# Supplementary material for: Thermochemical anomalies in the upper mantle control Gakkel Ridge accretion
Source: Nat Commun. 2021 Nov 29;12:6962. doi: 10.1038/s41467-021-27058-1 (PMC8630051; doi:10.1038/s41467-021-27058-1)
Supplement: Supplementary file 1 — Supplementary Information [file 41467_2021_27058_MOESM1_ESM.pdf]

Supplementary Information

Thermochemical anomalies in the upper mantle control Gakkel Ridge accretion

John M. O'Connor[1,2,3], Wilfried Jokat[2,4], Peter J. Michael[5], Mechita C. Schmidt-Aursch[2], Daniel P. Miggins[6], Anthony A.P. Koppers[6]

[1]GeoZentrum Nordbayern, University Erlangen-Nürnberg, Schlossgarten 5, 91054 Erlangen, Germany
[2]Alfred Wegener Institute Helmholtz Centre for Polar and Marine Research, Am Handelshafen 12, 27570 Bremerhaven Germany
[3]Faculty of Science, Vrije Universiteit Amsterdam, De Boelelaan 1085, 1081 HV Amsterdam, Netherlands
[4]University of Bremen, Fachbereich 5, 28359 Bremen, Germany
[5]College of Engineering & Natural Sciences, University of Tulsa, Oklahoma 74104, USA
[6]College of Earth, Ocean, and Atmospheric Sciences, Oregon State University, Corvallis, OR 97331-5503, USA

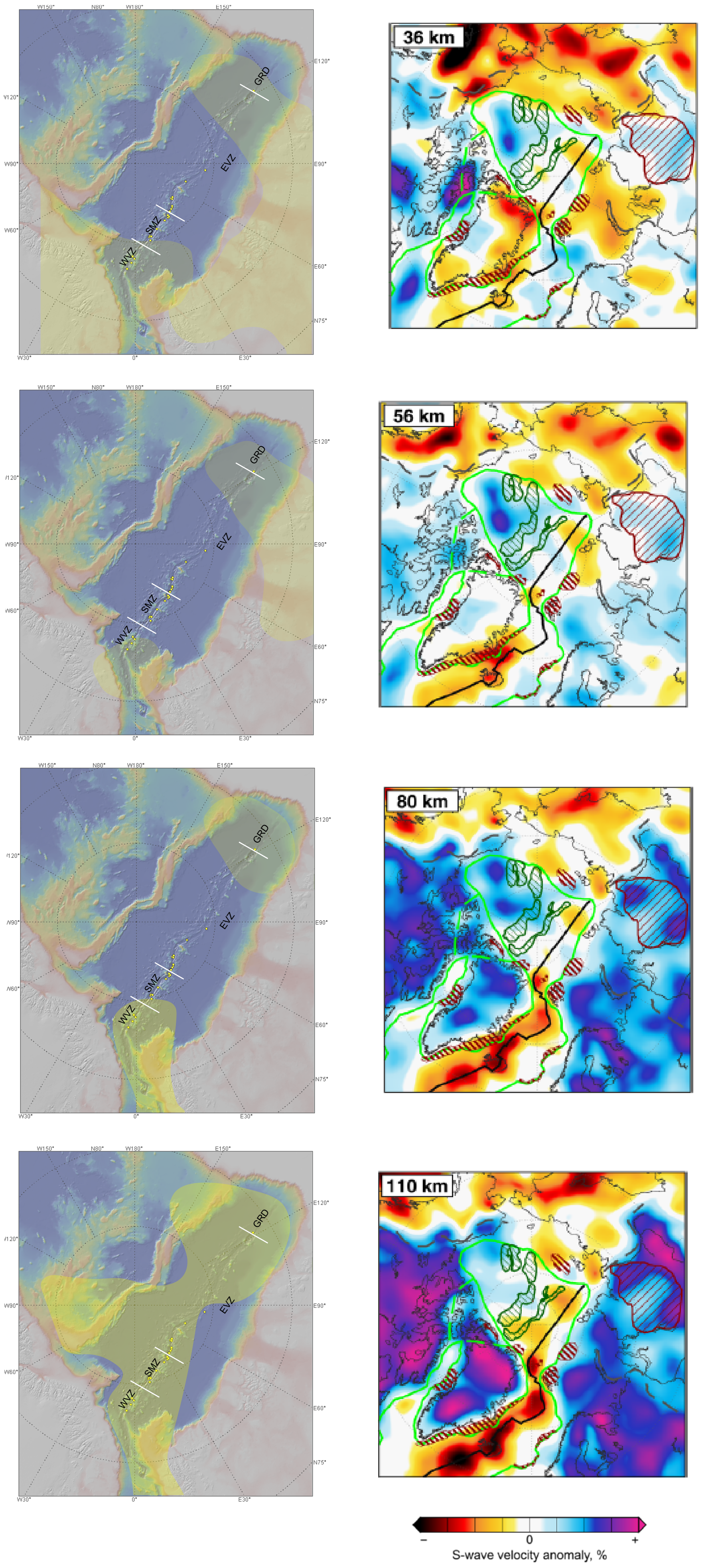

Supplementary Figure 1. Map views of AMISvArc tomographic model between 110 km to 36 km depths. This shows that the relation at 200 km and 150 km between the low-velocity anomalies and the Gakkel Ridge does not extend to shallower depths. Other details are the same as in Figure 6.

Supplementary Table 1. Results of $^{40}Ar/^{39}Ar$ groundmass incremental heating experiments

Sample Information					Results	Plateau										Normal Isochron							Inverse Isochron								Total Fusion			
Experiment Number	Sample Name	Zone	Age Type	Age Interpretation	Notes	Age	± 2σ (i)	± 2σ (f)	^{39}Ar	K/Ca	± 2σ	MSWD	P	n	N	Age	± 2σ (i)	± 2σ (f)	$^{40}Ar/^{36}Ar$ intercept	± 2σ	MSWD	P	Age	± 2σ (i)	± 2σ (f)	$^{40}Ar/^{36}Ar$ intercept	± 2σ	SF	MSWD	P	Age	± 2σ (i)	± 2σ (f)	K/Ca
18D00172	**PS59-223-27**	WVZ	**Plateau**	Crystallization Age	Mostly atmospheric	**947.6**	**± 160.1 ka**	± 161.6 ka	100%	0.013	± 0.002	0.51	98%	24	24	**917.4**	**± 304.0 ka**	± 304.7 ka	295.63	± 1.03	0.73	81%	**917**	**± 252.3 ka**	± 253.2 ka	295.64	± 1.03	1%	0.73	81%	**979**	**± 187.4 ka**	± 188.7 ka	0.031
17D20162	**PS59-226-23**	WVZ	**Plateau**	Crystallization Age		**1.65**	**± 0.09 Ma**	± 0.09 Ma	45%	0.02	± 0.007	1.85	5%	11	24	**2.45**	**± 0.63 Ma**	± 0.63 Ma	284.43	± 8.61	1.25	26%	**2.44**	**± 0.58 Ma**	± 0.58 Ma	284.59	± 8.54	3%	1.22	28%	**1.59**	**± 0.05 Ma**	± 0.06 Ma	0.026
17D30241	**PS59-231-20**	WVZ	**Plateau**	Crystallization Age	bumpy spectra	**791.6**	**± 66.3 ka**	± 68.6 ka	88%	0.012	± 0.005	1.83	1%	20	24	**889.9**	**± 80.5 ka**	± 83.0 ka	293.59	± 1.14	1.21	24%	**892.3**	**± 78.2 ka**	± 80.8 ka	293.59	± 1.13	9%	1.19	26%	**797.5**	**± 61.2 ka**	± 63.8 ka	0.033
17D19908	**HLY0102-D24-5**	WVZ	**Plateau**	Crystallization Age	Mostly atmospheric	**2.78**	**± 0.15 Ma**	± 0.16 Ma	91%	0.006	± 0.001	1.66	4%	19	24	**2.93**	**± 0.44 Ma**	± 0.44 Ma	294.06	± 3.86	1.71	3%	**2.94**	**± 0.41 Ma**	± 0.42 Ma	294.05	± 3.84	8%	1.69	4%	**2.85**	**± 0.12 Ma**	± 0.14 Ma	0.006
17D30446	**PS59-244-002**	SMZ	**Plateau**	Crystallization Age	Excess Initial 40Ar/36Ar = 296.73 ± 0.53 (SD).	**2.12**	**± 0.49 Ma**	± 0.49 Ma	100%	0.019	± 0.005	0.16	100%	24	24	**2.13**	**± 1.19 Ma**	± 1.19 Ma	296.73	± 1.06	0.22	100%	**2.13**	**± 1.01 Ma**	± 1.01 Ma	296.73	± 1.06	1%	0.22	100%	**2.34**	**± 0.73 Ma**	± 0.73 Ma	0.042
17D30134	**HLY0102-D35-20**	SMZ	**Plateau**	Crystallization Age	Excess Initial 40Ar/36Ar = 300.25 ± 2.41 (SD).	**3.13**	**± 0.09 Ma**	± 0.12 Ma	79%	0.069	± 0.014	0.8	63%	11	24	**2.94**	**± 0.30 Ma**	± 0.30 Ma	303.74	± 5.26	4.64	0%	**2.94**	**± 0.29 Ma**	± 0.30 Ma	303.82	± 5.26	9%	4.61	0%	**2.94**	**± 0.09 Ma**	± 0.11 Ma	0.062
17D19948	**HLY0102-D35-12**	SMZ	**Plateau**	Crystallization Age	Mostly atmospheric	**1.31**	**± 0.03 Ma**	± 0.04 Ma	68%	0.055	± 0.005	0.89	56%	13	24	**1.25**	**± 0.09 Ma**	± 0.09 Ma	297.24	± 2.52	0.8	64%	**1.25**	**± 0.09 Ma**	± 0.09 Ma	297.25	± 2.52	11%	0.8	64%	**1.29**	**± 0.03 Ma**	± 0.04 Ma	0.044
17D20015	**HLY0102-D37-8**	SMZ	**Plateau**	Crystallization Age	Excess Initial 40Ar/36Ar = 323.83 ± 2.94 (SD).	**2.18**	**± 0.09 Ma**	± 0.10 Ma	91%	0.047	± 0.011	1.32	20%	13	24	**2.21**	**± 0.26 Ma**	± 0.27 Ma	323.92	± 5.79	7.7	0%	**2.22**	**± 0.26 Ma**	± 0.26 Ma	323.83	± 5.88	16%	7.83	0%	**1.95**	**± 0.09 Ma**	± 0.10 Ma	0.063
17D19641	**HLY0102-D36-1**	SMZ	**Plateau**	Crystallization Age	age	**49**	**± 34.4 ka**	± 34.4 ka	99%	0.029	± 0.009	0.71	84%	23	24	**30**	**± 55.6 ka**	± 55.6 ka	296.75	± 0.80	0.83	69%	**30.7**	**± 14.2 ka**	± 14.2 ka	296.75	± 0.80	0%	0.83	68%	**95**	**± 100.7 ka**	± 100.7 ka	0.064
17D30553	**PS59-252-1**	SMZ	**Plateau**	Crystallization Age		**473.9**	**± 29.6 ka**	± 31.5 ka	83%	0.021	± 0.008	0.8	72%	21	24	**510.7**	**± 44.3 ka**	± 45.8 ka	294.53	± 0.95	0.87	62%	**511.2**	**± 43.6 ka**	± 45.1 ka	294.54	± 0.95	6%	0.87	63%	**509.6**	**± 44.6 ka**	± 46.0 ka	0.051
17D19681	**PS59-312-11**	SMZ	**Plateau**	Crystallization Age	Nice plateau	**1.68**	**± 0.05 Ma**	± 0.06 Ma	76%	0.024	± 0.008	1.69	6%	13	24	**1.95**	**± 0.52 Ma**	± 0.53 Ma	289.29	± 11.85	1.69	7%	**1.95**	**± 0.47 Ma**	± 0.47 Ma	289.38	± 11.82	3%	1.68	7%	**1.51**	**± 0.04 Ma**	± 0.05 Ma	0.032
17D19868	**PS59-311-2**	SMZ	**Plateau**	Crystallization Age	Mostly atmospheric	**661.3**	**± 67.8 ka**	± 69.5 ka	96%	0.016	± 0.004	2.4	0%	20	24	**604.5**	**± 160.6 ka**	± 161.2 ka	296.47	± 2.65	2.48	0%	**607.8**	**± 138.8 ka**	± 139.4 ka	296.48	± 2.65	4%	2.45	0%	**681**	**± 66.5 ka**	± 68.3 ka	0.025
17D20055	**PS59-310-1-1**	SMZ	**Plateau**	Crystallization Age	Excess Initial 40Ar/36Ar = 296.53 ± 0.42 (SD).	**294.8**	**± 52.8 ka**	± 53.2 ka	100%	0.03	± 0.006	0.81	72%	24	24	**292.5**	**± 85.6 ka**	± 85.8 ka	296.53	± 0.84	0.94	54%	**293.9**	**± 71.1 ka**	± 71.5 ka	296.53	± 0.84	2%	0.94	54%	**323.1**	**± 152.7 ka**	± 152.9 ka	0.063
18D00132	**HLY0102-D95-38**	SMZ	**Plateau**	Crystallization Age	Excess Initial 40Ar/36Ar = 297.99 ± 1.14 (SD).	**1.69**	**± 0.08 Ma**	± 0.09 Ma	79%	0.032	± 0.006	0.9	55%	13	24	**1.69**	**± 0.18 Ma**	± 0.18 Ma	297.95	± 2.26	1.66	8%	**1.69**	**± 0.18 Ma**	± 0.18 Ma	297.99	± 2.27	12%	1.66	7%	**1.52**	**± 0.11 Ma**	± 0.12 Ma	0.04
18D00504	**PS59-309-39**	EVZ	**Plateau**	Crystallization Age	Excess Initial 40Ar/36Ar = 301.16 ± 0.66 (SD). Mostly atmospheric	**589.9**	**± 116.2 ka**	± 117.0 ka	70%	0.018	± 0.002	0.86	59%	13	24	**489.6**	**± 168.3 ka**	± 168.7 ka	302.14	± 1.18	0.88	56%	**490.3**	**± 148.6 ka**	± 149.1 ka	302.15	± 1.18	3%	0.87	57%	**324.2**	**± 175.4 ka**	± 175.5 ka	0.018
17D24038	**HLY0102-D90-18**	EVZ	**Plateau**	Crystallization Age	Excess Initial 40Ar/36Ar = 297.96 ± 0.70 (SD).	**146.4**	**± 82.2 ka**	± 82.2 ka	100%	0.031	± 0.007	0.29	100%	30	30	**146.6**	**± 131.9 ka**	± 132.0 ka	297.95	± 1.40	0.37	100%	**146.7**	**± 75.1 ka**	± 75.1 ka	297.96	± 1.40	1%	0.37	100%	**103.8**	**± 117.0 ka**	± 117.0 ka	0.059
17D17883	**HLY0102-D89-004**	EVZ	**Plateau**	Crystallization Age		**1.41**	**± 0.06 Ma**	± 0.07 Ma	61%	0.026	± 0.004	1.03	41%	12	24	**1.52**	**± 0.38 Ma**	± 0.38 Ma	293.75	± 6.30	1.32	21%	**1.52**	**± 0.35 Ma**	± 0.35 Ma	293.73	± 6.31	4%	1.32	21%	**1.38**	**± 0.06 Ma**	± 0.07 Ma	0.023
17D30281	**HLY0102-D85-90**	EVZ	**Plateau**	Crystallization Age	Bumpy spectrum	**1.8**	**± 0.10 Ma**	± 0.11 Ma	92%	0.028	± 0.006	2.63	0%	17	24	**1.73**	**± 0.17 Ma**	± 0.17 Ma	296.3	± 1.59	2.62	0%	**1.73**	**± 0.17 Ma**	± 0.17 Ma	296.3	± 1.60	9%	2.63	0%	**1.72**	**± 0.13 Ma**	± 0.14 Ma	0.04
17D20349	**PS59-305-1**	EVZ	**Plateau**	Crystallization Age	Subatmospheric Initial 40Ar/36Ar = 291.41 ± 0.62 (SD).	**978.3**	**± 42.4 ka**	± 47.8 ka	71%	0.044	± 0.007	0.66	82%	16	24	**975.1**	**± 76.9 ka**	± 80.0 ka	291.42	± 1.23	0.81	66%	**976.9**	**± 76.2 ka**	± 79.3 ka	291.41	± 1.23	7%	0.82	65%	**1515.4**	**± 215.0 ka**	± 217.7 ka	0.071
17D19828	**PS59-305-20**	EVZ	**Plateau**	Crystallization Age	Mostly atmospheric	**757.9**	**± 28.9 ka**	± 33.6 ka	97%	0.02	± 0.006	1.05	40%	20	24	**839**	**± 236.6 ka**	± 237.3 ka	293.59	± 5.46	1.08	36%	**839.3**	**± 199.7 ka**	± 200.6 ka	293.61	± 5.45	3%	1.07	37%	**740.3**	**± 29.9 ka**	± 34.2 ka	0.063
17D19761	**PS59-300-18**	EVZ	**Plateau**	Crystallization Age	Mostly atmospheric	**3.58**	**± 0.07 Ma**	± 0.11 Ma	49%	0.017	± 0.001	1.11	35%	10	24	**3.67**	**± 0.28 Ma**	± 0.29 Ma	293.45	± 6.52	1.19	30%	**3.67**	**± 0.28 Ma**	± 0.29 Ma	293.37	± 6.47	13%	1.19	30%	**3.03**	**± 0.13 Ma**	± 0.15 Ma	0.024
18D00424	**PS59-299-1**	EVZ	**Plateau**	Crystallization Age	Mostly atmospheric	**1.0**	**± 0.30 Ma**	± 0.30 Ma	73%	0.004	± 0.000	0.91	54%	13	24	**1.0**	**± 0.67 Ma**	± 0.67 Ma	295.18	± 3.93	1.58	10%	**1.0**	**± 0.44 Ma**	± 0.44 Ma	295.27	± 3.89	3%	1.56	10%	**3.02**	**± 0.46 Ma**	± 0.46 Ma	0.004
18D00464	**PS59-271-1-14**	EVZ	**Plateau**	Crystallization Age	Excess Initial 40Ar/36Ar = 304.74 ± 1.70 (SD). Mostly atmospheric	**1.12**	**± 0.11 Ma**	± 0.11 Ma	88%	0.026	± 0.005	1.73	4%	16	24	**1.13**	**± 0.25 Ma**	± 0.25 Ma	305.1	± 3.40	3.28	0%	**1.14**	**± 0.22 Ma**	± 0.23 Ma	305.05	± 3.37	7%	3.21	0%	**1.06**	**± 0.11 Ma**	± 0.11 Ma	0.03
17D20095	**PS59-276-1-005**	EVZ	**Plateau**	Crystallization Age		**212.5**	**± 26.0 ka**	± 26.5 ka	80%	0.017	± 0.009	1.22	24%	17	24	**206.7**	**± 51.0 ka**	± 51.2 ka	295.68	± 1.57	1.29	20%	**207.1**	**± 46.1 ka**	± 46.3 ka	295.7	± 1.58	3%	1.3	19%	**319.5**	**± 50.5 ka**	± 51.1 ka	0.061
18D00611	**HLY0102-D66-32**	EVZ	**Plateau**	Crystallization Age	Mostly atmospheric	**1.47**	**± 0.01 Ma**	± 0.04 Ma	95%	0.097	± 0.015	1.0	45%	19	24	**1.49**	**± 0.02 Ma**	± 0.04 Ma	293.45	± 2.55	0.99	47%	**1.49**	**± 0.02 Ma**	± 0.04 Ma	293.43	± 2.56	40%	0.98	48%	**1.45**	**± 0.02 Ma**	± 0.04 Ma	0.108
18D00212	**PS59-229-13**		**Plateau**	Crystallization Age with high error	Subatmospheric Initial 40Ar/36Ar = 291.31 ± 1.43 (SD).	**5.59**	**± 1.41 Ma**	± 1.41 Ma	62%	0.02	± 0.005	0.15	100%	14	22	**3.37**	**± 3.29 Ma**	± 3.29 Ma	292.77	± 2.08	0.47	93%	**3.37**	**± 2.13 Ma**	± 2.13 Ma	292.77	± 2.08	1%	0.47	93%	**16.7**	**± 3.01 Ma**	± 3.03 Ma	0.025
18D00651	**HLY0102-D55-9**		**Plateau**	Crystallization Age with high error	Mostly atmospheric	**4.27**	**± 0.75 Ma**	± 0.76 Ma	71%	0.007	± 0.002	0.85	60%	14	24	**21.76**	**± 9.76 Ma**	± 9.77 Ma	281.31	± 7.93	0.52	90%	**21.75**	**± 8.38 Ma**	± 8.39 Ma	281.32	± 7.94	1%	0.52	90%	**9.69**	**± 0.83 Ma**	± 0.86 Ma	0.013
18D00544	**PS59-297-37**		**Plateau**	Crystallization Age with high error	Mostly atmospheric	**1.1**	**± 0.73 Ma**	± 0.73 Ma	100%	0.009	± 0.001	0.66	89%	24	24																**2.13**	**± 1.11 Ma**	± 1.11 Ma	0.012
17D23804	**PS59-225-18**			**No Age**	Mostly atmospheric										30																2388.5	± 555.4 ka	± 558.0 ka	0.019
17D24083	**HLY0102-D23-1**			**No Age**	Subatmospheric Initial 40Ar/36Ar = 294.90 ± 1.18 (SD).										30																5862.5	± 1402.9 ka	± 1409.2 ka	0.024
17D30593	**HLY0102-D36-9**			**No Age**	Mostly atmospheric										24																318.1	± 226.4 ka	± 226.6 ka	0.086
17D15594	**PS59-254-1**			**No Age**	Mostly atmospheric										41																80.1	± 86.8 ka	± 86.8 ka	0.042
18D00249	**PS59-260-1**			**No Age**	Mostly atmospheric																													0.062
17D19721	**HLY0102-D91-21**			**No Age**	Mostly atmospheric										24																145.1	± 207.1 ka	± 207.1 ka	0.04
17D23687	**HLY0102-D52-2**			**No Age**	Subatmospheric Initial 40Ar/36Ar = 293.71 ± 1.01 (SD). Mostly atmospheric	**74.3**	**± 241.9 ka**	± 241.9 ka	84%	0.021	± 0.006	0.59	92%	21	30	88.3	± 797.8 ka	± 797.8 ka	293.71	± 2.02	1.31	17%	91.1	± 41.7 ka	± 41.8 ka	293.71	± 2.01	0%	1.31	16%	910	± 378.8 ka	± 379.3 ka	0.03
17D24182	**HLY0102-D83-1**			**No Age**	Mostly atmospheric										30																0.13	± 0.59 Ma	± 0.59 Ma	0.024
18D00316	**HLY0102-D51-29**			**No Age**	Mostly atmospheric																													0.025
17D23732	**PS59-270-36**			**No Age**	Mostly atmospheric										30																42.1	± 144.6 ka	± 144.6 ka	0.039
17D20202	**PS59-295-1**			**No Age**	Mostly atmospheric	**28.7**	**± 71.5 ka**	± 71.5 ka	100%	0.021	± 0.006	1.16	27%	24	24	29.5	± 116.8 ka	± 116.8 ka	295.35	± 0.80	1.4	10%	29.7	± 12.6 ka	± 12.6 ka	295.36	± 0.79	0%	1.4	10%	74.6	± 120.9 ka	± 121.0 ka	0.048
17D20282	**PS59-293-17**			**No Age**	Mostly atmospheric										24																1.92	± 1.10 Ma	± 1.10 Ma	0.033
17D30633	**HLY0102-D57-2**			**No Age**	Mostly atmospheric	**6.16**	**± 0.03 Ma**			0.021	± 0.000				24	6.23	± 0.14 Ma		282.35	± 19.53			6.22	± 0.14 Ma		284.06	± 19.64				0.36	± 0.41 Ma	± 0.41 Ma	0.025
17D23921	**PS59-275-1**			**No Age**	Subatmospheric Initial 40Ar/36Ar = 287.32 ± 1.83 (SD). Mostly atmospheric										30																21.48	± 2.88 Ma	± 2.92 Ma	0.017
17D30486	**PS59-290-1-44**			**No Age**	Mostly atmospheric										24																1.37	± 1.73 Ma	± 1.73 Ma	0.016
17D23966	**HLY0102-D58-10**			**No Age**	Subatmospheric Initial 40Ar/36Ar = 292.35 ± 2.25 (SD). Mostly atmospheric										30																1939.3	± 787.0 ka	± 788.2 ka	0.038
18D00356	**PS59-289-69**			**No Age**	Mostly atmospheric																													0.025
