# Supplementary material for: Thermochemical anomalies in the upper mantle control Gakkel Ridge accretion
Source: Nat Commun. 2021 Nov 29;12:6962. doi: 10.1038/s41467-021-27058-1 (PMC8630051; doi:10.1038/s41467-021-27058-1)
Supplement: Supplementary file 3 — Description of Additional Supplementary Files [file 41467_2021_27058_MOESM3_ESM.pdf]

Description of Additional Supplementary Files

File name: Supplementary Data 1
Description: Summary versions of ArArCalc age files

File name: Supplementary Data 2
Description: Full versions of ArArCalc age files
