# Supplementary material for: Thermochemical anomalies in the upper mantle control Gakkel Ridge accretion
Source: Nat Commun. 2021 Nov 29;12:6962. doi: 10.1038/s41467-021-27058-1 (PMC8630051; doi:10.1038/s41467-021-27058-1)

Supplementary Data 1

Summary versions of ArArCalc age files

EXP#18D00172 > PS59-223-27 > Groundmass > O-CONNOR (16-22)
ARTIC OCEAN > GAKKEL RIDGE
17-OSU-05 (5B35-17) > Incremental Heating > Dan Miggins

Information on Analysis and Constants Used in Calculations

Project = **O-CONNOR (16-22)**
Sample = **PS59-223-27**
Material = **Groundmass**
Location = **Gakkel Ridge**
Region = **Artic Ocean**
Analyst = **Dan Miggins**
Irradiation = **17-OSU-05 (5B35-17)**
Position = **X: 0 | Y: 0 | Z/H: 51.18569 mm**
FCT-NM Age = **28.201 ± 0.023 Ma**
FCT-NM Reference = **Kuiper et al (2008)**
FCT-NM 40Ar/39Ar Ratio = **10.30417 ± 0.00793**
FCT-NM J-value = **0.00152534 ± 0.00000117**
Air Shot 40Ar/36Ar = **302.9450 ± 0.3969**
Air Shot MDF = **0.99384820 ± 0.00066120 (LIN)**
Experiment Type = **Incremental Heating**
Extraction Method = **Bulk Laser Heating**
Heating = **64 sec**
Isolation = **3.00 min**
Instrument = **ARGUS-VI-D**
Preferred Age = **Plateau Age**
Age Classification = **Crystallization Age**
IGSN = Undefined
Rock Class = Undefined
Lithology = **Basaltic Lava**
Lat-Lon = Undefined - Undefined
Age Equations = **Min et al. (2000)**
Negative Intensities = **Allowed**
Collector Calibrations = **36Ar**
Decay 40K = **5.530 ± 0.048 E-10 1/a**
Decay 39Ar = **2.940 ± 0.016 E-07 1/h**
Decay 37Ar = **8.230 ± 0.012 E-04 1/h**
Decay 36Cl = **2.257 ± 0.015 E-06 1/a**
Decay 40K(EC,β^+) = **0.580 ± 0.009 E-10 1/a**
Decay 40K(β^-) = **4.950 ± 0.043 E-10 1/a**
Atmospheric 40/36(a) = **295.50 ± 0.70**
Atmospheric 38/36(a) = **0.1869**
Production 39/37(ca) = **0.0006425 ± 0.0000059**
Production 38/37(ca) = **0.0001800 ± 0.0000173**
Production 36/37(ca) = **0.0002703 ± 0.0000005**
Production 40/39(k) = **0.000607 ± 0.000059**
Production 38/39(k) = **0.012077 ± 0.000011**
Production 36/38(cl) = **262.80 ± 1.71**
Scaling Ratio K/Ca = **0.430**
Abundance Ratio 40K/K = **1.1700 ± 0.0100 E-04**
Atomic Weight K = **39.0983 ± 0.0001 g**

Mostly atmospheric

Results

Results	40(a)/36(a) ± 2σ		40(r)/39(k) ± 2σ		Age ± 2σ (ka)		MSWD	39Ar(k) (%,n)	K/Ca ± 2σ
**Age Plateau**			0.34363	± 0.05808<br>± 16.90%	**947.6**	**± 160.1**<br>**± 16.90%**	0.51<br>98%	100.00<br>24	0.0130 ± 0.0020
					Full External Error	± 161.6	1.59	2σ Confidence Limit	
					Analytical Error	± 160.1	1.0000	Error Magnification	
**Total Fusion Age**			0.35504	± 0.06796<br>± 19.14%	**979.0**	**± 187.4**<br>**± 19.14%**		24	0.0314 ± 0.0002
					Full External Error	± 188.7			
					Analytical Error	± 187.4			
**Normal Isochron**	**295.63**	**± 1.03**<br>**± 0.35%**	0.33268	± 0.11028<br>± 33.15%	**917.4**	**± 304.0**<br>**± 33.14%**	0.73<br>81%	100.00<br>24	
					Full External Error	± 304.7	1.60	2σ Confidence Limit	
					Analytical Error	± 304.0	1.0000	Error Magnification	
**Inverse Isochron**<br>**Clustered Points**	**295.64**	**± 1.03**<br>**± 0.35%**	0.33253	± 0.09153<br>± 27.53%	**917.0**	**± 252.3**<br>**± 27.52%**	0.73<br>81%	100.00<br>24	
					Full External Error	± 253.2	1.60	2σ Confidence Limit	
					Analytical Error	± 252.3	1.0000	Error Magnification	
							1%	Spreading Factor	

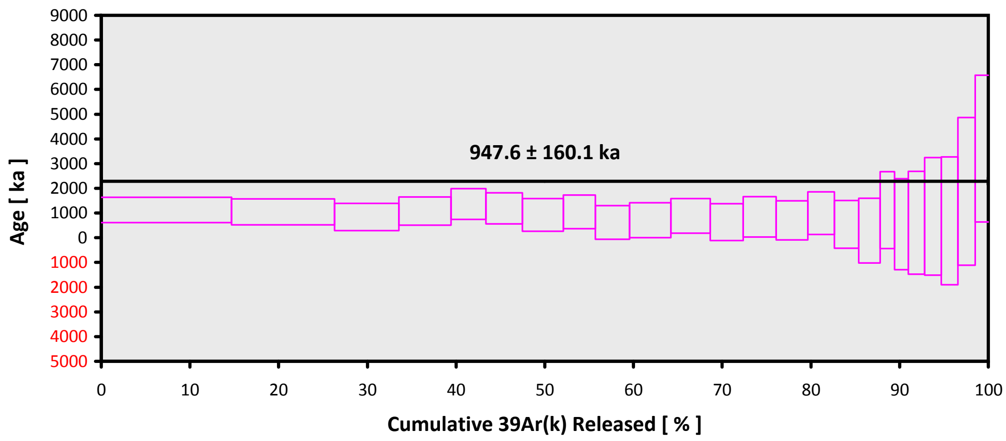

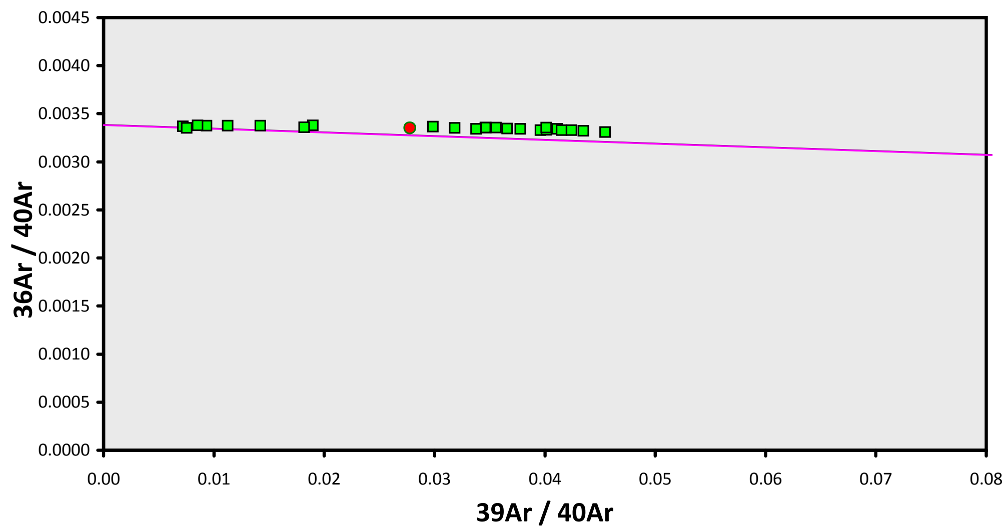

EXP#17D20162 > PS59-226-23 > Groundmass > O-CONNOR (16-22)
ARTIC OCEAN > GAKKEL RIDGE
17-OSU-01 (1F23-17) > Incremental Heating > Dan Miggins

Information on Analysis and Constants Used in Calculations

Project = **O-CONNOR (16-22)**
Sample = **PS59-226-23**
Material = **Groundmass**
Location = **Gakkel Ridge**
Region = **Artic Ocean**
Analyst = **Dan Miggins**
Irradiation = **17-OSU-01 (1F23-17)**
Position = **X: 0 | Y: 0 | Z/H: 30.0291 mm**
FCT-NM Age = **28.201 ± 0.023 Ma**
FCT-NM Reference = **Kuiper et al (2008)**
FCT-NM 40Ar/39Ar Ratio = **9.64936 ± 0.00917**
FCT-NM J-value = **0.00162885 ± 0.00000155**
Air Shot 40Ar/36Ar = **302.4490 ± 0.4597**
Air Shot MDF = **0.99424863 ± 0.00068860 (LIN)**
Experiment Type = **Incremental Heating**
Extraction Method = **Bulk Laser Heating**
Heating = **64 sec**
Isolation = **3.00 min**
Instrument = **ARGUS-VI-D**
Preferred Age = **Plateau Age**
Age Classification = **Crystallization Age**
IGSN = Undefined
Rock Class = **Igneous>Volcanic**
Lithology = **Basaltic Lava**
Lat-Lon = Undefined - Undefined
Age Equations = **Min et al. (2000)**
Negative Intensities = **Allowed**
Collector Calibrations = **36Ar**
Decay 40K = **5.530 ± 0.048 E-10 1/a**
Decay 39Ar = **2.940 ± 0.016 E-07 1/h**
Decay 37Ar = **8.230 ± 0.012 E-04 1/h**
Decay 36Cl = **2.257 ± 0.015 E-06 1/a**
Decay 40K(EC,β^+) = **0.580 ± 0.009 E-10 1/a**
Decay 40K(β^-) = **4.950 ± 0.043 E-10 1/a**
Atmospheric 40/36(a) = **295.50**
Atmospheric 38/36(a) = **0.1869**
Production 39/37(ca) = **0.0006425 ± 0.0000059**
Production 38/37(ca) = **0.0001800 ± 0.0000173**
Production 36/37(ca) = **0.0002703 ± 0.0000005**
Production 40/39(k) = **0.000607 ± 0.000059**
Production 38/39(k) = **0.012077 ± 0.000011**
Production 36/38(cl) = **262.80 ± 1.71**
Scaling Ratio K/Ca = **0.430**
Abundance Ratio 40K/K = **1.1700 ± 0.0100 E-04**
Atomic Weight K = **39.0983 ± 0.0001 g**

Results	40(a)/36(a) ± 2σ	40(r)/39(k) ± 2σ	Age ± 2σ (Ma)	MSWD	39Ar(k) (%,n)	K/Ca ± 2σ
**Age Plateau**		0.55967 ± 0.02960<br>± 5.29%	**1.65 ± 0.09**<br>**± 5.29%**	1.85<br>5%	44.75<br>11	0.0201 ± 0.0066
			Full External Error ± 0.09	1.89	2σ Confidence Limit	
			Analytical Error ± 0.09	1.3595	Error Magnification	
**Total Fusion Age**		0.53996 ± 0.01778<br>± 3.29%	**1.59 ± 0.05**<br>**± 3.30%**		24	0.0263 ± 0.0001
			Full External Error ± 0.06			
			Analytical Error ± 0.05			
**Normal Isochron**	**284.43 ± 8.61**<br>**± 3.03%**	0.83313 ± 0.21425<br>± 25.72%	**2.45 ± 0.63**<br>**± 25.70%**	1.25<br>26%	44.75<br>11	
			Full External Error ± 0.63	1.94	2σ Confidence Limit	
			Analytical Error ± 0.63	1.1169	Error Magnification	
**Inverse Isochron**<br>Clustered Points	**284.59 ± 8.54**<br>**± 3.00%**	0.82946 ± 0.19558<br>± 23.58%	**2.44 ± 0.58**<br>**± 23.56%**	1.22<br>28%	44.75<br>11	
			Full External Error ± 0.58	1.94	2σ Confidence Limit	
			Analytical Error ± 0.58	1.1056	Error Magnification	
				3%	Spreading Factor	

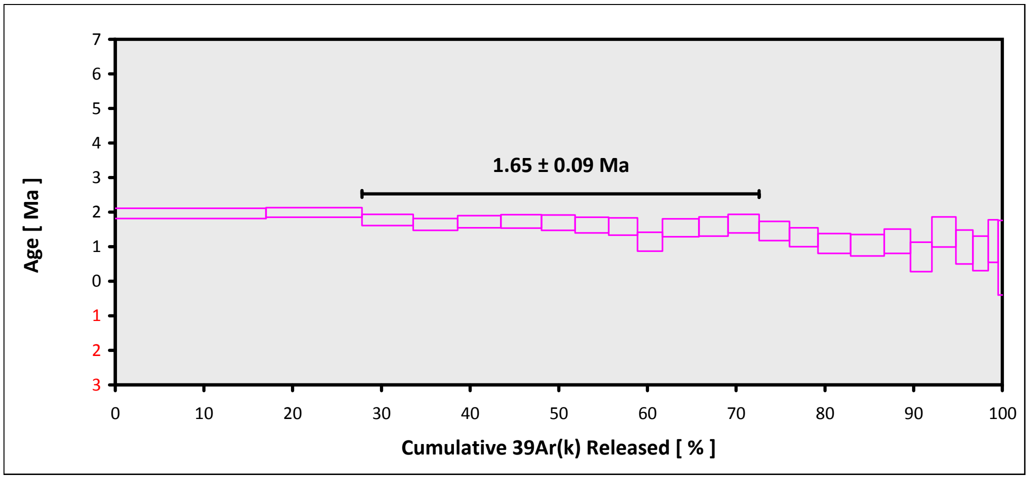

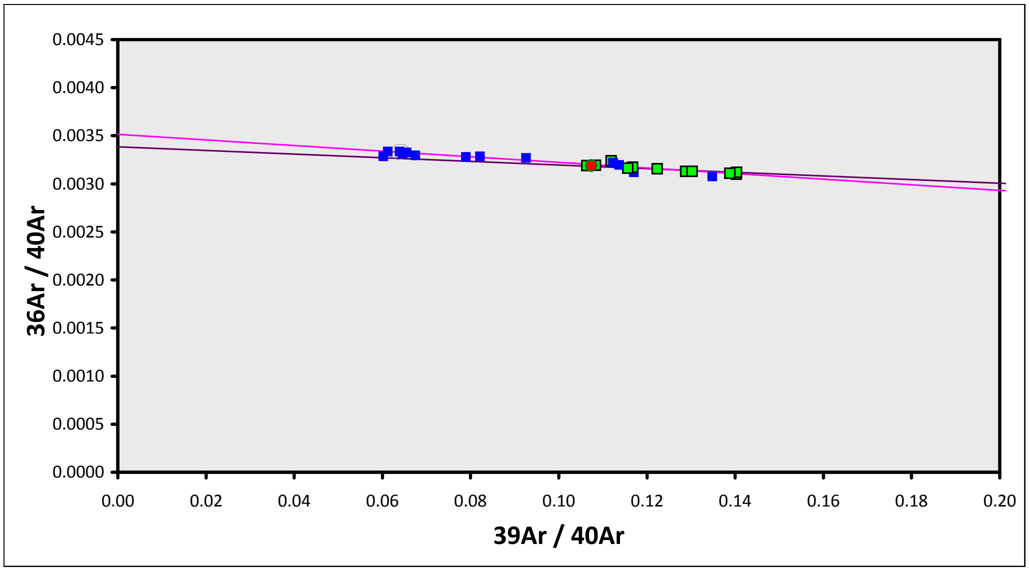

EXP#17D30241 > PS59-231-20 > Groundmass > O-CONNOR (16-22)
ARTIC OCEAN > GAKKEL RIDGE
17-OSU-05 (5B3-17) > Incremental Heating > Dan Miggins

Information on Analysis and Constants Used in Calculations

Project = **O-CONNOR (16-22)**
Sample = **PS59-231-20**
Material = **Groundmass**
Location = **Gakkel Ridge**
Region = **Artic Ocean**
Analyst = **Dan Miggins**
Irradiation = **17-OSU-05 (5B3-17)**
Position = **X: 0 | Y: 0 | Z/H: 4.863966 mm**
FCT-NM Age = **28.201 ± 0.023 Ma**
FCT-NM Reference = **Kuiper et al (2008)**
FCT-NM 40Ar/39Ar Ratio = **9.56637 ± 0.00794**
FCT-NM J-value = **0.00164298 ± 0.00000136**
Air Shot 40Ar/36Ar = **302.7710 ± 0.1605**
Air Shot MDF = **0.99398853 ± 0.00059332 (LIN)**
Experiment Type = **Incremental Heating**
Extraction Method = **Bulk Laser Heating**
Heating = **64 sec**
Isolation = **3.00 min**
Instrument = **ARGUS-VI-D**
Preferred Age = **Plateau Age**
Age Classification = **Crystallization Age**
IGSN = Undefined
Rock Class = **Igneous>Volcanic**
Lithology = **Basaltic Lava**
Lat-Lon = Undefined - Undefined
Age Equations = **Min et al. (2000)**
Negative Intensities = **Allowed**
Collector Calibrations = **36Ar**
Decay 40K = **5.530 ± 0.048 E-10 1/a**
Decay 39Ar = **2.940 ± 0.016 E-07 1/h**
Decay 37Ar = **8.230 ± 0.012 E-04 1/h**
Decay 36Cl = **2.257 ± 0.015 E-06 1/a**
Decay 40K(EC,β^+) = **0.580 ± 0.009 E-10 1/a**
Decay 40K(β^-) = **4.950 ± 0.043 E-10 1/a**
Atmospheric 40/36(a) = **295.50**
Atmospheric 38/36(a) = **0.1869**
Production 39/37(ca) = **0.0006425 ± 0.0000059**
Production 38/37(ca) = **0.0001800 ± 0.0000173**
Production 36/37(ca) = **0.0002703 ± 0.0000005**
Production 40/39(k) = **0.000607 ± 0.000059**
Production 38/39(k) = **0.012077 ± 0.000011**
Production 36/38(cl) = **262.80 ± 1.71**
Scaling Ratio K/Ca = **0.430**
Abundance Ratio 40K/K = **1.1700 ± 0.0100 E-04**
Atomic Weight K = **39.0983 ± 0.0001 g**

bumpy sepctra

Results

Results	40(a)/36(a) ± 2σ		40(r)/39(k) ± 2σ		Age ± 2σ (ka)		MSWD	39Ar(k) (%,n)	K/Ca ± 2σ
**Age Plateau**			0.26651	± 0.02231	**791.6**	**± 66.3**	1.83	88.28	0.0122 ± 0.0050
Error Mean				± 8.37%		**± 8.37%**	1%	20	
					Full External Error	± 68.6	1.65	2σ Confidence Limit	
					Analytical Error	± 66.3	1.3526	Error Magnification	
**Total Fusion Age**			0.26848	± 0.02061	**797.5**	**± 61.2**		24	0.0326 ± 0.0001
				± 7.68%		**± 7.68%**			
					Full External Error	± 63.8			
					Analytical Error	± 61.2			
**Normal Isochron**	**293.59**	**± 1.14**	0.29960	± 0.02710	**889.9**	**± 80.5**	1.21	88.28	
		**± 0.39%**		± 9.05%		**± 9.05%**	24%	20	
					Full External Error	± 83.0	1.67	2σ Confidence Limit	
					Analytical Error	± 80.5	1.0995	Error Magnification	
**Inverse Isochron**	**293.59**	**± 1.13**	0.30039	± 0.02634	**892.3**	**± 78.2**	1.19	88.28	
		**± 0.39%**		± 8.77%		**± 8.77%**	26%	20	
					Full External Error	± 80.8	1.67	2σ Confidence Limit	
					Analytical Error	± 78.2	1.0894	Error Magnification	
							9%	Spreading Factor	

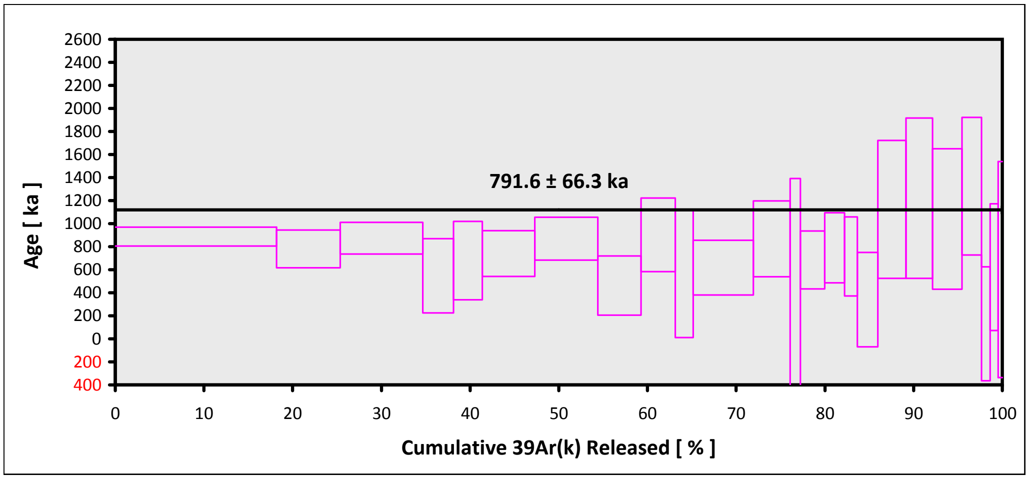

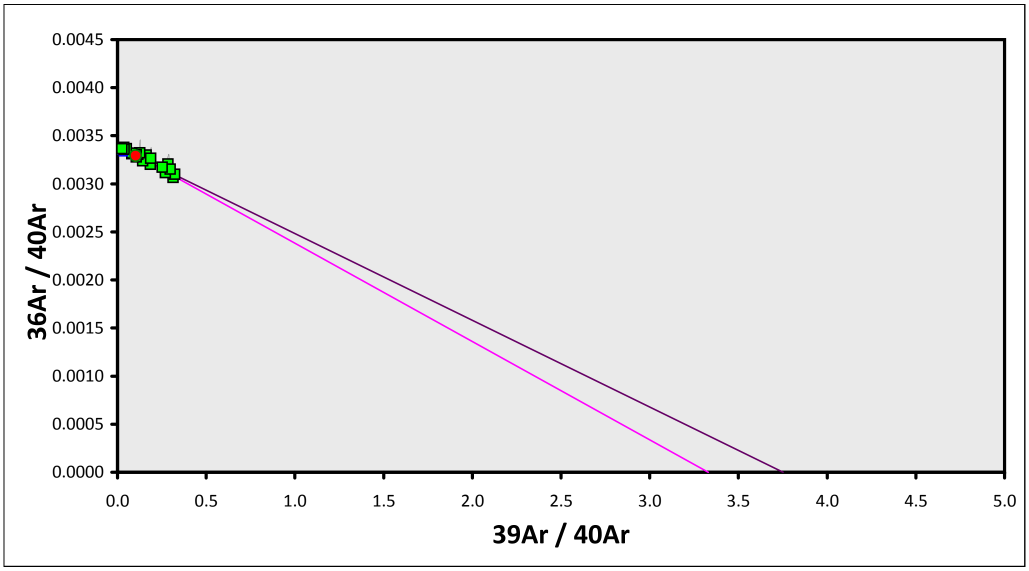

EXP#17D19908 > HLY0102-D24-5 > Groundmass > O-CONNOR (16-22)
ARTIC OCEAN > GAKKEL RIDGE
17-OSU-01 (1C17-17) > Incremental Heating > Dan Miggins

Information on Analysis and Constants Used in Calculations

Project = **O-CONNOR (16-22)**
Sample = **HLY0102-D24-5**
Material = **Groundmass**
Location = **Gakkel Ridge**
Region = **Artic Ocean**
Analyst = **Dan Miggins**
Irradiation = **17-OSU-01 (1C17-17)**
Position = **X: 0 | Y: 0 | Z/H: 26.42529 mm**
FCT-NM Age = **28.201 ± 0.023 Ma**
FCT-NM Reference = **Kuiper et al (2008)**
FCT-NM 40Ar/39Ar Ratio = **9.60352 ± 0.00855**
FCT-NM J-value = **0.00163663 ± 0.00000146**
Air Shot 40Ar/36Ar = **302.4660 ± 0.4204**
Air Shot MDF = **0.99423489 ± 0.00067193 (LIN)**
Experiment Type = **Incremental Heating**
Extraction Method = **Bulk Laser Heating**
Heating = **64 sec**
Isolation = **3.00 min**
Instrument = **ARGUS-VI-D**
Preferred Age = **Plateau Age**
Age Classification = **Crystallization Age**
IGSN = Undefined
Rock Class = **Igneous>Volcanic**
Lithology = **Basaltic Lava**
Lat-Lon = Undefined - Undefined
Age Equations = **Min et al. (2000)**
Negative Intensities = **Allowed**
Collector Calibrations = **36Ar**
Decay 40K = **5.530 ± 0.048 E-10 1/a**
Decay 39Ar = **2.940 ± 0.016 E-07 1/h**
Decay 37Ar = **8.230 ± 0.012 E-04 1/h**
Decay 36Cl = **2.257 ± 0.015 E-06 1/a**
Decay 40K(EC,β^+) = **0.580 ± 0.009 E-10 1/a**
Decay 40K(β^-) = **4.950 ± 0.043 E-10 1/a**
Atmospheric 40/36(a) = **295.50**
Atmospheric 38/36(a) = **0.1869**
Production 39/37(ca) = **0.0006425 ± 0.0000059**
Production 38/37(ca) = **0.0001800 ± 0.0000173**
Production 36/37(ca) = **0.0002703 ± 0.0000005**
Production 40/39(k) = **0.000607 ± 0.000059**
Production 38/39(k) = **0.012077 ± 0.000011**
Production 36/38(cl) = **262.80 ± 1.71**
Scaling Ratio K/Ca = **0.430**
Abundance Ratio 40K/K = **1.1700 ± 0.0100 E-04**
Atomic Weight K = **39.0983 ± 0.0001 g**

Mostly atmospheric

Results

Results	40(a)/36(a) ± 2σ		40(r)/39(k) ± 2σ		Age ± 2σ (Ma)		MSWD	39Ar(k) (%,n)	K/Ca ± 2σ
**Age Plateau**			0.94130	± 0.05042<br>± 5.36%	**2.78**	**± 0.15<br>± 5.36%**	1.66<br>4%	90.69<br>19	0.0060 ± 0.0010
					Full External Error	± 0.16	1.67	2σ Confidence Limit	
					Analytical Error	± 0.15	1.2872	Error Magnification	
**Total Fusion Age**			0.96445	± 0.04198<br>± 4.35%	**2.85**	**± 0.12<br>± 4.35%**		24	0.0060 ± 0.0000
					Full External Error	± 0.14			
					Analytical Error	± 0.12			
**Normal Isochron**<br>Error Chron	**294.06**	**± 3.86<br>± 1.31%**	0.99080	± 0.14823<br>± 14.96%	**2.93**	**± 0.44<br>± 14.95%**	1.71<br>3%	90.69<br>19	
					Full External Error	± 0.44	1.69	2σ Confidence Limit	
					Analytical Error	± 0.44	1.3058	Error Magnification	
**Inverse Isochron**<br>Error Chron	**294.05**	**± 3.84<br>± 1.30%**	0.99408	± 0.14017<br>± 14.10%	**2.94**	**± 0.41<br>± 14.09%**	1.69<br>4%	90.69<br>19	
					Full External Error	± 0.42	1.69	2σ Confidence Limit	
					Analytical Error	± 0.41	1.3012	Error Magnification	
							8%	Spreading Factor	

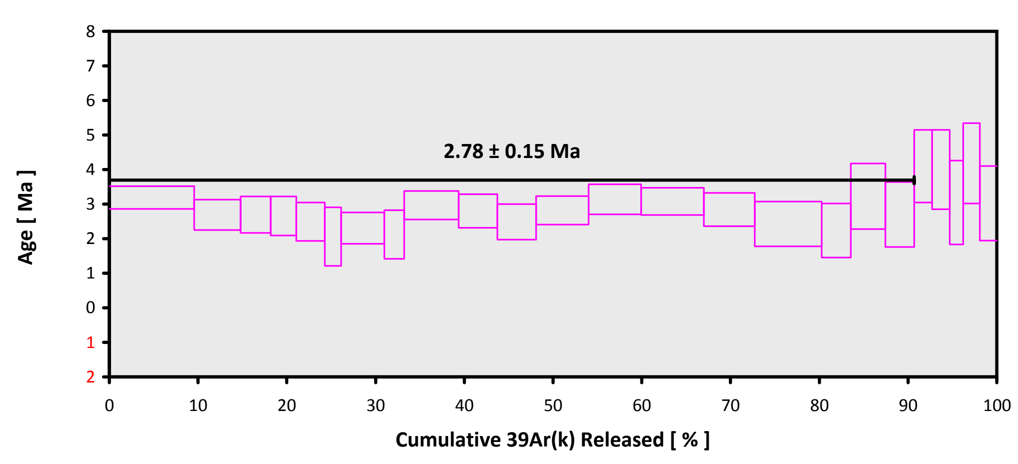

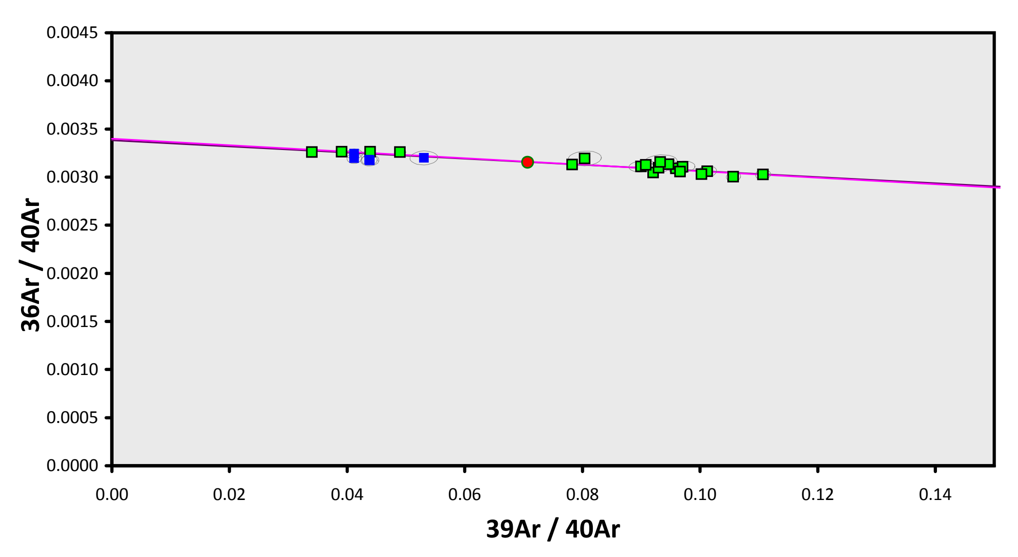

EXP#17D30446 > PS59-244-002 > Groundmass > O-CONNOR (16-22)
ARTIC OCEAN > GAKKEL RIDGE
17-OSU-05 (5A41-17) > Incremental Heating > Dan Miggins

Information on Analysis and Constants Used in Calculations

Project = **O-CONNOR (16-22)**
Sample = **PS59-244-002**
Material = **Groundmass**
Location = **Gakkel Ridge**
Region = **Artic Ocean**
Analyst = **Dan Miggins**
Irradiation = **17-OSU-05 (5A41-17)**
Position = **X: 0 | Y: 0 | Z/H: 60.23046 mm**
FCT-NM Age = **28.201 ± 0.023 Ma**
FCT-NM Reference = **Kuiper et al (2008)**
FCT-NM 40Ar/39Ar Ratio = **10.59431 ± 0.01112**
FCT-NM J-value = **0.00148357 ± 0.00000156**
Air Shot 40Ar/36Ar = **302.6380 ± 0.4540**
Air Shot MDF = **0.99409590 ± 0.00068555 (LIN)**
Experiment Type = **Incremental Heating**
Extraction Method = **Bulk Laser Heating**
Heating = **64 sec**
Isolation = **3.00 min**
Instrument = **ARGUS-VI-D**
Preferred Age = **Plateau Age**
Age Classification = **Crystallization Age**
IGSN = Undefined
Rock Class = **Igneous>Volcanic**
Lithology = **Basaltic Lava**
Lat-Lon = Undefined - Undefined
Age Equations = **Min et al. (2000)**
Negative Intensities = **Allowed**
Collector Calibrations = **36Ar**
Decay 40K = **5.530 ± 0.048 E-10 1/a**
Decay 39Ar = **2.940 ± 0.016 E-07 1/h**
Decay 37Ar = **8.230 ± 0.012 E-04 1/h**
Decay 36Cl = **2.257 ± 0.015 E-06 1/a**
Decay 40K(EC,β^+) = **0.580 ± 0.009 E-10 1/a**
Decay 40K(β^-) = **4.950 ± 0.043 E-10 1/a**
Atmospheric 40/36(a) = **296.73 ± 0.53**
Atmospheric 38/36(a) = **0.1869**
Production 39/37(ca) = **0.0006425 ± 0.0000059**
Production 38/37(ca) = **0.0001800 ± 0.0000173**
Production 36/37(ca) = **0.0002703 ± 0.0000005**
Production 40/39(k) = **0.000607 ± 0.000059**
Production 38/39(k) = **0.012077 ± 0.000011**
Production 36/38(cl) = **262.80 ± 1.71**
Scaling Ratio K/Ca = **0.430**
Abundance Ratio 40K/K = **1.1700 ± 0.0100 E-04**
Atomic Weight K = **39.0983 ± 0.0001 g**

Excess Initial 40Ar/36Ar = 296.73 ± 0.18 (%SD).

Results

Results	40(a)/36(a) ± 2σ		40(r)/39(k) ± 2σ		Age ± 2σ (Ma)		MSWD	39Ar(k) (%,n)	K/Ca ± 2σ
**Age Plateau**			0.79033	± 0.18228	**2.12**	**± 0.49**	0.16	100.00	0.0194 ± 0.0050
Overestimated				± 23.06%		**± 23.05%**	100%	24	
				Full External Error		± 0.49	1.59	2σ Confidence Limit	
				Analytical Error		± 0.49	1.0000	Error Magnification	
**Total Fusion Age**			0.87172	± 0.27061	**2.34**	**± 0.73**		24	0.0418 ± 0.0001
				± 31.04%		**± 31.02%**			
				Full External Error		± 0.73			
				Analytical Error		± 0.73			
**Normal Isochron**	**296.73**	**± 1.06**	0.79302	± 0.44320	**2.13**	**± 1.19**	0.22	100.00	
Overestimated		**± 0.36%**		± 55.89%		**± 55.86%**	100%	24	
				Full External Error		± 1.19	1.60	2σ Confidence Limit	
				Analytical Error		± 1.19	1.0000	Error Magnification	
**Inverse Isochron**	**296.73**	**± 1.06**	0.79331	± 0.37678	**2.13**	**± 1.01**	0.22	100.00	
Overestimated		**± 0.36%**		± 47.49%		**± 47.47%**	100%	24	
				Full External Error		± 1.01	1.60	2σ Confidence Limit	
				Analytical Error		± 1.01	1.0000	Error Magnification	
							1%	Spreading Factor	

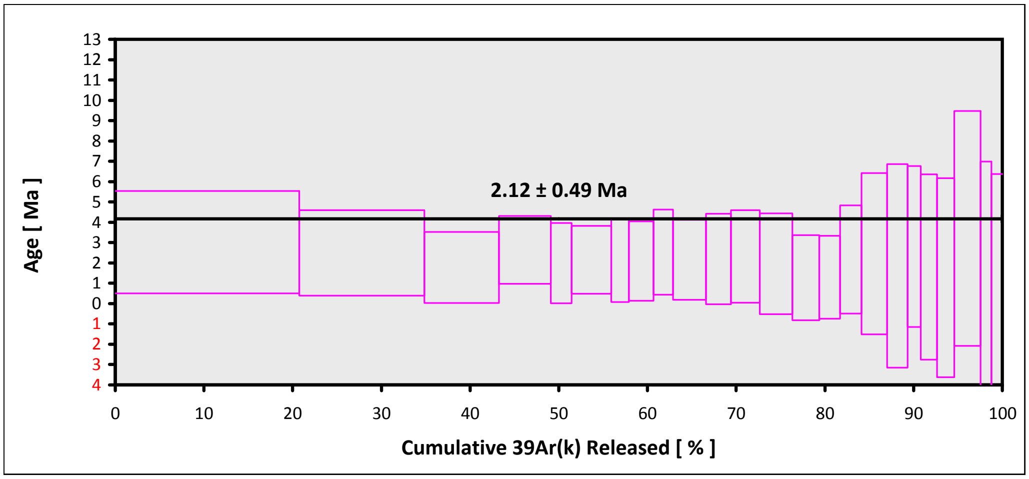

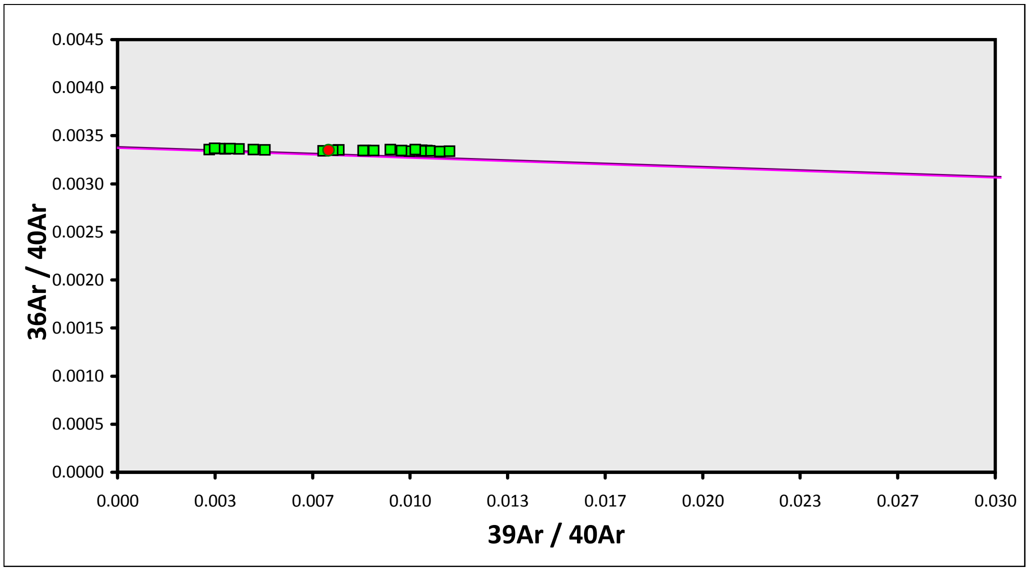

EXP#17D30134 > HLY0102-D35-1 > Groundmass > O-CONNOR (16-22)
ARTIC OCEAN > GAKKEL RIDGE
17-OSU-05 (5A29-17) > Incremental Heating > Dan Miggins

Information on Analysis and Constants Used in Calculations

Project = **O-CONNOR (16-22)**
Sample = **HLY0102-D35-1**
Material = **Groundmass**
Location = **Gakkel Ridge**
Region = **Artic Ocean**
Analyst = **Dan Miggins**
Irradiation = **17-OSU-05 (5A29-17)**
Position = **X: 0 | Y: 0 | Z/H: 42.47709 mm**
FCT-NM Age = **28.201 ± 0.023 Ma**
FCT-NM Reference = **Kuiper et al (2008)**
FCT-NM 40Ar/39Ar Ratio = **10.09639 ± 0.01030**
FCT-NM J-value = **0.00155673 ± 0.00000159**
Air Shot 40Ar/36Ar = **302.7650 ± 0.1605**
Air Shot MDF = **0.99399337 ± 0.00059333 (LIN)**
Experiment Type = **Incremental Heating**
Extraction Method = **Bulk Laser Heating**
Heating = **64 sec**
Isolation = **3.00 min**
Instrument = **ARGUS-VI-D**
Preferred Age = **Plateau Age**
Age Classification = **Crystallization Age**
IGSN = Undefined
Rock Class = **Igneous>Volcanic**
Lithology = **Basaltic Lava**
Lat-Lon = Undefined - Undefined
Age Equations = **Min et al. (2000)**
Negative Intensities = **Allowed**
Collector Calibrations = **36Ar**
Decay 40K = **5.530 ± 0.048 E-10 1/a**
Decay 39Ar = **2.940 ± 0.016 E-07 1/h**
Decay 37Ar = **8.230 ± 0.012 E-04 1/h**
Decay 36Cl = **2.257 ± 0.015 E-06 1/a**
Decay 40K(EC,β^+) = **0.580 ± 0.009 E-10 1/a**
Decay 40K(β^-) = **4.950 ± 0.043 E-10 1/a**
Atmospheric 40/36(a) = **300.25 ± 2.41**
Atmospheric 38/36(a) = **0.1869**
Production 39/37(ca) = **0.0006425 ± 0.0000059**
Production 38/37(ca) = **0.0001800 ± 0.0000173**
Production 36/37(ca) = **0.0002703 ± 0.0000005**
Production 40/39(k) = **0.000607 ± 0.000059**
Production 38/39(k) = **0.012077 ± 0.000011**
Production 36/38(cl) = **262.80 ± 1.71**
Scaling Ratio K/Ca = **0.430**
Abundance Ratio 40K/K = **1.1700 ± 0.0100 E-04**
Atomic Weight K = **39.0983 ± 0.0001 g**

Excess Initial 40Ar/36Ar = 300.25 ± 0.80 (%SD).

Results

Results	40(a)/36(a) ± 2σ		40(r)/39(k) ± 2σ		Age ± 2σ (Ma)		MSWD	39Ar(k) (%,n)	K/Ca ± 2σ
**Age Plateau**			1.11114	± 0.03227<br>± 2.90%	**3.13**	**± 0.09<br>± 2.91%**	0.80<br>63%	79.00<br>11	0.069 ± 0.014
					Full External Error	± 0.12	1.89	2σ Confidence Limit	
					Analytical Error	± 0.09	1.0000	Error Magnification	
**Total Fusion Age**			1.04576	± 0.03038<br>± 2.91%	**2.94**	**± 0.09<br>± 2.91%**		24	0.062 ± 0.000
					Full External Error	± 0.11			
					Analytical Error	± 0.09			
**Normal Isochron**<br>Error Chron	**303.74**	**± 5.26<br>± 1.73%**	1.04626	± 0.10498<br>± 10.03%	**2.94**	**± 0.30<br>± 10.03%**	4.64<br>0%	79.00<br>11	
					Full External Error	± 0.30	1.94	2σ Confidence Limit	
					Analytical Error	± 0.30	2.1538	Error Magnification	
**Inverse Isochron**<br>Error Chron	**303.82**	**± 5.26<br>± 1.73%**	1.04538	± 0.10345<br>± 9.90%	**2.94**	**± 0.29<br>± 9.89%**	4.61<br>0%	79.00<br>11	
					Full External Error	± 0.30	1.94	2σ Confidence Limit	
					Analytical Error	± 0.29	2.1475	Error Magnification	
							9%	Spreading Factor	

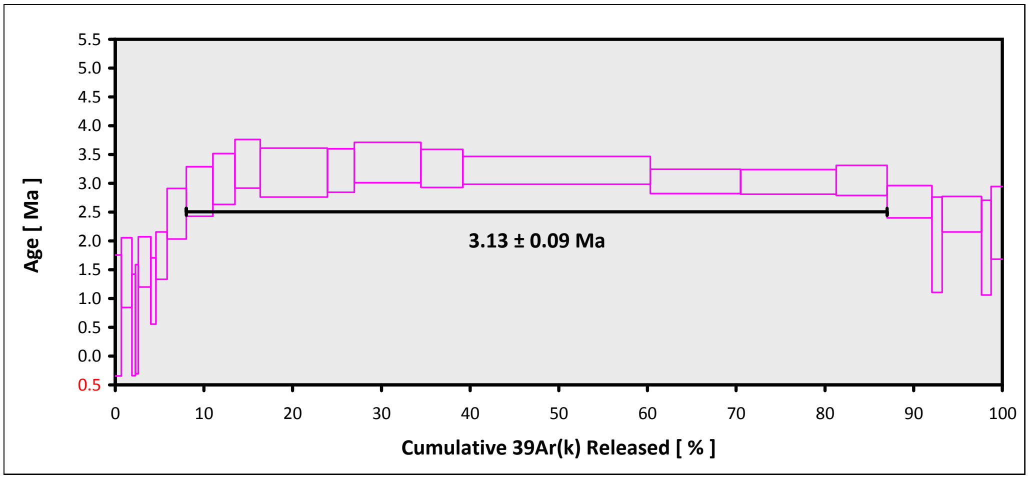

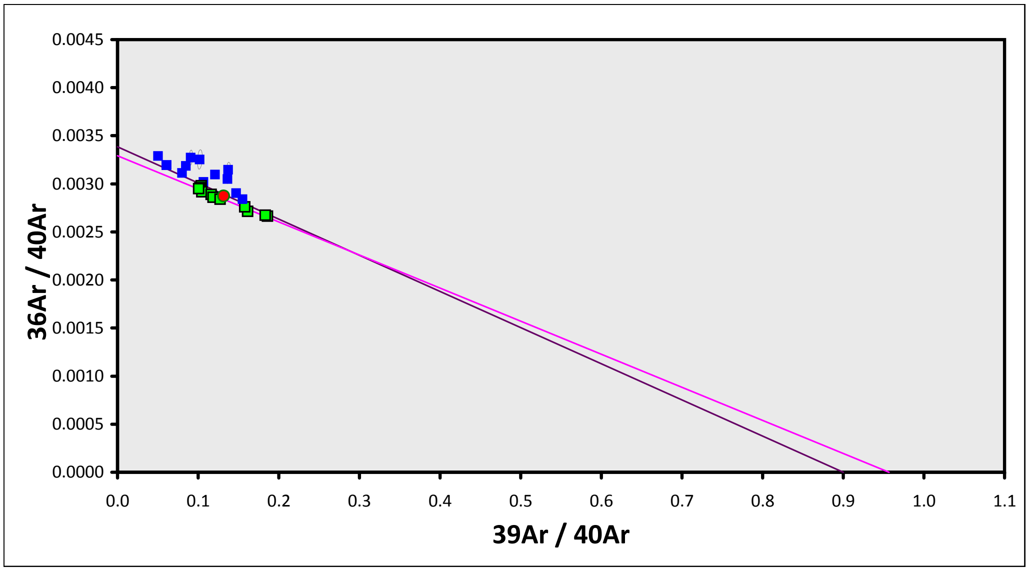

EXP#17D19948 > HLY0102-D35-12 > Groundmass > O-CONNOR (16-22)
ARTIC OCEAN > GAKKEL RIDGE
17-OSU-01 (1C32-17) > Incremental Heating > Dan Miggins

Information on Analysis and Constants Used in Calculations

Project = **O-CONNOR (16-22)**
Sample = **HLY0102-D35-12**
Material = **Groundmass**
Location = **Gakkel Ridge**
Region = **Artic Ocean**
Analyst = **Dan Miggins**
Irradiation = **17-OSU-01 (1C32-17)**
Position = **X: 0 | Y: 0 | Z/H: 49.18268 mm**
FCT-NM Age = **28.201 ± 0.023 Ma**
FCT-NM Reference = **Kuiper et al (2008)**
FCT-NM 40Ar/39Ar Ratio = **9.99322 ± 0.00849**
FCT-NM J-value = **0.00157281 ± 0.00000134**
Air Shot 40Ar/36Ar = **302.4220 ± 0.4355**
Air Shot MDF = **0.99427047 ± 0.00067830 (LIN)**
Experiment Type = **Incremental Heating**
Extraction Method = **Bulk Laser Heating**
Heating = **64 sec**
Isolation = **3.00 min**
Instrument = **ARGUS-VI-D**
Preferred Age = **Plateau Age**
Age Classification = **Crystallization Age**
IGSN = Undefined
Rock Class = **Igneous>Volcanic**
Lithology = **Basaltic Lava**
Lat-Lon = Undefined - Undefined
Age Equations = **Min et al. (2000)**
Negative Intensities = **Allowed**
Collector Calibrations = **36Ar**
Decay 40K = **5.530 ± 0.048 E-10 1/a**
Decay 39Ar = **2.940 ± 0.016 E-07 1/h**
Decay 37Ar = **8.230 ± 0.012 E-04 1/h**
Decay 36Cl = **2.257 ± 0.015 E-06 1/a**
Decay 40K(EC,β^+) = **0.580 ± 0.009 E-10 1/a**
Decay 40K(β^-) = **4.950 ± 0.043 E-10 1/a**
Atmospheric 40/36(a) = **295.50**
Atmospheric 38/36(a) = **0.1869**
Production 39/37(ca) = **0.0006425 ± 0.0000059**
Production 38/37(ca) = **0.0001800 ± 0.0000173**
Production 36/37(ca) = **0.0002703 ± 0.0000005**
Production 40/39(k) = **0.000607 ± 0.000059**
Production 38/39(k) = **0.012077 ± 0.000011**
Production 36/38(cl) = **262.80 ± 1.71**
Scaling Ratio K/Ca = **0.430**
Abundance Ratio 40K/K = **1.1700 ± 0.0100 E-04**
Atomic Weight K = **39.0983 ± 0.0001 g**

Mostly atmospheric

Results

Results	40(a)/36(a) ± 2σ		40(r)/39(k) ± 2σ		Age ± 2σ (Ma)		MSWD	39Ar(k) (%,n)	K/Ca ± 2σ
**Age Plateau**			0.45962	± 0.01014<br>± 2.21%	**1.31**	**± 0.03<br>± 2.21%**	0.89<br>56%	67.82<br>13	0.0554 ± 0.0053
				Full External Error	± 0.04		1.82	2σ Confidence Limit	
				Analytical Error	± 0.03		1.0000	Error Magnification	
**Total Fusion Age**			0.45416	± 0.01186<br>± 2.61%	**1.29**	**± 0.03<br>± 2.62%**		24	0.0441 ± 0.0001
				Full External Error	± 0.04				
				Analytical Error	± 0.03				
**Normal Isochron**	**297.24**	**± 2.52<br>± 0.85%**	0.43947	± 0.03065<br>± 6.97%	**1.25**	**± 0.09<br>± 6.97%**	0.80<br>64%	67.82<br>13	
				Full External Error	± 0.09		1.85	2σ Confidence Limit	
				Analytical Error	± 0.09		1.0000	Error Magnification	
**Inverse Isochron**	**297.25**	**± 2.52<br>± 0.85%**	0.43959	± 0.03047<br>± 6.93%	**1.25**	**± 0.09<br>± 6.93%**	0.80<br>64%	67.82<br>13	
				Full External Error	± 0.09		1.85	2σ Confidence Limit	
				Analytical Error	± 0.09		1.0000	Error Magnification	
							11%	Spreading Factor	

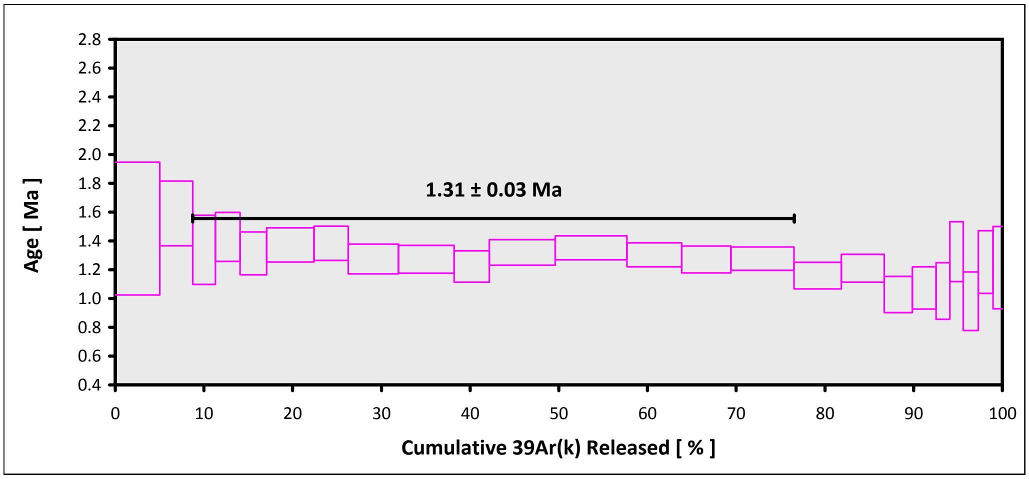

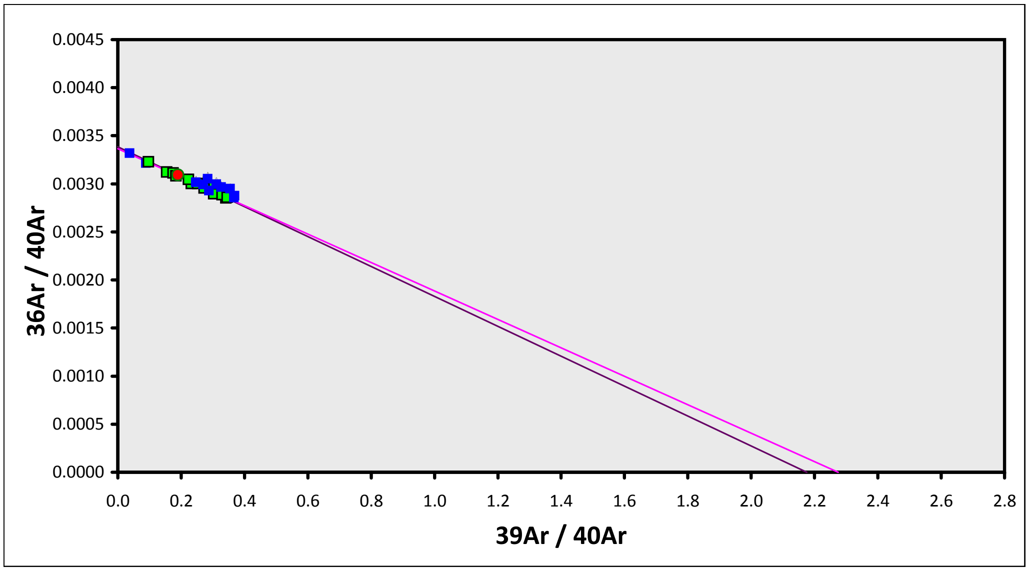

EXP#17D20015 > HLY0102-D37-8 > Groundmass > O-CONNOR (16-22)
ARTIC OCEAN > GAKKEL RIDGE
17-OSU-01 (1C41-17) > Incremental Heating > Dan Miggins

Information on Analysis and Constants Used in Calculations

Project = **O-CONNOR (16-22)**
Sample = **HLY0102-D37-8**
Material = **Groundmass**
Location = **Gakkel Ridge**
Region = **Artic Ocean**
Analyst = **Dan Miggins**
Irradiation = **17-OSU-01 (1C41-17)**
Position = **X: 0 | Y: 0 | Z/H: 62.18691 mm**
FCT-NM Age = **28.201 ± 0.023 Ma**
FCT-NM Reference = **Kuiper et al (2008)**
FCT-NM 40Ar/39Ar Ratio = **10.32999 ± 0.00847**
FCT-NM J-value = **0.00152153 ± 0.00000125**
Air Shot 40Ar/36Ar = **302.3710 ± 0.4324**
Air Shot MDF = **0.99431172 ± 0.00067715 (LIN)**
Experiment Type = **Incremental Heating**
Extraction Method = **Bulk Laser Heating**
Heating = **64 sec**
Isolation = **3.00 min**
Instrument = **ARGUS-VI-D**
Preferred Age = **Plateau Age**
Age Classification = **Crystallization Age**
IGSN = Undefined
Rock Class = **Igneous>Volcanic**
Lithology = **Basaltic Lava**
Lat-Lon = Undefined - Undefined
Age Equations = **Min et al. (2000)**
Negative Intensities = **Allowed**
Collector Calibrations = **36Ar**
Decay 40K = **5.530 ± 0.048 E-10 1/a**
Decay 39Ar = **2.940 ± 0.016 E-07 1/h**
Decay 37Ar = **8.230 ± 0.012 E-04 1/h**
Decay 36Cl = **2.257 ± 0.015 E-06 1/a**
Decay 40K(EC,β^+) = **0.580 ± 0.009 E-10 1/a**
Decay 40K(β^-) = **4.950 ± 0.043 E-10 1/a**
Atmospheric 40/36(a) = **323.83 ± 2.94**
Atmospheric 38/36(a) = **0.1869**
Production 39/37(ca) = **0.0006425 ± 0.0000059**
Production 38/37(ca) = **0.0001800 ± 0.0000173**
Production 36/37(ca) = **0.0002703 ± 0.0000005**
Production 40/39(k) = **0.000607 ± 0.000059**
Production 38/39(k) = **0.012077 ± 0.000011**
Production 36/38(cl) = **262.80 ± 1.71**
Scaling Ratio K/Ca = **0.430**
Abundance Ratio 40K/K = **1.1700 ± 0.0100 E-04**
Atomic Weight K = **39.0983 ± 0.0001 g**

Excess Initial 40Ar/36Ar = 323.83 ± 0.91 (%SD).

Results

Results	40(a)/36(a) ± 2σ		40(r)/39(k) ± 2σ		Age ± 2σ (Ma)		MSWD	39Ar(k) (%,n)	K/Ca ± 2σ
**Age Plateau**			0.79260	± 0.03093<br>± 3.90%	**2.18**	**± 0.09<br>± 3.90%**	1.32<br>20%	91.08<br>13	0.047 ± 0.011
				Full External Error		± 0.10	1.82	2σ Confidence Limit	
				Analytical Error		± 0.09	1.1476	Error Magnification	
**Total Fusion Age**			0.70766	± 0.03352<br>± 4.74%	**1.95**	**± 0.09<br>± 4.74%**		24	0.063 ± 0.000
				Full External Error		± 0.10			
				Analytical Error		± 0.09			
**Normal Isochron**<br>Error Chron	**323.92**	**± 5.79<br>± 1.79%**	0.80300	± 0.09550<br>± 11.89%	**2.21**	**± 0.26<br>± 11.89%**	7.70<br>0%	91.08<br>13	
				Full External Error		± 0.27	1.85	2σ Confidence Limit	
				Analytical Error		± 0.26	2.7741	Error Magnification	
**Inverse Isochron**<br>Error Chron	**323.83**	**± 5.88<br>± 1.81%**	0.80666	± 0.09465<br>± 11.73%	**2.22**	**± 0.26<br>± 11.73%**	7.83<br>0%	91.08<br>13	
				Full External Error		± 0.26	1.85	2σ Confidence Limit	
				Analytical Error		± 0.26	2.7979	Error Magnification	
							16%	Spreading Factor	

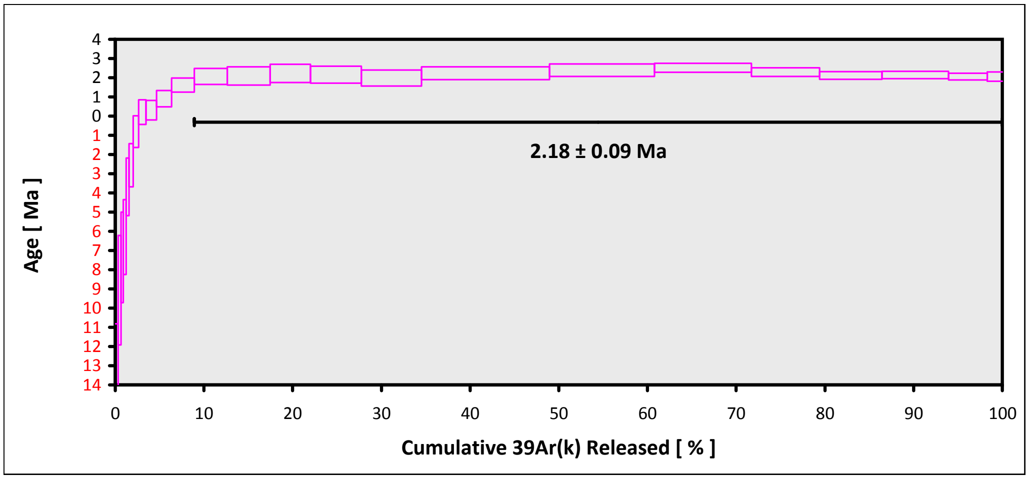

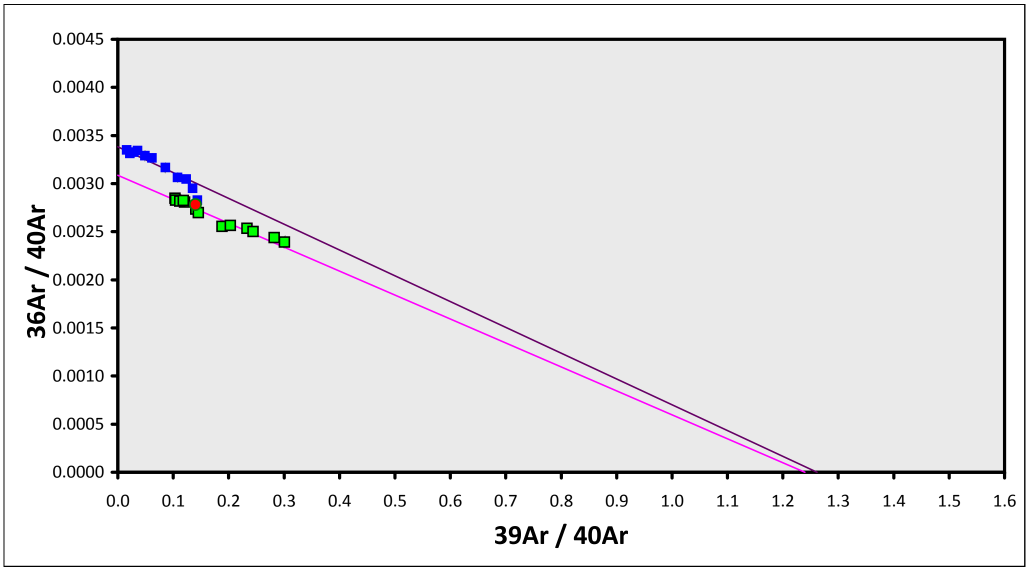

EXP#17D19641 > HLY0102-D36-1 > Groundmass > O-CONNOR (16-22)
ARTIC OCEAN > GAKKEL RIDGE
17-OSU-01 (1C5-17) > Incremental Heating > Dan Miggins

Information on Analysis and Constants Used in Calculations

Project = **O-CONNOR (16-22)**
Sample = **HLY0102-D36-1**
Material = **Groundmass**
Location = **Gakkel Ridge**
Region = **Artic Ocean**
Analyst = **Dan Miggins**
Irradiation = **17-OSU-01 (1C5-17)**
Position = **X: 0 | Y: 0 | Z/H: 8.270811 mm**
FCT-NM Age = **28.201 ± 0.023 Ma**
FCT-NM Reference = **Kuiper et al (2008)**
FCT-NM 40Ar/39Ar Ratio = **9.47435 ± 0.00853**
FCT-NM J-value = **0.00165894 ± 0.00000149**
Air Shot 40Ar/36Ar = **302.5570 ± 0.3268**
Air Shot MDF = **0.99416133 ± 0.00063675 (LIN)**
Experiment Type = **Incremental Heating**
Extraction Method = **Bulk Laser Heating**
Heating = **64 sec**
Isolation = **3.00 min**
Instrument = **ARGUS-VI-D**
Preferred Age = **Plateau Age**
Age Classification = **Crystallization Age**
IGSN = Undefined
Rock Class = **Igneous>Volcanic**
Lithology = **Basaltic Lava**
Lat-Lon = Undefined - Undefined
Age Equations = **Min et al. (2000)**
Negative Intensities = **Allowed**
Collector Calibrations = **36Ar**
Decay 40K = **5.530 ± 0.048 E-10 1/a**
Decay 39Ar = **2.940 ± 0.016 E-07 1/h**
Decay 37Ar = **8.230 ± 0.012 E-04 1/h**
Decay 36Cl = **2.257 ± 0.015 E-06 1/a**
Decay 40K(EC,β^+) = **0.580 ± 0.009 E-10 1/a**
Decay 40K(β^-) = **4.950 ± 0.043 E-10 1/a**
Atmospheric 40/36(a) = **296.41 ± 0.47**
Atmospheric 38/36(a) = **0.1869**
Production 39/37(ca) = **0.0006425 ± 0.0000059**
Production 38/37(ca) = **0.0001800 ± 0.0000173**
Production 36/37(ca) = **0.0002703 ± 0.0000005**
Production 40/39(k) = **0.000607 ± 0.000059**
Production 38/39(k) = **0.012077 ± 0.000011**
Production 36/38(cl) = **262.80 ± 1.71**
Scaling Ratio K/Ca = **0.430**
Abundance Ratio 40K/K = **1.1700 ± 0.0100 E-04**
Atomic Weight K = **39.0983 ± 0.0001 g**

Excess Initial 40Ar/36Ar = 296.41 ± 0.16 (%SD). Plateau Age essentially around zero age

Results

Results	40(a)/36(a) ± 2σ		40(r)/39(k) ± 2σ		Age ± 2σ (ka)		MSWD	39Ar(k) (%,n)	K/Ca ± 2σ
**Age Plateau**			0.01633	± 0.01145<br>± 70.14%	**49.0**	**± 34.4<br>± 70.14%**	0.71<br>84%	99.49<br>23	0.029 ± 0.009
				Full External Error		± 34.4	1.60	2σ Confidence Limit	
				Analytical Error		± 34.4	1.0000	Error Magnification	
**Total Fusion Age**			0.03168	± 0.03358<br>± 106.00%	**95.0**	**± 100.7<br>± 106.00%**		24	0.064 ± 0.000
				Full External Error		± 100.7			
				Analytical Error		± 100.7			
**Normal Isochron**	**296.75**	**± 0.80<br>± 0.27%**	0.01001	± 0.01852<br>± 185.01%	**30.0**	**± 55.6<br>± 185.01%**	0.83<br>69%	99.49<br>23	
				Full External Error		± 55.6	1.62	2σ Confidence Limit	
				Analytical Error		± 55.6	1.0000	Error Magnification	
**Inverse Isochron**<br>Clustered Points	**296.75**	**± 0.80<br>± 0.27%**	0.01023	± 0.00473<br>± 46.26%	**30.7**	**± 14.2<br>± 46.26%**	0.83<br>68%	99.49<br>23	
				Full External Error		± 14.2	1.62	2σ Confidence Limit	
				Analytical Error		± 14.2	1.0000	Error Magnification	
							0%	Spreading Factor	

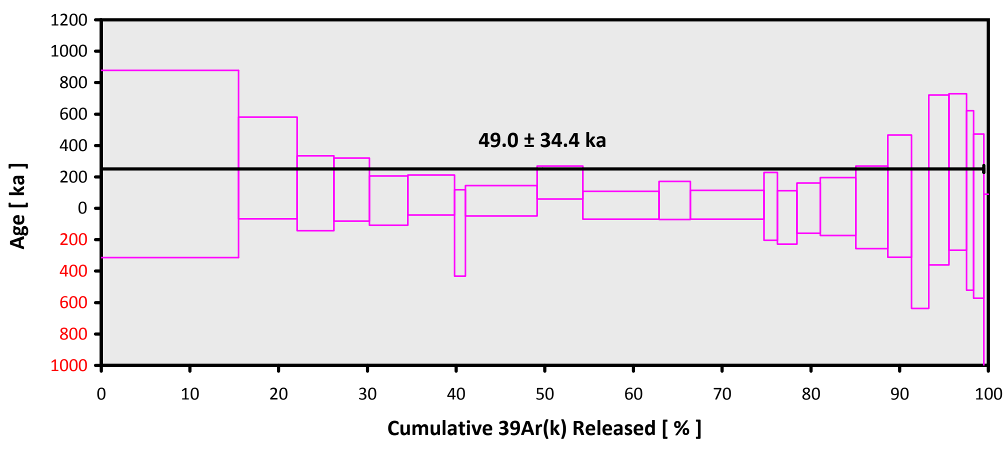

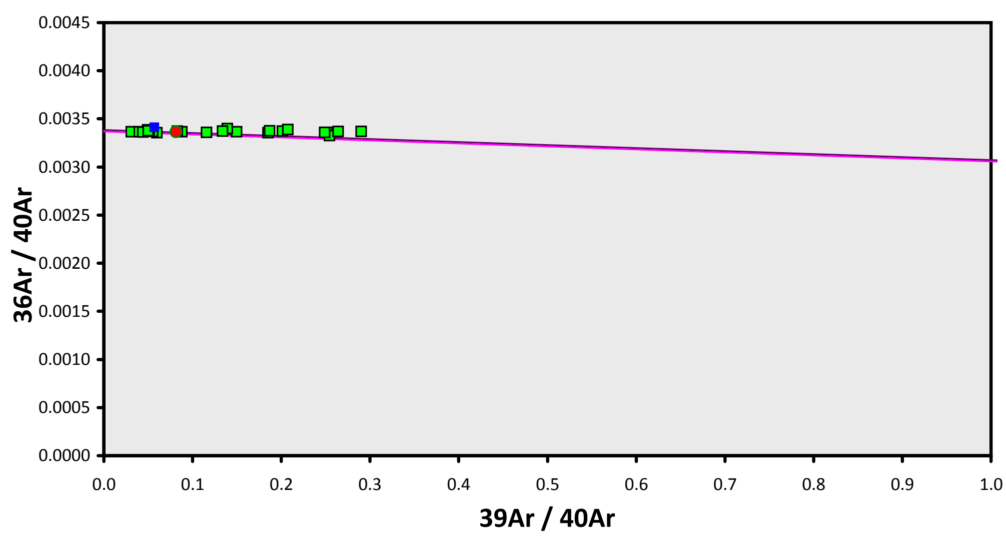

EXP#17D30553 > PS59-252-1 > Groundmass > O-CONNOR (16-22)
ARTIC OCEAN > GAKKEL RIDGE
17-OSU-05 (5A26-17) > Incremental Heating > Dan Miggins

Information on Analysis and Constants Used in Calculations

Project = **O-CONNOR (16-22)**
Sample = **PS59-252-1**
Material = **Groundmass**
Location = **Gakkel Ridge**
Region = **Artic Ocean**
Analyst = **Dan Miggins**
Irradiation = **17-OSU-05 (5A26-17)**
Position = **X: 0 | Y: 0 | Z/H: 37.93017 mm**
FCT-NM Age = **28.201 ± 0.023 Ma**
FCT-NM Reference = **Kuiper et al (2008)**
FCT-NM 40Ar/39Ar Ratio = **9.99585 ± 0.01030**
FCT-NM J-value = **0.00157239 ± 0.00000162**
Air Shot 40Ar/36Ar = **302.7790 ± 0.1665**
Air Shot MDF = **0.99398207 ± 0.00059439 (LIN)**
Experiment Type = **Incremental Heating**
Extraction Method = **Bulk Laser Heating**
Heating = **64 sec**
Isolation = **3.00 min**
Instrument = **ARGUS-VI-D**
Preferred Age = **Plateau Age**
Age Classification = **Crystallization Age**
IGSN = Undefined
Rock Class = **Igneous>Volcanic**
Lithology = **Basaltic Lava**
Lat-Lon = Undefined - Undefined
Age Equations = **Min et al. (2000)**
Negative Intensities = **Allowed**
Collector Calibrations = **36Ar**
Decay 40K = **5.530 ± 0.048 E-10 1/a**
Decay 39Ar = **2.940 ± 0.016 E-07 1/h**
Decay 37Ar = **8.230 ± 0.012 E-04 1/h**
Decay 36Cl = **2.257 ± 0.015 E-06 1/a**
Decay 40K(EC,β^+) = **0.580 ± 0.009 E-10 1/a**
Decay 40K(β^-) = **4.950 ± 0.043 E-10 1/a**
Atmospheric 40/36(a) = **295.50 ± 0.70**
Atmospheric 38/36(a) = **0.1869**
Production 39/37(ca) = **0.0006425 ± 0.0000059**
Production 38/37(ca) = **0.0001800 ± 0.0000173**
Production 36/37(ca) = **0.0002703 ± 0.0000005**
Production 40/39(k) = **0.000607 ± 0.000059**
Production 38/39(k) = **0.012077 ± 0.000011**
Production 36/38(cl) = **262.80 ± 1.71**
Scaling Ratio K/Ca = **0.430**
Abundance Ratio 40K/K = **1.1700 ± 0.0100 E-04**
Atomic Weight K = **39.0983 ± 0.0001 g**

Results

Results	40(a)/36(a) ± 2σ	40(r)/39(k) ± 2σ	Age ± 2σ (ka)	MSWD	39Ar(k) (%,n)	K/Ca ± 2σ
**Age Plateau**		0.16668 ± 0.01042 ± 6.25%	**473.9 ± 29.6 ± 6.25%**	0.80 / 72%	83.02 / 21	0.0213 ± 0.0078
			Full External Error ± 31.5	1.63	2σ Confidence Limit	
			Analytical Error ± 29.6	1.0000	Error Magnification	
**Total Fusion Age**		0.17926 ± 0.01567 ± 8.74%	**509.6 ± 44.6 ± 8.74%**		24	0.0510 ± 0.0001
			Full External Error ± 46.0			
			Analytical Error ± 44.5			
**Normal Isochron**	**294.53 ± 0.95 ± 0.32%**	0.17962 ± 0.01559 ± 8.68%	**510.7 ± 44.3 ± 8.68%**	0.87 / 62%	83.02 / 21	
			Full External Error ± 45.8	1.65	2σ Confidence Limit	
			Analytical Error ± 44.3	1.0000	Error Magnification	
**Inverse Isochron**	**294.54 ± 0.95 ± 0.32%**	0.17980 ± 0.01532 ± 8.52%	**511.2 ± 43.6 ± 8.52%**	0.87 / 63%	83.02 / 21	
			Full External Error ± 45.1	1.65	2σ Confidence Limit	
			Analytical Error ± 43.6	1.0000	Error Magnification	
				6%	Spreading Factor	

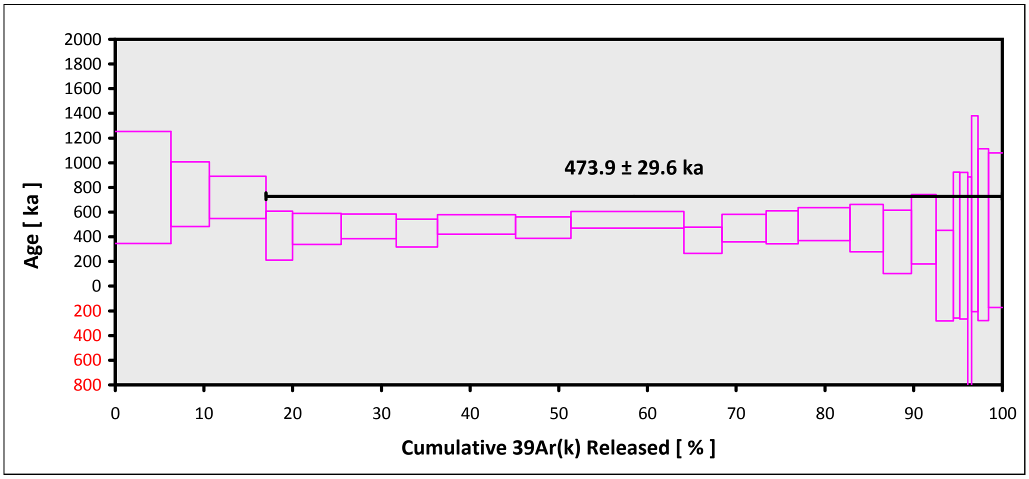

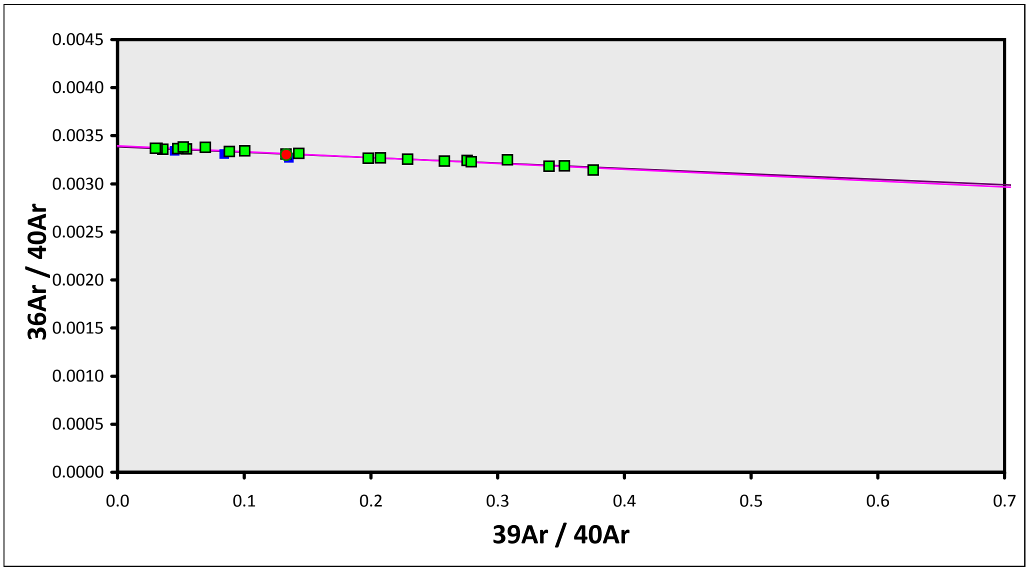

EXP#17D19681 > PS59-312-11 > Groundmass > O-CONNOR (16-22)
ARTIC OCEAN > GAKKEL RIDGE
17-OSU-01 (1C14-17) > Incremental Heating > Dan Miggins

Information on Analysis and Constants Used in Calculations

Project = **O-CONNOR (16-22)**
Sample = **PS59-312-11**
Material = **Groundmass**
Location = **Gakkel Ridge**
Region = **Artic Ocean**
Analyst = **Dan Miggins**
Irradiation = **17-OSU-01 (1C14-17)**
Position = **X: 0 | Y: 0 | Z/H: 22.04658 mm**
FCT-NM Age = **28.201 ± 0.023 Ma**
FCT-NM Reference = **Kuiper et al (2008)**
FCT-NM 40Ar/39Ar Ratio = **9.55759 ± 0.00851**
FCT-NM J-value = **0.00164449 ± 0.00000146**
Air Shot 40Ar/36Ar = **302.5400 ± 0.3328**
Air Shot MDF = **0.99417507 ± 0.00063883 (LIN)**
Experiment Type = **Incremental Heating**
Extraction Method = **Bulk Laser Heating**
Heating = **64 sec**
Isolation = **3.00 min**
Instrument = **ARGUS-VI-D**
Preferred Age = **Plateau Age**
Age Classification = **Crystallization Age**
IGSN = Undefined
Rock Class = **Igneous>Volcanic**
Lithology = **Basaltic Lava**
Lat-Lon = Undefined - Undefined
Age Equations = **Min et al. (2000)**
Negative Intensities = **Allowed**
Collector Calibrations = **36Ar**
Decay 40K = **5.530 ± 0.048 E-10 1/a**
Decay 39Ar = **2.940 ± 0.016 E-07 1/h**
Decay 37Ar = **8.230 ± 0.012 E-04 1/h**
Decay 36Cl = **2.257 ± 0.015 E-06 1/a**
Decay 40K(EC,β^+) = **0.580 ± 0.009 E-10 1/a**
Decay 40K(β^-) = **4.950 ± 0.043 E-10 1/a**
Atmospheric 40/36(a) = **295.50**
Atmospheric 38/36(a) = **0.1869**
Production 39/37(ca) = **0.0006425 ± 0.0000059**
Production 38/37(ca) = **0.0001800 ± 0.0000173**
Production 36/37(ca) = **0.0002703 ± 0.0000005**
Production 40/39(k) = **0.000607 ± 0.000059**
Production 38/39(k) = **0.012077 ± 0.000011**
Production 36/38(cl) = **262.80 ± 1.71**
Scaling Ratio K/Ca = **0.430**
Abundance Ratio 40K/K = **1.1700 ± 0.0100 E-04**
Atomic Weight K = **39.0983 ± 0.0001 g**

Nice plateau

Results

Results	40(a)/36(a) ± 2σ		40(r)/39(k) ± 2σ		Age ± 2σ (Ma)		MSWD	39Ar(k) (%,n)	K/Ca ± 2σ
**Age Plateau**			0.56425	± 0.01516<br>± 2.69%	**1.68**	**± 0.05<br>± 2.69%**	1.69<br>6%	75.62<br>13	0.0244 ± 0.0083
				Full External Error	± 0.06		1.82	2σ Confidence Limit	
				Analytical Error	± 0.05		1.2995	Error Magnification	
**Total Fusion Age**			0.50887	± 0.01371<br>± 2.69%	**1.51**	**± 0.04<br>± 2.70%**		24	0.0318 ± 0.0001
				Full External Error	± 0.05				
				Analytical Error	± 0.04				
**Normal Isochron**	**289.29**	**± 11.85<br>± 4.10%**	0.65613	± 0.17659<br>± 26.91%	**1.95**	**± 0.52<br>± 26.90%**	1.69<br>7%	75.62<br>13	
				Full External Error	± 0.53		1.85	2σ Confidence Limit	
				Analytical Error	± 0.52		1.3009	Error Magnification	
**Inverse Isochron**<br>Clustered Points	**289.38**	**± 11.82<br>± 4.08%**	0.65517	± 0.15692<br>± 23.95%	**1.95**	**± 0.47<br>± 23.94%**	1.68<br>7%	75.62<br>13	
				Full External Error	± 0.47		1.85	2σ Confidence Limit	
				Analytical Error	± 0.47		1.2971	Error Magnification	
							3%	Spreading Factor	

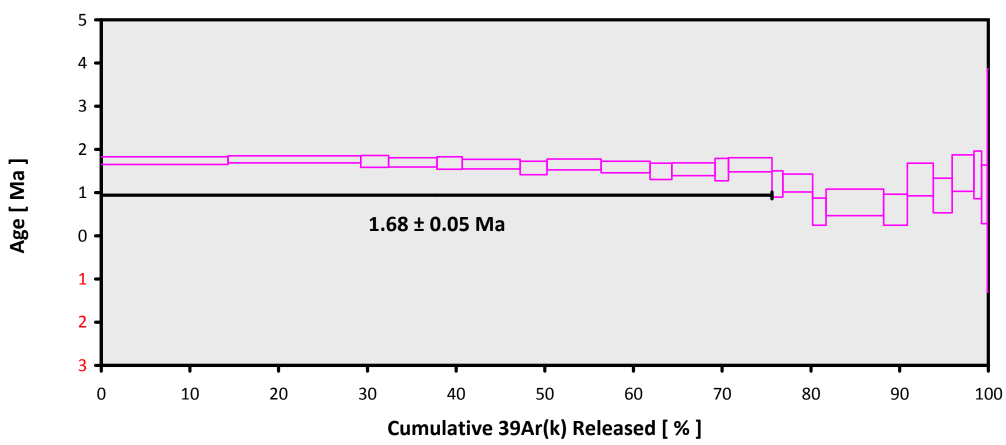

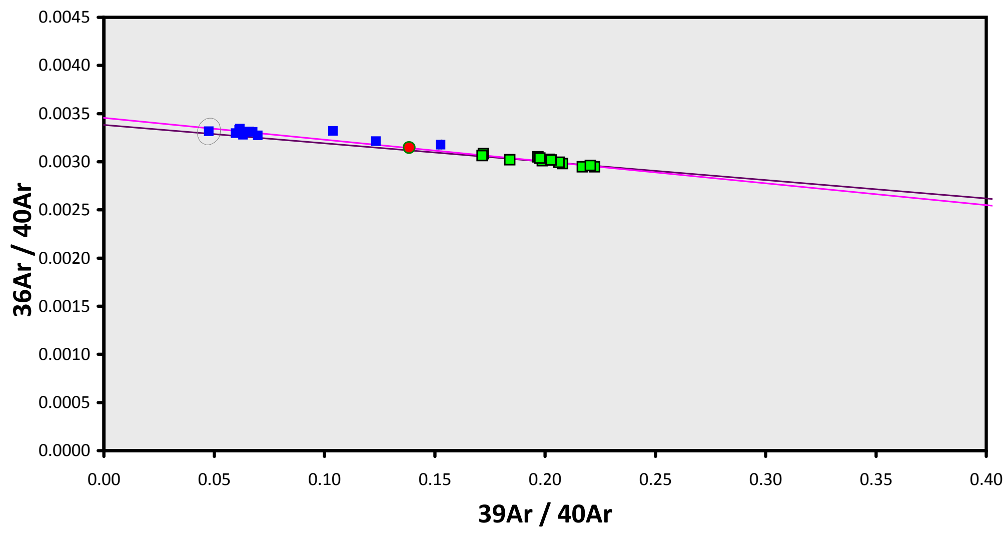

EXP#17D19868 > PS59-311-1 > Groundmass > O-CONNOR (16-22) ARTIC OCEAN > GAKKEL RIDGE 17-OSU-01 (1C8-17) > Incremental Heating > Dan Miggins

Information on Analysis and Constants Used in Calculations

Project = **O-CONNOR (16-22)**
Sample = **PS59-311-1**
Material = **Groundmass**
Location = **Gakkel Ridge**
Region = **Artic Ocean**
Analyst = **Dan Miggins**
Irradiation = **17-OSU-01 (1C8-17)**
Position = **X: 0 | Y: 0 | Z/H: 13.0264 mm**
FCT-NM Age = **28.201 ± 0.023 Ma**
FCT-NM Reference = **Kuiper et al (2008)**
FCT-NM 40Ar/39Ar Ratio = **9.49259 ± 0.00854**
FCT-NM J-value = **0.00165575 ± 0.00000149**
Air Shot 40Ar/36Ar = **302.4950 ± 0.4174**
Air Shot MDF = **0.99421144 ± 0.00067064 (LIN)**
Experiment Type = **Incremental Heating**
Extraction Method = **Bulk Laser Heating**
Heating = **64 sec**
Isolation = **3.00 min**
Instrument = **ARGUS-VI-D**
Preferred Age = **Plateau Age**
Age Classification = **Crystallization Age**
IGSN = Undefined
Rock Class = **Igneous>Volcanic**
Lithology = **Basaltic Lava**
Lat-Lon = Undefined - Undefined
Age Equations = **Min et al. (2000)**
Negative Intensities = **Allowed**
Collector Calibrations = **36Ar**
Decay 40K = **5.530 ± 0.048 E-10 1/a**
Decay 39Ar = **2.940 ± 0.016 E-07 1/h**
Decay 37Ar = **8.230 ± 0.012 E-04 1/h**
Decay 36Cl = **2.257 ± 0.015 E-06 1/a**
Decay 40K(EC,β^+) = **0.580 ± 0.009 E-10 1/a**
Decay 40K(β^-) = **4.950 ± 0.043 E-10 1/a**
Atmospheric 40/36(a) = **295.50**
Atmospheric 38/36(a) = **0.1869**
Production 39/37(ca) = **0.0006425 ± 0.0000059**
Production 38/37(ca) = **0.0001800 ± 0.0000173**
Production 36/37(ca) = **0.0002703 ± 0.0000005**
Production 40/39(k) = **0.000607 ± 0.000059**
Production 38/39(k) = **0.012077 ± 0.000011**
Production 36/38(cl) = **262.80 ± 1.71**
Scaling Ratio K/Ca = **0.430**
Abundance Ratio 40K/K = **1.1700 ± 0.0100 E-04**
Atomic Weight K = **39.0983 ± 0.0001 g**

Mostly atmospheric

Results

Results	40(a)/36(a) ± 2σ		40(r)/39(k) ± 2σ		Age ± 2σ (ka)		MSWD	39Ar(k) (%,n)	K/Ca ± 2σ
**Age Plateau**			0.22092	± 0.02266	**661.3**	**± 67.8**	2.40	96.09	0.0155 ± 0.0036
Error Mean				± 10.26%		**± 10.26%**	0%	20	
					Full External Error	± 69.5	1.65	2σ Confidence Limit	
					Analytical Error	± 67.8	1.5478	Error Magnification	
**Total Fusion Age**			0.22748	± 0.02223	**681.0**	**± 66.5**		24	0.0250 ± 0.0001
				± 9.77%		**± 9.77%**			
					Full External Error	± 68.3			
					Analytical Error	± 66.5			
**Normal Isochron**	**296.47**	**± 2.65**	0.20194	± 0.05366	**604.5**	**± 160.6**	2.48	96.09	
Error Chron		**± 0.90%**		± 26.57%		**± 26.57%**	0%	20	
					Full External Error	± 161.2	1.67	2σ Confidence Limit	
					Analytical Error	± 160.6	1.5736	Error Magnification	
**Inverse Isochron**	**296.48**	**± 2.65**	0.20304	± 0.04636	**607.8**	**± 138.8**	2.45	96.09	
Error Chron		**± 0.89%**		± 22.83%		**± 22.83%**	0%	20	
					Full External Error	± 139.4	1.67	2σ Confidence Limit	
					Analytical Error	± 138.8	1.5662	Error Magnification	
							4%	Spreading Factor	

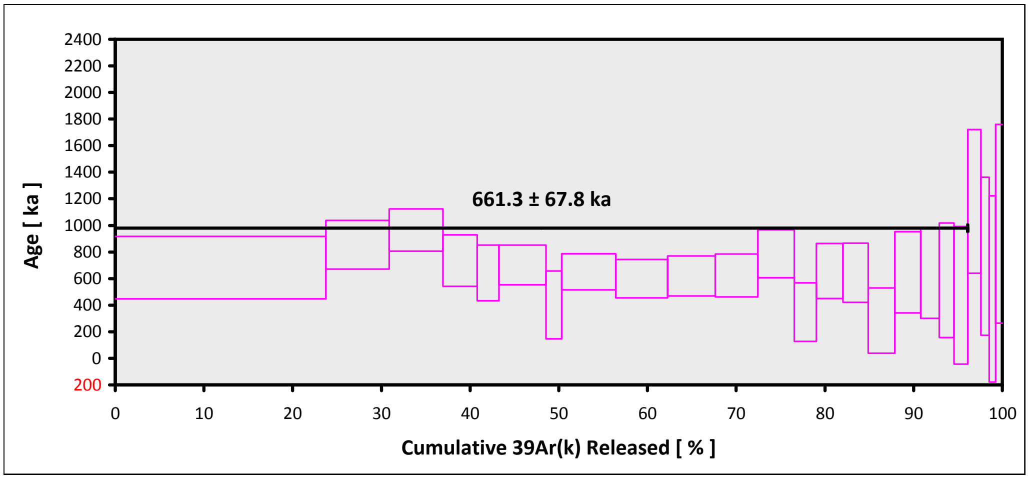

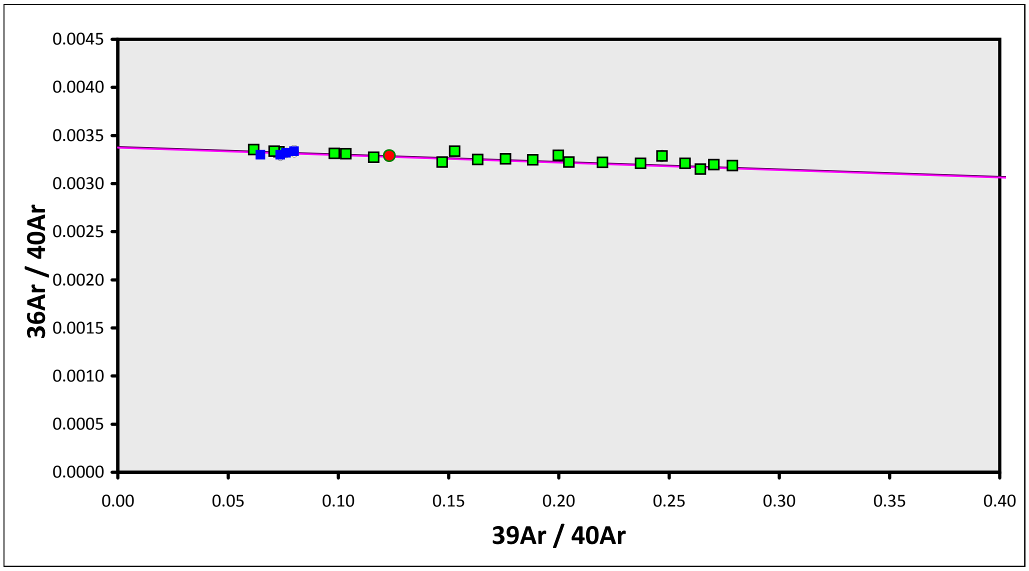

EXP#17D20055 > PS59-310-1-1 > Groundmass > O-CONNOR (16-22)
ARTIC OCEAN > GAKKEL RIDGE
17-OSU-01 (1C38-17) > Incremental Heating > Dan Miggins

Information on Analysis and Constants Used in Calculations

Project = **O-CONNOR (16-22)**
Sample = **PS59-310-1-1**
Material = **Groundmass**
Location = **Gakkel Ridge**
Region = **Artic Ocean**
Analyst = **Dan Miggins**
Irradiation = **17-OSU-01 (1C38-17)**
Position = **X: 0 | Y: 0 | Z/H: 57.84776 mm**
FCT-NM Age = **28.201 ± 0.023 Ma**
FCT-NM Reference = **Kuiper et al (2008)**
FCT-NM 40Ar/39Ar Ratio = **10.20843 ± 0.00847**
FCT-NM J-value = **0.00153965 ± 0.00000128**
Air Shot 40Ar/36Ar = **302.6230 ± 0.3510**
Air Shot MDF = **0.99410801 ± 0.00064497 (LIN)**
Experiment Type = **Incremental Heating**
Extraction Method = **Bulk Laser Heating**
Heating = **64 sec**
Isolation = **3.00 min**
Instrument = **ARGUS-VI-D**
Preferred Age = **Plateau Age**
Age Classification = **Crystallization Age**
IGSN = Undefined
Rock Class = **Igneous>Volcanic**
Lithology = **Basaltic Lava**
Lat-Lon = Undefined - Undefined
Age Equations = **Min et al. (2000)**
Negative Intensities = **Allowed**
Collector Calibrations = **36Ar**
Decay 40K = **5.530 ± 0.048 E-10 1/a**
Decay 39Ar = **2.940 ± 0.016 E-07 1/h**
Decay 37Ar = **8.230 ± 0.012 E-04 1/h**
Decay 36Cl = **2.257 ± 0.015 E-06 1/a**
Decay 40K(EC,β^+) = **0.580 ± 0.009 E-10 1/a**
Decay 40K(β^-) = **4.950 ± 0.043 E-10 1/a**
Atmospheric 40/36(a) = **296.53 ± 0.42**
Atmospheric 38/36(a) = **0.1869**
Production 39/37(ca) = **0.0006425 ± 0.0000059**
Production 38/37(ca) = **0.0001800 ± 0.0000173**
Production 36/37(ca) = **0.0002703 ± 0.0000005**
Production 40/39(k) = **0.000607 ± 0.000059**
Production 38/39(k) = **0.012077 ± 0.000011**
Production 36/38(cl) = **262.80 ± 1.71**
Scaling Ratio K/Ca = **0.430**
Abundance Ratio 40K/K = **1.1700 ± 0.0100 E-04**
Atomic Weight K = **39.0983 ± 0.0001 g**

Excess Initial 40Ar/36Ar = 296.53 ± 0.14 (%SD).

Results

Results	40(a)/36(a) ± 2σ		40(r)/39(k) ± 2σ		Age ± 2σ (ka)		MSWD	39Ar(k) (%,n)	K/Ca ± 2σ
**Age Plateau**			0.10590	± 0.01895<br>± 17.89%	**294.8**	**± 52.8**<br>**± 17.89%**	0.81<br>72%	100.00<br>24	0.030 ± 0.006
				Full External Error	± 53.2		1.59	2σ Confidence Limit	
				Analytical Error	± 52.7		1.0000	Error Magnification	
**Total Fusion Age**			0.11607	± 0.05486<br>± 47.26%	**323.1**	**± 152.7**<br>**± 47.26%**		24	0.063 ± 0.000
				Full External Error	± 152.9				
				Analytical Error	± 152.7				
**Normal Isochron**	**296.53**	**± 0.84**<br>**± 0.28%**	0.10506	± 0.03074<br>± 29.26%	**292.5**	**± 85.6**<br>**± 29.26%**	0.94<br>54%	100.00<br>24	
				Full External Error	± 85.8		1.60	2σ Confidence Limit	
				Analytical Error	± 85.6		1.0000	Error Magnification	
**Inverse Isochron**<br>Clustered Points	**296.53**	**± 0.84**<br>**± 0.28%**	0.10557	± 0.02556<br>± 24.21%	**293.9**	**± 71.1**<br>**± 24.21%**	0.94<br>54%	100.00<br>24	
				Full External Error	± 71.5		1.60	2σ Confidence Limit	
				Analytical Error	± 71.1		1.0000	Error Magnification	
							2%	Spreading Factor	

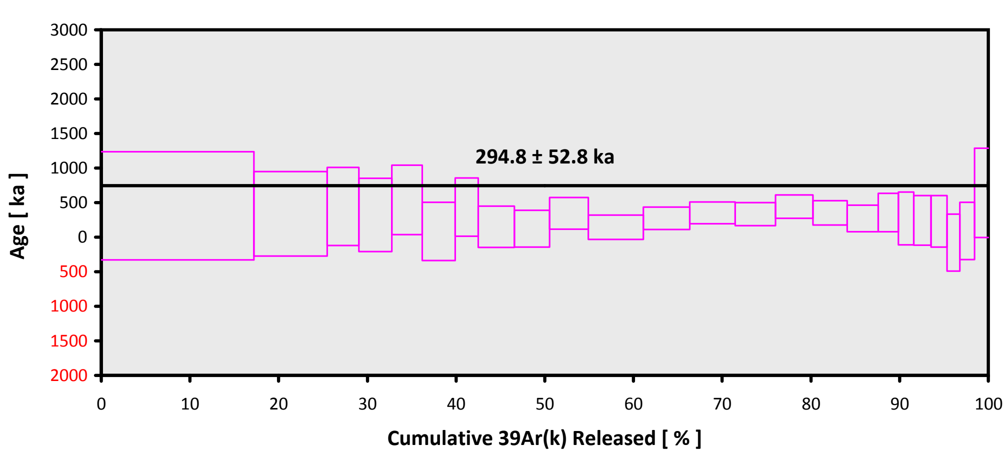

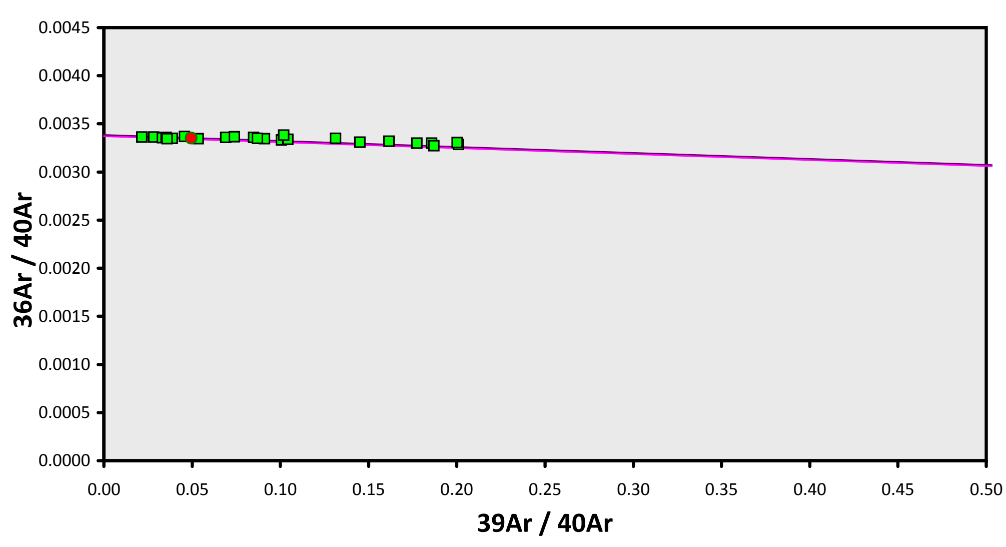

EXP#18D00132 > HLY0102-D95-38 > Groundmass > O-CONNOR (16-22)
ARTIC OCEAN > GAKKEL RIDGE
17-OSU-05 (5B20-17) > Incremental Heating > Dan Miggins

Information on Analysis and Constants Used in Calculations

Project = **O-CONNOR (16-22)**
Sample = **HLY0102-D95-38**
Material = **Groundmass**
Location = **Gakkel Ridge**
Region = **Artic Ocean**
Analyst = **Dan Miggins**
Irradiation = **17-OSU-05 (5B20-17)**
Position = **X: 0 | Y: 0 | Z/H: 29.19945 mm**
FCT-NM Age = **28.201 ± 0.023 Ma**
FCT-NM Reference = **Kuiper et al (2008)**
FCT-NM 40Ar/39Ar Ratio = **9.82234 ± 0.00796**
FCT-NM J-value = **0.00160017 ± 0.00000130**
Air Shot 40Ar/36Ar = **302.9270 ± 0.3968**
Air Shot MDF = **0.99386271 ± 0.00066124 (LIN)**
Experiment Type = **Incremental Heating**
Extraction Method = **Bulk Laser Heating**
Heating = **64 sec**
Isolation = **3.00 min**
Instrument = **ARGUS-VI-D**
Preferred Age = **Plateau Age**
Age Classification = **Crystallization Age**
IGSN = Undefined
Rock Class = **Igneous>Volcanic**
Lithology = **Basaltic Lava**
Lat-Lon = Undefined - Undefined
Age Equations = **Min et al. (2000)**
Negative Intensities = **Allowed**
Collector Calibrations = **36Ar**
Decay 40K = **5.530 ± 0.048 E-10 1/a**
Decay 39Ar = **2.940 ± 0.016 E-07 1/h**
Decay 37Ar = **8.230 ± 0.012 E-04 1/h**
Decay 36Cl = **2.257 ± 0.015 E-06 1/a**
Decay 40K(EC,β^+) = **0.580 ± 0.009 E-10 1/a**
Decay 40K(β^-) = **4.950 ± 0.043 E-10 1/a**
Atmospheric 40/36(a) = **297.99 ± 1.14**
Atmospheric 38/36(a) = **0.1869**
Production 39/37(ca) = **0.0006425 ± 0.0000059**
Production 38/37(ca) = **0.0001800 ± 0.0000173**
Production 36/37(ca) = **0.0002703 ± 0.0000005**
Production 40/39(k) = **0.000607 ± 0.000059**
Production 38/39(k) = **0.012077 ± 0.000011**
Production 36/38(cl) = **262.80 ± 1.71**
Scaling Ratio K/Ca = **0.430**
Abundance Ratio 40K/K = **1.1700 ± 0.0100 E-04**
Atomic Weight K = **39.0983 ± 0.0001 g**

Excess Initial 40Ar/36Ar = 297.99 ± 0.38 (%SD).

Results

Results	40(a)/36(a) ± 2σ		40(r)/39(k) ± 2σ		Age ± 2σ (Ma)		MSWD	39Ar(k) (%,n)	K/Ca ± 2σ
**Age Plateau**			0.58596	± 0.02844<br>± 4.85%	**1.69**	**± 0.08<br>± 4.85%**	0.90<br>55%	79.02<br>13	0.0320 ± 0.0056
				Full External Error	± 0.09		1.82	2σ Confidence Limit	
				Analytical Error	± 0.08		1.0000	Error Magnification	
**Total Fusion Age**			0.52712	± 0.03840<br>± 7.28%	**1.52**	**± 0.11<br>± 7.28%**		24	0.0396 ± 0.0004
				Full External Error	± 0.12				
				Analytical Error	± 0.11				
**Normal Isochron**	**297.95**	**± 2.26<br>± 0.76%**	0.58581	± 0.06160<br>± 10.52%	**1.69**	**± 0.18<br>± 10.51%**	1.66<br>8%	79.02<br>13	
				Full External Error	± 0.18		1.85	2σ Confidence Limit	
				Analytical Error	± 0.18		1.2886	Error Magnification	
**Inverse Isochron**	**297.99**	**± 2.27<br>± 0.76%**	0.58585	± 0.06083<br>± 10.38%	**1.69**	**± 0.18<br>± 10.38%**	1.66<br>7%	79.02<br>13	
				Full External Error	± 0.18		1.85	2σ Confidence Limit	
				Analytical Error	± 0.18		1.2896	Error Magnification	
							12%	Spreading Factor	

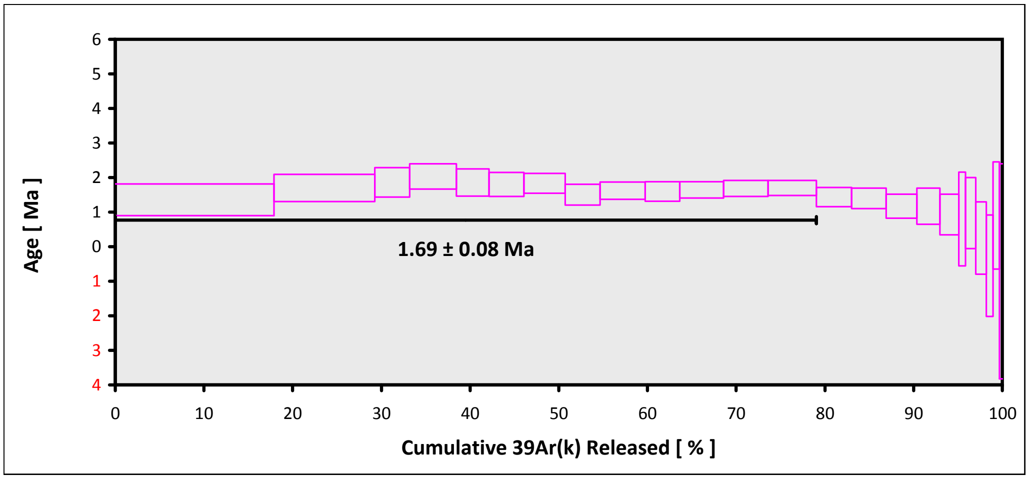

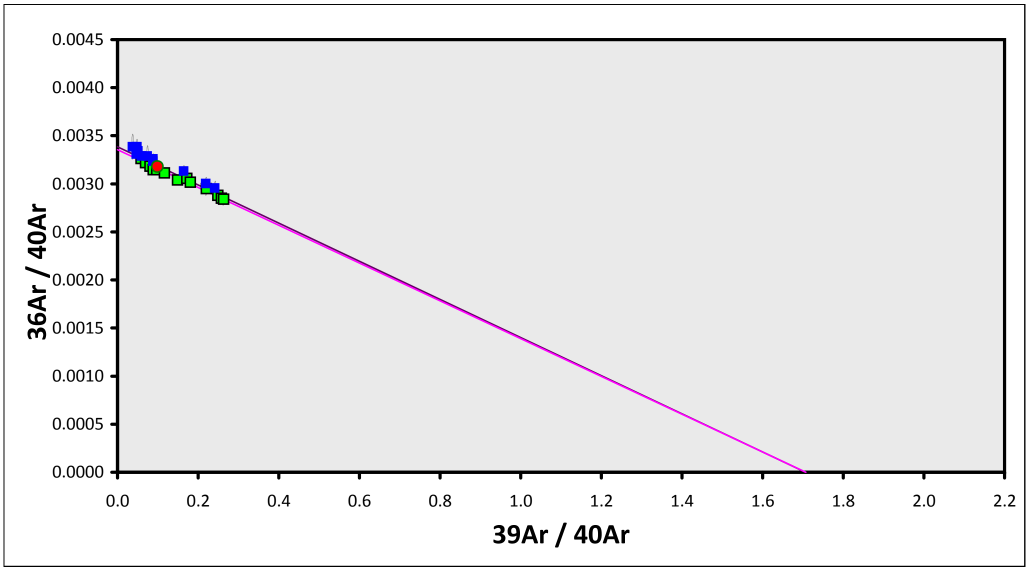

EXP#18D00504 > PS59-309-39 > Groundmass > O-CONNOR (16-22)
ARTIC OCEAN > GAKKEL RIDGE
17-OSU-05 (5B12-17) > Incremental Heating > Dan Miggins

Information on Analysis and Constants Used in Calculations

Project = **O-CONNOR (16-22)**
Sample = **PS59-309-39**
Material = **Groundmass**
Location = **Gakkel Ridge**
Region = **Artic Ocean**
Analyst = **Dan Miggins**
Irradiation = **17-OSU-05 (5B12-17)**
Position = **X: 0 | Y: 0 | Z/H: 18.17182 mm**
FCT-NM Age = **28.201 ± 0.023 Ma**
FCT-NM Reference = **Kuiper et al (2008)**
FCT-NM 40Ar/39Ar Ratio = **9.67023 ± 0.00793**
FCT-NM J-value = **0.00162534 ± 0.00000133**
Air Shot 40Ar/36Ar = **302.3400 ± 0.4686**
Air Shot MDF = **0.99433681 ± 0.00069284 (LIN)**
Experiment Type = **Incremental Heating**
Extraction Method = **Bulk Laser Heating**
Heating = **77 sec**
Isolation = **3.00 min**
Instrument = **ARGUS-VI-D**
Preferred Age = **Plateau Age**
Age Classification = **Crystallization Age**
IGSN = Undefined
Rock Class = Undefined
Lithology = **Basaltic Lava**
Lat-Lon = Undefined - Undefined
Age Equations = **Min et al. (2000)**
Negative Intensities = **Allowed**
Collector Calibrations = **36Ar**
Decay 40K = **5.530 ± 0.048 E-10 1/a**
Decay 39Ar = **2.940 ± 0.016 E-07 1/h**
Decay 37Ar = **8.230 ± 0.012 E-04 1/h**
Decay 36Cl = **2.257 ± 0.015 E-06 1/a**
Decay 40K(EC,β^+) = **0.580 ± 0.009 E-10 1/a**
Decay 40K(β^-) = **4.950 ± 0.043 E-10 1/a**
Atmospheric 40/36(a) = **301.16 ± 0.66**
Atmospheric 38/36(a) = **0.1869**
Production 39/37(ca) = **0.0006425 ± 0.0000059**
Production 38/37(ca) = **0.0001800 ± 0.0000173**
Production 36/37(ca) = **0.0002703 ± 0.0000005**
Production 40/39(k) = **0.000607 ± 0.000059**
Production 38/39(k) = **0.012077 ± 0.000011**
Production 36/38(cl) = **262.80 ± 1.71**
Scaling Ratio K/Ca = **0.430**
Abundance Ratio 40K/K = **1.1700 ± 0.0100 E-04**
Atomic Weight K = **39.0983 ± 0.0001 g**

Excess Initial 40Ar/36Ar = 301.16 ± 0.22 (%SD). Mostly atmospheric

Results

Results	40(a)/36(a) ± 2σ		40(r)/39(k) ± 2σ		Age ± 2σ (ka)		MSWD	39Ar(k) (%,n)	K/Ca ± 2σ
**Age Plateau**			0.20074	± 0.03955<br>± 19.70%	**589.9**	**± 116.2**<br>**± 19.70%**	0.86<br>59%	69.91<br>13	0.0175 ± 0.0018
				Full External Error	± 117.0		1.82	2σ Confidence Limit	
				Analytical Error	± 116.2		1.0000	Error Magnification	
**Total Fusion Age**			0.11030	± 0.05969<br>± 54.11%	**324.2**	**± 175.4**<br>**± 54.11%**		24	0.0178 ± 0.0002
				Full External Error	± 175.5				
				Analytical Error	± 175.4				
**Normal Isochron**	**302.14**	**± 1.18**<br>**± 0.39%**	0.16659	± 0.05728<br>± 34.38%	**489.6**	**± 168.3**<br>**± 34.38%**	0.88<br>56%	69.91<br>13	
				Full External Error	± 168.7		1.85	2σ Confidence Limit	
				Analytical Error	± 168.3		1.0000	Error Magnification	
**Inverse Isochron**<br>**Clustered Points**	**302.15**	**± 1.18**<br>**± 0.39%**	0.16683	± 0.05059<br>± 30.32%	**490.3**	**± 148.6**<br>**± 30.32%**	0.87<br>57%	69.91<br>13	
				Full External Error	± 149.1		1.85	2σ Confidence Limit	
				Analytical Error	± 148.6		1.0000	Error Magnification	
							3%	Spreading Factor	

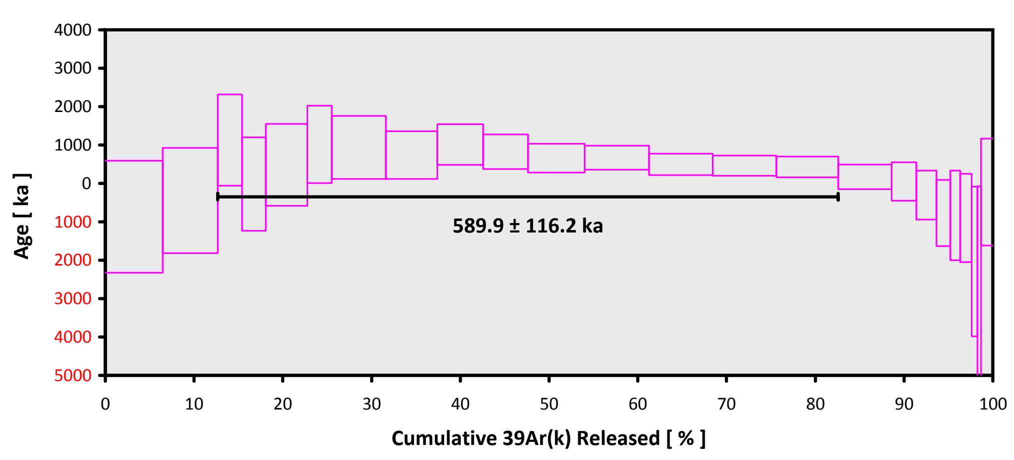

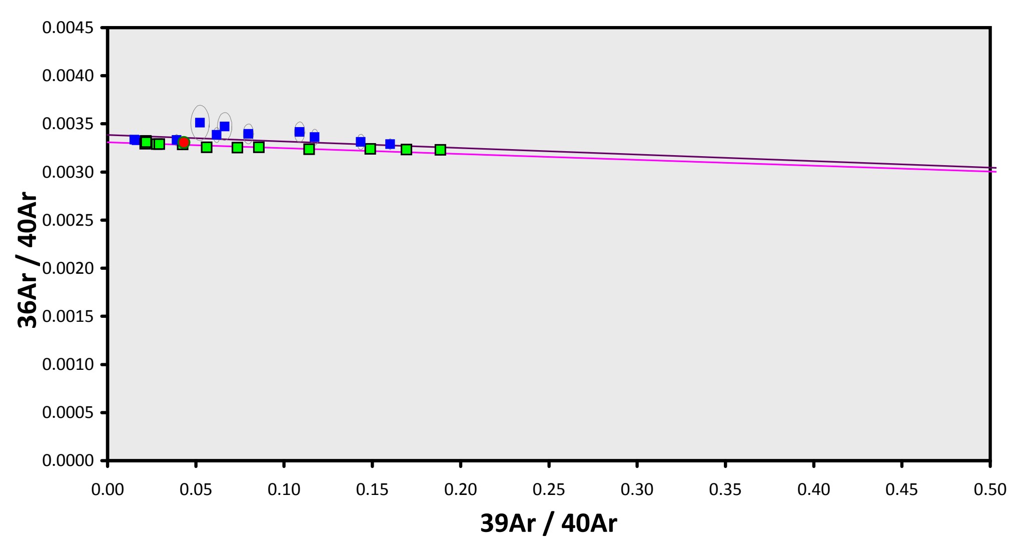

EXP#17D24038 > HLY0102-D90-18 > Groundmass > O-CONNOR (16-22)
ARTIC OCEAN > GAKKEL RIDGE
17-OSU-05 (5A14-17) > Incremental Heating > Dan Miggins

Information on Analysis and Constants Used in Calculations

Project = **O-CONNOR (16-22)**
Sample = **HLY0102-D90-18**
Material = **Groundmass**
Location = **Gakkel Ridge**
Region = **Artic Ocean**
Analyst = **Dan Miggins**
Irradiation = **17-OSU-05 (5A14-17)**
Position = **X: 0 | Y: 0 | Z/H: 20.1194 mm**
FCT-NM Age = **28.201 ± 0.023 Ma**
FCT-NM Reference = **Kuiper et al (2008)**
FCT-NM 40Ar/39Ar Ratio = **9.70619 ± 0.01116**
FCT-NM J-value = **0.00161932 ± 0.00000186**
Air Shot 40Ar/36Ar = **302.6970 ± 0.4268**
Air Shot MDF = **0.99404825 ± 0.00067391 (LIN)**
Experiment Type = **Incremental Heating**
Extraction Method = **Bulk Laser Heating**
Heating = **64 sec**
Isolation = **3.00 min**
Instrument = **ARGUS-VI-D**
Preferred Age = **Plateau Age**
Age Classification = **Crystallization Age**
IGSN = Undefined
Rock Class = **Igneous>Volcanic**
Lithology = **Basaltic Lava**
Lat-Lon = Undefined - Undefined
Age Equations = **Min et al. (2000)**
Negative Intensities = **Allowed**
Collector Calibrations = **36Ar**
Decay 40K = **5.530 ± 0.048 E-10 1/a**
Decay 39Ar = **2.940 ± 0.016 E-07 1/h**
Decay 37Ar = **8.230 ± 0.012 E-04 1/h**
Decay 36Cl = **2.257 ± 0.015 E-06 1/a**
Decay 40K(EC,β^+) = **0.580 ± 0.009 E-10 1/a**
Decay 40K(β^-) = **4.950 ± 0.043 E-10 1/a**
Atmospheric 40/36(a) = **297.96 ± 0.70**
Atmospheric 38/36(a) = **0.1869**
Production 39/37(ca) = **0.0006425 ± 0.0000059**
Production 38/37(ca) = **0.0001800 ± 0.0000173**
Production 36/37(ca) = **0.0002703 ± 0.0000005**
Production 40/39(k) = **0.000607 ± 0.000059**
Production 38/39(k) = **0.012077 ± 0.000011**
Production 36/38(cl) = **262.80 ± 1.71**
Scaling Ratio K/Ca = **0.430**
Abundance Ratio 40K/K = **1.1700 ± 0.0100 E-04**
Atomic Weight K = **39.0983 ± 0.0001 g**

Excess Initial 40Ar/36Ar = 297.96 ± 0.24 (%SD).

Results

Results	40(a)/36(a) ± 2σ		40(r)/39(k) ± 2σ		Age ± 2σ (ka)		MSWD	39Ar(k) (%,n)	K/Ca ± 2σ
**Age Plateau**			0.05000	± 0.02807	**146.4**	**± 82.2**	0.29	100.00	0.0308 ± 0.0068
Overestimated				± 56.14%		**± 56.14%**	100%	30	
					Full External Error	± 82.2	1.53	2σ Confidence Limit	
					Analytical Error	± 82.2	1.0000	Error Magnification	
**Total Fusion Age**			0.03544	± 0.03995	**103.8**	**± 117.0**		30	0.0589 ± 0.0001
				± 112.72%		**± 112.72%**			
					Full External Error	± 117.0			
					Analytical Error	± 117.0			
**Normal Isochron**	**297.95**	**± 1.40**	0.05005	± 0.04505	**146.6**	**± 131.9**	0.37	100.00	
		**± 0.47%**		± 90.01%		**± 90.00%**	100%	30	
					Full External Error	± 132.0	1.53	2σ Confidence Limit	
					Analytical Error	± 131.9	1.0000	Error Magnification	
**Inverse Isochron**	**297.96**	**± 1.40**	0.05010	± 0.02563	**146.7**	**± 75.1**	0.37	100.00	
Clustered Points		**± 0.47%**		± 51.16%		**± 51.16%**	100%	30	
					Full External Error	± 75.1	1.53	2σ Confidence Limit	
					Analytical Error	± 75.1	1.0000	Error Magnification	
							1%	Spreading Factor	

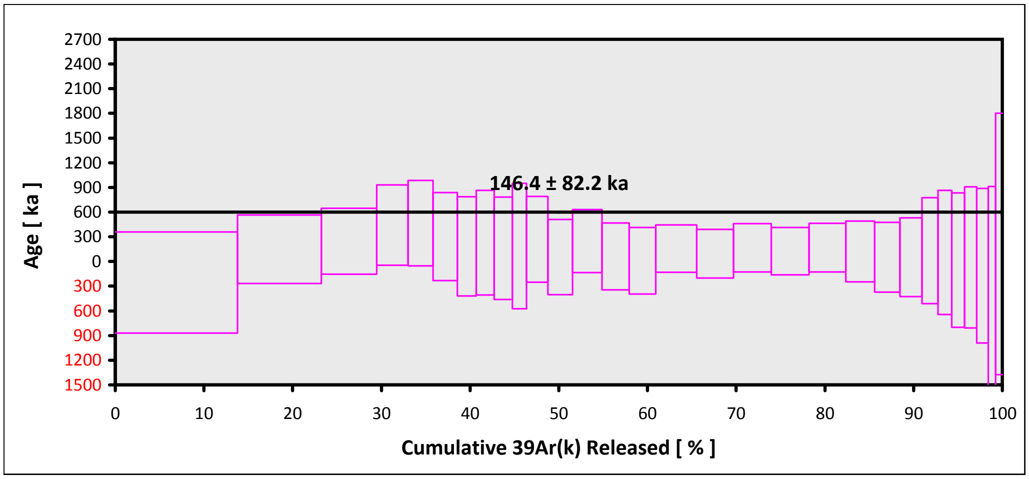

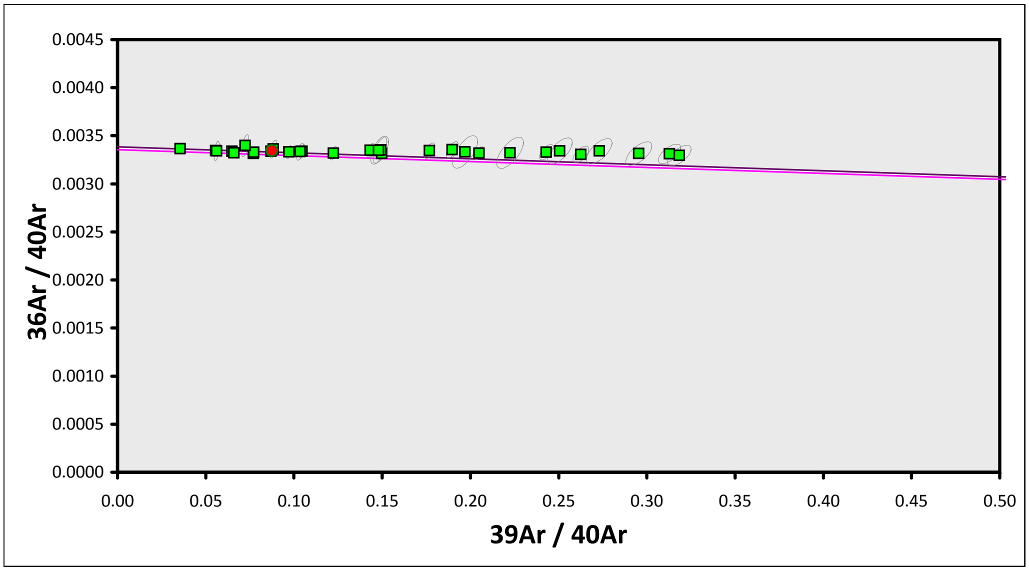

EXP#17D17883 > HLY0102-D89-004 > Groundmass > O-CONNOR (16-22)
ARTIC OCEAN > GAKKEL RIDGE
17-OSU-01 (1C2-17) > Incremental Heating > Dan Miggins

Information on Analysis and Constants Used in Calculations

Project = **O-CONNOR (16-22)**
Sample = **HLY0102-D89-004**
Material = **Groundmass**
Location = **Gakkel Ridge**
Region = **Artic Ocean**
Analyst = **Dan Miggins**
Irradiation = **17-OSU-01 (1C2-17)**
Position = **X: 0 | Y: 0 | Z/H: 3.663492 mm**
FCT-NM Age = **28.201 ± 0.023 Ma**
FCT-NM Reference = **Kuiper et al (2008)**
FCT-NM 40Ar/39Ar Ratio = **9.46726 ± 0.00852**
FCT-NM J-value = **0.00166018 ± 0.00000149**
Air Shot 40Ar/36Ar = **302.6190 ± 0.4025**
Air Shot MDF = **0.99411124 ± 0.00066430 (LIN)**
Experiment Type = **Incremental Heating**
Extraction Method = **Bulk Laser Heating**
Heating = **64 sec**
Isolation = **3.00 min**
Instrument = **ARGUS-VI-D**
Preferred Age = **Plateau Age**
Age Classification = **Crystallization Age**
IGSN = Undefined
Rock Class = **Igneous>Volcanic**
Lithology = **Basaltic Lava**
Lat-Lon = Undefined - Undefined
Age Equations = **Min et al. (2000)**
Negative Intensities = **Allowed**
Collector Calibrations = **36Ar**
Decay 40K = **5.530 ± 0.048 E-10 1/a**
Decay 39Ar = **2.940 ± 0.016 E-07 1/h**
Decay 37Ar = **8.230 ± 0.012 E-04 1/h**
Decay 36Cl = **2.257 ± 0.015 E-06 1/a**
Decay 40K(EC,β^+) = **0.580 ± 0.009 E-10 1/a**
Decay 40K(β^-) = **4.950 ± 0.043 E-10 1/a**
Atmospheric 40/36(a) = **295.50 ± 0.70**
Atmospheric 38/36(a) = **0.1869**
Production 39/37(ca) = **0.0006425 ± 0.0000059**
Production 38/37(ca) = **0.0001800 ± 0.0000173**
Production 36/37(ca) = **0.0002703 ± 0.0000005**
Production 40/39(k) = **0.000607 ± 0.000059**
Production 38/39(k) = **0.012077 ± 0.000011**
Production 36/38(cl) = **262.80 ± 1.71**
Scaling Ratio K/Ca = **0.430**
Abundance Ratio 40K/K = **1.1700 ± 0.0100 E-04**
Atomic Weight K = **39.0983 ± 0.0001 g**

Results

Results	40(a)/36(a) ± 2σ	40(r)/39(k) ± 2σ	Age ± 2σ (Ma)	MSWD	39Ar(k) (%,n)	K/Ca ± 2σ
**Age Plateau**		0.47147 ± 0.01977 ± 4.19%	**1.41 ± 0.06 ± 4.20%**	1.03 41%	61.14 12	0.0260 ± 0.0041
			Full External Error ± 0.07	1.85	2σ Confidence Limit	
			Analytical Error ± 0.06	1.0168	Error Magnification	
**Total Fusion Age**		0.45994 ± 0.01945 ± 4.23%	**1.38 ± 0.06 ± 4.23%**		24	0.0233 ± 0.0001
			Full External Error ± 0.07			
			Analytical Error ± 0.06			
**Normal Isochron**	**293.75 ± 6.30 ± 2.15%**	0.50541 ± 0.12509 ± 24.75%	**1.52 ± 0.38 ± 24.74%**	1.32 21%	61.14 12	
			Full External Error ± 0.38	1.89	2σ Confidence Limit	
			Analytical Error ± 0.38	1.1500	Error Magnification	
**Inverse Isochron** Clustered Points	**293.73 ± 6.31 ± 2.15%**	0.50637 ± 0.11529 ± 22.77%	**1.52 ± 0.35 ± 22.76%**	1.32 21%	61.14 12	
			Full External Error ± 0.35	1.89	2σ Confidence Limit	
			Analytical Error ± 0.35	1.1500	Error Magnification	
				4%	Spreading Factor	

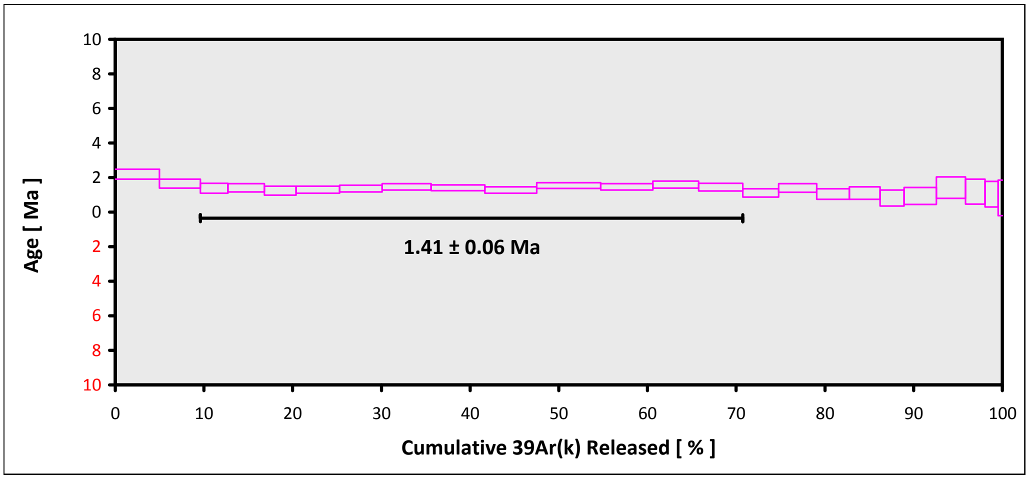

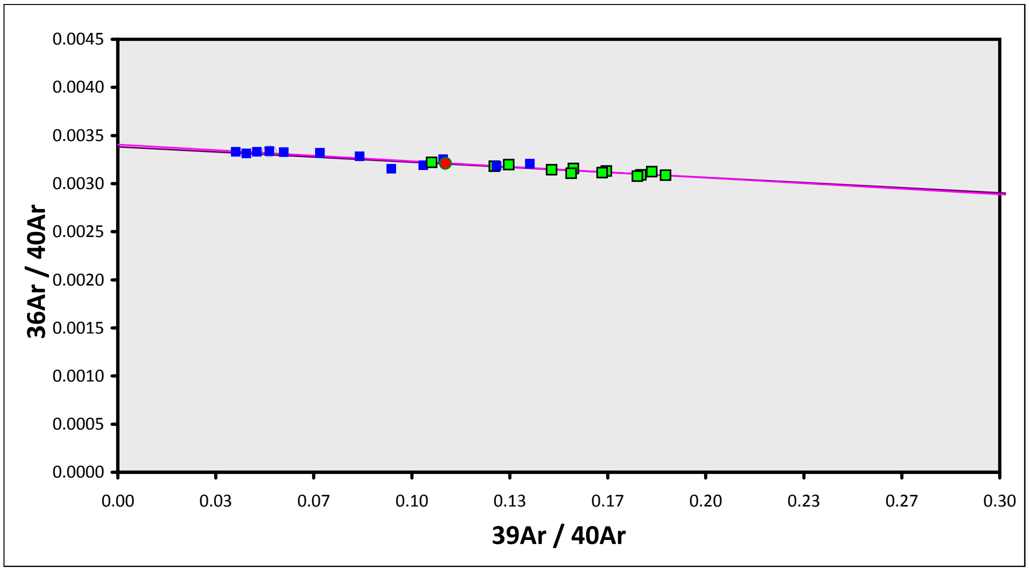

EXP#17D30281 > HLY0102-D85-90 > Groundmass > O-CONNOR (16-22) ARTIC OCEAN > GAKKEL RIDGE 17-OSU-05 (5B6-17) > Incremental Heating > Dan Miggins

Information on Analysis and Constants Used in Calculations

Project = **O-CONNOR (16-22)**
Sample = **HLY0102-D85-90**
Material = **Groundmass**
Location = **Gakkel Ridge**
Region = **Artic Ocean**
Analyst = **Dan Miggins**
Irradiation = **17-OSU-05 (5B6-17)**
Position = **X: 0 | Y: 0 | Z/H: 9.527553 mm**
FCT-NM Age = **28.201 ± 0.023 Ma**
FCT-NM Reference = **Kuiper et al (2008)**
FCT-NM 40Ar/39Ar Ratio = **9.59452 ± 0.00950**
FCT-NM J-value = **0.00163816 ± 0.00000162**
Air Shot 40Ar/36Ar = **302.8530 ± 0.4573**
Air Shot MDF = **0.99392238 ± 0.00068637 (LIN)**
Experiment Type = **Incremental Heating**
Extraction Method = **Bulk Laser Heating**
Heating = **64 sec**
Isolation = **3.00 min**
Instrument = **ARGUS-VI-D**
Preferred Age = **Plateau Age**
Age Classification = **Crystallization Age**
IGSN = Undefined
Rock Class = **Igneous>Volcanic**
Lithology = **Basaltic Lava**
Lat-Lon = Undefined - Undefined
Age Equations = **Min et al. (2000)**
Negative Intensities = **Allowed**
Collector Calibrations = **36Ar**
Decay 40K = **5.530 ± 0.048 E-10 1/a**
Decay 39Ar = **2.940 ± 0.016 E-07 1/h**
Decay 37Ar = **8.230 ± 0.012 E-04 1/h**
Decay 36Cl = **2.257 ± 0.015 E-06 1/a**
Decay 40K(EC,β^+) = **0.580 ± 0.009 E-10 1/a**
Decay 40K(β^-) = **4.950 ± 0.043 E-10 1/a**
Atmospheric 40/36(a) = **295.50**
Atmospheric 38/36(a) = **0.1869**
Production 39/37(ca) = **0.0006425 ± 0.0000059**
Production 38/37(ca) = **0.0001800 ± 0.0000173**
Production 36/37(ca) = **0.0002703 ± 0.0000005**
Production 40/39(k) = **0.000607 ± 0.000059**
Production 38/39(k) = **0.012077 ± 0.000011**
Production 36/38(cl) = **262.80 ± 1.71**
Scaling Ratio K/Ca = **0.430**
Abundance Ratio 40K/K = **1.1700 ± 0.0100 E-04**
Atomic Weight K = **39.0983 ± 0.0001 g**

Bumpy spectrum

Results

Results	40(a)/36(a) ± 2σ		40(r)/39(k) ± 2σ		Age ± 2σ (Ma)		MSWD	39Ar(k) (%,n)	K/Ca ± 2σ
**Age Plateau**			0.60740	± 0.03341	**1.80**	**± 0.10**	2.63	92.39	0.0276 ± 0.0061
Error Mean				± 5.50%		**± 5.50%**	0%	17	
					Full External Error	± 0.11	1.71	2σ Confidence Limit	
					Analytical Error	± 0.10	1.6209	Error Magnification	
**Total Fusion Age**			0.57978	± 0.04452	**1.72**	**± 0.13**		24	0.0402 ± 0.0001
				± 7.68%		**± 7.68%**			
					Full External Error	± 0.14			
					Analytical Error	± 0.13			
**Normal Isochron**	**296.30**	**± 1.59**	0.58326	± 0.05649	**1.73**	**± 0.17**	2.62	92.39	
Error Chron		**± 0.54%**		± 9.69%		**± 9.68%**	0%	17	
					Full External Error	± 0.17	1.73	2σ Confidence Limit	
					Analytical Error	± 0.17	1.6174	Error Magnification	
**Inverse Isochron**	**296.30**	**± 1.60**	0.58456	± 0.05575	**1.73**	**± 0.17**	2.63	92.39	
Error Chron		**± 0.54%**		± 9.54%		**± 9.53%**	0%	17	
					Full External Error	± 0.17	1.73	2σ Confidence Limit	
					Analytical Error	± 0.16	1.6204	Error Magnification	
							9%	Spreading Factor	

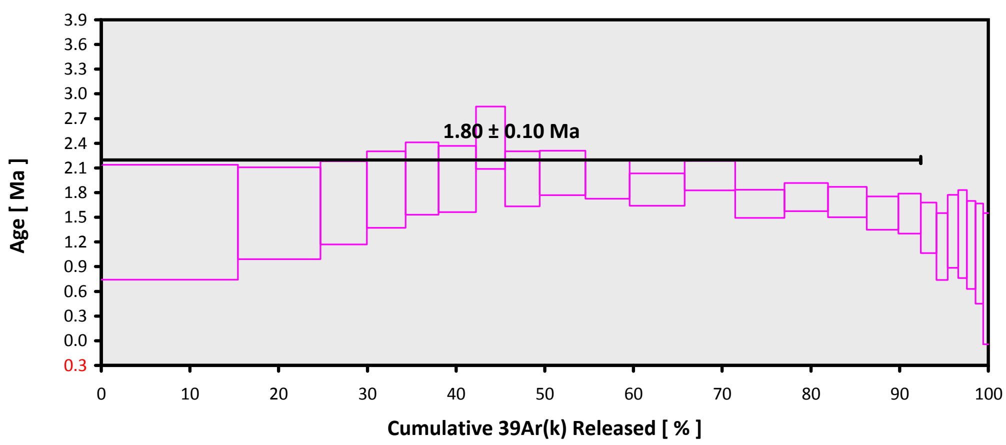

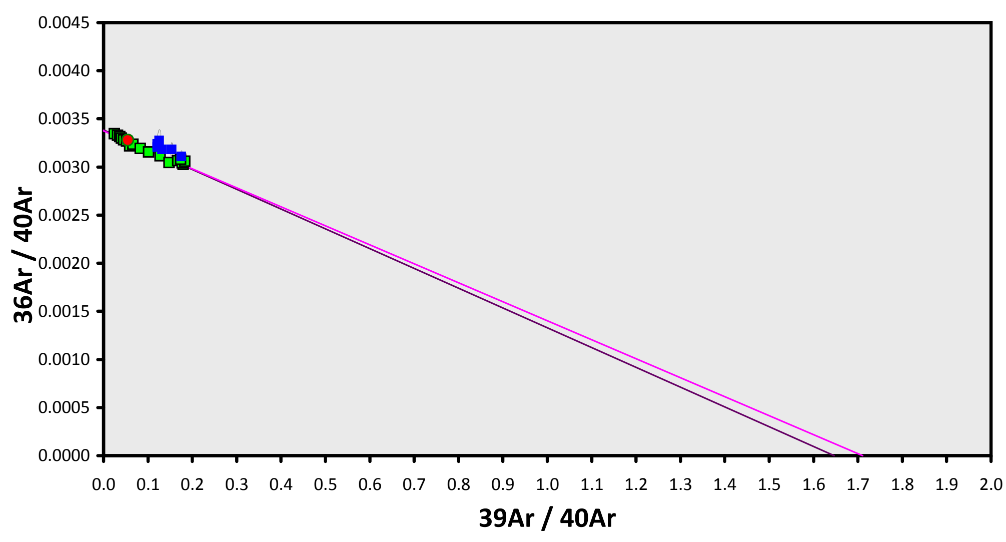

EXP#17D20349 > PS59-305-1 > Groundmass > O-CONNOR (16-22)
ARTIC OCEAN > GAKKEL RIDGE
17-OSU-01 (1C35-17) > Incremental Heating > Dan Miggins

Information on Analysis and Constants Used in Calculations

Project = **O-CONNOR (16-22)**
Sample = **PS59-305-1**
Material = **Groundmass**
Location = **Gakkel Ridge**
Region = **Artic Ocean**
Analyst = **Dan Miggins**
Irradiation = **17-OSU-01 (1C35-17)**
Position = **X: 0 | Y: 0 | Z/H: 53.4427 mm**
FCT-NM Age = **28.201 ± 0.023 Ma**
FCT-NM Reference = **Kuiper et al (2008)**
FCT-NM 40Ar/39Ar Ratio = **10.09436 ± 0.00848**
FCT-NM J-value = **0.00155705 ± 0.00000131**
Air Shot 40Ar/36Ar = **302.4360 ± 0.4537**
Air Shot MDF = **0.99425915 ± 0.00068600 (LIN)**
Experiment Type = **Incremental Heating**
Extraction Method = **Bulk Laser Heating**
Heating = **64 sec**
Isolation = **3.00 min**
Instrument = **ARGUS-VI-D**
Preferred Age = **Plateau Age**
Age Classification = **Crystallization Age**
IGSN = Undefined
Rock Class = **Igneous>Volcanic**
Lithology = **Basaltic Lava**
Lat-Lon = Undefined - Undefined
Age Equations = **Min et al. (2000)**
Negative Intensities = **Allowed**
Collector Calibrations = **36Ar**
Decay 40K = **5.530 ± 0.048 E-10 1/a**
Decay 39Ar = **2.940 ± 0.016 E-07 1/h**
Decay 37Ar = **8.230 ± 0.012 E-04 1/h**
Decay 36Cl = **2.257 ± 0.015 E-06 1/a**
Decay 40K(EC,β^+) = **0.580 ± 0.009 E-10 1/a**
Decay 40K(β^-) = **4.950 ± 0.043 E-10 1/a**
Atmospheric 40/36(a) = **291.41 ± 0.62**
Atmospheric 38/36(a) = **0.1869**
Production 39/37(ca) = **0.0006425 ± 0.0000059**
Production 38/37(ca) = **0.0001800 ± 0.0000173**
Production 36/37(ca) = **0.0002703 ± 0.0000005**
Production 40/39(k) = **0.000607 ± 0.000059**
Production 38/39(k) = **0.012077 ± 0.000011**
Production 36/38(cl) = **262.80 ± 1.71**
Scaling Ratio K/Ca = **0.430**
Abundance Ratio 40K/K = **1.1700 ± 0.0100 E-04**
Atomic Weight K = **39.0983 ± 0.0001 g**

Subatmospheric Initial 40Ar/36Ar = 291.41 ± 0.21 (%SD).

Results

Results	40(a)/36(a) ± 2σ		40(r)/39(k) ± 2σ		Age ± 2σ (ka)		MSWD	39Ar(k) (%,n)	K/Ca ± 2σ
**Age Plateau**			0.34754	± 0.01504<br>± 4.33%	**978.3**	**± 42.4<br>± 4.33%**	0.66<br>82%	70.83<br>16	0.044 ± 0.007
					Full External Error	± 47.8	1.73	2σ Confidence Limit	
					Analytical Error	± 42.3	1.0000	Error Magnification	
**Total Fusion Age**			0.53844	± 0.07642<br>± 14.19%	**1515.4**	**± 215.0<br>± 14.19%**		24	0.071 ± 0.000
					Full External Error	± 217.7			
					Analytical Error	± 215.0			
**Normal Isochron**	**291.42**	**± 1.23<br>± 0.42%**	0.34639	± 0.02734<br>± 7.89%	**975.1**	**± 76.9<br>± 7.89%**	0.81<br>66%	70.83<br>16	
					Full External Error	± 80.0	1.76	2σ Confidence Limit	
					Analytical Error	± 76.9	1.0000	Error Magnification	
**Inverse Isochron**	**291.41**	**± 1.23<br>± 0.42%**	0.34706	± 0.02706<br>± 7.80%	**976.9**	**± 76.2<br>± 7.80%**	0.82<br>65%	70.83<br>16	
					Full External Error	± 79.3	1.76	2σ Confidence Limit	
					Analytical Error	± 76.1	1.0000	Error Magnification	
							7%	Spreading Factor	

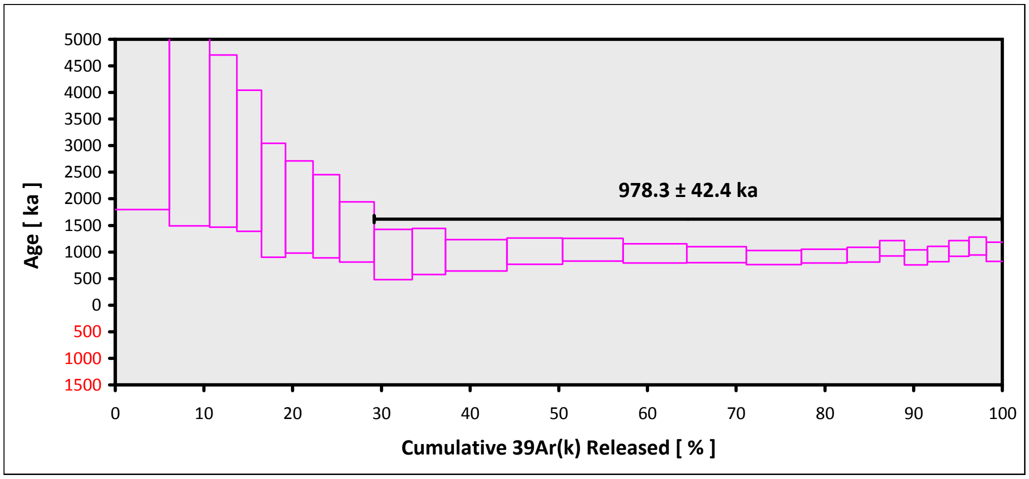

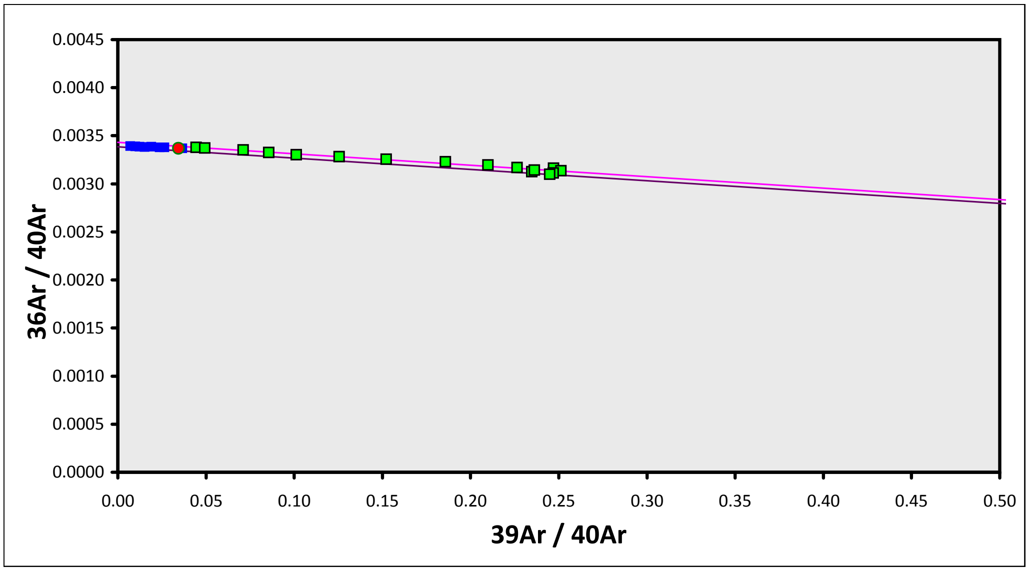

EXP#17D19828 > PS59-305-20 > Groundmass > O-CONNOR (16-22)
ARTIC OCEAN > GAKKEL RIDGE
17-OSU-01 (1F26-17) > Incremental Heating > Dan Miggins

Information on Analysis and Constants Used in Calculations

Project = **O-CONNOR (16-22)**
Sample = **PS59-305-20**
Material = **Groundmass**
Location = **Gakkel Ridge**
Region = **Artic Ocean**
Analyst = **Dan Miggins**
Irradiation = **17-OSU-01 (1F26-17)**
Position = **X: 0 | Y: 0 | Z/H: 34.82958 mm**
FCT-NM Age = **28.201 ± 0.023 Ma**
FCT-NM Reference = **Kuiper et al (2008)**
FCT-NM 40Ar/39Ar Ratio = **9.72237 ± 0.00914**
FCT-NM J-value = **0.00161662 ± 0.00000152**
Air Shot 40Ar/36Ar = **302.5200 ± 0.4205**
Air Shot MDF = **0.99419123 ± 0.00067181 (LIN)**
Experiment Type = **Incremental Heating**
Extraction Method = **Bulk Laser Heating**
Heating = **64 sec**
Isolation = **3.00 min**
Instrument = **ARGUS-VI-D**
Preferred Age = **Plateau Age**
Age Classification = **Crystallization Age**
IGSN = Undefined
Rock Class = **Igneous>Volcanic**
Lithology = **Basaltic Lava**
Lat-Lon = Undefined - Undefined
Age Equations = **Min et al. (2000)**
Negative Intensities = **Allowed**
Collector Calibrations = **36Ar**
Decay 40K = **5.530 ± 0.048 E-10 1/a**
Decay 39Ar = **2.940 ± 0.016 E-07 1/h**
Decay 37Ar = **8.230 ± 0.012 E-04 1/h**
Decay 36Cl = **2.257 ± 0.015 E-06 1/a**
Decay 40K(EC,β^+) = **0.580 ± 0.009 E-10 1/a**
Decay 40K(β^-) = **4.950 ± 0.043 E-10 1/a**
Atmospheric 40/36(a) = **295.50**
Atmospheric 38/36(a) = **0.1869**
Production 39/37(ca) = **0.0006425 ± 0.0000059**
Production 38/37(ca) = **0.0001800 ± 0.0000173**
Production 36/37(ca) = **0.0002703 ± 0.0000005**
Production 40/39(k) = **0.000607 ± 0.000059**
Production 38/39(k) = **0.012077 ± 0.000011**
Production 36/38(cl) = **262.80 ± 1.71**
Scaling Ratio K/Ca = **0.430**
Abundance Ratio 40K/K = **1.1700 ± 0.0100 E-04**
Atomic Weight K = **39.0983 ± 0.0001 g**

Mostly atmospheric

Results

Results	40(a)/36(a) ± 2σ		40(r)/39(k) ± 2σ		Age ± 2σ (ka)		MSWD	39Ar(k) (%,n)	K/Ca ± 2σ
**Age Plateau**			0.25930	± 0.00987<br>± 3.80%	**757.9**	**± 28.9<br>± 3.81%**	1.05<br>40%	96.61<br>20	0.020 ± 0.006
				Full External Error	± 33.6		1.65	2σ Confidence Limit	
				Analytical Error	± 28.8		1.0232	Error Magnification	
**Total Fusion Age**			0.25328	± 0.01021<br>± 4.03%	**740.3**	**± 29.9<br>± 4.03%**		24	0.063 ± 0.000
				Full External Error	± 34.2				
				Analytical Error	± 29.8				
**Normal Isochron**	**293.59**	**± 5.46<br>± 1.86%**	0.28707	± 0.08095<br>± 28.20%	**839.0**	**± 236.6<br>± 28.19%**	1.08<br>36%	96.61<br>20	
				Full External Error	± 237.3		1.67	2σ Confidence Limit	
				Analytical Error	± 236.5		1.0404	Error Magnification	
**Inverse Isochron**<br>Clustered Points	**293.61**	**± 5.45<br>± 1.86%**	0.28715	± 0.06836<br>± 23.80%	**839.3**	**± 199.7<br>± 23.80%**	1.07<br>37%	96.61<br>20	
				Full External Error	± 200.6		1.67	2σ Confidence Limit	
				Analytical Error	± 199.7		1.0367	Error Magnification	
							3%	Spreading Factor	

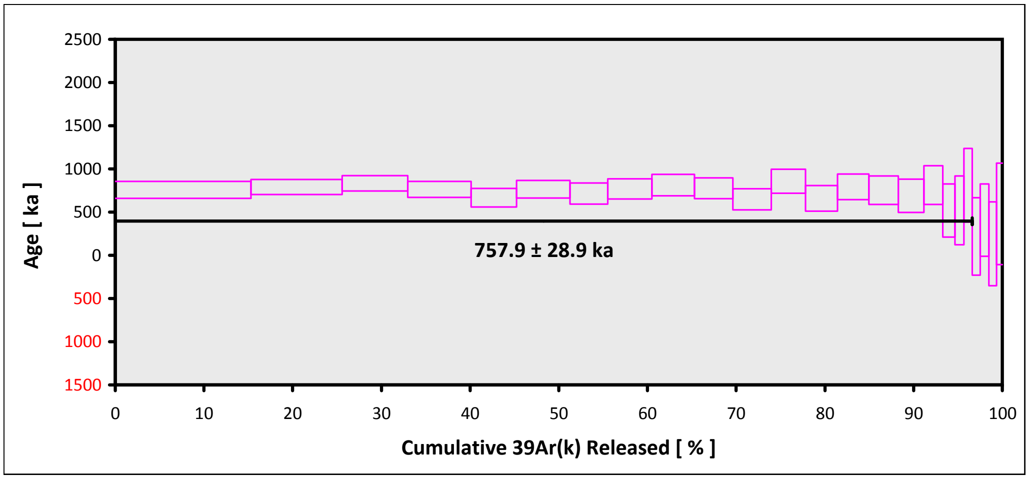

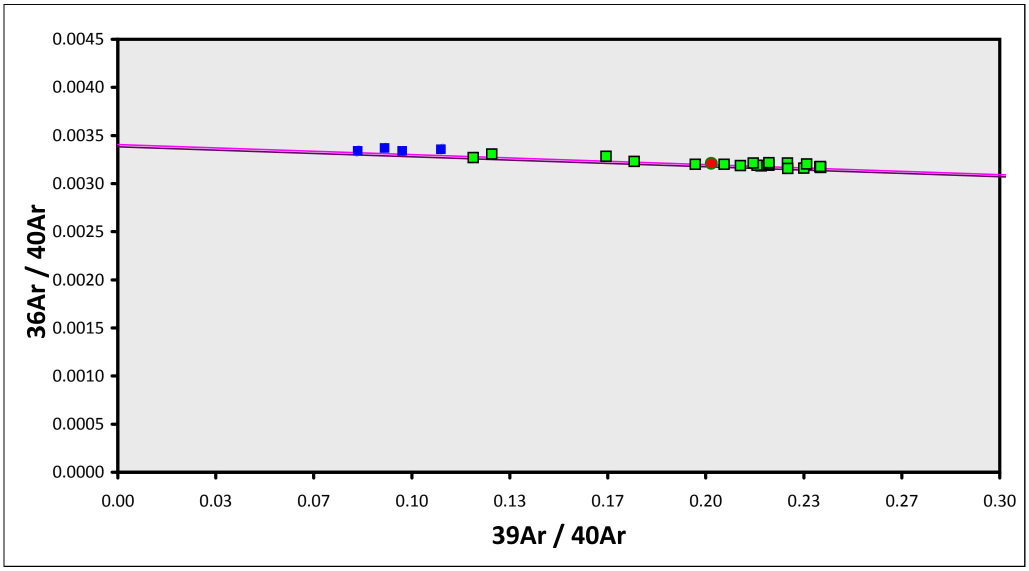

EXP#17D19761 > PS59-300-18 > Groundmass > O-CONNOR (16-22)
ARTIC OCEAN > GAKKEL RIDGE
17-OSU-01 (1F20-17) > Incremental Heating > Dan Miggins

Information on Analysis and Constants Used in Calculations

Project = **O-CONNOR (16-22)**
Sample = **PS59-300-18**
Material = **Groundmass**
Location = **Gakkel Ridge**
Region = **Artic Ocean**
Analyst = **Dan Miggins**
Irradiation = **17-OSU-01 (1F20-17)**
Position = **X: 0 | Y: 0 | Z/H: 25.3719 mm**
FCT-NM Age = **28.201 ± 0.023 Ma**
FCT-NM Reference = **Kuiper et al (2008)**
FCT-NM 40Ar/39Ar Ratio = **9.58986 ± 0.00921**
FCT-NM J-value = **0.00163896 ± 0.00000157**
Air Shot 40Ar/36Ar = **302.5630 ± 0.3358**
Air Shot MDF = **0.99415648 ± 0.00063982 (LIN)**
Experiment Type = **Incremental Heating**
Extraction Method = **Bulk Laser Heating**
Heating = **64 sec**
Isolation = **3.00 min**
Instrument = **ARGUS-VI-D**
Preferred Age = **Plateau Age**
Age Classification = **Crystallization Age**
IGSN = Undefined
Rock Class = **Igneous>Volcanic**
Lithology = **Basaltic Lava**
Lat-Lon = Undefined - Undefined
Age Equations = **Min et al. (2000)**
Negative Intensities = **Allowed**
Collector Calibrations = **36Ar**
Decay 40K = **5.530 ± 0.048 E-10 1/a**
Decay 39Ar = **2.940 ± 0.016 E-07 1/h**
Decay 37Ar = **8.230 ± 0.012 E-04 1/h**
Decay 36Cl = **2.257 ± 0.015 E-06 1/a**
Decay 40K(EC,β^+) = **0.580 ± 0.009 E-10 1/a**
Decay 40K(β^-) = **4.950 ± 0.043 E-10 1/a**
Atmospheric 40/36(a) = **295.50**
Atmospheric 38/36(a) = **0.1869**
Production 39/37(ca) = **0.0006425 ± 0.0000059**
Production 38/37(ca) = **0.0001800 ± 0.0000173**
Production 36/37(ca) = **0.0002703 ± 0.0000005**
Production 40/39(k) = **0.000607 ± 0.000059**
Production 38/39(k) = **0.012077 ± 0.000011**
Production 36/38(cl) = **262.80 ± 1.71**
Scaling Ratio K/Ca = **0.430**
Abundance Ratio 40K/K = **1.1700 ± 0.0100 E-04**
Atomic Weight K = **39.0983 ± 0.0001 g**

Mostly atmospheric

Results

Results	40(a)/36(a) ± 2σ		40(r)/39(k) ± 2σ		Age ± 2σ (Ma)		MSWD	39Ar(k) (%,n)	K/Ca ± 2σ
**Age Plateau**			1.20976	± 0.02396<br>± 1.98%	**3.58**	**± 0.07**<br>**± 1.99%**	1.11<br>35%	48.97<br>10	0.0172 ± 0.0015
					Full External Error	± 0.11	1.94	2σ Confidence Limit	
					Analytical Error	± 0.07	1.0540	Error Magnification	
**Total Fusion Age**			1.02183	± 0.04411<br>± 4.32%	**3.03**	**± 0.13**<br>**± 4.32%**		24	0.0242 ± 0.0001
					Full External Error	± 0.15			
					Analytical Error	± 0.13			
**Normal Isochron**	**293.45**	**± 6.52**<br>**± 2.22%**	1.23795	± 0.09561<br>± 7.72%	**3.67**	**± 0.28**<br>**± 7.72%**	1.19<br>30%	48.97<br>10	
					Full External Error	± 0.29	2.00	2σ Confidence Limit	
					Analytical Error	± 0.28	1.0929	Error Magnification	
**Inverse Isochron**	**293.37**	**± 6.47**<br>**± 2.21%**	1.24000	± 0.09396<br>± 7.58%	**3.67**	**± 0.28**<br>**± 7.57%**	1.19<br>30%	48.97<br>10	
					Full External Error	± 0.29	2.00	2σ Confidence Limit	
					Analytical Error	± 0.28	1.0888	Error Magnification	
							13%	Spreading Factor	

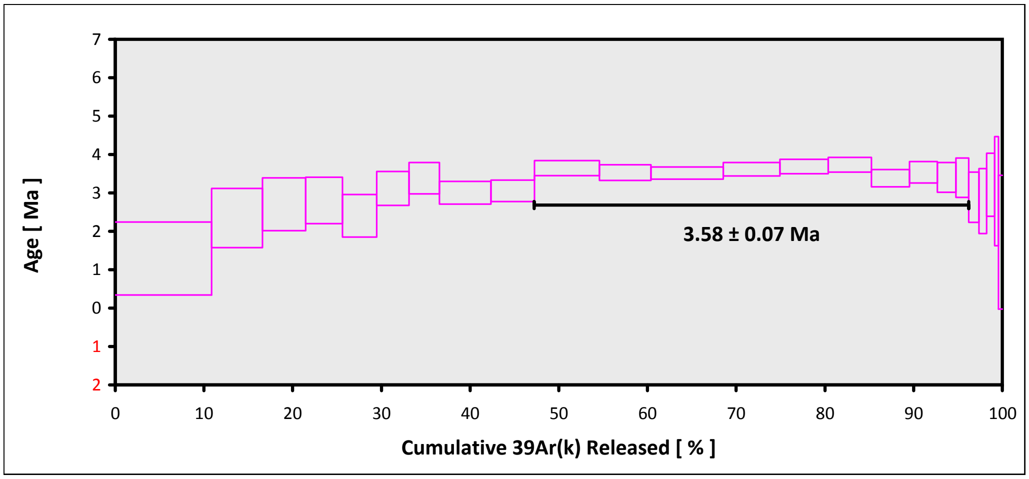

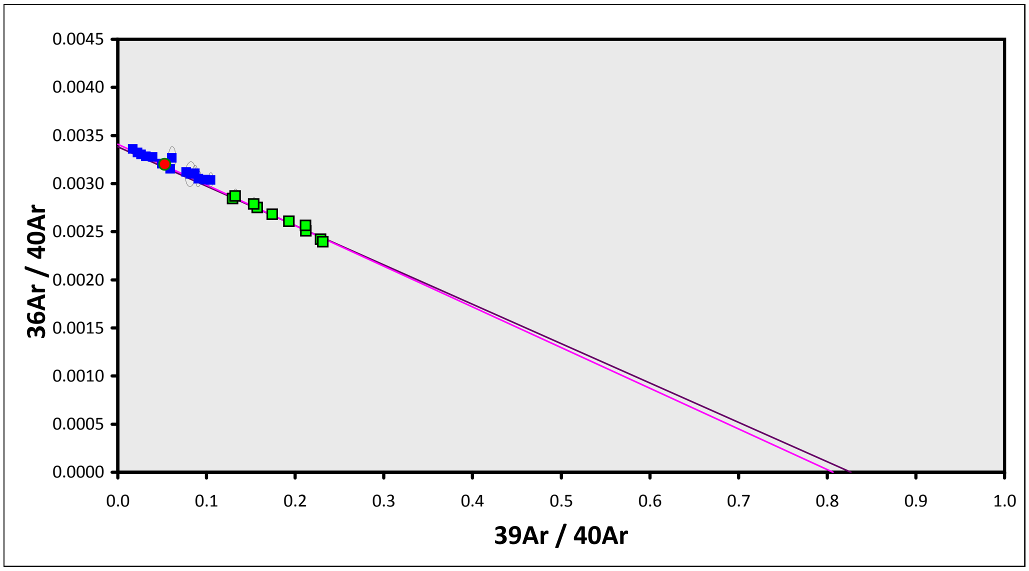

EXP#18D00424 > PS59-299-1 > Groundmass > O-CONNOR (16-22)
ARTIC OCEAN > GAKKEL RIDGE
17-OSU-05 (5B33-17) > Incremental Heating > Dan Miggins

Information on Analysis and Constants Used in Calculations

Project = **O-CONNOR (16-22)**
Sample = **PS59-299-1**
Material = **Groundmass**
Location = **Gakkel Ridge**
Region = **Artic Ocean**
Analyst = **Dan Miggins**
Irradiation = **17-OSU-05 (5B33-17)**
Position = **X: 0 | Y: 0 | Z/H: 48.74548 mm**
FCT-NM Age = **28.201 ± 0.023 Ma**
FCT-NM Reference = **Kuiper et al (2008)**
FCT-NM 40Ar/39Ar Ratio = **10.23897 ± 0.00788**
FCT-NM J-value = **0.00153506 ± 0.00000118**
Air Shot 40Ar/36Ar = **302.3240 ± 0.4686**
Air Shot MDF = **0.99434975 ± 0.00069288 (LIN)**
Experiment Type = **Incremental Heating**
Extraction Method = **Bulk Laser Heating**
Heating = **77 sec**
Isolation = **3.00 min**
Instrument = **ARGUS-VI-D**
Preferred Age = **Plateau Age**
Age Classification = **Crystallization Age**
IGSN = Undefined
Rock Class = Undefined
Lithology = **Basaltic Lava**
Lat-Lon = Undefined - Undefined
Age Equations = **Min et al. (2000)**
Negative Intensities = **Allowed**
Collector Calibrations = **36Ar**
Decay 40K = **5.530 ± 0.048 E-10 1/a**
Decay 39Ar = **2.940 ± 0.016 E-07 1/h**
Decay 37Ar = **8.230 ± 0.012 E-04 1/h**
Decay 36Cl = **2.257 ± 0.015 E-06 1/a**
Decay 40K(EC,β^+) = **0.580 ± 0.009 E-10 1/a**
Decay 40K(β^-) = **4.950 ± 0.043 E-10 1/a**
Atmospheric 40/36(a) = **295.27 ± 1.95**
Atmospheric 38/36(a) = **0.1869**
Production 39/37(ca) = **0.0006425 ± 0.0000059**
Production 38/37(ca) = **0.0001800 ± 0.0000173**
Production 36/37(ca) = **0.0002703 ± 0.0000005**
Production 40/39(k) = **0.000607 ± 0.000059**
Production 38/39(k) = **0.012077 ± 0.000011**
Production 36/38(cl) = **262.80 ± 1.71**
Scaling Ratio K/Ca = **0.430**
Abundance Ratio 40K/K = **1.1700 ± 0.0100 E-04**
Atomic Weight K = **39.0983 ± 0.0001 g**

Mostly atmospheric

Results

Results	40(a)/36(a) ± 2σ		40(r)/39(k) ± 2σ		Age ± 2σ (Ma)		MSWD	39Ar(k) (%,n)	K/Ca ± 2σ
**Age Plateau**			0.35991	± 0.10773<br>± 29.93%	**1.00**	**± 0.30**<br>**± 29.93%**	0.91<br>54%	72.78<br>13	0.0042 ± 0.0003
				Full External Error	± 0.30		1.82	2σ Confidence Limit	
				Analytical Error	± 0.30		1.0000	Error Magnification	
**Total Fusion Age**			1.09041	± 0.16476<br>± 15.11%	**3.02**	**± 0.46**<br>**± 15.10%**		24	0.0041 ± 0.0000
				Full External Error	± 0.46				
				Analytical Error	± 0.46				
**Normal Isochron**	**295.18**	**± 3.93**<br>**± 1.33%**	0.36165	± 0.24010<br>± 66.39%	**1.00**	**± 0.67**<br>**± 66.37%**	1.58<br>10%	72.78<br>13	
				Full External Error	± 0.67		1.85	2σ Confidence Limit	
				Analytical Error	± 0.67		1.2585	Error Magnification	
**Inverse Isochron**<br>Clustered Points	**295.27**	**± 3.89**<br>**± 1.32%**	0.36188	± 0.15946<br>± 44.07%	**1.00**	**± 0.44**<br>**± 44.05%**	1.56<br>10%	72.78<br>13	
				Full External Error	± 0.44		1.85	2σ Confidence Limit	
				Analytical Error	± 0.44		1.2508	Error Magnification	
							3%	Spreading Factor	

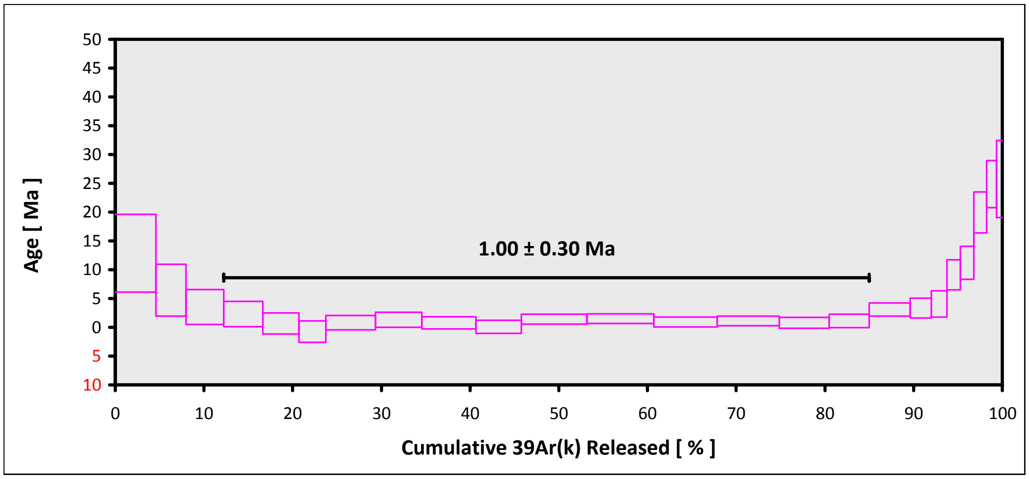

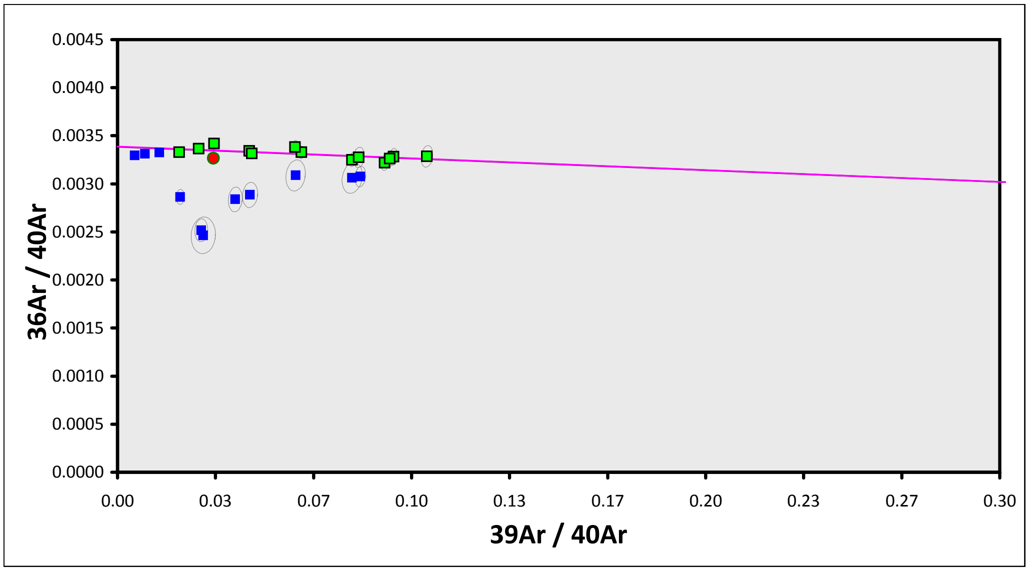

EXP#18D00464 > PS59-271-1-14 > Groundmass > O-CONNOR (16-22) > ARTIC OCEAN > GAKKEL RIDGE > 17-OSU-05 (5B9-17) > Incremental Heating > Dan Miggins

Information on Analysis and Constants Used in Calculations

Project = **O-CONNOR (16-22)**
Sample = **PS59-271-1-14**
Material = **Groundmass**
Location = **Gakkel Ridge**
Region = **Artic Ocean**
Analyst = **Dan Miggins**
Irradiation = **17-OSU-05 (5B9-17)**
Position = **X: 0 | Y: 0 | Z/H: 13.78676 mm**
FCT-NM Age = **28.201 ± 0.023 Ma**
FCT-NM Reference = **Kuiper et al (2008)**
FCT-NM 40Ar/39Ar Ratio = **9.62643 ± 0.00789**
FCT-NM J-value = **0.00163273 ± 0.00000134**
Air Shot 40Ar/36Ar = **302.3250 ± 0.4686**
Air Shot MDF = **0.99434894 ± 0.00069288 (LIN)**
Experiment Type = **Incremental Heating**
Extraction Method = **Bulk Laser Heating**
Heating = **77 sec**
Isolation = **3.00 min**
Instrument = **ARGUS-VI-D**
Preferred Age = **Plateau Age**
Age Classification = **Crystallization Age**
IGSN = Undefined
Rock Class = Undefined
Lithology = **Basaltic Lava**
Lat-Lon = Undefined - Undefined
Age Equations = **Min et al. (2000)**
Negative Intensities = **Allowed**
Collector Calibrations = **36Ar**
Decay 40K = **5.530 ± 0.048 E-10 1/a**
Decay 39Ar = **2.940 ± 0.016 E-07 1/h**
Decay 37Ar = **8.230 ± 0.012 E-04 1/h**
Decay 36Cl = **2.257 ± 0.015 E-06 1/a**
Decay 40K(EC,β^+) = **0.580 ± 0.009 E-10 1/a**
Decay 40K(β^-) = **4.950 ± 0.043 E-10 1/a**
Atmospheric 40/36(a) = **304.74 ± 1.70**
Atmospheric 38/36(a) = **0.1869**
Production 39/37(ca) = **0.0006425 ± 0.0000059**
Production 38/37(ca) = **0.0001800 ± 0.0000173**
Production 36/37(ca) = **0.0002703 ± 0.0000005**
Production 40/39(k) = **0.000607 ± 0.000059**
Production 38/39(k) = **0.012077 ± 0.000011**
Production 36/38(cl) = **262.80 ± 1.71**
Scaling Ratio K/Ca = **0.430**
Abundance Ratio 40K/K = **1.1700 ± 0.0100 E-04**
Atomic Weight K = **39.0983 ± 0.0001 g**

Excess Initial 40Ar/36Ar = 304.74 ± 0.56 (%SD). Mostly atmospheric

Results

Results	40(a)/36(a) ± 2σ		40(r)/39(k) ± 2σ		Age ± 2σ (Ma)		MSWD	39Ar(k) (%,n)	K/Ca ± 2σ
**Age Plateau**			0.37903	± 0.03725	**1.12**	**± 0.11**	1.73	88.27	0.0258 ± 0.0050
Error Mean				± 9.83%		**± 9.83%**	4%	16	
				Full External Error		± 0.11	1.73	2σ Confidence Limit	
				Analytical Error		± 0.11	1.3164	Error Magnification	
**Total Fusion Age**			0.35760	± 0.03632	**1.06**	**± 0.11**		24	0.0301 ± 0.0003
				± 10.16%		**± 10.15%**			
				Full External Error		± 0.11			
				Analytical Error		± 0.11			
**Normal Isochron**	**305.10**	**± 3.40**	0.38241	± 0.08343	**1.13**	**± 0.25**	3.28	88.27	
Error Chron		**± 1.12%**		± 21.82%		**± 21.81%**	0%	16	
				Full External Error		± 0.25	1.76	2σ Confidence Limit	
				Analytical Error		± 0.25	1.8111	Error Magnification	
**Inverse Isochron**	**305.05**	**± 3.37**	0.38599	± 0.07616	**1.14**	**± 0.22**	3.21	88.27	
Error Chron		**± 1.10%**		± 19.73%		**± 19.72%**	0%	16	
				Full External Error		± 0.23	1.76	2σ Confidence Limit	
				Analytical Error		± 0.22	1.7915	Error Magnification	
							7%	Spreading Factor	

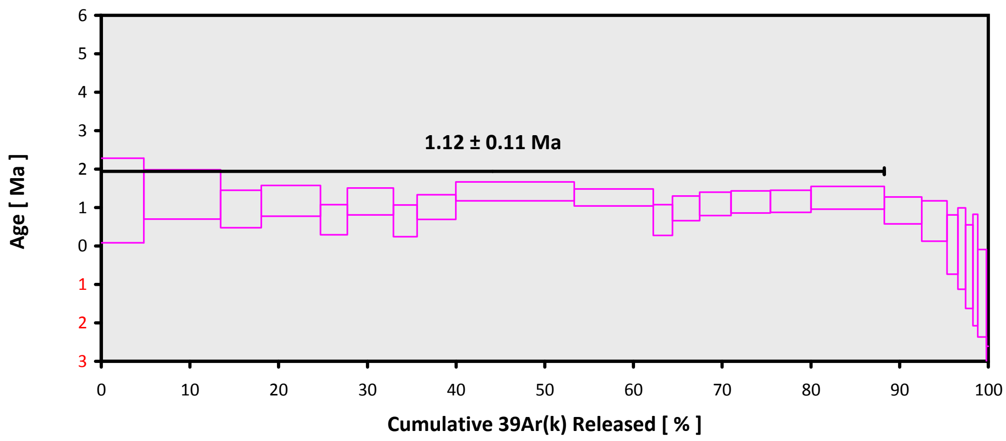

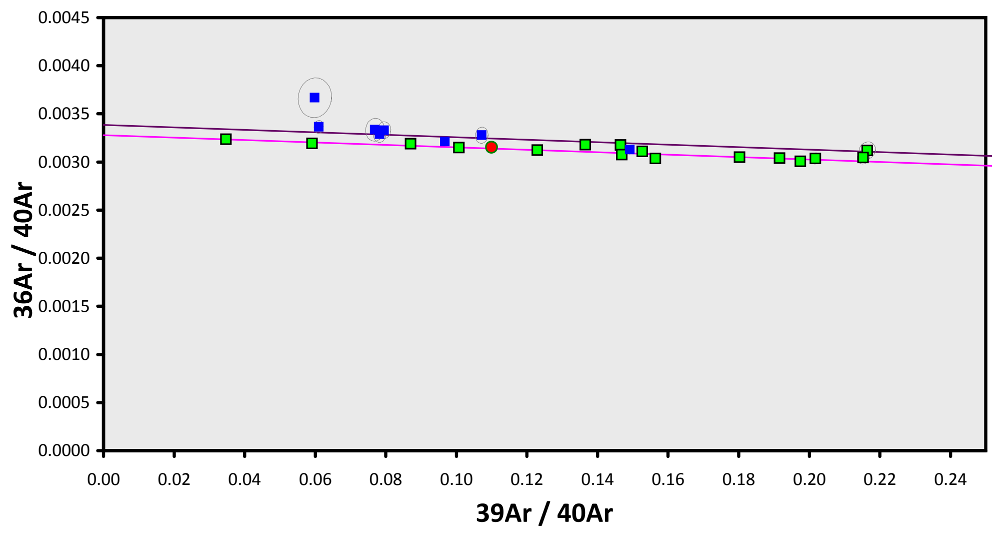

EXP#17D20095 > PS59-276-1-005 > Groundmass > O-CONNOR (16-22)
ARTIC OCEAN > GAKKEL RIDGE
17-OSU-01 (1C11-17) > Incremental Heating > Dan Miggins

Information on Analysis and Constants Used in Calculations

Project = **O-CONNOR (16-22)**
Sample = **PS59-276-1-005**
Material = **Groundmass**
Location = **Gakkel Ridge**
Region = **Artic Ocean**
Analyst = **Dan Miggins**
Irradiation = **17-OSU-01 (1C11-17)**
Position = **X: 0 | Y: 0 | Z/H: 17.68106 mm**
FCT-NM Age = **28.201 ± 0.023 Ma**
FCT-NM Reference = **Kuiper et al (2008)**
FCT-NM 40Ar/39Ar Ratio = **9.52112 ± 0.00847**
FCT-NM J-value = **0.00165079 ± 0.00000147**
Air Shot 40Ar/36Ar = **302.6420 ± 0.3511**
Air Shot MDF = **0.99409266 ± 0.00064493 (LIN)**
Experiment Type = **Incremental Heating**
Extraction Method = **Bulk Laser Heating**
Heating = **64 sec**
Isolation = **3.00 min**
Instrument = **ARGUS-VI-D**
Preferred Age = **Plateau Age**
Age Classification = **Crystallization Age**
IGSN = Undefined
Rock Class = **Igneous>Volcanic**
Lithology = **Basaltic Lava**
Lat-Lon = Undefined - Undefined
Age Equations = **Min et al. (2000)**
Negative Intensities = **Allowed**
Collector Calibrations = **36Ar**
Decay 40K = **5.530 ± 0.048 E-10 1/a**
Decay 39Ar = **2.940 ± 0.016 E-07 1/h**
Decay 37Ar = **8.230 ± 0.012 E-04 1/h**
Decay 36Cl = **2.257 ± 0.015 E-06 1/a**
Decay 40K(EC,β^+) = **0.580 ± 0.009 E-10 1/a**
Decay 40K(β^-) = **4.950 ± 0.043 E-10 1/a**
Atmospheric 40/36(a) = **295.50**
Atmospheric 38/36(a) = **0.1869**
Production 39/37(ca) = **0.0006425 ± 0.0000059**
Production 38/37(ca) = **0.0001800 ± 0.0000173**
Production 36/37(ca) = **0.0002703 ± 0.0000005**
Production 40/39(k) = **0.000607 ± 0.000059**
Production 38/39(k) = **0.012077 ± 0.000011**
Production 36/38(cl) = **262.80 ± 1.71**
Scaling Ratio K/Ca = **0.430**
Abundance Ratio 40K/K = **1.1700 ± 0.0100 E-04**
Atomic Weight K = **39.0983 ± 0.0001 g**

Results

Results	40(a)/36(a) ± 2σ		40(r)/39(k) ± 2σ		Age ± 2σ (ka)		MSWD	39Ar(k) (%,n)	K/Ca ± 2σ
**Age Plateau**			0.07120	± 0.00872<br>± 12.25%	**212.5**	**± 26.0**<br>**± 12.25%**	1.22<br>24%	79.64<br>17	0.017 ± 0.009
					Full External Error	± 26.5	1.71	2σ Confidence Limit	
					Analytical Error	± 26.0	1.1047	Error Magnification	
**Total Fusion Age**			0.10702	± 0.01693<br>± 15.82%	**319.5**	**± 50.5**<br>**± 15.82%**		24	0.061 ± 0.000
					Full External Error	± 51.1			
					Analytical Error	± 50.5			
**Normal Isochron**	**295.68**	**± 1.57**<br>**± 0.53%**	0.06924	± 0.01709<br>± 24.68%	**206.7**	**± 51.0**<br>**± 24.68%**	1.29<br>20%	79.64<br>17	
					Full External Error	± 51.2	1.73	2σ Confidence Limit	
					Analytical Error	± 51.0	1.1337	Error Magnification	
**Inverse Isochron**<br>Clustered Points	**295.70**	**± 1.58**<br>**± 0.53%**	0.06938	± 0.01543<br>± 22.24%	**207.1**	**± 46.1**<br>**± 22.24%**	1.30<br>19%	79.64<br>17	
					Full External Error	± 46.3	1.73	2σ Confidence Limit	
					Analytical Error	± 46.0	1.1386	Error Magnification	
							3%	Spreading Factor	

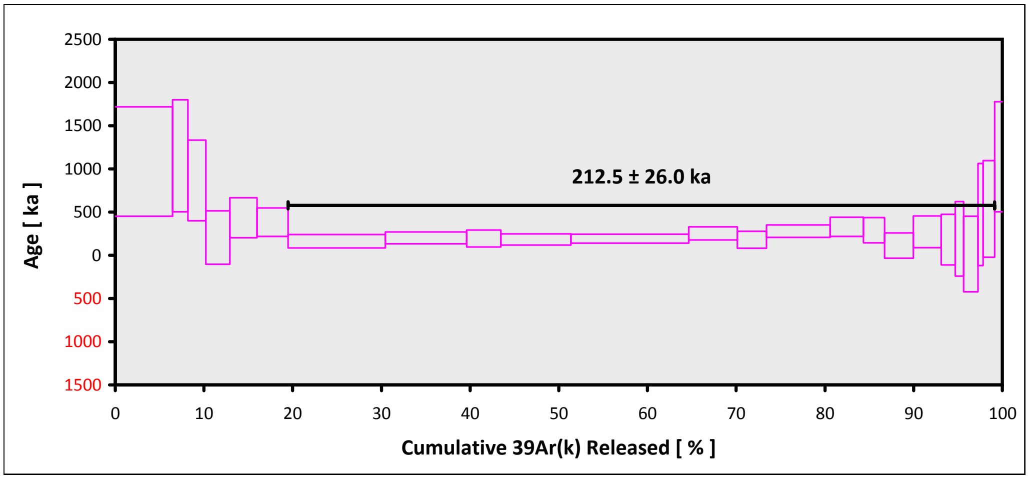

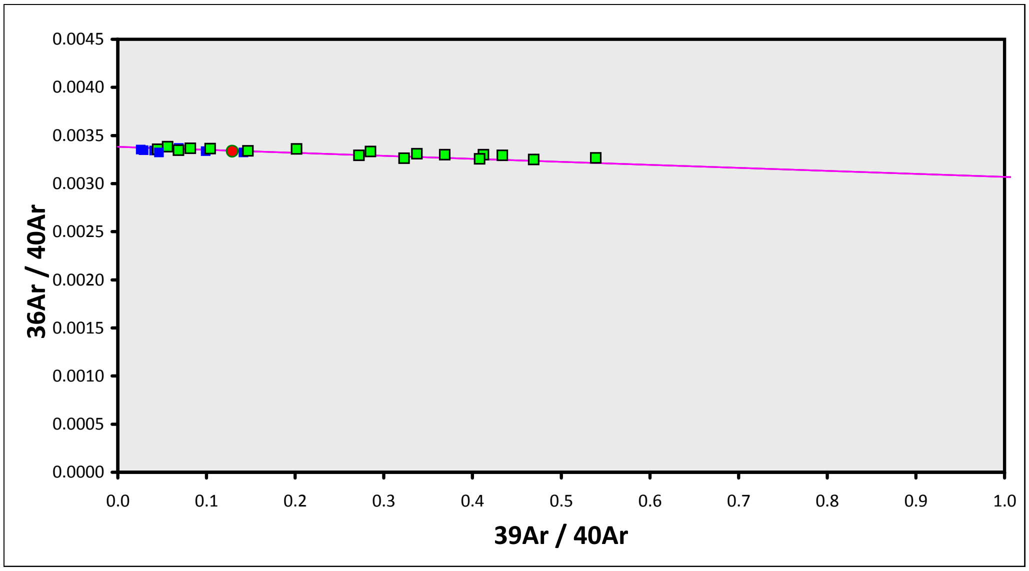

EXP#18D00611 > HLY0102-D66-32 > Groundmass > O-CONNOR (16-22)
ARTIC OCEAN > GAKKEL RIDGE
17-OSU-05 (5B11-17) > Incremental Heating > Dan Miggins

Information on Analysis and Constants Used in Calculations

Project = **O-CONNOR (16-22)**
Sample = **HLY0102-D66-32**
Material = **Groundmass**
Location = **Gakkel Ridge**
Region = **Artic Ocean**
Analyst = **Dan Miggins**
Irradiation = **17-OSU-05 (5B11-17)**
Position = **X: 0 | Y: 0 | Z/H: 16.47832 mm**
FCT-NM Age = **28.201 ± 0.023 Ma**
FCT-NM Reference = **Kuiper et al (2008)**
FCT-NM 40Ar/39Ar Ratio = **9.65222 ± 0.00791**
FCT-NM J-value = **0.00162837 ± 0.00000134**
Air Shot 40Ar/36Ar = **302.6400 ± 0.4600**
Air Shot MDF = **0.99409428 ± 0.00068817 (LIN)**
Experiment Type = **Incremental Heating**
Extraction Method = **Bulk Laser Heating**
Heating = **77 sec**
Isolation = **3.00 min**
Instrument = **ARGUS-VI-D**
Preferred Age = **Plateau Age**
Age Classification = **Crystallization Age**
IGSN = Undefined
Rock Class = Undefined
Lithology = **Basaltic Lava**
Lat-Lon = Undefined - Undefined
Age Equations = **Min et al. (2000)**
Negative Intensities = **Allowed**
Collector Calibrations = **36Ar**
Decay 40K = **5.530 ± 0.048 E-10 1/a**
Decay 39Ar = **2.940 ± 0.016 E-07 1/h**
Decay 37Ar = **8.230 ± 0.012 E-04 1/h**
Decay 36Cl = **2.257 ± 0.015 E-06 1/a**
Decay 40K(EC,β^+) = **0.580 ± 0.009 E-10 1/a**
Decay 40K(β^-) = **4.950 ± 0.043 E-10 1/a**
Atmospheric 40/36(a) = **295.50 ± 0.70**
Atmospheric 38/36(a) = **0.1869**
Production 39/37(ca) = **0.0006425 ± 0.0000059**
Production 38/37(ca) = **0.0001800 ± 0.0000173**
Production 36/37(ca) = **0.0002703 ± 0.0000005**
Production 40/39(k) = **0.000607 ± 0.000059**
Production 38/39(k) = **0.012077 ± 0.000011**
Production 36/38(cl) = **262.80 ± 1.71**
Scaling Ratio K/Ca = **0.430**
Abundance Ratio 40K/K = **1.1700 ± 0.0100 E-04**
Atomic Weight K = **39.0983 ± 0.0001 g**

Mostly atmospheric

Results

Results	40(a)/36(a) ± 2σ		40(r)/39(k) ± 2σ		Age ± 2σ (Ma)		MSWD	39Ar(k) (%,n)	K/Ca ± 2σ
**Age Plateau**			0.49948	± 0.00469<br>± 0.94%	**1.47**	**± 0.01<br>± 0.95%**	1.00<br>45%	94.84<br>19	0.097 ± 0.015
					Full External Error	± 0.04	1.67	2σ Confidence Limit	
					Analytical Error	± 0.01	1.0017	Error Magnification	
**Total Fusion Age**			0.49255	± 0.00534<br>± 1.09%	**1.45**	**± 0.02<br>± 1.10%**		24	0.108 ± 0.001
					Full External Error	± 0.04			
					Analytical Error	± 0.02			
**Normal Isochron**	**293.45**	**± 2.55<br>± 0.87%**	0.50495	± 0.00845<br>± 1.67%	**1.49**	**± 0.02<br>± 1.68%**	0.99<br>47%	94.84<br>19	
					Full External Error	± 0.04	1.69	2σ Confidence Limit	
					Analytical Error	± 0.02	1.0000	Error Magnification	
**Inverse Isochron**	**293.43**	**± 2.56<br>± 0.87%**	0.50532	± 0.00844<br>± 1.67%	**1.49**	**± 0.02<br>± 1.68%**	0.98<br>48%	94.84<br>19	
					Full External Error	± 0.04	1.69	2σ Confidence Limit	
					Analytical Error	± 0.02	1.0000	Error Magnification	
							40%	Spreading Factor	

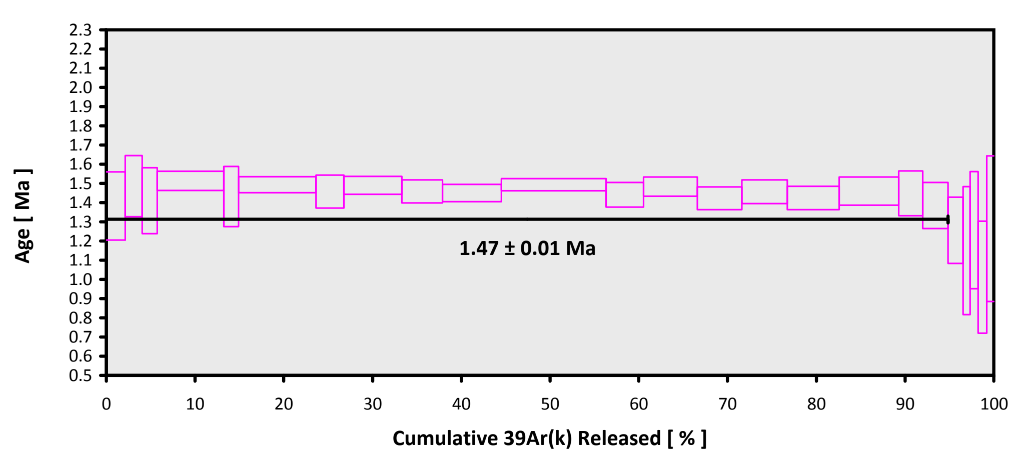

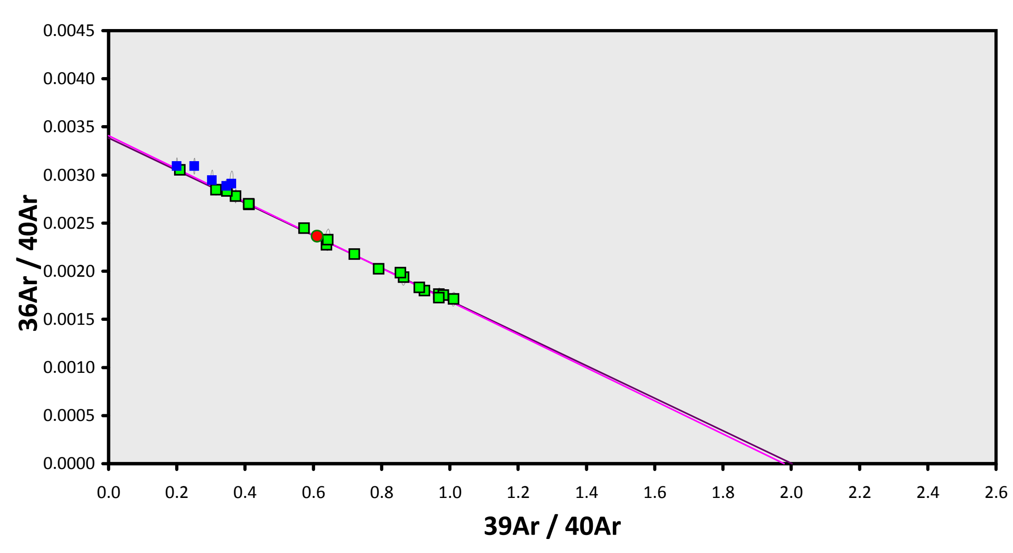

EXP#18D00212 > PS59-229-13 > Groundmass > O-CONNOR (16-22)
ARTIC OCEAN > GAKKEL RIDGE
17-OSU-05 (5B18-17) > Incremental Heating > Dan Miggins

Information on Analysis and Constants Used in Calculations

Project = **O-CONNOR (16-22)**
Sample = **PS59-229-13**
Material = **Groundmass**
Location = **Gakkel Ridge**
Region = **Artic Ocean**
Analyst = **Dan Miggins**
Irradiation = **17-OSU-05 (5B18-17)**
Position = **X: 0 | Y: 0 | Z/H: 26.49127 mm**
FCT-NM Age = **28.201 ± 0.023 Ma**
FCT-NM Reference = **Kuiper et al (2008)**
FCT-NM 40Ar/39Ar Ratio = **9.77942 ± 0.00792**
FCT-NM J-value = **0.00160719 ± 0.00000130**
Air Shot 40Ar/36Ar = **302.9580 ± 0.3969**
Air Shot MDF = **0.99383773 ± 0.00066118 (LIN)**
Experiment Type = **Incremental Heating**
Extraction Method = **Bulk Laser Heating**
Heating = **64 sec**
Isolation = **3.00 min**
Instrument = **ARGUS-VI-D**
Preferred Age = **Plateau Age**
Age Classification = **Crystallization Age**
IGSN = Undefined
Rock Class = **Igneous>Volcanic**
Lithology = **Basaltic Lava**
Lat-Lon = Undefined - Undefined
Age Equations = **Min et al. (2000)**
Negative Intensities = **Allowed**
Collector Calibrations = **36Ar**
Decay 40K = **5.530 ± 0.048 E-10 1/a**
Decay 39Ar = **2.940 ± 0.016 E-07 1/h**
Decay 37Ar = **8.230 ± 0.012 E-04 1/h**
Decay 36Cl = **2.257 ± 0.015 E-06 1/a**
Decay 40K(EC,β^+) = **0.580 ± 0.009 E-10 1/a**
Decay 40K(β^-) = **4.950 ± 0.043 E-10 1/a**
Atmospheric 40/36(a) = **291.31 ± 1.43**
Atmospheric 38/36(a) = **0.1869**
Production 39/37(ca) = **0.0006425 ± 0.0000059**
Production 38/37(ca) = **0.0001800 ± 0.0000173**
Production 36/37(ca) = **0.0002703 ± 0.0000005**
Production 40/39(k) = **0.000607 ± 0.000059**
Production 38/39(k) = **0.012077 ± 0.000011**
Production 36/38(cl) = **262.80 ± 1.71**
Scaling Ratio K/Ca = **0.430**
Abundance Ratio 40K/K = **1.1700 ± 0.0100 E-04**
Atomic Weight K = **39.0983 ± 0.0001 g**

Subatmospheric Initial 40Ar/36Ar = 291.31 ± 0.49 (%SD).

Results

Results	40(a)/36(a) ± 2σ		40(r)/39(k) ± 2σ		Age ± 2σ (Ma)		MSWD	39Ar(k) (%,n)	K/Ca ± 2σ
**Age Plateau** Overestimated			1.92560	± 0.48574<br>± 25.23%	**5.59**	**± 1.41**<br>**± 25.19%**	0.15<br>100%	61.56<br>14	0.0205 ± 0.0046
					Full External Error	± 1.41	1.78	2σ Confidence Limit	
					Analytical Error	± 1.41	1.0000	Error Magnification	
**Total Fusion Age**			5.77352	± 1.04369<br>± 18.08%	**16.70**	**± 3.01**<br>**± 17.99%**		22	0.0246 ± 0.0002
					Full External Error	± 3.03			
					Analytical Error	± 3.01			
**Normal Isochron**	**292.77**	**± 2.08**<br>**± 0.71%**	1.16228	± 1.13281<br>± 97.46%	**3.37**	**± 3.29**<br>**± 97.37%**	0.47<br>93%	61.56<br>14	
					Full External Error	± 3.29	1.82	2σ Confidence Limit	
					Analytical Error	± 3.29	1.0000	Error Magnification	
**Inverse Isochron** Clustered Points	**292.77**	**± 2.08**<br>**± 0.71%**	1.16218	± 0.73542<br>± 63.28%	**3.37**	**± 2.13**<br>**± 63.22%**	0.47<br>93%	61.56<br>14	
					Full External Error	± 2.13	1.82	2σ Confidence Limit	
					Analytical Error	± 2.13	1.0000	Error Magnification	
							1%	Spreading Factor	

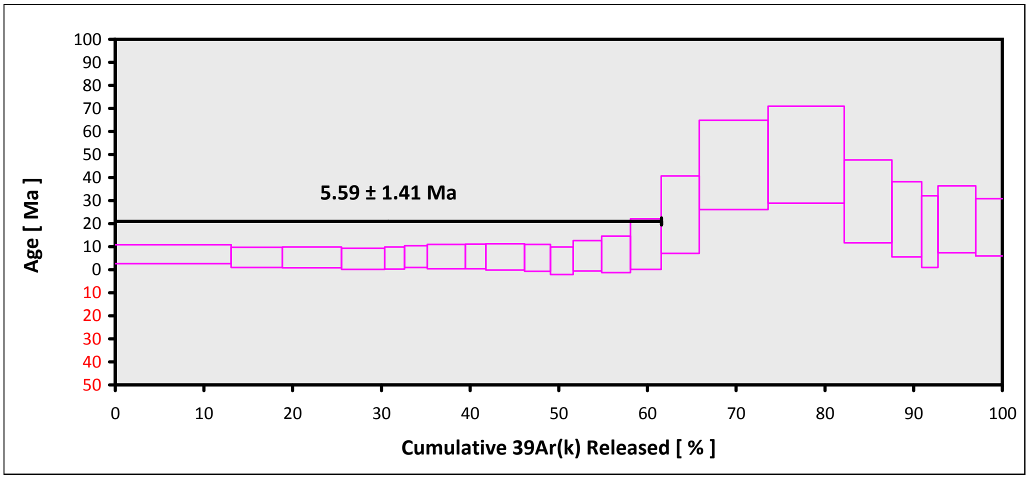

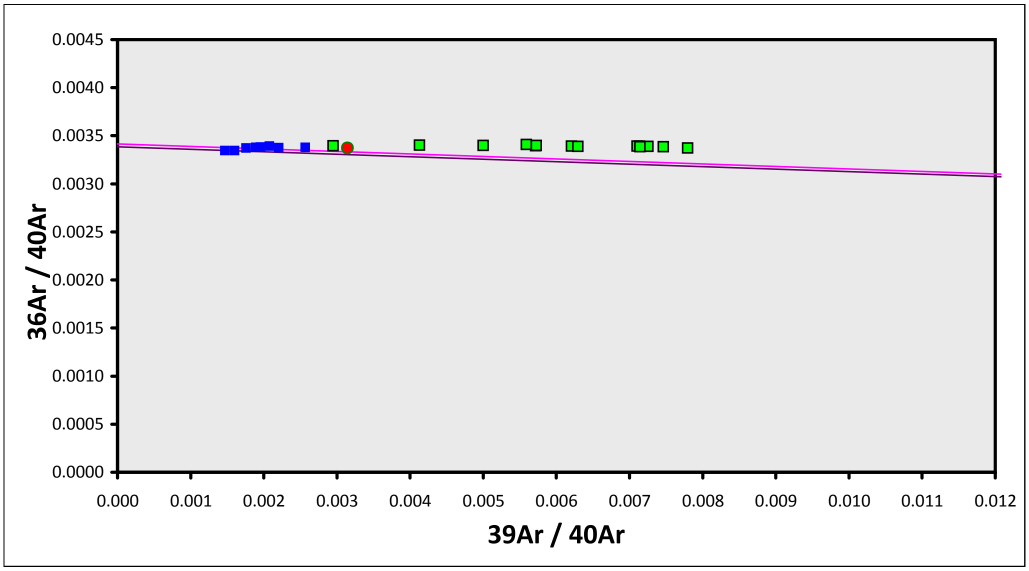

EXP#18D00651 > HLY0102-D55-9 > Groundmass > O-CONNOR (16-22) ARTIC OCEAN > GAKKEL RIDGE 17-OSU-05 (5B8-17) > Incremental Heating > Dan Miggins

Information on Analysis and Constants Used in Calculations

Project = **O-CONNOR (16-22)**
Sample = **HLY0102-D55-9**
Material = **Groundmass**
Location = **Gakkel Ridge**
Region = **Artic Ocean**
Analyst = **Dan Miggins**
Irradiation = **17-OSU-05 (5B8-17)**
Position = **X: 0 | Y: 0 | Z/H: 12.04491 mm**
FCT-NM Age = **28.201 ± 0.023 Ma**
FCT-NM Reference = **Kuiper et al (2008)**
FCT-NM 40Ar/39Ar Ratio = **9.61157 ± 0.00788**
FCT-NM J-value = **0.00163526 ± 0.00000134**
Air Shot 40Ar/36Ar = **302.8580 ± 0.4603**
Air Shot MDF = **0.99391835 ± 0.00068767 (LIN)**
Experiment Type = **Incremental Heating**
Extraction Method = **Bulk Laser Heating**
Heating = **77 sec**
Isolation = **3.00 min**
Instrument = **ARGUS-VI-D**
Preferred Age = **Plateau Age**
Age Classification = **Crystallization Age**
IGSN = Undefined
Rock Class = Undefined
Lithology = **Basaltic Lava**
Lat-Lon = Undefined - Undefined
Age Equations = **Min et al. (2000)**
Negative Intensities = **Allowed**
Collector Calibrations = **36Ar**
Decay 40K = **5.530 ± 0.048 E-10 1/a**
Decay 39Ar = **2.940 ± 0.016 E-07 1/h**
Decay 37Ar = **8.230 ± 0.012 E-04 1/h**
Decay 36Cl = **2.257 ± 0.015 E-06 1/a**
Decay 40K(EC,β^+) = **0.580 ± 0.009 E-10 1/a**
Decay 40K(β^-) = **4.950 ± 0.043 E-10 1/a**
Atmospheric 40/36(a) = **295.50 ± 0.70**
Atmospheric 38/36(a) = **0.1869**
Production 39/37(ca) = **0.0006425 ± 0.0000059**
Production 38/37(ca) = **0.0001800 ± 0.0000173**
Production 36/37(ca) = **0.0002703 ± 0.0000005**
Production 40/39(k) = **0.000607 ± 0.000059**
Production 38/39(k) = **0.012077 ± 0.000011**
Production 36/38(cl) = **262.80 ± 1.71**
Scaling Ratio K/Ca = **0.430**
Abundance Ratio 40K/K = **1.1700 ± 0.0100 E-04**
Atomic Weight K = **39.0983 ± 0.0001 g**

Mostly atmospheric

Results

Results	40(a)/36(a) ± 2σ		40(r)/39(k) ± 2σ		Age ± 2σ (Ma)		MSWD	39Ar(k) (%,n)	K/Ca ± 2σ
**Age Plateau**			1.44732	± 0.25526<br>± 17.64%	**4.27**	**± 0.75**<br>**± 17.62%**	0.85<br>60%	70.90<br>14	0.0069 ± 0.0018
					Full External Error	± 0.76	1.78	2σ Confidence Limit	
					Analytical Error	± 0.75	1.0000	Error Magnification	
**Total Fusion Age**			3.28476	± 0.28369<br>± 8.64%	**9.69**	**± 0.83**<br>**± 8.61%**		24	0.0125 ± 0.0001
					Full External Error	± 0.86			
					Analytical Error	± 0.83			
**Normal Isochron**	**281.31**	**± 7.93**<br>**± 2.82%**	7.40194	± 3.33979<br>± 45.12%	**21.76**	**± 9.76**<br>**± 44.85%**	0.52<br>90%	70.90<br>14	
					Full External Error	± 9.77	1.82	2σ Confidence Limit	
					Analytical Error	± 9.76	1.0000	Error Magnification	
**Inverse Isochron**<br>Clustered Points	**281.32**	**± 7.94**<br>**± 2.82%**	7.39809	± 2.86788<br>± 38.77%	**21.75**	**± 8.38**<br>**± 38.53%**	0.52<br>90%	70.90<br>14	
					Full External Error	± 8.39	1.82	2σ Confidence Limit	
					Analytical Error	± 8.38	1.0000	Error Magnification	
							1%	Spreading Factor	

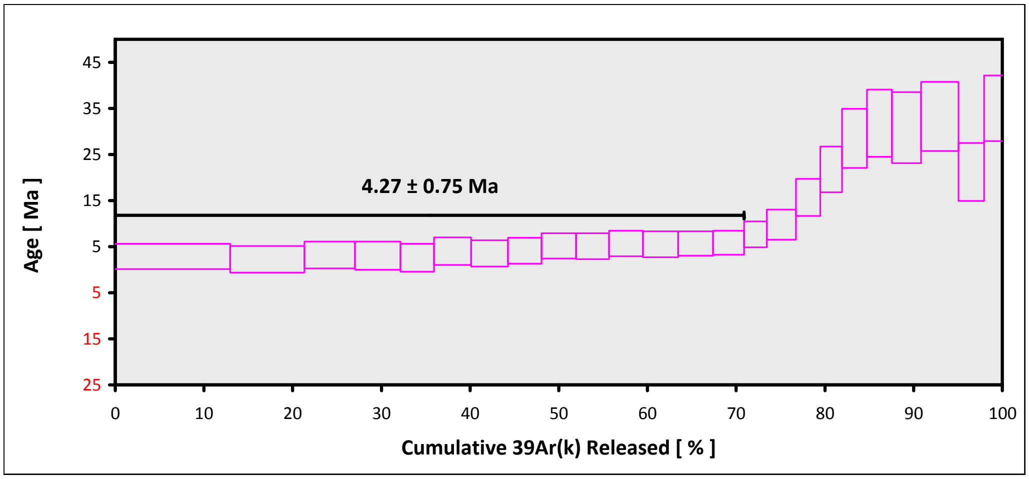

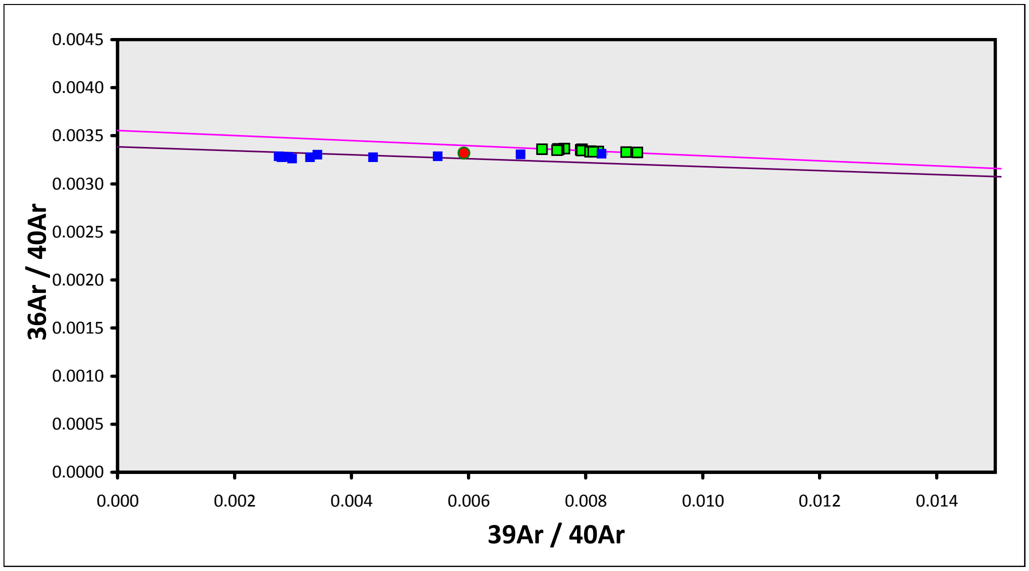

EXP#18D00544 > PS59-297-37 > Groundmass > O-CONNOR (16-22)
ARTIC OCEAN > GAKKEL RIDGE
17-OSU-05 (5B17-17) > Incremental Heating > Dan Miggins

Information on Analysis and Constants Used in Calculations

Project = **O-CONNOR (16-22)**
Sample = **PS59-297-37**
Material = **Groundmass**
Location = **Gakkel Ridge**
Region = **Artic Ocean**
Analyst = **Dan Miggins**
Irradiation = **17-OSU-05 (5B17-17)**
Position = **X: 0 | Y: 0 | Z/H: 24.94839 mm**
FCT-NM Age = **28.201 ± 0.023 Ma**
FCT-NM Reference = **Kuiper et al (2008)**
FCT-NM 40Ar/39Ar Ratio = **9.75664 ± 0.00790**
FCT-NM J-value = **0.00161094 ± 0.00000130**
Air Shot 40Ar/36Ar = **302.4190 ± 0.4597**
Air Shot MDF = **0.99427289 ± 0.00068867 (LIN)**
Experiment Type = **Incremental Heating**
Extraction Method = **Bulk Laser Heating**
Heating = **77 sec**
Isolation = **3.00 min**
Instrument = **ARGUS-VI-D**
Preferred Age = **Plateau Age**
Age Classification = **Crystallization Age**
IGSN = Undefined
Rock Class = Undefined
Lithology = **Basaltic Lava**
Lat-Lon = Undefined - Undefined
Age Equations = **Min et al. (2000)**
Negative Intensities = **Allowed**
Collector Calibrations = **36Ar**
Decay 40K = **5.530 ± 0.048 E-10 1/a**
Decay 39Ar = **2.940 ± 0.016 E-07 1/h**
Decay 37Ar = **8.230 ± 0.012 E-04 1/h**
Decay 36Cl = **2.257 ± 0.015 E-06 1/a**
Decay 40K(EC,β^+) = **0.580 ± 0.009 E-10 1/a**
Decay 40K(β^-) = **4.950 ± 0.043 E-10 1/a**
Atmospheric 40/36(a) = **295.50 ± 0.70**
Atmospheric 38/36(a) = **0.1869**
Production 39/37(ca) = **0.0006425 ± 0.0000059**
Production 38/37(ca) = **0.0001800 ± 0.0000173**
Production 36/37(ca) = **0.0002703 ± 0.0000005**
Production 40/39(k) = **0.000607 ± 0.000059**
Production 38/39(k) = **0.012077 ± 0.000011**
Production 36/38(cl) = **262.80 ± 1.71**
Scaling Ratio K/Ca = **0.430**
Abundance Ratio 40K/K = **1.1700 ± 0.0100 E-04**
Atomic Weight K = **39.0983 ± 0.0001 g**

Mostly atmospheric

Results

Results	40(a)/36(a) ± 2σ	40(r)/39(k) ± 2σ	Age ± 2σ (Ma)	MSWD	39Ar(k) (%,n)	K/Ca ± 2σ
**Age Plateau**		0.37810 ± 0.25075 ± 66.32%	**1.10 ± 0.73 ± 66.30%**	0.66	100.00	0.0089 ± 0.0012
				89%	24	
		Full External Error	± 0.73	1.59	2σ Confidence Limit	
		Analytical Error	± 0.73	1.0000	Error Magnification	
**Total Fusion Age**		0.73003 ± 0.37997 ± 52.05%	**2.13 ± 1.11 ± 52.02%**		24	0.0120 ± 0.0001
		Full External Error	± 1.11			
		Analytical Error	± 1.11			
**Normal Isochron**	**297.16 ± 0.93 ± 0.31%**	0.37913 ± 0.47314 ± 124.80%	**1.10 ± 1.38 ± 124.83%**	0.48	100.00	
				98%	24	
		Full External Error	± 1.38	1.60	2σ Confidence Limit	
		Analytical Error	± 1.38	1.0000	Error Magnification	
**Inverse Isochron** Clustered Points	**297.16 ± 0.93 ± 0.31%**	0.38037 ± 0.21075 ± 55.41%	**1.11 ± 0.61 ± 55.42%**	0.48	100.00	
				98%	24	
		Full External Error	± 0.61	1.60	2σ Confidence Limit	
		Analytical Error	± 0.61	1.0000	Error Magnification	
				0%	Spreading Factor	

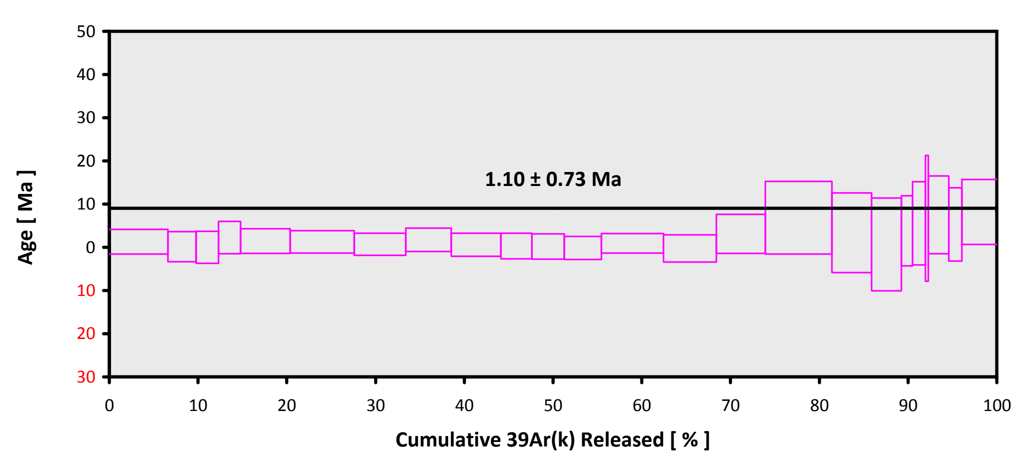

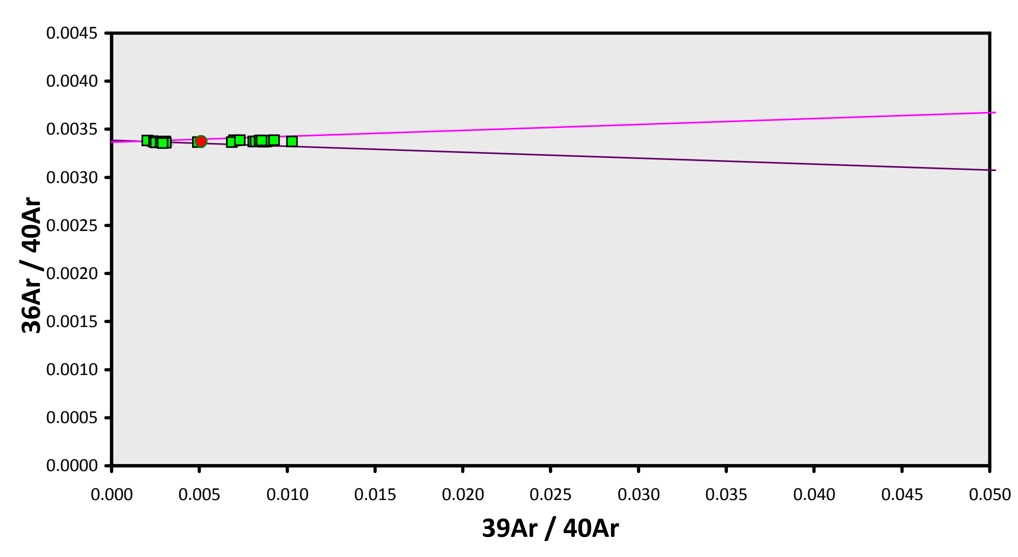

Supplement: Supplementary file 4 — Dataset 1 [file 41467_2021_27058_MOESM4_ESM.pdf]
