# Supplementary material for: Thermochemical anomalies in the upper mantle control Gakkel Ridge accretion
Source: Nat Commun. 2021 Nov 29;12:6962. doi: 10.1038/s41467-021-27058-1 (PMC8630051; doi:10.1038/s41467-021-27058-1)
Supplement: Supplementary file 5 — Dataset 2 [file 41467_2021_27058_MOESM5_ESM.pdf]

Supplementary Data 2

Full versions of ArArCalc age files

Relative Abundances			36Ar [fA]	%1σ	37Ar [fA]	%1σ	38Ar [fA]	%1σ	39Ar [fA]	%1σ	40Ar [fA]	%1σ	40(r)/39(k) ± 2σ	Age ± 2σ (ka)	40Ar(r) (%)	39Ar(k) (%)	K/Ca ± 2σ
18D00176	1.8 %	✓	4.440646	0.285	81.5783	3.426	1.496138	1.537	52.60371	0.085	1327.180	0.056	0.40827 ± 0.18711	1125.8 ± 515.8	1.62	14.68	0.2770 ± 0.0190
18D00178	1.9 %	✓	3.475289	0.293	86.0118	3.223	1.182357	2.022	41.66771	0.092	1035.882	0.071	0.37924 ± 0.18908	1045.8 ± 521.2	1.52	11.63	0.2080 ± 0.0134
18D00179	2.0 %	✓	2.116634	0.312	60.8084	4.718	0.707814	3.433	25.88632	0.119	628.482	0.118	0.30403 ± 0.19817	838.4 ± 546.4	1.25	7.22	0.1828 ± 0.0173
18D00181	2.2 %	✓	1.713710	0.329	70.2699	3.986	0.552052	4.245	21.18140	0.138	509.090	0.145	0.39214 ± 0.20677	1081.3 ± 570.0	1.63	5.91	0.1293 ± 0.0103
18D00182	2.4 %	✓	1.035775	0.392	52.8743	5.343	0.358995	6.295	14.07499	0.201	308.810	0.239	0.49520 ± 0.22735	1365.4 ± 626.6	2.25	3.92	0.1142 ± 0.0122
18D00184	2.7 %	✓	1.144201	0.372	69.9203	3.959	0.380910	6.231	14.78964	0.176	338.894	0.218	0.43124 ± 0.22681	1189.1 ± 625.2	1.88	4.12	0.0907 ± 0.0072
18D00185	3.0 %	✓	1.488142	0.342	102.8993	2.882	0.477863	4.988	16.57324	0.168	437.075	0.169	0.33552 ± 0.23949	925.2 ± 660.3	1.27	4.61	0.0690 ± 0.0040
18D00187	3.4 %	✓	1.037549	0.391	94.9300	2.935	0.389442	6.116	12.95130	0.213	303.906	0.243	0.37919 ± 0.24716	1045.6 ± 681.4	1.61	3.60	0.0584 ± 0.0034
18D00188	3.9 %	✓	1.197413	0.367	136.7854	2.271	0.389484	5.803	13.97534	0.195	346.048	0.213	0.22538 ± 0.24771	621.6 ± 683.0	0.90	3.88	0.0437 ± 0.0020
18D00190	4.5 %	✓	1.668163	0.331	227.1401	1.385	0.550107	4.321	16.76357	0.165	479.118	0.154	0.25933 ± 0.25631	715.2 ± 706.7	0.90	4.64	0.0315 ± 0.0009
18D00191	5.2 %	✓	1.522296	0.339	279.3834	1.233	0.490505	4.726	16.00688	0.175	432.613	0.171	0.32098 ± 0.25197	885.1 ± 694.7	1.17	4.42	0.0244 ± 0.0006
18D00193	6.0 %	✓	1.342165	0.349	295.6524	1.219	0.421703	5.622	13.57016	0.197	376.072	0.196	0.22934 ± 0.27245	632.5 ± 751.2	0.82	3.74	0.0195 ± 0.0005
18D00194	6.9 %	✓	1.498131	0.339	366.0062	1.020	0.447463	5.224	13.52604	0.206	417.557	0.177	0.30737 ± 0.29503	847.6 ± 813.4	0.98	3.71	0.0156 ± 0.0003
18D00196	7.9 %	✓	1.343827	0.352	406.4866	0.979	0.395682	6.065	12.99781	0.204	367.881	0.201	0.25439 ± 0.28770	701.6 ± 793.3	0.88	3.56	0.0135 ± 0.0003
18D00197	9.0 %	✓	1.169366	0.369	383.9321	1.003	0.391355	5.999	11.01082	0.244	318.776	0.234	0.36117 ± 0.31280	995.9 ± 862.3	1.22	3.01	0.0121 ± 0.0002
18D00199	10.3 %	✓	1.190885	0.366	350.0304	1.097	0.373392	6.591	9.95462	0.264	325.877	0.227	0.19766 ± 0.34974	545.1 ± 964.4	0.59	2.72	0.0120 ± 0.0003
18D00200	11.6 %	✓	1.630343	0.329	300.1561	1.200	0.400446	5.895	8.90375	0.298	458.712	0.161	0.10499 ± 0.47679	289.6 ± 1314.9	0.20	2.43	0.0125 ± 0.0003
18D00202	12.5 %	✓	1.128460	0.369	196.6788	1.615	0.305426	7.722	5.94971	0.420	320.113	0.231	0.40501 ± 0.56371	1116.8 ± 1553.9	0.74	1.63	0.0127 ± 0.0004
18D00203	13.4 %	✓	1.349065	0.348	173.2774	1.716	0.344190	6.774	5.59996	0.428	385.911	0.191	0.20030 ± 0.66812	552.4 ± 1842.3	0.28	1.53	0.0136 ± 0.0005
18D00205	14.6 %	✓	2.027228	0.320	199.6484	1.473	0.487642	4.619	6.70326	0.365	584.545	0.126	0.21929 ± 0.75597	604.8 ± 2084.5	0.25	1.84	0.0142 ± 0.0004
18D00206	16.0 %	✓	2.478757	0.303	197.6687	1.564	0.529790	4.293	6.83924	0.370	718.792	0.103	0.31337 ± 0.86398	864.2 ± 2382.0	0.29	1.88	0.0146 ± 0.0005
18D00208	17.6 %	✓	2.749686	0.302	218.2697	1.505	0.668003	3.591	6.94036	0.350	796.789	0.093	0.24874 ± 0.93869	686.0 ± 2588.2	0.21	1.90	0.0134 ± 0.0004
18D00209	19.3 %	✓	3.328765	0.295	274.0229	1.213	0.783539	2.990	7.13355	0.348	966.493	0.076	0.67990 ± 1.08382	1874.4 ± 2986.4	0.49	1.94	0.0109 ± 0.0003
18D00211	21.0 %	✓	2.407366	0.307	269.0359	1.235	0.535759	4.352	5.42849	0.471	696.770	0.106	1.30882 ± 1.08037	3606.5 ± 2974.1	0.99	1.47	0.0084 ± 0.0002
		Σ	44.483863	0.071	4893.4669	0.323	13.060057	0.882	361.03188	0.038	12881.384	0.028					

Information on Analysis and Constants Used in Calculations

Project = **O-CONNOR (16-22)**
Sample = **PS59-223-27**
Material = **Groundmass**
Location = **Gakkel Ridge**
Region = **Artic Ocean**
Analyst = **Dan Miggins**
Irradiation = **17-OSU-05 (5B35-17)**
Position = **X: 0 | Y: 0 | Z/H: 51.18569 mm**
FCT-NM Age = **28.201 ± 0.023 Ma**
FCT-NM Reference = **Kuiper et al (2008)**
FCT-NM 40Ar/39Ar Ratio = **10.30417 ± 0.00793**
FCT-NM J-value = **0.00152534 ± 0.00000117**
Air Shot 40Ar/36Ar = **302.9450 ± 0.3969**
Air Shot MDF = **0.99384820 ± 0.00066120 (LIN)**
Experiment Type = **Incremental Heating**
Extraction Method = **Bulk Laser Heating**
Heating = **64 sec**
Isolation = **3.00 min**
Instrument = **ARGUS-VI-D**
Preferred Age = **Plateau Age**
Age Classification = **Crystallization Age**
IGSN = Undefined
Rock Class = Undefined
Lithology = **Basaltic Lava**
Lat-Lon = Undefined - Undefined

Age Equations = **Min et al. (2000)**
Negative Intensities = **Allowed**
Collector Calibrations = **36Ar**
Decay 40K = **5.530 ± 0.048 E-10 1/a**
Decay 39Ar = **2.940 ± 0.016 E-07 1/h**
Decay 37Ar = **8.230 ± 0.012 E-04 1/h**
Decay 36Cl = **2.257 ± 0.015 E-06 1/a**
Decay 40K(EC,β⁺) = **0.580 ± 0.009 E-10 1/a**
Decay 40K(β⁻) = **4.950 ± 0.043 E-10 1/a**
Atmospheric 40/36(a) = **295.50 ± 0.70**
Atmospheric 38/36(a) = **0.1869**
Production 39/37(ca) = **0.0006425 ± 0.0000059**
Production 38/37(ca) = **0.0001800 ± 0.0000173**
Production 36/37(ca) = **0.0002703 ± 0.0000005**
Production 40/39(k) = **0.000607 ± 0.000059**
Production 38/39(k) = **0.012077 ± 0.000011**
Production 36/38(cl) = **262.80 ± 1.71**
Scaling Ratio K/Ca = **0.430**
Abundance Ratio 40K/K = **1.1700 ± 0.0100 E-04**
Atomic Weight K = **39.0983 ± 0.0001 g**

Results	40(a)/36(a) ± 2σ	40(r)/39(k) ± 2σ	Age ± 2σ (ka)	MSWD	39Ar(k) (%,n)	K/Ca ± 2σ
**Age Plateau**		0.34363 ± 0.05808 ± 16.90%	**947.6 ± 160.1 ± 16.90%**	0.51	100.00 24	0.0130 ± 0.0020
			Full External Error ± 161.6	1.59	2σ Confidence Limit	
			Analytical Error ± 160.1	1.0000	Error Magnification	
**Total Fusion Age**		0.35504 ± 0.06796 ± 19.14%	**979.0 ± 187.4 ± 19.14%**		24	0.0314 ± 0.0002
			Full External Error ± 188.7			
			Analytical Error ± 187.4			
**Normal Isochron**	**295.63 ± 1.03 ± 0.35%**	0.33268 ± 0.11028 ± 33.15%	**917.4 ± 304.0 ± 33.14%**	0.73 81%	100.00 24	
			Full External Error ± 304.7	1.60	2σ Confidence Limit	
			Analytical Error ± 304.0	1.0000	Error Magnification	
				3	Number of Iterations	
				0.0000016383	Convergence	
**Inverse Isochron** Clustered Points	**295.64 ± 1.03 ± 0.35%**	0.33253 ± 0.09153 ± 27.53%	**917.0 ± 252.3 ± 27.52%**	0.73 81%	100.00 24	
			Full External Error ± 253.2	1.60	2σ Confidence Limit	
			Analytical Error ± 252.3	1.0000	Error Magnification	
**Notes**				3	Number of Iterations	
Mostly atmospheric				0.0019545024	Convergence	
				1%	Spreading Factor	

Incremental Heating			36Ar(a) [fA]	37Ar(ca) [fA]	38Ar(cl) [fA]	39Ar(k) [fA]	40Ar(r) [fA]	Age ± 2σ (ka)	40Ar(r) (%)	39Ar(k) (%)	K/Ca ± 2σ
18D00176	1.8 %	✓	4.418588	81.5783	0.0209578	52.55129	21.45515	1125.8 ± 515.8	1.62	14.68	0.2770 ± 0.0190
18D00178	1.9 %	✓	3.452032	86.0118	0.0191363	41.61244	15.78130	1045.8 ± 521.2	1.52	11.63	0.2080 ± 0.0134
18D00179	2.0 %	✓	2.100197	60.8084	0.0000000	25.84725	7.85830	838.4 ± 546.4	1.25	7.22	0.1828 ± 0.0173
18D00181	2.2 %	✓	1.694716	70.2699	0.0000000	21.13625	8.28832	1081.3 ± 570.0	1.63	5.91	0.1293 ± 0.0103
18D00182	2.4 %	✓	1.021484	52.8743	0.0000000	14.04102	6.95316	1365.4 ± 626.6	2.25	3.92	0.1142 ± 0.0122
18D00184	2.7 %	✓	1.125301	69.9203	0.0000000	14.74472	6.35855	1189.1 ± 625.2	1.88	4.12	0.0907 ± 0.0072
18D00185	3.0 %	✓	1.460328	102.8993	0.0000000	16.50713	5.53844	925.2 ± 660.3	1.27	4.61	0.0690 ± 0.0040
18D00187	3.4 %	✓	1.011879	94.9300	0.0275579	12.89031	4.88787	1045.6 ± 681.4	1.61	3.60	0.0584 ± 0.0034
18D00188	3.9 %	✓	1.160440	136.7854	0.0000000	13.88746	3.12998	621.6 ± 683.0	0.90	3.88	0.0437 ± 0.0020
18D00190	4.5 %	✓	1.606764	227.1401	0.0082267	16.61763	4.30950	715.2 ± 706.7	0.90	4.64	0.0315 ± 0.0009
18D00191	5.2 %	✓	1.446778	279.3834	0.0000000	15.82738	5.08025	885.1 ± 694.7	1.17	4.42	0.0244 ± 0.0006
18D00193	6.0 %	✓	1.262250	295.6524	0.0000000	13.38020	3.06866	632.5 ± 751.2	0.82	3.74	0.0195 ± 0.0005
18D00194	6.9 %	✓	1.399199	366.0062	0.0000000	13.29089	4.08528	847.6 ± 813.4	0.98	3.71	0.0156 ± 0.0003
18D00196	7.9 %	✓	1.233954	406.4866	0.0000000	12.73664	3.24011	701.6 ± 793.3	0.88	3.56	0.0135 ± 0.0003
18D00197	9.0 %	✓	1.065589	383.9321	0.0000000	10.76414	3.88764	995.9 ± 862.3	1.22	3.01	0.0121 ± 0.0002
18D00199	10.3 %	✓	1.096272	350.0304	0.0000000	9.72973	1.92321	545.1 ± 964.4	0.59	2.72	0.0120 ± 0.0003
18D00200	11.6 %	✓	1.549211	300.1561	0.0000000	8.71090	0.91456	289.6 ± 1314.9	0.20	2.43	0.0125 ± 0.0003
18D00202	12.5 %	✓	1.075298	196.6788	0.0000000	5.82334	2.35848	1116.8 ± 1553.9	0.74	1.63	0.0127 ± 0.0004
18D00203	13.4 %	✓	1.302227	173.2774	0.0033277	5.48863	1.09936	552.4 ± 1842.3	0.28	1.53	0.0136 ± 0.0005
18D00205	14.6 %	✓	1.973262	199.6484	0.0034970	6.57498	1.44185	604.8 ± 2084.5	0.25	1.84	0.0142 ± 0.0004
18D00206	16.0 %	✓	2.425327	197.6687	0.0000000	6.71224	2.10339	864.2 ± 2382.0	0.29	1.88	0.0146 ± 0.0005
18D00208	17.6 %	✓	2.690671	218.2697	0.0437027	6.80013	1.69147	686.0 ± 2588.2	0.21	1.90	0.0134 ± 0.0004
18D00209	19.3 %	✓	3.254681	274.0229	0.0418893	6.95749	4.73040	1874.4 ± 2986.4	0.49	1.94	0.0109 ± 0.0003
18D00211	21.0 %	✓	2.334645	269.0359	0.0000000	5.25563	6.87869	3606.5 ± 2974.1	0.99	1.47	0.0084 ± 0.0002
		Σ	43.161094	4893.4669	0.1682955	357.88783	127.06393				

Information on Analysis

Project = **O-CONNOR (16-22)**
Sample = **PS59-223-27**
Material = **Groundmass**
Location = **Gakkel Ridge**
Region = **Artic Ocean**
Analyst = **Dan Miggins**
Irradiation = **17-OSU-05 (5B35-17)**
J = **0.00152534 ± 0.00000117**
FCT-NM = **28.201 ± 0.023 Ma**

Results	40(r)/39(k) ± 2σ		Age ± 2σ (ka)		MSWD	39Ar(k) (%,n)	K/Ca ± 2σ
**Age Plateau**	0.34363	± 0.05808<br>± 16.90%	**947.6**	**± 160.1**<br>**± 16.90%**	0.51<br>98%	100.00<br>24	0.0130 ± 0.0020
			Full External Error	± 161.6	1.59	2σ Confidence Limit	
			Analytical Error	± 160.1	1.0000	Error Magnification	
**Total Fusion Age**	0.35504	± 0.06796<br>± 19.14%	**979.0**	**± 187.4**<br>**± 19.14%**		24	0.0314 ± 0.0002
			Full External Error	± 188.7			
			Analytical Error	± 187.4			

Normal Isochron			39(k)/36(a) ± 2σ	40(a+r)/36(a) ± 2σ	r.i.
18D00176	1.8 %	✓	11.89 ± 0.07	300.36 ± 1.76	0.9414
18D00178	1.9 %	✓	12.05 ± 0.07	300.07 ± 1.82	0.9281
18D00179	2.0 %	✓	12.31 ± 0.08	299.24 ± 2.02	0.8768
18D00181	2.2 %	✓	12.47 ± 0.09	300.39 ± 2.20	0.8481
18D00182	2.4 %	✓	13.75 ± 0.12	302.31 ± 2.84	0.7701
18D00184	2.7 %	✓	13.10 ± 0.11	301.15 ± 2.66	0.7897
18D00185	3.0 %	✓	11.30 ± 0.09	299.29 ± 2.34	0.8128
18D00187	3.4 %	✓	12.74 ± 0.12	300.33 ± 2.85	0.7610
18D00188	3.9 %	✓	11.97 ± 0.10	298.20 ± 2.63	0.7794
18D00190	4.5 %	✓	10.34 ± 0.08	298.18 ± 2.27	0.8241
18D00191	5.2 %	✓	10.94 ± 0.09	299.01 ± 2.40	0.8121
18D00193	6.0 %	✓	10.60 ± 0.09	297.93 ± 2.54	0.7841
18D00194	6.9 %	✓	9.50 ± 0.08	298.42 ± 2.45	0.7831
18D00196	7.9 %	✓	10.32 ± 0.09	298.13 ± 2.64	0.7856
18D00197	9.0 %	✓	10.10 ± 0.10	299.15 ± 2.86	0.7462
18D00199	10.3 %	✓	8.88 ± 0.09	297.25 ± 2.78	0.7283
18D00200	11.6 %	✓	5.62 ± 0.05	296.09 ± 2.29	0.6848
18D00202	12.5 %	✓	5.42 ± 0.06	297.69 ± 2.73	0.5848
18D00203	13.4 %	✓	4.21 ± 0.05	296.34 ± 2.45	0.5685
18D00205	14.6 %	✓	3.33 ± 0.03	296.23 ± 2.10	0.6191
18D00206	16.0 %	✓	2.77 ± 0.03	296.37 ± 1.94	0.6033
18D00208	17.6 %	✓	2.53 ± 0.02	296.13 ± 1.92	0.6265
18D00209	19.3 %	✓	2.14 ± 0.02	296.95 ± 1.85	0.6253
18D00211	21.0 %	✓	2.25 ± 0.03	298.45 ± 2.01	0.5183

Results	40(a)/36(a) ± 2σ		40(r)/39(k) ± 2σ		Age ± 2σ (ka)		MSWD
Normal Isochron	**295.63**	**± 1.03**<br>**± 0.35%**	0.33268	± 0.11028<br>± 33.15%	**917.4**	**± 304.0**<br>**± 33.14%**	0.73<br>81%
					Full External Error	± 304.7	
					Analytical Error	± 304.0	

Statistics				
	2σ Confidence Limit	1.60	Convergence	0.000001638325
	Error Magnification	1.0000	Number of Iterations	3
	Number of Data Points	24	Calculated Line	Weighted York-2

Inverse Isochron			39(k)/40(a+r) ± 2σ		36(a)/40(a+r) ± 2σ		r.i.
18D00176	1.8 %	✓	0.0395972	± 0.0000804	0.00332939	± 0.00001947	0.1049
18D00178	1.9 %	✓	0.0401720	± 0.0000936	0.00333254	± 0.00002026	0.1441
18D00179	2.0 %	✓	0.0411275	± 0.0001379	0.00334178	± 0.00002254	0.2445
18D00181	2.2 %	✓	0.0415188	± 0.0001667	0.00332900	± 0.00002433	0.2872
18D00182	2.4 %	✓	0.0454694	± 0.0002845	0.00330790	± 0.00003108	0.3897
18D00184	2.7 %	✓	0.0435095	± 0.0002444	0.00332060	± 0.00002932	0.3829
18D00185	3.0 %	✓	0.0377681	± 0.0001808	0.00334121	± 0.00002613	0.3051
18D00187	3.4 %	✓	0.0424165	± 0.0002748	0.00332967	± 0.00003164	0.3837
18D00188	3.9 %	✓	0.0401325	± 0.0002332	0.00335349	± 0.00002959	0.3555
18D00190	4.5 %	✓	0.0346845	± 0.0001576	0.00335366	± 0.00002550	0.2752
18D00191	5.2 %	✓	0.0365864	± 0.0001805	0.00334435	± 0.00002681	0.2949
18D00193	6.0 %	✓	0.0355796	± 0.0002000	0.00335648	± 0.00002864	0.3215
18D00194	6.9 %	✓	0.0318307	± 0.0001755	0.00335098	± 0.00002748	0.2768
18D00196	7.9 %	✓	0.0346223	± 0.0002015	0.00335429	± 0.00002967	0.3141
18D00197	9.0 %	✓	0.0337678	± 0.0002322	0.00334282	± 0.00003195	0.3331
18D00199	10.3 %	✓	0.0298576	± 0.0002115	0.00336412	± 0.00003149	0.3115
18D00200	11.6 %	✓	0.0189901	± 0.0001316	0.00337735	± 0.00002612	0.1936
18D00202	12.5 %	✓	0.0181918	± 0.0001777	0.00335916	± 0.00003078	0.2377
18D00203	13.4 %	✓	0.0142227	± 0.0001359	0.00337445	± 0.00002788	0.1853
18D00205	14.6 %	✓	0.0112481	± 0.0000888	0.00337575	± 0.00002391	0.1142
18D00206	16.0 %	✓	0.0093383	± 0.0000732	0.00337419	± 0.00002212	0.0821
18D00208	17.6 %	✓	0.0085345	± 0.0000633	0.00337691	± 0.00002188	0.0716
18D00209	19.3 %	✓	0.0071987	± 0.0000528	0.00336753	± 0.00002103	0.0511
18D00211	21.0 %	✓	0.0075429	± 0.0000755	0.00335069	± 0.00002252	0.0668

Results	40(a)/36(a)	± 2σ	40(r)/39(k)	± 2σ	Age (ka)	± 2σ	MSWD
**Inverse Isochron**	**295.64**	**± 1.03**	0.33253	± 0.09153	**917.0**	**± 252.3**	0.73
Clustered Points		**± 0.35%**		± 27.53%		**± 27.52%**	81%
					Full External Error	± 253.2	
					Analytical Error	± 252.3	

Statistics				
2σ Confidence Limit	1.60	Convergence	0.0019545024
Error Magnification	1.0000	Number of Iterations	3
Number of Data Points	24	Calculated Line	Weighted York-2
Spreading Factor	1.3%		

**Degassing Patterns**			36Ar(a) [fA]	%1σ	36Ar(c) [fA]	%1σ	36Ar(ca) [fA]	%1σ	36Ar(cl) [fA]	%1σ	37Ar(ca) [fA]	%1σ	38Ar(a) [fA]	%1σ	38Ar(c) [fA]	%1σ	38Ar(k) [fA]	%1σ	38Ar(ca) [fA]	%1σ	38Ar(cl) [fA]	%1σ	39Ar(k) [fA]	%1σ	39Ar(ca) [fA]	%1σ	40Ar(r) [fA]	%1σ	40Ar(a) [fA]	%1σ	40Ar(c) [fA]	%1σ	40Ar(k) [fA]	%1σ
18D00176	1.8 %	✓	4.418588	0.29	0.0000000	0.00	0.0220506	3.43	0.0000080	110.58	81.5783	3.43	0.8258340	0.29	0.0000000	0.00	0.6346620	0.12	0.0146841	10.22	0.0209578	110.58	52.55129	0.08	0.0524141	3.55	21.45515	22.91	1305.693	0.37	0.0000000	0.00	0.0318986	9.65
18D00178	1.9 %	✓	3.452032	0.30	0.0000000	0.00	0.0232490	3.23	0.0000073	125.68	86.0118	3.22	0.6451848	0.30	0.0000000	0.00	0.5025535	0.13	0.0154821	10.15	0.0191363	125.68	41.61244	0.09	0.0552626	3.35	15.78130	24.93	1020.076	0.38	0.0000000	0.00	0.0252588	9.65
18D00179	2.0 %	✓	2.100197	0.32	0.0000000	0.00	0.0164365	4.72	0.0000000	0.00	60.8084	4.72	0.3925268	0.32	0.0000000	0.00	0.3121573	0.15	0.0109455	10.72	0.0000000	0.00	25.84725	0.12	0.0390694	4.81	7.85830	32.59	620.608	0.40	0.0000000	0.00	0.0156893	9.65
18D00181	2.2 %	✓	1.694716	0.34	0.0000000	0.00	0.0189940	3.99	0.0000000	0.00	70.2699	3.99	0.3167425	0.34	0.0000000	0.00	0.2552625	0.17	0.0126486	10.42	0.0000000	0.00	21.13625	0.14	0.0451484	4.09	8.28832	26.36	500.789	0.41	0.0000000	0.00	0.0128297	9.65
18D00182	2.4 %	✓	1.021484	0.40	0.0000000	0.00	0.0142919	5.35	0.0000000	0.00	52.8743	5.34	0.1909153	0.40	0.0000000	0.00	0.1695734	0.22	0.0095174	11.01	0.0000000	0.00	14.04102	0.20	0.0339717	5.42	6.95316	22.95	301.848	0.47	0.0000000	0.00	0.0085229	9.65
18D00184	2.7 %	✓	1.125301	0.38	0.0000000	0.00	0.0188995	3.96	0.0000000	0.00	69.9203	3.96	0.2103188	0.38	0.0000000	0.00	0.1780720	0.20	0.0125857	10.41	0.0000000	0.00	14.74472	0.18	0.0449238	4.06	6.35855	26.30	332.527	0.45	0.0000000	0.00	0.0089500	9.65
18D00185	3.0 %	✓	1.460328	0.35	0.0000000	0.00	0.0278137	2.89	0.0000000	0.00	102.8993	2.88	0.2729353	0.35	0.0000000	0.00	0.1993566	0.19	0.0185219	10.05	0.0000000	0.00	16.50713	0.17	0.0661128	3.03	5.53844	35.69	431.527	0.42	0.0000000	0.00	0.0100198	9.65
18D00187	3.4 %	✓	1.011879	0.41	0.0000000	0.00	0.0256596	2.94	0.0000106	86.72	94.9300	2.94	0.1891202	0.41	0.0000000	0.00	0.1556763	0.23	0.0170874	10.07	0.0275579	86.73	12.89031	0.21	0.0609925	3.08	4.88787	32.59	299.010	0.47	0.0000000	0.00	0.0078244	9.65
18D00188	3.9 %	✓	1.160440	0.39	0.0000000	0.00	0.0369731	2.28	0.0000000	0.00	136.7854	2.27	0.2168862	0.39	0.0000000	0.00	0.1677188	0.22	0.0246214	9.89	0.0000000	0.00	13.88746	0.20	0.0878846	2.45	3.12998	54.95	342.910	0.45	0.0000000	0.00	0.0084297	9.65
18D00190	4.5 %	✓	1.606764	0.35	0.0000000	0.00	0.0613960	1.40	0.0000032	293.30	227.1401	1.38	0.3003043	0.35	0.0000000	0.00	0.2006912	0.19	0.0408852	9.73	0.0082267	293.30	16.61763	0.17	0.1459375	1.66	4.30950	49.42	474.799	0.42	0.0000000	0.00	0.0100869	9.65
18D00191	5.2 %	✓	1.446778	0.36	0.0000000	0.00	0.0755173	1.24	0.0000000	0.00	279.3834	1.23	0.2704028	0.36	0.0000000	0.00	0.1911473	0.20	0.0502890	9.71	0.0000000	0.00	15.82738	0.18	0.1795039	1.54	5.08025	39.25	427.523	0.43	0.0000000	0.00	0.0096072	9.65
18D00193	6.0 %	✓	1.262250	0.38	0.0000000	0.00	0.0799149	1.23	0.0000000	0.00	295.6524	1.22	0.2359146	0.38	0.0000000	0.00	0.1615927	0.22	0.0532174	9.71	0.0000000	0.00	13.38020	0.20	0.1899567	1.53	3.06866	59.40	372.995	0.45	0.0000000	0.00	0.0081218	9.65
18D00194	6.9 %	✓	1.399199	0.37	0.0000000	0.00	0.0989315	1.03	0.0000000	0.00	366.0062	1.02	0.2615103	0.37	0.0000000	0.00	0.1605140	0.23	0.0658811	9.68	0.0000000	0.00	13.29089	0.21	0.2351590	1.37	4.08528	47.99	413.463	0.44	0.0000000	0.00	0.0080676	9.65
18D00196	7.9 %	✓	1.233954	0.39	0.0000000	0.00	0.1098733	0.99	0.0000000	0.00	406.4866	0.98	0.2306260	0.39	0.0000000	0.00	0.1538204	0.23	0.0731676	9.68	0.0000000	0.00	12.73664	0.21	0.2611676	1.34	3.24011	56.55	364.633	0.46	0.0000000	0.00	0.0077311	9.65
18D00197	9.0 %	✓	1.065589	0.42	0.0000000	0.00	0.1037768	1.02	0.0000000	0.00	383.9321	1.00	0.1991586	0.42	0.0000000	0.00	0.1299986	0.27	0.0691078	9.68	0.0000000	0.00	10.76414	0.25	0.2466764	1.36	3.88764	43.30	314.882	0.48	0.0000000	0.00	0.0065338	9.65
18D00199	10.3 %	✓	1.096272	0.41	0.0000000	0.00	0.0946132	1.11	0.0000000	0.00	350.0304	1.10	0.2048932	0.41	0.0000000	0.00	0.1175059	0.29	0.0630055	9.69	0.0000000	0.00	9.72973	0.27	0.2248946	1.43	1.92321	88.47	323.948	0.47	0.0000000	0.00	0.0059059	9.65
18D00200	11.6 %	✓	1.549211	0.35	0.0000000	0.00	0.0811322	1.21	0.0000000	0.00	300.1561	1.20	0.2895476	0.35	0.0000000	0.00	0.1052015	0.32	0.0540281	9.70	0.0000000	0.00	8.71090	0.31	0.1928503	1.51	0.91456	227.06	457.792	0.42	0.0000000	0.00	0.0052875	9.65
18D00202	12.5 %	✓	1.075298	0.40	0.0000000	0.00	0.0531623	1.62	0.0000000	0.00	196.6788	1.62	0.2009732	0.40	0.0000000	0.00	0.0703285	0.44	0.0354022	9.76	0.0000000	0.00	5.82334	0.43	0.1263661	1.86	2.35848	69.59	317.751	0.46	0.0000000	0.00	0.0035348	9.66
18D00203	13.4 %	✓	1.302227	0.37	0.0000000	0.00	0.0468369	1.72	0.0000013	707.21	173.2774	1.72	0.2433862	0.37	0.0000000	0.00	0.0662861	0.45	0.0311899	9.78	0.0033277	707.21	5.48863	0.44	0.1113307	1.95	1.09936	166.78	384.808	0.44	0.0000000	0.00	0.0033316	9.66
18D00205	14.6 %	✓	1.973262	0.33	0.0000000	0.00	0.0539650	1.48	0.0000013	652.85	199.6484	1.47	0.3688026	0.33	0.0000000	0.00	0.0794061	0.38	0.0359367	9.74	0.0034970	652.85	6.57498	0.37	0.1282741	1.74	1.44185	172.37	583.099	0.41	0.0000000	0.00	0.0039910	9.66
18D00206	16.0 %	✓	2.425327	0.31	0.0000000	0.00	0.0534299	1.57	0.0000000	0.00	197.6687	1.56	0.4532937	0.31	0.0000000	0.00	0.0810637	0.39	0.0355804	9.76	0.0000000	0.00	6.71224	0.38	0.1270022	1.81	2.10339	137.85	716.684	0.39	0.0000000	0.00	0.0040743	9.66
18D00208	17.6 %	✓	2.690671	0.31	0.0000000	0.00	0.0589983	1.51	0.0000168	55.72	218.2697	1.50	0.5028864	0.31	0.0000000	0.00	0.0821251	0.37	0.0392886	9.75	0.0437027	55.72	6.80013	0.36	0.1402383	1.76	1.69147	188.69	795.093	0.39	0.0000000	0.00	0.0041277	9.66
18D00209	19.3 %	✓	3.254681	0.30	0.0000000	0.00	0.0740684	1.22	0.0000161	57.27	274.0229	1.21	0.6082998	0.30	0.0000000	0.00	0.0840256	0.37	0.0493241	9.71	0.0418893	57.28	6.95749	0.36	0.1760597	1.52	4.73040	79.70	961.758	0.38	0.0000000	0.00	0.0042232	9.66
18D00211	21.0 %	✓	2.334645	0.32	0.0000000	0.00	0.0727204	1.25	0.0000000	0.00	269.0359	1.23	0.4363452	0.32	0.0000000	0.00	0.0634723	0.50	0.0484265	9.71	0.0000000	0.00	5.25563	0.49	0.1728556	1.54	6.87869	41.27	689.888	0.40	0.0000000	0.00	0.0031902	9.66
		Σ	43.161094	0.07	0.0000000	0.00	1.3227041	0.33	0.0000647	39.91	4893.4669	0.32	8.0668085	0.07	0.0000000	0.00	4.3222113	0.05	0.8808240	2.26	0.1682955	39.92	357.88783	0.04	3.1440525	0.39	127.06393	9.57	12754.103	0.09	0.0000000	0.00	0.2172379	2.45
		Σ							44.483863	0.07	4893.4669	0.32									13.438139	0.52			361.03188	0.04							12881.384	0.13

**Additional Parameters**			**40Ar/39Ar**	**1σ**	**37Ar/39Ar**	**1σ**	**36Ar/39Ar**	**1σ**	**Time (days)**	**37Ar (decay)**	**39Ar (decay)**	**40Ar (moles)**
18D00176	1.8 %	✓	25.229776	0.025582	1.550809	0.053142	0.084417	0.000251	236.419	107.733043	1.00167312	6.370E-11
18D00178	1.9 %	✓	24.860551	0.028911	2.064232	0.066555	0.083405	0.000256	236.433	107.762602	1.00167322	4.972E-11
18D00179	2.0 %	✓	24.278544	0.040626	2.349056	0.110873	0.081766	0.000273	236.440	107.777385	1.00167327	3.017E-11
18D00181	2.2 %	✓	24.034757	0.048152	3.317531	0.132309	0.080906	0.000288	236.453	107.806956	1.00167337	2.444E-11
18D00182	2.4 %	✓	21.940343	0.068519	3.756614	0.200848	0.073590	0.000324	236.460	107.821744	1.00167342	1.482E-11
18D00184	2.7 %	✓	22.914279	0.064207	4.727652	0.187350	0.077365	0.000318	236.474	107.851327	1.00167352	1.627E-11
18D00185	3.0 %	✓	26.372353	0.062903	6.208763	0.179267	0.089792	0.000342	236.481	107.866122	1.00167357	2.098E-11
18D00187	3.4 %	✓	23.465281	0.075764	7.329761	0.215704	0.080112	0.000357	236.495	107.895717	1.00167366	1.459E-11
18D00188	3.9 %	✓	24.761344	0.071642	9.787621	0.223133	0.085680	0.000357	236.502	107.910518	1.00167371	1.661E-11
18D00190	4.5 %	✓	28.580928	0.064476	13.549623	0.188949	0.099511	0.000368	236.516	107.940125	1.00167381	2.300E-11
18D00191	5.2 %	✓	27.026672	0.066129	17.453955	0.217413	0.095103	0.000363	236.523	107.954932	1.00167386	2.077E-11
18D00193	6.0 %	✓	27.713138	0.077116	21.786952	0.268965	0.098906	0.000396	236.537	107.986033	1.00167396	1.805E-11
18D00194	6.9 %	✓	30.870573	0.083934	27.059365	0.281640	0.110759	0.000439	236.544	108.000846	1.00167401	2.004E-11
18D00196	7.9 %	✓	28.303334	0.081146	31.273475	0.312698	0.103389	0.000421	236.558	108.030478	1.00167411	1.766E-11
18D00197	9.0 %	✓	28.951140	0.097938	34.868621	0.360070	0.106202	0.000470	236.565	108.045298	1.00167416	1.530E-11
18D00199	10.3 %	✓	32.736289	0.113923	35.162603	0.396645	0.119631	0.000540	236.579	108.074942	1.00167426	1.564E-11
18D00200	11.6 %	✓	51.518952	0.174735	33.711212	0.416716	0.183108	0.000813	236.586	108.089767	1.00167431	2.202E-11
18D00202	12.5 %	✓	53.803048	0.257572	33.056873	0.551713	0.189666	0.001060	236.600	108.119424	1.00167440	1.537E-11
18D00203	13.4 %	✓	68.913164	0.322757	30.942624	0.547260	0.240906	0.001328	236.607	108.134256	1.00167445	1.852E-11
18D00205	14.6 %	✓	87.203081	0.336992	29.783791	0.452078	0.302424	0.001468	236.622	108.165408	1.00167456	2.806E-11
18D00206	16.0 %	✓	105.098140	0.403382	28.902136	0.464506	0.362432	0.001732	236.628	108.180246	1.00167461	3.450E-11
18D00208	17.6 %	✓	114.805071	0.415728	31.449321	0.485936	0.396188	0.001832	236.642	108.209927	1.00167470	3.825E-11
18D00209	19.3 %	✓	135.485582	0.482438	38.413275	0.484761	0.466635	0.002127	236.649	108.224771	1.00167475	4.639E-11
18D00211	21.0 %	✓	128.354246	0.619635	49.560008	0.655047	0.443469	0.002493	236.663	108.254465	1.00167485	3.344E-11

Procedure Blanks		36Ar ± 1σ (SE) [fA]	37Ar ± 1σ (SE) [fA]	38Ar ± 1σ (SE) [fA]	39Ar ± 1σ (SE) [fA]	40Ar ± 1σ (SE) [fA]
18D00176	1.8 %	0.0319547 ± 0.0023117	0.0007078 ± 0.0180595	0.0081099 ± 0.0167667	0.0455932 ± 0.0191002	9.7148104 ± 0.7372988
18D00178	1.9 %	0.0347878 ± 0.0023117	0.0071875 ± 0.0180595	0.0172416 ± 0.0167667	0.0316330 ± 0.0191002	10.4479365 ± 0.7372988
18D00179	2.0 %	0.0357263 ± 0.0023117	0.0100783 ± 0.0180595	0.0201951 ± 0.0167667	0.0275078 ± 0.0191002	10.6904545 ± 0.7372988
18D00181	2.2 %	0.0368194 ± 0.0023117	0.0141069 ± 0.0180595	0.0234797 ± 0.0167667	0.0236487 ± 0.0191002	10.9713066 ± 0.7372988
18D00182	2.4 %	0.0370376 ± 0.0023117	0.0153781 ± 0.0180595	0.0240336 ± 0.0167667	0.0234356 ± 0.0191002	11.0259212 ± 0.7372988
18D00184	2.7 %	0.0369637 ± 0.0023117	0.0167408 ± 0.0180595	0.0234765 ± 0.0167667	0.0253632 ± 0.0191002	11.0010239 ± 0.7372988
18D00185	3.0 %	0.0367250 ± 0.0023117	0.0169438 ± 0.0180595	0.0225522 ± 0.0167667	0.0271204 ± 0.0191002	10.9352286 ± 0.7372988
18D00187	3.4 %	0.0359647 ± 0.0023117	0.0166449 ± 0.0180595	0.0198339 ± 0.0167667	0.0313820 ± 0.0191002	10.7280314 ± 0.7372988
18D00188	3.9 %	0.0354861 ± 0.0023117	0.0162326 ± 0.0180595	0.0181905 ± 0.0167667	0.0335985 ± 0.0191002	10.5977822 ± 0.7372988
18D00190	4.5 %	0.0344274 ± 0.0023117	0.0150797 ± 0.0180595	0.0146669 ± 0.0167667	0.0376031 ± 0.0191002	10.3086596 ± 0.7372988
18D00191	5.2 %	0.0338800 ± 0.0023117	0.0144068 ± 0.0180595	0.0129012 ± 0.0167667	0.0391990 ± 0.0191002	10.1583751 ± 0.7372988
18D00193	6.0 %	0.0327678 ± 0.0023117	0.0129419 ± 0.0180595	0.0094512 ± 0.0167667	0.0412717 ± 0.0191002	9.8505152 ± 0.7372988
18D00194	6.9 %	0.0322797 ± 0.0023117	0.0122679 ± 0.0180595	0.0080137 ± 0.0167667	0.0415494 ± 0.0191002	9.7139360 ± 0.7372988
18D00196	7.9 %	0.0314276 ± 0.0023117	0.0110539 ± 0.0180595	0.0056879 ± 0.0167667	0.0406143 ± 0.0191002	9.4721039 ± 0.7372988
18D00197	9.0 %	0.0310757 ± 0.0023117	0.0105373 ± 0.0180595	0.0048409 ± 0.0167667	0.0394042 ± 0.0191002	9.3702266 ± 0.7372988
18D00199	10.3 %	0.0305368 ± 0.0023117	0.0097163 ± 0.0180595	0.0038366 ± 0.0167667	0.0356246 ± 0.0191002	9.2097835 ± 0.7372988
18D00200	11.6 %	0.0303517 ± 0.0023117	0.0094135 ± 0.0180595	0.0036845 ± 0.0167667	0.0331536 ± 0.0191002	9.1520293 ± 0.7372988
18D00202	12.5 %	0.0301413 ± 0.0023117	0.0090001 ± 0.0180595	0.0040481 ± 0.0167667	0.0274145 ± 0.0191002	9.0802804 ± 0.7372988
18D00203	13.4 %	0.0301075 ± 0.0023117	0.0088693 ± 0.0180595	0.0045328 ± 0.0167667	0.0243406 ± 0.0191002	9.0645333 ± 0.7372988
18D00205	14.6 %	0.0301532 ± 0.0023117	0.0086725 ± 0.0180595	0.0060720 ± 0.0167667	0.0181133 ± 0.0191002	9.0666872 ± 0.7372988
18D00206	16.0 %	0.0302104 ± 0.0023117	0.0085709 ± 0.0180595	0.0069816 ± 0.0167667	0.0155651 ± 0.0191002	9.0798689 ± 0.7372988
18D00208	17.6 %	0.0303314 ± 0.0023117	0.0082161 ± 0.0180595	0.0089233 ± 0.0167667	0.0121437 ± 0.0191002	9.1153216 ± 0.7372988
18D00209	19.3 %	0.0303659 ± 0.0023117	0.0078983 ± 0.0180595	0.0098512 ± 0.0167667	0.0116591 ± 0.0191002	9.1306272 ± 0.7372988
18D00211	21.0 %	0.0302960 ± 0.0023117	0.0067926 ± 0.0180595	0.0113152 ± 0.0167667	0.0142341 ± 0.0191002	9.1357783 ± 0.7372988

Intercept Values		36Ar ± 1σ (SE) [fA]	r2	Regression (type,n)	37Ar ± 1σ (SE) [fA]	r2	Regression (type,n)	38Ar ± 1σ (SE) [fA]	r2	Regression (type,n)	39Ar ± 1σ (SE) [fA]	r2	Regression (type,n)	40Ar ± 1σ (SE) [fA]	r2	Regression (type,n)
18D00176	1.8 %	4.2537909 ± 0.0029713	0.9900	EXP 150 of 150	0.7439799 ± 0.0171941	0.0502	EXP 149 of 150	1.4858431 ± 0.0151824	0.1789	EXP 150 of 150	52.2389910 ± 0.0196616	0.9962	EXP 150 of 150	1336.89452 ± 0.06877	0.9998	EXP 150 of 150
18D00178	1.9 %	3.3388337 ± 0.0028512	0.9849	EXP 150 of 150	0.7762639 ± 0.0167903	0.0138	EXP 150 of 150	1.1850537 ± 0.0165601	0.1075	EXP 150 of 150	41.3743262 ± 0.0179103	0.9948	EXP 150 of 150	1046.33006 ± 0.06295	0.9996	EXP 150 of 150
18D00179	2.0 %	2.0480641 ± 0.0020646	0.9776	EXP 150 of 150	0.5437282 ± 0.0184913	0.0000	EXP 149 of 150	0.7193025 ± 0.0171509	0.0252	EXP 150 of 150	25.7119134 ± 0.0167163	0.9875	EXP 150 of 150	639.17269 ± 0.04955	0.9989	EXP 150 of 150
18D00181	2.2 %	1.6660875 ± 0.0019664	0.9702	EXP 150 of 150	0.6256940 ± 0.0174494	0.0465	EXP 150 of 150	0.5687404 ± 0.0159422	0.0001	EXP 150 of 150	21.0398275 ± 0.0168185	0.9804	EXP 150 of 150	520.06109 ± 0.04773	0.9984	EXP 150 of 150
18D00182	2.4 %	1.0217759 ± 0.0015600	0.9427	EXP 150 of 150	0.4659710 ± 0.0180028	0.0676	EXP 150 of 150	0.3786126 ± 0.0147264	0.0235	EXP 150 of 150	13.9886347 ± 0.0182729	0.9433	EXP 150 of 150	319.83599 ± 0.05258	0.9826	EXP 150 of 150
18D00184	2.7 %	1.1247848 ± 0.0015302	0.9553	EXP 150 of 150	0.6196146 ± 0.0170020	0.0822	EXP 150 of 150	0.3997003 ± 0.0163769	0.0124	EXP 150 of 150	14.6996429 ± 0.0144581	0.9710	EXP 150 of 150	349.89507 ± 0.03747	0.9960	EXP 150 of 150
18D00185	3.0 %	1.4515395 ± 0.0018346	0.9636	EXP 150 of 150	0.9194305 ± 0.0189782	0.0436	EXP 150 of 150	0.4945371 ± 0.0165144	0.0076	EXP 150 of 150	16.4710849 ± 0.0168017	0.9683	EXP 150 of 150	448.01064 ± 0.04221	0.9979	EXP 150 of 150
18D00187	3.4 %	1.0223893 ± 0.0015590	0.9458	EXP 150 of 150	0.8469719 ± 0.0167494	0.0564	EXP 150 of 150	0.4044849 ± 0.0164947	0.0503	EXP 150 of 150	12.8816582 ± 0.0175772	0.9421	EXP 150 of 150	314.63400 ± 0.04055	0.9922	EXP 150 of 150
18D00188	3.9 %	1.1738970 ± 0.0016281	0.9568	EXP 150 of 150	1.2279893 ± 0.0199523	0.1021	EXP 150 of 150	0.4028829 ± 0.0147322	0.0027	EXP 150 of 150	13.8999267 ± 0.0168509	0.9508	EXP 150 of 150	356.64608 ± 0.04446	0.9941	EXP 150 of 150
18D00190	4.5 %	1.6203931 ± 0.0019337	0.9690	EXP 150 of 150	2.0504568 ± 0.0169347	0.3024	EXP 150 of 150	0.5580071 ± 0.0164191	0.0757	EXP 150 of 150	16.6704080 ± 0.0162351	0.9706	EXP 150 of 150	489.42710 ± 0.04439	0.9984	EXP 150 of 150
18D00191	5.2 %	1.4811654 ± 0.0018377	0.9620	EXP 150 of 150	2.5258649 ± 0.0185734	0.3526	EXP 149 of 150	0.4973725 ± 0.0155808	0.0039	EXP 150 of 150	15.9212183 ± 0.0172757	0.9626	EXP 150 of 150	442.77119 ± 0.04692	0.9970	EXP 150 of 150
18D00193	6.0 %	1.3087984 ± 0.0015811	0.9657	EXP 150 of 150	2.6744801 ± 0.0199780	0.3664	EXP 150 of 150	0.4259668 ± 0.0163370	0.0050	EXP 150 of 150	13.5055732 ± 0.0161255	0.9533	EXP 149 of 150	385.92223 ± 0.04070	0.9966	EXP 150 of 150
18D00194	6.9 %	1.4565909 ± 0.0017426	0.9666	EXP 149 of 150	3.3141992 ± 0.0171228	0.5774	EXP 150 of 150	0.4499726 ± 0.0158643	0.0120	EXP 150 of 150	13.4620785 ± 0.0179874	0.9351	EXP 150 of 150	427.27067 ± 0.04496	0.9967	EXP 150 of 150
18D00196	7.9 %	1.3090387 ± 0.0017215	0.9592	EXP 150 of 150	3.6823084 ± 0.0180129	0.4683	EXP 150 of 150	0.3965025 ± 0.0167474	0.0025	EXP 149 of 150	12.9370251 ± 0.0160202	0.9446	EXP 150 of 150	377.35335 ± 0.05870	0.9899	EXP 150 of 150
18D00197	9.0 %	1.1428218 ± 0.0015501	0.9520	EXP 150 of 150	3.4774150 ± 0.0176990	0.5538	EXP 150 of 150	0.3913815 ± 0.0160116	0.0835	EXP 150 of 150	10.9643275 ± 0.0171886	0.9050	EXP 150 of 150	328.14599 ± 0.11099	0.8847	EXP 150 of 150
18D00199	10.3 %	1.1627416 ± 0.0015645	0.9547	EXP 150 of 150	3.1693735 ± 0.0200854	0.4145	EXP 149 of 150	0.3726351 ± 0.0175917	0.0324	EXP 150 of 150	9.9125898 ± 0.0164278	0.8902	EXP 150 of 150	335.08718 ± 0.06884	0.9721	EXP 150 of 150
18D00200	11.6 %	1.5803608 ± 0.0017225	0.9724	EXP 149 of 150	2.7163276 ± 0.0196335	0.3406	EXP 150 of 150	0.3992042 ± 0.0161921	0.0005	EXP 150 of 150	8.8674411 ± 0.0172053	0.8444	EXP 150 of 150	467.86375 ± 0.04893	0.9973	EXP 150 of 150
18D00202	12.5 %	1.1029972 ± 0.0013842	0.9625	EXP 150 of 150	1.7765656 ± 0.0187725	0.1870	EXP 150 of 150	0.3057172 ± 0.0161683	0.0117	EXP 150 of 150	5.9307091 ± 0.0152699	0.6732	EXP 150 of 150	329.19280 ± 0.03632	0.9957	EXP 150 of 150
18D00203	13.4 %	1.3126983 ± 0.0015975	0.9682	EXP 150 of 150	1.5640288 ± 0.0168365	0.2488	EXP 150 of 150	0.3444889 ± 0.0157786	0.0392	EXP 150 of 150	5.5806110 ± 0.0136310	0.7433	EXP 150 of 150	394.97532 ± 0.03613	0.9981	EXP 150 of 150
18D00205	14.6 %	1.9574909 ± 0.0023151	0.9712	EXP 150 of 150	1.8030827 ± 0.0151112	0.3166	EXP 149 of 150	0.4877158 ± 0.0146071	0.0299	EXP 150 of 150	6.6690760 ± 0.0143421	0.8174	EXP 149 of 150	593.61141 ± 0.04026	0.9994	EXP 150 of 150
18D00206	16.0 %	2.3868284 ± 0.0021942	0.9829	EXP 150 of 150	1.7849730 ± 0.0174910	0.1780	EXP 150 of 150	0.5302543 ± 0.0149312	0.0033	EXP 150 of 150	6.8014521 ± 0.0156403	0.7380	EXP 149 of 150	727.87160 ± 0.04216	0.9996	EXP 150 of 150
18D00208	17.6 %	2.6445286 ± 0.0026182	0.9803	EXP 150 of 150	1.9717073 ± 0.0193142	0.2471	EXP 150 of 150	0.6687087 ± 0.0167182	0.1090	EXP 150 of 150	6.8983616 ± 0.0139750	0.7992	EXP 150 of 150	805.90426 ± 0.04867	0.9996	EXP 150 of 150
18D00209	19.3 %	3.1951081 ± 0.0028599	0.9843	EXP 150 of 150	2.4774210 ± 0.0168967	0.4300	EXP 150 of 150	0.7837513 ± 0.0159141	0.1300	EXP 150 of 150	7.0895529 ± 0.0147986	0.7684	EXP 149 of 150	975.62339 ± 0.04936	0.9998	EXP 150 of 150
18D00211	21.0 %	2.3190402 ± 0.0023674	0.9784	EXP 150 of 150	2.4326268 ± 0.0171740	0.3759	EXP 149 of 150	0.5404835 ± 0.0157683	0.0227	EXP 150 of 150	5.4003710 ± 0.0163064	0.5534	EXP 150 of 150	705.90532 ± 0.04028	0.9996	EXP 150 of 150

Project Info		Analyst	Irradiation	X-pos	Y-pos	Z/H-pos	Project	Experiment	Nmb
18D00176	1.8 %	Dan Miggins	17-OSU-05	0.00	0.00	51.19	Arctic\O-Connor (16-22)	18D00172	01
18D00178	1.9 %	Dan Miggins	17-OSU-05	0.00	0.00	51.19	Arctic\O-Connor (16-22)	18D00172	01
18D00179	2.0 %	Dan Miggins	17-OSU-05	0.00	0.00	51.19	Arctic\O-Connor (16-22)	18D00172	01
18D00181	2.2 %	Dan Miggins	17-OSU-05	0.00	0.00	51.19	Arctic\O-Connor (16-22)	18D00172	01
18D00182	2.4 %	Dan Miggins	17-OSU-05	0.00	0.00	51.19	Arctic\O-Connor (16-22)	18D00172	01
18D00184	2.7 %	Dan Miggins	17-OSU-05	0.00	0.00	51.19	Arctic\O-Connor (16-22)	18D00172	01
18D00185	3.0 %	Dan Miggins	17-OSU-05	0.00	0.00	51.19	Arctic\O-Connor (16-22)	18D00172	01
18D00187	3.4 %	Dan Miggins	17-OSU-05	0.00	0.00	51.19	Arctic\O-Connor (16-22)	18D00172	01
18D00188	3.9 %	Dan Miggins	17-OSU-05	0.00	0.00	51.19	Arctic\O-Connor (16-22)	18D00172	01
18D00190	4.5 %	Dan Miggins	17-OSU-05	0.00	0.00	51.19	Arctic\O-Connor (16-22)	18D00172	01
18D00191	5.2 %	Dan Miggins	17-OSU-05	0.00	0.00	51.19	Arctic\O-Connor (16-22)	18D00172	01
18D00193	6.0 %	Dan Miggins	17-OSU-05	0.00	0.00	51.19	Arctic\O-Connor (16-22)	18D00172	01
18D00194	6.9 %	Dan Miggins	17-OSU-05	0.00	0.00	51.19	Arctic\O-Connor (16-22)	18D00172	01
18D00196	7.9 %	Dan Miggins	17-OSU-05	0.00	0.00	51.19	Arctic\O-Connor (16-22)	18D00172	01
18D00197	9.0 %	Dan Miggins	17-OSU-05	0.00	0.00	51.19	Arctic\O-Connor (16-22)	18D00172	01
18D00199	10.3 %	Dan Miggins	17-OSU-05	0.00	0.00	51.19	Arctic\O-Connor (16-22)	18D00172	01
18D00200	11.6 %	Dan Miggins	17-OSU-05	0.00	0.00	51.19	Arctic\O-Connor (16-22)	18D00172	01
18D00202	12.5 %	Dan Miggins	17-OSU-05	0.00	0.00	51.19	Arctic\O-Connor (16-22)	18D00172	01
18D00203	13.4 %	Dan Miggins	17-OSU-05	0.00	0.00	51.19	Arctic\O-Connor (16-22)	18D00172	01
18D00205	14.6 %	Dan Miggins	17-OSU-05	0.00	0.00	51.19	Arctic\O-Connor (16-22)	18D00172	01
18D00206	16.0 %	Dan Miggins	17-OSU-05	0.00	0.00	51.19	Arctic\O-Connor (16-22)	18D00172	01
18D00208	17.6 %	Dan Miggins	17-OSU-05	0.00	0.00	51.19	Arctic\O-Connor (16-22)	18D00172	01
18D00209	19.3 %	Dan Miggins	17-OSU-05	0.00	0.00	51.19	Arctic\O-Connor (16-22)	18D00172	01
18D00211	21.0 %	Dan Miggins	17-OSU-05	0.00	0.00	51.19	Arctic\O-Connor (16-22)	18D00172	01

**Sample Parameters**		Sample	Material	Location	Standard Name	Standard (in Ma)	%1σ	Standard Reference	Standard 40Ar/39Ar	%1σ	J	%1σ	Air 40Ar/36Ar	%1σ	MDF (lin)	%1σ	Volume Ratio	Sensitivity (mol/volt)	Day	Month	Year	Hour	Min	Resist
18D00176	1.8 %	PS59-223-27	Groundmass	Gakkel Ridge	FCT-NM (5B35-17)	28.201	0.082	Kuiper et al (2008)	10.30417	0.077	0.00152534	0.077	302.945	0.131	0.9938482	0.067	1	4.8E-14	2	JAN	2018	21	53	1
18D00178	1.9 %	PS59-223-27	Groundmass	Gakkel Ridge	FCT-NM (5B35-17)	28.201	0.082	Kuiper et al (2008)	10.30417	0.077	0.00152534	0.077	302.945	0.131	0.9938482	0.067	1	4.8E-14	2	JAN	2018	22	13	1
18D00179	2.0 %	PS59-223-27	Groundmass	Gakkel Ridge	FCT-NM (5B35-17)	28.201	0.082	Kuiper et al (2008)	10.30417	0.077	0.00152534	0.077	302.945	0.131	0.9938482	0.067	1	4.8E-14	2	JAN	2018	22	23	1
18D00181	2.2 %	PS59-223-27	Groundmass	Gakkel Ridge	FCT-NM (5B35-17)	28.201	0.082	Kuiper et al (2008)	10.30417	0.077	0.00152534	0.077	302.945	0.131	0.9938482	0.067	1	4.8E-14	2	JAN	2018	22	43	1
18D00182	2.4 %	PS59-223-27	Groundmass	Gakkel Ridge	FCT-NM (5B35-17)	28.201	0.082	Kuiper et al (2008)	10.30417	0.077	0.00152534	0.077	302.945	0.131	0.9938482	0.067	1	4.8E-14	2	JAN	2018	22	53	1
18D00184	2.7 %	PS59-223-27	Groundmass	Gakkel Ridge	FCT-NM (5B35-17)	28.201	0.082	Kuiper et al (2008)	10.30417	0.077	0.00152534	0.077	302.945	0.131	0.9938482	0.067	1	4.8E-14	2	JAN	2018	23	13	1
18D00185	3.0 %	PS59-223-27	Groundmass	Gakkel Ridge	FCT-NM (5B35-17)	28.201	0.082	Kuiper et al (2008)	10.30417	0.077	0.00152534	0.077	302.945	0.131	0.9938482	0.067	1	4.8E-14	2	JAN	2018	23	23	1
18D00187	3.4 %	PS59-223-27	Groundmass	Gakkel Ridge	FCT-NM (5B35-17)	28.201	0.082	Kuiper et al (2008)	10.30417	0.077	0.00152534	0.077	302.945	0.131	0.9938482	0.067	1	4.8E-14	2	JAN	2018	23	43	1
18D00188	3.9 %	PS59-223-27	Groundmass	Gakkel Ridge	FCT-NM (5B35-17)	28.201	0.082	Kuiper et al (2008)	10.30417	0.077	0.00152534	0.077	302.945	0.131	0.9938482	0.067	1	4.8E-14	2	JAN	2018	23	53	1
18D00190	4.5 %	PS59-223-27	Groundmass	Gakkel Ridge	FCT-NM (5B35-17)	28.201	0.082	Kuiper et al (2008)	10.30417	0.077	0.00152534	0.077	302.945	0.131	0.9938482	0.067	1	4.8E-14	3	JAN	2018	0	13	1
18D00191	5.2 %	PS59-223-27	Groundmass	Gakkel Ridge	FCT-NM (5B35-17)	28.201	0.082	Kuiper et al (2008)	10.30417	0.077	0.00152534	0.077	302.945	0.131	0.9938482	0.067	1	4.8E-14	3	JAN	2018	0	23	1
18D00193	6.0 %	PS59-223-27	Groundmass	Gakkel Ridge	FCT-NM (5B35-17)	28.201	0.082	Kuiper et al (2008)	10.30417	0.077	0.00152534	0.077	302.945	0.131	0.9938482	0.067	1	4.8E-14	3	JAN	2018	0	44	1
18D00194	6.9 %	PS59-223-27	Groundmass	Gakkel Ridge	FCT-NM (5B35-17)	28.201	0.082	Kuiper et al (2008)	10.30417	0.077	0.00152534	0.077	302.945	0.131	0.9938482	0.067	1	4.8E-14	3	JAN	2018	0	54	1
18D00196	7.9 %	PS59-223-27	Groundmass	Gakkel Ridge	FCT-NM (5B35-17)	28.201	0.082	Kuiper et al (2008)	10.30417	0.077	0.00152534	0.077	302.945	0.131	0.9938482	0.067	1	4.8E-14	3	JAN	2018	1	14	1
18D00197	9.0 %	PS59-223-27	Groundmass	Gakkel Ridge	FCT-NM (5B35-17)	28.201	0.082	Kuiper et al (2008)	10.30417	0.077	0.00152534	0.077	302.945	0.131	0.9938482	0.067	1	4.8E-14	3	JAN	2018	1	24	1
18D00199	10.3 %	PS59-223-27	Groundmass	Gakkel Ridge	FCT-NM (5B35-17)	28.201	0.082	Kuiper et al (2008)	10.30417	0.077	0.00152534	0.077	302.945	0.131	0.9938482	0.067	1	4.8E-14	3	JAN	2018	1	44	1
18D00200	11.6 %	PS59-223-27	Groundmass	Gakkel Ridge	FCT-NM (5B35-17)	28.201	0.082	Kuiper et al (2008)	10.30417	0.077	0.00152534	0.077	302.945	0.131	0.9938482	0.067	1	4.8E-14	3	JAN	2018	1	54	1
18D00202	12.5 %	PS59-223-27	Groundmass	Gakkel Ridge	FCT-NM (5B35-17)	28.201	0.082	Kuiper et al (2008)	10.30417	0.077	0.00152534	0.077	302.945	0.131	0.9938482	0.067	1	4.8E-14	3	JAN	2018	2	14	1
18D00203	13.4 %	PS59-223-27	Groundmass	Gakkel Ridge	FCT-NM (5B35-17)	28.201	0.082	Kuiper et al (2008)	10.30417	0.077	0.00152534	0.077	302.945	0.131	0.9938482	0.067	1	4.8E-14	3	JAN	2018	2	24	1
18D00205	14.6 %	PS59-223-27	Groundmass	Gakkel Ridge	FCT-NM (5B35-17)	28.201	0.082	Kuiper et al (2008)	10.30417	0.077	0.00152534	0.077	302.945	0.131	0.9938482	0.067	1	4.8E-14	3	JAN	2018	2	45	1
18D00206	16.0 %	PS59-223-27	Groundmass	Gakkel Ridge	FCT-NM (5B35-17)	28.201	0.082	Kuiper et al (2008)	10.30417	0.077	0.00152534	0.077	302.945	0.131	0.9938482	0.067	1	4.8E-14	3	JAN	2018	2	55	1
18D00208	17.6 %	PS59-223-27	Groundmass	Gakkel Ridge	FCT-NM (5B35-17)	28.201	0.082	Kuiper et al (2008)	10.30417	0.077	0.00152534	0.077	302.945	0.131	0.9938482	0.067	1	4.8E-14	3	JAN	2018	3	15	1
18D00209	19.3 %	PS59-223-27	Groundmass	Gakkel Ridge	FCT-NM (5B35-17)	28.201	0.082	Kuiper et al (2008)	10.30417	0.077	0.00152534	0.077	302.945	0.131	0.9938482	0.067	1	4.8E-14	3	JAN	2018	3	25	1
18D00211	21.0 %	PS59-223-27	Groundmass	Gakkel Ridge	FCT-NM (5B35-17)	28.201	0.082	Kuiper et al (2008)	10.30417	0.077	0.00152534	0.077	302.945	0.131	0.9938482	0.067	1	4.8E-14	3	JAN	2018	3	45	1

**Irradiation Constants**		**40/36(a)**	**%1σ**	**40/36(c)**	**%1σ**	**38/36(a)**	**%1σ**	**38/36(c)**	**%1σ**	**39/37(ca)**	**%1σ**	**38/37(ca)**	**%1σ**	**36/37(ca)**	**%1σ**	**40/39(k)**	**%1σ**	**38/39(k)**	**%1σ**	**36/38(cl)**	**%1σ**	**K/Ca**	**%1σ**	**K/Cl**	**%1σ**	**Ca/Cl**	**%1σ**
18D00176	1.8 %	295.5	0.237	0.018	35	0.1869	0	1.493	3	0.000643	0.92	0.00018	9.63	0.00027	0.17	0.000607	9.65	0.012077	0.09	0	0	0.43	0	0	0	0	0
18D00178	1.9 %	295.5	0.237	0.018	35	0.1869	0	1.493	3	0.000643	0.92	0.00018	9.63	0.00027	0.17	0.000607	9.65	0.012077	0.09	0	0	0.43	0	0	0	0	0
18D00179	2.0 %	295.5	0.237	0.018	35	0.1869	0	1.493	3	0.000643	0.92	0.00018	9.63	0.00027	0.17	0.000607	9.65	0.012077	0.09	0	0	0.43	0	0	0	0	0
18D00181	2.2 %	295.5	0.237	0.018	35	0.1869	0	1.493	3	0.000643	0.92	0.00018	9.63	0.00027	0.17	0.000607	9.65	0.012077	0.09	0	0	0.43	0	0	0	0	0
18D00182	2.4 %	295.5	0.237	0.018	35	0.1869	0	1.493	3	0.000643	0.92	0.00018	9.63	0.00027	0.17	0.000607	9.65	0.012077	0.09	0	0	0.43	0	0	0	0	0
18D00184	2.7 %	295.5	0.237	0.018	35	0.1869	0	1.493	3	0.000643	0.92	0.00018	9.63	0.00027	0.17	0.000607	9.65	0.012077	0.09	0	0	0.43	0	0	0	0	0
18D00185	3.0 %	295.5	0.237	0.018	35	0.1869	0	1.493	3	0.000643	0.92	0.00018	9.63	0.00027	0.17	0.000607	9.65	0.012077	0.09	0	0	0.43	0	0	0	0	0
18D00187	3.4 %	295.5	0.237	0.018	35	0.1869	0	1.493	3	0.000643	0.92	0.00018	9.63	0.00027	0.17	0.000607	9.65	0.012077	0.09	0	0	0.43	0	0	0	0	0
18D00188	3.9 %	295.5	0.237	0.018	35	0.1869	0	1.493	3	0.000643	0.92	0.00018	9.63	0.00027	0.17	0.000607	9.65	0.012077	0.09	0	0	0.43	0	0	0	0	0
18D00190	4.5 %	295.5	0.237	0.018	35	0.1869	0	1.493	3	0.000643	0.92	0.00018	9.63	0.00027	0.17	0.000607	9.65	0.012077	0.09	0	0	0.43	0	0	0	0	0
18D00191	5.2 %	295.5	0.237	0.018	35	0.1869	0	1.493	3	0.000643	0.92	0.00018	9.63	0.00027	0.17	0.000607	9.65	0.012077	0.09	0	0	0.43	0	0	0	0	0
18D00193	6.0 %	295.5	0.237	0.018	35	0.1869	0	1.493	3	0.000643	0.92	0.00018	9.63	0.00027	0.17	0.000607	9.65	0.012077	0.09	0	0	0.43	0	0	0	0	0
18D00194	6.9 %	295.5	0.237	0.018	35	0.1869	0	1.493	3	0.000643	0.92	0.00018	9.63	0.00027	0.17	0.000607	9.65	0.012077	0.09	0	0	0.43	0	0	0	0	0
18D00196	7.9 %	295.5	0.237	0.018	35	0.1869	0	1.493	3	0.000643	0.92	0.00018	9.63	0.00027	0.17	0.000607	9.65	0.012077	0.09	0	0	0.43	0	0	0	0	0
18D00197	9.0 %	295.5	0.237	0.018	35	0.1869	0	1.493	3	0.000643	0.92	0.00018	9.63	0.00027	0.17	0.000607	9.65	0.012077	0.09	0	0	0.43	0	0	0	0	0
18D00199	10.3 %	295.5	0.237	0.018	35	0.1869	0	1.493	3	0.000643	0.92	0.00018	9.63	0.00027	0.17	0.000607	9.65	0.012077	0.09	0	0	0.43	0	0	0	0	0
18D00200	11.6 %	295.5	0.237	0.018	35	0.1869	0	1.493	3	0.000643	0.92	0.00018	9.63	0.00027	0.17	0.000607	9.65	0.012077	0.09	0	0	0.43	0	0	0	0	0
18D00202	12.5 %	295.5	0.237	0.018	35	0.1869	0	1.493	3	0.000643	0.92	0.00018	9.63	0.00027	0.17	0.000607	9.65	0.012077	0.09	0	0	0.43	0	0	0	0	0
18D00203	13.4 %	295.5	0.237	0.018	35	0.1869	0	1.493	3	0.000643	0.92	0.00018	9.63	0.00027	0.17	0.000607	9.65	0.012077	0.09	0	0	0.43	0	0	0	0	0
18D00205	14.6 %	295.5	0.237	0.018	35	0.1869	0	1.493	3	0.000643	0.92	0.00018	9.63	0.00027	0.17	0.000607	9.65	0.012077	0.09	0	0	0.43	0	0	0	0	0
18D00206	16.0 %	295.5	0.237	0.018	35	0.1869	0	1.493	3	0.000643	0.92	0.00018	9.63	0.00027	0.17	0.000607	9.65	0.012077	0.09	0	0	0.43	0	0	0	0	0
18D00208	17.6 %	295.5	0.237	0.018	35	0.1869	0	1.493	3	0.000643	0.92	0.00018	9.63	0.00027	0.17	0.000607	9.65	0.012077	0.09	0	0	0.43	0	0	0	0	0
18D00209	19.3 %	295.5	0.237	0.018	35	0.1869	0	1.493	3	0.000643	0.92	0.00018	9.63	0.00027	0.17	0.000607	9.65	0.012077	0.09	0	0	0.43	0	0	0	0	0
18D00211	21.0 %	295.5	0.237	0.018	35	0.1869	0	1.493	3	0.000643	0.92	0.00018	9.63	0.00027	0.17	0.000607	9.65	0.012077	0.09	0	0	0.43	0	0	0	0	0

18D00172.AGE >>> PS59-223-27 >>> ARCTIC | O-CONNOR (16-22) PROJECT

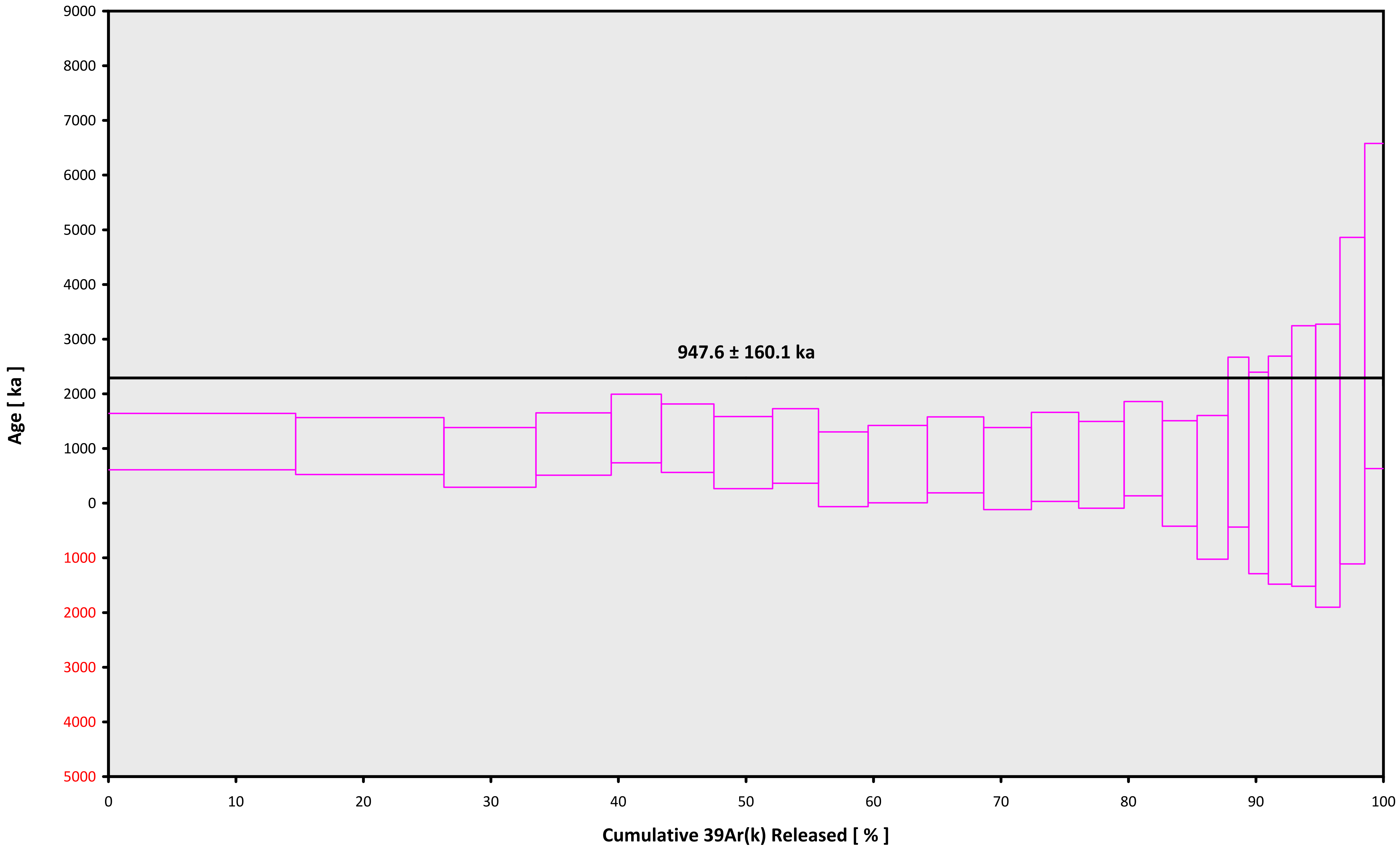

Ar-Ages in ka

WEIGHTED PLATEAU
947.6 ± 160.1
TOTAL FUSION
979.0 ± 187.4
NORMAL ISOCHRON
917.4 ± 304.0
INVERSE ISOCHRON
917.0 ± 252.3

MSWD (PROBABILITY)
0.51 (98%)

Sample Info

Groundmass
Gakkel Ridge
Dan Miggins

IRR = 17-OSU-05 (5B35-17)
J = 0.00152534 ± 0.00000117

18D00172.AGE >>> PS59-223-27 >>> ARCTIC | O-CONNOR (16-22) PROJECT

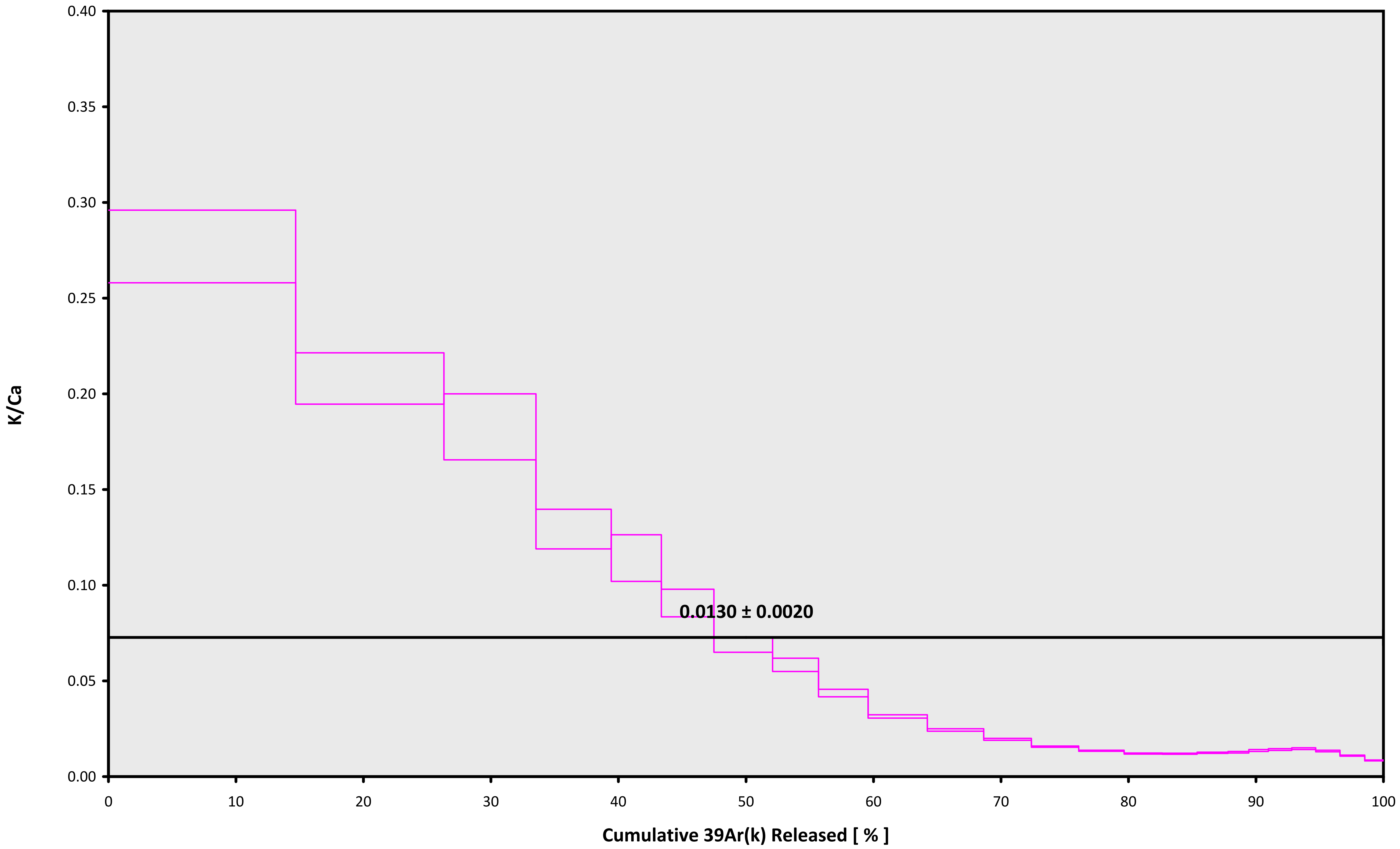

Ar-Ages in ka

WEIGHTED PLATEAU
947.6 ± 160.1
TOTAL FUSION
979.0 ± 187.4
NORMAL ISOCHRON
917.4 ± 304.0
INVERSE ISOCHRON
917.0 ± 252.3

Sample Info

Groundmass
Gakkel Ridge
Dan Miggins

IRR = 17-OSU-05 (5B35-17)
J = 0.00152534 ± 0.00000117

18D00172.AGE >>> PS59-223-27 >>> ARCTIC | O-CONNOR (16-22) PROJECT

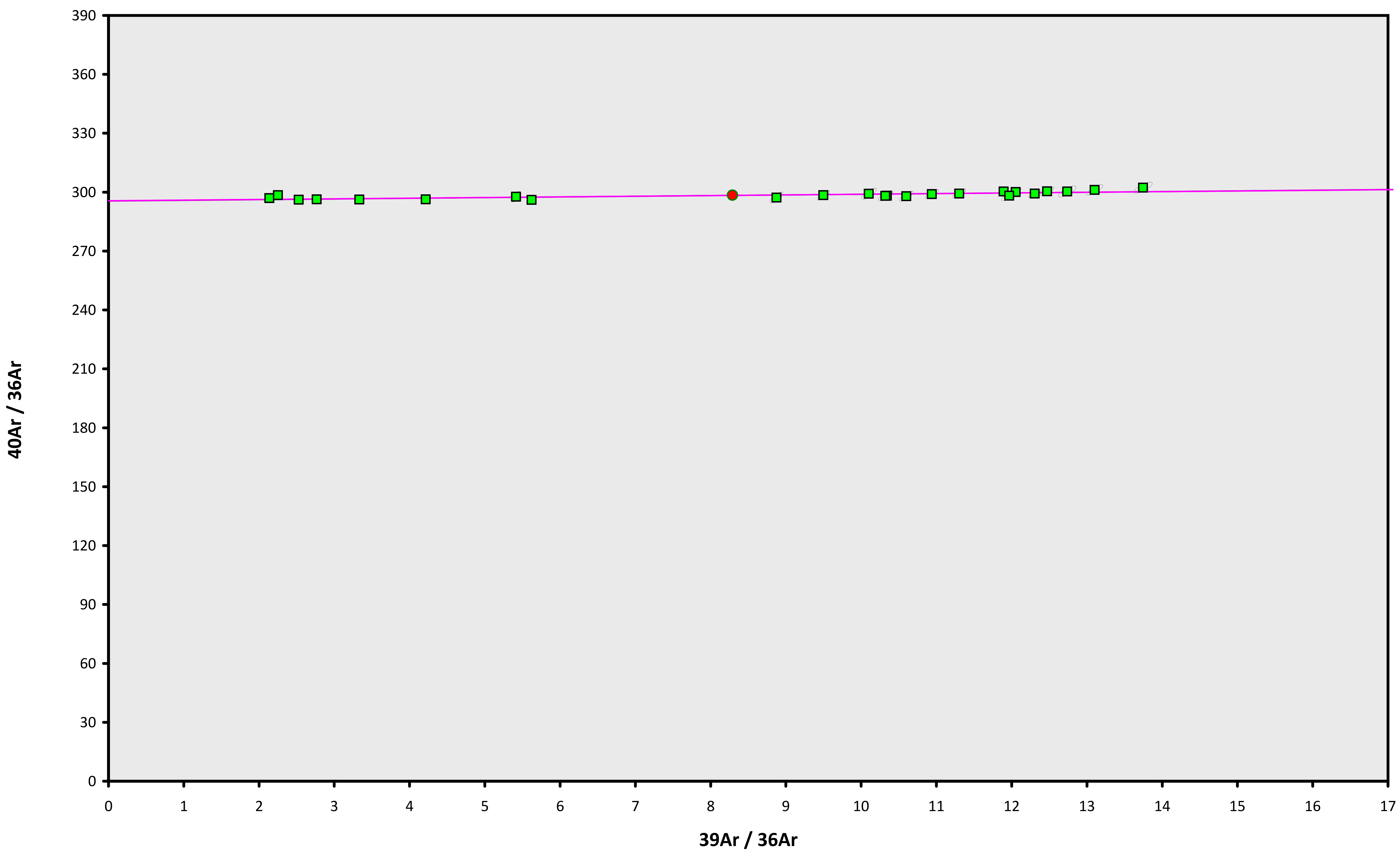

Ar-Ages in ka

WEIGHTED PLATEAU
947.6 ± 160.1
TOTAL FUSION
979.0 ± 187.4
NORMAL ISOCHRON
917.4 ± 304.0
INVERSE ISOCHRON
917.0 ± 252.3

MSWD (PROBABILITY)
0.73 (81%)

40AR/36AR INTERCEPT
295.6 ± 1.0

Sample Info

Groundmass
Gakkel Ridge
Dan Miggins

IRR = 17-OSU-05 (5B35-17)
J = 0.00152534 ± 0.00000117

18D00172.AGE >>> PS59-223-27 >>> ARCTIC | O-CONNOR (16-22) PROJECT

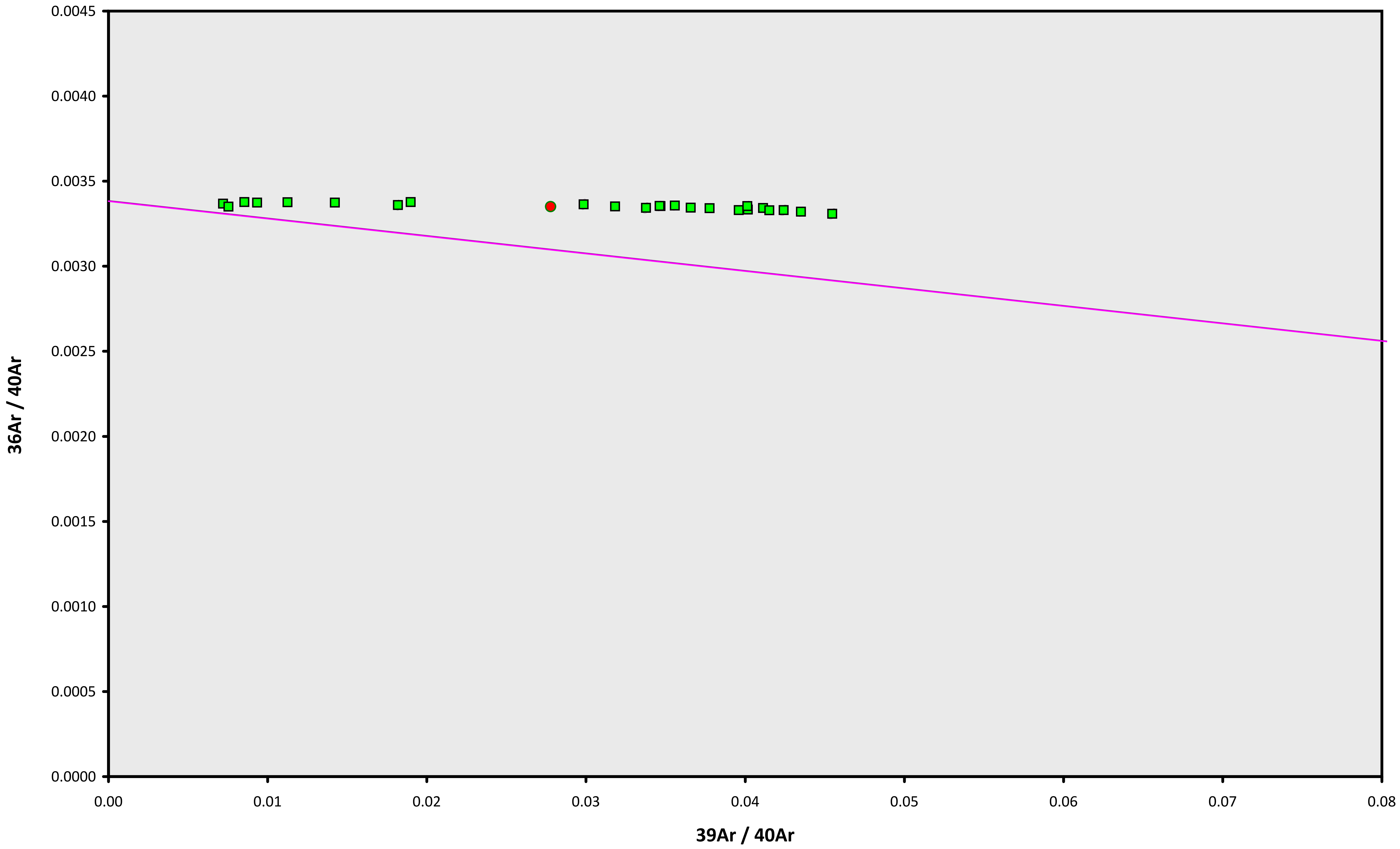

Ar-Ages in ka

WEIGHTED PLATEAU
947.6 ± 160.1
TOTAL FUSION
979.0 ± 187.4
NORMAL ISOCHRON
917.4 ± 304.0
INVERSE ISOCHRON
917.0 ± 252.3

MSWD (PROBABILITY)
0.73 (81%)

SPREADING FACTOR
1.3%

40AR/36AR INTERCEPT
295.6 ± 1.0

Sample Info

Groundmass
Gakkel Ridge
Dan Miggins

IRR = 17-OSU-05 (5B35-17)
J = 0.00152534 ± 0.00000117

Relative Abundances			36Ar [fA]	%1σ	37Ar [fA]	%1σ	38Ar [fA]	%1σ	39Ar [fA]	%1σ	40Ar [fA]	%1σ	40(r)/39(k) ± 2σ	Age ± 2σ (Ma)	40Ar(r) (%)	39Ar(k) (%)	K/Ca ± 2σ
17D20166	1.8 %		1.362274	0.311	111.0664	0.608	0.8899245	2.655	50.04852	0.084	427.0783	0.026	0.66768 ± 0.05043	1.97 ± 0.15	7.81	17.00	0.1935 ± 0.0024
17D20168	1.9 %		0.754905	0.332	103.4334	0.630	0.5479315	4.427	31.92712	0.100	236.3779	0.054	0.67627 ± 0.04739	1.99 ± 0.14	9.12	10.83	0.1325 ± 0.0017
17D20169	2.0 %	✓	0.389471	0.395	60.5543	0.895	0.2701889	8.730	16.92908	0.155	120.4159	0.087	0.60116 ± 0.05551	1.77 ± 0.16	8.43	5.74	0.1199 ± 0.0022
17D20171	2.2 %	✓	0.348459	0.402	70.8654	0.777	0.2606309	8.789	14.86198	0.166	105.5924	0.099	0.55848 ± 0.05801	1.64 ± 0.17	7.84	5.04	0.0899 ± 0.0014
17D20172	2.4 %	✓	0.344652	0.399	90.8509	0.661	0.2631973	9.263	14.35086	0.173	102.9551	0.102	0.58487 ± 0.05914	1.72 ± 0.17	8.12	4.86	0.0676 ± 0.0009
17D20174	2.7 %	✓	0.356104	0.397	111.0070	0.605	0.2342309	10.241	13.49888	0.191	104.2689	0.100	0.58824 ± 0.06463	1.73 ± 0.19	7.58	4.57	0.0520 ± 0.0007
17D20175	3.0 %	✓	0.299444	0.449	113.2133	0.614	0.1975410	12.322	11.25805	0.222	85.8894	0.122	0.57572 ± 0.07428	1.69 ± 0.22	7.50	3.80	0.0425 ± 0.0006
17D20177	3.4 %	✓	0.325802	0.426	144.5023	0.553	0.2095631	11.147	11.21292	0.226	90.8706	0.114	0.55138 ± 0.07710	1.62 ± 0.23	6.75	3.78	0.0331 ± 0.0004
17D20178	3.9 %	✓	0.296694	0.443	142.2920	0.557	0.1608603	15.066	9.60039	0.249	81.4265	0.128	0.53769 ± 0.08582	1.58 ± 0.25	6.28	3.23	0.0287 ± 0.0004
17D20180	4.5 %	✓	0.279631	0.445	147.8003	0.551	0.1590184	14.455	8.38035	0.286	74.0479	0.141	0.38830 ± 0.09366	1.14 ± 0.28	4.34	2.82	0.0241 ± 0.0003
17D20181	5.2 %	✓	0.439190	0.393	300.3240	0.484	0.2458929	9.524	12.34304	0.200	112.1766	0.093	0.52481 ± 0.08800	1.55 ± 0.26	5.68	4.13	0.0174 ± 0.0002
17D20183	6.0 %	✓	0.356581	0.403	254.3588	0.493	0.2001160	11.516	9.76451	0.237	90.2289	0.115	0.53849 ± 0.09377	1.59 ± 0.28	5.73	3.26	0.0162 ± 0.0002
17D20184	6.9 %	✓	0.366260	0.407	311.5313	0.485	0.1974875	11.974	10.51803	0.223	89.1925	0.117	0.56595 ± 0.09125	1.67 ± 0.27	6.55	3.51	0.0142 ± 0.0002
17D20186	7.9 %		0.368023	0.415	313.0129	0.482	0.2062887	10.919	10.29197	0.237	88.7339	0.117	0.49337 ± 0.09526	1.45 ± 0.28	5.61	3.43	0.0139 ± 0.0001
17D20187	9.0 %		0.345962	0.402	278.7806	0.488	0.1810041	12.749	9.62039	0.247	84.0594	0.124	0.43312 ± 0.09316	1.28 ± 0.27	4.86	3.21	0.0146 ± 0.0002
17D20189	10.3 %		0.463184	0.371	295.6470	0.486	0.2269610	10.406	11.04981	0.216	117.2851	0.089	0.37037 ± 0.09797	1.09 ± 0.29	3.43	3.69	0.0158 ± 0.0002
17D20190	11.6 %		0.528082	0.361	307.6709	0.481	0.2609724	9.464	11.32495	0.210	135.4231	0.078	0.35434 ± 0.10538	1.04 ± 0.31	2.91	3.78	0.0156 ± 0.0002
17D20192	12.5 %		0.429497	0.390	261.4397	0.491	0.1981185	12.315	8.80860	0.258	109.4415	0.095	0.39373 ± 0.11984	1.16 ± 0.35	3.11	2.94	0.0142 ± 0.0002
17D20193	13.4 %		0.456792	0.369	252.4960	0.494	0.1724663	13.750	7.30232	0.319	116.5246	0.089	0.23891 ± 0.14554	0.70 ± 0.43	1.46	2.43	0.0122 ± 0.0001
17D20195	14.6 %		0.522419	0.362	327.6385	0.481	0.2112405	11.274	8.16048	0.294	132.0606	0.080	0.48435 ± 0.14696	1.43 ± 0.43	2.92	2.70	0.0104 ± 0.0001
17D20196	16.0 %		0.358520	0.419	264.1702	0.490	0.1567698	15.693	5.77262	0.397	86.7348	0.120	0.33713 ± 0.16722	0.99 ± 0.49	2.18	1.91	0.0091 ± 0.0001
17D20198	17.6 %		0.337012	0.411	279.1899	0.486	0.1361922	17.179	5.33697	0.422	78.6977	0.132	0.27287 ± 0.16982	0.80 ± 0.50	1.79	1.75	0.0079 ± 0.0001
17D20199	19.3 %		0.213044	0.499	196.9944	0.519	0.0800204	29.224	3.40040	0.668	48.5179	0.214	0.39586 ± 0.20890	1.17 ± 0.61	2.67	1.11	0.0071 ± 0.0001
17D20201	21.0 %		0.089380	0.829	71.3120	0.780	0.0280023	83.235	1.38997	1.586	21.0274	0.490	0.23117 ± 0.36628	0.68 ± 1.08	1.48	0.46	0.0081 ± 0.0003
		Σ	10.031382	0.085	4810.1516	0.113	5.6946194	2.038	297.15219	0.042	2739.0268	0.019					

Information on Analysis and Constants Used in Calculations

Project = **O-CONNOR (16-22)**
Sample = **PS59-226-23**
Material = **Groundmass**
Location = **Gakkel Ridge**
Region = **Artic Ocean**
Analyst = **Dan Miggins**
Irradiation = **17-OSU-01 (1F23-17)**
Position = **X: 0 | Y: 0 | Z/H: 30.0291 mm**
FCT-NM Age = **28.201 ± 0.023 Ma**
FCT-NM Reference = **Kuiper et al (2008)**
FCT-NM 40Ar/39Ar Ratio = **9.64936 ± 0.00917**
FCT-NM J-value = **0.00162885 ± 0.00000155**
Air Shot 40Ar/36Ar = **302.4490 ± 0.4597**
Air Shot MDF = **0.99424863 ± 0.00068860 (LIN)**
Experiment Type = **Incremental Heating**
Extraction Method = **Bulk Laser Heating**
Heating = **64 sec**
Isolation = **3.00 min**
Instrument = **ARGUS-VI-D**
Preferred Age = **Plateau Age**
Age Classification = **Crystallization Age**
IGSN = Undefined
Rock Class = **Igneous>Volcanic**
Lithology = **Basaltic Lava**
Lat-Lon = Undefined - Undefined

Age Equations = **Min et al. (2000)**
Negative Intensities = **Allowed**
Collector Calibrations = **36Ar**
Decay 40K = **5.530 ± 0.048 E-10 1/a**
Decay 39Ar = **2.940 ± 0.016 E-07 1/h**
Decay 37Ar = **8.230 ± 0.012 E-04 1/h**
Decay 36Cl = **2.257 ± 0.015 E-06 1/a**
Decay 40K(EC,β^+) = **0.580 ± 0.009 E-10 1/a**
Decay 40K(β^-) = **4.950 ± 0.043 E-10 1/a**
Atmospheric 40/36(a) = **295.50**
Atmospheric 38/36(a) = **0.1869**
Production 39/37(ca) = **0.0006425 ± 0.0000059**
Production 38/37(ca) = **0.0001800 ± 0.0000173**
Production 36/37(ca) = **0.0002703 ± 0.0000005**
Production 40/39(k) = **0.000607 ± 0.000059**
Production 38/39(k) = **0.012077 ± 0.000011**
Production 36/38(cl) = **262.80 ± 1.71**
Scaling Ratio K/Ca = **0.430**
Abundance Ratio 40K/K = **1.1700 ± 0.0100 E-04**
Atomic Weight K = **39.0983 ± 0.0001 g**

Results

Results	40(a)/36(a) ± 2σ	40(r)/39(k) ± 2σ	Age ± 2σ (Ma)	MSWD	39Ar(k) (%,n)	K/Ca ± 2σ
**Age Plateau**		0.55967 ± 0.02960 ± 5.29%	**1.65 ± 0.09 ± 5.29%**	1.85 5%	44.75 11	0.0201 ± 0.0066
		Full External Error	± 0.09	1.89	2σ Confidence Limit	
		Analytical Error	± 0.09	1.3595	Error Magnification	
**Total Fusion Age**		0.53996 ± 0.01778 ± 3.29%	**1.59 ± 0.05 ± 3.30%**		24	0.0263 ± 0.0001
		Full External Error	± 0.06			
		Analytical Error	± 0.05			
**Normal Isochron**	**284.43 ± 8.61 ± 3.03%**	0.83313 ± 0.21425 ± 25.72%	**2.45 ± 0.63 ± 25.70%**	1.25 26%	44.75 11	
		Full External Error	± 0.63	1.94	2σ Confidence Limit	
		Analytical Error	± 0.63	1.1169	Error Magnification	
				5	Number of Iterations	
				0.0000024997	Convergence	
**Inverse Isochron** Clustered Points	**284.59 ± 8.54 ± 3.00%**	0.82946 ± 0.19558 ± 23.58%	**2.44 ± 0.58 ± 23.56%**	1.22 28%	44.75 11	
		Full External Error	± 0.58	1.94	2σ Confidence Limit	
		Analytical Error	± 0.58	1.1056	Error Magnification	
				3	Number of Iterations	
				0.0028313442	Convergence	
				3%	Spreading Factor	

Incremental Heating			36Ar(a) [fA]	37Ar(ca) [fA]	38Ar(cl) [fA]	39Ar(k) [fA]	40Ar(r) [fA]	Age ± 2σ (Ma)	40Ar(r) (%)	39Ar(k) (%)	K/Ca ± 2σ
17D20166	1.8 %		1.3322484	111.0664	0.0173611	49.97716	33.36858	1.97 ± 0.15	7.81	17.00	0.1935 ± 0.0024
17D20168	1.9 %		0.7269448	103.4334	0.0086663	31.86066	21.54641	1.99 ± 0.14	9.12	10.83	0.1325 ± 0.0017
17D20169	2.0 %	✓	0.3731031	60.5543	0.0000000	16.89017	10.15370	1.77 ± 0.16	8.43	5.74	0.1199 ± 0.0022
17D20171	2.2 %	✓	0.3293021	70.8654	0.0073904	14.81644	8.27467	1.64 ± 0.17	7.84	5.04	0.0899 ± 0.0014
17D20172	2.4 %	✓	0.3200917	90.8509	0.0144087	14.29249	8.35929	1.72 ± 0.17	8.12	4.86	0.0676 ± 0.0009
17D20174	2.7 %	✓	0.3260984	111.0070	0.0000000	13.42756	7.89866	1.73 ± 0.19	7.58	4.57	0.0520 ± 0.0007
17D20175	3.0 %	✓	0.2688424	113.2133	0.0000000	11.18531	6.43964	1.69 ± 0.22	7.50	3.80	0.0425 ± 0.0006
17D20177	3.4 %	✓	0.2867428	144.5023	0.0000000	11.12008	6.13137	1.62 ± 0.23	6.75	3.78	0.0331 ± 0.0004
17D20178	3.9 %	✓	0.2582327	142.2920	0.0000000	9.50897	5.11292	1.58 ± 0.25	6.28	3.23	0.0287 ± 0.0004
17D20180	4.5 %	✓	0.2396809	147.8003	0.0000000	8.28539	3.21720	1.14 ± 0.28	4.34	2.82	0.0241 ± 0.0003
17D20181	5.2 %	✓	0.3580124	300.3240	0.0000000	12.15008	6.37652	1.55 ± 0.26	5.68	4.13	0.0174 ± 0.0002
17D20183	6.0 %	✓	0.2878273	254.3588	0.0000000	9.60108	5.17005	1.59 ± 0.28	5.73	3.26	0.0162 ± 0.0002
17D20184	6.9 %	✓	0.2820535	311.5313	0.0000000	10.31787	5.83941	1.67 ± 0.27	6.55	3.51	0.0142 ± 0.0002
17D20186	7.9 %		0.2834153	313.0129	0.0000000	10.09086	4.97855	1.45 ± 0.28	5.61	3.43	0.0139 ± 0.0001
17D20187	9.0 %		0.2706072	278.7806	0.0000000	9.44127	4.08923	1.28 ± 0.27	4.86	3.21	0.0146 ± 0.0002
17D20189	10.3 %		0.3832702	295.6470	0.0000000	10.85986	4.02220	1.09 ± 0.29	3.43	3.69	0.0158 ± 0.0002
17D20190	11.6 %		0.4449187	307.6709	0.0000000	11.12727	3.94283	1.04 ± 0.31	2.91	3.78	0.0156 ± 0.0002
17D20192	12.5 %		0.3588296	261.4397	0.0000000	8.64063	3.40208	1.16 ± 0.35	3.11	2.94	0.0142 ± 0.0002
17D20193	13.4 %		0.3885428	252.4960	0.0000000	7.14009	1.70585	0.70 ± 0.43	1.46	2.43	0.0122 ± 0.0001
17D20195	14.6 %		0.4338587	327.6385	0.0000000	7.94997	3.85055	1.43 ± 0.43	2.92	2.70	0.0104 ± 0.0001
17D20196	16.0 %		0.2871152	264.1702	0.0000000	5.60289	1.88891	0.99 ± 0.49	2.18	1.91	0.0091 ± 0.0001
17D20198	17.6 %		0.2615473	279.1899	0.0000000	5.15759	1.40734	0.80 ± 0.50	1.79	1.75	0.0079 ± 0.0001
17D20199	19.3 %		0.1597966	196.9944	0.0000000	3.27384	1.29598	1.17 ± 0.61	2.67	1.11	0.0071 ± 0.0001
17D20201	21.0 %		0.0701043	71.3120	0.0000000	1.34415	0.31073	0.68 ± 1.08	1.48	0.46	0.0081 ± 0.0003
		Σ	8.7311866	4810.1516	0.0478265	294.06167	158.78265				

Information on Analysis

Project = **O-CONNOR (16-22)**
Sample = **PS59-226-23**
Material = **Groundmass**
Location = **Gakkel Ridge**
Region = **Artic Ocean**
Analyst = **Dan Miggins**
Irradiation = **17-OSU-01 (1F23-17)**
J = **0.00162885 ± 0.00000155**
FCT-NM = **28.201 ± 0.023 Ma**

Results	40(r)/39(k) ± 2σ		Age ± 2σ (Ma)		MSWD	39Ar(k) (%,n)	K/Ca ± 2σ
**Age Plateau**	0.55967	± 0.02960<br>± 5.29%	**1.65**	**± 0.09**<br>**± 5.29%**	1.85<br>5%	44.75<br>11	0.0201 ± 0.0066
		Full External Error		± 0.09	1.89	2σ Confidence Limit	
		Analytical Error		± 0.09	1.3595	Error Magnification	
**Total Fusion Age**	0.53996	± 0.01778<br>± 3.29%	**1.59**	**± 0.05**<br>**± 3.30%**		24	0.0263 ± 0.0001
		Full External Error		± 0.06			
		Analytical Error		± 0.05			

Normal Isochron			39(k)/36(a) ± 2σ	40(a+r)/36(a) ± 2σ	r.i.
17D20166	1.8 %		37.51 ± 0.25	320.55 ± 2.05	0.9634
17D20168	1.9 %		43.83 ± 0.32	325.14 ± 2.28	0.9488
17D20169	2.0 %	✓	45.27 ± 0.40	322.71 ± 2.73	0.9167
17D20171	2.2 %	✓	44.99 ± 0.41	320.63 ± 2.82	0.9085
17D20172	2.4 %	✓	44.65 ± 0.42	321.62 ± 2.86	0.9029
17D20174	2.7 %	✓	41.18 ± 0.39	319.72 ± 2.87	0.8927
17D20175	3.0 %	✓	41.61 ± 0.46	319.45 ± 3.32	0.8894
17D20177	3.4 %	✓	38.78 ± 0.42	316.88 ± 3.19	0.8830
17D20178	3.9 %	✓	36.82 ± 0.42	315.30 ± 3.36	0.8729
17D20180	4.5 %	✓	34.57 ± 0.42	308.92 ± 3.37	0.8469
17D20181	5.2 %	✓	33.94 ± 0.36	313.31 ± 3.16	0.9085
17D20183	6.0 %	✓	33.36 ± 0.38	313.46 ± 3.31	0.8835
17D20184	6.9 %	✓	36.58 ± 0.44	316.20 ± 3.56	0.9038
17D20186	7.9 %		35.60 ± 0.43	313.07 ± 3.58	0.8981
17D20187	9.0 %		34.89 ± 0.41	310.61 ± 3.41	0.8810
17D20189	10.3 %		28.33 ± 0.29	305.99 ± 2.87	0.8853
17D20190	11.6 %		25.01 ± 0.24	304.36 ± 2.71	0.8844
17D20192	12.5 %		24.08 ± 0.26	304.98 ± 2.97	0.8588
17D20193	13.4 %		18.38 ± 0.20	299.89 ± 2.71	0.7885
17D20195	14.6 %		18.32 ± 0.20	304.38 ± 2.77	0.8156
17D20196	16.0 %		19.51 ± 0.26	302.08 ± 3.33	0.7765
17D20198	17.6 %		19.72 ± 0.28	300.88 ± 3.41	0.7605
17D20199	19.3 %		20.49 ± 0.40	303.61 ± 4.39	0.6730
17D20201	21.0 %		19.17 ± 0.75	299.93 ± 7.11	0.5002

Results	40(a)/36(a) ± 2σ		40(r)/39(k) ± 2σ		Age ± 2σ (Ma)		MSWD
Normal Isochron	**284.43**	**± 8.61**<br>**± 3.03%**	0.83313	± 0.21425<br>± 25.72%	**2.45**	**± 0.63**<br>**± 25.70%**	1.25<br>26%
					Full External Error	± 0.63	
					Analytical Error	± 0.63	

Statistics				
	2σ Confidence Limit	1.94	Convergence	0.000002499687
	Error Magnification	1.1169	Number of Iterations	5
	Number of Data Points	11	Calculated Line	Weighted York-2

**Inverse Isochron**			**39(k)/40(a+r) ± 2σ**	**36(a)/40(a+r) ± 2σ**	**r.i.**
17D20166	1.8 %		0.1170294 ± 0.0002071	0.00311967 ± 0.00001996	0.0236
17D20168	1.9 %		0.1347980 ± 0.0003079	0.00307560 ± 0.00002155	0.0734
17D20169	2.0 %	✓	0.1402772 ± 0.0004984	0.00309872 ± 0.00002623	0.1003
17D20171	2.2 %	✓	0.1403292 ± 0.0005422	0.00311888 ± 0.00002741	0.1148
17D20172	2.4 %	✓	0.1388343 ± 0.0005603	0.00310930 ± 0.00002764	0.1155
17D20174	2.7 %	✓	0.1287882 ± 0.0005566	0.00312772 ± 0.00002803	0.1031
17D20175	3.0 %	✓	0.1302395 ± 0.0006624	0.00313035 ± 0.00003257	0.1123
17D20177	3.4 %	✓	0.1223817 ± 0.0006247	0.00315574 ± 0.00003179	0.1018
17D20178	3.9 %	✓	0.1167882 ± 0.0006588	0.00317159 ± 0.00003377	0.1091
17D20180	4.5 %	✓	0.1118998 ± 0.0007210	0.00323705 ± 0.00003535	0.1124
17D20181	5.2 %	✓	0.1083193 ± 0.0004865	0.00319172 ± 0.00003218	0.0769
17D20183	6.0 %	✓	0.1064150 ± 0.0005696	0.00319018 ± 0.00003366	0.0932
17D20184	6.9 %	✓	0.1156891 ± 0.0005928	0.00316252 ± 0.00003560	0.0942
17D20186	7.9 %		0.1137283 ± 0.0006130	0.00319421 ± 0.00003656	0.0888
17D20187	9.0 %		0.1123244 ± 0.0006310	0.00321946 ± 0.00003531	0.0994
17D20189	10.3 %		0.0925988 ± 0.0004408	0.00326803 ± 0.00003066	0.0713
17D20190	11.6 %		0.0821708 ± 0.0003751	0.00328556 ± 0.00002927	0.0603
17D20192	12.5 %		0.0789558 ± 0.0004428	0.00327889 ± 0.00003198	0.0661
17D20193	13.4 %		0.0612777 ± 0.0004161	0.00333455 ± 0.00003016	0.0521
17D20195	14.6 %		0.0602016 ± 0.0003772	0.00328542 ± 0.00002991	0.0448
17D20196	16.0 %		0.0646004 ± 0.0005519	0.00331039 ± 0.00003651	0.0611
17D20198	17.6 %		0.0655394 ± 0.0006004	0.00332358 ± 0.00003762	0.0671
17D20199	19.3 %		0.0674797 ± 0.0009810	0.00329370 ± 0.00004757	0.0871
17D20201	21.0 %		0.0639263 ± 0.0021896	0.00333409 ± 0.00007902	0.1183

**Results**	**40(a)/36(a) ± 2σ**	**40(r)/39(k) ± 2σ**	**Age ± 2σ (Ma)**	**MSWD**
**Inverse Isochron** Clustered Points	**284.59 ± 8.54 ± 3.00%**	0.82946 ± 0.19558 ± 23.58%	**2.44 ± 0.58 ± 23.56%**	1.22 28%
			Full External Error ± 0.58	
			Analytical Error ± 0.58	

**Statistics**				
	2σ Confidence Limit	1.94	Convergence	0.0028313442
	Error Magnification	1.1056	Number of Iterations	3
	Number of Data Points	11	Calculated Line	Weighted York-2
	Spreading Factor	2.8%		

**Degassing Patterns**			36Ar(a) [fA]	%1σ	36Ar(c) [fA]	%1σ	36Ar(ca) [fA]	%1σ	36Ar(cl) [fA]	%1σ	37Ar(ca) [fA]	%1σ	38Ar(a) [fA]	%1σ	38Ar(c) [fA]	%1σ	38Ar(k) [fA]	%1σ	38Ar(ca) [fA]	%1σ	38Ar(cl) [fA]	%1σ	39Ar(k) [fA]	%1σ	39Ar(ca) [fA]	%1σ	40Ar(r) [fA]	%1σ	40Ar(a) [fA]	%1σ	40Ar(c) [fA]	%1σ	40Ar(k) [fA]	%1σ
17D20166	1.8 %		1.3322484	0.32	0.0000000	0.00	0.0300213	0.63	0.0000041	136.70	111.0664	0.61	0.2489972	0.32	0.0000000	0.00	0.6035742	0.12	0.0199920	9.65	0.0173611	136.70	49.97716	0.08	0.0713602	1.10	33.36858	3.78	393.6794	0.32	0.0000000	0.00	0.0303361	9.65
17D20168	1.9 %		0.7269448	0.35	0.0000000	0.00	0.0279580	0.65	0.0000020	280.82	103.4334	0.63	0.1358660	0.35	0.0000000	0.00	0.3847812	0.13	0.0186180	9.65	0.0086663	280.82	31.86066	0.10	0.0664560	1.12	21.54641	3.50	214.8122	0.35	0.0000000	0.00	0.0193394	9.65
17D20169	2.0 %	✓	0.3731031	0.41	0.0000000	0.00	0.0163678	0.91	0.0000000	0.00	60.5543	0.90	0.0697330	0.41	0.0000000	0.00	0.2039826	0.18	0.0108998	9.67	0.0000000	0.00	16.89017	0.15	0.0389061	1.28	10.15370	4.61	110.2520	0.41	0.0000000	0.00	0.0102523	9.65
17D20171	2.2 %	✓	0.3293021	0.43	0.0000000	0.00	0.0191549	0.80	0.0000017	310.46	70.8654	0.78	0.0615466	0.43	0.0000000	0.00	0.1789382	0.19	0.0127558	9.66	0.0073904	310.46	14.81644	0.17	0.0455310	1.20	8.27467	5.19	97.3088	0.43	0.0000000	0.00	0.0089936	9.65
17D20172	2.4 %	✓	0.3200917	0.43	0.0000000	0.00	0.0245570	0.68	0.0000034	169.60	90.8509	0.66	0.0598251	0.43	0.0000000	0.00	0.1726104	0.20	0.0163532	9.65	0.0144087	169.60	14.29249	0.17	0.0583717	1.13	8.35929	5.05	94.5871	0.43	0.0000000	0.00	0.0086755	9.65
17D20174	2.7 %	✓	0.3260984	0.44	0.0000000	0.00	0.0300052	0.63	0.0000000	0.00	111.0070	0.60	0.0609478	0.44	0.0000000	0.00	0.1621646	0.21	0.0199813	9.65	0.0000000	0.00	13.42756	0.19	0.0713220	1.10	7.89866	5.49	96.3621	0.44	0.0000000	0.00	0.0081505	9.65
17D20175	3.0 %	✓	0.2688424	0.51	0.0000000	0.00	0.0306016	0.64	0.0000000	0.00	113.2133	0.61	0.0502466	0.51	0.0000000	0.00	0.1350849	0.24	0.0203784	9.65	0.0000000	0.00	11.18531	0.22	0.0727396	1.11	6.43964	6.45	79.4429	0.51	0.0000000	0.00	0.0067895	9.65
17D20177	3.4 %	✓	0.2867428	0.49	0.0000000	0.00	0.0390590	0.58	0.0000000	0.00	144.5023	0.55	0.0535922	0.49	0.0000000	0.00	0.1342972	0.25	0.0260104	9.65	0.0000000	0.00	11.12008	0.23	0.0928427	1.07	6.13137	6.99	84.7325	0.49	0.0000000	0.00	0.0067499	9.65
17D20178	3.9 %	✓	0.2582327	0.52	0.0000000	0.00	0.0384615	0.58	0.0000000	0.00	142.2920	0.56	0.0482637	0.52	0.0000000	0.00	0.1148399	0.27	0.0256126	9.65	0.0000000	0.00	9.50897	0.25	0.0914226	1.08	5.11292	7.98	76.3078	0.52	0.0000000	0.00	0.0057719	9.65
17D20180	4.5 %	✓	0.2396809	0.53	0.0000000	0.00	0.0399504	0.58	0.0000000	0.00	147.8003	0.55	0.0447964	0.53	0.0000000	0.00	0.1000626	0.30	0.0266041	9.65	0.0000000	0.00	8.28539	0.29	0.0949617	1.07	3.21720	12.06	70.8257	0.53	0.0000000	0.00	0.0050292	9.65
17D20181	5.2 %	✓	0.3580124	0.50	0.0000000	0.00	0.0811776	0.51	0.0000000	0.00	300.3240	0.48	0.0669125	0.50	0.0000000	0.00	0.1467365	0.22	0.0540583	9.64	0.0000000	0.00	12.15008	0.20	0.1929582	1.04	6.37652	8.38	105.7927	0.50	0.0000000	0.00	0.0073751	9.65
17D20183	6.0 %	✓	0.2878273	0.51	0.0000000	0.00	0.0687532	0.52	0.0000000	0.00	254.3588	0.49	0.0537949	0.51	0.0000000	0.00	0.1159523	0.26	0.0457846	9.64	0.0000000	0.00	9.60108	0.24	0.1634256	1.04	5.17005	8.70	85.0530	0.51	0.0000000	0.00	0.0058279	9.65
17D20184	6.9 %	✓	0.2820535	0.55	0.0000000	0.00	0.0842069	0.51	0.0000000	0.00	311.5313	0.49	0.0527158	0.55	0.0000000	0.00	0.1246089	0.25	0.0560756	9.64	0.0000000	0.00	10.31787	0.23	0.2001589	1.04	5.83941	8.06	83.3468	0.55	0.0000000	0.00	0.0062629	9.65
17D20186	7.9 %		0.2834153	0.56	0.0000000	0.00	0.0846074	0.51	0.0000000	0.00	313.0129	0.48	0.0529703	0.56	0.0000000	0.00	0.1218673	0.26	0.0563423	9.64	0.0000000	0.00	10.09086	0.24	0.2011108	1.04	4.97855	9.65	83.7492	0.56	0.0000000	0.00	0.0061252	9.65
17D20187	9.0 %		0.2706072	0.53	0.0000000	0.00	0.0753544	0.52	0.0000000	0.00	278.7806	0.49	0.0505765	0.53	0.0000000	0.00	0.1140223	0.27	0.0501805	9.64	0.0000000	0.00	9.44127	0.25	0.1791165	1.04	4.08923	10.75	79.9644	0.53	0.0000000	0.00	0.0057309	9.65
17D20189	10.3 %		0.3832702	0.46	0.0000000	0.00	0.0799134	0.51	0.0000000	0.00	295.6470	0.49	0.0716332	0.46	0.0000000	0.00	0.1311545	0.24	0.0532165	9.64	0.0000000	0.00	10.85986	0.22	0.1899532	1.04	4.02220	13.22	113.2563	0.46	0.0000000	0.00	0.0065919	9.65
17D20190	11.6 %		0.4449187	0.44	0.0000000	0.00	0.0831634	0.51	0.0000000	0.00	307.6709	0.48	0.0831553	0.44	0.0000000	0.00	0.1343840	0.23	0.0553808	9.64	0.0000000	0.00	11.12727	0.21	0.1976786	1.04	3.94283	14.87	131.4735	0.44	0.0000000	0.00	0.0067543	9.65
17D20192	12.5 %		0.3588296	0.48	0.0000000	0.00	0.0706671	0.52	0.0000000	0.00	261.4397	0.49	0.0670652	0.48	0.0000000	0.00	0.1043528	0.28	0.0470591	9.64	0.0000000	0.00	8.64063	0.26	0.1679750	1.04	3.40208	15.22	106.0341	0.48	0.0000000	0.00	0.0052449	9.65
17D20193	13.4 %		0.3885428	0.44	0.0000000	0.00	0.0682497	0.52	0.0000000	0.00	252.4960	0.49	0.0726186	0.44	0.0000000	0.00	0.0862308	0.34	0.0454493	9.64	0.0000000	0.00	7.14009	0.33	0.1622287	1.04	1.70585	30.46	114.8144	0.44	0.0000000	0.00	0.0043340	9.66
17D20195	14.6 %		0.4338587	0.45	0.0000000	0.00	0.0885607	0.51	0.0000000	0.00	327.6385	0.48	0.0810882	0.45	0.0000000	0.00	0.0960118	0.32	0.0589749	9.64	0.0000000	0.00	7.94997	0.30	0.2105077	1.04	3.85055	15.17	128.2053	0.45	0.0000000	0.00	0.0048256	9.65
17D20196	16.0 %		0.2871152	0.54	0.0000000	0.00	0.0714052	0.52	0.0000000	0.00	264.1702	0.49	0.0536618	0.54	0.0000000	0.00	0.0676661	0.42	0.0475506	9.64	0.0000000	0.00	5.60289	0.41	0.1697294	1.04	1.88891	24.80	84.8425	0.54	0.0000000	0.00	0.0034010	9.66
17D20198	17.6 %		0.2615473	0.55	0.0000000	0.00	0.0754650	0.51	0.0000000	0.00	279.1899	0.49	0.0488832	0.55	0.0000000	0.00	0.0622882	0.45	0.0502542	9.64	0.0000000	0.00	5.15759	0.44	0.1793795	1.04	1.40734	31.11	77.2872	0.55	0.0000000	0.00	0.0031307	9.66
17D20199	19.3 %		0.1597966	0.69	0.0000000	0.00	0.0532476	0.55	0.0000000	0.00	196.9944	0.52	0.0298660	0.69	0.0000000	0.00	0.0395381	0.70	0.0354590	9.64	0.0000000	0.00	3.27384	0.69	0.1265689	1.06	1.29598	26.38	47.2199	0.69	0.0000000	0.00	0.0019872	9.67
17D20201	21.0 %		0.0701043	1.08	0.0000000	0.00	0.0192756	0.80	0.0000000	0.00	71.3120	0.78	0.0131025	1.08	0.0000000	0.00	0.0162333	1.64	0.0128362	9.66	0.0000000	0.00	1.34415	1.64	0.0458179	1.21	0.31073	79.21	20.7158	1.08	0.0000000	0.00	0.0008159	9.79
		Σ	8.7311866	0.10	0.0000000	0.00	1.3001840	0.12	0.0000112	99.82	4810.1516	0.11	1.6318588	0.10	0.0000000	0.00	3.5513828	0.05	0.8658273	2.17	0.0478265	99.82	294.06167	0.04	3.0905224	0.24	158.78265	1.65	2580.0656	0.10	0.0000000	0.00	0.1784954	2.51
		Σ							10.031382	0.09	4810.1516	0.11									6.0968953	0.84			297.15219	0.04							2739.0268	0.13

**Additional Parameters**			**40Ar/39Ar**	**1σ**	**37Ar/39Ar**	**1σ**	**36Ar/39Ar**	**1σ**	**Time (days)**	**37Ar (decay)**	**39Ar (decay)**	**40Ar (moles)**
17D20166	1.8 %		8.533285	0.007538	2.219175	0.013631	0.027219	0.000088	144.281	17.327784	1.00101945	2.050E-11
17D20168	1.9 %		7.403673	0.008438	3.239672	0.020682	0.023645	0.000082	144.295	17.332538	1.00101955	1.135E-11
17D20169	2.0 %	✓	7.112964	0.012612	3.576941	0.032490	0.023006	0.000098	144.302	17.334916	1.00101960	5.780E-12
17D20171	2.2 %	✓	7.104873	0.013693	4.768235	0.037887	0.023446	0.000102	144.316	17.339672	1.00101970	5.068E-12
17D20172	2.4 %	✓	7.174140	0.014428	6.330697	0.043247	0.024016	0.000104	144.323	17.342051	1.00101974	4.942E-12
17D20174	2.7 %	✓	7.724264	0.016616	8.223427	0.052143	0.026380	0.000116	144.337	17.346809	1.00101984	5.005E-12
17D20175	3.0 %	✓	7.629153	0.019296	10.056214	0.065603	0.026598	0.000133	144.344	17.349189	1.00101989	4.123E-12
17D20177	3.4 %	✓	8.104099	0.020535	12.887126	0.076960	0.029056	0.000140	144.358	17.353949	1.00101999	4.362E-12
17D20178	3.9 %	✓	8.481575	0.023725	14.821476	0.090345	0.030904	0.000157	144.365	17.356329	1.00102004	3.908E-12
17D20180	4.5 %	✓	8.835902	0.028184	17.636532	0.109583	0.033368	0.000176	144.378	17.361091	1.00102014	3.554E-12
17D20181	5.2 %	✓	9.088243	0.020092	24.331443	0.127436	0.035582	0.000157	144.385	17.363473	1.00102019	5.384E-12
17D20183	6.0 %	✓	9.240491	0.024339	26.049323	0.142436	0.036518	0.000171	144.399	17.368237	1.00102028	4.331E-12
17D20184	6.9 %	✓	8.479961	0.021333	29.618793	0.158149	0.034822	0.000162	144.406	17.370619	1.00102033	4.281E-12
17D20186	7.9 %		8.621663	0.022801	30.413314	0.163480	0.035758	0.000171	144.420	17.375385	1.00102043	4.259E-12
17D20187	9.0 %		8.737628	0.024117	28.978096	0.158579	0.035961	0.000170	144.427	17.377769	1.00102048	4.035E-12
17D20189	10.3 %		10.614222	0.024820	26.755843	0.142193	0.041918	0.000180	144.442	17.382775	1.00102058	5.630E-12
17D20190	11.6 %		11.957943	0.026788	27.167537	0.142523	0.046630	0.000195	144.449	17.385160	1.00102063	6.500E-12
17D20192	12.5 %		12.424386	0.034165	29.680046	0.164715	0.048759	0.000228	144.463	17.389930	1.00102073	5.253E-12
17D20193	13.4 %		15.957206	0.052923	34.577517	0.203290	0.062554	0.000305	144.469	17.392315	1.00102078	5.593E-12
17D20195	14.6 %		16.182955	0.049283	40.149429	0.226442	0.064018	0.000298	144.483	17.397087	1.00102088	6.339E-12
17D20196	16.0 %		15.025217	0.062281	45.762638	0.288418	0.062107	0.000358	144.490	17.399474	1.00102093	4.163E-12
17D20198	17.6 %		14.745761	0.065263	52.312428	0.336886	0.063147	0.000372	144.504	17.404247	1.00102102	3.777E-12
17D20199	19.3 %		14.268265	0.100034	57.932649	0.489798	0.062653	0.000522	144.511	17.406635	1.00102107	2.329E-12
17D20201	21.0 %		15.127947	0.251179	51.304712	0.906897	0.064304	0.001151	144.525	17.411411	1.00102117	1.009E-12

Procedure Blanks		36Ar ± 1σ (SE) [fA]	37Ar ± 1σ (SE) [fA]	38Ar ± 1σ (SE) [fA]	39Ar ± 1σ (SE) [fA]	40Ar ± 1σ (SE) [fA]
17D20166	1.8 %	0.0081644 ± 0.0004666	0.1471009 ± 0.0180843	0.0252987 ± 0.0166821	0.0249743 ± 0.0156487	2.2940814 ± 0.1015967
17D20168	1.9 %	0.0090885 ± 0.0004666	0.1410047 ± 0.0180843	0.0165811 ± 0.0166821	0.0176298 ± 0.0156487	2.5779217 ± 0.1015967
17D20169	2.0 %	0.0093647 ± 0.0004666	0.1394675 ± 0.0180843	0.0144356 ± 0.0166821	0.0157731 ± 0.0156487	2.6622018 ± 0.1015967
17D20171	2.2 %	0.0096203 ± 0.0004666	0.1387496 ± 0.0180843	0.0134240 ± 0.0166821	0.0147967 ± 0.0156487	2.7390061 ± 0.1015967
17D20172	2.4 %	0.0096274 ± 0.0004666	0.1393254 ± 0.0180843	0.0141461 ± 0.0166821	0.0153530 ± 0.0156487	2.7401730 ± 0.1015967
17D20174	2.7 %	0.0094632 ± 0.0004666	0.1417931 ± 0.0180843	0.0171339 ± 0.0166821	0.0178293 ± 0.0156487	2.6877252 ± 0.1015967
17D20175	3.0 %	0.0093148 ± 0.0004666	0.1434859 ± 0.0180843	0.0190819 ± 0.0166821	0.0194928 ± 0.0156487	2.6412189 ± 0.1015967
17D20177	3.4 %	0.0089360 ± 0.0004666	0.1473476 ± 0.0180843	0.0232090 ± 0.0166821	0.0231136 ± 0.0156487	2.5234943 ± 0.1015967
17D20178	3.9 %	0.0087236 ± 0.0004666	0.1493621 ± 0.0180843	0.0251644 ± 0.0166821	0.0248819 ± 0.0156487	2.4578501 ± 0.1015967
17D20180	4.5 %	0.0082915 ± 0.0004666	0.1532276 ± 0.0180843	0.0284174 ± 0.0166821	0.0279463 ± 0.0156487	2.3250153 ± 0.1015967
17D20181	5.2 %	0.0080849 ± 0.0004666	0.1549689 ± 0.0180843	0.0295852 ± 0.0166821	0.0291208 ± 0.0156487	2.2618645 ± 0.1015967
17D20183	6.0 %	0.0077173 ± 0.0004666	0.1578492 ± 0.0180843	0.0307973 ± 0.0166821	0.0305350 ± 0.0156487	2.1502773 ± 0.1015967
17D20184	6.9 %	0.0075644 ± 0.0004666	0.1589229 ± 0.0180843	0.0308060 ± 0.0166821	0.0307205 ± 0.0156487	2.1043464 ± 0.1015967
17D20186	7.9 %	0.0073351 ± 0.0004666	0.1602301 ± 0.0180843	0.0296571 ± 0.0166821	0.0299978 ± 0.0156487	2.0365559 ± 0.1015967
17D20187	9.0 %	0.0072621 ± 0.0004666	0.1604428 ± 0.0180843	0.0285582 ± 0.0166821	0.0291029 ± 0.0156487	2.0156674 ± 0.1015967
17D20189	10.3 %	0.0072012 ± 0.0004666	0.1599381 ± 0.0180843	0.0254063 ± 0.0166821	0.0261978 ± 0.0156487	2.0005554 ± 0.1015967
17D20190	11.6 %	0.0072147 ± 0.0004666	0.1592707 ± 0.0180843	0.0236667 ± 0.0166821	0.0244128 ± 0.0156487	2.0064279 ± 0.1015967
17D20192	12.5 %	0.0073135 ± 0.0004666	0.1572300 ± 0.0180843	0.0202408 ± 0.0166821	0.0203618 ± 0.0156487	2.0399771 ± 0.1015967
17D20193	13.4 %	0.0073922 ± 0.0004666	0.1559265 ± 0.0180843	0.0188042 ± 0.0166821	0.0182465 ± 0.0156487	2.0655052 ± 0.1015967
17D20195	14.6 %	0.0075862 ± 0.0004666	0.1529839 ± 0.0180843	0.0172258 ± 0.0166821	0.0142967 ± 0.0156487	2.1267790 ± 0.1015967
17D20196	16.0 %	0.0076900 ± 0.0004666	0.1514593 ± 0.0180843	0.0174279 ± 0.0166821	0.0126804 ± 0.0156487	2.1588418 ± 0.1015967
17D20198	17.6 %	0.0078767 ± 0.0004666	0.1486452 ± 0.0180843	0.0207919 ± 0.0166821	0.0107942 ± 0.0156487	2.2146942 ± 0.1015967
17D20199	19.3 %	0.0079434 ± 0.0004666	0.1475148 ± 0.0180843	0.0243918 ± 0.0166821	0.0108098 ± 0.0156487	2.2332666 ± 0.1015967
17D20201	21.0 %	0.0079767 ± 0.0004666	0.1462608 ± 0.0180843	0.0366399 ± 0.0166821	0.0135566 ± 0.0156487	2.2367427 ± 0.1015967

**Intercept Values**		**36Ar ± 1σ (SE) [fA]**	**r2**	**Regression (type,n)**	**37Ar ± 1σ (SE) [fA]**	**r2**	**Regression (type,n)**	**38Ar ± 1σ (SE) [fA]**	**r2**	**Regression (type,n)**	**39Ar ± 1σ (SE) [fA]**	**r2**	**Regression (type,n)**	**40Ar ± 1σ (SE) [fA]**	**r2**	**Regression (type,n)**
17D20166	1.8 %	1.3191732 ± 0.0016821	0.9608	EXP 150 of 150	6.1521979 ± 0.0175154	0.8111	EXP 150 of 150	0.8543910 ± 0.0162994	0.0649	EXP 150 of 150	49.6855792 ± 0.0183680	0.9967	EXP 150 of 150	429.372402 ± 0.043071	0.9941	EXP 150 of 150
17D20168	1.9 %	0.7355849 ± 0.0011969	0.9265	EXP 150 of 150	5.7237656 ± 0.0177845	0.7860	EXP 150 of 150	0.5250489 ± 0.0172102	0.0620	EXP 150 of 150	31.6938871 ± 0.0169055	0.9927	EXP 150 of 150	238.955859 ± 0.077963	0.7374	EXP 150 of 150
17D20169	2.0 %	0.3841791 ± 0.0009275	0.7739	EXP 150 of 150	3.2935480 ± 0.0192120	0.5296	EXP 150 of 150	0.2526460 ± 0.0162840	0.0149	EXP 150 of 150	16.7989842 ± 0.0171947	0.9712	EXP 150 of 150	123.078133 ± 0.024778	0.9977	EXP 150 of 150
17D20171	2.2 %	0.3449659 ± 0.0008430	0.7577	EXP 149 of 150	3.8777316 ± 0.0175401	0.6718	EXP 150 of 150	0.2442094 ± 0.0153053	0.0265	EXP 150 of 150	14.7468145 ± 0.0157490	0.9692	EXP 149 of 150	108.331452 ± 0.023282	0.9975	EXP 150 of 150
17D20172	2.4 %	0.3413096 ± 0.0008124	0.7944	EXP 150 of 150	5.0091825 ± 0.0164971	0.7445	EXP 150 of 150	0.2460242 ± 0.0173894	0.0494	EXP 150 of 150	14.2385920 ± 0.0164123	0.9632	EXP 150 of 150	105.695238 ± 0.025607	0.9970	EXP 150 of 150
17D20174	2.7 %	0.3521660 ± 0.0008353	0.8054	EXP 150 of 150	6.1472318 ± 0.0169362	0.7878	EXP 149 of 150	0.2144031 ± 0.0168464	0.0052	EXP 150 of 150	13.3898873 ± 0.0179340	0.9505	EXP 150 of 150	106.956617 ± 0.023055	0.9967	EXP 150 of 150
17D20175	3.0 %	0.2974901 ± 0.0008948	0.6749	EXP 149 of 150	6.2696545 ± 0.0188490	0.7956	EXP 150 of 150	0.1761872 ± 0.0173379	0.0096	EXP 150 of 150	11.1625242 ± 0.0175924	0.9352	EXP 150 of 150	88.530572 ± 0.025277	0.9969	EXP 150 of 150
17D20177	3.4 %	0.3224772 ± 0.0008878	0.7443	EXP 150 of 150	8.0359608 ± 0.0175664	0.8680	EXP 150 of 150	0.1839440 ± 0.0159633	0.0240	EXP 150 of 150	11.1140826 ± 0.0181637	0.9305	EXP 150 of 150	93.394118 ± 0.021987	0.9967	EXP 150 of 150
17D20178	3.9 %	0.2942527 ± 0.0008594	0.7146	EXP 150 of 150	7.9076696 ± 0.0177814	0.8640	EXP 150 of 150	0.1338459 ± 0.0171924	0.0061	EXP 150 of 150	9.5106772 ± 0.0165573	0.9180	EXP 150 of 150	83.884313 ± 0.023279	0.9968	EXP 150 of 150
17D20180	4.5 %	0.2773998 ± 0.0008000	0.7251	EXP 150 of 150	8.2134041 ± 0.0180848	0.8765	EXP 150 of 150	0.1287722 ± 0.0154244	0.0001	EXP 150 of 150	8.2958052 ± 0.0170294	0.8787	EXP 150 of 150	76.372955 ± 0.022679	0.9967	EXP 150 of 150
17D20181	5.2 %	0.4307474 ± 0.0010580	0.8330	EXP 150 of 150	16.8433452 ± 0.0187464	0.9649	EXP 150 of 150	0.2134797 ± 0.0160458	0.0002	EXP 150 of 150	12.2305604 ± 0.0169430	0.9466	EXP 150 of 150	114.438423 ± 0.025067	0.9911	EXP 150 of 150
17D20183	6.0 %	0.3508790 ± 0.0008739	0.8136	EXP 149 of 150	14.2348921 ± 0.0183616	0.9525	EXP 150 of 150	0.1670172 ± 0.0155099	0.0056	EXP 150 of 150	9.6680276 ± 0.0154603	0.9274	EXP 150 of 150	92.379130 ± 0.019708	0.9959	EXP 150 of 150
17D20184	6.9 %	0.3600418 ± 0.0009263	0.7915	EXP 150 of 150	17.4664712 ± 0.0209724	0.9602	EXP 150 of 150	0.1644103 ± 0.0163722	0.0109	EXP 150 of 150	10.4162730 ± 0.0156661	0.9420	EXP 150 of 150	91.296823 ± 0.021957	0.9955	EXP 150 of 150
17D20186	7.9 %	0.3615084 ± 0.0009739	0.7846	EXP 150 of 150	17.5441284 ± 0.0190009	0.9654	EXP 150 of 150	0.1742591 ± 0.0147450	0.0063	EXP 150 of 150	10.1924618 ± 0.0171145	0.9222	EXP 150 of 150	90.770450 ± 0.021561	0.9947	EXP 150 of 150
17D20187	9.0 %	0.3402045 ± 0.0008357	0.8149	EXP 150 of 150	15.6055354 ± 0.0191005	0.9563	EXP 150 of 150	0.1503642 ± 0.0155555	0.0004	EXP 150 of 150	9.5263125 ± 0.0163497	0.9199	EXP 150 of 150	86.075062 ± 0.022214	0.9951	EXP 150 of 150
17D20189	10.3 %	0.4529543 ± 0.0009672	0.8725	EXP 150 of 150	16.5550748 ± 0.0192191	0.9628	EXP 150 of 150	0.1989445 ± 0.0163277	0.0041	EXP 150 of 150	10.9489815 ± 0.0161333	0.9365	EXP 150 of 150	119.285686 ± 0.025166	0.9673	EXP 150 of 150
17D20190	11.6 %	0.5154241 ± 0.0010461	0.9031	EXP 150 of 150	17.2331542 ± 0.0169870	0.9710	EXP 150 of 150	0.2343043 ± 0.0178242	0.0145	EXP 150 of 150	11.2240445 ± 0.0158768	0.9427	EXP 150 of 150	137.429482 ± 0.030368	0.7598	EXP 149 of 150
17D20192	12.5 %	0.4206474 ± 0.0010151	0.8409	EXP 150 of 150	14.6177225 ± 0.0184494	0.9527	EXP 150 of 150	0.1755992 ± 0.0174147	0.0004	EXP 150 of 150	8.7287451 ± 0.0151048	0.9140	EXP 150 of 150	111.481439 ± 0.022499	0.9658	EXP 150 of 150
17D20193	13.4 %	0.4469947 ± 0.0009384	0.8979	EXP 150 of 150	14.1116262 ± 0.0184545	0.9491	EXP 150 of 150	0.1516786 ± 0.0164670	0.0011	EXP 150 of 150	7.2347495 ± 0.0163273	0.8503	EXP 150 of 150	118.590078 ± 0.022992	0.9294	EXP 149 of 150
17D20195	14.6 %	0.5103460 ± 0.0010435	0.8967	EXP 149 of 150	18.3554966 ± 0.0196810	0.9656	EXP 150 of 150	0.1915853 ± 0.0166077	0.0000	EXP 150 of 150	8.0910621 ± 0.0170592	0.8821	EXP 150 of 150	134.187406 ± 0.028769	0.0359	EXP 150 of 150
17D20196	16.0 %	0.3527185 ± 0.0009612	0.8007	EXP 150 of 150	14.7696177 ± 0.0177698	0.9563	EXP 150 of 150	0.1375390 ± 0.0176935	0.0000	EXP 150 of 150	5.7209476 ± 0.0160304	0.7963	EXP 150 of 150	88.893681 ± 0.022328	0.9855	EXP 150 of 150
17D20198	17.6 %	0.3322066 ± 0.0008532	0.8320	EXP 150 of 150	15.6164583 ± 0.0173703	0.9644	EXP 149 of 150	0.1138340 ± 0.0160177	0.0095	EXP 150 of 150	5.2901282 ± 0.0155960	0.7658	EXP 150 of 150	80.912385 ± 0.021236	0.9881	EXP 150 of 150
17D20199	19.3 %	0.2129704 ± 0.0007043	0.6401	EXP 150 of 150	10.9747066 ± 0.0197725	0.9140	EXP 150 of 150	0.0547083 ± 0.0160022	0.0001	EXP 150 of 150	3.3666267 ± 0.0160688	0.5068	EXP 150 of 150	50.751139 ± 0.020984	0.9956	EXP 150 of 150
17D20201	21.0 %	0.0939932 ± 0.0004812	0.0335	EXP 150 of 150	3.8788777 ± 0.0177812	0.5901	EXP 150 of 150	0.0089597 ± 0.0158911	0.0053	EXP 149 of 150	1.3670238 ± 0.0152932	0.0171	EXP 150 of 150	23.264120 ± 0.017221	0.9982	EXP 150 of 150

Project Info		Analyst	Irradiation	X-pos	Y-pos	Z/H-pos	Project	Experiment	Nmb
17D20166	1.8 %	Dan Miggins	17-OSU-01	0.00	0.00	30.03	Arctic\O-Connor (16-22)	17D20162	01
17D20168	1.9 %	Dan Miggins	17-OSU-01	0.00	0.00	30.03	Arctic\O-Connor (16-22)	17D20162	01
17D20169	2.0 %	Dan Miggins	17-OSU-01	0.00	0.00	30.03	Arctic\O-Connor (16-22)	17D20162	01
17D20171	2.2 %	Dan Miggins	17-OSU-01	0.00	0.00	30.03	Arctic\O-Connor (16-22)	17D20162	01
17D20172	2.4 %	Dan Miggins	17-OSU-01	0.00	0.00	30.03	Arctic\O-Connor (16-22)	17D20162	01
17D20174	2.7 %	Dan Miggins	17-OSU-01	0.00	0.00	30.03	Arctic\O-Connor (16-22)	17D20162	01
17D20175	3.0 %	Dan Miggins	17-OSU-01	0.00	0.00	30.03	Arctic\O-Connor (16-22)	17D20162	01
17D20177	3.4 %	Dan Miggins	17-OSU-01	0.00	0.00	30.03	Arctic\O-Connor (16-22)	17D20162	01
17D20178	3.9 %	Dan Miggins	17-OSU-01	0.00	0.00	30.03	Arctic\O-Connor (16-22)	17D20162	01
17D20180	4.5 %	Dan Miggins	17-OSU-01	0.00	0.00	30.03	Arctic\O-Connor (16-22)	17D20162	01
17D20181	5.2 %	Dan Miggins	17-OSU-01	0.00	0.00	30.03	Arctic\O-Connor (16-22)	17D20162	01
17D20183	6.0 %	Dan Miggins	17-OSU-01	0.00	0.00	30.03	Arctic\O-Connor (16-22)	17D20162	01
17D20184	6.9 %	Dan Miggins	17-OSU-01	0.00	0.00	30.03	Arctic\O-Connor (16-22)	17D20162	01
17D20186	7.9 %	Dan Miggins	17-OSU-01	0.00	0.00	30.03	Arctic\O-Connor (16-22)	17D20162	01
17D20187	9.0 %	Dan Miggins	17-OSU-01	0.00	0.00	30.03	Arctic\O-Connor (16-22)	17D20162	01
17D20189	10.3 %	Dan Miggins	17-OSU-01	0.00	0.00	30.03	Arctic\O-Connor (16-22)	17D20162	01
17D20190	11.6 %	Dan Miggins	17-OSU-01	0.00	0.00	30.03	Arctic\O-Connor (16-22)	17D20162	01
17D20192	12.5 %	Dan Miggins	17-OSU-01	0.00	0.00	30.03	Arctic\O-Connor (16-22)	17D20162	01
17D20193	13.4 %	Dan Miggins	17-OSU-01	0.00	0.00	30.03	Arctic\O-Connor (16-22)	17D20162	01
17D20195	14.6 %	Dan Miggins	17-OSU-01	0.00	0.00	30.03	Arctic\O-Connor (16-22)	17D20162	01
17D20196	16.0 %	Dan Miggins	17-OSU-01	0.00	0.00	30.03	Arctic\O-Connor (16-22)	17D20162	01
17D20198	17.6 %	Dan Miggins	17-OSU-01	0.00	0.00	30.03	Arctic\O-Connor (16-22)	17D20162	01
17D20199	19.3 %	Dan Miggins	17-OSU-01	0.00	0.00	30.03	Arctic\O-Connor (16-22)	17D20162	01
17D20201	21.0 %	Dan Miggins	17-OSU-01	0.00	0.00	30.03	Arctic\O-Connor (16-22)	17D20162	01

**Sample Parameters**		Sample	Material	Location	Standard Name	Standard (in Ma)	%1σ	Standard Reference	Standard 40Ar/39Ar	%1σ	J	%1σ	Air 40Ar/36Ar	%1σ	MDF (lin)	%1σ	Volume Ratio	Sensitivity (mol/volt)	Day	Month	Year	Hour	Min	Resist
17D20166	1.8 %	PS59-226-23	Groundmass	Gakkel Ridge	FCT-NM (1F23-17)	28.201	0.082	Kuiper et al (2008)	9.64936	0.095	0.00162885	0.095	302.449	0.152	0.9942486	0.069	1	4.8E-14	12	JUN	2017	22	24	1
17D20168	1.9 %	PS59-226-23	Groundmass	Gakkel Ridge	FCT-NM (1F23-17)	28.201	0.082	Kuiper et al (2008)	9.64936	0.095	0.00162885	0.095	302.449	0.152	0.9942486	0.069	1	4.8E-14	12	JUN	2017	22	44	1
17D20169	2.0 %	PS59-226-23	Groundmass	Gakkel Ridge	FCT-NM (1F23-17)	28.201	0.082	Kuiper et al (2008)	9.64936	0.095	0.00162885	0.095	302.449	0.152	0.9942486	0.069	1	4.8E-14	12	JUN	2017	22	54	1
17D20171	2.2 %	PS59-226-23	Groundmass	Gakkel Ridge	FCT-NM (1F23-17)	28.201	0.082	Kuiper et al (2008)	9.64936	0.095	0.00162885	0.095	302.449	0.152	0.9942486	0.069	1	4.8E-14	12	JUN	2017	23	14	1
17D20172	2.4 %	PS59-226-23	Groundmass	Gakkel Ridge	FCT-NM (1F23-17)	28.201	0.082	Kuiper et al (2008)	9.64936	0.095	0.00162885	0.095	302.449	0.152	0.9942486	0.069	1	4.8E-14	12	JUN	2017	23	24	1
17D20174	2.7 %	PS59-226-23	Groundmass	Gakkel Ridge	FCT-NM (1F23-17)	28.201	0.082	Kuiper et al (2008)	9.64936	0.095	0.00162885	0.095	302.449	0.152	0.9942486	0.069	1	4.8E-14	12	JUN	2017	23	44	1
17D20175	3.0 %	PS59-226-23	Groundmass	Gakkel Ridge	FCT-NM (1F23-17)	28.201	0.082	Kuiper et al (2008)	9.64936	0.095	0.00162885	0.095	302.449	0.152	0.9942486	0.069	1	4.8E-14	12	JUN	2017	23	54	1
17D20177	3.4 %	PS59-226-23	Groundmass	Gakkel Ridge	FCT-NM (1F23-17)	28.201	0.082	Kuiper et al (2008)	9.64936	0.095	0.00162885	0.095	302.449	0.152	0.9942486	0.069	1	4.8E-14	13	JUN	2017	0	14	1
17D20178	3.9 %	PS59-226-23	Groundmass	Gakkel Ridge	FCT-NM (1F23-17)	28.201	0.082	Kuiper et al (2008)	9.64936	0.095	0.00162885	0.095	302.449	0.152	0.9942486	0.069	1	4.8E-14	13	JUN	2017	0	24	1
17D20180	4.5 %	PS59-226-23	Groundmass	Gakkel Ridge	FCT-NM (1F23-17)	28.201	0.082	Kuiper et al (2008)	9.64936	0.095	0.00162885	0.095	302.449	0.152	0.9942486	0.069	1	4.8E-14	13	JUN	2017	0	44	1
17D20181	5.2 %	PS59-226-23	Groundmass	Gakkel Ridge	FCT-NM (1F23-17)	28.201	0.082	Kuiper et al (2008)	9.64936	0.095	0.00162885	0.095	302.449	0.152	0.9942486	0.069	1	4.8E-14	13	JUN	2017	0	54	1
17D20183	6.0 %	PS59-226-23	Groundmass	Gakkel Ridge	FCT-NM (1F23-17)	28.201	0.082	Kuiper et al (2008)	9.64936	0.095	0.00162885	0.095	302.449	0.152	0.9942486	0.069	1	4.8E-14	13	JUN	2017	1	14	1
17D20184	6.9 %	PS59-226-23	Groundmass	Gakkel Ridge	FCT-NM (1F23-17)	28.201	0.082	Kuiper et al (2008)	9.64936	0.095	0.00162885	0.095	302.449	0.152	0.9942486	0.069	1	4.8E-14	13	JUN	2017	1	24	1
17D20186	7.9 %	PS59-226-23	Groundmass	Gakkel Ridge	FCT-NM (1F23-17)	28.201	0.082	Kuiper et al (2008)	9.64936	0.095	0.00162885	0.095	302.449	0.152	0.9942486	0.069	1	4.8E-14	13	JUN	2017	1	44	1
17D20187	9.0 %	PS59-226-23	Groundmass	Gakkel Ridge	FCT-NM (1F23-17)	28.201	0.082	Kuiper et al (2008)	9.64936	0.095	0.00162885	0.095	302.449	0.152	0.9942486	0.069	1	4.8E-14	13	JUN	2017	1	54	1
17D20189	10.3 %	PS59-226-23	Groundmass	Gakkel Ridge	FCT-NM (1F23-17)	28.201	0.082	Kuiper et al (2008)	9.64936	0.095	0.00162885	0.095	302.449	0.152	0.9942486	0.069	1	4.8E-14	13	JUN	2017	2	15	1
17D20190	11.6 %	PS59-226-23	Groundmass	Gakkel Ridge	FCT-NM (1F23-17)	28.201	0.082	Kuiper et al (2008)	9.64936	0.095	0.00162885	0.095	302.449	0.152	0.9942486	0.069	1	4.8E-14	13	JUN	2017	2	25	1
17D20192	12.5 %	PS59-226-23	Groundmass	Gakkel Ridge	FCT-NM (1F23-17)	28.201	0.082	Kuiper et al (2008)	9.64936	0.095	0.00162885	0.095	302.449	0.152	0.9942486	0.069	1	4.8E-14	13	JUN	2017	2	45	1
17D20193	13.4 %	PS59-226-23	Groundmass	Gakkel Ridge	FCT-NM (1F23-17)	28.201	0.082	Kuiper et al (2008)	9.64936	0.095	0.00162885	0.095	302.449	0.152	0.9942486	0.069	1	4.8E-14	13	JUN	2017	2	55	1
17D20195	14.6 %	PS59-226-23	Groundmass	Gakkel Ridge	FCT-NM (1F23-17)	28.201	0.082	Kuiper et al (2008)	9.64936	0.095	0.00162885	0.095	302.449	0.152	0.9942486	0.069	1	4.8E-14	13	JUN	2017	3	15	1
17D20196	16.0 %	PS59-226-23	Groundmass	Gakkel Ridge	FCT-NM (1F23-17)	28.201	0.082	Kuiper et al (2008)	9.64936	0.095	0.00162885	0.095	302.449	0.152	0.9942486	0.069	1	4.8E-14	13	JUN	2017	3	25	1
17D20198	17.6 %	PS59-226-23	Groundmass	Gakkel Ridge	FCT-NM (1F23-17)	28.201	0.082	Kuiper et al (2008)	9.64936	0.095	0.00162885	0.095	302.449	0.152	0.9942486	0.069	1	4.8E-14	13	JUN	2017	3	45	1
17D20199	19.3 %	PS59-226-23	Groundmass	Gakkel Ridge	FCT-NM (1F23-17)	28.201	0.082	Kuiper et al (2008)	9.64936	0.095	0.00162885	0.095	302.449	0.152	0.9942486	0.069	1	4.8E-14	13	JUN	2017	3	55	1
17D20201	21.0 %	PS59-226-23	Groundmass	Gakkel Ridge	FCT-NM (1F23-17)	28.201	0.082	Kuiper et al (2008)	9.64936	0.095	0.00162885	0.095	302.449	0.152	0.9942486	0.069	1	4.8E-14	13	JUN	2017	4	15	1

Irradiation Constants		40/36(a)	%1σ	40/36(c)	%1σ	38/36(a)	%1σ	38/36(c)	%1σ	39/37(ca)	%1σ	38/37(ca)	%1σ	36/37(ca)	%1σ	40/39(k)	%1σ	38/39(k)	%1σ	36/38(cl)	%1σ	K/Ca	%1σ	K/Cl	%1σ	Ca/Cl	%1σ
17D20166	1.8 %	295.5	0	0.018	35	0.1869	0	1.493	3	0.000643	0.92	0.00018	9.63	0.00027	0.17	0.000607	9.65	0.012077	0.09	0	0	0.43	0	0	0	0	0
17D20168	1.9 %	295.5	0	0.018	35	0.1869	0	1.493	3	0.000643	0.92	0.00018	9.63	0.00027	0.17	0.000607	9.65	0.012077	0.09	0	0	0.43	0	0	0	0	0
17D20169	2.0 %	295.5	0	0.018	35	0.1869	0	1.493	3	0.000643	0.92	0.00018	9.63	0.00027	0.17	0.000607	9.65	0.012077	0.09	0	0	0.43	0	0	0	0	0
17D20171	2.2 %	295.5	0	0.018	35	0.1869	0	1.493	3	0.000643	0.92	0.00018	9.63	0.00027	0.17	0.000607	9.65	0.012077	0.09	0	0	0.43	0	0	0	0	0
17D20172	2.4 %	295.5	0	0.018	35	0.1869	0	1.493	3	0.000643	0.92	0.00018	9.63	0.00027	0.17	0.000607	9.65	0.012077	0.09	0	0	0.43	0	0	0	0	0
17D20174	2.7 %	295.5	0	0.018	35	0.1869	0	1.493	3	0.000643	0.92	0.00018	9.63	0.00027	0.17	0.000607	9.65	0.012077	0.09	0	0	0.43	0	0	0	0	0
17D20175	3.0 %	295.5	0	0.018	35	0.1869	0	1.493	3	0.000643	0.92	0.00018	9.63	0.00027	0.17	0.000607	9.65	0.012077	0.09	0	0	0.43	0	0	0	0	0
17D20177	3.4 %	295.5	0	0.018	35	0.1869	0	1.493	3	0.000643	0.92	0.00018	9.63	0.00027	0.17	0.000607	9.65	0.012077	0.09	0	0	0.43	0	0	0	0	0
17D20178	3.9 %	295.5	0	0.018	35	0.1869	0	1.493	3	0.000643	0.92	0.00018	9.63	0.00027	0.17	0.000607	9.65	0.012077	0.09	0	0	0.43	0	0	0	0	0
17D20180	4.5 %	295.5	0	0.018	35	0.1869	0	1.493	3	0.000643	0.92	0.00018	9.63	0.00027	0.17	0.000607	9.65	0.012077	0.09	0	0	0.43	0	0	0	0	0
17D20181	5.2 %	295.5	0	0.018	35	0.1869	0	1.493	3	0.000643	0.92	0.00018	9.63	0.00027	0.17	0.000607	9.65	0.012077	0.09	0	0	0.43	0	0	0	0	0
17D20183	6.0 %	295.5	0	0.018	35	0.1869	0	1.493	3	0.000643	0.92	0.00018	9.63	0.00027	0.17	0.000607	9.65	0.012077	0.09	0	0	0.43	0	0	0	0	0
17D20184	6.9 %	295.5	0	0.018	35	0.1869	0	1.493	3	0.000643	0.92	0.00018	9.63	0.00027	0.17	0.000607	9.65	0.012077	0.09	0	0	0.43	0	0	0	0	0
17D20186	7.9 %	295.5	0	0.018	35	0.1869	0	1.493	3	0.000643	0.92	0.00018	9.63	0.00027	0.17	0.000607	9.65	0.012077	0.09	0	0	0.43	0	0	0	0	0
17D20187	9.0 %	295.5	0	0.018	35	0.1869	0	1.493	3	0.000643	0.92	0.00018	9.63	0.00027	0.17	0.000607	9.65	0.012077	0.09	0	0	0.43	0	0	0	0	0
17D20189	10.3 %	295.5	0	0.018	35	0.1869	0	1.493	3	0.000643	0.92	0.00018	9.63	0.00027	0.17	0.000607	9.65	0.012077	0.09	0	0	0.43	0	0	0	0	0
17D20190	11.6 %	295.5	0	0.018	35	0.1869	0	1.493	3	0.000643	0.92	0.00018	9.63	0.00027	0.17	0.000607	9.65	0.012077	0.09	0	0	0.43	0	0	0	0	0
17D20192	12.5 %	295.5	0	0.018	35	0.1869	0	1.493	3	0.000643	0.92	0.00018	9.63	0.00027	0.17	0.000607	9.65	0.012077	0.09	0	0	0.43	0	0	0	0	0
17D20193	13.4 %	295.5	0	0.018	35	0.1869	0	1.493	3	0.000643	0.92	0.00018	9.63	0.00027	0.17	0.000607	9.65	0.012077	0.09	0	0	0.43	0	0	0	0	0
17D20195	14.6 %	295.5	0	0.018	35	0.1869	0	1.493	3	0.000643	0.92	0.00018	9.63	0.00027	0.17	0.000607	9.65	0.012077	0.09	0	0	0.43	0	0	0	0	0
17D20196	16.0 %	295.5	0	0.018	35	0.1869	0	1.493	3	0.000643	0.92	0.00018	9.63	0.00027	0.17	0.000607	9.65	0.012077	0.09	0	0	0.43	0	0	0	0	0
17D20198	17.6 %	295.5	0	0.018	35	0.1869	0	1.493	3	0.000643	0.92	0.00018	9.63	0.00027	0.17	0.000607	9.65	0.012077	0.09	0	0	0.43	0	0	0	0	0
17D20199	19.3 %	295.5	0	0.018	35	0.1869	0	1.493	3	0.000643	0.92	0.00018	9.63	0.00027	0.17	0.000607	9.65	0.012077	0.09	0	0	0.43	0	0	0	0	0
17D20201	21.0 %	295.5	0	0.018	35	0.1869	0	1.493	3	0.000643	0.92	0.00018	9.63	0.00027	0.17	0.000607	9.65	0.012077	0.09	0	0	0.43	0	0	0	0	0

17D20162.AGE >>> PS59-226-23 >>> ARCTIC | O-CONNOR (16-22) PROJECT

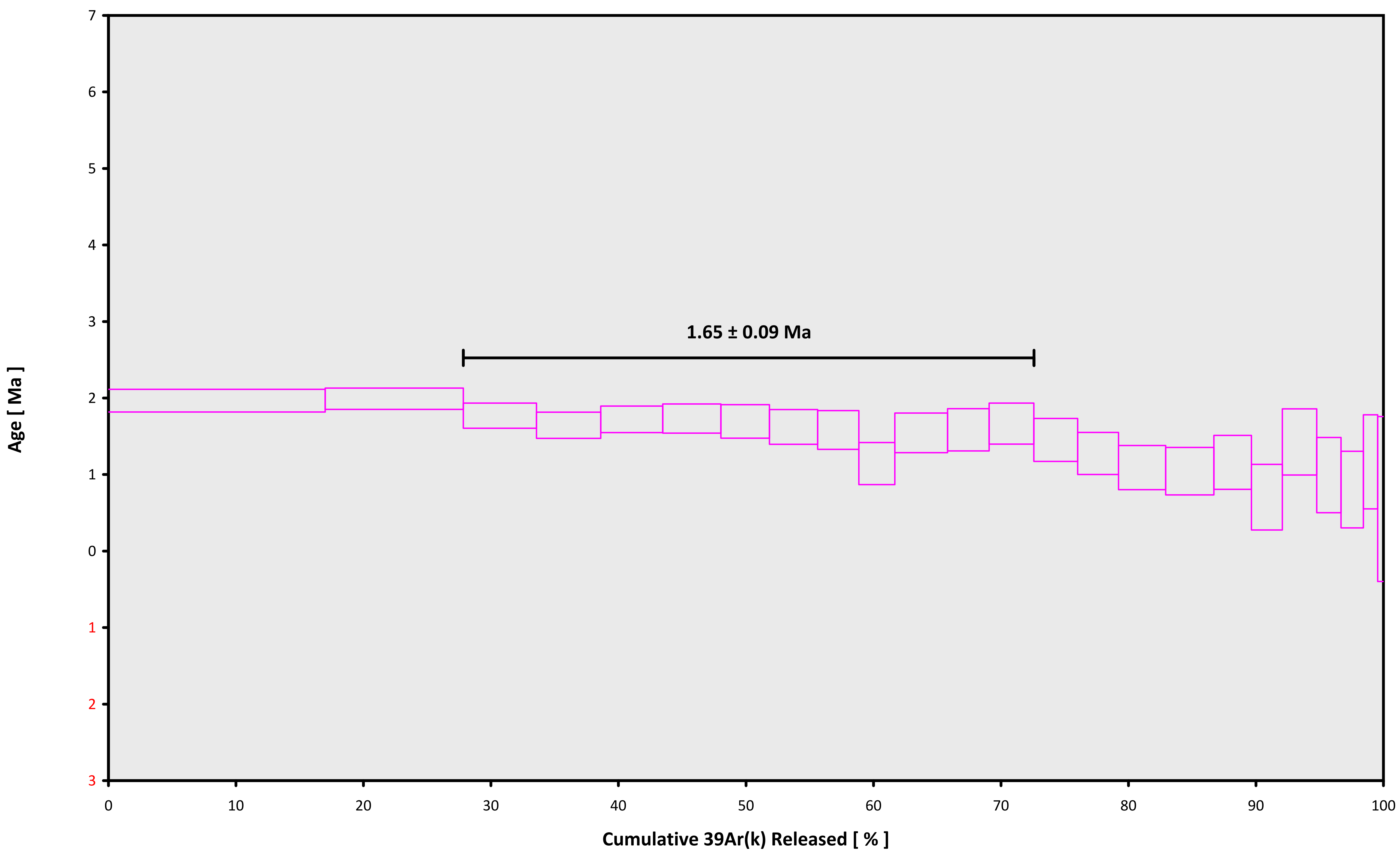

Ar-Ages in Ma

WEIGHTED PLATEAU
1.65 ± 0.09
TOTAL FUSION
1.59 ± 0.05
NORMAL ISOCHRON
2.45 ± 0.63
INVERSE ISOCHRON
2.44 ± 0.58

MSWD (PROBABILITY)
1.85 (5%)

Sample Info

Groundmass
Gakkel Ridge
Dan Miggins

IRR = 17-OSU-01 (1F23-17)
J = 0.00162885 ± 0.00000155

17D20162.AGE >>> PS59-226-23 >>> ARCTIC | O-CONNOR (16-22) PROJECT

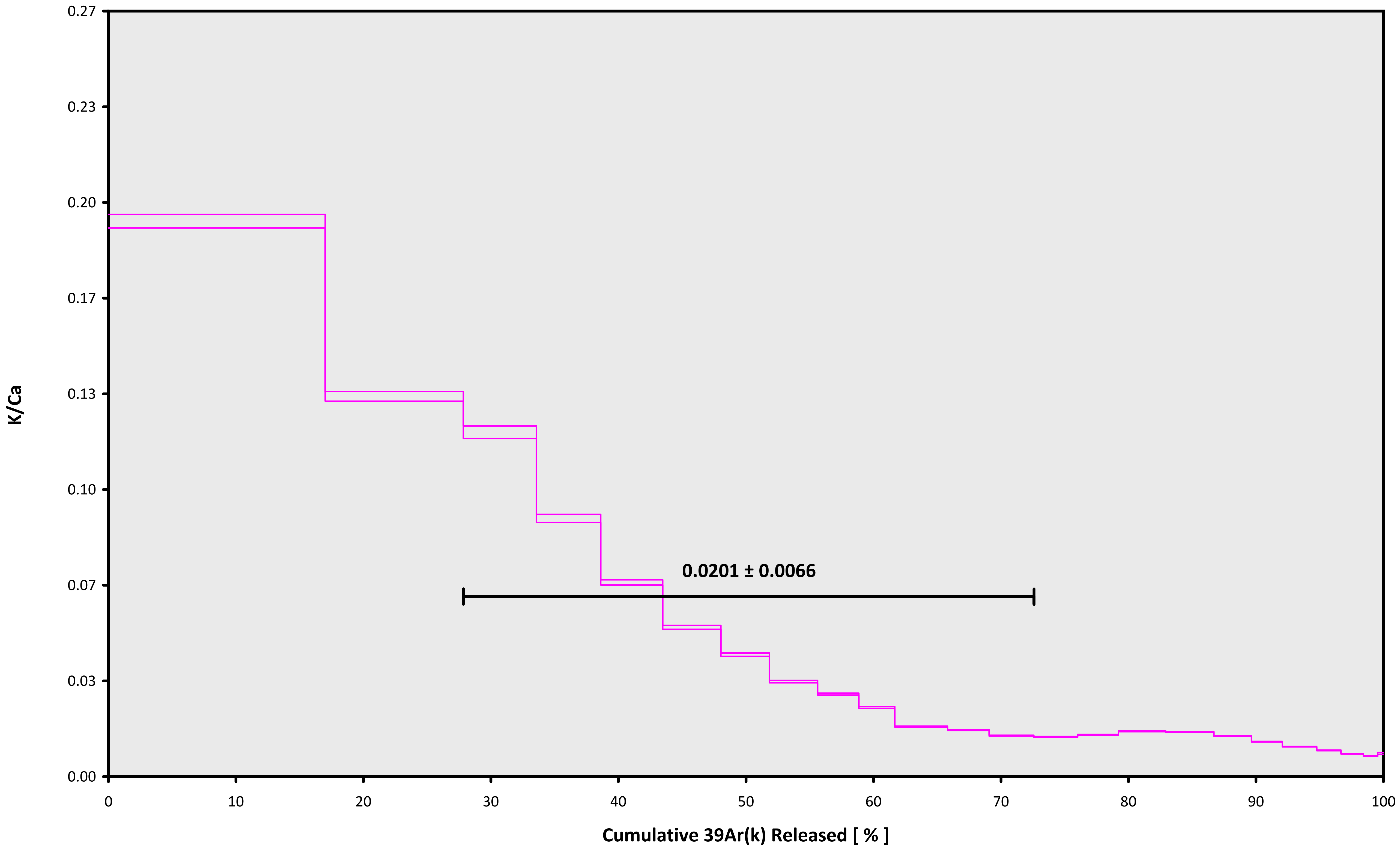

Ar-Ages in Ma

WEIGHTED PLATEAU
1.65 ± 0.09
TOTAL FUSION
1.59 ± 0.05
NORMAL ISOCHRON
2.45 ± 0.63
INVERSE ISOCHRON
2.44 ± 0.58

Sample Info

Groundmass
Gakkel Ridge
Dan Miggins

IRR = 17-OSU-01 (1F23-17)
J = 0.00162885 ± 0.00000155

17D20162.AGE >>> PS59-226-23 >>> ARCTIC | O-CONNOR (16-22) PROJECT

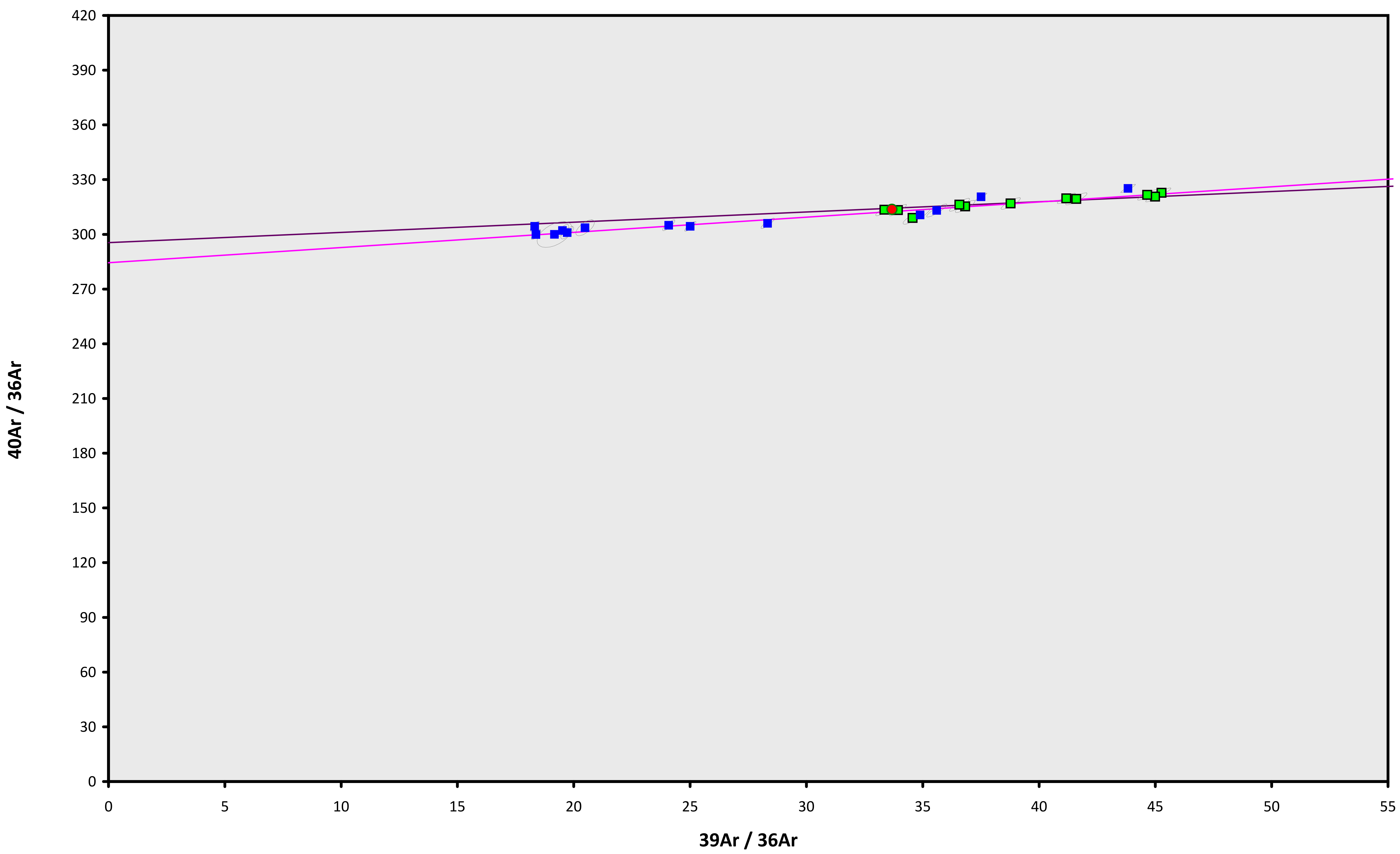

Ar-Ages in Ma

WEIGHTED PLATEAU
1.65 ± 0.09
TOTAL FUSION
1.59 ± 0.05
NORMAL ISOCHRON
2.45 ± 0.63
INVERSE ISOCHRON
2.44 ± 0.58

MSWD (PROBABILITY)
1.25 (26%)

40AR/36AR INTERCEPT
284.4 ± 8.6

Sample Info

Groundmass
Gakkel Ridge
Dan Miggins

IRR = 17-OSU-01 (1F23-17)
J = 0.00162885 ± 0.00000155

17D20162.AGE >>> PS59-226-23 >>> ARCTIC | O-CONNOR (16-22) PROJECT

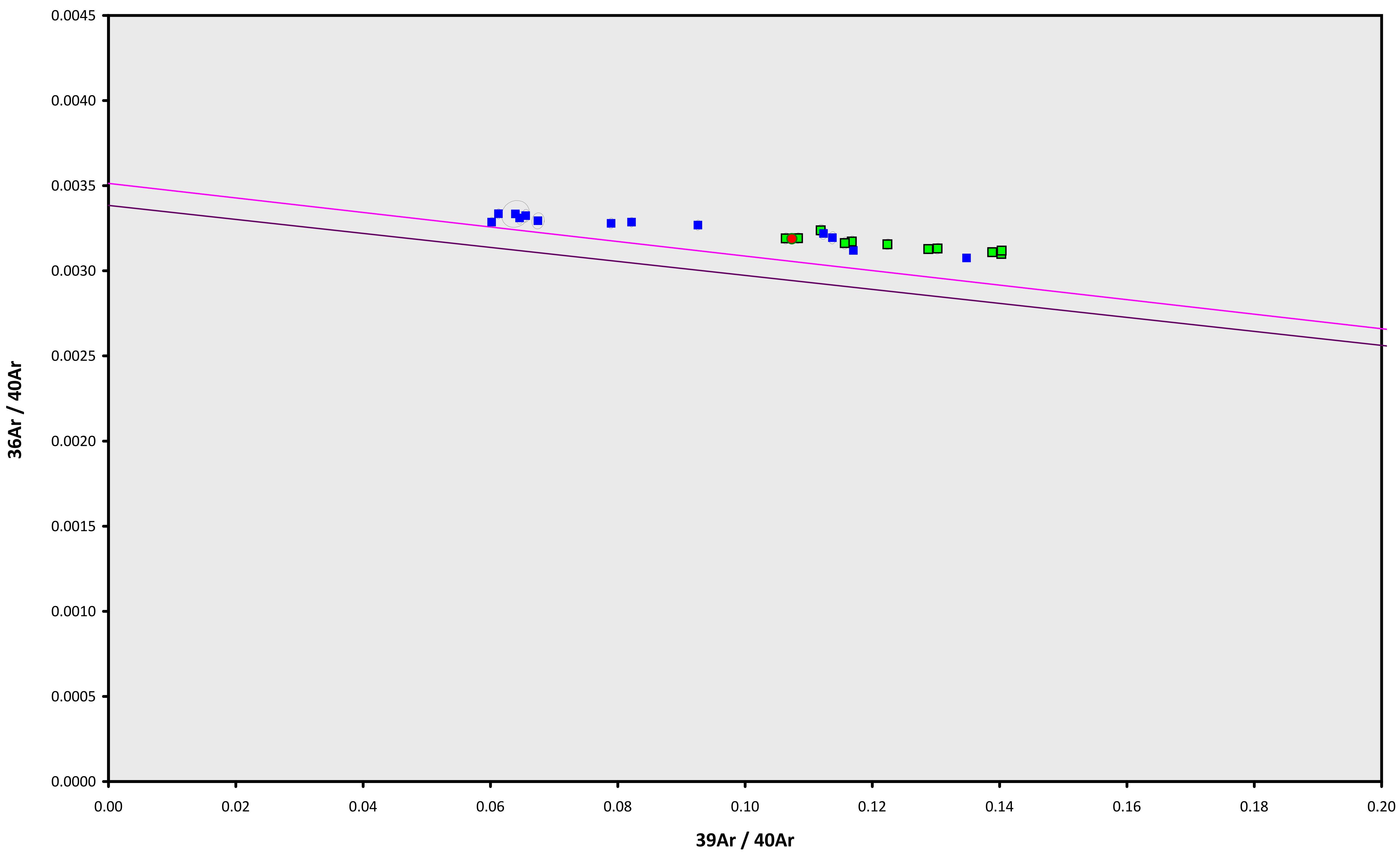

Ar-Ages in Ma

WEIGHTED PLATEAU
1.65 ± 0.09
TOTAL FUSION
1.59 ± 0.05
NORMAL ISOCHRON
2.45 ± 0.63
INVERSE ISOCHRON
2.44 ± 0.58

MSWD (PROBABILITY)
1.22 (28%)

SPREADING FACTOR
2.8%

40AR/36AR INTERCEPT
284.6 ± 8.5

Sample Info

Groundmass
Gakkel Ridge
Dan Miggins

IRR = 17-OSU-01 (1F23-17)
J = 0.00162885 ± 0.00000155

Relative Abundances

Relative Abundances			36Ar [fA]	%1σ	37Ar [fA]	%1σ	38Ar [fA]	%1σ	39Ar [fA]	%1σ	40Ar [fA]	%1σ	40(r)/39(k) ± 2σ	Age ± 2σ (ka)	40Ar(r) (%)	39Ar(k) (%)	K/Ca ± 2σ
17D30245	1.8 %	✓	0.714439	0.399	96.3804	0.532	1.0395620	2.302	70.60671	0.071	224.5608	0.215	0.29913 ± 0.02758	888.5 ± 81.9	9.40	18.18	0.3147 ± 0.0034
17D30247	1.9 %	✓	0.279239	0.747	40.6000	0.897	0.4109690	5.959	27.93576	0.102	86.6208	0.531	0.26273 ± 0.05518	780.4 ± 163.9	8.47	7.19	0.2956 ± 0.0053
17D30248	2.0 %	✓	0.437293	0.541	78.4665	0.606	0.5473268	4.284	36.11953	0.087	133.5788	0.348	0.29403 ± 0.04660	873.4 ± 138.4	7.94	9.29	0.1977 ± 0.0024
17D30250	2.2 %	✓	0.158711	1.213	26.2732	1.307	0.2167510	11.341	13.48839	0.183	47.2893	0.967	0.18423 ± 0.10844	547.3 ± 322.1	5.25	3.47	0.2205 ± 0.0058
17D30251	2.4 %	✓	0.141401	1.343	28.4674	1.249	0.2156087	10.748	12.66479	0.183	42.4108	1.078	0.22891 ± 0.11459	680.0 ± 340.3	6.83	3.26	0.1910 ± 0.0048
17D30253	2.7 %	✓	0.309551	0.671	81.9513	0.567	0.3488646	6.909	23.00133	0.113	90.6557	0.506	0.24906 ± 0.06689	739.8 ± 198.7	6.30	5.91	0.1204 ± 0.0014
17D30254	3.0 %	✓	0.514631	0.479	142.3699	0.460	0.5034518	4.979	27.61954	0.102	148.7690	0.311	0.29256 ± 0.06286	869.0 ± 186.7	5.41	7.09	0.0831 ± 0.0008
17D30256	3.4 %	✓	0.424895	0.543	137.5598	0.465	0.3283672	7.509	19.07184	0.128	117.5385	0.391	0.15582 ± 0.08677	462.9 ± 257.7	2.52	4.89	0.0593 ± 0.0006
17D30257	3.9 %	✓	0.376699	0.589	138.0593	0.473	0.2903129	8.643	15.04248	0.156	104.8392	0.437	0.30388 ± 0.10726	902.6 ± 318.5	4.33	3.85	0.0466 ± 0.0005
17D30259	4.5 %	✓	0.153238	1.238	66.4132	0.642	0.1253805	19.201	7.79785	0.272	41.4572	1.103	0.19023 ± 0.18678	565.1 ± 554.7	3.56	2.00	0.0502 ± 0.0007
17D30260	5.2 %	✓	0.892475	0.354	491.3504	0.400	0.5498554	4.311	26.67840	0.104	229.9723	0.200	0.20771 ± 0.08005	617.0 ± 237.7	2.38	6.79	0.0231 ± 0.0002
17D30262	6.0 %	✓	0.598711	0.428	381.0522	0.403	0.3386867	7.340	16.37401	0.154	151.2094	0.304	0.29242 ± 0.11110	868.6 ± 329.9	3.12	4.16	0.0182 ± 0.0002
17D30263	6.9 %	✓	0.145925	1.291	100.6688	0.524	0.1171746	21.498	4.54790	0.467	35.7364	1.279	0.14606 ± 0.32204	433.9 ± 956.6	1.83	1.16	0.0191 ± 0.0003
17D30265	7.9 %	✓	0.409058	0.361	296.9079	0.406	0.2543222	9.136	10.85906	0.222	99.6286	0.041	0.23070 ± 0.08454	685.3 ± 251.1	2.47	2.75	0.0155 ± 0.0001
17D30266	9.0 %	✓	0.424780	0.346	262.7818	0.411	0.2266263	10.998	8.86674	0.266	106.8521	0.041	0.26606 ± 0.10264	790.3 ± 304.8	2.17	2.24	0.0142 ± 0.0001
17D30268	10.3 %	✓	0.272658	0.389	169.5487	0.435	0.1311984	18.249	5.65765	0.388	68.3674	0.047	0.24078 ± 0.11583	715.2 ± 344.0	1.95	1.43	0.0141 ± 0.0002
17D30269	11.6 %	✓	0.662336	0.310	245.7710	0.414	0.2419021	10.071	9.05668	0.263	177.1143	0.019	0.11453 ± 0.13818	340.2 ± 410.5	0.58	2.29	0.0156 ± 0.0002
17D30271	12.5 %		1.526974	0.276	323.6471	0.405	0.4682139	4.979	12.62721	0.194	430.0746	0.009	0.37822 ± 0.20155	1123.3 ± 598.5	1.09	3.20	0.0165 ± 0.0001
17D30272	13.4 %		1.719517	0.266	339.3562	0.403	0.5259979	4.502	11.78847	0.209	485.7757	0.009	0.41124 ± 0.23457	1221.4 ± 696.4	0.98	2.98	0.0147 ± 0.0001
17D30274	14.6 %		1.637425	0.271	529.2090	0.397	0.5323861	4.169	13.26421	0.185	446.1243	0.009	0.35030 ± 0.20511	1040.5 ± 609.0	1.01	3.33	0.0105 ± 0.0001
17D30275	16.0 %		0.981361	0.291	521.0780	0.396	0.3535396	6.250	8.90607	0.273	252.2011	0.014	0.44615 ± 0.20123	1325.1 ± 597.4	1.52	2.21	0.0071 ± 0.0001
17D30277	17.6 %	✓	0.417492	0.248	212.2108	0.380	0.1474994	15.999	3.91831	0.561	106.5870	0.029	0.04379 ± 0.16647	130.1 ± 494.5	0.16	0.98	0.0077 ± 0.0001
17D30278	19.3 %	✓	0.393670	0.264	289.4555	0.367	0.1317194	16.977	3.66789	0.624	93.9420	0.034	0.20952 ± 0.18523	622.4 ± 550.1	0.78	0.90	0.0052 ± 0.0001
17D30280	21.0 %	✓	0.262650	0.354	112.1920	0.456	0.0915577	25.866	1.83998	1.107	69.0108	0.040	0.20244 ± 0.31594	601.3 ± 938.4	0.52	0.46	0.0068 ± 0.0002
		Σ	13.855131	0.088	5111.7705	0.103	8.1372742	1.440	391.40082	0.033	3790.3161	0.044					

Information on Analysis and Constants Used in Calculations

Project = **O-CONNOR (16-22)**
Sample = **PS59-231-20**
Material = **Groundmass**
Location = **Gakkel Ridge**
Region = **Artic Ocean**
Analyst = **Dan Miggins**
Irradiation = **17-OSU-05 (5B3-17)**
Position = **X: 0 | Y: 0 | Z/H: 4.863966 mm**
FCT-NM Age = **28.201 ± 0.023 Ma**
FCT-NM Reference = **Kuiper et al (2008)**
FCT-NM 40Ar/39Ar Ratio = **9.56637 ± 0.00794**
FCT-NM J-value = **0.00164298 ± 0.00000136**
Air Shot 40Ar/36Ar = **302.7710 ± 0.1605**
Air Shot MDF = **0.99398853 ± 0.00059332 (LIN)**
Experiment Type = **Incremental Heating**
Extraction Method = **Bulk Laser Heating**
Heating = **64 sec**
Isolation = **3.00 min**
Instrument = **ARGUS-VI-D**
Preferred Age = **Plateau Age**
Age Classification = **Crystallization Age**
IGSN = Undefined
Rock Class = **Igneous>Volcanic**
Lithology = **Basaltic Lava**
Lat-Lon = Undefined - Undefined

Age Equations = **Min et al. (2000)**
Negative Intensities = **Allowed**
Collector Calibrations = **36Ar**
Decay 40K = **5.530 ± 0.048 E-10 1/a**
Decay 39Ar = **2.940 ± 0.016 E-07 1/h**
Decay 37Ar = **8.230 ± 0.012 E-04 1/h**
Decay 36Cl = **2.257 ± 0.015 E-06 1/a**
Decay 40K(EC,β^+) = **0.580 ± 0.009 E-10 1/a**
Decay 40K(β^-) = **4.950 ± 0.043 E-10 1/a**
Atmospheric 40/36(a) = **295.50**
Atmospheric 38/36(a) = **0.1869**
Production 39/37(ca) = **0.0006425 ± 0.0000059**
Production 38/37(ca) = **0.0001800 ± 0.0000173**
Production 36/37(ca) = **0.0002703 ± 0.0000005**
Production 40/39(k) = **0.000607 ± 0.000059**
Production 38/39(k) = **0.012077 ± 0.000011**
Production 36/38(cl) = **262.80 ± 1.71**
Scaling Ratio K/Ca = **0.430**
Abundance Ratio 40K/K = **1.1700 ± 0.0100 E-04**
Atomic Weight K = **39.0983 ± 0.0001 g**

Results

Results	40(a)/36(a) ± 2σ	40(r)/39(k) ± 2σ	Age ± 2σ (ka)	MSWD	39Ar(k) (%,n)	K/Ca ± 2σ
**Age Plateau**		0.26651 ± 0.02231	**791.6 ± 66.3**	1.83	88.28	0.0122 ± 0.0050
Error Mean		± 8.37%	**± 8.37%**	1%	20	
		Full External Error	± 68.6	1.65	2σ Confidence Limit	
		Analytical Error	± 66.3	1.3526	Error Magnification	
**Total Fusion Age**		0.26848 ± 0.02061	**797.5 ± 61.2**		24	0.0326 ± 0.0001
		± 7.68%	**± 7.68%**			
		Full External Error	± 63.8			
		Analytical Error	± 61.2			
**Normal Isochron**	293.59 ± 1.14	0.29960 ± 0.02710	**889.9 ± 80.5**	1.21	88.28	
	± 0.39%	± 9.05%	**± 9.05%**	24%	20	
		Full External Error	± 83.0	1.67	2σ Confidence Limit	
		Analytical Error	± 80.5	1.0995	Error Magnification	
				5	Number of Iterations	
				0.0000003623	Convergence	
**Inverse Isochron**	293.59 ± 1.13	0.30039 ± 0.02634	**892.3 ± 78.2**	1.19	88.28	
	± 0.39%	± 8.77%	**± 8.77%**	26%	20	
		Full External Error	± 80.8	1.67	2σ Confidence Limit	
		Analytical Error	± 78.2	1.0894	Error Magnification	
				3	Number of Iterations	
				0.0052301509	Convergence	
				9%	Spreading Factor	

Notes
bumpy sepctra

Incremental Heating			36Ar(a) [fA]	37Ar(ca) [fA]	38Ar(cl) [fA]	39Ar(k) [fA]	40Ar(r) [fA]	Age ± 2σ (ka)	40Ar(r) (%)	39Ar(k) (%)	K/Ca ± 2σ
17D30245	1.8 %	✓	0.688379	96.3804	0.0415861	70.54478	21.10193	888.5 ± 81.9	9.40	18.18	0.3147 ± 0.0034
17D30247	1.9 %	✓	0.268261	40.6000	0.0164577	27.90968	7.33259	780.4 ± 163.9	8.47	7.19	0.2956 ± 0.0053
17D30248	2.0 %	✓	0.416079	78.4665	0.0198308	36.06912	10.60543	873.4 ± 138.4	7.94	9.29	0.1977 ± 0.0024
17D30250	2.2 %	✓	0.151605	26.2732	0.0209914	13.47151	2.48180	547.3 ± 322.1	5.25	3.47	0.2205 ± 0.0058
17D30251	2.4 %	✓	0.133700	28.4674	0.0327644	12.64650	2.89492	680.0 ± 340.3	6.83	3.26	0.1910 ± 0.0048
17D30253	2.7 %	✓	0.287399	81.9513	0.0032474	22.94868	5.71553	739.8 ± 198.7	6.30	5.91	0.1204 ± 0.0014
17D30254	3.0 %	✓	0.476137	142.3699	0.0563787	27.52807	8.05366	869.0 ± 186.7	5.41	7.09	0.0831 ± 0.0008
17D30256	3.4 %	✓	0.387712	137.5598	0.0018799	18.98346	2.95805	462.9 ± 257.7	2.52	4.89	0.0593 ± 0.0006
17D30257	3.9 %	✓	0.339377	138.0593	0.0214360	14.95377	4.54423	902.6 ± 318.5	4.33	3.85	0.0466 ± 0.0005
17D30259	4.5 %	✓	0.135287	66.4132	0.0000000	7.75518	1.47527	565.1 ± 554.7	3.56	2.00	0.0502 ± 0.0007
17D30260	5.2 %	✓	0.759663	491.3504	0.0010489	26.36271	5.47585	617.0 ± 237.7	2.38	6.79	0.0231 ± 0.0002
17D30262	6.0 %	✓	0.495713	381.0522	0.0000000	16.12918	4.71650	868.6 ± 329.9	3.12	4.16	0.0182 ± 0.0002
17D30263	6.9 %	✓	0.118710	100.6688	0.0227234	4.48322	0.65480	433.9 ± 956.6	1.83	1.16	0.0191 ± 0.0003
17D30265	7.9 %	✓	0.328802	296.9079	0.0105847	10.66830	2.46112	685.3 ± 251.1	2.47	2.75	0.0155 ± 0.0001
17D30266	9.0 %	✓	0.353749	262.7818	0.0081654	8.69791	2.31414	790.3 ± 304.8	2.17	2.24	0.0142 ± 0.0001
17D30268	10.3 %	✓	0.226829	169.5487	0.0000000	5.54871	1.33599	715.2 ± 344.0	1.95	1.43	0.0141 ± 0.0002
17D30269	11.6 %	✓	0.595904	245.7710	0.0000000	8.89877	1.01915	340.2 ± 410.5	0.58	2.29	0.0156 ± 0.0002
17D30271	12.5 %		1.439492	323.6471	0.0000000	12.41927	4.69717	1123.3 ± 598.5	1.09	3.20	0.0165 ± 0.0001
17D30272	13.4 %		1.627785	339.3562	0.0209445	11.57044	4.75820	1221.4 ± 696.4	0.98	2.98	0.0147 ± 0.0001
17D30274	14.6 %		1.494379	529.2090	0.0017435	12.92420	4.52740	1040.5 ± 609.0	1.01	3.33	0.0105 ± 0.0001
17D30275	16.0 %		0.840514	521.0780	0.0000000	8.57128	3.82410	1325.1 ± 597.4	1.52	2.21	0.0071 ± 0.0001
17D30277	17.6 %	✓	0.360132	212.4047	0.0000000	3.78542	0.16576	130.1 ± 494.5	0.16	0.98	0.0077 ± 0.0001
17D30278	19.3 %	✓	0.315430	289.7200	0.0000000	3.48510	0.73020	622.4 ± 550.1	0.78	0.90	0.0052 ± 0.0001
17D30280	21.0 %	✓	0.232323	112.2945	0.0065970	1.76951	0.35821	601.3 ± 938.4	0.52	0.46	0.0068 ± 0.0002
		Σ	12.473362	5112.3314	0.2863800	388.12475	104.20202				

Information on Analysis

Project = **O-CONNOR (16-22)**
Sample = **PS59-231-20**
Material = **Groundmass**
Location = **Gakkel Ridge**
Region = **Artic Ocean**
Analyst = **Dan Miggins**
Irradiation = **17-OSU-05 (5B3-17)**
J = **0.00164298 ± 0.00000136**
FCT-NM = **28.201 ± 0.023 Ma**

Results	40(r)/39(k) ± 2σ		Age ± 2σ (ka)		MSWD	39Ar(k) (%,n)	K/Ca ± 2σ
**Age Plateau**<br>Error Mean	0.26651	± 0.02231<br>± 8.37%	**791.6**	**± 66.3**<br>**± 8.37%**	1.83<br>1%	88.28<br>20	0.0122 ± 0.0050
			Full External Error	± 68.6	1.65	2σ Confidence Limit	
			Analytical Error	± 66.3	1.3526	Error Magnification	
**Total Fusion Age**	0.26848	± 0.02061<br>± 7.68%	**797.5**	**± 61.2**<br>**± 7.68%**		24	0.0326 ± 0.0001
			Full External Error	± 63.8			
			Analytical Error	± 61.2			

Normal Isochron			39(k)/36(a) ± 2σ	40(a+r)/36(a) ± 2σ	r.i.
17D30245	1.8 %	✓	102.48 ± 0.86	326.15 ± 3.05	0.8748
17D30247	1.9 %	✓	104.04 ± 1.63	322.83 ± 6.09	0.8190
17D30248	2.0 %	✓	86.69 ± 1.00	320.99 ± 4.28	0.8436
17D30250	2.2 %	✓	88.86 ± 2.28	311.87 ± 9.96	0.7878
17D30251	2.4 %	✓	94.59 ± 2.71	317.15 ± 11.32	0.7904
17D30253	2.7 %	✓	79.85 ± 1.17	315.39 ± 5.57	0.8100
17D30254	3.0 %	✓	57.82 ± 0.61	312.41 ± 3.78	0.8418
17D30256	3.4 %	✓	48.96 ± 0.60	303.13 ± 4.32	0.8178
17D30257	3.9 %	✓	44.06 ± 0.59	308.89 ± 4.87	0.8092
17D30259	4.5 %	✓	57.32 ± 1.64	306.40 ± 10.95	0.7722
17D30260	5.2 %	✓	34.70 ± 0.30	302.71 ± 2.83	0.8775
17D30262	6.0 %	✓	32.54 ± 0.36	305.01 ± 3.70	0.8287
17D30263	6.9 %	✓	37.77 ± 1.25	301.02 ± 12.30	0.7471
17D30265	7.9 %	✓	32.45 ± 0.33	302.99 ± 2.81	0.8942
17D30266	9.0 %	✓	24.59 ± 0.25	302.04 ± 2.58	0.8387
17D30268	10.3 %	✓	24.46 ± 0.30	301.39 ± 2.89	0.7653
17D30269	11.6 %	✓	14.93 ± 0.13	297.21 ± 2.08	0.7910
17D30271	12.5 %		8.63 ± 0.06	298.76 ± 1.76	0.8286
17D30272	13.4 %		7.11 ± 0.05	298.42 ± 1.68	0.7961
17D30274	14.6 %		8.65 ± 0.06	298.53 ± 1.79	0.8417
17D30275	16.0 %		10.20 ± 0.09	300.05 ± 2.08	0.7705
17D30277	17.6 %	✓	10.51 ± 0.14	295.96 ± 1.75	0.4492
17D30278	19.3 %	✓	11.05 ± 0.16	297.81 ± 2.06	0.4609
17D30280	21.0 %	✓	7.62 ± 0.19	297.04 ± 2.42	0.3299

Results	40(a)/36(a) ± 2σ		40(r)/39(k) ± 2σ		Age ± 2σ (ka)		MSWD
Normal Isochron	**293.59**	**± 1.14**<br>**± 0.39%**	0.29960	± 0.02710<br>± 9.05%	**889.9**	**± 80.5**<br>**± 9.05%**	1.21<br>24%
					Full External Error	± 83.0	
					Analytical Error	± 80.5	

Statistics				
2σ Confidence Limit	1.67	Convergence	0.000000362267
Error Magnification	1.0995	Number of Iterations	5
Number of Data Points	20	Calculated Line	Weighted York-2

Inverse Isochron			39(k)/40(a+r) ± 2σ	36(a)/40(a+r) ± 2σ	r.i.
17D30245	1.8 %	✓	0.3142055 ± 0.0014251	0.00306603 ± 0.00002866	0.4381
17D30247	1.9 %	✓	0.3222683 ± 0.0034876	0.00309757 ± 0.00005841	0.5536
17D30248	2.0 %	✓	0.2700656 ± 0.0019366	0.00311537 ± 0.00004158	0.5054
17D30250	2.2 %	✓	0.2849235 ± 0.0056082	0.00320646 ± 0.00010244	0.5947
17D30251	2.4 %	✓	0.2982445 ± 0.0065248	0.00315306 ± 0.00011257	0.5955
17D30253	2.7 %	✓	0.2531798 ± 0.0026260	0.00317071 ± 0.00005604	0.5589
17D30254	3.0 %	✓	0.1850598 ± 0.0012120	0.00320088 ± 0.00003877	0.4884
17D30256	3.4 %	✓	0.1615243 ± 0.0013290	0.00329892 ± 0.00004706	0.5203
17D30257	3.9 %	✓	0.1426477 ± 0.0013256	0.00323740 ± 0.00005105	0.5217
17D30259	4.5 %	✓	0.1870862 ± 0.0042513	0.00326366 ± 0.00011659	0.5993
17D30260	5.2 %	✓	0.1146423 ± 0.0005179	0.00330351 ± 0.00003092	0.3766
17D30262	6.0 %	✓	0.1066747 ± 0.0007307	0.00327853 ± 0.00003978	0.4458
17D30263	6.9 %	✓	0.1254622 ± 0.0034240	0.00332208 ± 0.00013573	0.5873
17D30265	7.9 %	✓	0.1070877 ± 0.0004936	0.00330049 ± 0.00003063	0.0159
17D30266	9.0 %	✓	0.0814054 ± 0.0004473	0.00331080 ± 0.00002827	0.0140
17D30268	10.3 %	✓	0.0811642 ± 0.0006481	0.00331796 ± 0.00003180	0.0116
17D30269	11.6 %	✓	0.0502446 ± 0.0002705	0.00336462 ± 0.00002349	0.0040
17D30271	12.5 %		0.0288775 ± 0.0001147	0.00334713 ± 0.00001970	0.0015
17D30272	13.4 %		0.0238188 ± 0.0001021	0.00335095 ± 0.00001891	0.0014
17D30274	14.6 %		0.0289705 ± 0.0001115	0.00334975 ± 0.00002011	0.0015
17D30275	16.0 %		0.0339866 ± 0.0001950	0.00333278 ± 0.00002314	0.0019
17D30277	17.6 %	✓	0.0355156 ± 0.0004142	0.00337883 ± 0.00002001	0.0050
17D30278	19.3 %	✓	0.0370992 ± 0.0004898	0.00335779 ± 0.00002325	0.0051
17D30280	21.0 %	✓	0.0256415 ± 0.0005917	0.00336653 ± 0.00002741	0.0034

Results	40(a)/36(a) ± 2σ		40(r)/39(k) ± 2σ		Age ± 2σ (ka)		MSWD
**Inverse Isochron**	**293.59**	**± 1.13**<br>**± 0.39%**	0.30039	± 0.02634<br>± 8.77%	**892.3**	**± 78.2**<br>**± 8.77%**	1.19<br>26%
					Full External Error	± 80.8	
					Analytical Error	± 78.2	

Statistics				
	2σ Confidence Limit	1.67	Convergence	0.0052301509
	Error Magnification	1.0894	Number of Iterations	3
	Number of Data Points	20	Calculated Line	Weighted York-2
	Spreading Factor	8.9%		

Degassing Patterns			36Ar(a) [fA]	%1σ	36Ar(c) [fA]	%1σ	36Ar(ca) [fA]	%1σ	36Ar(cl) [fA]	%1σ	37Ar(ca) [fA]	%1σ	38Ar(a) [fA]	%1σ	38Ar(c) [fA]	%1σ	38Ar(k) [fA]	%1σ	38Ar(ca) [fA]	%1σ	38Ar(cl) [fA]	%1σ	39Ar(k) [fA]	%1σ	39Ar(ca) [fA]	%1σ	40Ar(r) [fA]	%1σ	40Ar(a) [fA]	%1σ	40Ar(c) [fA]	%1σ	40Ar(k) [fA]	%1σ
17D30245	1.8 %	✓	0.688379	0.41	0.0000000	0.00	0.0260516	0.56	0.0000084	57.75	96.3804	0.53	0.1286581	0.41	0.0000000	0.00	0.8519693	0.11	0.0173485	9.64	0.0415861	57.76	70.54478	0.07	0.0619244	1.06	21.10193	4.61	203.4160	0.41	0.0000000	0.00	0.0428207	9.65
17D30247	1.9 %	✓	0.268261	0.78	0.0000000	0.00	0.0109742	0.91	0.0000033	148.92	40.6000	0.90	0.0501381	0.78	0.0000000	0.00	0.3370652	0.14	0.0073080	9.67	0.0164577	148.92	27.90968	0.10	0.0260855	1.28	7.33259	10.50	79.2713	0.78	0.0000000	0.00	0.0169412	9.65
17D30248	2.0 %	✓	0.416079	0.57	0.0000000	0.00	0.0212095	0.63	0.0000040	118.51	78.4665	0.61	0.0777652	0.57	0.0000000	0.00	0.4356067	0.13	0.0141240	9.65	0.0198308	118.51	36.06912	0.09	0.0504148	1.10	10.60543	7.92	122.9514	0.57	0.0000000	0.00	0.0218940	9.65
17D30250	2.2 %	✓	0.151605	1.27	0.0000000	0.00	0.0071016	1.32	0.0000042	117.16	26.2732	1.31	0.0283350	1.27	0.0000000	0.00	0.1626954	0.20	0.0047292	9.72	0.0209914	117.16	13.47151	0.18	0.0168805	1.60	2.48180	29.43	44.7993	1.27	0.0000000	0.00	0.0081772	9.65
17D30251	2.4 %	✓	0.133700	1.42	0.0000000	0.00	0.0076947	1.26	0.0000066	70.77	28.4674	1.25	0.0249884	1.42	0.0000000	0.00	0.1527318	0.20	0.0051241	9.71	0.0327644	70.77	12.64650	0.18	0.0182903	1.55	2.89492	25.03	39.5082	1.42	0.0000000	0.00	0.0076764	9.65
17D30253	2.7 %	✓	0.287399	0.72	0.0000000	0.00	0.0221514	0.59	0.0000007	743.72	81.9513	0.57	0.0537148	0.72	0.0000000	0.00	0.2771512	0.14	0.0147512	9.65	0.0032474	743.72	22.94868	0.11	0.0526537	1.08	5.71553	13.43	84.9263	0.72	0.0000000	0.00	0.0139298	9.65
17D30254	3.0 %	✓	0.476137	0.52	0.0000000	0.00	0.0384826	0.49	0.0000113	44.70	142.3699	0.46	0.0889901	0.52	0.0000000	0.00	0.3324565	0.14	0.0256266	9.64	0.0563787	44.71	27.52807	0.10	0.0914726	1.03	8.05366	10.74	140.6986	0.52	0.0000000	0.00	0.0167095	9.65
17D30256	3.4 %	✓	0.387712	0.60	0.0000000	0.00	0.0371824	0.49	0.0000004	######	137.5598	0.46	0.0724634	0.60	0.0000000	0.00	0.2292632	0.16	0.0247608	9.64	0.0018799	######	18.98346	0.13	0.0883822	1.03	2.95805	27.84	114.5689	0.60	0.0000000	0.00	0.0115230	9.65
17D30257	3.9 %	✓	0.339377	0.66	0.0000000	0.00	0.0373174	0.50	0.0000043	117.62	138.0593	0.47	0.0634295	0.66	0.0000000	0.00	0.1805967	0.18	0.0248507	9.64	0.0214360	117.63	14.95377	0.16	0.0887031	1.03	4.54423	17.65	100.2859	0.66	0.0000000	0.00	0.0090769	9.65
17D30259	4.5 %	✓	0.135287	1.41	0.0000000	0.00	0.0179515	0.66	0.0000000	0.00	66.4132	0.64	0.0252851	1.41	0.0000000	0.00	0.0936594	0.29	0.0119544	9.65	0.0000000	0.00	7.75518	0.27	0.0426705	1.12	1.47527	49.09	39.9772	1.41	0.0000000	0.00	0.0047074	9.65
17D30260	5.2 %	✓	0.759663	0.42	0.0000000	0.00	0.1328120	0.43	0.0000002	######	491.3504	0.40	0.1419810	0.42	0.0000000	0.00	0.3183824	0.14	0.0884431	9.64	0.0010489	######	26.36271	0.11	0.3156927	1.00	5.47585	19.27	224.4804	0.42	0.0000000	0.00	0.0160022	9.65
17D30262	6.0 %	✓	0.495713	0.52	0.0000000	0.00	0.1029984	0.44	0.0000000	0.00	381.0522	0.40	0.0926487	0.52	0.0000000	0.00	0.1947921	0.18	0.0685894	9.64	0.0000000	0.00	16.12918	0.16	0.2448261	1.00	4.71650	19.00	146.4832	0.52	0.0000000	0.00	0.0097904	9.65
17D30263	6.9 %	✓	0.118710	1.59	0.0000000	0.00	0.0272108	0.55	0.0000046	111.15	100.6688	0.52	0.0221869	1.59	0.0000000	0.00	0.0541439	0.48	0.0181204	9.64	0.0227234	111.15	4.48322	0.47	0.0646797	1.06	0.65480	110.25	35.0788	1.59	0.0000000	0.00	0.0027213	9.66
17D30265	7.9 %	✓	0.328802	0.46	0.0000000	0.00	0.0802542	0.44	0.0000021	224.89	296.9079	0.41	0.0614531	0.46	0.0000000	0.00	0.1288411	0.24	0.0534434	9.64	0.0105847	224.90	10.66830	0.23	0.1907633	1.01	2.46112	18.32	97.1610	0.46	0.0000000	0.00	0.0064757	9.65
17D30266	9.0 %	✓	0.353749	0.42	0.0000000	0.00	0.0710299	0.44	0.0000016	310.37	262.7818	0.41	0.0661156	0.42	0.0000000	0.00	0.1050446	0.29	0.0473007	9.64	0.0081654	310.37	8.69791	0.27	0.1688373	1.01	2.31414	19.29	104.5327	0.42	0.0000000	0.00	0.0052796	9.65
17D30268	10.3 %	✓	0.226829	0.48	0.0000000	0.00	0.0458290	0.47	0.0000000	0.00	169.5487	0.43	0.0423944	0.48	0.0000000	0.00	0.0670118	0.41	0.0305188	9.64	0.0000000	0.00	5.54871	0.40	0.1089350	1.02	1.33599	24.05	67.0281	0.48	0.0000000	0.00	0.0033681	9.66
17D30269	11.6 %	✓	0.595904	0.35	0.0000000	0.00	0.0664319	0.45	0.0000000	0.00	245.7710	0.41	0.1113745	0.35	0.0000000	0.00	0.1074705	0.28	0.0442388	9.64	0.0000000	0.00	8.89877	0.27	0.1579079	1.01	1.01915	60.33	176.0898	0.35	0.0000000	0.00	0.0054016	9.65
17D30271	12.5 %		1.439492	0.29	0.0000000	0.00	0.0874818	0.44	0.0000000	0.00	323.6471	0.41	0.2690411	0.29	0.0000000	0.00	0.1499875	0.22	0.0582565	9.64	0.0000000	0.00	12.41927	0.20	0.2079433	1.01	4.69717	26.64	425.3699	0.29	0.0000000	0.00	0.0075385	9.65
17D30272	13.4 %		1.627785	0.28	0.0000000	0.00	0.0917280	0.44	0.0000042	116.60	339.3562	0.40	0.3042330	0.28	0.0000000	0.00	0.1397362	0.23	0.0610841	9.64	0.0209445	116.60	11.57044	0.21	0.2180364	1.00	4.75820	28.52	481.0105	0.28	0.0000000	0.00	0.0070233	9.65
17D30274	14.6 %		1.494379	0.30	0.0000000	0.00	0.1430452	0.43	0.0000004	######	529.2090	0.40	0.2792995	0.30	0.0000000	0.00	0.1560855	0.21	0.0952576	9.64	0.0017435	######	12.92420	0.19	0.3400168	1.00	4.52740	29.28	441.5890	0.30	0.0000000	0.00	0.0078450	9.65
17D30275	16.0 %		0.840514	0.35	0.0000000	0.00	0.1408474	0.43	0.0000000	0.00	521.0780	0.40	0.1570920	0.35	0.0000000	0.00	0.1035154	0.30	0.0937940	9.64	0.0000000	0.00	8.57128	0.29	0.3347926	1.00	3.82410	22.55	248.3718	0.35	0.0000000	0.00	0.0052028	9.65
17D30277	17.6 %	✓	0.360132	0.29	0.0000000	0.00	0.0574130	0.42	0.0000000	0.00	212.4047	0.38	0.0673086	0.29	0.0000000	0.00	0.0457165	0.59	0.0382328	9.64	0.0000000	0.00	3.78542	0.58	0.1364700	1.00	0.16576	190.07	106.4190	0.29	0.0000000	0.00	0.0022977	9.67
17D30278	19.3 %	✓	0.315430	0.34	0.0000000	0.00	0.0783113	0.40	0.0000000	0.00	289.7200	0.37	0.0589539	0.34	0.0000000	0.00	0.0420895	0.67	0.0521496	9.64	0.0000000	0.00	3.48510	0.66	0.1861451	0.99	0.73020	44.20	93.2097	0.34	0.0000000	0.00	0.0021155	9.67
17D30280	21.0 %	✓	0.232323	0.41	0.0000000	0.00	0.0303532	0.49	0.0000013	360.57	112.2945	0.46	0.0434212	0.41	0.0000000	0.00	0.0213704	1.16	0.0202130	9.64	0.0065970	360.57	1.76951	1.15	0.0721492	1.03	0.35821	78.03	68.6515	0.41	0.0000000	0.00	0.0010741	9.72
		Σ	12.473362	0.10	0.0000000	0.00	1.3818632	0.11	0.0000576	34.15	5112.3314	0.10	2.3312713	0.10	0.0000000	0.00	4.6873827	0.04	0.9202197	2.42	0.2863800	34.15	388.12475	0.03	3.2846729	0.25	104.20202	3.84	3685.8784	0.10	0.0000000	0.00	0.2355917	2.63
		Σ							13.855283	0.09	5112.3314	0.10									8.2252536	1.22			391.40943	0.03							3790.3161	0.14

**Additional Parameters**			**40Ar/39Ar**	**1σ**	**37Ar/39Ar**	**1σ**	**36Ar/39Ar**	**1σ**	**Time (days)**	**37Ar (decay)**	**39Ar (decay)**	**40Ar (moles)**
17D30245	1.8 %	✓	3.180446	0.007210	1.365032	0.007325	0.010119	0.000041	123.785	11.645374	1.00087736	1.078E-11
17D30247	1.9 %	✓	3.100714	0.016774	1.453333	0.013120	0.009996	0.000075	123.799	11.648569	1.00087746	4.158E-12
17D30248	2.0 %	✓	3.698241	0.013256	2.172413	0.013293	0.012107	0.000066	123.806	11.650167	1.00087751	6.412E-12
17D30250	2.2 %	✓	3.505928	0.034497	1.947837	0.025712	0.011766	0.000144	123.820	11.653523	1.00087761	2.270E-12
17D30251	2.4 %	✓	3.348718	0.036623	2.247763	0.028366	0.011165	0.000151	123.827	11.655122	1.00087766	2.036E-12
17D30253	2.7 %	✓	3.941325	0.020434	3.562893	0.020593	0.013458	0.000092	123.841	11.658320	1.00087776	4.351E-12
17D30254	3.0 %	✓	5.386366	0.017629	5.154679	0.024278	0.018633	0.000091	123.848	11.659919	1.00087781	7.141E-12
17D30256	3.4 %	✓	6.162934	0.025338	7.212719	0.034759	0.022279	0.000124	123.862	11.663118	1.00087791	5.642E-12
17D30257	3.9 %	✓	6.969542	0.032357	9.177961	0.045729	0.025042	0.000153	123.869	11.664718	1.00087796	5.032E-12
17D30259	4.5 %	✓	5.316483	0.060379	8.516860	0.059348	0.019651	0.000249	123.883	11.667919	1.00087806	1.990E-12
17D30260	5.2 %	✓	8.620168	0.019394	18.417538	0.076030	0.033453	0.000124	123.890	11.669519	1.00087811	1.104E-11
17D30262	6.0 %	✓	9.234723	0.031498	23.271772	0.100378	0.036565	0.000166	123.903	11.672721	1.00087820	7.258E-12
17D30263	6.9 %	✓	7.857770	0.107026	22.135218	0.155406	0.032086	0.000441	123.910	11.674322	1.00087825	1.715E-12
17D30265	7.9 %	✓	9.174691	0.020724	27.341938	0.126590	0.037670	0.000160	123.924	11.677525	1.00087835	4.782E-12
17D30266	9.0 %	✓	12.050889	0.032409	29.636793	0.145076	0.047907	0.000209	123.931	11.679127	1.00087840	5.129E-12
17D30268	10.3 %	✓	12.084077	0.047274	29.968055	0.174731	0.048193	0.000265	123.945	11.682332	1.00087850	3.282E-12
17D30269	11.6 %	✓	19.556202	0.051621	27.136982	0.133212	0.073132	0.000298	123.952	11.683934	1.00087855	8.501E-12
17D30271	12.5 %		34.059344	0.066276	25.630924	0.115176	0.120927	0.000408	123.967	11.687300	1.00087865	2.064E-11
17D30272	13.4 %		41.207682	0.086309	28.787120	0.130670	0.145864	0.000494	123.974	11.688903	1.00087870	2.332E-11
17D30274	14.6 %		33.633680	0.062448	39.897507	0.174670	0.123447	0.000406	123.988	11.692110	1.00087880	2.141E-11
17D30275	16.0 %		28.317877	0.077442	58.508148	0.281568	0.110190	0.000439	123.994	11.693714	1.00087885	1.211E-11
17D30277	17.6 %	✓	27.202324	0.152812	54.158820	0.366908	0.106549	0.000653	124.008	11.696923	1.00087894	5.116E-12
17D30278	19.3 %	✓	25.611986	0.160013	78.916043	0.571118	0.107329	0.000727	124.015	11.698527	1.00087899	4.509E-12
17D30280	21.0 %	✓	37.506295	0.415551	60.974594	0.730162	0.142746	0.001659	124.029	11.701737	1.00087909	3.313E-12

Procedure Blanks		36Ar ± 1σ (SE) [fA]	37Ar ± 1σ (SE) [fA]	38Ar ± 1σ (SE) [fA]	39Ar ± 1σ (SE) [fA]	40Ar ± 1σ (SE) [fA]
17D30245	1.8 %	0.0163573 ± 0.0016797	0.0182330 ± 0.0219138	0.0115212 ± 0.0172984	0.0200119 ± 0.0151588	4.9177788 ± 0.4566886
17D30247	1.9 %	0.0171225 ± 0.0016797	0.0138029 ± 0.0219138	0.0150779 ± 0.0172984	0.0364287 ± 0.0151588	5.1422352 ± 0.4566886
17D30248	2.0 %	0.0169950 ± 0.0016797	0.0166109 ± 0.0219138	0.0166392 ± 0.0172984	0.0374532 ± 0.0151588	5.1092368 ± 0.4566886
17D30250	2.2 %	0.0162691 ± 0.0016797	0.0262318 ± 0.0219138	0.0191541 ± 0.0172984	0.0314401 ± 0.0151588	4.9077172 ± 0.4566886
17D30251	2.4 %	0.0158933 ± 0.0016797	0.0305023 ± 0.0219138	0.0199015 ± 0.0172984	0.0269551 ± 0.0151588	4.8016664 ± 0.4566886
17D30253	2.7 %	0.0153907 ± 0.0016797	0.0350187 ± 0.0219138	0.0203774 ± 0.0172984	0.0189148 ± 0.0151588	4.6560440 ± 0.4566886
17D30254	3.0 %	0.0153275 ± 0.0016797	0.0345481 ± 0.0219138	0.0200724 ± 0.0172984	0.0164113 ± 0.0151588	4.6340080 ± 0.4566886
17D30256	3.4 %	0.0155963 ± 0.0016797	0.0278960 ± 0.0219138	0.0183511 ± 0.0172984	0.0157290 ± 0.0151588	4.6955081 ± 0.4566886
17D30257	3.9 %	0.0158798 ± 0.0016797	0.0222287 ± 0.0219138	0.0169484 ± 0.0172984	0.0175317 ± 0.0151588	4.7647256 ± 0.4566886
17D30259	4.5 %	0.0164836 ± 0.0016797	0.0090354 ± 0.0219138	0.0131518 ± 0.0172984	0.0240412 ± 0.0151588	4.9044817 ± 0.4566886
17D30260	5.2 %	0.0166433 ± 0.0016797	0.0032543 ± 0.0219138	0.0108188 ± 0.0172984	0.0276594 ± 0.0151588	4.9288476 ± 0.4566886
17D30262	6.0 %	0.0161371 ± 0.0016797	0.0007759 ± 0.0219138	0.0054944 ± 0.0172984	0.0315638 ± 0.0151588	4.7313061 ± 0.4566886
17D30263	6.9 %	0.0151985 ± 0.0016797	0.0039506 ± 0.0219138	0.0026112 ± 0.0172984	0.0296914 ± 0.0151588	4.4313720 ± 0.4566886
17D30265	7.9 %	0.0138974 ± 0.0002268	0.0195524 ± 0.0191487	0.0027655 ± 0.0168440	0.0232105 ± 0.0142806	4.0214813 ± 0.0171261
17D30266	9.0 %	0.0138974 ± 0.0002268	0.0195524 ± 0.0191487	0.0027655 ± 0.0168440	0.0232105 ± 0.0142806	4.0214813 ± 0.0171261
17D30268	10.3 %	0.0140472 ± 0.0002161	0.0047824 ± 0.0176455	0.0150861 ± 0.0173975	0.0340377 ± 0.0154027	4.2291355 ± 0.0171954
17D30269	11.6 %	0.0140472 ± 0.0002161	0.0047824 ± 0.0176455	0.0150861 ± 0.0173975	0.0340377 ± 0.0154027	4.2291355 ± 0.0171954
17D30271	12.5 %	0.0138637 ± 0.0002333	0.0026369 ± 0.0180256	0.0385837 ± 0.0161771	0.0207347 ± 0.0156820	4.1661129 ± 0.0199956
17D30272	13.4 %	0.0138637 ± 0.0002333	0.0026369 ± 0.0180256	0.0385837 ± 0.0161771	0.0207347 ± 0.0156820	4.1661129 ± 0.0199956
17D30274	14.6 %	0.0141975 ± 0.0002394	0.0303211 ± 0.0164739	0.0265655 ± 0.0145976	0.0682784 ± 0.0162058	4.2939195 ± 0.0173754
17D30275	16.0 %	0.0141975 ± 0.0002394	0.0303211 ± 0.0164739	0.0265655 ± 0.0145976	0.0682784 ± 0.0162058	4.2939195 ± 0.0173754
17D30277	17.6 %	0.0143471 ± 0.0002233	0.0303675 ± 0.0184141	0.0204629 ± 0.0153669	0.0618807 ± 0.0158082	4.2256087 ± 0.0184925
17D30278	19.3 %	0.0143471 ± 0.0002233	0.0303675 ± 0.0184141	0.0204629 ± 0.0153669	0.0618807 ± 0.0158082	4.2256087 ± 0.0184925
17D30280	21.0 %	0.0142988 ± 0.0002380	0.0102723 ± 0.0189810	0.0215822 ± 0.0172845	0.0540301 ± 0.0148029	4.3211393 ± 0.0163432

**Intercept Values**		**36Ar ± 1σ (SE) [fA]**	**r2**	**Regression (type,n)**	**37Ar ± 1σ (SE) [fA]**	**r2**	**Regression (type,n)**	**38Ar ± 1σ (SE) [fA]**	**r2**	**Regression (type,n)**	**39Ar ± 1σ (SE) [fA]**	**r2**	**Regression (type,n)**	**40Ar ± 1σ (SE) [fA]**	**r2**	**Regression (type,n)**
17D30245	1.8 %	0.6993879 ± 0.0013630	0.8821	EXP 150 of 150	8.1454797 ± 0.0193714	0.8547	EXP 150 of 150	1.0385868 ± 0.0160676	0.0918	EXP 149 of 150	70.1415649 ± 0.0218000	0.9977	EXP 150 of 150	229.478574 ± 0.159664	0.0005	EXP 150 of 150
17D30247	1.9 %	0.2840855 ± 0.0008590	0.5567	EXP 150 of 150	3.4364408 ± 0.0168102	0.5224	EXP 149 of 150	0.4211067 ± 0.0169090	0.0138	EXP 150 of 150	27.7802373 ± 0.0171282	0.9903	EXP 150 of 150	91.763041 ± 0.057074	0.9745	EXP 150 of 150
17D30248	2.0 %	0.4350633 ± 0.0011247	0.7510	EXP 150 of 150	6.6305515 ± 0.0212832	0.7487	EXP 150 of 150	0.5573866 ± 0.0153981	0.0111	EXP 150 of 150	35.9087962 ± 0.0170648	0.9943	EXP 150 of 150	138.687989 ± 0.084507	0.8784	EXP 150 of 150
17D30250	2.2 %	0.1680028 ± 0.0006568	0.2788	EXP 150 of 150	2.2401591 ± 0.0167987	0.2968	EXP 150 of 150	0.2332996 ± 0.0170454	0.0180	EXP 150 of 150	13.4271423 ± 0.0175237	0.9532	EXP 150 of 150	52.197029 ± 0.021420	0.9971	EXP 150 of 150
17D30251	2.4 %	0.1510778 ± 0.0006075	0.1060	EXP 150 of 150	2.4290014 ± 0.0181189	0.3559	EXP 149 of 150	0.2329184 ± 0.0149960	0.0482	EXP 150 of 150	12.6047186 ± 0.0156655	0.9590	EXP 150 of 150	47.212470 ± 0.022864	0.9968	EXP 150 of 150
17D30253	2.7 %	0.3113327 ± 0.0007805	0.7629	EXP 150 of 150	6.9378575 ± 0.0178725	0.8051	EXP 150 of 150	0.3650484 ± 0.0163591	0.0105	EXP 150 of 150	22.8621887 ± 0.0157473	0.9872	EXP 150 of 150	95.311770 ± 0.043621	0.9718	EXP 150 of 150
17D30254	3.0 %	0.5073343 ± 0.0011446	0.8523	EXP 150 of 150	12.0248592 ± 0.0187939	0.9349	EXP 150 of 150	0.5174723 ± 0.0177119	0.0747	EXP 150 of 150	27.4461588 ± 0.0166860	0.9908	EXP 150 of 150	153.402966 ± 0.075632	0.4620	EXP 150 of 150
17D30256	3.4 %	0.4218117 ± 0.0010330	0.8238	EXP 150 of 150	11.6099297 ± 0.0188358	0.9270	EXP 150 of 150	0.3427711 ± 0.0171488	0.0087	EXP 150 of 150	18.9565098 ± 0.0152030	0.9826	EXP 150 of 150	122.233994 ± 0.048016	0.8670	EXP 150 of 150
17D30257	3.9 %	0.3760177 ± 0.0009561	0.8129	EXP 150 of 150	11.6447201 ± 0.0216401	0.9164	EXP 150 of 150	0.3037715 ± 0.0177546	0.0543	EXP 150 of 150	14.9566383 ± 0.0153716	0.9712	EXP 150 of 150	109.603888 ± 0.038624	0.9539	EXP 150 of 150
17D30259	4.5 %	0.1629849 ± 0.0005845	0.4461	EXP 150 of 150	5.5984870 ± 0.0180613	0.7242	EXP 150 of 150	0.1370251 ± 0.0163235	0.0013	EXP 150 of 150	7.7683101 ± 0.0138419	0.8934	EXP 150 of 150	46.361648 ± 0.020657	0.9965	EXP 150 of 150
17D30260	5.2 %	0.8698831 ± 0.0014251	0.9361	EXP 150 of 150	41.3504763 ± 0.0232125	0.9910	EXP 150 of 150	0.5540645 ± 0.0157706	0.0404	EXP 149 of 150	26.5227264 ± 0.0167335	0.9895	EXP 150 of 150	234.901120 ± 0.044233	0.9764	EXP 150 of 150
17D30262	6.0 %	0.5885275 ± 0.0011162	0.8964	EXP 150 of 150	32.0560366 ± 0.0202374	0.9881	EXP 150 of 150	0.3401098 ± 0.0174318	0.0125	EXP 150 of 150	16.2930503 ± 0.0174192	0.9697	EXP 150 of 150	155.940749 ± 0.056630	0.0717	EXP 149 of 150
17D30263	6.9 %	0.1547087 ± 0.0005565	0.3314	EXP 150 of 150	8.4717586 ± 0.0196957	0.8500	EXP 150 of 150	0.1183772 ± 0.0178926	0.0075	EXP 150 of 150	4.5463380 ± 0.0144224	0.6598	EXP 150 of 150	40.167725 ± 0.022269	0.9968	EXP 150 of 150
17D30265	7.9 %	0.4049724 ± 0.0010211	0.8031	EXP 150 of 150	24.9872683 ± 0.0184298	0.9839	EXP 150 of 150	0.2484995 ± 0.0155939	0.0119	EXP 150 of 150	10.8076490 ± 0.0181206	0.9167	EXP 150 of 150	103.650042 ± 0.037396	0.9453	EXP 150 of 150
17D30266	9.0 %	0.4200032 ± 0.0009748	0.8340	EXP 149 of 150	22.1144960 ± 0.0193960	0.9778	EXP 150 of 150	0.2211366 ± 0.0179615	0.0038	EXP 149 of 150	8.8290194 ± 0.0177940	0.8701	EXP 150 of 150	110.873616 ± 0.039763	0.9205	EXP 150 of 150
17D30268	10.3 %	0.2747189 ± 0.0007619	0.7300	EXP 150 of 150	14.2566855 ± 0.0201762	0.9429	EXP 150 of 150	0.1447074 ± 0.0160262	0.0001	EXP 150 of 150	5.6528040 ± 0.0150908	0.7656	EXP 150 of 150	72.596584 ± 0.027160	0.9872	EXP 150 of 150
17D30269	11.6 %	0.6472656 ± 0.0012048	0.9250	EXP 150 of 150	20.6609395 ± 0.0212754	0.9678	EXP 149 of 150	0.2540803 ± 0.0166287	0.0010	EXP 150 of 150	9.0284790 ± 0.0171655	0.8866	EXP 150 of 150	181.343446 ± 0.029948	0.9804	EXP 150 of 150
17D30271	12.5 %	1.4737079 ± 0.0019026	0.9674	EXP 150 of 150	27.1908884 ± 0.0209987	0.9822	EXP 150 of 150	0.5011693 ± 0.0163876	0.0147	EXP 150 of 150	12.5611675 ± 0.0170979	0.9476	EXP 150 of 150	434.240711 ± 0.035106	0.9993	EXP 150 of 150
17D30272	13.4 %	1.6577866 ± 0.0017646	0.9766	EXP 150 of 150	28.5068894 ± 0.0190184	0.9869	EXP 150 of 150	0.5582586 ± 0.0168909	0.0415	EXP 150 of 150	11.7281926 ± 0.0174804	0.9286	EXP 149 of 150	489.941829 ± 0.039660	0.9993	EXP 150 of 150
17D30274	14.6 %	1.5796368 ± 0.0018740	0.9712	EXP 150 of 150	44.4773014 ± 0.0202152	0.9936	EXP 150 of 150	0.5525519 ± 0.0163514	0.0115	EXP 150 of 150	13.2413306 ± 0.0165081	0.9504	EXP 150 of 150	450.418192 ± 0.037510	0.9992	EXP 150 of 150
17D30275	16.0 %	0.9524155 ± 0.0014782	0.9493	EXP 150 of 150	43.7883914 ± 0.0182356	0.9947	EXP 150 of 150	0.3758553 ± 0.0162249	0.0238	EXP 150 of 150	8.9131447 ± 0.0171222	0.8840	EXP 149 of 150	256.495050 ± 0.030474	0.9964	EXP 150 of 150
17D30277	17.6 %	0.4125240 ± 0.0009535	0.8783	EXP 150 of 150	17.8141218 ± 0.0194363	0.9649	EXP 150 of 150	0.1660156 ± 0.0174966	0.0014	EXP 150 of 150	3.9509574 ± 0.0150345	0.5956	EXP 150 of 150	110.812636 ± 0.025139	0.5902	EXP 150 of 150
17D30278	19.3 %	0.3898039 ± 0.0009601	0.8494	EXP 149 of 150	24.2840778 ± 0.0206160	0.9789	EXP 150 of 150	0.1504439 ± 0.0158367	0.0039	EXP 150 of 150	3.7024113 ± 0.0163039	0.4894	EXP 150 of 150	98.167605 ± 0.026042	0.9151	EXP 150 of 150
17D30280	21.0 %	0.2647973 ± 0.0008510	0.7213	EXP 150 of 150	9.4083534 ± 0.0200682	0.8804	EXP 150 of 150	0.1119315 ± 0.0157291	0.0047	EXP 150 of 150	1.8802841 ± 0.0137747	0.1107	EXP 149 of 150	73.331966 ± 0.022065	0.9806	EXP 150 of 150

Project Info		Analyst	Irradiation	X-pos	Y-pos	Z/H-pos	Project	Experiment	Nmb
17D30245	1.8 %	Dan Miggins	17-OSU-05	0.00	0.00	4.86	Arctic\O-Connor (16-22)	17D30241	01
17D30247	1.9 %	Dan Miggins	17-OSU-05	0.00	0.00	4.86	Arctic\O-Connor (16-22)	17D30241	01
17D30248	2.0 %	Dan Miggins	17-OSU-05	0.00	0.00	4.86	Arctic\O-Connor (16-22)	17D30241	01
17D30250	2.2 %	Dan Miggins	17-OSU-05	0.00	0.00	4.86	Arctic\O-Connor (16-22)	17D30241	01
17D30251	2.4 %	Dan Miggins	17-OSU-05	0.00	0.00	4.86	Arctic\O-Connor (16-22)	17D30241	01
17D30253	2.7 %	Dan Miggins	17-OSU-05	0.00	0.00	4.86	Arctic\O-Connor (16-22)	17D30241	01
17D30254	3.0 %	Dan Miggins	17-OSU-05	0.00	0.00	4.86	Arctic\O-Connor (16-22)	17D30241	01
17D30256	3.4 %	Dan Miggins	17-OSU-05	0.00	0.00	4.86	Arctic\O-Connor (16-22)	17D30241	01
17D30257	3.9 %	Dan Miggins	17-OSU-05	0.00	0.00	4.86	Arctic\O-Connor (16-22)	17D30241	01
17D30259	4.5 %	Dan Miggins	17-OSU-05	0.00	0.00	4.86	Arctic\O-Connor (16-22)	17D30241	01
17D30260	5.2 %	Dan Miggins	17-OSU-05	0.00	0.00	4.86	Arctic\O-Connor (16-22)	17D30241	01
17D30262	6.0 %	Dan Miggins	17-OSU-05	0.00	0.00	4.86	Arctic\O-Connor (16-22)	17D30241	01
17D30263	6.9 %	Dan Miggins	17-OSU-05	0.00	0.00	4.86	Arctic\O-Connor (16-22)	17D30241	01
17D30265	7.9 %	Dan Miggins	17-OSU-05	0.00	0.00	4.86	Arctic\O-Connor (16-22)	17D30241	01
17D30266	9.0 %	Dan Miggins	17-OSU-05	0.00	0.00	4.86	Arctic\O-Connor (16-22)	17D30241	01
17D30268	10.3 %	Dan Miggins	17-OSU-05	0.00	0.00	4.86	Arctic\O-Connor (16-22)	17D30241	01
17D30269	11.6 %	Dan Miggins	17-OSU-05	0.00	0.00	4.86	Arctic\O-Connor (16-22)	17D30241	01
17D30271	12.5 %	Dan Miggins	17-OSU-05	0.00	0.00	4.86	Arctic\O-Connor (16-22)	17D30241	01
17D30272	13.4 %	Dan Miggins	17-OSU-05	0.00	0.00	4.86	Arctic\O-Connor (16-22)	17D30241	01
17D30274	14.6 %	Dan Miggins	17-OSU-05	0.00	0.00	4.86	Arctic\O-Connor (16-22)	17D30241	01
17D30275	16.0 %	Dan Miggins	17-OSU-05	0.00	0.00	4.86	Arctic\O-Connor (16-22)	17D30241	01
17D30277	17.6 %	Dan Miggins	17-OSU-05	0.00	0.00	4.86	Arctic\O-Connor (16-22)	17D30241	01
17D30278	19.3 %	Dan Miggins	17-OSU-05	0.00	0.00	4.86	Arctic\O-Connor (16-22)	17D30241	01
17D30280	21.0 %	Dan Miggins	17-OSU-05	0.00	0.00	4.86	Arctic\O-Connor (16-22)	17D30241	01

**Sample Parameters**		Sample	Material	Location	Standard Name	Standard (in Ma)	%1σ	Standard Reference	Standard 40Ar/39Ar	%1σ	J	%1σ	Air 40Ar/36Ar	%1σ	MDF (lin)	%1σ	Volume Ratio	Sensitivity (mol/volt)	Day	Month	Year	Hour	Min	Resist
17D30245	1.8 %	PS59-231-20	Groundmass	Gakkel Ridge	FCT-NM (5B3-17)	28.201	0.082	Kuiper et al (2008)	9.56637	0.083	0.00164298	0.083	302.771	0.053	0.9939885	0.060	1	4.8E-14	12	SEP	2017	6	40	1
17D30247	1.9 %	PS59-231-20	Groundmass	Gakkel Ridge	FCT-NM (5B3-17)	28.201	0.082	Kuiper et al (2008)	9.56637	0.083	0.00164298	0.083	302.771	0.053	0.9939885	0.060	1	4.8E-14	12	SEP	2017	7	0	1
17D30248	2.0 %	PS59-231-20	Groundmass	Gakkel Ridge	FCT-NM (5B3-17)	28.201	0.082	Kuiper et al (2008)	9.56637	0.083	0.00164298	0.083	302.771	0.053	0.9939885	0.060	1	4.8E-14	12	SEP	2017	7	10	1
17D30250	2.2 %	PS59-231-20	Groundmass	Gakkel Ridge	FCT-NM (5B3-17)	28.201	0.082	Kuiper et al (2008)	9.56637	0.083	0.00164298	0.083	302.771	0.053	0.9939885	0.060	1	4.8E-14	12	SEP	2017	7	31	1
17D30251	2.4 %	PS59-231-20	Groundmass	Gakkel Ridge	FCT-NM (5B3-17)	28.201	0.082	Kuiper et al (2008)	9.56637	0.083	0.00164298	0.083	302.771	0.053	0.9939885	0.060	1	4.8E-14	12	SEP	2017	7	41	1
17D30253	2.7 %	PS59-231-20	Groundmass	Gakkel Ridge	FCT-NM (5B3-17)	28.201	0.082	Kuiper et al (2008)	9.56637	0.083	0.00164298	0.083	302.771	0.053	0.9939885	0.060	1	4.8E-14	12	SEP	2017	8	1	1
17D30254	3.0 %	PS59-231-20	Groundmass	Gakkel Ridge	FCT-NM (5B3-17)	28.201	0.082	Kuiper et al (2008)	9.56637	0.083	0.00164298	0.083	302.771	0.053	0.9939885	0.060	1	4.8E-14	12	SEP	2017	8	11	1
17D30256	3.4 %	PS59-231-20	Groundmass	Gakkel Ridge	FCT-NM (5B3-17)	28.201	0.082	Kuiper et al (2008)	9.56637	0.083	0.00164298	0.083	302.771	0.053	0.9939885	0.060	1	4.8E-14	12	SEP	2017	8	31	1
17D30257	3.9 %	PS59-231-20	Groundmass	Gakkel Ridge	FCT-NM (5B3-17)	28.201	0.082	Kuiper et al (2008)	9.56637	0.083	0.00164298	0.083	302.771	0.053	0.9939885	0.060	1	4.8E-14	12	SEP	2017	8	41	1
17D30259	4.5 %	PS59-231-20	Groundmass	Gakkel Ridge	FCT-NM (5B3-17)	28.201	0.082	Kuiper et al (2008)	9.56637	0.083	0.00164298	0.083	302.771	0.053	0.9939885	0.060	1	4.8E-14	12	SEP	2017	9	1	1
17D30260	5.2 %	PS59-231-20	Groundmass	Gakkel Ridge	FCT-NM (5B3-17)	28.201	0.082	Kuiper et al (2008)	9.56637	0.083	0.00164298	0.083	302.771	0.053	0.9939885	0.060	1	4.8E-14	12	SEP	2017	9	11	1
17D30262	6.0 %	PS59-231-20	Groundmass	Gakkel Ridge	FCT-NM (5B3-17)	28.201	0.082	Kuiper et al (2008)	9.56637	0.083	0.00164298	0.083	302.771	0.053	0.9939885	0.060	1	4.8E-14	12	SEP	2017	9	31	1
17D30263	6.9 %	PS59-231-20	Groundmass	Gakkel Ridge	FCT-NM (5B3-17)	28.201	0.082	Kuiper et al (2008)	9.56637	0.083	0.00164298	0.083	302.771	0.053	0.9939885	0.060	1	4.8E-14	12	SEP	2017	9	41	1
17D30265	7.9 %	PS59-231-20	Groundmass	Gakkel Ridge	FCT-NM (5B3-17)	28.201	0.082	Kuiper et al (2008)	9.56637	0.083	0.00164298	0.083	302.771	0.053	0.9939885	0.060	1	4.8E-14	12	SEP	2017	10	1	1
17D30266	9.0 %	PS59-231-20	Groundmass	Gakkel Ridge	FCT-NM (5B3-17)	28.201	0.082	Kuiper et al (2008)	9.56637	0.083	0.00164298	0.083	302.771	0.053	0.9939885	0.060	1	4.8E-14	12	SEP	2017	10	11	1
17D30268	10.3 %	PS59-231-20	Groundmass	Gakkel Ridge	FCT-NM (5B3-17)	28.201	0.082	Kuiper et al (2008)	9.56637	0.083	0.00164298	0.083	302.771	0.053	0.9939885	0.060	1	4.8E-14	12	SEP	2017	10	31	1
17D30269	11.6 %	PS59-231-20	Groundmass	Gakkel Ridge	FCT-NM (5B3-17)	28.201	0.082	Kuiper et al (2008)	9.56637	0.083	0.00164298	0.083	302.771	0.053	0.9939885	0.060	1	4.8E-14	12	SEP	2017	10	41	1
17D30271	12.5 %	PS59-231-20	Groundmass	Gakkel Ridge	FCT-NM (5B3-17)	28.201	0.082	Kuiper et al (2008)	9.56637	0.083	0.00164298	0.083	302.771	0.053	0.9939885	0.060	1	4.8E-14	12	SEP	2017	11	2	1
17D30272	13.4 %	PS59-231-20	Groundmass	Gakkel Ridge	FCT-NM (5B3-17)	28.201	0.082	Kuiper et al (2008)	9.56637	0.083	0.00164298	0.083	302.771	0.053	0.9939885	0.060	1	4.8E-14	12	SEP	2017	11	12	1
17D30274	14.6 %	PS59-231-20	Groundmass	Gakkel Ridge	FCT-NM (5B3-17)	28.201	0.082	Kuiper et al (2008)	9.56637	0.083	0.00164298	0.083	302.771	0.053	0.9939885	0.060	1	4.8E-14	12	SEP	2017	11	32	1
17D30275	16.0 %	PS59-231-20	Groundmass	Gakkel Ridge	FCT-NM (5B3-17)	28.201	0.082	Kuiper et al (2008)	9.56637	0.083	0.00164298	0.083	302.771	0.053	0.9939885	0.060	1	4.8E-14	12	SEP	2017	11	42	1
17D30277	17.6 %	PS59-231-20	Groundmass	Gakkel Ridge	FCT-NM (5B3-17)	28.201	0.0816				0.00164148	0.081			0.9934	0.007	1	4.8E-14	12	SEP	2017	12	2	1
17D30278	19.3 %	PS59-231-20	Groundmass	Gakkel Ridge	FCT-NM (5B3-17)	28.201	0.0816				0.00164148	0.081			0.9934	0.007	1	4.8E-14	12	SEP	2017	12	12	1
17D30280	21.0 %	PS59-231-20	Groundmass	Gakkel Ridge	FCT-NM (5B3-17)	28.201	0.0816				0.00164148	0.081			0.9934	0.007	1	4.8E-14	12	SEP	2017	12	32	1

Irradiation Constants		40/36(a)	%1σ	40/36(c)	%1σ	38/36(a)	%1σ	38/36(c)	%1σ	39/37(ca)	%1σ	38/37(ca)	%1σ	36/37(ca)	%1σ	40/39(k)	%1σ	38/39(k)	%1σ	36/38(cl)	%1σ	K/Ca	%1σ	K/Cl	%1σ	Ca/Cl	%1σ
17D30245	1.8 %	295.5	0	0.018	35	0.1869	0	1.493	3	0.000643	0.92	0.00018	9.63	0.00027	0.17	0.000607	9.65	0.012077	0.09	0	0	0.43	0	0	0	0	0
17D30247	1.9 %	295.5	0	0.018	35	0.1869	0	1.493	3	0.000643	0.92	0.00018	9.63	0.00027	0.17	0.000607	9.65	0.012077	0.09	0	0	0.43	0	0	0	0	0
17D30248	2.0 %	295.5	0	0.018	35	0.1869	0	1.493	3	0.000643	0.92	0.00018	9.63	0.00027	0.17	0.000607	9.65	0.012077	0.09	0	0	0.43	0	0	0	0	0
17D30250	2.2 %	295.5	0	0.018	35	0.1869	0	1.493	3	0.000643	0.92	0.00018	9.63	0.00027	0.17	0.000607	9.65	0.012077	0.09	0	0	0.43	0	0	0	0	0
17D30251	2.4 %	295.5	0	0.018	35	0.1869	0	1.493	3	0.000643	0.92	0.00018	9.63	0.00027	0.17	0.000607	9.65	0.012077	0.09	0	0	0.43	0	0	0	0	0
17D30253	2.7 %	295.5	0	0.018	35	0.1869	0	1.493	3	0.000643	0.92	0.00018	9.63	0.00027	0.17	0.000607	9.65	0.012077	0.09	0	0	0.43	0	0	0	0	0
17D30254	3.0 %	295.5	0	0.018	35	0.1869	0	1.493	3	0.000643	0.92	0.00018	9.63	0.00027	0.17	0.000607	9.65	0.012077	0.09	0	0	0.43	0	0	0	0	0
17D30256	3.4 %	295.5	0	0.018	35	0.1869	0	1.493	3	0.000643	0.92	0.00018	9.63	0.00027	0.17	0.000607	9.65	0.012077	0.09	0	0	0.43	0	0	0	0	0
17D30257	3.9 %	295.5	0	0.018	35	0.1869	0	1.493	3	0.000643	0.92	0.00018	9.63	0.00027	0.17	0.000607	9.65	0.012077	0.09	0	0	0.43	0	0	0	0	0
17D30259	4.5 %	295.5	0	0.018	35	0.1869	0	1.493	3	0.000643	0.92	0.00018	9.63	0.00027	0.17	0.000607	9.65	0.012077	0.09	0	0	0.43	0	0	0	0	0
17D30260	5.2 %	295.5	0	0.018	35	0.1869	0	1.493	3	0.000643	0.92	0.00018	9.63	0.00027	0.17	0.000607	9.65	0.012077	0.09	0	0	0.43	0	0	0	0	0
17D30262	6.0 %	295.5	0	0.018	35	0.1869	0	1.493	3	0.000643	0.92	0.00018	9.63	0.00027	0.17	0.000607	9.65	0.012077	0.09	0	0	0.43	0	0	0	0	0
17D30263	6.9 %	295.5	0	0.018	35	0.1869	0	1.493	3	0.000643	0.92	0.00018	9.63	0.00027	0.17	0.000607	9.65	0.012077	0.09	0	0	0.43	0	0	0	0	0
17D30265	7.9 %	295.5	0	0.018	35	0.1869	0	1.493	3	0.000643	0.92	0.00018	9.63	0.00027	0.17	0.000607	9.65	0.012077	0.09	0	0	0.43	0	0	0	0	0
17D30266	9.0 %	295.5	0	0.018	35	0.1869	0	1.493	3	0.000643	0.92	0.00018	9.63	0.00027	0.17	0.000607	9.65	0.012077	0.09	0	0	0.43	0	0	0	0	0
17D30268	10.3 %	295.5	0	0.018	35	0.1869	0	1.493	3	0.000643	0.92	0.00018	9.63	0.00027	0.17	0.000607	9.65	0.012077	0.09	0	0	0.43	0	0	0	0	0
17D30269	11.6 %	295.5	0	0.018	35	0.1869	0	1.493	3	0.000643	0.92	0.00018	9.63	0.00027	0.17	0.000607	9.65	0.012077	0.09	0	0	0.43	0	0	0	0	0
17D30271	12.5 %	295.5	0	0.018	35	0.1869	0	1.493	3	0.000643	0.92	0.00018	9.63	0.00027	0.17	0.000607	9.65	0.012077	0.09	0	0	0.43	0	0	0	0	0
17D30272	13.4 %	295.5	0	0.018	35	0.1869	0	1.493	3	0.000643	0.92	0.00018	9.63	0.00027	0.17	0.000607	9.65	0.012077	0.09	0	0	0.43	0	0	0	0	0
17D30274	14.6 %	295.5	0	0.018	35	0.1869	0	1.493	3	0.000643	0.92	0.00018	9.63	0.00027	0.17	0.000607	9.65	0.012077	0.09	0	0	0.43	0	0	0	0	0
17D30275	16.0 %	295.5	0	0.018	35	0.1869	0	1.493	3	0.000643	0.92	0.00018	9.63	0.00027	0.17	0.000607	9.65	0.012077	0.09	0	0	0.43	0	0	0	0	0
17D30277	17.6 %	295.5	0	0.018	35	0.1869	0	1.493	3	0.000643	0.92	0.00018	9.63	0.00027	0.17	0.000607	9.65	0.012077	0.09	0	0	0.43	0	0	0	0	0
17D30278	19.3 %	295.5	0	0.018	35	0.1869	0	1.493	3	0.000643	0.92	0.00018	9.63	0.00027	0.17	0.000607	9.65	0.012077	0.09	0	0	0.43	0	0	0	0	0
17D30280	21.0 %	295.5	0	0.018	35	0.1869	0	1.493	3	0.000643	0.92	0.00018	9.63	0.00027	0.17	0.000607	9.65	0.012077	0.09	0	0	0.43	0	0	0	0	0

17D30241.AGE >>> PS59-231-20 >>> ARCTIC | O-CONNOR (16-22) PROJECT

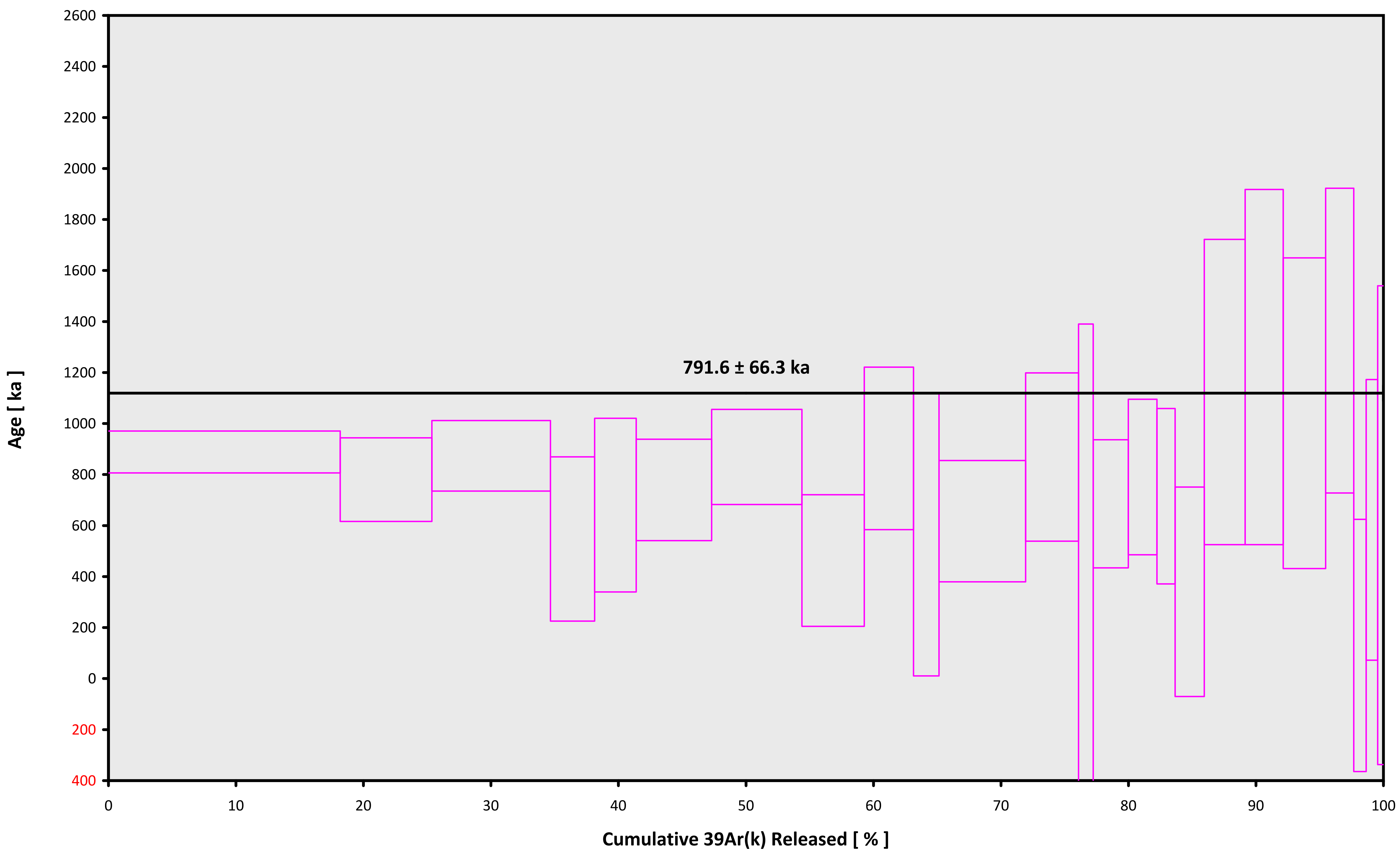

Ar-Ages in ka

WEIGHTED PLATEAU
791.6 ± 66.3
TOTAL FUSION
797.5 ± 61.2
NORMAL ISOCHRON
889.9 ± 80.5
INVERSE ISOCHRON
892.3 ± 78.2

MSWD (PROBABILITY)
1.83 (1%)

Sample Info

Groundmass
Gakkel Ridge
Dan Miggins

IRR = 17-OSU-05 (5B3-17)
J = 0.00164298 ± 0.00000136

17D30241.AGE >>> PS59-231-20 >>> ARCTIC | O-CONNOR (16-22) PROJECT

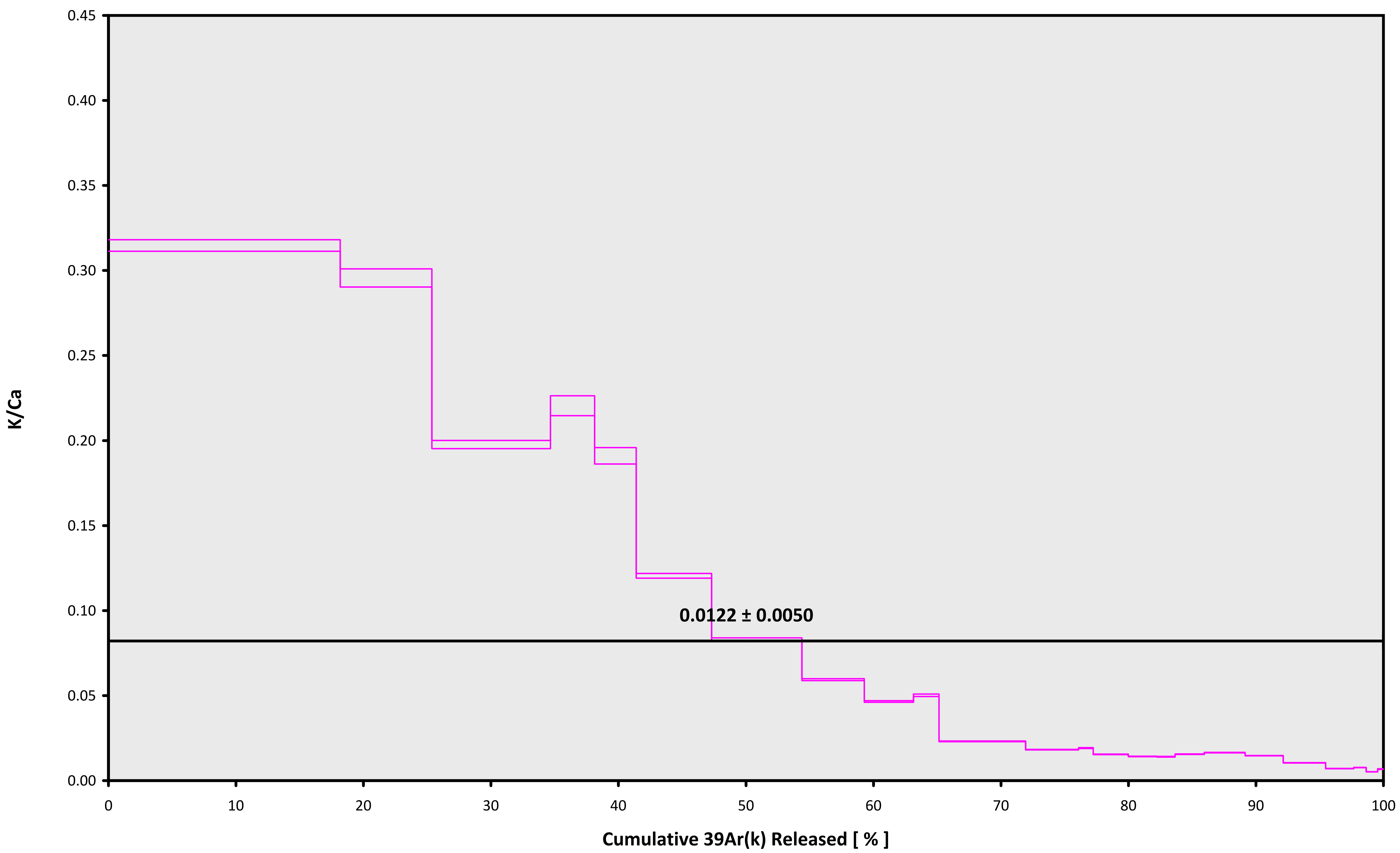

Ar-Ages in ka

WEIGHTED PLATEAU
791.6 ± 66.3
TOTAL FUSION
797.5 ± 61.2
NORMAL ISOCHRON
889.9 ± 80.5
INVERSE ISOCHRON
892.3 ± 78.2

Sample Info

Groundmass
Gakkel Ridge
Dan Miggins

IRR = 17-OSU-05 (5B3-17)
J = 0.00164298 ± 0.00000136

17D30241.AGE >>> PS59-231-20 >>> ARCTIC | O-CONNOR (16-22) PROJECT

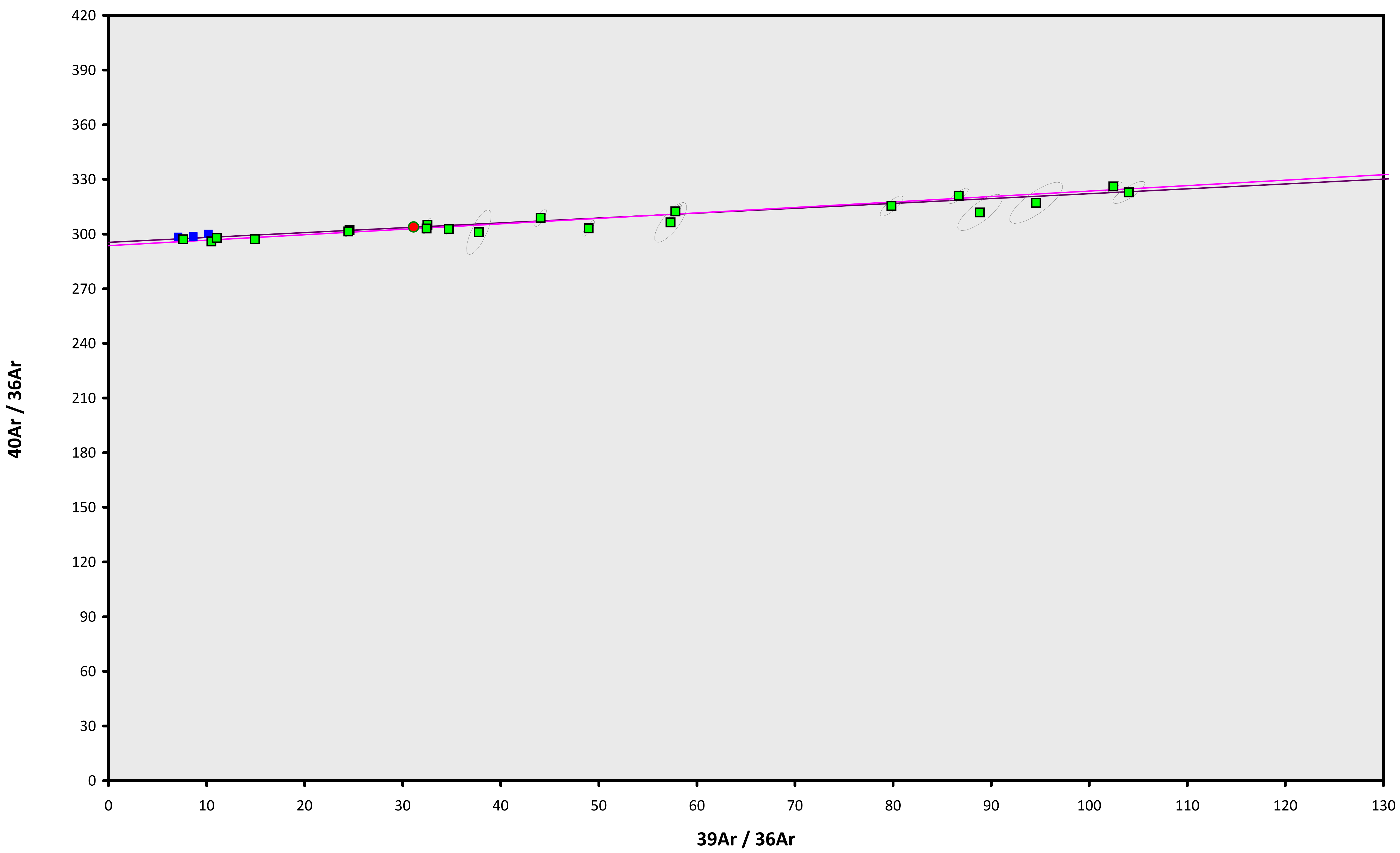

Ar-Ages in ka

WEIGHTED PLATEAU
791.6 ± 66.3
TOTAL FUSION
797.5 ± 61.2
NORMAL ISOCHRON
889.9 ± 80.5
INVERSE ISOCHRON
892.3 ± 78.2

MSWD (PROBABILITY)
1.21 (24%)

40AR/36AR INTERCEPT
293.6 ± 1.1

Sample Info

Groundmass
Gakkel Ridge
Dan Miggins

IRR = 17-OSU-05 (5B3-17)
J = 0.00164298 ± 0.00000136

17D30241.AGE >>> PS59-231-20 >>> ARCTIC | O-CONNOR (16-22) PROJECT

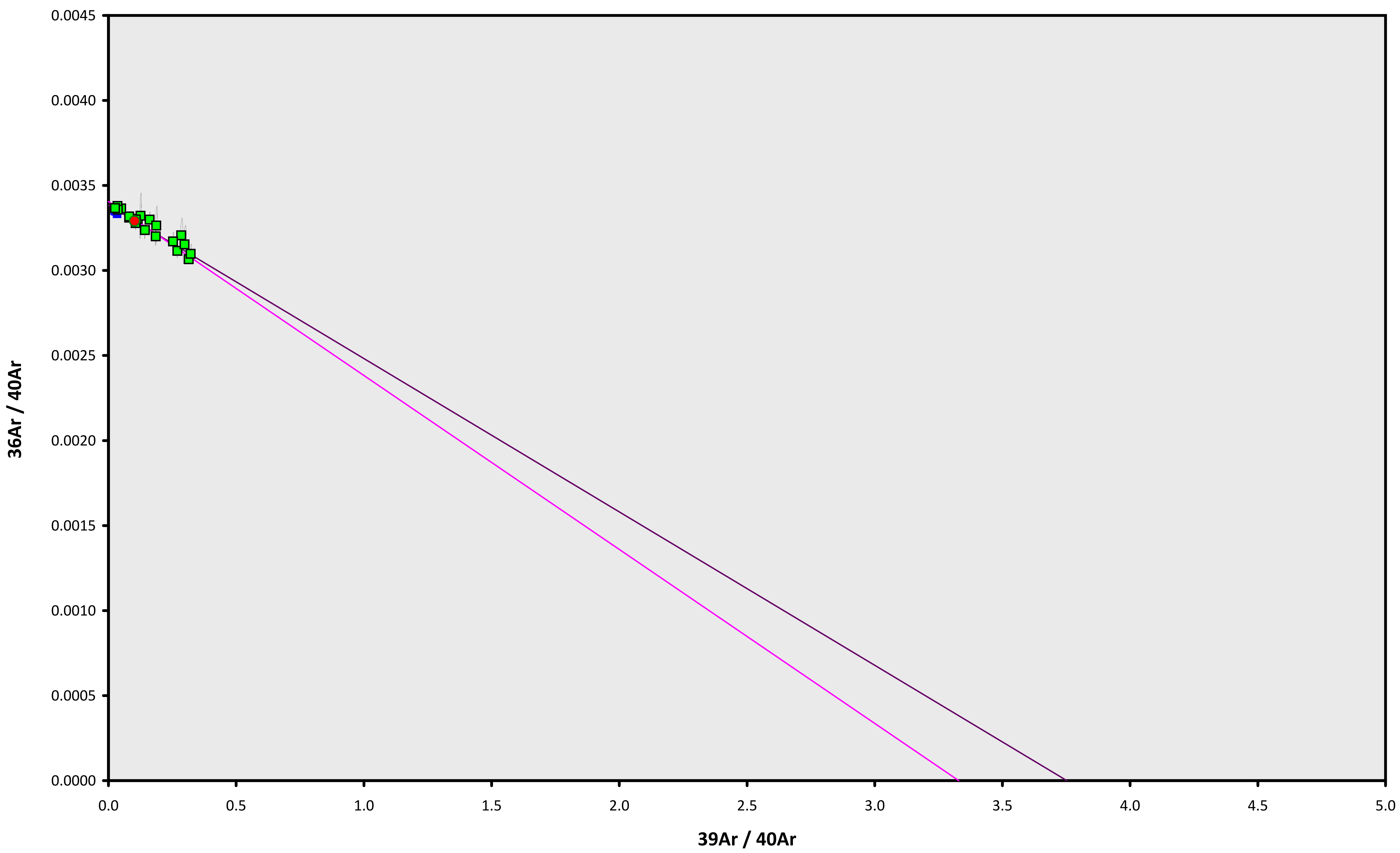

Ar-Ages in ka

WEIGHTED PLATEAU
791.6 ± 66.3
TOTAL FUSION
797.5 ± 61.2
NORMAL ISOCHRON
889.9 ± 80.5
INVERSE ISOCHRON
892.3 ± 78.2

MSWD (PROBABILITY)
1.19 (26%)

SPREADING FACTOR
8.9%

40AR/36AR INTERCEPT
293.6 ± 1.1

Sample Info

Groundmass
Gakkel Ridge
Dan Miggins

IRR = 17-OSU-05 (5B3-17)
J = 0.00164298 ± 0.00000136

Relative Abundances

Relative Abundances			36Ar [fA]	%1σ	37Ar [fA]	%1σ	38Ar [fA]	%1σ	39Ar [fA]	%1σ	40Ar [fA]	%1σ	40(r)/39(k) ± 2σ	Age ± 2σ (Ma)	40Ar(r) (%)	39Ar(k) (%)	K/Ca ± 2σ
17D19912	1.8 %	✓	0.2794132	0.415	220.2963	0.493	0.1256119	17.950	6.781008	0.341	72.13732	0.117	1.07879 ± 0.11001	3.19 ± 0.32	9.93	9.55	0.0130 ± 0.0002
17D19914	1.9 %	✓	0.1565925	0.546	144.2116	0.543	0.0644765	35.619	3.743050	0.612	38.07494	0.200	0.90904 ± 0.14951	2.69 ± 0.44	8.72	5.25	0.0109 ± 0.0002
17D19915	2.0 %	✓	0.1047519	0.607	98.2046	0.643	0.0238126	99.789	2.409762	0.898	25.25152	0.298	0.91188 ± 0.17888	2.70 ± 0.53	8.47	3.37	0.0103 ± 0.0002
17D19917	2.2 %	✓	0.0940330	0.618	89.0624	0.644	0.0059613	389.328	2.080783	1.066	22.49331	0.336	0.89894 ± 0.19216	2.66 ± 0.57	8.09	2.91	0.0098 ± 0.0002
17D19918	2.4 %	✓	0.0995555	0.627	105.3031	0.626	0.0451249	51.884	2.288354	0.965	22.87992	0.330	0.84247 ± 0.18680	2.49 ± 0.55	8.18	3.19	0.0091 ± 0.0002
17D19920	2.7 %	✓	0.0671518	0.814	59.7391	0.850	0.0025260	940.089	1.322071	1.640	15.96720	0.473	0.69692 ± 0.28624	2.06 ± 0.85	5.60	1.85	0.0092 ± 0.0003
17D19921	3.0 %	✓	0.1600562	0.502	177.7164	0.510	0.0509176	47.290	3.496485	0.634	35.74123	0.210	0.77977 ± 0.15443	2.31 ± 0.46	7.38	4.86	0.0082 ± 0.0001
17D19923	3.4 %	✓	0.0747852	0.733	82.1754	0.687	0.0032986	709.645	1.605541	1.412	16.65060	0.452	0.71762 ± 0.23859	2.12 ± 0.71	6.69	2.23	0.0081 ± 0.0003
17D19924	3.9 %	✓	0.2004270	0.457	242.8308	0.492	0.0830542	29.067	4.416243	0.502	44.10823	0.171	1.00352 ± 0.14047	2.97 ± 0.42	9.69	6.13	0.0075 ± 0.0001
17D19926	4.5 %	✓	0.1401084	0.540	179.1640	0.513	0.0450028	52.330	3.148396	0.718	29.96449	0.251	0.94653 ± 0.16416	2.80 ± 0.49	9.58	4.36	0.0073 ± 0.0001
17D19927	5.2 %	✓	0.1561427	0.524	189.7372	0.508	0.0506964	46.255	3.167271	0.720	33.54737	0.226	0.84074 ± 0.17507	2.49 ± 0.52	7.63	4.38	0.0069 ± 0.0001
17D19929	6.0 %	✓	0.1832488	0.470	260.8678	0.486	0.0693507	35.621	4.291004	0.519	37.25255	0.202	0.95467 ± 0.13918	2.82 ± 0.41	10.57	5.93	0.0068 ± 0.0001
17D19930	6.9 %	✓	0.1880328	0.483	264.3621	0.480	0.0743254	30.478	4.269191	0.520	38.80077	0.196	1.06118 ± 0.14624	3.14 ± 0.43	11.21	5.90	0.0067 ± 0.0001
17D19932	7.9 %	✓	0.2401244	0.409	338.6178	0.473	0.0791219	29.082	5.133579	0.445	49.03101	0.154	1.04107 ± 0.13437	3.08 ± 0.40	10.44	7.07	0.0062 ± 0.0001
17D19933	9.0 %	✓	0.2380751	0.420	289.5659	0.478	0.0687084	33.414	4.183548	0.550	51.06690	0.149	0.96109 ± 0.16399	2.84 ± 0.48	7.52	5.75	0.0059 ± 0.0001
17D19935	10.3 %	✓	0.5119273	0.359	452.4005	0.463	0.1753279	13.265	5.537169	0.391	119.44448	0.065	0.81991 ± 0.22021	2.42 ± 0.65	3.60	7.55	0.0050 ± 0.0001
17D19936	11.6 %	✓	0.2090445	0.449	215.0338	0.494	0.0434654	52.150	2.406382	0.914	46.31298	0.164	0.75587 ± 0.26598	2.24 ± 0.79	3.70	3.26	0.0045 ± 0.0001
17D19938	12.5 %	✓	0.3473718	0.401	320.2113	0.473	0.1010452	22.610	2.930018	0.760	80.04665	0.095	1.09132 ± 0.32192	3.23 ± 0.95	3.71	3.92	0.0037 ± 0.0001
17D19939	13.4 %	✓	0.2634349	0.423	280.3744	0.481	0.0640361	36.548	2.425637	0.949	57.50139	0.131	0.91273 ± 0.31835	2.70 ± 0.94	3.56	3.23	0.0034 ± 0.0001
17D19941	14.6 %		0.1513248	0.499	181.9386	0.517	0.0428559	53.194	1.523823	1.391	32.13637	0.234	1.38682 ± 0.35539	4.10 ± 1.05	6.07	2.02	0.0033 ± 0.0001
17D19942	16.0 %		0.1588818	0.523	188.3064	0.509	0.0202981	116.368	1.513552	1.368	33.79266	0.225	1.35214 ± 0.38904	4.00 ± 1.15	5.57	2.00	0.0032 ± 0.0001
17D19944	17.6 %		0.0980627	0.642	132.6079	0.556	0.0172775	135.271	1.116834	2.034	19.44887	0.389	1.03002 ± 0.40985	3.05 ± 1.21	5.46	1.48	0.0033 ± 0.0002
17D19945	19.3 %		0.1482302	0.522	198.4966	0.504	0.0145419	159.359	1.433901	1.610	29.79644	0.251	1.41481 ± 0.39343	4.18 ± 1.16	6.20	1.88	0.0028 ± 0.0001
17D19947	21.0 %		0.1684939	0.422	234.0215	0.492	0.0509163	46.843	1.488155	1.512	32.46700	0.231	1.02288 ± 0.36532	3.02 ± 1.08	4.21	1.92	0.0025 ± 0.0001
		Σ	4.4392707	0.101	4945.2456	0.110	1.3151563	8.703	72.711757	0.150	983.91419	0.038					

Information on Analysis and Constants Used in Calculations

Project = **O-CONNOR (16-22)**
Sample = **HLY0102-D24-5**
Material = **Groundmass**
Location = **Gakkel Ridge**
Region = **Artic Ocean**
Analyst = **Dan Miggins**
Irradiation = **17-OSU-01 (1C17-17)**
Position = **X: 0 | Y: 0 | Z/H: 26.42529 mm**
FCT-NM Age = **28.201 ± 0.023 Ma**
FCT-NM Reference = **Kuiper et al (2008)**
FCT-NM 40Ar/39Ar Ratio = **9.60352 ± 0.00855**
FCT-NM J-value = **0.00163663 ± 0.00000146**
Air Shot 40Ar/36Ar = **302.4660 ± 0.4204**
Air Shot MDF = **0.99423489 ± 0.00067193 (LIN)**
Experiment Type = **Incremental Heating**
Extraction Method = **Bulk Laser Heating**
Heating = **64 sec**
Isolation = **3.00 min**
Instrument = **ARGUS-VI-D**
Preferred Age = **Plateau Age**
Age Classification = **Crystallization Age**
IGSN = Undefined
Rock Class = **Igneous>Volcanic**
Lithology = **Basaltic Lava**
Lat-Lon = Undefined - Undefined

Age Equations = **Min et al. (2000)**
Negative Intensities = **Allowed**
Collector Calibrations = **36Ar**
Decay 40K = **5.530 ± 0.048 E-10 1/a**
Decay 39Ar = **2.940 ± 0.016 E-07 1/h**
Decay 37Ar = **8.230 ± 0.012 E-04 1/h**
Decay 36Cl = **2.257 ± 0.015 E-06 1/a**
Decay 40K(EC,β^+) = **0.580 ± 0.009 E-10 1/a**
Decay 40K(β^-) = **4.950 ± 0.043 E-10 1/a**
Atmospheric 40/36(a) = **295.50**
Atmospheric 38/36(a) = **0.1869**
Production 39/37(ca) = **0.0006425 ± 0.0000059**
Production 38/37(ca) = **0.0001800 ± 0.0000173**
Production 36/37(ca) = **0.0002703 ± 0.0000005**
Production 40/39(k) = **0.000607 ± 0.000059**
Production 38/39(k) = **0.012077 ± 0.000011**
Production 36/38(cl) = **262.80 ± 1.71**
Scaling Ratio K/Ca = **0.430**
Abundance Ratio 40K/K = **1.1700 ± 0.0100 E-04**
Atomic Weight K = **39.0983 ± 0.0001 g**

Results

Results	40(a)/36(a) ± 2σ	40(r)/39(k) ± 2σ	Age ± 2σ (Ma)	MSWD	39Ar(k) (%,n)	K/Ca ± 2σ
**Age Plateau**		0.94130 ± 0.05042 ± 5.36%	**2.78 ± 0.15 ± 5.36%**	1.66 4%	90.69 19	0.0060 ± 0.0010
			Full External Error ± 0.16	1.67	2σ Confidence Limit	
			Analytical Error ± 0.15	1.2872	Error Magnification	
**Total Fusion Age**		0.96445 ± 0.04198 ± 4.35%	**2.85 ± 0.12 ± 4.35%**		24	0.0060 ± 0.0000
			Full External Error ± 0.14			
			Analytical Error ± 0.12			
**Normal Isochron** Error Chron	**294.06 ± 3.86 ± 1.31%**	0.99080 ± 0.14823 ± 14.96%	**2.93 ± 0.44 ± 14.95%**	1.71 3%	90.69 19	
			Full External Error ± 0.44	1.69	2σ Confidence Limit	
			Analytical Error ± 0.44	1.3058	Error Magnification	
				4	Number of Iterations	
				0.0000025378	Convergence	
**Inverse Isochron** Error Chron	**294.05 ± 3.84 ± 1.30%**	0.99408 ± 0.14017 ± 14.10%	**2.94 ± 0.41 ± 14.09%**	1.69 4%	90.69 19	
			Full External Error ± 0.42	1.69	2σ Confidence Limit	
			Analytical Error ± 0.41	1.3012	Error Magnification	
**Notes**				3	Number of Iterations	
Mostly atmospheric				0.0000520800	Convergence	
				8%	Spreading Factor	

Incremental Heating			36Ar(a) [fA]	37Ar(ca) [fA]	38Ar(cl) [fA]	39Ar(k) [fA]	40Ar(r) [fA]	Age ± 2σ (Ma)	40Ar(r) (%)	39Ar(k) (%)	K/Ca ± 2σ
17D19912	1.8 %	✓	0.2198671	220.2963	0.0000000	6.639468	7.162573	3.19 ± 0.32	9.93	9.55	0.0130 ± 0.0002
17D19914	1.9 %	✓	0.1176121	144.2116	0.0000000	3.650394	3.318347	2.69 ± 0.44	8.72	5.25	0.0109 ± 0.0002
17D19915	2.0 %	✓	0.0782072	98.2046	0.0000000	2.346665	2.139877	2.70 ± 0.53	8.47	3.37	0.0103 ± 0.0002
17D19917	2.2 %	✓	0.0699595	89.0624	0.0000000	2.023560	1.819061	2.66 ± 0.57	8.09	2.91	0.0098 ± 0.0002
17D19918	2.4 %	✓	0.0710921	105.3031	0.0000000	2.220696	1.870860	2.49 ± 0.55	8.18	3.19	0.0091 ± 0.0002
17D19920	2.7 %	✓	0.0510044	59.7391	0.0000000	1.283689	0.894627	2.06 ± 0.85	5.60	1.85	0.0092 ± 0.0003
17D19921	3.0 %	✓	0.1120195	177.7164	0.0000000	3.382303	2.637419	2.31 ± 0.46	7.38	4.86	0.0082 ± 0.0001
17D19923	3.4 %	✓	0.0525732	82.1754	0.0000000	1.552743	1.114275	2.12 ± 0.71	6.69	2.23	0.0081 ± 0.0003
17D19924	3.9 %	✓	0.1347899	242.8308	0.0000000	4.260224	4.275229	2.97 ± 0.42	9.69	6.13	0.0075 ± 0.0001
17D19926	4.5 %	✓	0.0916804	179.1640	0.0000000	3.033283	2.871093	2.80 ± 0.49	9.58	4.36	0.0073 ± 0.0001
17D19927	5.2 %	✓	0.1048567	189.7372	0.0000000	3.045365	2.560349	2.49 ± 0.52	7.63	4.38	0.0069 ± 0.0001
17D19929	6.0 %	✓	0.1127363	260.8678	0.0000000	4.123397	3.936487	2.82 ± 0.41	10.57	5.93	0.0068 ± 0.0001
17D19930	6.9 %	✓	0.1165757	264.3621	0.0000000	4.099339	4.350156	3.14 ± 0.43	11.21	5.90	0.0067 ± 0.0001
17D19932	7.9 %	✓	0.1485960	338.6178	0.0000000	4.916017	5.117898	3.08 ± 0.40	10.44	7.07	0.0062 ± 0.0001
17D19933	9.0 %	✓	0.1598055	289.5659	0.0000000	3.997502	3.841953	2.84 ± 0.48	7.52	5.75	0.0059 ± 0.0001
17D19935	10.3 %	✓	0.3896435	452.4005	0.0000000	5.246502	4.301643	2.42 ± 0.65	3.60	7.55	0.0050 ± 0.0001
17D19936	11.6 %	✓	0.1509208	215.0338	0.0000000	2.268223	1.714488	2.24 ± 0.79	3.70	3.26	0.0045 ± 0.0001
17D19938	12.5 %	✓	0.2608187	320.2113	0.0000000	2.724282	2.973076	3.23 ± 0.95	3.71	3.92	0.0037 ± 0.0001
17D19939	13.4 %	✓	0.1876497	280.3744	0.0000000	2.245497	2.049531	2.70 ± 0.94	3.56	3.23	0.0034 ± 0.0001
17D19941	14.6 %		0.1021468	181.9386	0.0000000	1.406927	1.951150	4.10 ± 1.05	6.07	2.02	0.0033 ± 0.0001
17D19942	16.0 %		0.1079826	188.3064	0.0000000	1.392565	1.882949	4.00 ± 1.15	5.57	2.00	0.0032 ± 0.0001
17D19944	17.6 %		0.0622188	132.6079	0.0000000	1.031633	1.062606	3.05 ± 1.21	5.46	1.48	0.0033 ± 0.0002
17D19945	19.3 %		0.0945766	198.4966	0.0000000	1.306367	1.848259	4.18 ± 1.16	6.20	1.88	0.0028 ± 0.0001
17D19947	21.0 %		0.1052378	234.0215	0.0000000	1.337796	1.368411	3.02 ± 1.08	4.21	1.92	0.0025 ± 0.0001
		Σ	3.1025708	4945.2456	0.0000000	69.534437	67.062318				

Information on Analysis

Project = **O-CONNOR (16-22)**
Sample = **HLY0102-D24-5**
Material = **Groundmass**
Location = **Gakkel Ridge**
Region = **Artic Ocean**
Analyst = **Dan Miggins**
Irradiation = **17-OSU-01 (1C17-17)**
J = **0.00163663 ± 0.00000146**
FCT-NM = **28.201 ± 0.023 Ma**

Results	40(r)/39(k) ± 2σ		Age ± 2σ (Ma)		MSWD	39Ar(k) (%,n)	K/Ca ± 2σ
**Age Plateau**	0.94130	± 0.05042<br>± 5.36%	**2.78**	**± 0.15**<br>**± 5.36%**	1.66<br>4%	90.69<br>19	0.0060 ± 0.0010
		Full External Error		± 0.16	1.67	2σ Confidence Limit	
		Analytical Error		± 0.15	1.2872	Error Magnification	
**Total Fusion Age**	0.96445	± 0.04198<br>± 4.35%	**2.85**	**± 0.12**<br>**± 4.35%**		24	0.0060 ± 0.0000
		Full External Error		± 0.14			
		Analytical Error		± 0.12			

Normal Isochron			39(k)/36(a) ± 2σ	40(a+r)/36(a) ± 2σ	r.i.
17D19912	1.8 %	✓	30.20 ± 0.39	328.08 ± 3.66	0.8237
17D19914	1.9 %	✓	31.04 ± 0.61	323.71 ± 5.04	0.7418
17D19915	2.0 %	✓	30.01 ± 0.75	322.86 ± 5.78	0.6359
17D19917	2.2 %	✓	28.92 ± 0.81	321.50 ± 5.94	0.5754
17D19918	2.4 %	✓	31.24 ± 0.84	321.82 ± 6.26	0.6368
17D19920	2.7 %	✓	25.17 ± 1.02	313.04 ± 7.54	0.5040
17D19921	3.0 %	✓	30.19 ± 0.60	319.04 ± 4.99	0.7266
17D19923	3.4 %	✓	29.53 ± 1.07	316.69 ± 7.44	0.5502
17D19924	3.9 %	✓	31.61 ± 0.56	327.22 ± 4.87	0.7900
17D19926	4.5 %	✓	33.09 ± 0.76	326.82 ± 5.93	0.7305
17D19927	5.2 %	✓	29.04 ± 0.65	319.92 ± 5.46	0.7124
17D19929	6.0 %	✓	36.58 ± 0.72	330.42 ± 5.64	0.8132
17D19930	6.9 %	✓	35.16 ± 0.70	332.82 ± 5.73	0.8176
17D19932	7.9 %	✓	33.08 ± 0.57	329.94 ± 4.92	0.8244
17D19933	9.0 %	✓	25.01 ± 0.44	319.54 ± 4.41	0.7415
17D19935	10.3 %	✓	13.46 ± 0.17	306.54 ± 3.07	0.7600
17D19936	11.6 %	✓	15.03 ± 0.35	306.86 ± 4.14	0.5415
17D19938	12.5 %	✓	10.45 ± 0.21	306.90 ± 3.48	0.5552
17D19939	13.4 %	✓	11.97 ± 0.29	306.42 ± 3.94	0.5109
17D19941	14.6 %		13.77 ± 0.47	314.60 ± 5.15	0.4418
17D19942	16.0 %		12.90 ± 0.44	312.94 ± 5.26	0.4604
17D19944	17.6 %		16.58 ± 0.81	312.58 ± 7.09	0.4092
17D19945	19.3 %		13.81 ± 0.55	315.04 ± 5.72	0.4245
17D19947	21.0 %		12.71 ± 0.47	308.50 ± 4.81	0.3854

Results	40(a)/36(a) ± 2σ	40(r)/39(k) ± 2σ	Age ± 2σ (Ma)	MSWD
Normal Isochron<br>Error Chron	294.06 ± 3.86<br>± 1.31%	0.99080 ± 0.14823<br>± 14.96%	2.93 ± 0.44<br>± 14.95%	1.71<br>3%
			Full External Error ± 0.44	
			Analytical Error ± 0.44	

Statistics				
2σ Confidence Limit	1.69	Convergence	0.000002537820
Error Magnification	1.3058	Number of Iterations	4
Number of Data Points	19	Calculated Line	Weighted York-2

**Inverse Isochron**			**39(k)/40(a+r) ± 2σ**	**36(a)/40(a+r) ± 2σ**	**r.i.**
17D19912	1.8 %	✓	0.0920444 ± 0.0006775	0.00304807 ± 0.00003401	0.0665
17D19914	1.9 %	✓	0.0958795 ± 0.0012633	0.00308914 ± 0.00004805	0.0779
17D19915	2.0 %	✓	0.0929369 ± 0.0018032	0.00309730 ± 0.00005541	0.1026
17D19917	2.2 %	✓	0.0899677 ± 0.0020641	0.00311040 ± 0.00005750	0.1063
17D19918	2.4 %	✓	0.0970645 ± 0.0020346	0.00310737 ± 0.00006047	0.1071
17D19920	2.7 %	✓	0.0803993 ± 0.0028207	0.00319448 ± 0.00007689	0.1059
17D19921	3.0 %	✓	0.0946385 ± 0.0013041	0.00313436 ± 0.00004906	0.0819
17D19923	3.4 %	✓	0.0932598 ± 0.0028525	0.00315761 ± 0.00007420	0.1137
17D19924	3.9 %	✓	0.0965914 ± 0.0010604	0.00305607 ± 0.00004552	0.0715
17D19926	4.5 %	✓	0.1012355 ± 0.0015935	0.00305982 ± 0.00005556	0.0880
17D19927	5.2 %	✓	0.0907831 ± 0.0014228	0.00312580 ± 0.00005332	0.0762
17D19929	6.0 %	✓	0.1106951 ± 0.0012802	0.00302647 ± 0.00005163	0.0828
17D19930	6.9 %	✓	0.1056577 ± 0.0012194	0.00300466 ± 0.00005177	0.0773
17D19932	7.9 %	✓	0.1002695 ± 0.0009859	0.00303084 ± 0.00004524	0.0646
17D19933	9.0 %	✓	0.0782834 ± 0.0009332	0.00312948 ± 0.00004318	0.0538
17D19935	10.3 %	✓	0.0439254 ± 0.0003703	0.00326222 ± 0.00003270	0.0197
17D19936	11.6 %	✓	0.0489774 ± 0.0009649	0.00325881 ± 0.00004392	0.0404
17D19938	12.5 %	✓	0.0340344 ± 0.0005623	0.00325840 ± 0.00003698	0.0194
17D19939	13.4 %	✓	0.0390521 ± 0.0008095	0.00326347 ± 0.00004193	0.0257
17D19941	14.6 %		0.0437811 ± 0.0013368	0.00317863 ± 0.00005201	0.0438
17D19942	16.0 %		0.0412101 ± 0.0012417	0.00319553 ± 0.00005373	0.0399
17D19944	17.6 %		0.0530451 ± 0.0023738	0.00319920 ± 0.00007261	0.0596
17D19945	19.3 %		0.0438442 ± 0.0015677	0.00317418 ± 0.00005760	0.0390
17D19947	21.0 %		0.0412058 ± 0.0014028	0.00324146 ± 0.00005051	0.0404

**Results**	**40(a)/36(a) ± 2σ**		**40(r)/39(k) ± 2σ**		**Age ± 2σ (Ma)**		**MSWD**
**Inverse Isochron** Error Chron	**294.05**	**± 3.84** **± 1.30%**	0.99408	± 0.14017 ± 14.10%	**2.94**	**± 0.41** **± 14.09%**	1.69 4%
				Full External Error		± 0.42	
				Analytical Error		± 0.41	

**Statistics**				
2σ Confidence Limit	1.69	Convergence	0.0000520800
Error Magnification	1.3012	Number of Iterations	3
Number of Data Points	19	Calculated Line	Weighted York-2
Spreading Factor	7.6%		

**Degassing Patterns**			36Ar(a) [fA]	%1σ	36Ar(c) [fA]	%1σ	36Ar(ca) [fA]	%1σ	36Ar(cl) [fA]	%1σ	37Ar(ca) [fA]	%1σ	38Ar(a) [fA]	%1σ	38Ar(c) [fA]	%1σ	38Ar(k) [fA]	%1σ	38Ar(ca) [fA]	%1σ	38Ar(cl) [fA]	%1σ	39Ar(k) [fA]	%1σ	39Ar(ca) [fA]	%1σ	40Ar(r) [fA]	%1σ	40Ar(a) [fA]	%1σ	40Ar(c) [fA]	%1σ	40Ar(k) [fA]	%1σ
17D19912	1.8 %	✓	0.2198671	0.55	0.0000000	0.00	0.0595461	0.52	0.0000000	0.00	220.2963	0.49	0.0410932	0.55	0.0000000	0.00	0.0801849	0.36	0.0396533	9.64	0.0000000	0.00	6.639468	0.35	0.1415404	1.04	7.162573	5.09	64.97072	0.55	0.0000000	0.00	0.0040302	9.66
17D19914	1.9 %	✓	0.1176121	0.75	0.0000000	0.00	0.0389804	0.57	0.0000000	0.00	144.2116	0.54	0.0219817	0.75	0.0000000	0.00	0.0440858	0.63	0.0259581	9.65	0.0000000	0.00	3.650394	0.63	0.0926559	1.07	3.318347	8.20	34.75437	0.75	0.0000000	0.00	0.0022158	9.67
17D19915	2.0 %	✓	0.0782072	0.84	0.0000000	0.00	0.0265447	0.67	0.0000000	0.00	98.2046	0.64	0.0146169	0.84	0.0000000	0.00	0.0283407	0.93	0.0176768	9.65	0.0000000	0.00	2.346665	0.92	0.0630965	1.12	2.139877	9.76	23.11022	0.84	0.0000000	0.00	0.0014244	9.69
17D19917	2.2 %	✓	0.0699595	0.86	0.0000000	0.00	0.0240736	0.67	0.0000000	0.00	89.0624	0.64	0.0130754	0.86	0.0000000	0.00	0.0244385	1.10	0.0160312	9.65	0.0000000	0.00	2.023560	1.10	0.0572226	1.12	1.819061	10.63	20.67302	0.86	0.0000000	0.00	0.0012283	9.71
17D19918	2.4 %	✓	0.0710921	0.92	0.0000000	0.00	0.0284634	0.65	0.0000000	0.00	105.3031	0.63	0.0132871	0.92	0.0000000	0.00	0.0268194	1.00	0.0189546	9.65	0.0000000	0.00	2.220696	0.99	0.0676573	1.11	1.870860	11.04	21.00771	0.92	0.0000000	0.00	0.0013480	9.70
17D19920	2.7 %	✓	0.0510044	1.11	0.0000000	0.00	0.0161475	0.87	0.0000000	0.00	59.7391	0.85	0.0095327	1.11	0.0000000	0.00	0.0155031	1.69	0.0107530	9.67	0.0000000	0.00	1.283689	1.69	0.0383824	1.25	0.894627	20.47	15.07179	1.11	0.0000000	0.00	0.0007792	9.80
17D19921	3.0 %	✓	0.1120195	0.75	0.0000000	0.00	0.0480368	0.54	0.0000000	0.00	177.7164	0.51	0.0209364	0.75	0.0000000	0.00	0.0408481	0.66	0.0319890	9.64	0.0000000	0.00	3.382303	0.66	0.1141828	1.05	2.637419	9.88	33.10176	0.75	0.0000000	0.00	0.0020531	9.67
17D19923	3.4 %	✓	0.0525732	1.08	0.0000000	0.00	0.0222120	0.71	0.0000000	0.00	82.1754	0.69	0.0098259	1.08	0.0000000	0.00	0.0187525	1.46	0.0147916	9.65	0.0000000	0.00	1.552743	1.46	0.0527977	1.15	1.114275	16.56	15.53538	1.08	0.0000000	0.00	0.0009425	9.76
17D19924	3.9 %	✓	0.1347899	0.72	0.0000000	0.00	0.0656372	0.52	0.0000000	0.00	242.8308	0.49	0.0251922	0.72	0.0000000	0.00	0.0514507	0.53	0.0437095	9.64	0.0000000	0.00	4.260224	0.52	0.1560188	1.04	4.275229	6.98	39.83041	0.72	0.0000000	0.00	0.0025860	9.66
17D19926	4.5 %	✓	0.0916804	0.87	0.0000000	0.00	0.0484280	0.54	0.0000000	0.00	179.1640	0.51	0.0171351	0.87	0.0000000	0.00	0.0366330	0.75	0.0322495	9.64	0.0000000	0.00	3.033283	0.75	0.1151129	1.05	2.871093	8.64	27.09155	0.87	0.0000000	0.00	0.0018412	9.68
17D19927	5.2 %	✓	0.1048567	0.82	0.0000000	0.00	0.0512860	0.54	0.0000000	0.00	189.7372	0.51	0.0195977	0.82	0.0000000	0.00	0.0367789	0.76	0.0341527	9.64	0.0000000	0.00	3.045365	0.75	0.1219062	1.05	2.560349	10.38	30.98517	0.82	0.0000000	0.00	0.0018485	9.68
17D19929	6.0 %	✓	0.1127363	0.83	0.0000000	0.00	0.0705126	0.51	0.0000000	0.00	260.8678	0.49	0.0210704	0.83	0.0000000	0.00	0.0497983	0.55	0.0469562	9.64	0.0000000	0.00	4.123397	0.54	0.1676076	1.04	3.936487	7.27	33.31356	0.83	0.0000000	0.00	0.0025029	9.67
17D19930	6.9 %	✓	0.1165757	0.84	0.0000000	0.00	0.0714571	0.51	0.0000000	0.00	264.3621	0.48	0.0217880	0.84	0.0000000	0.00	0.0495077	0.55	0.0475852	9.64	0.0000000	0.00	4.099339	0.54	0.1698527	1.04	4.350156	6.87	34.44813	0.84	0.0000000	0.00	0.0024883	9.67
17D19932	7.9 %	✓	0.1485960	0.73	0.0000000	0.00	0.0915284	0.50	0.0000000	0.00	338.6178	0.47	0.0277726	0.73	0.0000000	0.00	0.0593707	0.48	0.0609512	9.64	0.0000000	0.00	4.916017	0.47	0.2175619	1.03	5.117898	6.44	43.91013	0.73	0.0000000	0.00	0.0029840	9.66
17D19933	9.0 %	✓	0.1598055	0.67	0.0000000	0.00	0.0782697	0.51	0.0000000	0.00	289.5659	0.48	0.0298676	0.67	0.0000000	0.00	0.0482778	0.58	0.0521219	9.64	0.0000000	0.00	3.997502	0.58	0.1860461	1.04	3.841953	8.51	47.22252	0.67	0.0000000	0.00	0.0024265	9.67
17D19935	10.3 %	✓	0.3896435	0.50	0.0000000	0.00	0.1222839	0.49	0.0000000	0.00	452.4005	0.46	0.0728244	0.50	0.0000000	0.00	0.0633620	0.43	0.0814321	9.64	0.0000000	0.00	5.246502	0.42	0.2906673	1.03	4.301643	13.42	115.13965	0.50	0.0000000	0.00	0.0031846	9.66
17D19936	11.6 %	✓	0.1509208	0.65	0.0000000	0.00	0.0581236	0.52	0.0000000	0.00	215.0338	0.49	0.0282071	0.65	0.0000000	0.00	0.0273933	0.98	0.0387061	9.64	0.0000000	0.00	2.268223	0.97	0.1381592	1.04	1.714488	17.57	44.59711	0.65	0.0000000	0.00	0.0013768	9.70
17D19938	12.5 %	✓	0.2608187	0.56	0.0000000	0.00	0.0865531	0.50	0.0000000	0.00	320.2113	0.47	0.0487470	0.56	0.0000000	0.00	0.0329012	0.83	0.0576380	9.64	0.0000000	0.00	2.724282	0.82	0.2057358	1.03	2.973076	14.73	77.07192	0.56	0.0000000	0.00	0.0016536	9.68
17D19939	13.4 %	✓	0.1876497	0.63	0.0000000	0.00	0.0757852	0.51	0.0000000	0.00	280.3744	0.48	0.0350717	0.63	0.0000000	0.00	0.0271189	1.03	0.0504674	9.64	0.0000000	0.00	2.245497	1.03	0.1801406	1.04	2.049531	17.41	55.45050	0.63	0.0000000	0.00	0.0013630	9.70
17D19941	14.6 %		0.1021468	0.78	0.0000000	0.00	0.0491780	0.54	0.0000000	0.00	181.9386	0.52	0.0190912	0.78	0.0000000	0.00	0.0169915	1.51	0.0327490	9.64	0.0000000	0.00	1.406927	1.51	0.1168956	1.06	1.951150	12.72	30.18437	0.78	0.0000000	0.00	0.0008540	9.77
17D19942	16.0 %		0.1079826	0.81	0.0000000	0.00	0.0508992	0.54	0.0000000	0.00	188.3064	0.51	0.0201820	0.81	0.0000000	0.00	0.0168180	1.49	0.0338952	9.64	0.0000000	0.00	1.392565	1.49	0.1209869	1.05	1.882949	14.31	31.90886	0.81	0.0000000	0.00	0.0008453	9.76
17D19944	17.6 %		0.0622188	1.07	0.0000000	0.00	0.0358439	0.58	0.0000000	0.00	132.6079	0.56	0.0116287	1.07	0.0000000	0.00	0.0124590	2.21	0.0238694	9.65	0.0000000	0.00	1.031633	2.20	0.0852006	1.08	1.062606	19.77	18.38564	1.07	0.0000000	0.00	0.0006262	9.90
17D19945	19.3 %		0.0945766	0.87	0.0000000	0.00	0.0536536	0.53	0.0000000	0.00	198.4966	0.50	0.0176764	0.87	0.0000000	0.00	0.0157770	1.77	0.0357294	9.64	0.0000000	0.00	1.306367	1.77	0.1275340	1.05	1.848259	13.79	27.94739	0.87	0.0000000	0.00	0.0007930	9.81
17D19947	21.0 %		0.1052378	0.74	0.0000000	0.00	0.0632560	0.52	0.0000000	0.00	234.0215	0.49	0.0196690	0.74	0.0000000	0.00	0.0161566	1.69	0.0421239	9.64	0.0000000	0.00	1.337796	1.69	0.1503588	1.04	1.368411	17.78	31.09778	0.74	0.0000000	0.00	0.0008120	9.80
		Σ	3.1025708	0.15	0.0000000	0.00	1.3366999	0.12	0.0000000	0.00	4945.2456	0.11	0.5798705	0.15	0.0000000	0.00	0.8397674	0.16	0.8901442	2.15	0.0000000	0.00	69.534437	0.16	3.1773203	0.23	67.062318	2.17	916.80967	0.15	0.0000000	0.00	0.0422074	2.21
		Σ							4.4392707	0.11	4945.2456	0.11									2.3097821	0.83			72.711757	0.15							983.91419	0.21

Additional Parameters			40Ar/39Ar	1σ	37Ar/39Ar	1σ	36Ar/39Ar	1σ	Time (days)	37Ar (decay)	39Ar (decay)	40Ar (moles)
17D19912	1.8 %	✓	10.638141	0.038345	32.487250	0.194653	0.041205	0.000221	142.657	16.780675	1.00100798	3.463E-12
17D19914	1.9 %	✓	10.172170	0.065453	38.527829	0.315186	0.041836	0.000343	142.671	16.785280	1.00100808	1.828E-12
17D19915	2.0 %	✓	10.478848	0.099202	40.752834	0.450326	0.043470	0.000471	142.678	16.787582	1.00100812	1.212E-12
17D19917	2.2 %	✓	10.810024	0.120843	42.802335	0.533137	0.045191	0.000557	142.692	16.792188	1.00100822	1.080E-12
17D19918	2.4 %	✓	9.998419	0.101950	46.016990	0.529207	0.043505	0.000500	142.699	16.794492	1.00100827	1.098E-12
17D19920	2.7 %	✓	12.077411	0.206116	45.185972	0.834496	0.050793	0.000930	142.713	16.799100	1.00100837	7.664E-13
17D19921	3.0 %	✓	10.222045	0.068256	50.827162	0.413512	0.045776	0.000370	142.719	16.801404	1.00100842	1.716E-12
17D19923	3.4 %	✓	10.370713	0.153798	51.182373	0.803939	0.046579	0.000741	142.733	16.806014	1.00100852	7.992E-13
17D19924	3.9 %	✓	9.987726	0.052950	54.985829	0.386336	0.045384	0.000308	142.740	16.808319	1.00100857	2.117E-12
17D19926	4.5 %	✓	9.517381	0.072357	56.906424	0.502002	0.044502	0.000400	142.754	16.812931	1.00100866	1.438E-12
17D19927	5.2 %	✓	10.591884	0.079960	59.905579	0.528096	0.049299	0.000439	142.761	16.815237	1.00100871	1.610E-12
17D19929	6.0 %	✓	8.681547	0.048356	60.794116	0.432296	0.042705	0.000299	142.775	16.819851	1.00100881	1.788E-12
17D19930	6.9 %	✓	9.088554	0.050466	61.923231	0.438112	0.044044	0.000312	142.782	16.822158	1.00100886	1.862E-12
17D19932	7.9 %	✓	9.551039	0.044969	65.961349	0.428134	0.046775	0.000283	142.796	16.826774	1.00100896	2.353E-12
17D19933	9.0 %	✓	12.206599	0.069498	69.215391	0.503966	0.056907	0.000394	142.803	16.829082	1.00100901	2.451E-12
17D19935	10.3 %	✓	21.571397	0.085476	81.702493	0.495219	0.092453	0.000491	142.817	16.833699	1.00100911	5.733E-12
17D19936	11.6 %	✓	19.245892	0.178638	89.359779	0.928110	0.086871	0.000884	142.824	16.836008	1.00100915	2.223E-12
17D19938	12.5 %	✓	27.319507	0.209128	109.286467	0.978062	0.118556	0.001018	142.838	16.840628	1.00100925	3.842E-12
17D19939	13.4 %	✓	23.705683	0.227026	115.587952	1.229392	0.108604	0.001128	142.844	16.842938	1.00100930	2.760E-12
17D19941	14.6 %		21.089311	0.297373	119.396168	1.771458	0.099306	0.001467	142.858	16.847559	1.00100940	1.543E-12
17D19942	16.0 %		22.326724	0.309539	124.413595	1.816177	0.104973	0.001537	142.865	16.849870	1.00100945	1.622E-12
17D19944	17.6 %		17.414293	0.360569	118.735586	2.503358	0.087804	0.001873	142.879	16.854493	1.00100955	9.335E-13
17D19945	19.3 %		20.779983	0.338589	138.431138	2.335230	0.103375	0.001749	142.886	16.856805	1.00100960	1.430E-12
17D19947	21.0 %		21.816954	0.333796	157.256180	2.501088	0.113223	0.001778	142.900	16.861430	1.00100969	1.558E-12

Procedure Blanks		36Ar ± 1σ (SE) [fA]	37Ar ± 1σ (SE) [fA]	38Ar ± 1σ (SE) [fA]	39Ar ± 1σ (SE) [fA]	40Ar ± 1σ (SE) [fA]
17D19912	1.8 %	0.0082831 ± 0.0001978	0.1461426 ± 0.0177309	0.0446348 ± 0.0164405	0.0219476 ± 0.0155046	2.3265650 ± 0.0729253
17D19914	1.9 %	0.0082242 ± 0.0001978	0.1428989 ± 0.0177309	0.0325587 ± 0.0164405	0.0129737 ± 0.0155046	2.2880449 ± 0.0729253
17D19915	2.0 %	0.0081930 ± 0.0001978	0.1415288 ± 0.0177309	0.0284308 ± 0.0164405	0.0104923 ± 0.0155046	2.2768103 ± 0.0729253
17D19917	2.2 %	0.0081296 ± 0.0001978	0.1393721 ± 0.0177309	0.0232602 ± 0.0164405	0.0087084 ± 0.0155046	2.2670750 ± 0.0729253
17D19918	2.4 %	0.0080983 ± 0.0001978	0.1386081 ± 0.0177309	0.0219472 ± 0.0164405	0.0090996 ± 0.0155046	2.2673545 ± 0.0729253
17D19920	2.7 %	0.0080384 ± 0.0001978	0.1377414 ± 0.0177309	0.0212482 ± 0.0164405	0.0117493 ± 0.0155046	2.2754258 ± 0.0729253
17D19921	3.0 %	0.0080104 ± 0.0001978	0.1376422 ± 0.0177309	0.0216389 ± 0.0164405	0.0137548 ± 0.0155046	2.2822119 ± 0.0729253
17D19923	3.4 %	0.0079598 ± 0.0001978	0.1380968 ± 0.0177309	0.0234010 ± 0.0164405	0.0185618 ± 0.0155046	2.2990389 ± 0.0729253
17D19924	3.9 %	0.0079376 ± 0.0001978	0.1386350 ± 0.0177309	0.0245961 ± 0.0164405	0.0211638 ± 0.0155046	2.3082883 ± 0.0729253
17D19926	4.5 %	0.0079003 ± 0.0001978	0.1402704 ± 0.0177309	0.0272324 ± 0.0164405	0.0263323 ± 0.0155046	2.3267474 ± 0.0729253
17D19927	5.2 %	0.0078853 ± 0.0001978	0.1413329 ± 0.0177309	0.0285444 ± 0.0164405	0.0287528 ± 0.0155046	2.3353799 ± 0.0729253
17D19929	6.0 %	0.0078632 ± 0.0001978	0.1438367 ± 0.0177309	0.0308915 ± 0.0164405	0.0329675 ± 0.0155046	2.3502749 ± 0.0729253
17D19930	6.9 %	0.0078559 ± 0.0001978	0.1452243 ± 0.0177309	0.0318446 ± 0.0164405	0.0346689 ± 0.0155046	2.3561743 ± 0.0729253
17D19932	7.9 %	0.0078488 ± 0.0001978	0.1481126 ± 0.0177309	0.0331628 ± 0.0164405	0.0370948 ± 0.0155046	2.3642363 ± 0.0729253
17D19933	9.0 %	0.0078487 ± 0.0001978	0.1495404 ± 0.0177309	0.0334928 ± 0.0164405	0.0377798 ± 0.0155046	2.3662498 ± 0.0729253
17D19935	10.3 %	0.0078546 ± 0.0001978	0.1521573 ± 0.0177309	0.0334658 ± 0.0164405	0.0380621 ± 0.0155046	2.3661372 ± 0.0729253
17D19936	11.6 %	0.0078601 ± 0.0001978	0.1532545 ± 0.0177309	0.0331206 ± 0.0164405	0.0376734 ± 0.0155046	2.3640761 ± 0.0729253
17D19938	12.5 %	0.0078748 ± 0.0001978	0.1547725 ± 0.0177309	0.0318557 ± 0.0164405	0.0359380 ± 0.0155046	2.3563745 ± 0.0729253
17D19939	13.4 %	0.0078834 ± 0.0001978	0.1550823 ± 0.0177309	0.0309949 ± 0.0164405	0.0346587 ± 0.0155046	2.3510133 ± 0.0729253
17D19941	14.6 %	0.0079009 ± 0.0001978	0.1545021 ± 0.0177309	0.0290229 ± 0.0164405	0.0315117 ± 0.0155046	2.3382359 ± 0.0729253
17D19942	16.0 %	0.0079089 ± 0.0001978	0.1534819 ± 0.0177309	0.0280177 ± 0.0164405	0.0297648 ± 0.0155046	2.3313130 ± 0.0729253
17D19944	17.6 %	0.0079211 ± 0.0001978	0.1496324 ± 0.0177309	0.0262930 ± 0.0164405	0.0262928 ± 0.0155046	2.3179000 ± 0.0729253
17D19945	19.3 %	0.0079241 ± 0.0001978	0.1466538 ± 0.0177309	0.0257266 ± 0.0164405	0.0247417 ± 0.0155046	2.3121174 ± 0.0729253
17D19947	21.0 %	0.0079209 ± 0.0001978	0.1381920 ± 0.0177309	0.0256272 ± 0.0164405	0.0225116 ± 0.0155046	2.3044366 ± 0.0729253

Intercept Values		36Ar ± 1σ (SE) [fA]	r2	Regression (type,n)	37Ar ± 1σ (SE) [fA]	r2	Regression (type,n)	38Ar ± 1σ (SE) [fA]	r2	Regression (type,n)	39Ar ± 1σ (SE) [fA]	r2	Regression (type,n)	40Ar ± 1σ (SE) [fA]	r2	Regression (type,n)
17D19912	1.8 %	0.2772437 ± 0.0008116	0.7008	EXP 150 of 150	12.755113 ± 0.017892	0.9406	EXP 150 of 150	0.0795290 ± 0.0150477	0.0011	EXP 150 of 150	6.7132537 ± 0.0163236	0.8428	EXP 149 of 150	74.463887 ± 0.042206	0.9721	EXP 150 of 150
17D19914	1.9 %	0.1589587 ± 0.0006840	0.3192	EXP 150 of 150	8.300275 ± 0.018186	0.8672	EXP 150 of 150	0.0311744 ± 0.0156541	0.0004	EXP 150 of 150	3.7047906 ± 0.0164444	0.4910	EXP 150 of 150	40.362980 ± 0.021643	0.9952	EXP 150 of 150
17D19915	2.0 %	0.1090262 ± 0.0005082	0.0829	EXP 150 of 150	5.607281 ± 0.019420	0.7247	EXP 150 of 150	0.0048927 ± 0.0167756	0.0108	EXP 150 of 150	2.3829906 ± 0.0148128	0.3096	EXP 150 of 150	27.528335 ± 0.018956	0.9969	EXP 150 of 150
17D19917	2.2 %	0.0986449 ± 0.0004600	0.0131	EXP 149 of 150	5.072828 ± 0.015993	0.7552	EXP 149 of 150	0.0173676 ± 0.0160009	0.0160	EXP 150 of 150	2.0580177 ± 0.0155975	0.2026	EXP 150 of 150	24.760386 ± 0.019643	0.9964	EXP 150 of 150
17D19918	2.4 %	0.1039296 ± 0.0005021	0.0700	EXP 150 of 150	6.023208 ± 0.019923	0.7568	EXP 150 of 150	0.0226575 ± 0.0162876	0.0056	EXP 150 of 150	2.2637952 ± 0.0154263	0.4027	EXP 150 of 150	25.147273 ± 0.019957	0.9962	EXP 150 of 150
17D19920	2.7 %	0.0726781 ± 0.0004544	0.0082	EXP 150 of 150	3.356932 ± 0.017803	0.5159	EXP 150 of 150	0.0187514 ± 0.0167533	0.0009	EXP 150 of 150	1.3013905 ± 0.0149165	0.0711	EXP 150 of 150	18.242621 ± 0.019523	0.9965	EXP 150 of 150
17D19921	3.0 %	0.1620791 ± 0.0006169	0.4994	EXP 150 of 150	10.257160 ± 0.016875	0.9219	EXP 150 of 150	0.0286917 ± 0.0172110	0.0025	EXP 150 of 150	3.4591098 ± 0.0154467	0.5573	EXP 150 of 150	38.023442 ± 0.018015	0.9957	EXP 150 of 150
17D19923	3.4 %	0.0799474 ± 0.0004472	0.0135	EXP 150 of 150	4.667100 ± 0.017405	0.6987	EXP 150 of 150	0.0266616 ± 0.0162821	0.0272	EXP 150 of 150	1.5761322 ± 0.0163037	0.0689	EXP 150 of 150	18.949638 ± 0.018543	0.9966	EXP 150 of 150
17D19924	3.9 %	0.2008669 ± 0.0006754	0.6203	EXP 150 of 150	14.058924 ± 0.020795	0.9350	EXP 150 of 150	0.0575006 ± 0.0172953	0.0014	EXP 150 of 150	4.3652444 ± 0.0153421	0.7090	EXP 150 of 150	46.416514 ± 0.019246	0.9934	EXP 150 of 150
17D19926	4.5 %	0.1427674 ± 0.0005942	0.3666	EXP 150 of 150	10.332015 ± 0.018023	0.9230	EXP 150 of 150	0.0172515 ± 0.0164799	0.0002	EXP 150 of 150	3.1007939 ± 0.0160892	0.5551	EXP 150 of 150	32.291236 ± 0.018162	0.9957	EXP 150 of 150
17D19927	5.2 %	0.1581869 ± 0.0006401	0.4772	EXP 150 of 150	10.947446 ± 0.018478	0.9211	EXP 150 of 150	0.0215676 ± 0.0163397	0.0000	EXP 150 of 150	3.1171205 ± 0.0163919	0.4833	EXP 150 of 150	35.882747 ± 0.020375	0.9940	EXP 150 of 150
17D19929	6.0 %	0.1842568 ± 0.0006424	0.6372	EXP 150 of 150	15.097833 ± 0.020343	0.9505	EXP 150 of 150	0.0376596 ± 0.0180543	0.0000	EXP 150 of 150	4.2290466 ± 0.0155161	0.7199	EXP 150 of 150	39.602829 ± 0.018637	0.9941	EXP 150 of 150
17D19930	6.9 %	0.1888546 ± 0.0006905	0.5443	EXP 149 of 150	15.298489 ± 0.017349	0.9611	EXP 150 of 150	0.0416240 ± 0.0152013	0.0038	EXP 150 of 150	4.2056796 ± 0.0153850	0.6550	EXP 149 of 150	41.156949 ± 0.021643	0.9923	EXP 150 of 150
17D19932	7.9 %	0.2389904 ± 0.0006729	0.7492	EXP 150 of 150	19.628099 ± 0.020122	0.9682	EXP 150 of 150	0.0450470 ± 0.0157171	0.0014	EXP 150 of 150	5.0618008 ± 0.0162023	0.7502	EXP 150 of 150	51.395248 ± 0.019459	0.9905	EXP 150 of 150
17D19933	9.0 %	0.2370177 ± 0.0007016	0.7406	EXP 149 of 150	16.759588 ± 0.018663	0.9634	EXP 150 of 150	0.0344235 ± 0.0156430	0.0006	EXP 150 of 150	4.1175036 ± 0.0165309	0.6492	EXP 150 of 150	53.433146 ± 0.021175	0.9873	EXP 150 of 150
17D19935	10.3 %	0.5006312 ± 0.0011247	0.8849	EXP 150 of 150	26.258411 ± 0.018803	0.9847	EXP 150 of 150	0.1398409 ± 0.0160677	0.0260	EXP 150 of 150	5.4616965 ± 0.0144284	0.8034	EXP 150 of 150	121.810615 ± 0.024870	0.9482	EXP 149 of 150
17D19936	11.6 %	0.2090844 ± 0.0006866	0.6797	EXP 150 of 150	12.398424 ± 0.017333	0.9414	EXP 150 of 150	0.0098437 ± 0.0152227	0.0006	EXP 150 of 150	2.3524507 ± 0.0152927	0.2727	EXP 150 of 150	48.677052 ± 0.020746	0.9882	EXP 150 of 150
17D19938	12.5 %	0.3422518 ± 0.0009557	0.8198	EXP 150 of 150	18.531067 ± 0.018737	0.9692	EXP 150 of 150	0.0680247 ± 0.0154810	0.0058	EXP 150 of 150	2.8742836 ± 0.0156311	0.4444	EXP 150 of 150	82.403024 ± 0.022655	0.5991	EXP 150 of 150
17D19939	13.4 %	0.2614635 ± 0.0007924	0.7583	EXP 150 of 150	16.203844 ± 0.019639	0.9570	EXP 150 of 150	0.0323030 ± 0.0162751	0.0010	EXP 150 of 150	2.3745899 ± 0.0167154	0.2900	EXP 150 of 150	59.852403 ± 0.018371	0.9794	EXP 150 of 150
17D19941	14.6 %	0.1535647 ± 0.0005729	0.5304	EXP 150 of 150	10.458106 ± 0.019799	0.8883	EXP 150 of 150	0.0133390 ± 0.0154103	0.0000	EXP 150 of 150	1.4820154 ± 0.0141948	0.1573	EXP 150 of 150	34.474610 ± 0.018328	0.9928	EXP 150 of 150
17D19942	16.0 %	0.1608472 ± 0.0006513	0.5303	EXP 150 of 150	10.829059 ± 0.018451	0.9190	EXP 150 of 150	0.0079536 ± 0.0165785	0.0088	EXP 150 of 150	1.4735606 ± 0.0134746	0.0698	EXP 150 of 150	36.123970 ± 0.021157	0.9900	EXP 150 of 150
17D19944	17.6 %	0.1023154 ± 0.0005110	0.2029	EXP 150 of 150	7.582299 ± 0.017553	0.8678	EXP 150 of 150	0.0092147 ± 0.0162301	0.0033	EXP 150 of 150	1.0829950 ± 0.0163701	0.0238	EXP 150 of 150	21.766773 ± 0.020052	0.9943	EXP 150 of 150
17D19945	19.3 %	0.1506092 ± 0.0006013	0.4537	EXP 150 of 150	11.425439 ± 0.018453	0.9223	EXP 150 of 150	0.0113523 ± 0.0159507	0.0051	EXP 150 of 150	1.3994708 ± 0.0168636	0.1212	EXP 149 of 150	32.108557 ± 0.017239	0.9944	EXP 150 of 150
17D19947	21.0 %	0.1701115 ± 0.0004793	0.7510	EXP 150 of 150	13.501218 ± 0.019403	0.9384	EXP 150 of 150	0.0247022 ± 0.0168974	0.0027	EXP 150 of 150	1.4555879 ± 0.0160726	0.1430	EXP 150 of 150	34.771439 ± 0.018193	0.9928	EXP 150 of 150

Project Info		Analyst	Irradiation	X-pos	Y-pos	Z/H-pos	Project	Experiment	Nmb
17D19912	1.8 %	Dan Miggins	17-OSU-01	0.00	0.00	26.43	Arctic\O-Connor (16-22)	17D19908	01
17D19914	1.9 %	Dan Miggins	17-OSU-01	0.00	0.00	26.43	Arctic\O-Connor (16-22)	17D19908	01
17D19915	2.0 %	Dan Miggins	17-OSU-01	0.00	0.00	26.43	Arctic\O-Connor (16-22)	17D19908	01
17D19917	2.2 %	Dan Miggins	17-OSU-01	0.00	0.00	26.43	Arctic\O-Connor (16-22)	17D19908	01
17D19918	2.4 %	Dan Miggins	17-OSU-01	0.00	0.00	26.43	Arctic\O-Connor (16-22)	17D19908	01
17D19920	2.7 %	Dan Miggins	17-OSU-01	0.00	0.00	26.43	Arctic\O-Connor (16-22)	17D19908	01
17D19921	3.0 %	Dan Miggins	17-OSU-01	0.00	0.00	26.43	Arctic\O-Connor (16-22)	17D19908	01
17D19923	3.4 %	Dan Miggins	17-OSU-01	0.00	0.00	26.43	Arctic\O-Connor (16-22)	17D19908	01
17D19924	3.9 %	Dan Miggins	17-OSU-01	0.00	0.00	26.43	Arctic\O-Connor (16-22)	17D19908	01
17D19926	4.5 %	Dan Miggins	17-OSU-01	0.00	0.00	26.43	Arctic\O-Connor (16-22)	17D19908	01
17D19927	5.2 %	Dan Miggins	17-OSU-01	0.00	0.00	26.43	Arctic\O-Connor (16-22)	17D19908	01
17D19929	6.0 %	Dan Miggins	17-OSU-01	0.00	0.00	26.43	Arctic\O-Connor (16-22)	17D19908	01
17D19930	6.9 %	Dan Miggins	17-OSU-01	0.00	0.00	26.43	Arctic\O-Connor (16-22)	17D19908	01
17D19932	7.9 %	Dan Miggins	17-OSU-01	0.00	0.00	26.43	Arctic\O-Connor (16-22)	17D19908	01
17D19933	9.0 %	Dan Miggins	17-OSU-01	0.00	0.00	26.43	Arctic\O-Connor (16-22)	17D19908	01
17D19935	10.3 %	Dan Miggins	17-OSU-01	0.00	0.00	26.43	Arctic\O-Connor (16-22)	17D19908	01
17D19936	11.6 %	Dan Miggins	17-OSU-01	0.00	0.00	26.43	Arctic\O-Connor (16-22)	17D19908	01
17D19938	12.5 %	Dan Miggins	17-OSU-01	0.00	0.00	26.43	Arctic\O-Connor (16-22)	17D19908	01
17D19939	13.4 %	Dan Miggins	17-OSU-01	0.00	0.00	26.43	Arctic\O-Connor (16-22)	17D19908	01
17D19941	14.6 %	Dan Miggins	17-OSU-01	0.00	0.00	26.43	Arctic\O-Connor (16-22)	17D19908	01
17D19942	16.0 %	Dan Miggins	17-OSU-01	0.00	0.00	26.43	Arctic\O-Connor (16-22)	17D19908	01
17D19944	17.6 %	Dan Miggins	17-OSU-01	0.00	0.00	26.43	Arctic\O-Connor (16-22)	17D19908	01
17D19945	19.3 %	Dan Miggins	17-OSU-01	0.00	0.00	26.43	Arctic\O-Connor (16-22)	17D19908	01
17D19947	21.0 %	Dan Miggins	17-OSU-01	0.00	0.00	26.43	Arctic\O-Connor (16-22)	17D19908	01

**Sample Parameters**		Sample	Material	Location	Standard Name	Standard (in Ma)	%1σ	Standard Reference	Standard 40Ar/39Ar	%1σ	J	%1σ	Air 40Ar/36Ar	%1σ	MDF (lin)	%1σ	Volume Ratio	Sensitivity (mol/volt)	Day	Month	Year	Hour	Min	Resist
17D19912	1.8 %	HLY0102-D24-5	Groundmass	Gakkel Ridge	FCT-NM (1C17-17)	28.201	0.082	Kuiper et al (2008)	9.60352	0.089	0.00163663	0.089	302.466	0.139	0.9942349	0.068	1	4.8E-14	11	JUN	2017	7	25	1
17D19914	1.9 %	HLY0102-D24-5	Groundmass	Gakkel Ridge	FCT-NM (1C17-17)	28.201	0.082	Kuiper et al (2008)	9.60352	0.089	0.00163663	0.089	302.466	0.139	0.9942349	0.068	1	4.8E-14	11	JUN	2017	7	45	1
17D19915	2.0 %	HLY0102-D24-5	Groundmass	Gakkel Ridge	FCT-NM (1C17-17)	28.201	0.082	Kuiper et al (2008)	9.60352	0.089	0.00163663	0.089	302.466	0.139	0.9942349	0.068	1	4.8E-14	11	JUN	2017	7	55	1
17D19917	2.2 %	HLY0102-D24-5	Groundmass	Gakkel Ridge	FCT-NM (1C17-17)	28.201	0.082	Kuiper et al (2008)	9.60352	0.089	0.00163663	0.089	302.466	0.139	0.9942349	0.068	1	4.8E-14	11	JUN	2017	8	15	1
17D19918	2.4 %	HLY0102-D24-5	Groundmass	Gakkel Ridge	FCT-NM (1C17-17)	28.201	0.082	Kuiper et al (2008)	9.60352	0.089	0.00163663	0.089	302.466	0.139	0.9942349	0.068	1	4.8E-14	11	JUN	2017	8	25	1
17D19920	2.7 %	HLY0102-D24-5	Groundmass	Gakkel Ridge	FCT-NM (1C17-17)	28.201	0.082	Kuiper et al (2008)	9.60352	0.089	0.00163663	0.089	302.466	0.139	0.9942349	0.068	1	4.8E-14	11	JUN	2017	8	45	1
17D19921	3.0 %	HLY0102-D24-5	Groundmass	Gakkel Ridge	FCT-NM (1C17-17)	28.201	0.082	Kuiper et al (2008)	9.60352	0.089	0.00163663	0.089	302.466	0.139	0.9942349	0.068	1	4.8E-14	11	JUN	2017	8	55	1
17D19923	3.4 %	HLY0102-D24-5	Groundmass	Gakkel Ridge	FCT-NM (1C17-17)	28.201	0.082	Kuiper et al (2008)	9.60352	0.089	0.00163663	0.089	302.466	0.139	0.9942349	0.068	1	4.8E-14	11	JUN	2017	9	15	1
17D19924	3.9 %	HLY0102-D24-5	Groundmass	Gakkel Ridge	FCT-NM (1C17-17)	28.201	0.082	Kuiper et al (2008)	9.60352	0.089	0.00163663	0.089	302.466	0.139	0.9942349	0.068	1	4.8E-14	11	JUN	2017	9	25	1
17D19926	4.5 %	HLY0102-D24-5	Groundmass	Gakkel Ridge	FCT-NM (1C17-17)	28.201	0.082	Kuiper et al (2008)	9.60352	0.089	0.00163663	0.089	302.466	0.139	0.9942349	0.068	1	4.8E-14	11	JUN	2017	9	45	1
17D19927	5.2 %	HLY0102-D24-5	Groundmass	Gakkel Ridge	FCT-NM (1C17-17)	28.201	0.082	Kuiper et al (2008)	9.60352	0.089	0.00163663	0.089	302.466	0.139	0.9942349	0.068	1	4.8E-14	11	JUN	2017	9	55	1
17D19929	6.0 %	HLY0102-D24-5	Groundmass	Gakkel Ridge	FCT-NM (1C17-17)	28.201	0.082	Kuiper et al (2008)	9.60352	0.089	0.00163663	0.089	302.466	0.139	0.9942349	0.068	1	4.8E-14	11	JUN	2017	10	15	1
17D19930	6.9 %	HLY0102-D24-5	Groundmass	Gakkel Ridge	FCT-NM (1C17-17)	28.201	0.082	Kuiper et al (2008)	9.60352	0.089	0.00163663	0.089	302.466	0.139	0.9942349	0.068	1	4.8E-14	11	JUN	2017	10	25	1
17D19932	7.9 %	HLY0102-D24-5	Groundmass	Gakkel Ridge	FCT-NM (1C17-17)	28.201	0.082	Kuiper et al (2008)	9.60352	0.089	0.00163663	0.089	302.466	0.139	0.9942349	0.068	1	4.8E-14	11	JUN	2017	10	45	1
17D19933	9.0 %	HLY0102-D24-5	Groundmass	Gakkel Ridge	FCT-NM (1C17-17)	28.201	0.082	Kuiper et al (2008)	9.60352	0.089	0.00163663	0.089	302.466	0.139	0.9942349	0.068	1	4.8E-14	11	JUN	2017	10	55	1
17D19935	10.3 %	HLY0102-D24-5	Groundmass	Gakkel Ridge	FCT-NM (1C17-17)	28.201	0.082	Kuiper et al (2008)	9.60352	0.089	0.00163663	0.089	302.466	0.139	0.9942349	0.068	1	4.8E-14	11	JUN	2017	11	15	1
17D19936	11.6 %	HLY0102-D24-5	Groundmass	Gakkel Ridge	FCT-NM (1C17-17)	28.201	0.082	Kuiper et al (2008)	9.60352	0.089	0.00163663	0.089	302.466	0.139	0.9942349	0.068	1	4.8E-14	11	JUN	2017	11	25	1
17D19938	12.5 %	HLY0102-D24-5	Groundmass	Gakkel Ridge	FCT-NM (1C17-17)	28.201	0.082	Kuiper et al (2008)	9.60352	0.089	0.00163663	0.089	302.466	0.139	0.9942349	0.068	1	4.8E-14	11	JUN	2017	11	45	1
17D19939	13.4 %	HLY0102-D24-5	Groundmass	Gakkel Ridge	FCT-NM (1C17-17)	28.201	0.082	Kuiper et al (2008)	9.60352	0.089	0.00163663	0.089	302.466	0.139	0.9942349	0.068	1	4.8E-14	11	JUN	2017	11	55	1
17D19941	14.6 %	HLY0102-D24-5	Groundmass	Gakkel Ridge	FCT-NM (1C17-17)	28.201	0.082	Kuiper et al (2008)	9.60352	0.089	0.00163663	0.089	302.466	0.139	0.9942349	0.068	1	4.8E-14	11	JUN	2017	12	15	1
17D19942	16.0 %	HLY0102-D24-5	Groundmass	Gakkel Ridge	FCT-NM (1C17-17)	28.201	0.082	Kuiper et al (2008)	9.60352	0.089	0.00163663	0.089	302.466	0.139	0.9942349	0.068	1	4.8E-14	11	JUN	2017	12	25	1
17D19944	17.6 %	HLY0102-D24-5	Groundmass	Gakkel Ridge	FCT-NM (1C17-17)	28.201	0.082	Kuiper et al (2008)	9.60352	0.089	0.00163663	0.089	302.466	0.139	0.9942349	0.068	1	4.8E-14	11	JUN	2017	12	45	1
17D19945	19.3 %	HLY0102-D24-5	Groundmass	Gakkel Ridge	FCT-NM (1C17-17)	28.201	0.082	Kuiper et al (2008)	9.60352	0.089	0.00163663	0.089	302.466	0.139	0.9942349	0.068	1	4.8E-14	11	JUN	2017	12	55	1
17D19947	21.0 %	HLY0102-D24-5	Groundmass	Gakkel Ridge	FCT-NM (1C17-17)	28.201	0.082	Kuiper et al (2008)	9.60352	0.089	0.00163663	0.089	302.466	0.139	0.9942349	0.068	1	4.8E-14	11	JUN	2017	13	15	1

**Irradiation Constants**		**40/36(a)**	**%1σ**	**40/36(c)**	**%1σ**	**38/36(a)**	**%1σ**	**38/36(c)**	**%1σ**	**39/37(ca)**	**%1σ**	**38/37(ca)**	**%1σ**	**36/37(ca)**	**%1σ**	**40/39(k)**	**%1σ**	**38/39(k)**	**%1σ**	**36/38(cl)**	**%1σ**	**K/Ca**	**%1σ**	**K/Cl**	**%1σ**	**Ca/Cl**	**%1σ**
17D19912	1.8 %	295.5	0	0.018	35	0.1869	0	1.493	3	0.000643	0.92	0.00018	9.63	0.00027	0.17	0.000607	9.65	0.012077	0.09	0	0	0.43	0	0	0	0	0
17D19914	1.9 %	295.5	0	0.018	35	0.1869	0	1.493	3	0.000643	0.92	0.00018	9.63	0.00027	0.17	0.000607	9.65	0.012077	0.09	0	0	0.43	0	0	0	0	0
17D19915	2.0 %	295.5	0	0.018	35	0.1869	0	1.493	3	0.000643	0.92	0.00018	9.63	0.00027	0.17	0.000607	9.65	0.012077	0.09	0	0	0.43	0	0	0	0	0
17D19917	2.2 %	295.5	0	0.018	35	0.1869	0	1.493	3	0.000643	0.92	0.00018	9.63	0.00027	0.17	0.000607	9.65	0.012077	0.09	0	0	0.43	0	0	0	0	0
17D19918	2.4 %	295.5	0	0.018	35	0.1869	0	1.493	3	0.000643	0.92	0.00018	9.63	0.00027	0.17	0.000607	9.65	0.012077	0.09	0	0	0.43	0	0	0	0	0
17D19920	2.7 %	295.5	0	0.018	35	0.1869	0	1.493	3	0.000643	0.92	0.00018	9.63	0.00027	0.17	0.000607	9.65	0.012077	0.09	0	0	0.43	0	0	0	0	0
17D19921	3.0 %	295.5	0	0.018	35	0.1869	0	1.493	3	0.000643	0.92	0.00018	9.63	0.00027	0.17	0.000607	9.65	0.012077	0.09	0	0	0.43	0	0	0	0	0
17D19923	3.4 %	295.5	0	0.018	35	0.1869	0	1.493	3	0.000643	0.92	0.00018	9.63	0.00027	0.17	0.000607	9.65	0.012077	0.09	0	0	0.43	0	0	0	0	0
17D19924	3.9 %	295.5	0	0.018	35	0.1869	0	1.493	3	0.000643	0.92	0.00018	9.63	0.00027	0.17	0.000607	9.65	0.012077	0.09	0	0	0.43	0	0	0	0	0
17D19926	4.5 %	295.5	0	0.018	35	0.1869	0	1.493	3	0.000643	0.92	0.00018	9.63	0.00027	0.17	0.000607	9.65	0.012077	0.09	0	0	0.43	0	0	0	0	0
17D19927	5.2 %	295.5	0	0.018	35	0.1869	0	1.493	3	0.000643	0.92	0.00018	9.63	0.00027	0.17	0.000607	9.65	0.012077	0.09	0	0	0.43	0	0	0	0	0
17D19929	6.0 %	295.5	0	0.018	35	0.1869	0	1.493	3	0.000643	0.92	0.00018	9.63	0.00027	0.17	0.000607	9.65	0.012077	0.09	0	0	0.43	0	0	0	0	0
17D19930	6.9 %	295.5	0	0.018	35	0.1869	0	1.493	3	0.000643	0.92	0.00018	9.63	0.00027	0.17	0.000607	9.65	0.012077	0.09	0	0	0.43	0	0	0	0	0
17D19932	7.9 %	295.5	0	0.018	35	0.1869	0	1.493	3	0.000643	0.92	0.00018	9.63	0.00027	0.17	0.000607	9.65	0.012077	0.09	0	0	0.43	0	0	0	0	0
17D19933	9.0 %	295.5	0	0.018	35	0.1869	0	1.493	3	0.000643	0.92	0.00018	9.63	0.00027	0.17	0.000607	9.65	0.012077	0.09	0	0	0.43	0	0	0	0	0
17D19935	10.3 %	295.5	0	0.018	35	0.1869	0	1.493	3	0.000643	0.92	0.00018	9.63	0.00027	0.17	0.000607	9.65	0.012077	0.09	0	0	0.43	0	0	0	0	0
17D19936	11.6 %	295.5	0	0.018	35	0.1869	0	1.493	3	0.000643	0.92	0.00018	9.63	0.00027	0.17	0.000607	9.65	0.012077	0.09	0	0	0.43	0	0	0	0	0
17D19938	12.5 %	295.5	0	0.018	35	0.1869	0	1.493	3	0.000643	0.92	0.00018	9.63	0.00027	0.17	0.000607	9.65	0.012077	0.09	0	0	0.43	0	0	0	0	0
17D19939	13.4 %	295.5	0	0.018	35	0.1869	0	1.493	3	0.000643	0.92	0.00018	9.63	0.00027	0.17	0.000607	9.65	0.012077	0.09	0	0	0.43	0	0	0	0	0
17D19941	14.6 %	295.5	0	0.018	35	0.1869	0	1.493	3	0.000643	0.92	0.00018	9.63	0.00027	0.17	0.000607	9.65	0.012077	0.09	0	0	0.43	0	0	0	0	0
17D19942	16.0 %	295.5	0	0.018	35	0.1869	0	1.493	3	0.000643	0.92	0.00018	9.63	0.00027	0.17	0.000607	9.65	0.012077	0.09	0	0	0.43	0	0	0	0	0
17D19944	17.6 %	295.5	0	0.018	35	0.1869	0	1.493	3	0.000643	0.92	0.00018	9.63	0.00027	0.17	0.000607	9.65	0.012077	0.09	0	0	0.43	0	0	0	0	0
17D19945	19.3 %	295.5	0	0.018	35	0.1869	0	1.493	3	0.000643	0.92	0.00018	9.63	0.00027	0.17	0.000607	9.65	0.012077	0.09	0	0	0.43	0	0	0	0	0
17D19947	21.0 %	295.5	0	0.018	35	0.1869	0	1.493	3	0.000643	0.92	0.00018	9.63	0.00027	0.17	0.000607	9.65	0.012077	0.09	0	0	0.43	0	0	0	0	0

17D19908.AGE >>> HLY0102-D24-5 >>> ARCTIC | O-CONNOR (16-22) PROJECT

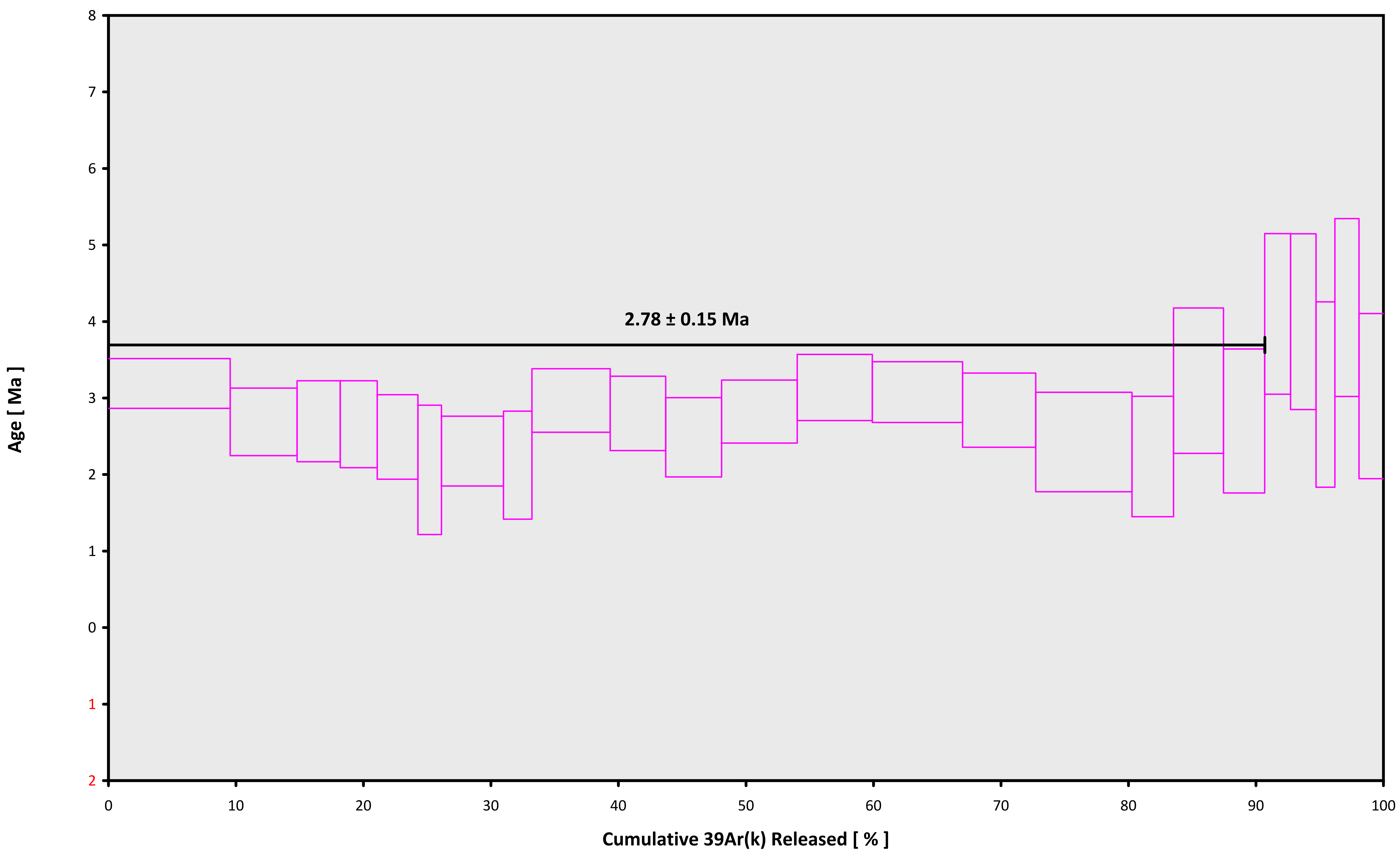

Ar-Ages in Ma

WEIGHTED PLATEAU
2.78 ± 0.15
TOTAL FUSION
2.85 ± 0.12
NORMAL ISOCHRON
2.93 ± 0.44
INVERSE ISOCHRON
2.94 ± 0.41

MSWD (PROBABILITY)
1.66 (4%)

Sample Info

Groundmass
Gakkel Ridge
Dan Miggins

IRR = 17-OSU-01 (1C17-17)
J = 0.00163663 ± 0.00000146

17D19908.AGE >>> HLY0102-D24-5 >>> ARCTIC | O-CONNOR (16-22) PROJECT

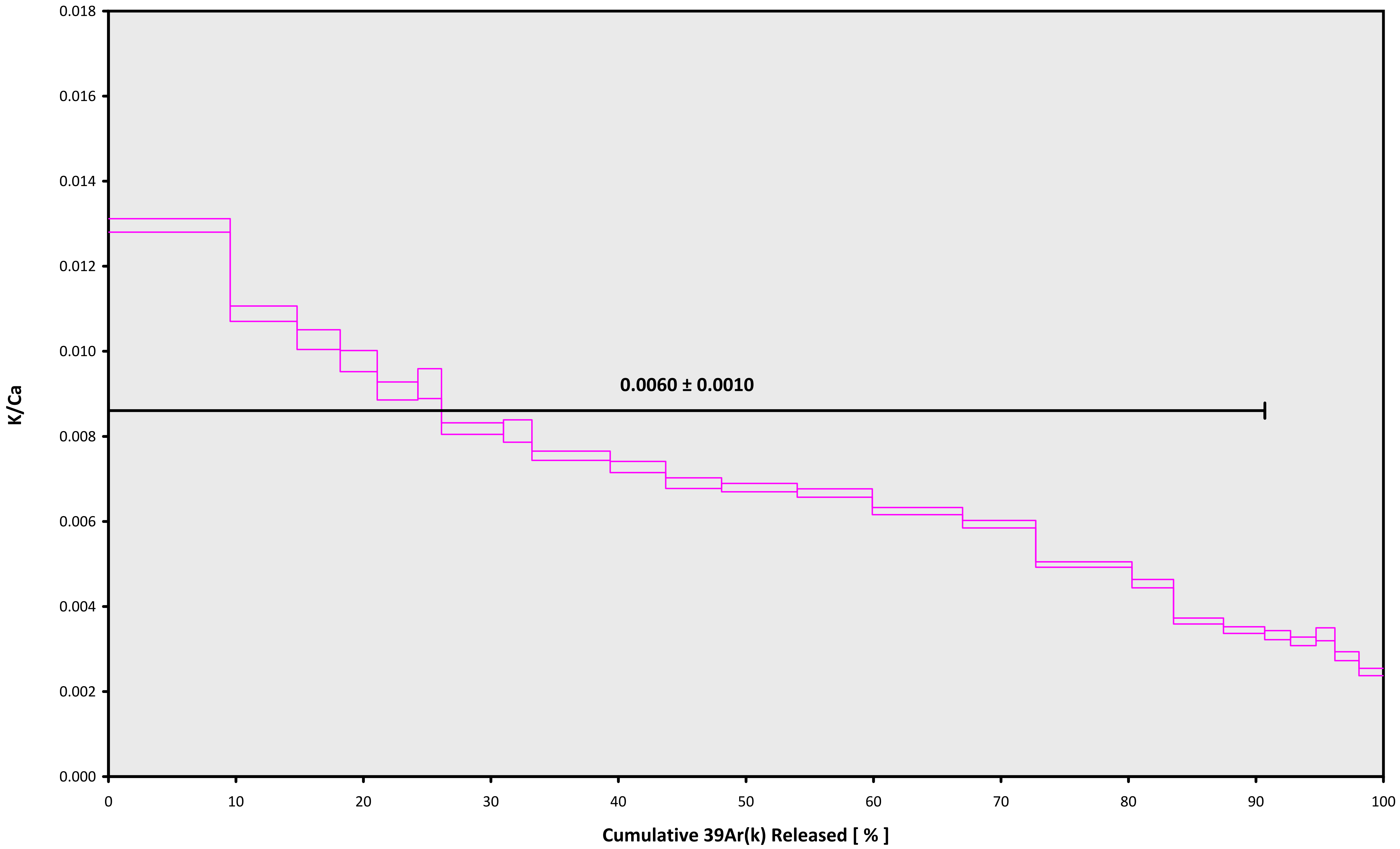

Ar-Ages in Ma

WEIGHTED PLATEAU
2.78 ± 0.15
TOTAL FUSION
2.85 ± 0.12
NORMAL ISOCHRON
2.93 ± 0.44
INVERSE ISOCHRON
2.94 ± 0.41

Sample Info

Groundmass
Gakkel Ridge
Dan Miggins

IRR = 17-OSU-01 (1C17-17)
J = 0.00163663 ± 0.00000146

17D19908.AGE >>> HLY0102-D24-5 >>> ARCTIC | O-CONNOR (16-22) PROJECT

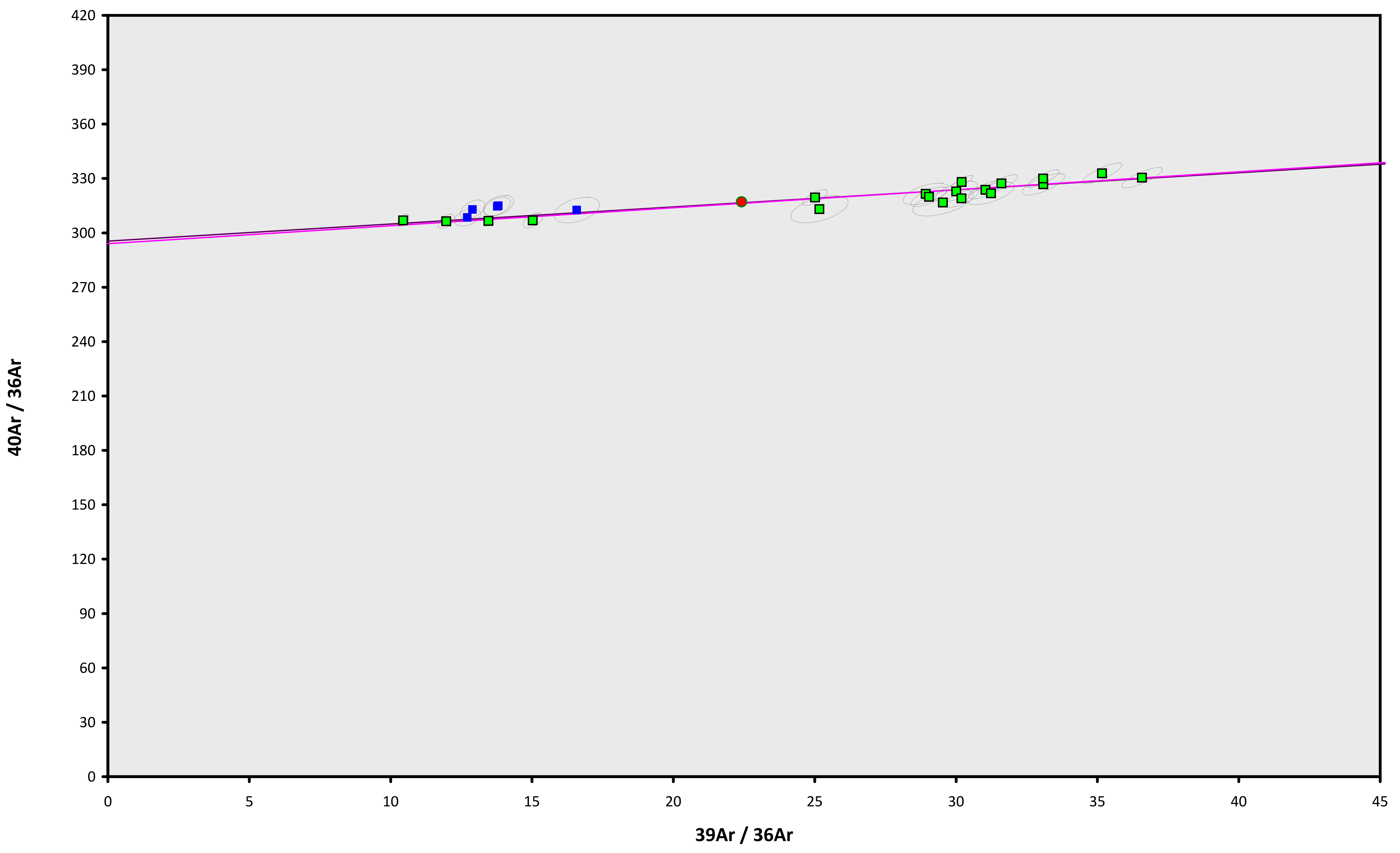

Ar-Ages in Ma

WEIGHTED PLATEAU
2.78 ± 0.15
TOTAL FUSION
2.85 ± 0.12
NORMAL ISOCHRON
2.93 ± 0.44
INVERSE ISOCHRON
2.94 ± 0.41

MSWD (PROBABILITY)
1.71 (3%)

40AR/36AR INTERCEPT
294.1 ± 3.9

Sample Info

Groundmass
Gakkel Ridge
Dan Miggins

IRR = 17-OSU-01 (1C17-17)
J = 0.00163663 ± 0.00000146

17D19908.AGE >>> HLY0102-D24-5 >>> ARCTIC | O-CONNOR (16-22) PROJECT

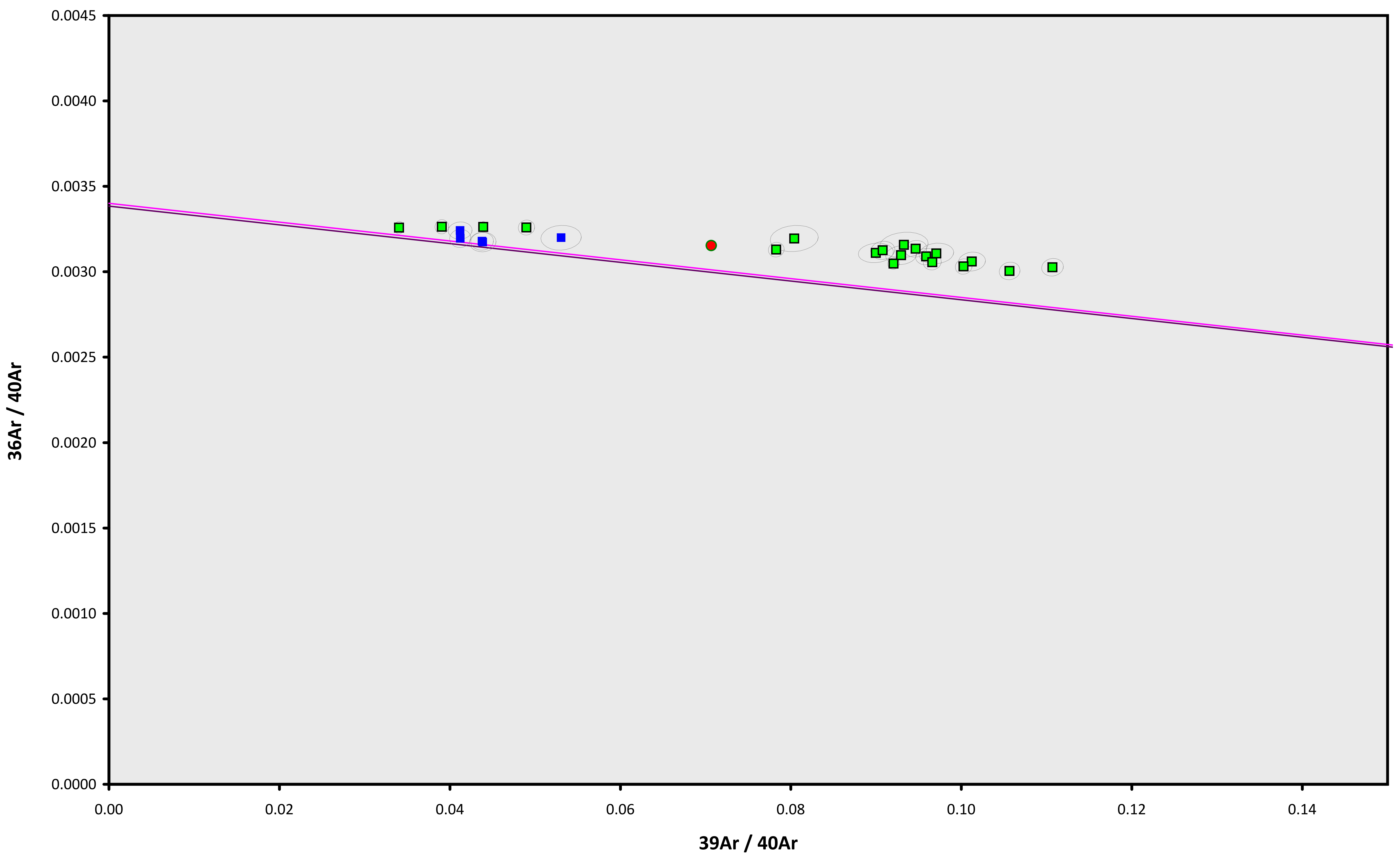

Ar-Ages in Ma

WEIGHTED PLATEAU
2.78 ± 0.15
TOTAL FUSION
2.85 ± 0.12
NORMAL ISOCHRON
2.93 ± 0.44
INVERSE ISOCHRON
2.94 ± 0.41

MSWD (PROBABILITY)
1.69 (4%)

SPREADING FACTOR
7.6%

40AR/36AR INTERCEPT
294.1 ± 3.8

Sample Info

Groundmass
Gakkel Ridge
Dan Miggins

IRR = 17-OSU-01 (1C17-17)
J = 0.00163663 ± 0.00000146

Relative Abundances			36Ar [fA]	%1σ	37Ar [fA]	%1σ	38Ar [fA]	%1σ	39Ar [fA]	%1σ	40Ar [fA]	%1σ	40(r)/39(k) ± 2σ	Age ± 2σ (Ma)	40Ar(r) (%)	39Ar(k) (%)	K/Ca ± 2σ
17D30450	1.8 %	✓	28.26268	0.282	88.9284	0.564	6.135697	0.416	59.48418	0.081	8446.197	0.010	1.12599 ± 0.94144	3.02 ± 2.52	0.79	20.73	0.2874 ± 0.0033
17D30452	1.9 %	✓	15.99678	0.284	96.7830	0.535	3.602963	0.711	40.54459	0.092	4776.684	0.016	0.93134 ± 0.78561	2.50 ± 2.10	0.79	14.12	0.1799 ± 0.0020
17D30453	2.0 %	✓	7.77804	0.288	98.8134	0.536	1.790684	1.329	24.15560	0.122	2315.971	0.034	0.66016 ± 0.65189	1.77 ± 1.75	0.69	8.40	0.1048 ± 0.0012
17D30455	2.2 %	✓	5.09600	0.292	90.0623	0.541	1.186561	2.008	16.83022	0.159	1521.431	0.051	0.98442 ± 0.62293	2.64 ± 1.67	1.09	5.85	0.0801 ± 0.0009
17D30456	2.4 %	✓	2.13501	0.325	46.1535	0.824	0.475966	5.146	6.74044	0.342	634.794	0.122	0.74078 ± 0.73578	1.99 ± 1.97	0.78	2.34	0.0625 ± 0.0011
17D30458	2.7 %	✓	3.78616	0.300	100.6258	0.546	0.885757	2.640	12.84158	0.190	1125.678	0.069	0.80419 ± 0.62357	2.16 ± 1.67	0.91	4.46	0.0546 ± 0.0006
17D30459	3.0 %	✓	1.81658	0.336	54.9562	0.744	0.433062	5.542	5.78821	0.404	539.166	0.144	0.78847 ± 0.76071	2.11 ± 2.04	0.84	2.01	0.0450 ± 0.0008
17D30461	3.4 %	✓	2.56361	0.317	84.2594	0.568	0.608394	3.881	7.93925	0.302	760.089	0.102	0.77919 ± 0.72694	2.09 ± 1.95	0.81	2.75	0.0402 ± 0.0005
17D30462	3.9 %	✓	2.12718	0.327	71.7106	0.621	0.510566	4.726	6.35427	0.362	631.412	0.123	0.94543 ± 0.78325	2.53 ± 2.10	0.94	2.20	0.0378 ± 0.0005
17D30464	4.5 %	✓	3.72089	0.301	132.2924	0.487	0.864695	2.873	10.78709	0.226	1102.086	0.070	0.80279 ± 0.73432	2.15 ± 1.97	0.78	3.73	0.0348 ± 0.0004
17D30465	5.2 %	✓	3.12035	0.306	115.5989	0.511	0.720424	3.182	8.16659	0.289	923.250	0.084	0.81764 ± 0.83079	2.19 ± 2.23	0.72	2.82	0.0301 ± 0.0004
17D30467	6.0 %	✓	3.72280	0.300	134.7389	0.483	0.848612	2.806	9.34171	0.254	1101.885	0.070	0.86662 ± 0.84785	2.32 ± 2.27	0.73	3.23	0.0295 ± 0.0003
17D30468	6.9 %	✓	4.71965	0.295	165.5648	0.458	1.049797	2.154	10.66471	0.223	1394.890	0.056	0.72952 ± 0.92296	1.96 ± 2.47	0.55	3.68	0.0274 ± 0.0003
17D30470	7.9 %	✓	3.14691	0.305	142.4583	0.474	0.727195	3.326	8.73391	0.254	926.465	0.084	0.47495 ± 0.78211	1.27 ± 2.10	0.44	3.02	0.0261 ± 0.0003
17D30471	9.0 %	✓	2.26266	0.321	113.8209	0.503	0.553649	4.385	6.85133	0.326	665.561	0.116	0.48548 ± 0.76092	1.30 ± 2.04	0.49	2.36	0.0256 ± 0.0003
17D30473	10.3 %	✓	3.19119	0.307	119.7889	0.499	0.739070	3.233	7.01909	0.328	942.927	0.082	0.80819 ± 0.99220	2.17 ± 2.66	0.60	2.42	0.0249 ± 0.0003
17D30474	11.6 %	✓	5.97627	0.291	177.2056	0.456	1.290408	1.881	8.33425	0.264	1766.662	0.044	0.91652 ± 1.48210	2.46 ± 3.97	0.43	2.87	0.0199 ± 0.0002
17D30476	12.5 %	✓	6.11159	0.290	186.4005	0.454	1.287010	1.828	6.75683	0.344	1803.138	0.043	0.69233 ± 1.87012	1.86 ± 5.01	0.25	2.32	0.0153 ± 0.0002
17D30477	13.4 %	✓	2.83321	0.313	89.5608	0.556	0.639141	3.855	4.28193	0.518	837.930	0.092	1.04533 ± 1.47773	2.80 ± 3.96	0.53	1.47	0.0203 ± 0.0003
17D30479	14.6 %	✓	4.31549	0.297	140.7700	0.478	0.911594	2.724	5.37480	0.437	1272.802	0.061	0.67291 ± 1.70146	1.80 ± 4.56	0.28	1.84	0.0161 ± 0.0002
17D30480	16.0 %	✓	4.92831	0.296	149.6624	0.469	1.056363	2.259	5.68550	0.398	1453.043	0.053	0.47735 ± 1.82607	1.28 ± 4.90	0.18	1.95	0.0161 ± 0.0002
17D30482	17.6 %	✓	9.12037	0.286	293.8776	0.428	1.878130	1.385	8.65498	0.261	2694.394	0.029	1.37880 ± 2.15627	3.70 ± 5.77	0.43	2.95	0.0124 ± 0.0001
17D30483	19.3 %	✓	3.43376	0.305	121.8653	0.497	0.714295	3.525	3.60272	0.641	1010.972	0.077	0.52325 ± 2.08583	1.40 ± 5.59	0.18	1.23	0.0124 ± 0.0002
17D30485	21.0 %	✓	3.60598	0.302	132.5027	0.483	0.763164	3.188	3.60632	0.615	1060.102	0.073	0.20663 ± 2.16955	0.55 ± 5.82	0.07	1.23	0.0114 ± 0.0002
		Σ	133.77147	0.083	2948.4007	0.107	29.673197	0.400	288.54011	0.044	39707.526	0.010					

Information on Analysis and Constants Used in Calculations

Project = **O-CONNOR (16-22)**
Sample = **PS59-244-002**
Material = **Groundmass**
Location = **Gakkel Ridge**
Region = **Artic Ocean**
Analyst = **Dan Miggins**
Irradiation = **17-OSU-05 (5A41-17)**
Position = **X: 0 | Y: 0 | Z/H: 60.23046 mm**
FCT-NM Age = **28.201 ± 0.023 Ma**
FCT-NM Reference = **Kuiper et al (2008)**
FCT-NM 40Ar/39Ar Ratio = **10.59431 ± 0.01112**
FCT-NM J-value = **0.00148357 ± 0.00000156**
Air Shot 40Ar/36Ar = **302.6380 ± 0.4540**
Air Shot MDF = **0.99409590 ± 0.00068555 (LIN)**
Experiment Type = **Incremental Heating**
Extraction Method = **Bulk Laser Heating**
Heating = **64 sec**
Isolation = **3.00 min**
Instrument = **ARGUS-VI-D**
Preferred Age = **Plateau Age**
Age Classification = **Crystallization Age**
IGSN = Undefined
Rock Class = **Igneous>Volcanic**
Lithology = **Basaltic Lava**
Lat-Lon = Undefined - Undefined

Age Equations = **Min et al. (2000)**
Negative Intensities = **Allowed**
Collector Calibrations = **36Ar**
Decay 40K = **5.530 ± 0.048 E-10 1/a**
Decay 39Ar = **2.940 ± 0.016 E-07 1/h**
Decay 37Ar = **8.230 ± 0.012 E-04 1/h**
Decay 36Cl = **2.257 ± 0.015 E-06 1/a**
Decay 40K(EC,β^+) = **0.580 ± 0.009 E-10 1/a**
Decay 40K(β^-) = **4.950 ± 0.043 E-10 1/a**
Atmospheric 40/36(a) = **296.73 ± 0.53**
Atmospheric 38/36(a) = **0.1869**
Production 39/37(ca) = **0.0006425 ± 0.0000059**
Production 38/37(ca) = **0.0001800 ± 0.0000173**
Production 36/37(ca) = **0.0002703 ± 0.0000005**
Production 40/39(k) = **0.000607 ± 0.000059**
Production 38/39(k) = **0.012077 ± 0.000011**
Production 36/38(cl) = **262.80 ± 1.71**
Scaling Ratio K/Ca = **0.430**
Abundance Ratio 40K/K = **1.1700 ± 0.0100 E-04**
Atomic Weight K = **39.0983 ± 0.0001 g**

Results

Results	40(a)/36(a) ± 2σ	40(r)/39(k) ± 2σ	Age ± 2σ (Ma)	MSWD	39Ar(k) (%,n)	K/Ca ± 2σ
**Age Plateau**		0.79033 ± 0.18228	**2.12** ± 0.49	0.16	100.00	0.0194 ± 0.0050
Overestimated Error		± 23.06%	± 23.05%	100%	24	
			Full External Error ± 0.49	1.59	2σ Confidence Limit	
			Analytical Error ± 0.49	1.0000	Error Magnification	
**Total Fusion Age**		0.87172 ± 0.27061	**2.34** ± 0.73		24	0.0418 ± 0.0001
		± 31.04%	± 31.02%			
			Full External Error ± 0.73			
			Analytical Error ± 0.73			
**Normal Isochron**	**296.73** ± 1.06	0.79302 ± 0.44320	**2.13** ± 1.19	0.22	100.00	
Overestimated Error	± 0.36%	± 55.89%	± 55.86%	100%	24	
			Full External Error ± 1.19	1.60	2σ Confidence Limit	
			Analytical Error ± 1.19	1.0000	Error Magnification	
				2	Number of Iterations	
				0.0000040247	Convergence	
**Inverse Isochron**	**296.73** ± 1.06	0.79331 ± 0.37678	**2.13** ± 1.01	0.22	100.00	
Overestimated Error	± 0.36%	± 47.49%	± 47.47%	100%	24	
			Full External Error ± 1.01	1.60	2σ Confidence Limit	
			Analytical Error ± 1.01	1.0000	Error Magnification	
				3	Number of Iterations	
				0.0000052755	Convergence	
				1%	Spreading Factor	

Notes

Excess Initial 40Ar/36Ar = 296.73 ± 0.18 (%SD).

**Incremental Heating**			36Ar(a) [fA]	37Ar(ca) [fA]	38Ar(cl) [fA]	39Ar(k) [fA]	40Ar(r) [fA]	Age ± 2σ (Ma)	40Ar(r) (%)	39Ar(k) (%)	K/Ca ± 2σ
17D30450	1.8 %	✓	28.23862	88.9284	0.1241909	59.42705	66.91451	3.02 ± 2.52	0.79	20.73	0.2874 ± 0.0033
17D30452	1.9 %	✓	15.97060	96.7830	0.1117307	40.48241	37.70277	2.50 ± 2.10	0.79	14.12	0.1799 ± 0.0020
17D30453	2.0 %	✓	7.75133	98.8134	0.0332141	24.09212	15.90475	1.77 ± 1.75	0.69	8.40	0.1048 ± 0.0012
17D30455	2.2 %	✓	5.07165	90.0623	0.0198989	16.77236	16.51107	2.64 ± 1.67	1.09	5.85	0.0801 ± 0.0009
17D30456	2.4 %	✓	2.12253	46.1535	0.0000000	6.71079	4.97124	1.99 ± 1.97	0.78	2.34	0.0625 ± 0.0011
17D30458	2.7 %	✓	3.75896	100.6258	0.0107884	12.77693	10.27503	2.16 ± 1.67	0.91	4.46	0.0546 ± 0.0006
17D30459	3.0 %	✓	1.80173	54.9562	0.0169490	5.75290	4.53601	2.11 ± 2.04	0.84	2.01	0.0450 ± 0.0008
17D30461	3.4 %	✓	2.54083	84.2594	0.0231181	7.88511	6.14397	2.09 ± 1.95	0.81	2.75	0.0402 ± 0.0005
17D30462	3.9 %	✓	2.10779	71.7106	0.0275277	6.30820	5.96398	2.53 ± 2.10	0.94	2.20	0.0378 ± 0.0005
17D30464	4.5 %	✓	3.68513	132.2924	0.0228828	10.70209	8.59150	2.15 ± 1.97	0.78	3.73	0.0348 ± 0.0004
17D30465	5.2 %	✓	3.08910	115.5989	0.0245332	8.09232	6.61662	2.19 ± 2.23	0.72	2.82	0.0301 ± 0.0004
17D30467	6.0 %	✓	3.68638	134.7389	0.0236006	9.25514	8.02072	2.32 ± 2.27	0.73	3.23	0.0295 ± 0.0003
17D30468	6.9 %	✓	4.67489	165.5648	0.0187449	10.55833	7.70249	1.96 ± 2.47	0.55	3.68	0.0274 ± 0.0003
17D30470	7.9 %	✓	3.10840	142.4583	0.0162193	8.64238	4.10470	1.27 ± 2.10	0.44	3.02	0.0261 ± 0.0003
17D30471	9.0 %	✓	2.23188	113.8209	0.0341620	6.77820	3.29067	1.30 ± 2.04	0.49	2.36	0.0256 ± 0.0003
17D30473	10.3 %	✓	3.15880	119.7889	0.0432871	6.94212	5.61056	2.17 ± 2.66	0.60	2.42	0.0249 ± 0.0003
17D30474	11.6 %	✓	5.92836	177.2056	0.0512232	8.22039	7.53419	2.46 ± 3.97	0.43	2.87	0.0199 ± 0.0002
17D30476	12.5 %	✓	6.06120	186.4005	0.0404650	6.63707	4.59502	1.86 ± 5.01	0.25	2.32	0.0153 ± 0.0002
17D30477	13.4 %	✓	2.80899	89.5608	0.0470025	4.22439	4.41587	2.80 ± 3.96	0.53	1.47	0.0203 ± 0.0003
17D30479	14.6 %	✓	4.27743	140.7700	0.0229837	5.28435	3.55588	1.80 ± 4.56	0.28	1.84	0.0161 ± 0.0002
17D30480	16.0 %	✓	4.88785	149.6624	0.0483814	5.58935	2.66805	1.28 ± 4.90	0.18	1.95	0.0161 ± 0.0002
17D30482	17.6 %	✓	9.04093	293.8776	0.0332366	8.46617	11.67313	3.70 ± 5.77	0.43	2.95	0.0124 ± 0.0001
17D30483	19.3 %	✓	3.40082	121.8653	0.0141818	3.52443	1.84414	1.40 ± 5.59	0.18	1.23	0.0124 ± 0.0002
17D30485	21.0 %	✓	3.57016	132.5027	0.0295259	3.52119	0.72759	0.55 ± 5.82	0.07	1.23	0.0114 ± 0.0002
		Σ	132.97435	2948.4007	0.8378477	286.64576	249.87448				

Information on Analysis

Project = **O-CONNOR (16-22)**
Sample = **PS59-244-002**
Material = **Groundmass**
Location = **Gakkel Ridge**
Region = **Artic Ocean**
Analyst = **Dan Miggins**
Irradiation = **17-OSU-05 (5A41-17)**
J = **0.00148357 ± 0.00000156**
FCT-NM = **28.201 ± 0.023 Ma**

**Results**	40(r)/39(k) ± 2σ		Age ± 2σ (Ma)		MSWD	39Ar(k) (%,n)	K/Ca ± 2σ
**Age Plateau**<br>Overestimated Error	0.79033	± 0.18228<br>± 23.06%	**2.12**	**± 0.49**<br>**± 23.05%**	0.16<br>100%	100.00<br>24	0.0194 ± 0.0050
			Full External Error	± 0.49	1.59	2σ Confidence Limit	
			Analytical Error	± 0.49	1.0000	Error Magnification	
**Total Fusion Age**	0.87172	± 0.27061<br>± 31.04%	**2.34**	**± 0.73**<br>**± 31.02%**		24	0.0418 ± 0.0001
			Full External Error	± 0.73			
			Analytical Error	± 0.73			

Normal Isochron			39(k)/36(a) ± 2σ	40(a+r)/36(a) ± 2σ	r.i.
17D30450	1.8 %	✓	2.10 ± 0.01	299.10 ± 1.69	0.9610
17D30452	1.9 %	✓	2.53 ± 0.02	299.09 ± 1.70	0.9497
17D30453	2.0 %	✓	3.11 ± 0.02	298.78 ± 1.74	0.9146
17D30455	2.2 %	✓	3.31 ± 0.02	299.99 ± 1.79	0.8656
17D30456	2.4 %	✓	3.16 ± 0.03	299.07 ± 2.09	0.6457
17D30458	2.7 %	✓	3.40 ± 0.02	299.46 ± 1.85	0.8243
17D30459	3.0 %	✓	3.19 ± 0.03	299.25 ± 2.20	0.5896
17D30461	3.4 %	✓	3.10 ± 0.03	299.15 ± 2.01	0.6899
17D30462	3.9 %	✓	2.99 ± 0.03	299.56 ± 2.11	0.6290
17D30464	4.5 %	✓	2.90 ± 0.02	299.06 ± 1.87	0.7791
17D30465	5.2 %	✓	2.62 ± 0.02	298.87 ± 1.92	0.7019
17D30467	6.0 %	✓	2.51 ± 0.02	298.91 ± 1.86	0.7434
17D30468	6.9 %	✓	2.26 ± 0.02	298.38 ± 1.81	0.7838
17D30470	7.9 %	✓	2.78 ± 0.02	298.05 ± 1.91	0.7429
17D30471	9.0 %	✓	3.04 ± 0.03	298.20 ± 2.06	0.6621
17D30473	10.3 %	✓	2.20 ± 0.02	298.51 ± 1.92	0.6611
17D30474	11.6 %	✓	1.39 ± 0.01	298.00 ± 1.77	0.7310
17D30476	12.5 %	✓	1.10 ± 0.01	297.49 ± 1.76	0.6338
17D30477	13.4 %	✓	1.50 ± 0.02	298.30 ± 1.96	0.4945
17D30479	14.6 %	✓	1.24 ± 0.01	297.56 ± 1.82	0.5477
17D30480	16.0 %	✓	1.14 ± 0.01	297.28 ± 1.80	0.5839
17D30482	17.6 %	✓	0.94 ± 0.01	298.02 ± 1.73	0.7296
17D30483	19.3 %	✓	1.04 ± 0.02	297.27 ± 1.89	0.4128
17D30485	21.0 %	✓	0.99 ± 0.01	296.93 ± 1.86	0.4236

Results	40(a)/36(a) ± 2σ		40(r)/39(k) ± 2σ		Age ± 2σ (Ma)		MSWD
**Normal Isochron**<br>Overestimated Error	**296.73**	**± 1.06**<br>**± 0.36%**	0.79302	± 0.44320<br>± 55.89%	**2.13**	**± 1.19**<br>**± 55.86%**	0.22<br>100%
				Full External Error	± 1.19		
				Analytical Error	± 1.19		

Statistics				
	2σ Confidence Limit	1.60	Convergence	0.000004024713
	Error Magnification	1.0000	Number of Iterations	2
	Number of Data Points	24	Calculated Line	Weighted York-2

Inverse Isochron			39(k)/40(a+r) ± 2σ	36(a)/40(a+r) ± 2σ	r.i.
17D30450	1.8 %	✓	0.0070360 ± 0.0000114	0.00334337 ± 0.00001889	0.0040
17D30452	1.9 %	✓	0.0084750 ± 0.0000159	0.00334347 ± 0.00001902	0.0101
17D30453	2.0 %	✓	0.0104027 ± 0.0000265	0.00334692 ± 0.00001950	0.0304
17D30455	2.2 %	✓	0.0110241 ± 0.0000369	0.00333349 ± 0.00001986	0.0521
17D30456	2.4 %	✓	0.0105717 ± 0.0000771	0.00334367 ± 0.00002333	0.1171
17D30458	2.7 %	✓	0.0113505 ± 0.0000460	0.00333931 ± 0.00002067	0.0757
17D30459	3.0 %	✓	0.0106701 ± 0.0000920	0.00334171 ± 0.00002460	0.1301
17D30461	3.4 %	✓	0.0103740 ± 0.0000666	0.00334283 ± 0.00002244	0.0964
17D30462	3.9 %	✓	0.0099907 ± 0.0000769	0.00333824 ± 0.00002351	0.1110
17D30464	4.5 %	✓	0.0097108 ± 0.0000464	0.00334379 ± 0.00002087	0.0663
17D30465	5.2 %	✓	0.0087651 ± 0.0000532	0.00334591 ± 0.00002145	0.0724
17D30467	6.0 %	✓	0.0083994 ± 0.0000447	0.00334554 ± 0.00002083	0.0597
17D30468	6.9 %	✓	0.0075693 ± 0.0000351	0.00335146 ± 0.00002029	0.0440
17D30470	7.9 %	✓	0.0093284 ± 0.0000503	0.00335514 ± 0.00002148	0.0810
17D30471	9.0 %	✓	0.0101842 ± 0.0000712	0.00335340 ± 0.00002321	0.1119
17D30473	10.3 %	✓	0.0073623 ± 0.0000503	0.00335001 ± 0.00002153	0.0615
17D30474	11.6 %	✓	0.0046531 ± 0.0000252	0.00335569 ± 0.00001993	0.0240
17D30476	12.5 %	✓	0.0036809 ± 0.0000260	0.00336148 ± 0.00001987	0.0177
17D30477	13.4 %	✓	0.0050415 ± 0.0000538	0.00335231 ± 0.00002208	0.0486
17D30479	14.6 %	✓	0.0041518 ± 0.0000373	0.00336065 ± 0.00002058	0.0270
17D30480	16.0 %	✓	0.0038467 ± 0.0000315	0.00336388 ± 0.00002042	0.0229
17D30482	17.6 %	✓	0.0031421 ± 0.0000169	0.00335547 ± 0.00001946	0.0107
17D30483	19.3 %	✓	0.0034862 ± 0.0000461	0.00336392 ± 0.00002138	0.0280
17D30485	21.0 %	✓	0.0033216 ± 0.0000421	0.00336775 ± 0.00002112	0.0269

Results	40(a)/36(a) ± 2σ		40(r)/39(k) ± 2σ		Age ± 2σ (Ma)		MSWD
**Inverse Isochron**<br>Overestimated Error	**296.73**	**± 1.06**<br>**± 0.36%**	0.79331	± 0.37678<br>± 47.49%	**2.13**	**± 1.01**<br>**± 47.47%**	0.22<br>100%
					Full External Error	± 1.01	
					Analytical Error	± 1.01	

Statistics				
	2σ Confidence Limit	1.60	Convergence	0.0000052755
	Error Magnification	1.0000	Number of Iterations	3
	Number of Data Points	24	Calculated Line	Weighted York-2
	Spreading Factor	0.7%		

Degassing Patterns			36Ar(a) [fA]	%1σ	36Ar(c) [fA]	%1σ	36Ar(ca) [fA]	%1σ	36Ar(cl) [fA]	%1σ	37Ar(ca) [fA]	%1σ	38Ar(a) [fA]	%1σ	38Ar(c) [fA]	%1σ	38Ar(k) [fA]	%1σ	38Ar(ca) [fA]	%1σ	38Ar(cl) [fA]	%1σ	39Ar(k) [fA]	%1σ	39Ar(ca) [fA]	%1σ	40Ar(r) [fA]	%1σ	40Ar(a) [fA]	%1σ	40Ar(c) [fA]	%1σ	40Ar(k) [fA]	%1σ
17D30450	1.8 %	✓	28.23862	0.28	0.0000000	0.00	0.0240373	0.59	0.0000255	23.86	88.9284	0.56	5.277798	0.28	0.0000000	0.00	0.7177004	0.12	0.0160071	9.65	0.1241909	23.88	59.42705	0.08	0.0571365	1.08	66.91451	41.80	8379.246	0.33	0.0000000	0.00	0.0360722	9.65
17D30452	1.9 %	✓	15.97060	0.28	0.0000000	0.00	0.0261604	0.56	0.0000229	24.23	96.7830	0.54	2.984905	0.28	0.0000000	0.00	0.4889061	0.13	0.0174209	9.64	0.1117307	24.25	40.48241	0.09	0.0621831	1.06	37.70277	42.18	4738.956	0.34	0.0000000	0.00	0.0245728	9.65
17D30453	2.0 %	✓	7.75133	0.29	0.0000000	0.00	0.0267093	0.56	0.0000068	72.95	98.8134	0.54	1.448723	0.29	0.0000000	0.00	0.2909605	0.15	0.0177864	9.64	0.0332141	72.96	24.09212	0.12	0.0634876	1.06	15.90475	49.37	2300.051	0.34	0.0000000	0.00	0.0146239	9.65
17D30455	2.2 %	✓	5.07165	0.29	0.0000000	0.00	0.0243438	0.57	0.0000041	120.84	90.0623	0.54	0.947891	0.29	0.0000000	0.00	0.2025598	0.18	0.0162112	9.65	0.0198989	120.84	16.77236	0.16	0.0578650	1.07	16.51107	31.64	1504.910	0.34	0.0000000	0.00	0.0101808	9.65
17D30456	2.4 %	✓	2.12253	0.33	0.0000000	0.00	0.0124753	0.84	0.0000000	0.00	46.1535	0.82	0.396701	0.33	0.0000000	0.00	0.0810462	0.36	0.0083076	9.67	0.0000000	0.00	6.71079	0.34	0.0296536	1.24	4.97124	49.66	629.819	0.37	0.0000000	0.00	0.0040734	9.66
17D30458	2.7 %	✓	3.75896	0.30	0.0000000	0.00	0.0271992	0.57	0.0000022	218.23	100.6258	0.55	0.702549	0.30	0.0000000	0.00	0.1543070	0.21	0.0181126	9.65	0.0107884	218.24	12.77693	0.19	0.0646521	1.07	10.27503	38.77	1115.395	0.35	0.0000000	0.00	0.0077556	9.65
17D30459	3.0 %	✓	1.80173	0.34	0.0000000	0.00	0.0148547	0.76	0.0000035	141.90	54.9562	0.74	0.336743	0.34	0.0000000	0.00	0.0694777	0.42	0.0098921	9.66	0.0169490	141.91	5.75290	0.41	0.0353094	1.18	4.53601	48.24	534.626	0.38	0.0000000	0.00	0.0034920	9.66
17D30461	3.4 %	✓	2.54083	0.32	0.0000000	0.00	0.0227753	0.59	0.0000047	102.57	84.2594	0.57	0.474881	0.32	0.0000000	0.00	0.0952285	0.32	0.0151667	9.65	0.0231181	102.57	7.88511	0.30	0.0541367	1.08	6.14397	46.65	753.940	0.37	0.0000000	0.00	0.0047863	9.65
17D30462	3.9 %	✓	2.10779	0.33	0.0000000	0.00	0.0193834	0.64	0.0000056	87.91	71.7106	0.62	0.393946	0.33	0.0000000	0.00	0.0761841	0.38	0.0129079	9.65	0.0275277	87.91	6.30820	0.36	0.0460740	1.11	5.96398	41.42	625.444	0.38	0.0000000	0.00	0.0038291	9.66
17D30464	4.5 %	✓	3.68513	0.30	0.0000000	0.00	0.0357586	0.52	0.0000047	109.42	132.2924	0.49	0.688750	0.30	0.0000000	0.00	0.1292492	0.25	0.0238126	9.64	0.0228828	109.42	10.70209	0.23	0.0849979	1.04	8.59150	45.74	1093.488	0.35	0.0000000	0.00	0.0064962	9.65
17D30465	5.2 %	✓	3.08910	0.31	0.0000000	0.00	0.0312464	0.54	0.0000050	94.11	115.5989	0.51	0.577352	0.31	0.0000000	0.00	0.0977309	0.31	0.0208078	9.64	0.0245332	94.11	8.09232	0.29	0.0742723	1.05	6.61662	50.80	916.628	0.36	0.0000000	0.00	0.0049120	9.65
17D30467	6.0 %	✓	3.68638	0.30	0.0000000	0.00	0.0364199	0.51	0.0000048	101.79	134.7389	0.48	0.688984	0.30	0.0000000	0.00	0.1117743	0.27	0.0242530	9.64	0.0236006	101.79	9.25514	0.26	0.0865697	1.04	8.02072	48.92	1093.859	0.35	0.0000000	0.00	0.0056179	9.65
17D30468	6.9 %	✓	4.67489	0.30	0.0000000	0.00	0.0447522	0.49	0.0000038	122.43	165.5648	0.46	0.873738	0.30	0.0000000	0.00	0.1275130	0.24	0.0298017	9.64	0.0187449	122.44	10.55833	0.23	0.1063754	1.03	7.70249	63.26	1387.181	0.35	0.0000000	0.00	0.0064089	9.65
17D30470	7.9 %	✓	3.10840	0.31	0.0000000	0.00	0.0385065	0.50	0.0000033	150.31	142.4583	0.47	0.580960	0.31	0.0000000	0.00	0.1043740	0.27	0.0256425	9.64	0.0162193	150.31	8.64238	0.26	0.0915295	1.03	4.10470	82.34	922.355	0.36	0.0000000	0.00	0.0052459	9.65
17D30471	9.0 %	✓	2.23188	0.33	0.0000000	0.00	0.0307658	0.53	0.0000070	71.42	113.8209	0.50	0.417139	0.33	0.0000000	0.00	0.0818603	0.34	0.0204878	9.64	0.0341620	71.43	6.77820	0.33	0.0731299	1.05	3.29067	78.37	662.267	0.37	0.0000000	0.00	0.0041144	9.66
17D30473	10.3 %	✓	3.15880	0.31	0.0000000	0.00	0.0323789	0.53	0.0000089	55.59	119.7889	0.50	0.590380	0.31	0.0000000	0.00	0.0838400	0.34	0.0215620	9.64	0.0432871	55.59	6.94212	0.33	0.0769644	1.05	5.61056	61.38	937.312	0.36	0.0000000	0.00	0.0042139	9.66
17D30474	11.6 %	✓	5.92836	0.29	0.0000000	0.00	0.0478987	0.49	0.0000105	48.20	177.2056	0.46	1.108011	0.29	0.0000000	0.00	0.0992777	0.28	0.0318970	9.64	0.0512232	48.21	8.22039	0.27	0.1138546	1.03	7.53419	80.85	1759.122	0.34	0.0000000	0.00	0.0049898	9.65
17D30476	12.5 %	✓	6.06120	0.29	0.0000000	0.00	0.0503840	0.48	0.0000083	59.27	186.4005	0.45	1.132837	0.29	0.0000000	0.00	0.0801558	0.36	0.0335521	9.64	0.0404650	59.28	6.63707	0.35	0.1197623	1.03	4.59502	135.06	1798.539	0.34	0.0000000	0.00	0.0040287	9.66
17D30477	13.4 %	✓	2.80899	0.32	0.0000000	0.00	0.0242083	0.58	0.0000097	52.66	89.5608	0.56	0.525000	0.32	0.0000000	0.00	0.0510179	0.53	0.0161210	9.65	0.0470025	52.67	4.22439	0.53	0.0575428	1.08	4.41587	70.68	833.511	0.36	0.0000000	0.00	0.0025642	9.66
17D30479	14.6 %	✓	4.27743	0.30	0.0000000	0.00	0.0380501	0.51	0.0000047	109.10	140.7700	0.48	0.799452	0.30	0.0000000	0.00	0.0638192	0.45	0.0253386	9.64	0.0229837	109.10	5.28435	0.45	0.0904447	1.04	3.55588	126.43	1269.243	0.35	0.0000000	0.00	0.0032076	9.66
17D30480	16.0 %	✓	4.88785	0.30	0.0000000	0.00	0.0404538	0.50	0.0000099	49.94	149.6624	0.47	0.913539	0.30	0.0000000	0.00	0.0675025	0.42	0.0269392	9.64	0.0483814	49.95	5.58935	0.41	0.0961581	1.03	2.66805	191.27	1450.372	0.35	0.0000000	0.00	0.0033927	9.66
17D30482	17.6 %	✓	9.04093	0.29	0.0000000	0.00	0.0794351	0.46	0.0000068	81.08	293.8776	0.43	1.689750	0.29	0.0000000	0.00	0.1022459	0.28	0.0528980	9.64	0.0332366	81.08	8.46617	0.27	0.1888164	1.01	11.67313	78.19	2682.715	0.34	0.0000000	0.00	0.0051390	9.65
17D30483	19.3 %	✓	3.40082	0.31	0.0000000	0.00	0.0329402	0.52	0.0000029	178.74	121.8653	0.50	0.635613	0.31	0.0000000	0.00	0.0425645	0.66	0.0219358	9.64	0.0141818	178.74	3.52443	0.66	0.0782985	1.05	1.84414	199.32	1009.125	0.36	0.0000000	0.00	0.0021393	9.67
17D30485	21.0 %	✓	3.57016	0.30	0.0000000	0.00	0.0358155	0.51	0.0000061	83.08	132.5027	0.48	0.667262	0.30	0.0000000	0.00	0.0425254	0.64	0.0238505	9.64	0.0295259	83.08	3.52119	0.63	0.0851330	1.04	0.72759	524.98	1059.372	0.35	0.0000000	0.00	0.0021374	9.67
		Σ	132.97435	0.08	0.0000000	0.00	0.7969527	0.11	0.0001720	14.16	2948.4007	0.11	24.852905	0.08	0.0000000	0.00	3.4618208	0.05	0.5307121	2.12	0.8378477	14.16	286.64576	0.04	1.8943475	0.23	249.87448	15.52	39457.478	0.10	0.0000000	0.00	0.1739940	2.86
		Σ							133.77147	0.08	2948.4007	0.11									29.683286	0.41			288.54011	0.04							39707.526	0.14

Additional Parameters			40Ar/39Ar	1σ	37Ar/39Ar	1σ	36Ar/39Ar	1σ	Time (days)	37Ar (decay)	39Ar (decay)	40Ar (moles)
17D30450	1.8 %	✓	141.990636	0.115216	1.494992	0.008510	0.475129	0.001394	126.285	12.234857	1.00089502	4.054E-10
17D30452	1.9 %	✓	117.813086	0.110006	2.387075	0.012964	0.394548	0.001176	126.299	12.238214	1.00089512	2.293E-10
17D30453	2.0 %	✓	95.877157	0.121601	4.090701	0.022486	0.321997	0.001009	126.306	12.239893	1.00089517	1.112E-10
17D30455	2.2 %	✓	90.398775	0.150926	5.351222	0.030180	0.302788	0.001007	126.319	12.243251	1.00089527	7.303E-11
17D30456	2.4 %	✓	94.176900	0.342011	6.847249	0.061119	0.316746	0.001494	126.326	12.244931	1.00089531	3.047E-11
17D30458	2.7 %	✓	87.658827	0.176769	7.835935	0.045321	0.294836	0.001045	126.340	12.248290	1.00089541	5.403E-11
17D30459	3.0 %	✓	93.149039	0.399330	9.494510	0.080366	0.313842	0.001649	126.347	12.249971	1.00089546	2.588E-11
17D30461	3.4 %	✓	95.738105	0.305497	10.613022	0.068294	0.322903	0.001414	126.361	12.253332	1.00089556	3.648E-11
17D30462	3.9 %	✓	99.368137	0.379853	11.285410	0.081151	0.334764	0.001633	126.369	12.255181	1.00089561	3.031E-11
17D30464	4.5 %	✓	102.167096	0.242096	12.263961	0.065876	0.344939	0.001299	126.383	12.258543	1.00089571	5.290E-11
17D30465	5.2 %	✓	113.052063	0.340275	14.155099	0.083098	0.382087	0.001609	126.390	12.260225	1.00089576	4.432E-11
17D30467	6.0 %	✓	117.953267	0.311109	14.423366	0.078693	0.398514	0.001568	126.403	12.263588	1.00089586	5.289E-11
17D30468	6.9 %	✓	130.794972	0.300295	15.524555	0.079062	0.442548	0.001635	126.410	12.265271	1.00089591	6.695E-11
17D30470	7.9 %	✓	106.076786	0.283170	16.310947	0.087627	0.360309	0.001430	126.424	12.268636	1.00089601	4.447E-11
17D30471	9.0 %	✓	97.143406	0.336293	16.612972	0.099607	0.330251	0.001512	126.431	12.270319	1.00089606	3.195E-11
17D30473	10.3 %	✓	134.337483	0.453706	17.066166	0.101937	0.454645	0.002042	126.445	12.273685	1.00089615	4.526E-11
17D30474	11.6 %	✓	211.976169	0.566615	21.262347	0.112036	0.717074	0.002818	126.452	12.275369	1.00089620	8.480E-11
17D30476	12.5 %	✓	266.861540	0.924618	27.586979	0.157144	0.904505	0.004068	126.466	12.278737	1.00089630	8.655E-11
17D30477	13.4 %	✓	195.689776	1.030642	20.916005	0.159041	0.661666	0.004008	126.473	12.280421	1.00089635	4.022E-11
17D30479	14.6 %	✓	236.809135	1.045676	26.190748	0.169603	0.802911	0.004246	126.487	12.283791	1.00089645	6.109E-11
17D30480	16.0 %	✓	255.569847	1.027012	26.323513	0.161921	0.866821	0.004303	126.494	12.285644	1.00089650	6.975E-11
17D30482	17.6 %	✓	311.311319	0.816808	33.954737	0.170056	1.053772	0.004078	126.508	12.289015	1.00089660	1.293E-10
17D30483	19.3 %	✓	280.613106	1.812739	33.825881	0.274385	0.953102	0.006771	126.515	12.290701	1.00089665	4.853E-11
17D30485	21.0 %	✓	293.956729	1.819519	36.741805	0.287125	0.999905	0.006847	126.529	12.294073	1.00089675	5.088E-11

Procedure Blanks		36Ar ± 1σ (SE) [fA]	37Ar ± 1σ (SE) [fA]	38Ar ± 1σ (SE) [fA]	39Ar ± 1σ (SE) [fA]	40Ar ± 1σ (SE) [fA]
17D30450	1.8 %	0.0125032 ± 0.0026337	0.0332332 ± 0.0191953	0.0349522 ± 0.0170025	0.0450213 ± 0.0156534	3.8070090 ± 0.7730966
17D30452	1.9 %	0.0150639 ± 0.0026337	0.0202109 ± 0.0191953	0.0231362 ± 0.0170025	0.0507892 ± 0.0156534	4.5228647 ± 0.7730966
17D30453	2.0 %	0.0161508 ± 0.0026337	0.0151806 ± 0.0191953	0.0176900 ± 0.0170025	0.0514133 ± 0.0156534	4.8349111 ± 0.7730966
17D30455	2.2 %	0.0179511 ± 0.0026337	0.0076668 ± 0.0191953	0.0078207 ± 0.0170025	0.0494449 ± 0.0156534	5.3659833 ± 0.7730966
17D30456	2.4 %	0.0186708 ± 0.0026337	0.0050283 ± 0.0191953	0.0034287 ± 0.0170025	0.0473188 ± 0.0156534	5.5849127 ± 0.7730966
17D30458	2.7 %	0.0197662 ± 0.0026337	0.0016265 ± 0.0191953	0.0042133 ± 0.0170025	0.0418112 ± 0.0156534	5.9303328 ± 0.7730966
17D30459	3.0 %	0.0201498 ± 0.0026337	0.0007295 ± 0.0191953	0.0074495 ± 0.0170025	0.0387861 ± 0.0156534	6.0575402 ± 0.7730966
17D30461	3.4 %	0.0206096 ± 0.0026337	0.0002352 ± 0.0191953	0.0127386 ± 0.0170025	0.0329473 ± 0.0156534	6.2237577 ± 0.7730966
17D30462	3.9 %	0.0206987 ± 0.0026337	0.0005711 ± 0.0191953	0.0149792 ± 0.0170025	0.0301423 ± 0.0156534	6.2668420 ± 0.7730966
17D30464	4.5 %	0.0205890 ± 0.0026337	0.0020195 ± 0.0191953	0.0178694 ± 0.0170025	0.0262814 ± 0.0156534	6.2624324 ± 0.7730966
17D30465	5.2 %	0.0204138 ± 0.0026337	0.0030560 ± 0.0191953	0.0187632 ± 0.0170025	0.0250878 ± 0.0156534	6.2226121 ± 0.7730966
17D30467	6.0 %	0.0198514 ± 0.0026337	0.0055543 ± 0.0191953	0.0195225 ± 0.0170025	0.0243698 ± 0.0156534	6.0746819 ± 0.7730966
17D30468	6.9 %	0.0194767 ± 0.0026337	0.0069469 ± 0.0191953	0.0194261 ± 0.0170025	0.0248686 ± 0.0156534	5.9697550 ± 0.7730966
17D30470	7.9 %	0.0185725 ± 0.0026337	0.0098727 ± 0.0191953	0.0183983 ± 0.0170025	0.0275025 ± 0.0156534	5.7069518 ± 0.7730966
17D30471	9.0 %	0.0180569 ± 0.0026337	0.0113581 ± 0.0191953	0.0175223 ± 0.0170025	0.0295510 ± 0.0156534	5.5530717 ± 0.7730966
17D30473	10.3 %	0.0169353 ± 0.0026337	0.0142809 ± 0.0191953	0.0152063 ± 0.0170025	0.0347570 ± 0.0156534	5.2113612 ± 0.7730966
17D30474	11.6 %	0.0163447 ± 0.0026337	0.0156918 ± 0.0191953	0.0138389 ± 0.0170025	0.0377180 ± 0.0156534	5.0283402 ± 0.7730966
17D30476	12.5 %	0.0151439 ± 0.0026337	0.0183729 ± 0.0191953	0.0108883 ± 0.0170025	0.0437275 ± 0.0156534	4.6510063 ± 0.7730966
17D30477	13.4 %	0.0145506 ± 0.0026337	0.0196380 ± 0.0191953	0.0093949 ± 0.0170025	0.0464696 ± 0.0156534	4.4623158 ± 0.7730966
17D30479	14.6 %	0.0134225 ± 0.0026337	0.0220309 ± 0.0191953	0.0066182 ± 0.0170025	0.0505254 ± 0.0156534	4.0999604 ± 0.7730966
17D30480	16.0 %	0.0128566 ± 0.0026337	0.0232879 ± 0.0191953	0.0053352 ± 0.0170025	0.0514582 ± 0.0156534	3.9167102 ± 0.7730966
17D30482	17.6 %	0.0119776 ± 0.0026337	0.0255377 ± 0.0191953	0.0037520 ± 0.0170025	0.0495480 ± 0.0156534	3.6307661 ± 0.7730966
17D30483	19.3 %	0.0116311 ± 0.0026337	0.0266842 ± 0.0191953	0.0034508 ± 0.0170025	0.0463060 ± 0.0156534	3.5181019 ± 0.7730966
17D30485	21.0 %	0.0111757 ± 0.0026337	0.0291427 ± 0.0191953	0.0041628 ± 0.0170025	0.0337864 ± 0.0156534	3.3725949 ± 0.7730966

Intercept Values		36Ar ± 1σ (SE) [fA]	r2	Regression (type,n)	37Ar ± 1σ (SE) [fA]	r2	Regression (type,n)	38Ar ± 1σ (SE) [fA]	r2	Regression (type,n)	39Ar ± 1σ (SE) [fA]	r2	Regression (type,n)	40Ar ± 1σ (SE) [fA]	r2	Regression (type,n)
17D30450	1.8 %	27.0218041 ± 0.0075113	0.9986	EXP 150 of 150	7.173126 ± 0.019681	0.7919	EXP 150 of 150	6.0982101 ± 0.0166372	0.8096	EXP 150 of 150	59.1258016 ± 0.0191768	0.9958	EXP 150 of 150	8450.0039 ± 0.2300	1.0000	EXP 150 of 150
17D30452	1.9 %	15.3024285 ± 0.0057384	0.9974	EXP 150 of 150	7.788602 ± 0.018400	0.8504	EXP 150 of 150	3.5835621 ± 0.0181138	0.5720	EXP 150 of 150	40.3204202 ± 0.0188979	0.9912	EXP 150 of 150	4781.2067 ± 0.1155	1.0000	EXP 150 of 150
17D30453	2.0 %	7.4492557 ± 0.0042278	0.9940	EXP 150 of 150	7.945455 ± 0.019305	0.8493	EXP 150 of 150	1.7872329 ± 0.0160585	0.2491	EXP 150 of 150	24.0431981 ± 0.0185324	0.9741	EXP 150 of 150	2320.8055 ± 0.0749	0.9999	EXP 150 of 150
17D30455	2.2 %	4.8879518 ± 0.0029745	0.9924	EXP 150 of 150	7.233638 ± 0.016613	0.8552	EXP 150 of 150	1.1803729 ± 0.0162093	0.0791	EXP 150 of 150	16.7655256 ± 0.0181326	0.9491	EXP 150 of 150	1526.7974 ± 0.0667	0.9998	EXP 150 of 150
17D30456	2.4 %	2.0589947 ± 0.0020643	0.9771	EXP 150 of 150	3.707556 ± 0.018199	0.5952	EXP 150 of 150	0.4737756 ± 0.0172152	0.0028	EXP 150 of 150	6.7420481 ± 0.0160651	0.6775	EXP 149 of 150	640.3789 ± 0.0492	0.9980	EXP 150 of 150
17D30458	2.7 %	3.6380179 ± 0.0027427	0.9884	EXP 150 of 150	8.071824 ± 0.021747	0.8081	EXP 150 of 150	0.8710866 ± 0.0155957	0.0485	EXP 149 of 150	12.7963036 ± 0.0161918	0.9292	EXP 150 of 150	1131.6086 ± 0.0641	0.9996	EXP 150 of 150
17D30459	3.0 %	1.7561734 ± 0.0018400	0.9755	EXP 150 of 150	4.407615 ± 0.019419	0.6266	EXP 150 of 150	0.4204993 ± 0.0165264	0.0119	EXP 150 of 150	5.7877366 ± 0.0166890	0.6012	EXP 150 of 150	545.2234 ± 0.0472	0.9974	EXP 150 of 150
17D30461	3.4 %	2.4705273 ± 0.0024730	0.9786	EXP 150 of 150	6.755065 ± 0.018181	0.8212	EXP 150 of 150	0.5884726 ± 0.0159616	0.0267	EXP 149 of 150	7.9183495 ± 0.0171472	0.7715	EXP 150 of 150	766.3123 ± 0.0517	0.9993	EXP 150 of 150
17D30462	3.9 %	2.0535421 ± 0.0021825	0.9767	EXP 150 of 150	5.748530 ± 0.018625	0.7768	EXP 149 of 150	0.4895586 ± 0.0167000	0.0281	EXP 150 of 150	6.3413190 ± 0.0160698	0.7009	EXP 150 of 150	637.6791 ± 0.0446	0.9990	EXP 150 of 150
17D30464	4.5 %	3.5764660 ± 0.0028611	0.9870	EXP 150 of 150	10.603008 ± 0.019843	0.9112	EXP 150 of 150	0.8366166 ± 0.0176659	0.0703	EXP 150 of 150	10.7402086 ± 0.0169823	0.8855	EXP 150 of 150	1108.3480 ± 0.0511	0.9998	EXP 150 of 150
17D30465	5.2 %	3.0023833 ± 0.0025392	0.9855	EXP 150 of 150	9.265067 ± 0.020421	0.8789	EXP 149 of 150	0.6931557 ± 0.0149406	0.0529	EXP 150 of 150	8.1362872 ± 0.0165380	0.7860	EXP 150 of 150	929.4723 ± 0.0510	0.9996	EXP 150 of 150
17D30467	6.0 %	3.5775557 ± 0.0027515	0.9885	EXP 150 of 150	10.798140 ± 0.019269	0.9098	EXP 150 of 150	0.8190704 ± 0.0162275	0.0761	EXP 150 of 150	9.3027179 ± 0.0164467	0.8537	EXP 150 of 150	1107.9597 ± 0.0536	0.9998	EXP 150 of 150
17D30468	6.9 %	4.5298211 ± 0.0031242	0.9907	EXP 150 of 150	13.266875 ± 0.018381	0.9392	EXP 150 of 150	1.0179770 ± 0.0144345	0.0552	EXP 150 of 150	10.6172425 ± 0.0160812	0.8833	EXP 150 of 150	1400.8599 ± 0.0546	0.9999	EXP 150 of 150
17D30470	7.9 %	3.0259225 ± 0.0024832	0.9865	EXP 150 of 150	11.416091 ± 0.018549	0.9261	EXP 150 of 150	0.7002118 ± 0.0167644	0.0340	EXP 150 of 150	8.7021720 ± 0.0142451	0.8650	EXP 149 of 150	932.1719 ± 0.0525	0.9996	EXP 150 of 150
17D30471	9.0 %	2.1803692 ± 0.0021449	0.9787	EXP 150 of 150	9.123415 ± 0.018044	0.8904	EXP 150 of 150	0.5295903 ± 0.0169059	0.0179	EXP 150 of 150	6.8344092 ± 0.0150092	0.7498	EXP 150 of 150	671.1145 ± 0.0462	0.9992	EXP 150 of 150
17D30473	10.3 %	3.0666050 ± 0.0027920	0.9831	EXP 150 of 150	9.601481 ± 0.019113	0.8863	EXP 150 of 150	0.7151377 ± 0.0163548	0.0549	EXP 150 of 150	7.0062358 ± 0.0159220	0.7313	EXP 150 of 150	948.1380 ± 0.0509	0.9997	EXP 150 of 150
17D30474	11.6 %	5.7275806 ± 0.0036563	0.9922	EXP 150 of 150	14.196243 ± 0.020137	0.9415	EXP 150 of 150	1.2613348 ± 0.0168250	0.2111	EXP 150 of 150	8.3154342 ± 0.0141018	0.8174	EXP 150 of 150	1771.6899 ± 0.0692	0.9999	EXP 150 of 150
17D30476	12.5 %	5.8556973 ± 0.0034047	0.9936	EXP 149 of 150	14.930634 ± 0.021115	0.9387	EXP 150 of 150	1.2609275 ± 0.0157575	0.1857	EXP 150 of 150	6.7547248 ± 0.0163082	0.6179	EXP 150 of 150	1807.7886 ± 0.0664	0.9999	EXP 150 of 150
17D30477	13.4 %	2.7221111 ± 0.0027135	0.9796	EXP 150 of 150	7.183629 ± 0.018663	0.8314	EXP 150 of 150	0.6222006 ± 0.0174073	0.0683	EXP 150 of 150	4.2993538 ± 0.0152529	0.3283	EXP 150 of 150	842.3919 ± 0.0474	0.9996	EXP 150 of 150
17D30479	14.6 %	4.1375281 ± 0.0031097	0.9892	EXP 150 of 150	11.279167 ± 0.019266	0.9219	EXP 150 of 150	0.8942130 ± 0.0176549	0.0620	EXP 150 of 150	5.3888673 ± 0.0169279	0.4030	EXP 150 of 150	1276.9016 ± 0.0606	0.9998	EXP 150 of 150
17D30480	16.0 %	4.7226120 ± 0.0036462	0.9886	EXP 150 of 150	11.989730 ± 0.018644	0.9319	EXP 150 of 150	1.0385557 ± 0.0162731	0.1393	EXP 149 of 150	5.6983969 ± 0.0156760	0.4498	EXP 150 of 150	1456.9602 ± 0.0519	0.9999	EXP 150 of 150
17D30482	17.6 %	8.7278833 ± 0.0040840	0.9958	EXP 150 of 150	23.516434 ± 0.018789	0.9812	EXP 150 of 150	1.8522049 ± 0.0190936	0.2466	EXP 150 of 150	8.6458208 ± 0.0149170	0.7095	EXP 150 of 150	2698.0245 ± 0.0842	0.9999	EXP 150 of 150
17D30483	19.3 %	3.2931150 ± 0.0029608	0.9842	EXP 150 of 150	9.766566 ± 0.018976	0.8956	EXP 150 of 150	0.7024115 ± 0.0181423	0.0137	EXP 150 of 150	3.6245921 ± 0.0166045	0.0738	EXP 150 of 150	1014.4898 ± 0.0520	0.9997	EXP 150 of 150
17D30485	21.0 %	3.4572363 ± 0.0027985	0.9878	EXP 149 of 150	10.616294 ± 0.018411	0.9191	EXP 149 of 150	0.7499910 ± 0.0169701	0.0845	EXP 150 of 150	3.6156434 ± 0.0152834	0.0990	EXP 150 of 150	1063.4746 ± 0.0545	0.9997	EXP 150 of 150

Project Info		Analyst	Irradiation	X-pos	Y-pos	Z/H-pos	Project	Experiment	Nmb
17D30450	1.8 %	Dan Miggins	17-OSU-05	0.00	0.00	60.23	Arctic\O-Connor (16-22)	17D30446	01
17D30452	1.9 %	Dan Miggins	17-OSU-05	0.00	0.00	60.23	Arctic\O-Connor (16-22)	17D30446	01
17D30453	2.0 %	Dan Miggins	17-OSU-05	0.00	0.00	60.23	Arctic\O-Connor (16-22)	17D30446	01
17D30455	2.2 %	Dan Miggins	17-OSU-05	0.00	0.00	60.23	Arctic\O-Connor (16-22)	17D30446	01
17D30456	2.4 %	Dan Miggins	17-OSU-05	0.00	0.00	60.23	Arctic\O-Connor (16-22)	17D30446	01
17D30458	2.7 %	Dan Miggins	17-OSU-05	0.00	0.00	60.23	Arctic\O-Connor (16-22)	17D30446	01
17D30459	3.0 %	Dan Miggins	17-OSU-05	0.00	0.00	60.23	Arctic\O-Connor (16-22)	17D30446	01
17D30461	3.4 %	Dan Miggins	17-OSU-05	0.00	0.00	60.23	Arctic\O-Connor (16-22)	17D30446	01
17D30462	3.9 %	Dan Miggins	17-OSU-05	0.00	0.00	60.23	Arctic\O-Connor (16-22)	17D30446	01
17D30464	4.5 %	Dan Miggins	17-OSU-05	0.00	0.00	60.23	Arctic\O-Connor (16-22)	17D30446	01
17D30465	5.2 %	Dan Miggins	17-OSU-05	0.00	0.00	60.23	Arctic\O-Connor (16-22)	17D30446	01
17D30467	6.0 %	Dan Miggins	17-OSU-05	0.00	0.00	60.23	Arctic\O-Connor (16-22)	17D30446	01
17D30468	6.9 %	Dan Miggins	17-OSU-05	0.00	0.00	60.23	Arctic\O-Connor (16-22)	17D30446	01
17D30470	7.9 %	Dan Miggins	17-OSU-05	0.00	0.00	60.23	Arctic\O-Connor (16-22)	17D30446	01
17D30471	9.0 %	Dan Miggins	17-OSU-05	0.00	0.00	60.23	Arctic\O-Connor (16-22)	17D30446	01
17D30473	10.3 %	Dan Miggins	17-OSU-05	0.00	0.00	60.23	Arctic\O-Connor (16-22)	17D30446	01
17D30474	11.6 %	Dan Miggins	17-OSU-05	0.00	0.00	60.23	Arctic\O-Connor (16-22)	17D30446	01
17D30476	12.5 %	Dan Miggins	17-OSU-05	0.00	0.00	60.23	Arctic\O-Connor (16-22)	17D30446	01
17D30477	13.4 %	Dan Miggins	17-OSU-05	0.00	0.00	60.23	Arctic\O-Connor (16-22)	17D30446	01
17D30479	14.6 %	Dan Miggins	17-OSU-05	0.00	0.00	60.23	Arctic\O-Connor (16-22)	17D30446	01
17D30480	16.0 %	Dan Miggins	17-OSU-05	0.00	0.00	60.23	Arctic\O-Connor (16-22)	17D30446	01
17D30482	17.6 %	Dan Miggins	17-OSU-05	0.00	0.00	60.23	Arctic\O-Connor (16-22)	17D30446	01
17D30483	19.3 %	Dan Miggins	17-OSU-05	0.00	0.00	60.23	Arctic\O-Connor (16-22)	17D30446	01
17D30485	21.0 %	Dan Miggins	17-OSU-05	0.00	0.00	60.23	Arctic\O-Connor (16-22)	17D30446	01

**Sample Parameters**		Sample	Material	Location	Standard Name	Standard (in Ma)	%1σ	Standard Reference	Standard 40Ar/39Ar	%1σ	J	%1σ	Air 40Ar/36Ar	%1σ	MDF (lin)	%1σ	Volume Ratio	Sensitivity (mol/volt)	Day	Month	Year	Hour	Min	Resist
17D30450	1.8 %	PS59-244-002	Groundmass	Gakkel Ridge	FCT-NM (5A41-17)	28.201	0.082	Kuiper et al (2008)	10.59431	0.105	0.00148357	0.105	302.638	0.15	0.9940959	0.069	1	4.8E-14	14	SEP	2017	18	40	1
17D30452	1.9 %	PS59-244-002	Groundmass	Gakkel Ridge	FCT-NM (5A41-17)	28.201	0.082	Kuiper et al (2008)	10.59431	0.105	0.00148357	0.105	302.638	0.15	0.9940959	0.069	1	4.8E-14	14	SEP	2017	19	0	1
17D30453	2.0 %	PS59-244-002	Groundmass	Gakkel Ridge	FCT-NM (5A41-17)	28.201	0.082	Kuiper et al (2008)	10.59431	0.105	0.00148357	0.105	302.638	0.15	0.9940959	0.069	1	4.8E-14	14	SEP	2017	19	10	1
17D30455	2.2 %	PS59-244-002	Groundmass	Gakkel Ridge	FCT-NM (5A41-17)	28.201	0.082	Kuiper et al (2008)	10.59431	0.105	0.00148357	0.105	302.638	0.15	0.9940959	0.069	1	4.8E-14	14	SEP	2017	19	30	1
17D30456	2.4 %	PS59-244-002	Groundmass	Gakkel Ridge	FCT-NM (5A41-17)	28.201	0.082	Kuiper et al (2008)	10.59431	0.105	0.00148357	0.105	302.638	0.15	0.9940959	0.069	1	4.8E-14	14	SEP	2017	19	40	1
17D30458	2.7 %	PS59-244-002	Groundmass	Gakkel Ridge	FCT-NM (5A41-17)	28.201	0.082	Kuiper et al (2008)	10.59431	0.105	0.00148357	0.105	302.638	0.15	0.9940959	0.069	1	4.8E-14	14	SEP	2017	20	0	1
17D30459	3.0 %	PS59-244-002	Groundmass	Gakkel Ridge	FCT-NM (5A41-17)	28.201	0.082	Kuiper et al (2008)	10.59431	0.105	0.00148357	0.105	302.638	0.15	0.9940959	0.069	1	4.8E-14	14	SEP	2017	20	10	1
17D30461	3.4 %	PS59-244-002	Groundmass	Gakkel Ridge	FCT-NM (5A41-17)	28.201	0.082	Kuiper et al (2008)	10.59431	0.105	0.00148357	0.105	302.638	0.15	0.9940959	0.069	1	4.8E-14	14	SEP	2017	20	30	1
17D30462	3.9 %	PS59-244-002	Groundmass	Gakkel Ridge	FCT-NM (5A41-17)	28.201	0.082	Kuiper et al (2008)	10.59431	0.105	0.00148357	0.105	302.638	0.15	0.9940959	0.069	1	4.8E-14	14	SEP	2017	20	41	1
17D30464	4.5 %	PS59-244-002	Groundmass	Gakkel Ridge	FCT-NM (5A41-17)	28.201	0.082	Kuiper et al (2008)	10.59431	0.105	0.00148357	0.105	302.638	0.15	0.9940959	0.069	1	4.8E-14	14	SEP	2017	21	1	1
17D30465	5.2 %	PS59-244-002	Groundmass	Gakkel Ridge	FCT-NM (5A41-17)	28.201	0.082	Kuiper et al (2008)	10.59431	0.105	0.00148357	0.105	302.638	0.15	0.9940959	0.069	1	4.8E-14	14	SEP	2017	21	11	1
17D30467	6.0 %	PS59-244-002	Groundmass	Gakkel Ridge	FCT-NM (5A41-17)	28.201	0.082	Kuiper et al (2008)	10.59431	0.105	0.00148357	0.105	302.638	0.15	0.9940959	0.069	1	4.8E-14	14	SEP	2017	21	31	1
17D30468	6.9 %	PS59-244-002	Groundmass	Gakkel Ridge	FCT-NM (5A41-17)	28.201	0.082	Kuiper et al (2008)	10.59431	0.105	0.00148357	0.105	302.638	0.15	0.9940959	0.069	1	4.8E-14	14	SEP	2017	21	41	1
17D30470	7.9 %	PS59-244-002	Groundmass	Gakkel Ridge	FCT-NM (5A41-17)	28.201	0.082	Kuiper et al (2008)	10.59431	0.105	0.00148357	0.105	302.638	0.15	0.9940959	0.069	1	4.8E-14	14	SEP	2017	22	1	1
17D30471	9.0 %	PS59-244-002	Groundmass	Gakkel Ridge	FCT-NM (5A41-17)	28.201	0.082	Kuiper et al (2008)	10.59431	0.105	0.00148357	0.105	302.638	0.15	0.9940959	0.069	1	4.8E-14	14	SEP	2017	22	11	1
17D30473	10.3 %	PS59-244-002	Groundmass	Gakkel Ridge	FCT-NM (5A41-17)	28.201	0.082	Kuiper et al (2008)	10.59431	0.105	0.00148357	0.105	302.638	0.15	0.9940959	0.069	1	4.8E-14	14	SEP	2017	22	31	1
17D30474	11.6 %	PS59-244-002	Groundmass	Gakkel Ridge	FCT-NM (5A41-17)	28.201	0.082	Kuiper et al (2008)	10.59431	0.105	0.00148357	0.105	302.638	0.15	0.9940959	0.069	1	4.8E-14	14	SEP	2017	22	41	1
17D30476	12.5 %	PS59-244-002	Groundmass	Gakkel Ridge	FCT-NM (5A41-17)	28.201	0.082	Kuiper et al (2008)	10.59431	0.105	0.00148357	0.105	302.638	0.15	0.9940959	0.069	1	4.8E-14	14	SEP	2017	23	1	1
17D30477	13.4 %	PS59-244-002	Groundmass	Gakkel Ridge	FCT-NM (5A41-17)	28.201	0.082	Kuiper et al (2008)	10.59431	0.105	0.00148357	0.105	302.638	0.15	0.9940959	0.069	1	4.8E-14	14	SEP	2017	23	11	1
17D30479	14.6 %	PS59-244-002	Groundmass	Gakkel Ridge	FCT-NM (5A41-17)	28.201	0.082	Kuiper et al (2008)	10.59431	0.105	0.00148357	0.105	302.638	0.15	0.9940959	0.069	1	4.8E-14	14	SEP	2017	23	31	1
17D30480	16.0 %	PS59-244-002	Groundmass	Gakkel Ridge	FCT-NM (5A41-17)	28.201	0.082	Kuiper et al (2008)	10.59431	0.105	0.00148357	0.105	302.638	0.15	0.9940959	0.069	1	4.8E-14	14	SEP	2017	23	42	1
17D30482	17.6 %	PS59-244-002	Groundmass	Gakkel Ridge	FCT-NM (5A41-17)	28.201	0.082	Kuiper et al (2008)	10.59431	0.105	0.00148357	0.105	302.638	0.15	0.9940959	0.069	1	4.8E-14	15	SEP	2017	0	2	1
17D30483	19.3 %	PS59-244-002	Groundmass	Gakkel Ridge	FCT-NM (5A41-17)	28.201	0.082	Kuiper et al (2008)	10.59431	0.105	0.00148357	0.105	302.638	0.15	0.9940959	0.069	1	4.8E-14	15	SEP	2017	0	12	1
17D30485	21.0 %	PS59-244-002	Groundmass	Gakkel Ridge	FCT-NM (5A41-17)	28.201	0.082	Kuiper et al (2008)	10.59431	0.105	0.00148357	0.105	302.638	0.15	0.9940959	0.069	1	4.8E-14	15	SEP	2017	0	32	1

**Irradiation Constants**		40/36(a)	%1σ	40/36(c)	%1σ	38/36(a)	%1σ	38/36(c)	%1σ	39/37(ca)	%1σ	38/37(ca)	%1σ	36/37(ca)	%1σ	40/39(k)	%1σ	38/39(k)	%1σ	36/38(cl)	%1σ	K/Ca	%1σ	K/Cl	%1σ	Ca/Cl	%1σ
17D30450	1.8 %	296.73	0.178	0.018	35	0.1869	0	1.493	3	0.000643	0.92	0.00018	9.63	0.00027	0.17	0.000607	9.65	0.012077	0.09	0	0	0.43	0	0	0	0	0
17D30452	1.9 %	296.73	0.178	0.018	35	0.1869	0	1.493	3	0.000643	0.92	0.00018	9.63	0.00027	0.17	0.000607	9.65	0.012077	0.09	0	0	0.43	0	0	0	0	0
17D30453	2.0 %	296.73	0.178	0.018	35	0.1869	0	1.493	3	0.000643	0.92	0.00018	9.63	0.00027	0.17	0.000607	9.65	0.012077	0.09	0	0	0.43	0	0	0	0	0
17D30455	2.2 %	296.73	0.178	0.018	35	0.1869	0	1.493	3	0.000643	0.92	0.00018	9.63	0.00027	0.17	0.000607	9.65	0.012077	0.09	0	0	0.43	0	0	0	0	0
17D30456	2.4 %	296.73	0.178	0.018	35	0.1869	0	1.493	3	0.000643	0.92	0.00018	9.63	0.00027	0.17	0.000607	9.65	0.012077	0.09	0	0	0.43	0	0	0	0	0
17D30458	2.7 %	296.73	0.178	0.018	35	0.1869	0	1.493	3	0.000643	0.92	0.00018	9.63	0.00027	0.17	0.000607	9.65	0.012077	0.09	0	0	0.43	0	0	0	0	0
17D30459	3.0 %	296.73	0.178	0.018	35	0.1869	0	1.493	3	0.000643	0.92	0.00018	9.63	0.00027	0.17	0.000607	9.65	0.012077	0.09	0	0	0.43	0	0	0	0	0
17D30461	3.4 %	296.73	0.178	0.018	35	0.1869	0	1.493	3	0.000643	0.92	0.00018	9.63	0.00027	0.17	0.000607	9.65	0.012077	0.09	0	0	0.43	0	0	0	0	0
17D30462	3.9 %	296.73	0.178	0.018	35	0.1869	0	1.493	3	0.000643	0.92	0.00018	9.63	0.00027	0.17	0.000607	9.65	0.012077	0.09	0	0	0.43	0	0	0	0	0
17D30464	4.5 %	296.73	0.178	0.018	35	0.1869	0	1.493	3	0.000643	0.92	0.00018	9.63	0.00027	0.17	0.000607	9.65	0.012077	0.09	0	0	0.43	0	0	0	0	0
17D30465	5.2 %	296.73	0.178	0.018	35	0.1869	0	1.493	3	0.000643	0.92	0.00018	9.63	0.00027	0.17	0.000607	9.65	0.012077	0.09	0	0	0.43	0	0	0	0	0
17D30467	6.0 %	296.73	0.178	0.018	35	0.1869	0	1.493	3	0.000643	0.92	0.00018	9.63	0.00027	0.17	0.000607	9.65	0.012077	0.09	0	0	0.43	0	0	0	0	0
17D30468	6.9 %	296.73	0.178	0.018	35	0.1869	0	1.493	3	0.000643	0.92	0.00018	9.63	0.00027	0.17	0.000607	9.65	0.012077	0.09	0	0	0.43	0	0	0	0	0
17D30470	7.9 %	296.73	0.178	0.018	35	0.1869	0	1.493	3	0.000643	0.92	0.00018	9.63	0.00027	0.17	0.000607	9.65	0.012077	0.09	0	0	0.43	0	0	0	0	0
17D30471	9.0 %	296.73	0.178	0.018	35	0.1869	0	1.493	3	0.000643	0.92	0.00018	9.63	0.00027	0.17	0.000607	9.65	0.012077	0.09	0	0	0.43	0	0	0	0	0
17D30473	10.3 %	296.73	0.178	0.018	35	0.1869	0	1.493	3	0.000643	0.92	0.00018	9.63	0.00027	0.17	0.000607	9.65	0.012077	0.09	0	0	0.43	0	0	0	0	0
17D30474	11.6 %	296.73	0.178	0.018	35	0.1869	0	1.493	3	0.000643	0.92	0.00018	9.63	0.00027	0.17	0.000607	9.65	0.012077	0.09	0	0	0.43	0	0	0	0	0
17D30476	12.5 %	296.73	0.178	0.018	35	0.1869	0	1.493	3	0.000643	0.92	0.00018	9.63	0.00027	0.17	0.000607	9.65	0.012077	0.09	0	0	0.43	0	0	0	0	0
17D30477	13.4 %	296.73	0.178	0.018	35	0.1869	0	1.493	3	0.000643	0.92	0.00018	9.63	0.00027	0.17	0.000607	9.65	0.012077	0.09	0	0	0.43	0	0	0	0	0
17D30479	14.6 %	296.73	0.178	0.018	35	0.1869	0	1.493	3	0.000643	0.92	0.00018	9.63	0.00027	0.17	0.000607	9.65	0.012077	0.09	0	0	0.43	0	0	0	0	0
17D30480	16.0 %	296.73	0.178	0.018	35	0.1869	0	1.493	3	0.000643	0.92	0.00018	9.63	0.00027	0.17	0.000607	9.65	0.012077	0.09	0	0	0.43	0	0	0	0	0
17D30482	17.6 %	296.73	0.178	0.018	35	0.1869	0	1.493	3	0.000643	0.92	0.00018	9.63	0.00027	0.17	0.000607	9.65	0.012077	0.09	0	0	0.43	0	0	0	0	0
17D30483	19.3 %	296.73	0.178	0.018	35	0.1869	0	1.493	3	0.000643	0.92	0.00018	9.63	0.00027	0.17	0.000607	9.65	0.012077	0.09	0	0	0.43	0	0	0	0	0
17D30485	21.0 %	296.73	0.178	0.018	35	0.1869	0	1.493	3	0.000643	0.92	0.00018	9.63	0.00027	0.17	0.000607	9.65	0.012077	0.09	0	0	0.43	0	0	0	0	0

OSU Argon Geochronology Lab
CEOAS Oregon State University, Corvallis, USA

17D30446.AGE >>> PS59-244-002 >>> ARCTIC | O-CONNOR (16-22) PROJECT

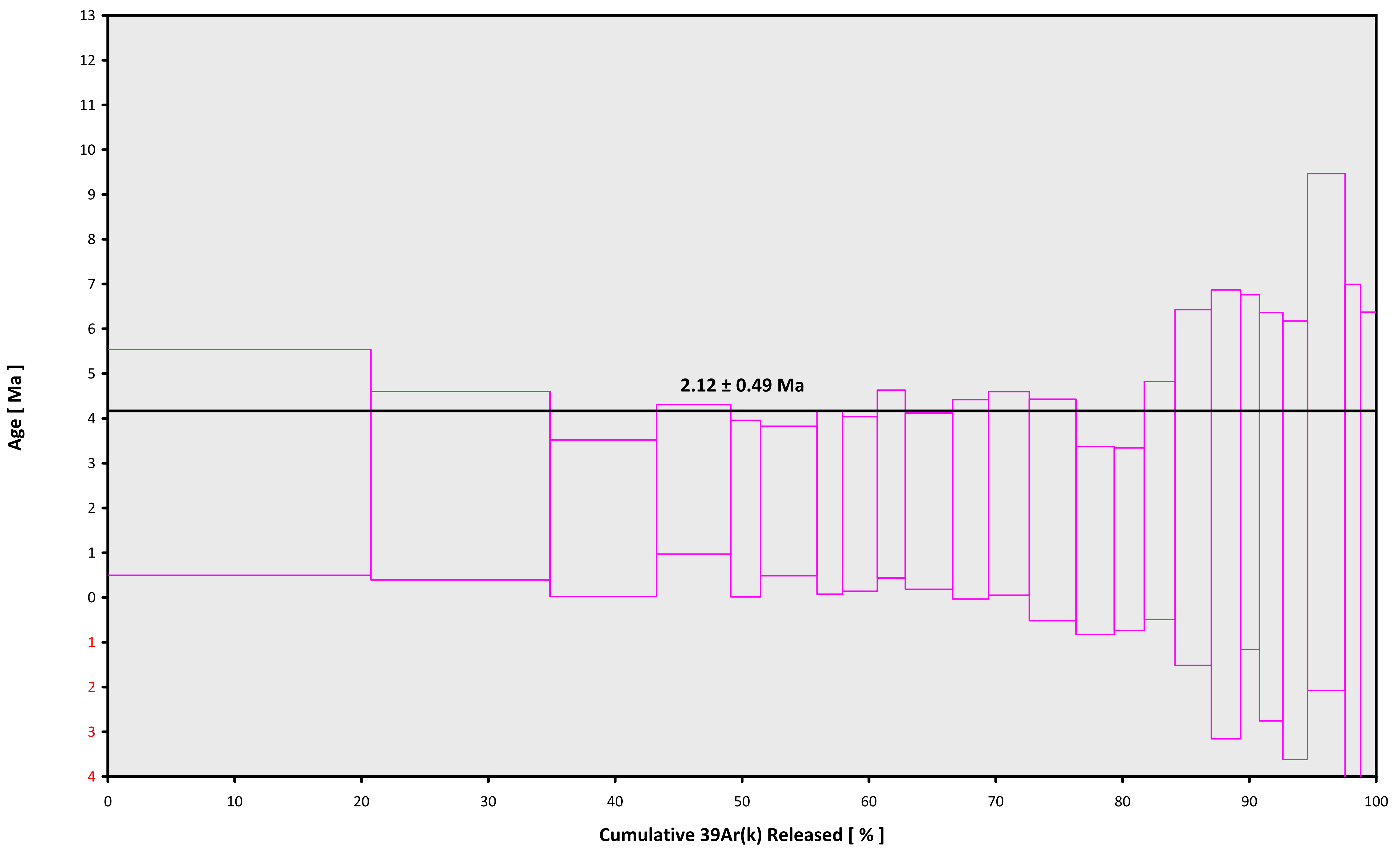

Ar-Ages in Ma

WEIGHTED PLATEAU
2.12 ± 0.49
TOTAL FUSION
2.34 ± 0.73
NORMAL ISOCHRON
2.13 ± 1.19
INVERSE ISOCHRON
2.13 ± 1.01

MSWD (PROBABILITY)
0.16 (100%)

Sample Info

Groundmass
Gakkel Ridge
Dan Miggins

IRR = 17-OSU-05 (5A41-17)
J = 0.00148357 ± 0.00000156

17D30446.full.xls printed at 17-09-2017 (14:32)
ArArCALC v2.7.0 -- Beta Version

17D30446.AGE >>> PS59-244-002 >>> ARCTIC | O-CONNOR (16-22) PROJECT

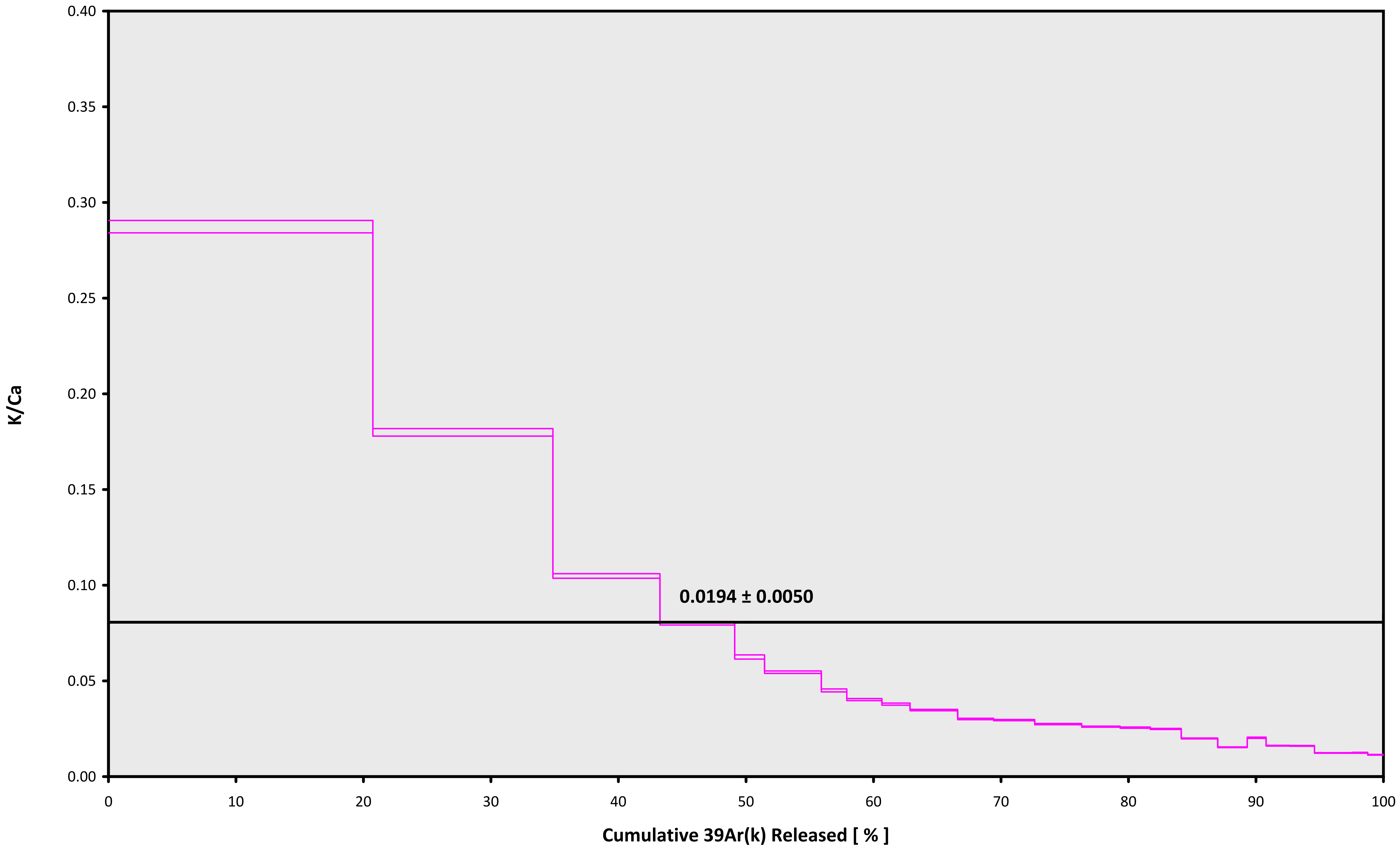

Ar-Ages in Ma

WEIGHTED PLATEAU
2.12 ± 0.49
TOTAL FUSION
2.34 ± 0.73
NORMAL ISOCHRON
2.13 ± 1.19
INVERSE ISOCHRON
2.13 ± 1.01

Sample Info

Groundmass
Gakkel Ridge
Dan Miggins

IRR = 17-OSU-05 (5A41-17)
J = 0.00148357 ± 0.00000156

17D30446.AGE >>> PS59-244-002 >>> ARCTIC | O-CONNOR (16-22) PROJECT

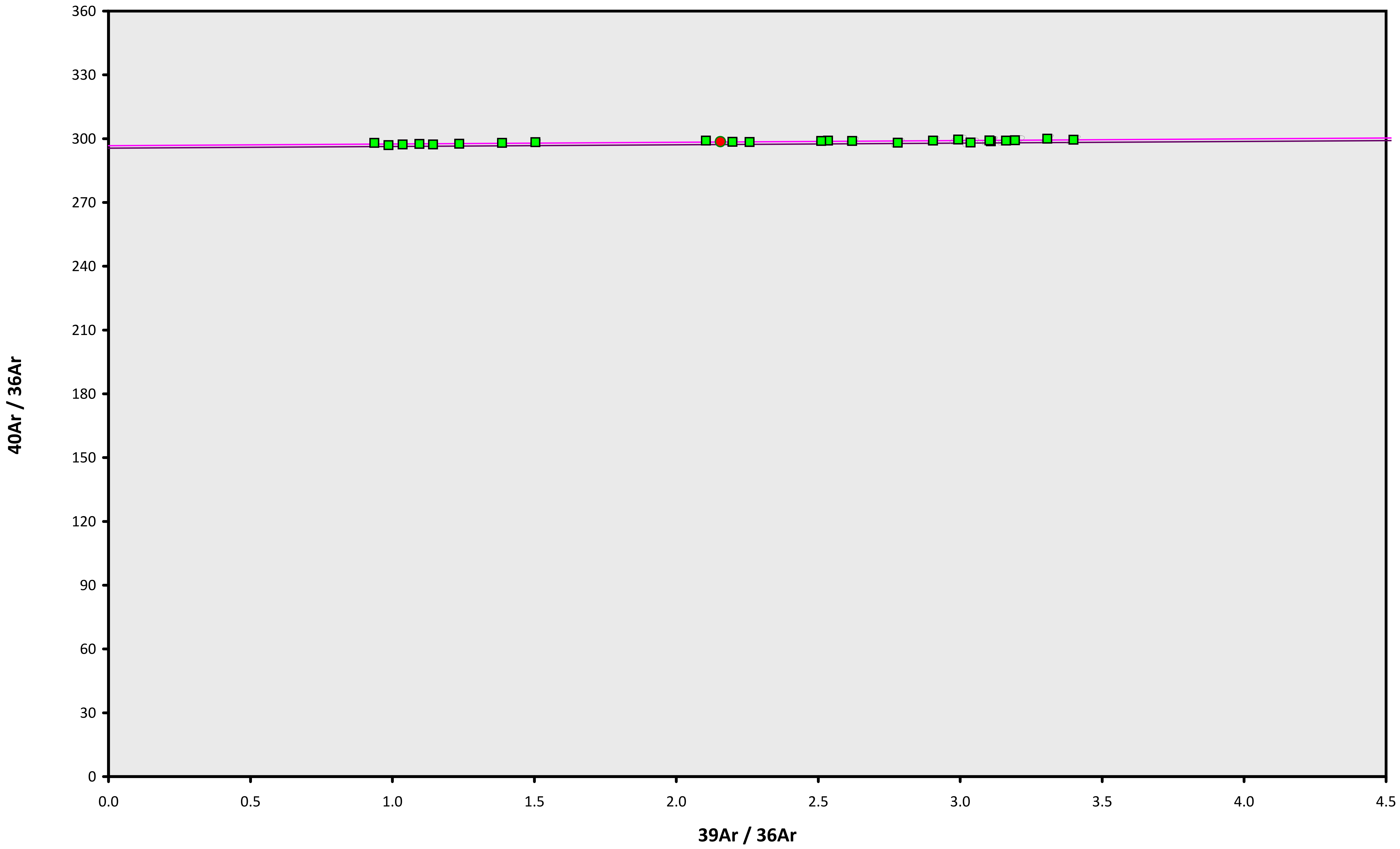

Ar-Ages in Ma

WEIGHTED PLATEAU
2.12 ± 0.49
TOTAL FUSION
2.34 ± 0.73
NORMAL ISOCHRON
2.13 ± 1.19
INVERSE ISOCHRON
2.13 ± 1.01

MSWD (PROBABILITY)
0.22 (100%)

40AR/36AR INTERCEPT
296.7 ± 1.1

Sample Info

Groundmass
Gakkel Ridge
Dan Miggins

IRR = 17-OSU-05 (5A41-17)
J = 0.00148357 ± 0.00000156

17D30446.AGE >>> PS59-244-002 >>> ARCTIC | O-CONNOR (16-22) PROJECT

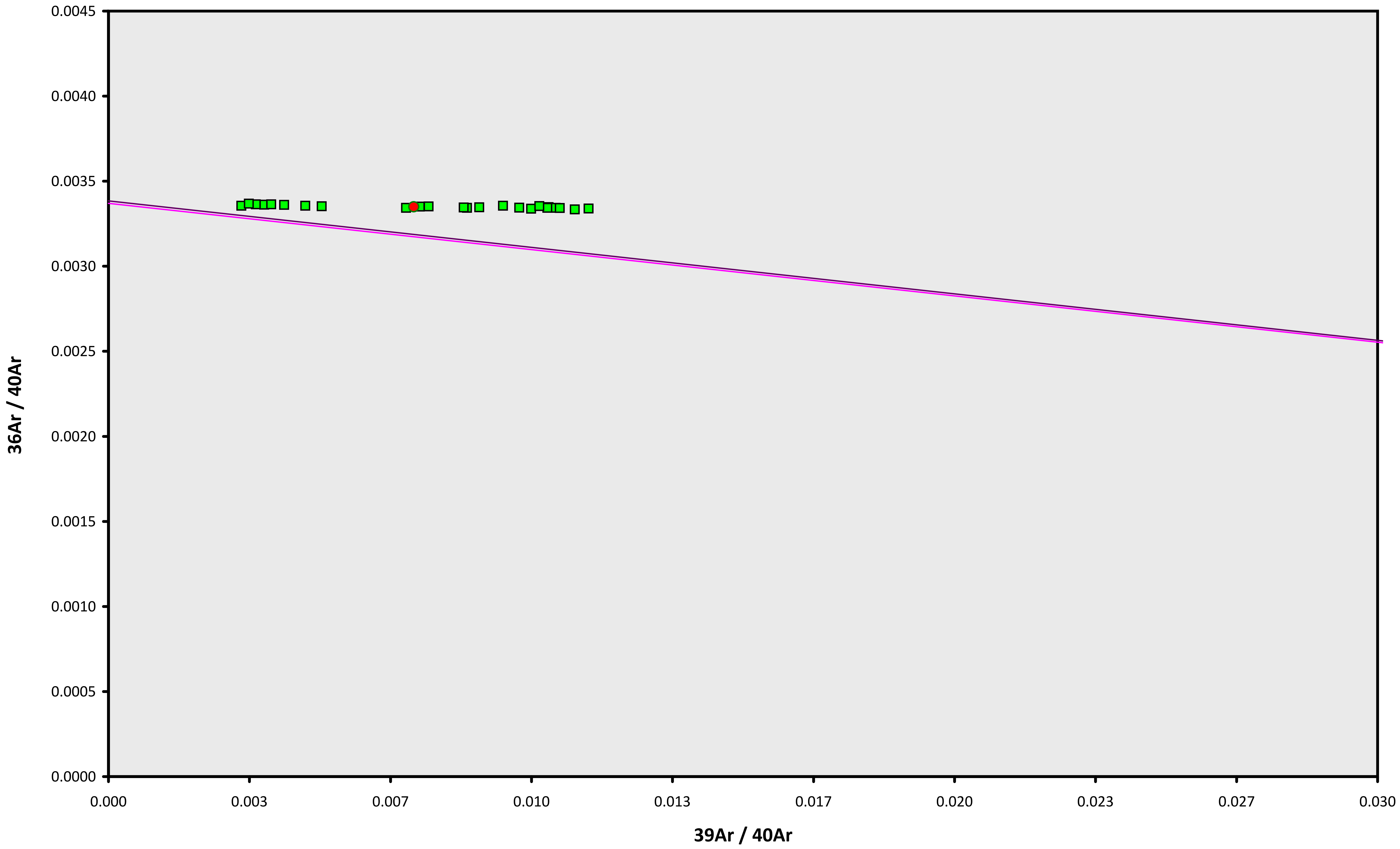

Ar-Ages in Ma

WEIGHTED PLATEAU
2.12 ± 0.49
TOTAL FUSION
2.34 ± 0.73
NORMAL ISOCHRON
2.13 ± 1.19
INVERSE ISOCHRON
2.13 ± 1.01

MSWD (PROBABILITY)
0.22 (100%)

SPREADING FACTOR
0.7%

40AR/36AR INTERCEPT
296.7 ± 1.1

Sample Info

Groundmass
Gakkel Ridge
Dan Miggins

IRR = 17-OSU-05 (5A41-17)
J = 0.00148357 ± 0.00000156

Relative Abundances			36Ar [fA]	%1σ	37Ar [fA]	%1σ	38Ar [fA]	%1σ	39Ar [fA]	%1σ	40Ar [fA]	%1σ	40(r)/39(k) ± 2σ	Age ± 2σ (Ma)	40Ar(r) (%)	39Ar(k) (%)	K/Ca ± 2σ
17D30138	1.8 %		0.242022	0.467	13.5253	2.453	0.115310	19.685	3.66919	0.614	72.4869	0.194	0.25041 ± 0.37265	0.70 ± 1.05	1.26	0.69	0.116 ± 0.006
17D30140	1.9 %		0.242303	0.473	22.9498	1.517	0.170119	13.914	6.32416	0.370	74.1377	0.191	0.51475 ± 0.21563	1.45 ± 0.61	4.38	1.19	0.118 ± 0.004
17D30141	2.0 %		0.077781	1.013	7.7365	4.416	0.054988	43.847	2.10465	1.086	23.1296	0.575	0.19205 ± 0.31257	0.54 ± 0.88	1.74	0.40	0.117 ± 0.011
17D30143	2.2 %		0.058608	1.308	6.6134	5.208	0.033389	69.636	1.78795	1.287	17.4688	0.761	0.22831 ± 0.33667	0.64 ± 0.95	2.33	0.34	0.116 ± 0.012
17D30144	2.4 %		0.194690	0.537	26.6236	1.362	0.168541	14.960	7.34289	0.324	60.5501	0.228	0.58058 ± 0.15507	1.63 ± 0.44	7.02	1.38	0.118 ± 0.003
17D30146	2.7 %		0.075919	1.042	12.3241	2.847	0.052390	46.188	3.17632	0.729	23.0691	0.577	0.40174 ± 0.20505	1.13 ± 0.58	5.52	0.60	0.111 ± 0.006
17D30147	3.0 %		0.152814	0.643	23.3375	1.515	0.172371	14.408	6.58450	0.346	48.0637	0.280	0.62031 ± 0.14624	1.75 ± 0.41	8.48	1.24	0.121 ± 0.004
17D30149	3.4 %		0.337622	0.396	40.8724	0.933	0.253285	9.469	11.55317	0.211	108.1878	0.134	0.87878 ± 0.15536	2.47 ± 0.44	9.36	2.17	0.121 ± 0.002
17D30150	3.9 %	✓	0.469415	0.354	56.7437	0.707	0.404989	5.762	15.91626	0.153	152.4726	0.102	1.01598 ± 0.15271	2.86 ± 0.43	10.58	2.99	0.120 ± 0.002
17D30152	4.5 %	✓	0.398688	0.390	48.5295	0.840	0.310837	7.798	13.38488	0.187	130.3701	0.116	1.09322 ± 0.15737	3.07 ± 0.44	11.20	2.52	0.118 ± 0.002
17D30153	5.2 %	✓	0.433920	0.373	54.4794	0.749	0.329675	7.363	15.07416	0.168	143.7181	0.108	1.18687 ± 0.15051	3.34 ± 0.42	12.42	2.83	0.119 ± 0.002
17D30155	6.0 %	✓	1.218821	0.293	154.0347	0.455	0.950448	2.550	40.31562	0.085	398.9924	0.035	1.13215 ± 0.15091	3.18 ± 0.42	11.41	7.58	0.112 ± 0.001
17D30156	6.9 %	✓	0.414792	0.369	63.5278	0.684	0.376912	6.534	16.02704	0.158	137.6935	0.114	1.14500 ± 0.13442	3.22 ± 0.38	13.29	3.01	0.108 ± 0.002
17D30158	7.9 %	✓	1.005357	0.296	165.5168	0.447	0.911699	2.799	39.94690	0.085	336.0673	0.041	1.19554 ± 0.12470	3.36 ± 0.35	14.17	7.51	0.104 ± 0.001
17D30159	9.0 %	✓	0.588140	0.331	107.2273	0.515	0.557136	4.347	25.18557	0.115	196.9887	0.071	1.15837 ± 0.11749	3.26 ± 0.33	14.77	4.73	0.101 ± 0.001
17D30161	10.3 %	✓	2.042027	0.268	583.8906	0.395	2.429109	0.981	112.59284	0.064	694.3747	0.020	1.14609 ± 0.08611	3.22 ± 0.24	18.52	21.15	0.083 ± 0.001
17D30162	11.6 %	✓	0.872650	0.300	368.8453	0.403	1.157821	2.078	54.24287	0.077	290.3145	0.047	1.07804 ± 0.07516	3.03 ± 0.21	20.05	10.18	0.063 ± 0.001
17D30164	12.5 %	✓	0.942241	0.300	404.9680	0.401	1.235131	2.014	57.35891	0.074	311.4919	0.044	1.07594 ± 0.07661	3.03 ± 0.22	19.72	10.76	0.061 ± 0.000
17D30165	13.4 %	✓	0.607257	0.326	276.8137	0.412	0.690235	3.407	30.62864	0.097	192.8973	0.070	1.08456 ± 0.09350	3.05 ± 0.26	17.12	5.74	0.047 ± 0.000
17D30167	14.6 %		0.591911	0.330	376.6802	0.402	0.603976	4.073	27.02263	0.105	172.6765	0.078	0.95280 ± 0.09943	2.68 ± 0.28	14.78	5.05	0.031 ± 0.000
17D30168	16.0 %		0.353917	0.414	108.9844	0.500	0.210014	11.365	6.23231	0.357	101.6521	0.132	0.68692 ± 0.29462	1.93 ± 0.83	4.16	1.16	0.024 ± 0.000
17D30170	17.6 %		0.602376	0.343	508.0088	0.397	0.622475	3.853	23.94897	0.116	160.3194	0.084	0.87543 ± 0.11003	2.46 ± 0.31	12.90	4.45	0.020 ± 0.000
17D30171	19.3 %		0.323575	0.410	101.1713	0.514	0.177008	13.649	5.72313	0.402	92.7321	0.144	0.66953 ± 0.29305	1.88 ± 0.82	4.09	1.07	0.024 ± 0.000
17D30173	21.0 %		0.302058	0.434	147.4137	0.460	0.227270	10.662	6.85496	0.341	84.2870	0.159	0.82216 ± 0.22439	2.31 ± 0.63	6.59	1.27	0.020 ± 0.000
		Σ	12.548904	0.082	3680.8180	0.127	12.215130	0.969	532.99851	0.028	4024.1418	0.017					

Information on Analysis and Constants Used in Calculations

Project = **O-CONNOR (16-22)**
Sample = **HLY0102-D35-1**
Material = **Groundmass**
Location = **Gakkel Ridge**
Region = **Artic Ocean**
Analyst = **Dan Miggins**
Irradiation = **17-OSU-05 (5A29-17)**
Position = **X: 0 | Y: 0 | Z/H: 42.47709 mm**
FCT-NM Age = **28.201 ± 0.023 Ma**
FCT-NM Reference = **Kuiper et al (2008)**
FCT-NM 40Ar/39Ar Ratio = **10.09639 ± 0.01030**
FCT-NM J-value = **0.00155673 ± 0.00000159**
Air Shot 40Ar/36Ar = **302.7650 ± 0.1605**
Air Shot MDF = **0.99399337 ± 0.00059333 (LIN)**
Experiment Type = **Incremental Heating**
Extraction Method = **Bulk Laser Heating**
Heating = **64 sec**
Isolation = **3.00 min**
Instrument = **ARGUS-VI-D**
Preferred Age = **Plateau Age**
Age Classification = **Crystallization Age**
IGSN = Undefined
Rock Class = **Igneous>Volcanic**
Lithology = **Basaltic Lava**
Lat-Lon = Undefined - Undefined

Age Equations = **Min et al. (2000)**
Negative Intensities = **Allowed**
Collector Calibrations = **36Ar**
Decay 40K = **5.530 ± 0.048 E-10 1/a**
Decay 39Ar = **2.940 ± 0.016 E-07 1/h**
Decay 37Ar = **8.230 ± 0.012 E-04 1/h**
Decay 36Cl = **2.257 ± 0.015 E-06 1/a**
Decay 40K(EC,β⁺) = **0.580 ± 0.009 E-10 1/a**
Decay 40K(β⁻) = **4.950 ± 0.043 E-10 1/a**
Atmospheric 40/36(a) = **300.25 ± 2.41**
Atmospheric 38/36(a) = **0.1869**
Production 39/37(ca) = **0.0006425 ± 0.0000059**
Production 38/37(ca) = **0.0001800 ± 0.0000173**
Production 36/37(ca) = **0.0002703 ± 0.0000005**
Production 40/39(k) = **0.000607 ± 0.000059**
Production 38/39(k) = **0.012077 ± 0.000011**
Production 36/38(cl) = **262.80 ± 1.71**
Scaling Ratio K/Ca = **0.430**
Abundance Ratio 40K/K = **1.1700 ± 0.0100 E-04**
Atomic Weight K = **39.0983 ± 0.0001 g**

Results

Results	40(a)/36(a) ± 2σ	40(r)/39(k) ± 2σ	Age ± 2σ (Ma)	MSWD	39Ar(k) (%,n)	K/Ca ± 2σ
**Age Plateau**		1.11114 ± 0.03227<br>± 2.90%	**3.13** ± 0.09<br>± 2.91%	0.80<br>63%	79.00<br>11	0.069 ± 0.014
			Full External Error ± 0.12	1.89	2σ Confidence Limit	
			Analytical Error ± 0.09	1.0000	Error Magnification	
**Total Fusion Age**		1.04576 ± 0.03038<br>± 2.91%	**2.94** ± 0.09<br>± 2.91%		24	0.062 ± 0.000
			Full External Error ± 0.11			
			Analytical Error ± 0.09			
**Normal Isochron**<br>Error Chron	**303.74** ± 5.26<br>± 1.73%	1.04626 ± 0.10498<br>± 10.03%	**2.94** ± 0.30<br>± 10.03%	4.64<br>0%	79.00<br>11	
			Full External Error ± 0.30	1.94	2σ Confidence Limit	
			Analytical Error ± 0.30	2.1538	Error Magnification	
				4	Number of Iterations	
				0.0000091804	Convergence	
**Inverse Isochron**<br>Error Chron	**303.82** ± 5.26<br>± 1.73%	1.04538 ± 0.10345<br>± 9.90%	**2.94** ± 0.29<br>± 9.89%	4.61<br>0%	79.00<br>11	
			Full External Error ± 0.30	1.94	2σ Confidence Limit	
			Analytical Error ± 0.29	2.1475	Error Magnification	
				3	Number of Iterations	
				0.0002506894	Convergence	
				9%	Spreading Factor	

Notes
Excess Initial 40Ar/36Ar = 300.25 ± 0.80 (%SD).

Incremental Heating			36Ar(a) [fA]	37Ar(ca) [fA]	38Ar(cl) [fA]	39Ar(k) [fA]	40Ar(r) [fA]	Age ± 2σ (Ma)	40Ar(r) (%)	39Ar(k) (%)	K/Ca ± 2σ
17D30138	1.8 %		0.238361	13.5253	0.0241181	3.66050	0.91661	0.70 ± 1.05	1.26	0.69	0.116 ± 0.006
17D30140	1.9 %		0.236090	22.9498	0.0456643	6.30941	3.24774	1.45 ± 0.61	4.38	1.19	0.118 ± 0.004
17D30141	2.0 %		0.075687	7.7365	0.0140916	2.09968	0.40324	0.54 ± 0.88	1.74	0.40	0.117 ± 0.011
17D30143	2.2 %		0.056821	6.6134	0.0000366	1.78370	0.40723	0.64 ± 0.95	2.33	0.34	0.116 ± 0.012
17D30144	2.4 %		0.187485	26.6236	0.0402346	7.32579	4.25321	1.63 ± 0.44	7.02	1.38	0.118 ± 0.003
17D30146	2.7 %		0.072587	12.3241	0.0000000	3.16840	1.27287	1.13 ± 0.58	5.52	0.60	0.111 ± 0.006
17D30147	3.0 %		0.146493	23.3375	0.0614505	6.56950	4.07513	1.75 ± 0.41	8.48	1.24	0.121 ± 0.004
17D30149	3.4 %		0.326565	40.8724	0.0456822	11.52691	10.12962	2.47 ± 0.44	9.36	2.17	0.121 ± 0.002
17D30150	3.9 %	✓	0.454053	56.7437	0.1181328	15.87980	16.13350	2.86 ± 0.43	10.58	2.99	0.120 ± 0.002
17D30152	4.5 %	✓	0.385557	48.5295	0.0687687	13.35370	14.59852	3.07 ± 0.44	11.20	2.52	0.118 ± 0.002
17D30153	5.2 %	✓	0.419182	54.4794	0.0598955	15.03915	17.84947	3.34 ± 0.42	12.42	2.83	0.119 ± 0.002
17D30155	6.0 %	✓	1.177142	154.0347	0.2170179	40.21665	45.53114	3.18 ± 0.42	11.41	7.58	0.112 ± 0.001
17D30156	6.9 %	✓	0.397600	63.5278	0.0981003	15.98622	18.30425	3.22 ± 0.38	13.29	3.01	0.108 ± 0.002
17D30158	7.9 %	✓	0.960573	165.5168	0.2212202	39.84056	47.63108	3.36 ± 0.35	14.17	7.51	0.104 ± 0.001
17D30159	9.0 %	✓	0.559131	107.2273	0.1299995	25.11667	29.09451	3.26 ± 0.33	14.77	4.73	0.101 ± 0.001
17D30161	10.3 %	✓	1.884078	583.8906	0.6166216	112.21769	128.61201	3.22 ± 0.24	18.52	21.15	0.083 ± 0.001
17D30162	11.6 %	✓	0.772893	368.8453	0.2947460	54.00589	58.22071	3.03 ± 0.21	20.05	10.18	0.063 ± 0.001
17D30164	12.5 %	✓	0.832715	404.9680	0.3170207	57.09872	61.43459	3.03 ± 0.22	19.72	10.76	0.061 ± 0.000
17D30165	13.4 %	✓	0.532400	276.8137	0.1731492	30.45079	33.02569	3.05 ± 0.26	17.12	5.74	0.047 ± 0.000
17D30167	14.6 %		0.490070	376.6802	0.1211501	26.78061	25.51664	2.68 ± 0.28	14.78	5.05	0.031 ± 0.000
17D30168	16.0 %		0.324448	108.9844	0.0553352	6.16228	4.23298	1.93 ± 0.83	4.16	1.16	0.024 ± 0.000
17D30170	17.6 %		0.465029	508.0088	0.1588298	23.62258	20.68002	2.46 ± 0.31	12.90	4.45	0.020 ± 0.000
17D30171	19.3 %		0.296221	101.1713	0.0351004	5.65813	3.78828	1.88 ± 0.82	4.09	1.07	0.024 ± 0.000
17D30173	21.0 %		0.262198	147.4137	0.0700873	6.76025	5.55798	2.31 ± 0.63	6.59	1.27	0.020 ± 0.000
		Σ	11.553381	3680.8180	2.9864533	530.63358	554.91701				

Information on Analysis

Project = **O-CONNOR (16-22)**
Sample = **HLY0102-D35-1**
Material = **Groundmass**
Location = **Gakkel Ridge**
Region = **Artic Ocean**
Analyst = **Dan Miggins**
Irradiation = **17-OSU-05 (5A29-17)**
J = **0.00155673 ± 0.00000159**
FCT-NM = **28.201 ± 0.023 Ma**

Results	40(r)/39(k) ± 2σ		Age ± 2σ (Ma)		MSWD	39Ar(k) (%,n)	K/Ca ± 2σ
**Age Plateau**	1.11114	± 0.03227<br>± 2.90%	**3.13**	**± 0.09<br>± 2.91%**	0.80<br>63%	79.00<br>11	0.069 ± 0.014
		Full External Error		± 0.12	1.89	2σ Confidence Limit	
		Analytical Error		± 0.09	1.0000	Error Magnification	
**Total Fusion Age**	1.04576	± 0.03038<br>± 2.91%	**2.94**	**± 0.09<br>± 2.91%**		24	0.062 ± 0.000
		Full External Error		± 0.11			
		Analytical Error		± 0.09			

Normal Isochron			39(k)/36(a) ± 2σ	40(a+r)/36(a) ± 2σ	r.i.
17D30138	1.8 %		15.36 ± 0.24	304.10 ± 3.12	0.5659
17D30140	1.9 %		26.72 ± 0.33	314.01 ± 3.29	0.7409
17D30141	2.0 %		27.74 ± 0.84	305.58 ± 7.31	0.6081
17D30143	2.2 %		31.39 ± 1.18	307.42 ± 9.58	0.6328
17D30144	2.4 %		39.07 ± 0.51	322.94 ± 3.91	0.8014
17D30146	2.7 %		43.65 ± 1.15	317.79 ± 7.88	0.7367
17D30147	3.0 %		44.85 ± 0.68	328.07 ± 4.79	0.8212
17D30149	3.4 %		35.30 ± 0.33	331.27 ± 2.86	0.8448
17D30150	3.9 %	✓	34.97 ± 0.28	335.78 ± 2.56	0.8888
17D30152	4.5 %	✓	34.63 ± 0.31	338.11 ± 2.84	0.8718
17D30153	5.2 %	✓	35.88 ± 0.30	342.83 ± 2.75	0.8832
17D30155	6.0 %	✓	34.16 ± 0.22	338.93 ± 2.07	0.9566
17D30156	6.9 %	✓	40.21 ± 0.34	346.29 ± 2.79	0.8871
17D30158	7.9 %	✓	41.48 ± 0.27	349.84 ± 2.19	0.9563
17D30159	9.0 %	✓	44.92 ± 0.33	352.29 ± 2.51	0.9302
17D30161	10.3 %	✓	59.56 ± 0.36	368.51 ± 2.16	0.9742
17D30162	11.6 %	✓	69.88 ± 0.49	375.58 ± 2.60	0.9667
17D30164	12.5 %	✓	68.57 ± 0.48	374.03 ± 2.60	0.9693
17D30165	13.4 %	✓	57.20 ± 0.45	362.28 ± 2.78	0.9520
17D30167	14.6 %		54.65 ± 0.46	352.32 ± 2.93	0.9507
17D30168	16.0 %		18.99 ± 0.22	313.30 ± 2.96	0.7509
17D30170	17.6 %		50.80 ± 0.48	344.72 ± 3.24	0.9535
17D30171	19.3 %		19.10 ± 0.23	313.04 ± 2.96	0.7067
17D30173	21.0 %		25.78 ± 0.32	321.45 ± 3.41	0.7872

Results	40(a)/36(a) ± 2σ		40(r)/39(k) ± 2σ		Age ± 2σ (Ma)		MSWD
Normal Isochron<br>Error Chron	**303.74**	**± 5.26**<br>**± 1.73%**	1.04626	± 0.10498<br>± 10.03%	**2.94**	**± 0.30**<br>**± 10.03%**	4.64<br>0%
					Full External Error	± 0.30	
					Analytical Error	± 0.30	

Statistics				
	2σ Confidence Limit	1.94	Convergence	0.000009180366
	Error Magnification	2.1538	Number of Iterations	4
	Number of Data Points	11	Calculated Line	Weighted York-2

Inverse Isochron			39(k)/40(a+r) ± 2σ	36(a)/40(a+r) ± 2σ	r.i.
17D30138	1.8 %		0.0505003 ± 0.0006521	0.00328844 ± 0.00003379	0.1139
17D30140	1.9 %		0.0851084 ± 0.0007097	0.00318465 ± 0.00003334	0.1677
17D30141	2.0 %		0.0907841 ± 0.0022350	0.00327249 ± 0.00007826	0.2250
17D30143	2.2 %		0.1021145 ± 0.0030591	0.00325291 ± 0.00010132	0.2480
17D30144	2.4 %		0.1209962 ± 0.0009602	0.00309659 ± 0.00003748	0.2174
17D30146	2.7 %		0.1373550 ± 0.0025572	0.00314678 ± 0.00007801	0.2884
17D30147	3.0 %		0.1366945 ± 0.0012187	0.00304815 ± 0.00004451	0.2417
17D30149	3.4 %		0.1065523 ± 0.0005339	0.00301870 ± 0.00002606	0.1661
17D30150	3.9 %	✓	0.1041551 ± 0.0003838	0.00297812 ± 0.00002267	0.1491
17D30152	4.5 %	✓	0.1024355 ± 0.0004516	0.00295759 ± 0.00002485	0.1446
17D30153	5.2 %	✓	0.1046501 ± 0.0004187	0.00291688 ± 0.00002343	0.1445
17D30155	6.0 %	✓	0.1008017 ± 0.0001856	0.00295047 ± 0.00001805	0.0430
17D30156	6.9 %	✓	0.1161082 ± 0.0004536	0.00288778 ± 0.00002324	0.1646
17D30158	7.9 %	✓	0.1185579 ± 0.0002233	0.00285848 ± 0.00001788	0.0568
17D30159	9.0 %	✓	0.1275130 ± 0.0003462	0.00283861 ± 0.00002023	0.1046
17D30161	10.3 %	✓	0.1616256 ± 0.0002182	0.00271361 ± 0.00001589	0.0200
17D30162	11.6 %	✓	0.1860465 ± 0.0003361	0.00266256 ± 0.00001845	0.0710
17D30164	12.5 %	✓	0.1833276 ± 0.0003187	0.00267361 ± 0.00001858	0.0641
17D30165	13.4 %	✓	0.1578753 ± 0.0003788	0.00276028 ± 0.00002118	0.1055
17D30167	14.6 %		0.1551058 ± 0.0004086	0.00283835 ± 0.00002361	0.1100
17D30168	16.0 %		0.0606235 ± 0.0004667	0.00319186 ± 0.00003017	0.0964
17D30170	17.6 %		0.1473602 ± 0.0004262	0.00290090 ± 0.00002725	0.1027
17D30171	19.3 %		0.0610181 ± 0.0005270	0.00319449 ± 0.00003021	0.1013
17D30173	21.0 %		0.0802091 ± 0.0006109	0.00311093 ± 0.00003297	0.1249

Results	40(a)/36(a) ± 2σ	40(r)/39(k) ± 2σ	Age ± 2σ (Ma)	MSWD
**Inverse Isochron**<br>Error Chron	**303.82** **± 5.26**<br>**± 1.73%**	1.04538 ± 0.10345<br>± 9.90%	**2.94** **± 0.29**<br>**± 9.89%**<br>Full External Error ± 0.30<br>Analytical Error ± 0.29	4.61<br>0%

Statistics				
	2σ Confidence Limit	1.94	Convergence	0.0002506894
	Error Magnification	2.1475	Number of Iterations	3
	Number of Data Points	11	Calculated Line	Weighted York-2
	Spreading Factor	8.9%		

**Degassing Patterns**			36Ar(a) [fA]	%1σ	36Ar(c) [fA]	%1σ	36Ar(ca) [fA]	%1σ	36Ar(cl) [fA]	%1σ	37Ar(ca) [fA]	%1σ	38Ar(a) [fA]	%1σ	38Ar(c) [fA]	%1σ	38Ar(k) [fA]	%1σ	38Ar(ca) [fA]	%1σ	38Ar(cl) [fA]	%1σ	39Ar(k) [fA]	%1σ	39Ar(ca) [fA]	%1σ	40Ar(r) [fA]	%1σ	40Ar(a) [fA]	%1σ	40Ar(c) [fA]	%1σ	40Ar(k) [fA]	%1σ
17D30138	1.8 %		0.238361	0.48	0.0000000	0.00	0.0036559	2.46	0.0000048	94.14	13.5253	2.45	0.0445498	0.48	0.0000000	0.00	0.0442078	0.62	0.0024346	9.94	0.0241181	94.15	3.66050	0.62	0.0086900	2.62	0.91661	74.41	71.5680	0.93	0.0000000	0.00	0.0022219	9.67
17D30140	1.9 %		0.236090	0.49	0.0000000	0.00	0.0062033	1.53	0.0000091	51.86	22.9498	1.52	0.0441253	0.49	0.0000000	0.00	0.0761988	0.38	0.0041310	9.75	0.0456643	51.87	6.30941	0.37	0.0147453	1.77	3.24774	20.94	70.8861	0.94	0.0000000	0.00	0.0038298	9.66
17D30141	2.0 %		0.075687	1.05	0.0000000	0.00	0.0020912	4.42	0.0000028	171.13	7.7365	4.42	0.0141459	1.05	0.0000000	0.00	0.0253578	1.09	0.0013926	10.59	0.0140916	171.13	2.09968	1.09	0.0049707	4.51	0.40324	81.37	22.7250	1.32	0.0000000	0.00	0.0012745	9.71
17D30143	2.2 %		0.056821	1.36	0.0000000	0.00	0.0017876	5.21	0.0000000	######	6.6134	5.21	0.0106198	1.36	0.0000000	0.00	0.0215418	1.29	0.0011904	10.95	0.0000366	######	1.78370	1.29	0.0042491	5.29	0.40723	73.72	17.0605	1.58	0.0000000	0.00	0.0010827	9.74
17D30144	2.4 %		0.187485	0.56	0.0000000	0.00	0.0071964	1.37	0.0000080	62.69	26.6236	1.36	0.0350410	0.56	0.0000000	0.00	0.0884736	0.34	0.0047922	9.73	0.0402346	62.70	7.32579	0.32	0.0171057	1.64	4.25321	13.35	56.2924	0.98	0.0000000	0.00	0.0044468	9.66
17D30146	2.7 %		0.072587	1.10	0.0000000	0.00	0.0033312	2.85	0.0000000	0.00	12.3241	2.85	0.0135666	1.10	0.0000000	0.00	0.0382647	0.74	0.0022183	10.04	0.0000000	0.00	3.16840	0.73	0.0079182	2.99	1.27287	25.51	21.7944	1.36	0.0000000	0.00	0.0019232	9.68
17D30147	3.0 %		0.146493	0.67	0.0000000	0.00	0.0063081	1.52	0.0000123	40.44	23.3375	1.52	0.0273796	0.67	0.0000000	0.00	0.0793399	0.36	0.0042008	9.75	0.0614505	40.45	6.56950	0.35	0.0149944	1.77	4.07513	11.78	43.9846	1.05	0.0000000	0.00	0.0039877	9.66
17D30149	3.4 %		0.326565	0.41	0.0000000	0.00	0.0110478	0.95	0.0000091	52.54	40.8724	0.93	0.0610351	0.41	0.0000000	0.00	0.1392105	0.23	0.0073570	9.68	0.0456822	52.55	11.52691	0.21	0.0262605	1.31	10.12962	8.84	98.0512	0.90	0.0000000	0.00	0.0069968	9.65
17D30150	3.9 %	✓	0.454053	0.37	0.0000000	0.00	0.0153378	0.73	0.0000236	19.80	56.7437	0.71	0.0848625	0.37	0.0000000	0.00	0.1917803	0.18	0.0102139	9.66	0.1181328	19.82	15.87980	0.15	0.0364579	1.16	16.13350	7.51	136.3295	0.88	0.0000000	0.00	0.0096390	9.65
17D30152	4.5 %	✓	0.385557	0.40	0.0000000	0.00	0.0131175	0.86	0.0000138	35.29	48.5295	0.84	0.0720606	0.40	0.0000000	0.00	0.1612727	0.21	0.0087353	9.67	0.0687687	35.30	13.35370	0.19	0.0311802	1.25	14.59852	7.20	115.7635	0.90	0.0000000	0.00	0.0081057	9.65
17D30153	5.2 %	✓	0.419182	0.39	0.0000000	0.00	0.0147258	0.77	0.0000120	40.58	54.4794	0.75	0.0783452	0.39	0.0000000	0.00	0.1816279	0.19	0.0098063	9.66	0.0598955	40.59	15.03915	0.17	0.0350030	1.19	17.84947	6.34	125.8595	0.89	0.0000000	0.00	0.0091288	9.65
17D30155	6.0 %	✓	1.177142	0.30	0.0000000	0.00	0.0416356	0.49	0.0000434	11.28	154.0347	0.45	0.2200078	0.30	0.0000000	0.00	0.4856965	0.12	0.0277262	9.64	0.2170179	11.32	40.21665	0.09	0.0989673	1.03	45.53114	6.66	353.4369	0.86	0.0000000	0.00	0.0244115	9.65
17D30156	6.9 %	✓	0.397600	0.39	0.0000000	0.00	0.0171716	0.70	0.0000196	25.15	63.5278	0.68	0.0743115	0.39	0.0000000	0.00	0.1930656	0.18	0.0114350	9.65	0.0981003	25.17	15.98622	0.16	0.0408166	1.15	18.30425	5.87	119.3795	0.89	0.0000000	0.00	0.0097036	9.65
17D30158	7.9 %	✓	0.960573	0.31	0.0000000	0.00	0.0447392	0.48	0.0000443	11.65	165.5168	0.45	0.1795311	0.31	0.0000000	0.00	0.4811544	0.12	0.0297930	9.64	0.2212202	11.69	39.84056	0.08	0.1063446	1.02	47.63108	5.21	288.4121	0.86	0.0000000	0.00	0.0241832	9.65
17D30159	9.0 %	✓	0.559131	0.35	0.0000000	0.00	0.0289835	0.54	0.0000260	18.71	107.2273	0.51	0.1045015	0.35	0.0000000	0.00	0.3033340	0.15	0.0193009	9.64	0.1299995	18.74	25.11667	0.12	0.0688936	1.05	29.09451	5.07	167.8790	0.87	0.0000000	0.00	0.0152458	9.65
17D30161	10.3 %	✓	1.884078	0.29	0.0000000	0.00	0.1578256	0.43	0.0001234	4.31	583.8906	0.40	0.3521343	0.29	0.0000000	0.00	1.3552530	0.11	0.1051003	9.64	0.6166216	4.41	112.21769	0.06	0.3751497	1.00	128.61201	3.76	565.6945	0.85	0.0000000	0.00	0.0681161	9.65
17D30162	11.6 %	✓	0.772893	0.34	0.0000000	0.00	0.0996989	0.44	0.0000590	8.50	368.8453	0.40	0.1444536	0.34	0.0000000	0.00	0.6522291	0.12	0.0663922	9.64	0.2947460	8.55	54.00589	0.08	0.2369831	1.00	58.22071	3.49	232.0610	0.87	0.0000000	0.00	0.0327816	9.65
17D30164	12.5 %	✓	0.832715	0.34	0.0000000	0.00	0.1094629	0.44	0.0000635	8.21	404.9680	0.40	0.1556344	0.34	0.0000000	0.00	0.6895812	0.12	0.0728942	9.64	0.3170207	8.26	57.09872	0.07	0.2601919	1.00	61.43459	3.56	250.0226	0.87	0.0000000	0.00	0.0346589	9.65
17D30165	13.4 %	✓	0.532400	0.38	0.0000000	0.00	0.0748228	0.45	0.0000347	13.90	276.8137	0.41	0.0995055	0.38	0.0000000	0.00	0.3677542	0.13	0.0498265	9.64	0.1731492	13.93	30.45079	0.10	0.1778528	1.01	33.02569	4.31	159.8531	0.89	0.0000000	0.00	0.0184836	9.65
17D30167	14.6 %		0.490070	0.41	0.0000000	0.00	0.1018166	0.44	0.0000243	21.04	376.6802	0.40	0.0915941	0.41	0.0000000	0.00	0.3234294	0.14	0.0678024	9.64	0.1211501	21.06	26.78061	0.11	0.2420170	1.00	25.51664	5.22	147.1436	0.90	0.0000000	0.00	0.0162558	9.65
17D30168	16.0 %		0.324448	0.45	0.0000000	0.00	0.0294585	0.53	0.0000111	43.29	108.9844	0.50	0.0606393	0.45	0.0000000	0.00	0.0744219	0.37	0.0196172	9.64	0.0553352	43.30	6.16228	0.36	0.0700225	1.05	4.23298	21.44	97.4154	0.92	0.0000000	0.00	0.0037405	9.66
17D30170	17.6 %		0.465029	0.46	0.0000000	0.00	0.1373148	0.43	0.0000318	16.12	508.0088	0.40	0.0869140	0.46	0.0000000	0.00	0.2852899	0.15	0.0914416	9.64	0.1588298	16.14	23.62258	0.12	0.3263957	1.00	20.68002	6.28	139.6250	0.93	0.0000000	0.00	0.0143389	9.65
17D30171	19.3 %		0.296221	0.45	0.0000000	0.00	0.0273466	0.54	0.0000070	69.03	101.1713	0.51	0.0553637	0.45	0.0000000	0.00	0.0683332	0.42	0.0182108	9.64	0.0351004	69.04	5.65813	0.41	0.0650026	1.05	3.78828	21.88	88.9404	0.92	0.0000000	0.00	0.0034345	9.66
17D30173	21.0 %		0.262198	0.51	0.0000000	0.00	0.0398459	0.49	0.0000140	34.78	147.4137	0.46	0.0490048	0.51	0.0000000	0.00	0.0816435	0.36	0.0265345	9.64	0.0700873	34.79	6.76025	0.35	0.0947133	1.03	5.55798	13.64	78.7249	0.95	0.0000000	0.00	0.0041035	9.66
		Σ	11.553381	0.09	0.0000000	0.00	0.9949251	0.14	0.0005978	3.94	3680.8180	0.13	2.1593270	0.09	0.0000000	0.00	6.4084618	0.04	0.6625472	2.92	2.9864533	3.95	530.63358	0.03	2.3649255	0.31	554.91701	1.45	3468.9027	0.23	0.0000000	0.00	0.3220946	2.94
		Σ							12.548904	0.08	3680.8180	0.13									12.216789	0.98			532.99851	0.03							4024.1418	0.28

**Additional Parameters**			**40Ar/39Ar**	**1σ**	**37Ar/39Ar**	**1σ**	**36Ar/39Ar**	**1σ**	**Time (days)**	**37Ar (decay)**	**39Ar (decay)**	**40Ar (moles)**
17D30138	1.8 %		19.755569	0.127267	3.686186	0.093210	0.065961	0.000509	123.106	11.490353	1.00087257	3.479E-12
17D30140	1.9 %		11.722931	0.048788	3.628912	0.056647	0.038314	0.000230	123.120	11.493506	1.00087267	3.559E-12
17D30141	2.0 %		10.989740	0.135023	3.675888	0.167174	0.036957	0.000549	123.127	11.495083	1.00087272	1.110E-12
17D30143	2.2 %		9.770264	0.146080	3.698866	0.198424	0.032780	0.000602	123.141	11.498237	1.00087282	8.385E-13
17D30144	2.4 %		8.246077	0.032665	3.625763	0.050757	0.026514	0.000166	123.148	11.499814	1.00087287	2.906E-12
17D30146	2.7 %		7.262864	0.067499	3.879993	0.114015	0.023901	0.000304	123.162	11.502969	1.00087297	1.107E-12
17D30147	3.0 %		7.299532	0.032493	3.544313	0.055086	0.023208	0.000169	123.169	11.504547	1.00087301	2.307E-12
17D30149	3.4 %		9.364339	0.023421	3.537764	0.033828	0.029223	0.000131	123.183	11.507703	1.00087311	5.193E-12
17D30150	3.9 %	✓	9.579679	0.017618	3.565144	0.025786	0.029493	0.000114	123.190	11.509282	1.00087316	7.319E-12
17D30152	4.5 %	✓	9.740103	0.021432	3.625697	0.031206	0.029786	0.000129	123.203	11.512440	1.00087326	6.258E-12
17D30153	5.2 %	✓	9.534072	0.019041	3.614095	0.027754	0.028786	0.000118	123.210	11.514019	1.00087331	6.898E-12
17D30155	6.0 %	✓	9.896721	0.009088	3.820720	0.017668	0.030232	0.000092	123.225	11.517336	1.00087341	1.915E-11
17D30156	6.9 %	✓	8.591324	0.016750	3.963786	0.027831	0.025881	0.000104	123.232	11.518916	1.00087346	6.609E-12
17D30158	7.9 %	✓	8.412851	0.007901	4.143421	0.018841	0.025167	0.000077	123.246	11.522077	1.00087356	1.613E-11
17D30159	9.0 %	✓	7.821492	0.010594	4.257491	0.022460	0.023352	0.000082	123.253	11.523657	1.00087361	9.455E-12
17D30161	10.3 %	✓	6.167130	0.004145	5.185859	0.020758	0.018136	0.000050	123.267	11.526819	1.00087371	3.333E-11
17D30162	11.6 %	✓	5.352122	0.004812	6.799885	0.027879	0.016088	0.000050	123.274	11.528400	1.00087376	1.394E-11
17D30164	12.5 %	✓	5.430575	0.004697	7.060246	0.028776	0.016427	0.000051	123.287	11.531563	1.00087385	1.495E-11
17D30165	13.4 %	✓	6.297937	0.007517	9.037742	0.038262	0.019826	0.000067	123.294	11.533145	1.00087390	9.259E-12
17D30167	14.6 %		6.390071	0.008348	13.939435	0.057931	0.021904	0.000076	123.308	11.536309	1.00087400	8.288E-12
17D30168	16.0 %		16.310517	0.062124	17.487015	0.107440	0.056788	0.000310	123.315	11.537892	1.00087405	4.879E-12
17D30170	17.6 %		6.694207	0.009549	21.212135	0.087734	0.025152	0.000091	123.329	11.541058	1.00087415	7.695E-12
17D30171	19.3 %		16.203032	0.069241	17.677615	0.115427	0.056538	0.000325	123.336	11.542641	1.00087420	4.451E-12
17D30173	21.0 %		12.295760	0.046260	21.504663	0.123220	0.044064	0.000243	123.350	11.545808	1.00087429	4.046E-12

Procedure Blanks		36Ar ± 1σ (SE) [fA]	37Ar ± 1σ (SE) [fA]	38Ar ± 1σ (SE) [fA]	39Ar ± 1σ (SE) [fA]	40Ar ± 1σ (SE) [fA]
17D30138	1.8 %	0.0126247 ± 0.0005682	0.0386997 ± 0.0229085	0.0501713 ± 0.0168270	0.0540749 ± 0.0158319	3.8007150 ± 0.1312479
17D30140	1.9 %	0.0128897 ± 0.0005682	0.0255688 ± 0.0229085	0.0386038 ± 0.0168270	0.0524648 ± 0.0158319	3.9158804 ± 0.1312479
17D30141	2.0 %	0.0129714 ± 0.0005682	0.0203287 ± 0.0229085	0.0341084 ± 0.0168270	0.0521123 ± 0.0158319	3.9516052 ± 0.1312479
17D30143	2.2 %	0.0130665 ± 0.0005682	0.0124170 ± 0.0229085	0.0274101 ± 0.0168270	0.0520593 ± 0.0158319	3.9912415 ± 0.1312479
17D30144	2.4 %	0.0130926 ± 0.0005682	0.0096984 ± 0.0229085	0.0250940 ± 0.0168270	0.0522679 ± 0.0158319	3.9996488 ± 0.1312479
17D30146	2.7 %	0.0131310 ± 0.0005682	0.0065814 ± 0.0229085	0.0222450 ± 0.0168270	0.0529537 ± 0.0158319	4.0042186 ± 0.1312479
17D30147	3.0 %	0.0131542 ± 0.0005682	0.0061070 ± 0.0229085	0.0215996 ± 0.0168270	0.0533603 ± 0.0158319	4.0043469 ± 0.1312479
17D30149	3.4 %	0.0132336 ± 0.0005682	0.0071005 ± 0.0229085	0.0215862 ± 0.0168270	0.0541500 ± 0.0158319	4.0095417 ± 0.1312479
17D30150	3.9 %	0.0132989 ± 0.0005682	0.0084635 ± 0.0229085	0.0221061 ± 0.0168270	0.0544831 ± 0.0158319	4.0180438 ± 0.1312479
17D30152	4.5 %	0.0135014 ± 0.0005682	0.0126238 ± 0.0229085	0.0239204 ± 0.0168270	0.0549260 ± 0.0158319	4.0547839 ± 0.1312479
17D30153	5.2 %	0.0136460 ± 0.0005682	0.0152873 ± 0.0229085	0.0251034 ± 0.0168270	0.0550060 ± 0.0158319	4.0859273 ± 0.1312479
17D30155	6.0 %	0.0140619 ± 0.0005682	0.0217364 ± 0.0229085	0.0278795 ± 0.0168270	0.0548138 ± 0.0158319	4.1863398 ± 0.1312479
17D30156	6.9 %	0.0143194 ± 0.0005682	0.0250421 ± 0.0229085	0.0292233 ± 0.0168270	0.0545413 ± 0.0158319	4.2533338 ± 0.1312479
17D30158	7.9 %	0.0149615 ± 0.0005682	0.0316548 ± 0.0229085	0.0316719 ± 0.0168270	0.0536494 ± 0.0158319	4.4297889 ± 0.1312479
17D30159	9.0 %	0.0153501 ± 0.0005682	0.0347695 ± 0.0229085	0.0326668 ± 0.0168270	0.0530419 ± 0.0158319	4.5410776 ± 0.1312479
17D30161	10.3 %	0.0162700 ± 0.0005682	0.0400984 ± 0.0229085	0.0339242 ± 0.0168270	0.0515588 ± 0.0158319	4.8136835 ± 0.1312479
17D30162	11.6 %	0.0168035 ± 0.0005682	0.0420915 ± 0.0229085	0.0340773 ± 0.0168270	0.0507154 ± 0.0158319	4.9762980 ± 0.1312479
17D30164	12.5 %	0.0180209 ± 0.0005682	0.0441460 ± 0.0229085	0.0331605 ± 0.0168270	0.0489308 ± 0.0158319	5.3567312 ± 0.1312479
17D30165	13.4 %	0.0187053 ± 0.0005682	0.0439573 ± 0.0229085	0.0319820 ± 0.0168270	0.0480423 ± 0.0158319	5.5753172 ± 0.1312479
17D30167	14.6 %	0.0202245 ± 0.0005682	0.0404871 ± 0.0229085	0.0279143 ± 0.0168270	0.0464299 ± 0.0158319	6.0704832 ± 0.1312479
17D30168	16.0 %	0.0210580 ± 0.0005682	0.0369267 ± 0.0229085	0.0249173 ± 0.0168270	0.0457791 ± 0.0158319	6.3473005 ± 0.1312479
17D30170	17.6 %	0.0228677 ± 0.0005682	0.0254219 ± 0.0229085	0.0167282 ± 0.0168270	0.0449962 ± 0.0158319	6.9593338 ± 0.1312479
17D30171	19.3 %	0.0238409 ± 0.0005682	0.0171698 ± 0.0229085	0.0114290 ± 0.0168270	0.0449575 ± 0.0158319	7.2942569 ± 0.1312479
17D30173	21.0 %	0.0259144 ± 0.0005682	0.0051389 ± 0.0229085	0.0018459 ± 0.0168270	0.0458450 ± 0.0158319	8.0205207 ± 0.1312479

**Intercept Values**		**36Ar ± 1σ (SE) [fA]**	**r2**	**Regression (type,n)**	**37Ar ± 1σ (SE) [fA]**	**r2**	**Regression (type,n)**	**38Ar ± 1σ (SE) [fA]**	**r2**	**Regression (type,n)**	**39Ar ± 1σ (SE) [fA]**	**r2**	**Regression (type,n)**	**40Ar ± 1σ (SE) [fA]**	**r2**	**Regression (type,n)**
17D30138	1.8 %	0.2440115 ± 0.0007271	0.6714	EXP 150 of 150	1.194620 ± 0.016087	0.1921	EXP 150 of 150	0.1640966 ± 0.0148252	0.0362	EXP 150 of 150	3.6980846 ± 0.0156698	0.4856	EXP 150 of 150	76.287577 ± 0.051237	0.9423	EXP 150 of 150
17D30140	1.9 %	0.2445448 ± 0.0007495	0.6328	EXP 150 of 150	1.986403 ± 0.017351	0.2279	EXP 150 of 150	0.2066798 ± 0.0162392	0.0258	EXP 150 of 150	6.3332280 ± 0.0165608	0.7973	EXP 150 of 150	78.053546 ± 0.053696	0.9528	EXP 150 of 150
17D30141	2.0 %	0.0873344 ± 0.0004603	0.0576	EXP 150 of 150	0.681242 ± 0.017903	0.0475	EXP 150 of 150	0.0884358 ± 0.0168611	0.0042	EXP 150 of 150	2.1423203 ± 0.0162060	0.0120	EXP 150 of 150	27.081158 ± 0.022124	0.9965	EXP 150 of 150
17D30143	2.2 %	0.0690995 ± 0.0004425	0.1271	EXP 150 of 150	0.577234 ± 0.018318	0.0165	EXP 150 of 150	0.0603978 ± 0.0156373	0.0041	EXP 150 of 150	1.8277447 ± 0.0164534	0.0866	EXP 150 of 150	21.460024 ± 0.020611	0.9966	EXP 150 of 150
17D30144	2.4 %	0.1992268 ± 0.0006876	0.4224	EXP 150 of 150	2.283173 ± 0.018850	0.2943	EXP 150 of 150	0.1916109 ± 0.0183684	0.0011	EXP 150 of 150	7.3447761 ± 0.0169525	0.8347	EXP 150 of 150	64.549719 ± 0.043705	0.9732	EXP 150 of 150
17D30146	2.7 %	0.0857134 ± 0.0004665	0.0485	EXP 150 of 150	1.058686 ± 0.018852	0.1395	EXP 150 of 150	0.0740059 ± 0.0169829	0.0185	EXP 150 of 150	3.2074737 ± 0.0165606	0.3427	EXP 150 of 150	27.073368 ± 0.021889	0.9960	EXP 150 of 150
17D30147	3.0 %	0.1592528 ± 0.0006590	0.2464	EXP 150 of 150	1.998154 ± 0.018053	0.2500	EXP 150 of 150	0.1919000 ± 0.0178572	0.0046	EXP 150 of 150	6.5926734 ± 0.0156569	0.8531	EXP 150 of 150	52.068091 ± 0.030721	0.9887	EXP 150 of 150
17D30149	3.4 %	0.3360195 ± 0.0008332	0.7976	EXP 149 of 150	3.494936 ± 0.018654	0.5552	EXP 150 of 150	0.2718287 ± 0.0166810	0.0022	EXP 150 of 150	11.5280450 ± 0.0170219	0.9423	EXP 150 of 150	112.197387 ± 0.061598	0.5752	EXP 150 of 150
17D30150	3.9 %	0.4620858 ± 0.0010057	0.8554	EXP 150 of 150	4.850009 ± 0.017022	0.7461	EXP 150 of 150	0.4222312 ± 0.0157553	0.0284	EXP 150 of 150	15.8615176 ± 0.0156388	0.9741	EXP 150 of 150	156.490654 ± 0.084113	0.2388	EXP 150 of 150
17D30152	4.5 %	0.3946699 ± 0.0010123	0.8089	EXP 150 of 150	4.152173 ± 0.020581	0.5873	EXP 150 of 150	0.3310242 ± 0.0170372	0.0036	EXP 150 of 150	13.3479575 ± 0.0174808	0.9514	EXP 150 of 150	134.424918 ± 0.074399	0.0716	EXP 150 of 150
17D30153	5.2 %	0.4284981 ± 0.0010272	0.8339	EXP 150 of 150	4.661723 ± 0.018933	0.6610	EXP 150 of 150	0.3508185 ± 0.0170830	0.0001	EXP 150 of 150	15.0257183 ± 0.0174234	0.9632	EXP 150 of 150	147.804016 ± 0.082151	0.0092	EXP 150 of 150
17D30155	6.0 %	1.1793234 ± 0.0018239	0.9492	EXP 150 of 150	13.155248 ± 0.020280	0.9364	EXP 150 of 150	0.9669120 ± 0.0169927	0.0557	EXP 150 of 150	40.0937668 ± 0.0183884	0.9948	EXP 150 of 150	403.178749 ± 0.045118	0.9979	EXP 150 of 150
17D30156	6.9 %	0.4108835 ± 0.0009429	0.8087	EXP 150 of 150	5.440888 ± 0.020018	0.6860	EXP 150 of 150	0.4016086 ± 0.0175690	0.0169	EXP 150 of 150	15.9715948 ± 0.0171656	0.9679	EXP 150 of 150	141.946820 ± 0.085451	0.4782	EXP 150 of 150
17D30158	7.9 %	0.9761391 ± 0.0015161	0.9411	EXP 150 of 150	14.138365 ± 0.020372	0.9393	EXP 150 of 150	0.9324201 ± 0.0187388	0.0638	EXP 150 of 150	39.7264104 ± 0.0177590	0.9951	EXP 150 of 150	340.497110 ± 0.040943	0.9971	EXP 150 of 150
17D30159	9.0 %	0.5776453 ± 0.0011279	0.9019	EXP 150 of 150	9.172314 ± 0.020383	0.8654	EXP 149 of 150	0.5831109 ± 0.0169974	0.0212	EXP 150 of 150	25.0657674 ± 0.0189363	0.9852	EXP 150 of 150	201.529786 ± 0.049023	0.9494	EXP 150 of 150
17D30161	10.3 %	1.9685635 ± 0.0021157	0.9752	EXP 150 of 150	49.783602 ± 0.019553	0.9954	EXP 150 of 150	2.4338569 ± 0.0162194	0.3754	EXP 150 of 150	111.8717002 ± 0.0215901	0.9991	EXP 150 of 150	699.188352 ± 0.042352	0.9996	EXP 150 of 150
17D30162	11.6 %	0.8511066 ± 0.0013536	0.9379	EXP 150 of 150	31.460892 ± 0.020994	0.9865	EXP 150 of 150	1.1779915 ± 0.0167314	0.1258	EXP 150 of 150	53.9213309 ± 0.0205130	0.9965	EXP 150 of 150	295.290782 ± 0.038608	0.9951	EXP 150 of 150
17D30164	12.5 %	0.9188566 ± 0.0014874	0.9378	EXP 150 of 150	34.530469 ± 0.020926	0.9890	EXP 150 of 150	1.2534557 ± 0.0178528	0.1654	EXP 150 of 150	57.0141902 ± 0.0199288	0.9971	EXP 150 of 150	316.848590 ± 0.039237	0.9970	EXP 150 of 150
17D30165	13.4 %	0.5992776 ± 0.0011291	0.9080	EXP 150 of 150	23.613667 ± 0.020957	0.9776	EXP 150 of 150	0.7139269 ± 0.0159970	0.0946	EXP 149 of 150	30.4664793 ± 0.0170248	0.9920	EXP 150 of 150	198.472570 ± 0.028881	0.9855	EXP 150 of 150
17D30167	14.6 %	0.5861250 ± 0.0011277	0.9143	EXP 150 of 150	32.104673 ± 0.020410	0.9881	EXP 149 of 150	0.6246360 ± 0.0175263	0.0167	EXP 150 of 150	26.8836005 ± 0.0170081	0.9900	EXP 150 of 150	178.746994 ± 0.026949	0.9836	EXP 150 of 150
17D30168	16.0 %	0.3594228 ± 0.0009799	0.8096	EXP 149 of 150	9.312747 ± 0.017704	0.9037	EXP 150 of 150	0.2324084 ± 0.0165192	0.0284	EXP 150 of 150	6.2353123 ± 0.0149780	0.8030	EXP 150 of 150	107.999434 ± 0.029937	0.7797	EXP 150 of 150
17D30170	17.6 %	0.5987730 ± 0.0012739	0.8908	EXP 150 of 150	43.250919 ± 0.020865	0.9931	EXP 150 of 150	0.6317268 ± 0.0166654	0.1326	EXP 150 of 150	23.8296018 ± 0.0174669	0.9867	EXP 150 of 150	167.278721 ± 0.026502	0.9844	EXP 150 of 150
17D30171	19.3 %	0.3331966 ± 0.0008486	0.8278	EXP 150 of 150	8.624464 ± 0.017430	0.8733	EXP 150 of 150	0.1863111 ± 0.0169287	0.0012	EXP 150 of 150	5.7288110 ± 0.0161568	0.7137	EXP 150 of 150	100.026360 ± 0.023520	0.7977	EXP 150 of 150
17D30173	21.0 %	0.3146988 ± 0.0008695	0.8042	EXP 150 of 150	12.532850 ± 0.020217	0.9269	EXP 150 of 150	0.2226944 ± 0.0170260	0.0275	EXP 150 of 150	6.8537612 ± 0.0164954	0.8445	EXP 150 of 150	92.307514 ± 0.026136	0.7931	EXP 150 of 150

Project Info		Analyst	Irradiation	X-pos	Y-pos	Z/H-pos	Project	Experiment	Nmb
17D30138	1.8 %	Dan Miggins	17-OSU-05	0.00	0.00	42.48	Arctic\O-Connor (16-22)	17D30134	01
17D30140	1.9 %	Dan Miggins	17-OSU-05	0.00	0.00	42.48	Arctic\O-Connor (16-22)	17D30134	01
17D30141	2.0 %	Dan Miggins	17-OSU-05	0.00	0.00	42.48	Arctic\O-Connor (16-22)	17D30134	01
17D30143	2.2 %	Dan Miggins	17-OSU-05	0.00	0.00	42.48	Arctic\O-Connor (16-22)	17D30134	01
17D30144	2.4 %	Dan Miggins	17-OSU-05	0.00	0.00	42.48	Arctic\O-Connor (16-22)	17D30134	01
17D30146	2.7 %	Dan Miggins	17-OSU-05	0.00	0.00	42.48	Arctic\O-Connor (16-22)	17D30134	01
17D30147	3.0 %	Dan Miggins	17-OSU-05	0.00	0.00	42.48	Arctic\O-Connor (16-22)	17D30134	01
17D30149	3.4 %	Dan Miggins	17-OSU-05	0.00	0.00	42.48	Arctic\O-Connor (16-22)	17D30134	01
17D30150	3.9 %	Dan Miggins	17-OSU-05	0.00	0.00	42.48	Arctic\O-Connor (16-22)	17D30134	01
17D30152	4.5 %	Dan Miggins	17-OSU-05	0.00	0.00	42.48	Arctic\O-Connor (16-22)	17D30134	01
17D30153	5.2 %	Dan Miggins	17-OSU-05	0.00	0.00	42.48	Arctic\O-Connor (16-22)	17D30134	01
17D30155	6.0 %	Dan Miggins	17-OSU-05	0.00	0.00	42.48	Arctic\O-Connor (16-22)	17D30134	01
17D30156	6.9 %	Dan Miggins	17-OSU-05	0.00	0.00	42.48	Arctic\O-Connor (16-22)	17D30134	01
17D30158	7.9 %	Dan Miggins	17-OSU-05	0.00	0.00	42.48	Arctic\O-Connor (16-22)	17D30134	01
17D30159	9.0 %	Dan Miggins	17-OSU-05	0.00	0.00	42.48	Arctic\O-Connor (16-22)	17D30134	01
17D30161	10.3 %	Dan Miggins	17-OSU-05	0.00	0.00	42.48	Arctic\O-Connor (16-22)	17D30134	01
17D30162	11.6 %	Dan Miggins	17-OSU-05	0.00	0.00	42.48	Arctic\O-Connor (16-22)	17D30134	01
17D30164	12.5 %	Dan Miggins	17-OSU-05	0.00	0.00	42.48	Arctic\O-Connor (16-22)	17D30134	01
17D30165	13.4 %	Dan Miggins	17-OSU-05	0.00	0.00	42.48	Arctic\O-Connor (16-22)	17D30134	01
17D30167	14.6 %	Dan Miggins	17-OSU-05	0.00	0.00	42.48	Arctic\O-Connor (16-22)	17D30134	01
17D30168	16.0 %	Dan Miggins	17-OSU-05	0.00	0.00	42.48	Arctic\O-Connor (16-22)	17D30134	01
17D30170	17.6 %	Dan Miggins	17-OSU-05	0.00	0.00	42.48	Arctic\O-Connor (16-22)	17D30134	01
17D30171	19.3 %	Dan Miggins	17-OSU-05	0.00	0.00	42.48	Arctic\O-Connor (16-22)	17D30134	01
17D30173	21.0 %	Dan Miggins	17-OSU-05	0.00	0.00	42.48	Arctic\O-Connor (16-22)	17D30134	01

**Sample Parameters**		Sample	Material	Location	Standard Name	Standard (in Ma)	%1σ	Standard Reference	Standard 40Ar/39Ar	%1σ	J	%1σ	Air 40Ar/36Ar	%1σ	MDF (lin)	%1σ	Volume Ratio	Sensitivity (mol/volt)	Day	Month	Year	Hour	Min	Resist
17D30138	1.8 %	HLY0102-D35-1	Groundmass	Gakkel Ridge	FCT-NM (5A29-17)	28.201	0.082	Kuiper et al (2008)	10.09639	0.102	0.00155673	0.102	302.765	0.053	0.9939934	0.060	1	4.8E-14	11	SEP	2017	14	23	1
17D30140	1.9 %	HLY0102-D35-1	Groundmass	Gakkel Ridge	FCT-NM (5A29-17)	28.201	0.082	Kuiper et al (2008)	10.09639	0.102	0.00155673	0.102	302.765	0.053	0.9939934	0.060	1	4.8E-14	11	SEP	2017	14	43	1
17D30141	2.0 %	HLY0102-D35-1	Groundmass	Gakkel Ridge	FCT-NM (5A29-17)	28.201	0.082	Kuiper et al (2008)	10.09639	0.102	0.00155673	0.102	302.765	0.053	0.9939934	0.060	1	4.8E-14	11	SEP	2017	14	53	1
17D30143	2.2 %	HLY0102-D35-1	Groundmass	Gakkel Ridge	FCT-NM (5A29-17)	28.201	0.082	Kuiper et al (2008)	10.09639	0.102	0.00155673	0.102	302.765	0.053	0.9939934	0.060	1	4.8E-14	11	SEP	2017	15	13	1
17D30144	2.4 %	HLY0102-D35-1	Groundmass	Gakkel Ridge	FCT-NM (5A29-17)	28.201	0.082	Kuiper et al (2008)	10.09639	0.102	0.00155673	0.102	302.765	0.053	0.9939934	0.060	1	4.8E-14	11	SEP	2017	15	23	1
17D30146	2.7 %	HLY0102-D35-1	Groundmass	Gakkel Ridge	FCT-NM (5A29-17)	28.201	0.082	Kuiper et al (2008)	10.09639	0.102	0.00155673	0.102	302.765	0.053	0.9939934	0.060	1	4.8E-14	11	SEP	2017	15	43	1
17D30147	3.0 %	HLY0102-D35-1	Groundmass	Gakkel Ridge	FCT-NM (5A29-17)	28.201	0.082	Kuiper et al (2008)	10.09639	0.102	0.00155673	0.102	302.765	0.053	0.9939934	0.060	1	4.8E-14	11	SEP	2017	15	53	1
17D30149	3.4 %	HLY0102-D35-1	Groundmass	Gakkel Ridge	FCT-NM (5A29-17)	28.201	0.082	Kuiper et al (2008)	10.09639	0.102	0.00155673	0.102	302.765	0.053	0.9939934	0.060	1	4.8E-14	11	SEP	2017	16	13	1
17D30150	3.9 %	HLY0102-D35-1	Groundmass	Gakkel Ridge	FCT-NM (5A29-17)	28.201	0.082	Kuiper et al (2008)	10.09639	0.102	0.00155673	0.102	302.765	0.053	0.9939934	0.060	1	4.8E-14	11	SEP	2017	16	23	1
17D30152	4.5 %	HLY0102-D35-1	Groundmass	Gakkel Ridge	FCT-NM (5A29-17)	28.201	0.082	Kuiper et al (2008)	10.09639	0.102	0.00155673	0.102	302.765	0.053	0.9939934	0.060	1	4.8E-14	11	SEP	2017	16	43	1
17D30153	5.2 %	HLY0102-D35-1	Groundmass	Gakkel Ridge	FCT-NM (5A29-17)	28.201	0.082	Kuiper et al (2008)	10.09639	0.102	0.00155673	0.102	302.765	0.053	0.9939934	0.060	1	4.8E-14	11	SEP	2017	16	53	1
17D30155	6.0 %	HLY0102-D35-1	Groundmass	Gakkel Ridge	FCT-NM (5A29-17)	28.201	0.082	Kuiper et al (2008)	10.09639	0.102	0.00155673	0.102	302.765	0.053	0.9939934	0.060	1	4.8E-14	11	SEP	2017	17	14	1
17D30156	6.9 %	HLY0102-D35-1	Groundmass	Gakkel Ridge	FCT-NM (5A29-17)	28.201	0.082	Kuiper et al (2008)	10.09639	0.102	0.00155673	0.102	302.765	0.053	0.9939934	0.060	1	4.8E-14	11	SEP	2017	17	24	1
17D30158	7.9 %	HLY0102-D35-1	Groundmass	Gakkel Ridge	FCT-NM (5A29-17)	28.201	0.082	Kuiper et al (2008)	10.09639	0.102	0.00155673	0.102	302.765	0.053	0.9939934	0.060	1	4.8E-14	11	SEP	2017	17	44	1
17D30159	9.0 %	HLY0102-D35-1	Groundmass	Gakkel Ridge	FCT-NM (5A29-17)	28.201	0.082	Kuiper et al (2008)	10.09639	0.102	0.00155673	0.102	302.765	0.053	0.9939934	0.060	1	4.8E-14	11	SEP	2017	17	54	1
17D30161	10.3 %	HLY0102-D35-1	Groundmass	Gakkel Ridge	FCT-NM (5A29-17)	28.201	0.082	Kuiper et al (2008)	10.09639	0.102	0.00155673	0.102	302.765	0.053	0.9939934	0.060	1	4.8E-14	11	SEP	2017	18	14	1
17D30162	11.6 %	HLY0102-D35-1	Groundmass	Gakkel Ridge	FCT-NM (5A29-17)	28.201	0.082	Kuiper et al (2008)	10.09639	0.102	0.00155673	0.102	302.765	0.053	0.9939934	0.060	1	4.8E-14	11	SEP	2017	18	24	1
17D30164	12.5 %	HLY0102-D35-1	Groundmass	Gakkel Ridge	FCT-NM (5A29-17)	28.201	0.082	Kuiper et al (2008)	10.09639	0.102	0.00155673	0.102	302.765	0.053	0.9939934	0.060	1	4.8E-14	11	SEP	2017	18	44	1
17D30165	13.4 %	HLY0102-D35-1	Groundmass	Gakkel Ridge	FCT-NM (5A29-17)	28.201	0.082	Kuiper et al (2008)	10.09639	0.102	0.00155673	0.102	302.765	0.053	0.9939934	0.060	1	4.8E-14	11	SEP	2017	18	54	1
17D30167	14.6 %	HLY0102-D35-1	Groundmass	Gakkel Ridge	FCT-NM (5A29-17)	28.201	0.082	Kuiper et al (2008)	10.09639	0.102	0.00155673	0.102	302.765	0.053	0.9939934	0.060	1	4.8E-14	11	SEP	2017	19	14	1
17D30168	16.0 %	HLY0102-D35-1	Groundmass	Gakkel Ridge	FCT-NM (5A29-17)	28.201	0.082	Kuiper et al (2008)	10.09639	0.102	0.00155673	0.102	302.765	0.053	0.9939934	0.060	1	4.8E-14	11	SEP	2017	19	24	1
17D30170	17.6 %	HLY0102-D35-1	Groundmass	Gakkel Ridge	FCT-NM (5A29-17)	28.201	0.082	Kuiper et al (2008)	10.09639	0.102	0.00155673	0.102	302.765	0.053	0.9939934	0.060	1	4.8E-14	11	SEP	2017	19	44	1
17D30171	19.3 %	HLY0102-D35-1	Groundmass	Gakkel Ridge	FCT-NM (5A29-17)	28.201	0.082	Kuiper et al (2008)	10.09639	0.102	0.00155673	0.102	302.765	0.053	0.9939934	0.060	1	4.8E-14	11	SEP	2017	19	54	1
17D30173	21.0 %	HLY0102-D35-1	Groundmass	Gakkel Ridge	FCT-NM (5A29-17)	28.201	0.082	Kuiper et al (2008)	10.09639	0.102	0.00155673	0.102	302.765	0.053	0.9939934	0.060	1	4.8E-14	11	SEP	2017	20	14	1

Irradiation Constants		40/36(a)	%1σ	40/36(c)	%1σ	38/36(a)	%1σ	38/36(c)	%1σ	39/37(ca)	%1σ	38/37(ca)	%1σ	36/37(ca)	%1σ	40/39(k)	%1σ	38/39(k)	%1σ	36/38(cl)	%1σ	K/Ca	%1σ	K/Cl	%1σ	Ca/Cl	%1σ
17D30138	1.8 %	300.25	0.802	0.018	35	0.1869	0	1.493	3	0.000643	0.92	0.00018	9.63	0.00027	0.17	0.000607	9.65	0.012077	0.09	0	0	0.43	0	0	0	0	0
17D30140	1.9 %	300.25	0.802	0.018	35	0.1869	0	1.493	3	0.000643	0.92	0.00018	9.63	0.00027	0.17	0.000607	9.65	0.012077	0.09	0	0	0.43	0	0	0	0	0
17D30141	2.0 %	300.25	0.802	0.018	35	0.1869	0	1.493	3	0.000643	0.92	0.00018	9.63	0.00027	0.17	0.000607	9.65	0.012077	0.09	0	0	0.43	0	0	0	0	0
17D30143	2.2 %	300.25	0.802	0.018	35	0.1869	0	1.493	3	0.000643	0.92	0.00018	9.63	0.00027	0.17	0.000607	9.65	0.012077	0.09	0	0	0.43	0	0	0	0	0
17D30144	2.4 %	300.25	0.802	0.018	35	0.1869	0	1.493	3	0.000643	0.92	0.00018	9.63	0.00027	0.17	0.000607	9.65	0.012077	0.09	0	0	0.43	0	0	0	0	0
17D30146	2.7 %	300.25	0.802	0.018	35	0.1869	0	1.493	3	0.000643	0.92	0.00018	9.63	0.00027	0.17	0.000607	9.65	0.012077	0.09	0	0	0.43	0	0	0	0	0
17D30147	3.0 %	300.25	0.802	0.018	35	0.1869	0	1.493	3	0.000643	0.92	0.00018	9.63	0.00027	0.17	0.000607	9.65	0.012077	0.09	0	0	0.43	0	0	0	0	0
17D30149	3.4 %	300.25	0.802	0.018	35	0.1869	0	1.493	3	0.000643	0.92	0.00018	9.63	0.00027	0.17	0.000607	9.65	0.012077	0.09	0	0	0.43	0	0	0	0	0
17D30150	3.9 %	300.25	0.802	0.018	35	0.1869	0	1.493	3	0.000643	0.92	0.00018	9.63	0.00027	0.17	0.000607	9.65	0.012077	0.09	0	0	0.43	0	0	0	0	0
17D30152	4.5 %	300.25	0.802	0.018	35	0.1869	0	1.493	3	0.000643	0.92	0.00018	9.63	0.00027	0.17	0.000607	9.65	0.012077	0.09	0	0	0.43	0	0	0	0	0
17D30153	5.2 %	300.25	0.802	0.018	35	0.1869	0	1.493	3	0.000643	0.92	0.00018	9.63	0.00027	0.17	0.000607	9.65	0.012077	0.09	0	0	0.43	0	0	0	0	0
17D30155	6.0 %	300.25	0.802	0.018	35	0.1869	0	1.493	3	0.000643	0.92	0.00018	9.63	0.00027	0.17	0.000607	9.65	0.012077	0.09	0	0	0.43	0	0	0	0	0
17D30156	6.9 %	300.25	0.802	0.018	35	0.1869	0	1.493	3	0.000643	0.92	0.00018	9.63	0.00027	0.17	0.000607	9.65	0.012077	0.09	0	0	0.43	0	0	0	0	0
17D30158	7.9 %	300.25	0.802	0.018	35	0.1869	0	1.493	3	0.000643	0.92	0.00018	9.63	0.00027	0.17	0.000607	9.65	0.012077	0.09	0	0	0.43	0	0	0	0	0
17D30159	9.0 %	300.25	0.802	0.018	35	0.1869	0	1.493	3	0.000643	0.92	0.00018	9.63	0.00027	0.17	0.000607	9.65	0.012077	0.09	0	0	0.43	0	0	0	0	0
17D30161	10.3 %	300.25	0.802	0.018	35	0.1869	0	1.493	3	0.000643	0.92	0.00018	9.63	0.00027	0.17	0.000607	9.65	0.012077	0.09	0	0	0.43	0	0	0	0	0
17D30162	11.6 %	300.25	0.802	0.018	35	0.1869	0	1.493	3	0.000643	0.92	0.00018	9.63	0.00027	0.17	0.000607	9.65	0.012077	0.09	0	0	0.43	0	0	0	0	0
17D30164	12.5 %	300.25	0.802	0.018	35	0.1869	0	1.493	3	0.000643	0.92	0.00018	9.63	0.00027	0.17	0.000607	9.65	0.012077	0.09	0	0	0.43	0	0	0	0	0
17D30165	13.4 %	300.25	0.802	0.018	35	0.1869	0	1.493	3	0.000643	0.92	0.00018	9.63	0.00027	0.17	0.000607	9.65	0.012077	0.09	0	0	0.43	0	0	0	0	0
17D30167	14.6 %	300.25	0.802	0.018	35	0.1869	0	1.493	3	0.000643	0.92	0.00018	9.63	0.00027	0.17	0.000607	9.65	0.012077	0.09	0	0	0.43	0	0	0	0	0
17D30168	16.0 %	300.25	0.802	0.018	35	0.1869	0	1.493	3	0.000643	0.92	0.00018	9.63	0.00027	0.17	0.000607	9.65	0.012077	0.09	0	0	0.43	0	0	0	0	0
17D30170	17.6 %	300.25	0.802	0.018	35	0.1869	0	1.493	3	0.000643	0.92	0.00018	9.63	0.00027	0.17	0.000607	9.65	0.012077	0.09	0	0	0.43	0	0	0	0	0
17D30171	19.3 %	300.25	0.802	0.018	35	0.1869	0	1.493	3	0.000643	0.92	0.00018	9.63	0.00027	0.17	0.000607	9.65	0.012077	0.09	0	0	0.43	0	0	0	0	0
17D30173	21.0 %	300.25	0.802	0.018	35	0.1869	0	1.493	3	0.000643	0.92	0.00018	9.63	0.00027	0.17	0.000607	9.65	0.012077	0.09	0	0	0.43	0	0	0	0	0

17D30134.AGE >>> HLY0102-D35-1 >>> ARCTIC | O-CONNOR (16-22) PROJECT

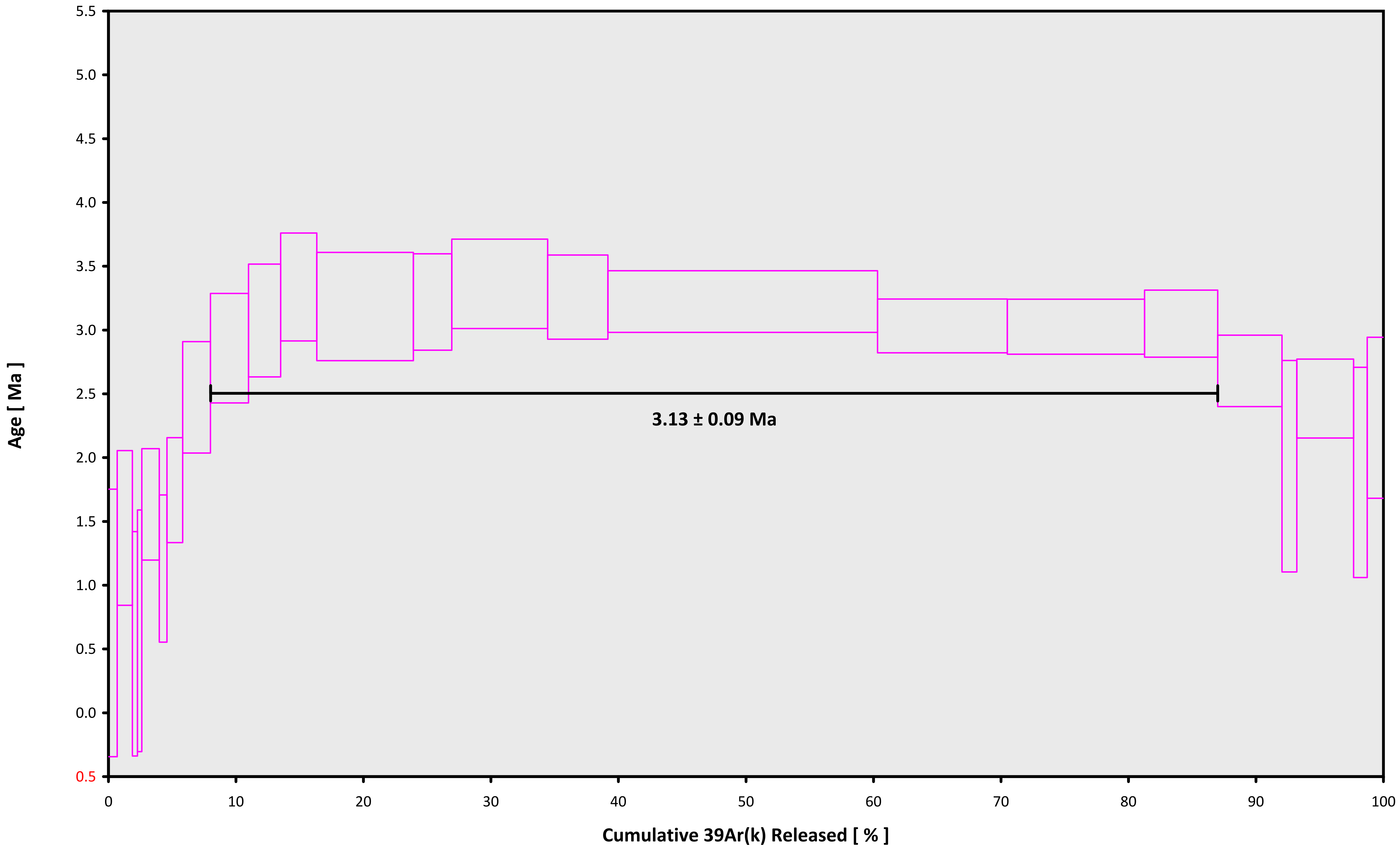

Ar-Ages in Ma

WEIGHTED PLATEAU
3.13 ± 0.09
TOTAL FUSION
2.94 ± 0.09
NORMAL ISOCHRON
2.94 ± 0.30
INVERSE ISOCHRON
2.94 ± 0.29

MSWD (PROBABILITY)
0.80 (63%)

Sample Info

Groundmass
Gakkel Ridge
Dan Miggins

IRR = 17-OSU-05 (5A29-17)
J = 0.00155673 ± 0.00000159

17D30134.AGE >>> HLY0102-D35-1 >>> ARCTIC | O-CONNOR (16-22) PROJECT

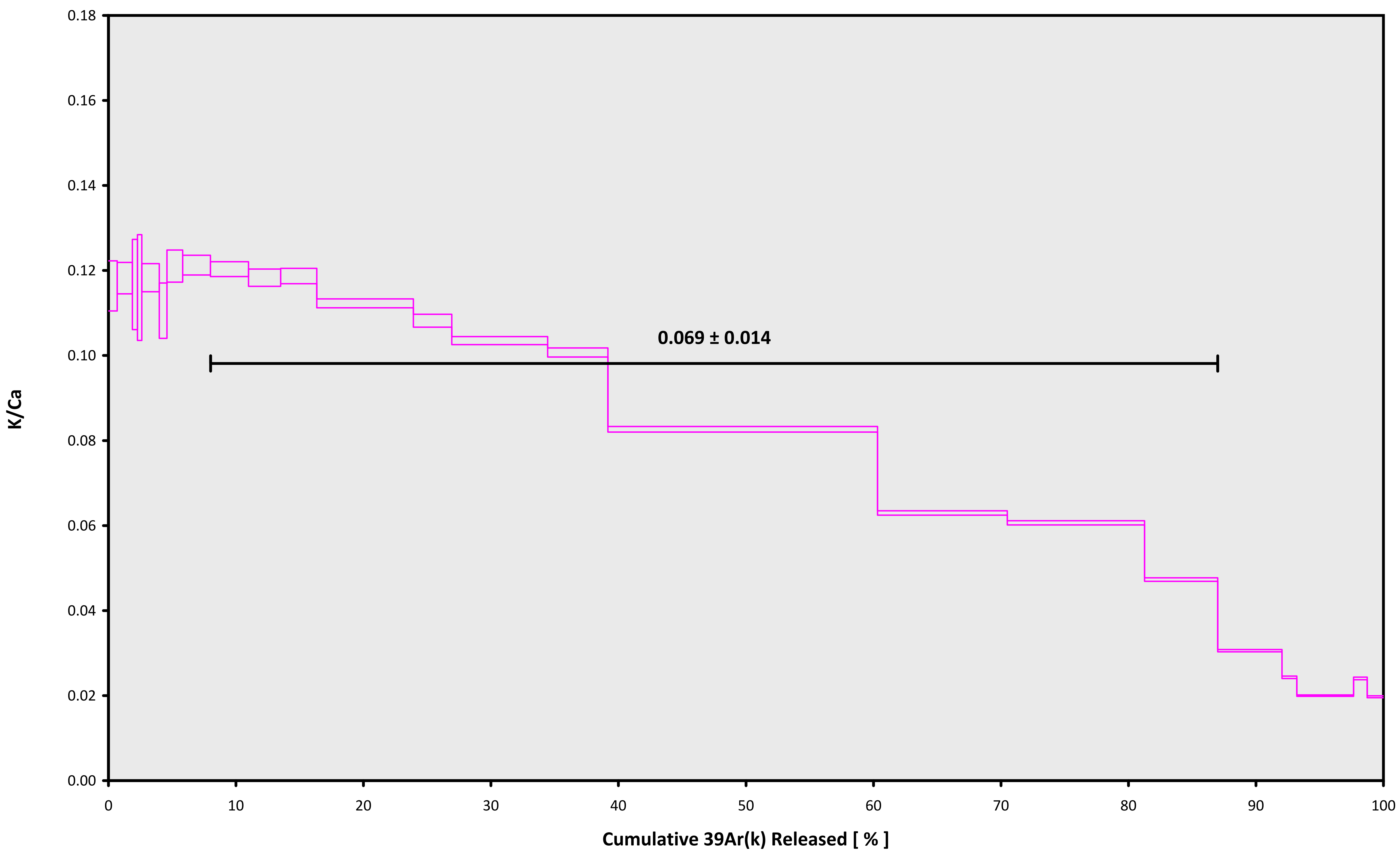

Ar-Ages in Ma

WEIGHTED PLATEAU
3.13 ± 0.09
TOTAL FUSION
2.94 ± 0.09
NORMAL ISOCHRON
2.94 ± 0.30
INVERSE ISOCHRON
2.94 ± 0.29

Sample Info

Groundmass
Gakkel Ridge
Dan Miggins

IRR = 17-OSU-05 (5A29-17)
J = 0.00155673 ± 0.00000159

17D30134.AGE >>> HLY0102-D35-1 >>> ARCTIC | O-CONNOR (16-22) PROJECT

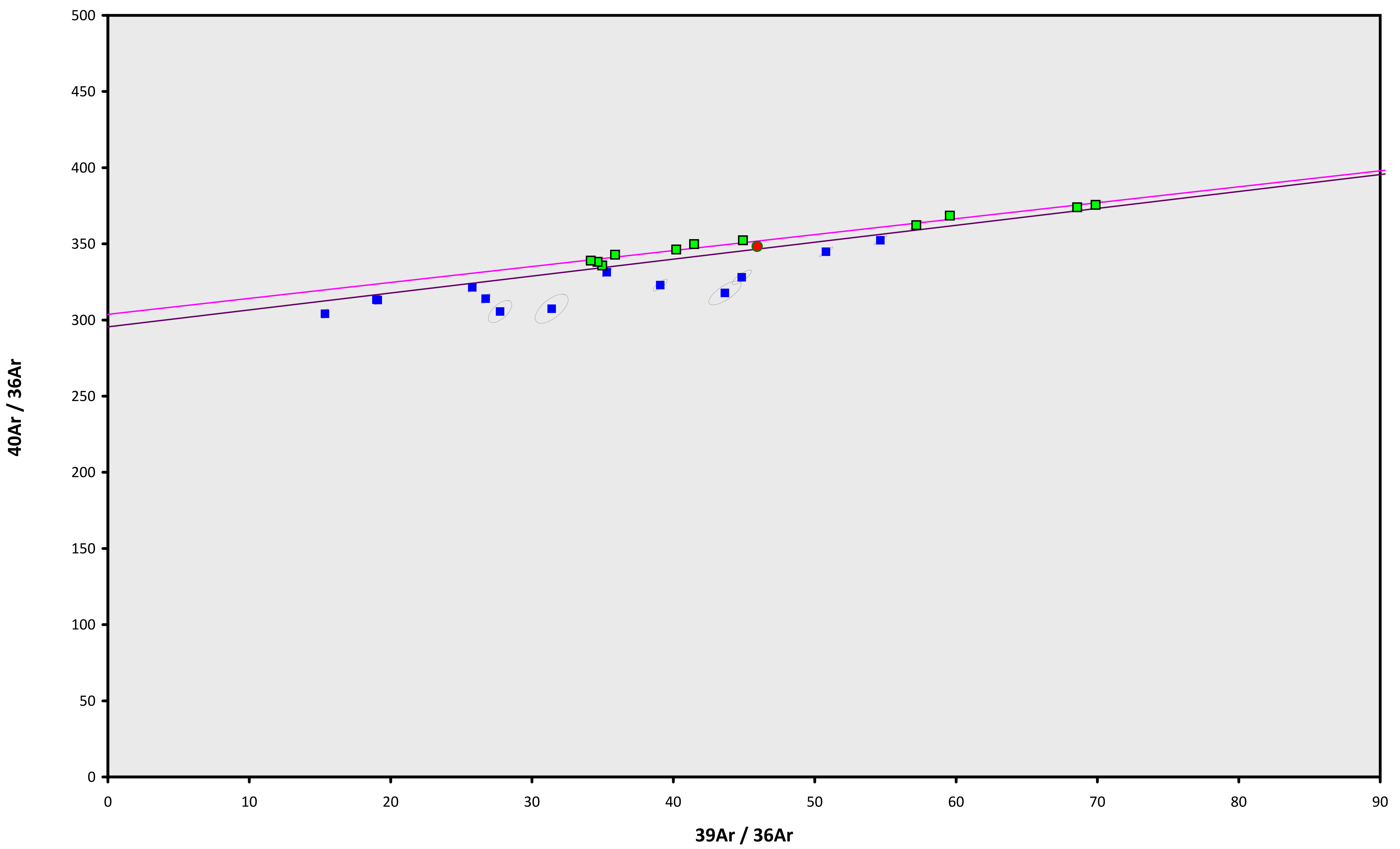

Ar-Ages in Ma

WEIGHTED PLATEAU
3.13 ± 0.09
TOTAL FUSION
2.94 ± 0.09
NORMAL ISOCHRON
2.94 ± 0.30
INVERSE ISOCHRON
2.94 ± 0.29

MSWD (PROBABILITY)
4.64 (0%)

40AR/36AR INTERCEPT
303.7 ± 5.3

Sample Info

Groundmass
Gakkel Ridge
Dan Miggins

IRR = 17-OSU-05 (5A29-17)
J = 0.00155673 ± 0.00000159

17D30134.AGE >>> HLY0102-D35-1 >>> ARCTIC | O-CONNOR (16-22) PROJECT

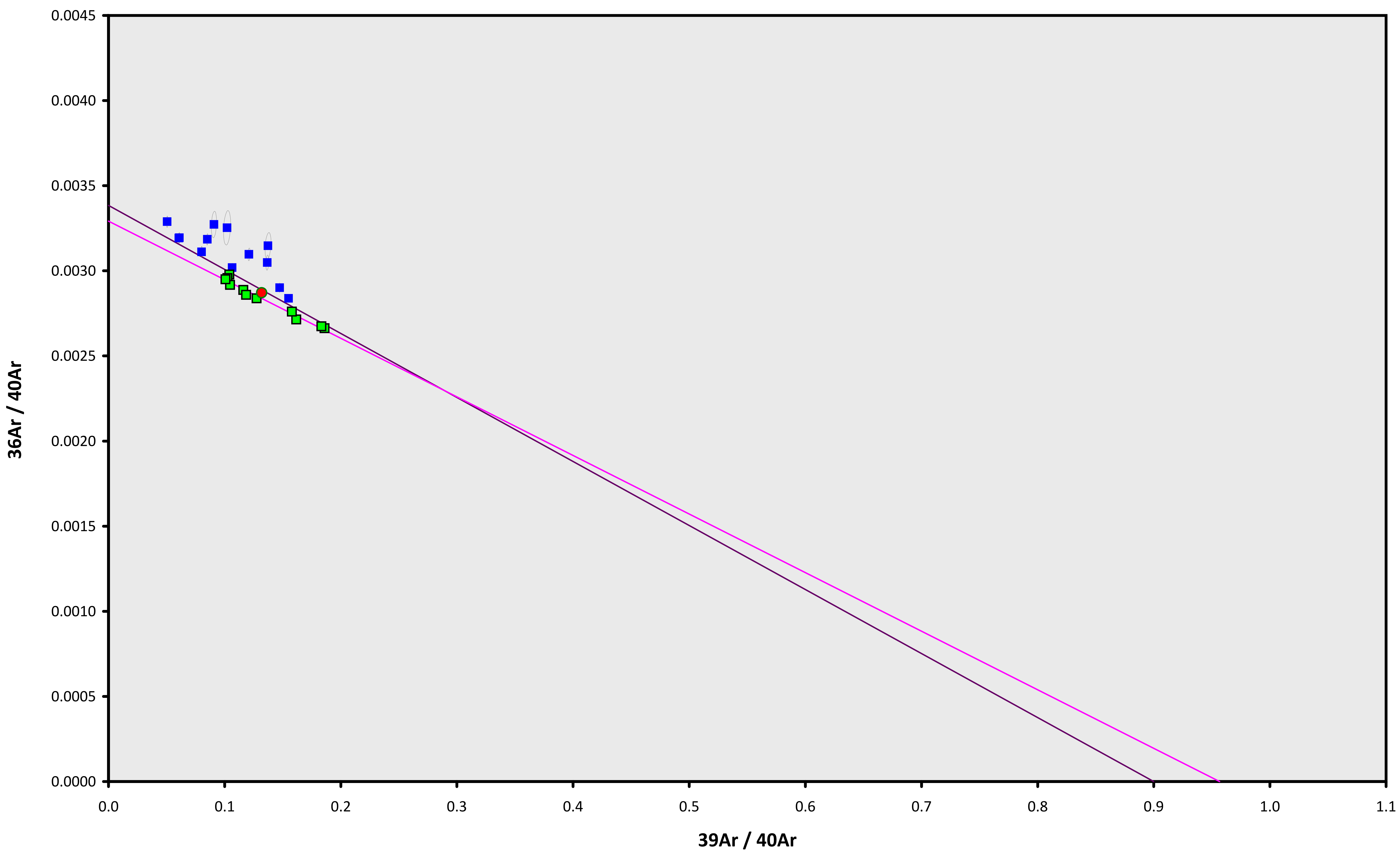

Ar-Ages in Ma

WEIGHTED PLATEAU
3.13 ± 0.09
TOTAL FUSION
2.94 ± 0.09
NORMAL ISOCHRON
2.94 ± 0.30
INVERSE ISOCHRON
2.94 ± 0.29

MSWD (PROBABILITY)
4.61 (0%)

SPREADING FACTOR
8.9%

40AR/36AR INTERCEPT
303.8 ± 5.3

Sample Info

Groundmass
Gakkel Ridge
Dan Miggins

IRR = 17-OSU-05 (5A29-17)
J = 0.00155673 ± 0.00000159

Relative Abundances			36Ar [fA]	%1σ	37Ar [fA]	%1σ	38Ar [fA]	%1σ	39Ar [fA]	%1σ	40Ar [fA]	%1σ	40(r)/39(k) ± 2σ	Age ± 2σ (Ma)	40Ar(r) (%)	39Ar(k) (%)	K/Ca ± 2σ
17D19952	1.8 %		1.6518569	0.303	98.5978	0.623	0.5398125	4.455	18.37320	0.143	489.8273	0.013	0.52255 ± 0.16198	1.49 ± 0.46	1.95	5.01	0.0799 ± 0.0010
17D19954	1.9 %		0.5092907	0.352	74.5231	0.783	0.2446837	10.032	13.61542	0.180	152.1408	0.045	0.55939 ± 0.07902	1.59 ± 0.22	4.99	3.71	0.0783 ± 0.0013
17D19955	2.0 %	✓	0.3248033	0.404	50.9846	0.965	0.1421715	16.941	9.39009	0.253	96.3141	0.067	0.47036 ± 0.08441	1.34 ± 0.24	4.57	2.56	0.0789 ± 0.0016
17D19957	2.2 %	✓	0.2212819	0.456	58.5104	0.920	0.1606975	14.983	10.24449	0.227	65.8482	0.080	0.50228 ± 0.06002	1.43 ± 0.17	7.79	2.79	0.0750 ± 0.0014
17D19958	2.4 %	✓	0.2136182	0.444	65.5860	0.800	0.1398540	16.634	11.04266	0.216	62.9731	0.081	0.46187 ± 0.05240	1.31 ± 0.15	8.07	3.01	0.0721 ± 0.0012
17D19960	2.7 %	✓	0.3596386	0.377	123.2205	0.577	0.2652373	9.069	19.59274	0.134	105.8645	0.060	0.48282 ± 0.04205	1.37 ± 0.12	8.90	5.33	0.0681 ± 0.0008
17D19961	3.0 %	✓	0.2076269	0.471	91.2004	0.669	0.1882404	12.070	14.21244	0.175	60.9647	0.084	0.48658 ± 0.04207	1.38 ± 0.12	11.30	3.87	0.0667 ± 0.0009
17D19963	3.4 %	✓	0.3196963	0.386	139.2780	0.563	0.2576352	9.274	20.74079	0.128	92.6129	0.063	0.44814 ± 0.03637	1.27 ± 0.10	9.99	5.65	0.0638 ± 0.0007
17D19964	3.9 %	✓	0.3159696	0.407	162.5242	0.530	0.3237312	7.639	23.03779	0.119	90.6582	0.064	0.44724 ± 0.03414	1.27 ± 0.10	11.31	6.27	0.0607 ± 0.0007
17D19966	4.5 %	✓	0.1793754	0.509	103.7117	0.616	0.1629756	14.955	14.66526	0.165	51.0058	0.098	0.42986 ± 0.03827	1.22 ± 0.11	12.30	3.99	0.0605 ± 0.0008
17D19967	5.2 %	✓	0.3499085	0.396	203.1597	0.510	0.4084806	5.891	27.31579	0.111	99.8069	0.060	0.46420 ± 0.03110	1.32 ± 0.09	12.64	7.43	0.0575 ± 0.0006
17D19969	6.0 %	✓	0.3438523	0.410	230.0841	0.496	0.3829418	6.425	29.61907	0.105	97.2594	0.058	0.47540 ± 0.02931	1.35 ± 0.08	14.41	8.06	0.0551 ± 0.0006
17D19970	6.9 %	✓	0.2391974	0.446	186.5063	0.516	0.3117146	7.516	22.74018	0.122	66.1710	0.081	0.45850 ± 0.02920	1.30 ± 0.08	15.67	6.18	0.0522 ± 0.0006
17D19972	7.9 %	✓	0.2251249	0.480	174.7125	0.520	0.2661205	9.417	20.39040	0.144	61.6462	0.082	0.44700 ± 0.03280	1.27 ± 0.09	14.70	5.54	0.0499 ± 0.0005
17D19973	9.0 %	✓	0.2873821	0.416	265.2539	0.486	0.3552358	6.422	26.24860	0.111	75.4580	0.071	0.44894 ± 0.02864	1.28 ± 0.08	15.52	7.13	0.0423 ± 0.0004
17D19975	10.3 %		0.2115231	0.471	221.2796	0.500	0.2983211	7.781	19.61572	0.138	52.7788	0.095	0.40754 ± 0.03214	1.16 ± 0.09	15.04	5.32	0.0378 ± 0.0004
17D19976	11.6 %		0.1988170	0.477	225.5800	0.499	0.2546517	9.345	17.86333	0.143	48.2857	0.102	0.42568 ± 0.03390	1.21 ± 0.10	15.62	4.84	0.0338 ± 0.0004
17D19978	12.5 %		0.1462930	0.546	184.2174	0.517	0.1752701	13.104	11.71883	0.203	32.7160	0.150	0.36149 ± 0.04386	1.03 ± 0.12	12.82	3.17	0.0271 ± 0.0003
17D19979	13.4 %		0.1378600	0.574	180.8252	0.516	0.1545710	15.976	9.89758	0.243	29.9943	0.162	0.37764 ± 0.05145	1.07 ± 0.15	12.32	2.67	0.0233 ± 0.0003
17D19981	14.6 %		0.0876827	0.697	121.7957	0.586	0.1126681	21.832	5.76149	0.426	18.2880	0.267	0.37011 ± 0.06912	1.05 ± 0.20	11.50	1.55	0.0201 ± 0.0003
17D19982	16.0 %		0.0913022	0.687	129.9330	0.569	0.0915924	27.760	5.60118	0.411	19.1786	0.252	0.46645 ± 0.07306	1.33 ± 0.21	13.42	1.51	0.0183 ± 0.0003
17D19984	17.6 %		0.1136951	0.605	173.8981	0.523	0.1055974	24.214	6.31087	0.354	21.8500	0.222	0.34509 ± 0.07182	0.98 ± 0.20	9.79	1.69	0.0153 ± 0.0002
17D19985	19.3 %		0.1223336	0.586	178.7634	0.521	0.1060425	23.115	6.17523	0.378	24.5467	0.199	0.44088 ± 0.07628	1.25 ± 0.22	10.88	1.66	0.0146 ± 0.0002
17D19987	21.0 %		0.0758260	0.780	124.4558	0.575	0.0578369	41.654	3.88628	0.616	14.0952	0.343	0.42747 ± 0.10042	1.22 ± 0.29	11.54	1.04	0.0132 ± 0.0002
		Σ	6.9339557	0.104	3568.6014	0.117	5.5060833	2.146	368.05944	0.034	1930.2844	0.014					

Information on Analysis and Constants Used in Calculations

Project = **O-CONNOR (16-22)**
Sample = **HLY0102-D35-12**
Material = **Groundmass**
Location = **Gakkel Ridge**
Region = **Artic Ocean**
Analyst = **Dan Miggins**
Irradiation = **17-OSU-01 (1C32-17)**
Position = **X: 0 | Y: 0 | Z/H: 49.18268 mm**
FCT-NM Age = **28.201 ± 0.023 Ma**
FCT-NM Reference = **Kuiper et al (2008)**
FCT-NM 40Ar/39Ar Ratio = **9.99322 ± 0.00849**
FCT-NM J-value = **0.00157281 ± 0.00000134**
Air Shot 40Ar/36Ar = **302.4220 ± 0.4355**
Air Shot MDF = **0.99427047 ± 0.00067830 (LIN)**
Experiment Type = **Incremental Heating**
Extraction Method = **Bulk Laser Heating**
Heating = **64 sec**
Isolation = **3.00 min**
Instrument = **ARGUS-VI-D**
Preferred Age = **Plateau Age**
Age Classification = **Crystallization Age**
IGSN = Undefined
Rock Class = **Igneous>Volcanic**
Lithology = **Basaltic Lava**
Lat-Lon = Undefined - Undefined

Age Equations = **Min et al. (2000)**
Negative Intensities = **Allowed**
Collector Calibrations = **36Ar**
Decay 40K = **5.530 ± 0.048 E-10 1/a**
Decay 39Ar = **2.940 ± 0.016 E-07 1/h**
Decay 37Ar = **8.230 ± 0.012 E-04 1/h**
Decay 36Cl = **2.257 ± 0.015 E-06 1/a**
Decay 40K(EC,β⁺) = **0.580 ± 0.009 E-10 1/a**
Decay 40K(β⁻) = **4.950 ± 0.043 E-10 1/a**
Atmospheric 40/36(a) = **295.50**
Atmospheric 38/36(a) = **0.1869**
Production 39/37(ca) = **0.0006425 ± 0.0000059**
Production 38/37(ca) = **0.0001800 ± 0.0000173**
Production 36/37(ca) = **0.0002703 ± 0.0000005**
Production 40/39(k) = **0.000607 ± 0.000059**
Production 38/39(k) = **0.012077 ± 0.000011**
Production 36/38(cl) = **262.80 ± 1.71**
Scaling Ratio K/Ca = **0.430**
Abundance Ratio 40K/K = **1.1700 ± 0.0100 E-04**
Atomic Weight K = **39.0983 ± 0.0001 g**

Results	40(a)/36(a) ± 2σ	40(r)/39(k) ± 2σ	Age ± 2σ (Ma)	MSWD	39Ar(k) (%,n)	K/Ca ± 2σ
**Age Plateau**		0.45962 ± 0.01014<br>± 2.21%	**1.31 ± 0.03**<br>**± 2.21%**	0.89<br>56%	67.82<br>13	0.0554 ± 0.0053
		Full External Error	± 0.04	1.82	2σ Confidence Limit	
		Analytical Error	± 0.03	1.0000	Error Magnification	
**Total Fusion Age**		0.45416 ± 0.01186<br>± 2.61%	**1.29 ± 0.03**<br>**± 2.62%**		24	0.0441 ± 0.0001
		Full External Error	± 0.04			
		Analytical Error	± 0.03			
**Normal Isochron**	**297.24** ± 2.52<br>± 0.85%	0.43947 ± 0.03065<br>± 6.97%	**1.25 ± 0.09**<br>**± 6.97%**	0.80<br>64%	67.82<br>13	
		Full External Error	± 0.09	1.85	2σ Confidence Limit	
		Analytical Error	± 0.09	1.0000	Error Magnification	
				4	Number of Iterations	
				0.0000033882	Convergence	
**Inverse Isochron**	**297.25** ± 2.52<br>± 0.85%	0.43959 ± 0.03047<br>± 6.93%	**1.25 ± 0.09**<br>**± 6.93%**	0.80<br>64%	67.82<br>13	
		Full External Error	± 0.09	1.85	2σ Confidence Limit	
		Analytical Error	± 0.09	1.0000	Error Magnification	
				3	Number of Iterations	
				0.0000146858	Convergence	
				11%	Spreading Factor	

Notes
Mostly atmospheric

Incremental Heating			36Ar(a) [fA]	37Ar(ca) [fA]	38Ar(cl) [fA]	39Ar(k) [fA]	40Ar(r) [fA]	Age ± 2σ (Ma)	40Ar(r) (%)	39Ar(k) (%)	K/Ca ± 2σ
17D19952	1.8 %		1.6252059	98.5978	0.0000000	18.30985	9.56780	1.49 ± 0.46	1.95	5.01	0.0799 ± 0.0010
17D19954	1.9 %		0.4891471	74.5231	0.0000000	13.56754	7.58959	1.59 ± 0.22	4.99	3.71	0.0783 ± 0.0013
17D19955	2.0 %	✓	0.3110222	50.9846	0.0000000	9.35734	4.40131	1.34 ± 0.24	4.57	2.56	0.0789 ± 0.0016
17D19957	2.2 %	✓	0.2054665	58.5104	0.0000000	10.20690	5.12668	1.43 ± 0.17	7.79	2.79	0.0750 ± 0.0014
17D19958	2.4 %	✓	0.1958904	65.5860	0.0000000	11.00052	5.08083	1.31 ± 0.15	8.07	3.01	0.0721 ± 0.0012
17D19960	2.7 %	✓	0.3263321	123.2205	0.0000000	19.51357	9.42149	1.37 ± 0.12	8.90	5.33	0.0681 ± 0.0008
17D19961	3.0 %	✓	0.1829754	91.2004	0.0000000	14.15384	6.88691	1.38 ± 0.12	11.30	3.87	0.0667 ± 0.0009
17D19963	3.4 %	✓	0.2820495	139.2780	0.0000000	20.65131	9.25471	1.27 ± 0.10	9.99	5.65	0.0638 ± 0.0007
17D19964	3.9 %	✓	0.2720393	162.5242	0.0000000	22.93337	10.25672	1.27 ± 0.10	11.31	6.27	0.0607 ± 0.0007
17D19966	4.5 %	✓	0.1513421	103.7117	0.0000000	14.59863	6.27537	1.22 ± 0.11	12.30	3.99	0.0605 ± 0.0008
17D19967	5.2 %	✓	0.2949945	203.1597	0.0000000	27.18526	12.61951	1.32 ± 0.09	12.64	7.43	0.0575 ± 0.0006
17D19969	6.0 %	✓	0.2816606	230.0841	0.0000000	29.47124	14.01077	1.35 ± 0.08	14.41	8.06	0.0551 ± 0.0006
17D19970	6.9 %	✓	0.1887847	186.5063	0.0000000	22.62035	10.37137	1.30 ± 0.08	15.67	6.18	0.0522 ± 0.0006
17D19972	7.9 %	✓	0.1779001	174.7125	0.0000000	20.27815	9.06439	1.27 ± 0.09	14.70	5.54	0.0499 ± 0.0005
17D19973	9.0 %	✓	0.2156840	265.2539	0.0000000	26.07818	11.70761	1.28 ± 0.08	15.52	7.13	0.0423 ± 0.0004
17D19975	10.3 %		0.1517112	221.2796	0.0000000	19.47354	7.93632	1.16 ± 0.09	15.04	5.32	0.0378 ± 0.0004
17D19976	11.6 %		0.1378427	225.5800	0.0000000	17.71839	7.54243	1.21 ± 0.10	15.62	4.84	0.0338 ± 0.0004
17D19978	12.5 %		0.0964990	184.2174	0.0000000	11.60047	4.19350	1.03 ± 0.12	12.82	3.17	0.0271 ± 0.0003
17D19979	13.4 %		0.0889830	180.8252	0.0000000	9.78140	3.69387	1.07 ± 0.15	12.32	2.67	0.0233 ± 0.0003
17D19981	14.6 %		0.0547585	121.7957	0.0118740	5.68324	2.10343	1.05 ± 0.20	11.50	1.55	0.0201 ± 0.0003
17D19982	16.0 %		0.0561813	129.9330	0.0000000	5.51769	2.57372	1.33 ± 0.21	13.42	1.51	0.0183 ± 0.0003
17D19984	17.6 %		0.0666905	173.8981	0.0000000	6.19914	2.13925	0.98 ± 0.20	9.79	1.69	0.0153 ± 0.0002
17D19985	19.3 %		0.0740139	178.7634	0.0000000	6.06037	2.67188	1.25 ± 0.22	10.88	1.66	0.0146 ± 0.0002
17D19987	21.0 %		0.0421856	124.4558	0.0000000	3.80632	1.62709	1.22 ± 0.29	11.54	1.04	0.0132 ± 0.0002
		Σ	5.9693600	3568.6014	0.0118740	365.76661	166.11652				

Information on Analysis
Project = **O-CONNOR (16-22)**
Sample = **HLY0102-D35-12**
Material = **Groundmass**
Location = **Gakkel Ridge**
Region = **Artic Ocean**
Analyst = **Dan Miggins**
Irradiation = **17-OSU-01 (1C32-17)**
J = **0.00157281 ± 0.00000134**
FCT-NM = **28.201 ± 0.023 Ma**

Results	40(r)/39(k) ± 2σ		Age ± 2σ (Ma)		MSWD	39Ar(k) (%,n)	K/Ca ± 2σ
**Age Plateau**	0.45962	± 0.01014<br>± 2.21%	**1.31**	**± 0.03**<br>**± 2.21%**	0.89<br>56%	67.82<br>13	0.0554 ± 0.0053
			Full External Error	± 0.04	1.82	2σ Confidence Limit	
			Analytical Error	± 0.03	1.0000	Error Magnification	
**Total Fusion Age**	0.45416	± 0.01186<br>± 2.61%	**1.29**	**± 0.03**<br>**± 2.62%**		24	0.0441 ± 0.0001
			Full External Error	± 0.04			
			Analytical Error	± 0.03			

**Normal Isochron**			**39(k)/36(a) ± 2σ**	**40(a+r)/36(a) ± 2σ**	**r.i.**
17D19952	1.8 %		11.27 ± 0.08	301.39 ± 1.86	0.9059
17D19954	1.9 %		27.74 ± 0.23	311.02 ± 2.30	0.8911
17D19955	2.0 %	✓	30.09 ± 0.30	309.65 ± 2.66	0.8475
17D19957	2.2 %	✓	49.68 ± 0.54	320.45 ± 3.22	0.8973
17D19958	2.4 %	✓	56.16 ± 0.60	321.44 ± 3.19	0.9019
17D19960	2.7 %	✓	59.80 ± 0.53	324.37 ± 2.75	0.9426
17D19961	3.0 %	✓	77.35 ± 0.88	333.14 ± 3.65	0.9400
17D19963	3.4 %	✓	73.22 ± 0.68	328.31 ± 2.95	0.9513
17D19964	3.9 %	✓	84.30 ± 0.84	333.20 ± 3.24	0.9620
17D19966	4.5 %	✓	96.46 ± 1.23	336.96 ± 4.19	0.9533
17D19967	5.2 %	✓	92.16 ± 0.91	338.28 ± 3.27	0.9665
17D19969	6.0 %	✓	104.63 ± 1.10	345.24 ± 3.57	0.9732
17D19970	6.9 %	✓	119.82 ± 1.43	350.44 ± 4.13	0.9693
17D19972	7.9 %	✓	113.99 ± 1.46	346.45 ± 4.37	0.9658
17D19973	9.0 %	✓	120.91 ± 1.43	349.78 ± 4.08	0.9746
17D19975	10.3 %		128.36 ± 1.80	347.81 ± 4.84	0.9709
17D19976	11.6 %		128.54 ± 1.90	350.22 ± 5.14	0.9713
17D19978	12.5 %		120.21 ± 2.16	338.96 ± 6.02	0.9595
17D19979	13.4 %		109.92 ± 2.13	337.01 ± 6.42	0.9532
17D19981	14.6 %		103.79 ± 2.60	333.91 ± 8.04	0.9151
17D19982	16.0 %		98.21 ± 2.45	341.31 ± 8.22	0.9217
17D19984	17.6 %		92.95 ± 2.16	327.58 ± 7.36	0.9317
17D19985	19.3 %		81.88 ± 1.80	331.60 ± 6.97	0.9196
17D19987	21.0 %		90.23 ± 2.90	334.07 ± 10.16	0.8966

**Results**	**40(a)/36(a) ± 2σ**		**40(r)/39(k) ± 2σ**		**Age ± 2σ (Ma)**		**MSWD**
**Normal Isochron**	**297.24**	**± 2.52**<br>**± 0.85%**	0.43947	± 0.03065<br>± 6.97%	**1.25**	**± 0.09**<br>**± 6.97%**	0.80<br>64%
					Full External Error	± 0.09	
					Analytical Error	± 0.09	

**Statistics**				
2σ Confidence Limit	1.85	Convergence	0.000003388158
Error Magnification	1.0000	Number of Iterations	4
Number of Data Points	13	Calculated Line	Weighted York-2

Inverse Isochron			39(k)/40(a+r) ± 2σ	36(a)/40(a+r) ± 2σ	r.i.
17D19952	1.8 %		0.0373811 ± 0.0001077	0.00331799 ± 0.00002049	0.0035
17D19954	1.9 %		0.0891824 ± 0.0003316	0.00321527 ± 0.00002382	0.0295
17D19955	2.0 %	✓	0.0971602 ± 0.0005097	0.00322944 ± 0.00002771	0.0400
17D19957	2.2 %	✓	0.1550210 ± 0.0007489	0.00312060 ± 0.00003139	0.0531
17D19958	2.4 %	✓	0.1747045 ± 0.0008097	0.00311103 ± 0.00003088	0.0576
17D19960	2.7 %	✓	0.1843466 ± 0.0005440	0.00308289 ± 0.00002617	0.0579
17D19961	3.0 %	✓	0.2321972 ± 0.0009050	0.00300176 ± 0.00003293	0.0662
17D19963	3.4 %	✓	0.2230155 ± 0.0006380	0.00304588 ± 0.00002737	0.0625
17D19964	3.9 %	✓	0.2530039 ± 0.0006865	0.00300117 ± 0.00002915	0.0617
17D19966	4.5 %	✓	0.2862647 ± 0.0011037	0.00296767 ± 0.00003691	0.0794
17D19967	5.2 %	✓	0.2724237 ± 0.0006905	0.00295614 ± 0.00002859	0.0580
17D19969	6.0 %	✓	0.3030727 ± 0.0007329	0.00289651 ± 0.00002997	0.0545
17D19970	6.9 %	✓	0.3419179 ± 0.0010066	0.00285358 ± 0.00003364	0.0751
17D19972	7.9 %	✓	0.3290099 ± 0.0010970	0.00288640 ± 0.00003638	0.0647
17D19973	9.0 %	✓	0.3456709 ± 0.0009165	0.00285893 ± 0.00003339	0.0661
17D19975	10.3 %		0.3690479 ± 0.0012464	0.00287512 ± 0.00003997	0.0768
17D19976	11.6 %		0.3670308 ± 0.0012978	0.00285537 ± 0.00004191	0.0805
17D19978	12.5 %		0.3546570 ± 0.0018046	0.00295023 ± 0.00005237	0.1000
17D19979	13.4 %		0.3261734 ± 0.0019228	0.00296725 ± 0.00005651	0.0932
17D19981	14.6 %		0.3108216 ± 0.0031586	0.00299479 ± 0.00007214	0.1162
17D19982	16.0 %		0.2877502 ± 0.0028039	0.00292988 ± 0.00007053	0.1082
17D19984	17.6 %		0.2837619 ± 0.0024035	0.00305271 ± 0.00006862	0.1035
17D19985	19.3 %		0.2469290 ± 0.0021461	0.00301568 ± 0.00006339	0.0871
17D19987	21.0 %		0.2700869 ± 0.0038715	0.00299339 ± 0.00009101	0.1078

Results	40(a)/36(a) ± 2σ	40(r)/39(k) ± 2σ	Age ± 2σ (Ma)	MSWD
**Inverse Isochron**	**297.25 ± 2.52 ± 0.85%**	0.43959 ± 0.03047 ± 6.93%	**1.25 ± 0.09 ± 6.93%**	0.80 64%
			Full External Error ± 0.09	
			Analytical Error ± 0.09	

Statistics				
	2σ Confidence Limit	1.85	Convergence	0.0000146858
	Error Magnification	1.0000	Number of Iterations	3
	Number of Data Points	13	Calculated Line	Weighted York-2
	Spreading Factor	10.9%		

Degassing Patterns

			36Ar(a) [fA]	%1σ	36Ar(c) [fA]	%1σ	36Ar(ca) [fA]	%1σ	36Ar(cl) [fA]	%1σ	37Ar(ca) [fA]	%1σ
17D19952	1.8 %		1.6252059	0.31	0.0000000	0.00	0.0266510	0.65	0.0000000	0.00	98.5978	0.62
17D19954	1.9 %		0.4891471	0.37	0.0000000	0.00	0.0201436	0.80	0.0000000	0.00	74.5231	0.78
17D19955	2.0 %	✓	0.3110222	0.42	0.0000000	0.00	0.0137811	0.98	0.0000000	0.00	50.9846	0.97
17D19957	2.2 %	✓	0.2054665	0.50	0.0000000	0.00	0.0158154	0.94	0.0000000	0.00	58.5104	0.92
17D19958	2.4 %	✓	0.1958904	0.49	0.0000000	0.00	0.0177279	0.82	0.0000000	0.00	65.5860	0.80
17D19960	2.7 %	✓	0.3263321	0.42	0.0000000	0.00	0.0333065	0.60	0.0000000	0.00	123.2205	0.58
17D19961	3.0 %	✓	0.1829754	0.54	0.0000000	0.00	0.0246515	0.69	0.0000000	0.00	91.2004	0.67
17D19963	3.4 %	✓	0.2820495	0.44	0.0000000	0.00	0.0376468	0.59	0.0000000	0.00	139.2780	0.56
17D19964	3.9 %	✓	0.2720393	0.48	0.0000000	0.00	0.0439303	0.56	0.0000000	0.00	162.5242	0.53
17D19966	4.5 %	✓	0.1513421	0.61	0.0000000	0.00	0.0280333	0.64	0.0000000	0.00	103.7117	0.62
17D19967	5.2 %	✓	0.2949945	0.48	0.0000000	0.00	0.0549141	0.54	0.0000000	0.00	203.1597	0.51
17D19969	6.0 %	✓	0.2816606	0.51	0.0000000	0.00	0.0621917	0.52	0.0000000	0.00	230.0841	0.50
17D19970	6.9 %	✓	0.1887847	0.58	0.0000000	0.00	0.0504126	0.54	0.0000000	0.00	186.5063	0.52
17D19972	7.9 %	✓	0.1779001	0.62	0.0000000	0.00	0.0472248	0.55	0.0000000	0.00	174.7125	0.52
17D19973	9.0 %	✓	0.2156840	0.58	0.0000000	0.00	0.0716981	0.51	0.0000000	0.00	265.2539	0.49
17D19975	10.3 %		0.1517112	0.69	0.0000000	0.00	0.0598119	0.53	0.0000000	0.00	221.2796	0.50
17D19976	11.6 %		0.1378427	0.73	0.0000000	0.00	0.0609743	0.53	0.0000000	0.00	225.5800	0.50
17D19978	12.5 %		0.0964990	0.87	0.0000000	0.00	0.0497940	0.54	0.0000000	0.00	184.2174	0.52
17D19979	13.4 %		0.0889830	0.94	0.0000000	0.00	0.0488771	0.54	0.0000000	0.00	180.8252	0.52
17D19981	14.6 %		0.0547585	1.17	0.0000000	0.00	0.0329214	0.61	0.0000028	207.95	121.7957	0.59
17D19982	16.0 %		0.0561813	1.18	0.0000000	0.00	0.0351209	0.59	0.0000000	0.00	129.9330	0.57
17D19984	17.6 %		0.0666905	1.10	0.0000000	0.00	0.0470047	0.55	0.0000000	0.00	173.8981	0.52
17D19985	19.3 %		0.0740139	1.03	0.0000000	0.00	0.0483198	0.55	0.0000000	0.00	178.7634	0.52
17D19987	21.0 %		0.0421856	1.48	0.0000000	0.00	0.0336404	0.60	0.0000000	0.00	124.4558	0.57
		Σ	5.9693600	0.12	0.0000000	0.00	0.9645930	0.12	0.0000028	207.95	3568.6014	0.12
		Σ							6.9339557	0.11	3568.6014	0.12

	38Ar(a) [fA]	%1σ	38Ar(c) [fA]	%1σ	38Ar(k) [fA]	%1σ	38Ar(ca) [fA]	%1σ	38Ar(cl) [fA]	%1σ	39Ar(k) [fA]	%1σ	39Ar(ca) [fA]	%1σ
17D19952	0.3037510	0.31	0.0000000	0.00	0.2211280	0.17	0.0177476	9.65	0.0000000	0.00	18.30985	0.14	0.0633491	1.11
17D19954	0.0914216	0.37	0.0000000	0.00	0.1638552	0.20	0.0134142	9.66	0.0000000	0.00	13.56754	0.18	0.0478811	1.21
17D19955	0.0581301	0.42	0.0000000	0.00	0.1130086	0.27	0.0091772	9.68	0.0000000	0.00	9.35734	0.25	0.0327576	1.33
17D19957	0.0384017	0.50	0.0000000	0.00	0.1232687	0.24	0.0105319	9.67	0.0000000	0.00	10.20690	0.23	0.0375929	1.30
17D19958	0.0366119	0.49	0.0000000	0.00	0.1328532	0.23	0.0118055	9.66	0.0000000	0.00	11.00052	0.22	0.0421390	1.22
17D19960	0.0609915	0.42	0.0000000	0.00	0.2356654	0.16	0.0221797	9.65	0.0000000	0.00	19.51357	0.13	0.0791692	1.09
17D19961	0.0341981	0.54	0.0000000	0.00	0.1709360	0.20	0.0164161	9.65	0.0000000	0.00	14.15384	0.18	0.0585963	1.14
17D19963	0.0527150	0.44	0.0000000	0.00	0.2494058	0.16	0.0250700	9.65	0.0000000	0.00	20.65131	0.13	0.0894861	1.08
17D19964	0.0508441	0.48	0.0000000	0.00	0.2769663	0.15	0.0292544	9.64	0.0000000	0.00	22.93337	0.12	0.1044218	1.06
17D19966	0.0282858	0.61	0.0000000	0.00	0.1763076	0.19	0.0186681	9.65	0.0000000	0.00	14.59863	0.17	0.0666348	1.11
17D19967	0.0551345	0.48	0.0000000	0.00	0.3283164	0.14	0.0365687	9.64	0.0000000	0.00	27.18526	0.11	0.1305301	1.05
17D19969	0.0526424	0.51	0.0000000	0.00	0.3559241	0.14	0.0414151	9.64	0.0000000	0.00	29.47124	0.11	0.1478290	1.04
17D19970	0.0352839	0.58	0.0000000	0.00	0.2731860	0.15	0.0335711	9.64	0.0000000	0.00	22.62035	0.12	0.1198303	1.06
17D19972	0.0332495	0.62	0.0000000	0.00	0.2448992	0.17	0.0314483	9.64	0.0000000	0.00	20.27815	0.14	0.1122528	1.06
17D19973	0.0403113	0.58	0.0000000	0.00	0.3149462	0.14	0.0477457	9.64	0.0000000	0.00	26.07818	0.11	0.1704256	1.04
17D19975	0.0283548	0.69	0.0000000	0.00	0.2351820	0.17	0.0398303	9.64	0.0000000	0.00	19.47354	0.14	0.1421721	1.05
17D19976	0.0257628	0.73	0.0000000	0.00	0.2139850	0.17	0.0406044	9.64	0.0000000	0.00	17.71839	0.14	0.1449351	1.05
17D19978	0.0180357	0.87	0.0000000	0.00	0.1400988	0.22	0.0331591	9.64	0.0000000	0.00	11.60047	0.21	0.1183597	1.06
17D19979	0.0166309	0.94	0.0000000	0.00	0.1181299	0.26	0.0325485	9.64	0.0000000	0.00	9.78140	0.25	0.1161802	1.05
17D19981	0.0102344	1.17	0.0000000	0.00	0.0686365	0.44	0.0219232	9.65	0.0118740	207.95	5.68324	0.43	0.0782537	1.09
17D19982	0.0105003	1.18	0.0000000	0.00	0.0666372	0.43	0.0233879	9.65	0.0000000	0.00	5.51769	0.42	0.0834819	1.08
17D19984	0.0124644	1.10	0.0000000	0.00	0.0748671	0.37	0.0313017	9.64	0.0000000	0.00	6.19914	0.36	0.1117295	1.06
17D19985	0.0138332	1.03	0.0000000	0.00	0.0731911	0.40	0.0321774	9.64	0.0000000	0.00	6.06037	0.39	0.1148555	1.06
17D19987	0.0078845	1.48	0.0000000	0.00	0.0459689	0.64	0.0224020	9.65	0.0000000	0.00	3.80632	0.63	0.0799629	1.08
Σ	1.1156734	0.12	0.0000000	0.00	4.4173633	0.04	0.6423482	2.11	0.0118740	207.95	365.76661	0.03	2.2928264	0.23
Σ									6.1872590	0.46			368.05944	0.03

	40Ar(r) [fA]	%1σ	40Ar(a) [fA]	%1σ	40Ar(c) [fA]	%1σ	40Ar(k) [fA]	%1σ
17D19952	9.56780	15.50	480.2484	0.31	0.0000000	0.00	0.0111141	9.65
17D19954	7.58959	7.06	144.5430	0.37	0.0000000	0.00	0.0082355	9.65
17D19955	4.40131	8.97	91.9071	0.42	0.0000000	0.00	0.0056799	9.65
17D19957	5.12668	5.97	60.7153	0.50	0.0000000	0.00	0.0061956	9.65
17D19958	5.08083	5.67	57.8856	0.49	0.0000000	0.00	0.0066773	9.65
17D19960	9.42149	4.35	96.4311	0.42	0.0000000	0.00	0.0118447	9.65
17D19961	6.88691	4.32	54.0692	0.54	0.0000000	0.00	0.0085914	9.65
17D19963	9.25471	4.06	83.3456	0.44	0.0000000	0.00	0.0125353	9.65
17D19964	10.25672	3.81	80.3876	0.48	0.0000000	0.00	0.0139206	9.65
17D19966	6.27537	4.45	44.7216	0.61	0.0000000	0.00	0.0088614	9.65
17D19967	12.61951	3.35	87.1709	0.48	0.0000000	0.00	0.0165015	9.65
17D19969	14.01077	3.08	83.2307	0.51	0.0000000	0.00	0.0178890	9.65
17D19970	10.37137	3.18	55.7859	0.58	0.0000000	0.00	0.0137306	9.65
17D19972	9.06439	3.67	52.5695	0.62	0.0000000	0.00	0.0123088	9.65
17D19973	11.70761	3.19	63.7346	0.58	0.0000000	0.00	0.0158295	9.65
17D19975	7.93632	3.94	44.8307	0.69	0.0000000	0.00	0.0118204	9.65
17D19976	7.54243	3.98	40.7325	0.73	0.0000000	0.00	0.0107551	9.65
17D19978	4.19350	6.06	28.5155	0.87	0.0000000	0.00	0.0070415	9.65
17D19979	3.69387	6.81	26.2945	0.94	0.0000000	0.00	0.0059373	9.65
17D19981	2.10343	9.33	16.1812	1.17	0.0000000	0.00	0.0034497	9.66
17D19982	2.57372	7.82	16.6016	1.18	0.0000000	0.00	0.0033492	9.66
17D19984	2.13925	10.40	19.7070	1.10	0.0000000	0.00	0.0037629	9.66
17D19985	2.67188	8.64	21.8711	1.03	0.0000000	0.00	0.0036786	9.66
17D19987	1.62709	11.73	12.4658	1.48	0.0000000	0.00	0.0023104	9.67
Σ	166.11652	1.31	1763.9459	0.12	0.0000000	0.00	0.2220203	2.19
Σ							1930.2844	0.16

**Additional Parameters**			40Ar/39Ar	1σ	37Ar/39Ar	1σ	36Ar/39Ar	1σ	Time (days)	37Ar (decay)	39Ar (decay)	40Ar (moles)
17D19952	1.8 %		26.659880	0.038278	5.366394	0.034315	0.089906	0.000302	142.927	16.870453	1.00100989	2.351E-11
17D19954	1.9 %		11.174152	0.020701	5.473437	0.043988	0.037405	0.000148	142.941	16.875082	1.00100998	7.303E-12
17D19955	2.0 %	✓	10.256984	0.026813	5.429615	0.054188	0.034590	0.000165	142.948	16.877396	1.00101003	4.623E-12
17D19957	2.2 %	✓	6.427672	0.015471	5.711402	0.054137	0.021600	0.000110	142.962	16.882027	1.00101013	3.161E-12
17D19958	2.4 %	✓	5.702714	0.013168	5.939328	0.049213	0.019345	0.000095	142.969	16.884343	1.00101018	3.023E-12
17D19960	2.7 %	✓	5.403249	0.007942	6.289090	0.037285	0.018356	0.000073	142.983	16.888975	1.00101028	5.081E-12
17D19961	3.0 %	✓	4.289533	0.008329	6.416943	0.044345	0.014609	0.000073	142.990	16.891292	1.00101033	2.926E-12
17D19963	3.4 %	✓	4.465252	0.006361	6.715171	0.038750	0.015414	0.000063	143.003	16.895927	1.00101042	4.445E-12
17D19964	3.9 %	✓	3.935198	0.005317	7.054680	0.038344	0.013715	0.000058	143.010	16.898244	1.00101047	4.352E-12
17D19966	4.5 %	✓	3.478003	0.006679	7.071931	0.045109	0.012231	0.000065	143.024	16.902881	1.00101057	2.448E-12
17D19967	5.2 %	✓	3.653816	0.004609	7.437445	0.038803	0.012810	0.000053	143.031	16.905199	1.00101062	4.791E-12
17D19969	6.0 %	✓	3.283674	0.003951	7.768108	0.039353	0.011609	0.000049	143.045	16.909838	1.00101072	4.668E-12
17D19970	6.9 %	✓	2.909871	0.004264	8.201617	0.043525	0.010519	0.000049	143.052	16.912157	1.00101077	3.176E-12
17D19972	7.9 %	✓	3.023294	0.005015	8.568371	0.046248	0.011041	0.000055	143.067	16.917030	1.00101087	2.959E-12
17D19973	9.0 %	✓	2.874745	0.003788	10.105446	0.050382	0.010948	0.000047	143.074	16.919350	1.00101092	3.622E-12
17D19975	10.3 %		2.690639	0.004516	11.280730	0.058535	0.010783	0.000053	143.088	16.923992	1.00101102	2.533E-12
17D19976	11.6 %		2.703063	0.004747	12.628102	0.065540	0.011130	0.000055	143.094	16.926314	1.00101107	2.318E-12
17D19978	12.5 %		2.791748	0.007049	15.719782	0.087321	0.012484	0.000073	143.108	16.930958	1.00101117	1.570E-12
17D19979	13.4 %		3.030466	0.008850	18.269643	0.104270	0.013929	0.000087	143.115	16.933281	1.00101121	1.440E-12
17D19981	14.6 %		3.174181	0.015962	21.139600	0.153233	0.015219	0.000124	143.129	16.937927	1.00101131	8.778E-13
17D19982	16.0 %		3.424038	0.016491	23.197451	0.162813	0.016301	0.000130	143.136	16.940250	1.00101136	9.206E-13
17D19984	17.6 %		3.462286	0.014460	27.555313	0.173895	0.018016	0.000126	143.150	16.944898	1.00101146	1.049E-12
17D19985	19.3 %		3.975020	0.017002	28.948472	0.186340	0.019810	0.000138	143.157	16.947222	1.00101151	1.178E-12
17D19987	21.0 %		3.626925	0.025569	32.024406	0.269820	0.019511	0.000194	143.171	16.951872	1.00101161	6.766E-13

**Procedure Blanks**		**36Ar ± 1σ (SE) [fA]**	**37Ar ± 1σ (SE) [fA]**	**38Ar ± 1σ (SE) [fA]**	**39Ar ± 1σ (SE) [fA]**	**40Ar ± 1σ (SE) [fA]**
17D19952	1.8 %	0.0079786 ± 0.0002898	0.1385639 ± 0.0182604	0.0353145 ± 0.0171844	0.0190765 ± 0.0159696	2.3259105 ± 0.0451842
17D19954	1.9 %	0.0081128 ± 0.0002898	0.1647341 ± 0.0182604	0.0385123 ± 0.0171844	0.0178560 ± 0.0159696	2.3122083 ± 0.0451842
17D19955	2.0 %	0.0081496 ± 0.0002898	0.1721113 ± 0.0182604	0.0382525 ± 0.0171844	0.0181642 ± 0.0159696	2.3030896 ± 0.0451842
17D19957	2.2 %	0.0081770 ± 0.0002898	0.1779956 ± 0.0182604	0.0353153 ± 0.0171844	0.0198883 ± 0.0159696	2.2832681 ± 0.0451842
17D19958	2.4 %	0.0081729 ± 0.0002898	0.1774294 ± 0.0182604	0.0330956 ± 0.0171844	0.0210515 ± 0.0159696	2.2735891 ± 0.0451842
17D19960	2.7 %	0.0081414 ± 0.0002898	0.1713673 ± 0.0182604	0.0281415 ± 0.0171844	0.0234458 ± 0.0159696	2.2568622 ± 0.0451842
17D19961	3.0 %	0.0081183 ± 0.0002898	0.1666148 ± 0.0182604	0.0257399 ± 0.0171844	0.0245017 ± 0.0159696	2.2505245 ± 0.0451842
17D19963	3.4 %	0.0080670 ± 0.0002898	0.1552960 ± 0.0182604	0.0217632 ± 0.0171844	0.0259907 ± 0.0159696	2.2432849 ± 0.0451842
17D19964	3.9 %	0.0080422 ± 0.0002898	0.1492895 ± 0.0182604	0.0203960 ± 0.0171844	0.0263265 ± 0.0159696	2.2427797 ± 0.0451842
17D19966	4.5 %	0.0080013 ± 0.0002898	0.1377535 ± 0.0182604	0.0192674 ± 0.0171844	0.0260342 ± 0.0159696	2.2485989 ± 0.0451842
17D19967	5.2 %	0.0079877 ± 0.0002898	0.1326005 ± 0.0182604	0.0195891 ± 0.0171844	0.0253865 ± 0.0159696	2.2550068 ± 0.0451842
17D19969	6.0 %	0.0079789 ± 0.0002898	0.1242372 ± 0.0182604	0.0220557 ± 0.0171844	0.0231360 ± 0.0159696	2.2746357 ± 0.0451842
17D19970	6.9 %	0.0079852 ± 0.0002898	0.1212201 ± 0.0182604	0.0241588 ± 0.0171844	0.0215912 ± 0.0159696	2.2876267 ± 0.0451842
17D19972	7.9 %	0.0080238 ± 0.0002898	0.1176886 ± 0.0182604	0.0301714 ± 0.0171844	0.0177065 ± 0.0159696	2.3207236 ± 0.0451842
17D19973	9.0 %	0.0080548 ± 0.0002898	0.1173497 ± 0.0182604	0.0336159 ± 0.0171844	0.0156967 ± 0.0159696	2.3386723 ± 0.0451842
17D19975	10.3 %	0.0081412 ± 0.0002898	0.1190507 ± 0.0182604	0.0410378 ± 0.0171844	0.0118177 ± 0.0159696	2.3769996 ± 0.0451842
17D19976	11.6 %	0.0081959 ± 0.0002898	0.1209114 ± 0.0182604	0.0447194 ± 0.0171844	0.0101644 ± 0.0159696	2.3965108 ± 0.0451842
17D19978	12.5 %	0.0083259 ± 0.0002898	0.1259750 ± 0.0182604	0.0511290 ± 0.0171844	0.0080675 ± 0.0159696	2.4336679 ± 0.0451842
17D19979	13.4 %	0.0083996 ± 0.0002898	0.1288154 ± 0.0182604	0.0534363 ± 0.0171844	0.0079176 ± 0.0159696	2.4501331 ± 0.0451842
17D19981	14.6 %	0.0085594 ± 0.0002898	0.1339770 ± 0.0182604	0.0550485 ± 0.0171844	0.0102460 ± 0.0159696	2.4754928 ± 0.0451842
17D19982	16.0 %	0.0086430 ± 0.0002898	0.1357522 ± 0.0182604	0.0538080 ± 0.0171844	0.0130958 ± 0.0159696	2.4828930 ± 0.0451842
17D19984	17.6 %	0.0088099 ± 0.0002898	0.1360973 ± 0.0182604	0.0457142 ± 0.0171844	0.0231918 ± 0.0159696	2.4830067 ± 0.0451842
17D19985	19.3 %	0.0088897 ± 0.0002898	0.1339378 ± 0.0182604	0.0381908 ± 0.0171844	0.0308872 ± 0.0159696	2.4739124 ± 0.0451842
17D19987	21.0 %	0.0090324 ± 0.0002898	0.1229021 ± 0.0182604	0.0143588 ± 0.0171844	0.0527923 ± 0.0159696	2.4325107 ± 0.0451842

Intercept Values		36Ar ± 1σ (SE) [fA]	r2	Regression (type,n)	37Ar ± 1σ (SE) [fA]	r2	Regression (type,n)	38Ar ± 1σ (SE) [fA]	r2	Regression (type,n)	39Ar ± 1σ (SE) [fA]	r2	Regression (type,n)	40Ar ± 1σ (SE) [fA]	r2	Regression (type,n)
17D19952	1.8 %	1.5982726 ± 0.0019333	0.9683	EXP 149 of 150	5.6055343 ± 0.0163852	0.7508	EXP 150 of 150	0.4983134 ± 0.0164115	0.0742	EXP 150 of 150	18.2306248 ± 0.0164887	0.9780	EXP 150 of 150	492.153177 ± 0.041736	0.9992	EXP 150 of 150
17D19954	1.9 %	0.4984227 ± 0.0010203	0.8913	EXP 150 of 150	4.1756343 ± 0.0208410	0.5604	EXP 150 of 150	0.2033680 ± 0.0171281	0.0000	EXP 150 of 150	13.5060461 ± 0.0158359	0.9634	EXP 150 of 150	154.452983 ± 0.051548	0.8281	EXP 150 of 150
17D19955	2.0 %	0.3208478 ± 0.0008699	0.7736	EXP 150 of 150	2.7969206 ± 0.0175113	0.3920	EXP 150 of 150	0.1022902 ± 0.0164787	0.0056	EXP 150 of 150	9.3088132 ± 0.0161230	0.9153	EXP 150 of 150	98.617143 ± 0.046204	0.8278	EXP 150 of 150
17D19957	2.2 %	0.2212119 ± 0.0007152	0.5565	EXP 149 of 150	3.2283585 ± 0.0202533	0.3791	EXP 150 of 150	0.1235410 ± 0.0164658	0.0007	EXP 150 of 150	10.1557405 ± 0.0151701	0.9398	EXP 150 of 150	68.131495 ± 0.027435	0.9764	EXP 150 of 150
17D19958	2.4 %	0.2138299 ± 0.0006511	0.6827	EXP 150 of 150	3.6403252 ± 0.0172853	0.6057	EXP 150 of 150	0.1051562 ± 0.0152811	0.0016	EXP 150 of 150	10.9473768 ± 0.0158454	0.9454	EXP 150 of 150	65.246698 ± 0.024134	0.9843	EXP 150 of 150
17D19960	2.7 %	0.3543767 ± 0.0008361	0.8504	EXP 150 of 150	6.9993245 ± 0.0179287	0.8433	EXP 150 of 150	0.2340569 ± 0.0164333	0.0011	EXP 150 of 150	19.4375962 ± 0.0158232	0.9832	EXP 150 of 150	108.121344 ± 0.044937	0.2895	EXP 150 of 150
17D19961	3.0 %	0.2080071 ± 0.0007024	0.5260	EXP 150 of 150	5.1399712 ± 0.0185636	0.7281	EXP 150 of 150	0.1603438 ± 0.0144613	0.0015	EXP 150 of 150	14.0924014 ± 0.0162160	0.9656	EXP 150 of 150	63.215251 ± 0.024207	0.9861	EXP 150 of 150
17D19963	3.4 %	0.3158486 ± 0.0007752	0.8215	EXP 150 of 150	7.9465084 ± 0.0198011	0.8391	EXP 150 of 150	0.2329202 ± 0.0161993	0.0017	EXP 150 of 150	20.5753802 ± 0.0154617	0.9856	EXP 150 of 150	94.856150 ± 0.037440	0.7962	EXP 150 of 150
17D19964	3.9 %	0.3122359 ± 0.0008593	0.7918	EXP 150 of 150	9.3034536 ± 0.0183588	0.8930	EXP 150 of 150	0.2996262 ± 0.0173837	0.0276	EXP 150 of 150	22.8565988 ± 0.0156644	0.9885	EXP 150 of 150	92.901029 ± 0.035987	0.8688	EXP 150 of 150
17D19966	4.5 %	0.1806916 ± 0.0006766	0.5073	EXP 150 of 150	5.8926786 ± 0.0172262	0.7961	EXP 150 of 150	0.1418409 ± 0.0168869	0.0001	EXP 150 of 150	14.5406448 ± 0.0150717	0.9739	EXP 150 of 150	53.254422 ± 0.020843	0.9905	EXP 150 of 150
17D19967	5.2 %	0.3448556 ± 0.0009052	0.8106	EXP 150 of 150	11.6787230 ± 0.0203547	0.9159	EXP 150 of 150	0.3842116 ± 0.0164387	0.0652	EXP 150 of 150	27.1067814 ± 0.0177073	0.9897	EXP 150 of 150	102.061879 ± 0.038704	0.6190	EXP 150 of 150
17D19969	6.0 %	0.3390162 ± 0.0009582	0.7555	EXP 150 of 150	13.2487515 ± 0.0192143	0.9396	EXP 150 of 150	0.3564988 ± 0.0172018	0.0040	EXP 150 of 150	29.3968198 ± 0.0173823	0.9916	EXP 150 of 150	99.533992 ± 0.034335	0.7412	EXP 150 of 150
17D19970	6.9 %	0.2382679 ± 0.0007514	0.6562	EXP 150 of 150	10.7174425 ± 0.0193870	0.9137	EXP 150 of 150	0.2839845 ± 0.0155212	0.0224	EXP 150 of 150	22.5657207 ± 0.0164801	0.9868	EXP 150 of 150	68.458610 ± 0.028463	0.9764	EXP 150 of 150
17D19972	7.9 %	0.2247585 ± 0.0007987	0.5629	EXP 150 of 150	10.0326672 ± 0.0181343	0.9105	EXP 150 of 150	0.2329001 ± 0.0178397	0.0001	EXP 149 of 150	20.2356210 ± 0.0201306	0.9754	EXP 150 of 150	63.966901 ± 0.023194	0.9813	EXP 150 of 150
17D19973	9.0 %	0.2847265 ± 0.0008055	0.7482	EXP 150 of 150	15.2911144 ± 0.0194515	0.9540	EXP 150 of 150	0.3175499 ± 0.0145976	0.0011	EXP 150 of 150	26.0564492 ± 0.0162030	0.9904	EXP 150 of 150	77.796721 ± 0.029481	0.9526	EXP 150 of 150
17D19975	10.3 %	0.2117810 ± 0.0007183	0.6027	EXP 150 of 150	12.7314444 ± 0.0197117	0.9345	EXP 150 of 150	0.2538654 ± 0.0152026	0.0598	EXP 150 of 150	19.4720277 ± 0.0172118	0.9807	EXP 150 of 150	55.155806 ± 0.021646	0.9881	EXP 150 of 150
17D19976	11.6 %	0.1996032 ± 0.0006845	0.5751	EXP 150 of 150	12.9775239 ± 0.0199231	0.9336	EXP 150 of 150	0.2070147 ± 0.0160611	0.0004	EXP 150 of 150	17.7330744 ± 0.0155366	0.9804	EXP 150 of 150	50.682222 ± 0.019791	0.9911	EXP 150 of 150
17D19978	12.5 %	0.1491667 ± 0.0005965	0.4426	EXP 150 of 150	10.5677841 ± 0.0190675	0.9084	EXP 150 of 150	0.1221331 ± 0.0148356	0.0028	EXP 150 of 150	11.6319742 ± 0.0155025	0.9564	EXP 150 of 150	35.149678 ± 0.019307	0.9935	EXP 150 of 150
17D19979	13.4 %	0.1411217 ± 0.0006011	0.4106	EXP 150 of 150	10.3665884 ± 0.0181471	0.9136	EXP 150 of 150	0.0993638 ± 0.0173358	0.0002	EXP 150 of 150	9.8231190 ± 0.0164828	0.9287	EXP 150 of 150	32.444402 ± 0.017651	0.9943	EXP 150 of 150
17D19981	14.6 %	0.0929742 ± 0.0004551	0.1374	EXP 150 of 150	6.9333144 ± 0.0187501	0.8304	EXP 150 of 150	0.0563288 ± 0.0172028	0.0010	EXP 150 of 150	5.7125144 ± 0.0180341	0.7851	EXP 150 of 150	20.763520 ± 0.018303	0.9953	EXP 150 of 150
17D19982	16.0 %	0.0965424 ± 0.0004707	0.0588	EXP 150 of 150	7.4026780 ± 0.0182963	0.8523	EXP 150 of 150	0.0367350 ± 0.0183426	0.0014	EXP 150 of 150	5.5504226 ± 0.0158838	0.7769	EXP 150 of 150	21.661533 ± 0.017053	0.9957	EXP 150 of 150
17D19984	17.6 %	0.1182677 ± 0.0005123	0.4202	EXP 150 of 150	9.9503255 ± 0.0185478	0.9041	EXP 149 of 150	0.0586733 ± 0.0185352	0.0010	EXP 150 of 150	6.2452527 ± 0.0147826	0.8759	EXP 150 of 150	24.333053 ± 0.017617	0.9948	EXP 150 of 150
17D19985	19.3 %	0.1266641 ± 0.0005339	0.2902	EXP 147 of 150	10.2332609 ± 0.0190103	0.9019	EXP 150 of 150	0.0666368 ± 0.0170831	0.0055	EXP 150 of 150	6.1028237 ± 0.0163146	0.8113	EXP 150 of 150	27.020567 ± 0.018832	0.9935	EXP 150 of 150
17D19987	21.0 %	0.0820324 ± 0.0004463	0.0326	EXP 150 of 150	7.0928035 ± 0.0176310	0.8407	EXP 150 of 150	0.0428154 ± 0.0164880	0.0111	EXP 150 of 150	3.8073591 ± 0.0174260	0.5778	EXP 150 of 150	16.527757 ± 0.017070	0.9961	EXP 150 of 150

Project Info		Analyst	Irradiation	X-pos	Y-pos	Z/H-pos	Project	Experiment	Nmb
17D19952	1.8 %	Dan Miggins	17-OSU-01	0.00	0.00	49.18	Arctic\O-Connor (16-22)	17D19948	01
17D19954	1.9 %	Dan Miggins	17-OSU-01	0.00	0.00	49.18	Arctic\O-Connor (16-22)	17D19948	01
17D19955	2.0 %	Dan Miggins	17-OSU-01	0.00	0.00	49.18	Arctic\O-Connor (16-22)	17D19948	01
17D19957	2.2 %	Dan Miggins	17-OSU-01	0.00	0.00	49.18	Arctic\O-Connor (16-22)	17D19948	01
17D19958	2.4 %	Dan Miggins	17-OSU-01	0.00	0.00	49.18	Arctic\O-Connor (16-22)	17D19948	01
17D19960	2.7 %	Dan Miggins	17-OSU-01	0.00	0.00	49.18	Arctic\O-Connor (16-22)	17D19948	01
17D19961	3.0 %	Dan Miggins	17-OSU-01	0.00	0.00	49.18	Arctic\O-Connor (16-22)	17D19948	01
17D19963	3.4 %	Dan Miggins	17-OSU-01	0.00	0.00	49.18	Arctic\O-Connor (16-22)	17D19948	01
17D19964	3.9 %	Dan Miggins	17-OSU-01	0.00	0.00	49.18	Arctic\O-Connor (16-22)	17D19948	01
17D19966	4.5 %	Dan Miggins	17-OSU-01	0.00	0.00	49.18	Arctic\O-Connor (16-22)	17D19948	01
17D19967	5.2 %	Dan Miggins	17-OSU-01	0.00	0.00	49.18	Arctic\O-Connor (16-22)	17D19948	01
17D19969	6.0 %	Dan Miggins	17-OSU-01	0.00	0.00	49.18	Arctic\O-Connor (16-22)	17D19948	01
17D19970	6.9 %	Dan Miggins	17-OSU-01	0.00	0.00	49.18	Arctic\O-Connor (16-22)	17D19948	01
17D19972	7.9 %	Dan Miggins	17-OSU-01	0.00	0.00	49.18	Arctic\O-Connor (16-22)	17D19948	01
17D19973	9.0 %	Dan Miggins	17-OSU-01	0.00	0.00	49.18	Arctic\O-Connor (16-22)	17D19948	01
17D19975	10.3 %	Dan Miggins	17-OSU-01	0.00	0.00	49.18	Arctic\O-Connor (16-22)	17D19948	01
17D19976	11.6 %	Dan Miggins	17-OSU-01	0.00	0.00	49.18	Arctic\O-Connor (16-22)	17D19948	01
17D19978	12.5 %	Dan Miggins	17-OSU-01	0.00	0.00	49.18	Arctic\O-Connor (16-22)	17D19948	01
17D19979	13.4 %	Dan Miggins	17-OSU-01	0.00	0.00	49.18	Arctic\O-Connor (16-22)	17D19948	01
17D19981	14.6 %	Dan Miggins	17-OSU-01	0.00	0.00	49.18	Arctic\O-Connor (16-22)	17D19948	01
17D19982	16.0 %	Dan Miggins	17-OSU-01	0.00	0.00	49.18	Arctic\O-Connor (16-22)	17D19948	01
17D19984	17.6 %	Dan Miggins	17-OSU-01	0.00	0.00	49.18	Arctic\O-Connor (16-22)	17D19948	01
17D19985	19.3 %	Dan Miggins	17-OSU-01	0.00	0.00	49.18	Arctic\O-Connor (16-22)	17D19948	01
17D19987	21.0 %	Dan Miggins	17-OSU-01	0.00	0.00	49.18	Arctic\O-Connor (16-22)	17D19948	01

Sample Parameters		Sample	Material	Location	Standard Name	Standard (in Ma)	%1σ	Standard Reference	Standard 40Ar/39Ar	%1σ	J	%1σ	Air 40Ar/36Ar	%1σ	MDF (lin)	%1σ	Volume Ratio	Sensitivity (mol/volt)	Day	Month	Year	Hour	Min	Resist
17D19952	1.8 %	HLY0102-D35-12	Groundmass	Gakkel Ridge	FCT-NM (1C32-17)	28.201	0.082	Kuiper et al (2008)	9.99322	0.085	0.00157281	0.085	302.422	0.144	0.9942705	0.068	1	4.8E-14	11	JUN	2017	13	54	1
17D19954	1.9 %	HLY0102-D35-12	Groundmass	Gakkel Ridge	FCT-NM (1C32-17)	28.201	0.082	Kuiper et al (2008)	9.99322	0.085	0.00157281	0.085	302.422	0.144	0.9942705	0.068	1	4.8E-14	11	JUN	2017	14	14	1
17D19955	2.0 %	HLY0102-D35-12	Groundmass	Gakkel Ridge	FCT-NM (1C32-17)	28.201	0.082	Kuiper et al (2008)	9.99322	0.085	0.00157281	0.085	302.422	0.144	0.9942705	0.068	1	4.8E-14	11	JUN	2017	14	24	1
17D19957	2.2 %	HLY0102-D35-12	Groundmass	Gakkel Ridge	FCT-NM (1C32-17)	28.201	0.082	Kuiper et al (2008)	9.99322	0.085	0.00157281	0.085	302.422	0.144	0.9942705	0.068	1	4.8E-14	11	JUN	2017	14	44	1
17D19958	2.4 %	HLY0102-D35-12	Groundmass	Gakkel Ridge	FCT-NM (1C32-17)	28.201	0.082	Kuiper et al (2008)	9.99322	0.085	0.00157281	0.085	302.422	0.144	0.9942705	0.068	1	4.8E-14	11	JUN	2017	14	54	1
17D19960	2.7 %	HLY0102-D35-12	Groundmass	Gakkel Ridge	FCT-NM (1C32-17)	28.201	0.082	Kuiper et al (2008)	9.99322	0.085	0.00157281	0.085	302.422	0.144	0.9942705	0.068	1	4.8E-14	11	JUN	2017	15	14	1
17D19961	3.0 %	HLY0102-D35-12	Groundmass	Gakkel Ridge	FCT-NM (1C32-17)	28.201	0.082	Kuiper et al (2008)	9.99322	0.085	0.00157281	0.085	302.422	0.144	0.9942705	0.068	1	4.8E-14	11	JUN	2017	15	24	1
17D19963	3.4 %	HLY0102-D35-12	Groundmass	Gakkel Ridge	FCT-NM (1C32-17)	28.201	0.082	Kuiper et al (2008)	9.99322	0.085	0.00157281	0.085	302.422	0.144	0.9942705	0.068	1	4.8E-14	11	JUN	2017	15	44	1
17D19964	3.9 %	HLY0102-D35-12	Groundmass	Gakkel Ridge	FCT-NM (1C32-17)	28.201	0.082	Kuiper et al (2008)	9.99322	0.085	0.00157281	0.085	302.422	0.144	0.9942705	0.068	1	4.8E-14	11	JUN	2017	15	54	1
17D19966	4.5 %	HLY0102-D35-12	Groundmass	Gakkel Ridge	FCT-NM (1C32-17)	28.201	0.082	Kuiper et al (2008)	9.99322	0.085	0.00157281	0.085	302.422	0.144	0.9942705	0.068	1	4.8E-14	11	JUN	2017	16	14	1
17D19967	5.2 %	HLY0102-D35-12	Groundmass	Gakkel Ridge	FCT-NM (1C32-17)	28.201	0.082	Kuiper et al (2008)	9.99322	0.085	0.00157281	0.085	302.422	0.144	0.9942705	0.068	1	4.8E-14	11	JUN	2017	16	24	1
17D19969	6.0 %	HLY0102-D35-12	Groundmass	Gakkel Ridge	FCT-NM (1C32-17)	28.201	0.082	Kuiper et al (2008)	9.99322	0.085	0.00157281	0.085	302.422	0.144	0.9942705	0.068	1	4.8E-14	11	JUN	2017	16	44	1
17D19970	6.9 %	HLY0102-D35-12	Groundmass	Gakkel Ridge	FCT-NM (1C32-17)	28.201	0.082	Kuiper et al (2008)	9.99322	0.085	0.00157281	0.085	302.422	0.144	0.9942705	0.068	1	4.8E-14	11	JUN	2017	16	54	1
17D19972	7.9 %	HLY0102-D35-12	Groundmass	Gakkel Ridge	FCT-NM (1C32-17)	28.201	0.082	Kuiper et al (2008)	9.99322	0.085	0.00157281	0.085	302.422	0.144	0.9942705	0.068	1	4.8E-14	11	JUN	2017	17	15	1
17D19973	9.0 %	HLY0102-D35-12	Groundmass	Gakkel Ridge	FCT-NM (1C32-17)	28.201	0.082	Kuiper et al (2008)	9.99322	0.085	0.00157281	0.085	302.422	0.144	0.9942705	0.068	1	4.8E-14	11	JUN	2017	17	25	1
17D19975	10.3 %	HLY0102-D35-12	Groundmass	Gakkel Ridge	FCT-NM (1C32-17)	28.201	0.082	Kuiper et al (2008)	9.99322	0.085	0.00157281	0.085	302.422	0.144	0.9942705	0.068	1	4.8E-14	11	JUN	2017	17	45	1
17D19976	11.6 %	HLY0102-D35-12	Groundmass	Gakkel Ridge	FCT-NM (1C32-17)	28.201	0.082	Kuiper et al (2008)	9.99322	0.085	0.00157281	0.085	302.422	0.144	0.9942705	0.068	1	4.8E-14	11	JUN	2017	17	55	1
17D19978	12.5 %	HLY0102-D35-12	Groundmass	Gakkel Ridge	FCT-NM (1C32-17)	28.201	0.082	Kuiper et al (2008)	9.99322	0.085	0.00157281	0.085	302.422	0.144	0.9942705	0.068	1	4.8E-14	11	JUN	2017	18	15	1
17D19979	13.4 %	HLY0102-D35-12	Groundmass	Gakkel Ridge	FCT-NM (1C32-17)	28.201	0.082	Kuiper et al (2008)	9.99322	0.085	0.00157281	0.085	302.422	0.144	0.9942705	0.068	1	4.8E-14	11	JUN	2017	18	25	1
17D19981	14.6 %	HLY0102-D35-12	Groundmass	Gakkel Ridge	FCT-NM (1C32-17)	28.201	0.082	Kuiper et al (2008)	9.99322	0.085	0.00157281	0.085	302.422	0.144	0.9942705	0.068	1	4.8E-14	11	JUN	2017	18	45	1
17D19982	16.0 %	HLY0102-D35-12	Groundmass	Gakkel Ridge	FCT-NM (1C32-17)	28.201	0.082	Kuiper et al (2008)	9.99322	0.085	0.00157281	0.085	302.422	0.144	0.9942705	0.068	1	4.8E-14	11	JUN	2017	18	55	1
17D19984	17.6 %	HLY0102-D35-12	Groundmass	Gakkel Ridge	FCT-NM (1C32-17)	28.201	0.082	Kuiper et al (2008)	9.99322	0.085	0.00157281	0.085	302.422	0.144	0.9942705	0.068	1	4.8E-14	11	JUN	2017	19	15	1
17D19985	19.3 %	HLY0102-D35-12	Groundmass	Gakkel Ridge	FCT-NM (1C32-17)	28.201	0.082	Kuiper et al (2008)	9.99322	0.085	0.00157281	0.085	302.422	0.144	0.9942705	0.068	1	4.8E-14	11	JUN	2017	19	25	1
17D19987	21.0 %	HLY0102-D35-12	Groundmass	Gakkel Ridge	FCT-NM (1C32-17)	28.201	0.082	Kuiper et al (2008)	9.99322	0.085	0.00157281	0.085	302.422	0.144	0.9942705	0.068	1	4.8E-14	11	JUN	2017	19	45	1

Irradiation Constants		40/36(a)	%1σ	40/36(c)	%1σ	38/36(a)	%1σ	38/36(c)	%1σ	39/37(ca)	%1σ	38/37(ca)	%1σ	36/37(ca)	%1σ	40/39(k)	%1σ	38/39(k)	%1σ	36/38(cl)	%1σ	K/Ca	%1σ	K/Cl	%1σ	Ca/Cl	%1σ
17D19952	1.8 %	295.5	0	0.018	35	0.1869	0	1.493	3	0.000643	0.92	0.00018	9.63	0.00027	0.17	0.000607	9.65	0.012077	0.09	0	0	0.43	0	0	0	0	0
17D19954	1.9 %	295.5	0	0.018	35	0.1869	0	1.493	3	0.000643	0.92	0.00018	9.63	0.00027	0.17	0.000607	9.65	0.012077	0.09	0	0	0.43	0	0	0	0	0
17D19955	2.0 %	295.5	0	0.018	35	0.1869	0	1.493	3	0.000643	0.92	0.00018	9.63	0.00027	0.17	0.000607	9.65	0.012077	0.09	0	0	0.43	0	0	0	0	0
17D19957	2.2 %	295.5	0	0.018	35	0.1869	0	1.493	3	0.000643	0.92	0.00018	9.63	0.00027	0.17	0.000607	9.65	0.012077	0.09	0	0	0.43	0	0	0	0	0
17D19958	2.4 %	295.5	0	0.018	35	0.1869	0	1.493	3	0.000643	0.92	0.00018	9.63	0.00027	0.17	0.000607	9.65	0.012077	0.09	0	0	0.43	0	0	0	0	0
17D19960	2.7 %	295.5	0	0.018	35	0.1869	0	1.493	3	0.000643	0.92	0.00018	9.63	0.00027	0.17	0.000607	9.65	0.012077	0.09	0	0	0.43	0	0	0	0	0
17D19961	3.0 %	295.5	0	0.018	35	0.1869	0	1.493	3	0.000643	0.92	0.00018	9.63	0.00027	0.17	0.000607	9.65	0.012077	0.09	0	0	0.43	0	0	0	0	0
17D19963	3.4 %	295.5	0	0.018	35	0.1869	0	1.493	3	0.000643	0.92	0.00018	9.63	0.00027	0.17	0.000607	9.65	0.012077	0.09	0	0	0.43	0	0	0	0	0
17D19964	3.9 %	295.5	0	0.018	35	0.1869	0	1.493	3	0.000643	0.92	0.00018	9.63	0.00027	0.17	0.000607	9.65	0.012077	0.09	0	0	0.43	0	0	0	0	0
17D19966	4.5 %	295.5	0	0.018	35	0.1869	0	1.493	3	0.000643	0.92	0.00018	9.63	0.00027	0.17	0.000607	9.65	0.012077	0.09	0	0	0.43	0	0	0	0	0
17D19967	5.2 %	295.5	0	0.018	35	0.1869	0	1.493	3	0.000643	0.92	0.00018	9.63	0.00027	0.17	0.000607	9.65	0.012077	0.09	0	0	0.43	0	0	0	0	0
17D19969	6.0 %	295.5	0	0.018	35	0.1869	0	1.493	3	0.000643	0.92	0.00018	9.63	0.00027	0.17	0.000607	9.65	0.012077	0.09	0	0	0.43	0	0	0	0	0
17D19970	6.9 %	295.5	0	0.018	35	0.1869	0	1.493	3	0.000643	0.92	0.00018	9.63	0.00027	0.17	0.000607	9.65	0.012077	0.09	0	0	0.43	0	0	0	0	0
17D19972	7.9 %	295.5	0	0.018	35	0.1869	0	1.493	3	0.000643	0.92	0.00018	9.63	0.00027	0.17	0.000607	9.65	0.012077	0.09	0	0	0.43	0	0	0	0	0
17D19973	9.0 %	295.5	0	0.018	35	0.1869	0	1.493	3	0.000643	0.92	0.00018	9.63	0.00027	0.17	0.000607	9.65	0.012077	0.09	0	0	0.43	0	0	0	0	0
17D19975	10.3 %	295.5	0	0.018	35	0.1869	0	1.493	3	0.000643	0.92	0.00018	9.63	0.00027	0.17	0.000607	9.65	0.012077	0.09	0	0	0.43	0	0	0	0	0
17D19976	11.6 %	295.5	0	0.018	35	0.1869	0	1.493	3	0.000643	0.92	0.00018	9.63	0.00027	0.17	0.000607	9.65	0.012077	0.09	0	0	0.43	0	0	0	0	0
17D19978	12.5 %	295.5	0	0.018	35	0.1869	0	1.493	3	0.000643	0.92	0.00018	9.63	0.00027	0.17	0.000607	9.65	0.012077	0.09	0	0	0.43	0	0	0	0	0
17D19979	13.4 %	295.5	0	0.018	35	0.1869	0	1.493	3	0.000643	0.92	0.00018	9.63	0.00027	0.17	0.000607	9.65	0.012077	0.09	0	0	0.43	0	0	0	0	0
17D19981	14.6 %	295.5	0	0.018	35	0.1869	0	1.493	3	0.000643	0.92	0.00018	9.63	0.00027	0.17	0.000607	9.65	0.012077	0.09	0	0	0.43	0	0	0	0	0
17D19982	16.0 %	295.5	0	0.018	35	0.1869	0	1.493	3	0.000643	0.92	0.00018	9.63	0.00027	0.17	0.000607	9.65	0.012077	0.09	0	0	0.43	0	0	0	0	0
17D19984	17.6 %	295.5	0	0.018	35	0.1869	0	1.493	3	0.000643	0.92	0.00018	9.63	0.00027	0.17	0.000607	9.65	0.012077	0.09	0	0	0.43	0	0	0	0	0
17D19985	19.3 %	295.5	0	0.018	35	0.1869	0	1.493	3	0.000643	0.92	0.00018	9.63	0.00027	0.17	0.000607	9.65	0.012077	0.09	0	0	0.43	0	0	0	0	0
17D19987	21.0 %	295.5	0	0.018	35	0.1869	0	1.493	3	0.000643	0.92	0.00018	9.63	0.00027	0.17	0.000607	9.65	0.012077	0.09	0	0	0.43	0	0	0	0	0

17D19948.AGE >>> HLY0102-D35-12 >>> ARCTIC | O-CONNOR (16-22) PROJECT

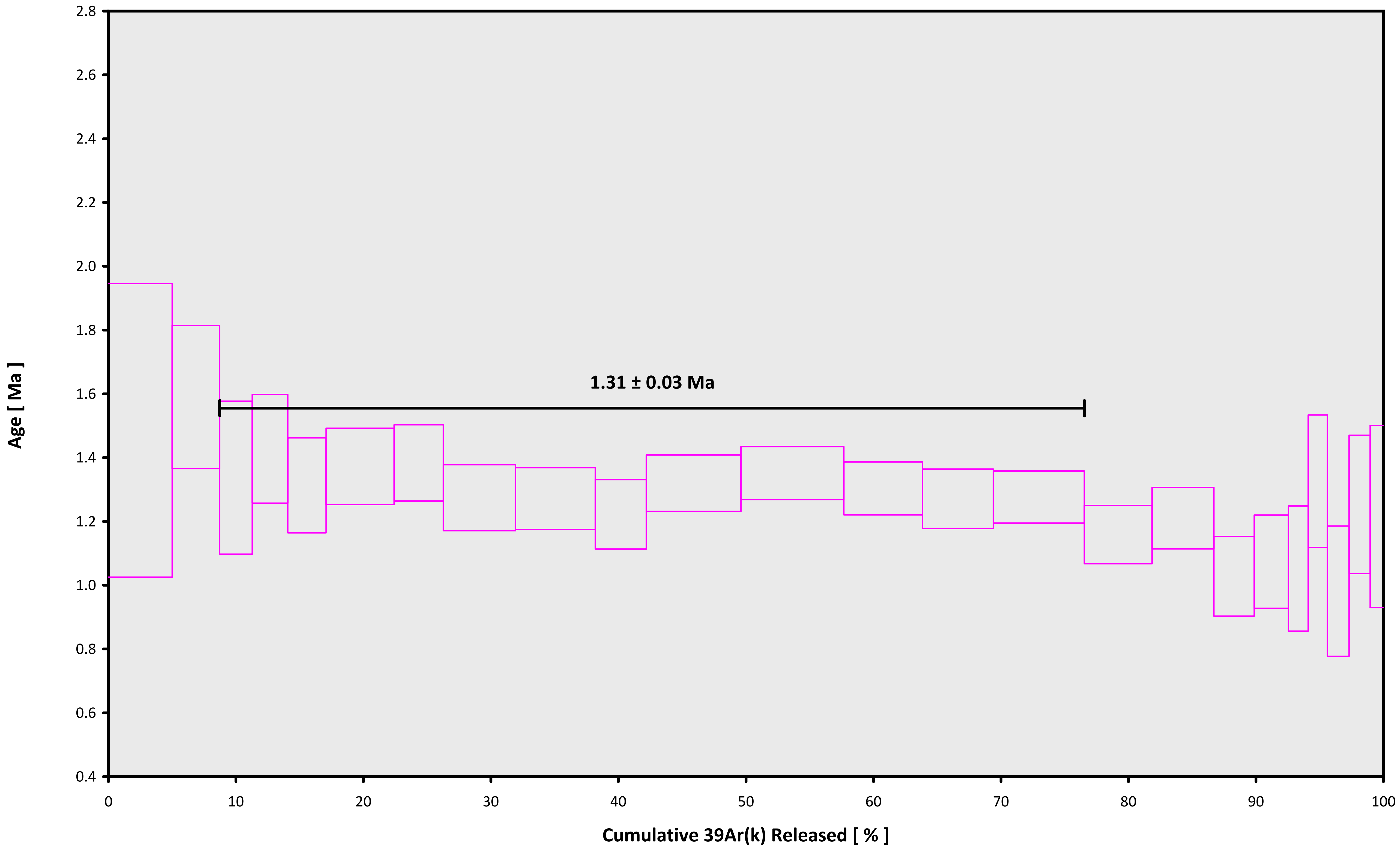

Ar-Ages in Ma

WEIGHTED PLATEAU
1.31 ± 0.03
TOTAL FUSION
1.29 ± 0.03
NORMAL ISOCHRON
1.25 ± 0.09
INVERSE ISOCHRON
1.25 ± 0.09

MSWD (PROBABILITY)
0.89 (56%)

Sample Info

Groundmass
Gakkel Ridge
Dan Miggins

IRR = 17-OSU-01 (1C32-17)
J = 0.00157281 ± 0.00000134

17D19948.AGE >>> HLY0102-D35-12 >>> ARCTIC | O-CONNOR (16-22) PROJECT

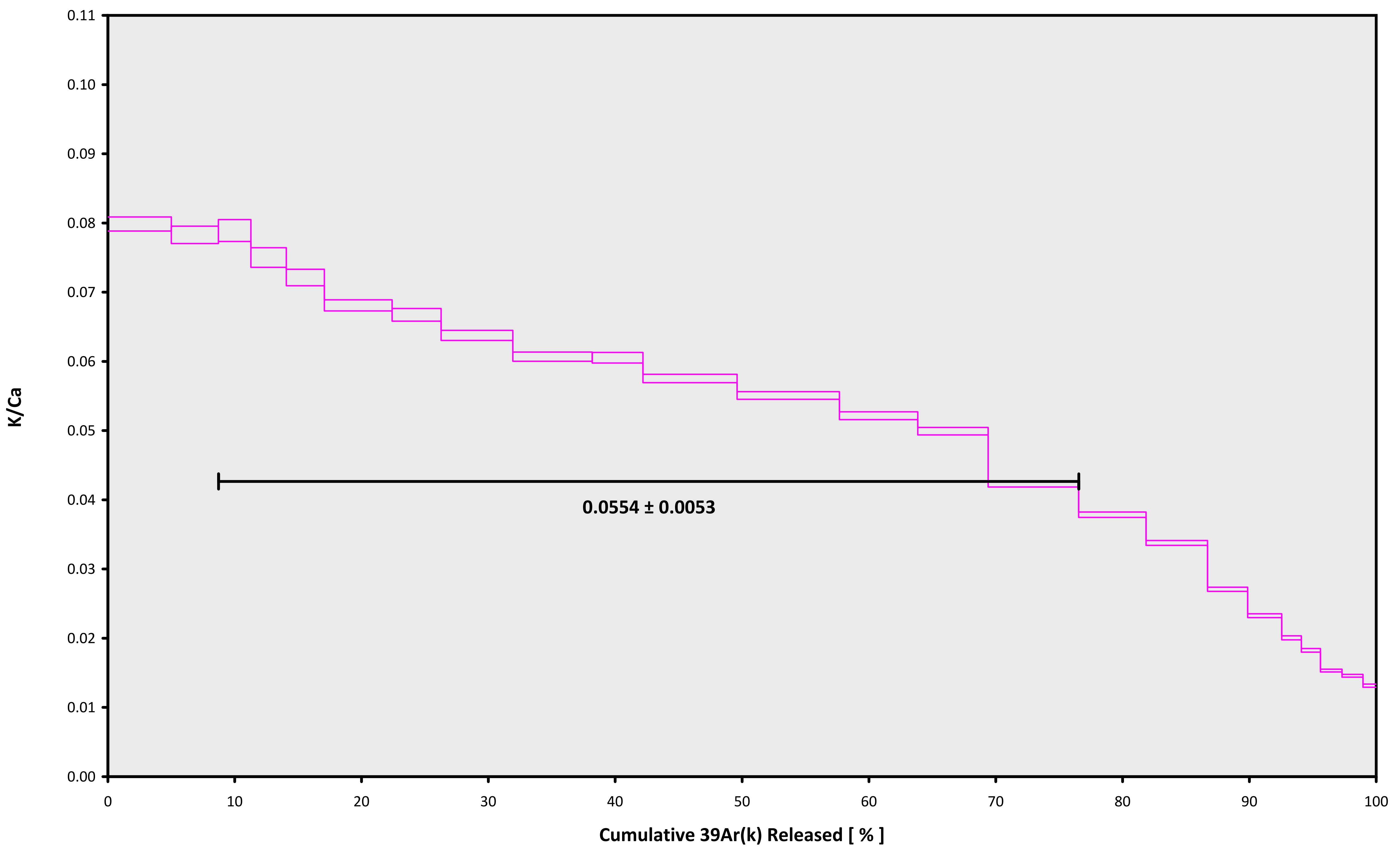

Ar-Ages in Ma

WEIGHTED PLATEAU
1.31 ± 0.03
TOTAL FUSION
1.29 ± 0.03
NORMAL ISOCHRON
1.25 ± 0.09
INVERSE ISOCHRON
1.25 ± 0.09

Sample Info

Groundmass
Gakkel Ridge
Dan Miggins

IRR = 17-OSU-01 (1C32-17)
J = 0.00157281 ± 0.00000134

17D19948.AGE >>> HLY0102-D35-12 >>> ARCTIC | O-CONNOR (16-22) PROJECT

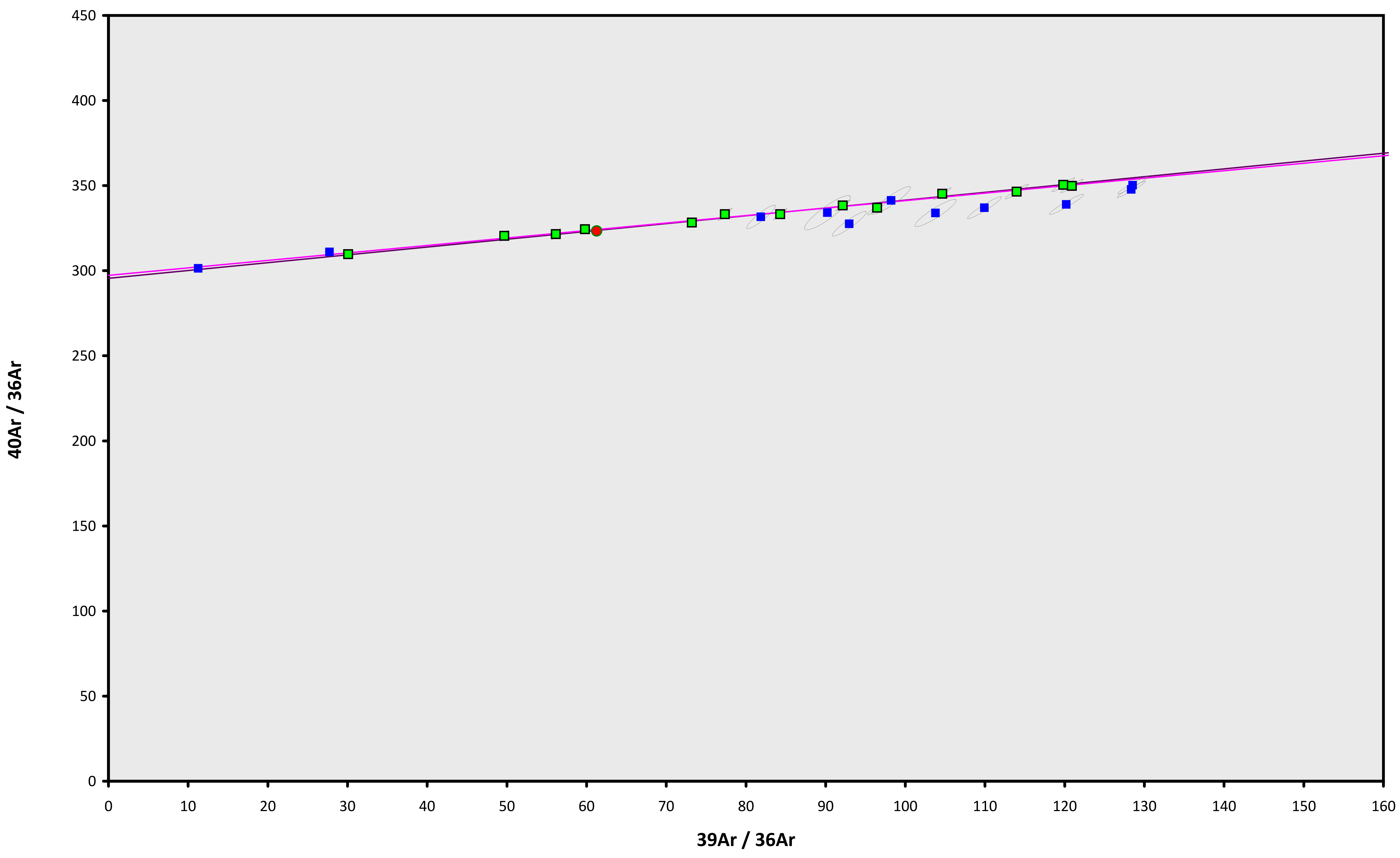

Ar-Ages in Ma

WEIGHTED PLATEAU
1.31 ± 0.03
TOTAL FUSION
1.29 ± 0.03
NORMAL ISOCHRON
1.25 ± 0.09
INVERSE ISOCHRON
1.25 ± 0.09

MSWD (PROBABILITY)
0.80 (64%)

40AR/36AR INTERCEPT
297.2 ± 2.5

Sample Info

Groundmass
Gakkel Ridge
Dan Miggins

IRR = 17-OSU-01 (1C32-17)
J = 0.00157281 ± 0.00000134

17D19948.AGE >>> HLY0102-D35-12 >>> ARCTIC | O-CONNOR (16-22) PROJECT

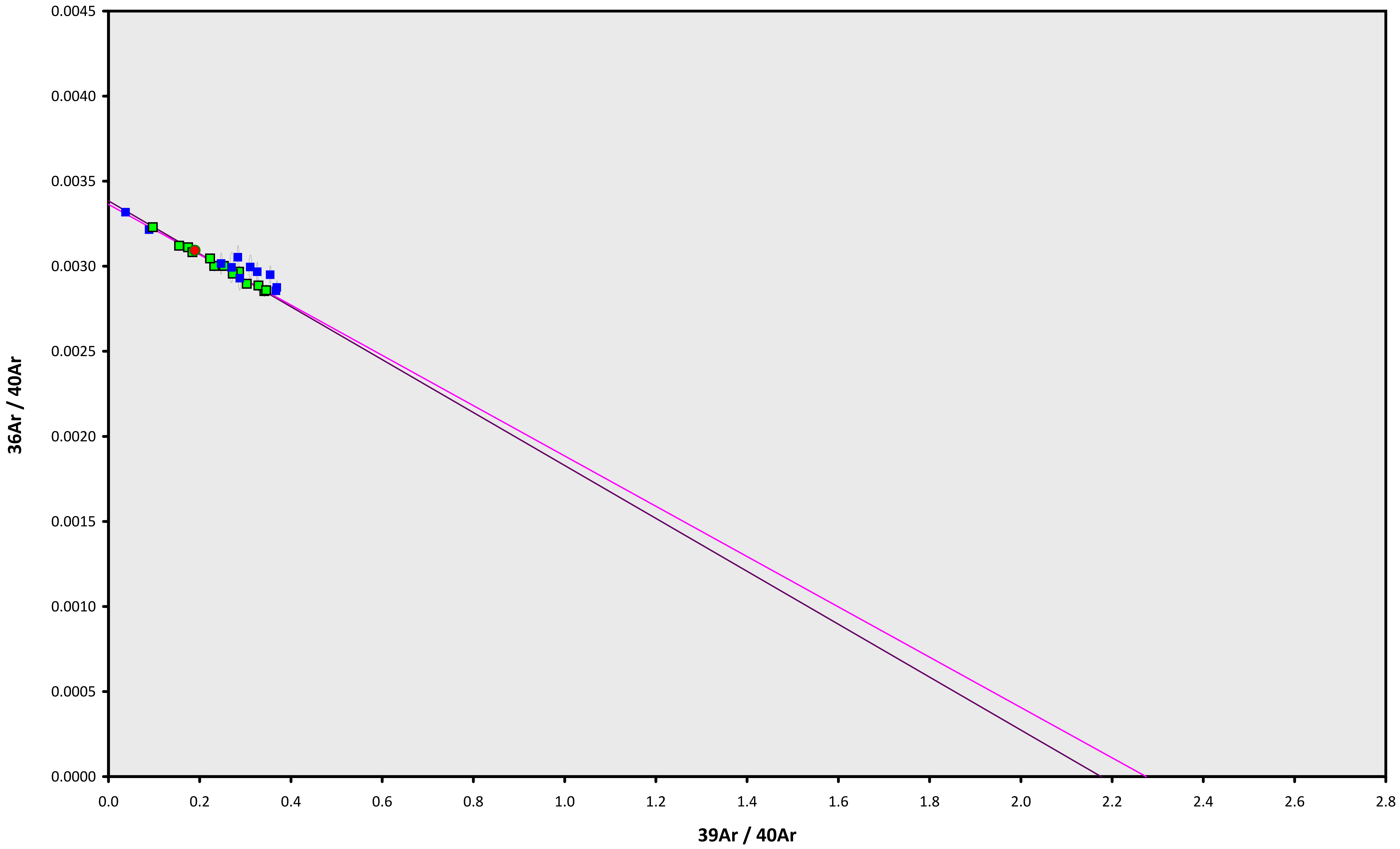

Ar-Ages in Ma

WEIGHTED PLATEAU
1.31 ± 0.03
TOTAL FUSION
1.29 ± 0.03
NORMAL ISOCHRON
1.25 ± 0.09
INVERSE ISOCHRON
1.25 ± 0.09

MSWD (PROBABILITY)
0.80 (64%)

SPREADING FACTOR
10.9%

40AR/36AR INTERCEPT
297.2 ± 2.5

Sample Info

Groundmass
Gakkel Ridge
Dan Miggins

IRR = 17-OSU-01 (1C32-17)
J = 0.00157281 ± 0.00000134

**Relative Abundances**			36Ar [fA]	%1σ	37Ar [fA]	%1σ	38Ar [fA]	%1σ	39Ar [fA]	%1σ	40Ar [fA]	%1σ	40(r)/39(k) ± 2σ	Age ± 2σ (Ma)	40Ar(r) (%)	39Ar(k) (%)	K/Ca ± 2σ
17D20019	1.8 %		0.328067	0.418	6.0867	7.489	0.0612733	39.734	1.56877	1.379	97.4331	0.121	5.28662 ± 1.36787	14.60 ± 3.79	8.49	0.34	0.111 ± 0.017
17D20021	1.9 %		0.209676	0.509	5.6415	7.968	0.0767179	30.959	1.39433	1.544	62.8303	0.188	3.28932 ± 1.03070	9.07 ± 2.85	7.28	0.30	0.106 ± 0.017
17D20022	2.0 %		0.147950	0.624	4.1333	10.633	0.0141366	170.859	1.27290	1.723	44.1568	0.266	2.67095 ± 0.85386	7.36 ± 2.36	7.68	0.27	0.132 ± 0.028
17D20024	2.2 %		0.133856	0.664	4.9986	9.040	0.0172972	134.406	1.42481	1.550	39.6580	0.297	2.28759 ± 0.70674	6.31 ± 1.95	8.20	0.30	0.122 ± 0.022
17D20025	2.4 %		0.108367	0.760	5.8226	7.651	0.0292655	81.526	1.58874	1.448	32.4665	0.362	1.33581 ± 0.54396	3.68 ± 1.50	6.52	0.34	0.117 ± 0.018
17D20027	2.7 %		0.120640	0.694	8.6580	4.933	0.0322640	74.302	2.24999	0.926	36.2303	0.324	0.92676 ± 0.40820	2.55 ± 1.12	5.74	0.48	0.111 ± 0.011
17D20028	3.0 %		0.107698	0.770	10.7663	4.329	0.0521506	45.746	2.84609	0.845	33.1008	0.355	0.29387 ± 0.30087	0.81 ± 0.83	2.52	0.61	0.113 ± 0.010
17D20030	3.4 %		0.114376	0.778	15.2049	2.764	0.0993422	24.951	3.90127	0.561	36.0000	0.327	0.07516 ± 0.23157	0.21 ± 0.64	0.81	0.83	0.110 ± 0.006
17D20031	3.9 %		0.143454	0.618	22.8484	1.956	0.0963953	23.801	5.58517	0.416	45.0822	0.261	0.11204 ± 0.18318	0.31 ± 0.50	1.38	1.19	0.105 ± 0.004
17D20033	4.5 %		0.180528	0.539	32.8626	1.490	0.1733653	12.957	7.87494	0.298	58.1678	0.202	0.32877 ± 0.15477	0.90 ± 0.43	4.44	1.68	0.103 ± 0.003
17D20034	5.2 %		0.248295	0.480	50.0586	1.024	0.2520680	9.623	11.97387	0.209	83.0653	0.144	0.58941 ± 0.13405	1.62 ± 0.37	8.47	2.56	0.103 ± 0.002
17D20036	6.0 %	✓	0.422468	0.391	74.1946	0.781	0.3566608	6.895	17.34822	0.158	143.2871	0.091	0.74955 ± 0.15085	2.06 ± 0.41	9.05	3.71	0.100 ± 0.002
17D20037	6.9 %	✓	0.648702	0.350	96.9695	0.636	0.5117336	4.802	22.62789	0.131	218.7472	0.063	0.76046 ± 0.17516	2.09 ± 0.48	7.84	4.84	0.100 ± 0.001
17D20039	7.9 %	✓	0.601181	0.360	95.5259	0.656	0.4761112	5.048	21.31931	0.128	203.5447	0.068	0.81006 ± 0.17267	2.23 ± 0.47	8.46	4.56	0.096 ± 0.001
17D20040	9.0 %	✓	0.706729	0.350	127.9140	0.576	0.6048335	4.083	26.82152	0.112	238.6524	0.055	0.78471 ± 0.15973	2.16 ± 0.44	8.79	5.73	0.090 ± 0.001
17D20042	10.3 %	✓	0.793775	0.330	159.6267	0.531	0.6783911	3.552	31.60011	0.100	265.7836	0.045	0.72064 ± 0.15030	1.98 ± 0.41	8.54	6.75	0.085 ± 0.001
17D20043	11.6 %	✓	1.407231	0.307	364.8351	0.472	1.4605481	1.691	67.60495	0.078	478.5807	0.026	0.81335 ± 0.12159	2.24 ± 0.33	11.45	14.45	0.079 ± 0.001
17D20045	12.5 %	✓	1.102821	0.318	297.5200	0.478	1.1573846	2.177	55.33941	0.081	379.0212	0.032	0.86897 ± 0.11664	2.39 ± 0.32	12.64	11.83	0.080 ± 0.001
17D20046	13.4 %	✓	0.783585	0.330	334.3773	0.474	1.0550747	2.277	51.23290	0.085	271.2326	0.045	0.91615 ± 0.08664	2.52 ± 0.24	17.23	10.94	0.066 ± 0.001
17D20048	14.6 %	✓	0.534464	0.372	304.3481	0.478	0.7408452	3.185	36.00824	0.096	176.2441	0.068	0.83209 ± 0.08305	2.29 ± 0.23	16.91	7.68	0.051 ± 0.000
17D20049	16.0 %	✓	0.454873	0.382	361.9109	0.472	0.6799789	3.797	33.07698	0.100	140.9427	0.086	0.77064 ± 0.07349	2.12 ± 0.20	17.96	7.04	0.039 ± 0.000
17D20051	17.6 %	✓	0.468944	0.378	413.1330	0.471	0.7155875	3.383	35.12761	0.097	142.9157	0.083	0.78051 ± 0.06975	2.15 ± 0.19	19.04	7.48	0.036 ± 0.000
17D20052	19.3 %	✓	0.249135	0.458	271.4633	0.485	0.4504718	5.556	20.51785	0.132	72.1218	0.164	0.74732 ± 0.06462	2.06 ± 0.18	21.08	4.36	0.032 ± 0.000
17D20054	21.0 %	✓	0.091459	0.874	103.5800	0.633	0.1645545	13.911	8.04781	0.281	26.5227	0.442	0.74798 ± 0.08656	2.06 ± 0.24	22.51	1.71	0.033 ± 0.000
		Σ	10.108272	0.092	3172.4798	0.152	9.9564514	1.188	468.35368	0.031	3325.7878	0.018					

Information on Analysis and Constants Used in Calculations

Project = **O-CONNOR (16-22)**
Sample = **HLY0102-D37-8**
Material = **Groundmass**
Location = **Gakkel Ridge**
Region = **Artic Ocean**
Analyst = **Dan Miggins**
Irradiation = **17-OSU-01 (1C41-17)**
Position = **X: 0 | Y: 0 | Z/H: 62.18691 mm**
FCT-NM Age = **28.201 ± 0.023 Ma**
FCT-NM Reference = **Kuiper et al (2008)**
FCT-NM 40Ar/39Ar Ratio = **10.32999 ± 0.00847**
FCT-NM J-value = **0.00152153 ± 0.00000125**
Air Shot 40Ar/36Ar = **302.3710 ± 0.4324**
Air Shot MDF = **0.99431172 ± 0.00067715 (LIN)**
Experiment Type = **Incremental Heating**
Extraction Method = **Bulk Laser Heating**
Heating = **64 sec**
Isolation = **3.00 min**
Instrument = **ARGUS-VI-D**
Preferred Age = **Plateau Age**
Age Classification = **Crystallization Age**
IGSN = Undefined
Rock Class = **Igneous>Volcanic**
Lithology = **Basaltic Lava**
Lat-Lon = Undefined - Undefined

Age Equations = **Min et al. (2000)**
Negative Intensities = **Allowed**
Collector Calibrations = **36Ar**
Decay 40K = **5.530 ± 0.048 E-10 1/a**
Decay 39Ar = **2.940 ± 0.016 E-07 1/h**
Decay 37Ar = **8.230 ± 0.012 E-04 1/h**
Decay 36Cl = **2.257 ± 0.015 E-06 1/a**
Decay 40K(EC,β⁺) = **0.580 ± 0.009 E-10 1/a**
Decay 40K(β⁻) = **4.950 ± 0.043 E-10 1/a**
Atmospheric 40/36(a) = **323.83 ± 2.94**
Atmospheric 38/36(a) = **0.1869**
Production 39/37(ca) = **0.0006425 ± 0.0000059**
Production 38/37(ca) = **0.0001800 ± 0.0000173**
Production 36/37(ca) = **0.0002703 ± 0.0000005**
Production 40/39(k) = **0.000607 ± 0.000059**
Production 38/39(k) = **0.012077 ± 0.000011**
Production 36/38(cl) = **262.80 ± 1.71**
Scaling Ratio K/Ca = **0.430**
Abundance Ratio 40K/K = **1.1700 ± 0.0100 E-04**
Atomic Weight K = **39.0983 ± 0.0001 g**

**Results**	40(a)/36(a) ± 2σ	40(r)/39(k) ± 2σ	Age ± 2σ (Ma)	MSWD	39Ar(k) (%,n)	K/Ca ± 2σ
**Age Plateau**		0.79260 ± 0.03093 ± 3.90%	**2.18** ± 0.09 ± 3.90%	1.32 20%	91.08 13	0.047 ± 0.011
		Full External Error	± 0.10	1.82	2σ Confidence Limit	
		Analytical Error	± 0.09	1.1476	Error Magnification	
**Total Fusion Age**		0.70766 ± 0.03352 ± 4.74%	**1.95** ± 0.09 ± 4.74%		24	0.063 ± 0.000
		Full External Error	± 0.10			
		Analytical Error	± 0.09			
**Normal Isochron** Error Chron	**323.92** ± 5.79 ± 1.79%	0.80300 ± 0.09550 ± 11.89%	**2.21** ± 0.26 ± 11.89%	7.70 0%	91.08 13	
		Full External Error	± 0.27	1.85	2σ Confidence Limit	
		Analytical Error	± 0.26	2.7741	Error Magnification	
				6	Number of Iterations	
				0.0000059488	Convergence	
**Inverse Isochron** Error Chron	**323.83** ± 5.88 ± 1.81%	0.80666 ± 0.09465 ± 11.73%	**2.22** ± 0.26 ± 11.73%	7.83 0%	91.08 13	
		Full External Error	± 0.26	1.85	2σ Confidence Limit	
		Analytical Error	± 0.26	2.7979	Error Magnification	
				3	Number of Iterations	
				0.0002333611	Convergence	
				16%	Spreading Factor	

Notes

Excess Initial 40Ar/36Ar = 323.83 ± 0.91 (%SD).

Incremental Heating			36Ar(a) [fA]	37Ar(ca) [fA]	38Ar(cl) [fA]	39Ar(k) [fA]	40Ar(r) [fA]	Age ± 2σ (Ma)	40Ar(r) (%)	39Ar(k) (%)	K/Ca ± 2σ
17D20019	1.8 %		0.3264213	6.0867	0.0000000	1.56486	8.27281	14.60 ± 3.79	8.49	0.34	0.111 ± 0.017
17D20021	1.9 %		0.2081460	5.6415	0.0200044	1.39070	4.57446	9.07 ± 2.85	7.28	0.30	0.106 ± 0.017
17D20022	2.0 %		0.1468326	4.1333	0.0000000	1.27024	3.39275	7.36 ± 2.36	7.68	0.27	0.132 ± 0.028
17D20024	2.2 %		0.1325054	4.9986	0.0000000	1.42160	3.25203	6.31 ± 1.95	8.20	0.30	0.122 ± 0.022
17D20025	2.4 %		0.1067932	5.8226	0.0000000	1.58500	2.11726	3.68 ± 1.50	6.52	0.34	0.117 ± 0.018
17D20027	2.7 %		0.1182998	8.6580	0.0000000	2.24442	2.08005	2.55 ± 1.12	5.74	0.48	0.111 ± 0.011
17D20028	3.0 %		0.1047876	10.7663	0.0000000	2.83918	0.83434	0.81 ± 0.83	2.52	0.61	0.113 ± 0.010
17D20030	3.4 %		0.1102589	15.2049	0.0290002	3.89150	0.29249	0.21 ± 0.64	0.81	0.83	0.110 ± 0.006
17D20031	3.9 %		0.1372779	22.8484	0.0000000	5.57049	0.62414	0.31 ± 0.50	1.38	1.19	0.105 ± 0.004
17D20033	4.5 %		0.1716362	32.8626	0.0405206	7.85383	2.58209	0.90 ± 0.43	4.44	1.68	0.103 ± 0.003
17D20034	5.2 %		0.2347512	50.0586	0.0549625	11.94170	7.03855	1.62 ± 0.37	8.47	2.56	0.103 ± 0.002
17D20036	6.0 %	✓	0.4023992	74.1946	0.0591585	17.30055	12.96769	2.06 ± 0.41	9.05	3.71	0.100 ± 0.002
17D20037	6.9 %	✓	0.6224664	96.9695	0.1054154	22.56559	17.16022	2.09 ± 0.48	7.84	4.84	0.100 ± 0.001
17D20039	7.9 %	✓	0.5753380	95.5259	0.0946538	21.25793	17.22015	2.23 ± 0.47	8.46	4.56	0.096 ± 0.001
17D20040	9.0 %	✓	0.6721229	127.9140	0.1332583	26.73934	20.98261	2.16 ± 0.44	8.79	5.73	0.090 ± 0.001
17D20042	10.3 %	✓	0.7505980	159.6267	0.1289757	31.49755	22.69830	1.98 ± 0.41	8.54	6.75	0.085 ± 0.001
17D20043	11.6 %	✓	1.3085373	364.8351	0.3366781	67.37054	54.79615	2.24 ± 0.33	11.45	14.45	0.079 ± 0.001
17D20045	12.5 %	✓	1.0223434	297.5200	0.2467295	55.14826	47.92223	2.39 ± 0.32	12.64	11.83	0.080 ± 0.001
17D20046	13.4 %	✓	0.6931451	334.3773	0.2491929	51.01806	46.74042	2.52 ± 0.24	17.23	10.94	0.066 ± 0.001
17D20048	14.6 %	✓	0.4521597	304.3481	0.1690439	35.81270	29.79950	2.29 ± 0.23	16.91	7.68	0.051 ± 0.000
17D20049	16.0 %	✓	0.3570135	361.9109	0.1514467	32.84445	25.31111	2.12 ± 0.20	17.96	7.04	0.039 ± 0.000
17D20051	17.6 %	✓	0.3572380	413.1330	0.1534254	34.86217	27.21019	2.15 ± 0.19	19.04	7.48	0.036 ± 0.000
17D20052	19.3 %	✓	0.1757294	271.4633	0.1230769	20.34343	15.20304	2.06 ± 0.18	21.08	4.36	0.032 ± 0.000
17D20054	21.0 %	✓	0.0634530	103.5800	0.0376610	7.98126	5.96984	2.06 ± 0.24	22.51	1.71	0.033 ± 0.000
		Σ	9.2502539	3172.4798	2.1332038	466.31536	329.99502				

Information on Analysis

Project = **O-CONNOR (16-22)**
Sample = **HLY0102-D37-8**
Material = **Groundmass**
Location = **Gakkel Ridge**
Region = **Artic Ocean**
Analyst = **Dan Miggins**
Irradiation = **17-OSU-01 (1C41-17)**
J = **0.00152153 ± 0.00000125**
FCT-NM = **28.201 ± 0.023 Ma**

Results	40(r)/39(k) ± 2σ		Age ± 2σ (Ma)		MSWD	39Ar(k) (%,n)	K/Ca ± 2σ
**Age Plateau**	0.79260	± 0.03093<br>± 3.90%	**2.18**	**± 0.09**<br>**± 3.90%**	1.32<br>20%	91.08<br>13	0.047 ± 0.011
		Full External Error		± 0.10	1.82	2σ Confidence Limit	
		Analytical Error		± 0.09	1.1476	Error Magnification	
**Total Fusion Age**	0.70766	± 0.03352<br>± 4.74%	**1.95**	**± 0.09**<br>**± 4.74%**		24	0.063 ± 0.000
		Full External Error		± 0.10			
		Analytical Error		± 0.09			

Normal Isochron			39(k)/36(a) ± 2σ	40(a+r)/36(a) ± 2σ	r.i.
17D20019	1.8 %		4.79 ± 0.14	298.49 ± 2.62	0.2807
17D20021	1.9 %		6.68 ± 0.22	301.85 ± 3.31	0.2972
17D20022	2.0 %		8.65 ± 0.32	300.72 ± 4.14	0.3177
17D20024	2.2 %		10.73 ± 0.36	299.29 ± 4.43	0.3662
17D20025	2.4 %		14.84 ± 0.49	304.00 ± 5.23	0.4293
17D20027	2.7 %		18.97 ± 0.44	306.25 ± 4.81	0.5557
17D20028	3.0 %		27.09 ± 0.63	315.87 ± 5.53	0.6283
17D20030	3.4 %		35.29 ± 0.70	326.48 ± 5.72	0.7634
17D20031	3.9 %		40.58 ± 0.63	328.38 ± 4.61	0.7818
17D20033	4.5 %		45.76 ± 0.59	338.87 ± 4.12	0.8360
17D20034	5.2 %		50.87 ± 0.56	353.81 ± 3.75	0.8905
17D20036	6.0 %	✓	42.99 ± 0.38	356.06 ± 3.01	0.9115
17D20037	6.9 %	✓	36.25 ± 0.28	351.40 ± 2.61	0.9279
17D20039	7.9 %	✓	36.95 ± 0.29	353.76 ± 2.71	0.9315
17D20040	9.0 %	✓	39.78 ± 0.31	355.05 ± 2.65	0.9460
17D20042	10.3 %	✓	41.96 ± 0.31	354.07 ± 2.51	0.9535
17D20043	11.6 %	✓	51.49 ± 0.35	365.71 ± 2.44	0.9702
17D20045	12.5 %	✓	53.94 ± 0.38	370.70 ± 2.57	0.9694
17D20046	13.4 %	✓	73.60 ± 0.57	391.26 ± 2.99	0.9690
17D20048	14.6 %	✓	79.20 ± 0.73	389.73 ± 3.54	0.9669
17D20049	16.0 %	✓	92.00 ± 0.95	394.73 ± 4.05	0.9667
17D20051	17.6 %	✓	97.59 ± 1.03	400.00 ± 4.22	0.9704
17D20052	19.3 %	✓	115.77 ± 1.61	410.34 ± 5.77	0.9545
17D20054	21.0 %	✓	125.78 ± 3.33	417.91 ± 11.42	0.9243

Results	40(a)/36(a) ± 2σ		40(r)/39(k) ± 2σ		Age ± 2σ (Ma)		MSWD
Normal Isochron<br>Error Chron	**323.92**	**± 5.79**<br>**± 1.79%**	0.80300	± 0.09550<br>± 11.89%	**2.21**	**± 0.26**<br>**± 11.89%**	7.70<br>0%
					Full External Error	± 0.27	
					Analytical Error	± 0.26	

Statistics				
	2σ Confidence Limit	1.85	Convergence	0.000005948779
	Error Magnification	2.7741	Number of Iterations	6
	Number of Data Points	13	Calculated Line	Weighted York-2

Inverse Isochron			39(k)/40(a+r) ± 2σ	36(a)/40(a+r) ± 2σ	r.i.
17D20019	1.8 %		0.0160610 ± 0.0004459	0.00335024 ± 0.00002944	0.0241
17D20021	1.9 %		0.0221345 ± 0.0006902	0.00331287 ± 0.00003638	0.0412
17D20022	2.0 %		0.0287671 ± 0.0010052	0.00332531 ± 0.00004573	0.0590
17D20024	2.2 %		0.0358471 ± 0.0011337	0.00334127 ± 0.00004942	0.0752
17D20025	2.4 %		0.0488211 ± 0.0014609	0.00328943 ± 0.00005655	0.1017
17D20027	2.7 %		0.0619511 ± 0.0012184	0.00326534 ± 0.00005124	0.1359
17D20028	3.0 %		0.0857782 ± 0.0015751	0.00316588 ± 0.00005546	0.1563
17D20030	3.4 %		0.1081044 ± 0.0014053	0.00306295 ± 0.00005369	0.1872
17D20031	3.9 %		0.1235721 ± 0.0012161	0.00304528 ± 0.00004275	0.1974
17D20033	4.5 %		0.1350313 ± 0.0009743	0.00295095 ± 0.00003585	0.1870
17D20034	5.2 %		0.1437754 ± 0.0007309	0.00282635 ± 0.00003000	0.1536
17D20036	6.0 %	✓	0.1207493 ± 0.0004409	0.00280855 ± 0.00002371	0.1085
17D20037	6.9 %	✓	0.1031648 ± 0.0002997	0.00284578 ± 0.00002113	0.0734
17D20039	7.9 %	✓	0.1044452 ± 0.0003037	0.00282677 ± 0.00002165	0.0823
17D20040	9.0 %	✓	0.1120506 ± 0.0002810	0.00281652 ± 0.00002102	0.0651
17D20042	10.3 %	✓	0.1185168 ± 0.0002612	0.00282430 ± 0.00001999	0.0531
17D20043	11.6 %	✓	0.1407836 ± 0.0002331	0.00273444 ± 0.00001824	0.0238
17D20045	12.5 %	✓	0.1455146 ± 0.0002537	0.00269756 ± 0.00001874	0.0334
17D20046	13.4 %	✓	0.1881186 ± 0.0003612	0.00255583 ± 0.00001950	0.0542
17D20048	14.6 %	✓	0.2032245 ± 0.0004782	0.00256585 ± 0.00002331	0.0857
17D20049	16.0 %	✓	0.2330670 ± 0.0006189	0.00253340 ± 0.00002599	0.1078
17D20051	17.6 %	✓	0.2439713 ± 0.0006276	0.00250001 ± 0.00002635	0.1022
17D20052	19.3 %	✓	0.2821187 ± 0.0011914	0.00243698 ± 0.00003426	0.1805
17D20054	21.0 %	✓	0.3009774 ± 0.0031598	0.00239284 ± 0.00006538	0.2725

Results	40(a)/36(a) ± 2σ	40(r)/39(k) ± 2σ	Age ± 2σ (Ma)	MSWD
Inverse Isochron<br>Error Chron	**323.83** **± 5.88**<br>**± 1.81%**	0.80666 ± 0.09465<br>± 11.73%	**2.22** **± 0.26**<br>**± 11.73%**<br>Full External Error ± 0.26<br>Analytical Error ± 0.26	7.83<br>0%

Statistics				
	2σ Confidence Limit	1.85	Convergence	0.0002333611
	Error Magnification	2.7979	Number of Iterations	3
	Number of Data Points	13	Calculated Line	Weighted York-2
	Spreading Factor	16.0%		

Degassing Patterns			36Ar(a) [fA]	%1σ	36Ar(c) [fA]	%1σ	36Ar(ca) [fA]	%1σ	36Ar(cl) [fA]	%1σ	37Ar(ca) [fA]	%1σ	38Ar(a) [fA]	%1σ	38Ar(c) [fA]	%1σ	38Ar(k) [fA]	%1σ	38Ar(ca) [fA]	%1σ	38Ar(cl) [fA]	%1σ	39Ar(k) [fA]	%1σ	39Ar(ca) [fA]	%1σ	40Ar(r) [fA]	%1σ	40Ar(a) [fA]	%1σ	40Ar(c) [fA]	%1σ	40Ar(k) [fA]	%1σ
17D20019	1.8 %		0.3264213	0.42	0.0000000	0.00	0.0016452	7.49	0.0000000	0.00	6.0867	7.49	0.0610081	0.42	0.0000000	0.00	0.0188988	1.39	0.0010956	12.20	0.0000000	0.00	1.56486	1.38	0.0039107	7.54	8.27281	12.86	105.7050	1.00	0.0000000	0.00	0.0009499	9.75
17D20021	1.9 %		0.2081460	0.52	0.0000000	0.00	0.0015249	7.97	0.0000047	118.75	5.6415	7.97	0.0389025	0.52	0.0000000	0.00	0.0167955	1.55	0.0010155	12.50	0.0200044	118.76	1.39070	1.55	0.0036247	8.02	4.57446	15.59	67.4039	1.04	0.0000000	0.00	0.0008442	9.77
17D20022	2.0 %		0.1468326	0.63	0.0000000	0.00	0.0011172	10.63	0.0000000	0.00	4.1333	10.63	0.0274430	0.63	0.0000000	0.00	0.0153407	1.73	0.0007440	14.35	0.0000000	0.00	1.27024	1.73	0.0026556	10.67	3.39275	15.89	47.5488	1.11	0.0000000	0.00	0.0007710	9.80
17D20024	2.2 %		0.1325054	0.68	0.0000000	0.00	0.0013511	9.04	0.0000000	0.00	4.9986	9.04	0.0247653	0.68	0.0000000	0.00	0.0171686	1.56	0.0008997	13.21	0.0000000	0.00	1.42160	1.55	0.0032116	9.09	3.25203	15.37	42.9092	1.13	0.0000000	0.00	0.0008629	9.77
17D20025	2.4 %		0.1067932	0.78	0.0000000	0.00	0.0015738	7.65	0.0000000	0.00	5.8226	7.65	0.0199596	0.78	0.0000000	0.00	0.0191421	1.45	0.0010481	12.30	0.0000000	0.00	1.58500	1.45	0.0037410	7.71	2.11726	20.31	34.5828	1.20	0.0000000	0.00	0.0009621	9.76
17D20027	2.7 %		0.1182998	0.71	0.0000000	0.00	0.0023403	4.94	0.0000000	0.00	8.6580	4.93	0.0221102	0.71	0.0000000	0.00	0.0271059	0.93	0.0015584	10.82	0.0000000	0.00	2.24442	0.93	0.0055628	5.02	2.08005	22.00	38.3090	1.15	0.0000000	0.00	0.0013624	9.69
17D20028	3.0 %		0.1047876	0.80	0.0000000	0.00	0.0029101	4.33	0.0000000	0.00	10.7663	4.33	0.0195848	0.80	0.0000000	0.00	0.0342887	0.85	0.0019379	10.56	0.0000000	0.00	2.83918	0.85	0.0069173	4.43	0.83434	51.18	33.9334	1.21	0.0000000	0.00	0.0017234	9.69
17D20030	3.4 %		0.1102589	0.81	0.0000000	0.00	0.0041099	2.77	0.0000068	85.49	15.2049	2.76	0.0206074	0.81	0.0000000	0.00	0.0469977	0.57	0.0027369	10.02	0.0290002	85.50	3.89150	0.56	0.0097691	2.91	0.29249	154.05	35.7052	1.22	0.0000000	0.00	0.0023621	9.67
17D20031	3.9 %		0.1372779	0.65	0.0000000	0.00	0.0061759	1.96	0.0000000	0.00	22.8484	1.96	0.0256572	0.65	0.0000000	0.00	0.0672748	0.43	0.0041127	9.83	0.0000000	0.00	5.57049	0.42	0.0146801	2.16	0.62414	81.75	44.4547	1.12	0.0000000	0.00	0.0033813	9.66
17D20033	4.5 %		0.1716362	0.57	0.0000000	0.00	0.0088828	1.50	0.0000094	55.47	32.8626	1.49	0.0320788	0.57	0.0000000	0.00	0.0948507	0.31	0.0059153	9.74	0.0405206	55.48	7.85383	0.30	0.0211142	1.75	2.58209	23.54	55.5809	1.07	0.0000000	0.00	0.0047673	9.65
17D20034	5.2 %		0.2347512	0.51	0.0000000	0.00	0.0135308	1.04	0.0000128	44.18	50.0586	1.02	0.0438750	0.51	0.0000000	0.00	0.1442199	0.23	0.0090105	9.68	0.0549625	44.19	11.94170	0.21	0.0321626	1.38	7.03855	11.37	76.0195	1.04	0.0000000	0.00	0.0072486	9.65
17D20036	6.0 %	✓	0.4023992	0.41	0.0000000	0.00	0.0200548	0.80	0.0000138	41.65	74.1946	0.78	0.0752084	0.41	0.0000000	0.00	0.2089388	0.18	0.0133550	9.66	0.0591585	41.66	17.30055	0.16	0.0476701	1.21	12.96769	10.06	130.3089	1.00	0.0000000	0.00	0.0105014	9.65
17D20037	6.9 %	✓	0.6224664	0.37	0.0000000	0.00	0.0262109	0.66	0.0000246	23.39	96.9695	0.64	0.1163390	0.37	0.0000000	0.00	0.2725246	0.16	0.0174545	9.65	0.1054154	23.41	22.56559	0.13	0.0623029	1.12	17.16022	11.52	201.5733	0.98	0.0000000	0.00	0.0136973	9.65
17D20039	7.9 %	✓	0.5753380	0.38	0.0000000	0.00	0.0258207	0.68	0.0000221	25.47	95.5259	0.66	0.1075307	0.38	0.0000000	0.00	0.2567320	0.16	0.0171947	9.65	0.0946538	25.49	21.25793	0.13	0.0613754	1.13	17.22015	10.66	186.3117	0.98	0.0000000	0.00	0.0129036	9.65
17D20040	9.0 %	✓	0.6721229	0.37	0.0000000	0.00	0.0345751	0.60	0.0000310	18.64	127.9140	0.58	0.1256198	0.37	0.0000000	0.00	0.3229310	0.14	0.0230245	9.65	0.1332583	18.66	26.73934	0.11	0.0821847	1.09	20.98261	10.18	217.6536	0.98	0.0000000	0.00	0.0162308	9.65
17D20042	10.3 %	✓	0.7505980	0.35	0.0000000	0.00	0.0431471	0.56	0.0000301	18.84	159.6267	0.53	0.1402868	0.35	0.0000000	0.00	0.3803959	0.13	0.0287328	9.64	0.1289757	18.86	31.49755	0.10	0.1025601	1.06	22.69830	10.43	243.0661	0.97	0.0000000	0.00	0.0191190	9.65
17D20043	11.6 %	✓	1.3085373	0.33	0.0000000	0.00	0.0986149	0.50	0.0000785	7.64	364.8351	0.47	0.2445656	0.33	0.0000000	0.00	0.8136341	0.12	0.0656703	9.64	0.3366781	7.69	67.37054	0.08	0.2344066	1.03	54.79615	7.47	423.7436	0.97	0.0000000	0.00	0.0408939	9.65
17D20045	12.5 %	✓	1.0223434	0.35	0.0000000	0.00	0.0804197	0.51	0.0000575	10.47	297.5200	0.48	0.1910760	0.35	0.0000000	0.00	0.6660255	0.12	0.0535536	9.64	0.2467295	10.51	55.14826	0.08	0.1911566	1.04	47.92223	6.71	331.0655	0.97	0.0000000	0.00	0.0334750	9.65
17D20046	13.4 %	✓	0.6931451	0.38	0.0000000	0.00	0.0903822	0.50	0.0000581	9.97	334.3773	0.47	0.1295488	0.38	0.0000000	0.00	0.6161451	0.12	0.0601879	9.64	0.2491929	10.01	51.01806	0.09	0.2148374	1.03	46.74042	4.73	224.4612	0.98	0.0000000	0.00	0.0309680	9.65
17D20048	14.6 %	✓	0.4521597	0.45	0.0000000	0.00	0.0822653	0.51	0.0000394	14.34	304.3481	0.48	0.0845087	0.45	0.0000000	0.00	0.4325100	0.13	0.0547827	9.64	0.1690439	14.37	35.81270	0.10	0.1955436	1.04	29.79950	4.99	146.4229	1.01	0.0000000	0.00	0.0217383	9.65
17D20049	16.0 %	✓	0.3570135	0.51	0.0000000	0.00	0.0978245	0.50	0.0000353	17.57	361.9109	0.47	0.0667258	0.51	0.0000000	0.00	0.3966624	0.14	0.0651440	9.64	0.1514467	17.60	32.84445	0.10	0.2325277	1.03	25.31111	4.77	115.6117	1.04	0.0000000	0.00	0.0199366	9.65
17D20051	17.6 %	✓	0.3572380	0.52	0.0000000	0.00	0.1116699	0.50	0.0000358	16.49	413.1330	0.47	0.0667678	0.52	0.0000000	0.00	0.4210304	0.13	0.0743639	9.64	0.1534254	16.51	34.86217	0.10	0.2654380	1.03	27.21019	4.47	115.6844	1.05	0.0000000	0.00	0.0211613	9.65
17D20052	19.3 %	✓	0.1757294	0.68	0.0000000	0.00	0.0733765	0.51	0.0000287	20.72	271.4633	0.49	0.0328438	0.68	0.0000000	0.00	0.2456877	0.16	0.0488634	9.64	0.1230769	20.74	20.34343	0.13	0.1744152	1.04	15.20304	4.32	56.9065	1.14	0.0000000	0.00	0.0123485	9.65
17D20054	21.0 %	✓	0.0634530	1.29	0.0000000	0.00	0.0279977	0.66	0.0000088	60.98	103.5800	0.63	0.0118594	1.29	0.0000000	0.00	0.0963897	0.30	0.0186444	9.65	0.0376610	60.99	7.98126	0.28	0.0665502	1.12	5.96984	5.78	20.5480	1.58	0.0000000	0.00	0.0048446	9.65
		Σ	9.2502539	0.10	0.0000000	0.00	0.8575213	0.16	0.0004971	4.77	3172.4798	0.15	1.7288725	0.10	0.0000000	0.00	5.6316906	0.04	0.5710464	2.86	2.1332038	4.77	466.31536	0.03	2.0383183	0.31	329.99502	2.37	2995.5097	0.26	0.0000000	0.00	0.2830534	2.74
		Σ							10.108272	0.09	3172.4798	0.15									10.064813	1.03			468.35368	0.03							3325.7878	0.33

Additional Parameters			40Ar/39Ar	1σ	37Ar/39Ar	1σ	36Ar/39Ar	1σ	Time (days)	37Ar (decay)	39Ar (decay)	40Ar (moles)
17D20019	1.8 %		62.108025	0.859931	3.879894	0.295436	0.209124	0.003014	143.333	17.006370	1.00101275	4.677E-12
17D20021	1.9 %		45.061419	0.700707	4.046076	0.328398	0.150378	0.002444	143.347	17.011036	1.00101285	3.016E-12
17D20022	2.0 %		34.690069	0.604778	3.247143	0.349779	0.116231	0.002130	143.354	17.013370	1.00101290	2.120E-12
17D20024	2.2 %		27.833957	0.439135	3.508235	0.321779	0.093947	0.001584	143.369	17.018271	1.00101300	1.904E-12
17D20025	2.4 %		20.435339	0.305043	3.664897	0.285382	0.068209	0.001116	143.376	17.020606	1.00101305	1.558E-12
17D20027	2.7 %		16.102461	0.157976	3.848012	0.193142	0.053618	0.000621	143.390	17.025276	1.00101315	1.739E-12
17D20028	3.0 %		11.630249	0.106551	3.782822	0.166861	0.037841	0.000433	143.397	17.027611	1.00101320	1.589E-12
17D20030	3.4 %		9.227759	0.059862	3.897417	0.109910	0.029318	0.000281	143.410	17.032283	1.00101330	1.728E-12
17D20031	3.9 %		8.071778	0.039639	4.090907	0.081788	0.025685	0.000191	143.417	17.034619	1.00101335	2.164E-12
17D20033	4.5 %		7.386441	0.026595	4.173056	0.063408	0.022924	0.000141	143.431	17.039293	1.00101345	2.792E-12
17D20034	5.2 %		6.937214	0.017599	4.180653	0.043711	0.020736	0.000108	143.438	17.041630	1.00101350	3.987E-12
17D20036	6.0 %	✓	8.259469	0.015044	4.276786	0.034085	0.024352	0.000103	143.452	17.046306	1.00101359	6.878E-12
17D20037	6.9 %	✓	9.667149	0.014006	4.285399	0.027830	0.028668	0.000107	143.459	17.048645	1.00101364	1.050E-11
17D20039	7.9 %	✓	9.547437	0.013844	4.480723	0.029948	0.028199	0.000108	143.473	17.053322	1.00101374	9.770E-12
17D20040	9.0 %	✓	8.897796	0.011125	4.769080	0.027985	0.026349	0.000097	143.480	17.055661	1.00101379	1.146E-11
17D20042	10.3 %	✓	8.410844	0.009238	5.051460	0.027287	0.025119	0.000087	143.494	17.060341	1.00101389	1.276E-11
17D20043	11.6 %	✓	7.079077	0.005837	5.396574	0.025832	0.020815	0.000066	143.501	17.062681	1.00101394	2.297E-11
17D20045	12.5 %	✓	6.849028	0.005948	5.376277	0.026085	0.019928	0.000065	143.515	17.067363	1.00101403	1.819E-11
17D20046	13.4 %	✓	5.294109	0.005060	6.526612	0.031420	0.015295	0.000052	143.522	17.069704	1.00101408	1.302E-11
17D20048	14.6 %	✓	4.894549	0.005731	8.452178	0.041230	0.014843	0.000057	143.535	17.074388	1.00101418	8.460E-12
17D20049	16.0 %	✓	4.261052	0.005625	10.941474	0.052796	0.013752	0.000054	143.542	17.076730	1.00101423	6.765E-12
17D20051	17.6 %	✓	4.068473	0.005199	11.760921	0.056536	0.013350	0.000052	143.556	17.081415	1.00101433	6.860E-12
17D20052	19.3 %	✓	3.515078	0.007389	13.230593	0.066543	0.012142	0.000058	143.563	17.083758	1.00101438	3.462E-12
17D20054	21.0 %	✓	3.295635	0.017253	12.870581	0.089064	0.011365	0.000104	143.577	17.088446	1.00101448	1.273E-12

Procedure Blanks		36Ar ± 1σ (SE) [fA]	37Ar ± 1σ (SE) [fA]	38Ar ± 1σ (SE) [fA]	39Ar ± 1σ (SE) [fA]	40Ar ± 1σ (SE) [fA]
17D20019	1.8 %	0.0082489 ± 0.0005409	0.1586230 ± 0.0183598	0.0317944 ± 0.0169271	0.0338258 ± 0.0155341	2.2592057 ± 0.1158466
17D20021	1.9 %	0.0078652 ± 0.0005409	0.1357518 ± 0.0183598	0.0272675 ± 0.0169271	0.0362247 ± 0.0155341	2.2840851 ± 0.1158466
17D20022	2.0 %	0.0077911 ± 0.0005409	0.1283882 ± 0.0183598	0.0254699 ± 0.0169271	0.0370791 ± 0.0155341	2.3077965 ± 0.1158466
17D20024	2.2 %	0.0078299 ± 0.0005409	0.1198239 ± 0.0183598	0.0226565 ± 0.0169271	0.0379517 ± 0.0155341	2.3749541 ± 0.1158466
17D20025	2.4 %	0.0079199 ± 0.0005409	0.1183596 ± 0.0183598	0.0217530 ± 0.0169271	0.0378820 ± 0.0155341	2.4127193 ± 0.1158466
17D20027	2.7 %	0.0081926 ± 0.0005409	0.1190434 ± 0.0183598	0.0207315 ± 0.0169271	0.0367403 ± 0.0155341	2.4940323 ± 0.1158466
17D20028	3.0 %	0.0083592 ± 0.0005409	0.1206902 ± 0.0183598	0.0205879 ± 0.0169271	0.0356622 ± 0.0155341	2.5356769 ± 0.1158466
17D20030	3.4 %	0.0087183 ± 0.0005409	0.1255039 ± 0.0183598	0.0209642 ± 0.0169271	0.0325226 ± 0.0155341	2.6167273 ± 0.1158466
17D20031	3.9 %	0.0088986 ± 0.0005409	0.1283000 ± 0.0183598	0.0214533 ± 0.0169271	0.0304920 ± 0.0155341	2.6546638 ± 0.1158466
17D20033	4.5 %	0.0092358 ± 0.0005409	0.1339071 ± 0.0183598	0.0229499 ± 0.0169271	0.0256331 ± 0.0155341	2.7222291 ± 0.1158466
17D20034	5.2 %	0.0093846 ± 0.0005409	0.1364778 ± 0.0183598	0.0239215 ± 0.0169271	0.0228727 ± 0.0155341	2.7508225 ± 0.1158466
17D20036	6.0 %	0.0096273 ± 0.0005409	0.1407162 ± 0.0183598	0.0262146 ± 0.0169271	0.0169062 ± 0.0155341	2.7955853 ± 0.1158466
17D20037	6.9 %	0.0097171 ± 0.0005409	0.1422741 ± 0.0183598	0.0274951 ± 0.0169271	0.0138051 ± 0.0155341	2.8111533 ± 0.1158466
17D20039	7.9 %	0.0098279 ± 0.0005409	0.1441558 ± 0.0183598	0.0302147 ± 0.0169271	0.0076758 ± 0.0155341	2.8277013 ± 0.1158466
17D20040	9.0 %	0.0098489 ± 0.0005409	0.1445004 ± 0.0183598	0.0316075 ± 0.0169271	0.0047896 ± 0.0155341	2.8285140 ± 0.1158466
17D20042	10.3 %	0.0098260 ± 0.0005409	0.1442118 ± 0.0183598	0.0343370 ± 0.0169271	0.0002247 ± 0.0155341	2.8153401 ± 0.1158466
17D20043	11.6 %	0.0097860 ± 0.0005409	0.1437297 ± 0.0183598	0.0356223 ± 0.0169271	0.0021739 ± 0.0155341	2.8016200 ± 0.1158466
17D20045	12.5 %	0.0096631 ± 0.0005409	0.1426312 ± 0.0183598	0.0378991 ± 0.0169271	0.0044623 ± 0.0155341	2.7611221 ± 0.1158466
17D20046	13.4 %	0.0095878 ± 0.0005409	0.1422964 ± 0.0183598	0.0388339 ± 0.0169271	0.0045856 ± 0.0155341	2.7350446 ± 0.1158466
17D20048	14.6 %	0.0094338 ± 0.0005409	0.1429228 ± 0.0183598	0.0401487 ± 0.0169271	0.0022041 ± 0.0155341	2.6735256 ± 0.1158466
17D20049	16.0 %	0.0093667 ± 0.0005409	0.1442962 ± 0.0183598	0.0404669 ± 0.0169271	0.0005537 ± 0.0155341	2.6392184 ± 0.1158466
17D20051	17.6 %	0.0092861 ± 0.0005409	0.1503565 ± 0.0183598	0.0402643 ± 0.0169271	0.0098822 ± 0.0155341	2.5668865 ± 0.1158466
17D20052	19.3 %	0.0092882 ± 0.0005409	0.1555860 ± 0.0183598	0.0396766 ± 0.0169271	0.0167429 ± 0.0155341	2.5304299 ± 0.1158466
17D20054	21.0 %	0.0094210 ± 0.0005409	0.1719635 ± 0.0183598	0.0373547 ± 0.0169271	0.0356285 ± 0.0155341	2.4613982 ± 0.1158466

**Intercept Values**		**36Ar ± 1σ (SE) [fA]**	**r2**	**Regression (type,n)**	**37Ar ± 1σ (SE) [fA]**	**r2**	**Regression (type,n)**	**38Ar ± 1σ (SE) [fA]**	**r2**	**Regression (type,n)**	**39Ar ± 1σ (SE) [fA]**	**r2**	**Regression (type,n)**	**40Ar ± 1σ (SE) [fA]**	**r2**	**Regression (type,n)**
17D20019	1.8 %	0.3241121 ± 0.0008306	0.7930	EXP 150 of 150	0.1931829 ± 0.0188263	0.0124	EXP 150 of 150	0.0287819 ± 0.0171112	0.0080	EXP 150 of 150	1.5244588 ± 0.0148157	0.0567	EXP 150 of 150	99.692353 ± 0.023332	0.9979	EXP 150 of 150
17D20021	1.9 %	0.2097413 ± 0.0006713	0.5194	EXP 150 of 150	0.1902375 ± 0.0183157	0.0240	EXP 150 of 150	0.0485777 ± 0.0162736	0.0012	EXP 149 of 150	1.3487821 ± 0.0146587	0.0000	EXP 150 of 150	65.114373 ± 0.022435	0.9985	EXP 150 of 150
17D20022	2.0 %	0.1502375 ± 0.0005847	0.2072	EXP 150 of 150	0.1104147 ± 0.0175075	0.0015	EXP 150 of 150	0.0114941 ± 0.0168427	0.0352	EXP 150 of 150	1.2273088 ± 0.0152484	0.0040	EXP 150 of 150	46.464617 ± 0.020046	0.9990	EXP 150 of 150
17D20024	2.2 %	0.1367073 ± 0.0005597	0.1600	EXP 150 of 150	0.1688886 ± 0.0185047	0.0013	EXP 150 of 150	0.0055560 ± 0.0155481	0.0057	EXP 150 of 150	1.3773337 ± 0.0154508	0.0047	EXP 150 of 150	42.033001 ± 0.020287	0.9988	EXP 150 of 150
17D20025	2.4 %	0.1122560 ± 0.0005033	0.0043	EXP 149 of 150	0.2179016 ± 0.0179578	0.0229	EXP 150 of 150	0.0071797 ± 0.0164270	0.0017	EXP 150 of 150	1.5402444 ± 0.0167305	0.0429	EXP 150 of 150	34.879260 ± 0.019035	0.9991	EXP 150 of 150
17D20027	2.7 %	0.1243451 ± 0.0005044	0.1435	EXP 149 of 150	0.3808279 ± 0.0163039	0.0287	EXP 149 of 150	0.0111654 ± 0.0165883	0.0002	EXP 150 of 150	2.1982088 ± 0.0135939	0.1607	EXP 149 of 150	38.724365 ± 0.018384	0.9989	EXP 150 of 150
17D20028	3.0 %	0.1120509 ± 0.0005131	0.0069	EXP 150 of 150	0.5008184 ± 0.0194663	0.0215	EXP 150 of 150	0.0309695 ± 0.0164235	0.0003	EXP 150 of 150	2.7914081 ± 0.0180374	0.3129	EXP 150 of 150	35.636445 ± 0.018801	0.9990	EXP 150 of 150
17D20030	3.4 %	0.1188393 ± 0.0005897	0.0000	EXP 150 of 150	0.7519943 ± 0.0153348	0.0515	EXP 149 of 150	0.0772480 ± 0.0177181	0.0151	EXP 150 of 150	3.8426746 ± 0.0149530	0.6300	EXP 150 of 150	38.616730 ± 0.019972	0.9986	EXP 150 of 150
17D20031	3.9 %	0.1470163 ± 0.0005376	0.2156	EXP 150 of 150	1.1901364 ± 0.0170781	0.2011	EXP 150 of 150	0.0738456 ± 0.0150978	0.0025	EXP 150 of 150	5.5173438 ± 0.0166401	0.7612	EXP 150 of 150	47.736885 ± 0.020887	0.9984	EXP 150 of 150
17D20033	4.5 %	0.1830490 ± 0.0005958	0.4130	EXP 150 of 150	1.7618643 ± 0.0196574	0.2154	EXP 150 of 150	0.1484435 ± 0.0143721	0.0201	EXP 149 of 150	7.7966747 ± 0.0165218	0.8753	EXP 150 of 150	60.890035 ± 0.021010	0.9978	EXP 150 of 150
17D20034	5.2 %	0.2484435 ± 0.0007640	0.5801	EXP 150 of 150	2.7508976 ± 0.0191140	0.4116	EXP 150 of 150	0.2252793 ± 0.0169821	0.0027	EXP 150 of 150	11.8709594 ± 0.0176258	0.9425	EXP 150 of 150	85.816091 ± 0.029553	0.9944	EXP 150 of 150
17D20036	6.0 %	0.4163803 ± 0.0009818	0.8222	EXP 150 of 150	4.1376516 ± 0.0200291	0.5862	EXP 149 of 150	0.3263893 ± 0.0174447	0.0049	EXP 150 of 150	17.2153595 ± 0.0189358	0.9683	EXP 150 of 150	146.082704 ± 0.061118	0.8698	EXP 150 of 150
17D20037	6.9 %	0.6342888 ± 0.0012242	0.8917	EXP 150 of 150	5.4486221 ± 0.0167549	0.7702	EXP 150 of 150	0.4784177 ± 0.0174098	0.0244	EXP 150 of 150	22.4628407 ± 0.0196448	0.9802	EXP 150 of 150	221.558374 ± 0.074157	0.6360	EXP 150 of 150
17D20039	7.9 %	0.5886462 ± 0.0012134	0.8744	EXP 150 of 150	5.3619956 ± 0.0184281	0.7312	EXP 150 of 150	0.4404810 ± 0.0166596	0.0117	EXP 150 of 150	21.1691280 ± 0.0169980	0.9833	EXP 150 of 150	206.372448 ± 0.074579	0.4456	EXP 150 of 150
17D20040	9.0 %	0.6902894 ± 0.0013508	0.9001	EXP 150 of 150	7.2274995 ± 0.0184469	0.8357	EXP 150 of 150	0.5663463 ± 0.0175739	0.0433	EXP 150 of 150	26.6374468 ± 0.0180055	0.9887	EXP 149 of 150	241.480915 ± 0.062609	0.8791	EXP 150 of 150
17D20042	10.3 %	0.7740746 ± 0.0012696	0.9311	EXP 149 of 150	9.0529462 ± 0.0171565	0.9048	EXP 150 of 150	0.6363377 ± 0.0167388	0.0065	EXP 150 of 150	31.3891034 ± 0.0169767	0.9927	EXP 150 of 150	268.598906 ± 0.034634	0.9901	EXP 150 of 150
17D20043	11.6 %	1.3646711 ± 0.0017243	0.9642	EXP 150 of 150	20.8739737 ± 0.0190307	0.9749	EXP 150 of 150	1.4083127 ± 0.0174765	0.2448	EXP 150 of 150	67.1552221 ± 0.0210785	0.9977	EXP 150 of 150	481.382298 ± 0.040500	0.9988	EXP 150 of 150
17D20045	12.5 %	1.0714615 ± 0.0015820	0.9501	EXP 150 of 150	16.9924319 ± 0.0173077	0.9690	EXP 150 of 150	1.1063208 ± 0.0182066	0.0726	EXP 150 of 150	54.9739559 ± 0.0183473	0.9974	EXP 150 of 150	381.782307 ± 0.032903	0.9985	EXP 150 of 150
17D20046	13.4 %	0.7640257 ± 0.0012438	0.9270	EXP 150 of 150	19.1128427 ± 0.0175425	0.9753	EXP 150 of 150	1.0042398 ± 0.0166033	0.0859	EXP 150 of 150	50.8950124 ± 0.0203698	0.9961	EXP 150 of 150	273.967612 ± 0.034006	0.9923	EXP 150 of 150
17D20048	14.6 %	0.5240174 ± 0.0011578	0.8773	EXP 150 of 150	17.3781762 ± 0.0180376	0.9684	EXP 150 of 150	0.6922697 ± 0.0160240	0.0954	EXP 150 of 150	35.7697431 ± 0.0183319	0.9936	EXP 150 of 150	178.917641 ± 0.028142	0.9044	EXP 150 of 150
17D20049	16.0 %	0.4473198 ± 0.0010180	0.8644	EXP 150 of 150	20.6877943 ± 0.0183076	0.9768	EXP 150 of 150	0.6317775 ± 0.0190809	0.0134	EXP 150 of 150	32.8553104 ± 0.0186897	0.9919	EXP 150 of 150	143.581939 ± 0.033932	0.6692	EXP 150 of 150
17D20051	17.6 %	0.4607862 ± 0.0010295	0.8546	EXP 150 of 150	23.6236284 ± 0.0218254	0.9742	EXP 150 of 150	0.6671838 ± 0.0168882	0.0226	EXP 150 of 150	34.8829037 ± 0.0184849	0.9931	EXP 150 of 150	145.482621 ± 0.026843	0.1102	EXP 150 of 150
17D20052	19.3 %	0.2491556 ± 0.0006872	0.7405	EXP 150 of 150	15.4637893 ± 0.0187524	0.9571	EXP 150 of 150	0.4056713 ± 0.0180377	0.0482	EXP 150 of 150	20.3639468 ± 0.0170409	0.9820	EXP 150 of 150	74.652274 ± 0.022484	0.9929	EXP 150 of 150
17D20054	21.0 %	0.0974783 ± 0.0004903	0.0705	EXP 150 of 150	5.7861587 ± 0.0186366	0.7874	EXP 149 of 150	0.1253280 ± 0.0150187	0.0029	EXP 150 of 150	7.9583855 ± 0.0152424	0.9060	EXP 150 of 150	28.984055 ± 0.017887	0.9981	EXP 150 of 150

**Project Info**		Analyst	Irradiation	X-pos	Y-pos	Z/H-pos	Project	Experiment	Nmb
17D20019	1.8 %	Dan Miggins	17-OSU-01	0.00	0.00	62.19	Arctic\O-Connor (16-22)	17D20015	01
17D20021	1.9 %	Dan Miggins	17-OSU-01	0.00	0.00	62.19	Arctic\O-Connor (16-22)	17D20015	01
17D20022	2.0 %	Dan Miggins	17-OSU-01	0.00	0.00	62.19	Arctic\O-Connor (16-22)	17D20015	01
17D20024	2.2 %	Dan Miggins	17-OSU-01	0.00	0.00	62.19	Arctic\O-Connor (16-22)	17D20015	01
17D20025	2.4 %	Dan Miggins	17-OSU-01	0.00	0.00	62.19	Arctic\O-Connor (16-22)	17D20015	01
17D20027	2.7 %	Dan Miggins	17-OSU-01	0.00	0.00	62.19	Arctic\O-Connor (16-22)	17D20015	01
17D20028	3.0 %	Dan Miggins	17-OSU-01	0.00	0.00	62.19	Arctic\O-Connor (16-22)	17D20015	01
17D20030	3.4 %	Dan Miggins	17-OSU-01	0.00	0.00	62.19	Arctic\O-Connor (16-22)	17D20015	01
17D20031	3.9 %	Dan Miggins	17-OSU-01	0.00	0.00	62.19	Arctic\O-Connor (16-22)	17D20015	01
17D20033	4.5 %	Dan Miggins	17-OSU-01	0.00	0.00	62.19	Arctic\O-Connor (16-22)	17D20015	01
17D20034	5.2 %	Dan Miggins	17-OSU-01	0.00	0.00	62.19	Arctic\O-Connor (16-22)	17D20015	01
17D20036	6.0 %	Dan Miggins	17-OSU-01	0.00	0.00	62.19	Arctic\O-Connor (16-22)	17D20015	01
17D20037	6.9 %	Dan Miggins	17-OSU-01	0.00	0.00	62.19	Arctic\O-Connor (16-22)	17D20015	01
17D20039	7.9 %	Dan Miggins	17-OSU-01	0.00	0.00	62.19	Arctic\O-Connor (16-22)	17D20015	01
17D20040	9.0 %	Dan Miggins	17-OSU-01	0.00	0.00	62.19	Arctic\O-Connor (16-22)	17D20015	01
17D20042	10.3 %	Dan Miggins	17-OSU-01	0.00	0.00	62.19	Arctic\O-Connor (16-22)	17D20015	01
17D20043	11.6 %	Dan Miggins	17-OSU-01	0.00	0.00	62.19	Arctic\O-Connor (16-22)	17D20015	01
17D20045	12.5 %	Dan Miggins	17-OSU-01	0.00	0.00	62.19	Arctic\O-Connor (16-22)	17D20015	01
17D20046	13.4 %	Dan Miggins	17-OSU-01	0.00	0.00	62.19	Arctic\O-Connor (16-22)	17D20015	01
17D20048	14.6 %	Dan Miggins	17-OSU-01	0.00	0.00	62.19	Arctic\O-Connor (16-22)	17D20015	01
17D20049	16.0 %	Dan Miggins	17-OSU-01	0.00	0.00	62.19	Arctic\O-Connor (16-22)	17D20015	01
17D20051	17.6 %	Dan Miggins	17-OSU-01	0.00	0.00	62.19	Arctic\O-Connor (16-22)	17D20015	01
17D20052	19.3 %	Dan Miggins	17-OSU-01	0.00	0.00	62.19	Arctic\O-Connor (16-22)	17D20015	01
17D20054	21.0 %	Dan Miggins	17-OSU-01	0.00	0.00	62.19	Arctic\O-Connor (16-22)	17D20015	01

**Sample Parameters**		Sample	Material	Location	Standard Name	Standard (in Ma)	%1σ	Standard Reference	Standard 40Ar/39Ar	%1σ	J	%1σ	Air 40Ar/36Ar	%1σ	MDF (lin)	%1σ	Volume Ratio	Sensitivity (mol/volt)	Day	Month	Year	Hour	Min	Resist
17D20019	1.8 %	HLY0102-D37-8	Groundmass	Gakkel Ridge	FCT-NM (1C41-17)	28.201	0.082	Kuiper et al (2008)	10.32999	0.082	0.00152153	0.082	302.371	0.143	0.9943117	0.068	1	4.8E-14	11	JUN	2017	23	39	1
17D20021	1.9 %	HLY0102-D37-8	Groundmass	Gakkel Ridge	FCT-NM (1C41-17)	28.201	0.082	Kuiper et al (2008)	10.32999	0.082	0.00152153	0.082	302.371	0.143	0.9943117	0.068	1	4.8E-14	11	JUN	2017	23	59	1
17D20022	2.0 %	HLY0102-D37-8	Groundmass	Gakkel Ridge	FCT-NM (1C41-17)	28.201	0.082	Kuiper et al (2008)	10.32999	0.082	0.00152153	0.082	302.371	0.143	0.9943117	0.068	1	4.8E-14	12	JUN	2017	0	9	1
17D20024	2.2 %	HLY0102-D37-8	Groundmass	Gakkel Ridge	FCT-NM (1C41-17)	28.201	0.082	Kuiper et al (2008)	10.32999	0.082	0.00152153	0.082	302.371	0.143	0.9943117	0.068	1	4.8E-14	12	JUN	2017	0	30	1
17D20025	2.4 %	HLY0102-D37-8	Groundmass	Gakkel Ridge	FCT-NM (1C41-17)	28.201	0.082	Kuiper et al (2008)	10.32999	0.082	0.00152153	0.082	302.371	0.143	0.9943117	0.068	1	4.8E-14	12	JUN	2017	0	40	1
17D20027	2.7 %	HLY0102-D37-8	Groundmass	Gakkel Ridge	FCT-NM (1C41-17)	28.201	0.082	Kuiper et al (2008)	10.32999	0.082	0.00152153	0.082	302.371	0.143	0.9943117	0.068	1	4.8E-14	12	JUN	2017	1	0	1
17D20028	3.0 %	HLY0102-D37-8	Groundmass	Gakkel Ridge	FCT-NM (1C41-17)	28.201	0.082	Kuiper et al (2008)	10.32999	0.082	0.00152153	0.082	302.371	0.143	0.9943117	0.068	1	4.8E-14	12	JUN	2017	1	10	1
17D20030	3.4 %	HLY0102-D37-8	Groundmass	Gakkel Ridge	FCT-NM (1C41-17)	28.201	0.082	Kuiper et al (2008)	10.32999	0.082	0.00152153	0.082	302.371	0.143	0.9943117	0.068	1	4.8E-14	12	JUN	2017	1	30	1
17D20031	3.9 %	HLY0102-D37-8	Groundmass	Gakkel Ridge	FCT-NM (1C41-17)	28.201	0.082	Kuiper et al (2008)	10.32999	0.082	0.00152153	0.082	302.371	0.143	0.9943117	0.068	1	4.8E-14	12	JUN	2017	1	40	1
17D20033	4.5 %	HLY0102-D37-8	Groundmass	Gakkel Ridge	FCT-NM (1C41-17)	28.201	0.082	Kuiper et al (2008)	10.32999	0.082	0.00152153	0.082	302.371	0.143	0.9943117	0.068	1	4.8E-14	12	JUN	2017	2	0	1
17D20034	5.2 %	HLY0102-D37-8	Groundmass	Gakkel Ridge	FCT-NM (1C41-17)	28.201	0.082	Kuiper et al (2008)	10.32999	0.082	0.00152153	0.082	302.371	0.143	0.9943117	0.068	1	4.8E-14	12	JUN	2017	2	10	1
17D20036	6.0 %	HLY0102-D37-8	Groundmass	Gakkel Ridge	FCT-NM (1C41-17)	28.201	0.082	Kuiper et al (2008)	10.32999	0.082	0.00152153	0.082	302.371	0.143	0.9943117	0.068	1	4.8E-14	12	JUN	2017	2	30	1
17D20037	6.9 %	HLY0102-D37-8	Groundmass	Gakkel Ridge	FCT-NM (1C41-17)	28.201	0.082	Kuiper et al (2008)	10.32999	0.082	0.00152153	0.082	302.371	0.143	0.9943117	0.068	1	4.8E-14	12	JUN	2017	2	40	1
17D20039	7.9 %	HLY0102-D37-8	Groundmass	Gakkel Ridge	FCT-NM (1C41-17)	28.201	0.082	Kuiper et al (2008)	10.32999	0.082	0.00152153	0.082	302.371	0.143	0.9943117	0.068	1	4.8E-14	12	JUN	2017	3	0	1
17D20040	9.0 %	HLY0102-D37-8	Groundmass	Gakkel Ridge	FCT-NM (1C41-17)	28.201	0.082	Kuiper et al (2008)	10.32999	0.082	0.00152153	0.082	302.371	0.143	0.9943117	0.068	1	4.8E-14	12	JUN	2017	3	10	1
17D20042	10.3 %	HLY0102-D37-8	Groundmass	Gakkel Ridge	FCT-NM (1C41-17)	28.201	0.082	Kuiper et al (2008)	10.32999	0.082	0.00152153	0.082	302.371	0.143	0.9943117	0.068	1	4.8E-14	12	JUN	2017	3	30	1
17D20043	11.6 %	HLY0102-D37-8	Groundmass	Gakkel Ridge	FCT-NM (1C41-17)	28.201	0.082	Kuiper et al (2008)	10.32999	0.082	0.00152153	0.082	302.371	0.143	0.9943117	0.068	1	4.8E-14	12	JUN	2017	3	40	1
17D20045	12.5 %	HLY0102-D37-8	Groundmass	Gakkel Ridge	FCT-NM (1C41-17)	28.201	0.082	Kuiper et al (2008)	10.32999	0.082	0.00152153	0.082	302.371	0.143	0.9943117	0.068	1	4.8E-14	12	JUN	2017	4	0	1
17D20046	13.4 %	HLY0102-D37-8	Groundmass	Gakkel Ridge	FCT-NM (1C41-17)	28.201	0.082	Kuiper et al (2008)	10.32999	0.082	0.00152153	0.082	302.371	0.143	0.9943117	0.068	1	4.8E-14	12	JUN	2017	4	10	1
17D20048	14.6 %	HLY0102-D37-8	Groundmass	Gakkel Ridge	FCT-NM (1C41-17)	28.201	0.082	Kuiper et al (2008)	10.32999	0.082	0.00152153	0.082	302.371	0.143	0.9943117	0.068	1	4.8E-14	12	JUN	2017	4	30	1
17D20049	16.0 %	HLY0102-D37-8	Groundmass	Gakkel Ridge	FCT-NM (1C41-17)	28.201	0.082	Kuiper et al (2008)	10.32999	0.082	0.00152153	0.082	302.371	0.143	0.9943117	0.068	1	4.8E-14	12	JUN	2017	4	40	1
17D20051	17.6 %	HLY0102-D37-8	Groundmass	Gakkel Ridge	FCT-NM (1C41-17)	28.201	0.082	Kuiper et al (2008)	10.32999	0.082	0.00152153	0.082	302.371	0.143	0.9943117	0.068	1	4.8E-14	12	JUN	2017	5	0	1
17D20052	19.3 %	HLY0102-D37-8	Groundmass	Gakkel Ridge	FCT-NM (1C41-17)	28.201	0.082	Kuiper et al (2008)	10.32999	0.082	0.00152153	0.082	302.371	0.143	0.9943117	0.068	1	4.8E-14	12	JUN	2017	5	10	1
17D20054	21.0 %	HLY0102-D37-8	Groundmass	Gakkel Ridge	FCT-NM (1C41-17)	28.201	0.082	Kuiper et al (2008)	10.32999	0.082	0.00152153	0.082	302.371	0.143	0.9943117	0.068	1	4.8E-14	12	JUN	2017	5	30	1

Irradiation Constants		40/36(a)	%1σ	40/36(c)	%1σ	38/36(a)	%1σ	38/36(c)	%1σ	39/37(ca)	%1σ	38/37(ca)	%1σ	36/37(ca)	%1σ	40/39(k)	%1σ	38/39(k)	%1σ	36/38(cl)	%1σ	K/Ca	%1σ	K/Cl	%1σ	Ca/Cl	%1σ
17D20019	1.8 %	323.83	0.907	0.018	35	0.1869	0	1.493	3	0.000643	0.92	0.00018	9.63	0.00027	0.17	0.000607	9.65	0.012077	0.09	0	0	0.43	0	0	0	0	0
17D20021	1.9 %	323.83	0.907	0.018	35	0.1869	0	1.493	3	0.000643	0.92	0.00018	9.63	0.00027	0.17	0.000607	9.65	0.012077	0.09	0	0	0.43	0	0	0	0	0
17D20022	2.0 %	323.83	0.907	0.018	35	0.1869	0	1.493	3	0.000643	0.92	0.00018	9.63	0.00027	0.17	0.000607	9.65	0.012077	0.09	0	0	0.43	0	0	0	0	0
17D20024	2.2 %	323.83	0.907	0.018	35	0.1869	0	1.493	3	0.000643	0.92	0.00018	9.63	0.00027	0.17	0.000607	9.65	0.012077	0.09	0	0	0.43	0	0	0	0	0
17D20025	2.4 %	323.83	0.907	0.018	35	0.1869	0	1.493	3	0.000643	0.92	0.00018	9.63	0.00027	0.17	0.000607	9.65	0.012077	0.09	0	0	0.43	0	0	0	0	0
17D20027	2.7 %	323.83	0.907	0.018	35	0.1869	0	1.493	3	0.000643	0.92	0.00018	9.63	0.00027	0.17	0.000607	9.65	0.012077	0.09	0	0	0.43	0	0	0	0	0
17D20028	3.0 %	323.83	0.907	0.018	35	0.1869	0	1.493	3	0.000643	0.92	0.00018	9.63	0.00027	0.17	0.000607	9.65	0.012077	0.09	0	0	0.43	0	0	0	0	0
17D20030	3.4 %	323.83	0.907	0.018	35	0.1869	0	1.493	3	0.000643	0.92	0.00018	9.63	0.00027	0.17	0.000607	9.65	0.012077	0.09	0	0	0.43	0	0	0	0	0
17D20031	3.9 %	323.83	0.907	0.018	35	0.1869	0	1.493	3	0.000643	0.92	0.00018	9.63	0.00027	0.17	0.000607	9.65	0.012077	0.09	0	0	0.43	0	0	0	0	0
17D20033	4.5 %	323.83	0.907	0.018	35	0.1869	0	1.493	3	0.000643	0.92	0.00018	9.63	0.00027	0.17	0.000607	9.65	0.012077	0.09	0	0	0.43	0	0	0	0	0
17D20034	5.2 %	323.83	0.907	0.018	35	0.1869	0	1.493	3	0.000643	0.92	0.00018	9.63	0.00027	0.17	0.000607	9.65	0.012077	0.09	0	0	0.43	0	0	0	0	0
17D20036	6.0 %	323.83	0.907	0.018	35	0.1869	0	1.493	3	0.000643	0.92	0.00018	9.63	0.00027	0.17	0.000607	9.65	0.012077	0.09	0	0	0.43	0	0	0	0	0
17D20037	6.9 %	323.83	0.907	0.018	35	0.1869	0	1.493	3	0.000643	0.92	0.00018	9.63	0.00027	0.17	0.000607	9.65	0.012077	0.09	0	0	0.43	0	0	0	0	0
17D20039	7.9 %	323.83	0.907	0.018	35	0.1869	0	1.493	3	0.000643	0.92	0.00018	9.63	0.00027	0.17	0.000607	9.65	0.012077	0.09	0	0	0.43	0	0	0	0	0
17D20040	9.0 %	323.83	0.907	0.018	35	0.1869	0	1.493	3	0.000643	0.92	0.00018	9.63	0.00027	0.17	0.000607	9.65	0.012077	0.09	0	0	0.43	0	0	0	0	0
17D20042	10.3 %	323.83	0.907	0.018	35	0.1869	0	1.493	3	0.000643	0.92	0.00018	9.63	0.00027	0.17	0.000607	9.65	0.012077	0.09	0	0	0.43	0	0	0	0	0
17D20043	11.6 %	323.83	0.907	0.018	35	0.1869	0	1.493	3	0.000643	0.92	0.00018	9.63	0.00027	0.17	0.000607	9.65	0.012077	0.09	0	0	0.43	0	0	0	0	0
17D20045	12.5 %	323.83	0.907	0.018	35	0.1869	0	1.493	3	0.000643	0.92	0.00018	9.63	0.00027	0.17	0.000607	9.65	0.012077	0.09	0	0	0.43	0	0	0	0	0
17D20046	13.4 %	323.83	0.907	0.018	35	0.1869	0	1.493	3	0.000643	0.92	0.00018	9.63	0.00027	0.17	0.000607	9.65	0.012077	0.09	0	0	0.43	0	0	0	0	0
17D20048	14.6 %	323.83	0.907	0.018	35	0.1869	0	1.493	3	0.000643	0.92	0.00018	9.63	0.00027	0.17	0.000607	9.65	0.012077	0.09	0	0	0.43	0	0	0	0	0
17D20049	16.0 %	323.83	0.907	0.018	35	0.1869	0	1.493	3	0.000643	0.92	0.00018	9.63	0.00027	0.17	0.000607	9.65	0.012077	0.09	0	0	0.43	0	0	0	0	0
17D20051	17.6 %	323.83	0.907	0.018	35	0.1869	0	1.493	3	0.000643	0.92	0.00018	9.63	0.00027	0.17	0.000607	9.65	0.012077	0.09	0	0	0.43	0	0	0	0	0
17D20052	19.3 %	323.83	0.907	0.018	35	0.1869	0	1.493	3	0.000643	0.92	0.00018	9.63	0.00027	0.17	0.000607	9.65	0.012077	0.09	0	0	0.43	0	0	0	0	0
17D20054	21.0 %	323.83	0.907	0.018	35	0.1869	0	1.493	3	0.000643	0.92	0.00018	9.63	0.00027	0.17	0.000607	9.65	0.012077	0.09	0	0	0.43	0	0	0	0	0

17D20015.AGE >>> HLY0102-D37-8 >>> ARCTIC | O-CONNOR (16-22) PROJECT

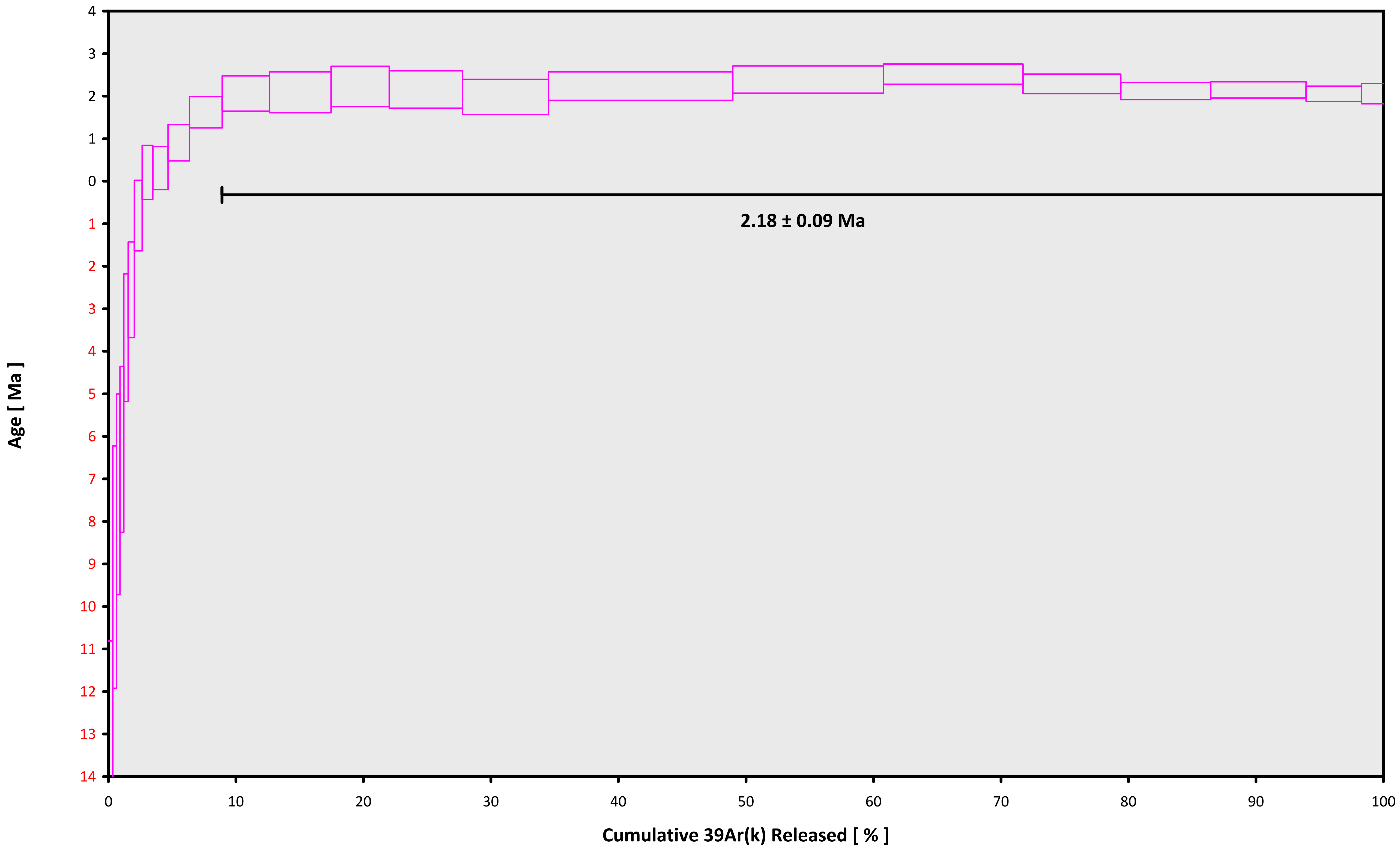

Ar-Ages in Ma

WEIGHTED PLATEAU
2.18 ± 0.09
TOTAL FUSION
1.95 ± 0.09
NORMAL ISOCHRON
2.21 ± 0.26
INVERSE ISOCHRON
2.22 ± 0.26

MSWD (PROBABILITY)
1.32 (20%)

Sample Info

Groundmass
Gakkel Ridge
Dan Miggins

IRR = 17-OSU-01 (1C41-17)
J = 0.00152153 ± 0.00000125

17D20015.AGE >>> HLY0102-D37-8 >>> ARCTIC | O-CONNOR (16-22) PROJECT

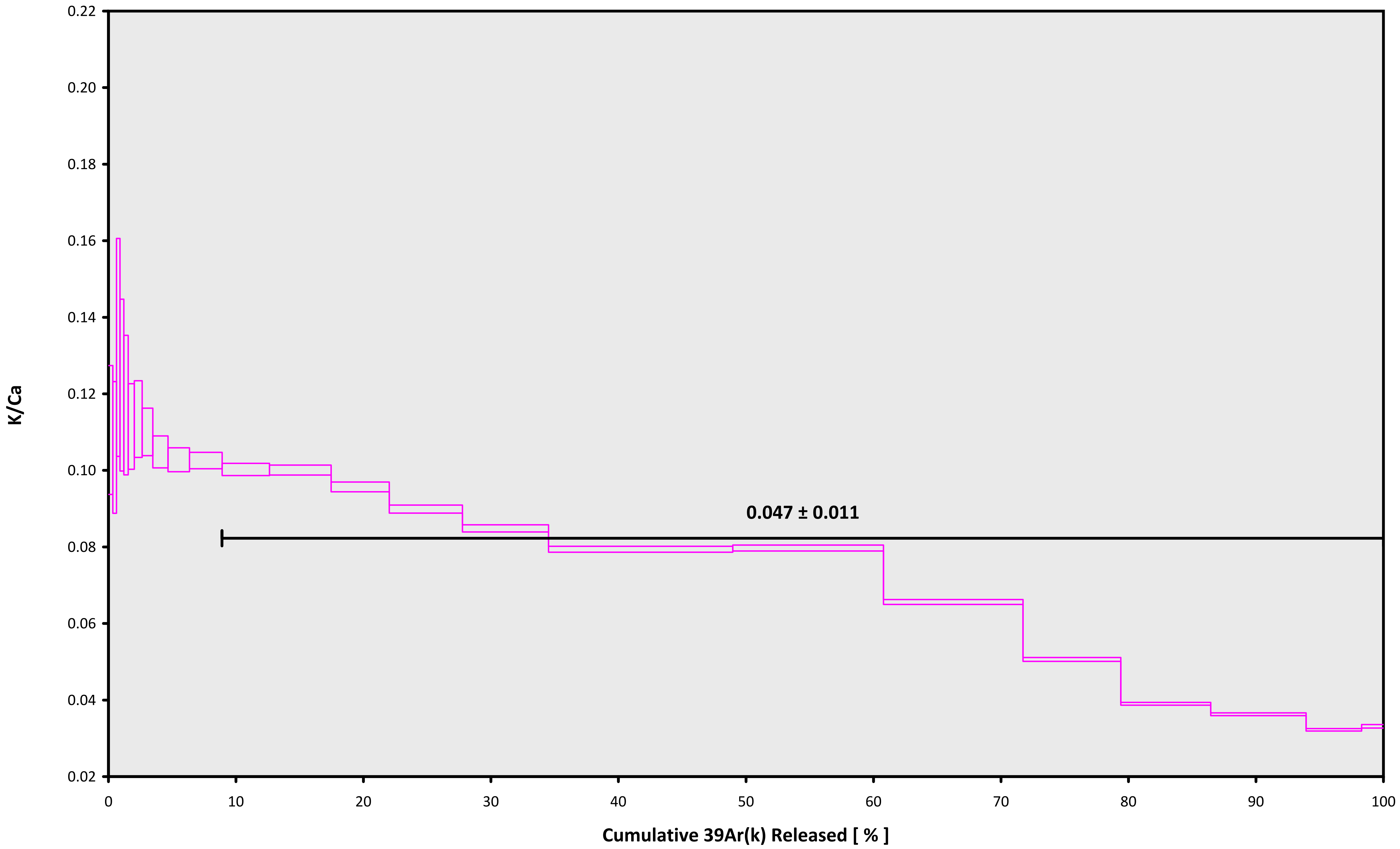

Ar-Ages in Ma

WEIGHTED PLATEAU
2.18 ± 0.09
TOTAL FUSION
1.95 ± 0.09
NORMAL ISOCHRON
2.21 ± 0.26
INVERSE ISOCHRON
2.22 ± 0.26

Sample Info

Groundmass
Gakkel Ridge
Dan Miggins

IRR = 17-OSU-01 (1C41-17)
J = 0.00152153 ± 0.00000125

17D20015.AGE >>> HLY0102-D37-8 >>> ARCTIC | O-CONNOR (16-22) PROJECT

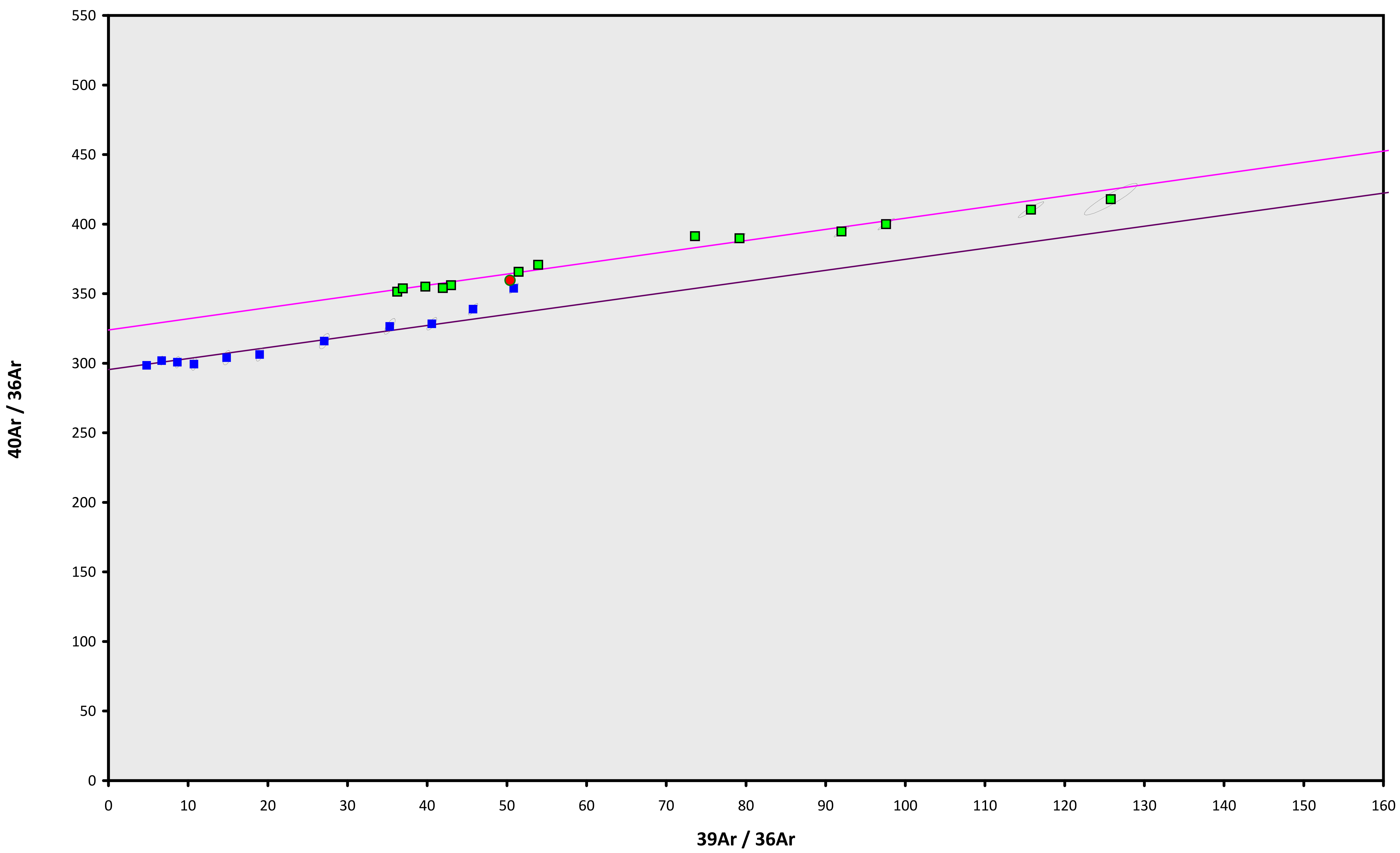

Ar-Ages in Ma

WEIGHTED PLATEAU
2.18 ± 0.09
TOTAL FUSION
1.95 ± 0.09
NORMAL ISOCHRON
2.21 ± 0.26
INVERSE ISOCHRON
2.22 ± 0.26

MSWD (PROBABILITY)
7.70 (0%)

40AR/36AR INTERCEPT
323.9 ± 5.8

Sample Info

Groundmass
Gakkel Ridge
Dan Miggins

IRR = 17-OSU-01 (1C41-17)
J = 0.00152153 ± 0.00000125

17D20015.AGE >>> HLY0102-D37-8 >>> ARCTIC | O-CONNOR (16-22) PROJECT

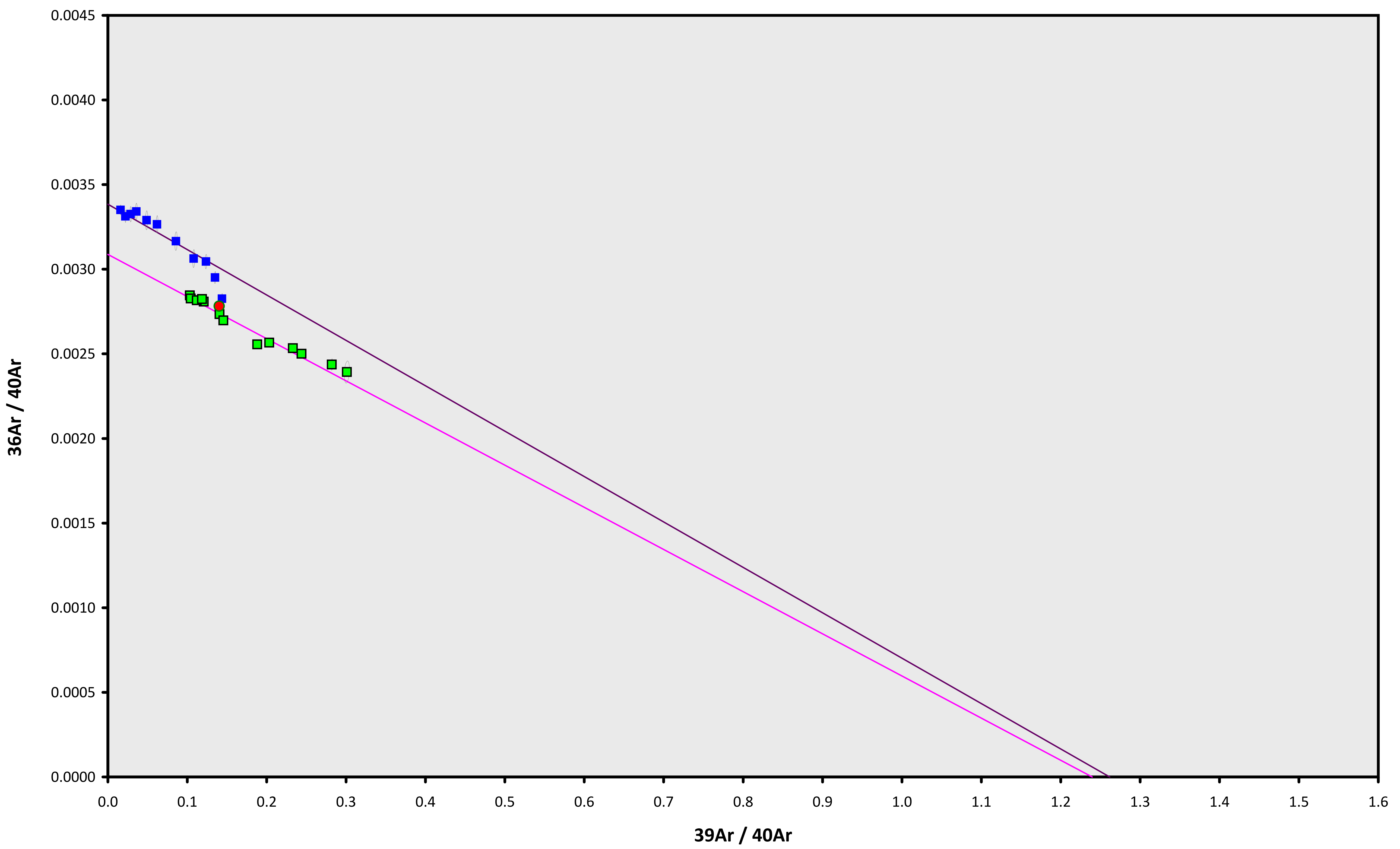

Ar-Ages in Ma

WEIGHTED PLATEAU
2.18 ± 0.09
TOTAL FUSION
1.95 ± 0.09
NORMAL ISOCHRON
2.21 ± 0.26
INVERSE ISOCHRON
2.22 ± 0.26

MSWD (PROBABILITY)
7.83 (0%)

SPREADING FACTOR
16.0%

40AR/36AR INTERCEPT
323.8 ± 5.9

Sample Info

Groundmass
Gakkel Ridge
Dan Miggins

IRR = 17-OSU-01 (1C41-17)
J = 0.00152153 ± 0.00000125

Relative Abundances			36Ar [fA]	%1σ	37Ar [fA]	%1σ	38Ar [fA]	%1σ	39Ar [fA]	%1σ	40Ar [fA]	%1σ	40(r)/39(k) ± 2σ	Age ± 2σ (ka)	40Ar(r) (%)	39Ar(k) (%)	K/Ca ± 2σ
17D19645	1.8 %	✓	10.448246	0.264	166.2026	0.511	3.536550	0.711	95.90772	0.069	3092.6744	0.005	0.09389 ± 0.19875	281.7 ± 596.1	0.29	15.47	0.248 ± 0.003
17D19647	1.9 %	✓	2.325876	0.280	115.7355	0.554	1.032556	2.453	41.01550	0.085	683.6715	0.021	0.08579 ± 0.10809	257.3 ± 324.2	0.51	6.61	0.152 ± 0.002
17D19648	2.0 %	✓	1.005497	0.303	90.9446	0.630	0.527446	4.357	25.76745	0.109	291.5900	0.047	0.03200 ± 0.07961	96.0 ± 238.8	0.28	4.15	0.122 ± 0.002
17D19650	2.2 %	✓	0.740993	0.337	100.9294	0.600	0.452853	5.339	24.70310	0.117	212.5454	0.064	0.03975 ± 0.06689	119.2 ± 200.7	0.46	3.98	0.105 ± 0.001
17D19651	2.4 %	✓	0.635532	0.332	121.8420	0.575	0.452979	5.368	26.91525	0.106	179.0667	0.077	0.01618 ± 0.05237	48.6 ± 157.1	0.24	4.33	0.095 ± 0.001
17D19653	2.7 %	✓	0.636837	0.327	160.5399	0.510	0.506652	4.708	32.83672	0.100	176.8385	0.077	0.02799 ± 0.04245	84.0 ± 127.3	0.52	5.29	0.088 ± 0.001
17D19654	3.0 %	✓	0.195254	0.533	35.2158	1.275	0.122809	19.448	7.63301	0.316	54.6617	0.245	0.05214 ± 0.09180	156.4 ± 275.4	0.73	1.23	0.093 ± 0.002
17D19656	3.4 %	✓	0.741541	0.329	275.0338	0.471	0.737467	3.155	50.22281	0.080	198.6107	0.068	0.01630 ± 0.03221	48.9 ± 96.6	0.41	8.08	0.078 ± 0.001
17D19657	3.9 %	✓	0.465691	0.361	182.7812	0.498	0.455706	5.284	31.95538	0.098	125.1605	0.108	0.05497 ± 0.03498	164.9 ± 104.9	1.40	5.14	0.075 ± 0.001
17D19659	4.5 %	✓	0.706438	0.337	327.8727	0.461	0.784912	3.168	53.41189	0.078	183.4932	0.074	0.00629 ± 0.02950	18.9 ± 88.5	0.18	8.59	0.070 ± 0.001
17D19660	5.2 %	✓	0.332113	0.397	140.0970	0.532	0.336768	7.160	21.89949	0.124	87.5989	0.153	0.01689 ± 0.04033	50.7 ± 121.0	0.42	3.52	0.067 ± 0.001
17D19662	6.0 %	✓	0.749596	0.315	346.9206	0.462	0.754355	3.120	51.65926	0.080	194.8150	0.069	0.00760 ± 0.03067	22.8 ± 92.0	0.20	8.31	0.064 ± 0.001
17D19663	6.9 %	✓	0.172517	0.574	64.9450	0.777	0.098554	25.083	9.30379	0.244	45.9764	0.291	0.00415 ± 0.07192	12.5 ± 215.7	0.08	1.50	0.061 ± 0.001
17D19665	7.9 %	✓	0.249435	0.462	99.8903	0.608	0.230781	11.164	13.69011	0.174	65.6771	0.204	0.01928 ± 0.05649	57.8 ± 169.5	0.40	2.20	0.059 ± 0.001
17D19666	9.0 %	✓	0.332439	0.398	126.8224	0.546	0.263955	9.101	16.59042	0.149	88.3930	0.152	0.00035 ± 0.05344	1.1 ± 160.3	0.01	2.67	0.056 ± 0.001
17D19668	10.3 %	✓	0.678934	0.338	214.2830	0.486	0.465966	5.003	24.78067	0.114	184.1780	0.073	0.00363 ± 0.06152	10.9 ± 184.6	0.05	3.98	0.049 ± 0.000
17D19669	11.6 %	✓	0.972284	0.302	234.9355	0.478	0.488722	4.916	22.54354	0.114	269.4349	0.050	0.00223 ± 0.08771	6.7 ± 263.1	0.02	3.62	0.041 ± 0.000
17D19671	12.5 %	✓	1.061273	0.301	214.1812	0.483	0.472259	5.078	16.59434	0.152	297.8452	0.046	0.02593 ± 0.12983	77.8 ± 389.5	0.14	2.66	0.033 ± 0.000
17D19672	13.4 %	✓	0.875201	0.306	158.6113	0.513	0.349714	6.860	12.26791	0.198	245.9253	0.055	0.06504 ± 0.14750	195.1 ± 442.5	0.32	1.96	0.033 ± 0.000
17D19674	14.6 %	✓	1.239976	0.304	246.1216	0.473	0.477133	4.984	14.08629	0.174	348.6649	0.039	0.06012 ± 0.18057	180.3 ± 541.6	0.24	2.25	0.024 ± 0.000
17D19675	16.0 %	✓	1.017944	0.300	279.9183	0.467	0.398120	6.227	12.48098	0.184	280.2544	0.048	0.07697 ± 0.16606	230.9 ± 498.1	0.34	1.99	0.019 ± 0.000
17D19677	17.6 %	✓	0.363615	0.397	127.6019	0.555	0.166106	15.246	5.09814	0.436	97.6425	0.138	0.01698 ± 0.19053	51.0 ± 571.5	0.09	0.81	0.017 ± 0.000
17D19678	19.3 %	✓	0.535315	0.348	207.5987	0.490	0.209213	11.597	7.22923	0.322	141.9277	0.095	0.01643 ± 0.17404	49.3 ± 522.1	0.08	1.15	0.015 ± 0.000
17D19680	21.0 %		0.216077	0.494	97.7944	0.601	0.078528	31.261	3.22370	0.742	55.5936	0.241	0.19632 ± 0.22655	589.0 ± 679.9	1.12	0.51	0.014 ± 0.000
		Σ	26.698624	0.113	4136.8187	0.111	13.400105	0.887	621.81667	0.025	7602.2394	0.009					

Information on Analysis and Constants Used in Calculations

Project = **O-CONNOR (16-22)**
Sample = **HLY0102-D36-1**
Material = **Groundmass**
Location = **Gakkel Ridge**
Region = **Artic Ocean**
Analyst = **Dan Miggins**
Irradiation = **17-OSU-01 (1C5-17)**
Position = **X: 0 | Y: 0 | Z/H: 8.270811 mm**
FCT-NM Age = **28.201 ± 0.023 Ma**
FCT-NM Reference = **Kuiper et al (2008)**
FCT-NM 40Ar/39Ar Ratio = **9.47435 ± 0.00853**
FCT-NM J-value = **0.00165894 ± 0.00000149**
Air Shot 40Ar/36Ar = **302.5570 ± 0.3268**
Air Shot MDF = **0.99416133 ± 0.00063675 (LIN)**
Experiment Type = **Incremental Heating**
Extraction Method = **Bulk Laser Heating**
Heating = **64 sec**
Isolation = **3.00 min**
Instrument = **ARGUS-VI-D**
Preferred Age = **Plateau Age**
Age Classification = **Crystallization Age**
IGSN = Undefined
Rock Class = **Igneous>Volcanic**
Lithology = **Basaltic Lava**
Lat-Lon = Undefined - Undefined

Age Equations = **Min et al. (2000)**
Negative Intensities = **Allowed**
Collector Calibrations = **36Ar**
Decay 40K = **5.530 ± 0.048 E-10 1/a**
Decay 39Ar = **2.940 ± 0.016 E-07 1/h**
Decay 37Ar = **8.230 ± 0.012 E-04 1/h**
Decay 36Cl = **2.257 ± 0.015 E-06 1/a**
Decay 40K(EC,β⁺) = **0.580 ± 0.009 E-10 1/a**
Decay 40K(β⁻) = **4.950 ± 0.043 E-10 1/a**
Atmospheric 40/36(a) = **296.41 ± 0.47**
Atmospheric 38/36(a) = **0.1869**
Production 39/37(ca) = **0.0006425 ± 0.0000059**
Production 38/37(ca) = **0.0001800 ± 0.0000173**
Production 36/37(ca) = **0.0002703 ± 0.0000005**
Production 40/39(k) = **0.000607 ± 0.000059**
Production 38/39(k) = **0.012077 ± 0.000011**
Production 36/38(cl) = **262.80 ± 1.71**
Scaling Ratio K/Ca = **0.430**
Abundance Ratio 40K/K = **1.1700 ± 0.0100 E-04**
Atomic Weight K = **39.0983 ± 0.0001 g**

Results

Results	40(a)/36(a) ± 2σ	40(r)/39(k) ± 2σ	Age ± 2σ (ka)	MSWD	39Ar(k) (%,n)	K/Ca ± 2σ
**Age Plateau**		0.01633 ± 0.01145 ± 70.14%	**49.0 ± 34.4 ± 70.14%**	0.71 84%	99.49 23	0.029 ± 0.009
		Full External Error	± 34.4	1.60	2σ Confidence Limit	
		Analytical Error	± 34.4	1.0000	Error Magnification	
**Total Fusion Age**		0.03168 ± 0.03358 ± 106.00%	**95.0 ± 100.7 ± 106.00%**		24	0.064 ± 0.000
		Full External Error	± 100.7			
		Analytical Error	± 100.7			
**Normal Isochron**	**296.75 ± 0.80 ± 0.27%**	0.01001 ± 0.01852 ± 185.01%	**30.0 ± 55.6 ± 185.01%**	0.83 69%	99.49 23	
		Full External Error	± 55.6	1.62	2σ Confidence Limit	
		Analytical Error	± 55.6	1.0000	Error Magnification	
				3	Number of Iterations	
				0.0000000112	Convergence	
**Inverse Isochron** Clustered Points	**296.75 ± 0.80 ± 0.27%**	0.01023 ± 0.00473 ± 46.26%	**30.7 ± 14.2 ± 46.26%**	0.83 68%	99.49 23	
		Full External Error	± 14.2	1.62	2σ Confidence Limit	
		Analytical Error	± 14.2	1.0000	Error Magnification	
				3	Number of Iterations	
				0.0139257771	Convergence	
				0%	Spreading Factor	

Notes

Excess Initial 40Ar/36Ar = 296.41 ± 0.16 (%SD). Plateau Age essentially around zero age

Incremental Heating			36Ar(a) [fA]	37Ar(ca) [fA]	38Ar(cl) [fA]	39Ar(k) [fA]	40Ar(r) [fA]	Age ± 2σ (ka)	40Ar(r) (%)	39Ar(k) (%)	K/Ca ± 2σ
17D19645	1.8 %	✓	10.403229	166.2026	0.4052821	95.80093	8.995171	281.7 ± 596.1	0.29	15.47	0.248 ± 0.003
17D19647	1.9 %	✓	2.294573	115.7355	0.0884217	40.94114	3.512318	257.3 ± 324.2	0.51	6.61	0.152 ± 0.002
17D19648	2.0 %	✓	0.980910	90.9446	0.0172563	25.70901	0.822688	96.0 ± 238.8	0.28	4.15	0.122 ± 0.002
17D19650	2.2 %	✓	0.713711	100.9294	0.0037372	24.63825	0.979274	119.2 ± 200.7	0.46	3.98	0.105 ± 0.001
17D19651	2.4 %	✓	0.602598	121.8420	0.0000000	26.83697	0.434351	48.6 ± 157.1	0.24	4.33	0.095 ± 0.001
17D19653	2.7 %	✓	0.593443	160.5399	0.0000000	32.73357	0.916106	84.0 ± 127.3	0.52	5.29	0.088 ± 0.001
17D19654	3.0 %	✓	0.185735	35.2158	0.0000000	7.61038	0.396784	156.4 ± 275.4	0.73	1.23	0.093 ± 0.002
17D19656	3.4 %	✓	0.667200	275.0338	0.0000000	50.04610	0.815657	48.9 ± 96.6	0.41	8.08	0.078 ± 0.001
17D19657	3.9 %	✓	0.416285	182.7812	0.0000000	31.83794	1.750079	164.9 ± 104.9	1.40	5.14	0.075 ± 0.001
17D19659	4.5 %	✓	0.617814	327.8727	0.0000000	53.20123	0.334700	18.9 ± 88.5	0.18	8.59	0.070 ± 0.001
17D19660	5.2 %	✓	0.294245	140.0970	0.0000000	21.80947	0.368436	50.7 ± 121.0	0.42	3.52	0.067 ± 0.001
17D19662	6.0 %	✓	0.655824	346.9206	0.0000000	51.43636	0.391054	22.8 ± 92.0	0.20	8.31	0.064 ± 0.001
17D19663	6.9 %	✓	0.154962	64.9450	0.0000000	9.26207	0.038455	12.5 ± 215.7	0.08	1.50	0.061 ± 0.001
17D19665	7.9 %	✓	0.222433	99.8903	0.0066672	13.62593	0.262657	57.8 ± 169.5	0.40	2.20	0.059 ± 0.001
17D19666	9.0 %	✓	0.298159	126.8224	0.0000000	16.50894	0.005828	1.1 ± 160.3	0.01	2.67	0.056 ± 0.001
17D19668	10.3 %	✓	0.621010	214.2830	0.0137145	24.64299	0.089518	10.9 ± 184.6	0.05	3.98	0.049 ± 0.000
17D19669	11.6 %	✓	0.908779	234.9355	0.0061477	22.39260	0.050041	6.7 ± 263.1	0.02	3.62	0.041 ± 0.000
17D19671	12.5 %	✓	1.003369	214.1812	0.0474286	16.45673	0.426665	77.8 ± 389.5	0.14	2.66	0.033 ± 0.000
17D19672	13.4 %	✓	0.832324	158.6113	0.0186736	12.16600	0.791219	195.1 ± 442.5	0.32	1.96	0.033 ± 0.000
17D19674	14.6 %	✓	1.173439	246.1216	0.0453050	13.92816	0.837365	180.3 ± 541.6	0.24	2.25	0.024 ± 0.000
17D19675	16.0 %	✓	0.942277	279.9183	0.0230625	12.30113	0.946804	230.9 ± 498.1	0.34	1.99	0.019 ± 0.000
17D19677	17.6 %	✓	0.329119	127.6019	0.0210451	5.01615	0.085198	51.0 ± 571.5	0.09	0.81	0.017 ± 0.000
17D19678	19.3 %	✓	0.479201	207.5987	0.0000000	7.09585	0.116556	49.3 ± 522.1	0.08	1.15	0.015 ± 0.000
17D19680	21.0 %		0.189644	97.7944	0.0000000	3.16087	0.620556	589.0 ± 679.9	1.12	0.51	0.014 ± 0.000
		Σ	25.580283	4136.8187	0.6967415	619.15877	19.611935				

Information on Analysis

Project = **O-CONNOR (16-22)**
Sample = **HLY0102-D36-1**
Material = **Groundmass**
Location = **Gakkel Ridge**
Region = **Artic Ocean**
Analyst = **Dan Miggins**
Irradiation = **17-OSU-01 (1C5-17)**
J = **0.00165894 ± 0.00000149**
FCT-NM = **28.201 ± 0.023 Ma**

Results	40(r)/39(k) ± 2σ		Age ± 2σ (ka)		MSWD	39Ar(k) (%,n)	K/Ca ± 2σ
**Age Plateau**	0.01633	± 0.01145<br>± 70.14%	**49.0**	**± 34.4**<br>**± 70.14%**	0.71<br>84%	99.49<br>23	0.029 ± 0.009
		Full External Error	± 34.4		1.60	2σ Confidence Limit	
		Analytical Error	± 34.4		1.0000	Error Magnification	
**Total Fusion Age**	0.03168	± 0.03358<br>± 106.00%	**95.0**	**± 100.7**<br>**± 106.00%**		24	0.064 ± 0.000
		Full External Error	± 100.7				
		Analytical Error	± 100.7				

**Normal Isochron**			39(k)/36(a) ± 2σ	40(a+r)/36(a) ± 2σ	r.i.
17D19645	1.8 %	✓	9.21 ± 0.05	297.27 ± 1.58	0.9673
17D19647	1.9 %	✓	17.84 ± 0.11	297.94 ± 1.69	0.9549
17D19648	2.0 %	✓	26.21 ± 0.17	297.25 ± 1.87	0.9332
17D19650	2.2 %	✓	34.52 ± 0.25	297.78 ± 2.12	0.9330
17D19651	2.4 %	✓	44.54 ± 0.33	297.13 ± 2.14	0.9348
17D19653	2.7 %	✓	55.16 ± 0.41	297.95 ± 2.16	0.9401
17D19654	3.0 %	✓	40.97 ± 0.53	294.27 ± 3.62	0.7992
17D19656	3.4 %	✓	75.01 ± 0.57	297.63 ± 2.24	0.9609
17D19657	3.9 %	✓	76.48 ± 0.64	300.61 ± 2.54	0.9395
17D19659	4.5 %	✓	86.11 ± 0.69	296.95 ± 2.37	0.9637
17D19660	5.2 %	✓	74.12 ± 0.70	297.66 ± 2.85	0.9135
17D19662	6.0 %	✓	78.43 ± 0.59	297.01 ± 2.22	0.9598
17D19663	6.9 %	✓	59.77 ± 0.82	296.66 ± 4.20	0.8518
17D19665	7.9 %	✓	61.26 ± 0.68	295.23 ± 3.32	0.8842
17D19666	9.0 %	✓	55.37 ± 0.52	296.43 ± 2.81	0.8988
17D19668	10.3 %	✓	39.68 ± 0.31	296.55 ± 2.26	0.9381
17D19669	11.6 %	✓	24.64 ± 0.17	296.47 ± 1.95	0.9315
17D19671	12.5 %	✓	16.40 ± 0.12	296.84 ± 1.92	0.8920
17D19672	13.4 %	✓	14.62 ± 0.11	295.46 ± 1.94	0.8382
17D19674	14.6 %	✓	11.87 ± 0.09	297.12 ± 1.93	0.8717
17D19675	16.0 %	✓	13.05 ± 0.10	297.41 ± 1.96	0.8581
17D19677	17.6 %	✓	15.24 ± 0.19	296.67 ± 2.75	0.6748
17D19678	19.3 %	✓	14.81 ± 0.15	296.17 ± 2.40	0.7467
17D19680	21.0 %		16.67 ± 0.32	293.14 ± 3.62	0.5536

**Results**	40(a)/36(a) ± 2σ	40(r)/39(k) ± 2σ	Age ± 2σ (ka)	MSWD
**Normal Isochron**	**296.75** **± 0.80** **± 0.27%**	0.01001 ± 0.01852 ± 185.01%	**30.0** **± 55.6** **± 185.01%**	0.83 69%
		Full External Error	± 55.6	
		Analytical Error	± 55.6	

**Statistics**				
	2σ Confidence Limit	1.62	Convergence	0.000000011249
	Error Magnification	1.0000	Number of Iterations	3
	Number of Data Points	23	Calculated Line	Weighted York-2

Inverse Isochron			39(k)/40(a+r) ± 2σ	36(a)/40(a+r) ± 2σ	r.i.
17D19645	1.8 %	✓	0.0309773 ± 0.0000431	0.00336389 ± 0.00001784	0.0014
17D19647	1.9 %	✓	0.0598864 ± 0.0001054	0.00335637 ± 0.00001909	0.0168
17D19648	2.0 %	✓	0.0881731 ± 0.0002091	0.00336419 ± 0.00002116	0.0597
17D19650	2.2 %	✓	0.1159281 ± 0.0003093	0.00335816 ± 0.00002391	0.0860
17D19651	2.4 %	✓	0.1498850 ± 0.0003946	0.00336552 ± 0.00002425	0.1254
17D19653	2.7 %	✓	0.1851251 ± 0.0004677	0.00335623 ± 0.00002429	0.1307
17D19654	3.0 %	✓	0.1392388 ± 0.0011174	0.00339820 ± 0.00004181	0.2440
17D19656	3.4 %	✓	0.2520194 ± 0.0005311	0.00335985 ± 0.00002524	0.1166
17D19657	3.9 %	✓	0.2544162 ± 0.0007452	0.00332652 ± 0.00002810	0.1900
17D19659	4.5 %	✓	0.2899868 ± 0.0006234	0.00336755 ± 0.00002683	0.1268
17D19660	5.2 %	✓	0.2490074 ± 0.0009838	0.00335951 ± 0.00003216	0.2477
17D19662	6.0 %	✓	0.2640690 ± 0.0005615	0.00336693 ± 0.00002516	0.1211
17D19663	6.9 %	✓	0.2014771 ± 0.0015346	0.00337088 ± 0.00004770	0.3146
17D19665	7.9 %	✓	0.2074948 ± 0.0011135	0.00338720 ± 0.00003809	0.2752
17D19666	9.0 %	✓	0.1867885 ± 0.0007952	0.00337348 ± 0.00003194	0.2280
17D19668	10.3 %	✓	0.1338107 ± 0.0003639	0.00337207 ± 0.00002565	0.1044
17D19669	11.6 %	✓	0.0831137 ± 0.0002086	0.00337308 ± 0.00002216	0.0617
17D19671	12.5 %	✓	0.0552545 ± 0.0001772	0.00336887 ± 0.00002173	0.0408
17D19672	13.4 %	✓	0.0494718 ± 0.0002052	0.00338456 ± 0.00002218	0.0444
17D19674	14.6 %	✓	0.0399481 ± 0.0001440	0.00336560 ± 0.00002189	0.0260
17D19675	16.0 %	✓	0.0438939 ± 0.0001696	0.00336231 ± 0.00002217	0.0369
17D19677	17.6 %	✓	0.0513743 ± 0.0004769	0.00337076 ± 0.00003125	0.0881
17D19678	19.3 %	✓	0.0499977 ± 0.0003417	0.00337648 ± 0.00002735	0.0650
17D19680	21.0 %		0.0568586 ± 0.0009032	0.00341137 ± 0.00004217	0.1180

Results	40(a)/36(a) ± 2σ	40(r)/39(k) ± 2σ	Age ± 2σ (ka)	MSWD
**Inverse Isochron**<br>Clustered Points	**296.75 ± 0.80**<br>**± 0.27%**	0.01023 ± 0.00473<br>± 46.26%	**30.7 ± 14.2**<br>**± 46.26%**<br>Full External Error ± 14.2<br>Analytical Error ± 14.2	0.83<br>68%

Statistics				
	2σ Confidence Limit	1.62	Convergence	0.0139257771
	Error Magnification	1.0000	Number of Iterations	3
	Number of Data Points	23	Calculated Line	Weighted York-2
	Spreading Factor	0.3%		

Degassing Patterns			36Ar(a) [fA]	%1σ	36Ar(c) [fA]	%1σ	36Ar(ca) [fA]	%1σ	36Ar(cl) [fA]	%1σ	37Ar(ca) [fA]	%1σ	38Ar(a) [fA]	%1σ	38Ar(c) [fA]	%1σ	38Ar(k) [fA]	%1σ	38Ar(ca) [fA]	%1σ	38Ar(cl) [fA]	%1σ	39Ar(k) [fA]	%1σ	39Ar(ca) [fA]	%1σ	40Ar(r) [fA]	%1σ	40Ar(a) [fA]	%1σ	40Ar(c) [fA]	%1σ	40Ar(k) [fA]	%1σ
17D19645	1.8 %	✓	10.403229	0.27	0.0000000	0.00	0.0449246	0.54	0.0000927	6.44	166.2026	0.51	1.9443635	0.27	0.0000000	0.00	1.1569878	0.11	0.0299165	9.64	0.4052821	6.51	95.80093	0.07	0.1067852	1.05	8.995171	105.84	3083.6210	0.31	0.0000000	0.00	0.0581512	9.65
17D19647	1.9 %	✓	2.294573	0.28	0.0000000	0.00	0.0312833	0.58	0.0000202	28.79	115.7355	0.55	0.4288557	0.28	0.0000000	0.00	0.4944462	0.12	0.0208324	9.65	0.0884217	28.81	40.94114	0.09	0.0743601	1.07	3.512318	63.00	680.1343	0.32	0.0000000	0.00	0.0248513	9.65
17D19648	2.0 %	✓	0.980910	0.31	0.0000000	0.00	0.0245823	0.65	0.0000039	133.55	90.9446	0.63	0.1833322	0.31	0.0000000	0.00	0.3104878	0.14	0.0163700	9.65	0.0172563	133.56	25.70901	0.11	0.0584319	1.11	0.822688	124.39	290.7517	0.35	0.0000000	0.00	0.0156054	9.65
17D19650	2.2 %	✓	0.713711	0.35	0.0000000	0.00	0.0272812	0.62	0.0000009	648.91	100.9294	0.60	0.1333926	0.35	0.0000000	0.00	0.2975562	0.15	0.0181673	9.65	0.0037372	648.91	24.63825	0.12	0.0648472	1.10	0.979274	84.15	211.5512	0.38	0.0000000	0.00	0.0149554	9.65
17D19651	2.4 %	✓	0.602598	0.35	0.0000000	0.00	0.0329339	0.60	0.0000000	0.00	121.8420	0.57	0.1126256	0.35	0.0000000	0.00	0.3241100	0.14	0.0219316	9.65	0.0000000	0.00	26.83697	0.11	0.0782835	1.08	0.434351	161.78	178.6161	0.39	0.0000000	0.00	0.0162900	9.65
17D19653	2.7 %	✓	0.593443	0.35	0.0000000	0.00	0.0433939	0.54	0.0000000	0.00	160.5399	0.51	0.1109146	0.35	0.0000000	0.00	0.3953233	0.13	0.0288972	9.64	0.0000000	0.00	32.73357	0.10	0.1031469	1.05	0.916106	75.84	175.9025	0.39	0.0000000	0.00	0.0198693	9.65
17D19654	3.0 %	✓	0.185735	0.56	0.0000000	0.00	0.0095188	1.29	0.0000000	0.00	35.2158	1.27	0.0347140	0.56	0.0000000	0.00	0.0919106	0.33	0.0063388	9.71	0.0000000	0.00	7.61038	0.32	0.0226262	1.57	0.396784	88.04	55.0539	0.59	0.0000000	0.00	0.0046195	9.66
17D19656	3.4 %	✓	0.667200	0.37	0.0000000	0.00	0.0743416	0.50	0.0000000	0.00	275.0338	0.47	0.1246996	0.37	0.0000000	0.00	0.6044067	0.12	0.0495061	9.64	0.0000000	0.00	50.04610	0.08	0.1767092	1.03	0.815657	98.80	197.7646	0.40	0.0000000	0.00	0.0303780	9.65
17D19657	3.9 %	✓	0.416285	0.41	0.0000000	0.00	0.0494057	0.53	0.0000000	0.00	182.7812	0.50	0.0778037	0.41	0.0000000	0.00	0.3845068	0.13	0.0329006	9.64	0.0000000	0.00	31.83794	0.10	0.1174369	1.05	1.750079	31.82	123.3911	0.44	0.0000000	0.00	0.0193256	9.65
17D19659	4.5 %	✓	0.617814	0.39	0.0000000	0.00	0.0886240	0.49	0.0000000	0.00	327.8727	0.46	0.1154694	0.39	0.0000000	0.00	0.6425112	0.12	0.0590171	9.64	0.0000000	0.00	53.20123	0.08	0.2106582	1.03	0.334700	234.47	183.1262	0.42	0.0000000	0.00	0.0322931	9.65
17D19660	5.2 %	✓	0.294245	0.45	0.0000000	0.00	0.0378682	0.56	0.0000000	0.00	140.0970	0.53	0.0549944	0.45	0.0000000	0.00	0.2633930	0.15	0.0252175	9.64	0.0000000	0.00	21.80947	0.12	0.0900123	1.06	0.368436	119.37	87.2172	0.48	0.0000000	0.00	0.0132384	9.65
17D19662	6.0 %	✓	0.655824	0.37	0.0000000	0.00	0.0937726	0.49	0.0000000	0.00	346.9206	0.46	0.1225735	0.37	0.0000000	0.00	0.6211969	0.12	0.0624457	9.64	0.0000000	0.00	51.43636	0.08	0.2228965	1.03	0.391054	201.70	194.3927	0.40	0.0000000	0.00	0.0312219	9.65
17D19663	6.9 %	✓	0.154962	0.64	0.0000000	0.00	0.0175546	0.80	0.0000000	0.00	64.9450	0.78	0.0289624	0.64	0.0000000	0.00	0.1118580	0.26	0.0116901	9.66	0.0000000	0.00	9.26207	0.25	0.0417271	1.20	0.038455	866.06	45.9324	0.66	0.0000000	0.00	0.0056221	9.65
17D19665	7.9 %	✓	0.222433	0.52	0.0000000	0.00	0.0270003	0.63	0.0000015	387.36	99.8903	0.61	0.0415728	0.52	0.0000000	0.00	0.1645604	0.20	0.0179802	9.65	0.0066672	387.36	13.62593	0.17	0.0641795	1.10	0.262657	146.52	65.9314	0.55	0.0000000	0.00	0.0082709	9.65
17D19666	9.0 %	✓	0.298159	0.45	0.0000000	0.00	0.0342801	0.57	0.0000000	0.00	126.8224	0.55	0.0557258	0.45	0.0000000	0.00	0.1993784	0.17	0.0228280	9.65	0.0000000	0.00	16.50894	0.15	0.0814834	1.07	0.005828	######	88.3772	0.48	0.0000000	0.00	0.0100209	9.65
17D19668	10.3 %	✓	0.621010	0.37	0.0000000	0.00	0.0579207	0.51	0.0000031	172.19	214.2830	0.49	0.1160667	0.37	0.0000000	0.00	0.2976134	0.15	0.0385709	9.64	0.0137145	172.19	24.64299	0.11	0.1376768	1.04	0.089518	846.82	184.0735	0.41	0.0000000	0.00	0.0149583	9.65
17D19669	11.6 %	✓	0.908779	0.32	0.0000000	0.00	0.0635031	0.51	0.0000014	396.56	234.9355	0.48	0.1698508	0.32	0.0000000	0.00	0.2704354	0.15	0.0422884	9.64	0.0061477	396.56	22.39260	0.11	0.1509461	1.04	0.050041	######	269.3712	0.36	0.0000000	0.00	0.0135923	9.65
17D19671	12.5 %	✓	1.003369	0.32	0.0000000	0.00	0.0578932	0.51	0.0000109	51.19	214.1812	0.48	0.1875296	0.32	0.0000000	0.00	0.1987480	0.18	0.0385526	9.64	0.0474286	51.20	16.45673	0.15	0.1376114	1.04	0.426665	250.38	297.4086	0.36	0.0000000	0.00	0.0099892	9.65
17D19672	13.4 %	✓	0.832324	0.32	0.0000000	0.00	0.0428726	0.54	0.0000043	129.37	158.6113	0.51	0.1555613	0.32	0.0000000	0.00	0.1469288	0.22	0.0285500	9.64	0.0186736	129.37	12.16600	0.20	0.1019077	1.05	0.791219	113.40	246.7091	0.36	0.0000000	0.00	0.0073848	9.65
17D19674	14.6 %	✓	1.173439	0.32	0.0000000	0.00	0.0665267	0.50	0.0000104	53.37	246.1216	0.47	0.2193158	0.32	0.0000000	0.00	0.1682103	0.20	0.0443019	9.64	0.0453050	53.37	13.92816	0.18	0.1581331	1.03	0.837365	150.17	347.8191	0.36	0.0000000	0.00	0.0084544	9.65
17D19675	16.0 %	✓	0.942277	0.33	0.0000000	0.00	0.0756619	0.50	0.0000053	109.58	279.9183	0.47	0.1761115	0.33	0.0000000	0.00	0.1485608	0.21	0.0503853	9.64	0.0230625	109.59	12.30113	0.19	0.1798475	1.03	0.946804	107.87	279.3002	0.36	0.0000000	0.00	0.0074668	9.65
17D19677	17.6 %	✓	0.329119	0.44	0.0000000	0.00	0.0344908	0.58	0.0000048	120.81	127.6019	0.55	0.0615124	0.44	0.0000000	0.00	0.0605801	0.45	0.0229683	9.65	0.0210451	120.82	5.01615	0.44	0.0819842	1.07	0.085198	560.87	97.5542	0.47	0.0000000	0.00	0.0030448	9.66
17D19678	19.3 %	✓	0.479201	0.39	0.0000000	0.00	0.0561139	0.52	0.0000000	0.00	207.5987	0.49	0.0895627	0.39	0.0000000	0.00	0.0856966	0.34	0.0373678	9.64	0.0000000	0.00	7.09585	0.33	0.1333822	1.04	0.116556	529.77	142.0400	0.42	0.0000000	0.00	0.0043072	9.66
17D19680	21.0 %		0.189644	0.57	0.0000000	0.00	0.0264338	0.62	0.0000000	0.00	97.7944	0.60	0.0354444	0.57	0.0000000	0.00	0.0381738	0.76	0.0176030	9.65	0.0000000	0.00	3.16087	0.76	0.0628329	1.10	0.620556	57.69	56.2122	0.59	0.0000000	0.00	0.0019186	9.68
		Σ	25.580283	0.12	0.0000000	0.00	1.1181821	0.12	0.0001595	12.28	4136.8187	0.11	4.7809549	0.12	0.0000000	0.00	7.4775804	0.03	0.7446274	2.17	0.6967415	12.29	619.15877	0.02	2.6579060	0.24	19.611935	53.00	7582.2516	0.14	0.0000000	0.00	0.3758294	2.51
		Σ							26.698624	0.11	4136.8187	0.11									13.699904	0.64			621.81667	0.02							7602.2394	0.19

**Additional Parameters**			**40Ar/39Ar**	**1σ**	**37Ar/39Ar**	**1σ**	**36Ar/39Ar**	**1σ**	**Time (days)**	**37Ar (decay)**	**39Ar (decay)**	**40Ar (moles)**
17D19645	1.8 %	✓	32.246356	0.022388	1.732943	0.008930	0.108941	0.000297	140.895	16.206765	1.00099553	1.484E-10
17D19647	1.9 %	✓	16.668612	0.014634	2.821751	0.015814	0.056707	0.000166	140.909	16.211212	1.00099563	3.282E-11
17D19648	2.0 %	✓	11.316215	0.013388	3.529438	0.022559	0.039022	0.000126	140.916	16.213436	1.00099568	1.400E-11
17D19650	2.2 %	✓	8.603996	0.011453	4.085699	0.024984	0.029996	0.000107	140.930	16.217884	1.00099578	1.020E-11
17D19651	2.4 %	✓	6.652983	0.008738	4.526877	0.026460	0.023612	0.000082	140.937	16.220109	1.00099583	8.595E-12
17D19653	2.7 %	✓	5.385390	0.006787	4.889036	0.025407	0.019394	0.000066	140.951	16.224559	1.00099593	8.488E-12
17D19654	3.0 %	✓	7.161223	0.028678	4.613623	0.060607	0.025580	0.000159	140.958	16.226785	1.00099597	2.624E-12
17D19656	3.4 %	✓	3.954592	0.004156	5.476273	0.026150	0.014765	0.000050	140.972	16.231460	1.00099608	9.533E-12
17D19657	3.9 %	✓	3.916727	0.005724	5.719888	0.029020	0.014573	0.000054	140.979	16.233686	1.00099613	6.008E-12
17D19659	4.5 %	✓	3.435437	0.003681	6.138573	0.028693	0.013226	0.000046	140.993	16.238140	1.00099622	8.808E-12
17D19660	5.2 %	✓	4.000042	0.007886	6.397274	0.034975	0.015165	0.000063	141.000	16.240368	1.00099627	4.205E-12
17D19662	6.0 %	✓	3.771154	0.003995	6.715556	0.031518	0.014510	0.000047	141.014	16.244824	1.00099637	9.351E-12
17D19663	6.9 %	✓	4.941687	0.018781	6.980483	0.056853	0.018543	0.000116	141.021	16.247052	1.00099642	2.207E-12
17D19665	7.9 %	✓	4.797409	0.012843	7.296529	0.046171	0.018220	0.000090	141.035	16.251510	1.00099652	3.152E-12
17D19666	9.0 %	✓	5.327957	0.011309	7.644315	0.043270	0.020038	0.000085	141.042	16.253739	1.00099657	4.243E-12
17D19668	10.3 %	✓	7.432326	0.010056	8.647184	0.043135	0.027398	0.000098	141.056	16.258199	1.00099667	8.841E-12
17D19669	11.6 %	✓	11.951752	0.014888	10.421410	0.051169	0.043129	0.000139	141.063	16.260429	1.00099672	1.293E-11
17D19671	12.5 %	✓	17.948598	0.028515	12.906882	0.065371	0.063954	0.000215	141.076	16.264890	1.00099681	1.430E-11
17D19672	13.4 %	✓	20.046232	0.041208	12.928962	0.071095	0.071341	0.000260	141.083	16.267121	1.00099686	1.180E-11
17D19674	14.6 %	✓	24.752076	0.044043	17.472419	0.087985	0.088027	0.000308	141.097	16.271585	1.00099696	1.674E-11
17D19675	16.0 %	✓	22.454523	0.042670	22.427586	0.112603	0.081560	0.000287	141.104	16.273817	1.00099701	1.345E-11
17D19677	17.6 %	✓	19.152578	0.087523	25.029128	0.176602	0.071323	0.000420	141.118	16.278282	1.00099711	4.687E-12
17D19678	19.3 %	✓	19.632483	0.065833	28.716577	0.168333	0.074049	0.000351	141.125	16.280515	1.00099716	6.813E-12
17D19680	21.0 %		17.245284	0.134500	30.336080	0.289558	0.067028	0.000597	141.139	16.284982	1.00099725	2.668E-12

**Procedure Blanks**		$^{36}Ar \pm 1\sigma$ (SE) [fA]	$^{37}Ar \pm 1\sigma$ (SE) [fA]	$^{38}Ar \pm 1\sigma$ (SE) [fA]	$^{39}Ar \pm 1\sigma$ (SE) [fA]	$^{40}Ar \pm 1\sigma$ (SE) [fA]
17D19645	1.8 %	0.0084288 ± 0.0004901	0.1174022 ± 0.0174239	0.0252646 ± 0.0171691	0.0218893 ± 0.0154536	2.4208078 ± 0.1322727
17D19647	1.9 %	0.0088871 ± 0.0004901	0.1023448 ± 0.0174239	0.0258456 ± 0.0171691	0.0200707 ± 0.0154536	2.5604789 ± 0.1322727
17D19648	2.0 %	0.0090466 ± 0.0004901	0.0973167 ± 0.0174239	0.0252504 ± 0.0171691	0.0178698 ± 0.0154536	2.6084670 ± 0.1322727
17D19650	2.2 %	0.0092494 ± 0.0004901	0.0914269 ± 0.0174239	0.0230119 ± 0.0171691	0.0118368 ± 0.0154536	2.6681201 ± 0.1322727
17D19651	2.4 %	0.0093012 ± 0.0004901	0.0902528 ± 0.0174239	0.0216193 ± 0.0171691	0.0083436 ± 0.0154536	2.6825610 ± 0.1322727
17D19653	2.7 %	0.0093260 ± 0.0004901	0.0907193 ± 0.0174239	0.0188177 ± 0.0171691	0.0011478 ± 0.0154536	2.6871780 ± 0.1322727
17D19654	3.0 %	0.0093065 ± 0.0004901	0.0920905 ± 0.0174239	0.0175823 ± 0.0171691	0.0022991 ± 0.0154536	2.6797841 ± 0.1322727
17D19656	3.4 %	0.0092159 ± 0.0004901	0.0967550 ± 0.0174239	0.0157183 ± 0.0171691	0.0086192 ± 0.0154536	2.6494383 ± 0.1322727
17D19657	3.9 %	0.0091561 ± 0.0004901	0.0995873 ± 0.0174239	0.0152791 ± 0.0171691	0.0110102 ± 0.0154536	2.6301325 ± 0.1322727
17D19659	4.5 %	0.0090194 ± 0.0004901	0.1059153 ± 0.0174239	0.0154111 ± 0.0171691	0.0142665 ± 0.0154536	2.5869737 ± 0.1322727
17D19660	5.2 %	0.0089479 ± 0.0004901	0.1092293 ± 0.0174239	0.0159989 ± 0.0171691	0.0150453 ± 0.0154536	2.5648469 ± 0.1322727
17D19662	6.0 %	0.0088113 ± 0.0004901	0.1157568 ± 0.0174239	0.0181629 ± 0.0171691	0.0147922 ± 0.0154536	2.5233818 ± 0.1322727
17D19663	6.9 %	0.0087506 ± 0.0004901	0.1188316 ± 0.0174239	0.0196785 ± 0.0171691	0.0137571 ± 0.0154536	2.5054237 ± 0.1322727
17D19665	7.9 %	0.0086542 ± 0.0004901	0.1243103 ± 0.0174239	0.0233292 ± 0.0171691	0.0099658 ± 0.0154536	2.4780746 ± 0.1322727
17D19666	9.0 %	0.0086218 ± 0.0004901	0.1266187 ± 0.0174239	0.0253263 ± 0.0171691	0.0072896 ± 0.0154536	2.4697178 ± 0.1322727
17D19668	10.3 %	0.0085961 ± 0.0004901	0.1301882 ± 0.0174239	0.0292235 ± 0.0171691	0.0006808 ± 0.0154536	2.4657929 ± 0.1322727
17D19669	11.6 %	0.0086051 ± 0.0004901	0.1313967 ± 0.0174239	0.0309085 ± 0.0171691	0.0030886 ± 0.0154536	2.4709131 ± 0.1322727
17D19671	12.5 %	0.0086715 ± 0.0004901	0.1325839 ± 0.0174239	0.0331175 ± 0.0171691	0.0110448 ± 0.0154536	2.4966069 ± 0.1322727
17D19672	13.4 %	0.0087302 ± 0.0004901	0.1325530 ± 0.0174239	0.0333493 ± 0.0171691	0.0149850 ± 0.0154536	2.5175228 ± 0.1322727
17D19674	14.6 %	0.0089007 ± 0.0004901	0.1312724 ± 0.0174239	0.0312408 ± 0.0171691	0.0220689 ± 0.0154536	2.5759156 ± 0.1322727
17D19675	16.0 %	0.0090127 ± 0.0004901	0.1300563 ± 0.0174239	0.0285310 ± 0.0171691	0.0248827 ± 0.0154536	2.6133888 ± 0.1322727
17D19677	17.6 %	0.0092900 ± 0.0004901	0.1266100 ± 0.0174239	0.0187808 ± 0.0171691	0.0281252 ± 0.0154536	2.7044470 ± 0.1322727
17D19678	19.3 %	0.0094544 ± 0.0004901	0.1244566 ± 0.0174239	0.0112938 ± 0.0171691	0.0281406 ± 0.0154536	2.7576823 ± 0.1322727
17D19680	21.0 %	0.0098318 ± 0.0004901	0.1195344 ± 0.0174239	0.0101167 ± 0.0171691	0.0238230 ± 0.0154536	2.8782584 ± 0.1322727

**Intercept Values**		**36Ar ± 1σ (SE) [fA]**	**r2**	**Regression (type,n)**	**37Ar ± 1σ (SE) [fA]**	**r2**	**Regression (type,n)**	**38Ar ± 1σ (SE) [fA]**	**r2**	**Regression (type,n)**	**39Ar ± 1σ (SE) [fA]**	**r2**	**Regression (type,n)**	**40Ar ± 1σ (SE) [fA]**	**r2**	**Regression (type,n)**
17D19645	1.8 %	10.0646638 ± 0.0043380	0.9966	EXP 150 of 150	9.9583673 ± 0.0187154	0.9119	EXP 150 of 150	3.4699949 ± 0.0173741	0.5722	EXP 150 of 150	95.2321055 ± 0.0200789	0.9989	EXP 150 of 150	3095.095179 ± 0.086703	0.9999	EXP 150 of 150
17D19647	1.9 %	2.2474981 ± 0.0022305	0.9790	EXP 150 of 150	6.9120145 ± 0.0154794	0.8675	EXP 149 of 150	0.9946549 ± 0.0181687	0.0670	EXP 150 of 150	40.7158598 ± 0.0170939	0.9956	EXP 150 of 150	686.231962 ± 0.046650	0.9994	EXP 150 of 150
17D19648	2.0 %	0.9768178 ± 0.0014140	0.9490	EXP 150 of 150	5.4137881 ± 0.0174624	0.7803	EXP 150 of 150	0.4960378 ± 0.0148515	0.0065	EXP 150 of 150	25.5739382 ± 0.0162672	0.9896	EXP 150 of 150	294.198433 ± 0.037623	0.9904	EXP 150 of 150
17D19650	2.2 %	0.7224412 ± 0.0014387	0.9036	EXP 150 of 150	6.0230666 ± 0.0175799	0.8395	EXP 150 of 150	0.4245543 ± 0.0166106	0.0014	EXP 150 of 150	24.5228787 ± 0.0183267	0.9862	EXP 150 of 150	215.213501 ± 0.030962	0.9739	EXP 150 of 150
17D19651	2.4 %	0.6209884 ± 0.0011624	0.9118	EXP 150 of 150	7.2901511 ± 0.0206342	0.8152	EXP 150 of 150	0.4260715 ± 0.0168036	0.0159	EXP 150 of 150	26.7234403 ± 0.0166490	0.9903	EXP 150 of 150	181.749266 ± 0.039530	0.6630	EXP 150 of 150
17D19653	2.7 %	0.6222695 ± 0.0011131	0.9189	EXP 150 of 150	9.6310854 ± 0.0172676	0.9011	EXP 150 of 150	0.4819187 ± 0.0161419	0.0059	EXP 150 of 150	32.6117347 ± 0.0195071	0.9913	EXP 150 of 150	179.525689 ± 0.034329	0.8919	EXP 150 of 150
17D19654	3.0 %	0.1972350 ± 0.0007235	0.5659	EXP 150 of 150	2.0401798 ± 0.0186048	0.3040	EXP 150 of 150	0.1037932 ± 0.0161985	0.0006	EXP 150 of 150	7.5832775 ± 0.0176999	0.8510	EXP 150 of 150	57.341471 ± 0.022285	0.9962	EXP 150 of 150
17D19656	3.4 %	0.7229351 ± 0.0013425	0.9187	EXP 150 of 150	16.5513681 ± 0.0197790	0.9563	EXP 149 of 150	0.7131386 ± 0.0152703	0.0292	EXP 150 of 150	49.8890722 ± 0.0185489	0.9968	EXP 150 of 150	201.260122 ± 0.026432	0.9845	EXP 150 of 150
17D19657	3.9 %	0.4573746 ± 0.0010046	0.8709	EXP 150 of 150	10.9628574 ± 0.0179914	0.9263	EXP 150 of 150	0.4351067 ± 0.0164705	0.0142	EXP 150 of 150	31.7485547 ± 0.0178215	0.9922	EXP 150 of 150	127.790626 ± 0.030111	0.8938	EXP 150 of 150
17D19659	4.5 %	0.6889520 ± 0.0013662	0.9097	EXP 150 of 150	19.7324483 ± 0.0179075	0.9764	EXP 150 of 150	0.7603371 ± 0.0175505	0.0712	EXP 150 of 150	53.0620528 ± 0.0177917	0.9974	EXP 150 of 150	186.080139 ± 0.027583	0.9708	EXP 150 of 150
17D19660	5.2 %	0.3286006 ± 0.0008217	0.8042	EXP 150 of 150	8.3663588 ± 0.0178977	0.8834	EXP 150 of 150	0.3168373 ± 0.0165202	0.0346	EXP 150 of 150	21.7652453 ± 0.0172906	0.9845	EXP 150 of 150	90.163719 ± 0.021633	0.9895	EXP 150 of 150
17D19662	6.0 %	0.7302835 ± 0.0011848	0.9436	EXP 150 of 150	20.8664862 ± 0.0213297	0.9693	EXP 150 of 150	0.7273852 ± 0.0156665	0.0162	EXP 150 of 150	51.3218877 ± 0.0193621	0.9966	EXP 150 of 150	197.338407 ± 0.027305	0.9854	EXP 149 of 150
17D19663	6.9 %	0.1747948 ± 0.0006927	0.4269	EXP 150 of 150	3.8085924 ± 0.0180057	0.6028	EXP 150 of 150	0.0777253 ± 0.0173817	0.0215	EXP 150 of 150	9.2541265 ± 0.0153649	0.9332	EXP 150 of 150	48.481858 ± 0.020413	0.9966	EXP 150 of 150
17D19665	7.9 %	0.2487307 ± 0.0007748	0.7027	EXP 150 of 150	5.9147076 ± 0.0181511	0.7827	EXP 150 of 150	0.2047570 ± 0.0188010	0.0204	EXP 150 of 150	13.6067477 ± 0.0155944	0.9677	EXP 150 of 150	68.155134 ± 0.020204	0.9929	EXP 150 of 150
17D19666	9.0 %	0.3285877 ± 0.0008285	0.8272	EXP 150 of 150	7.5395698 ± 0.0171389	0.8553	EXP 150 of 150	0.2355468 ± 0.0163958	0.0021	EXP 150 of 150	16.4846092 ± 0.0158085	0.9759	EXP 150 of 150	90.862765 ± 0.021193	0.9803	EXP 150 of 150
17D19668	10.3 %	0.6620567 ± 0.0013248	0.9102	EXP 150 of 150	12.8192869 ± 0.0188126	0.9379	EXP 150 of 150	0.4313018 ± 0.0153513	0.0287	EXP 150 of 150	24.6124154 ± 0.0171968	0.9880	EXP 150 of 150	186.643812 ± 0.028308	0.9875	EXP 150 of 150
17D19669	11.6 %	0.9444093 ± 0.0013367	0.9576	EXP 150 of 150	14.0641949 ± 0.0181359	0.9490	EXP 150 of 150	0.4521079 ± 0.0163908	0.0193	EXP 150 of 150	22.3867691 ± 0.0143754	0.9897	EXP 150 of 150	271.905767 ± 0.031183	0.9972	EXP 150 of 150
17D19671	12.5 %	1.0301262 ± 0.0014522	0.9597	EXP 150 of 150	12.8054150 ± 0.0175997	0.9492	EXP 150 of 150	0.4336275 ± 0.0163257	0.0644	EXP 150 of 150	16.4701692 ± 0.0166871	0.9730	EXP 150 of 150	300.341833 ± 0.034919	0.9977	EXP 150 of 150
17D19672	13.4 %	0.8510940 ± 0.0012612	0.9530	EXP 150 of 150	9.4473292 ± 0.0174792	0.9052	EXP 150 of 150	0.3122814 ± 0.0163478	0.0051	EXP 149 of 150	12.1692842 ± 0.0168237	0.9507	EXP 150 of 150	248.442797 ± 0.027496	0.9970	EXP 150 of 150
17D19674	14.6 %	1.2023537 ± 0.0018134	0.9568	EXP 150 of 150	14.7300211 ± 0.0168742	0.9616	EXP 150 of 150	0.4403216 ± 0.0160370	0.0316	EXP 150 of 150	13.9681862 ± 0.0164659	0.9613	EXP 150 of 150	351.240818 ± 0.031619	0.9989	EXP 150 of 150
17D19675	16.0 %	0.9887639 ± 0.0013668	0.9592	EXP 150 of 150	16.7696291 ± 0.0176183	0.9695	EXP 150 of 150	0.3649409 ± 0.0174726	0.0069	EXP 150 of 150	12.3710069 ± 0.0147365	0.9624	EXP 150 of 150	282.867838 ± 0.030893	0.9977	EXP 150 of 150
17D19677	17.6 %	0.3592622 ± 0.0009265	0.8438	EXP 150 of 150	7.5750701 ± 0.0188580	0.8407	EXP 149 of 150	0.1453857 ± 0.0182098	0.0327	EXP 150 of 150	5.0352541 ± 0.0154104	0.7586	EXP 150 of 150	100.346912 ± 0.024152	0.8893	EXP 150 of 150
17D19678	19.3 %	0.5246847 ± 0.0010857	0.9049	EXP 150 of 150	12.4038792 ± 0.0193913	0.9327	EXP 149 of 150	0.1954766 ± 0.0167370	0.0065	EXP 150 of 150	7.1518019 ± 0.0165359	0.8610	EXP 150 of 150	144.685408 ± 0.024800	0.9524	EXP 150 of 150
17D19680	21.0 %	0.2178021 ± 0.0007217	0.7153	EXP 150 of 150	5.7806220 ± 0.0163288	0.8152	EXP 150 of 150	0.0877275 ± 0.0171425	0.0307	EXP 150 of 150	3.1778975 ± 0.0179216	0.4834	EXP 150 of 150	58.471864 ± 0.020024	0.9921	EXP 150 of 150

Project Info		Analyst	Irradiation	X-pos	Y-pos	Z/H-pos	Project	Experiment	Nmb
17D19645	1.8 %	Dan Miggins	17-OSU-01	0.00	0.00	8.27	Arctic\O-Connor (16-22)	17D19641	01
17D19647	1.9 %	Dan Miggins	17-OSU-01	0.00	0.00	8.27	Arctic\O-Connor (16-22)	17D19641	01
17D19648	2.0 %	Dan Miggins	17-OSU-01	0.00	0.00	8.27	Arctic\O-Connor (16-22)	17D19641	01
17D19650	2.2 %	Dan Miggins	17-OSU-01	0.00	0.00	8.27	Arctic\O-Connor (16-22)	17D19641	01
17D19651	2.4 %	Dan Miggins	17-OSU-01	0.00	0.00	8.27	Arctic\O-Connor (16-22)	17D19641	01
17D19653	2.7 %	Dan Miggins	17-OSU-01	0.00	0.00	8.27	Arctic\O-Connor (16-22)	17D19641	01
17D19654	3.0 %	Dan Miggins	17-OSU-01	0.00	0.00	8.27	Arctic\O-Connor (16-22)	17D19641	01
17D19656	3.4 %	Dan Miggins	17-OSU-01	0.00	0.00	8.27	Arctic\O-Connor (16-22)	17D19641	01
17D19657	3.9 %	Dan Miggins	17-OSU-01	0.00	0.00	8.27	Arctic\O-Connor (16-22)	17D19641	01
17D19659	4.5 %	Dan Miggins	17-OSU-01	0.00	0.00	8.27	Arctic\O-Connor (16-22)	17D19641	01
17D19660	5.2 %	Dan Miggins	17-OSU-01	0.00	0.00	8.27	Arctic\O-Connor (16-22)	17D19641	01
17D19662	6.0 %	Dan Miggins	17-OSU-01	0.00	0.00	8.27	Arctic\O-Connor (16-22)	17D19641	01
17D19663	6.9 %	Dan Miggins	17-OSU-01	0.00	0.00	8.27	Arctic\O-Connor (16-22)	17D19641	01
17D19665	7.9 %	Dan Miggins	17-OSU-01	0.00	0.00	8.27	Arctic\O-Connor (16-22)	17D19641	01
17D19666	9.0 %	Dan Miggins	17-OSU-01	0.00	0.00	8.27	Arctic\O-Connor (16-22)	17D19641	01
17D19668	10.3 %	Dan Miggins	17-OSU-01	0.00	0.00	8.27	Arctic\O-Connor (16-22)	17D19641	01
17D19669	11.6 %	Dan Miggins	17-OSU-01	0.00	0.00	8.27	Arctic\O-Connor (16-22)	17D19641	01
17D19671	12.5 %	Dan Miggins	17-OSU-01	0.00	0.00	8.27	Arctic\O-Connor (16-22)	17D19641	01
17D19672	13.4 %	Dan Miggins	17-OSU-01	0.00	0.00	8.27	Arctic\O-Connor (16-22)	17D19641	01
17D19674	14.6 %	Dan Miggins	17-OSU-01	0.00	0.00	8.27	Arctic\O-Connor (16-22)	17D19641	01
17D19675	16.0 %	Dan Miggins	17-OSU-01	0.00	0.00	8.27	Arctic\O-Connor (16-22)	17D19641	01
17D19677	17.6 %	Dan Miggins	17-OSU-01	0.00	0.00	8.27	Arctic\O-Connor (16-22)	17D19641	01
17D19678	19.3 %	Dan Miggins	17-OSU-01	0.00	0.00	8.27	Arctic\O-Connor (16-22)	17D19641	01
17D19680	21.0 %	Dan Miggins	17-OSU-01	0.00	0.00	8.27	Arctic\O-Connor (16-22)	17D19641	01

**Sample Parameters**		Sample	Material	Location	Standard Name	Standard (in Ma)	%1σ	Standard Reference	Standard 40Ar/39Ar	%1σ	J	%1σ	Air 40Ar/36Ar	%1σ	MDF (lin)	%1σ	Volume Ratio	Sensitivity (mol/volt)	Day	Month	Year	Hour	Min	Resist
17D19645	1.8 %	HLY0102-D36-1	Groundmass	Gakkel Ridge	FCT-NM (1C5-17)	28.201	0.082	Kuiper et al (2008)	9.47435	0.09	0.00165894	0.090	302.557	0.108	0.9941613	0.064	1	4.8E-14	9	JUN	2017	13	8	1
17D19647	1.9 %	HLY0102-D36-1	Groundmass	Gakkel Ridge	FCT-NM (1C5-17)	28.201	0.082	Kuiper et al (2008)	9.47435	0.09	0.00165894	0.090	302.557	0.108	0.9941613	0.064	1	4.8E-14	9	JUN	2017	13	28	1
17D19648	2.0 %	HLY0102-D36-1	Groundmass	Gakkel Ridge	FCT-NM (1C5-17)	28.201	0.082	Kuiper et al (2008)	9.47435	0.09	0.00165894	0.090	302.557	0.108	0.9941613	0.064	1	4.8E-14	9	JUN	2017	13	38	1
17D19650	2.2 %	HLY0102-D36-1	Groundmass	Gakkel Ridge	FCT-NM (1C5-17)	28.201	0.082	Kuiper et al (2008)	9.47435	0.09	0.00165894	0.090	302.557	0.108	0.9941613	0.064	1	4.8E-14	9	JUN	2017	13	58	1
17D19651	2.4 %	HLY0102-D36-1	Groundmass	Gakkel Ridge	FCT-NM (1C5-17)	28.201	0.082	Kuiper et al (2008)	9.47435	0.09	0.00165894	0.090	302.557	0.108	0.9941613	0.064	1	4.8E-14	9	JUN	2017	14	8	1
17D19653	2.7 %	HLY0102-D36-1	Groundmass	Gakkel Ridge	FCT-NM (1C5-17)	28.201	0.082	Kuiper et al (2008)	9.47435	0.09	0.00165894	0.090	302.557	0.108	0.9941613	0.064	1	4.8E-14	9	JUN	2017	14	28	1
17D19654	3.0 %	HLY0102-D36-1	Groundmass	Gakkel Ridge	FCT-NM (1C5-17)	28.201	0.082	Kuiper et al (2008)	9.47435	0.09	0.00165894	0.090	302.557	0.108	0.9941613	0.064	1	4.8E-14	9	JUN	2017	14	38	1
17D19656	3.4 %	HLY0102-D36-1	Groundmass	Gakkel Ridge	FCT-NM (1C5-17)	28.201	0.082	Kuiper et al (2008)	9.47435	0.09	0.00165894	0.090	302.557	0.108	0.9941613	0.064	1	4.8E-14	9	JUN	2017	14	59	1
17D19657	3.9 %	HLY0102-D36-1	Groundmass	Gakkel Ridge	FCT-NM (1C5-17)	28.201	0.082	Kuiper et al (2008)	9.47435	0.09	0.00165894	0.090	302.557	0.108	0.9941613	0.064	1	4.8E-14	9	JUN	2017	15	9	1
17D19659	4.5 %	HLY0102-D36-1	Groundmass	Gakkel Ridge	FCT-NM (1C5-17)	28.201	0.082	Kuiper et al (2008)	9.47435	0.09	0.00165894	0.090	302.557	0.108	0.9941613	0.064	1	4.8E-14	9	JUN	2017	15	29	1
17D19660	5.2 %	HLY0102-D36-1	Groundmass	Gakkel Ridge	FCT-NM (1C5-17)	28.201	0.082	Kuiper et al (2008)	9.47435	0.09	0.00165894	0.090	302.557	0.108	0.9941613	0.064	1	4.8E-14	9	JUN	2017	15	39	1
17D19662	6.0 %	HLY0102-D36-1	Groundmass	Gakkel Ridge	FCT-NM (1C5-17)	28.201	0.082	Kuiper et al (2008)	9.47435	0.09	0.00165894	0.090	302.557	0.108	0.9941613	0.064	1	4.8E-14	9	JUN	2017	15	59	1
17D19663	6.9 %	HLY0102-D36-1	Groundmass	Gakkel Ridge	FCT-NM (1C5-17)	28.201	0.082	Kuiper et al (2008)	9.47435	0.09	0.00165894	0.090	302.557	0.108	0.9941613	0.064	1	4.8E-14	9	JUN	2017	16	9	1
17D19665	7.9 %	HLY0102-D36-1	Groundmass	Gakkel Ridge	FCT-NM (1C5-17)	28.201	0.082	Kuiper et al (2008)	9.47435	0.09	0.00165894	0.090	302.557	0.108	0.9941613	0.064	1	4.8E-14	9	JUN	2017	16	29	1
17D19666	9.0 %	HLY0102-D36-1	Groundmass	Gakkel Ridge	FCT-NM (1C5-17)	28.201	0.082	Kuiper et al (2008)	9.47435	0.09	0.00165894	0.090	302.557	0.108	0.9941613	0.064	1	4.8E-14	9	JUN	2017	16	39	1
17D19668	10.3 %	HLY0102-D36-1	Groundmass	Gakkel Ridge	FCT-NM (1C5-17)	28.201	0.082	Kuiper et al (2008)	9.47435	0.09	0.00165894	0.090	302.557	0.108	0.9941613	0.064	1	4.8E-14	9	JUN	2017	16	59	1
17D19669	11.6 %	HLY0102-D36-1	Groundmass	Gakkel Ridge	FCT-NM (1C5-17)	28.201	0.082	Kuiper et al (2008)	9.47435	0.09	0.00165894	0.090	302.557	0.108	0.9941613	0.064	1	4.8E-14	9	JUN	2017	17	9	1
17D19671	12.5 %	HLY0102-D36-1	Groundmass	Gakkel Ridge	FCT-NM (1C5-17)	28.201	0.082	Kuiper et al (2008)	9.47435	0.09	0.00165894	0.090	302.557	0.108	0.9941613	0.064	1	4.8E-14	9	JUN	2017	17	29	1
17D19672	13.4 %	HLY0102-D36-1	Groundmass	Gakkel Ridge	FCT-NM (1C5-17)	28.201	0.082	Kuiper et al (2008)	9.47435	0.09	0.00165894	0.090	302.557	0.108	0.9941613	0.064	1	4.8E-14	9	JUN	2017	17	39	1
17D19674	14.6 %	HLY0102-D36-1	Groundmass	Gakkel Ridge	FCT-NM (1C5-17)	28.201	0.082	Kuiper et al (2008)	9.47435	0.09	0.00165894	0.090	302.557	0.108	0.9941613	0.064	1	4.8E-14	9	JUN	2017	17	59	1
17D19675	16.0 %	HLY0102-D36-1	Groundmass	Gakkel Ridge	FCT-NM (1C5-17)	28.201	0.082	Kuiper et al (2008)	9.47435	0.09	0.00165894	0.090	302.557	0.108	0.9941613	0.064	1	4.8E-14	9	JUN	2017	18	9	1
17D19677	17.6 %	HLY0102-D36-1	Groundmass	Gakkel Ridge	FCT-NM (1C5-17)	28.201	0.082	Kuiper et al (2008)	9.47435	0.09	0.00165894	0.090	302.557	0.108	0.9941613	0.064	1	4.8E-14	9	JUN	2017	18	29	1
17D19678	19.3 %	HLY0102-D36-1	Groundmass	Gakkel Ridge	FCT-NM (1C5-17)	28.201	0.082	Kuiper et al (2008)	9.47435	0.09	0.00165894	0.090	302.557	0.108	0.9941613	0.064	1	4.8E-14	9	JUN	2017	18	39	1
17D19680	21.0 %	HLY0102-D36-1	Groundmass	Gakkel Ridge	FCT-NM (1C5-17)	28.201	0.082	Kuiper et al (2008)	9.47435	0.09	0.00165894	0.090	302.557	0.108	0.9941613	0.064	1	4.8E-14	9	JUN	2017	18	59	1

Irradiation Constants		40/36(a)	%1σ	40/36(c)	%1σ	38/36(a)	%1σ	38/36(c)	%1σ	39/37(ca)	%1σ	38/37(ca)	%1σ	36/37(ca)	%1σ	40/39(k)	%1σ	38/39(k)	%1σ	36/38(cl)	%1σ	K/Ca	%1σ	K/Cl	%1σ	Ca/Cl	%1σ
17D19645	1.8 %	296.41	0.158	0.018	35	0.1869	0	1.493	3	0.000643	0.92	0.00018	9.63	0.00027	0.17	0.000607	9.65	0.012077	0.09	0	0	0.43	0	0	0	0	0
17D19647	1.9 %	296.41	0.158	0.018	35	0.1869	0	1.493	3	0.000643	0.92	0.00018	9.63	0.00027	0.17	0.000607	9.65	0.012077	0.09	0	0	0.43	0	0	0	0	0
17D19648	2.0 %	296.41	0.158	0.018	35	0.1869	0	1.493	3	0.000643	0.92	0.00018	9.63	0.00027	0.17	0.000607	9.65	0.012077	0.09	0	0	0.43	0	0	0	0	0
17D19650	2.2 %	296.41	0.158	0.018	35	0.1869	0	1.493	3	0.000643	0.92	0.00018	9.63	0.00027	0.17	0.000607	9.65	0.012077	0.09	0	0	0.43	0	0	0	0	0
17D19651	2.4 %	296.41	0.158	0.018	35	0.1869	0	1.493	3	0.000643	0.92	0.00018	9.63	0.00027	0.17	0.000607	9.65	0.012077	0.09	0	0	0.43	0	0	0	0	0
17D19653	2.7 %	296.41	0.158	0.018	35	0.1869	0	1.493	3	0.000643	0.92	0.00018	9.63	0.00027	0.17	0.000607	9.65	0.012077	0.09	0	0	0.43	0	0	0	0	0
17D19654	3.0 %	296.41	0.158	0.018	35	0.1869	0	1.493	3	0.000643	0.92	0.00018	9.63	0.00027	0.17	0.000607	9.65	0.012077	0.09	0	0	0.43	0	0	0	0	0
17D19656	3.4 %	296.41	0.158	0.018	35	0.1869	0	1.493	3	0.000643	0.92	0.00018	9.63	0.00027	0.17	0.000607	9.65	0.012077	0.09	0	0	0.43	0	0	0	0	0
17D19657	3.9 %	296.41	0.158	0.018	35	0.1869	0	1.493	3	0.000643	0.92	0.00018	9.63	0.00027	0.17	0.000607	9.65	0.012077	0.09	0	0	0.43	0	0	0	0	0
17D19659	4.5 %	296.41	0.158	0.018	35	0.1869	0	1.493	3	0.000643	0.92	0.00018	9.63	0.00027	0.17	0.000607	9.65	0.012077	0.09	0	0	0.43	0	0	0	0	0
17D19660	5.2 %	296.41	0.158	0.018	35	0.1869	0	1.493	3	0.000643	0.92	0.00018	9.63	0.00027	0.17	0.000607	9.65	0.012077	0.09	0	0	0.43	0	0	0	0	0
17D19662	6.0 %	296.41	0.158	0.018	35	0.1869	0	1.493	3	0.000643	0.92	0.00018	9.63	0.00027	0.17	0.000607	9.65	0.012077	0.09	0	0	0.43	0	0	0	0	0
17D19663	6.9 %	296.41	0.158	0.018	35	0.1869	0	1.493	3	0.000643	0.92	0.00018	9.63	0.00027	0.17	0.000607	9.65	0.012077	0.09	0	0	0.43	0	0	0	0	0
17D19665	7.9 %	296.41	0.158	0.018	35	0.1869	0	1.493	3	0.000643	0.92	0.00018	9.63	0.00027	0.17	0.000607	9.65	0.012077	0.09	0	0	0.43	0	0	0	0	0
17D19666	9.0 %	296.41	0.158	0.018	35	0.1869	0	1.493	3	0.000643	0.92	0.00018	9.63	0.00027	0.17	0.000607	9.65	0.012077	0.09	0	0	0.43	0	0	0	0	0
17D19668	10.3 %	296.41	0.158	0.018	35	0.1869	0	1.493	3	0.000643	0.92	0.00018	9.63	0.00027	0.17	0.000607	9.65	0.012077	0.09	0	0	0.43	0	0	0	0	0
17D19669	11.6 %	296.41	0.158	0.018	35	0.1869	0	1.493	3	0.000643	0.92	0.00018	9.63	0.00027	0.17	0.000607	9.65	0.012077	0.09	0	0	0.43	0	0	0	0	0
17D19671	12.5 %	296.41	0.158	0.018	35	0.1869	0	1.493	3	0.000643	0.92	0.00018	9.63	0.00027	0.17	0.000607	9.65	0.012077	0.09	0	0	0.43	0	0	0	0	0
17D19672	13.4 %	296.41	0.158	0.018	35	0.1869	0	1.493	3	0.000643	0.92	0.00018	9.63	0.00027	0.17	0.000607	9.65	0.012077	0.09	0	0	0.43	0	0	0	0	0
17D19674	14.6 %	296.41	0.158	0.018	35	0.1869	0	1.493	3	0.000643	0.92	0.00018	9.63	0.00027	0.17	0.000607	9.65	0.012077	0.09	0	0	0.43	0	0	0	0	0
17D19675	16.0 %	296.41	0.158	0.018	35	0.1869	0	1.493	3	0.000643	0.92	0.00018	9.63	0.00027	0.17	0.000607	9.65	0.012077	0.09	0	0	0.43	0	0	0	0	0
17D19677	17.6 %	296.41	0.158	0.018	35	0.1869	0	1.493	3	0.000643	0.92	0.00018	9.63	0.00027	0.17	0.000607	9.65	0.012077	0.09	0	0	0.43	0	0	0	0	0
17D19678	19.3 %	296.41	0.158	0.018	35	0.1869	0	1.493	3	0.000643	0.92	0.00018	9.63	0.00027	0.17	0.000607	9.65	0.012077	0.09	0	0	0.43	0	0	0	0	0
17D19680	21.0 %	296.41	0.158	0.018	35	0.1869	0	1.493	3	0.000643	0.92	0.00018	9.63	0.00027	0.17	0.000607	9.65	0.012077	0.09	0	0	0.43	0	0	0	0	0

17D19641.AGE >>> HLY0102-D36-1 >>> ARCTIC | O-CONNOR (16-22) PROJECT

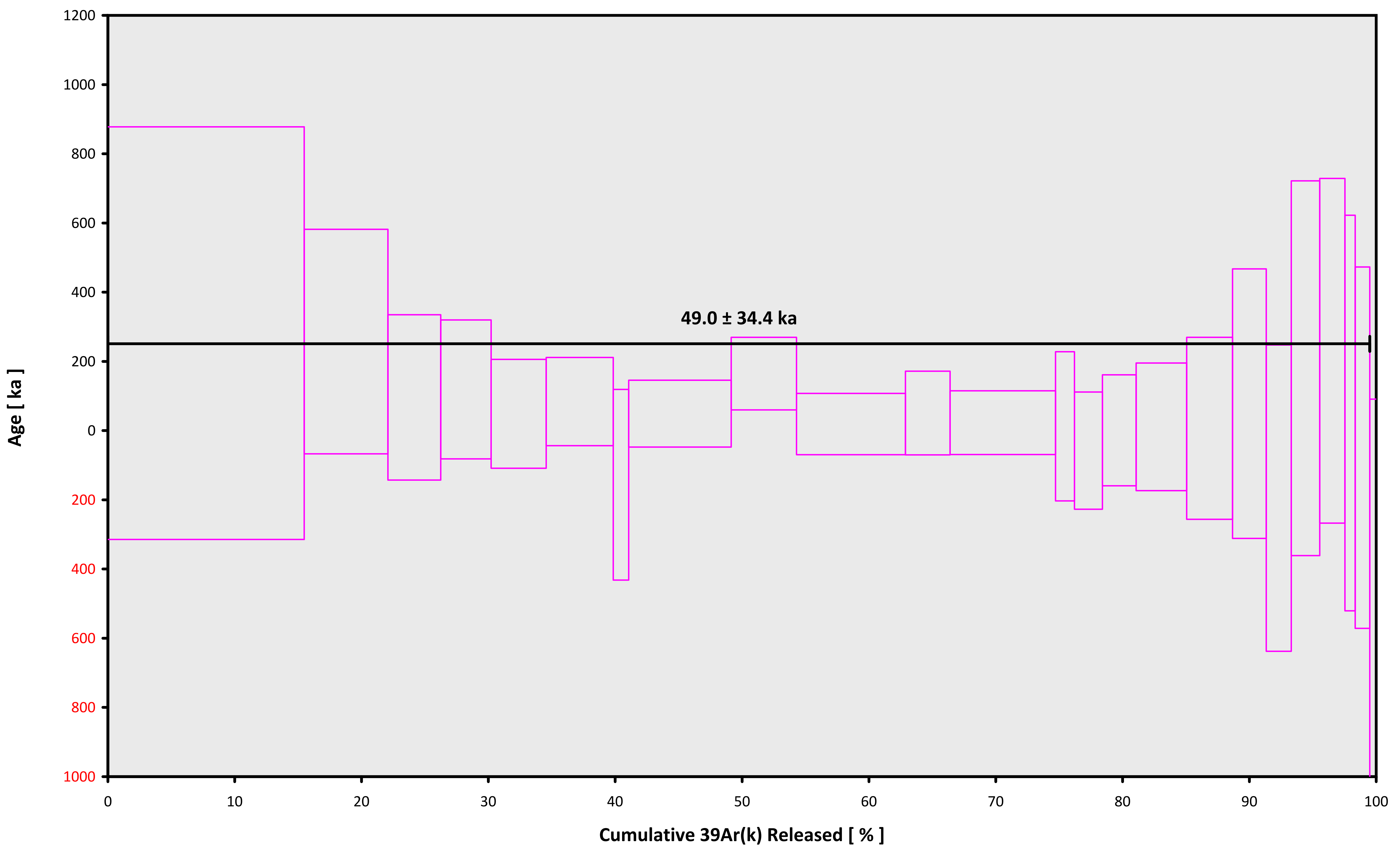

Ar-Ages in ka

WEIGHTED PLATEAU
49.0 ± 34.4
TOTAL FUSION
95.0 ± 100.7
NORMAL ISOCHRON
30.0 ± 55.6
INVERSE ISOCHRON
30.7 ± 14.2

MSWD (PROBABILITY)
0.71 (84%)

Sample Info

Groundmass
Gakkel Ridge
Dan Miggins

IRR = 17-OSU-01 (1C5-17)
J = 0.00165894 ± 0.00000149

17D19641.AGE >>> HLY0102-D36-1 >>> ARCTIC | O-CONNOR (16-22) PROJECT

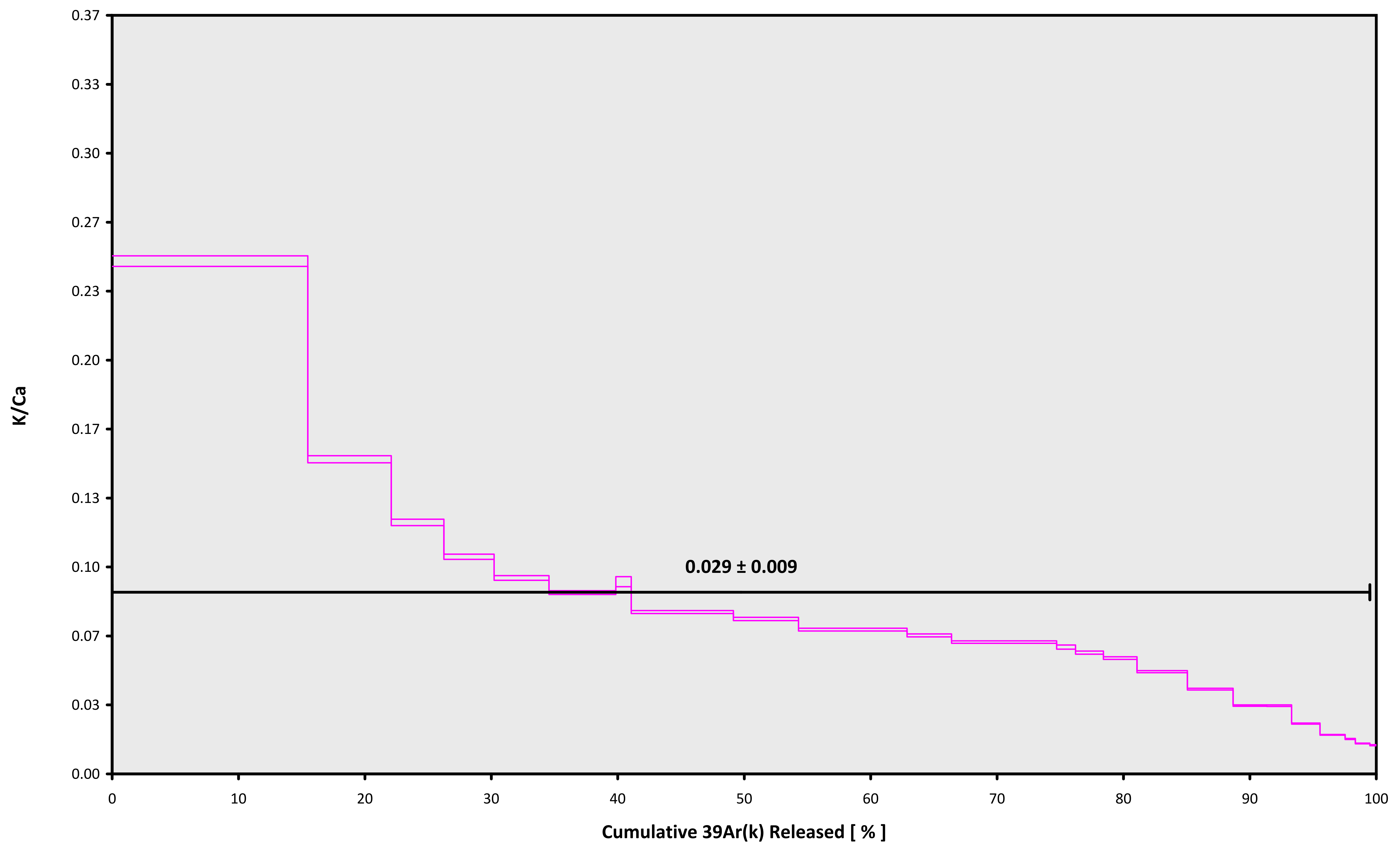

Ar-Ages in ka

WEIGHTED PLATEAU
49.0 ± 34.4
TOTAL FUSION
95.0 ± 100.7
NORMAL ISOCHRON
30.0 ± 55.6
INVERSE ISOCHRON
30.7 ± 14.2

Sample Info

Groundmass
Gakkel Ridge
Dan Miggins

IRR = 17-OSU-01 (1C5-17)
J = 0.00165894 ± 0.00000149

17D19641.AGE >>> HLY0102-D36-1 >>> ARCTIC | O-CONNOR (16-22) PROJECT

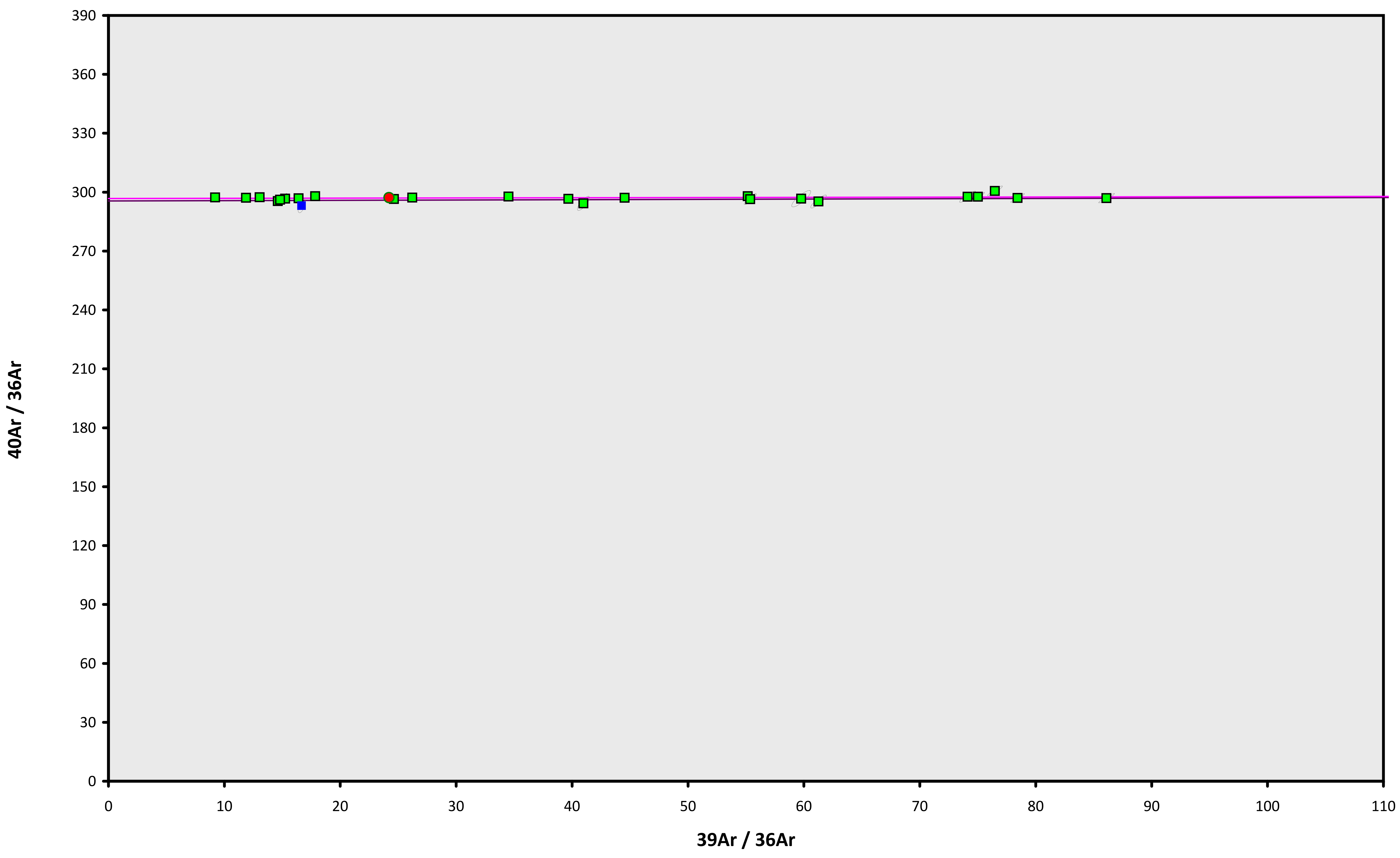

Ar-Ages in ka

WEIGHTED PLATEAU
49.0 ± 34.4
TOTAL FUSION
95.0 ± 100.7
NORMAL ISOCHRON
30.0 ± 55.6
INVERSE ISOCHRON
30.7 ± 14.2

MSWD (PROBABILITY)
0.83 (69%)

40AR/36AR INTERCEPT
296.7 ± 0.8

Sample Info

Groundmass
Gakkel Ridge
Dan Miggins

IRR = 17-OSU-01 (1C5-17)
J = 0.00165894 ± 0.00000149

OSU Argon Geochronology Lab
CEOAS Oregon State University, Corvallis, USA

17D19641.AGE >>> HLY0102-D36-1 >>> ARCTIC | O-CONNOR (16-22) PROJECT

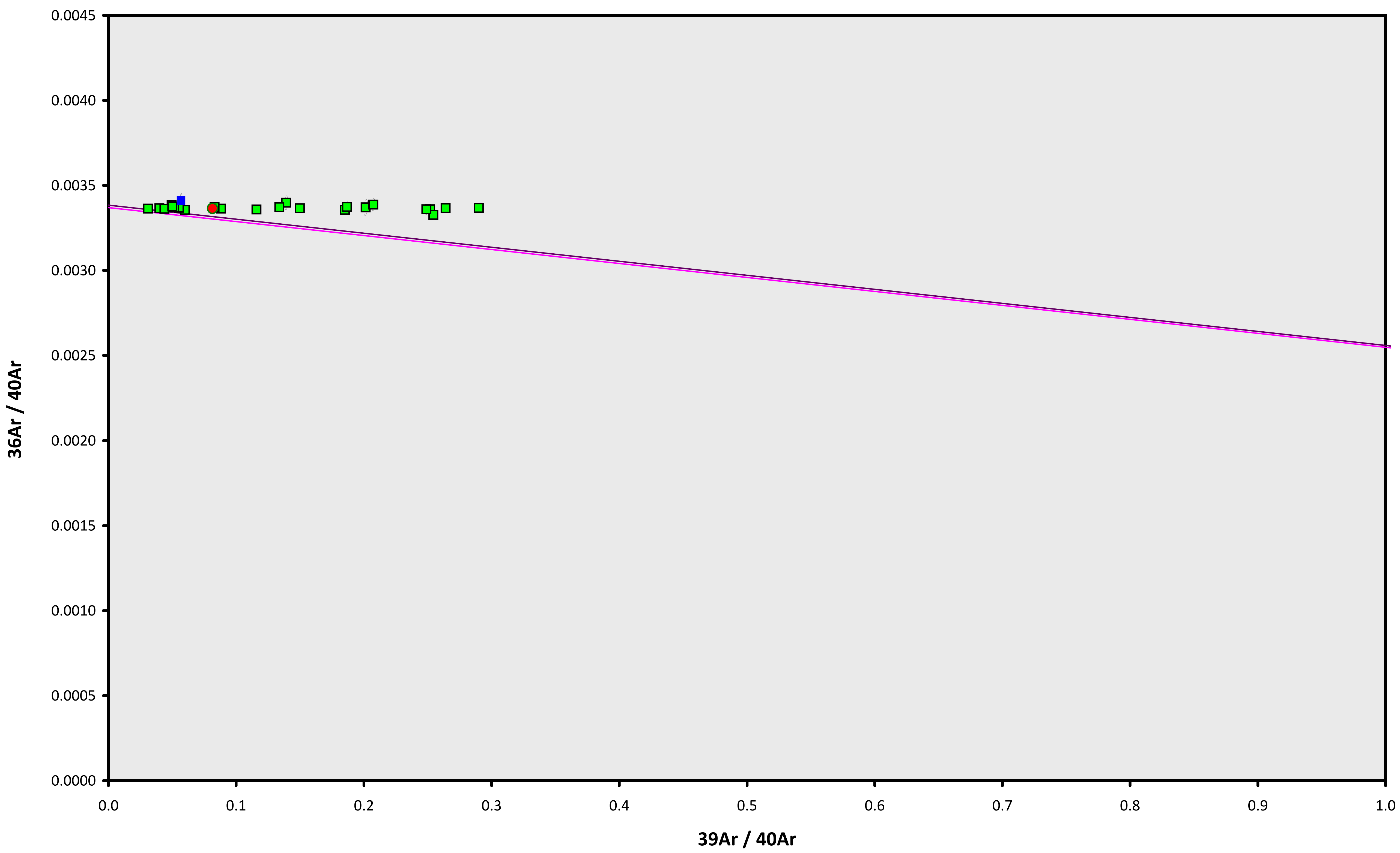

Ar-Ages in ka

WEIGHTED PLATEAU
49.0 ± 34.4
TOTAL FUSION
95.0 ± 100.7
NORMAL ISOCHRON
30.0 ± 55.6
INVERSE ISOCHRON
30.7 ± 14.2

MSWD (PROBABILITY)
0.83 (68%)

SPREADING FACTOR
0.3%

40AR/36AR INTERCEPT
296.8 ± 0.8

Sample Info

Groundmass
Gakkel Ridge
Dan Miggins

IRR = 17-OSU-01 (1C5-17)
J = 0.00165894 ± 0.00000149

Relative Abundances

			36Ar [fA]	%1σ	37Ar [fA]	%1σ	38Ar [fA]	%1σ	39Ar [fA]	%1σ	40Ar [fA]	%1σ	40(r)/39(k) ± 2σ	Age ± 2σ (ka)	40Ar(r) (%)	39Ar(k) (%)	K/Ca ± 2σ
17D30557	1.8 %		1.933454	0.272	75.2381	0.604	0.7515262	3.082	25.92361	0.111	572.6131	0.044	0.28118 ± 0.15994	799.3 ± 454.6	1.27	6.27	0.1479 ± 0.0018
17D30559	1.9 %		0.721672	0.304	65.6847	0.633	0.3825660	6.537	17.96562	0.147	212.7220	0.059	0.26250 ± 0.09210	746.2 ± 261.8	2.21	4.34	0.1173 ± 0.0015
17D30560	2.0 %		0.665211	0.327	113.8299	0.491	0.4562098	5.270	26.37415	0.103	194.1569	0.065	0.25334 ± 0.06029	720.2 ± 171.4	3.43	6.37	0.0994 ± 0.0010
17D30562	2.2 %	✓	0.300118	0.410	54.3267	0.706	0.2387566	9.740	12.36666	0.188	86.1279	0.116	0.14406 ± 0.06941	409.6 ± 197.3	2.06	2.99	0.0976 ± 0.0014
17D30563	2.4 %	✓	0.386995	0.368	112.4846	0.494	0.3550245	7.081	22.70324	0.120	109.0738	0.094	0.16295 ± 0.04442	463.3 ± 126.3	3.38	5.48	0.0865 ± 0.0009
17D30565	2.7 %	✓	0.359915	0.352	138.1745	0.469	0.4184282	5.797	25.82874	0.105	99.7110	0.102	0.17010 ± 0.03516	483.6 ± 99.9	4.39	6.24	0.0801 ± 0.0008
17D30566	3.0 %	✓	0.255006	0.436	108.1421	0.498	0.3019486	7.962	19.28044	0.131	69.6300	0.143	0.15108 ± 0.03966	429.5 ± 112.7	4.17	4.65	0.0764 ± 0.0008
17D30568	3.4 %	✓	0.399647	0.359	224.6949	0.427	0.5234191	4.489	36.43870	0.087	106.5585	0.095	0.17601 ± 0.02770	500.4 ± 78.8	5.99	8.79	0.0695 ± 0.0006
17D30569	3.9 %	✓	0.278867	0.409	170.2959	0.445	0.3747033	6.195	25.90135	0.114	73.1268	0.136	0.16704 ± 0.03049	474.9 ± 86.7	5.89	6.25	0.0651 ± 0.0006
17D30571	4.5 %	✓	0.543609	0.318	383.1541	0.411	0.7736574	3.045	52.80213	0.076	140.0126	0.080	0.18929 ± 0.02369	538.1 ± 67.3	7.11	12.73	0.0590 ± 0.0005
17D30572	5.2 %	✓	0.222817	0.427	137.1792	0.462	0.2703640	8.788	17.69035	0.136	57.2032	0.174	0.13107 ± 0.03745	372.7 ± 106.5	4.03	4.26	0.0552 ± 0.0005
17D30574	6.0 %	✓	0.283748	0.412	168.5820	0.444	0.3402999	7.177	20.71970	0.131	73.8011	0.136	0.16532 ± 0.03898	470.0 ± 110.8	4.62	4.99	0.0526 ± 0.0005
17D30575	6.9 %	✓	0.246046	0.402	128.9345	0.473	0.2384678	10.207	14.95299	0.160	64.9106	0.153	0.16769 ± 0.04661	476.7 ± 132.5	3.84	3.60	0.0496 ± 0.0005
17D30577	7.9 %	✓	0.460343	0.350	235.1097	0.425	0.4075667	5.892	24.21955	0.118	121.5241	0.090	0.17688 ± 0.04725	502.9 ± 134.3	3.50	5.83	0.0440 ± 0.0004
17D30578	9.0 %	✓	0.434932	0.337	175.1726	0.443	0.2794983	8.582	15.69685	0.162	117.1209	0.088	0.16559 ± 0.06747	470.8 ± 191.8	2.20	3.78	0.0383 ± 0.0004
17D30580	10.3 %	✓	0.480738	0.348	161.7521	0.446	0.2925733	8.097	13.23449	0.186	130.8034	0.076	0.12630 ± 0.09039	359.1 ± 257.0	1.27	3.18	0.0349 ± 0.0003
17D30581	11.6 %	✓	0.466430	0.334	140.2053	0.461	0.2990372	8.399	11.44705	0.210	128.4735	0.078	0.16190 ± 0.09888	460.3 ± 281.1	1.43	2.75	0.0348 ± 0.0004
17D30583	12.5 %	✓	0.426241	0.341	122.4003	0.480	0.2198564	10.599	8.16201	0.274	116.4218	0.085	0.02980 ± 0.12922	84.7 ± 367.4	0.21	1.96	0.0284 ± 0.0003
17D30584	13.4 %	✓	0.197474	0.441	58.8556	0.679	0.0848142	29.498	2.97786	0.756	53.9979	0.183	0.11694 ± 0.20778	332.5 ± 590.7	0.64	0.71	0.0215 ± 0.0004
17D30586	14.6 %	✓	0.292325	0.380	98.4058	0.527	0.0972777	25.159	3.82773	0.609	78.9585	0.125	0.11531 ± 0.20868	327.9 ± 593.3	0.55	0.91	0.0164 ± 0.0003
17D30587	16.0 %	✓	0.122895	0.616	43.2673	0.845	0.0485152	49.494	1.73767	1.279	32.8769	0.301	0.00948 ± 0.30217	27.0 ± 859.2	0.05	0.41	0.0170 ± 0.0005
17D30589	17.6 %	✓	0.316383	0.388	107.7847	0.499	0.1191532	20.420	3.14130	0.692	85.5179	0.116	0.20659 ± 0.27902	587.3 ± 793.1	0.74	0.74	0.0123 ± 0.0002
17D30590	19.3 %	✓	0.577964	0.342	197.1238	0.433	0.2226585	10.399	5.01151	0.459	155.7632	0.068	0.14710 ± 0.24480	418.2 ± 695.9	0.46	1.18	0.0107 ± 0.0001
17D30592	21.0 %	✓	0.788283	0.297	258.3394	0.421	0.3026491	7.341	6.57387	0.323	213.3274	0.048	0.15964 ± 0.22056	453.9 ± 627.0	0.48	1.55	0.0107 ± 0.0001
		Σ	11.161112	0.080	3479.1376	0.103	7.7989714	1.506	414.97758	0.031	3094.4330	0.018					

Information on Analysis and Constants Used in Calculations

Project = **O-CONNOR (16-22)**
Sample = **PS59-252-1**
Material = **Groundmass**
Location = **Gakkel Ridge**
Region = **Artic Ocean**
Analyst = **Dan Miggins**
Irradiation = **17-OSU-05 (5A26-17)**
Position = **X: 0 | Y: 0 | Z/H: 37.93017 mm**
FCT-NM Age = **28.201 ± 0.023 Ma**
FCT-NM Reference = **Kuiper et al (2008)**
FCT-NM 40Ar/39Ar Ratio = **9.99585 ± 0.01030**
FCT-NM J-value = **0.00157239 ± 0.00000162**
Air Shot 40Ar/36Ar = **302.7790 ± 0.1665**
Air Shot MDF = **0.99398207 ± 0.00059439 (LIN)**
Experiment Type = **Incremental Heating**
Extraction Method = **Bulk Laser Heating**
Heating = **64 sec**
Isolation = **3.00 min**
Instrument = **ARGUS-VI-D**
Preferred Age = **Plateau Age**
Age Classification = **Crystallization Age**
IGSN = Undefined
Rock Class = **Igneous>Volcanic**
Lithology = **Basaltic Lava**
Lat-Lon = Undefined - Undefined

Age Equations = **Min et al. (2000)**
Negative Intensities = **Allowed**
Collector Calibrations = **36Ar**
Decay 40K = **5.530 ± 0.048 E-10 1/a**
Decay 39Ar = **2.940 ± 0.016 E-07 1/h**
Decay 37Ar = **8.230 ± 0.012 E-04 1/h**
Decay 36Cl = **2.257 ± 0.015 E-06 1/a**
Decay 40K(EC,β^+) = **0.580 ± 0.009 E-10 1/a**
Decay 40K(β^-) = **4.950 ± 0.043 E-10 1/a**
Atmospheric 40/36(a) = **295.50 ± 0.70**
Atmospheric 38/36(a) = **0.1869**
Production 39/37(ca) = **0.0006425 ± 0.0000059**
Production 38/37(ca) = **0.0001800 ± 0.0000173**
Production 36/37(ca) = **0.0002703 ± 0.0000005**
Production 40/39(k) = **0.000607 ± 0.000059**
Production 38/39(k) = **0.012077 ± 0.000011**
Production 36/38(cl) = **262.80 ± 1.71**
Scaling Ratio K/Ca = **0.430**
Abundance Ratio 40K/K = **1.1700 ± 0.0100 E-04**
Atomic Weight K = **39.0983 ± 0.0001 g**

Results

Results	40(a)/36(a) ± 2σ	40(r)/39(k) ± 2σ	Age ± 2σ (ka)	MSWD	39Ar(k) (%,n)	K/Ca ± 2σ
**Age Plateau**		0.16668 ± 0.01042<br>± 6.25%	**473.9 ± 29.6**<br>**± 6.25%**	0.80<br>72%	83.02<br>21	0.0213 ± 0.0078
			Full External Error ± 31.5	1.63	2σ Confidence Limit	
			Analytical Error ± 29.6	1.0000	Error Magnification	
**Total Fusion Age**		0.17926 ± 0.01567<br>± 8.74%	**509.6 ± 44.6**<br>**± 8.74%**		24	0.0510 ± 0.0001
			Full External Error ± 46.0			
			Analytical Error ± 44.5			
**Normal Isochron**	**294.53** ± 0.95<br>± 0.32%	0.17962 ± 0.01559<br>± 8.68%	**510.7 ± 44.3**<br>**± 8.68%**	0.87<br>62%	83.02<br>21	
			Full External Error ± 45.8	1.65	2σ Confidence Limit	
			Analytical Error ± 44.3	1.0000	Error Magnification	
				4	Number of Iterations	
				0.0000000918	Convergence	
**Inverse Isochron**	**294.54** ± 0.95<br>± 0.32%	0.17980 ± 0.01532<br>± 8.52%	**511.2 ± 43.6**<br>**± 8.52%**	0.87<br>63%	83.02<br>21	
			Full External Error ± 45.1	1.65	2σ Confidence Limit	
			Analytical Error ± 43.6	1.0000	Error Magnification	
				3	Number of Iterations	
				0.0001787145	Convergence	
				6%	Spreading Factor	

Incremental Heating			36Ar(a) [fA]	37Ar(ca) [fA]	38Ar(cl) [fA]	39Ar(k) [fA]	40Ar(r) [fA]	Age ± 2σ (ka)	40Ar(r) (%)	39Ar(k) (%)	K/Ca ± 2σ
17D30557	1.8 %		1.913103	75.2381	0.0679288	25.87527	7.275538	799.3 ± 454.6	1.27	6.27	0.1479 ± 0.0018
17D30559	1.9 %		0.703912	65.6847	0.0227203	17.92342	4.704942	746.2 ± 261.8	2.21	4.34	0.1173 ± 0.0015
17D30560	2.0 %		0.634442	113.8299	0.0000000	26.30102	6.663164	720.2 ± 171.4	3.43	6.37	0.0994 ± 0.0010
17D30562	2.2 %	✓	0.285428	54.3267	0.0267007	12.33175	1.776558	409.6 ± 197.3	2.06	2.99	0.0976 ± 0.0014
17D30563	2.4 %	✓	0.356590	112.4846	0.0000000	22.63097	3.687754	463.3 ± 126.3	3.38	5.48	0.0865 ± 0.0009
17D30565	2.7 %	✓	0.322562	138.1745	0.0224084	25.73997	4.378264	483.6 ± 99.9	4.39	6.24	0.0801 ± 0.0008
17D30566	3.0 %	✓	0.225773	108.1421	0.0082753	19.21096	2.902416	429.5 ± 112.7	4.17	4.65	0.0764 ± 0.0008
17D30568	3.4 %	✓	0.338912	224.6949	0.0000000	36.29433	6.388105	500.4 ± 78.8	5.99	8.79	0.0695 ± 0.0006
17D30569	3.9 %	✓	0.232836	170.2959	0.0000000	25.79193	4.308169	474.9 ± 86.7	5.89	6.25	0.0651 ± 0.0006
17D30571	4.5 %	✓	0.440043	383.1541	0.0000000	52.55596	9.948109	538.1 ± 67.3	7.11	12.73	0.0590 ± 0.0005
17D30572	5.2 %	✓	0.185737	137.1792	0.0000000	17.60221	2.307166	372.7 ± 106.5	4.03	4.26	0.0552 ± 0.0005
17D30574	6.0 %	✓	0.238177	168.5820	0.0165162	20.61139	3.407414	470.0 ± 110.8	4.62	4.99	0.0526 ± 0.0005
17D30575	6.9 %	✓	0.211195	128.9345	0.0000000	14.87015	2.493594	476.7 ± 132.5	3.84	3.60	0.0496 ± 0.0005
17D30577	7.9 %	✓	0.396793	235.1097	0.0004112	24.06849	4.257187	502.9 ± 134.3	3.50	5.83	0.0440 ± 0.0004
17D30578	9.0 %	✓	0.387583	175.1726	0.0000000	15.58430	2.580595	470.8 ± 191.8	2.20	3.78	0.0383 ± 0.0004
17D30580	10.3 %	✓	0.437012	161.7521	0.0232027	13.13056	1.658437	359.1 ± 257.0	1.27	3.18	0.0349 ± 0.0003
17D30581	11.6 %	✓	0.428521	140.2053	0.0565517	11.35696	1.838737	460.3 ± 281.1	1.43	2.75	0.0348 ± 0.0004
17D30583	12.5 %	✓	0.393151	122.4003	0.0267217	8.08337	0.240850	84.7 ± 367.4	0.21	1.96	0.0284 ± 0.0003
17D30584	13.4 %	✓	0.181564	58.8556	0.0047789	2.94004	0.343814	332.5 ± 590.7	0.64	0.71	0.0215 ± 0.0004
17D30586	14.6 %	✓	0.265726	98.4058	0.0000000	3.76450	0.434098	327.9 ± 593.3	0.55	0.91	0.0164 ± 0.0003
17D30587	16.0 %	✓	0.111200	43.2673	0.0000000	1.70987	0.016218	27.0 ± 859.2	0.05	0.41	0.0170 ± 0.0005
17D30589	17.6 %	✓	0.287247	107.7847	0.0089644	3.07205	0.634658	587.3 ± 793.1	0.74	0.74	0.0123 ± 0.0002
17D30590	19.3 %	✓	0.524676	197.1238	0.0301199	4.88485	0.718567	418.2 ± 695.9	0.46	1.18	0.0107 ± 0.0001
17D30592	21.0 %	✓	0.718445	258.3394	0.0444826	6.40788	1.022986	453.9 ± 627.0	0.48	1.55	0.0107 ± 0.0001
		Σ	10.220627	3479.1376	0.3597827	412.74224	73.987341				

Information on Analysis

Project = **O-CONNOR (16-22)**
Sample = **PS59-252-1**
Material = **Groundmass**
Location = **Gakkel Ridge**
Region = **Artic Ocean**
Analyst = **Dan Miggins**
Irradiation = **17-OSU-05 (5A26-17)**
J = **0.00157239 ± 0.00000162**
FCT-NM = **28.201 ± 0.023 Ma**

Results	40(r)/39(k) ± 2σ		Age ± 2σ (ka)		MSWD	39Ar(k) (%,n)	K/Ca ± 2σ
**Age Plateau**	0.16668	± 0.01042<br>± 6.25%	**473.9**	**± 29.6**<br>**± 6.25%**	0.80<br>72%	83.02<br>21	0.0213 ± 0.0078
			Full External Error	± 31.5	1.63	2σ Confidence Limit	
			Analytical Error	± 29.6	1.0000	Error Magnification	
**Total Fusion Age**	0.17926	± 0.01567<br>± 8.74%	**509.6**	**± 44.6**<br>**± 8.74%**		24	0.0510 ± 0.0001
			Full External Error	± 46.0			
			Analytical Error	± 44.5			

**Normal Isochron**			**39(k)/36(a) ± 2σ**	**40(a+r)/36(a) ± 2σ**	**r.i.**
17D30557	1.8 %		13.53 ± 0.08	299.30 ± 1.67	0.9159
17D30559	1.9 %		25.46 ± 0.18	302.18 ± 1.92	0.8887
17D30560	2.0 %		41.46 ± 0.30	306.00 ± 2.14	0.9409
17D30562	2.2 %	✓	43.20 ± 0.41	301.72 ± 2.70	0.8856
17D30563	2.4 %	✓	63.46 ± 0.53	305.84 ± 2.53	0.9331
17D30565	2.7 %	✓	79.80 ± 0.66	309.07 ± 2.54	0.9361
17D30566	3.0 %	✓	85.09 ± 0.88	308.36 ± 3.19	0.9294
17D30568	3.4 %	✓	107.09 ± 0.94	314.35 ± 2.77	0.9567
17D30569	3.9 %	✓	110.77 ± 1.14	314.00 ± 3.25	0.9402
17D30571	4.5 %	✓	119.43 ± 0.99	318.11 ± 2.64	0.9641
17D30572	5.2 %	✓	94.77 ± 1.02	307.92 ± 3.38	0.9171
17D30574	6.0 %	✓	86.54 ± 0.89	309.81 ± 3.21	0.9329
17D30575	6.9 %	✓	70.41 ± 0.71	307.31 ± 3.07	0.9015
17D30577	7.9 %	✓	60.66 ± 0.52	306.23 ± 2.59	0.9390
17D30578	9.0 %	✓	40.21 ± 0.33	302.16 ± 2.37	0.8963
17D30580	10.3 %	✓	30.05 ± 0.26	299.29 ± 2.36	0.8822
17D30581	11.6 %	✓	26.50 ± 0.22	299.79 ± 2.25	0.8470
17D30583	12.5 %	✓	20.56 ± 0.19	296.11 ± 2.26	0.7823
17D30584	13.4 %	✓	16.19 ± 0.29	297.39 ± 3.08	0.4999
17D30586	14.6 %	✓	14.17 ± 0.21	297.13 ± 2.62	0.5400
17D30587	16.0 %	✓	15.38 ± 0.45	295.65 ± 4.43	0.4274
17D30589	17.6 %	✓	10.69 ± 0.18	297.71 ± 2.65	0.5014
17D30590	19.3 %	✓	9.31 ± 0.11	296.87 ± 2.29	0.6168
17D30592	21.0 %	✓	8.92 ± 0.08	296.92 ± 1.98	0.6965

**Results**	**40(a)/36(a) ± 2σ**	**40(r)/39(k) ± 2σ**	**Age ± 2σ (ka)**	**MSWD**
**Normal Isochron**	**294.53 ± 0.95 ± 0.32%**	0.17962 ± 0.01559 ± 8.68%	**510.7 ± 44.3 ± 8.68%**	0.87 62%
			Full External Error ± 45.8	
			Analytical Error ± 44.3	

**Statistics**				
	2σ Confidence Limit	1.65	Convergence	0.000000091765
	Error Magnification	1.0000	Number of Iterations	4
	Number of Data Points	21	Calculated Line	Weighted York-2

Inverse Isochron			39(k)/40(a+r) ± 2σ	36(a)/40(a+r) ± 2σ	r.i.
17D30557	1.8 %		0.0451893 ± 0.0001079	0.00334110 ± 0.00001863	0.0587
17D30559	1.9 %		0.0842618 ± 0.0002675	0.00330924 ± 0.00002105	0.0698
17D30560	2.0 %		0.1354739 ± 0.0003310	0.00326795 ± 0.00002286	0.0989
17D30562	2.2 %	✓	0.1431919 ± 0.0006334	0.00331429 ± 0.00002969	0.1364
17D30563	2.4 %	✓	0.2075092 ± 0.0006330	0.00326967 ± 0.00002702	0.1406
17D30565	2.7 %	✓	0.2581862 ± 0.0007576	0.00323548 ± 0.00002654	0.1729
17D30566	3.0 %	✓	0.2759468 ± 0.0010704	0.00324301 ± 0.00003358	0.2032
17D30568	3.4 %	✓	0.3406753 ± 0.0008825	0.00318118 ± 0.00002807	0.1594
17D30569	3.9 %	✓	0.3527771 ± 0.0012577	0.00318468 ± 0.00003297	0.2013
17D30571	4.5 %	✓	0.3754515 ± 0.0008337	0.00314359 ± 0.00002607	0.1401
17D30572	5.2 %	✓	0.3077713 ± 0.0013651	0.00324758 ± 0.00003569	0.2496
17D30574	6.0 %	✓	0.2793302 ± 0.0010576	0.00322782 ± 0.00003341	0.1895
17D30575	6.9 %	✓	0.2291185 ± 0.0010199	0.00325407 ± 0.00003256	0.2117
17D30577	7.9 %	✓	0.1980791 ± 0.0005901	0.00326553 ± 0.00002759	0.1289
17D30578	9.0 %	✓	0.1330724 ± 0.0004936	0.00330952 ± 0.00002599	0.1073
17D30580	10.3 %	✓	0.1003901 ± 0.0004070	0.00334119 ± 0.00002630	0.0730
17D30581	11.6 %	✓	0.0884040 ± 0.0003987	0.00333566 ± 0.00002498	0.0714
17D30583	12.5 %	✓	0.0694347 ± 0.0004021	0.00337709 ± 0.00002580	0.0661
17D30584	13.4 %	✓	0.0544492 ± 0.0008569	0.00336255 ± 0.00003478	0.0819
17D30586	14.6 %	✓	0.0476784 ± 0.0006023	0.00336549 ± 0.00002964	0.0565
17D30587	16.0 %	✓	0.0520098 ± 0.0013884	0.00338243 ± 0.00005071	0.0908
17D30589	17.6 %	✓	0.0359237 ± 0.0005155	0.00335898 ± 0.00002993	0.0420
17D30590	19.3 %	✓	0.0313614 ± 0.0002992	0.00336848 ± 0.00002598	0.0250
17D30592	21.0 %	✓	0.0300383 ± 0.0002017	0.00336787 ± 0.00002242	0.0207

Results	40(a)/36(a) ± 2σ	40(r)/39(k) ± 2σ	Age ± 2σ (ka)	MSWD
**Inverse Isochron**	**294.54 ± 0.95 ± 0.32%**	0.17980 ± 0.01532 ± 8.52%	**511.2 ± 43.6 ± 8.52%**	0.87 63%
			Full External Error ± 45.1	
			Analytical Error ± 43.6	

Statistics					
	2σ Confidence Limit	1.65	Convergence	0.0001787145
	Error Magnification	1.0000	Number of Iterations	3
	Number of Data Points	21	Calculated Line	Weighted York-2
	Spreading Factor	6.2%		

Degassing Patterns

			36Ar(a) [fA]	%1σ	36Ar(c) [fA]	%1σ	36Ar(ca) [fA]	%1σ	36Ar(cl) [fA]	%1σ	37Ar(ca) [fA]	%1σ	38Ar(a) [fA]	%1σ	38Ar(c) [fA]	%1σ
17D30557	1.8 %		1.913103	0.28	0.0000000	0.00	0.0203369	0.63	0.0000140	34.21	75.2381	0.60	0.3575589	0.28	0.0000000	0.00
17D30559	1.9 %		0.703912	0.31	0.0000000	0.00	0.0177546	0.66	0.0000047	110.22	65.6847	0.63	0.1315612	0.31	0.0000000	0.00
17D30560	2.0 %		0.634442	0.34	0.0000000	0.00	0.0307682	0.52	0.0000000	0.00	113.8299	0.49	0.1185773	0.34	0.0000000	0.00
17D30562	2.2 %	✓	0.285428	0.43	0.0000000	0.00	0.0146845	0.73	0.0000055	87.19	54.3267	0.71	0.0533464	0.43	0.0000000	0.00
17D30563	2.4 %	✓	0.356590	0.40	0.0000000	0.00	0.0304046	0.52	0.0000000	0.00	112.4846	0.49	0.0666467	0.40	0.0000000	0.00
17D30565	2.7 %	✓	0.322562	0.40	0.0000000	0.00	0.0373486	0.50	0.0000046	108.80	138.1745	0.47	0.0602869	0.40	0.0000000	0.00
17D30566	3.0 %	✓	0.225773	0.50	0.0000000	0.00	0.0292308	0.53	0.0000017	291.44	108.1421	0.50	0.0421970	0.50	0.0000000	0.00
17D30568	3.4 %	✓	0.338912	0.43	0.0000000	0.00	0.0607350	0.46	0.0000000	0.00	224.6949	0.43	0.0633426	0.43	0.0000000	0.00
17D30569	3.9 %	✓	0.232836	0.50	0.0000000	0.00	0.0460310	0.48	0.0000000	0.00	170.2959	0.45	0.0435170	0.50	0.0000000	0.00
17D30571	4.5 %	✓	0.440043	0.41	0.0000000	0.00	0.1035666	0.44	0.0000000	0.00	383.1541	0.41	0.0822440	0.41	0.0000000	0.00
17D30572	5.2 %	✓	0.185737	0.52	0.0000000	0.00	0.0370795	0.49	0.0000000	0.00	137.1792	0.46	0.0347143	0.52	0.0000000	0.00
17D30574	6.0 %	✓	0.238177	0.50	0.0000000	0.00	0.0455677	0.48	0.0000034	148.96	168.5820	0.44	0.0445152	0.50	0.0000000	0.00
17D30575	6.9 %	✓	0.211195	0.48	0.0000000	0.00	0.0348510	0.50	0.0000000	0.00	128.9345	0.47	0.0394723	0.48	0.0000000	0.00
17D30577	7.9 %	✓	0.396793	0.41	0.0000000	0.00	0.0635502	0.46	0.0000001	######	235.1097	0.42	0.0741606	0.41	0.0000000	0.00
17D30578	9.0 %	✓	0.387583	0.38	0.0000000	0.00	0.0473491	0.47	0.0000000	0.00	175.1726	0.44	0.0724393	0.38	0.0000000	0.00
17D30580	10.3 %	✓	0.437012	0.39	0.0000000	0.00	0.0437216	0.48	0.0000048	102.84	161.7521	0.45	0.0816775	0.39	0.0000000	0.00
17D30581	11.6 %	✓	0.428521	0.37	0.0000000	0.00	0.0378975	0.49	0.0000117	44.64	140.2053	0.46	0.0800905	0.37	0.0000000	0.00
17D30583	12.5 %	✓	0.393151	0.37	0.0000000	0.00	0.0330848	0.51	0.0000055	87.58	122.4003	0.48	0.0734799	0.37	0.0000000	0.00
17D30584	13.4 %	✓	0.181564	0.48	0.0000000	0.00	0.0159087	0.70	0.0000010	524.01	58.8556	0.68	0.0339344	0.48	0.0000000	0.00
17D30586	14.6 %	✓	0.265726	0.42	0.0000000	0.00	0.0265991	0.55	0.0000000	0.00	98.4058	0.53	0.0496642	0.42	0.0000000	0.00
17D30587	16.0 %	✓	0.111200	0.69	0.0000000	0.00	0.0116952	0.86	0.0000000	0.00	43.2673	0.85	0.0207833	0.69	0.0000000	0.00
17D30589	17.6 %	✓	0.287247	0.43	0.0000000	0.00	0.0291342	0.53	0.0000019	272.26	107.7847	0.50	0.0536864	0.43	0.0000000	0.00
17D30590	19.3 %	✓	0.524676	0.38	0.0000000	0.00	0.0532826	0.47	0.0000062	77.73	197.1238	0.43	0.0980619	0.38	0.0000000	0.00
17D30592	21.0 %	✓	0.718445	0.33	0.0000000	0.00	0.0698292	0.45	0.0000092	50.97	258.3394	0.42	0.1342774	0.33	0.0000000	0.00
		Σ	10.220627	0.09	0.0000000	0.00	0.9404109	0.11	0.0000743	25.05	3479.1376	0.10	1.9102351	0.09	0.0000000	0.00
		Σ							11.161112	0.08	3479.1376	0.10				

	38Ar(k) [fA]	%1σ	38Ar(ca) [fA]	%1σ	38Ar(cl) [fA]	%1σ	39Ar(k) [fA]	%1σ	39Ar(ca) [fA]	%1σ	40Ar(r) [fA]	%1σ	40Ar(a) [fA]	%1σ	40Ar(c) [fA]	%1σ	40Ar(k) [fA]	%1σ
17D30557	0.3124956	0.14	0.0135429	9.65	0.0679288	34.22	25.87527	0.11	0.0483405	1.10	7.275538	28.44	565.3219	0.36	0.0000000	0.00	0.0157063	9.65
17D30559	0.2164612	0.17	0.0118232	9.65	0.0227203	110.23	17.92342	0.15	0.0422024	1.12	4.704942	17.54	208.0061	0.39	0.0000000	0.00	0.0108795	9.65
17D30560	0.3176374	0.14	0.0204894	9.64	0.0000000	0.00	26.30102	0.10	0.0731357	1.04	6.663164	11.90	187.4777	0.42	0.0000000	0.00	0.0159647	9.65
17D30562	0.1489306	0.21	0.0097788	9.66	0.0267007	87.19	12.33175	0.19	0.0349049	1.16	1.776558	24.09	84.3439	0.49	0.0000000	0.00	0.0074854	9.65
17D30563	0.2733142	0.15	0.0202472	9.64	0.0000000	0.00	22.63097	0.12	0.0722713	1.04	3.687754	13.63	105.3723	0.47	0.0000000	0.00	0.0137370	9.65
17D30565	0.3108616	0.14	0.0248714	9.64	0.0224084	108.80	25.73997	0.11	0.0887771	1.03	4.378264	10.33	95.3171	0.46	0.0000000	0.00	0.0156242	9.65
17D30566	0.2320108	0.16	0.0194656	9.64	0.0082753	291.45	19.21096	0.13	0.0694813	1.05	2.902416	13.12	66.7159	0.55	0.0000000	0.00	0.0116611	9.65
17D30568	0.4383267	0.13	0.0404451	9.64	0.0000000	0.00	36.29433	0.09	0.1443665	1.01	6.388105	7.87	100.1484	0.49	0.0000000	0.00	0.0220307	9.65
17D30569	0.3114892	0.15	0.0306533	9.64	0.0000000	0.00	25.79193	0.11	0.1094151	1.02	4.308169	9.13	68.8030	0.55	0.0000000	0.00	0.0156557	9.65
17D30571	0.6347183	0.12	0.0689677	9.64	0.0000000	0.00	52.55596	0.08	0.2461765	1.01	9.948109	6.26	130.0326	0.47	0.0000000	0.00	0.0319015	9.65
17D30572	0.2125819	0.16	0.0246922	9.64	0.0000000	0.00	17.60221	0.14	0.0881376	1.03	2.307166	14.29	54.8853	0.57	0.0000000	0.00	0.0106845	9.65
17D30574	0.2489238	0.16	0.0303448	9.64	0.0165162	148.97	20.61139	0.13	0.1083139	1.02	3.407414	11.79	70.3812	0.55	0.0000000	0.00	0.0125111	9.65
17D30575	0.1795868	0.18	0.0232082	9.64	0.0000000	0.00	14.87015	0.16	0.0828404	1.03	2.493594	13.90	62.4080	0.53	0.0000000	0.00	0.0090262	9.65
17D30577	0.2906752	0.15	0.0423198	9.64	0.0004112	######	24.06849	0.12	0.1510580	1.01	4.257187	13.36	117.2523	0.48	0.0000000	0.00	0.0146096	9.65
17D30578	0.1882116	0.19	0.0315311	9.64	0.0000000	0.00	15.58430	0.16	0.1125484	1.02	2.580595	20.37	114.5309	0.45	0.0000000	0.00	0.0094597	9.65
17D30580	0.1585778	0.21	0.0291154	9.64	0.0232027	102.84	13.13056	0.19	0.1039257	1.02	1.658437	35.78	129.1369	0.45	0.0000000	0.00	0.0079703	9.65
17D30581	0.1371580	0.23	0.0252369	9.64	0.0565517	44.65	11.35696	0.21	0.0900819	1.03	1.838737	30.54	126.6279	0.44	0.0000000	0.00	0.0068937	9.65
17D30583	0.0976229	0.29	0.0220321	9.64	0.0267217	87.59	8.08337	0.28	0.0786422	1.04	0.240850	216.84	116.1760	0.44	0.0000000	0.00	0.0049066	9.65
17D30584	0.0355069	0.77	0.0105940	9.65	0.0047789	524.02	2.94004	0.77	0.0378148	1.14	0.343814	88.84	53.6523	0.54	0.0000000	0.00	0.0017846	9.68
17D30586	0.0454639	0.63	0.0177130	9.64	0.0000000	0.00	3.76450	0.62	0.0632257	1.06	0.434098	90.48	78.5221	0.48	0.0000000	0.00	0.0022851	9.67
17D30587	0.0206501	1.30	0.0077881	9.67	0.0000000	0.00	1.70987	1.30	0.0277992	1.25	0.016218	######	32.8597	0.73	0.0000000	0.00	0.0010379	9.74
17D30589	0.0371012	0.71	0.0194012	9.64	0.0089644	272.27	3.07205	0.71	0.0692517	1.05	0.634658	67.53	84.8814	0.49	0.0000000	0.00	0.0018647	9.68
17D30590	0.0589944	0.48	0.0354823	9.64	0.0301199	77.74	4.88485	0.47	0.1266521	1.02	0.718567	83.21	155.0417	0.38	0.0000000	0.00	0.0029651	9.66
17D30592	0.0773880	0.34	0.0465011	9.64	0.0444826	50.98	6.40788	0.33	0.1659831	1.01	1.022986	69.08	212.3005	0.33	0.0000000	0.00	0.0038896	9.66
Σ	4.9846880	0.04	0.6262448	2.21	0.3597827	25.05	412.74224	0.03	2.2353459	0.24	73.987341	4.37	3020.1952	0.11	0.0000000	0.00	0.2505345	2.38
Σ					7.8809506	1.16			414.97758	0.03							3094.4330	0.15

Additional Parameters			40Ar/39Ar	1σ	37Ar/39Ar	1σ	36Ar/39Ar	1σ	Time (days)	37Ar (decay)	39Ar (decay)	40Ar (moles)
17D30557	1.8 %		22.088481	0.026321	2.902300	0.017830	0.074583	0.000219	126.964	12.400092	1.00089982	2.749E-11
17D30559	1.9 %		11.840500	0.018756	3.656131	0.023771	0.040170	0.000136	126.978	12.403495	1.00089991	1.021E-11
17D30560	2.0 %		7.361633	0.008973	4.315963	0.021646	0.025222	0.000086	126.985	12.405196	1.00089996	9.320E-12
17D30562	2.2 %	✓	6.964528	0.015369	4.392993	0.032085	0.024268	0.000109	126.999	12.408600	1.00090006	4.134E-12
17D30563	2.4 %	✓	4.804328	0.007311	4.954560	0.025168	0.017046	0.000066	127.006	12.410302	1.00090011	5.236E-12
17D30565	2.7 %	✓	3.860467	0.005652	5.349642	0.025698	0.013935	0.000051	127.019	12.413707	1.00090021	4.786E-12
17D30566	3.0 %	✓	3.611432	0.006991	5.608900	0.028899	0.013226	0.000060	127.026	12.415410	1.00090026	3.342E-12
17D30568	3.4 %	✓	2.924322	0.003778	6.166380	0.026848	0.010968	0.000040	127.040	12.418816	1.00090036	5.115E-12
17D30569	3.9 %	✓	2.823281	0.005021	6.574788	0.030231	0.010766	0.000046	127.047	12.420520	1.00090041	3.510E-12
17D30571	4.5 %	✓	2.651647	0.002934	7.256413	0.030310	0.010295	0.000034	127.061	12.423928	1.00090050	6.721E-12
17D30572	5.2 %	✓	3.233581	0.007154	7.754465	0.037354	0.012595	0.000056	127.068	12.425632	1.00090055	2.746E-12
17D30574	6.0 %	✓	3.561881	0.006723	8.136313	0.037679	0.013695	0.000059	127.083	12.429212	1.00090066	3.542E-12
17D30575	6.9 %	✓	4.340978	0.009630	8.622652	0.043037	0.016455	0.000071	127.090	12.430917	1.00090070	3.116E-12
17D30577	7.9 %	✓	5.017604	0.007437	9.707436	0.042772	0.019007	0.000070	127.103	12.434327	1.00090080	5.833E-12
17D30578	9.0 %	✓	7.461428	0.013751	11.159728	0.052610	0.027708	0.000104	127.110	12.436033	1.00090085	5.622E-12
17D30580	10.3 %	✓	9.883521	0.019884	12.222010	0.059054	0.036325	0.000143	127.124	12.439445	1.00090095	6.279E-12
17D30581	11.6 %	✓	11.223293	0.025119	12.248162	0.062023	0.040747	0.000161	127.131	12.441151	1.00090100	6.167E-12
17D30583	12.5 %	✓	14.263855	0.040912	14.996344	0.082904	0.052223	0.000228	127.145	12.444565	1.00090110	5.588E-12
17D30584	13.4 %	✓	18.133131	0.140949	19.764425	0.200771	0.066314	0.000580	127.152	12.446272	1.00090115	2.592E-12
17D30586	14.6 %	✓	20.628025	0.128178	25.708665	0.206889	0.076370	0.000548	127.166	12.449687	1.00090124	3.790E-12
17D30587	16.0 %	✓	18.920159	0.248674	24.899647	0.381826	0.070724	0.001004	127.173	12.451395	1.00090129	1.578E-12
17D30589	17.6 %	✓	27.223683	0.191041	34.312076	0.292698	0.100717	0.000799	127.187	12.454811	1.00090139	4.105E-12
17D30590	19.3 %	✓	31.081113	0.144365	39.334250	0.248441	0.115327	0.000661	127.194	12.456520	1.00090144	7.477E-12
17D30592	21.0 %	✓	32.450822	0.105948	39.297936	0.208473	0.119912	0.000526	127.208	12.459937	1.00090154	1.024E-11

**Procedure Blanks**		36Ar ± 1σ (SE) [fA]	37Ar ± 1σ (SE) [fA]	38Ar ± 1σ (SE) [fA]	39Ar ± 1σ (SE) [fA]	40Ar ± 1σ (SE) [fA]
17D30557	1.8 %	0.0095069 ± 0.0002998	0.0320158 ± 0.0180117	0.0098392 ± 0.0168902	0.0270575 ± 0.0155226	2.9466627 ± 0.0965938
17D30559	1.9 %	0.0102011 ± 0.0002998	0.0225457 ± 0.0180117	0.0155469 ± 0.0168902	0.0415065 ± 0.0155226	3.1674019 ± 0.0965938
17D30560	2.0 %	0.0104222 ± 0.0002998	0.0193537 ± 0.0180117	0.0172095 ± 0.0168902	0.0465773 ± 0.0155226	3.2355358 ± 0.0965938
17D30562	2.2 %	0.0106639 ± 0.0002998	0.0154360 ± 0.0180117	0.0185901 ± 0.0168902	0.0532057 ± 0.0155226	3.3041885 ± 0.0965938
17D30563	2.4 %	0.0107033 ± 0.0002998	0.0144830 ± 0.0180117	0.0184716 ± 0.0168902	0.0550571 ± 0.0155226	3.3109072 ± 0.0965938
17D30565	2.7 %	0.0106617 ± 0.0002998	0.0140720 ± 0.0180117	0.0169972 ± 0.0168902	0.0565092 ± 0.0155226	3.2832722 ± 0.0965938
17D30566	3.0 %	0.0105961 ± 0.0002998	0.0144278 ± 0.0180117	0.0157819 ± 0.0168902	0.0563560 ± 0.0155226	3.2540331 ± 0.0965938
17D30568	3.4 %	0.0104093 ± 0.0002998	0.0158479 ± 0.0180117	0.0127179 ± 0.0168902	0.0548467 ± 0.0155226	3.1761414 ± 0.0965938
17D30569	3.9 %	0.0102999 ± 0.0002998	0.0167672 ± 0.0180117	0.0109869 ± 0.0168902	0.0536890 ± 0.0155226	3.1315179 ± 0.0965938
17D30571	4.5 %	0.0100745 ± 0.0002998	0.0187127 ± 0.0180117	0.0073923 ± 0.0168902	0.0510036 ± 0.0155226	3.0396323 ± 0.0965938
17D30572	5.2 %	0.0099668 ± 0.0002998	0.0196351 ± 0.0180117	0.0056235 ± 0.0168902	0.0496266 ± 0.0155226	2.9953139 ± 0.0965938
17D30574	6.0 %	0.0097698 ± 0.0002998	0.0212328 ± 0.0180117	0.0022003 ± 0.0168902	0.0470102 ± 0.0155226	2.9123890 ± 0.0965938
17D30575	6.9 %	0.0096951 ± 0.0002998	0.0217661 ± 0.0180117	0.0007845 ± 0.0168902	0.0460035 ± 0.0155226	2.8796806 ± 0.0965938
17D30577	7.9 %	0.0095903 ± 0.0002998	0.0222874 ± 0.0180117	0.0014797 ± 0.0168902	0.0446517 ± 0.0155226	2.8306721 ± 0.0965938
17D30578	9.0 %	0.0095615 ± 0.0002998	0.0222549 ± 0.0180117	0.0022802 ± 0.0168902	0.0443602 ± 0.0155226	2.8151090 ± 0.0965938
17D30580	10.3 %	0.0095501 ± 0.0002998	0.0216042 ± 0.0180117	0.0031265 ± 0.0168902	0.0446204 ± 0.0155226	2.8023509 ± 0.0965938
17D30581	11.6 %	0.0095654 ± 0.0002998	0.0210066 ± 0.0180117	0.0031472 ± 0.0168902	0.0451779 ± 0.0155226	2.8048077 ± 0.0965938
17D30583	12.5 %	0.0096278 ± 0.0002998	0.0193702 ± 0.0180117	0.0023498 ± 0.0168902	0.0471031 ± 0.0155226	2.8251655 ± 0.0965938
17D30584	13.4 %	0.0096694 ± 0.0002998	0.0183932 ± 0.0180117	0.0015294 ± 0.0168902	0.0484288 ± 0.0155226	2.8416328 ± 0.0965938
17D30586	14.6 %	0.0097549 ± 0.0002998	0.0163276 ± 0.0180117	0.0009311 ± 0.0168902	0.0516423 ± 0.0155226	2.8822038 ± 0.0965938
17D30587	16.0 %	0.0097896 ± 0.0002998	0.0153419 ± 0.0180117	0.0025505 ± 0.0168902	0.0534405 ± 0.0155226	2.9037884 ± 0.0965938
17D30589	17.6 %	0.0098159 ± 0.0002998	0.0137734 ± 0.0180117	0.0064868 ± 0.0168902	0.0571363 ± 0.0155226	2.9419018 ± 0.0965938
17D30590	19.3 %	0.0097948 ± 0.0002998	0.0133346 ± 0.0180117	0.0087599 ± 0.0168902	0.0588967 ± 0.0155226	2.9548263 ± 0.0965938
17D30592	21.0 %	0.0096483 ± 0.0002998	0.0135595 ± 0.0180117	0.0137782 ± 0.0168902	0.0618392 ± 0.0155226	2.9580436 ± 0.0965938

**Intercept Values**		**36Ar ± 1σ (SE) [fA]**	**r2**	**Regression (type,n)**	**37Ar ± 1σ (SE) [fA]**	**r2**	**Regression (type,n)**	**38Ar ± 1σ (SE) [fA]**	**r2**	**Regression (type,n)**	**39Ar ± 1σ (SE) [fA]**	**r2**	**Regression (type,n)**	**40Ar ± 1σ (SE) [fA]**	**r2**	**Regression (type,n)**
17D30557	1.8 %	1.8564405 ± 0.0022410	0.9617	EXP 150 of 150	5.990176 ± 0.020112	0.7519	EXP 150 of 150	0.7326433 ± 0.0154158	0.0068	EXP 150 of 150	25.7717950 ± 0.0182920	0.9845	EXP 150 of 150	575.55981 ± 0.23392	0.6679	EXP 150 of 150
17D30559	1.9 %	0.6995787 ± 0.0012237	0.8872	EXP 150 of 150	5.222737 ± 0.018113	0.7197	EXP 150 of 150	0.3624154 ± 0.0180282	0.0144	EXP 150 of 150	17.8831664 ± 0.0182302	0.9664	EXP 150 of 150	215.88936 ± 0.08124	0.9875	EXP 150 of 150
17D30560	2.0 %	0.6458653 ± 0.0013547	0.8096	EXP 150 of 150	9.029918 ± 0.018262	0.8902	EXP 150 of 150	0.4335104 ± 0.0166944	0.0190	EXP 150 of 150	26.2387485 ± 0.0156249	0.9900	EXP 150 of 150	197.39239 ± 0.08118	0.9899	EXP 150 of 150
17D30562	2.2 %	0.2973517 ± 0.0008970	0.4344	EXP 150 of 150	4.314655 ± 0.017348	0.6705	EXP 150 of 150	0.2172933 ± 0.0155722	0.0117	EXP 150 of 150	12.3345333 ± 0.0153649	0.9525	EXP 150 of 150	89.43213 ± 0.02627	0.9992	EXP 150 of 150
17D30563	2.4 %	0.3803802 ± 0.0009781	0.6622	EXP 150 of 150	8.914893 ± 0.018428	0.8915	EXP 150 of 150	0.3322806 ± 0.0182025	0.0010	EXP 150 of 150	22.6016416 ± 0.0174622	0.9836	EXP 150 of 150	112.38473 ± 0.03474	0.9983	EXP 150 of 150
17D30565	2.7 %	0.3544712 ± 0.0008228	0.7122	EXP 150 of 150	10.944216 ± 0.019740	0.9136	EXP 150 of 150	0.3963958 ± 0.0169913	0.0295	EXP 150 of 150	25.7070261 ± 0.0158280	0.9904	EXP 150 of 150	102.99427 ± 0.03178	0.9983	EXP 150 of 150
17D30566	3.0 %	0.2541905 ± 0.0008301	0.3143	EXP 150 of 150	8.567716 ± 0.017960	0.8796	EXP 149 of 150	0.2825332 ± 0.0166937	0.0072	EXP 149 of 150	19.2037590 ± 0.0159508	0.9819	EXP 150 of 150	72.88406 ± 0.02361	0.9992	EXP 150 of 150
17D30568	3.4 %	0.3921720 ± 0.0009595	0.7362	EXP 150 of 150	17.782778 ± 0.019231	0.9663	EXP 150 of 150	0.5044025 ± 0.0159098	0.0036	EXP 150 of 150	36.2421044 ± 0.0169249	0.9945	EXP 150 of 150	109.73463 ± 0.03167	0.9979	EXP 150 of 150
17D30569	3.9 %	0.2766876 ± 0.0008246	0.4029	EXP 150 of 150	13.480446 ± 0.019255	0.9421	EXP 150 of 150	0.3592073 ± 0.0155083	0.0089	EXP 150 of 150	25.7763051 ± 0.0197048	0.9850	EXP 150 of 150	76.25830 ± 0.02441	0.9990	EXP 150 of 150
17D30571	4.5 %	0.5293576 ± 0.0010221	0.8609	EXP 150 of 150	30.302763 ± 0.021638	0.9852	EXP 150 of 150	0.7569551 ± 0.0159834	0.0594	EXP 149 of 150	52.4887694 ± 0.0193708	0.9967	EXP 150 of 150	143.05223 ± 0.05740	0.9901	EXP 150 of 150
17D30572	5.2 %	0.2228126 ± 0.0006828	0.3256	EXP 150 of 150	10.860628 ± 0.017432	0.9263	EXP 150 of 150	0.2614870 ± 0.0162981	0.0001	EXP 150 of 150	17.6178979 ± 0.0148892	0.9803	EXP 150 of 150	60.19849 ± 0.02486	0.9990	EXP 150 of 150
17D30574	6.0 %	0.2808202 ± 0.0008500	0.5180	EXP 150 of 150	13.340092 ± 0.018372	0.9460	EXP 150 of 150	0.3340046 ± 0.0172270	0.0237	EXP 150 of 150	20.6237328 ± 0.0181947	0.9801	EXP 150 of 150	76.71351 ± 0.02782	0.9983	EXP 150 of 150
17D30575	6.9 %	0.2447304 ± 0.0006911	0.3260	EXP 150 of 150	10.206865 ± 0.018251	0.9143	EXP 150 of 150	0.2348136 ± 0.0171152	0.0041	EXP 150 of 150	14.8958089 ± 0.0156895	0.9672	EXP 150 of 150	67.79030 ± 0.02443	0.9987	EXP 150 of 150
17D30577	7.9 %	0.4493336 ± 0.0010654	0.7905	EXP 150 of 150	18.589543 ± 0.019252	0.9688	EXP 150 of 150	0.4041419 ± 0.0166554	0.0218	EXP 150 of 150	24.0970660 ± 0.0188422	0.9838	EXP 150 of 150	124.35478 ± 0.05140	0.9902	EXP 150 of 150
17D30578	9.0 %	0.4250311 ± 0.0009210	0.8096	EXP 150 of 150	13.854213 ± 0.019070	0.9402	EXP 150 of 150	0.2784151 ± 0.0166183	0.0002	EXP 150 of 150	15.6328876 ± 0.0175501	0.9630	EXP 150 of 150	119.93602 ± 0.03718	0.9942	EXP 150 of 150
17D30580	10.3 %	0.4687756 ± 0.0011041	0.8395	EXP 150 of 150	12.790349 ± 0.017490	0.9445	EXP 150 of 150	0.2921791 ± 0.0161975	0.0150	EXP 150 of 150	13.1877777 ± 0.0172073	0.9527	EXP 150 of 150	133.60570 ± 0.02521	0.9938	EXP 150 of 150
17D30581	11.6 %	0.4551231 ± 0.0009759	0.8569	EXP 150 of 150	11.087324 ± 0.017745	0.9306	EXP 150 of 150	0.2985859 ± 0.0181742	0.0492	EXP 150 of 150	11.4132260 ± 0.0168049	0.9346	EXP 150 of 150	131.27835 ± 0.02496	0.9927	EXP 150 of 150
17D30583	12.5 %	0.4167949 ± 0.0009260	0.8470	EXP 150 of 150	9.677707 ± 0.018245	0.8978	EXP 150 of 150	0.2195605 ± 0.0156410	0.0105	EXP 150 of 150	8.1527881 ± 0.0151041	0.8931	EXP 149 of 150	119.24693 ± 0.02403	0.9906	EXP 150 of 150
17D30584	13.4 %	0.1983067 ± 0.0006264	0.4431	EXP 150 of 150	4.661923 ± 0.018006	0.6499	EXP 149 of 150	0.0853230 ± 0.0180458	0.0003	EXP 149 of 150	3.0057354 ± 0.0159744	0.2981	EXP 149 of 150	56.83951 ± 0.01981	0.9985	EXP 150 of 150
17D30586	14.6 %	0.2889990 ± 0.0007594	0.7509	EXP 149 of 150	7.778114 ± 0.019502	0.8230	EXP 150 of 150	0.0951760 ± 0.0173018	0.0105	EXP 150 of 150	3.8529551 ± 0.0170035	0.4852	EXP 150 of 150	81.84070 ± 0.02159	0.9967	EXP 150 of 150
17D30587	16.0 %	0.1271856 ± 0.0005922	0.0222	EXP 150 of 150	3.427595 ± 0.017925	0.5458	EXP 150 of 150	0.0453809 ± 0.0166582	0.0031	EXP 150 of 150	1.7791158 ± 0.0156646	0.0455	EXP 150 of 150	35.78073 ± 0.02204	0.9985	EXP 150 of 150
17D30589	17.6 %	0.3120406 ± 0.0008613	0.7479	EXP 149 of 150	8.511824 ± 0.017715	0.8868	EXP 150 of 150	0.1112326 ± 0.0171042	0.0003	EXP 150 of 150	3.1767613 ± 0.0148923	0.4293	EXP 150 of 150	88.45978 ± 0.02193	0.9958	EXP 150 of 150
17D30590	19.3 %	0.5618959 ± 0.0012918	0.8537	EXP 150 of 150	15.553006 ± 0.018436	0.9590	EXP 150 of 150	0.2112191 ± 0.0154258	0.0160	EXP 150 of 150	5.0358159 ± 0.0165290	0.6565	EXP 150 of 150	158.71802 ± 0.04281	0.8961	EXP 150 of 150
17D30592	21.0 %	0.7626569 ± 0.0012531	0.9396	EXP 150 of 150	20.373394 ± 0.019311	0.9733	EXP 150 of 150	0.2852289 ± 0.0140120	0.0605	EXP 149 of 150	6.5903367 ± 0.0137232	0.8403	EXP 150 of 150	216.28546 ± 0.03455	0.9568	EXP 150 of 150

Project Info		Analyst	Irradiation	X-pos	Y-pos	Z/H-pos	Project	Experiment	Nmb
17D30557	1.8 %	Dan Miggins	17-OSU-05	0.00	0.00	37.93	Arctic\O-Connor (16-22)	17D30553	01
17D30559	1.9 %	Dan Miggins	17-OSU-05	0.00	0.00	37.93	Arctic\O-Connor (16-22)	17D30553	01
17D30560	2.0 %	Dan Miggins	17-OSU-05	0.00	0.00	37.93	Arctic\O-Connor (16-22)	17D30553	01
17D30562	2.2 %	Dan Miggins	17-OSU-05	0.00	0.00	37.93	Arctic\O-Connor (16-22)	17D30553	01
17D30563	2.4 %	Dan Miggins	17-OSU-05	0.00	0.00	37.93	Arctic\O-Connor (16-22)	17D30553	01
17D30565	2.7 %	Dan Miggins	17-OSU-05	0.00	0.00	37.93	Arctic\O-Connor (16-22)	17D30553	01
17D30566	3.0 %	Dan Miggins	17-OSU-05	0.00	0.00	37.93	Arctic\O-Connor (16-22)	17D30553	01
17D30568	3.4 %	Dan Miggins	17-OSU-05	0.00	0.00	37.93	Arctic\O-Connor (16-22)	17D30553	01
17D30569	3.9 %	Dan Miggins	17-OSU-05	0.00	0.00	37.93	Arctic\O-Connor (16-22)	17D30553	01
17D30571	4.5 %	Dan Miggins	17-OSU-05	0.00	0.00	37.93	Arctic\O-Connor (16-22)	17D30553	01
17D30572	5.2 %	Dan Miggins	17-OSU-05	0.00	0.00	37.93	Arctic\O-Connor (16-22)	17D30553	01
17D30574	6.0 %	Dan Miggins	17-OSU-05	0.00	0.00	37.93	Arctic\O-Connor (16-22)	17D30553	01
17D30575	6.9 %	Dan Miggins	17-OSU-05	0.00	0.00	37.93	Arctic\O-Connor (16-22)	17D30553	01
17D30577	7.9 %	Dan Miggins	17-OSU-05	0.00	0.00	37.93	Arctic\O-Connor (16-22)	17D30553	01
17D30578	9.0 %	Dan Miggins	17-OSU-05	0.00	0.00	37.93	Arctic\O-Connor (16-22)	17D30553	01
17D30580	10.3 %	Dan Miggins	17-OSU-05	0.00	0.00	37.93	Arctic\O-Connor (16-22)	17D30553	01
17D30581	11.6 %	Dan Miggins	17-OSU-05	0.00	0.00	37.93	Arctic\O-Connor (16-22)	17D30553	01
17D30583	12.5 %	Dan Miggins	17-OSU-05	0.00	0.00	37.93	Arctic\O-Connor (16-22)	17D30553	01
17D30584	13.4 %	Dan Miggins	17-OSU-05	0.00	0.00	37.93	Arctic\O-Connor (16-22)	17D30553	01
17D30586	14.6 %	Dan Miggins	17-OSU-05	0.00	0.00	37.93	Arctic\O-Connor (16-22)	17D30553	01
17D30587	16.0 %	Dan Miggins	17-OSU-05	0.00	0.00	37.93	Arctic\O-Connor (16-22)	17D30553	01
17D30589	17.6 %	Dan Miggins	17-OSU-05	0.00	0.00	37.93	Arctic\O-Connor (16-22)	17D30553	01
17D30590	19.3 %	Dan Miggins	17-OSU-05	0.00	0.00	37.93	Arctic\O-Connor (16-22)	17D30553	01
17D30592	21.0 %	Dan Miggins	17-OSU-05	0.00	0.00	37.93	Arctic\O-Connor (16-22)	17D30553	01

**Sample Parameters**		Sample	Material	Location	Standard Name	Standard (in Ma)	%1σ	Standard Reference	Standard 40Ar/39Ar	%1σ	J	%1σ	Air 40Ar/36Ar	%1σ	MDF (lin)	%1σ	Volume Ratio	Sensitivity (mol/volt)	Day	Month	Year	Hour	Min	Resist
17D30557	1.8 %	PS59-252-1	Groundmass	Gakkel Ridge	FCT-NM (5A26-17)	28.201	0.082	Kuiper et al (2008)	9.99585	0.103	0.00157239	0.103	302.779	0.055	0.9939821	0.060	1	4.8E-14	15	SEP	2017	10	58	1
17D30559	1.9 %	PS59-252-1	Groundmass	Gakkel Ridge	FCT-NM (5A26-17)	28.201	0.082	Kuiper et al (2008)	9.99585	0.103	0.00157239	0.103	302.779	0.055	0.9939821	0.060	1	4.8E-14	15	SEP	2017	11	18	1
17D30560	2.0 %	PS59-252-1	Groundmass	Gakkel Ridge	FCT-NM (5A26-17)	28.201	0.082	Kuiper et al (2008)	9.99585	0.103	0.00157239	0.103	302.779	0.055	0.9939821	0.060	1	4.8E-14	15	SEP	2017	11	28	1
17D30562	2.2 %	PS59-252-1	Groundmass	Gakkel Ridge	FCT-NM (5A26-17)	28.201	0.082	Kuiper et al (2008)	9.99585	0.103	0.00157239	0.103	302.779	0.055	0.9939821	0.060	1	4.8E-14	15	SEP	2017	11	48	1
17D30563	2.4 %	PS59-252-1	Groundmass	Gakkel Ridge	FCT-NM (5A26-17)	28.201	0.082	Kuiper et al (2008)	9.99585	0.103	0.00157239	0.103	302.779	0.055	0.9939821	0.060	1	4.8E-14	15	SEP	2017	11	58	1
17D30565	2.7 %	PS59-252-1	Groundmass	Gakkel Ridge	FCT-NM (5A26-17)	28.201	0.082	Kuiper et al (2008)	9.99585	0.103	0.00157239	0.103	302.779	0.055	0.9939821	0.060	1	4.8E-14	15	SEP	2017	12	18	1
17D30566	3.0 %	PS59-252-1	Groundmass	Gakkel Ridge	FCT-NM (5A26-17)	28.201	0.082	Kuiper et al (2008)	9.99585	0.103	0.00157239	0.103	302.779	0.055	0.9939821	0.060	1	4.8E-14	15	SEP	2017	12	28	1
17D30568	3.4 %	PS59-252-1	Groundmass	Gakkel Ridge	FCT-NM (5A26-17)	28.201	0.082	Kuiper et al (2008)	9.99585	0.103	0.00157239	0.103	302.779	0.055	0.9939821	0.060	1	4.8E-14	15	SEP	2017	12	48	1
17D30569	3.9 %	PS59-252-1	Groundmass	Gakkel Ridge	FCT-NM (5A26-17)	28.201	0.082	Kuiper et al (2008)	9.99585	0.103	0.00157239	0.103	302.779	0.055	0.9939821	0.060	1	4.8E-14	15	SEP	2017	12	58	1
17D30571	4.5 %	PS59-252-1	Groundmass	Gakkel Ridge	FCT-NM (5A26-17)	28.201	0.082	Kuiper et al (2008)	9.99585	0.103	0.00157239	0.103	302.779	0.055	0.9939821	0.060	1	4.8E-14	15	SEP	2017	13	18	1
17D30572	5.2 %	PS59-252-1	Groundmass	Gakkel Ridge	FCT-NM (5A26-17)	28.201	0.082	Kuiper et al (2008)	9.99585	0.103	0.00157239	0.103	302.779	0.055	0.9939821	0.060	1	4.8E-14	15	SEP	2017	13	28	1
17D30574	6.0 %	PS59-252-1	Groundmass	Gakkel Ridge	FCT-NM (5A26-17)	28.201	0.082	Kuiper et al (2008)	9.99585	0.103	0.00157239	0.103	302.779	0.055	0.9939821	0.060	1	4.8E-14	15	SEP	2017	13	49	1
17D30575	6.9 %	PS59-252-1	Groundmass	Gakkel Ridge	FCT-NM (5A26-17)	28.201	0.082	Kuiper et al (2008)	9.99585	0.103	0.00157239	0.103	302.779	0.055	0.9939821	0.060	1	4.8E-14	15	SEP	2017	13	59	1
17D30577	7.9 %	PS59-252-1	Groundmass	Gakkel Ridge	FCT-NM (5A26-17)	28.201	0.082	Kuiper et al (2008)	9.99585	0.103	0.00157239	0.103	302.779	0.055	0.9939821	0.060	1	4.8E-14	15	SEP	2017	14	19	1
17D30578	9.0 %	PS59-252-1	Groundmass	Gakkel Ridge	FCT-NM (5A26-17)	28.201	0.082	Kuiper et al (2008)	9.99585	0.103	0.00157239	0.103	302.779	0.055	0.9939821	0.060	1	4.8E-14	15	SEP	2017	14	29	1
17D30580	10.3 %	PS59-252-1	Groundmass	Gakkel Ridge	FCT-NM (5A26-17)	28.201	0.082	Kuiper et al (2008)	9.99585	0.103	0.00157239	0.103	302.779	0.055	0.9939821	0.060	1	4.8E-14	15	SEP	2017	14	49	1
17D30581	11.6 %	PS59-252-1	Groundmass	Gakkel Ridge	FCT-NM (5A26-17)	28.201	0.082	Kuiper et al (2008)	9.99585	0.103	0.00157239	0.103	302.779	0.055	0.9939821	0.060	1	4.8E-14	15	SEP	2017	14	59	1
17D30583	12.5 %	PS59-252-1	Groundmass	Gakkel Ridge	FCT-NM (5A26-17)	28.201	0.082	Kuiper et al (2008)	9.99585	0.103	0.00157239	0.103	302.779	0.055	0.9939821	0.060	1	4.8E-14	15	SEP	2017	15	19	1
17D30584	13.4 %	PS59-252-1	Groundmass	Gakkel Ridge	FCT-NM (5A26-17)	28.201	0.082	Kuiper et al (2008)	9.99585	0.103	0.00157239	0.103	302.779	0.055	0.9939821	0.060	1	4.8E-14	15	SEP	2017	15	29	1
17D30586	14.6 %	PS59-252-1	Groundmass	Gakkel Ridge	FCT-NM (5A26-17)	28.201	0.082	Kuiper et al (2008)	9.99585	0.103	0.00157239	0.103	302.779	0.055	0.9939821	0.060	1	4.8E-14	15	SEP	2017	15	49	1
17D30587	16.0 %	PS59-252-1	Groundmass	Gakkel Ridge	FCT-NM (5A26-17)	28.201	0.082	Kuiper et al (2008)	9.99585	0.103	0.00157239	0.103	302.779	0.055	0.9939821	0.060	1	4.8E-14	15	SEP	2017	15	59	1
17D30589	17.6 %	PS59-252-1	Groundmass	Gakkel Ridge	FCT-NM (5A26-17)	28.201	0.082	Kuiper et al (2008)	9.99585	0.103	0.00157239	0.103	302.779	0.055	0.9939821	0.060	1	4.8E-14	15	SEP	2017	16	19	1
17D30590	19.3 %	PS59-252-1	Groundmass	Gakkel Ridge	FCT-NM (5A26-17)	28.201	0.082	Kuiper et al (2008)	9.99585	0.103	0.00157239	0.103	302.779	0.055	0.9939821	0.060	1	4.8E-14	15	SEP	2017	16	29	1
17D30592	21.0 %	PS59-252-1	Groundmass	Gakkel Ridge	FCT-NM (5A26-17)	28.201	0.082	Kuiper et al (2008)	9.99585	0.103	0.00157239	0.103	302.779	0.055	0.9939821	0.060	1	4.8E-14	15	SEP	2017	16	49	1

**Irradiation Constants**		**40/36(a)**	**%1σ**	**40/36(c)**	**%1σ**	**38/36(a)**	**%1σ**	**38/36(c)**	**%1σ**	**39/37(ca)**	**%1σ**	**38/37(ca)**	**%1σ**	**36/37(ca)**	**%1σ**	**40/39(k)**	**%1σ**	**38/39(k)**	**%1σ**	**36/38(cl)**	**%1σ**	**K/Ca**	**%1σ**	**K/Cl**	**%1σ**	**Ca/Cl**	**%1σ**
17D30557	1.8 %	295.5	0.237	0.018	35	0.1869	0	1.493	3	0.000643	0.92	0.00018	9.63	0.00027	0.17	0.000607	9.65	0.012077	0.09	0	0	0.43	0	0	0	0	0
17D30559	1.9 %	295.5	0.237	0.018	35	0.1869	0	1.493	3	0.000643	0.92	0.00018	9.63	0.00027	0.17	0.000607	9.65	0.012077	0.09	0	0	0.43	0	0	0	0	0
17D30560	2.0 %	295.5	0.237	0.018	35	0.1869	0	1.493	3	0.000643	0.92	0.00018	9.63	0.00027	0.17	0.000607	9.65	0.012077	0.09	0	0	0.43	0	0	0	0	0
17D30562	2.2 %	295.5	0.237	0.018	35	0.1869	0	1.493	3	0.000643	0.92	0.00018	9.63	0.00027	0.17	0.000607	9.65	0.012077	0.09	0	0	0.43	0	0	0	0	0
17D30563	2.4 %	295.5	0.237	0.018	35	0.1869	0	1.493	3	0.000643	0.92	0.00018	9.63	0.00027	0.17	0.000607	9.65	0.012077	0.09	0	0	0.43	0	0	0	0	0
17D30565	2.7 %	295.5	0.237	0.018	35	0.1869	0	1.493	3	0.000643	0.92	0.00018	9.63	0.00027	0.17	0.000607	9.65	0.012077	0.09	0	0	0.43	0	0	0	0	0
17D30566	3.0 %	295.5	0.237	0.018	35	0.1869	0	1.493	3	0.000643	0.92	0.00018	9.63	0.00027	0.17	0.000607	9.65	0.012077	0.09	0	0	0.43	0	0	0	0	0
17D30568	3.4 %	295.5	0.237	0.018	35	0.1869	0	1.493	3	0.000643	0.92	0.00018	9.63	0.00027	0.17	0.000607	9.65	0.012077	0.09	0	0	0.43	0	0	0	0	0
17D30569	3.9 %	295.5	0.237	0.018	35	0.1869	0	1.493	3	0.000643	0.92	0.00018	9.63	0.00027	0.17	0.000607	9.65	0.012077	0.09	0	0	0.43	0	0	0	0	0
17D30571	4.5 %	295.5	0.237	0.018	35	0.1869	0	1.493	3	0.000643	0.92	0.00018	9.63	0.00027	0.17	0.000607	9.65	0.012077	0.09	0	0	0.43	0	0	0	0	0
17D30572	5.2 %	295.5	0.237	0.018	35	0.1869	0	1.493	3	0.000643	0.92	0.00018	9.63	0.00027	0.17	0.000607	9.65	0.012077	0.09	0	0	0.43	0	0	0	0	0
17D30574	6.0 %	295.5	0.237	0.018	35	0.1869	0	1.493	3	0.000643	0.92	0.00018	9.63	0.00027	0.17	0.000607	9.65	0.012077	0.09	0	0	0.43	0	0	0	0	0
17D30575	6.9 %	295.5	0.237	0.018	35	0.1869	0	1.493	3	0.000643	0.92	0.00018	9.63	0.00027	0.17	0.000607	9.65	0.012077	0.09	0	0	0.43	0	0	0	0	0
17D30577	7.9 %	295.5	0.237	0.018	35	0.1869	0	1.493	3	0.000643	0.92	0.00018	9.63	0.00027	0.17	0.000607	9.65	0.012077	0.09	0	0	0.43	0	0	0	0	0
17D30578	9.0 %	295.5	0.237	0.018	35	0.1869	0	1.493	3	0.000643	0.92	0.00018	9.63	0.00027	0.17	0.000607	9.65	0.012077	0.09	0	0	0.43	0	0	0	0	0
17D30580	10.3 %	295.5	0.237	0.018	35	0.1869	0	1.493	3	0.000643	0.92	0.00018	9.63	0.00027	0.17	0.000607	9.65	0.012077	0.09	0	0	0.43	0	0	0	0	0
17D30581	11.6 %	295.5	0.237	0.018	35	0.1869	0	1.493	3	0.000643	0.92	0.00018	9.63	0.00027	0.17	0.000607	9.65	0.012077	0.09	0	0	0.43	0	0	0	0	0
17D30583	12.5 %	295.5	0.237	0.018	35	0.1869	0	1.493	3	0.000643	0.92	0.00018	9.63	0.00027	0.17	0.000607	9.65	0.012077	0.09	0	0	0.43	0	0	0	0	0
17D30584	13.4 %	295.5	0.237	0.018	35	0.1869	0	1.493	3	0.000643	0.92	0.00018	9.63	0.00027	0.17	0.000607	9.65	0.012077	0.09	0	0	0.43	0	0	0	0	0
17D30586	14.6 %	295.5	0.237	0.018	35	0.1869	0	1.493	3	0.000643	0.92	0.00018	9.63	0.00027	0.17	0.000607	9.65	0.012077	0.09	0	0	0.43	0	0	0	0	0
17D30587	16.0 %	295.5	0.237	0.018	35	0.1869	0	1.493	3	0.000643	0.92	0.00018	9.63	0.00027	0.17	0.000607	9.65	0.012077	0.09	0	0	0.43	0	0	0	0	0
17D30589	17.6 %	295.5	0.237	0.018	35	0.1869	0	1.493	3	0.000643	0.92	0.00018	9.63	0.00027	0.17	0.000607	9.65	0.012077	0.09	0	0	0.43	0	0	0	0	0
17D30590	19.3 %	295.5	0	0.018	35	0.1869	0	1.493	3	0.000643	0.92	0.00018	9.63	0.00027	0.17	0.000607	9.65	0.012077	0.09	0	0	0.43	0	0	0	0	0
17D30592	21.0 %	295.5	0	0.018	35	0.1869	0	1.493	3	0.000643	0.92	0.00018	9.63	0.00027	0.17	0.000607	9.65	0.012077	0.09	0	0	0.43	0	0	0	0	0

17D30553.AGE >>> PS59-252-1 >>> ARCTIC | O-CONNOR (16-22) PROJECT

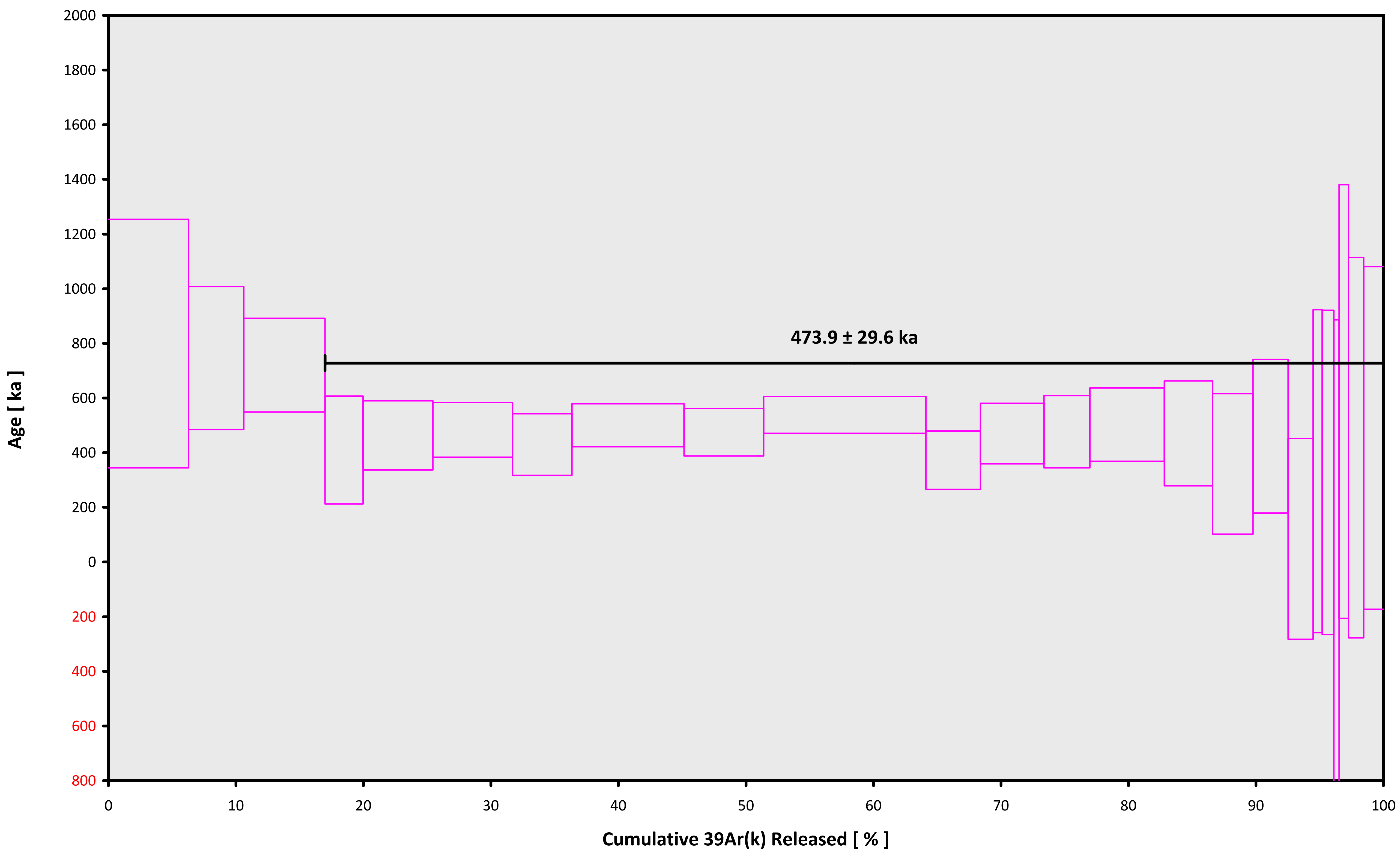

Ar-Ages in ka

WEIGHTED PLATEAU
473.9 ± 29.6
TOTAL FUSION
509.6 ± 44.6
NORMAL ISOCHRON
510.7 ± 44.3
INVERSE ISOCHRON
511.2 ± 43.6

MSWD (PROBABILITY)
0.80 (72%)

Sample Info

Groundmass
Gakkel Ridge
Dan Miggins

IRR = 17-OSU-05 (5A26-17)
J = 0.00157239 ± 0.00000162

17D30553.AGE >>> PS59-252-1 >>> ARCTIC | O-CONNOR (16-22) PROJECT

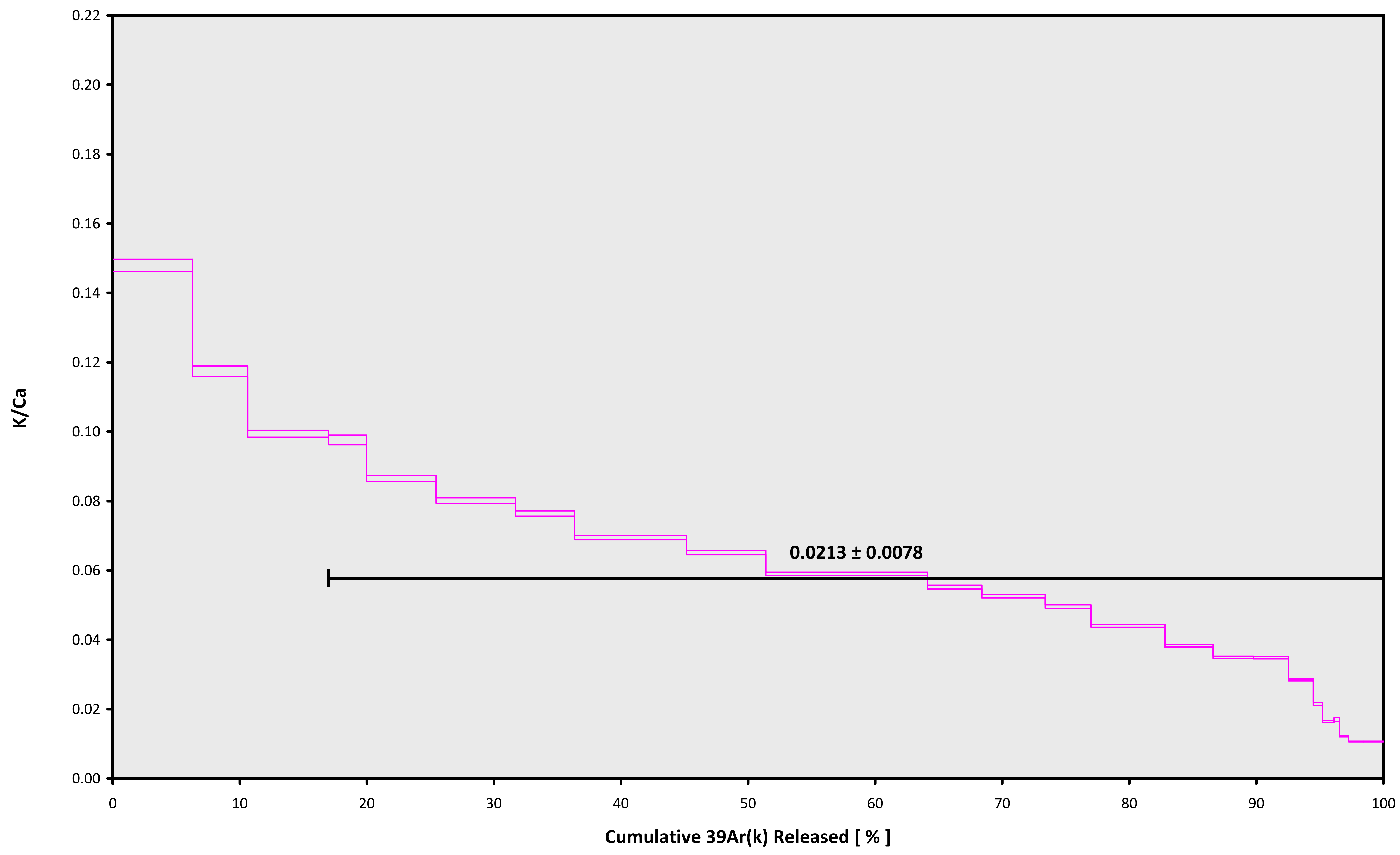

Ar-Ages in ka

WEIGHTED PLATEAU
473.9 ± 29.6
TOTAL FUSION
509.6 ± 44.6
NORMAL ISOCHRON
510.7 ± 44.3
INVERSE ISOCHRON
511.2 ± 43.6

Sample Info

Groundmass
Gakkel Ridge
Dan Miggins

IRR = 17-OSU-05 (5A26-17)
J = 0.00157239 ± 0.00000162

17D30553.AGE >>> PS59-252-1 >>> ARCTIC | O-CONNOR (16-22) PROJECT

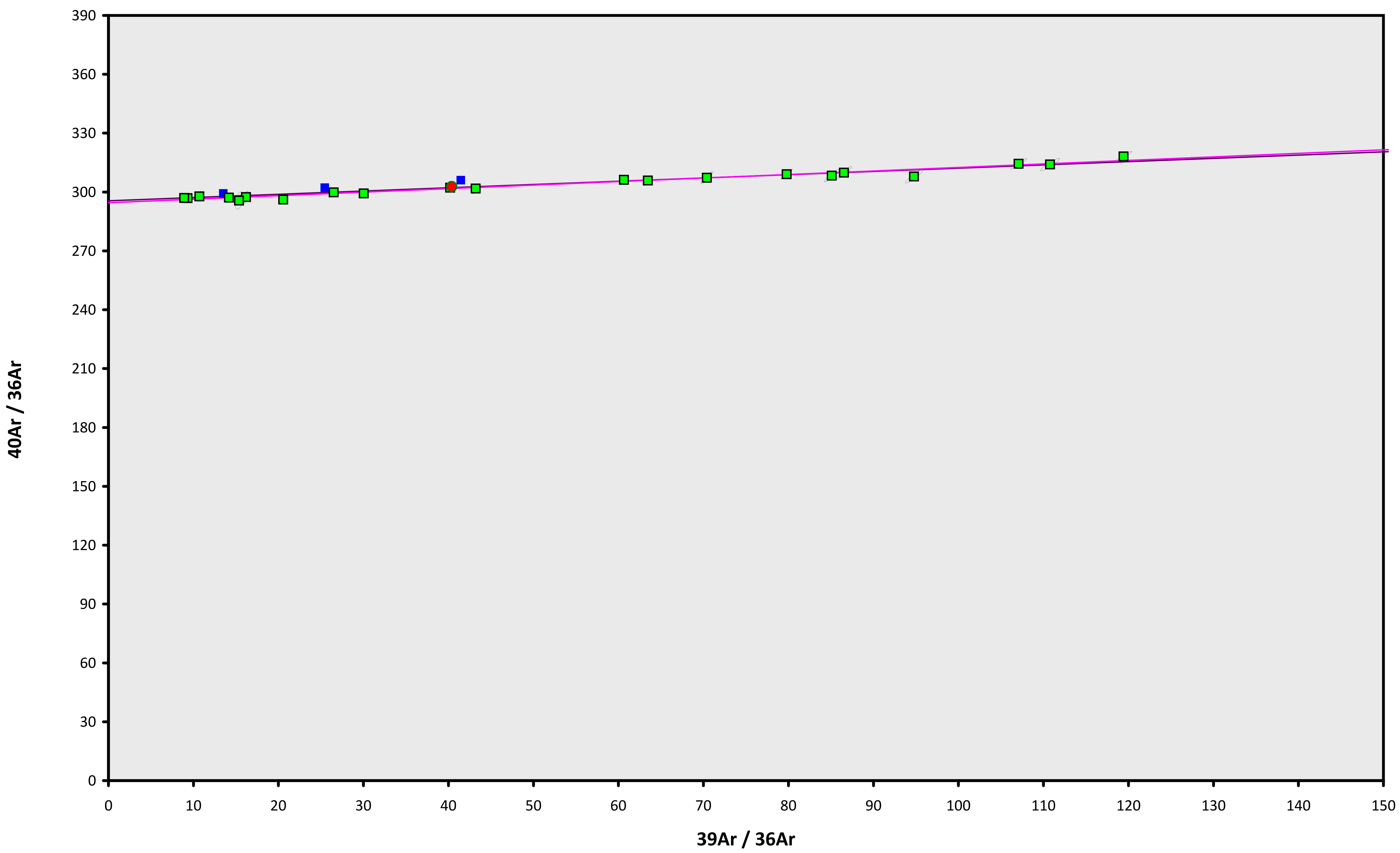

Ar-Ages in ka

WEIGHTED PLATEAU
473.9 ± 29.6
TOTAL FUSION
509.6 ± 44.6
NORMAL ISOCHRON
510.7 ± 44.3
INVERSE ISOCHRON
511.2 ± 43.6

MSWD (PROBABILITY)
0.87 (62%)

40AR/36AR INTERCEPT
294.5 ± 1.0

Sample Info

Groundmass
Gakkel Ridge
Dan Miggins

IRR = 17-OSU-05 (5A26-17)
J = 0.00157239 ± 0.00000162

17D30553.AGE >>> PS59-252-1 >>> ARCTIC | O-CONNOR (16-22) PROJECT

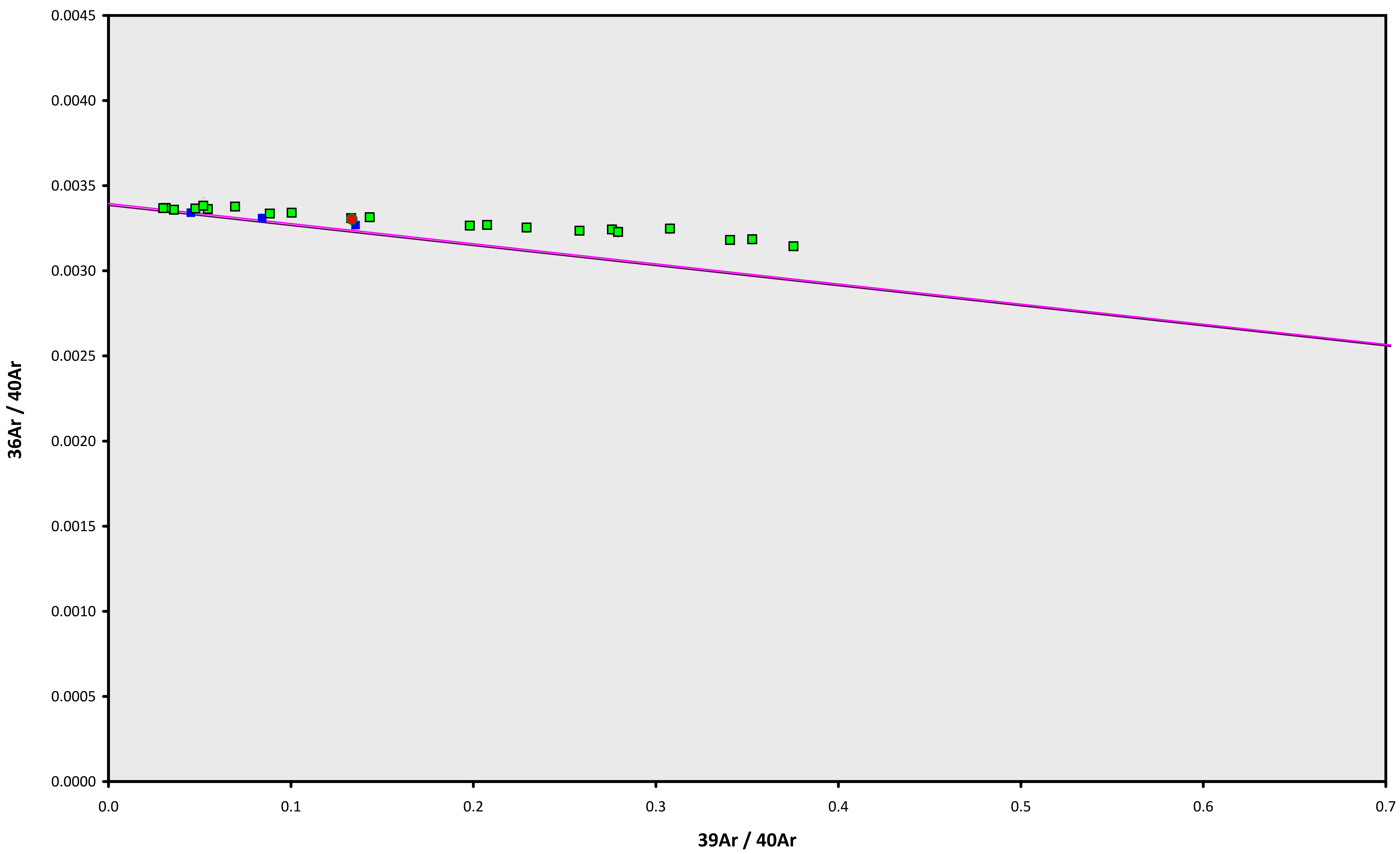

Ar-Ages in ka

WEIGHTED PLATEAU
473.9 ± 29.6
TOTAL FUSION
509.6 ± 44.6
NORMAL ISOCHRON
510.7 ± 44.3
INVERSE ISOCHRON
511.2 ± 43.6

MSWD (PROBABILITY)
0.87 (63%)

SPREADING FACTOR
6.2%

40AR/36AR INTERCEPT
294.5 ± 1.0

Sample Info

Groundmass
Gakkel Ridge
Dan Miggins

IRR = 17-OSU-05 (5A26-17)
J = 0.00157239 ± 0.00000162

Relative Abundances			36Ar [fA]	%1σ	37Ar [fA]	%1σ	38Ar [fA]	%1σ	39Ar [fA]	%1σ	40Ar [fA]	%1σ	40(r)/39(k) ± 2σ	Age ± 2σ (Ma)	40Ar(r) (%)	39Ar(k) (%)	K/Ca ± 2σ
17D19685	1.8 %	✓	0.910763	0.305	74.1757	0.718	0.9371080	2.466	54.33135	0.077	295.0441	0.032	0.58603 ± 0.03054	1.74 ± 0.09	10.78	14.27	0.3147 ± 0.0045
17D19687	1.9 %	✓	0.812022	0.321	137.3312	0.549	0.9659953	2.501	57.13972	0.077	262.9823	0.037	0.59545 ± 0.02730	1.77 ± 0.08	12.92	15.00	0.1786 ± 0.0020
17D19688	2.0 %	✓	0.164170	0.517	25.9798	1.686	0.2186142	9.900	11.87607	0.204	53.3152	0.172	0.57961 ± 0.04551	1.72 ± 0.14	12.89	3.12	0.1963 ± 0.0067
17D19690	2.2 %	✓	0.317301	0.372	75.9287	0.713	0.3660989	6.083	20.74557	0.132	99.5456	0.096	0.57199 ± 0.03519	1.70 ± 0.10	11.89	5.44	0.1172 ± 0.0017
17D19691	2.4 %	✓	0.157781	0.534	42.4350	1.055	0.1971601	12.338	10.92865	0.224	49.4178	0.186	0.56677 ± 0.04922	1.68 ± 0.15	12.50	2.87	0.1105 ± 0.0024
17D19693	2.7 %	✓	0.406871	0.376	173.0104	0.513	0.4779189	5.007	24.92962	0.115	120.2866	0.083	0.55867 ± 0.03777	1.66 ± 0.11	11.53	6.53	0.0617 ± 0.0006
17D19694	3.0 %	✓	0.195972	0.486	86.3326	0.660	0.2157720	11.140	11.58218	0.208	57.1087	0.162	0.52830 ± 0.05207	1.57 ± 0.15	10.66	3.03	0.0574 ± 0.0008
17D19696	3.4 %	✓	0.424020	0.372	270.3436	0.472	0.4017729	5.956	23.34600	0.123	116.5855	0.084	0.55529 ± 0.04213	1.65 ± 0.13	11.04	6.09	0.0369 ± 0.0004
17D19697	3.9 %	✓	0.400263	0.380	326.0511	0.463	0.3817281	6.216	21.18971	0.132	103.4903	0.091	0.53591 ± 0.04547	1.59 ± 0.14	10.86	5.52	0.0277 ± 0.0003
17D19699	4.5 %	✓	0.189457	0.486	165.9784	0.515	0.1855750	12.637	9.43373	0.246	47.4060	0.193	0.50114 ± 0.06353	1.49 ± 0.19	9.86	2.45	0.0242 ± 0.0003
17D19700	5.2 %	✓	0.391965	0.366	394.4565	0.457	0.3239365	7.181	18.82256	0.137	93.9704	0.099	0.51914 ± 0.04966	1.54 ± 0.15	10.26	4.88	0.0202 ± 0.0002
17D19702	6.0 %	✓	0.137842	0.559	129.2383	0.545	0.0992324	23.248	5.82793	0.398	33.3793	0.274	0.51632 ± 0.08795	1.53 ± 0.26	8.89	1.51	0.0191 ± 0.0003
17D19703	6.9 %	✓	0.451348	0.355	436.7280	0.455	0.3314683	7.003	18.94007	0.135	108.8120	0.086	0.55256 ± 0.05485	1.64 ± 0.16	9.48	4.91	0.0184 ± 0.0002
17D19705	7.9 %		0.128659	0.563	115.2115	0.584	0.0989653	23.547	4.76082	0.472	30.7092	0.298	0.40329 ± 0.10231	1.20 ± 0.30	6.16	1.23	0.0175 ± 0.0003
17D19706	9.0 %		0.404278	0.355	265.1827	0.472	0.2377016	9.572	12.94143	0.183	103.5397	0.090	0.41100 ± 0.07004	1.22 ± 0.21	5.07	3.36	0.0207 ± 0.0002
17D19708	10.3 %		0.210882	0.454	108.7268	0.589	0.1347132	18.004	5.75202	0.398	54.7030	0.169	0.18816 ± 0.10649	0.56 ± 0.32	1.95	1.49	0.0225 ± 0.0003
17D19709	11.6 %		1.464607	0.294	455.4462	0.456	0.6761675	3.430	25.04730	0.112	402.8806	0.024	0.26078 ± 0.10420	0.78 ± 0.31	1.60	6.51	0.0234 ± 0.0002
17D19711	12.5 %		0.617663	0.328	244.2303	0.479	0.2979623	8.116	10.33584	0.227	165.0912	0.056	0.20385 ± 0.12054	0.61 ± 0.36	1.26	2.68	0.0179 ± 0.0002
17D19712	13.4 %		0.707870	0.328	360.1525	0.461	0.3226991	7.164	11.30327	0.207	185.2637	0.051	0.43793 ± 0.12767	1.30 ± 0.38	2.62	2.91	0.0132 ± 0.0001
17D19714	14.6 %		0.490193	0.356	315.4252	0.466	0.2413412	9.198	8.24949	0.270	122.1854	0.076	0.31360 ± 0.13383	0.93 ± 0.40	2.07	2.12	0.0110 ± 0.0001
17D19715	16.0 %		0.629785	0.336	534.2349	0.452	0.3128390	7.470	9.68134	0.240	147.9899	0.063	0.48777 ± 0.14246	1.45 ± 0.42	3.08	2.46	0.0075 ± 0.0001
17D19717	17.6 %		0.208463	0.448	208.2263	0.492	0.1096309	21.143	3.38332	0.654	46.5130	0.198	0.47463 ± 0.18683	1.41 ± 0.56	3.32	0.85	0.0067 ± 0.0001
17D19718	19.3 %		0.171676	0.522	173.5988	0.506	0.1187028	20.163	2.65188	0.888	37.6811	0.244	0.32187 ± 0.22826	0.96 ± 0.68	2.17	0.67	0.0063 ± 0.0001
17D19720	21.0 %		0.036166	1.338	30.7199	1.465	0.0288793	81.564	0.42013	5.201	8.4055	1.083	0.43159 ± 0.86705	1.28 ± 2.58	2.06	0.11	0.0056 ± 0.0006
		Σ	10.030018	0.085	5149.1442	0.116	7.6819829	1.492	383.62001	0.034	2746.3059	0.017					

Information on Analysis and Constants Used in Calculations

Project = **O-CONNOR (16-22)**
Sample = **PS59-312-11**
Material = **Groundmass**
Location = **Gakkel Ridge**
Region = **Artic Ocean**
Analyst = **Dan Miggins**
Irradiation = **17-OSU-01 (1C14-17)**
Position = **X: 0 | Y: 0 | Z/H: 22.04658 mm**
FCT-NM Age = **28.201 ± 0.023 Ma**
FCT-NM Reference = **Kuiper et al (2008)**
FCT-NM 40Ar/39Ar Ratio = **9.55759 ± 0.00851**
FCT-NM J-value = **0.00164449 ± 0.00000146**
Air Shot 40Ar/36Ar = **302.5400 ± 0.3328**
Air Shot MDF = **0.99417507 ± 0.00063883 (LIN)**
Experiment Type = **Incremental Heating**
Extraction Method = **Bulk Laser Heating**
Heating = **64 sec**
Isolation = **3.00 min**
Instrument = **ARGUS-VI-D**
Preferred Age = **Plateau Age**
Age Classification = **Crystallization Age**
IGSN = Undefined
Rock Class = **Igneous>Volcanic**
Lithology = **Basaltic Lava**
Lat-Lon = Undefined - Undefined

Age Equations = **Min et al. (2000)**
Negative Intensities = **Allowed**
Collector Calibrations = **36Ar**
Decay 40K = **5.530 ± 0.048 E-10 1/a**
Decay 39Ar = **2.940 ± 0.016 E-07 1/h**
Decay 37Ar = **8.230 ± 0.012 E-04 1/h**
Decay 36Cl = **2.257 ± 0.015 E-06 1/a**
Decay 40K(EC,β⁺) = **0.580 ± 0.009 E-10 1/a**
Decay 40K(β⁻) = **4.950 ± 0.043 E-10 1/a**
Atmospheric 40/36(a) = **295.50**
Atmospheric 38/36(a) = **0.1869**
Production 39/37(ca) = **0.0006425 ± 0.0000059**
Production 38/37(ca) = **0.0001800 ± 0.0000173**
Production 36/37(ca) = **0.0002703 ± 0.0000005**
Production 40/39(k) = **0.000607 ± 0.000059**
Production 38/39(k) = **0.012077 ± 0.000011**
Production 36/38(cl) = **262.80 ± 1.71**
Scaling Ratio K/Ca = **0.430**
Abundance Ratio 40K/K = **1.1700 ± 0.0100 E-04**
Atomic Weight K = **39.0983 ± 0.0001 g**

Results

Results	40(a)/36(a) ± 2σ		40(r)/39(k) ± 2σ		Age ± 2σ (Ma)		MSWD	39Ar(k) (%,n)	K/Ca ± 2σ
**Age Plateau**			0.56425	± 0.01516<br>± 2.69%	**1.68**	**± 0.05<br>± 2.69%**	1.69<br>6%	75.62<br>13	0.0244 ± 0.0083
				Full External Error	± 0.06		1.82	2σ Confidence Limit	
				Analytical Error	± 0.05		1.2995	Error Magnification	
**Total Fusion Age**			0.50887	± 0.01371<br>± 2.69%	**1.51**	**± 0.04<br>± 2.70%**		24	0.0318 ± 0.0001
				Full External Error	± 0.05				
				Analytical Error	± 0.04				
**Normal Isochron**	**289.29**	**± 11.85<br>± 4.10%**	0.65613	± 0.17659<br>± 26.91%	**1.95**	**± 0.52<br>± 26.90%**	1.69<br>7%	75.62<br>13	
				Full External Error	± 0.53		1.85	2σ Confidence Limit	
				Analytical Error	± 0.52		1.3009	Error Magnification	
							6	Number of Iterations	
							0.0000032012	Convergence	
**Inverse Isochron**<br>Clustered Points	**289.38**	**± 11.82<br>± 4.08%**	0.65517	± 0.15692<br>± 23.95%	**1.95**	**± 0.47<br>± 23.94%**	1.68<br>7%	75.62<br>13	
				Full External Error	± 0.47		1.85	2σ Confidence Limit	
				Analytical Error	± 0.47		1.2971	Error Magnification	
**Notes**							4	Number of Iterations	
Nice plateau							0.0000617151	Convergence	
							3%	Spreading Factor	

Incremental Heating			36Ar(a) [fA]	37Ar(ca) [fA]	38Ar(cl) [fA]	39Ar(k) [fA]	40Ar(r) [fA]	Age ± 2σ (Ma)	40Ar(r) (%)	39Ar(k) (%)	K/Ca ± 2σ
17D19685	1.8 %	✓	0.8906904	74.1757	0.1017022	54.28370	31.81210	1.74 ± 0.09	10.78	14.27	0.3147 ± 0.0045
17D19687	1.9 %	✓	0.7748771	137.3312	0.1074403	57.05149	33.97145	1.77 ± 0.08	12.92	15.00	0.1786 ± 0.0020
17D19688	2.0 %	✓	0.1571378	25.9798	0.0413430	11.85938	6.87376	1.72 ± 0.14	12.89	3.12	0.1963 ± 0.0067
17D19690	2.2 %	✓	0.2967667	75.9287	0.0470110	20.69678	11.83844	1.70 ± 0.10	11.89	5.44	0.1172 ± 0.0017
17D19691	2.4 %	✓	0.1463034	42.4350	0.0305216	10.90138	6.17855	1.68 ± 0.15	12.50	2.87	0.1105 ± 0.0024
17D19693	2.7 %	✓	0.3600885	173.0104	0.0797440	24.81846	13.86536	1.66 ± 0.11	11.53	6.53	0.0617 ± 0.0006
17D19694	3.0 %	✓	0.1726299	86.3326	0.0287595	11.52671	6.08955	1.57 ± 0.15	10.66	3.03	0.0574 ± 0.0008
17D19696	3.4 %	✓	0.3509443	270.3436	0.0076677	23.17230	12.86742	1.65 ± 0.13	11.04	6.09	0.0369 ± 0.0004
17D19697	3.9 %	✓	0.3121285	326.0511	0.0113239	20.98023	11.24357	1.59 ± 0.14	10.86	5.52	0.0277 ± 0.0003
17D19699	4.5 %	✓	0.1445895	165.9784	0.0160319	9.32709	4.67413	1.49 ± 0.19	9.86	2.45	0.0242 ± 0.0003
17D19700	5.2 %	✓	0.2853439	394.4565	0.0000000	18.56913	9.64003	1.54 ± 0.15	10.26	4.88	0.0202 ± 0.0002
17D19702	6.0 %	✓	0.1029090	129.2383	0.0000000	5.74489	2.96622	1.53 ± 0.26	8.89	1.51	0.0191 ± 0.0003
17D19703	6.9 %	✓	0.3333002	436.7280	0.0000000	18.65947	10.31043	1.64 ± 0.16	9.48	4.91	0.0184 ± 0.0002
17D19705	7.9 %		0.0975167	115.2115	0.0033989	4.68680	1.89016	1.20 ± 0.30	6.16	1.23	0.0175 ± 0.0003
17D19706	9.0 %		0.3325992	265.1827	0.0000000	12.77105	5.24890	1.22 ± 0.21	5.07	3.36	0.0207 ± 0.0002
17D19708	10.3 %		0.1814904	108.7268	0.0125984	5.68216	1.06914	0.56 ± 0.32	1.95	1.49	0.0225 ± 0.0003
17D19709	11.6 %		1.3414893	455.4462	0.0445006	24.75468	6.45548	0.78 ± 0.31	1.60	6.51	0.0234 ± 0.0002
17D19711	12.5 %		0.5516414	244.2303	0.0279682	10.17892	2.07500	0.61 ± 0.36	1.26	2.68	0.0179 ± 0.0002
17D19712	13.4 %		0.6105188	360.1525	0.0100507	11.07187	4.84871	1.30 ± 0.38	2.62	2.91	0.0132 ± 0.0001
17D19714	14.6 %		0.4049307	315.4252	0.0117015	8.04683	2.52347	0.93 ± 0.40	2.07	2.12	0.0110 ± 0.0001
17D19715	16.0 %		0.4853786	534.2349	0.0131832	9.33810	4.55486	1.45 ± 0.42	3.08	2.46	0.0075 ± 0.0001
17D19717	17.6 %		0.1521782	208.2263	0.0044635	3.24953	1.54234	1.41 ± 0.56	3.32	0.85	0.0067 ± 0.0001
17D19718	19.3 %		0.1247443	173.5988	0.0334607	2.54034	0.81766	0.96 ± 0.68	2.17	0.67	0.0063 ± 0.0001
17D19720	21.0 %		0.0278595	30.7199	0.0133072	0.40039	0.17280	1.28 ± 2.58	2.06	0.11	0.0056 ± 0.0006
		Σ	8.6380561	5149.1442	0.6461781	380.31169	193.52953				

Information on Analysis

Project = **O-CONNOR (16-22)**
Sample = **PS59-312-11**
Material = **Groundmass**
Location = **Gakkel Ridge**
Region = **Artic Ocean**
Analyst = **Dan Miggins**
Irradiation = **17-OSU-01 (1C14-17)**
J = **0.00164449 ± 0.00000146**
FCT-NM = **28.201 ± 0.023 Ma**

Results	40(r)/39(k) ± 2σ		Age ± 2σ (Ma)		MSWD	39Ar(k) (%,n)	K/Ca ± 2σ
**Age Plateau**	0.56425	± 0.01516<br>± 2.69%	**1.68**	**± 0.05**<br>**± 2.69%**	1.69<br>6%	75.62<br>13	0.0244 ± 0.0083
			Full External Error	± 0.06	1.82	2σ Confidence Limit	
			Analytical Error	± 0.05	1.2995	Error Magnification	
**Total Fusion Age**	0.50887	± 0.01371<br>± 2.69%	**1.51**	**± 0.04**<br>**± 2.70%**		24	0.0318 ± 0.0001
			Full External Error	± 0.05			
			Analytical Error	± 0.04			

Normal Isochron			39(k)/36(a) ± 2σ	40(a+r)/36(a) ± 2σ	r.i.
17D19685	1.8 %	✓	60.95 ± 0.39	331.22 ± 2.08	0.9659
17D19687	1.9 %	✓	73.63 ± 0.51	339.34 ± 2.30	0.9687
17D19688	2.0 %	✓	75.47 ± 0.88	339.24 ± 3.88	0.8932
17D19690	2.2 %	✓	69.74 ± 0.59	335.39 ± 2.76	0.9237
17D19691	2.4 %	✓	74.51 ± 0.93	337.73 ± 4.13	0.8890
17D19693	2.7 %	✓	68.92 ± 0.61	334.01 ± 2.93	0.9484
17D19694	3.0 %	✓	66.77 ± 0.80	330.78 ± 3.85	0.8996
17D19696	3.4 %	✓	66.03 ± 0.63	332.17 ± 3.11	0.9500
17D19697	3.9 %	✓	67.22 ± 0.70	331.52 ± 3.41	0.9515
17D19699	4.5 %	✓	64.51 ± 0.91	327.83 ± 4.50	0.8977
17D19700	5.2 %	✓	65.08 ± 0.72	329.28 ± 3.59	0.9512
17D19702	6.0 %	✓	55.82 ± 0.97	324.32 ± 5.32	0.8353
17D19703	6.9 %	✓	55.98 ± 0.59	326.43 ± 3.38	0.9519
17D19705	7.9 %		48.06 ± 0.87	314.88 ± 5.19	0.7907
17D19706	9.0 %		38.40 ± 0.37	311.28 ± 2.83	0.9040
17D19708	10.3 %		31.31 ± 0.42	301.39 ± 3.39	0.7630
17D19709	11.6 %		18.45 ± 0.13	300.31 ± 1.95	0.9408
17D19711	12.5 %		18.45 ± 0.16	299.26 ± 2.25	0.8395
17D19712	13.4 %		18.14 ± 0.16	303.44 ± 2.38	0.8702
17D19714	14.6 %		19.87 ± 0.21	301.73 ± 2.71	0.8354
17D19715	16.0 %		19.24 ± 0.20	304.88 ± 2.83	0.8689
17D19717	17.6 %		21.35 ± 0.40	305.64 ± 4.11	0.6553
17D19718	19.3 %		20.36 ± 0.49	302.05 ± 4.74	0.5952
17D19720	21.0 %		14.37 ± 1.65	301.70 ± 12.63	0.2668

Results	40(a)/36(a) ± 2σ	40(r)/39(k) ± 2σ	Age ± 2σ (Ma)	MSWD
Normal Isochron	**289.29** **± 11.85** **± 4.10%**	0.65613 ± 0.17659 ± 26.91%	**1.95** **± 0.52** **± 26.90%**	1.69 7%
			Full External Error ± 0.53	
			Analytical Error ± 0.52	

Statistics				
2σ Confidence Limit	1.85	Convergence	0.000003201201
Error Magnification	1.3009	Number of Iterations	6
Number of Data Points	13	Calculated Line	Weighted York-2

**Inverse Isochron**			**39(k)/40(a+r) ± 2σ**	**36(a)/40(a+r) ± 2σ**	**r.i.**
17D19685	1.8 %	✓	0.1840056 ± 0.0003068	0.00301918 ± 0.00001898	0.0399
17D19687	1.9 %	✓	0.2169690 ± 0.0003730	0.00294689 ± 0.00002000	0.0474
17D19688	2.0 %	✓	0.2224692 ± 0.0011884	0.00294773 ± 0.00003374	0.1944
17D19690	2.2 %	✓	0.2079389 ± 0.0006780	0.00298159 ± 0.00002455	0.1363
17D19691	2.4 %	✓	0.2206258 ± 0.0012855	0.00296094 ± 0.00003620	0.1933
17D19693	2.7 %	✓	0.2063536 ± 0.0005862	0.00299396 ± 0.00002623	0.1104
17D19694	3.0 %	✓	0.2018629 ± 0.0010684	0.00302320 ± 0.00003520	0.1696
17D19696	3.4 %	✓	0.1987819 ± 0.0005960	0.00301055 ± 0.00002821	0.0994
17D19697	3.9 %	✓	0.2027515 ± 0.0006571	0.00301639 ± 0.00003106	0.0999
17D19699	4.5 %	✓	0.1967726 ± 0.0012397	0.00305039 ± 0.00004189	0.1730
17D19700	5.2 %	✓	0.1976298 ± 0.0006785	0.00303689 ± 0.00003307	0.1059
17D19702	6.0 %	✓	0.1721274 ± 0.0016812	0.00308334 ± 0.00005060	0.1877
17D19703	6.9 %	✓	0.1715015 ± 0.0005581	0.00306340 ± 0.00003172	0.0888
17D19705	7.9 %		0.1526330 ± 0.0017250	0.00317578 ± 0.00005234	0.1905
17D19706	9.0 %		0.1233537 ± 0.0005107	0.00321253 ± 0.00002917	0.0868
17D19708	10.3 %		0.1038795 ± 0.0009081	0.00331795 ± 0.00003736	0.1156
17D19709	11.6 %		0.0614465 ± 0.0001433	0.00332987 ± 0.00002166	0.0152
17D19711	12.5 %		0.0616587 ± 0.0002937	0.00334156 ± 0.00002514	0.0354
17D19712	13.4 %		0.0597649 ± 0.0002607	0.00329552 ± 0.00002581	0.0299
17D19714	14.6 %		0.0658602 ± 0.0003790	0.00331420 ± 0.00002981	0.0443
17D19715	16.0 %		0.0631020 ± 0.0003273	0.00327993 ± 0.00003040	0.0325
17D19717	17.6 %		0.0698660 ± 0.0009931	0.00327188 ± 0.00004401	0.0817
17D19718	19.3 %		0.0674195 ± 0.0012945	0.00331066 ± 0.00005195	0.0789
17D19720	21.0 %		0.0476356 ± 0.0053018	0.00331452 ± 0.00013878	0.1008

**Results**	**40(a)/36(a) ± 2σ**		**40(r)/39(k) ± 2σ**		**Age ± 2σ (Ma)**		**MSWD**
**Inverse Isochron** Clustered Points	**289.38**	**± 11.82** **± 4.08%**	0.65517	± 0.15692 ± 23.95%	**1.95**	**± 0.47** **± 23.94%**	1.68 7%
					Full External Error	± 0.47	
					Analytical Error	± 0.47	

**Statistics**				
2σ Confidence Limit	1.85	Convergence	0.0000617151
Error Magnification	1.2971	Number of Iterations	4
Number of Data Points	13	Calculated Line	Weighted York-2
Spreading Factor	3.3%		

Degassing Patterns			36Ar(a) [fA]	%1σ	36Ar(c) [fA]	%1σ	36Ar(ca) [fA]	%1σ	36Ar(cl) [fA]	%1σ	37Ar(ca) [fA]	%1σ	38Ar(a) [fA]	%1σ	38Ar(c) [fA]	%1σ	38Ar(k) [fA]	%1σ	38Ar(ca) [fA]	%1σ	38Ar(cl) [fA]	%1σ	39Ar(k) [fA]	%1σ	39Ar(ca) [fA]	%1σ	40Ar(r) [fA]	%1σ	40Ar(a) [fA]	%1σ	40Ar(c) [fA]	%1σ	40Ar(k) [fA]	%1σ
17D19685	1.8 %	✓	0.8906904	0.31	0.0000000	0.00	0.0200497	0.74	0.0000233	22.80	74.1757	0.72	0.1664700	0.31	0.0000000	0.00	0.6555842	0.12	0.0133516	9.66	0.1017022	22.82	54.28370	0.08	0.0476579	1.17	31.81210	2.60	263.1990	0.31	0.0000000	0.00	0.0329502	9.65
17D19687	1.9 %	✓	0.7748771	0.34	0.0000000	0.00	0.0371206	0.57	0.0000246	22.63	137.3312	0.55	0.1448245	0.34	0.0000000	0.00	0.6890108	0.12	0.0247196	9.65	0.1074403	22.65	57.05149	0.08	0.0882353	1.07	33.97145	2.29	228.9762	0.34	0.0000000	0.00	0.0346303	9.65
17D19688	2.0 %	✓	0.1571378	0.55	0.0000000	0.00	0.0070223	1.69	0.0000095	52.38	25.9798	1.69	0.0293690	0.55	0.0000000	0.00	0.1432258	0.22	0.0046764	9.78	0.0413430	52.39	11.85938	0.20	0.0166920	1.92	6.87376	3.92	46.4342	0.55	0.0000000	0.00	0.0071986	9.65
17D19690	2.2 %	✓	0.2967667	0.40	0.0000000	0.00	0.0205235	0.73	0.0000108	47.47	75.9287	0.71	0.0554657	0.40	0.0000000	0.00	0.2499550	0.16	0.0136672	9.66	0.0470110	47.48	20.69678	0.13	0.0487842	1.16	11.83844	3.07	87.6945	0.40	0.0000000	0.00	0.0125629	9.65
17D19691	2.4 %	✓	0.1463034	0.58	0.0000000	0.00	0.0114702	1.07	0.0000070	79.75	42.4350	1.06	0.0273441	0.58	0.0000000	0.00	0.1316560	0.24	0.0076383	9.69	0.0305216	79.76	10.90138	0.22	0.0272645	1.40	6.17855	4.34	43.2327	0.58	0.0000000	0.00	0.0066171	9.65
17D19693	2.7 %	✓	0.3600885	0.43	0.0000000	0.00	0.0467647	0.54	0.0000183	30.27	173.0104	0.51	0.0673005	0.43	0.0000000	0.00	0.2997325	0.15	0.0311419	9.64	0.0797440	30.28	24.81846	0.12	0.1111592	1.05	13.86536	3.38	106.4061	0.43	0.0000000	0.00	0.0150648	9.65
17D19694	3.0 %	✓	0.1726299	0.56	0.0000000	0.00	0.0233357	0.68	0.0000066	83.76	86.3326	0.66	0.0322645	0.56	0.0000000	0.00	0.1392081	0.23	0.0155399	9.65	0.0287595	83.77	11.52671	0.21	0.0554687	1.13	6.08955	4.92	51.0121	0.56	0.0000000	0.00	0.0069967	9.65
17D19696	3.4 %	✓	0.3509443	0.46	0.0000000	0.00	0.0730739	0.50	0.0000018	318.09	270.3436	0.47	0.0655915	0.46	0.0000000	0.00	0.2798519	0.15	0.0486619	9.64	0.0076677	318.10	23.17230	0.12	0.1736958	1.03	12.86742	3.79	103.7041	0.46	0.0000000	0.00	0.0140656	9.65
17D19697	3.9 %	✓	0.3121285	0.51	0.0000000	0.00	0.0881316	0.49	0.0000026	215.46	326.0511	0.46	0.0583368	0.51	0.0000000	0.00	0.2533782	0.16	0.0586892	9.64	0.0113239	215.46	20.98023	0.13	0.2094878	1.03	11.24357	4.24	92.2340	0.51	0.0000000	0.00	0.0127350	9.65
17D19699	4.5 %	✓	0.1445895	0.66	0.0000000	0.00	0.0448640	0.54	0.0000037	147.40	165.9784	0.52	0.0270238	0.66	0.0000000	0.00	0.1126432	0.26	0.0298761	9.64	0.0160319	147.41	9.32709	0.25	0.1066411	1.05	4.67413	6.33	42.7262	0.66	0.0000000	0.00	0.0056615	9.65
17D19700	5.2 %	✓	0.2853439	0.54	0.0000000	0.00	0.1066216	0.49	0.0000000	0.00	394.4565	0.46	0.0533308	0.54	0.0000000	0.00	0.2242593	0.17	0.0710022	9.64	0.0000000	0.00	18.56913	0.14	0.2534383	1.03	9.64003	4.78	84.3191	0.54	0.0000000	0.00	0.0112715	9.65
17D19702	6.0 %	✓	0.1029090	0.77	0.0000000	0.00	0.0349331	0.57	0.0000000	0.00	129.2383	0.54	0.0192337	0.77	0.0000000	0.00	0.0693811	0.41	0.0232629	9.65	0.0000000	0.00	5.74489	0.40	0.0830356	1.07	2.96622	8.51	30.4096	0.77	0.0000000	0.00	0.0034872	9.66
17D19703	6.9 %	✓	0.3333002	0.51	0.0000000	0.00	0.1180476	0.49	0.0000000	0.00	436.7280	0.45	0.0622938	0.51	0.0000000	0.00	0.2253504	0.16	0.0786110	9.64	0.0000000	0.00	18.65947	0.14	0.2805977	1.03	10.31043	4.96	98.4902	0.51	0.0000000	0.00	0.0113263	9.65
17D19705	7.9 %		0.0975167	0.77	0.0000000	0.00	0.0311417	0.61	0.0000008	688.22	115.2115	0.58	0.0182259	0.77	0.0000000	0.00	0.0566025	0.49	0.0207381	9.65	0.0033989	688.22	4.68680	0.48	0.0740234	1.09	1.89016	12.67	28.8162	0.77	0.0000000	0.00	0.0028449	9.66
17D19706	9.0 %		0.3325992	0.44	0.0000000	0.00	0.0716789	0.50	0.0000000	0.00	265.1827	0.47	0.0621628	0.44	0.0000000	0.00	0.1542359	0.21	0.0477329	9.64	0.0000000	0.00	12.77105	0.19	0.1703799	1.03	5.24890	8.52	98.2831	0.44	0.0000000	0.00	0.0077520	9.65
17D19708	10.3 %		0.1814904	0.54	0.0000000	0.00	0.0293888	0.61	0.0000029	193.13	108.7268	0.59	0.0339206	0.54	0.0000000	0.00	0.0686235	0.41	0.0195708	9.65	0.0125984	193.13	5.68216	0.40	0.0698569	1.09	1.06914	28.29	53.6304	0.54	0.0000000	0.00	0.0034491	9.66
17D19709	11.6 %		1.3414893	0.32	0.0000000	0.00	0.1231071	0.49	0.0000102	55.11	455.4462	0.46	0.2507244	0.32	0.0000000	0.00	0.2989622	0.15	0.0819803	9.64	0.0445006	55.12	24.75468	0.11	0.2926242	1.03	6.45548	19.98	396.4101	0.32	0.0000000	0.00	0.0150261	9.65
17D19711	12.5 %		0.5516414	0.37	0.0000000	0.00	0.0660154	0.51	0.0000064	87.80	244.2303	0.48	0.1031018	0.37	0.0000000	0.00	0.1229309	0.25	0.0439614	9.64	0.0279682	87.81	10.17892	0.23	0.1569179	1.04	2.07500	29.56	163.0100	0.37	0.0000000	0.00	0.0061786	9.65
17D19712	13.4 %		0.6105188	0.39	0.0000000	0.00	0.0973492	0.49	0.0000023	238.34	360.1525	0.46	0.1141060	0.39	0.0000000	0.00	0.1337150	0.23	0.0648275	9.64	0.0100507	238.34	11.07187	0.21	0.2313980	1.03	4.84871	14.58	180.4083	0.39	0.0000000	0.00	0.0067206	9.65
17D19714	14.6 %		0.4049307	0.44	0.0000000	0.00	0.0852594	0.50	0.0000027	195.43	315.4252	0.47	0.0756815	0.44	0.0000000	0.00	0.0971816	0.29	0.0567765	9.64	0.0117015	195.43	8.04683	0.28	0.2026607	1.03	2.52347	21.34	119.6570	0.44	0.0000000	0.00	0.0048844	9.65
17D19715	16.0 %		0.4853786	0.46	0.0000000	0.00	0.1444037	0.48	0.0000030	190.75	534.2349	0.45	0.0907173	0.46	0.0000000	0.00	0.1127762	0.27	0.0961623	9.64	0.0131832	190.75	9.33810	0.25	0.3432459	1.03	4.55486	14.60	143.4294	0.46	0.0000000	0.00	0.0056682	9.65
17D19717	17.6 %		0.1521782	0.64	0.0000000	0.00	0.0562836	0.52	0.0000010	525.66	208.2263	0.49	0.0284421	0.64	0.0000000	0.00	0.0392446	0.69	0.0374807	9.64	0.0044635	525.66	3.24953	0.68	0.1337854	1.04	1.54234	19.67	44.9686	0.64	0.0000000	0.00	0.0019725	9.67
17D19718	19.3 %		0.1247443	0.75	0.0000000	0.00	0.0469238	0.53	0.0000077	72.11	173.5988	0.51	0.0233147	0.75	0.0000000	0.00	0.0306797	0.93	0.0312478	9.64	0.0334607	72.11	2.54034	0.93	0.1115372	1.05	0.81766	35.45	36.8619	0.75	0.0000000	0.00	0.0015420	9.69
17D19720	21.0 %		0.0278595	1.79	0.0000000	0.00	0.0083036	1.47	0.0000031	177.08	30.7199	1.46	0.0052069	1.79	0.0000000	0.00	0.0048355	5.46	0.0055296	9.74	0.0133072	177.08	0.40039	5.46	0.0197375	1.73	0.17280	100.30	8.2325	1.79	0.0000000	0.00	0.0002430	11.09
		Σ	8.6380561	0.10	0.0000000	0.00	1.3918137	0.12	0.0001482	16.50	5149.1442	0.12	1.6144527	0.10	0.0000000	0.00	4.5930242	0.04	0.9268460	2.36	0.6461781	16.50	380.31169	0.03	3.3083251	0.25	193.52953	1.35	2552.5456	0.10	0.0000000	0.00	0.2308492	2.62
		Σ							10.030018	0.09	5149.1442	0.12									7.7805010	1.40			383.62001	0.03							2746.3059	0.13

**Additional Parameters**			**40Ar/39Ar**	**1σ**	**37Ar/39Ar**	**1σ**	**36Ar/39Ar**	**1σ**	**Time (days)**	**37Ar (decay)**	**39Ar (decay)**	**40Ar (moles)**
17D19685	1.8 %	✓	5.430457	0.004524	1.365247	0.009860	0.016763	0.000053	141.166	16.293696	1.00099745	1.416E-11
17D19687	1.9 %	✓	4.602442	0.003950	2.403427	0.013320	0.014211	0.000047	141.180	16.298166	1.00099754	1.262E-11
17D19688	2.0 %	✓	4.489292	0.011979	2.187573	0.037156	0.013824	0.000077	141.187	16.300402	1.00099759	2.559E-12
17D19690	2.2 %	✓	4.798401	0.007809	3.659997	0.026535	0.015295	0.000060	141.201	16.304874	1.00099769	4.778E-12
17D19691	2.4 %	✓	4.521860	0.013153	3.882910	0.041889	0.014437	0.000084	141.208	16.307111	1.00099774	2.372E-12
17D19693	2.7 %	✓	4.825047	0.006829	6.939952	0.036460	0.016321	0.000064	141.222	16.311585	1.00099784	5.774E-12
17D19694	3.0 %	✓	4.930737	0.013006	7.453912	0.051615	0.016920	0.000089	141.228	16.313823	1.00099789	2.741E-12
17D19696	3.4 %	✓	4.993812	0.007438	11.579870	0.056484	0.018162	0.000071	141.242	16.318299	1.00099799	5.596E-12
17D19697	3.9 %	✓	4.883986	0.007845	15.387232	0.074113	0.018889	0.000076	141.249	16.320537	1.00099803	4.968E-12
17D19699	4.5 %	✓	5.025159	0.015707	17.594146	0.100397	0.020083	0.000109	141.263	16.325015	1.00099813	2.275E-12
17D19700	5.2 %	✓	4.992434	0.008465	20.956577	0.100036	0.020824	0.000081	141.270	16.327255	1.00099818	4.511E-12
17D19702	6.0 %	✓	5.727474	0.027683	22.175681	0.149631	0.023652	0.000162	141.284	16.331734	1.00099828	1.602E-12
17D19703	6.9 %	✓	5.745067	0.009207	23.058414	0.109372	0.023830	0.000090	141.291	16.333975	1.00099833	5.223E-12
17D19705	7.9 %		6.450393	0.036023	24.199914	0.181763	0.027025	0.000199	141.305	16.338456	1.00099843	1.474E-12
17D19706	9.0 %		8.000641	0.016348	20.490994	0.103839	0.031239	0.000125	141.312	16.340697	1.00099848	4.970E-12
17D19708	10.3 %		9.510228	0.041118	18.902371	0.134328	0.036662	0.000221	141.326	16.345181	1.00099857	2.626E-12
17D19709	11.6 %		16.084791	0.018448	18.183443	0.085334	0.058474	0.000184	141.333	16.347423	1.00099862	1.934E-11
17D19711	12.5 %		15.972689	0.037410	23.629447	0.125201	0.059759	0.000238	141.347	16.351908	1.00099872	7.924E-12
17D19712	13.4 %		16.390277	0.034879	31.862685	0.160896	0.062625	0.000243	141.353	16.354151	1.00099877	8.893E-12
17D19714	14.6 %		14.811256	0.041472	38.235703	0.205839	0.059421	0.000265	141.368	16.358863	1.00099887	5.865E-12
17D19715	16.0 %		15.286094	0.037920	55.181907	0.282593	0.065051	0.000269	141.375	16.361107	1.00099892	7.104E-12
17D19717	17.6 %		13.747727	0.093976	61.544948	0.503798	0.061615	0.000489	141.389	16.365596	1.00099902	2.233E-12
17D19718	19.3 %		14.209238	0.130906	65.462657	0.669390	0.064737	0.000667	141.396	16.367841	1.00099907	1.809E-12
17D19720	21.0 %		20.007060	1.062984	73.120352	3.951254	0.086084	0.004623	141.410	16.372332	1.00099917	4.035E-13

Procedure Blanks		36Ar ± 1σ (SE) [fA]	37Ar ± 1σ (SE) [fA]	38Ar ± 1σ (SE) [fA]	39Ar ± 1σ (SE) [fA]	40Ar ± 1σ (SE) [fA]
17D19685	1.8 %	0.0095857 ± 0.0002899	0.1229757 ± 0.0178539	0.0237143 ± 0.0159607	0.0161448 ± 0.0158101	2.6917148 ± 0.0896248
17D19687	1.9 %	0.0108822 ± 0.0002899	0.1412513 ± 0.0178539	0.0428471 ± 0.0159607	0.0152599 ± 0.0158101	3.1073687 ± 0.0896248
17D19688	2.0 %	0.0113292 ± 0.0002899	0.1476916 ± 0.0178539	0.0495407 ± 0.0159607	0.0144828 ± 0.0158101	3.2531501 ± 0.0896248
17D19690	2.2 %	0.0118862 ± 0.0002899	0.1562075 ± 0.0178539	0.0581936 ± 0.0159607	0.0126254 ± 0.0158101	3.4406691 ± 0.0896248
17D19691	2.4 %	0.0120207 ± 0.0002899	0.1586614 ± 0.0178539	0.0605283 ± 0.0159607	0.0116712 ± 0.0158101	3.4898899 ± 0.0896248
17D19693	2.7 %	0.0120586 ± 0.0002899	0.1608219 ± 0.0178539	0.0620794 ± 0.0159607	0.0099766 ± 0.0158101	3.5166233 ± 0.0896248
17D19694	3.0 %	0.0119830 ± 0.0002899	0.1608391 ± 0.0178539	0.0616128 ± 0.0159607	0.0093221 ± 0.0158101	3.5005481 ± 0.0896248
17D19696	3.4 %	0.0116910 ± 0.0002899	0.1594386 ± 0.0178539	0.0589147 ± 0.0159607	0.0085627 ± 0.0158101	3.4242057 ± 0.0896248
17D19697	3.9 %	0.0114923 ± 0.0002899	0.1582637 ± 0.0178539	0.0569417 ± 0.0159607	0.0085032 ± 0.0158101	3.3692795 ± 0.0896248
17D19699	4.5 %	0.0110290 ± 0.0002899	0.1554866 ± 0.0178539	0.0523213 ± 0.0159607	0.0090878 ± 0.0158101	3.2379310 ± 0.0896248
17D19700	5.2 %	0.0107787 ± 0.0002899	0.1540593 ± 0.0178539	0.0498740 ± 0.0159607	0.0097369 ± 0.0158101	3.1657788 ± 0.0896248
17D19702	6.0 %	0.0102716 ± 0.0002899	0.1514798 ± 0.0178539	0.0451325 ± 0.0159607	0.0117113 ± 0.0158101	3.0178547 ± 0.0896248
17D19703	6.9 %	0.0100257 ± 0.0002899	0.1504348 ± 0.0178539	0.0429802 ± 0.0159607	0.0130012 ± 0.0158101	2.9452817 ± 0.0896248
17D19705	7.9 %	0.0095715 ± 0.0002899	0.1490172 ± 0.0178539	0.0393935 ± 0.0159607	0.0160478 ± 0.0158101	2.8095723 ± 0.0896248
17D19706	9.0 %	0.0093706 ± 0.0002899	0.1486840 ± 0.0178539	0.0380425 ± 0.0159607	0.0177289 ± 0.0158101	2.7485638 ± 0.0896248
17D19708	10.3 %	0.0090350 ± 0.0002899	0.1487826 ± 0.0178539	0.0363607 ± 0.0159607	0.0211671 ± 0.0158101	2.6442201 ± 0.0896248
17D19709	11.6 %	0.0089042 ± 0.0002899	0.1491860 ± 0.0178539	0.0360548 ± 0.0159607	0.0228082 ± 0.0158101	2.6019416 ± 0.0896248
17D19711	12.5 %	0.0087222 ± 0.0002899	0.1505450 ± 0.0178539	0.0365024 ± 0.0159607	0.0255940 ± 0.0158101	2.5384746 ± 0.0896248
17D19712	13.4 %	0.0086713 ± 0.0002899	0.1514044 ± 0.0178539	0.0372224 ± 0.0159607	0.0265824 ± 0.0158101	2.5172718 ± 0.0896248
17D19714	14.6 %	0.0086479 ± 0.0002899	0.1532382 ± 0.0178539	0.0396366 ± 0.0159607	0.0272907 ± 0.0158101	2.4940514 ± 0.0896248
17D19715	16.0 %	0.0086733 ± 0.0002899	0.1539504 ± 0.0178539	0.0411175 ± 0.0159607	0.0267679 ± 0.0158101	2.4919712 ± 0.0896248
17D19717	17.6 %	0.0087825 ± 0.0002899	0.1545541 ± 0.0178539	0.0444081 ± 0.0159607	0.0235020 ± 0.0158101	2.5009840 ± 0.0896248
17D19718	19.3 %	0.0088598 ± 0.0002899	0.1542117 ± 0.0178539	0.0460655 ± 0.0159607	0.0205204 ± 0.0158101	2.5098849 ± 0.0896248
17D19720	21.0 %	0.0090391 ± 0.0002899	0.1515682 ± 0.0178539	0.0489512 ± 0.0159607	0.0112137 ± 0.0158101	2.5296566 ± 0.0896248

**Intercept Values**		**36Ar ± 1σ (SE) [fA]**	**r2**	**Regression (type,n)**	**37Ar ± 1σ (SE) [fA]**	**r2**	**Regression (type,n)**	**38Ar ± 1σ (SE) [fA]**	**r2**	**Regression (type,n)**	**39Ar ± 1σ (SE) [fA]**	**r2**	**Regression (type,n)**	**40Ar ± 1σ (SE) [fA]**	**r2**	**Regression (type,n)**
17D19685	1.8 %	0.8862272 ± 0.0013543	0.9475	EXP 150 of 150	4.3500081 ± 0.0178430	0.7229	EXP 150 of 150	0.9024785 ± 0.0162980	0.1362	EXP 150 of 150	53.9455199 ± 0.0163891	0.9978	EXP 150 of 150	297.735790 ± 0.032664	0.9971	EXP 150 of 150
17D19687	1.9 %	0.7924819 ± 0.0014248	0.9216	EXP 150 of 150	8.1378917 ± 0.0198409	0.8531	EXP 150 of 150	0.9118964 ± 0.0177194	0.0705	EXP 150 of 150	56.7356586 ± 0.0187873	0.9974	EXP 150 of 150	266.089638 ± 0.038859	0.9913	EXP 150 of 150
17D19688	2.0 %	0.1693481 ± 0.0006430	0.3691	EXP 150 of 150	1.4183097 ± 0.0181704	0.1769	EXP 150 of 150	0.1665271 ± 0.0142385	0.0038	EXP 150 of 150	11.7807812 ± 0.0164371	0.9467	EXP 150 of 150	56.568319 ± 0.020285	0.9966	EXP 150 of 150
17D19690	2.2 %	0.3172994 ± 0.0007528	0.8354	EXP 149 of 150	4.4193451 ± 0.0182313	0.7159	EXP 150 of 150	0.3036410 ± 0.0151477	0.0244	EXP 150 of 150	20.5917756 ± 0.0176549	0.9822	EXP 150 of 150	102.986225 ± 0.032043	0.9348	EXP 150 of 150
17D19691	2.4 %	0.1638900 ± 0.0006458	0.4092	EXP 150 of 150	2.3981684 ± 0.0167417	0.3744	EXP 150 of 150	0.1343353 ± 0.0179774	0.0011	EXP 150 of 150	10.8426122 ± 0.0171132	0.9350	EXP 150 of 150	52.907709 ± 0.019300	0.9960	EXP 150 of 150
17D19693	2.7 %	0.4036865 ± 0.0010160	0.8334	EXP 150 of 150	10.2606949 ± 0.0198187	0.9094	EXP 150 of 150	0.4102728 ± 0.0174425	0.0507	EXP 150 of 150	24.7500007 ± 0.0174657	0.9878	EXP 150 of 150	123.803187 ± 0.043577	0.4387	EXP 150 of 150
17D19694	3.0 %	0.2006131 ± 0.0007162	0.5579	EXP 150 of 150	5.0388083 ± 0.0180716	0.6913	EXP 150 of 150	0.1516459 ± 0.0175960	0.0001	EXP 149 of 150	11.4940450 ± 0.0164537	0.9475	EXP 150 of 150	60.609241 ± 0.022049	0.9934	EXP 150 of 150
17D19696	3.4 %	0.4198250 ± 0.0010389	0.8379	EXP 148 of 150	16.1183833 ± 0.0187853	0.9592	EXP 150 of 150	0.3381785 ± 0.0174438	0.0015	EXP 150 of 150	23.1785694 ± 0.0186081	0.9840	EXP 149 of 150	120.009743 ± 0.038137	0.4852	EXP 150 of 150
17D19697	3.9 %	0.3967591 ± 0.0010221	0.8191	EXP 150 of 150	19.4710976 ± 0.0184783	0.9741	EXP 150 of 150	0.3203401 ± 0.0171740	0.0113	EXP 150 of 150	21.0370172 ± 0.0184809	0.9818	EXP 150 of 150	106.859545 ± 0.029830	0.9175	EXP 149 of 150
17D19699	4.5 %	0.1933881 ± 0.0006890	0.5958	EXP 150 of 150	9.8342250 ± 0.0189458	0.8988	EXP 150 of 150	0.1310922 ± 0.0168057	0.0015	EXP 150 of 150	9.3604433 ± 0.0155932	0.9284	EXP 150 of 150	50.643917 ± 0.019350	0.9948	EXP 150 of 150
17D19700	5.2 %	0.3880590 ± 0.0009244	0.8488	EXP 150 of 150	23.5837681 ± 0.0182608	0.9821	EXP 150 of 150	0.2702894 ± 0.0165421	0.0118	EXP 150 of 150	18.6847378 ± 0.0162836	0.9804	EXP 149 of 150	97.136192 ± 0.026585	0.9381	EXP 150 of 150
17D19702	6.0 %	0.1429494 ± 0.0005880	0.3082	EXP 150 of 150	7.6237624 ± 0.0167955	0.8706	EXP 150 of 150	0.0529440 ± 0.0162832	0.0000	EXP 150 of 150	5.7765577 ± 0.0163427	0.8158	EXP 150 of 150	36.397167 ± 0.018553	0.9962	EXP 150 of 150
17D19703	6.9 %	0.4444635 ± 0.0010028	0.8628	EXP 150 of 150	26.1204157 ± 0.0179681	0.9859	EXP 150 of 150	0.2846272 ± 0.0164739	0.0119	EXP 150 of 150	18.7981764 ± 0.0157718	0.9827	EXP 150 of 150	111.757239 ± 0.028617	0.3685	EXP 150 of 150
17D19705	7.9 %	0.1334104 ± 0.0005461	0.3761	EXP 150 of 150	6.7794932 ± 0.0192325	0.8059	EXP 150 of 150	0.0584191 ± 0.0166045	0.0028	EXP 150 of 150	4.7123765 ± 0.0154856	0.7471	EXP 150 of 150	33.518751 ± 0.018278	0.9963	EXP 150 of 150
17D19706	9.0 %	0.3985023 ± 0.0008892	0.8607	EXP 150 of 150	15.7965040 ± 0.0182757	0.9603	EXP 150 of 150	0.1968903 ± 0.0158391	0.0001	EXP 150 of 150	12.8356277 ± 0.0153995	0.9621	EXP 150 of 150	106.288287 ± 0.026626	0.2092	EXP 150 of 150
17D19708	10.3 %	0.2120164 ± 0.0006966	0.6979	EXP 150 of 150	6.3870626 ± 0.0178219	0.7882	EXP 150 of 150	0.0967834 ± 0.0178845	0.0030	EXP 150 of 150	5.6917052 ± 0.0159322	0.8132	EXP 150 of 150	57.347217 ± 0.021942	0.9870	EXP 150 of 150
17D19709	11.6 %	1.4186391 ± 0.0018878	0.9661	EXP 150 of 150	27.2250983 ± 0.0209006	0.9825	EXP 150 of 150	0.6322368 ± 0.0164306	0.0310	EXP 150 of 150	24.8540339 ± 0.0165477	0.9889	EXP 150 of 150	405.482550 ± 0.036685	0.9991	EXP 150 of 150
17D19711	12.5 %	0.6032445 ± 0.0011401	0.9205	EXP 150 of 150	14.5247225 ± 0.0189343	0.9510	EXP 150 of 150	0.2579893 ± 0.0177849	0.0008	EXP 150 of 150	10.2399070 ± 0.0158543	0.9382	EXP 150 of 150	167.629679 ± 0.024692	0.9899	EXP 150 of 150
17D19712	13.4 %	0.6900210 ± 0.0013196	0.9174	EXP 150 of 150	21.4864137 ± 0.0191405	0.9771	EXP 150 of 150	0.2817179 ± 0.0163436	0.0009	EXP 150 of 150	11.1997613 ± 0.0153882	0.9528	EXP 150 of 150	187.780997 ± 0.026853	0.9930	EXP 150 of 150
17D19714	14.6 %	0.4804755 ± 0.0011024	0.8740	EXP 150 of 150	18.7919234 ± 0.0195091	0.9688	EXP 150 of 150	0.1988934 ± 0.0150494	0.0018	EXP 150 of 150	8.1660597 ± 0.0145012	0.9176	EXP 150 of 150	124.679425 ± 0.022797	0.9302	EXP 149 of 150
17D19715	16.0 %	0.6148635 ± 0.0012481	0.9104	EXP 150 of 150	31.9290208 ± 0.0199453	0.9886	EXP 150 of 150	0.2680775 ± 0.0166889	0.0074	EXP 150 of 150	9.5886843 ± 0.0156459	0.9259	EXP 150 of 150	150.481872 ± 0.023120	0.9830	EXP 149 of 150
17D19717	17.6 %	0.2094351 ± 0.0006697	0.6775	EXP 149 of 150	12.3468466 ± 0.0192666	0.9294	EXP 150 of 150	0.0639459 ± 0.0164339	0.0014	EXP 150 of 150	3.3367914 ± 0.0151307	0.5954	EXP 150 of 150	49.013944 ± 0.020369	0.9910	EXP 150 of 150
17D19718	19.3 %	0.1741036 ± 0.0006878	0.5652	EXP 150 of 150	10.2668126 ± 0.0178095	0.9136	EXP 150 of 150	0.0712547 ± 0.0174583	0.0366	EXP 150 of 150	2.6133067 ± 0.0171684	0.3437	EXP 150 of 150	40.191017 ± 0.020085	0.9933	EXP 150 of 150
17D19720	21.0 %	0.0438503 ± 0.0003528	0.2558	EXP 150 of 150	1.6920224 ± 0.0185319	0.2717	EXP 150 of 150	0.0204083 ± 0.0169483	0.0066	EXP 150 of 150	0.4060550 ± 0.0148670	0.0131	EXP 149 of 150	10.935185 ± 0.016146	0.9978	EXP 150 of 150

**Project Info**		Analyst	Irradiation	X-pos	Y-pos	Z/H-pos	Project	Experiment	Nmb
17D19685	1.8 %	Dan Miggins	17-OSU-01	0.00	0.00	22.05	Arctic\O-Connor (16-22)	17D19681	01
17D19687	1.9 %	Dan Miggins	17-OSU-01	0.00	0.00	22.05	Arctic\O-Connor (16-22)	17D19681	01
17D19688	2.0 %	Dan Miggins	17-OSU-01	0.00	0.00	22.05	Arctic\O-Connor (16-22)	17D19681	01
17D19690	2.2 %	Dan Miggins	17-OSU-01	0.00	0.00	22.05	Arctic\O-Connor (16-22)	17D19681	01
17D19691	2.4 %	Dan Miggins	17-OSU-01	0.00	0.00	22.05	Arctic\O-Connor (16-22)	17D19681	01
17D19693	2.7 %	Dan Miggins	17-OSU-01	0.00	0.00	22.05	Arctic\O-Connor (16-22)	17D19681	01
17D19694	3.0 %	Dan Miggins	17-OSU-01	0.00	0.00	22.05	Arctic\O-Connor (16-22)	17D19681	01
17D19696	3.4 %	Dan Miggins	17-OSU-01	0.00	0.00	22.05	Arctic\O-Connor (16-22)	17D19681	01
17D19697	3.9 %	Dan Miggins	17-OSU-01	0.00	0.00	22.05	Arctic\O-Connor (16-22)	17D19681	01
17D19699	4.5 %	Dan Miggins	17-OSU-01	0.00	0.00	22.05	Arctic\O-Connor (16-22)	17D19681	01
17D19700	5.2 %	Dan Miggins	17-OSU-01	0.00	0.00	22.05	Arctic\O-Connor (16-22)	17D19681	01
17D19702	6.0 %	Dan Miggins	17-OSU-01	0.00	0.00	22.05	Arctic\O-Connor (16-22)	17D19681	01
17D19703	6.9 %	Dan Miggins	17-OSU-01	0.00	0.00	22.05	Arctic\O-Connor (16-22)	17D19681	01
17D19705	7.9 %	Dan Miggins	17-OSU-01	0.00	0.00	22.05	Arctic\O-Connor (16-22)	17D19681	01
17D19706	9.0 %	Dan Miggins	17-OSU-01	0.00	0.00	22.05	Arctic\O-Connor (16-22)	17D19681	01
17D19708	10.3 %	Dan Miggins	17-OSU-01	0.00	0.00	22.05	Arctic\O-Connor (16-22)	17D19681	01
17D19709	11.6 %	Dan Miggins	17-OSU-01	0.00	0.00	22.05	Arctic\O-Connor (16-22)	17D19681	01
17D19711	12.5 %	Dan Miggins	17-OSU-01	0.00	0.00	22.05	Arctic\O-Connor (16-22)	17D19681	01
17D19712	13.4 %	Dan Miggins	17-OSU-01	0.00	0.00	22.05	Arctic\O-Connor (16-22)	17D19681	01
17D19714	14.6 %	Dan Miggins	17-OSU-01	0.00	0.00	22.05	Arctic\O-Connor (16-22)	17D19681	01
17D19715	16.0 %	Dan Miggins	17-OSU-01	0.00	0.00	22.05	Arctic\O-Connor (16-22)	17D19681	01
17D19717	17.6 %	Dan Miggins	17-OSU-01	0.00	0.00	22.05	Arctic\O-Connor (16-22)	17D19681	01
17D19718	19.3 %	Dan Miggins	17-OSU-01	0.00	0.00	22.05	Arctic\O-Connor (16-22)	17D19681	01
17D19720	21.0 %	Dan Miggins	17-OSU-01	0.00	0.00	22.05	Arctic\O-Connor (16-22)	17D19681	01

**Sample Parameters**		Sample	Material	Location	Standard Name	Standard (in Ma)	%1σ	Standard Reference	Standard 40Ar/39Ar	%1σ	J	%1σ	Air 40Ar/36Ar	%1σ	MDF (lin)	%1σ	Volume Ratio	Sensitivity (mol/volt)	Day	Month	Year	Hour	Min	Resist
17D19685	1.8 %	PS59-312-11	Groundmass	Gakkel Ridge	FCT-NM (1C14-17)	28.201	0.082	Kuiper et al (2008)	9.55759	0.089	0.00164449	0.089	302.54	0.11	0.9941751	0.064	1	4.8E-14	9	JUN	2017	19	38	1
17D19687	1.9 %	PS59-312-11	Groundmass	Gakkel Ridge	FCT-NM (1C14-17)	28.201	0.082	Kuiper et al (2008)	9.55759	0.089	0.00164449	0.089	302.54	0.11	0.9941751	0.064	1	4.8E-14	9	JUN	2017	19	58	1
17D19688	2.0 %	PS59-312-11	Groundmass	Gakkel Ridge	FCT-NM (1C14-17)	28.201	0.082	Kuiper et al (2008)	9.55759	0.089	0.00164449	0.089	302.54	0.11	0.9941751	0.064	1	4.8E-14	9	JUN	2017	20	8	1
17D19690	2.2 %	PS59-312-11	Groundmass	Gakkel Ridge	FCT-NM (1C14-17)	28.201	0.082	Kuiper et al (2008)	9.55759	0.089	0.00164449	0.089	302.54	0.11	0.9941751	0.064	1	4.8E-14	9	JUN	2017	20	28	1
17D19691	2.4 %	PS59-312-11	Groundmass	Gakkel Ridge	FCT-NM (1C14-17)	28.201	0.082	Kuiper et al (2008)	9.55759	0.089	0.00164449	0.089	302.54	0.11	0.9941751	0.064	1	4.8E-14	9	JUN	2017	20	38	1
17D19693	2.7 %	PS59-312-11	Groundmass	Gakkel Ridge	FCT-NM (1C14-17)	28.201	0.082	Kuiper et al (2008)	9.55759	0.089	0.00164449	0.089	302.54	0.11	0.9941751	0.064	1	4.8E-14	9	JUN	2017	20	58	1
17D19694	3.0 %	PS59-312-11	Groundmass	Gakkel Ridge	FCT-NM (1C14-17)	28.201	0.082	Kuiper et al (2008)	9.55759	0.089	0.00164449	0.089	302.54	0.11	0.9941751	0.064	1	4.8E-14	9	JUN	2017	21	8	1
17D19696	3.4 %	PS59-312-11	Groundmass	Gakkel Ridge	FCT-NM (1C14-17)	28.201	0.082	Kuiper et al (2008)	9.55759	0.089	0.00164449	0.089	302.54	0.11	0.9941751	0.064	1	4.8E-14	9	JUN	2017	21	28	1
17D19697	3.9 %	PS59-312-11	Groundmass	Gakkel Ridge	FCT-NM (1C14-17)	28.201	0.082	Kuiper et al (2008)	9.55759	0.089	0.00164449	0.089	302.54	0.11	0.9941751	0.064	1	4.8E-14	9	JUN	2017	21	38	1
17D19699	4.5 %	PS59-312-11	Groundmass	Gakkel Ridge	FCT-NM (1C14-17)	28.201	0.082	Kuiper et al (2008)	9.55759	0.089	0.00164449	0.089	302.54	0.11	0.9941751	0.064	1	4.8E-14	9	JUN	2017	21	58	1
17D19700	5.2 %	PS59-312-11	Groundmass	Gakkel Ridge	FCT-NM (1C14-17)	28.201	0.082	Kuiper et al (2008)	9.55759	0.089	0.00164449	0.089	302.54	0.11	0.9941751	0.064	1	4.8E-14	9	JUN	2017	22	8	1
17D19702	6.0 %	PS59-312-11	Groundmass	Gakkel Ridge	FCT-NM (1C14-17)	28.201	0.082	Kuiper et al (2008)	9.55759	0.089	0.00164449	0.089	302.54	0.11	0.9941751	0.064	1	4.8E-14	9	JUN	2017	22	28	1
17D19703	6.9 %	PS59-312-11	Groundmass	Gakkel Ridge	FCT-NM (1C14-17)	28.201	0.082	Kuiper et al (2008)	9.55759	0.089	0.00164449	0.089	302.54	0.11	0.9941751	0.064	1	4.8E-14	9	JUN	2017	22	38	1
17D19705	7.9 %	PS59-312-11	Groundmass	Gakkel Ridge	FCT-NM (1C14-17)	28.201	0.082	Kuiper et al (2008)	9.55759	0.089	0.00164449	0.089	302.54	0.11	0.9941751	0.064	1	4.8E-14	9	JUN	2017	22	58	1
17D19706	9.0 %	PS59-312-11	Groundmass	Gakkel Ridge	FCT-NM (1C14-17)	28.201	0.082	Kuiper et al (2008)	9.55759	0.089	0.00164449	0.089	302.54	0.11	0.9941751	0.064	1	4.8E-14	9	JUN	2017	23	8	1
17D19708	10.3 %	PS59-312-11	Groundmass	Gakkel Ridge	FCT-NM (1C14-17)	28.201	0.082	Kuiper et al (2008)	9.55759	0.089	0.00164449	0.089	302.54	0.11	0.9941751	0.064	1	4.8E-14	9	JUN	2017	23	28	1
17D19709	11.6 %	PS59-312-11	Groundmass	Gakkel Ridge	FCT-NM (1C14-17)	28.201	0.082	Kuiper et al (2008)	9.55759	0.089	0.00164449	0.089	302.54	0.11	0.9941751	0.064	1	4.8E-14	9	JUN	2017	23	38	1
17D19711	12.5 %	PS59-312-11	Groundmass	Gakkel Ridge	FCT-NM (1C14-17)	28.201	0.082	Kuiper et al (2008)	9.55759	0.089	0.00164449	0.089	302.54	0.11	0.9941751	0.064	1	4.8E-14	9	JUN	2017	23	58	1
17D19712	13.4 %	PS59-312-11	Groundmass	Gakkel Ridge	FCT-NM (1C14-17)	28.201	0.082	Kuiper et al (2008)	9.55759	0.089	0.00164449	0.089	302.54	0.11	0.9941751	0.064	1	4.8E-14	10	JUN	2017	0	8	1
17D19714	14.6 %	PS59-312-11	Groundmass	Gakkel Ridge	FCT-NM (1C14-17)	28.201	0.082	Kuiper et al (2008)	9.55759	0.089	0.00164449	0.089	302.54	0.11	0.9941751	0.064	1	4.8E-14	10	JUN	2017	0	29	1
17D19715	16.0 %	PS59-312-11	Groundmass	Gakkel Ridge	FCT-NM (1C14-17)	28.201	0.082	Kuiper et al (2008)	9.55759	0.089	0.00164449	0.089	302.54	0.11	0.9941751	0.064	1	4.8E-14	10	JUN	2017	0	39	1
17D19717	17.6 %	PS59-312-11	Groundmass	Gakkel Ridge	FCT-NM (1C14-17)	28.201	0.082	Kuiper et al (2008)	9.55759	0.089	0.00164449	0.089	302.54	0.11	0.9941751	0.064	1	4.8E-14	10	JUN	2017	0	59	1
17D19718	19.3 %	PS59-312-11	Groundmass	Gakkel Ridge	FCT-NM (1C14-17)	28.201	0.082	Kuiper et al (2008)	9.55759	0.089	0.00164449	0.089	302.54	0.11	0.9941751	0.064	1	4.8E-14	10	JUN	2017	1	9	1
17D19720	21.0 %	PS59-312-11	Groundmass	Gakkel Ridge	FCT-NM (1C14-17)	28.201	0.082	Kuiper et al (2008)	9.55759	0.089	0.00164449	0.089	302.54	0.11	0.9941751	0.064	1	4.8E-14	10	JUN	2017	1	29	1

Irradiation Constants		40/36(a)	%1σ	40/36(c)	%1σ	38/36(a)	%1σ	38/36(c)	%1σ	39/37(ca)	%1σ	38/37(ca)	%1σ	36/37(ca)	%1σ	40/39(k)	%1σ	38/39(k)	%1σ	36/38(cl)	%1σ	K/Ca	%1σ	K/Cl	%1σ	Ca/Cl	%1σ
17D19685	1.8 %	295.5	0	0.018	35	0.1869	0	1.493	3	0.000643	0.92	0.00018	9.63	0.00027	0.17	0.000607	9.65	0.012077	0.09	0	0	0.43	0	0	0	0	0
17D19687	1.9 %	295.5	0	0.018	35	0.1869	0	1.493	3	0.000643	0.92	0.00018	9.63	0.00027	0.17	0.000607	9.65	0.012077	0.09	0	0	0.43	0	0	0	0	0
17D19688	2.0 %	295.5	0	0.018	35	0.1869	0	1.493	3	0.000643	0.92	0.00018	9.63	0.00027	0.17	0.000607	9.65	0.012077	0.09	0	0	0.43	0	0	0	0	0
17D19690	2.2 %	295.5	0	0.018	35	0.1869	0	1.493	3	0.000643	0.92	0.00018	9.63	0.00027	0.17	0.000607	9.65	0.012077	0.09	0	0	0.43	0	0	0	0	0
17D19691	2.4 %	295.5	0	0.018	35	0.1869	0	1.493	3	0.000643	0.92	0.00018	9.63	0.00027	0.17	0.000607	9.65	0.012077	0.09	0	0	0.43	0	0	0	0	0
17D19693	2.7 %	295.5	0	0.018	35	0.1869	0	1.493	3	0.000643	0.92	0.00018	9.63	0.00027	0.17	0.000607	9.65	0.012077	0.09	0	0	0.43	0	0	0	0	0
17D19694	3.0 %	295.5	0	0.018	35	0.1869	0	1.493	3	0.000643	0.92	0.00018	9.63	0.00027	0.17	0.000607	9.65	0.012077	0.09	0	0	0.43	0	0	0	0	0
17D19696	3.4 %	295.5	0	0.018	35	0.1869	0	1.493	3	0.000643	0.92	0.00018	9.63	0.00027	0.17	0.000607	9.65	0.012077	0.09	0	0	0.43	0	0	0	0	0
17D19697	3.9 %	295.5	0	0.018	35	0.1869	0	1.493	3	0.000643	0.92	0.00018	9.63	0.00027	0.17	0.000607	9.65	0.012077	0.09	0	0	0.43	0	0	0	0	0
17D19699	4.5 %	295.5	0	0.018	35	0.1869	0	1.493	3	0.000643	0.92	0.00018	9.63	0.00027	0.17	0.000607	9.65	0.012077	0.09	0	0	0.43	0	0	0	0	0
17D19700	5.2 %	295.5	0	0.018	35	0.1869	0	1.493	3	0.000643	0.92	0.00018	9.63	0.00027	0.17	0.000607	9.65	0.012077	0.09	0	0	0.43	0	0	0	0	0
17D19702	6.0 %	295.5	0	0.018	35	0.1869	0	1.493	3	0.000643	0.92	0.00018	9.63	0.00027	0.17	0.000607	9.65	0.012077	0.09	0	0	0.43	0	0	0	0	0
17D19703	6.9 %	295.5	0	0.018	35	0.1869	0	1.493	3	0.000643	0.92	0.00018	9.63	0.00027	0.17	0.000607	9.65	0.012077	0.09	0	0	0.43	0	0	0	0	0
17D19705	7.9 %	295.5	0	0.018	35	0.1869	0	1.493	3	0.000643	0.92	0.00018	9.63	0.00027	0.17	0.000607	9.65	0.012077	0.09	0	0	0.43	0	0	0	0	0
17D19706	9.0 %	295.5	0	0.018	35	0.1869	0	1.493	3	0.000643	0.92	0.00018	9.63	0.00027	0.17	0.000607	9.65	0.012077	0.09	0	0	0.43	0	0	0	0	0
17D19708	10.3 %	295.5	0	0.018	35	0.1869	0	1.493	3	0.000643	0.92	0.00018	9.63	0.00027	0.17	0.000607	9.65	0.012077	0.09	0	0	0.43	0	0	0	0	0
17D19709	11.6 %	295.5	0	0.018	35	0.1869	0	1.493	3	0.000643	0.92	0.00018	9.63	0.00027	0.17	0.000607	9.65	0.012077	0.09	0	0	0.43	0	0	0	0	0
17D19711	12.5 %	295.5	0	0.018	35	0.1869	0	1.493	3	0.000643	0.92	0.00018	9.63	0.00027	0.17	0.000607	9.65	0.012077	0.09	0	0	0.43	0	0	0	0	0
17D19712	13.4 %	295.5	0	0.018	35	0.1869	0	1.493	3	0.000643	0.92	0.00018	9.63	0.00027	0.17	0.000607	9.65	0.012077	0.09	0	0	0.43	0	0	0	0	0
17D19714	14.6 %	295.5	0	0.018	35	0.1869	0	1.493	3	0.000643	0.92	0.00018	9.63	0.00027	0.17	0.000607	9.65	0.012077	0.09	0	0	0.43	0	0	0	0	0
17D19715	16.0 %	295.5	0	0.018	35	0.1869	0	1.493	3	0.000643	0.92	0.00018	9.63	0.00027	0.17	0.000607	9.65	0.012077	0.09	0	0	0.43	0	0	0	0	0
17D19717	17.6 %	295.5	0	0.018	35	0.1869	0	1.493	3	0.000643	0.92	0.00018	9.63	0.00027	0.17	0.000607	9.65	0.012077	0.09	0	0	0.43	0	0	0	0	0
17D19718	19.3 %	295.5	0	0.018	35	0.1869	0	1.493	3	0.000643	0.92	0.00018	9.63	0.00027	0.17	0.000607	9.65	0.012077	0.09	0	0	0.43	0	0	0	0	0
17D19720	21.0 %	295.5	0	0.018	35	0.1869	0	1.493	3	0.000643	0.92	0.00018	9.63	0.00027	0.17	0.000607	9.65	0.012077	0.09	0	0	0.43	0	0	0	0	0

17D19681.AGE >>> PS59-312-11 >>> ARCTIC | O-CONNOR (16-22) PROJECT

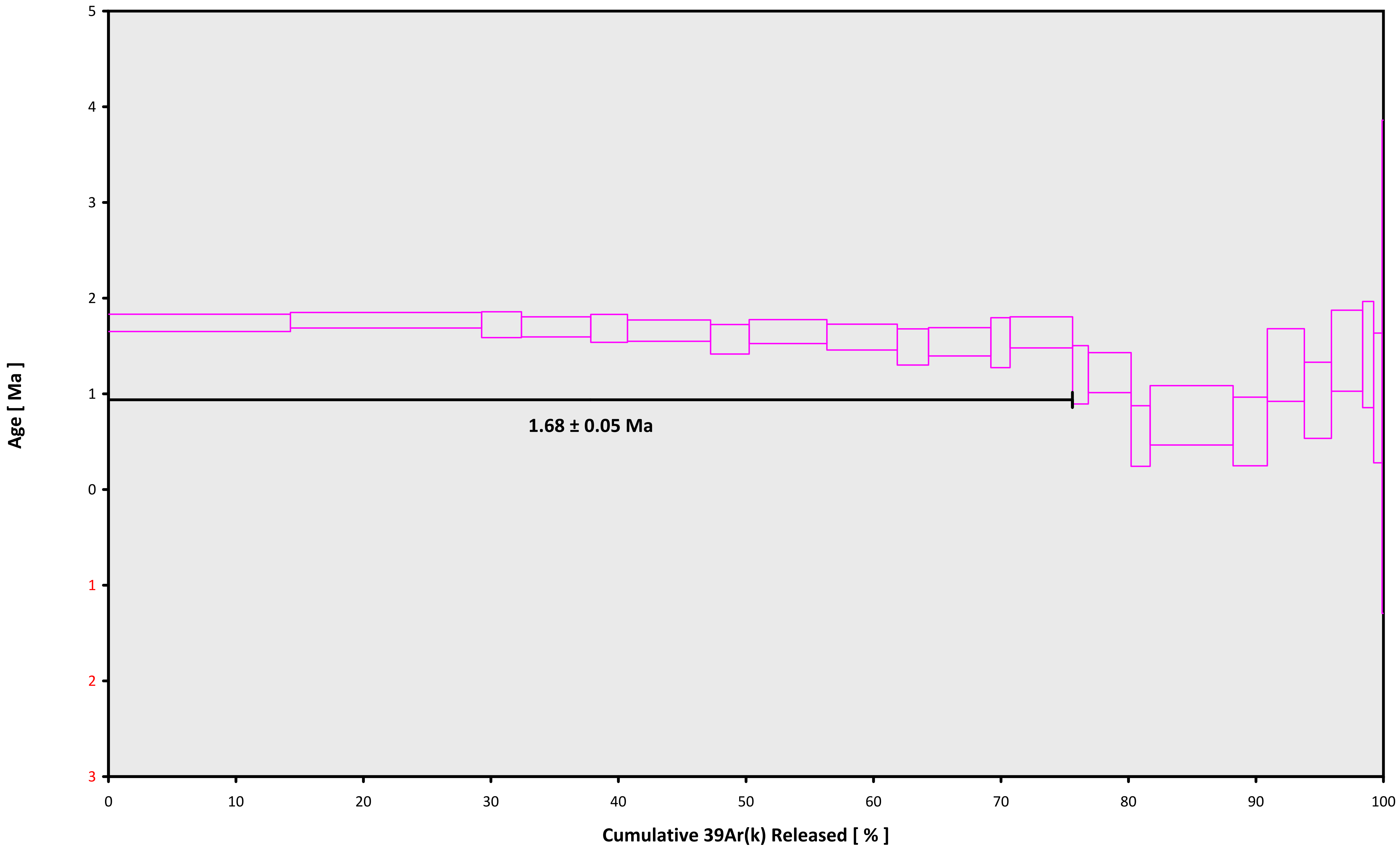

Ar-Ages in Ma

WEIGHTED PLATEAU
1.68 ± 0.05
TOTAL FUSION
1.51 ± 0.04
NORMAL ISOCHRON
1.95 ± 0.52
INVERSE ISOCHRON
1.95 ± 0.47

MSWD (PROBABILITY)
1.69 (6%)

Sample Info

Groundmass
Gakkel Ridge
Dan Miggins

IRR = 17-OSU-01 (1C14-17)
J = 0.00164449 ± 0.00000146

OSU Argon Geochronology Lab
CEOAS Oregon State University, Corvallis, USA

17D19681.AGE >>> PS59-312-11 >>> ARCTIC | O-CONNOR (16-22) PROJECT

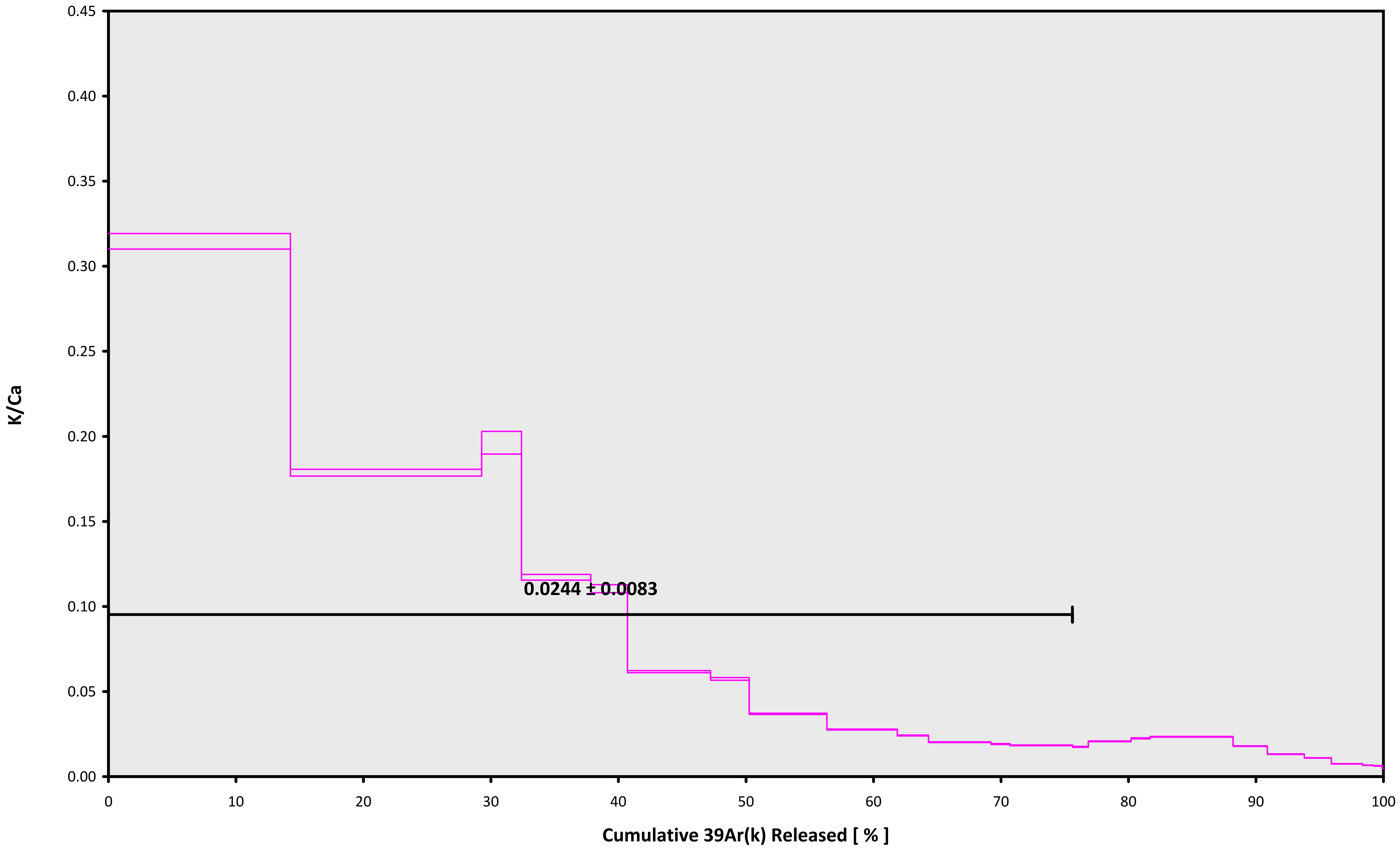

Ar-Ages in Ma

WEIGHTED PLATEAU
1.68 ± 0.05
TOTAL FUSION
1.51 ± 0.04
NORMAL ISOCHRON
1.95 ± 0.52
INVERSE ISOCHRON
1.95 ± 0.47

Sample Info

Groundmass
Gakkel Ridge
Dan Miggins

IRR = 17-OSU-01 (1C14-17)
J = 0.00164449 ± 0.00000146

17D19681.full.xls printed at 17-09-2017 (14:21)
ArArCALC v2.7.0 -- Beta Version

17D19681.AGE >>> PS59-312-11 >>> ARCTIC | O-CONNOR (16-22) PROJECT

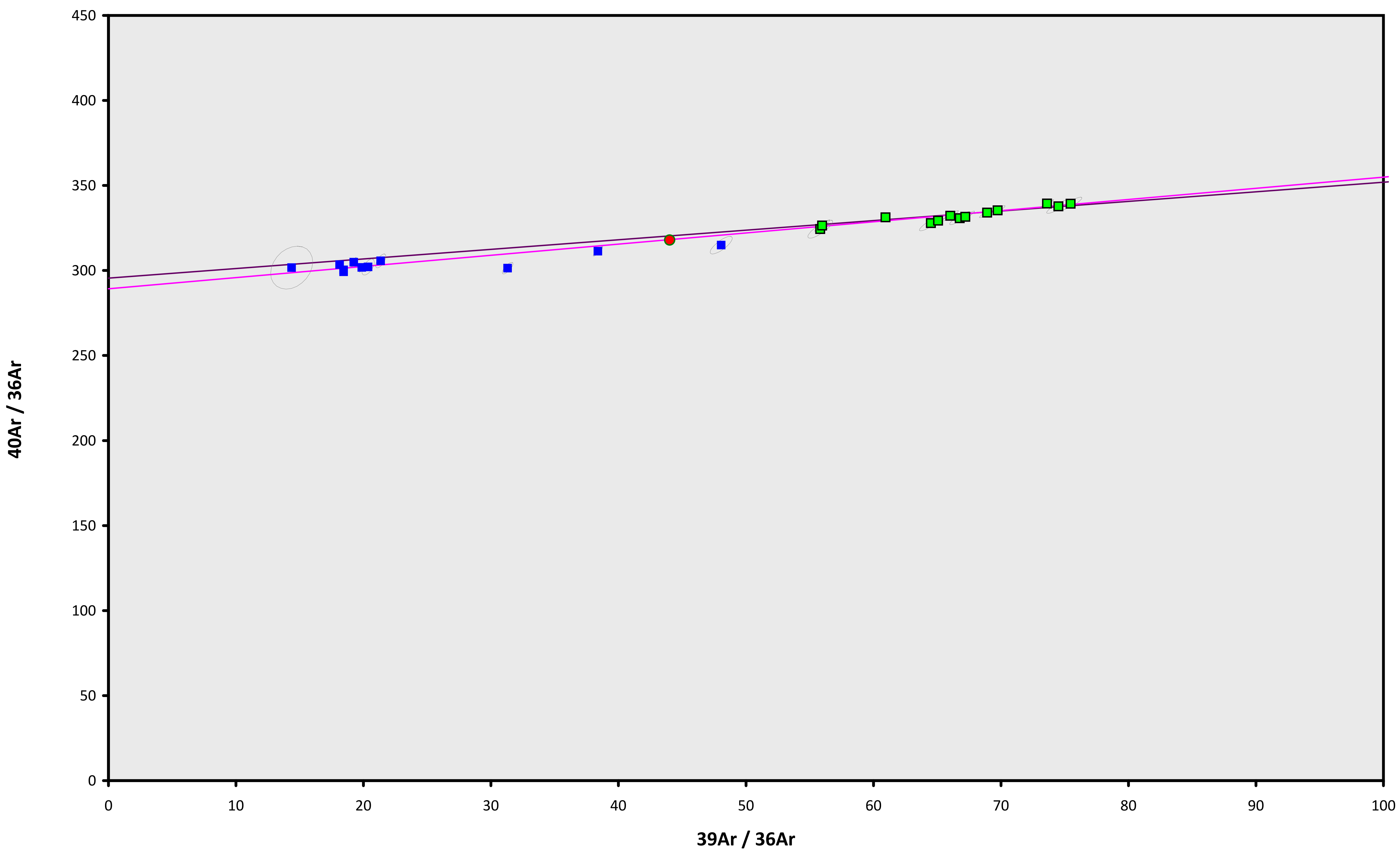

Ar-Ages in Ma

WEIGHTED PLATEAU
1.68 ± 0.05
TOTAL FUSION
1.51 ± 0.04
NORMAL ISOCHRON
1.95 ± 0.52
INVERSE ISOCHRON
1.95 ± 0.47

MSWD (PROBABILITY)
1.69 (7%)

40AR/36AR INTERCEPT
289.3 ± 11.9

Sample Info

Groundmass
Gakkel Ridge
Dan Miggins

IRR = 17-OSU-01 (1C14-17)
J = 0.00164449 ± 0.00000146

17D19681.AGE >>> PS59-312-11 >>> ARCTIC | O-CONNOR (16-22) PROJECT

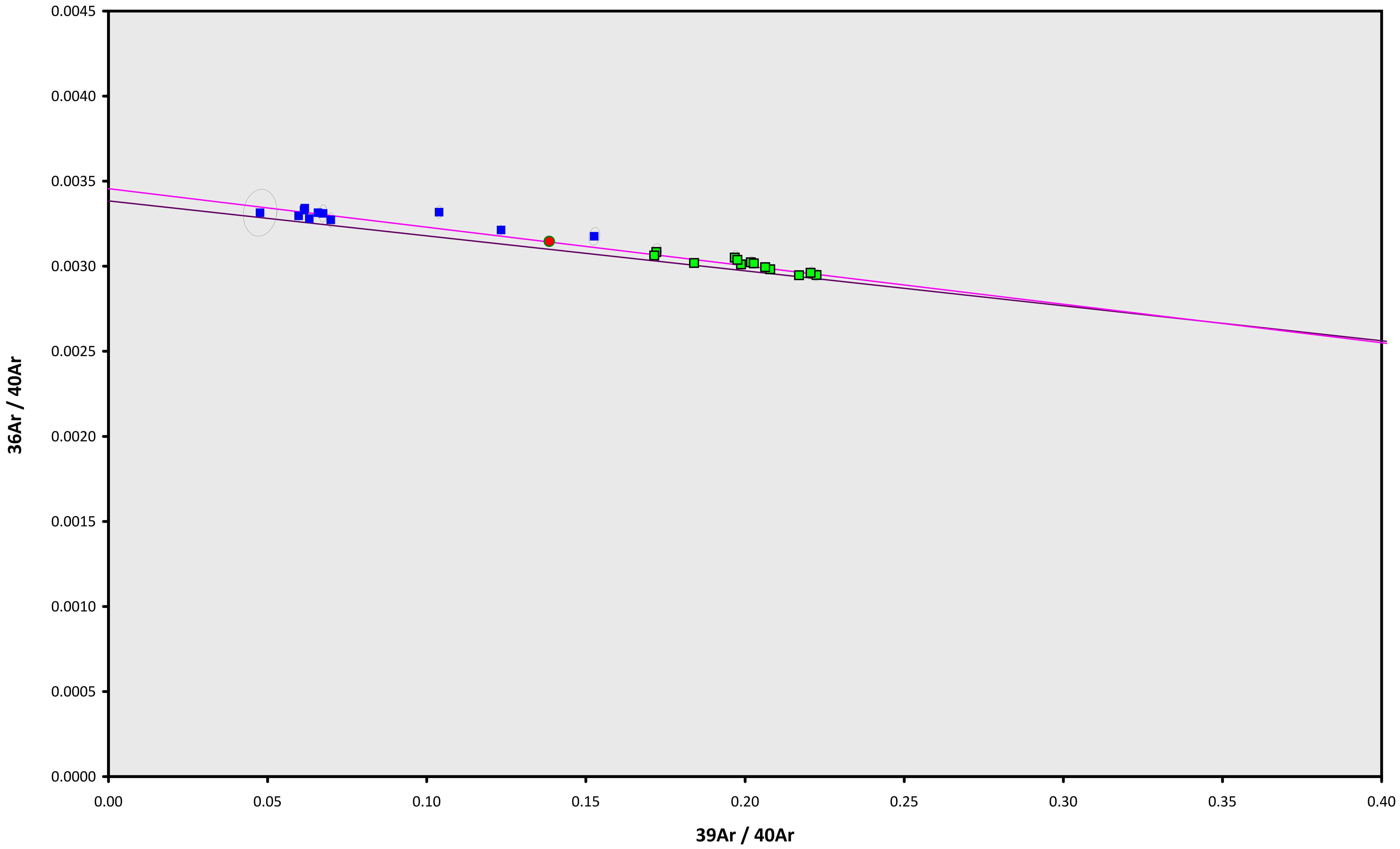

Ar-Ages in Ma

WEIGHTED PLATEAU
1.68 ± 0.05
TOTAL FUSION
1.51 ± 0.04
NORMAL ISOCHRON
1.95 ± 0.52
INVERSE ISOCHRON
1.95 ± 0.47

MSWD (PROBABILITY)
1.68 (7%)

SPREADING FACTOR
3.3%

40AR/36AR INTERCEPT
289.4 ± 11.8

Sample Info

Groundmass
Gakkel Ridge
Dan Miggins

IRR = 17-OSU-01 (1C14-17)
J = 0.00164449 ± 0.00000146

Relative Abundances			36Ar [fA]	%1σ	37Ar [fA]	%1σ	38Ar [fA]	%1σ	39Ar [fA]	%1σ	40Ar [fA]	%1σ	40(r)/39(k) ± 2σ	Age ± 2σ (ka)	40Ar(r) (%)	39Ar(k) (%)	K/Ca ± 2σ
17D19872	1.8 %	✓	2.8441718	0.288	143.2736	0.541	1.4153096	1.626	61.79969	0.079	843.0961	0.006	0.22781 ± 0.07857	682.0 ± 235.2	1.67	23.73	0.1852 ± 0.0020
17D19874	1.9 %	✓	0.5393742	0.351	60.8876	0.856	0.3464671	6.776	18.58998	0.142	159.8269	0.036	0.28542 ± 0.06084	854.4 ± 182.1	3.31	7.13	0.1310 ± 0.0023
17D19875	2.0 %	✓	0.3675873	0.382	84.0488	0.700	0.2704202	9.413	15.80571	0.163	106.9942	0.035	0.32225 ± 0.05321	964.6 ± 159.2	4.74	6.06	0.0806 ± 0.0012
17D19877	2.2 %	✓	0.2180766	0.498	67.3714	0.764	0.1823517	13.069	10.08975	0.232	61.5336	0.045	0.24564 ± 0.06465	735.3 ± 193.5	4.01	3.86	0.0641 ± 0.0010
17D19878	2.4 %	✓	0.1324941	0.564	50.1399	0.974	0.0759205	31.497	6.45779	0.362	36.5303	0.069	0.21465 ± 0.07032	642.6 ± 210.5	3.78	2.47	0.0551 ± 0.0011
17D19880	2.7 %	✓	0.2593541	0.439	155.3400	0.535	0.2326954	10.877	13.90548	0.177	67.4821	0.043	0.23484 ± 0.04997	703.0 ± 149.6	4.80	5.31	0.0382 ± 0.0004
17D19881	3.0 %	✓	0.0891032	0.716	51.7774	0.980	0.0770375	30.178	4.59203	0.510	22.8084	0.112	0.13409 ± 0.08541	401.4 ± 255.7	2.68	1.75	0.0379 ± 0.0008
17D19883	3.4 %	✓	0.2810186	0.416	247.2287	0.486	0.2409315	9.844	15.98388	0.153	66.7416	0.041	0.21725 ± 0.04560	650.4 ± 136.5	5.15	6.09	0.0275 ± 0.0003
17D19884	3.9 %	✓	0.2688429	0.434	293.5956	0.477	0.2188287	10.240	15.38230	0.166	59.0419	0.049	0.20009 ± 0.04819	599.0 ± 144.2	5.15	5.84	0.0223 ± 0.0002
17D19886	4.5 %	✓	0.2513039	0.439	317.9594	0.472	0.2238049	10.991	14.20014	0.172	51.7686	0.051	0.20695 ± 0.05015	619.5 ± 150.1	5.59	5.38	0.0189 ± 0.0002
17D19887	5.2 %	✓	0.2296603	0.459	320.1407	0.473	0.2327040	10.480	12.72764	0.200	44.9105	0.060	0.20836 ± 0.05399	623.7 ± 161.6	5.81	4.82	0.0168 ± 0.0002
17D19889	6.0 %	✓	0.2060994	0.490	293.1296	0.478	0.1851952	13.071	10.83891	0.215	40.2954	0.069	0.26289 ± 0.06055	787.0 ± 181.2	6.95	4.10	0.0156 ± 0.0002
17D19890	6.9 %	✓	0.1363867	0.554	186.6720	0.500	0.0812846	29.230	6.57208	0.347	26.1449	0.098	0.11607 ± 0.07386	347.5 ± 221.1	2.86	2.48	0.0149 ± 0.0002
17D19892	7.9 %	✓	0.1744229	0.486	224.4846	0.491	0.1422905	17.539	7.91016	0.295	35.3228	0.073	0.21974 ± 0.06915	657.8 ± 207.0	4.83	2.99	0.0149 ± 0.0002
17D19893	9.0 %	✓	0.1864942	0.476	214.2641	0.497	0.1093844	22.284	7.59297	0.307	39.6028	0.065	0.21506 ± 0.07465	643.8 ± 223.4	4.05	2.87	0.0150 ± 0.0002
17D19895	10.3 %	✓	0.2306440	0.446	225.7090	0.495	0.1514710	15.444	7.91975	0.300	50.8672	0.055	0.09458 ± 0.08217	283.2 ± 246.0	1.45	2.99	0.0148 ± 0.0002
17D19896	11.6 %	✓	0.3083495	0.412	238.7690	0.488	0.1695812	14.108	7.77894	0.301	73.6998	0.036	0.21628 ± 0.10209	647.5 ± 305.6	2.24	2.93	0.0137 ± 0.0002
17D19898	12.5 %	✓	0.2290488	0.439	172.1995	0.515	0.1282386	18.810	5.52573	0.428	55.0919	0.045	0.21400 ± 0.11345	640.6 ± 339.6	2.10	2.08	0.0135 ± 0.0002
17D19899	13.4 %	✓	0.2488078	0.404	175.1667	0.519	0.1274360	18.858	4.40109	0.513	60.3743	0.047	0.19603 ± 0.14373	586.8 ± 430.2	1.39	1.65	0.0105 ± 0.0002
17D19901	14.6 %	✓	0.2711190	0.422	192.3469	0.506	0.1259028	17.982	4.15550	0.548	65.3940	0.042	0.15859 ± 0.17302	474.8 ± 517.9	0.98	1.55	0.0090 ± 0.0001
17D19902	16.0 %		0.2644415	0.424	252.8763	0.483	0.1273507	18.516	4.01000	0.555	59.4637	0.045	0.39431 ± 0.18082	1180.2 ± 541.0	2.55	1.48	0.0065 ± 0.0001
17D19904	17.6 %		0.1558514	0.502	180.1995	0.515	0.0621341	40.470	2.58253	0.851	32.2950	0.076	0.25646 ± 0.19891	767.7 ± 595.3	1.96	0.95	0.0059 ± 0.0001
17D19905	19.3 %		0.1151693	0.599	142.2285	0.541	0.0798059	29.530	1.93026	1.163	22.9928	0.108	0.17437 ± 0.23397	522.0 ± 700.3	1.39	0.71	0.0056 ± 0.0001
17D19907	21.0 %		0.1393143	0.576	182.0193	0.513	0.0812476	28.160	2.12998	1.060	27.3104	0.091	0.33820 ± 0.24972	1012.3 ± 747.3	2.49	0.77	0.0048 ± 0.0001
		Σ	8.1471357	0.119	4471.8283	0.111	5.0877938	2.304	262.88230	0.047	2109.5890	0.007					

Information on Analysis and Constants Used in Calculations

Project = **O-CONNOR (16-22)**
Sample = **PS59-311-1**
Material = **Groundmass**
Location = **Gakkel Ridge**
Region = **Artic Ocean**
Analyst = **Dan Miggins**
Irradiation = **17-OSU-01 (1C8-17)**
Position = **X: 0 | Y: 0 | Z/H: 13.0264 mm**
FCT-NM Age = **28.201 ± 0.023 Ma**
FCT-NM Reference = **Kuiper et al (2008)**
FCT-NM 40Ar/39Ar Ratio = **9.49259 ± 0.00854**
FCT-NM J-value = **0.00165575 ± 0.00000149**
Air Shot 40Ar/36Ar = **302.4950 ± 0.4174**
Air Shot MDF = **0.99421144 ± 0.00067064 (LIN)**
Experiment Type = **Incremental Heating**
Extraction Method = **Bulk Laser Heating**
Heating = **64 sec**
Isolation = **3.00 min**
Instrument = **ARGUS-VI-D**
Preferred Age = **Plateau Age**
Age Classification = **Crystallization Age**
IGSN = Undefined
Rock Class = **Igneous>Volcanic**
Lithology = **Basaltic Lava**
Lat-Lon = Undefined - Undefined

Age Equations = **Min et al. (2000)**
Negative Intensities = **Allowed**
Collector Calibrations = **36Ar**
Decay 40K = **5.530 ± 0.048 E-10 1/a**
Decay 39Ar = **2.940 ± 0.016 E-07 1/h**
Decay 37Ar = **8.230 ± 0.012 E-04 1/h**
Decay 36Cl = **2.257 ± 0.015 E-06 1/a**
Decay 40K(EC,β^+) = **0.580 ± 0.009 E-10 1/a**
Decay 40K(β^-) = **4.950 ± 0.043 E-10 1/a**
Atmospheric 40/36(a) = **295.50**
Atmospheric 38/36(a) = **0.1869**
Production 39/37(ca) = **0.0006425 ± 0.0000059**
Production 38/37(ca) = **0.0001800 ± 0.0000173**
Production 36/37(ca) = **0.0002703 ± 0.0000005**
Production 40/39(k) = **0.000607 ± 0.000059**
Production 38/39(k) = **0.012077 ± 0.000011**
Production 36/38(cl) = **262.80 ± 1.71**
Scaling Ratio K/Ca = **0.430**
Abundance Ratio 40K/K = **1.1700 ± 0.0100 E-04**
Atomic Weight K = **39.0983 ± 0.0001 g**

Results	40(a)/36(a) ± 2σ	40(r)/39(k) ± 2σ	Age ± 2σ (ka)	MSWD	39Ar(k) (%,n)	K/Ca ± 2σ
**Age Plateau**		0.22092 ± 0.02266	**661.3 ± 67.8**	2.40	96.09	0.0155 ± 0.0036
Error Mean		± 10.26%	**± 10.26%**	0%	20	
			Full External Error ± 69.5	1.65	2σ Confidence Limit	
			Analytical Error ± 67.8	1.5478	Error Magnification	
**Total Fusion Age**		0.22748 ± 0.02223	**681.0 ± 66.5**		24	0.0250 ± 0.0001
		± 9.77%	**± 9.77%**			
			Full External Error ± 68.3			
			Analytical Error ± 66.5			
**Normal Isochron**	**296.47 ± 2.65**	0.20194 ± 0.05366	**604.5 ± 160.6**	2.48	96.09	
Error Chron	**± 0.90%**	± 26.57%	**± 26.57%**	0%	20	
			Full External Error ± 161.2	1.67	2σ Confidence Limit	
			Analytical Error ± 160.6	1.5736	Error Magnification	
				3	Number of Iterations	
				0.0000003535	Convergence	
**Inverse Isochron**	**296.48 ± 2.65**	0.20304 ± 0.04636	**607.8 ± 138.8**	2.45	96.09	
Error Chron	**± 0.89%**	± 22.83%	**± 22.83%**	0%	20	
			Full External Error ± 139.4	1.67	2σ Confidence Limit	
			Analytical Error ± 138.8	1.5662	Error Magnification	
				3	Number of Iterations	
				0.0000017022	Convergence	
				4%	Spreading Factor	

Notes
Mostly atmospheric

Incremental Heating			36Ar(a) [fA]	37Ar(ca) [fA]	38Ar(cl) [fA]	39Ar(k) [fA]	40Ar(r) [fA]	Age ± 2σ (ka)	40Ar(r) (%)	39Ar(k) (%)	K/Ca ± 2σ
17D19872	1.8 %	✓	2.8054172	143.2736	0.1199447	61.70764	14.057877	682.0 ± 235.2	1.67	23.73	0.1852 ± 0.0020
17D19874	1.9 %	✓	0.5229131	60.8876	0.0137362	18.55086	5.294788	854.4 ± 182.1	3.31	7.13	0.1310 ± 0.0023
17D19875	2.0 %	✓	0.3448688	84.0488	0.0006020	15.75171	5.075923	964.6 ± 159.2	4.74	6.06	0.0806 ± 0.0012
17D19877	2.2 %	✓	0.1998634	67.3714	0.0115392	10.04646	2.467815	735.3 ± 193.5	4.01	3.86	0.0641 ± 0.0010
17D19878	2.4 %	✓	0.1189412	50.1399	0.0000000	6.42557	1.379251	642.6 ± 210.5	3.78	2.47	0.0551 ± 0.0011
17D19880	2.7 %	✓	0.2173657	155.3400	0.0000000	13.80567	3.242152	703.0 ± 149.6	4.80	5.31	0.0382 ± 0.0004
17D19881	3.0 %	✓	0.0751078	51.7774	0.0000000	4.55877	0.611280	401.4 ± 255.7	2.68	1.75	0.0379 ± 0.0008
17D19883	3.4 %	✓	0.2141927	247.2287	0.0000000	15.82503	3.438066	650.4 ± 136.5	5.15	6.09	0.0275 ± 0.0003
17D19884	3.9 %	✓	0.1894840	293.5956	0.0000000	15.19367	3.040129	599.0 ± 144.2	5.15	5.84	0.0223 ± 0.0002
17D19886	4.5 %	✓	0.1653594	317.9594	0.0000000	13.99585	2.896394	619.5 ± 150.1	5.59	5.38	0.0189 ± 0.0002
17D19887	5.2 %	✓	0.1431263	320.1407	0.0000000	12.52195	2.609047	623.7 ± 161.6	5.81	4.82	0.0168 ± 0.0002
17D19889	6.0 %	✓	0.1268665	293.1296	0.0000000	10.65058	2.799946	787.0 ± 181.2	6.95	4.10	0.0156 ± 0.0002
17D19890	6.9 %	✓	0.0859292	186.6720	0.0000000	6.45214	0.748887	347.5 ± 221.1	2.86	2.48	0.0149 ± 0.0002
17D19892	7.9 %	✓	0.1137447	224.4846	0.0000000	7.76593	1.706473	657.8 ± 207.0	4.83	2.99	0.0149 ± 0.0002
17D19893	9.0 %	✓	0.1285786	214.2641	0.0000000	7.45530	1.603323	643.8 ± 223.4	4.05	2.87	0.0150 ± 0.0002
17D19895	10.3 %	✓	0.1696349	225.7090	0.0000000	7.77473	0.735360	283.2 ± 246.0	1.45	2.99	0.0148 ± 0.0002
17D19896	11.6 %	✓	0.2438102	238.7690	0.0000000	7.62553	1.649257	647.5 ± 305.6	2.24	2.93	0.0137 ± 0.0002
17D19898	12.5 %	✓	0.1825033	172.1995	0.0000000	5.41509	1.158857	640.6 ± 339.6	2.10	2.08	0.0135 ± 0.0002
17D19899	13.4 %	✓	0.2014588	175.1667	0.0064606	4.28854	0.840663	586.8 ± 430.2	1.39	1.65	0.0105 ± 0.0002
17D19901	14.6 %	✓	0.2191272	192.3469	0.0016320	4.03191	0.639420	474.8 ± 517.9	0.98	1.55	0.0090 ± 0.0001
17D19902	16.0 %		0.1960890	252.8763	0.0000000	3.84753	1.517112	1180.2 ± 541.0	2.55	1.48	0.0065 ± 0.0001
17D19904	17.6 %		0.1071435	180.1995	0.0000000	2.46675	0.632610	767.7 ± 595.3	1.96	0.95	0.0059 ± 0.0001
17D19905	19.3 %		0.0767209	142.2285	0.0176575	1.83888	0.320643	522.0 ± 700.3	1.39	0.71	0.0056 ± 0.0001
17D19907	21.0 %		0.0901128	182.0193	0.0073307	2.01303	0.680806	1012.3 ± 747.3	2.49	0.77	0.0048 ± 0.0001
		Σ	6.9383591	4471.8283	0.1789029	260.00915	59.146080				

Information on Analysis

Project = **O-CONNOR (16-22)**
Sample = **PS59-311-1**
Material = **Groundmass**
Location = **Gakkel Ridge**
Region = **Artic Ocean**
Analyst = **Dan Miggins**
Irradiation = **17-OSU-01 (1C8-17)**
J = **0.00165575 ± 0.00000149**
FCT-NM = **28.201 ± 0.023 Ma**

Results	40(r)/39(k) ± 2σ		Age ± 2σ (ka)		MSWD	39Ar(k) (%,n)	K/Ca ± 2σ
**Age Plateau**	0.22092	± 0.02266<br>± 10.26%	**661.3**	**± 67.8**<br>**± 10.26%**	2.40<br>0%	96.09<br>20	0.0155 ± 0.0036
Error Mean			Full External Error	± 69.5	1.65	2σ Confidence Limit	
			Analytical Error	± 67.8	1.5478	Error Magnification	
**Total Fusion Age**	0.22748	± 0.02223<br>± 9.77%	**681.0**	**± 66.5**<br>**± 9.77%**		24	0.0250 ± 0.0001
			Full External Error	± 68.3			
			Analytical Error	± 66.5			

Normal Isochron			39(k)/36(a) ± 2σ	40(a+r)/36(a) ± 2σ	r.i.
17D19872	1.8 %	✓	22.00 ± 0.13	300.51 ± 1.76	0.9649
17D19874	1.9 %	✓	35.48 ± 0.28	305.63 ± 2.23	0.9267
17D19875	2.0 %	✓	45.67 ± 0.40	310.22 ± 2.55	0.9251
17D19877	2.2 %	✓	50.27 ± 0.60	307.85 ± 3.38	0.9168
17D19878	2.4 %	✓	54.02 ± 0.79	307.10 ± 3.95	0.8639
17D19880	2.7 %	✓	63.51 ± 0.72	310.42 ± 3.33	0.9456
17D19881	3.0 %	✓	60.70 ± 1.23	303.64 ± 5.32	0.8540
17D19883	3.4 %	✓	73.88 ± 0.87	311.55 ± 3.55	0.9623
17D19884	3.9 %	✓	80.18 ± 1.08	311.54 ± 4.07	0.9655
17D19886	4.5 %	✓	84.64 ± 1.25	313.02 ± 4.49	0.9690
17D19887	5.2 %	✓	87.49 ± 1.44	313.73 ± 5.01	0.9660
17D19889	6.0 %	✓	83.95 ± 1.48	317.57 ± 5.46	0.9656
17D19890	6.9 %	✓	75.09 ± 1.50	304.22 ± 5.71	0.9297
17D19892	7.9 %	✓	68.28 ± 1.16	310.50 ± 4.96	0.9313
17D19893	9.0 %	✓	57.98 ± 0.92	307.97 ± 4.51	0.9151
17D19895	10.3 %	✓	45.83 ± 0.65	299.83 ± 3.82	0.8973
17D19896	11.6 %	✓	31.28 ± 0.39	302.26 ± 3.27	0.8667
17D19898	12.5 %	✓	29.67 ± 0.43	301.85 ± 3.44	0.7900
17D19899	13.4 %	✓	21.29 ± 0.31	299.67 ± 3.10	0.6959
17D19901	14.6 %	✓	18.40 ± 0.29	298.42 ± 3.21	0.6866
17D19902	16.0 %		19.62 ± 0.33	303.24 ± 3.64	0.7157
17D19904	17.6 %		23.02 ± 0.54	301.40 ± 4.67	0.6504
17D19905	19.3 %		23.97 ± 0.74	299.68 ± 5.69	0.6067
17D19907	21.0 %		22.34 ± 0.65	303.06 ± 5.72	0.6384

Results	40(a)/36(a) ± 2σ		40(r)/39(k) ± 2σ		Age ± 2σ (ka)		MSWD
**Normal Isochron**<br>Error Chron	**296.47**	**± 2.65**<br>**± 0.90%**	0.20194	± 0.05366<br>± 26.57%	**604.5**	**± 160.6**<br>**± 26.57%**	2.48<br>0%
					Full External Error	± 161.2	
					Analytical Error	± 160.6	

Statistics

2σ Confidence Limit	1.67	Convergence	0.000000353532
Error Magnification	1.5736	Number of Iterations	3
Number of Data Points	20	Calculated Line	Weighted York-2

Inverse Isochron			39(k)/40(a+r) ± 2σ	36(a)/40(a+r) ± 2σ	r.i.
17D19872	1.8 %	✓	0.0731950 ± 0.0001164	0.00332767 ± 0.00001946	0.0017
17D19874	1.9 %	✓	0.1160766 ± 0.0003405	0.00327198 ± 0.00002389	0.0237
17D19875	2.0 %	✓	0.1472334 ± 0.0004933	0.00322354 ± 0.00002650	0.0175
17D19877	2.2 %	✓	0.1632842 ± 0.0007764	0.00324836 ± 0.00003571	0.0158
17D19878	2.4 %	✓	0.1759159 ± 0.0013023	0.00325631 ± 0.00004183	0.0202
17D19880	2.7 %	✓	0.2046081 ± 0.0007510	0.00322149 ± 0.00003459	0.0186
17D19881	3.0 %	✓	0.1998965 ± 0.0021007	0.00329339 ± 0.00005774	0.0272
17D19883	3.4 %	✓	0.2371431 ± 0.0007597	0.00320974 ± 0.00003658	0.0189
17D19884	3.9 %	✓	0.2573774 ± 0.0009020	0.00320982 ± 0.00004196	0.0208
17D19886	4.5 %	✓	0.2703984 ± 0.0009855	0.00319473 ± 0.00004587	0.0202
17D19887	5.2 %	✓	0.2788675 ± 0.0011867	0.00318746 ± 0.00005092	0.0211
17D19889	6.0 %	✓	0.2643546 ± 0.0012162	0.00314891 ± 0.00005413	0.0242
17D19890	6.9 %	✓	0.2468212 ± 0.0018145	0.00328715 ± 0.00006167	0.0280
17D19892	7.9 %	✓	0.2198857 ± 0.0013618	0.00322058 ± 0.00005142	0.0214
17D19893	9.0 %	✓	0.1882733 ± 0.0012050	0.00324707 ± 0.00004754	0.0180
17D19895	10.3 %	✓	0.1528580 ± 0.0009510	0.00333517 ± 0.00004250	0.0152
17D19896	11.6 %	✓	0.1034739 ± 0.0006405	0.00330836 ± 0.00003574	0.0078
17D19898	12.5 %	✓	0.0982979 ± 0.0008634	0.00331291 ± 0.00003773	0.0080
17D19899	13.4 %	✓	0.0710357 ± 0.0007526	0.00333697 ± 0.00003454	0.0080
17D19901	14.6 %	✓	0.0616580 ± 0.0006990	0.00335100 ± 0.00003610	0.0057
17D19902	16.0 %		0.0647063 ± 0.0007536	0.00329775 ± 0.00003958	0.0057
17D19904	17.6 %		0.0763853 ± 0.0013687	0.00331780 ± 0.00005139	0.0084
17D19905	19.3 %		0.0799803 ± 0.0019627	0.00333690 ± 0.00006331	0.0099
17D19907	21.0 %		0.0737128 ± 0.0016611	0.00329973 ± 0.00006225	0.0078

Results	40(a)/36(a) ± 2σ	40(r)/39(k) ± 2σ	Age ± 2σ (ka)	MSWD
**Inverse Isochron**<br>Error Chron	**296.48** **± 2.65**<br>**± 0.89%**	0.20304 ± 0.04636<br>± 22.83%	**607.8** **± 138.8**<br>**± 22.83%**<br>Full External Error ± 139.4<br>Analytical Error ± 138.8	2.45<br>0%

Statistics				
	2σ Confidence Limit	1.67	Convergence	0.0000017022
	Error Magnification	1.5662	Number of Iterations	3
	Number of Data Points	20	Calculated Line	Weighted York-2
	Spreading Factor	4.4%		

Degassing Patterns			36Ar(a) [fA]	%1σ	36Ar(c) [fA]	%1σ	36Ar(ca) [fA]	%1σ	36Ar(cl) [fA]	%1σ	37Ar(ca) [fA]	%1σ	38Ar(a) [fA]	%1σ	38Ar(c) [fA]	%1σ	38Ar(k) [fA]	%1σ	38Ar(ca) [fA]	%1σ	38Ar(cl) [fA]	%1σ	39Ar(k) [fA]	%1σ	39Ar(ca) [fA]	%1σ	40Ar(r) [fA]	%1σ	40Ar(a) [fA]	%1σ	40Ar(c) [fA]	%1σ	40Ar(k) [fA]	%1σ
17D19872	1.8 %	✓	2.8054172	0.29	0.0000000	0.00	0.0387269	0.57	0.0000277	19.38	143.2736	0.54	0.5243325	0.29	0.0000000	0.00	0.7452432	0.12	0.0257893	9.65	0.1199447	19.40	61.70764	0.08	0.0920533	1.07	14.057877	17.24	829.0008	0.29	0.0000000	0.00	0.0374565	9.65
17D19874	1.9 %	✓	0.5229131	0.36	0.0000000	0.00	0.0164579	0.87	0.0000032	171.14	60.8876	0.86	0.0977325	0.36	0.0000000	0.00	0.2240387	0.17	0.0109598	9.67	0.0137362	171.14	18.55086	0.14	0.0391203	1.26	5.294788	10.66	154.5208	0.36	0.0000000	0.00	0.0112604	9.65
17D19875	2.0 %	✓	0.3448688	0.41	0.0000000	0.00	0.0227184	0.72	0.0000001	######	84.0488	0.70	0.0644560	0.41	0.0000000	0.00	0.1902334	0.19	0.0151288	9.66	0.0006020	######	15.75171	0.16	0.0540013	1.16	5.075923	8.26	101.9087	0.41	0.0000000	0.00	0.0095613	9.65
17D19877	2.2 %	✓	0.1998634	0.55	0.0000000	0.00	0.0182105	0.78	0.0000027	206.82	67.3714	0.76	0.0373545	0.55	0.0000000	0.00	0.1213311	0.25	0.0121269	9.66	0.0115392	206.82	10.04646	0.23	0.0432862	1.20	2.467815	13.16	59.0596	0.55	0.0000000	0.00	0.0060982	9.65
17D19878	2.4 %	✓	0.1189412	0.64	0.0000000	0.00	0.0135528	0.99	0.0000000	0.00	50.1399	0.97	0.0222301	0.64	0.0000000	0.00	0.0776016	0.37	0.0090252	9.68	0.0000000	0.00	6.42557	0.36	0.0322149	1.34	1.379251	16.38	35.1471	0.64	0.0000000	0.00	0.0039003	9.66
17D19880	2.7 %	✓	0.2173657	0.54	0.0000000	0.00	0.0419884	0.56	0.0000000	0.00	155.3400	0.53	0.0406256	0.54	0.0000000	0.00	0.1667311	0.20	0.0279612	9.64	0.0000000	0.00	13.80567	0.18	0.0998059	1.06	3.242152	10.64	64.2316	0.54	0.0000000	0.00	0.0083800	9.65
17D19881	3.0 %	✓	0.0751078	0.87	0.0000000	0.00	0.0139954	1.00	0.0000000	0.00	51.7774	0.98	0.0140376	0.87	0.0000000	0.00	0.0550562	0.52	0.0093199	9.68	0.0000000	0.00	4.55877	0.51	0.0332670	1.34	0.611280	31.84	22.1943	0.87	0.0000000	0.00	0.0027672	9.66
17D19883	3.4 %	✓	0.2141927	0.57	0.0000000	0.00	0.0668259	0.51	0.0000000	0.00	247.2287	0.49	0.0400326	0.57	0.0000000	0.00	0.1911189	0.18	0.0445012	9.64	0.0000000	0.00	15.82503	0.15	0.1588444	1.04	3.438066	10.49	63.2940	0.57	0.0000000	0.00	0.0096058	9.65
17D19884	3.9 %	✓	0.1894840	0.65	0.0000000	0.00	0.0793589	0.51	0.0000000	0.00	293.5956	0.48	0.0354146	0.65	0.0000000	0.00	0.1834939	0.19	0.0528472	9.64	0.0000000	0.00	15.19367	0.17	0.1886352	1.04	3.040129	12.04	55.9925	0.65	0.0000000	0.00	0.0092226	9.65
17D19886	4.5 %	✓	0.1653594	0.72	0.0000000	0.00	0.0859444	0.50	0.0000000	0.00	317.9594	0.47	0.0309057	0.72	0.0000000	0.00	0.1690279	0.20	0.0572327	9.64	0.0000000	0.00	13.99585	0.17	0.2042889	1.03	2.896394	12.12	48.8637	0.72	0.0000000	0.00	0.0084955	9.65
17D19887	5.2 %	✓	0.1431263	0.80	0.0000000	0.00	0.0865340	0.50	0.0000000	0.00	320.1407	0.47	0.0267503	0.80	0.0000000	0.00	0.1512276	0.22	0.0576253	9.64	0.0000000	0.00	12.52195	0.20	0.2056904	1.03	2.609047	12.95	42.2938	0.80	0.0000000	0.00	0.0076008	9.65
17D19889	6.0 %	✓	0.1268665	0.86	0.0000000	0.00	0.0792329	0.51	0.0000000	0.00	293.1296	0.48	0.0237113	0.86	0.0000000	0.00	0.1286270	0.24	0.0527633	9.64	0.0000000	0.00	10.65058	0.22	0.1883357	1.04	2.799946	11.51	37.4890	0.86	0.0000000	0.00	0.0064649	9.65
17D19890	6.9 %	✓	0.0859292	0.93	0.0000000	0.00	0.0504574	0.53	0.0000000	0.00	186.6720	0.50	0.0160602	0.93	0.0000000	0.00	0.0779225	0.37	0.0336010	9.64	0.0000000	0.00	6.45214	0.35	0.1199368	1.05	0.748887	31.81	25.3921	0.93	0.0000000	0.00	0.0039165	9.66
17D19892	7.9 %	✓	0.1137447	0.80	0.0000000	0.00	0.0606782	0.52	0.0000000	0.00	224.4846	0.49	0.0212589	0.80	0.0000000	0.00	0.0937892	0.31	0.0404072	9.64	0.0000000	0.00	7.76593	0.30	0.1442314	1.04	1.706473	15.73	33.6116	0.80	0.0000000	0.00	0.0047139	9.65
17D19893	9.0 %	✓	0.1285786	0.73	0.0000000	0.00	0.0579156	0.52	0.0000000	0.00	214.2641	0.50	0.0240313	0.73	0.0000000	0.00	0.0900377	0.33	0.0385675	9.64	0.0000000	0.00	7.45530	0.31	0.1376647	1.05	1.603323	17.35	37.9950	0.73	0.0000000	0.00	0.0045254	9.66
17D19895	10.3 %	✓	0.1696349	0.63	0.0000000	0.00	0.0610092	0.52	0.0000000	0.00	225.7090	0.50	0.0317048	0.63	0.0000000	0.00	0.0938955	0.32	0.0406276	9.64	0.0000000	0.00	7.77473	0.31	0.1450181	1.04	0.735360	43.44	50.1271	0.63	0.0000000	0.00	0.0047193	9.65
17D19896	11.6 %	✓	0.2438102	0.54	0.0000000	0.00	0.0645393	0.52	0.0000000	0.00	238.7690	0.49	0.0455681	0.54	0.0000000	0.00	0.0920935	0.32	0.0429784	9.64	0.0000000	0.00	7.62553	0.31	0.1534091	1.04	1.649257	23.60	72.0459	0.54	0.0000000	0.00	0.0046287	9.65
17D19898	12.5 %	✓	0.1825033	0.57	0.0000000	0.00	0.0465455	0.54	0.0000000	0.00	172.1995	0.52	0.0341099	0.57	0.0000000	0.00	0.0653981	0.45	0.0309959	9.64	0.0000000	0.00	5.41509	0.44	0.1106382	1.05	1.158857	26.50	53.9297	0.57	0.0000000	0.00	0.0032870	9.66
17D19899	13.4 %	✓	0.2014588	0.52	0.0000000	0.00	0.0473476	0.55	0.0000015	374.99	175.1667	0.52	0.0376526	0.52	0.0000000	0.00	0.0517928	0.54	0.0315300	9.64	0.0064606	374.99	4.28854	0.53	0.1125446	1.06	0.840663	36.66	59.5311	0.52	0.0000000	0.00	0.0026031	9.66
17D19901	14.6 %	✓	0.2191272	0.54	0.0000000	0.00	0.0519914	0.53	0.0000004	######	192.3469	0.51	0.0409549	0.54	0.0000000	0.00	0.0486934	0.57	0.0346224	9.64	0.0016320	######	4.03191	0.57	0.1235829	1.05	0.639420	54.55	64.7521	0.54	0.0000000	0.00	0.0024474	9.67
17D19902	16.0 %		0.1960890	0.60	0.0000000	0.00	0.0683525	0.51	0.0000000	0.00	252.8763	0.48	0.0366490	0.60	0.0000000	0.00	0.0464666	0.59	0.0455177	9.64	0.0000000	0.00	3.84753	0.58	0.1624731	1.04	1.517112	22.92	57.9443	0.60	0.0000000	0.00	0.0023355	9.67
17D19904	17.6 %		0.1071435	0.77	0.0000000	0.00	0.0487079	0.54	0.0000000	0.00	180.1995	0.51	0.0200251	0.77	0.0000000	0.00	0.0297909	0.90	0.0324359	9.64	0.0000000	0.00	2.46675	0.89	0.1157782	1.05	0.632610	38.77	31.6609	0.77	0.0000000	0.00	0.0014973	9.69
17D19905	19.3 %		0.0767209	0.94	0.0000000	0.00	0.0384444	0.57	0.0000041	134.21	142.2285	0.54	0.0143391	0.94	0.0000000	0.00	0.0222082	1.23	0.0256011	9.65	0.0176575	134.22	1.83888	1.22	0.0913818	1.07	0.320643	67.08	22.6710	0.94	0.0000000	0.00	0.0011162	9.73
17D19907	21.0 %		0.0901128	0.94	0.0000000	0.00	0.0491998	0.54	0.0000017	315.11	182.0193	0.51	0.0168421	0.94	0.0000000	0.00	0.0243114	1.13	0.0327635	9.64	0.0073307	315.11	2.01303	1.12	0.1169474	1.05	0.680806	36.90	26.6283	0.94	0.0000000	0.00	0.0012219	9.72
		Σ	6.9383591	0.14	0.0000000	0.00	1.2087352	0.12	0.0000414	37.59	4471.8283	0.11	1.2967793	0.14	0.0000000	0.00	3.1401305	0.05	0.8049291	2.14	0.1789029	37.58	260.00915	0.05	2.8731497	0.23	59.146080	4.89	2050.2851	0.14	0.0000000	0.00	0.1578256	2.89
		Σ							8.1471357	0.12	4471.8283	0.11									5.4207418	1.28			262.88230	0.05							2109.5890	0.19

**Additional Parameters**			40Ar/39Ar	1σ	37Ar/39Ar	1σ	36Ar/39Ar	1σ	Time (days)	37Ar (decay)	39Ar (decay)	40Ar (moles)
17D19872	1.8 %	✓	13.642400	0.010830	2.318355	0.012687	0.046022	0.000138	142.384	16.690460	1.00100605	4.047E-11
17D19874	1.9 %	✓	8.597474	0.012581	3.275293	0.028432	0.029014	0.000110	142.398	16.695039	1.00100615	7.672E-12
17D19875	2.0 %	✓	6.769338	0.011299	5.317618	0.038210	0.023257	0.000097	142.405	16.697330	1.00100620	5.136E-12
17D19877	2.2 %	✓	6.098623	0.014437	6.677220	0.053342	0.021614	0.000119	142.419	16.701911	1.00100629	2.954E-12
17D19878	2.4 %	✓	5.656781	0.020835	7.764260	0.080695	0.020517	0.000138	142.426	16.704202	1.00100634	1.753E-12
17D19880	2.7 %	✓	4.852915	0.008837	11.171138	0.062938	0.018651	0.000088	142.440	16.708785	1.00100644	3.239E-12
17D19881	3.0 %	✓	4.966949	0.025914	11.275481	0.124586	0.019404	0.000171	142.447	16.711077	1.00100649	1.095E-12
17D19883	3.4 %	✓	4.175559	0.006612	15.467376	0.078764	0.017581	0.000078	142.460	16.715662	1.00100659	3.204E-12
17D19884	3.9 %	✓	3.838299	0.006631	19.086587	0.096316	0.017477	0.000081	142.467	16.717955	1.00100664	2.834E-12
17D19886	4.5 %	✓	3.645640	0.006533	22.391286	0.112560	0.017697	0.000083	142.481	16.722542	1.00100674	2.485E-12
17D19887	5.2 %	✓	3.528577	0.007372	25.153178	0.129212	0.018044	0.000090	142.488	16.724836	1.00100679	2.156E-12
17D19889	6.0 %	✓	3.717665	0.008390	27.044185	0.141680	0.019015	0.000102	142.503	16.729654	1.00100689	1.934E-12
17D19890	6.9 %	✓	3.978174	0.014354	28.403789	0.173008	0.020752	0.000136	142.512	16.732638	1.00100695	1.255E-12
17D19892	7.9 %	✓	4.465489	0.013563	28.379264	0.162638	0.022050	0.000125	142.526	16.737229	1.00100705	1.695E-12
17D19893	9.0 %	✓	5.215724	0.016371	28.218760	0.164758	0.024561	0.000139	142.533	16.739525	1.00100710	1.901E-12
17D19895	10.3 %	✓	6.422825	0.019587	28.499507	0.164984	0.029123	0.000156	142.547	16.744117	1.00100720	2.442E-12
17D19896	11.6 %	✓	9.474277	0.028688	30.694296	0.175813	0.039639	0.000202	142.553	16.746414	1.00100725	3.538E-12
17D19898	12.5 %	✓	9.970057	0.042866	31.163205	0.208685	0.041451	0.000254	142.567	16.751009	1.00100734	2.644E-12
17D19899	13.4 %	✓	13.718041	0.070727	39.800757	0.290525	0.056533	0.000369	142.574	16.753307	1.00100739	2.898E-12
17D19901	14.6 %	✓	15.736740	0.086428	46.287342	0.345246	0.065243	0.000451	142.588	16.757904	1.00100749	3.139E-12
17D19902	16.0 %		14.828851	0.082635	63.061374	0.464219	0.065945	0.000461	142.595	16.760202	1.00100754	2.854E-12
17D19904	17.6 %		12.505198	0.106890	69.776451	0.694127	0.060348	0.000596	142.609	16.764801	1.00100764	1.550E-12
17D19905	19.3 %		11.911737	0.139164	73.683512	0.945469	0.059665	0.000781	142.616	16.767101	1.00100769	1.104E-12
17D19907	21.0 %		12.821879	0.136389	85.455847	1.006265	0.065406	0.000789	142.630	16.771701	1.00100779	1.311E-12

Procedure Blanks		36Ar ± 1σ (SE) [fA]		37Ar ± 1σ (SE) [fA]		38Ar ± 1σ (SE) [fA]		39Ar ± 1σ (SE) [fA]		40Ar ± 1σ (SE) [fA]	
17D19872	1.8 %	0.0082921	± 0.0002665	0.1137869	± 0.0177005	0.0344109	± 0.0170171	0.0211092	± 0.0156056	2.4191750	± 0.0167414
17D19874	1.9 %	0.0081485	± 0.0002665	0.1404421	± 0.0177005	0.0392569	± 0.0170171	0.0335692	± 0.0156056	2.3889701	± 0.0167414
17D19875	2.0 %	0.0080866	± 0.0002665	0.1496980	± 0.0177005	0.0408436	± 0.0170171	0.0368434	± 0.0156056	2.3773379	± 0.0167414
17D19877	2.2 %	0.0079820	± 0.0002665	0.1615814	± 0.0177005	0.0426807	± 0.0170171	0.0387664	± 0.0156056	2.3600318	± 0.0167414
17D19878	2.4 %	0.0079390	± 0.0002665	0.1647674	± 0.0177005	0.0430549	± 0.0170171	0.0378871	± 0.0156056	2.3539866	± 0.0167414
17D19880	2.7 %	0.0078710	± 0.0002665	0.1669059	± 0.0177005	0.0429987	± 0.0170171	0.0335214	± 0.0156056	2.3462388	± 0.0167414
17D19881	3.0 %	0.0078456	± 0.0002665	0.1663218	± 0.0177005	0.0426712	± 0.0170171	0.0304221	± 0.0156056	2.3442085	± 0.0167414
17D19883	3.4 %	0.0078113	± 0.0002665	0.1628870	± 0.0177005	0.0416492	± 0.0170171	0.0232530	± 0.0156056	2.3430703	± 0.0167414
17D19884	3.9 %	0.0078019	± 0.0002665	0.1604048	± 0.0177005	0.0410368	± 0.0170171	0.0194857	± 0.0156056	2.3436783	± 0.0167414
17D19886	4.5 %	0.0077975	± 0.0002665	0.1547135	± 0.0177005	0.0397883	± 0.0170171	0.0122358	± 0.0156056	2.3465927	± 0.0167414
17D19887	5.2 %	0.0078020	± 0.0002665	0.1517777	± 0.0177005	0.0392136	± 0.0170171	0.0089709	± 0.0156056	2.3486584	± 0.0167414
17D19889	6.0 %	0.0078244	± 0.0002665	0.1460403	± 0.0177005	0.0382540	± 0.0170171	0.0033792	± 0.0156056	2.3537129	± 0.0167414
17D19890	6.9 %	0.0078462	± 0.0002665	0.1430618	± 0.0177005	0.0378920	± 0.0170171	0.0009860	± 0.0156056	2.3570117	± 0.0167414
17D19892	7.9 %	0.0078896	± 0.0002665	0.1397030	± 0.0177005	0.0377656	± 0.0170171	0.0008505	± 0.0156056	2.3618296	± 0.0167414
17D19893	9.0 %	0.0079151	± 0.0002665	0.1386586	± 0.0177005	0.0379166	± 0.0170171	0.0008881	± 0.0156056	2.3639617	± 0.0167414
17D19895	10.3 %	0.0079719	± 0.0002665	0.1378975	± 0.0177005	0.0386626	± 0.0170171	0.0007844	± 0.0156056	2.3673573	± 0.0167414
17D19896	11.6 %	0.0080024	± 0.0002665	0.1381567	± 0.0177005	0.0392533	± 0.0170171	0.0024466	± 0.0156056	2.3685165	± 0.0167414
17D19898	12.5 %	0.0080656	± 0.0002665	0.1397751	± 0.0177005	0.0408336	± 0.0170171	0.0071972	± 0.0156056	2.3695520	± 0.0167414
17D19899	13.4 %	0.0080975	± 0.0002665	0.1410151	± 0.0177005	0.0417979	± 0.0170171	0.0101530	± 0.0156056	2.3693675	± 0.0167414
17D19901	14.6 %	0.0081596	± 0.0002665	0.1439398	± 0.0177005	0.0439863	± 0.0170171	0.0167876	± 0.0156056	2.3674970	± 0.0167414
17D19902	16.0 %	0.0081890	± 0.0002665	0.1454102	± 0.0177005	0.0451642	± 0.0170171	0.0202492	± 0.0156056	2.3657938	± 0.0167414
17D19904	17.6 %	0.0082417	± 0.0002665	0.1477127	± 0.0177005	0.0475464	± 0.0170171	0.0268111	± 0.0156056	2.3608630	± 0.0167414
17D19905	19.3 %	0.0082641	± 0.0002665	0.1482355	± 0.0177005	0.0486838	± 0.0170171	0.0296092	± 0.0156056	2.3576619	± 0.0167414
17D19907	21.0 %	0.0082984	± 0.0002665	0.1471323	± 0.0177005	0.0506575	± 0.0170171	0.0333784	± 0.0156056	2.3499086	± 0.0167414

**Intercept Values**		36Ar ± 1σ (SE) [fA]	r2	Regression (type,n)	37Ar ± 1σ (SE) [fA]	r2	Regression (type,n)	38Ar ± 1σ (SE) [fA]	r2	Regression (type,n)	39Ar ± 1σ (SE) [fA]	r2	Regression (type,n)	40Ar ± 1σ (SE) [fA]	r2	Regression (type,n)
17D19872	1.8 %	2.7458039 ± 0.0024154	0.9843	EXP 150 of 150	8.321526 ± 0.017963	0.8770	EXP 150 of 150	1.3645163 ± 0.0149751	0.2661	EXP 150 of 150	61.3597907 ± 0.0201946	0.9974	EXP 150 of 150	845.515289 ± 0.050918	0.9996	EXP 150 of 150
17D19874	1.9 %	0.5272955 ± 0.0011071	0.8854	EXP 150 of 150	3.443366 ± 0.019152	0.6278	EXP 150 of 150	0.3031999 ± 0.0157698	0.0000	EXP 150 of 150	18.4304314 ± 0.0169914	0.9763	EXP 150 of 150	162.215834 ± 0.054441	0.0022	EXP 150 of 150
17D19875	2.0 %	0.3618890 ± 0.0009005	0.7667	EXP 150 of 150	4.796681 ± 0.019645	0.6944	EXP 150 of 150	0.2264465 ± 0.0185269	0.0000	EXP 150 of 150	15.6617592 ± 0.0173710	0.9677	EXP 150 of 150	109.371562 ± 0.033110	0.9755	EXP 150 of 150
17D19877	2.2 %	0.2178804 ± 0.0008301	0.5399	EXP 150 of 150	3.802229 ± 0.016859	0.6496	EXP 150 of 150	0.1375602 ± 0.0162869	0.0028	EXP 150 of 150	9.9826021 ± 0.0159041	0.9374	EXP 150 of 150	63.893582 ± 0.022413	0.9951	EXP 150 of 150
17D19878	2.4 %	0.1354643 ± 0.0005697	0.1070	EXP 150 of 150	2.784820 ± 0.018308	0.4424	EXP 149 of 150	0.0319867 ± 0.0164032	0.0163	EXP 150 of 150	6.3761356 ± 0.0166190	0.8316	EXP 150 of 150	38.884273 ± 0.018953	0.9977	EXP 150 of 150
17D19880	2.7 %	0.2574990 ± 0.0008137	0.6822	EXP 150 of 150	8.968788 ± 0.019294	0.8726	EXP 149 of 150	0.1870032 ± 0.0183351	0.0050	EXP 150 of 150	13.7777164 ± 0.0163591	0.9635	EXP 150 of 150	69.828330 ± 0.023531	0.9930	EXP 150 of 150
17D19881	3.0 %	0.0936074 ± 0.0005007	0.0007	EXP 150 of 150	2.878338 ± 0.019714	0.4431	EXP 150 of 150	0.0334746 ± 0.0154420	0.0000	EXP 150 of 150	4.5304907 ± 0.0169462	0.6250	EXP 150 of 150	25.152605 ± 0.019282	0.9979	EXP 150 of 150
17D19883	3.4 %	0.2782914 ± 0.0008011	0.7136	EXP 150 of 150	14.370887 ± 0.019023	0.9479	EXP 150 of 150	0.1964935 ± 0.0161211	0.0010	EXP 150 of 150	15.8523005 ± 0.0151923	0.9760	EXP 150 of 150	69.084693 ± 0.022026	0.9931	EXP 150 of 150
17D19884	3.9 %	0.2665629 ± 0.0008299	0.7405	EXP 150 of 150	17.096767 ± 0.019412	0.9632	EXP 149 of 150	0.1752590 ± 0.0141741	0.0057	EXP 150 of 150	15.2585672 ± 0.0170813	0.9697	EXP 150 of 150	61.385548 ± 0.023458	0.9939	EXP 150 of 150
17D19886	4.5 %	0.2496772 ± 0.0007845	0.6524	EXP 150 of 150	18.529403 ± 0.018891	0.9690	EXP 150 of 150	0.1814260 ± 0.0173640	0.0052	EXP 150 of 150	14.0916669 ± 0.0158879	0.9669	EXP 150 of 150	54.115198 ± 0.020695	0.9953	EXP 150 of 150
17D19887	5.2 %	0.2288498 ± 0.0007683	0.6683	EXP 149 of 150	18.657935 ± 0.019647	0.9676	EXP 150 of 150	0.1907968 ± 0.0170686	0.0301	EXP 150 of 150	12.6324126 ± 0.0180080	0.9481	EXP 150 of 150	47.259128 ± 0.021063	0.9959	EXP 150 of 150
17D19889	6.0 %	0.2061948 ± 0.0007601	0.5442	EXP 150 of 150	17.071687 ± 0.020122	0.9633	EXP 150 of 150	0.1447976 ± 0.0168172	0.0133	EXP 150 of 150	10.7620732 ± 0.0154475	0.9480	EXP 150 of 150	42.649161 ± 0.022305	0.9952	EXP 150 of 150
17D19890	6.9 %	0.1391182 ± 0.0005733	0.2868	EXP 150 of 150	10.819649 ± 0.015615	0.9330	EXP 150 of 150	0.0424517 ± 0.0161837	0.0151	EXP 150 of 150	6.5265528 ± 0.0158351	0.8096	EXP 150 of 150	28.501897 ± 0.019491	0.9973	EXP 150 of 150
17D19892	7.9 %	0.1757715 ± 0.0006184	0.5387	EXP 150 of 150	13.040018 ± 0.018303	0.9473	EXP 150 of 150	0.1028779 ± 0.0178565	0.0163	EXP 150 of 150	7.8574025 ± 0.0162878	0.8911	EXP 150 of 150	37.684583 ± 0.019492	0.9962	EXP 150 of 150
17D19893	9.0 %	0.1874155 ± 0.0006444	0.5458	EXP 150 of 150	12.439281 ± 0.018916	0.9318	EXP 150 of 150	0.0702017 ± 0.0170555	0.0004	EXP 149 of 150	7.5423928 ± 0.0163429	0.8703	EXP 150 of 150	41.966780 ± 0.019476	0.9957	EXP 150 of 150
17D19895	10.3 %	0.2299664 ± 0.0007335	0.6855	EXP 150 of 150	13.108258 ± 0.020129	0.9329	EXP 149 of 150	0.1110552 ± 0.0156527	0.0028	EXP 150 of 150	7.8652897 ± 0.0168883	0.8794	EXP 150 of 150	53.234541 ± 0.022353	0.9909	EXP 150 of 150
17D19896	11.6 %	0.3047883 ± 0.0008736	0.7865	EXP 150 of 150	13.872525 ± 0.018556	0.9486	EXP 150 of 150	0.1283650 ± 0.0164180	0.0072	EXP 150 of 150	7.7237662 ± 0.0163989	0.8864	EXP 150 of 150	76.068323 ± 0.020760	0.9773	EXP 150 of 150
17D19898	12.5 %	0.2285248 ± 0.0007061	0.6939	EXP 150 of 150	9.961918 ± 0.017682	0.9064	EXP 150 of 150	0.0859206 ± 0.0166985	0.0021	EXP 150 of 150	5.4810824 ± 0.0171345	0.7761	EXP 150 of 150	57.461423 ± 0.018107	0.9915	EXP 150 of 150
17D19899	13.4 %	0.2475747 ± 0.0006593	0.7753	EXP 150 of 150	10.133330 ± 0.019293	0.8995	EXP 150 of 150	0.0841630 ± 0.0165717	0.0011	EXP 150 of 150	4.3611062 ± 0.0158601	0.6962	EXP 150 of 150	62.743695 ± 0.022730	0.9825	EXP 150 of 150
17D19901	14.6 %	0.2691113 ± 0.0007925	0.7754	EXP 150 of 150	11.135009 ± 0.018712	0.9210	EXP 150 of 150	0.0804591 ± 0.0145308	0.0012	EXP 149 of 150	4.1105423 ± 0.0161124	0.6477	EXP 150 of 150	67.761460 ± 0.021531	0.9754	EXP 150 of 150
17D19902	16.0 %	0.2627135 ± 0.0007770	0.7672	EXP 150 of 150	14.680867 ± 0.018038	0.9566	EXP 150 of 150	0.0807123 ± 0.0159248	0.0012	EXP 150 of 150	3.9625744 ± 0.0154504	0.6069	EXP 150 of 150	61.829543 ± 0.020585	0.9853	EXP 150 of 150
17D19904	17.6 %	0.1582485 ± 0.0005718	0.5487	EXP 150 of 150	10.414585 ± 0.019005	0.9047	EXP 150 of 150	0.0138685 ± 0.0181151	0.0079	EXP 150 of 150	2.5382104 ± 0.0151776	0.4247	EXP 150 of 150	34.655867 ± 0.018047	0.9955	EXP 150 of 150
17D19905	19.3 %	0.1191144 ± 0.0005261	0.3574	EXP 150 of 150	8.187267 ± 0.017422	0.8657	EXP 150 of 150	0.0301984 ± 0.0159060	0.0006	EXP 150 of 150	1.8875691 ± 0.0158818	0.2195	EXP 150 of 150	25.350435 ± 0.018199	0.9966	EXP 150 of 150
17D19907	21.0 %	0.1423882 ± 0.0006256	0.3847	EXP 150 of 150	10.517441 ± 0.018896	0.9137	EXP 150 of 150	0.0296497 ± 0.0148940	0.0001	EXP 150 of 150	2.0821644 ± 0.0160358	0.2909	EXP 150 of 150	29.660263 ± 0.018321	0.9960	EXP 150 of 150

Project Info		Analyst	Irradiation	X-pos	Y-pos	Z/H-pos	Project	Experiment	Nmb
17D19872	1.8 %	Dan Miggins	17-OSU-01	0.00	0.00	13.03	Arctic\O-Connor (16-22)	17D19868	01
17D19874	1.9 %	Dan Miggins	17-OSU-01	0.00	0.00	13.03	Arctic\O-Connor (16-22)	17D19868	01
17D19875	2.0 %	Dan Miggins	17-OSU-01	0.00	0.00	13.03	Arctic\O-Connor (16-22)	17D19868	01
17D19877	2.2 %	Dan Miggins	17-OSU-01	0.00	0.00	13.03	Arctic\O-Connor (16-22)	17D19868	01
17D19878	2.4 %	Dan Miggins	17-OSU-01	0.00	0.00	13.03	Arctic\O-Connor (16-22)	17D19868	01
17D19880	2.7 %	Dan Miggins	17-OSU-01	0.00	0.00	13.03	Arctic\O-Connor (16-22)	17D19868	01
17D19881	3.0 %	Dan Miggins	17-OSU-01	0.00	0.00	13.03	Arctic\O-Connor (16-22)	17D19868	01
17D19883	3.4 %	Dan Miggins	17-OSU-01	0.00	0.00	13.03	Arctic\O-Connor (16-22)	17D19868	01
17D19884	3.9 %	Dan Miggins	17-OSU-01	0.00	0.00	13.03	Arctic\O-Connor (16-22)	17D19868	01
17D19886	4.5 %	Dan Miggins	17-OSU-01	0.00	0.00	13.03	Arctic\O-Connor (16-22)	17D19868	01
17D19887	5.2 %	Dan Miggins	17-OSU-01	0.00	0.00	13.03	Arctic\O-Connor (16-22)	17D19868	01
17D19889	6.0 %	Dan Miggins	17-OSU-01	0.00	0.00	13.03	Arctic\O-Connor (16-22)	17D19868	01
17D19890	6.9 %	Dan Miggins	17-OSU-01	0.00	0.00	13.03	Arctic\O-Connor (16-22)	17D19868	01
17D19892	7.9 %	Dan Miggins	17-OSU-01	0.00	0.00	13.03	Arctic\O-Connor (16-22)	17D19868	01
17D19893	9.0 %	Dan Miggins	17-OSU-01	0.00	0.00	13.03	Arctic\O-Connor (16-22)	17D19868	01
17D19895	10.3 %	Dan Miggins	17-OSU-01	0.00	0.00	13.03	Arctic\O-Connor (16-22)	17D19868	01
17D19896	11.6 %	Dan Miggins	17-OSU-01	0.00	0.00	13.03	Arctic\O-Connor (16-22)	17D19868	01
17D19898	12.5 %	Dan Miggins	17-OSU-01	0.00	0.00	13.03	Arctic\O-Connor (16-22)	17D19868	01
17D19899	13.4 %	Dan Miggins	17-OSU-01	0.00	0.00	13.03	Arctic\O-Connor (16-22)	17D19868	01
17D19901	14.6 %	Dan Miggins	17-OSU-01	0.00	0.00	13.03	Arctic\O-Connor (16-22)	17D19868	01
17D19902	16.0 %	Dan Miggins	17-OSU-01	0.00	0.00	13.03	Arctic\O-Connor (16-22)	17D19868	01
17D19904	17.6 %	Dan Miggins	17-OSU-01	0.00	0.00	13.03	Arctic\O-Connor (16-22)	17D19868	01
17D19905	19.3 %	Dan Miggins	17-OSU-01	0.00	0.00	13.03	Arctic\O-Connor (16-22)	17D19868	01
17D19907	21.0 %	Dan Miggins	17-OSU-01	0.00	0.00	13.03	Arctic\O-Connor (16-22)	17D19868	01

**Sample Parameters**		Sample	Material	Location	Standard Name	Standard (in Ma)	%1σ	Standard Reference	Standard 40Ar/39Ar	%1σ	J	%1σ	Air 40Ar/36Ar	%1σ	MDF (lin)	%1σ	Volume Ratio	Sensitivity (mol/volt)	Day	Month	Year	Hour	Min	Resist
17D19872	1.8 %	PS59-311-1	Groundmass	Gakkel Ridge	FCT-NM (1C8-17)	28.201	0.082	Kuiper et al (2008)	9.49259	0.09	0.00165575	0.090	302.495	0.138	0.9942114	0.067	1	4.8E-14	11	JUN	2017	0	52	1
17D19874	1.9 %	PS59-311-1	Groundmass	Gakkel Ridge	FCT-NM (1C8-17)	28.201	0.082	Kuiper et al (2008)	9.49259	0.09	0.00165575	0.090	302.495	0.138	0.9942114	0.067	1	4.8E-14	11	JUN	2017	1	12	1
17D19875	2.0 %	PS59-311-1	Groundmass	Gakkel Ridge	FCT-NM (1C8-17)	28.201	0.082	Kuiper et al (2008)	9.49259	0.09	0.00165575	0.090	302.495	0.138	0.9942114	0.067	1	4.8E-14	11	JUN	2017	1	22	1
17D19877	2.2 %	PS59-311-1	Groundmass	Gakkel Ridge	FCT-NM (1C8-17)	28.201	0.082	Kuiper et al (2008)	9.49259	0.09	0.00165575	0.090	302.495	0.138	0.9942114	0.067	1	4.8E-14	11	JUN	2017	1	42	1
17D19878	2.4 %	PS59-311-1	Groundmass	Gakkel Ridge	FCT-NM (1C8-17)	28.201	0.082	Kuiper et al (2008)	9.49259	0.09	0.00165575	0.090	302.495	0.138	0.9942114	0.067	1	4.8E-14	11	JUN	2017	1	52	1
17D19880	2.7 %	PS59-311-1	Groundmass	Gakkel Ridge	FCT-NM (1C8-17)	28.201	0.082	Kuiper et al (2008)	9.49259	0.09	0.00165575	0.090	302.495	0.138	0.9942114	0.067	1	4.8E-14	11	JUN	2017	2	12	1
17D19881	3.0 %	PS59-311-1	Groundmass	Gakkel Ridge	FCT-NM (1C8-17)	28.201	0.082	Kuiper et al (2008)	9.49259	0.09	0.00165575	0.090	302.495	0.138	0.9942114	0.067	1	4.8E-14	11	JUN	2017	2	22	1
17D19883	3.4 %	PS59-311-1	Groundmass	Gakkel Ridge	FCT-NM (1C8-17)	28.201	0.082	Kuiper et al (2008)	9.49259	0.09	0.00165575	0.090	302.495	0.138	0.9942114	0.067	1	4.8E-14	11	JUN	2017	2	42	1
17D19884	3.9 %	PS59-311-1	Groundmass	Gakkel Ridge	FCT-NM (1C8-17)	28.201	0.082	Kuiper et al (2008)	9.49259	0.09	0.00165575	0.090	302.495	0.138	0.9942114	0.067	1	4.8E-14	11	JUN	2017	2	52	1
17D19886	4.5 %	PS59-311-1	Groundmass	Gakkel Ridge	FCT-NM (1C8-17)	28.201	0.082	Kuiper et al (2008)	9.49259	0.09	0.00165575	0.090	302.495	0.138	0.9942114	0.067	1	4.8E-14	11	JUN	2017	3	12	1
17D19887	5.2 %	PS59-311-1	Groundmass	Gakkel Ridge	FCT-NM (1C8-17)	28.201	0.082	Kuiper et al (2008)	9.49259	0.09	0.00165575	0.090	302.495	0.138	0.9942114	0.067	1	4.8E-14	11	JUN	2017	3	22	1
17D19889	6.0 %	PS59-311-1	Groundmass	Gakkel Ridge	FCT-NM (1C8-17)	28.201	0.082	Kuiper et al (2008)	9.49259	0.09	0.00165575	0.090	302.495	0.138	0.9942114	0.067	1	4.8E-14	11	JUN	2017	3	43	1
17D19890	6.9 %	PS59-311-1	Groundmass	Gakkel Ridge	FCT-NM (1C8-17)	28.201	0.082	Kuiper et al (2008)	9.49259	0.09	0.00165575	0.090	302.495	0.138	0.9942114	0.067	1	4.8E-14	11	JUN	2017	3	56	1
17D19892	7.9 %	PS59-311-1	Groundmass	Gakkel Ridge	FCT-NM (1C8-17)	28.201	0.082	Kuiper et al (2008)	9.49259	0.09	0.00165575	0.090	302.495	0.138	0.9942114	0.067	1	4.8E-14	11	JUN	2017	4	16	1
17D19893	9.0 %	PS59-311-1	Groundmass	Gakkel Ridge	FCT-NM (1C8-17)	28.201	0.082	Kuiper et al (2008)	9.49259	0.09	0.00165575	0.090	302.495	0.138	0.9942114	0.067	1	4.8E-14	11	JUN	2017	4	26	1
17D19895	10.3 %	PS59-311-1	Groundmass	Gakkel Ridge	FCT-NM (1C8-17)	28.201	0.082	Kuiper et al (2008)	9.49259	0.09	0.00165575	0.090	302.495	0.138	0.9942114	0.067	1	4.8E-14	11	JUN	2017	4	46	1
17D19896	11.6 %	PS59-311-1	Groundmass	Gakkel Ridge	FCT-NM (1C8-17)	28.201	0.082	Kuiper et al (2008)	9.49259	0.09	0.00165575	0.090	302.495	0.138	0.9942114	0.067	1	4.8E-14	11	JUN	2017	4	56	1
17D19898	12.5 %	PS59-311-1	Groundmass	Gakkel Ridge	FCT-NM (1C8-17)	28.201	0.082	Kuiper et al (2008)	9.49259	0.09	0.00165575	0.090	302.495	0.138	0.9942114	0.067	1	4.8E-14	11	JUN	2017	5	16	1
17D19899	13.4 %	PS59-311-1	Groundmass	Gakkel Ridge	FCT-NM (1C8-17)	28.201	0.082	Kuiper et al (2008)	9.49259	0.09	0.00165575	0.090	302.495	0.138	0.9942114	0.067	1	4.8E-14	11	JUN	2017	5	26	1
17D19901	14.6 %	PS59-311-1	Groundmass	Gakkel Ridge	FCT-NM (1C8-17)	28.201	0.082	Kuiper et al (2008)	9.49259	0.09	0.00165575	0.090	302.495	0.138	0.9942114	0.067	1	4.8E-14	11	JUN	2017	5	46	1
17D19902	16.0 %	PS59-311-1	Groundmass	Gakkel Ridge	FCT-NM (1C8-17)	28.201	0.082	Kuiper et al (2008)	9.49259	0.09	0.00165575	0.090	302.495	0.138	0.9942114	0.067	1	4.8E-14	11	JUN	2017	5	56	1
17D19904	17.6 %	PS59-311-1	Groundmass	Gakkel Ridge	FCT-NM (1C8-17)	28.201	0.082	Kuiper et al (2008)	9.49259	0.09	0.00165575	0.090	302.495	0.138	0.9942114	0.067	1	4.8E-14	11	JUN	2017	6	16	1
17D19905	19.3 %	PS59-311-1	Groundmass	Gakkel Ridge	FCT-NM (1C8-17)	28.201	0.082	Kuiper et al (2008)	9.49259	0.09	0.00165575	0.090	302.495	0.138	0.9942114	0.067	1	4.8E-14	11	JUN	2017	6	26	1
17D19907	21.0 %	PS59-311-1	Groundmass	Gakkel Ridge	FCT-NM (1C8-17)	28.201	0.082	Kuiper et al (2008)	9.49259	0.09	0.00165575	0.090	302.495	0.138	0.9942114	0.067	1	4.8E-14	11	JUN	2017	6	46	1

Irradiation Constants		40/36(a)	%1σ	40/36(c)	%1σ	38/36(a)	%1σ	38/36(c)	%1σ	39/37(ca)	%1σ	38/37(ca)	%1σ	36/37(ca)	%1σ	40/39(k)	%1σ	38/39(k)	%1σ	36/38(cl)	%1σ	K/Ca	%1σ	K/Cl	%1σ	Ca/Cl	%1σ
17D19872	1.8 %	295.5	0	0.018	35	0.1869	0	1.493	3	0.000643	0.92	0.00018	9.63	0.00027	0.17	0.000607	9.65	0.012077	0.09	0	0	0.43	0	0	0	0	0
17D19874	1.9 %	295.5	0	0.018	35	0.1869	0	1.493	3	0.000643	0.92	0.00018	9.63	0.00027	0.17	0.000607	9.65	0.012077	0.09	0	0	0.43	0	0	0	0	0
17D19875	2.0 %	295.5	0	0.018	35	0.1869	0	1.493	3	0.000643	0.92	0.00018	9.63	0.00027	0.17	0.000607	9.65	0.012077	0.09	0	0	0.43	0	0	0	0	0
17D19877	2.2 %	295.5	0	0.018	35	0.1869	0	1.493	3	0.000643	0.92	0.00018	9.63	0.00027	0.17	0.000607	9.65	0.012077	0.09	0	0	0.43	0	0	0	0	0
17D19878	2.4 %	295.5	0	0.018	35	0.1869	0	1.493	3	0.000643	0.92	0.00018	9.63	0.00027	0.17	0.000607	9.65	0.012077	0.09	0	0	0.43	0	0	0	0	0
17D19880	2.7 %	295.5	0	0.018	35	0.1869	0	1.493	3	0.000643	0.92	0.00018	9.63	0.00027	0.17	0.000607	9.65	0.012077	0.09	0	0	0.43	0	0	0	0	0
17D19881	3.0 %	295.5	0	0.018	35	0.1869	0	1.493	3	0.000643	0.92	0.00018	9.63	0.00027	0.17	0.000607	9.65	0.012077	0.09	0	0	0.43	0	0	0	0	0
17D19883	3.4 %	295.5	0	0.018	35	0.1869	0	1.493	3	0.000643	0.92	0.00018	9.63	0.00027	0.17	0.000607	9.65	0.012077	0.09	0	0	0.43	0	0	0	0	0
17D19884	3.9 %	295.5	0	0.018	35	0.1869	0	1.493	3	0.000643	0.92	0.00018	9.63	0.00027	0.17	0.000607	9.65	0.012077	0.09	0	0	0.43	0	0	0	0	0
17D19886	4.5 %	295.5	0	0.018	35	0.1869	0	1.493	3	0.000643	0.92	0.00018	9.63	0.00027	0.17	0.000607	9.65	0.012077	0.09	0	0	0.43	0	0	0	0	0
17D19887	5.2 %	295.5	0	0.018	35	0.1869	0	1.493	3	0.000643	0.92	0.00018	9.63	0.00027	0.17	0.000607	9.65	0.012077	0.09	0	0	0.43	0	0	0	0	0
17D19889	6.0 %	295.5	0	0.018	35	0.1869	0	1.493	3	0.000643	0.92	0.00018	9.63	0.00027	0.17	0.000607	9.65	0.012077	0.09	0	0	0.43	0	0	0	0	0
17D19890	6.9 %	295.5	0	0.018	35	0.1869	0	1.493	3	0.000643	0.92	0.00018	9.63	0.00027	0.17	0.000607	9.65	0.012077	0.09	0	0	0.43	0	0	0	0	0
17D19892	7.9 %	295.5	0	0.018	35	0.1869	0	1.493	3	0.000643	0.92	0.00018	9.63	0.00027	0.17	0.000607	9.65	0.012077	0.09	0	0	0.43	0	0	0	0	0
17D19893	9.0 %	295.5	0	0.018	35	0.1869	0	1.493	3	0.000643	0.92	0.00018	9.63	0.00027	0.17	0.000607	9.65	0.012077	0.09	0	0	0.43	0	0	0	0	0
17D19895	10.3 %	295.5	0	0.018	35	0.1869	0	1.493	3	0.000643	0.92	0.00018	9.63	0.00027	0.17	0.000607	9.65	0.012077	0.09	0	0	0.43	0	0	0	0	0
17D19896	11.6 %	295.5	0	0.018	35	0.1869	0	1.493	3	0.000643	0.92	0.00018	9.63	0.00027	0.17	0.000607	9.65	0.012077	0.09	0	0	0.43	0	0	0	0	0
17D19898	12.5 %	295.5	0	0.018	35	0.1869	0	1.493	3	0.000643	0.92	0.00018	9.63	0.00027	0.17	0.000607	9.65	0.012077	0.09	0	0	0.43	0	0	0	0	0
17D19899	13.4 %	295.5	0	0.018	35	0.1869	0	1.493	3	0.000643	0.92	0.00018	9.63	0.00027	0.17	0.000607	9.65	0.012077	0.09	0	0	0.43	0	0	0	0	0
17D19901	14.6 %	295.5	0	0.018	35	0.1869	0	1.493	3	0.000643	0.92	0.00018	9.63	0.00027	0.17	0.000607	9.65	0.012077	0.09	0	0	0.43	0	0	0	0	0
17D19902	16.0 %	295.5	0	0.018	35	0.1869	0	1.493	3	0.000643	0.92	0.00018	9.63	0.00027	0.17	0.000607	9.65	0.012077	0.09	0	0	0.43	0	0	0	0	0
17D19904	17.6 %	295.5	0	0.018	35	0.1869	0	1.493	3	0.000643	0.92	0.00018	9.63	0.00027	0.17	0.000607	9.65	0.012077	0.09	0	0	0.43	0	0	0	0	0
17D19905	19.3 %	295.5	0	0.018	35	0.1869	0	1.493	3	0.000643	0.92	0.00018	9.63	0.00027	0.17	0.000607	9.65	0.012077	0.09	0	0	0.43	0	0	0	0	0
17D19907	21.0 %	295.5	0	0.018	35	0.1869	0	1.493	3	0.000643	0.92	0.00018	9.63	0.00027	0.17	0.000607	9.65	0.012077	0.09	0	0	0.43	0	0	0	0	0

17D19868.AGE >>> PS59-311-1 >>> ARCTIC | O-CONNOR (16-22) PROJECT

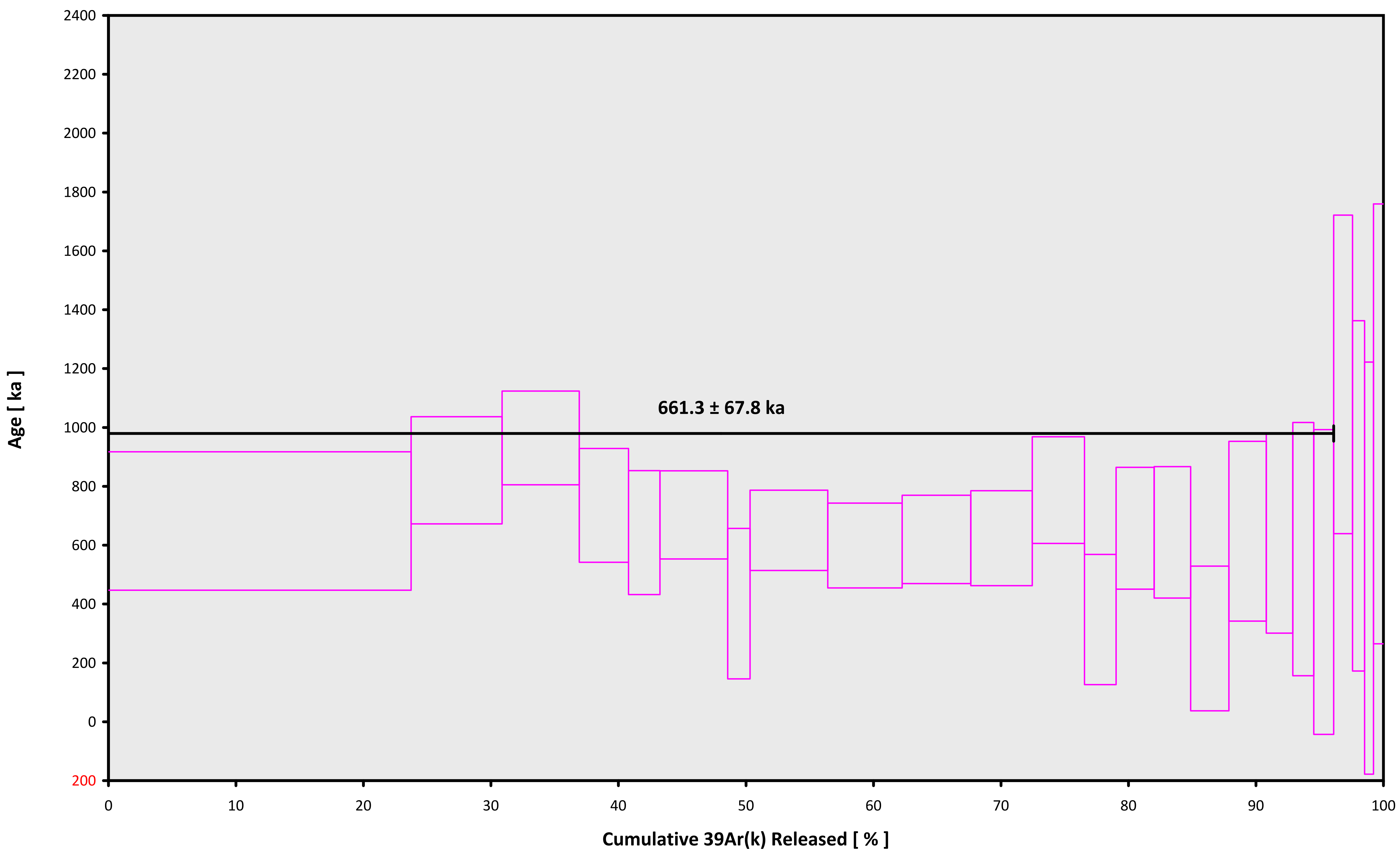

Ar-Ages in ka

WEIGHTED PLATEAU
661.3 ± 67.8
TOTAL FUSION
681.0 ± 66.5
NORMAL ISOCHRON
604.5 ± 160.6
INVERSE ISOCHRON
607.8 ± 138.8

MSWD (PROBABILITY)
2.40 (0%)

Sample Info

Groundmass
Gakkel Ridge
Dan Miggins

IRR = 17-OSU-01 (1C8-17)
J = 0.00165575 ± 0.00000149

17D19868.AGE >>> PS59-311-1 >>> ARCTIC | O-CONNOR (16-22) PROJECT

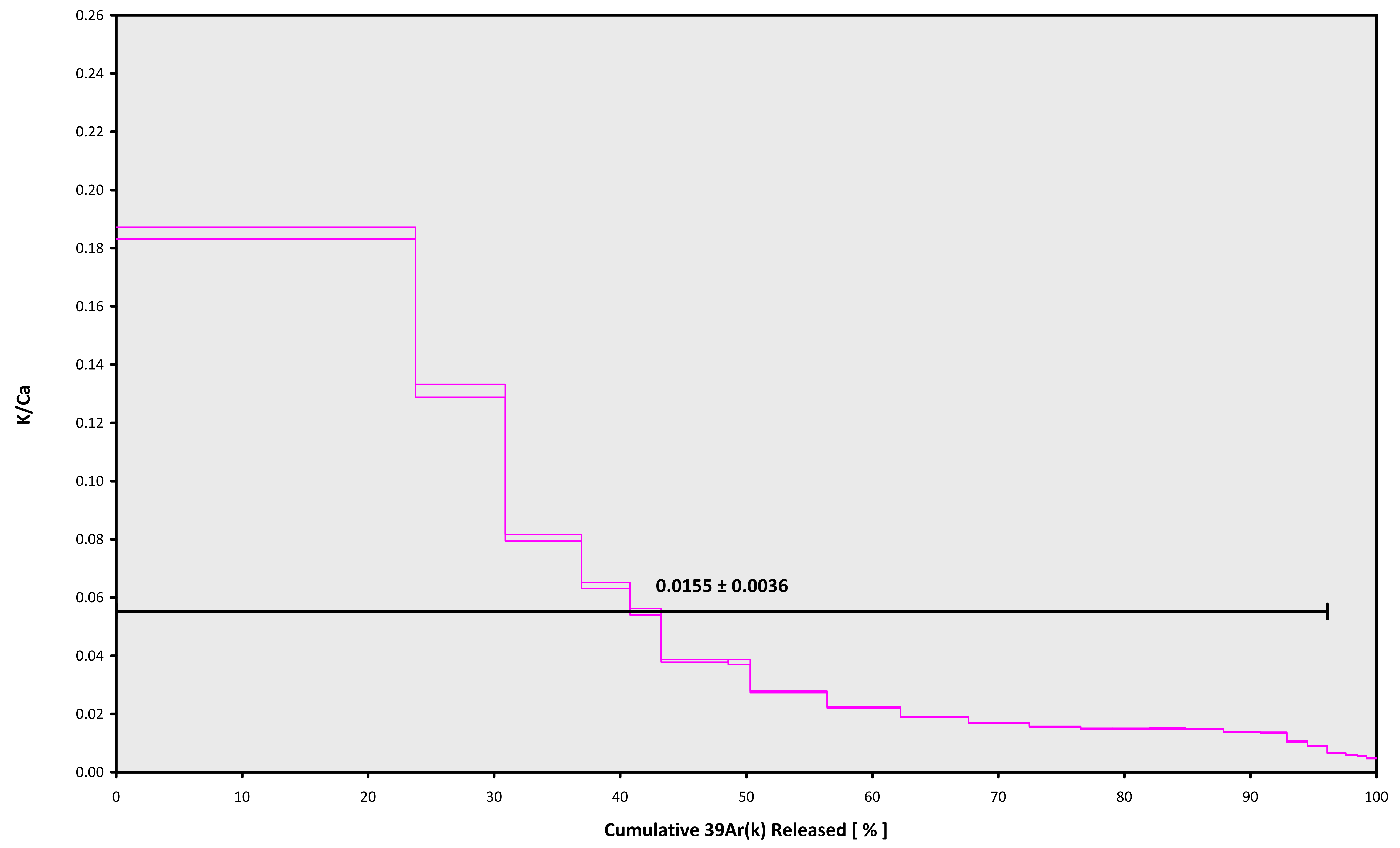

Ar-Ages in ka

WEIGHTED PLATEAU
661.3 ± 67.8
TOTAL FUSION
681.0 ± 66.5
NORMAL ISOCHRON
604.5 ± 160.6
INVERSE ISOCHRON
607.8 ± 138.8

Sample Info

Groundmass
Gakkel Ridge
Dan Miggins

IRR = 17-OSU-01 (1C8-17)
J = 0.00165575 ± 0.00000149

OSU Argon Geochronology Lab
CEOAS Oregon State University, Corvallis, USA

17D19868.AGE >>> PS59-311-1 >>> ARCTIC | O-CONNOR (16-22) PROJECT

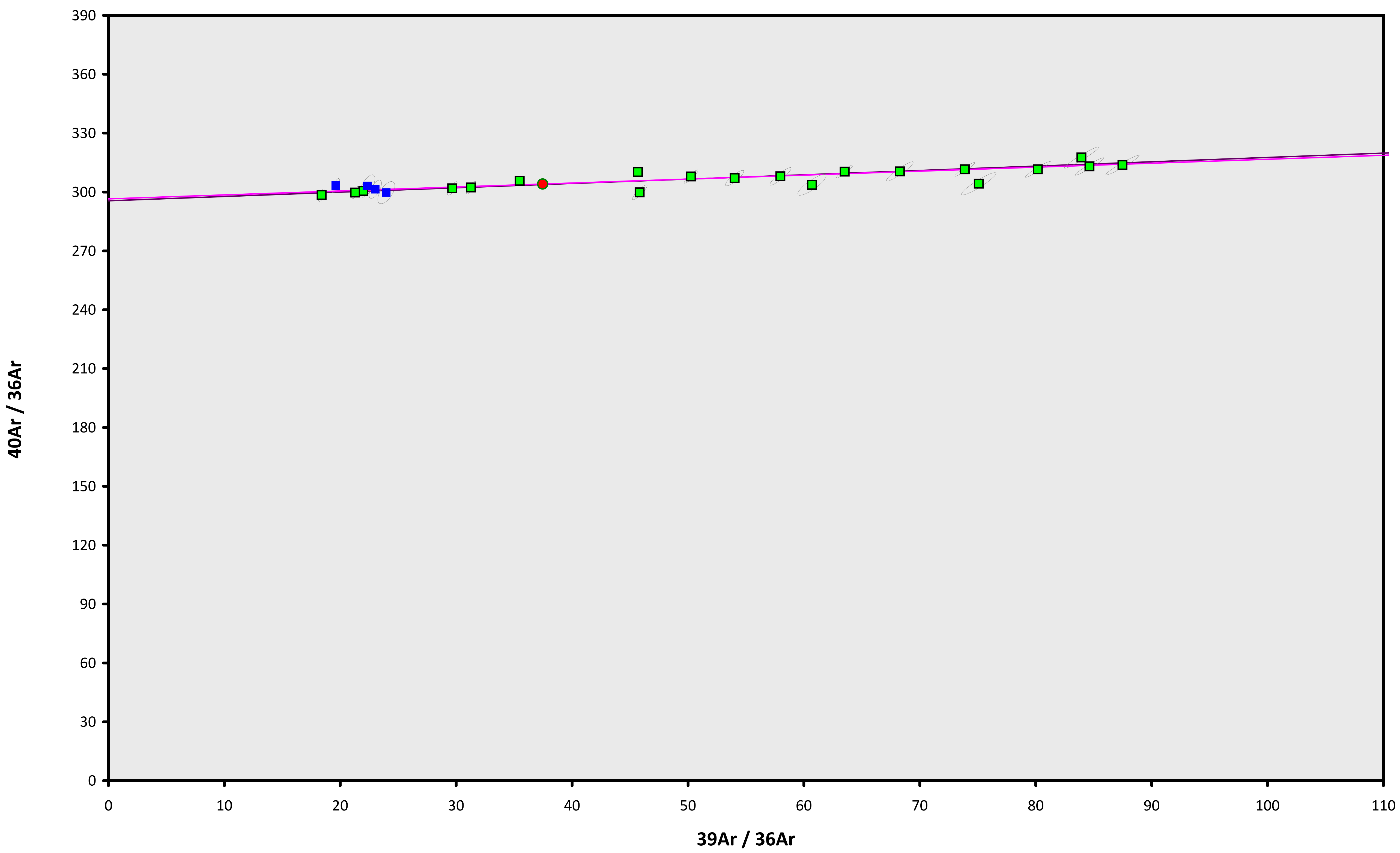

Ar-Ages in ka

WEIGHTED PLATEAU
661.3 ± 67.8
TOTAL FUSION
681.0 ± 66.5
NORMAL ISOCHRON
604.5 ± 160.6
INVERSE ISOCHRON
607.8 ± 138.8

MSWD (PROBABILITY)
2.48 (0%)

40AR/36AR INTERCEPT
296.5 ± 2.7

Sample Info

Groundmass
Gakkel Ridge
Dan Miggins

IRR = 17-OSU-01 (1C8-17)
J = 0.00165575 ± 0.00000149

17D19868.AGE >>> PS59-311-1 >>> ARCTIC | O-CONNOR (16-22) PROJECT

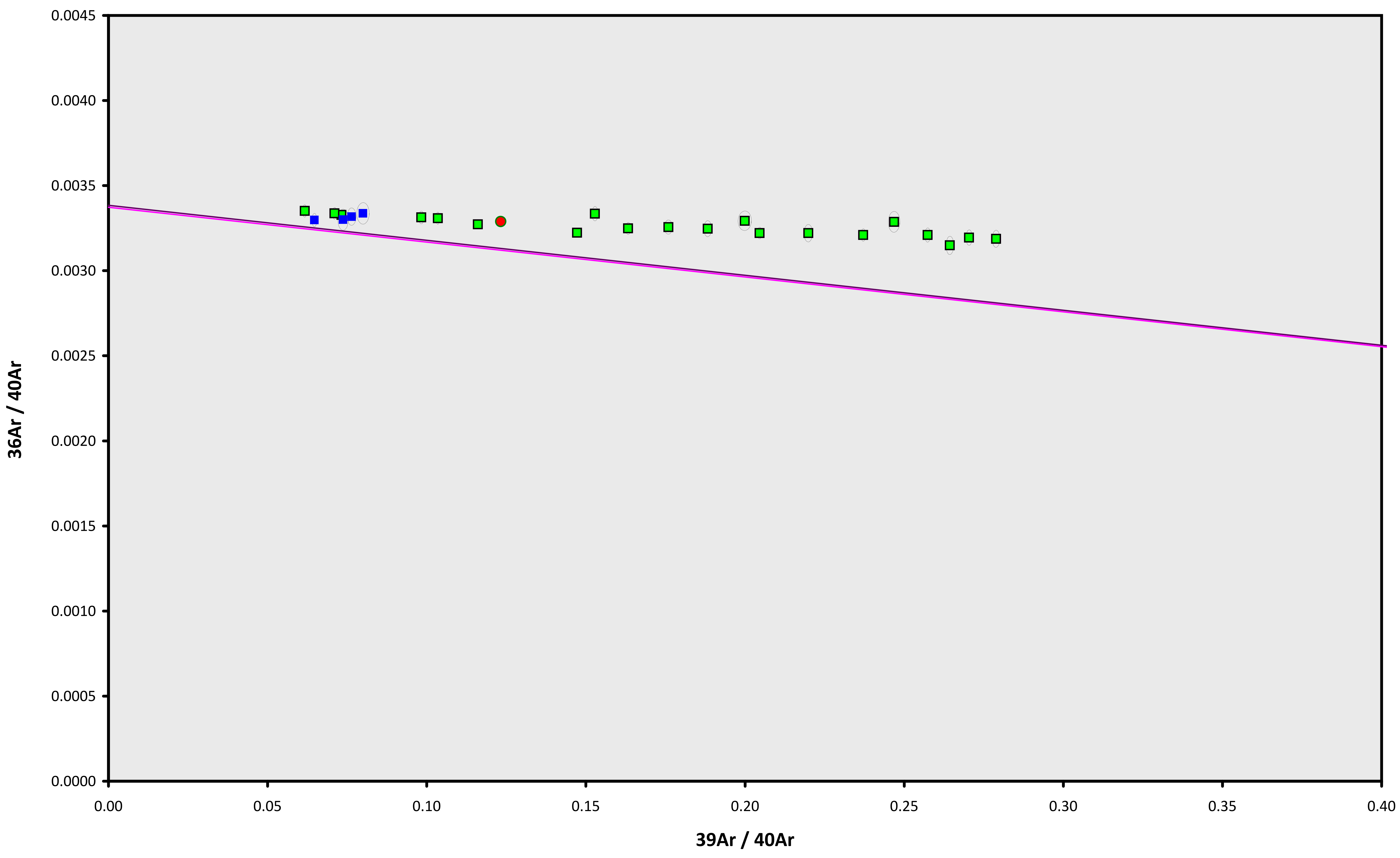

Ar-Ages in ka

WEIGHTED PLATEAU
661.3 ± 67.8
TOTAL FUSION
681.0 ± 66.5
NORMAL ISOCHRON
604.5 ± 160.6
INVERSE ISOCHRON
607.8 ± 138.8

MSWD (PROBABILITY)
2.45 (0%)

SPREADING FACTOR
4.4%

40AR/36AR INTERCEPT
296.5 ± 2.6

Sample Info

Groundmass
Gakkel Ridge
Dan Miggins

IRR = 17-OSU-01 (1C8-17)
J = 0.00165575 ± 0.00000149

Relative Abundances

			36Ar [fA]	%1σ	37Ar [fA]	%1σ	38Ar [fA]	%1σ	39Ar [fA]	%1σ	40Ar [fA]	%1σ	40(r)/39(k) ± 2σ	Age ± 2σ (ka)	40Ar(r) (%)	39Ar(k) (%)	K/Ca ± 2σ
17D20059	1.8 %	✓	10.347450	0.268	44.4243	1.092	2.884950	0.852	66.16595	0.074	3075.6029	0.007	0.16336 ± 0.28119	454.8 ± 782.7	0.35	17.20	0.640 ± 0.014
17D20061	1.9 %	✓	3.789713	0.276	32.5381	1.416	1.152856	2.074	31.79398	0.097	1125.0310	0.017	0.12149 ± 0.21969	338.2 ± 611.5	0.34	8.26	0.420 ± 0.012
17D20062	2.0 %	✓	1.398009	0.303	17.1391	2.584	0.471672	5.158	13.76582	0.174	415.3796	0.045	0.15968 ± 0.20316	444.5 ± 565.5	0.53	3.58	0.345 ± 0.018
17D20064	2.2 %	✓	1.363324	0.302	25.8699	1.779	0.469751	4.988	14.32140	0.168	403.8521	0.047	0.11556 ± 0.18987	321.7 ± 528.5	0.41	3.72	0.238 ± 0.008
17D20065	2.4 %	✓	1.145918	0.315	30.9576	1.500	0.391051	6.002	13.19749	0.186	339.8773	0.055	0.19370 ± 0.17999	539.2 ± 501.0	0.75	3.43	0.183 ± 0.006
17D20067	2.7 %	✓	1.063455	0.307	44.0032	1.147	0.399140	6.027	14.30601	0.175	312.2511	0.060	0.02973 ± 0.15152	82.8 ± 421.8	0.14	3.71	0.140 ± 0.003
17D20068	3.0 %	✓	0.628924	0.363	34.7733	1.370	0.252395	9.553	9.94336	0.229	185.2603	0.101	0.15595 ± 0.15103	434.1 ± 420.4	0.84	2.58	0.123 ± 0.003
17D20070	3.4 %	✓	0.784891	0.325	69.1407	0.813	0.383523	6.048	15.80793	0.158	228.0545	0.082	0.05363 ± 0.10702	149.3 ± 298.0	0.37	4.10	0.098 ± 0.002
17D20071	3.9 %	✓	0.624172	0.356	76.7871	0.734	0.324085	7.402	15.30585	0.161	179.6187	0.104	0.04453 ± 0.09595	124.0 ± 267.1	0.38	3.97	0.085 ± 0.001
17D20073	4.5 %	✓	0.578919	0.360	96.4669	0.646	0.327740	7.211	16.77938	0.143	166.0227	0.112	0.12430 ± 0.08221	346.0 ± 228.8	1.25	4.35	0.075 ± 0.001
17D20074	5.2 %	✓	0.653841	0.354	163.9944	0.527	0.425044	5.609	24.01282	0.117	181.9820	0.102	0.05139 ± 0.06351	143.1 ± 176.8	0.68	6.22	0.063 ± 0.001
17D20076	6.0 %	✓	0.455935	0.386	162.3518	0.527	0.361858	6.349	20.17760	0.132	124.1628	0.150	0.09794 ± 0.05820	272.7 ± 162.0	1.58	5.22	0.053 ± 0.001
17D20077	6.9 %	✓	0.415705	0.401	184.9263	0.513	0.349133	6.777	19.80244	0.138	110.9430	0.168	0.12624 ± 0.05641	351.4 ± 157.0	2.24	5.12	0.046 ± 0.000
17D20079	7.9 %	✓	0.361686	0.433	189.2479	0.503	0.295829	8.256	17.61775	0.146	94.1979	0.197	0.12032 ± 0.05976	335.0 ± 166.3	2.23	4.55	0.040 ± 0.000
17D20080	9.0 %	✓	0.338004	0.437	202.6424	0.501	0.319504	7.330	16.31992	0.148	86.5649	0.214	0.15883 ± 0.06149	442.2 ± 171.2	2.97	4.21	0.034 ± 0.000
17D20082	10.3 %	✓	0.299102	0.465	207.6110	0.498	0.280751	8.645	14.99444	0.168	73.9451	0.251	0.12684 ± 0.06350	353.1 ± 176.8	2.55	3.87	0.031 ± 0.000
17D20083	11.6 %	✓	0.276090	0.497	202.3989	0.498	0.250597	9.283	13.54257	0.181	66.9644	0.277	0.09773 ± 0.06923	272.1 ± 192.7	1.96	3.49	0.028 ± 0.000
17D20085	12.5 %	✓	0.239159	0.538	143.1507	0.547	0.160448	15.421	8.88005	0.250	60.5754	0.306	0.12814 ± 0.09959	356.7 ± 277.2	1.86	2.29	0.026 ± 0.000
17D20086	13.4 %	✓	0.241813	0.559	111.3050	0.618	0.158796	14.663	6.68209	0.339	63.4256	0.292	0.09673 ± 0.13745	269.3 ± 382.6	1.01	1.72	0.026 ± 0.000
17D20088	14.6 %	✓	0.309545	0.464	129.5770	0.572	0.181941	12.941	7.55022	0.307	82.0608	0.226	0.08757 ± 0.12914	243.8 ± 359.5	0.80	1.94	0.025 ± 0.000
17D20089	16.0 %	✓	0.303929	0.455	127.5548	0.568	0.122460	19.839	7.08413	0.318	80.4793	0.230	0.08211 ± 0.13362	228.6 ± 372.0	0.71	1.82	0.024 ± 0.000
17D20091	17.6 %	✓	0.210365	0.579	89.4775	0.670	0.117406	20.430	5.67286	0.394	55.0551	0.337	0.02779 ± 0.14833	77.4 ± 413.0	0.28	1.46	0.027 ± 0.000
17D20092	19.3 %	✓	0.313343	0.437	106.0102	0.619	0.176437	13.839	6.32677	0.363	84.6272	0.219	0.03301 ± 0.14866	91.9 ± 413.9	0.24	1.63	0.025 ± 0.000
17D20094	21.0 %	✓	0.594833	0.357	131.0478	0.564	0.250579	9.257	6.10301	0.375	167.2696	0.111	0.23050 ± 0.23255	641.6 ± 647.2	0.83	1.57	0.020 ± 0.000
		Σ	26.738126	0.118	2623.3959	0.135	10.507946	1.111	386.15384	0.033	7763.2031	0.012					

Information on Analysis and Constants Used in Calculations

Project = **O-CONNOR (16-22)**
Sample = **PS59-310-1-1**
Material = **Groundmass**
Location = **Gakkel Ridge**
Region = **Artic Ocean**
Analyst = **Dan Miggins**
Irradiation = **17-OSU-01 (1C38-17)**
Position = **X: 0 | Y: 0 | Z/H: 57.84776 mm**
FCT-NM Age = **28.201 ± 0.023 Ma**
FCT-NM Reference = **Kuiper et al (2008)**
FCT-NM 40Ar/39Ar Ratio = **10.20843 ± 0.00847**
FCT-NM J-value = **0.00153965 ± 0.00000128**
Air Shot 40Ar/36Ar = **302.6230 ± 0.3510**
Air Shot MDF = **0.99410801 ± 0.00064497 (LIN)**
Experiment Type = **Incremental Heating**
Extraction Method = **Bulk Laser Heating**
Heating = **64 sec**
Isolation = **3.00 min**
Instrument = **ARGUS-VI-D**
Preferred Age = **Plateau Age**
Age Classification = **Crystallization Age**
IGSN = Undefined
Rock Class = **Igneous>Volcanic**
Lithology = **Basaltic Lava**
Lat-Lon = Undefined - Undefined

Age Equations = **Min et al. (2000)**
Negative Intensities = **Allowed**
Collector Calibrations = **36Ar**
Decay 40K = **5.530 ± 0.048 E-10 1/a**
Decay 39Ar = **2.940 ± 0.016 E-07 1/h**
Decay 37Ar = **8.230 ± 0.012 E-04 1/h**
Decay 36Cl = **2.257 ± 0.015 E-06 1/a**
Decay 40K(EC,β^+) = **0.580 ± 0.009 E-10 1/a**
Decay 40K(β^-) = **4.950 ± 0.043 E-10 1/a**
Atmospheric 40/36(a) = **296.53 ± 0.42**
Atmospheric 38/36(a) = **0.1869**
Production 39/37(ca) = **0.0006425 ± 0.0000059**
Production 38/37(ca) = **0.0001800 ± 0.0000173**
Production 36/37(ca) = **0.0002703 ± 0.0000005**
Production 40/39(k) = **0.000607 ± 0.000059**
Production 38/39(k) = **0.012077 ± 0.000011**
Production 36/38(cl) = **262.80 ± 1.71**
Scaling Ratio K/Ca = **0.430**
Abundance Ratio 40K/K = **1.1700 ± 0.0100 E-04**
Atomic Weight K = **39.0983 ± 0.0001 g**

Results

Results	40(a)/36(a) ± 2σ	40(r)/39(k) ± 2σ	Age ± 2σ (ka)	MSWD	39Ar(k) (%,n)	K/Ca ± 2σ
**Age Plateau**		0.10590 ± 0.01895 ± 17.89%	**294.8 ± 52.8 ± 17.89%**	0.81 72%	100.00 24	0.030 ± 0.006
		Full External Error	± 53.2	1.59	2σ Confidence Limit	
		Analytical Error	± 52.7	1.0000	Error Magnification	
**Total Fusion Age**		0.11607 ± 0.05486 ± 47.26%	**323.1 ± 152.7 ± 47.26%**		24	0.063 ± 0.000
		Full External Error	± 152.9			
		Analytical Error	± 152.7			
**Normal Isochron**	**296.53 ± 0.84 ± 0.28%**	0.10506 ± 0.03074 ± 29.26%	**292.5 ± 85.6 ± 29.26%**	0.94 54%	100.00 24	
		Full External Error	± 85.8	1.60	2σ Confidence Limit	
		Analytical Error	± 85.6	1.0000	Error Magnification	
				4	Number of Iterations	
				0.0000000993	Convergence	
**Inverse Isochron** Clustered Points	**296.53 ± 0.84 ± 0.28%**	0.10557 ± 0.02556 ± 24.21%	**293.9 ± 71.1 ± 24.21%**	0.94 54%	100.00 24	
		Full External Error	± 71.5	1.60	2σ Confidence Limit	
		Analytical Error	± 71.1	1.0000	Error Magnification	
				3	Number of Iterations	
				0.0174119224	Convergence	
				2%	Spreading Factor	

Notes
Excess Initial 40Ar/36Ar = 296.53 ± 0.14 (%SD).

Incremental Heating			36Ar(a) [fA]	37Ar(ca) [fA]	38Ar(cl) [fA]	39Ar(k) [fA]	40Ar(r) [fA]	Age ± 2σ (ka)	40Ar(r) (%)	39Ar(k) (%)	K/Ca ± 2σ
17D20059	1.8 %	✓	10.335408	44.4243	0.1465245	66.13740	10.804255	454.8 ± 782.7	0.35	17.20	0.640 ± 0.014
17D20061	1.9 %	✓	3.780905	32.5381	0.0566247	31.77307	3.860031	338.2 ± 611.5	0.34	8.26	0.420 ± 0.012
17D20062	2.0 %	✓	1.393366	17.1391	0.0420501	13.75480	2.196310	444.5 ± 565.5	0.53	3.58	0.345 ± 0.018
17D20064	2.2 %	✓	1.356323	25.8699	0.0388389	14.30478	1.652996	321.7 ± 528.5	0.41	3.72	0.238 ± 0.008
17D20065	2.4 %	✓	1.137547	30.9576	0.0137256	13.17760	2.552436	539.2 ± 501.0	0.75	3.43	0.183 ± 0.006
17D20067	2.7 %	✓	1.051556	44.0032	0.0222511	14.27774	0.424420	82.8 ± 421.8	0.14	3.71	0.140 ± 0.003
17D20068	3.0 %	✓	0.619523	34.7733	0.0105306	9.92102	1.547197	434.1 ± 420.4	0.84	2.58	0.123 ± 0.003
17D20070	3.4 %	✓	0.766194	69.1407	0.0375002	15.76351	0.845467	149.3 ± 298.0	0.37	4.10	0.098 ± 0.002
17D20071	3.9 %	✓	0.603413	76.7871	0.0132321	15.25651	0.679330	124.0 ± 267.1	0.38	3.97	0.085 ± 0.001
17D20073	4.5 %	✓	0.552843	96.4669	0.0051538	16.71740	2.077914	346.0 ± 228.8	1.25	4.35	0.075 ± 0.001
17D20074	5.2 %	✓	0.609513	163.9944	0.0000000	23.90746	1.228647	143.1 ± 176.8	0.68	6.22	0.063 ± 0.001
17D20076	6.0 %	✓	0.412048	162.3518	0.0131975	20.07329	1.965972	272.7 ± 162.0	1.58	5.22	0.053 ± 0.001
17D20077	6.9 %	✓	0.365718	184.9263	0.0097749	19.68363	2.484807	351.4 ± 157.0	2.24	5.12	0.046 ± 0.000
17D20079	7.9 %	✓	0.310532	189.2479	0.0000000	17.49616	2.105204	335.0 ± 166.3	2.23	4.55	0.040 ± 0.000
17D20080	9.0 %	✓	0.283221	202.6424	0.0345705	16.18973	2.571468	442.2 ± 171.2	2.97	4.21	0.034 ± 0.000
17D20082	10.3 %	✓	0.242981	207.6110	0.0184913	14.86105	1.884994	353.1 ± 176.8	2.55	3.87	0.031 ± 0.000
17D20083	11.6 %	✓	0.221379	202.3989	0.0108069	13.41252	1.310834	272.1 ± 192.7	1.96	3.49	0.028 ± 0.000
17D20085	12.5 %	✓	0.200465	143.1507	0.0000000	8.78808	1.126128	356.7 ± 277.2	1.86	2.29	0.026 ± 0.000
17D20086	13.4 %	✓	0.211723	111.3050	0.0193543	6.61058	0.639452	269.3 ± 382.6	1.01	1.72	0.026 ± 0.000
17D20088	14.6 %	✓	0.274517	129.5770	0.0171309	7.46697	0.653864	243.8 ± 359.5	0.80	1.94	0.025 ± 0.000
17D20089	16.0 %	✓	0.269450	127.5548	0.0000000	7.00218	0.574957	228.6 ± 372.0	0.71	1.82	0.024 ± 0.000
17D20091	17.6 %	✓	0.186179	89.4775	0.0000000	5.61537	0.156073	77.4 ± 413.0	0.28	1.46	0.027 ± 0.000
17D20092	19.3 %	✓	0.284682	106.0102	0.0285627	6.25866	0.206612	91.9 ± 413.9	0.24	1.63	0.025 ± 0.000
17D20094	21.0 %	✓	0.559399	131.0478	0.0497497	6.01881	1.387354	641.6 ± 647.2	0.83	1.57	0.020 ± 0.000
		Σ	26.028885	2623.3959	0.5880703	384.46831	44.624577				

Information on Analysis

Project = **O-CONNOR (16-22)**
Sample = **PS59-310-1-1**
Material = **Groundmass**
Location = **Gakkel Ridge**
Region = **Artic Ocean**
Analyst = **Dan Miggins**
Irradiation = **17-OSU-01 (1C38-17)**
J = **0.00153965 ± 0.00000128**
FCT-NM = **28.201 ± 0.023 Ma**

Results	40(r)/39(k) ± 2σ		Age ± 2σ (ka)		MSWD	39Ar(k) (%,n)	K/Ca ± 2σ
**Age Plateau**	0.10590	± 0.01895<br>± 17.89%	**294.8**	**± 52.8**<br>**± 17.89%**	0.81<br>72%	100.00<br>24	0.030 ± 0.006
		Full External Error	± 53.2		1.59	2σ Confidence Limit	
		Analytical Error	± 52.7		1.0000	Error Magnification	
**Total Fusion Age**	0.11607	± 0.05486<br>± 47.26%	**323.1**	**± 152.7**<br>**± 47.26%**		24	0.063 ± 0.000
		Full External Error	± 152.9				
		Analytical Error	± 152.7				

Normal Isochron			39(k)/36(a) ± 2σ	40(a+r)/36(a) ± 2σ	r.i.
17D20059	1.8 %	✓	6.40 ± 0.04	297.58 ± 1.60	0.9636
17D20061	1.9 %	✓	8.40 ± 0.05	297.55 ± 1.65	0.9423
17D20062	2.0 %	✓	9.87 ± 0.07	298.11 ± 1.83	0.8580
17D20064	2.2 %	✓	10.55 ± 0.07	297.75 ± 1.83	0.8642
17D20065	2.4 %	✓	11.58 ± 0.09	298.77 ± 1.92	0.8496
17D20067	2.7 %	✓	13.58 ± 0.10	296.93 ± 1.88	0.8551
17D20068	3.0 %	✓	16.01 ± 0.14	299.03 ± 2.29	0.8195
17D20070	3.4 %	✓	20.57 ± 0.15	297.63 ± 2.04	0.8771
17D20071	3.9 %	✓	25.28 ± 0.20	297.66 ± 2.29	0.8824
17D20073	4.5 %	✓	30.24 ± 0.24	300.29 ± 2.37	0.8963
17D20074	5.2 %	✓	39.22 ± 0.31	298.55 ± 2.36	0.9234
17D20076	6.0 %	✓	48.72 ± 0.44	301.30 ± 2.75	0.9025
17D20077	6.9 %	✓	53.82 ± 0.52	303.32 ± 2.98	0.8997
17D20079	7.9 %	✓	56.34 ± 0.60	303.31 ± 3.33	0.8969
17D20080	9.0 %	✓	57.16 ± 0.63	305.61 ± 3.50	0.8930
17D20082	10.3 %	✓	61.16 ± 0.75	304.29 ± 3.88	0.8830
17D20083	11.6 %	✓	60.59 ± 0.80	302.45 ± 4.18	0.8801
17D20085	12.5 %	✓	43.84 ± 0.61	302.15 ± 4.35	0.8442
17D20086	13.4 %	✓	31.22 ± 0.46	299.55 ± 4.24	0.8044
17D20088	14.6 %	✓	27.20 ± 0.33	298.91 ± 3.43	0.7928
17D20089	16.0 %	✓	25.99 ± 0.32	298.66 ± 3.39	0.7768
17D20091	17.6 %	✓	30.16 ± 0.47	295.69 ± 4.38	0.7631
17D20092	19.3 %	✓	21.98 ± 0.27	297.26 ± 3.17	0.7268
17D20094	21.0 %	✓	10.76 ± 0.12	299.01 ± 2.38	0.6800

Results	40(a)/36(a) ± 2σ		40(r)/39(k) ± 2σ		Age ± 2σ (ka)		MSWD
**Normal Isochron**	**296.53**	**± 0.84**<br>**± 0.28%**	0.10506	± 0.03074<br>± 29.26%	**292.5**	**± 85.6**<br>**± 29.26%**	0.94<br>54%
					Full External Error	± 85.8	
					Analytical Error	± 85.6	

Statistics				
	2σ Confidence Limit	1.60	Convergence	0.000000099292
	Error Magnification	1.0000	Number of Iterations	4
	Number of Data Points	24	Calculated Line	Weighted York-2

Inverse Isochron			39(k)/40(a+r) ± 2σ	36(a)/40(a+r) ± 2σ	r.i.
17D20059	1.8 %	✓	0.0215042 ± 0.0000320	0.00336049 ± 0.00001806	0.0022
17D20061	1.9 %	✓	0.0282424 ± 0.0000555	0.00336077 ± 0.00001865	0.0106
17D20062	2.0 %	✓	0.0331145 ± 0.0001193	0.00335451 ± 0.00002062	0.0365
17D20064	2.2 %	✓	0.0354216 ± 0.0001237	0.00335854 ± 0.00002061	0.0405
17D20065	2.4 %	✓	0.0387726 ± 0.0001506	0.00334701 ± 0.00002155	0.0486
17D20067	2.7 %	✓	0.0457265 ± 0.0001698	0.00336776 ± 0.00002135	0.0611
17D20068	3.0 %	✓	0.0535535 ± 0.0002681	0.00334418 ± 0.00002558	0.1060
17D20070	3.4 %	✓	0.0691246 ± 0.0002468	0.00335984 ± 0.00002307	0.1085
17D20071	3.9 %	✓	0.0849427 ± 0.0003258	0.00335958 ± 0.00002579	0.1462
17D20073	4.5 %	✓	0.1006997 ± 0.0003668	0.00333013 ± 0.00002626	0.1752
17D20074	5.2 %	✓	0.1313831 ± 0.0004092	0.00334957 ± 0.00002650	0.1698
17D20076	6.0 %	✓	0.1616849 ± 0.0006476	0.00331894 ± 0.00003027	0.2464
17D20077	6.9 %	✓	0.1774402 ± 0.0007736	0.00329680 ± 0.00003236	0.2634
17D20079	7.9 %	✓	0.1857592 ± 0.0009133	0.00329696 ± 0.00003615	0.2873
17D20080	9.0 %	✓	0.1870454 ± 0.0009770	0.00327215 ± 0.00003751	0.3065
17D20082	10.3 %	✓	0.2009985 ± 0.0012163	0.00328636 ± 0.00004186	0.3263
17D20083	11.6 %	✓	0.2003178 ± 0.0013299	0.00330632 ± 0.00004568	0.3340
17D20085	12.5 %	✓	0.1450894 ± 0.0011509	0.00330964 ± 0.00004766	0.3273
17D20086	13.4 %	✓	0.1042323 ± 0.0009394	0.00333834 ± 0.00004731	0.2678
17D20088	14.6 %	✓	0.0909981 ± 0.0006985	0.00334547 ± 0.00003844	0.2313
17D20089	16.0 %	✓	0.0870105 ± 0.0006885	0.00334825 ± 0.00003801	0.2360
17D20091	17.6 %	✓	0.1020019 ± 0.0010638	0.00338190 ± 0.00005015	0.2931
17D20092	19.3 %	✓	0.0739590 ± 0.0006325	0.00336411 ± 0.00003583	0.2111
17D20094	21.0 %	✓	0.0359835 ± 0.0002852	0.00334437 ± 0.00002658	0.0783

Results	40(a)/36(a) ± 2σ		40(r)/39(k) ± 2σ		Age ± 2σ (ka)		MSWD
**Inverse Isochron**<br>Clustered Points	**296.53**	**± 0.84**<br>**± 0.28%**	0.10557	± 0.02556<br>± 24.21%	**293.9**	**± 71.1**<br>**± 24.21%**	0.94<br>54%
					Full External Error	± 71.5	
					Analytical Error	± 71.1	

Statistics				
	2σ Confidence Limit	1.60	Convergence	0.0174119224
	Error Magnification	1.0000	Number of Iterations	3
	Number of Data Points	24	Calculated Line	Weighted York-2
	Spreading Factor	1.9%		

Degassing Patterns			36Ar(a) [fA]	%1σ	36Ar(c) [fA]	%1σ	36Ar(ca) [fA]	%1σ	36Ar(cl) [fA]	%1σ	37Ar(ca) [fA]	%1σ	38Ar(a) [fA]	%1σ	38Ar(c) [fA]	%1σ	38Ar(k) [fA]	%1σ	38Ar(ca) [fA]	%1σ	38Ar(cl) [fA]	%1σ	39Ar(k) [fA]	%1σ	39Ar(ca) [fA]	%1σ	40Ar(r) [fA]	%1σ	40Ar(a) [fA]	%1σ	40Ar(c) [fA]	%1σ	40Ar(k) [fA]	%1σ
17D20059	1.8 %	✓	10.335408	0.27	0.0000000	0.00	0.0120079	1.11	0.0000342	17.20	44.4243	1.09	1.9316877	0.27	0.0000000	0.00	0.7987414	0.12	0.0079964	9.69	0.1465245	17.22	66.13740	0.07	0.0285426	1.43	10.804255	86.06	3064.7585	0.30	0.0000000	0.00	0.0401454	9.65
17D20061	1.9 %	✓	3.780905	0.28	0.0000000	0.00	0.0087951	1.43	0.0000132	42.39	32.5381	1.42	0.7066511	0.28	0.0000000	0.00	0.3837234	0.13	0.0058569	9.73	0.0566247	42.40	31.77307	0.10	0.0209057	1.69	3.860031	90.42	1121.1517	0.31	0.0000000	0.00	0.0192863	9.65
17D20062	2.0 %	✓	1.393366	0.30	0.0000000	0.00	0.0046327	2.59	0.0000098	57.91	17.1391	2.58	0.2604202	0.30	0.0000000	0.00	0.1661168	0.20	0.0030850	9.97	0.0420501	57.92	13.75480	0.17	0.0110119	2.74	2.196310	63.62	413.1749	0.34	0.0000000	0.00	0.0083492	9.65
17D20064	2.2 %	✓	1.356323	0.30	0.0000000	0.00	0.0069926	1.79	0.0000091	60.39	25.8699	1.78	0.2534967	0.30	0.0000000	0.00	0.1727588	0.19	0.0046566	9.79	0.0388389	60.40	14.30478	0.17	0.0166214	2.00	1.652996	82.16	402.1904	0.33	0.0000000	0.00	0.0086830	9.65
17D20065	2.4 %	✓	1.137547	0.32	0.0000000	0.00	0.0083678	1.51	0.0000032	171.15	30.9576	1.50	0.2126076	0.32	0.0000000	0.00	0.1591459	0.21	0.0055724	9.75	0.0137256	171.15	13.17760	0.19	0.0198903	1.76	2.552436	46.46	337.3168	0.35	0.0000000	0.00	0.0079988	9.65
17D20067	2.7 %	✓	1.051556	0.31	0.0000000	0.00	0.0118941	1.16	0.0000052	108.23	44.0032	1.15	0.1965359	0.31	0.0000000	0.00	0.1724322	0.20	0.0079206	9.70	0.0222511	108.23	14.27774	0.18	0.0282721	1.47	0.424420	254.87	311.8180	0.34	0.0000000	0.00	0.0086666	9.65
17D20068	3.0 %	✓	0.619523	0.37	0.0000000	0.00	0.0093992	1.38	0.0000025	229.09	34.7733	1.37	0.1157888	0.37	0.0000000	0.00	0.1198161	0.25	0.0062592	9.73	0.0105306	229.10	9.92102	0.23	0.0223418	1.65	1.547197	48.42	183.7070	0.39	0.0000000	0.00	0.0060221	9.65
17D20070	3.4 %	✓	0.766194	0.33	0.0000000	0.00	0.0186887	0.83	0.0000087	61.97	69.1407	0.81	0.1432017	0.33	0.0000000	0.00	0.1903759	0.18	0.0124453	9.66	0.0375002	61.98	15.76351	0.16	0.0444229	1.23	0.845467	99.77	227.1995	0.36	0.0000000	0.00	0.0095685	9.65
17D20071	3.9 %	✓	0.603413	0.37	0.0000000	0.00	0.0207556	0.75	0.0000031	181.63	76.7871	0.73	0.1127779	0.37	0.0000000	0.00	0.1842529	0.18	0.0138217	9.66	0.0132321	181.64	15.25651	0.16	0.0493357	1.18	0.679330	107.74	178.9301	0.40	0.0000000	0.00	0.0092607	9.65
17D20073	4.5 %	✓	0.552843	0.38	0.0000000	0.00	0.0260750	0.67	0.0000012	459.82	96.4669	0.65	0.1033264	0.38	0.0000000	0.00	0.2018961	0.17	0.0173640	9.65	0.0051538	459.82	16.71740	0.14	0.0619800	1.12	2.077914	33.07	163.9346	0.40	0.0000000	0.00	0.0101475	9.65
17D20074	5.2 %	✓	0.609513	0.38	0.0000000	0.00	0.0443277	0.55	0.0000000	0.00	163.9944	0.53	0.1139180	0.38	0.0000000	0.00	0.2887304	0.15	0.0295190	9.64	0.0000000	0.00	23.90746	0.12	0.1053664	1.06	1.228647	61.79	180.7389	0.41	0.0000000	0.00	0.0145118	9.65
17D20076	6.0 %	✓	0.412048	0.43	0.0000000	0.00	0.0438837	0.55	0.0000031	175.43	162.3518	0.53	0.0770118	0.43	0.0000000	0.00	0.2424251	0.16	0.0292233	9.64	0.0131975	175.43	20.07329	0.13	0.1043110	1.06	1.965972	29.71	122.1847	0.45	0.0000000	0.00	0.0121845	9.65
17D20077	6.9 %	✓	0.365718	0.46	0.0000000	0.00	0.0499856	0.54	0.0000023	244.35	184.9263	0.51	0.0683526	0.46	0.0000000	0.00	0.2377192	0.17	0.0332867	9.64	0.0097749	244.35	19.68363	0.14	0.1188151	1.05	2.484807	22.34	108.4462	0.48	0.0000000	0.00	0.0119480	9.65
17D20079	7.9 %	✓	0.310532	0.51	0.0000000	0.00	0.0511537	0.53	0.0000000	0.00	189.2479	0.50	0.0580385	0.51	0.0000000	0.00	0.2113011	0.17	0.0340646	9.64	0.0000000	0.00	17.49616	0.15	0.1215918	1.05	2.105204	24.83	92.0821	0.53	0.0000000	0.00	0.0106202	9.65
17D20080	9.0 %	✓	0.283221	0.53	0.0000000	0.00	0.0547742	0.53	0.0000081	68.52	202.6424	0.50	0.0529340	0.53	0.0000000	0.00	0.1955233	0.17	0.0364756	9.64	0.0345705	68.53	16.18973	0.15	0.1301978	1.05	2.571468	19.36	83.9836	0.55	0.0000000	0.00	0.0098272	9.65
17D20082	10.3 %	✓	0.242981	0.59	0.0000000	0.00	0.0561173	0.53	0.0000043	132.72	207.6110	0.50	0.0454131	0.59	0.0000000	0.00	0.1794769	0.19	0.0373700	9.64	0.0184913	132.72	14.86105	0.17	0.1333901	1.05	1.884994	25.03	72.0511	0.60	0.0000000	0.00	0.0090207	9.65
17D20083	11.6 %	✓	0.221379	0.63	0.0000000	0.00	0.0547084	0.53	0.0000025	217.74	202.3989	0.50	0.0413757	0.63	0.0000000	0.00	0.1619831	0.20	0.0364318	9.64	0.0108069	217.75	13.41252	0.18	0.1300413	1.05	1.310834	35.42	65.6454	0.65	0.0000000	0.00	0.0081414	9.65
17D20085	12.5 %	✓	0.200465	0.65	0.0000000	0.00	0.0386936	0.57	0.0000000	0.00	143.1507	0.55	0.0374670	0.65	0.0000000	0.00	0.1061336	0.27	0.0257671	9.65	0.0000000	0.00	8.78808	0.25	0.0919744	1.07	1.126128	38.86	59.4440	0.67	0.0000000	0.00	0.0053344	9.65
17D20086	13.4 %	✓	0.211723	0.65	0.0000000	0.00	0.0300858	0.64	0.0000045	120.74	111.3050	0.62	0.0395710	0.65	0.0000000	0.00	0.0798359	0.35	0.0200349	9.65	0.0193543	120.75	6.61058	0.34	0.0715135	1.11	0.639452	71.05	62.7821	0.66	0.0000000	0.00	0.0040126	9.66
17D20088	14.6 %	✓	0.274517	0.53	0.0000000	0.00	0.0350247	0.60	0.0000040	138.10	129.5770	0.57	0.0513072	0.53	0.0000000	0.00	0.0901786	0.32	0.0233239	9.65	0.0171309	138.10	7.46697	0.31	0.0832532	1.08	0.653864	73.74	81.4024	0.55	0.0000000	0.00	0.0045324	9.65
17D20089	16.0 %	✓	0.269450	0.52	0.0000000	0.00	0.0344781	0.59	0.0000000	0.00	127.5548	0.57	0.0503603	0.52	0.0000000	0.00	0.0845653	0.33	0.0229599	9.65	0.0000000	0.00	7.00218	0.32	0.0819540	1.08	0.574957	81.36	79.9001	0.54	0.0000000	0.00	0.0042503	9.66
17D20091	17.6 %	✓	0.186179	0.66	0.0000000	0.00	0.0241858	0.69	0.0000000	0.00	89.4775	0.67	0.0347969	0.66	0.0000000	0.00	0.0678169	0.41	0.0161059	9.65	0.0000000	0.00	5.61537	0.40	0.0574893	1.14	0.156073	266.83	55.2077	0.68	0.0000000	0.00	0.0034085	9.66
17D20092	19.3 %	✓	0.284682	0.49	0.0000000	0.00	0.0286545	0.64	0.0000067	85.75	106.0102	0.62	0.0532071	0.49	0.0000000	0.00	0.0755858	0.38	0.0190818	9.65	0.0285627	85.75	6.25866	0.37	0.0681115	1.11	0.206612	225.15	84.4168	0.51	0.0000000	0.00	0.0037990	9.66
17D20094	21.0 %	✓	0.559399	0.38	0.0000000	0.00	0.0354222	0.59	0.0000116	46.87	131.0478	0.56	0.1045517	0.38	0.0000000	0.00	0.0726892	0.39	0.0235886	9.65	0.0497497	46.88	6.01881	0.38	0.0841982	1.08	1.387354	50.44	165.8786	0.41	0.0000000	0.00	0.0036534	9.66
		Σ	26.028885	0.12	0.0000000	0.00	0.7091039	0.14	0.0001372	17.69	2623.3959	0.13	4.8647986	0.12	0.0000000	0.00	4.6432238	0.04	0.4722113	2.26	0.5880703	17.69	384.46831	0.03	1.6855318	0.25	44.624577	23.63	7718.3452	0.14	0.0000000	0.00	0.2333723	2.47
		Σ							26.738126	0.12	2623.3959	0.13									10.568304	0.99			386.15384	0.03							7763.2031	0.19

**Additional Parameters**			**40Ar/39Ar**	**1σ**	**37Ar/39Ar**	**1σ**	**36Ar/39Ar**	**1σ**	**Time (days)**	**37Ar (decay)**	**39Ar (decay)**	**40Ar (moles)**
17D20059	1.8 %	✓	46.483170	0.034585	0.671407	0.007352	0.156386	0.000435	143.604	17.097589	1.00101467	1.476E-10
17D20061	1.9 %	✓	35.385036	0.034726	1.023405	0.014522	0.119196	0.000349	143.618	17.102281	1.00101477	5.400E-11
17D20062	2.0 %	✓	30.174716	0.054314	1.245049	0.032245	0.101557	0.000355	143.625	17.104627	1.00101481	1.994E-11
17D20064	2.2 %	✓	28.199204	0.049192	1.806379	0.032278	0.095195	0.000329	143.639	17.109320	1.00101491	1.938E-11
17D20065	2.4 %	✓	25.753177	0.049944	2.345720	0.035453	0.086828	0.000317	143.646	17.111667	1.00101496	1.631E-11
17D20067	2.7 %	✓	21.826566	0.040450	3.075854	0.035684	0.074336	0.000263	143.660	17.116361	1.00101506	1.499E-11
17D20068	3.0 %	✓	18.631554	0.046537	3.497134	0.048569	0.063251	0.000271	143.667	17.118709	1.00101511	8.892E-12
17D20070	3.4 %	✓	14.426588	0.025693	4.373795	0.036229	0.049652	0.000180	143.681	17.123406	1.00101521	1.095E-11
17D20071	3.9 %	✓	11.735295	0.022452	5.016848	0.037673	0.040780	0.000159	143.688	17.125755	1.00101526	8.622E-12
17D20073	4.5 %	✓	9.894443	0.017976	5.749134	0.038053	0.034502	0.000134	143.701	17.130454	1.00101535	7.969E-12
17D20074	5.2 %	✓	7.578536	0.011766	6.829452	0.036856	0.027229	0.000102	143.708	17.132804	1.00101540	8.735E-12
17D20076	6.0 %	✓	6.153498	0.012291	8.046138	0.043684	0.022596	0.000092	143.723	17.137740	1.00101551	5.960E-12
17D20077	6.9 %	✓	5.602489	0.012178	9.338559	0.049623	0.020993	0.000089	143.730	17.140091	1.00101556	5.325E-12
17D20079	7.9 %	✓	5.346763	0.013105	10.741889	0.056290	0.020530	0.000094	143.744	17.144793	1.00101565	4.521E-12
17D20080	9.0 %	✓	5.304245	0.013809	12.416874	0.064861	0.020711	0.000096	143.751	17.147145	1.00101570	4.155E-12
17D20082	10.3 %	✓	4.931505	0.014871	13.845868	0.072762	0.019948	0.000099	143.765	17.151850	1.00101580	3.549E-12
17D20083	11.6 %	✓	4.944734	0.016357	14.945388	0.079217	0.020387	0.000108	143.772	17.154203	1.00101585	3.214E-12
17D20085	12.5 %	✓	6.821518	0.026931	16.120484	0.097005	0.026932	0.000160	143.785	17.158909	1.00101595	2.908E-12
17D20086	13.4 %	✓	9.491877	0.042491	16.657222	0.117401	0.036188	0.000237	143.792	17.161263	1.00101600	3.044E-12
17D20088	14.6 %	✓	10.868667	0.041393	17.162014	0.111367	0.040998	0.000228	143.806	17.165972	1.00101609	3.939E-12
17D20089	16.0 %	✓	11.360507	0.044583	18.005708	0.117118	0.042903	0.000238	143.813	17.168326	1.00101614	3.863E-12
17D20091	17.6 %	✓	9.704986	0.050295	15.772896	0.122617	0.037083	0.000260	143.827	17.173037	1.00101624	2.643E-12
17D20092	19.3 %	✓	13.376050	0.056718	16.755810	0.120283	0.049527	0.000281	143.834	17.175393	1.00101629	4.062E-12
17D20094	21.0 %	✓	27.407714	0.107141	21.472641	0.145427	0.097465	0.000505	143.848	17.180105	1.00101639	8.029E-12

Procedure Blanks		36Ar ± 1σ (SE) [fA]	37Ar ± 1σ (SE) [fA]	38Ar ± 1σ (SE) [fA]	39Ar ± 1σ (SE) [fA]	40Ar ± 1σ (SE) [fA]
17D20059	1.8 %	0.0089239 ± 0.0007529	0.1442885 ± 0.0177778	0.0355507 ± 0.0164620	0.0133863 ± 0.0155013	2.5847407 ± 0.1841114
17D20061	1.9 %	0.0098683 ± 0.0007529	0.1508292 ± 0.0177778	0.0381958 ± 0.0164620	0.0086554 ± 0.0155013	2.8615512 ± 0.1841114
17D20062	2.0 %	0.0100980 ± 0.0007529	0.1517856 ± 0.0177778	0.0389289 ± 0.0164620	0.0075183 ± 0.0155013	2.9239336 ± 0.1841114
17D20064	2.2 %	0.0101946 ± 0.0007529	0.1503831 ± 0.0177778	0.0394284 ± 0.0164620	0.0070409 ± 0.0155013	2.9357051 ± 0.1841114
17D20065	2.4 %	0.0101054 ± 0.0007529	0.1484934 ± 0.0177778	0.0392748 ± 0.0164620	0.0074656 ± 0.0155013	2.8991533 ± 0.1841114
17D20067	2.7 %	0.0097500 ± 0.0007529	0.1433698 ± 0.0177778	0.0383495 ± 0.0164620	0.0091280 ± 0.0155013	2.7724177 ± 0.1841114
17D20068	3.0 %	0.0095183 ± 0.0007529	0.1404932 ± 0.0177778	0.0376482 ± 0.0164620	0.0101890 ± 0.0155013	2.6931696 ± 0.1841114
17D20070	3.4 %	0.0090205 ± 0.0007529	0.1348638 ± 0.0177778	0.0359330 ± 0.0164620	0.0124022 ± 0.0155013	2.5263468 ± 0.1841114
17D20071	3.9 %	0.0087793 ± 0.0007529	0.1323563 ± 0.0177778	0.0349800 ± 0.0164620	0.0134362 ± 0.0155013	2.4465843 ± 0.1841114
17D20073	4.5 %	0.0083622 ± 0.0007529	0.1284293 ± 0.0177778	0.0330237 ± 0.0164620	0.0151363 ± 0.0155013	2.3099822 ± 0.1841114
17D20074	5.2 %	0.0082014 ± 0.0007529	0.1271434 ± 0.0177778	0.0320718 ± 0.0164620	0.0157424 ± 0.0155013	2.2578311 ± 0.1841114
17D20076	6.0 %	0.0079960 ± 0.0007529	0.1261252 ± 0.0177778	0.0302589 ± 0.0164620	0.0164022 ± 0.0155013	2.1919378 ± 0.1841114
17D20077	6.9 %	0.0079669 ± 0.0007529	0.1264623 ± 0.0177778	0.0295283 ± 0.0164620	0.0164238 ± 0.0155013	2.1829356 ± 0.1841114
17D20079	7.9 %	0.0080428 ± 0.0007529	0.1286330 ± 0.0177778	0.0284179 ± 0.0164620	0.0159745 ± 0.0155013	2.2080477 ± 0.1841114
17D20080	9.0 %	0.0081434 ± 0.0007529	0.1303726 ± 0.0177778	0.0280701 ± 0.0164620	0.0155623 ± 0.0155013	2.2404991 ± 0.1841114
17D20082	10.3 %	0.0084473 ± 0.0007529	0.1347854 ± 0.0177778	0.0278574 ± 0.0164620	0.0145820 ± 0.0155013	2.3371310 ± 0.1841114
17D20083	11.6 %	0.0086365 ± 0.0007529	0.1372529 ± 0.0177778	0.0280149 ± 0.0164620	0.0141310 ± 0.0155013	2.3965251 ± 0.1841114
17D20085	12.5 %	0.0090426 ± 0.0007529	0.1420553 ± 0.0177778	0.0289016 ± 0.0164620	0.0136723 ± 0.0155013	2.5215993 ± 0.1841114
17D20086	13.4 %	0.0092358 ± 0.0007529	0.1440726 ± 0.0177778	0.0296438 ± 0.0164620	0.0138398 ± 0.0155013	2.5793693 ± 0.1841114
17D20088	14.6 %	0.0095316 ± 0.0007529	0.1464052 ± 0.0177778	0.0317459 ± 0.0164620	0.0154797 ± 0.0155013	2.6616961 ± 0.1841114
17D20089	16.0 %	0.0096011 ± 0.0007529	0.1462910 ± 0.0177778	0.0331092 ± 0.0164620	0.0171856 ± 0.0155013	2.6752193 ± 0.1841114
17D20091	17.6 %	0.0094879 ± 0.0007529	0.1422875 ± 0.0177778	0.0364565 ± 0.0164620	0.0230260 ± 0.0155013	2.6154970 ± 0.1841114
17D20092	19.3 %	0.0092625 ± 0.0007529	0.1378568 ± 0.0177778	0.0384343 ± 0.0164620	0.0274524 ± 0.0155013	2.5280944 ± 0.1841114
17D20094	21.0 %	0.0083550 ± 0.0007529	0.1226442 ± 0.0177778	0.0429707 ± 0.0164620	0.0401200 ± 0.0155013	2.1989092 ± 0.1841114

Intercept Values		36Ar ± 1σ (SE) [fA]	r2	Regression (type,n)	37Ar ± 1σ (SE) [fA]	r2	Regression (type,n)	38Ar ± 1σ (SE) [fA]	r2	Regression (type,n)	39Ar ± 1σ (SE) [fA]	r2	Regression (type,n)	40Ar ± 1σ (SE) [fA]	r2	Regression (type,n)
17D20059	1.8 %	9.9635753 ± 0.0047480	0.9957	EXP 150 of 150	2.4081288 ± 0.0181351	0.3787	EXP 150 of 150	2.8154091 ± 0.0174848	0.3594	EXP 150 of 150	65.6967887 ± 0.0178945	0.9980	EXP 150 of 150	3078.18763 ± 0.09033	0.9999	EXP 150 of 150
17D20061	1.9 %	3.6557204 ± 0.0028967	0.9879	EXP 150 of 150	1.7181511 ± 0.0176906	0.1736	EXP 150 of 150	1.1010773 ± 0.0168785	0.0757	EXP 150 of 150	31.5663114 ± 0.0165232	0.9924	EXP 150 of 150	1127.89254 ± 0.05161	0.9998	EXP 150 of 150
17D20062	2.0 %	1.3550372 ± 0.0018529	0.9609	EXP 150 of 150	0.8325447 ± 0.0176394	0.0521	EXP 150 of 150	0.4271859 ± 0.0175137	0.0167	EXP 150 of 150	13.6634719 ± 0.0157824	0.9625	EXP 150 of 150	418.30351 ± 0.03133	0.9989	EXP 150 of 150
17D20064	2.2 %	1.3217660 ± 0.0017597	0.9638	EXP 150 of 150	1.3349633 ± 0.0183642	0.1939	EXP 150 of 150	0.4247881 ± 0.0162746	0.0229	EXP 150 of 150	14.2157052 ± 0.0157033	0.9654	EXP 150 of 150	406.78777 ± 0.03840	0.9985	EXP 150 of 150
17D20065	2.4 %	1.1125234 ± 0.0017345	0.9472	EXP 150 of 150	1.6287272 ± 0.0181727	0.2194	EXP 150 of 150	0.3471692 ± 0.0163324	0.0023	EXP 150 of 150	13.0991112 ± 0.0167793	0.9515	EXP 150 of 150	342.77643 ± 0.03482	0.9977	EXP 150 of 150
17D20067	2.7 %	1.0328358 ± 0.0014284	0.9594	EXP 150 of 150	2.3820815 ± 0.0198208	0.3637	EXP 150 of 150	0.3560877 ± 0.0171452	0.0031	EXP 150 of 150	14.1983315 ± 0.0171992	0.9611	EXP 150 of 150	315.02347 ± 0.03366	0.9975	EXP 150 of 150
17D20068	3.0 %	0.6145680 ± 0.0013051	0.8925	EXP 150 of 150	1.8549557 ± 0.0187103	0.2536	EXP 150 of 150	0.2117728 ± 0.0172219	0.0029	EXP 150 of 150	9.8646738 ± 0.0151087	0.9377	EXP 150 of 150	187.95342 ± 0.03031	0.9589	EXP 150 of 150
17D20070	3.4 %	0.7641168 ± 0.0012191	0.9440	EXP 150 of 150	3.8316552 ± 0.0200881	0.5695	EXP 150 of 150	0.3430715 ± 0.0159447	0.0321	EXP 150 of 150	15.6866331 ± 0.0165570	0.9714	EXP 150 of 150	230.58090 ± 0.02640	0.9947	EXP 150 of 150
17D20071	3.9 %	0.6092569 ± 0.0012260	0.8982	EXP 150 of 150	4.2722271 ± 0.0182378	0.6208	EXP 150 of 150	0.2852863 ± 0.0170542	0.0239	EXP 150 of 150	15.1869722 ± 0.0161206	0.9702	EXP 150 of 150	182.06524 ± 0.02871	0.9637	EXP 150 of 150
17D20073	4.5 %	0.5653053 ± 0.0011350	0.9108	EXP 150 of 150	5.4034896 ± 0.0184031	0.7373	EXP 150 of 150	0.2908552 ± 0.0165591	0.0005	EXP 150 of 150	16.6486556 ± 0.0145065	0.9802	EXP 149 of 150	168.33265 ± 0.02800	0.9529	EXP 149 of 150
17D20074	5.2 %	0.6372217 ± 0.0012813	0.9082	EXP 150 of 150	9.2758657 ± 0.0183562	0.8943	EXP 150 of 150	0.3879646 ± 0.0168433	0.0003	EXP 150 of 150	23.8316559 ± 0.0172454	0.9870	EXP 150 of 150	184.23987 ± 0.02697	0.9748	EXP 150 of 150
17D20076	6.0 %	0.4466234 ± 0.0009762	0.8833	EXP 150 of 150	9.1800180 ± 0.0179269	0.9026	EXP 150 of 150	0.3273355 ± 0.0156266	0.0052	EXP 150 of 150	20.0221945 ± 0.0170284	0.9814	EXP 150 of 150	126.35477 ± 0.02800	0.5674	EXP 150 of 150
17D20077	6.9 %	0.4078917 ± 0.0009408	0.8642	EXP 150 of 150	10.4722165 ± 0.0187760	0.9145	EXP 150 of 150	0.3154916 ± 0.0165995	0.0048	EXP 150 of 150	19.6495957 ± 0.0183019	0.9780	EXP 150 of 150	113.12590 ± 0.02803	0.9232	EXP 150 of 150
17D20079	7.9 %	0.3559987 ± 0.0009268	0.7951	EXP 150 of 150	10.7147547 ± 0.0162839	0.9335	EXP 150 of 150	0.2639254 ± 0.0176454	0.0005	EXP 150 of 150	17.4803990 ± 0.0168875	0.9755	EXP 150 of 150	96.40596 ± 0.02136	0.9743	EXP 150 of 150
17D20080	9.0 %	0.3333161 ± 0.0008472	0.7926	EXP 150 of 150	11.4788935 ± 0.0178656	0.9326	EXP 150 of 150	0.2876690 ± 0.0162619	0.0518	EXP 150 of 150	16.1919279 ± 0.0149940	0.9776	EXP 150 of 150	88.80538 ± 0.02191	0.9823	EXP 150 of 150
17D20082	10.3 %	0.2961956 ± 0.0008053	0.7714	EXP 150 of 150	11.7558644 ± 0.0175869	0.9352	EXP 150 of 150	0.2495861 ± 0.0174391	0.0118	EXP 150 of 150	14.8765515 ± 0.0170094	0.9682	EXP 150 of 150	76.28228 ± 0.02179	0.9874	EXP 150 of 150
17D20083	11.6 %	0.2742455 ± 0.0008259	0.7499	EXP 150 of 150	11.4532912 ± 0.0166682	0.9417	EXP 149 of 150	0.2196300 ± 0.0160425	0.0096	EXP 150 of 150	13.4351339 ± 0.0166824	0.9598	EXP 149 of 150	69.36091 ± 0.02084	0.9911	EXP 150 of 150
17D20085	12.5 %	0.2391228 ± 0.0007743	0.6726	EXP 150 of 150	8.0533446 ± 0.0180918	0.8750	EXP 150 of 150	0.1296564 ± 0.0180781	0.0012	EXP 150 of 150	8.8052007 ± 0.0145744	0.9287	EXP 150 of 150	63.09704 ± 0.01992	0.9912	EXP 150 of 150
17D20086	13.4 %	0.2418692 ± 0.0008659	0.6546	EXP 150 of 150	6.2272820 ± 0.0201555	0.7614	EXP 150 of 150	0.1272814 ± 0.0160750	0.0076	EXP 149 of 150	6.6222119 ± 0.0157233	0.8435	EXP 150 of 150	66.00493 ± 0.02226	0.9867	EXP 150 of 150
17D20088	14.6 %	0.3073264 ± 0.0008496	0.8019	EXP 150 of 150	7.2688439 ± 0.0189936	0.8324	EXP 150 of 150	0.1480510 ± 0.0164428	0.0166	EXP 150 of 150	7.4827225 ± 0.0162758	0.8709	EXP 150 of 150	84.72254 ± 0.02105	0.9566	EXP 150 of 150
17D20089	16.0 %	0.3019922 ± 0.0007796	0.8178	EXP 150 of 150	7.1522351 ± 0.0176926	0.8264	EXP 150 of 150	0.0879077 ± 0.0174758	0.0222	EXP 150 of 150	7.0181396 ± 0.0154576	0.8672	EXP 150 of 150	83.15457 ± 0.02061	0.9636	EXP 150 of 150
17D20091	17.6 %	0.2118672 ± 0.0007226	0.6921	EXP 149 of 150	4.9760956 ± 0.0180560	0.7250	EXP 150 of 150	0.0795658 ± 0.0170529	0.0021	EXP 149 of 150	5.6107513 ± 0.0154640	0.8035	EXP 150 of 150	57.67055 ± 0.02131	0.9884	EXP 150 of 150
17D20092	19.3 %	0.3107110 ± 0.0007317	0.8412	EXP 150 of 150	5.9254147 ± 0.0185492	0.7894	EXP 149 of 150	0.1359243 ± 0.0176397	0.0169	EXP 150 of 150	6.2557269 ± 0.0162248	0.8349	EXP 150 of 150	87.15529 ± 0.02284	0.9231	EXP 150 of 150
17D20094	21.0 %	0.5806075 ± 0.0011528	0.9137	EXP 150 of 150	7.3706023 ± 0.0180247	0.8423	EXP 150 of 150	0.2046562 ± 0.0159475	0.0669	EXP 150 of 150	6.0208412 ± 0.0161369	0.8146	EXP 150 of 150	169.46849 ± 0.02427	0.9920	EXP 150 of 150

Project Info		Analyst	Irradiation	X-pos	Y-pos	Z/H-pos	Project	Experiment	Nmb
17D20059	1.8 %	Dan Miggins	17-OSU-01	0.00	0.00	57.85	Arctic\O-Connor (16-22)	17D20055	01
17D20061	1.9 %	Dan Miggins	17-OSU-01	0.00	0.00	57.85	Arctic\O-Connor (16-22)	17D20055	01
17D20062	2.0 %	Dan Miggins	17-OSU-01	0.00	0.00	57.85	Arctic\O-Connor (16-22)	17D20055	01
17D20064	2.2 %	Dan Miggins	17-OSU-01	0.00	0.00	57.85	Arctic\O-Connor (16-22)	17D20055	01
17D20065	2.4 %	Dan Miggins	17-OSU-01	0.00	0.00	57.85	Arctic\O-Connor (16-22)	17D20055	01
17D20067	2.7 %	Dan Miggins	17-OSU-01	0.00	0.00	57.85	Arctic\O-Connor (16-22)	17D20055	01
17D20068	3.0 %	Dan Miggins	17-OSU-01	0.00	0.00	57.85	Arctic\O-Connor (16-22)	17D20055	01
17D20070	3.4 %	Dan Miggins	17-OSU-01	0.00	0.00	57.85	Arctic\O-Connor (16-22)	17D20055	01
17D20071	3.9 %	Dan Miggins	17-OSU-01	0.00	0.00	57.85	Arctic\O-Connor (16-22)	17D20055	01
17D20073	4.5 %	Dan Miggins	17-OSU-01	0.00	0.00	57.85	Arctic\O-Connor (16-22)	17D20055	01
17D20074	5.2 %	Dan Miggins	17-OSU-01	0.00	0.00	57.85	Arctic\O-Connor (16-22)	17D20055	01
17D20076	6.0 %	Dan Miggins	17-OSU-01	0.00	0.00	57.85	Arctic\O-Connor (16-22)	17D20055	01
17D20077	6.9 %	Dan Miggins	17-OSU-01	0.00	0.00	57.85	Arctic\O-Connor (16-22)	17D20055	01
17D20079	7.9 %	Dan Miggins	17-OSU-01	0.00	0.00	57.85	Arctic\O-Connor (16-22)	17D20055	01
17D20080	9.0 %	Dan Miggins	17-OSU-01	0.00	0.00	57.85	Arctic\O-Connor (16-22)	17D20055	01
17D20082	10.3 %	Dan Miggins	17-OSU-01	0.00	0.00	57.85	Arctic\O-Connor (16-22)	17D20055	01
17D20083	11.6 %	Dan Miggins	17-OSU-01	0.00	0.00	57.85	Arctic\O-Connor (16-22)	17D20055	01
17D20085	12.5 %	Dan Miggins	17-OSU-01	0.00	0.00	57.85	Arctic\O-Connor (16-22)	17D20055	01
17D20086	13.4 %	Dan Miggins	17-OSU-01	0.00	0.00	57.85	Arctic\O-Connor (16-22)	17D20055	01
17D20088	14.6 %	Dan Miggins	17-OSU-01	0.00	0.00	57.85	Arctic\O-Connor (16-22)	17D20055	01
17D20089	16.0 %	Dan Miggins	17-OSU-01	0.00	0.00	57.85	Arctic\O-Connor (16-22)	17D20055	01
17D20091	17.6 %	Dan Miggins	17-OSU-01	0.00	0.00	57.85	Arctic\O-Connor (16-22)	17D20055	01
17D20092	19.3 %	Dan Miggins	17-OSU-01	0.00	0.00	57.85	Arctic\O-Connor (16-22)	17D20055	01
17D20094	21.0 %	Dan Miggins	17-OSU-01	0.00	0.00	57.85	Arctic\O-Connor (16-22)	17D20055	01

**Sample Parameters**		Sample	Material	Location	Standard Name	Standard (in Ma)	%1σ	Standard Reference	Standard 40Ar/39Ar	%1σ	J	%1σ	Air 40Ar/36Ar	%1σ	MDF (lin)	%1σ	Volume Ratio	Sensitivity (mol/volt)	Day	Month	Year	Hour	Min	Resist
17D20059	1.8 %	PS59-310-1-1	Groundmass	Gakkel Ridge	FCT-NM (1C38-17)	28.201	0.082	Kuiper et al (2008)	10.20843	0.083	0.00153965	0.083	302.623	0.116	0.994108	0.065	1	4.8E-14	12	JUN	2017	6	9	1
17D20061	1.9 %	PS59-310-1-1	Groundmass	Gakkel Ridge	FCT-NM (1C38-17)	28.201	0.082	Kuiper et al (2008)	10.20843	0.083	0.00153965	0.083	302.623	0.116	0.994108	0.065	1	4.8E-14	12	JUN	2017	6	29	1
17D20062	2.0 %	PS59-310-1-1	Groundmass	Gakkel Ridge	FCT-NM (1C38-17)	28.201	0.082	Kuiper et al (2008)	10.20843	0.083	0.00153965	0.083	302.623	0.116	0.994108	0.065	1	4.8E-14	12	JUN	2017	6	39	1
17D20064	2.2 %	PS59-310-1-1	Groundmass	Gakkel Ridge	FCT-NM (1C38-17)	28.201	0.082	Kuiper et al (2008)	10.20843	0.083	0.00153965	0.083	302.623	0.116	0.994108	0.065	1	4.8E-14	12	JUN	2017	6	59	1
17D20065	2.4 %	PS59-310-1-1	Groundmass	Gakkel Ridge	FCT-NM (1C38-17)	28.201	0.082	Kuiper et al (2008)	10.20843	0.083	0.00153965	0.083	302.623	0.116	0.994108	0.065	1	4.8E-14	12	JUN	2017	7	9	1
17D20067	2.7 %	PS59-310-1-1	Groundmass	Gakkel Ridge	FCT-NM (1C38-17)	28.201	0.082	Kuiper et al (2008)	10.20843	0.083	0.00153965	0.083	302.623	0.116	0.994108	0.065	1	4.8E-14	12	JUN	2017	7	29	1
17D20068	3.0 %	PS59-310-1-1	Groundmass	Gakkel Ridge	FCT-NM (1C38-17)	28.201	0.082	Kuiper et al (2008)	10.20843	0.083	0.00153965	0.083	302.623	0.116	0.994108	0.065	1	4.8E-14	12	JUN	2017	7	39	1
17D20070	3.4 %	PS59-310-1-1	Groundmass	Gakkel Ridge	FCT-NM (1C38-17)	28.201	0.082	Kuiper et al (2008)	10.20843	0.083	0.00153965	0.083	302.623	0.116	0.994108	0.065	1	4.8E-14	12	JUN	2017	7	59	1
17D20071	3.9 %	PS59-310-1-1	Groundmass	Gakkel Ridge	FCT-NM (1C38-17)	28.201	0.082	Kuiper et al (2008)	10.20843	0.083	0.00153965	0.083	302.623	0.116	0.994108	0.065	1	4.8E-14	12	JUN	2017	8	9	1
17D20073	4.5 %	PS59-310-1-1	Groundmass	Gakkel Ridge	FCT-NM (1C38-17)	28.201	0.082	Kuiper et al (2008)	10.20843	0.083	0.00153965	0.083	302.623	0.116	0.994108	0.065	1	4.8E-14	12	JUN	2017	8	29	1
17D20074	5.2 %	PS59-310-1-1	Groundmass	Gakkel Ridge	FCT-NM (1C38-17)	28.201	0.082	Kuiper et al (2008)	10.20843	0.083	0.00153965	0.083	302.623	0.116	0.994108	0.065	1	4.8E-14	12	JUN	2017	8	39	1
17D20076	6.0 %	PS59-310-1-1	Groundmass	Gakkel Ridge	FCT-NM (1C38-17)	28.201	0.082	Kuiper et al (2008)	10.20843	0.083	0.00153965	0.083	302.623	0.116	0.994108	0.065	1	4.8E-14	12	JUN	2017	9	0	1
17D20077	6.9 %	PS59-310-1-1	Groundmass	Gakkel Ridge	FCT-NM (1C38-17)	28.201	0.082	Kuiper et al (2008)	10.20843	0.083	0.00153965	0.083	302.623	0.116	0.994108	0.065	1	4.8E-14	12	JUN	2017	9	10	1
17D20079	7.9 %	PS59-310-1-1	Groundmass	Gakkel Ridge	FCT-NM (1C38-17)	28.201	0.082	Kuiper et al (2008)	10.20843	0.083	0.00153965	0.083	302.623	0.116	0.994108	0.065	1	4.8E-14	12	JUN	2017	9	30	1
17D20080	9.0 %	PS59-310-1-1	Groundmass	Gakkel Ridge	FCT-NM (1C38-17)	28.201	0.082	Kuiper et al (2008)	10.20843	0.083	0.00153965	0.083	302.623	0.116	0.994108	0.065	1	4.8E-14	12	JUN	2017	9	40	1
17D20082	10.3 %	PS59-310-1-1	Groundmass	Gakkel Ridge	FCT-NM (1C38-17)	28.201	0.082	Kuiper et al (2008)	10.20843	0.083	0.00153965	0.083	302.623	0.116	0.994108	0.065	1	4.8E-14	12	JUN	2017	10	0	1
17D20083	11.6 %	PS59-310-1-1	Groundmass	Gakkel Ridge	FCT-NM (1C38-17)	28.201	0.082	Kuiper et al (2008)	10.20843	0.083	0.00153965	0.083	302.623	0.116	0.994108	0.065	1	4.8E-14	12	JUN	2017	10	10	1
17D20085	12.5 %	PS59-310-1-1	Groundmass	Gakkel Ridge	FCT-NM (1C38-17)	28.201	0.082	Kuiper et al (2008)	10.20843	0.083	0.00153965	0.083	302.623	0.116	0.994108	0.065	1	4.8E-14	12	JUN	2017	10	30	1
17D20086	13.4 %	PS59-310-1-1	Groundmass	Gakkel Ridge	FCT-NM (1C38-17)	28.201	0.082	Kuiper et al (2008)	10.20843	0.083	0.00153965	0.083	302.623	0.116	0.994108	0.065	1	4.8E-14	12	JUN	2017	10	40	1
17D20088	14.6 %	PS59-310-1-1	Groundmass	Gakkel Ridge	FCT-NM (1C38-17)	28.201	0.082	Kuiper et al (2008)	10.20843	0.083	0.00153965	0.083	302.623	0.116	0.994108	0.065	1	4.8E-14	12	JUN	2017	11	0	1
17D20089	16.0 %	PS59-310-1-1	Groundmass	Gakkel Ridge	FCT-NM (1C38-17)	28.201	0.082	Kuiper et al (2008)	10.20843	0.083	0.00153965	0.083	302.623	0.116	0.994108	0.065	1	4.8E-14	12	JUN	2017	11	10	1
17D20091	17.6 %	PS59-310-1-1	Groundmass	Gakkel Ridge	FCT-NM (1C38-17)	28.201	0.082	Kuiper et al (2008)	10.20843	0.083	0.00153965	0.083	302.623	0.116	0.994108	0.065	1	4.8E-14	12	JUN	2017	11	30	1
17D20092	19.3 %	PS59-310-1-1	Groundmass	Gakkel Ridge	FCT-NM (1C38-17)	28.201	0.082	Kuiper et al (2008)	10.20843	0.083	0.00153965	0.083	302.623	0.116	0.994108	0.065	1	4.8E-14	12	JUN	2017	11	40	1
17D20094	21.0 %	PS59-310-1-1	Groundmass	Gakkel Ridge	FCT-NM (1C38-17)	28.201	0.082	Kuiper et al (2008)	10.20843	0.083	0.00153965	0.083	302.623	0.116	0.994108	0.065	1	4.8E-14	12	JUN	2017	12	0	1

Irradiation Constants		40/36(a)	%1σ	40/36(c)	%1σ	38/36(a)	%1σ	38/36(c)	%1σ	39/37(ca)	%1σ	38/37(ca)	%1σ	36/37(ca)	%1σ	40/39(k)	%1σ	38/39(k)	%1σ	36/38(cl)	%1σ	K/Ca	%1σ	K/Cl	%1σ	Ca/Cl	%1σ
17D20059	1.8 %	296.53	0.141	0.018	35	0.1869	0	1.493	3	0.000643	0.92	0.00018	9.63	0.00027	0.17	0.000607	9.65	0.012077	0.09	0	0	0.43	0	0	0	0	0
17D20061	1.9 %	296.53	0.141	0.018	35	0.1869	0	1.493	3	0.000643	0.92	0.00018	9.63	0.00027	0.17	0.000607	9.65	0.012077	0.09	0	0	0.43	0	0	0	0	0
17D20062	2.0 %	296.53	0.141	0.018	35	0.1869	0	1.493	3	0.000643	0.92	0.00018	9.63	0.00027	0.17	0.000607	9.65	0.012077	0.09	0	0	0.43	0	0	0	0	0
17D20064	2.2 %	296.53	0.141	0.018	35	0.1869	0	1.493	3	0.000643	0.92	0.00018	9.63	0.00027	0.17	0.000607	9.65	0.012077	0.09	0	0	0.43	0	0	0	0	0
17D20065	2.4 %	296.53	0.141	0.018	35	0.1869	0	1.493	3	0.000643	0.92	0.00018	9.63	0.00027	0.17	0.000607	9.65	0.012077	0.09	0	0	0.43	0	0	0	0	0
17D20067	2.7 %	296.53	0.141	0.018	35	0.1869	0	1.493	3	0.000643	0.92	0.00018	9.63	0.00027	0.17	0.000607	9.65	0.012077	0.09	0	0	0.43	0	0	0	0	0
17D20068	3.0 %	296.53	0.141	0.018	35	0.1869	0	1.493	3	0.000643	0.92	0.00018	9.63	0.00027	0.17	0.000607	9.65	0.012077	0.09	0	0	0.43	0	0	0	0	0
17D20070	3.4 %	296.53	0.141	0.018	35	0.1869	0	1.493	3	0.000643	0.92	0.00018	9.63	0.00027	0.17	0.000607	9.65	0.012077	0.09	0	0	0.43	0	0	0	0	0
17D20071	3.9 %	296.53	0.141	0.018	35	0.1869	0	1.493	3	0.000643	0.92	0.00018	9.63	0.00027	0.17	0.000607	9.65	0.012077	0.09	0	0	0.43	0	0	0	0	0
17D20073	4.5 %	296.53	0.141	0.018	35	0.1869	0	1.493	3	0.000643	0.92	0.00018	9.63	0.00027	0.17	0.000607	9.65	0.012077	0.09	0	0	0.43	0	0	0	0	0
17D20074	5.2 %	296.53	0.141	0.018	35	0.1869	0	1.493	3	0.000643	0.92	0.00018	9.63	0.00027	0.17	0.000607	9.65	0.012077	0.09	0	0	0.43	0	0	0	0	0
17D20076	6.0 %	296.53	0.141	0.018	35	0.1869	0	1.493	3	0.000643	0.92	0.00018	9.63	0.00027	0.17	0.000607	9.65	0.012077	0.09	0	0	0.43	0	0	0	0	0
17D20077	6.9 %	296.53	0.141	0.018	35	0.1869	0	1.493	3	0.000643	0.92	0.00018	9.63	0.00027	0.17	0.000607	9.65	0.012077	0.09	0	0	0.43	0	0	0	0	0
17D20079	7.9 %	296.53	0.141	0.018	35	0.1869	0	1.493	3	0.000643	0.92	0.00018	9.63	0.00027	0.17	0.000607	9.65	0.012077	0.09	0	0	0.43	0	0	0	0	0
17D20080	9.0 %	296.53	0.141	0.018	35	0.1869	0	1.493	3	0.000643	0.92	0.00018	9.63	0.00027	0.17	0.000607	9.65	0.012077	0.09	0	0	0.43	0	0	0	0	0
17D20082	10.3 %	296.53	0.141	0.018	35	0.1869	0	1.493	3	0.000643	0.92	0.00018	9.63	0.00027	0.17	0.000607	9.65	0.012077	0.09	0	0	0.43	0	0	0	0	0
17D20083	11.6 %	296.53	0.141	0.018	35	0.1869	0	1.493	3	0.000643	0.92	0.00018	9.63	0.00027	0.17	0.000607	9.65	0.012077	0.09	0	0	0.43	0	0	0	0	0
17D20085	12.5 %	296.53	0.141	0.018	35	0.1869	0	1.493	3	0.000643	0.92	0.00018	9.63	0.00027	0.17	0.000607	9.65	0.012077	0.09	0	0	0.43	0	0	0	0	0
17D20086	13.4 %	296.53	0.141	0.018	35	0.1869	0	1.493	3	0.000643	0.92	0.00018	9.63	0.00027	0.17	0.000607	9.65	0.012077	0.09	0	0	0.43	0	0	0	0	0
17D20088	14.6 %	296.53	0.141	0.018	35	0.1869	0	1.493	3	0.000643	0.92	0.00018	9.63	0.00027	0.17	0.000607	9.65	0.012077	0.09	0	0	0.43	0	0	0	0	0
17D20089	16.0 %	296.53	0.141	0.018	35	0.1869	0	1.493	3	0.000643	0.92	0.00018	9.63	0.00027	0.17	0.000607	9.65	0.012077	0.09	0	0	0.43	0	0	0	0	0
17D20091	17.6 %	296.53	0.141	0.018	35	0.1869	0	1.493	3	0.000643	0.92	0.00018	9.63	0.00027	0.17	0.000607	9.65	0.012077	0.09	0	0	0.43	0	0	0	0	0
17D20092	19.3 %	296.53	0.141	0.018	35	0.1869	0	1.493	3	0.000643	0.92	0.00018	9.63	0.00027	0.17	0.000607	9.65	0.012077	0.09	0	0	0.43	0	0	0	0	0
17D20094	21.0 %	296.53	0.141	0.018	35	0.1869	0	1.493	3	0.000643	0.92	0.00018	9.63	0.00027	0.17	0.000607	9.65	0.012077	0.09	0	0	0.43	0	0	0	0	0

OSU Argon Geochronology Lab
CEOAS Oregon State University, Corvallis, USA

17D20055.AGE >>> PS59-310-1-1 >>> ARCTIC | O-CONNOR (16-22) PROJECT

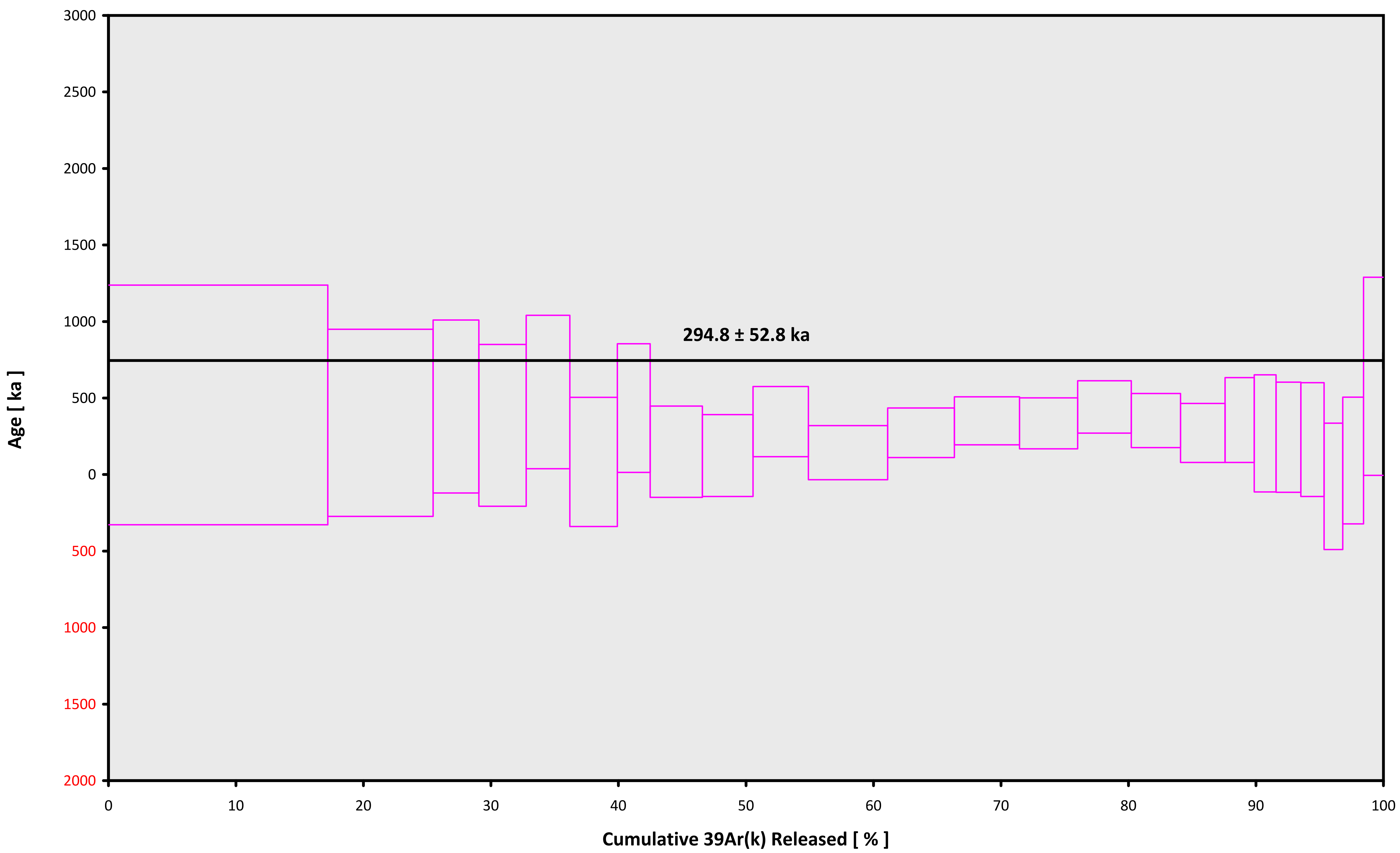

Ar-Ages in ka

WEIGHTED PLATEAU
294.8 ± 52.8
TOTAL FUSION
323.1 ± 152.7
NORMAL ISOCHRON
292.5 ± 85.6
INVERSE ISOCHRON
293.9 ± 71.1

MSWD (PROBABILITY)
0.81 (72%)

Sample Info

Groundmass
Gakkel Ridge
Dan Miggins

IRR = 17-OSU-01 (1C38-17)
J = 0.00153965 ± 0.00000128

17D20055.full.xls printed at 17-09-2017 (14:24)
ArArCALC v2.7.0 -- Beta Version

17D20055.AGE >>> PS59-310-1-1 >>> ARCTIC | O-CONNOR (16-22) PROJECT

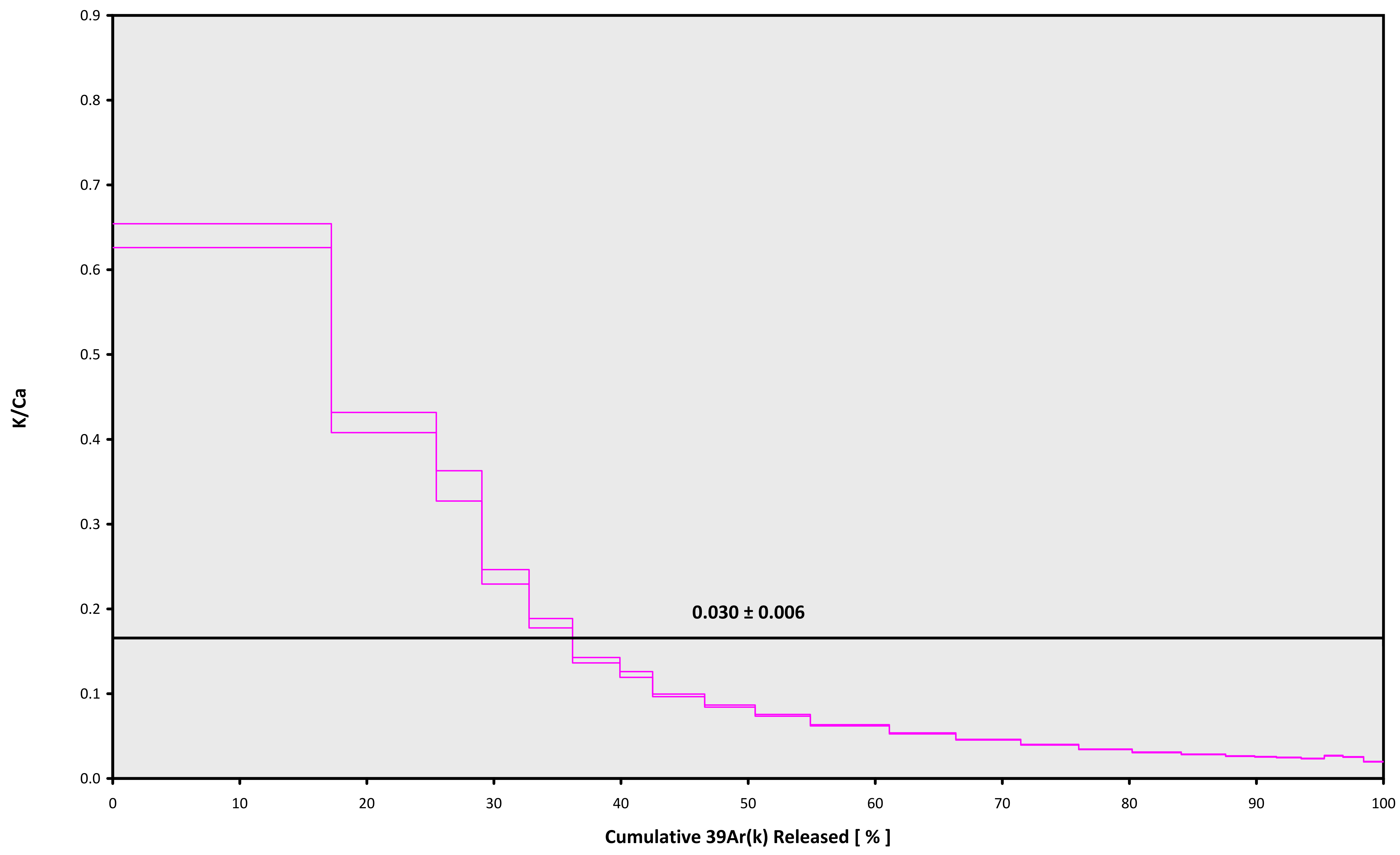

Ar-Ages in ka

WEIGHTED PLATEAU
294.8 ± 52.8
TOTAL FUSION
323.1 ± 152.7
NORMAL ISOCHRON
292.5 ± 85.6
INVERSE ISOCHRON
293.9 ± 71.1

Sample Info

Groundmass
Gakkel Ridge
Dan Miggins

IRR = 17-OSU-01 (1C38-17)
J = 0.00153965 ± 0.00000128

OSU Argon Geochronology Lab
CEOAS Oregon State University, Corvallis, USA

17D20055.AGE >>> PS59-310-1-1 >>> ARCTIC | O-CONNOR (16-22) PROJECT

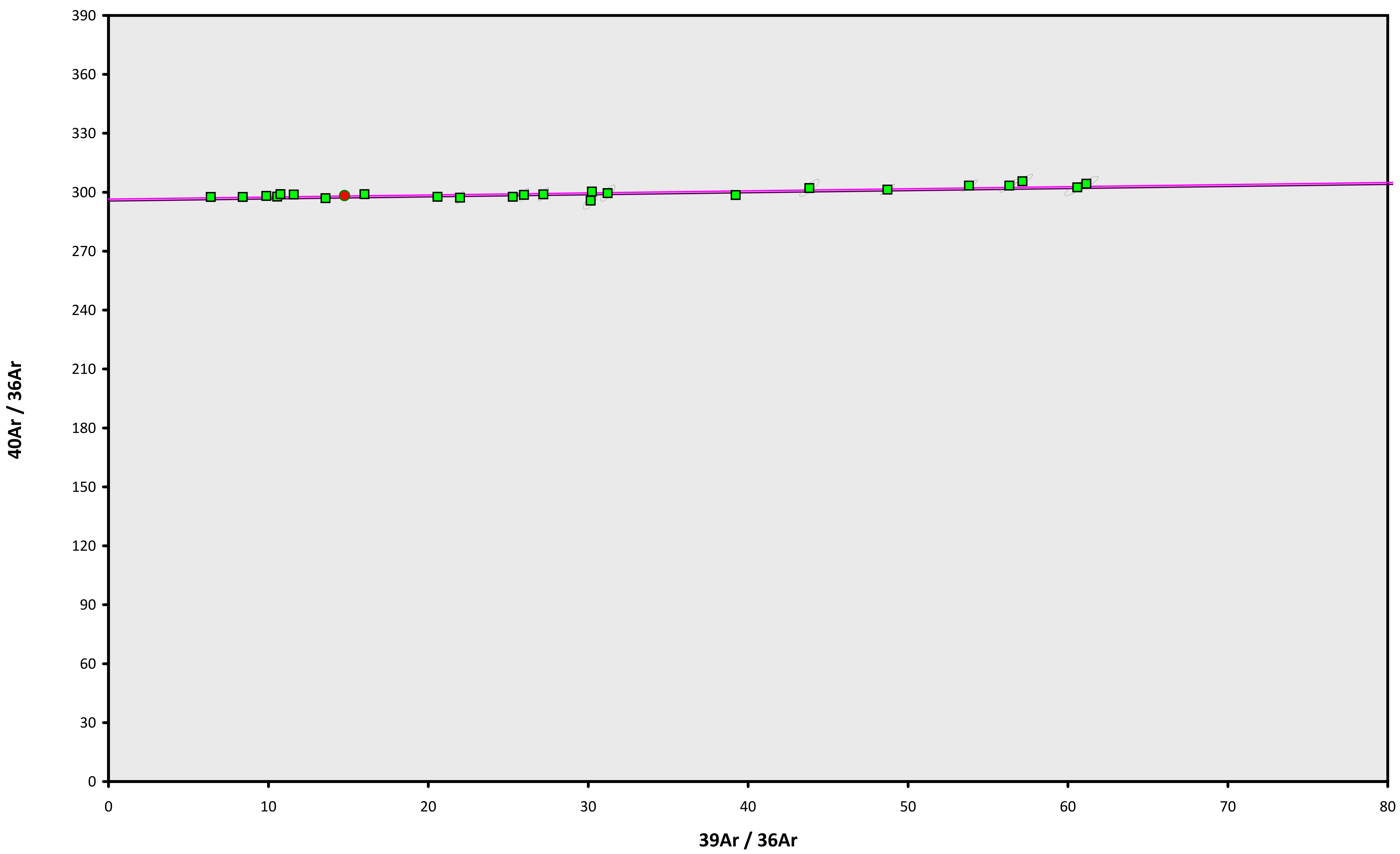

Ar-Ages in ka

WEIGHTED PLATEAU
294.8 ± 52.8
TOTAL FUSION
323.1 ± 152.7
NORMAL ISOCHRON
292.5 ± 85.6
INVERSE ISOCHRON
293.9 ± 71.1

MSWD (PROBABILITY)
0.94 (54%)

40AR/36AR INTERCEPT
296.5 ± 0.8

Sample Info

Groundmass
Gakkel Ridge
Dan Miggins

IRR = 17-OSU-01 (1C38-17)
J = 0.00153965 ± 0.00000128

17D20055.AGE >>> PS59-310-1-1 >>> ARCTIC | O-CONNOR (16-22) PROJECT

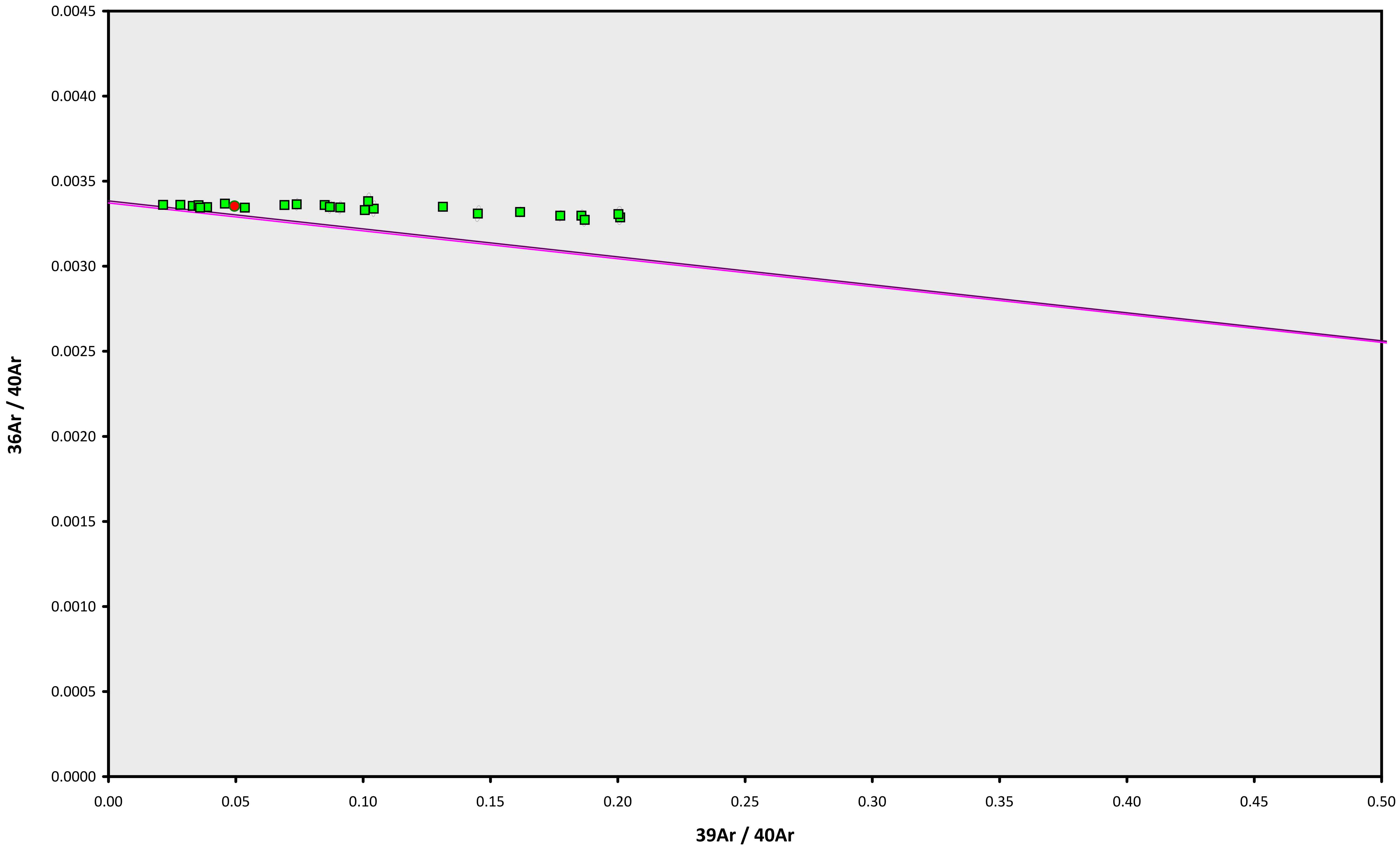

Ar-Ages in ka

WEIGHTED PLATEAU
294.8 ± 52.8
TOTAL FUSION
323.1 ± 152.7
NORMAL ISOCHRON
292.5 ± 85.6
INVERSE ISOCHRON
293.9 ± 71.1

MSWD (PROBABILITY)
0.94 (54%)

SPREADING FACTOR
1.9%

40AR/36AR INTERCEPT
296.5 ± 0.8

Sample Info

Groundmass
Gakkel Ridge
Dan Miggins

IRR = 17-OSU-01 (1C38-17)
J = 0.00153965 ± 0.00000128

Relative Abundances			36Ar [fA]	%1σ	37Ar [fA]	%1σ	38Ar [fA]	%1σ	39Ar [fA]	%1σ	40Ar [fA]	%1σ	40(r)/39(k) ± 2σ	Age ± 2σ (Ma)	40Ar(r) (%)	39Ar(k) (%)	K/Ca ± 2σ
18D00136	1.8 %	✓	2.923634	0.288	58.5331	4.987	1.2839743	2.035	52.53850	0.085	891.0760	0.034	0.46772 ± 0.15884	1.35 ± 0.46	2.76	17.89	0.3857 ± 0.0385
18D00138	1.9 %	✓	1.554825	0.303	72.3636	3.992	0.7359713	3.471	33.36224	0.098	477.0722	0.063	0.58717 ± 0.13653	1.70 ± 0.39	4.10	11.35	0.1980 ± 0.0158
18D00139	2.0 %	✓	0.463921	0.412	26.4327	11.047	0.2358924	10.512	11.59569	0.212	143.5687	0.209	0.64322 ± 0.14865	1.86 ± 0.43	5.19	3.95	0.1884 ± 0.0416
18D00141	2.2 %	✓	0.563864	0.386	62.1029	4.643	0.3156647	7.885	15.47046	0.170	173.8836	0.176	0.70329 ± 0.12679	2.03 ± 0.37	6.24	5.26	0.1068 ± 0.0099
18D00142	2.4 %	✓	0.364333	0.461	57.5102	4.855	0.2270709	11.279	10.88200	0.233	110.9052	0.269	0.64229 ± 0.13655	1.86 ± 0.39	6.28	3.70	0.0811 ± 0.0079
18D00144	2.7 %	✓	0.325194	0.483	75.0259	3.600	0.2342915	11.255	11.51998	0.214	98.0178	0.303	0.62346 ± 0.12024	1.80 ± 0.35	7.30	3.91	0.0657 ± 0.0047
18D00145	3.0 %	✓	0.310351	0.512	113.4052	2.573	0.2601443	10.046	13.78179	0.185	92.0357	0.324	0.63337 ± 0.10008	1.83 ± 0.29	9.43	4.67	0.0520 ± 0.0027
18D00147	3.4 %	✓	0.233258	0.583	104.9218	2.765	0.2175814	11.694	11.63118	0.219	67.0843	0.439	0.52079 ± 0.10387	1.51 ± 0.30	8.98	3.94	0.0474 ± 0.0026
18D00148	3.9 %	✓	0.292439	0.501	161.0480	1.840	0.2765583	9.566	15.01758	0.171	82.5546	0.359	0.56161 ± 0.08652	1.62 ± 0.25	10.15	5.08	0.0398 ± 0.0015
18D00150	4.5 %	✓	0.190022	0.676	139.5192	2.033	0.2145092	12.150	11.48291	0.223	51.6794	0.569	0.55192 ± 0.09864	1.60 ± 0.29	12.17	3.88	0.0351 ± 0.0014
18D00151	5.2 %	✓	0.220402	0.608	197.9559	1.566	0.2671544	9.732	14.59289	0.174	57.9679	0.508	0.56886 ± 0.08129	1.65 ± 0.24	14.20	4.93	0.0314 ± 0.0010
18D00153	6.0 %	✓	0.222832	0.611	219.7101	1.403	0.2520527	9.630	14.92668	0.173	57.3452	0.514	0.58381 ± 0.07987	1.69 ± 0.23	15.05	5.04	0.0289 ± 0.0008
18D00154	6.9 %	✓	0.238731	0.567	249.4742	1.316	0.2773345	9.313	16.05666	0.158	60.3773	0.488	0.58652 ± 0.07537	1.70 ± 0.22	15.44	5.42	0.0274 ± 0.0007
18D00156	7.9 %		0.194575	0.650	187.3860	1.631	0.1896521	13.202	11.85411	0.221	48.7029	0.604	0.49497 ± 0.09595	1.43 ± 0.28	11.92	4.00	0.0269 ± 0.0009
18D00157	9.0 %		0.205551	0.647	184.4489	1.622	0.2018975	11.741	11.47213	0.217	51.8801	0.567	0.48249 ± 0.10176	1.40 ± 0.29	10.56	3.87	0.0265 ± 0.0009
18D00159	10.3 %		0.243836	0.566	183.9198	1.725	0.2080690	12.169	10.27649	0.239	61.9578	0.475	0.40429 ± 0.11984	1.17 ± 0.35	6.63	3.46	0.0237 ± 0.0008
18D00160	11.6 %		0.329598	0.482	188.4415	1.658	0.1924102	13.095	7.73014	0.326	86.1216	0.342	0.40480 ± 0.18132	1.17 ± 0.52	3.58	2.59	0.0174 ± 0.0006
18D00162	12.5 %		0.286761	0.515	196.3418	1.593	0.1525504	16.933	6.46484	0.373	71.6874	0.411	0.32283 ± 0.20325	0.93 ± 0.59	2.85	2.16	0.0139 ± 0.0005
18D00163	13.4 %		0.115404	0.978	67.9358	4.061	0.0742032	34.841	2.22045	1.072	29.5196	0.996	0.27705 ± 0.46956	0.80 ± 1.36	2.04	0.74	0.0138 ± 0.0012
18D00165	14.6 %		0.224219	0.606	148.2621	2.058	0.1408792	18.497	3.44632	0.689	55.9961	0.525	0.33586 ± 0.35583	0.97 ± 1.03	2.01	1.14	0.0097 ± 0.0004
18D00166	16.0 %		0.281355	0.522	176.5027	1.770	0.1239593	20.597	3.67255	0.653	69.9333	0.420	0.08640 ± 0.36080	0.25 ± 1.04	0.44	1.21	0.0087 ± 0.0003
18D00168	17.6 %		0.185792	0.655	117.6424	2.540	0.0763004	34.296	2.25986	1.036	45.4770	0.647	0.18901 ± 0.50714	0.55 ± 1.47	0.91	0.74	0.0080 ± 0.0004
18D00169	19.3 %		0.189574	0.693	141.7825	2.143	0.0876549	30.140	2.23128	1.051	45.7397	0.643	0.31227 ± 0.53661	0.90 ± 1.55	1.46	0.73	0.0065 ± 0.0003
18D00171	21.0 %		0.101503	1.098	57.1283	4.864	0.0396026	65.798	0.97991	2.424	25.4131	1.157	0.24668 ± 1.07392	0.71 ± 3.11	0.92	0.32	0.0071 ± 0.0008
		Σ	10.261976	0.115	3187.7947	0.458	6.2853789	1.997	295.46662	0.044	2955.9966	0.049					

Information on Analysis and Constants Used in Calculations

Project = **O-CONNOR (16-22)**
Sample = **HLY0102-D95-38**
Material = **Groundmass**
Location = **Gakkel Ridge**
Region = **Artic Ocean**
Analyst = **Dan Miggins**
Irradiation = **17-OSU-05 (5B20-17)**
Position = **X: 0 | Y: 0 | Z/H: 29.19945 mm**
FCT-NM Age = **28.201 ± 0.023 Ma**
FCT-NM Reference = **Kuiper et al (2008)**
FCT-NM 40Ar/39Ar Ratio = **9.82234 ± 0.00796**
FCT-NM J-value = **0.00160017 ± 0.00000130**
Air Shot 40Ar/36Ar = **302.9270 ± 0.3968**
Air Shot MDF = **0.99386271 ± 0.00066124 (LIN)**
Experiment Type = **Incremental Heating**
Extraction Method = **Bulk Laser Heating**
Heating = **64 sec**
Isolation = **3.00 min**
Instrument = **ARGUS-VI-D**
Preferred Age = **Plateau Age**
Age Classification = **Crystallization Age**
IGSN = Undefined
Rock Class = **Igneous>Volcanic**
Lithology = **Basaltic Lava**
Lat-Lon = Undefined - Undefined

Age Equations = **Min et al. (2000)**
Negative Intensities = **Allowed**
Collector Calibrations = **36Ar**
Decay 40K = **5.530 ± 0.048 E-10 1/a**
Decay 39Ar = **2.940 ± 0.016 E-07 1/h**
Decay 37Ar = **8.230 ± 0.012 E-04 1/h**
Decay 36Cl = **2.257 ± 0.015 E-06 1/a**
Decay 40K(EC,β^+) = **0.580 ± 0.009 E-10 1/a**
Decay 40K(β^-) = **4.950 ± 0.043 E-10 1/a**
Atmospheric 40/36(a) = **297.99 ± 1.14**
Atmospheric 38/36(a) = **0.1869**
Production 39/37(ca) = **0.0006425 ± 0.0000059**
Production 38/37(ca) = **0.0001800 ± 0.0000173**
Production 36/37(ca) = **0.0002703 ± 0.0000005**
Production 40/39(k) = **0.000607 ± 0.000059**
Production 38/39(k) = **0.012077 ± 0.000011**
Production 36/38(cl) = **262.80 ± 1.71**
Scaling Ratio K/Ca = **0.430**
Abundance Ratio 40K/K = **1.1700 ± 0.0100 E-04**
Atomic Weight K = **39.0983 ± 0.0001 g**

Results

Results	40(a)/36(a) ± 2σ	40(r)/39(k) ± 2σ	Age ± 2σ (Ma)	MSWD	39Ar(k) (%,n)	K/Ca ± 2σ
**Age Plateau**		0.58596 ± 0.02844<br>± 4.85%	**1.69** ± 0.08<br>**± 4.85%**	0.90<br>55%	79.02<br>13	0.0320 ± 0.0056
		Full External Error	± 0.09	1.82	2σ Confidence Limit	
		Analytical Error	± 0.08	1.0000	Error Magnification	
**Total Fusion Age**		0.52712 ± 0.03840<br>± 7.28%	**1.52** ± 0.11<br>**± 7.28%**		24	0.0396 ± 0.0004
		Full External Error	± 0.12			
		Analytical Error	± 0.11			
**Normal Isochron**	**297.95** ± 2.26<br>± 0.76%	0.58581 ± 0.06160<br>± 10.52%	**1.69** ± 0.18<br>**± 10.51%**	1.66<br>8%	79.02<br>13	
		Full External Error	± 0.18	1.85	2σ Confidence Limit	
		Analytical Error	± 0.18	1.2886	Error Magnification	
				5	Number of Iterations	
				0.0000020788	Convergence	
**Inverse Isochron**	**297.99** ± 2.27<br>± 0.76%	0.58585 ± 0.06083<br>± 10.38%	**1.69** ± 0.18<br>**± 10.38%**	1.66<br>7%	79.02<br>13	
		Full External Error	± 0.18	1.85	2σ Confidence Limit	
		Analytical Error	± 0.18	1.2896	Error Magnification	
				4	Number of Iterations	
				0.0001741492	Convergence	
				12%	Spreading Factor	

Notes
Excess Initial 40Ar/36Ar = 297.99 ± 0.38 (%SD).

Incremental Heating			36Ar(a) [fA]	37Ar(ca) [fA]	38Ar(cl) [fA]	39Ar(k) [fA]	40Ar(r) [fA]	Age ± 2σ (Ma)	40Ar(r) (%)	39Ar(k) (%)	K/Ca ± 2σ
18D00136	1.8 %	✓	2.9077762	58.5331	0.0959217	52.50090	24.55596	1.35 ± 0.46	2.76	17.89	0.3857 ± 0.0385
18D00138	1.9 %	✓	1.5352523	72.3636	0.0336529	33.31575	19.56214	1.70 ± 0.39	4.10	11.35	0.1980 ± 0.0158
18D00139	2.0 %	✓	0.4567739	26.4327	0.0059274	11.57871	7.44762	1.86 ± 0.43	5.19	3.95	0.1884 ± 0.0416
18D00141	2.2 %	✓	0.5470720	62.1029	0.0158836	15.43056	10.85223	2.03 ± 0.37	6.24	5.26	0.1068 ± 0.0099
18D00142	2.4 %	✓	0.3487801	57.5102	0.0205565	10.84505	6.96566	1.86 ± 0.39	6.28	3.70	0.0811 ± 0.0079
18D00144	2.7 %	✓	0.3049052	75.0259	0.0252553	11.47178	7.15216	1.80 ± 0.35	7.30	3.91	0.0657 ± 0.0047
18D00145	3.0 %	✓	0.2796891	113.4052	0.0218948	13.70893	8.68278	1.83 ± 0.29	9.43	4.67	0.0520 ± 0.0027
18D00147	3.4 %	✓	0.2048893	104.9218	0.0207461	11.56376	6.02229	1.51 ± 0.30	8.98	3.94	0.0474 ± 0.0026
18D00148	3.9 %	✓	0.2489000	161.0480	0.0209326	14.91410	8.37585	1.62 ± 0.25	10.15	5.08	0.0398 ± 0.0015
18D00150	4.5 %	✓	0.1523014	139.5192	0.0233341	11.39327	6.28821	1.60 ± 0.29	12.17	3.88	0.0351 ± 0.0014
18D00151	5.2 %	✓	0.1668851	197.9559	0.0256293	14.46570	8.22902	1.65 ± 0.24	14.20	4.93	0.0314 ± 0.0010
18D00153	6.0 %	✓	0.1634427	219.7101	0.0033927	14.78552	8.63197	1.69 ± 0.23	15.05	5.04	0.0289 ± 0.0008
18D00154	6.9 %	✓	0.1712947	249.4742	0.0084337	15.89637	9.32349	1.70 ± 0.22	15.44	5.42	0.0274 ± 0.0007
18D00156	7.9 %		0.1439244	187.3860	0.0000000	11.73372	5.80778	1.43 ± 0.28	11.92	4.00	0.0269 ± 0.0009
18D00157	9.0 %		0.1556939	184.4489	0.0024799	11.35362	5.47796	1.40 ± 0.29	10.56	3.87	0.0265 ± 0.0009
18D00159	10.3 %		0.1941163	183.9198	0.0160011	10.15832	4.10693	1.17 ± 0.35	6.63	3.46	0.0237 ± 0.0008
18D00160	11.6 %		0.2786563	188.4415	0.0145152	7.60907	3.08016	1.17 ± 0.52	3.58	2.59	0.0174 ± 0.0006
18D00162	12.5 %		0.2336901	196.3418	0.0000000	6.33869	2.04630	0.93 ± 0.59	2.85	2.16	0.0139 ± 0.0005
18D00163	13.4 %		0.0970343	67.9358	0.0175498	2.17680	0.60307	0.80 ± 1.36	2.04	0.74	0.0138 ± 0.0012
18D00165	14.6 %		0.1841288	148.2621	0.0393076	3.35106	1.12550	0.97 ± 1.03	2.01	1.14	0.0097 ± 0.0004
18D00166	16.0 %		0.2336441	176.5027	0.0055369	3.55915	0.30751	0.25 ± 1.04	0.44	1.21	0.0087 ± 0.0003
18D00168	17.6 %		0.1539936	117.6424	0.0000000	2.18427	0.41284	0.55 ± 1.47	0.91	0.74	0.0080 ± 0.0004
18D00169	19.3 %		0.1512471	141.7825	0.0080189	2.14019	0.66832	0.90 ± 1.55	1.46	0.73	0.0065 ± 0.0003
18D00171	21.0 %		0.0860607	57.1283	0.0018437	0.94320	0.23267	0.71 ± 3.11	0.92	0.32	0.0071 ± 0.0008
		Σ	9.4001514	3187.7947	0.4268139	293.41846	154.66739				

Information on Analysis

Project = **O-CONNOR (16-22)**
Sample = **HLY0102-D95-38**
Material = **Groundmass**
Location = **Gakkel Ridge**
Region = **Artic Ocean**
Analyst = **Dan Miggins**
Irradiation = **17-OSU-05 (5B20-17)**
J = **0.00160017 ± 0.00000130**
FCT-NM = **28.201 ± 0.023 Ma**

Results	40(r)/39(k) ± 2σ		Age ± 2σ (Ma)		MSWD	39Ar(k) (%,n)	K/Ca ± 2σ
**Age Plateau**	0.58596	± 0.02844<br>± 4.85%	**1.69**	**± 0.08**<br>**± 4.85%**	0.90<br>55%	79.02<br>13	0.0320 ± 0.0056
		Full External Error		± 0.09	1.82	2σ Confidence Limit	
		Analytical Error		± 0.08	1.0000	Error Magnification	
**Total Fusion Age**	0.52712	± 0.03840<br>± 7.28%	**1.52**	**± 0.11**<br>**± 7.28%**		24	0.0396 ± 0.0004
		Full External Error		± 0.12			
		Analytical Error		± 0.11			

Normal Isochron			39(k)/36(a) ± 2σ	40(a+r)/36(a) ± 2σ	r.i.
18D00136	1.8 %	✓	18.06 ± 0.11	306.43 ± 1.79	0.9535
18D00138	1.9 %	✓	21.70 ± 0.14	310.73 ± 1.97	0.9342
18D00139	2.0 %	✓	25.35 ± 0.25	314.29 ± 3.14	0.8210
18D00141	2.2 %	✓	28.21 ± 0.26	317.83 ± 2.91	0.8560
18D00142	2.4 %	✓	31.09 ± 0.36	317.96 ± 3.77	0.8145
18D00144	2.7 %	✓	37.62 ± 0.46	321.45 ± 4.14	0.8252
18D00145	3.0 %	✓	49.01 ± 0.65	329.03 ± 4.69	0.8544
18D00147	3.4 %	✓	56.44 ± 0.90	327.38 ± 5.79	0.8339
18D00148	3.9 %	✓	59.92 ± 0.83	331.64 ± 5.05	0.8540
18D00150	4.5 %	✓	74.81 ± 1.51	339.28 ± 7.71	0.8433
18D00151	5.2 %	✓	86.68 ± 1.67	347.30 ± 7.48	0.8666
18D00153	6.0 %	✓	90.46 ± 1.80	350.80 ± 7.75	0.8715
18D00154	6.9 %	✓	92.80 ± 1.78	352.42 ± 7.51	0.8763
18D00156	7.9 %		81.53 ± 1.75	338.34 ± 8.21	0.8481
18D00157	9.0 %		72.92 ± 1.49	333.17 ± 7.67	0.8498
18D00159	10.3 %		52.33 ± 0.91	319.15 ± 6.15	0.8357
18D00160	11.6 %		27.31 ± 0.40	309.04 ± 4.52	0.7863
18D00162	12.5 %		27.12 ± 0.45	306.75 ± 5.13	0.7717
18D00163	13.4 %		22.43 ± 0.80	304.21 ± 10.42	0.6398
18D00165	14.6 %		18.20 ± 0.41	304.10 ± 6.15	0.6598
18D00166	16.0 %		15.23 ± 0.30	299.31 ± 5.02	0.6327
18D00168	17.6 %		14.18 ± 0.41	295.31 ± 6.78	0.5464
18D00169	19.3 %		14.15 ± 0.43	302.41 ± 7.32	0.5777
18D00171	21.0 %		10.96 ± 0.65	295.29 ± 11.48	0.4226

Results	40(a)/36(a) ± 2σ		40(r)/39(k) ± 2σ		Age ± 2σ (Ma)		MSWD
Normal Isochron	**297.95**	**± 2.26**<br>**± 0.76%**	0.58581	± 0.06160<br>± 10.52%	**1.69**	**± 0.18**<br>**± 10.51%**	1.66<br>8%
					Full External Error	± 0.18	
					Analytical Error	± 0.18	

Statistics				
	2σ Confidence Limit	1.85	Convergence	0.000002078794
	Error Magnification	1.2886	Number of Iterations	5
	Number of Data Points	13	Calculated Line	Weighted York-2

**Inverse Isochron**			39(k)/40(a+r) ± 2σ	36(a)/40(a+r) ± 2σ	r.i.
18D00136	1.8 %	✓	0.0589206 ± 0.0001076	0.00326334 ± 0.00001909	0.0428
18D00138	1.9 %	✓	0.0698367 ± 0.0001636	0.00321821 ± 0.00002044	0.1084
18D00139	2.0 %	✓	0.0806532 ± 0.0004822	0.00318173 ± 0.00003174	0.2939
18D00141	2.2 %	✓	0.0887455 ± 0.0004354	0.00314637 ± 0.00002882	0.2745
18D00142	2.4 %	✓	0.0977924 ± 0.0006978	0.00314503 ± 0.00003729	0.3423
18D00144	2.7 %	✓	0.1170460 ± 0.0008701	0.00311093 ± 0.00004008	0.3838
18D00145	3.0 %	✓	0.1489658 ± 0.0011145	0.00303920 ± 0.00004333	0.3948
18D00147	3.4 %	✓	0.1723947 ± 0.0016950	0.00305453 ± 0.00005399	0.4444
18D00148	3.9 %	✓	0.1806772 ± 0.0014397	0.00301530 ± 0.00004591	0.4246
18D00150	4.5 %	✓	0.2204900 ± 0.0027009	0.00294744 ± 0.00006695	0.4663
18D00151	5.2 %	✓	0.2495846 ± 0.0026861	0.00287936 ± 0.00006198	0.4466
18D00153	6.0 %	✓	0.2578738 ± 0.0027999	0.00285060 ± 0.00006300	0.4398
18D00154	6.9 %	✓	0.2633262 ± 0.0027076	0.00283753 ± 0.00006048	0.4352
18D00156	7.9 %		0.2409594 ± 0.0031046	0.00295558 ± 0.00007168	0.4672
18D00157	9.0 %		0.2188726 ± 0.0026621	0.00300143 ± 0.00006906	0.4596
18D00159	10.3 %		0.1639717 ± 0.0017488	0.00313335 ± 0.00006036	0.4389
18D00160	11.6 %		0.0883574 ± 0.0008430	0.00323579 ± 0.00004735	0.3345
18D00162	12.5 %		0.0884259 ± 0.0009916	0.00326002 ± 0.00005454	0.3594
18D00163	13.4 %		0.0737441 ± 0.0021842	0.00328726 ± 0.00011265	0.3907
18D00165	14.6 %		0.0598467 ± 0.0010588	0.00328836 ± 0.00006652	0.3082
18D00166	16.0 %		0.0508950 ± 0.0008109	0.00334106 ± 0.00005603	0.2646
18D00168	17.6 %		0.0480317 ± 0.0012063	0.00338628 ± 0.00007776	0.2901
18D00169	19.3 %		0.0467919 ± 0.0011924	0.00330678 ± 0.00008004	0.2681
18D00171	21.0 %		0.0371155 ± 0.0020623	0.00338654 ± 0.00013165	0.2479

**Results**	40(a)/36(a) ± 2σ		40(r)/39(k) ± 2σ		Age ± 2σ (Ma)		MSWD
**Inverse Isochron**	**297.99**	**± 2.27**<br>**± 0.76%**	0.58585	± 0.06083<br>± 10.38%	**1.69**	**± 0.18**<br>**± 10.38%**	1.66<br>7%
					Full External Error	± 0.18	
					Analytical Error	± 0.18	

**Statistics**				
2σ Confidence Limit	1.85	Convergence	0.0001741492
Error Magnification	1.2896	Number of Iterations	4
Number of Data Points	13	Calculated Line	Weighted York-2
Spreading Factor	12.0%		

Degassing Patterns			36Ar(a) [fA]	%1σ	36Ar(c) [fA]	%1σ	36Ar(ca) [fA]	%1σ	36Ar(cl) [fA]	%1σ	37Ar(ca) [fA]	%1σ	38Ar(a) [fA]	%1σ	38Ar(c) [fA]	%1σ	38Ar(k) [fA]	%1σ	38Ar(ca) [fA]	%1σ	38Ar(cl) [fA]	%1σ	39Ar(k) [fA]	%1σ	39Ar(ca) [fA]	%1σ	40Ar(r) [fA]	%1σ	40Ar(a) [fA]	%1σ	40Ar(c) [fA]	%1σ	40Ar(k) [fA]	%1σ
18D00136	1.8 %	✓	2.9077762	0.29	0.0000000	0.00	0.0158215	4.99	0.0000368	27.35	58.5331	4.99	0.5434634	0.29	0.0000000	0.00	0.6340533	0.12	0.0105360	10.84	0.0959217	27.37	52.50090	0.08	0.0376075	5.07	24.55596	16.98	866.4882	0.48	0.0000000	0.00	0.0318680	9.65
18D00138	1.9 %	✓	1.5352523	0.31	0.0000000	0.00	0.0195599	4.00	0.0000129	76.09	72.3636	3.99	0.2869387	0.31	0.0000000	0.00	0.4023543	0.13	0.0130254	10.42	0.0336529	76.10	33.31575	0.10	0.0464936	4.10	19.56214	11.63	457.4898	0.49	0.0000000	0.00	0.0202227	9.65
18D00139	2.0 %	✓	0.4567739	0.45	0.0000000	0.00	0.0071448	11.05	0.0000023	418.62	26.4327	11.05	0.0853710	0.45	0.0000000	0.00	0.1398360	0.23	0.0047579	14.65	0.0059274	418.62	11.57871	0.21	0.0169830	11.08	7.44762	11.55	136.1140	0.59	0.0000000	0.00	0.0070283	9.65
18D00141	2.2 %	✓	0.5470720	0.42	0.0000000	0.00	0.0167864	4.65	0.0000061	156.94	62.1029	4.64	0.1022478	0.42	0.0000000	0.00	0.1863548	0.19	0.0111785	10.69	0.0158836	156.94	15.43056	0.17	0.0399011	4.73	10.85223	9.01	163.0220	0.57	0.0000000	0.00	0.0093663	9.65
18D00142	2.4 %	✓	0.3487801	0.53	0.0000000	0.00	0.0155450	4.86	0.0000079	124.74	57.5102	4.85	0.0651870	0.53	0.0000000	0.00	0.1309756	0.25	0.0103518	10.78	0.0205565	124.75	10.84505	0.23	0.0369503	4.94	6.96566	10.63	103.9330	0.65	0.0000000	0.00	0.0065829	9.65
18D00144	2.7 %	✓	0.3049052	0.57	0.0000000	0.00	0.0202795	3.60	0.0000097	104.59	75.0259	3.60	0.0569868	0.57	0.0000000	0.00	0.1385447	0.23	0.0135047	10.28	0.0252553	104.59	11.47178	0.22	0.0482042	3.72	7.15216	9.64	90.8587	0.68	0.0000000	0.00	0.0069634	9.65
18D00145	3.0 %	✓	0.2796891	0.63	0.0000000	0.00	0.0306534	2.58	0.0000084	119.76	113.4052	2.57	0.0522739	0.63	0.0000000	0.00	0.1655627	0.21	0.0204129	9.97	0.0218948	119.76	13.70893	0.19	0.0728628	2.73	8.68278	7.90	83.3446	0.74	0.0000000	0.00	0.0083213	9.65
18D00147	3.4 %	✓	0.2048893	0.77	0.0000000	0.00	0.0283604	2.77	0.0000080	123.02	104.9218	2.76	0.0382938	0.77	0.0000000	0.00	0.1396556	0.24	0.0188859	10.02	0.0207461	123.02	11.56376	0.22	0.0674122	2.91	6.02229	9.97	61.0550	0.86	0.0000000	0.00	0.0070192	9.65
18D00148	3.9 %	✓	0.2489000	0.67	0.0000000	0.00	0.0435313	1.85	0.0000080	127.15	161.0480	1.84	0.0465194	0.67	0.0000000	0.00	0.1801176	0.19	0.0289886	9.80	0.0209326	127.15	14.91410	0.17	0.1034733	2.06	8.37585	7.70	74.1697	0.77	0.0000000	0.00	0.0090529	9.65
18D00150	4.5 %	✓	0.1523014	0.98	0.0000000	0.00	0.0377120	2.04	0.0000090	112.23	139.5192	2.03	0.0284651	0.98	0.0000000	0.00	0.1375965	0.24	0.0251135	9.84	0.0233341	112.23	11.39327	0.23	0.0896411	2.23	6.28821	8.93	45.3843	1.05	0.0000000	0.00	0.0069157	9.65
18D00151	5.2 %	✓	0.1668851	0.95	0.0000000	0.00	0.0535075	1.58	0.0000098	102.38	197.9559	1.57	0.0311908	0.95	0.0000000	0.00	0.1747023	0.20	0.0356321	9.76	0.0256293	102.38	14.46570	0.18	0.1271867	1.82	8.22902	7.14	49.7301	1.02	0.0000000	0.00	0.0087807	9.65
18D00153	6.0 %	✓	0.1634427	0.98	0.0000000	0.00	0.0593876	1.41	0.0000013	724.58	219.7101	1.40	0.0305474	0.98	0.0000000	0.00	0.1785647	0.20	0.0395478	9.73	0.0033927	724.58	14.78552	0.18	0.1411638	1.68	8.63197	6.84	48.7043	1.05	0.0000000	0.00	0.0089748	9.65
18D00154	6.9 %	✓	0.1712947	0.95	0.0000000	0.00	0.0674329	1.33	0.0000032	310.66	249.4742	1.32	0.0320150	0.95	0.0000000	0.00	0.1919805	0.18	0.0449054	9.72	0.0084337	310.66	15.89637	0.16	0.1602872	1.61	9.32349	6.42	51.0441	1.02	0.0000000	0.00	0.0096491	9.65
18D00156	7.9 %		0.1439244	1.05	0.0000000	0.00	0.0506504	1.64	0.0000000	0.00	187.3860	1.63	0.0268995	1.05	0.0000000	0.00	0.1417081	0.24	0.0337295	9.77	0.0000000	0.00	11.73372	0.22	0.1203955	1.87	5.80778	9.69	42.8880	1.12	0.0000000	0.00	0.0071224	9.65
18D00157	9.0 %		0.1556939	1.00	0.0000000	0.00	0.0498565	1.63	0.0000010	965.03	184.4489	1.62	0.0290992	1.00	0.0000000	0.00	0.1371176	0.24	0.0332008	9.77	0.0024799	965.03	11.35362	0.22	0.1185084	1.86	5.47796	10.54	46.3952	1.07	0.0000000	0.00	0.0068916	9.65
18D00159	10.3 %		0.1941163	0.84	0.0000000	0.00	0.0497135	1.73	0.0000061	159.56	183.9198	1.72	0.0362803	0.84	0.0000000	0.00	0.1226820	0.26	0.0331056	9.78	0.0160011	159.57	10.15832	0.24	0.1181685	1.95	4.10693	14.82	57.8447	0.92	0.0000000	0.00	0.0061661	9.65
18D00160	11.6 %		0.2786563	0.65	0.0000000	0.00	0.0509357	1.67	0.0000056	175.13	188.4415	1.66	0.0520809	0.65	0.0000000	0.00	0.0918947	0.34	0.0339195	9.77	0.0145152	175.13	7.60907	0.33	0.1210737	1.90	3.08016	22.39	83.0368	0.75	0.0000000	0.00	0.0046187	9.66
18D00162	12.5 %		0.2336901	0.73	0.0000000	0.00	0.0530712	1.60	0.0000000	0.00	196.3418	1.59	0.0436767	0.73	0.0000000	0.00	0.0765523	0.39	0.0353415	9.76	0.0000000	0.00	6.33869	0.38	0.1261496	1.84	2.04630	31.48	69.6373	0.82	0.0000000	0.00	0.0038476	9.66
18D00163	13.4 %		0.0970343	1.39	0.0000000	0.00	0.0183631	4.06	0.0000067	147.52	67.9358	4.06	0.0181357	1.39	0.0000000	0.00	0.0262892	1.10	0.0122285	10.45	0.0175498	147.52	2.17680	1.10	0.0436488	4.16	0.60307	84.74	28.9152	1.45	0.0000000	0.00	0.0013213	9.71
18D00165	14.6 %		0.1841288	0.86	0.0000000	0.00	0.0400753	2.07	0.0000151	66.65	148.2621	2.06	0.0344137	0.86	0.0000000	0.00	0.0404707	0.72	0.0266872	9.85	0.0393076	66.66	3.35106	0.71	0.0952584	2.25	1.12550	52.97	54.8685	0.95	0.0000000	0.00	0.0020341	9.68
18D00166	16.0 %		0.2336441	0.73	0.0000000	0.00	0.0477087	1.78	0.0000021	464.62	176.5027	1.77	0.0436681	0.73	0.0000000	0.00	0.0429838	0.68	0.0317705	9.79	0.0055369	464.62	3.55915	0.68	0.1134030	1.99	0.30751	208.80	69.6236	0.82	0.0000000	0.00	0.0021604	9.67
18D00168	17.6 %		0.1539936	0.95	0.0000000	0.00	0.0317987	2.55	0.0000000	0.00	117.6424	2.54	0.0287814	0.95	0.0000000	0.00	0.0263795	1.08	0.0211756	9.96	0.0000000	0.00	2.18427	1.08	0.0755852	2.70	0.41284	134.15	45.8885	1.02	0.0000000	0.00	0.0013259	9.71
18D00169	19.3 %		0.1512471	1.03	0.0000000	0.00	0.0383238	2.15	0.0000031	331.01	141.7825	2.14	0.0282681	1.03	0.0000000	0.00	0.0258470	1.10	0.0255208	9.87	0.0080189	331.01	2.14019	1.10	0.0910952	2.33	0.66832	85.91	45.0701	1.09	0.0000000	0.00	0.0012991	9.71
18D00171	21.0 %		0.0860607	1.56	0.0000000	0.00	0.0154418	4.87	0.0000007	######	57.1283	4.86	0.0160848	1.56	0.0000000	0.00	0.0113910	2.53	0.0102831	10.79	0.0018437	######	0.94320	2.53	0.0367049	4.95	0.23267	217.66	25.6452	1.61	0.0000000	0.00	0.0005725	9.98
		Σ	9.4001514	0.13	0.0000000	0.00	0.8616609	0.46	0.0001637	27.65	3187.7947	0.46	1.7568883	0.13	0.0000000	0.00	3.5436147	0.05	0.5738030	2.21	0.4268139	27.65	293.41846	0.04	2.0481581	0.50	154.66739	3.64	2801.1511	0.19	0.0000000	0.00	0.1781050	2.62
		Σ							10.261976	0.13	3187.7947	0.46									6.3011200	1.88			295.46662	0.04							2955.9966	0.26

Additional Parameters			40Ar/39Ar	1σ	37Ar/39Ar	1σ	36Ar/39Ar	1σ	Time (days)	37Ar (decay)	39Ar (decay)	40Ar (moles)
18D00136	1.8 %	✓	16.960438	0.015464	1.114099	0.055571	0.055647	0.000167	236.147	107.156794	1.00167120	4.277E-11
18D00138	1.9 %	✓	14.299764	0.016715	2.169027	0.086619	0.046604	0.000148	236.161	107.186195	1.00167130	2.290E-11
18D00139	2.0 %	✓	12.381212	0.036932	2.279532	0.251857	0.040008	0.000185	236.168	107.200898	1.00167135	6.891E-12
18D00141	2.2 %	✓	11.239717	0.027502	4.014288	0.186502	0.036448	0.000154	236.182	107.230311	1.00167145	8.346E-12
18D00142	2.4 %	✓	10.191623	0.036265	5.284894	0.256861	0.033480	0.000173	236.189	107.245021	1.00167150	5.323E-12
18D00144	2.7 %	✓	8.508504	0.031553	6.512676	0.234838	0.028229	0.000149	236.203	107.274446	1.00167160	4.705E-12
18D00145	3.0 %	✓	6.678063	0.024928	8.228625	0.212306	0.022519	0.000123	236.210	107.289161	1.00167165	4.418E-12
18D00147	3.4 %	✓	5.767625	0.028301	9.020734	0.250196	0.020055	0.000125	236.224	107.318598	1.00167174	3.220E-12
18D00148	3.9 %	✓	5.497201	0.021857	10.723966	0.198179	0.019473	0.000103	236.231	107.333320	1.00167179	3.963E-12
18D00150	4.5 %	✓	4.500551	0.027521	12.150166	0.248476	0.016548	0.000118	236.244	107.362769	1.00167189	2.481E-12
18D00151	5.2 %	✓	3.972338	0.021343	13.565236	0.213730	0.015103	0.000095	236.252	107.378969	1.00167195	2.782E-12
18D00153	6.0 %	✓	3.841793	0.020824	14.719287	0.208037	0.014928	0.000095	236.266	107.408431	1.00167204	2.753E-12
18D00154	6.9 %	✓	3.760263	0.019301	15.537120	0.205929	0.014868	0.000088	236.273	107.423165	1.00167209	2.898E-12
18D00156	7.9 %		4.108528	0.026420	15.807682	0.260131	0.016414	0.000113	236.287	107.452639	1.00167219	2.338E-12
18D00157	9.0 %		4.522272	0.027447	16.078006	0.263039	0.017917	0.000122	236.294	107.467378	1.00167224	2.490E-12
18D00159	10.3 %		6.029085	0.032043	17.897147	0.311653	0.023728	0.000146	236.308	107.496864	1.00167234	2.974E-12
18D00160	11.6 %		11.141010	0.052639	24.377502	0.412001	0.042638	0.000248	236.315	107.511610	1.00167239	4.134E-12
18D00162	12.5 %		11.088823	0.061486	30.370718	0.496949	0.044357	0.000282	236.328	107.541108	1.00167249	3.441E-12
18D00163	13.4 %		13.294439	0.194463	30.595522	1.285121	0.051973	0.000754	236.335	107.555861	1.00167253	1.417E-12
18D00165	14.6 %		16.248090	0.140801	43.020456	0.933839	0.065060	0.000597	236.349	107.585371	1.00167263	2.688E-12
18D00166	16.0 %		19.042158	0.147867	48.059985	0.906765	0.076610	0.000640	236.356	107.600129	1.00167268	3.357E-12
18D00168	17.6 %		20.123839	0.245846	52.057429	1.428216	0.082214	0.001008	236.371	107.631128	1.00167279	2.183E-12
18D00169	19.3 %		20.499313	0.252523	63.543067	1.516868	0.084962	0.001069	236.378	107.645892	1.00167283	2.196E-12
18D00171	21.0 %		25.934289	0.696622	58.299788	3.168562	0.103585	0.002757	236.392	107.675427	1.00167293	1.220E-12

Procedure Blanks		36Ar ± 1σ (SE) [fA]	37Ar ± 1σ (SE) [fA]	38Ar ± 1σ (SE) [fA]	39Ar ± 1σ (SE) [fA]	40Ar ± 1σ (SE) [fA]
18D00136	1.8 %	0.0286795 ± 0.0008534	0.0019240 ± 0.0180210	0.0409974 ± 0.0191869	0.0184427 ± 0.0176170	8.6418199 ± 0.2933613
18D00138	1.9 %	0.0313239 ± 0.0008534	0.0026754 ± 0.0180210	0.0338283 ± 0.0191869	0.0306455 ± 0.0176170	9.3196661 ± 0.2933613
18D00139	2.0 %	0.0320557 ± 0.0008534	0.0037425 ± 0.0180210	0.0306926 ± 0.0191869	0.0332340 ± 0.0176170	9.5052380 ± 0.2933613
18D00141	2.2 %	0.0326082 ± 0.0008534	0.0067134 ± 0.0180210	0.0252365 ± 0.0191869	0.0332840 ± 0.0176170	9.6394875 ± 0.2933613
18D00142	2.4 %	0.0325272 ± 0.0008534	0.0084246 ± 0.0180210	0.0228849 ± 0.0191869	0.0314264 ± 0.0176170	9.6136169 ± 0.2933613
18D00144	2.7 %	0.0318717 ± 0.0008534	0.0118823 ± 0.0180210	0.0188614 ± 0.0191869	0.0254519 ± 0.0176170	9.4333359 ± 0.2933613
18D00145	3.0 %	0.0313761 ± 0.0008534	0.0134882 ± 0.0180210	0.0171620 ± 0.0191869	0.0218587 ± 0.0176170	9.2994347 ± 0.2933613
18D00147	3.4 %	0.0302224 ± 0.0008534	0.0161676 ± 0.0180210	0.0143236 ± 0.0191869	0.0145734 ± 0.0176170	8.9892055 ± 0.2933613
18D00148	3.9 %	0.0296237 ± 0.0008534	0.0171526 ± 0.0180210	0.0131606 ± 0.0191869	0.0112479 ± 0.0176170	8.8284438 ± 0.2933613
18D00150	4.5 %	0.0285072 ± 0.0008534	0.0182564 ± 0.0180210	0.0112921 ± 0.0191869	0.0059513 ± 0.0176170	8.5283643 ± 0.2933613
18D00151	5.2 %	0.0279858 ± 0.0008534	0.0183291 ± 0.0180210	0.0105008 ± 0.0191869	0.0040504 ± 0.0176170	8.3878374 ± 0.2933613
18D00153	6.0 %	0.0272855 ± 0.0008534	0.0174637 ± 0.0180210	0.0094433 ± 0.0191869	0.0027726 ± 0.0176170	8.1981745 ± 0.2933613
18D00154	6.9 %	0.0270762 ± 0.0008534	0.0165654 ± 0.0180210	0.0090821 ± 0.0191869	0.0032373 ± 0.0176170	8.1408980 ± 0.2933613
18D00156	7.9 %	0.0269652 ± 0.0008534	0.0139449 ± 0.0180210	0.0086579 ± 0.0191869	0.0062951 ± 0.0176170	8.1090671 ± 0.2933613
18D00157	9.0 %	0.0270643 ± 0.0008534	0.0122917 ± 0.0180210	0.0085820 ± 0.0191869	0.0087781 ± 0.0176170	8.1350865 ± 0.2933613
18D00159	10.3 %	0.0275499 ± 0.0008534	0.0085371 ± 0.0180210	0.0086750 ± 0.0191869	0.0151806 ± 0.0176170	8.2662507 ± 0.2933613
18D00160	11.6 %	0.0279177 ± 0.0008534	0.0065566 ± 0.0180210	0.0088345 ± 0.0191869	0.0188328 ± 0.0176170	8.3670266 ± 0.2933613
18D00162	12.5 %	0.0288329 ± 0.0008534	0.0027563 ± 0.0180210	0.0093612 ± 0.0191869	0.0261747 ± 0.0176170	8.6218650 ± 0.2933613
18D00163	13.4 %	0.0293424 ± 0.0008534	0.0011095 ± 0.0180210	0.0097226 ± 0.0191869	0.0294399 ± 0.0176170	8.7666159 ± 0.2933613
18D00165	14.6 %	0.0303463 ± 0.0008534	0.0011803 ± 0.0180210	0.0106320 ± 0.0191869	0.0339015 ± 0.0176170	9.0613235 ± 0.2933613
18D00166	16.0 %	0.0307835 ± 0.0008534	0.0015985 ± 0.0180210	0.0111780 ± 0.0191869	0.0345162 ± 0.0176170	9.1970259 ± 0.2933613
18D00168	17.6 %	0.0313752 ± 0.0008534	0.0002043 ± 0.0180210	0.0125220 ± 0.0191869	0.0304704 ± 0.0176170	9.4103277 ± 0.2933613
18D00169	19.3 %	0.0314202 ± 0.0008534	0.0018358 ± 0.0180210	0.0132579 ± 0.0191869	0.0252172 ± 0.0176170	9.4573498 ± 0.2933613
18D00171	21.0 %	0.0308306 ± 0.0008534	0.0093548 ± 0.0180210	0.0149239 ± 0.0191869	0.0061783 ± 0.0176170	9.3902434 ± 0.2933613

**Intercept Values**		36Ar ± 1σ (SE) [fA]	r2	Regression (type,n)	37Ar ± 1σ (SE) [fA]	r2	Regression (type,n)	38Ar ± 1σ (SE) [fA]	r2	Regression (type,n)	39Ar ± 1σ (SE) [fA]	r2	Regression (type,n)	40Ar ± 1σ (SE) [fA]	r2	Regression (type,n)
18D00136	1.8 %	2.8085300 ± 0.0025654	0.9815	EXP 150 of 150	0.5342715 ± 0.0194049	0.0029	EXP 150 of 150	1.3092142 ± 0.0171844	0.1796	EXP 150 of 150	52.1480060 ± 0.0209718	0.9958	EXP 150 of 150	899.717868 ± 0.068303	0.9992	EXP 150 of 150
18D00138	1.9 %	1.5096830 ± 0.0018221	0.9644	EXP 150 of 150	0.6600330 ± 0.0188189	0.0681	EXP 150 of 150	0.7607675 ± 0.0163594	0.0312	EXP 150 of 150	33.1332050 ± 0.0161840	0.9936	EXP 150 of 150	486.391874 ± 0.075293	0.9904	EXP 150 of 150
18D00139	2.0 %	0.4731611 ± 0.0010712	0.8146	EXP 150 of 150	0.2382963 ± 0.0196800	0.0002	EXP 150 of 150	0.2636901 ± 0.0152202	0.0095	EXP 150 of 150	11.5386652 ± 0.0151315	0.9367	EXP 150 of 150	153.073928 ± 0.065371	0.9756	EXP 150 of 150
18D00141	2.2 %	0.5687419 ± 0.0012062	0.8652	EXP 150 of 150	0.5617931 ± 0.0188792	0.0153	EXP 150 of 150	0.3370272 ± 0.0153663	0.0205	EXP 150 of 150	15.3833219 ± 0.0164293	0.9651	EXP 150 of 150	183.523056 ± 0.084675	0.8745	EXP 150 of 150
18D00142	2.4 %	0.3789423 ± 0.0009717	0.7416	EXP 150 of 150	0.5179671 ± 0.0177482	0.0325	EXP 150 of 150	0.2471691 ± 0.0164841	0.0152	EXP 150 of 150	10.8287201 ± 0.0164448	0.9296	EXP 150 of 150	120.518838 ± 0.054526	0.9854	EXP 150 of 150
18D00144	2.7 %	0.3410731 ± 0.0008959	0.7223	EXP 150 of 150	0.6746426 ± 0.0162265	0.0389	EXP 149 of 150	0.2502776 ± 0.0176129	0.0156	EXP 150 of 150	11.4557627 ± 0.0151141	0.9460	EXP 150 of 150	107.451159 ± 0.046994	0.9883	EXP 150 of 150
18D00145	3.0 %	0.3264640 ± 0.0009574	0.6068	EXP 150 of 150	1.0240838 ± 0.0183460	0.1112	EXP 150 of 150	0.2741138 ± 0.0172658	0.0097	EXP 150 of 150	13.6963694 ± 0.0156346	0.9612	EXP 150 of 150	101.335088 ± 0.055648	0.9878	EXP 150 of 150
18D00147	3.4 %	0.2520084 ± 0.0007648	0.5926	EXP 150 of 150	0.9435242 ± 0.0183073	0.0322	EXP 150 of 150	0.2292347 ± 0.0162295	0.0352	EXP 150 of 150	11.5552115 ± 0.0163356	0.9400	EXP 150 of 150	76.073476 ± 0.028441	0.9967	EXP 150 of 150
18D00148	3.9 %	0.3076809 ± 0.0008030	0.7202	EXP 150 of 150	1.4557090 ± 0.0174826	0.1448	EXP 150 of 150	0.2863248 ± 0.0177364	0.0169	EXP 150 of 150	14.9119234 ± 0.0155710	0.9709	EXP 149 of 150	91.383069 ± 0.041273	0.9923	EXP 150 of 150
18D00150	4.5 %	0.2091843 ± 0.0007226	0.4445	EXP 150 of 150	1.2573644 ± 0.0164199	0.1450	EXP 150 of 150	0.2231688 ± 0.0171617	0.0194	EXP 150 of 150	11.3994726 ± 0.0166340	0.9373	EXP 150 of 150	60.207778 ± 0.023503	0.9978	EXP 150 of 150
18D00151	5.2 %	0.2375488 ± 0.0007569	0.5554	EXP 150 of 150	1.7913041 ± 0.0179208	0.2005	EXP 150 of 150	0.2743766 ± 0.0170659	0.0501	EXP 150 of 150	14.4833405 ± 0.0151339	0.9682	EXP 150 of 150	66.355716 ± 0.028626	0.9967	EXP 150 of 150
18D00153	6.0 %	0.2391583 ± 0.0007860	0.5405	EXP 150 of 150	1.9904865 ± 0.0165837	0.3294	EXP 150 of 150	0.2584027 ± 0.0143726	0.0350	EXP 150 of 150	14.8132589 ± 0.0158858	0.9676	EXP 150 of 150	65.543406 ± 0.025913	0.9969	EXP 150 of 150
18D00154	6.9 %	0.2540663 ± 0.0007416	0.6292	EXP 150 of 150	2.2630890 ± 0.0180471	0.3458	EXP 150 of 150	0.2830130 ± 0.0168087	0.0186	EXP 150 of 150	15.9349035 ± 0.0146411	0.9761	EXP 150 of 150	68.518153 ± 0.029003	0.9963	EXP 150 of 150
18D00156	7.9 %	0.2119709 ± 0.0006837	0.5787	EXP 150 of 150	1.6978879 ± 0.0177161	0.1703	EXP 150 of 150	0.1959825 ± 0.0156006	0.0020	EXP 150 of 150	11.7681260 ± 0.0173808	0.9363	EXP 150 of 150	56.812013 ± 0.021878	0.9977	EXP 150 of 150
18D00157	9.0 %	0.2225067 ± 0.0007679	0.4614	EXP 150 of 150	1.6724783 ± 0.0168936	0.2073	EXP 150 of 150	0.2080018 ± 0.0134178	0.0188	EXP 150 of 150	11.3915967 ± 0.0155012	0.9451	EXP 150 of 150	60.015159 ± 0.021522	0.9975	EXP 150 of 150
18D00159	10.3 %	0.2593941 ± 0.0007740	0.6890	EXP 150 of 150	1.6709394 ± 0.0194691	0.1829	EXP 150 of 150	0.2141904 ± 0.0160381	0.0051	EXP 150 of 150	10.2116679 ± 0.0153892	0.9341	EXP 150 of 150	70.224071 ± 0.021711	0.9959	EXP 150 of 150
18D00160	11.6 %	0.3413057 ± 0.0009153	0.7982	EXP 150 of 150	1.7139740 ± 0.0186317	0.2889	EXP 150 of 150	0.1988834 ± 0.0158485	0.0022	EXP 150 of 150	7.6887945 ± 0.0170389	0.8481	EXP 150 of 150	94.488587 ± 0.022531	0.9914	EXP 150 of 150
18D00162	12.5 %	0.3014913 ± 0.0008335	0.7502	EXP 150 of 150	1.7894147 ± 0.0183398	0.2725	EXP 150 of 150	0.1600394 ± 0.0168164	0.0002	EXP 150 of 150	6.4406848 ± 0.0155907	0.8222	EXP 150 of 150	80.309311 ± 0.023490	0.9925	EXP 150 of 150
18D00163	13.4 %	0.1390710 ± 0.0005784	0.2128	EXP 150 of 150	0.6189111 ± 0.0170554	0.1131	EXP 150 of 150	0.0830151 ± 0.0168501	0.0008	EXP 150 of 150	2.2326041 ± 0.0156470	0.1716	EXP 150 of 150	38.286257 ± 0.018213	0.9981	EXP 150 of 150
18D00165	14.6 %	0.2435384 ± 0.0007809	0.6620	EXP 149 of 150	1.3539325 ± 0.0190431	0.1500	EXP 150 of 150	0.1497823 ± 0.0171563	0.0178	EXP 150 of 150	3.4533888 ± 0.0154976	0.4658	EXP 150 of 150	65.057392 ± 0.019667	0.9952	EXP 150 of 150
18D00166	16.0 %	0.2983014 ± 0.0008326	0.7779	EXP 150 of 150	1.6117984 ± 0.0190565	0.0586	EXP 150 of 150	0.1336160 ± 0.0163645	0.0013	EXP 150 of 150	3.6784753 ± 0.0158064	0.5223	EXP 150 of 150	79.130305 ± 0.018791	0.9935	EXP 150 of 150
18D00168	17.6 %	0.2080303 ± 0.0006164	0.7275	EXP 150 of 150	1.0731240 ± 0.0190462	0.0754	EXP 150 of 150	0.0878861 ± 0.0173178	0.0048	EXP 150 of 150	2.2727351 ± 0.0150849	0.1493	EXP 150 of 150	54.887350 ± 0.020562	0.9960	EXP 150 of 150
18D00169	19.3 %	0.2116709 ± 0.0007705	0.5408	EXP 150 of 150	1.2910681 ± 0.0190512	0.1780	EXP 150 of 150	0.0998370 ± 0.0176855	0.0020	EXP 149 of 150	2.2391274 ± 0.0151206	0.1905	EXP 150 of 150	55.197083 ± 0.020665	0.9961	EXP 150 of 150
18D00171	21.0 %	0.1273419 ± 0.0005708	0.2173	EXP 150 of 150	0.5114508 ± 0.0174362	0.0158	EXP 150 of 150	0.0540405 ± 0.0171551	0.0004	EXP 150 of 150	0.9784545 ± 0.0156444	0.0156	EXP 150 of 150	34.803387 ± 0.019736	0.9975	EXP 150 of 150

Project Info		Analyst	Irradiation	X-pos	Y-pos	Z/H-pos	Project	Experiment	Nmb
18D00136	1.8 %	Dan Miggins	17-OSU-05	0.00	0.00	29.20	Arctic\O-Connor (16-22)	18D00132	01
18D00138	1.9 %	Dan Miggins	17-OSU-05	0.00	0.00	29.20	Arctic\O-Connor (16-22)	18D00132	01
18D00139	2.0 %	Dan Miggins	17-OSU-05	0.00	0.00	29.20	Arctic\O-Connor (16-22)	18D00132	01
18D00141	2.2 %	Dan Miggins	17-OSU-05	0.00	0.00	29.20	Arctic\O-Connor (16-22)	18D00132	01
18D00142	2.4 %	Dan Miggins	17-OSU-05	0.00	0.00	29.20	Arctic\O-Connor (16-22)	18D00132	01
18D00144	2.7 %	Dan Miggins	17-OSU-05	0.00	0.00	29.20	Arctic\O-Connor (16-22)	18D00132	01
18D00145	3.0 %	Dan Miggins	17-OSU-05	0.00	0.00	29.20	Arctic\O-Connor (16-22)	18D00132	01
18D00147	3.4 %	Dan Miggins	17-OSU-05	0.00	0.00	29.20	Arctic\O-Connor (16-22)	18D00132	01
18D00148	3.9 %	Dan Miggins	17-OSU-05	0.00	0.00	29.20	Arctic\O-Connor (16-22)	18D00132	01
18D00150	4.5 %	Dan Miggins	17-OSU-05	0.00	0.00	29.20	Arctic\O-Connor (16-22)	18D00132	01
18D00151	5.2 %	Dan Miggins	17-OSU-05	0.00	0.00	29.20	Arctic\O-Connor (16-22)	18D00132	01
18D00153	6.0 %	Dan Miggins	17-OSU-05	0.00	0.00	29.20	Arctic\O-Connor (16-22)	18D00132	01
18D00154	6.9 %	Dan Miggins	17-OSU-05	0.00	0.00	29.20	Arctic\O-Connor (16-22)	18D00132	01
18D00156	7.9 %	Dan Miggins	17-OSU-05	0.00	0.00	29.20	Arctic\O-Connor (16-22)	18D00132	01
18D00157	9.0 %	Dan Miggins	17-OSU-05	0.00	0.00	29.20	Arctic\O-Connor (16-22)	18D00132	01
18D00159	10.3 %	Dan Miggins	17-OSU-05	0.00	0.00	29.20	Arctic\O-Connor (16-22)	18D00132	01
18D00160	11.6 %	Dan Miggins	17-OSU-05	0.00	0.00	29.20	Arctic\O-Connor (16-22)	18D00132	01
18D00162	12.5 %	Dan Miggins	17-OSU-05	0.00	0.00	29.20	Arctic\O-Connor (16-22)	18D00132	01
18D00163	13.4 %	Dan Miggins	17-OSU-05	0.00	0.00	29.20	Arctic\O-Connor (16-22)	18D00132	01
18D00165	14.6 %	Dan Miggins	17-OSU-05	0.00	0.00	29.20	Arctic\O-Connor (16-22)	18D00132	01
18D00166	16.0 %	Dan Miggins	17-OSU-05	0.00	0.00	29.20	Arctic\O-Connor (16-22)	18D00132	01
18D00168	17.6 %	Dan Miggins	17-OSU-05	0.00	0.00	29.20	Arctic\O-Connor (16-22)	18D00132	01
18D00169	19.3 %	Dan Miggins	17-OSU-05	0.00	0.00	29.20	Arctic\O-Connor (16-22)	18D00132	01
18D00171	21.0 %	Dan Miggins	17-OSU-05	0.00	0.00	29.20	Arctic\O-Connor (16-22)	18D00132	01

**Sample Parameters**		Sample	Material	Location	Standard Name	Standard (in Ma)	%1σ	Standard Reference	Standard 40Ar/39Ar	%1σ	J	%1σ	Air 40Ar/36Ar	%1σ	MDF (lin)	%1σ	Volume Ratio	Sensitivity (mol/volt)	Day	Month	Year	Hour	Min	Resist
18D00136	1.8 %	HLY0102-D95-38	Groundmass	Gakkel Ridge	FCT-NM (5B20-17)	28.201	0.082	Kuiper et al (2008)	9.82234	0.081	0.00160017	0.081	302.927	0.131	0.9938627	0.067	1	4.8E-14	2	JAN	2018	15	22	1
18D00138	1.9 %	HLY0102-D95-38	Groundmass	Gakkel Ridge	FCT-NM (5B20-17)	28.201	0.082	Kuiper et al (2008)	9.82234	0.081	0.00160017	0.081	302.927	0.131	0.9938627	0.067	1	4.8E-14	2	JAN	2018	15	42	1
18D00139	2.0 %	HLY0102-D95-38	Groundmass	Gakkel Ridge	FCT-NM (5B20-17)	28.201	0.082	Kuiper et al (2008)	9.82234	0.081	0.00160017	0.081	302.927	0.131	0.9938627	0.067	1	4.8E-14	2	JAN	2018	15	52	1
18D00141	2.2 %	HLY0102-D95-38	Groundmass	Gakkel Ridge	FCT-NM (5B20-17)	28.201	0.082	Kuiper et al (2008)	9.82234	0.081	0.00160017	0.081	302.927	0.131	0.9938627	0.067	1	4.8E-14	2	JAN	2018	16	12	1
18D00142	2.4 %	HLY0102-D95-38	Groundmass	Gakkel Ridge	FCT-NM (5B20-17)	28.201	0.082	Kuiper et al (2008)	9.82234	0.081	0.00160017	0.081	302.927	0.131	0.9938627	0.067	1	4.8E-14	2	JAN	2018	16	22	1
18D00144	2.7 %	HLY0102-D95-38	Groundmass	Gakkel Ridge	FCT-NM (5B20-17)	28.201	0.082	Kuiper et al (2008)	9.82234	0.081	0.00160017	0.081	302.927	0.131	0.9938627	0.067	1	4.8E-14	2	JAN	2018	16	42	1
18D00145	3.0 %	HLY0102-D95-38	Groundmass	Gakkel Ridge	FCT-NM (5B20-17)	28.201	0.082	Kuiper et al (2008)	9.82234	0.081	0.00160017	0.081	302.927	0.131	0.9938627	0.067	1	4.8E-14	2	JAN	2018	16	52	1
18D00147	3.4 %	HLY0102-D95-38	Groundmass	Gakkel Ridge	FCT-NM (5B20-17)	28.201	0.082	Kuiper et al (2008)	9.82234	0.081	0.00160017	0.081	302.927	0.131	0.9938627	0.067	1	4.8E-14	2	JAN	2018	17	12	1
18D00148	3.9 %	HLY0102-D95-38	Groundmass	Gakkel Ridge	FCT-NM (5B20-17)	28.201	0.082	Kuiper et al (2008)	9.82234	0.081	0.00160017	0.081	302.927	0.131	0.9938627	0.067	1	4.8E-14	2	JAN	2018	17	22	1
18D00150	4.5 %	HLY0102-D95-38	Groundmass	Gakkel Ridge	FCT-NM (5B20-17)	28.201	0.082	Kuiper et al (2008)	9.82234	0.081	0.00160017	0.081	302.927	0.131	0.9938627	0.067	1	4.8E-14	2	JAN	2018	17	42	1
18D00151	5.2 %	HLY0102-D95-38	Groundmass	Gakkel Ridge	FCT-NM (5B20-17)	28.201	0.082	Kuiper et al (2008)	9.82234	0.081	0.00160017	0.081	302.927	0.131	0.9938627	0.067	1	4.8E-14	2	JAN	2018	17	53	1
18D00153	6.0 %	HLY0102-D95-38	Groundmass	Gakkel Ridge	FCT-NM (5B20-17)	28.201	0.082	Kuiper et al (2008)	9.82234	0.081	0.00160017	0.081	302.927	0.131	0.9938627	0.067	1	4.8E-14	2	JAN	2018	18	13	1
18D00154	6.9 %	HLY0102-D95-38	Groundmass	Gakkel Ridge	FCT-NM (5B20-17)	28.201	0.082	Kuiper et al (2008)	9.82234	0.081	0.00160017	0.081	302.927	0.131	0.9938627	0.067	1	4.8E-14	2	JAN	2018	18	23	1
18D00156	7.9 %	HLY0102-D95-38	Groundmass	Gakkel Ridge	FCT-NM (5B20-17)	28.201	0.082	Kuiper et al (2008)	9.82234	0.081	0.00160017	0.081	302.927	0.131	0.9938627	0.067	1	4.8E-14	2	JAN	2018	18	43	1
18D00157	9.0 %	HLY0102-D95-38	Groundmass	Gakkel Ridge	FCT-NM (5B20-17)	28.201	0.082	Kuiper et al (2008)	9.82234	0.081	0.00160017	0.081	302.927	0.131	0.9938627	0.067	1	4.8E-14	2	JAN	2018	18	53	1
18D00159	10.3 %	HLY0102-D95-38	Groundmass	Gakkel Ridge	FCT-NM (5B20-17)	28.201	0.082	Kuiper et al (2008)	9.82234	0.081	0.00160017	0.081	302.927	0.131	0.9938627	0.067	1	4.8E-14	2	JAN	2018	19	13	1
18D00160	11.6 %	HLY0102-D95-38	Groundmass	Gakkel Ridge	FCT-NM (5B20-17)	28.201	0.082	Kuiper et al (2008)	9.82234	0.081	0.00160017	0.081	302.927	0.131	0.9938627	0.067	1	4.8E-14	2	JAN	2018	19	23	1
18D00162	12.5 %	HLY0102-D95-38	Groundmass	Gakkel Ridge	FCT-NM (5B20-17)	28.201	0.082	Kuiper et al (2008)	9.82234	0.081	0.00160017	0.081	302.927	0.131	0.9938627	0.067	1	4.8E-14	2	JAN	2018	19	43	1
18D00163	13.4 %	HLY0102-D95-38	Groundmass	Gakkel Ridge	FCT-NM (5B20-17)	28.201	0.082	Kuiper et al (2008)	9.82234	0.081	0.00160017	0.081	302.927	0.131	0.9938627	0.067	1	4.8E-14	2	JAN	2018	19	53	1
18D00165	14.6 %	HLY0102-D95-38	Groundmass	Gakkel Ridge	FCT-NM (5B20-17)	28.201	0.082	Kuiper et al (2008)	9.82234	0.081	0.00160017	0.081	302.927	0.131	0.9938627	0.067	1	4.8E-14	2	JAN	2018	20	13	1
18D00166	16.0 %	HLY0102-D95-38	Groundmass	Gakkel Ridge	FCT-NM (5B20-17)	28.201	0.082	Kuiper et al (2008)	9.82234	0.081	0.00160017	0.081	302.927	0.131	0.9938627	0.067	1	4.8E-14	2	JAN	2018	20	23	1
18D00168	17.6 %	HLY0102-D95-38	Groundmass	Gakkel Ridge	FCT-NM (5B20-17)	28.201	0.082	Kuiper et al (2008)	9.82234	0.081	0.00160017	0.081	302.927	0.131	0.9938627	0.067	1	4.8E-14	2	JAN	2018	20	44	1
18D00169	19.3 %	HLY0102-D95-38	Groundmass	Gakkel Ridge	FCT-NM (5B20-17)	28.201	0.082	Kuiper et al (2008)	9.82234	0.081	0.00160017	0.081	302.927	0.131	0.9938627	0.067	1	4.8E-14	2	JAN	2018	20	54	1
18D00171	21.0 %	HLY0102-D95-38	Groundmass	Gakkel Ridge	FCT-NM (5B20-17)	28.201	0.082	Kuiper et al (2008)	9.82234	0.081	0.00160017	0.081	302.927	0.131	0.9938627	0.067	1	4.8E-14	2	JAN	2018	21	14	1

Irradiation Constants		40/36(a)	%1σ	40/36(c)	%1σ	38/36(a)	%1σ	38/36(c)	%1σ	39/37(ca)	%1σ	38/37(ca)	%1σ	36/37(ca)	%1σ	40/39(k)	%1σ	38/39(k)	%1σ	36/38(cl)	%1σ	K/Ca	%1σ	K/Cl	%1σ	Ca/Cl	%1σ
18D00136	1.8 %	297.99	0.382	0.018	35	0.1869	0	1.493	3	0.000643	0.92	0.00018	9.63	0.00027	0.17	0.000607	9.65	0.012077	0.09	0	0	0.43	0	0	0	0	0
18D00138	1.9 %	297.99	0.382	0.018	35	0.1869	0	1.493	3	0.000643	0.92	0.00018	9.63	0.00027	0.17	0.000607	9.65	0.012077	0.09	0	0	0.43	0	0	0	0	0
18D00139	2.0 %	297.99	0.382	0.018	35	0.1869	0	1.493	3	0.000643	0.92	0.00018	9.63	0.00027	0.17	0.000607	9.65	0.012077	0.09	0	0	0.43	0	0	0	0	0
18D00141	2.2 %	297.99	0.382	0.018	35	0.1869	0	1.493	3	0.000643	0.92	0.00018	9.63	0.00027	0.17	0.000607	9.65	0.012077	0.09	0	0	0.43	0	0	0	0	0
18D00142	2.4 %	297.99	0.382	0.018	35	0.1869	0	1.493	3	0.000643	0.92	0.00018	9.63	0.00027	0.17	0.000607	9.65	0.012077	0.09	0	0	0.43	0	0	0	0	0
18D00144	2.7 %	297.99	0.382	0.018	35	0.1869	0	1.493	3	0.000643	0.92	0.00018	9.63	0.00027	0.17	0.000607	9.65	0.012077	0.09	0	0	0.43	0	0	0	0	0
18D00145	3.0 %	297.99	0.382	0.018	35	0.1869	0	1.493	3	0.000643	0.92	0.00018	9.63	0.00027	0.17	0.000607	9.65	0.012077	0.09	0	0	0.43	0	0	0	0	0
18D00147	3.4 %	297.99	0.382	0.018	35	0.1869	0	1.493	3	0.000643	0.92	0.00018	9.63	0.00027	0.17	0.000607	9.65	0.012077	0.09	0	0	0.43	0	0	0	0	0
18D00148	3.9 %	297.99	0.382	0.018	35	0.1869	0	1.493	3	0.000643	0.92	0.00018	9.63	0.00027	0.17	0.000607	9.65	0.012077	0.09	0	0	0.43	0	0	0	0	0
18D00150	4.5 %	297.99	0.382	0.018	35	0.1869	0	1.493	3	0.000643	0.92	0.00018	9.63	0.00027	0.17	0.000607	9.65	0.012077	0.09	0	0	0.43	0	0	0	0	0
18D00151	5.2 %	297.99	0.382	0.018	35	0.1869	0	1.493	3	0.000643	0.92	0.00018	9.63	0.00027	0.17	0.000607	9.65	0.012077	0.09	0	0	0.43	0	0	0	0	0
18D00153	6.0 %	297.99	0.382	0.018	35	0.1869	0	1.493	3	0.000643	0.92	0.00018	9.63	0.00027	0.17	0.000607	9.65	0.012077	0.09	0	0	0.43	0	0	0	0	0
18D00154	6.9 %	297.99	0.382	0.018	35	0.1869	0	1.493	3	0.000643	0.92	0.00018	9.63	0.00027	0.17	0.000607	9.65	0.012077	0.09	0	0	0.43	0	0	0	0	0
18D00156	7.9 %	297.99	0.382	0.018	35	0.1869	0	1.493	3	0.000643	0.92	0.00018	9.63	0.00027	0.17	0.000607	9.65	0.012077	0.09	0	0	0.43	0	0	0	0	0
18D00157	9.0 %	297.99	0.382	0.018	35	0.1869	0	1.493	3	0.000643	0.92	0.00018	9.63	0.00027	0.17	0.000607	9.65	0.012077	0.09	0	0	0.43	0	0	0	0	0
18D00159	10.3 %	297.99	0.382	0.018	35	0.1869	0	1.493	3	0.000643	0.92	0.00018	9.63	0.00027	0.17	0.000607	9.65	0.012077	0.09	0	0	0.43	0	0	0	0	0
18D00160	11.6 %	297.99	0.382	0.018	35	0.1869	0	1.493	3	0.000643	0.92	0.00018	9.63	0.00027	0.17	0.000607	9.65	0.012077	0.09	0	0	0.43	0	0	0	0	0
18D00162	12.5 %	297.99	0.382	0.018	35	0.1869	0	1.493	3	0.000643	0.92	0.00018	9.63	0.00027	0.17	0.000607	9.65	0.012077	0.09	0	0	0.43	0	0	0	0	0
18D00163	13.4 %	297.99	0.382	0.018	35	0.1869	0	1.493	3	0.000643	0.92	0.00018	9.63	0.00027	0.17	0.000607	9.65	0.012077	0.09	0	0	0.43	0	0	0	0	0
18D00165	14.6 %	297.99	0.382	0.018	35	0.1869	0	1.493	3	0.000643	0.92	0.00018	9.63	0.00027	0.17	0.000607	9.65	0.012077	0.09	0	0	0.43	0	0	0	0	0
18D00166	16.0 %	297.99	0.382	0.018	35	0.1869	0	1.493	3	0.000643	0.92	0.00018	9.63	0.00027	0.17	0.000607	9.65	0.012077	0.09	0	0	0.43	0	0	0	0	0
18D00168	17.6 %	297.99	0.382	0.018	35	0.1869	0	1.493	3	0.000643	0.92	0.00018	9.63	0.00027	0.17	0.000607	9.65	0.012077	0.09	0	0	0.43	0	0	0	0	0
18D00169	19.3 %	297.99	0.382	0.018	35	0.1869	0	1.493	3	0.000643	0.92	0.00018	9.63	0.00027	0.17	0.000607	9.65	0.012077	0.09	0	0	0.43	0	0	0	0	0
18D00171	21.0 %	297.99	0.382	0.018	35	0.1869	0	1.493	3	0.000643	0.92	0.00018	9.63	0.00027	0.17	0.000607	9.65	0.012077	0.09	0	0	0.43	0	0	0	0	0

18D00132.AGE >>> HLY0102-D95-38 >>> ARCTIC | O-CONNOR (16-22) PROJECT

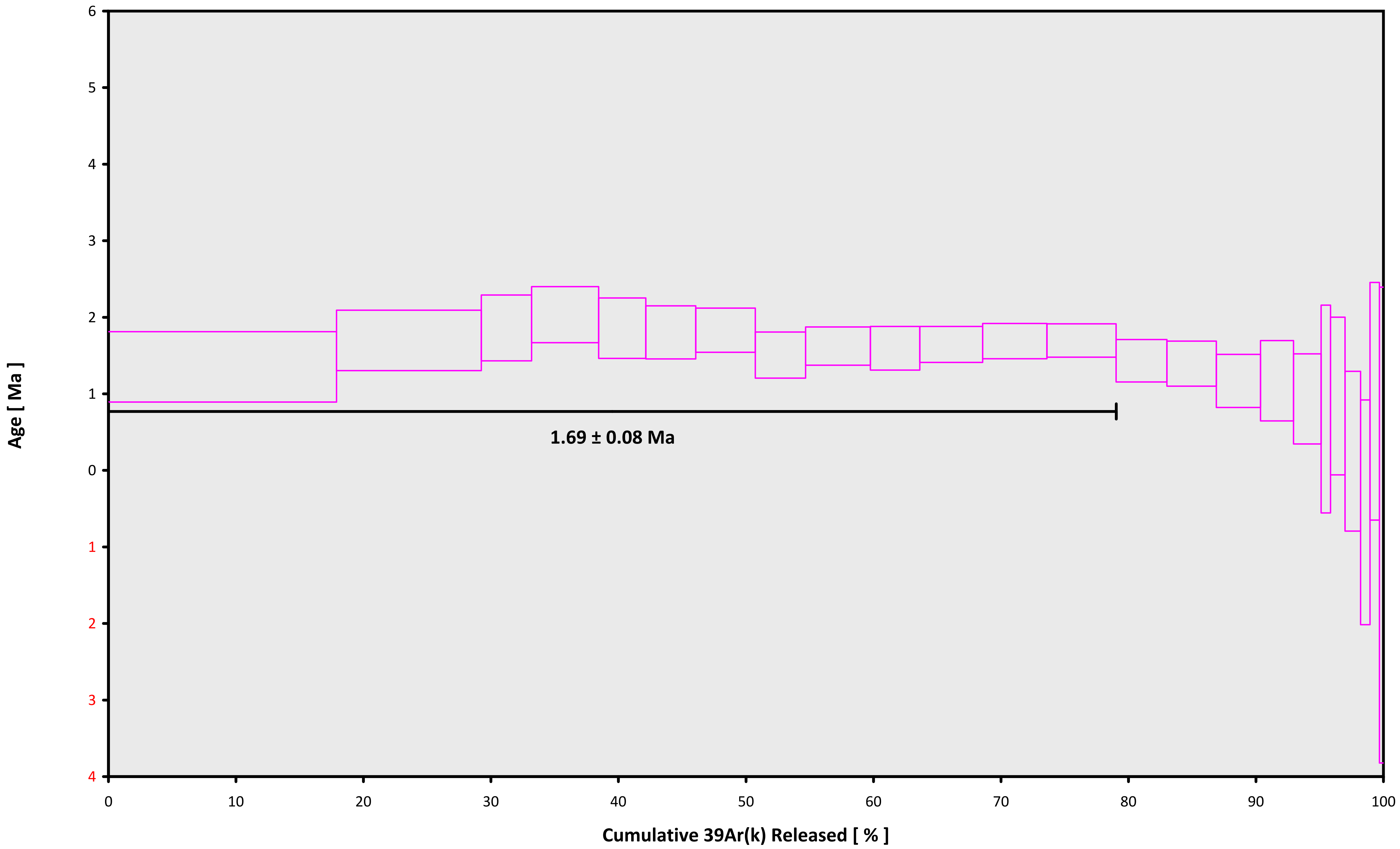

Ar-Ages in Ma

WEIGHTED PLATEAU
1.69 ± 0.08
TOTAL FUSION
1.52 ± 0.11
NORMAL ISOCHRON
1.69 ± 0.18
INVERSE ISOCHRON
1.69 ± 0.18

MSWD (PROBABILITY)
0.90 (55%)

Sample Info

Groundmass
Gakkel Ridge
Dan Miggins

IRR = 17-OSU-05 (5B20-17)
J = 0.00160017 ± 0.00000130

18D00132.AGE >>> HLY0102-D95-38 >>> ARCTIC | O-CONNOR (16-22) PROJECT

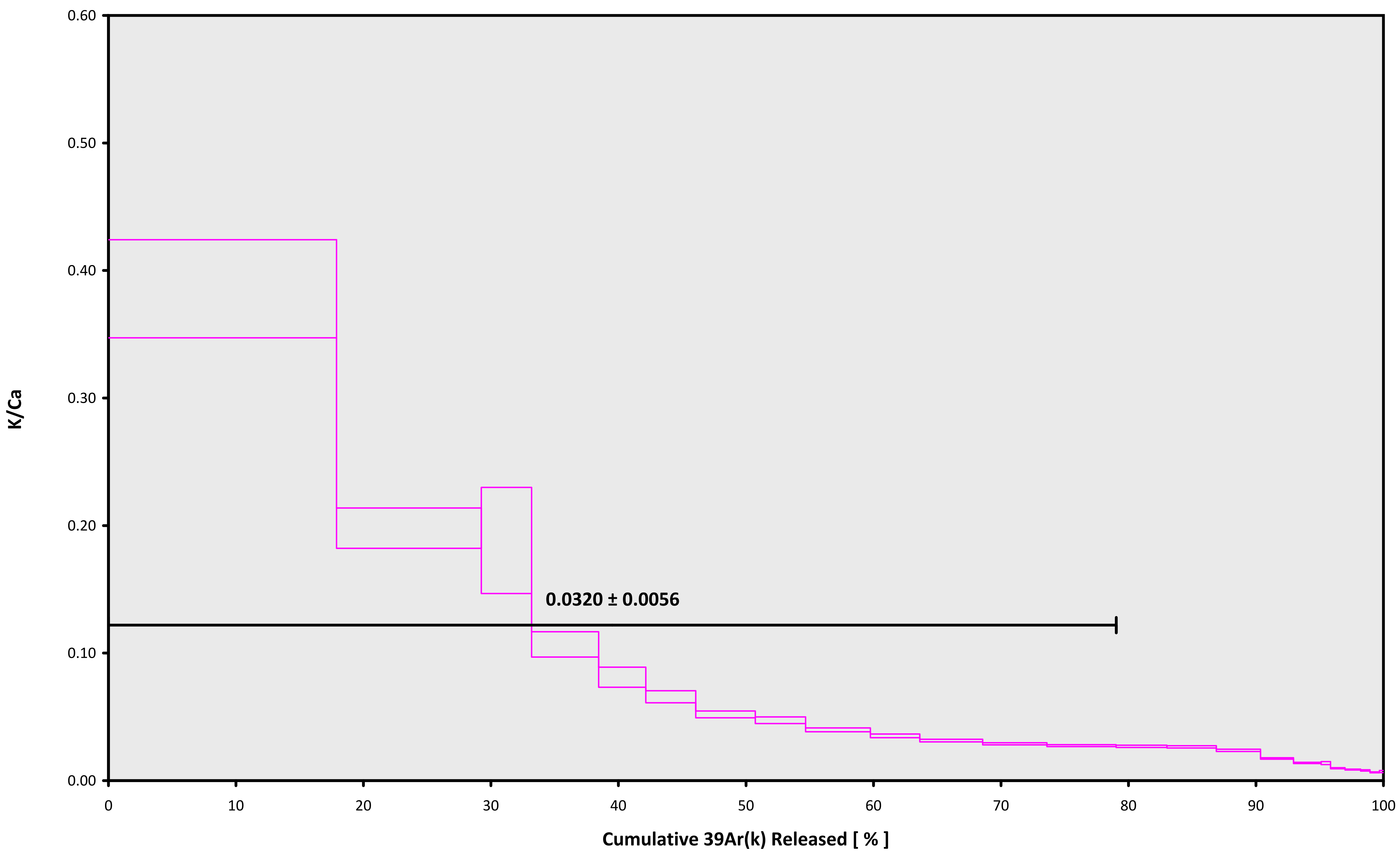

Ar-Ages in Ma

WEIGHTED PLATEAU
1.69 ± 0.08
TOTAL FUSION
1.52 ± 0.11
NORMAL ISOCHRON
1.69 ± 0.18
INVERSE ISOCHRON
1.69 ± 0.18

Sample Info

Groundmass
Gakkel Ridge
Dan Miggins

IRR = 17-OSU-05 (5B20-17)
J = 0.00160017 ± 0.00000130

18D00132.AGE >>> HLY0102-D95-38 >>> ARCTIC | O-CONNOR (16-22) PROJECT

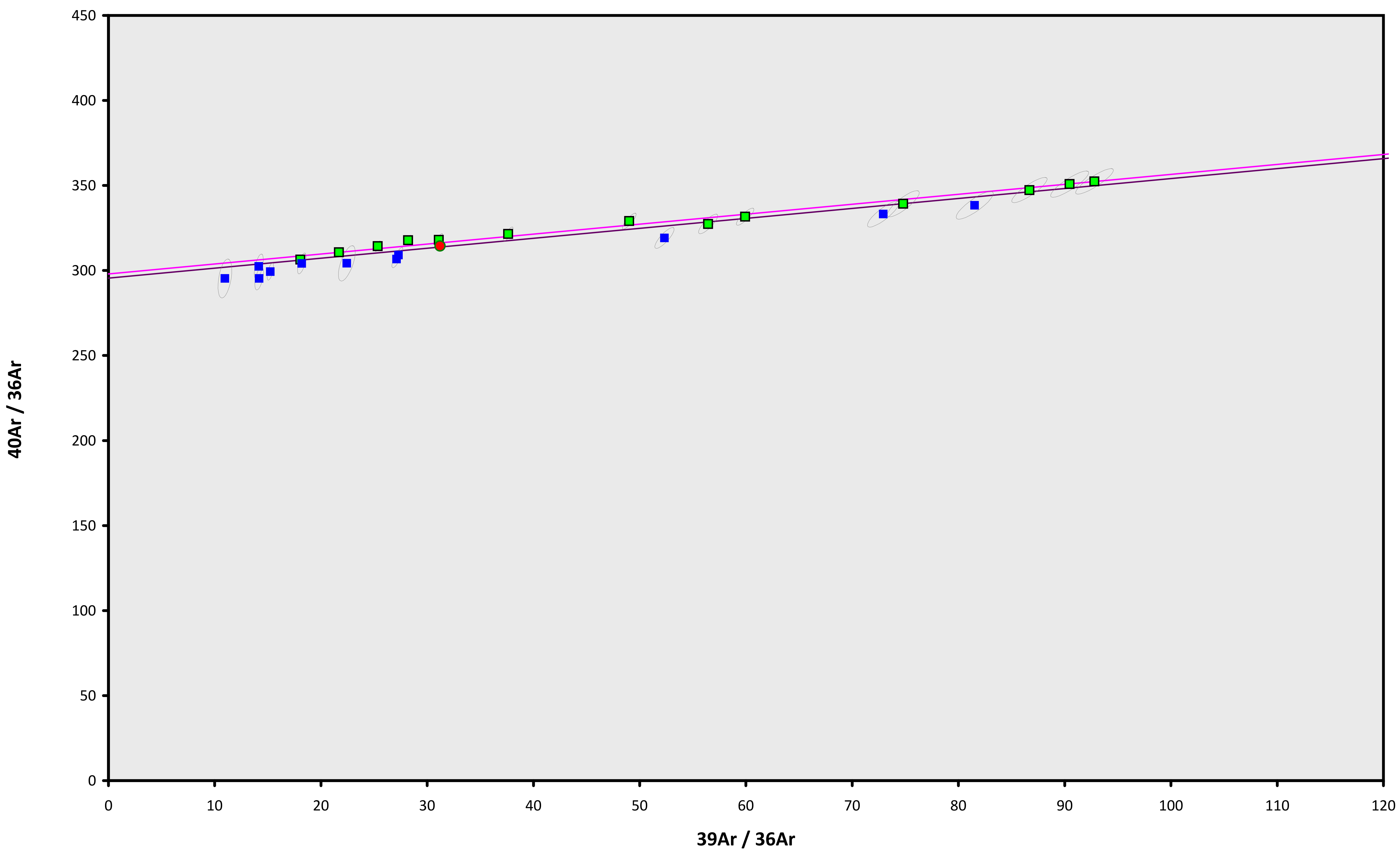

Ar-Ages in Ma

WEIGHTED PLATEAU
1.69 ± 0.08
TOTAL FUSION
1.52 ± 0.11
NORMAL ISOCHRON
1.69 ± 0.18
INVERSE ISOCHRON
1.69 ± 0.18

MSWD (PROBABILITY)
1.66 (8%)

40AR/36AR INTERCEPT
298.0 ± 2.3

Sample Info

Groundmass
Gakkel Ridge
Dan Miggins

IRR = 17-OSU-05 (5B20-17)
J = 0.00160017 ± 0.00000130

18D00132.AGE >>> HLY0102-D95-38 >>> ARCTIC | O-CONNOR (16-22) PROJECT

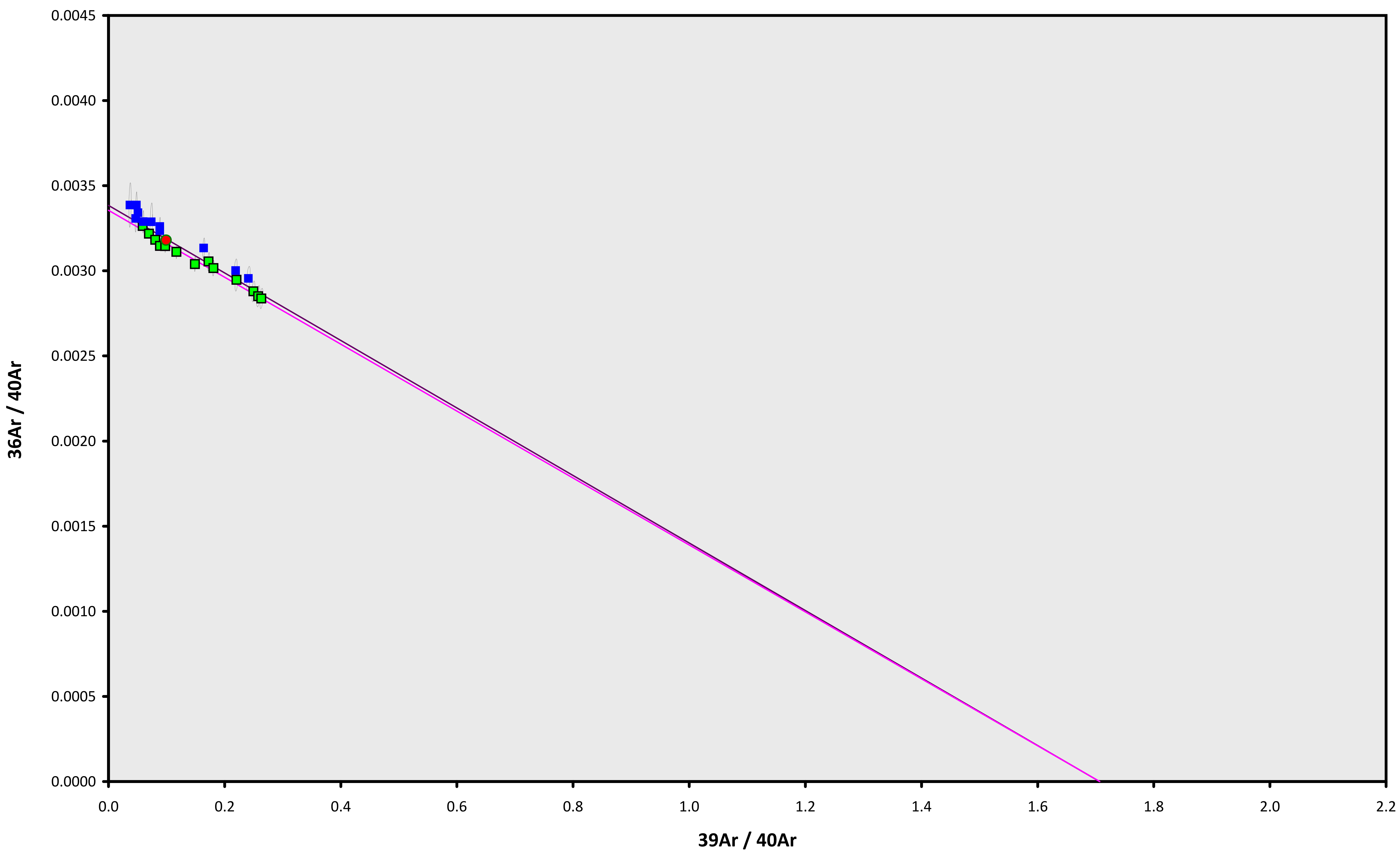

Ar-Ages in Ma

WEIGHTED PLATEAU
1.69 ± 0.08
TOTAL FUSION
1.52 ± 0.11
NORMAL ISOCHRON
1.69 ± 0.18
INVERSE ISOCHRON
1.69 ± 0.18

MSWD (PROBABILITY)
1.66 (7%)

SPREADING FACTOR
12.0%

40AR/36AR INTERCEPT
298.0 ± 2.3

Sample Info

Groundmass
Gakkel Ridge
Dan Miggins

IRR = 17-OSU-05 (5B20-17)
J = 0.00160017 ± 0.00000130

**Relative Abundances**			36Ar [fA]	%1σ	37Ar [fA]	%1σ	38Ar [fA]	%1σ	39Ar [fA]	%1σ	40Ar [fA]	%1σ	40(r)/39(k) ± 2σ	Age ± 2σ (ka)	40Ar(r) (%)	39Ar(k) (%)	K/Ca ± 2σ
18D00508	1.8 %		2.031309	0.303	44.2186	6.328	0.5048255	4.801	9.29493	0.317	605.4236	0.014	0.29467 ± 0.49662	866.3 ± 1460.3	0.45	6.45	0.0901 ± 0.0114
18D00510	1.9 %		1.825559	0.306	64.2290	4.407	0.4707106	5.131	8.96393	0.332	543.2042	0.016	0.15204 ± 0.46622	446.9 ± 1370.6	0.25	6.21	0.0597 ± 0.0053
18D00511	2.0 %	✓	0.620259	0.342	31.1824	9.184	0.1746020	14.410	3.99648	0.725	185.7880	0.065	0.38412 ± 0.40307	1128.6 ± 1183.9	0.82	2.77	0.0548 ± 0.0101
18D00513	2.2 %	✓	0.588370	0.361	30.9388	9.405	0.1588871	16.664	3.82911	0.762	174.6542	0.056	0.00609 ± 0.41450	17.9 ± 1218.3	0.01	2.65	0.0529 ± 0.0100
18D00514	2.4 %	✓	1.036367	0.316	84.1681	3.440	0.2916730	7.934	6.78335	0.430	306.3669	0.034	0.16387 ± 0.36317	481.6 ± 1067.1	0.36	4.68	0.0344 ± 0.0024
18D00516	2.7 %	✓	0.480144	0.379	50.2903	5.873	0.1635438	14.696	3.98954	0.734	141.8800	0.058	0.34709 ± 0.34316	1019.9 ± 1008.0	0.97	2.75	0.0338 ± 0.0040
18D00517	3.0 %	✓	1.020517	0.327	140.2983	2.238	0.3191923	7.490	8.86103	0.335	298.7179	0.034	0.31868 ± 0.28031	936.4 ± 823.4	0.94	6.10	0.0269 ± 0.0012
18D00519	3.4 %	✓	0.684130	0.351	147.4761	2.055	0.2476704	9.772	8.45171	0.344	196.1200	0.060	0.24977 ± 0.21143	734.0 ± 621.2	1.06	5.81	0.0244 ± 0.0010
18D00520	3.9 %	✓	0.470413	0.384	149.1058	2.127	0.1821023	14.036	7.53045	0.384	132.1021	0.062	0.34510 ± 0.18061	1014.0 ± 530.5	1.94	5.17	0.0214 ± 0.0009
18D00522	4.5 %	✓	0.362613	0.399	154.1312	2.075	0.1372841	17.274	7.35815	0.421	98.6936	0.066	0.27987 ± 0.15273	822.4 ± 448.7	2.06	5.05	0.0203 ± 0.0009
18D00523	5.2 %	✓	0.405456	0.375	211.5136	1.570	0.2055123	12.192	9.31562	0.316	106.9542	0.067	0.22434 ± 0.12737	659.2 ± 374.2	1.93	6.39	0.0187 ± 0.0006
18D00525	6.0 %	✓	0.362277	0.399	253.6913	1.297	0.1771221	13.550	10.53993	0.288	90.8341	0.068	0.22894 ± 0.10636	672.8 ± 312.5	2.62	7.22	0.0176 ± 0.0005
18D00526	6.9 %	✓	0.297090	0.421	267.9819	1.232	0.2176193	10.996	10.50112	0.287	69.3961	0.080	0.16779 ± 0.09496	493.1 ± 279.0	2.50	7.18	0.0166 ± 0.0004
18D00528	7.9 %	✓	0.272367	0.418	277.5200	1.228	0.1633545	14.770	10.52827	0.274	61.0788	0.085	0.15823 ± 0.08975	465.0 ± 263.7	2.68	7.20	0.0160 ± 0.0004
18D00529	9.0 %	✓	0.246917	0.453	281.4088	1.247	0.1653104	14.943	10.16216	0.290	52.9268	0.097	0.14697 ± 0.09223	431.9 ± 271.0	2.77	6.94	0.0153 ± 0.0004
18D00531	10.3 %		0.257969	0.446	294.5796	1.223	0.1612137	15.038	8.86960	0.332	54.2199	0.094	0.05812 ± 0.10908	170.8 ± 320.6	0.93	6.04	0.0127 ± 0.0003
18D00532	11.6 %		0.128095	0.592	133.0253	2.222	0.0623586	38.526	4.07644	0.716	27.8203	0.179	0.01739 ± 0.17115	51.1 ± 503.0	0.25	2.78	0.0129 ± 0.0006
18D00534	12.5 %		0.129230	0.598	130.0697	2.450	0.0462335	49.939	3.36758	0.833	27.9929	0.177	0.10346 ± 0.21802	304.1 ± 640.9	1.21	2.28	0.0109 ± 0.0006
18D00535	13.4 %		0.091857	0.748	82.9800	3.558	0.0232347	99.317	2.26459	1.273	20.3309	0.244	0.26190 ± 0.29348	769.9 ± 862.9	2.85	1.54	0.0115 ± 0.0009
18D00537	14.6 %		0.089247	0.739	79.1786	3.653	0.0352901	67.017	1.64485	1.757	19.9805	0.249	0.28406 ± 0.39594	835.1 ± 1164.2	2.27	1.11	0.0087 ± 0.0007
18D00538	16.0 %		0.141822	0.568	145.7681	2.189	0.0591567	40.177	1.96520	1.463	30.2716	0.168	0.30702 ± 0.39125	902.6 ± 1150.5	1.90	1.30	0.0055 ± 0.0003
18D00540	17.6 %		0.065597	0.924	63.4084	4.610	0.0411322	58.364	0.96618	2.941	13.9527	0.356	0.69204 ± 0.66233	2035.1 ± 1948.9	4.59	0.64	0.0063 ± 0.0007
18D00541	19.3 %		0.053766	1.036	55.8322	5.478	0.0220712	104.926	0.61328	4.674	11.0188	0.451	1.08873 ± 1.06349	3202.7 ± 3131.3	5.71	0.40	0.0044 ± 0.0007
18D00543	21.0 %		0.243764	0.454	293.9520	1.189	0.0555081	41.141	2.11734	1.368	49.3370	0.104	0.07639 ± 0.47324	224.5 ± 1391.1	0.30	1.34	0.0028 ± 0.0001
		Σ	11.905136	0.095	3466.9481	0.442	4.0856087	2.891	145.99086	0.098	3319.0652	0.011					

Information on Analysis and Constants Used in Calculations

Project = **O-CONNOR (16-22)**
Sample = **PS59-309-39**
Material = **Groundmass**
Location = **Gakkel Ridge**
Region = **Artic Ocean**
Analyst = **Dan Miggins**
Irradiation = **17-OSU-05 (5B12-17)**
Position = **X: 0 | Y: 0 | Z/H: 18.17182 mm**
FCT-NM Age = **28.201 ± 0.023 Ma**
FCT-NM Reference = **Kuiper et al (2008)**
FCT-NM 40Ar/39Ar Ratio = **9.67023 ± 0.00793**
FCT-NM J-value = **0.00162534 ± 0.00000133**
Air Shot 40Ar/36Ar = **302.3400 ± 0.4686**
Air Shot MDF = **0.99433681 ± 0.00069284 (LIN)**
Experiment Type = **Incremental Heating**
Extraction Method = **Bulk Laser Heating**
Heating = **77 sec**
Isolation = **3.00 min**
Instrument = **ARGUS-VI-D**
Preferred Age = **Plateau Age**
Age Classification = **Crystallization Age**
IGSN = Undefined
Rock Class = Undefined
Lithology = **Basaltic Lava**
Lat-Lon = Undefined - Undefined

Age Equations = **Min et al. (2000)**
Negative Intensities = **Allowed**
Collector Calibrations = **36Ar**
Decay 40K = **5.530 ± 0.048 E-10 1/a**
Decay 39Ar = **2.940 ± 0.016 E-07 1/h**
Decay 37Ar = **8.230 ± 0.012 E-04 1/h**
Decay 36Cl = **2.257 ± 0.015 E-06 1/a**
Decay 40K(EC,β^+) = **0.580 ± 0.009 E-10 1/a**
Decay 40K(β^-) = **4.950 ± 0.043 E-10 1/a**
Atmospheric 40/36(a) = **301.16 ± 0.66**
Atmospheric 38/36(a) = **0.1869**
Production 39/37(ca) = **0.0006425 ± 0.0000059**
Production 38/37(ca) = **0.0001800 ± 0.0000173**
Production 36/37(ca) = **0.0002703 ± 0.0000005**
Production 40/39(k) = **0.000607 ± 0.000059**
Production 38/39(k) = **0.012077 ± 0.000011**
Production 36/38(cl) = **262.80 ± 1.71**
Scaling Ratio K/Ca = **0.430**
Abundance Ratio 40K/K = **1.1700 ± 0.0100 E-04**
Atomic Weight K = **39.0983 ± 0.0001 g**

**Results**	40(a)/36(a) ± 2σ	40(r)/39(k) ± 2σ	Age ± 2σ (ka)	MSWD	39Ar(k) (%,n)	K/Ca ± 2σ
**Age Plateau**		0.20074 ± 0.03955 ± 19.70%	**589.9** ± 116.2 ± 19.70%	0.86 59%	69.91 13	0.0175 ± 0.0018
			Full External Error ± 117.0	1.82	2σ Confidence Limit	
			Analytical Error ± 116.2	1.0000	Error Magnification	
**Total Fusion Age**		0.11030 ± 0.05969 ± 54.11%	**324.2** ± 175.4 ± 54.11%		24	0.0178 ± 0.0002
			Full External Error ± 175.5			
			Analytical Error ± 175.4			
**Normal Isochron**	**302.14** ± 1.18 ± 0.39%	0.16659 ± 0.05728 ± 34.38%	**489.6** ± 168.3 ± 34.38%	0.88 56%	69.91 13	
			Full External Error ± 168.7	1.85	2σ Confidence Limit	
			Analytical Error ± 168.3	1.0000	Error Magnification	
				4	Number of Iterations	
				0.0000000227	Convergence	
**Inverse Isochron** Clustered Points	**302.15** ± 1.18 ± 0.39%	0.16683 ± 0.05059 ± 30.32%	**490.3** ± 148.6 ± 30.32%	0.87 57%	69.91 13	
			Full External Error ± 149.1	1.85	2σ Confidence Limit	
			Analytical Error ± 148.6	1.0000	Error Magnification	
				2	Number of Iterations	
				0.0089907160	Convergence	
				3%	Spreading Factor	

Notes
Excess Initial 40Ar/36Ar = 301.16 ± 0.22 (%SD). Mostly atmospheric

Incremental Heating			36Ar(a) [fA]	37Ar(ca) [fA]	38Ar(cl) [fA]	39Ar(k) [fA]	40Ar(r) [fA]	Age ± 2σ (ka)	40Ar(r) (%)	39Ar(k) (%)	K/Ca ± 2σ
18D00508	1.8 %		2.019354	44.2186	0.0075373	9.26651	2.730610	866.3 ± 1460.3	0.45	6.45	0.0901 ± 0.0114
18D00510	1.9 %		1.808193	64.2290	0.0134391	8.92267	1.356571	446.9 ± 1370.6	0.25	6.21	0.0597 ± 0.0053
18D00511	2.0 %	✓	0.611828	31.1824	0.0066150	3.97644	1.527426	1128.6 ± 1183.9	0.82	2.77	0.0548 ± 0.0101
18D00513	2.2 %	✓	0.580008	30.9388	0.0000000	3.80923	0.023214	17.9 ± 1218.3	0.01	2.65	0.0529 ± 0.0100
18D00514	2.4 %	✓	1.013614	84.1681	0.0058088	6.72927	1.102708	481.6 ± 1067.1	0.36	4.68	0.0344 ± 0.0024
18D00516	2.7 %	✓	0.466543	50.2903	0.0195032	3.95723	1.373531	1019.9 ± 1008.0	0.97	2.75	0.0338 ± 0.0040
18D00517	3.0 %	✓	0.982592	140.2983	0.0043660	8.77089	2.795078	936.4 ± 823.4	0.94	6.10	0.0269 ± 0.0012
18D00519	3.4 %	✓	0.644267	147.4761	0.0000000	8.35695	2.087327	734.0 ± 621.2	1.06	5.81	0.0244 ± 0.0010
18D00520	3.9 %	✓	0.430110	149.1058	0.0000000	7.43465	2.565726	1014.0 ± 530.5	1.94	5.17	0.0214 ± 0.0009
18D00522	4.5 %	✓	0.320951	154.1312	0.0000000	7.25912	2.031584	822.4 ± 448.7	2.06	5.05	0.0203 ± 0.0009
18D00523	5.2 %	✓	0.348284	211.5136	0.0000000	9.17972	2.059380	659.2 ± 374.2	1.93	6.39	0.0187 ± 0.0006
18D00525	6.0 %	✓	0.293704	253.6913	0.0000000	10.37693	2.375732	672.8 ± 312.5	2.62	7.22	0.0176 ± 0.0005
18D00526	6.9 %	✓	0.224654	267.9819	0.0026521	10.32895	1.733140	493.1 ± 279.0	2.50	7.18	0.0166 ± 0.0004
18D00528	7.9 %	✓	0.197353	277.5200	0.0000000	10.34997	1.637648	465.0 ± 263.7	2.68	7.20	0.0160 ± 0.0004
18D00529	9.0 %	✓	0.170852	281.4088	0.0000000	9.98135	1.466967	431.9 ± 271.0	2.77	6.94	0.0153 ± 0.0004
18D00531	10.3 %		0.178344	294.5796	0.0000000	8.68033	0.504475	170.8 ± 320.6	0.93	6.04	0.0127 ± 0.0003
18D00532	11.6 %		0.092139	133.0253	0.0000000	3.99097	0.069394	51.1 ± 503.0	0.25	2.78	0.0129 ± 0.0006
18D00534	12.5 %		0.094072	130.0697	0.0000000	3.28401	0.339777	304.1 ± 640.9	1.21	2.28	0.0109 ± 0.0006
18D00535	13.4 %		0.069427	82.9800	0.0000000	2.21127	0.579140	769.9 ± 862.9	2.85	1.54	0.0115 ± 0.0009
18D00537	14.6 %		0.067845	79.1786	0.0000000	1.59397	0.452777	835.1 ± 1164.2	2.27	1.11	0.0087 ± 0.0007
18D00538	16.0 %		0.102421	145.7681	0.0000000	1.87154	0.574605	902.6 ± 1150.5	1.90	1.30	0.0055 ± 0.0003
18D00540	17.6 %		0.048454	63.4084	0.0094860	0.92544	0.640440	2035.1 ± 1948.9	4.59	0.64	0.0063 ± 0.0007
18D00541	19.3 %		0.038674	55.8322	0.0000000	0.57741	0.628644	3202.7 ± 3131.3	5.71	0.40	0.0044 ± 0.0007
18D00543	21.0 %		0.164308	293.9520	0.0000000	1.92848	0.147319	224.5 ± 1391.1	0.30	1.34	0.0028 ± 0.0001
		Σ	10.967993	3466.9481	0.0694075	143.76334	15.857015				

Information on Analysis

Project = **O-CONNOR (16-22)**
Sample = **PS59-309-39**
Material = **Groundmass**
Location = **Gakkel Ridge**
Region = **Artic Ocean**
Analyst = **Dan Miggins**
Irradiation = **17-OSU-05 (5B12-17)**
J = **0.00162534 ± 0.00000133**
FCT-NM = **28.201 ± 0.023 Ma**

Results	40(r)/39(k) ± 2σ		Age ± 2σ (ka)		MSWD	39Ar(k) (%,n)	K/Ca ± 2σ
Age Plateau	0.20074	± 0.03955<br>± 19.70%	**589.9**	**± 116.2**<br>**± 19.70%**	0.86<br>59%	69.91<br>13	0.0175 ± 0.0018
			Full External Error	± 117.0	1.82	2σ Confidence Limit	
			Analytical Error	± 116.2	1.0000	Error Magnification	
Total Fusion Age	0.11030	± 0.05969<br>± 54.11%	**324.2**	**± 175.4**<br>**± 54.11%**		24	0.0178 ± 0.0002
			Full External Error	± 175.5			
			Analytical Error	± 175.4			

Normal Isochron			39(k)/36(a) ± 2σ	40(a+r)/36(a) ± 2σ	r.i.
18D00508	1.8 %		4.59 ± 0.04	299.81 ± 1.85	0.6939
18D00510	1.9 %		4.93 ± 0.05	300.41 ± 1.88	0.6814
18D00511	2.0 %	✓	6.50 ± 0.11	303.66 ± 2.28	0.4446
18D00513	2.2 %	✓	6.57 ± 0.11	301.12 ± 2.38	0.4491
18D00514	2.4 %	✓	6.64 ± 0.07	302.25 ± 2.02	0.6050
18D00516	2.7 %	✓	8.48 ± 0.15	304.10 ± 2.62	0.4937
18D00517	3.0 %	✓	8.93 ± 0.09	304.00 ± 2.14	0.7149
18D00519	3.4 %	✓	12.97 ± 0.14	304.40 ± 2.43	0.7407
18D00520	3.9 %	✓	17.29 ± 0.21	307.13 ± 2.88	0.7596
18D00522	4.5 %	✓	22.62 ± 0.31	307.49 ± 3.26	0.7693
18D00523	5.2 %	✓	26.36 ± 0.32	307.07 ± 3.14	0.8375
18D00525	6.0 %	✓	35.33 ± 0.46	309.25 ± 3.61	0.8859
18D00526	6.9 %	✓	45.98 ± 0.69	308.87 ± 4.26	0.9132
18D00528	7.9 %	✓	52.44 ± 0.83	309.46 ± 4.64	0.9298
18D00529	9.0 %	✓	58.42 ± 1.06	309.75 ± 5.37	0.9395
18D00531	10.3 %		48.67 ± 0.89	303.99 ± 5.19	0.9222
18D00532	11.6 %		43.31 ± 1.22	301.91 ± 7.31	0.8433
18D00534	12.5 %		34.91 ± 1.05	297.55 ± 7.41	0.8127
18D00535	13.4 %		31.85 ± 1.28	292.82 ± 9.00	0.7482
18D00537	14.6 %		23.49 ± 1.11	294.49 ± 9.01	0.6303
18D00538	16.0 %		18.27 ± 0.70	295.55 ± 6.90	0.5935
18D00540	17.6 %		19.10 ± 1.41	287.94 ± 12.02	0.5475
18D00541	19.3 %		14.93 ± 1.67	284.91 ± 14.91	0.4531
18D00543	21.0 %		11.74 ± 0.41	300.26 ± 5.38	0.5043

Results	40(a)/36(a) ± 2σ		40(r)/39(k) ± 2σ		Age ± 2σ (ka)		MSWD
Normal Isochron	**302.14**	**± 1.18**<br>**± 0.39%**	0.16659	± 0.05728<br>± 34.38%	**489.6**	**± 168.3**<br>**± 34.38%**	0.88<br>56%
					Full External Error	± 168.7	
					Analytical Error	± 168.3	

Statistics					
Statistics	2σ Confidence Limit	1.85	Convergence	0.000000022690	
	Error Magnification	1.0000	Number of Iterations	4	
	Number of Data Points	13	Calculated Line	Weighted York-2	

Inverse Isochron			39(k)/40(a+r) ± 2σ	36(a)/40(a+r) ± 2σ	r.i.
18D00508	1.8 %		0.0153060 ± 0.0000976	0.00333547 ± 0.00002053	0.0019
18D00510	1.9 %		0.0164262 ± 0.0001099	0.00332879 ± 0.00002079	0.0024
18D00511	2.0 %	✓	0.0214034 ± 0.0003138	0.00329319 ± 0.00002470	0.0153
18D00513	2.2 %	✓	0.0218104 ± 0.0003358	0.00332094 ± 0.00002622	0.0104
18D00514	2.4 %	✓	0.0219650 ± 0.0001913	0.00330854 ± 0.00002213	0.0080
18D00516	2.7 %	✓	0.0278918 ± 0.0004148	0.00328835 ± 0.00002829	0.0107
18D00517	3.0 %	✓	0.0293623 ± 0.0002005	0.00328942 ± 0.00002318	0.0098
18D00519	3.4 %	✓	0.0426125 ± 0.0003013	0.00328515 ± 0.00002618	0.0253
18D00520	3.9 %	✓	0.0562815 ± 0.0004443	0.00325600 ± 0.00003055	0.0209
18D00522	4.5 %	✓	0.0735554 ± 0.0006368	0.00325214 ± 0.00003443	0.0189
18D00523	5.2 %	✓	0.0858330 ± 0.0005637	0.00325656 ± 0.00003334	0.0268
18D00525	6.0 %	✓	0.1142485 ± 0.0006890	0.00323364 ± 0.00003774	0.0263
18D00526	6.9 %	✓	0.1488538 ± 0.0009055	0.00323756 ± 0.00004470	0.0306
18D00528	7.9 %	✓	0.1694701 ± 0.0009934	0.00323146 ± 0.00004844	0.0330
18D00529	9.0 %	✓	0.1886093 ± 0.0011783	0.00322845 ± 0.00005597	0.0346
18D00531	10.3 %		0.1601104 ± 0.0011326	0.00328960 ± 0.00005615	0.0293
18D00532	11.6 %		0.1434680 ± 0.0021668	0.00331221 ± 0.00008022	0.0352
18D00534	12.5 %		0.1173242 ± 0.0020521	0.00336080 ± 0.00008366	0.0287
18D00535	13.4 %		0.1087713 ± 0.0028914	0.00341509 ± 0.00010497	0.0293
18D00537	14.6 %		0.0797803 ± 0.0029265	0.00339574 ± 0.00010384	0.0221
18D00538	16.0 %		0.0618272 ± 0.0019161	0.00338352 ± 0.00007894	0.0156
18D00540	17.6 %		0.0663301 ± 0.0041094	0.00347291 ± 0.00014495	0.0196
18D00541	19.3 %		0.0524041 ± 0.0052371	0.00350994 ± 0.00018373	0.0155
18D00543	21.0 %		0.0390888 ± 0.0011827	0.00333041 ± 0.00005965	0.0080

Results	40(a)/36(a) ± 2σ	40(r)/39(k) ± 2σ	Age ± 2σ (ka)	MSWD
**Inverse Isochron** Clustered Points	**302.15 ± 1.18 ± 0.39%**	0.16683 ± 0.05059 ± 30.32%	**490.3 ± 148.6 ± 30.32%** Full External Error ± 149.1 Analytical Error ± 148.6	0.87 57%

Statistics				
	2σ Confidence Limit	1.85	Convergence	0.0089907160
	Error Magnification	1.0000	Number of Iterations	2
	Number of Data Points	13	Calculated Line	Weighted York-2
	Spreading Factor	2.8%		

Degassing Patterns			36Ar(a) [fA]	%1σ	36Ar(c) [fA]	%1σ	36Ar(ca) [fA]	%1σ	36Ar(cl) [fA]	%1σ	37Ar(ca) [fA]	%1σ	38Ar(a) [fA]	%1σ	38Ar(c) [fA]	%1σ	38Ar(k) [fA]	%1σ	38Ar(ca) [fA]	%1σ	38Ar(cl) [fA]	%1σ	39Ar(k) [fA]	%1σ	39Ar(ca) [fA]	%1σ	40Ar(r) [fA]	%1σ	40Ar(a) [fA]	%1σ	40Ar(c) [fA]	%1σ	40Ar(k) [fA]	%1σ
18D00508	1.8 %		2.019354	0.31	0.0000000	0.00	0.0119523	6.33	0.0000029	322.20	44.2186	6.33	0.3774172	0.31	0.0000000	0.00	0.1119117	0.33	0.0079593	11.52	0.0075373	322.20	9.26651	0.32	0.0284104	6.39	2.730610	84.27	608.1486	0.38	0.0000000	0.00	0.0056248	9.66
18D00510	1.9 %		1.808193	0.31	0.0000000	0.00	0.0173611	4.41	0.0000052	180.16	64.2290	4.41	0.3379512	0.31	0.0000000	0.00	0.1077590	0.35	0.0115612	10.59	0.0134391	180.16	8.92267	0.33	0.0412672	4.50	1.356571	153.32	544.5553	0.38	0.0000000	0.00	0.0054161	9.66
18D00511	2.0 %	✓	0.611828	0.37	0.0000000	0.00	0.0084286	9.19	0.0000026	380.62	31.1824	9.18	0.1143507	0.37	0.0000000	0.00	0.0480235	0.74	0.0056128	13.31	0.0066150	380.62	3.97644	0.73	0.0200347	9.23	1.527426	52.46	184.2582	0.43	0.0000000	0.00	0.0024137	9.68
18D00513	2.2 %	✓	0.580008	0.39	0.0000000	0.00	0.0083628	9.41	0.0000000	0.00	30.9388	9.40	0.1084034	0.39	0.0000000	0.00	0.0460041	0.77	0.0055690	13.46	0.0000000	0.00	3.80923	0.77	0.0198782	9.45	0.023214	######	174.6751	0.45	0.0000000	0.00	0.0023122	9.68
18D00514	2.4 %	✓	1.013614	0.33	0.0000000	0.00	0.0227506	3.44	0.0000023	399.49	84.1681	3.44	0.1894445	0.33	0.0000000	0.00	0.0812694	0.44	0.0151503	10.23	0.0058088	399.49	6.72927	0.43	0.0540780	3.56	1.102708	110.81	305.2601	0.40	0.0000000	0.00	0.0040847	9.66
18D00516	2.7 %	✓	0.466543	0.43	0.0000000	0.00	0.0135935	5.88	0.0000076	123.38	50.2903	5.87	0.0871969	0.43	0.0000000	0.00	0.0477914	0.75	0.0090523	11.28	0.0195032	123.39	3.95723	0.74	0.0323115	5.94	1.373531	49.43	140.5041	0.48	0.0000000	0.00	0.0024020	9.68
18D00517	3.0 %	✓	0.982592	0.35	0.0000000	0.00	0.0379226	2.24	0.0000017	550.89	140.2983	2.24	0.1836465	0.35	0.0000000	0.00	0.1059260	0.35	0.0252537	9.89	0.0043660	550.89	8.77089	0.34	0.0901417	2.42	2.795078	43.98	295.9175	0.41	0.0000000	0.00	0.0053239	9.66
18D00519	3.4 %	✓	0.644267	0.39	0.0000000	0.00	0.0398628	2.06	0.0000000	0.00	147.4761	2.06	0.1204136	0.39	0.0000000	0.00	0.1009269	0.36	0.0265457	9.85	0.0000000	0.00	8.35695	0.35	0.0947534	2.25	2.087327	42.32	194.0276	0.45	0.0000000	0.00	0.0050727	9.66
18D00520	3.9 %	✓	0.430110	0.46	0.0000000	0.00	0.0403033	2.13	0.0000000	0.00	149.1058	2.13	0.0803875	0.46	0.0000000	0.00	0.0897883	0.40	0.0268390	9.86	0.0000000	0.00	7.43465	0.39	0.0958005	2.32	2.565726	26.16	129.5319	0.51	0.0000000	0.00	0.0045128	9.66
18D00522	4.5 %	✓	0.320951	0.53	0.0000000	0.00	0.0416617	2.08	0.0000000	0.00	154.1312	2.07	0.0599857	0.53	0.0000000	0.00	0.0876684	0.44	0.0277436	9.85	0.0000000	0.00	7.25912	0.43	0.0990293	2.27	2.031584	27.28	96.6576	0.57	0.0000000	0.00	0.0044063	9.66
18D00523	5.2 %	✓	0.348284	0.51	0.0000000	0.00	0.0571721	1.58	0.0000000	0.00	211.5136	1.57	0.0650943	0.51	0.0000000	0.00	0.1108635	0.33	0.0380724	9.76	0.0000000	0.00	9.17972	0.32	0.1358975	1.82	2.059380	28.39	104.8893	0.55	0.0000000	0.00	0.0055721	9.66
18D00525	6.0 %	✓	0.293704	0.58	0.0000000	0.00	0.0685728	1.31	0.0000000	0.00	253.6913	1.30	0.0548934	0.58	0.0000000	0.00	0.1253222	0.31	0.0456644	9.72	0.0000000	0.00	10.37693	0.29	0.1629967	1.59	2.375732	23.23	88.4520	0.62	0.0000000	0.00	0.0062988	9.65
18D00526	6.9 %	✓	0.224654	0.69	0.0000000	0.00	0.0724355	1.24	0.0000010	919.66	267.9819	1.23	0.0419878	0.69	0.0000000	0.00	0.1247427	0.31	0.0482367	9.71	0.0026521	919.66	10.32895	0.29	0.1721784	1.54	1.733140	28.29	67.6567	0.72	0.0000000	0.00	0.0062697	9.65
18D00528	7.9 %	✓	0.197353	0.74	0.0000000	0.00	0.0750136	1.24	0.0000000	0.00	277.5200	1.23	0.0368853	0.74	0.0000000	0.00	0.1249966	0.29	0.0499536	9.71	0.0000000	0.00	10.34997	0.28	0.1783066	1.53	1.637648	28.36	59.4349	0.78	0.0000000	0.00	0.0062824	9.65
18D00529	9.0 %	✓	0.170852	0.86	0.0000000	0.00	0.0760648	1.26	0.0000000	0.00	281.4088	1.25	0.0319323	0.86	0.0000000	0.00	0.1205448	0.31	0.0506536	9.71	0.0000000	0.00	9.98135	0.30	0.1808052	1.55	1.466967	31.38	51.4538	0.89	0.0000000	0.00	0.0060587	9.65
18D00531	10.3 %		0.178344	0.85	0.0000000	0.00	0.0796249	1.24	0.0000000	0.00	294.5796	1.22	0.0333326	0.85	0.0000000	0.00	0.1048324	0.35	0.0530243	9.71	0.0000000	0.00	8.68033	0.34	0.1892674	1.53	0.504475	93.84	53.7102	0.88	0.0000000	0.00	0.0052690	9.66
18D00532	11.6 %		0.092139	1.20	0.0000000	0.00	0.0359567	2.23	0.0000000	0.00	133.0253	2.22	0.0172207	1.20	0.0000000	0.00	0.0481990	0.74	0.0239446	9.88	0.0000000	0.00	3.99097	0.73	0.0854688	2.41	0.069394	492.16	27.7485	1.22	0.0000000	0.00	0.0024225	9.68
18D00534	12.5 %		0.094072	1.23	0.0000000	0.00	0.0351579	2.46	0.0000000	0.00	130.0697	2.45	0.0175820	1.23	0.0000000	0.00	0.0396610	0.86	0.0234126	9.94	0.0000000	0.00	3.28401	0.86	0.0835698	2.62	0.339777	105.36	28.3307	1.25	0.0000000	0.00	0.0019934	9.69
18D00535	13.4 %		0.069427	1.52	0.0000000	0.00	0.0224295	3.56	0.0000000	0.00	82.9800	3.56	0.0129760	1.52	0.0000000	0.00	0.0267056	1.31	0.0149364	10.27	0.0000000	0.00	2.21127	1.31	0.0533146	3.67	0.579140	56.01	20.9087	1.53	0.0000000	0.00	0.0013422	9.74
18D00537	14.6 %		0.067845	1.51	0.0000000	0.00	0.0214020	3.66	0.0000000	0.00	79.1786	3.65	0.0126803	1.51	0.0000000	0.00	0.0192504	1.82	0.0142522	10.30	0.0000000	0.00	1.59397	1.82	0.0508723	3.77	0.452777	69.67	20.4323	1.52	0.0000000	0.00	0.0009675	9.82
18D00538	16.0 %		0.102421	1.15	0.0000000	0.00	0.0394011	2.20	0.0000000	0.00	145.7681	2.19	0.0191425	1.15	0.0000000	0.00	0.0226026	1.54	0.0262383	9.88	0.0000000	0.00	1.87154	1.54	0.0936560	2.37	0.574605	63.70	30.8451	1.18	0.0000000	0.00	0.0011360	9.77
18D00540	17.6 %		0.048454	2.06	0.0000000	0.00	0.0171393	4.61	0.0000037	253.45	63.4084	4.61	0.0090561	2.06	0.0000000	0.00	0.0111766	3.08	0.0114135	10.68	0.0094860	253.45	0.92544	3.08	0.0407399	4.70	0.640440	47.76	14.5925	2.07	0.0000000	0.00	0.0005617	10.13
18D00541	19.3 %		0.038674	2.58	0.0000000	0.00	0.0150915	5.48	0.0000000	0.00	55.8322	5.48	0.0072282	2.58	0.0000000	0.00	0.0069734	4.98	0.0100498	11.08	0.0000000	0.00	0.57741	4.98	0.0358722	5.55	0.628644	48.59	11.6471	2.59	0.0000000	0.00	0.0003505	10.86
18D00543	21.0 %		0.164308	0.89	0.0000000	0.00	0.0794552	1.20	0.0000000	0.00	293.9520	1.19	0.0307093	0.89	0.0000000	0.00	0.0232902	1.51	0.0529114	9.70	0.0000000	0.00	1.92848	1.51	0.1888642	1.50	0.147319	309.74	49.4831	0.92	0.0000000	0.00	0.0011706	9.77
		Σ	10.967993	0.11	0.0000000	0.00	0.9371161	0.44	0.0000269	98.55	3466.9481	0.44	2.0499180	0.11	0.0000000	0.00	1.7362299	0.10	0.6240507	2.35	0.0694075	98.55	143.76334	0.10	2.2275142	0.49	15.857015	27.06	3303.1209	0.13	0.0000000	0.00	0.0872643	2.26
		Σ							11.905136	0.11	3466.9481	0.44									4.4796061	1.56			145.99086	0.10							3319.0652	0.18

**Additional Parameters**			40Ar/39Ar	1σ	37Ar/39Ar	1σ	36Ar/39Ar	1σ	Time (days)	37Ar (decay)	39Ar (decay)	40Ar (moles)
18D00508	1.8 %		65.134851	0.206606	4.757280	0.301423	0.218540	0.000959	238.759	112.829916	1.00168966	2.906E-11
18D00510	1.9 %		60.598864	0.201409	7.165275	0.316680	0.203656	0.000919	238.773	112.860873	1.00168976	2.607E-11
18D00511	2.0 %	✓	46.487946	0.338444	7.802473	0.718772	0.155202	0.001245	238.780	112.876355	1.00168981	8.918E-12
18D00513	2.2 %	✓	45.612255	0.348570	8.079904	0.762385	0.153657	0.001296	238.794	112.908873	1.00168991	8.383E-12
18D00514	2.4 %	✓	45.164538	0.194742	12.408035	0.430108	0.152781	0.000815	238.801	112.924362	1.00168996	1.471E-11
18D00516	2.7 %	✓	35.563010	0.261776	12.605540	0.746117	0.120351	0.000994	238.815	112.955345	1.00169006	6.810E-12
18D00517	3.0 %	✓	33.711412	0.113617	15.833180	0.358238	0.115169	0.000539	238.822	112.970840	1.00169011	1.434E-11
18D00519	3.4 %	✓	23.204774	0.080919	17.449264	0.363564	0.080946	0.000398	238.836	113.001836	1.00169021	9.414E-12
18D00520	3.9 %	✓	17.542386	0.068187	19.800371	0.428029	0.062468	0.000339	238.843	113.017337	1.00169026	6.341E-12
18D00522	4.5 %	✓	13.412819	0.057149	20.946993	0.443491	0.049280	0.000286	238.857	113.048345	1.00169036	4.737E-12
18D00523	5.2 %	✓	11.481166	0.037049	22.705258	0.363665	0.043524	0.000213	238.864	113.063853	1.00169041	5.134E-12
18D00525	6.0 %	✓	8.618090	0.025517	24.069543	0.319889	0.034372	0.000169	238.878	113.094874	1.00169050	4.360E-12
18D00526	6.9 %	✓	6.608446	0.019722	25.519354	0.322936	0.028291	0.000144	238.885	113.110388	1.00169055	3.331E-12
18D00528	7.9 %	✓	5.801409	0.016672	26.359491	0.331643	0.025870	0.000129	238.899	113.141422	1.00169065	2.932E-12
18D00529	9.0 %	✓	5.208229	0.015942	27.691836	0.354425	0.024298	0.000131	238.906	113.156943	1.00169070	2.540E-12
18D00531	10.3 %		6.113007	0.021099	33.212276	0.420980	0.029085	0.000162	238.920	113.189542	1.00169080	2.603E-12
18D00532	11.6 %		6.824649	0.050399	32.632703	0.761924	0.031423	0.000292	238.927	113.205069	1.00169085	1.335E-12
18D00534	12.5 %		8.312463	0.070760	38.624047	0.999396	0.038375	0.000393	238.941	113.236129	1.00169095	1.344E-12
18D00535	13.4 %		8.977756	0.116351	36.642392	1.384502	0.040562	0.000599	238.948	113.251663	1.00169100	9.759E-13
18D00537	14.6 %		12.147348	0.215567	48.137385	1.951120	0.054259	0.001034	238.962	113.282736	1.00169110	9.591E-13
18D00538	16.0 %		15.403872	0.226783	74.174864	1.953054	0.072167	0.001132	238.969	113.298275	1.00169115	1.453E-12
18D00540	17.6 %		14.440999	0.427773	65.627637	3.588333	0.067893	0.002093	238.983	113.329361	1.00169125	6.697E-13
18D00541	19.3 %		17.966889	0.843680	91.038156	6.555679	0.087668	0.004197	238.990	113.344907	1.00169129	5.289E-13
18D00543	21.0 %		23.301385	0.319698	138.830710	2.516078	0.115127	0.001659	239.003	113.376006	1.00169139	2.368E-12

Procedure Blanks		36Ar ± 1σ (SE) [fA]	37Ar ± 1σ (SE) [fA]	38Ar ± 1σ (SE) [fA]	39Ar ± 1σ (SE) [fA]	40Ar ± 1σ (SE) [fA]
18D00508	1.8 %	0.0180242 ± 0.0002545	0.0440634 ± 0.0176067	0.0187091 ± 0.0170868	0.0274620 ± 0.0238724	5.4379086 ± 0.0455976
18D00510	1.9 %	0.0183540 ± 0.0002545	0.0336869 ± 0.0176067	0.0115343 ± 0.0170868	0.0276238 ± 0.0238724	5.4780222 ± 0.0455976
18D00511	2.0 %	0.0184507 ± 0.0002545	0.0296292 ± 0.0176067	0.0093213 ± 0.0170868	0.0285290 ± 0.0238724	5.4855046 ± 0.0455976
18D00513	2.2 %	0.0185343 ± 0.0002545	0.0230699 ± 0.0176067	0.0070813 ± 0.0170868	0.0314434 ± 0.0238724	5.4797727 ± 0.0455976
18D00514	2.4 %	0.0185273 ± 0.0002545	0.0207109 ± 0.0176067	0.0069571 ± 0.0170868	0.0330566 ± 0.0238724	5.4688965 ± 0.0455976
18D00516	2.7 %	0.0184452 ± 0.0002545	0.0171088 ± 0.0176067	0.0080816 ± 0.0170868	0.0361942 ± 0.0238724	5.4359354 ± 0.0455976
18D00517	3.0 %	0.0183781 ± 0.0002545	0.0157404 ± 0.0176067	0.0091686 ± 0.0170868	0.0375499 ± 0.0238724	5.4154767 ± 0.0455976
18D00519	3.4 %	0.0182106 ± 0.0002545	0.0135989 ± 0.0176067	0.0120260 ± 0.0170868	0.0395043 ± 0.0238724	5.3702705 ± 0.0455976
18D00520	3.9 %	0.0181168 ± 0.0002545	0.0127350 ± 0.0176067	0.0136650 ± 0.0170868	0.0400073 ± 0.0238724	5.3468311 ± 0.0455976
18D00522	4.5 %	0.0179237 ± 0.0002545	0.0112368 ± 0.0176067	0.0170734 ± 0.0170868	0.0399164 ± 0.0238724	5.3011518 ± 0.0455976
18D00523	5.2 %	0.0178298 ± 0.0002545	0.0105461 ± 0.0176067	0.0187417 ± 0.0170868	0.0392998 ± 0.0238724	5.2799016 ± 0.0455976
18D00525	6.0 %	0.0176582 ± 0.0002545	0.0091834 ± 0.0176067	0.0217918 ± 0.0170868	0.0369586 ± 0.0238724	5.2426570 ± 0.0455976
18D00526	6.9 %	0.0175843 ± 0.0002545	0.0084894 ± 0.0176067	0.0231030 ± 0.0170868	0.0352841 ± 0.0238724	5.2273341 ± 0.0455976
18D00528	7.9 %	0.0174683 ± 0.0002545	0.0070639 ± 0.0176067	0.0251585 ± 0.0170868	0.0311440 ± 0.0238724	5.2045682 ± 0.0455976
18D00529	9.0 %	0.0174284 ± 0.0002545	0.0063447 ± 0.0176067	0.0258626 ± 0.0170868	0.0288016 ± 0.0238724	5.1974786 ± 0.0455976
18D00531	10.3 %	0.0173885 ± 0.0002545	0.0049033 ± 0.0176067	0.0265693 ± 0.0170868	0.0237349 ± 0.0238724	5.1924373 ± 0.0455976
18D00532	11.6 %	0.0173913 ± 0.0002545	0.0042998 ± 0.0176067	0.0265281 ± 0.0170868	0.0214610 ± 0.0238724	5.1947572 ± 0.0455976
18D00534	12.5 %	0.0174395 ± 0.0002545	0.0034186 ± 0.0176067	0.0257298 ± 0.0170868	0.0177795 ± 0.0238724	5.2081972 ± 0.0455976
18D00535	13.4 %	0.0174844 ± 0.0002545	0.0032231 ± 0.0176067	0.0249937 ± 0.0170868	0.0166435 ± 0.0238724	5.2190236 ± 0.0455976
18D00537	14.6 %	0.0176123 ± 0.0002545	0.0035711 ± 0.0176067	0.0229389 ± 0.0170868	0.0165513 ± 0.0238724	5.2477575 ± 0.0455976
18D00538	16.0 %	0.0176935 ± 0.0002545	0.0042314 ± 0.0176067	0.0216717 ± 0.0170868	0.0179395 ± 0.0238724	5.2650531 ± 0.0455976
18D00540	17.6 %	0.0178831 ± 0.0002545	0.0068590 ± 0.0176067	0.0188242 ± 0.0170868	0.0245374 ± 0.0238724	5.3035742 ± 0.0455976
18D00541	19.3 %	0.0179882 ± 0.0002545	0.0089775 ± 0.0176067	0.0173258 ± 0.0170868	0.0301645 ± 0.0238724	5.3238697 ± 0.0455976
18D00543	21.0 %	0.0182086 ± 0.0002545	0.0152446 ± 0.0176067	0.0144222 ± 0.0170868	0.0472101 ± 0.0238724	5.3638075 ± 0.0455976

Intercept Values		36Ar ± 1σ (SE) [fA]	r2	Regression (type,n)	37Ar ± 1σ (SE) [fA]	r2	Regression (type,n)	38Ar ± 1σ (SE) [fA]	r2	Regression (type,n)	39Ar ± 1σ (SE) [fA]	r2	Regression (type,n)	40Ar ± 1σ (SE) [fA]	r2	Regression (type,n)
18D00508	1.8 %	1.9526722 ± 0.0020931	0.9723	EXP 150 of 150	0.3411927 ± 0.0166445	0.0008	EXP 150 of 150	0.5178178 ± 0.0167828	0.0026	EXP 150 of 150	9.2542597 ± 0.0156180	0.8287	EXP 150 of 150	610.861480 ± 0.068514	0.9975	EXP 150 of 150
18D00510	1.9 %	1.7570429 ± 0.0019995	0.9679	EXP 150 of 150	0.5257578 ± 0.0168068	0.0014	EXP 150 of 150	0.4769144 ± 0.0166680	0.0004	EXP 150 of 150	8.9258546 ± 0.0162615	0.8276	EXP 150 of 150	548.682196 ± 0.073877	0.9956	EXP 150 of 150
18D00511	2.0 %	0.6091947 ± 0.0011075	0.8714	EXP 150 of 150	0.2419370 ± 0.0175595	0.0043	EXP 149 of 150	0.1819461 ± 0.0180755	0.0000	EXP 150 of 150	3.9957131 ± 0.0158136	0.2746	EXP 150 of 150	191.273516 ± 0.111342	0.8283	EXP 150 of 150
18D00513	2.2 %	0.5789068 ± 0.0012314	0.8650	EXP 150 of 150	0.2462973 ± 0.0181162	0.0073	EXP 150 of 150	0.1641691 ± 0.0198311	0.0010	EXP 150 of 150	3.8324840 ± 0.0161970	0.3553	EXP 150 of 150	180.133986 ± 0.086693	0.7290	EXP 150 of 150
18D00514	2.4 %	1.0055786 ± 0.0013705	0.9510	EXP 150 of 150	0.7119935 ± 0.0172821	0.0564	EXP 150 of 150	0.2953272 ± 0.0152089	0.0018	EXP 150 of 150	6.7666869 ± 0.0156805	0.7509	EXP 150 of 150	311.835822 ± 0.094198	0.9038	EXP 150 of 150
18D00516	2.7 %	0.4757412 ± 0.0011246	0.8020	EXP 150 of 150	0.4205609 ± 0.0184752	0.0161	EXP 150 of 150	0.1697734 ± 0.0165114	0.0148	EXP 150 of 150	3.9964895 ± 0.0163393	0.3990	EXP 150 of 150	147.315928 ± 0.069220	0.9226	EXP 150 of 150
18D00517	3.0 %	0.9903330 ± 0.0015716	0.9311	EXP 150 of 150	1.2050897 ± 0.0190449	0.1819	EXP 149 of 150	0.3247462 ± 0.0163278	0.0284	EXP 150 of 150	8.8336308 ± 0.0161982	0.8653	EXP 150 of 150	304.133381 ± 0.092049	0.9013	EXP 150 of 150
18D00519	3.4 %	0.6697861 ± 0.0013279	0.8842	EXP 150 of 150	1.2693377 ± 0.0174324	0.1077	EXP 150 of 150	0.2568917 ± 0.0167477	0.0179	EXP 149 of 150	8.4292606 ± 0.0150630	0.8833	EXP 149 of 150	201.490247 ± 0.107919	0.3736	EXP 150 of 150
18D00520	3.9 %	0.4661451 ± 0.0011322	0.7849	EXP 150 of 150	1.2842009 ± 0.0191904	0.1413	EXP 150 of 150	0.1937052 ± 0.0186157	0.0000	EXP 150 of 150	7.5152616 ± 0.0150243	0.8147	EXP 150 of 150	137.448953 ± 0.068364	0.9578	EXP 150 of 150
18D00522	4.5 %	0.3632812 ± 0.0009351	0.7176	EXP 150 of 150	1.3290427 ± 0.0193584	0.1313	EXP 150 of 150	0.1528028 ± 0.0160545	0.0000	EXP 149 of 150	7.3441323 ± 0.0187014	0.7770	EXP 150 of 150	103.994724 ± 0.046283	0.9851	EXP 150 of 150
18D00523	5.2 %	0.4039922 ± 0.0009129	0.7916	EXP 150 of 150	1.8284619 ± 0.0188945	0.2308	EXP 150 of 150	0.2219267 ± 0.0179348	0.0088	EXP 150 of 150	9.2866357 ± 0.0155124	0.9147	EXP 150 of 150	112.234104 ± 0.055492	0.9773	EXP 150 of 150
18D00525	6.0 %	0.3626963 ± 0.0009374	0.7229	EXP 150 of 150	2.1959344 ± 0.0163858	0.3072	EXP 150 of 150	0.1969081 ± 0.0164623	0.0001	EXP 150 of 150	10.4996279 ± 0.0169180	0.9179	EXP 150 of 150	96.076731 ± 0.041646	0.9883	EXP 150 of 150
18D00526	6.9 %	0.3005374 ± 0.0008421	0.6575	EXP 150 of 150	2.3205245 ± 0.0156868	0.4093	EXP 150 of 150	0.2382578 ± 0.0163607	0.0556	EXP 150 of 150	10.4594307 ± 0.0165995	0.9134	EXP 150 of 150	74.623452 ± 0.031864	0.9953	EXP 150 of 150
18D00528	7.9 %	0.2768744 ± 0.0007553	0.6604	EXP 150 of 150	2.4041832 ± 0.0167195	0.3416	EXP 150 of 150	0.1866632 ± 0.0166445	0.0007	EXP 150 of 150	10.4822409 ± 0.0141460	0.9384	EXP 150 of 150	66.283394 ± 0.025039	0.9967	EXP 150 of 150
18D00529	9.0 %	0.2525956 ± 0.0007910	0.5485	EXP 150 of 150	2.4383554 ± 0.0179887	0.3785	EXP 150 of 150	0.1893009 ± 0.0174488	0.0019	EXP 150 of 150	10.1164650 ± 0.0154586	0.9288	EXP 149 of 150	58.124328 ± 0.023382	0.9974	EXP 150 of 150
18D00531	10.3 %	0.2630821 ± 0.0008062	0.6479	EXP 150 of 150	2.5534791 ± 0.0186083	0.4440	EXP 150 of 150	0.1859573 ± 0.0168070	0.0081	EXP 150 of 150	8.8283146 ± 0.0157357	0.9089	EXP 150 of 150	59.412360 ± 0.022732	0.9968	EXP 150 of 150
18D00532	11.6 %	0.1393911 ± 0.0005811	0.0617	EXP 149 of 150	1.1508478 ± 0.0168249	0.1706	EXP 150 of 150	0.0881806 ± 0.0164983	0.0000	EXP 150 of 150	4.0680193 ± 0.0162011	0.4488	EXP 149 of 150	33.015039 ± 0.020305	0.9982	EXP 150 of 150
18D00534	12.5 %	0.1405198 ± 0.0005966	0.1479	EXP 150 of 150	1.1257541 ± 0.0198063	0.0549	EXP 150 of 150	0.0714397 ± 0.0151368	0.0022	EXP 150 of 150	3.3606768 ± 0.0141253	0.4050	EXP 150 of 150	33.201119 ± 0.019256	0.9981	EXP 150 of 150
18D00535	13.4 %	0.1049701 ± 0.0005492	0.0152	EXP 150 of 150	0.7170510 ± 0.0179165	0.0316	EXP 150 of 150	0.0479653 ± 0.0151178	0.0019	EXP 150 of 150	2.2646313 ± 0.0156920	0.2512	EXP 150 of 150	25.549951 ± 0.019775	0.9983	EXP 150 of 150
18D00537	14.6 %	0.1026129 ± 0.0005216	0.0100	EXP 150 of 150	0.6835185 ± 0.0172199	0.0602	EXP 150 of 150	0.0578295 ± 0.0159622	0.0004	EXP 150 of 150	1.6493399 ± 0.0158709	0.0051	EXP 150 of 150	25.228286 ± 0.019829	0.9981	EXP 150 of 150
18D00538	16.0 %	0.1527668 ± 0.0006147	0.1625	EXP 150 of 150	1.2605290 ± 0.0194364	0.0891	EXP 150 of 150	0.0801585 ± 0.0161307	0.0001	EXP 150 of 150	1.9687278 ± 0.0155703	0.0248	EXP 150 of 150	35.536668 ± 0.022287	0.9974	EXP 150 of 150
18D00540	17.6 %	0.0803591 ± 0.0004873	0.1577	EXP 150 of 150	0.5431546 ± 0.0178281	0.0160	EXP 150 of 150	0.0594907 ± 0.0164734	0.0018	EXP 150 of 150	0.9836385 ± 0.0150058	0.0129	EXP 150 of 150	19.256242 ± 0.019674	0.9984	EXP 150 of 150
18D00541	19.3 %	0.0691953 ± 0.0004422	0.4761	EXP 150 of 150	0.4752530 ± 0.0195458	0.0107	EXP 150 of 150	0.0391471 ± 0.0152405	0.0004	EXP 150 of 150	0.6389523 ± 0.0154800	0.2417	EXP 150 of 150	16.342674 ± 0.019626	0.9987	EXP 150 of 150
18D00543	21.0 %	0.2503727 ± 0.0007840	0.6098	EXP 150 of 150	2.5334889 ± 0.0169410	0.3787	EXP 150 of 150	0.0693017 ± 0.0147580	0.0118	EXP 150 of 150	2.1490290 ± 0.0159605	0.0662	EXP 150 of 150	54.700799 ± 0.023976	0.9961	EXP 150 of 150

Project Info		Analyst	Irradiation	X-pos	Y-pos	Z/H-pos	Project	Experiment	Nmb
18D00508	1.8 %	Dan Miggins	17-OSU-05	0.00	0.00	18.17	Arctic\O-Connor (16-22)	18D00504	01
18D00510	1.9 %	Dan Miggins	17-OSU-05	0.00	0.00	18.17	Arctic\O-Connor (16-22)	18D00504	01
18D00511	2.0 %	Dan Miggins	17-OSU-05	0.00	0.00	18.17	Arctic\O-Connor (16-22)	18D00504	01
18D00513	2.2 %	Dan Miggins	17-OSU-05	0.00	0.00	18.17	Arctic\O-Connor (16-22)	18D00504	01
18D00514	2.4 %	Dan Miggins	17-OSU-05	0.00	0.00	18.17	Arctic\O-Connor (16-22)	18D00504	01
18D00516	2.7 %	Dan Miggins	17-OSU-05	0.00	0.00	18.17	Arctic\O-Connor (16-22)	18D00504	01
18D00517	3.0 %	Dan Miggins	17-OSU-05	0.00	0.00	18.17	Arctic\O-Connor (16-22)	18D00504	01
18D00519	3.4 %	Dan Miggins	17-OSU-05	0.00	0.00	18.17	Arctic\O-Connor (16-22)	18D00504	01
18D00520	3.9 %	Dan Miggins	17-OSU-05	0.00	0.00	18.17	Arctic\O-Connor (16-22)	18D00504	01
18D00522	4.5 %	Dan Miggins	17-OSU-05	0.00	0.00	18.17	Arctic\O-Connor (16-22)	18D00504	01
18D00523	5.2 %	Dan Miggins	17-OSU-05	0.00	0.00	18.17	Arctic\O-Connor (16-22)	18D00504	01
18D00525	6.0 %	Dan Miggins	17-OSU-05	0.00	0.00	18.17	Arctic\O-Connor (16-22)	18D00504	01
18D00526	6.9 %	Dan Miggins	17-OSU-05	0.00	0.00	18.17	Arctic\O-Connor (16-22)	18D00504	01
18D00528	7.9 %	Dan Miggins	17-OSU-05	0.00	0.00	18.17	Arctic\O-Connor (16-22)	18D00504	01
18D00529	9.0 %	Dan Miggins	17-OSU-05	0.00	0.00	18.17	Arctic\O-Connor (16-22)	18D00504	01
18D00531	10.3 %	Dan Miggins	17-OSU-05	0.00	0.00	18.17	Arctic\O-Connor (16-22)	18D00504	01
18D00532	11.6 %	Dan Miggins	17-OSU-05	0.00	0.00	18.17	Arctic\O-Connor (16-22)	18D00504	01
18D00534	12.5 %	Dan Miggins	17-OSU-05	0.00	0.00	18.17	Arctic\O-Connor (16-22)	18D00504	01
18D00535	13.4 %	Dan Miggins	17-OSU-05	0.00	0.00	18.17	Arctic\O-Connor (16-22)	18D00504	01
18D00537	14.6 %	Dan Miggins	17-OSU-05	0.00	0.00	18.17	Arctic\O-Connor (16-22)	18D00504	01
18D00538	16.0 %	Dan Miggins	17-OSU-05	0.00	0.00	18.17	Arctic\O-Connor (16-22)	18D00504	01
18D00540	17.6 %	Dan Miggins	17-OSU-05	0.00	0.00	18.17	Arctic\O-Connor (16-22)	18D00504	01
18D00541	19.3 %	Dan Miggins	17-OSU-05	0.00	0.00	18.17	Arctic\O-Connor (16-22)	18D00504	01
18D00543	21.0 %	Dan Miggins	17-OSU-05	0.00	0.00	18.17	Arctic\O-Connor (16-22)	18D00504	01

Sample Parameters		Sample	Material	Location	Standard Name	Standard (in Ma)	%1σ	Standard Reference	Standard 40Ar/39Ar	%1σ	J	%1σ	Air 40Ar/36Ar	%1σ	MDF (lin)	%1σ	Volume Ratio	Sensitivity (mol/volt)	Day	Month	Year	Hour	Min	Resist
18D00508	1.8 %	PS59-309-39	Groundmass	Gakkel Ridge	FCT-NM (5B12-17)	28.201	0.082	Kuiper et al (2008)	9.67023	0.082	0.00162534	0.082	302.34	0.155	0.9943368	0.070	1	4.8E-14	5	JAN	2018	6	3	1
18D00510	1.9 %	PS59-309-39	Groundmass	Gakkel Ridge	FCT-NM (5B12-17)	28.201	0.082	Kuiper et al (2008)	9.67023	0.082	0.00162534	0.082	302.34	0.155	0.9943368	0.070	1	4.8E-14	5	JAN	2018	6	23	1
18D00511	2.0 %	PS59-309-39	Groundmass	Gakkel Ridge	FCT-NM (5B12-17)	28.201	0.082	Kuiper et al (2008)	9.67023	0.082	0.00162534	0.082	302.34	0.155	0.9943368	0.070	1	4.8E-14	5	JAN	2018	6	33	1
18D00513	2.2 %	PS59-309-39	Groundmass	Gakkel Ridge	FCT-NM (5B12-17)	28.201	0.082	Kuiper et al (2008)	9.67023	0.082	0.00162534	0.082	302.34	0.155	0.9943368	0.070	1	4.8E-14	5	JAN	2018	6	54	1
18D00514	2.4 %	PS59-309-39	Groundmass	Gakkel Ridge	FCT-NM (5B12-17)	28.201	0.082	Kuiper et al (2008)	9.67023	0.082	0.00162534	0.082	302.34	0.155	0.9943368	0.070	1	4.8E-14	5	JAN	2018	7	4	1
18D00516	2.7 %	PS59-309-39	Groundmass	Gakkel Ridge	FCT-NM (5B12-17)	28.201	0.082	Kuiper et al (2008)	9.67023	0.082	0.00162534	0.082	302.34	0.155	0.9943368	0.070	1	4.8E-14	5	JAN	2018	7	24	1
18D00517	3.0 %	PS59-309-39	Groundmass	Gakkel Ridge	FCT-NM (5B12-17)	28.201	0.082	Kuiper et al (2008)	9.67023	0.082	0.00162534	0.082	302.34	0.155	0.9943368	0.070	1	4.8E-14	5	JAN	2018	7	34	1
18D00519	3.4 %	PS59-309-39	Groundmass	Gakkel Ridge	FCT-NM (5B12-17)	28.201	0.082	Kuiper et al (2008)	9.67023	0.082	0.00162534	0.082	302.34	0.155	0.9943368	0.070	1	4.8E-14	5	JAN	2018	7	54	1
18D00520	3.9 %	PS59-309-39	Groundmass	Gakkel Ridge	FCT-NM (5B12-17)	28.201	0.082	Kuiper et al (2008)	9.67023	0.082	0.00162534	0.082	302.34	0.155	0.9943368	0.070	1	4.8E-14	5	JAN	2018	8	4	1
18D00522	4.5 %	PS59-309-39	Groundmass	Gakkel Ridge	FCT-NM (5B12-17)	28.201	0.082	Kuiper et al (2008)	9.67023	0.082	0.00162534	0.082	302.34	0.155	0.9943368	0.070	1	4.8E-14	5	JAN	2018	8	24	1
18D00523	5.2 %	PS59-309-39	Groundmass	Gakkel Ridge	FCT-NM (5B12-17)	28.201	0.082	Kuiper et al (2008)	9.67023	0.082	0.00162534	0.082	302.34	0.155	0.9943368	0.070	1	4.8E-14	5	JAN	2018	8	34	1
18D00525	6.0 %	PS59-309-39	Groundmass	Gakkel Ridge	FCT-NM (5B12-17)	28.201	0.082	Kuiper et al (2008)	9.67023	0.082	0.00162534	0.082	302.34	0.155	0.9943368	0.070	1	4.8E-14	5	JAN	2018	8	54	1
18D00526	6.9 %	PS59-309-39	Groundmass	Gakkel Ridge	FCT-NM (5B12-17)	28.201	0.082	Kuiper et al (2008)	9.67023	0.082	0.00162534	0.082	302.34	0.155	0.9943368	0.070	1	4.8E-14	5	JAN	2018	9	4	1
18D00528	7.9 %	PS59-309-39	Groundmass	Gakkel Ridge	FCT-NM (5B12-17)	28.201	0.082	Kuiper et al (2008)	9.67023	0.082	0.00162534	0.082	302.34	0.155	0.9943368	0.070	1	4.8E-14	5	JAN	2018	9	24	1
18D00529	9.0 %	PS59-309-39	Groundmass	Gakkel Ridge	FCT-NM (5B12-17)	28.201	0.082	Kuiper et al (2008)	9.67023	0.082	0.00162534	0.082	302.34	0.155	0.9943368	0.070	1	4.8E-14	5	JAN	2018	9	34	1
18D00531	10.3 %	PS59-309-39	Groundmass	Gakkel Ridge	FCT-NM (5B12-17)	28.201	0.082	Kuiper et al (2008)	9.67023	0.082	0.00162534	0.082	302.34	0.155	0.9943368	0.070	1	4.8E-14	5	JAN	2018	9	55	1
18D00532	11.6 %	PS59-309-39	Groundmass	Gakkel Ridge	FCT-NM (5B12-17)	28.201	0.082	Kuiper et al (2008)	9.67023	0.082	0.00162534	0.082	302.34	0.155	0.9943368	0.070	1	4.8E-14	5	JAN	2018	10	5	1
18D00534	12.5 %	PS59-309-39	Groundmass	Gakkel Ridge	FCT-NM (5B12-17)	28.201	0.082	Kuiper et al (2008)	9.67023	0.082	0.00162534	0.082	302.34	0.155	0.9943368	0.070	1	4.8E-14	5	JAN	2018	10	25	1
18D00535	13.4 %	PS59-309-39	Groundmass	Gakkel Ridge	FCT-NM (5B12-17)	28.201	0.082	Kuiper et al (2008)	9.67023	0.082	0.00162534	0.082	302.34	0.155	0.9943368	0.070	1	4.8E-14	5	JAN	2018	10	35	1
18D00537	14.6 %	PS59-309-39	Groundmass	Gakkel Ridge	FCT-NM (5B12-17)	28.201	0.082	Kuiper et al (2008)	9.67023	0.082	0.00162534	0.082	302.34	0.155	0.9943368	0.070	1	4.8E-14	5	JAN	2018	10	55	1
18D00538	16.0 %	PS59-309-39	Groundmass	Gakkel Ridge	FCT-NM (5B12-17)	28.201	0.082	Kuiper et al (2008)	9.67023	0.082	0.00162534	0.082	302.34	0.155	0.9943368	0.070	1	4.8E-14	5	JAN	2018	11	5	1
18D00540	17.6 %	PS59-309-39	Groundmass	Gakkel Ridge	FCT-NM (5B12-17)	28.201	0.082	Kuiper et al (2008)	9.67023	0.082	0.00162534	0.082	302.34	0.155	0.9943368	0.070	1	4.8E-14	5	JAN	2018	11	25	1
18D00541	19.3 %	PS59-309-39	Groundmass	Gakkel Ridge	FCT-NM (5B12-17)	28.201	0.082	Kuiper et al (2008)	9.67023	0.082	0.00162534	0.082	302.34	0.155	0.9943368	0.070	1	4.8E-14	5	JAN	2018	11	35	1
18D00543	21.0 %	PS59-309-39	Groundmass	Gakkel Ridge	FCT-NM (5B12-17)	28.201	0.082	Kuiper et al (2008)	9.67023	0.082	0.00162534	0.082	302.34	0.155	0.9943368	0.070	1	4.8E-14	5	JAN	2018	11	55	1

Irradiation Constants		40/36(a)	%1σ	40/36(c)	%1σ	38/36(a)	%1σ	38/36(c)	%1σ	39/37(ca)	%1σ	38/37(ca)	%1σ	36/37(ca)	%1σ	40/39(k)	%1σ	38/39(k)	%1σ	36/38(cl)	%1σ	K/Ca	%1σ	K/Cl	%1σ	Ca/Cl	%1σ
18D00508	1.8 %	301.16	0.22	0.018	35	0.1869	0	1.493	3	0.000643	0.92	0.00018	9.63	0.00027	0.17	0.000607	9.65	0.012077	0.09	0	0	0.43	0	0	0	0	0
18D00510	1.9 %	301.16	0.22	0.018	35	0.1869	0	1.493	3	0.000643	0.92	0.00018	9.63	0.00027	0.17	0.000607	9.65	0.012077	0.09	0	0	0.43	0	0	0	0	0
18D00511	2.0 %	301.16	0.22	0.018	35	0.1869	0	1.493	3	0.000643	0.92	0.00018	9.63	0.00027	0.17	0.000607	9.65	0.012077	0.09	0	0	0.43	0	0	0	0	0
18D00513	2.2 %	301.16	0.22	0.018	35	0.1869	0	1.493	3	0.000643	0.92	0.00018	9.63	0.00027	0.17	0.000607	9.65	0.012077	0.09	0	0	0.43	0	0	0	0	0
18D00514	2.4 %	301.16	0.22	0.018	35	0.1869	0	1.493	3	0.000643	0.92	0.00018	9.63	0.00027	0.17	0.000607	9.65	0.012077	0.09	0	0	0.43	0	0	0	0	0
18D00516	2.7 %	301.16	0.22	0.018	35	0.1869	0	1.493	3	0.000643	0.92	0.00018	9.63	0.00027	0.17	0.000607	9.65	0.012077	0.09	0	0	0.43	0	0	0	0	0
18D00517	3.0 %	301.16	0.22	0.018	35	0.1869	0	1.493	3	0.000643	0.92	0.00018	9.63	0.00027	0.17	0.000607	9.65	0.012077	0.09	0	0	0.43	0	0	0	0	0
18D00519	3.4 %	301.16	0.22	0.018	35	0.1869	0	1.493	3	0.000643	0.92	0.00018	9.63	0.00027	0.17	0.000607	9.65	0.012077	0.09	0	0	0.43	0	0	0	0	0
18D00520	3.9 %	301.16	0.22	0.018	35	0.1869	0	1.493	3	0.000643	0.92	0.00018	9.63	0.00027	0.17	0.000607	9.65	0.012077	0.09	0	0	0.43	0	0	0	0	0
18D00522	4.5 %	301.16	0.22	0.018	35	0.1869	0	1.493	3	0.000643	0.92	0.00018	9.63	0.00027	0.17	0.000607	9.65	0.012077	0.09	0	0	0.43	0	0	0	0	0
18D00523	5.2 %	301.16	0.22	0.018	35	0.1869	0	1.493	3	0.000643	0.92	0.00018	9.63	0.00027	0.17	0.000607	9.65	0.012077	0.09	0	0	0.43	0	0	0	0	0
18D00525	6.0 %	301.16	0.22	0.018	35	0.1869	0	1.493	3	0.000643	0.92	0.00018	9.63	0.00027	0.17	0.000607	9.65	0.012077	0.09	0	0	0.43	0	0	0	0	0
18D00526	6.9 %	301.16	0.22	0.018	35	0.1869	0	1.493	3	0.000643	0.92	0.00018	9.63	0.00027	0.17	0.000607	9.65	0.012077	0.09	0	0	0.43	0	0	0	0	0
18D00528	7.9 %	301.16	0.22	0.018	35	0.1869	0	1.493	3	0.000643	0.92	0.00018	9.63	0.00027	0.17	0.000607	9.65	0.012077	0.09	0	0	0.43	0	0	0	0	0
18D00529	9.0 %	301.16	0.22	0.018	35	0.1869	0	1.493	3	0.000643	0.92	0.00018	9.63	0.00027	0.17	0.000607	9.65	0.012077	0.09	0	0	0.43	0	0	0	0	0
18D00531	10.3 %	301.16	0.22	0.018	35	0.1869	0	1.493	3	0.000643	0.92	0.00018	9.63	0.00027	0.17	0.000607	9.65	0.012077	0.09	0	0	0.43	0	0	0	0	0
18D00532	11.6 %	301.16	0.22	0.018	35	0.1869	0	1.493	3	0.000643	0.92	0.00018	9.63	0.00027	0.17	0.000607	9.65	0.012077	0.09	0	0	0.43	0	0	0	0	0
18D00534	12.5 %	301.16	0.22	0.018	35	0.1869	0	1.493	3	0.000643	0.92	0.00018	9.63	0.00027	0.17	0.000607	9.65	0.012077	0.09	0	0	0.43	0	0	0	0	0
18D00535	13.4 %	301.16	0.22	0.018	35	0.1869	0	1.493	3	0.000643	0.92	0.00018	9.63	0.00027	0.17	0.000607	9.65	0.012077	0.09	0	0	0.43	0	0	0	0	0
18D00537	14.6 %	301.16	0.22	0.018	35	0.1869	0	1.493	3	0.000643	0.92	0.00018	9.63	0.00027	0.17	0.000607	9.65	0.012077	0.09	0	0	0.43	0	0	0	0	0
18D00538	16.0 %	301.16	0.22	0.018	35	0.1869	0	1.493	3	0.000643	0.92	0.00018	9.63	0.00027	0.17	0.000607	9.65	0.012077	0.09	0	0	0.43	0	0	0	0	0
18D00540	17.6 %	301.16	0.22	0.018	35	0.1869	0	1.493	3	0.000643	0.92	0.00018	9.63	0.00027	0.17	0.000607	9.65	0.012077	0.09	0	0	0.43	0	0	0	0	0
18D00541	19.3 %	301.16	0.22	0.018	35	0.1869	0	1.493	3	0.000643	0.92	0.00018	9.63	0.00027	0.17	0.000607	9.65	0.012077	0.09	0	0	0.43	0	0	0	0	0
18D00543	21.0 %	301.16	0.22	0.018	35	0.1869	0	1.493	3	0.000643	0.92	0.00018	9.63	0.00027	0.17	0.000607	9.65	0.012077	0.09	0	0	0.43	0	0	0	0	0

18D00504.AGE >>> PS59-309-39 >>> ARCTIC | O-CONNOR (16-22) PROJECT

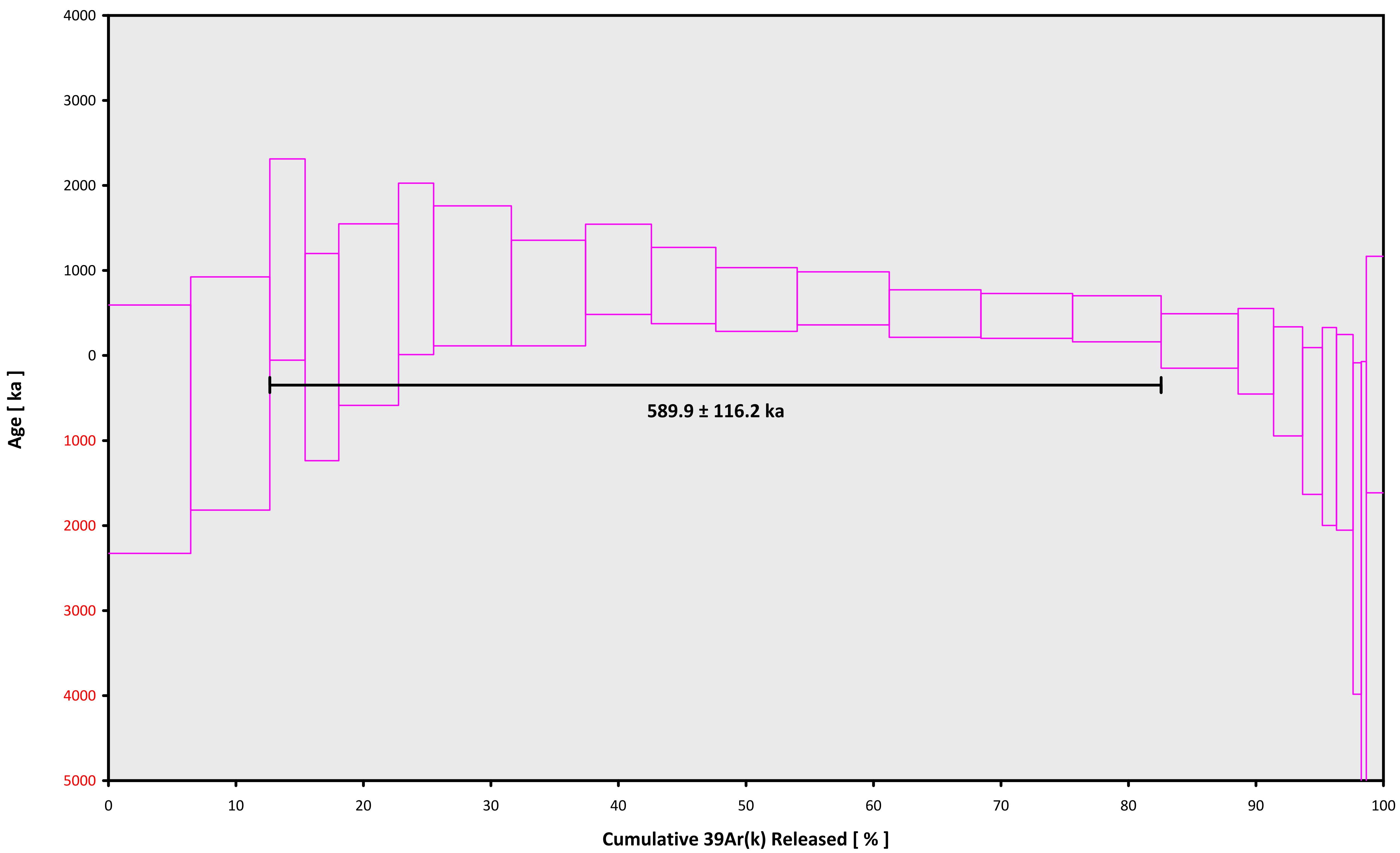

Ar-Ages in ka

WEIGHTED PLATEAU
589.9 ± 116.2
TOTAL FUSION
324.2 ± 175.4
NORMAL ISOCHRON
489.6 ± 168.3
INVERSE ISOCHRON
490.3 ± 148.6

MSWD (PROBABILITY)
0.86 (59%)

Sample Info

Groundmass
Gakkel Ridge
Dan Miggins

IRR = 17-OSU-05 (5B12-17)
J = 0.00162534 ± 0.00000133

18D00504.AGE >>> PS59-309-39 >>> ARCTIC | O-CONNOR (16-22) PROJECT

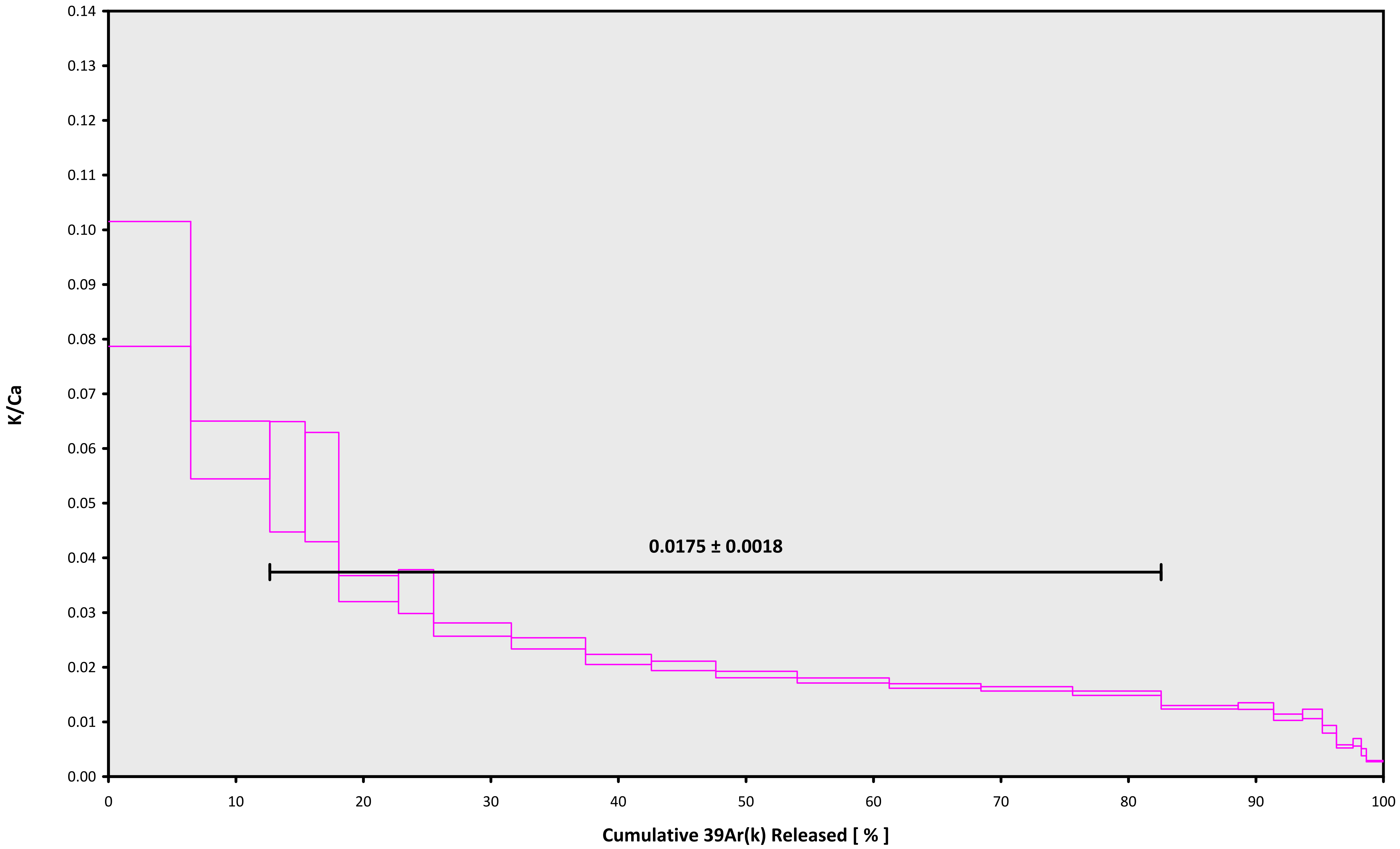

Ar-Ages in ka

WEIGHTED PLATEAU
589.9 ± 116.2
TOTAL FUSION
324.2 ± 175.4
NORMAL ISOCHRON
489.6 ± 168.3
INVERSE ISOCHRON
490.3 ± 148.6

Sample Info

Groundmass
Gakkel Ridge
Dan Miggins

IRR = 17-OSU-05 (5B12-17)
J = 0.00162534 ± 0.00000133

18D00504.AGE >>> PS59-309-39 >>> ARCTIC | O-CONNOR (16-22) PROJECT

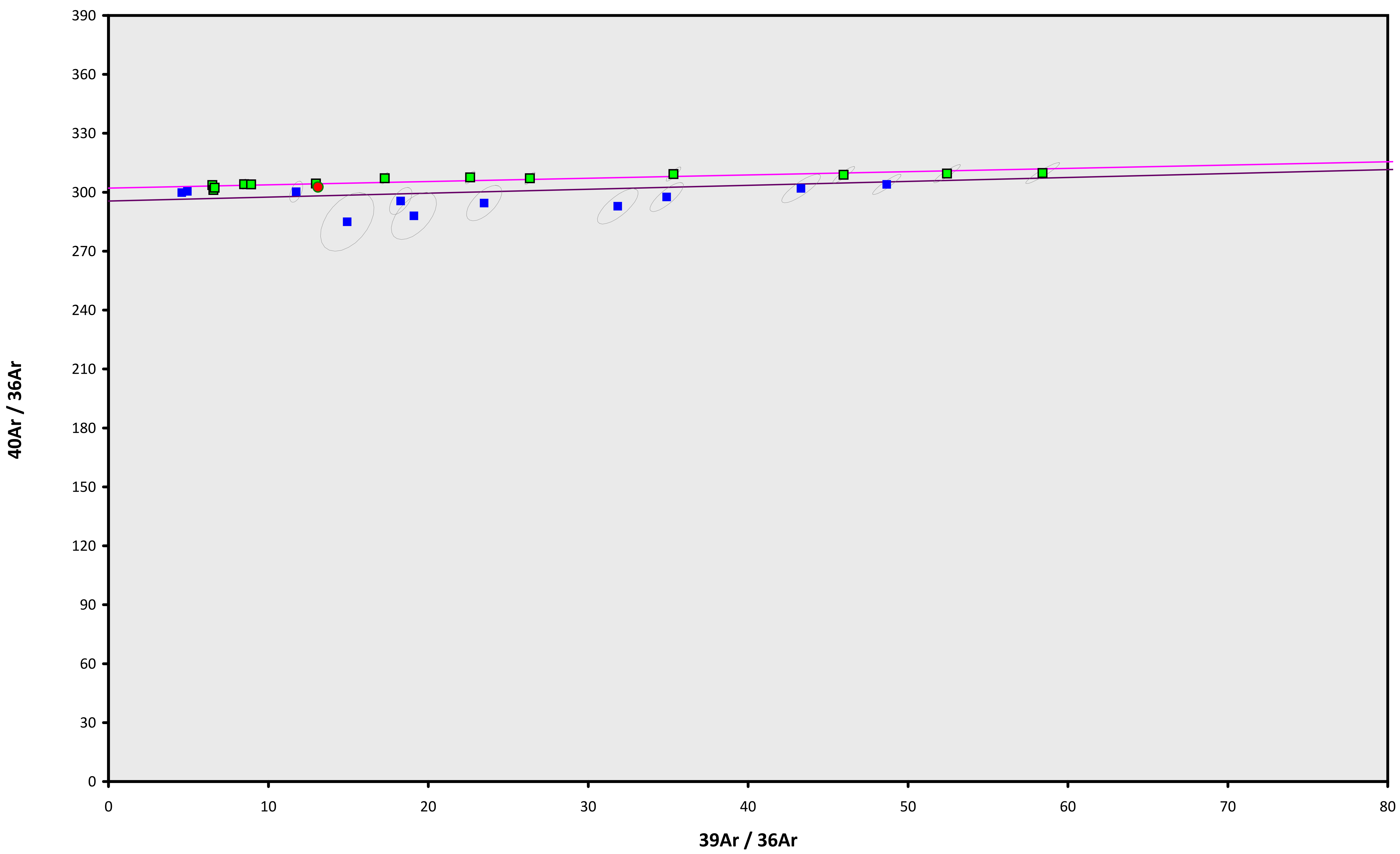

Ar-Ages in ka

WEIGHTED PLATEAU
589.9 ± 116.2
TOTAL FUSION
324.2 ± 175.4
NORMAL ISOCHRON
489.6 ± 168.3
INVERSE ISOCHRON
490.3 ± 148.6

MSWD (PROBABILITY)
0.88 (56%)

40AR/36AR INTERCEPT
302.1 ± 1.2

Sample Info

Groundmass
Gakkel Ridge
Dan Miggins

IRR = 17-OSU-05 (5B12-17)
J = 0.00162534 ± 0.00000133

18D00504.AGE >>> PS59-309-39 >>> ARCTIC | O-CONNOR (16-22) PROJECT

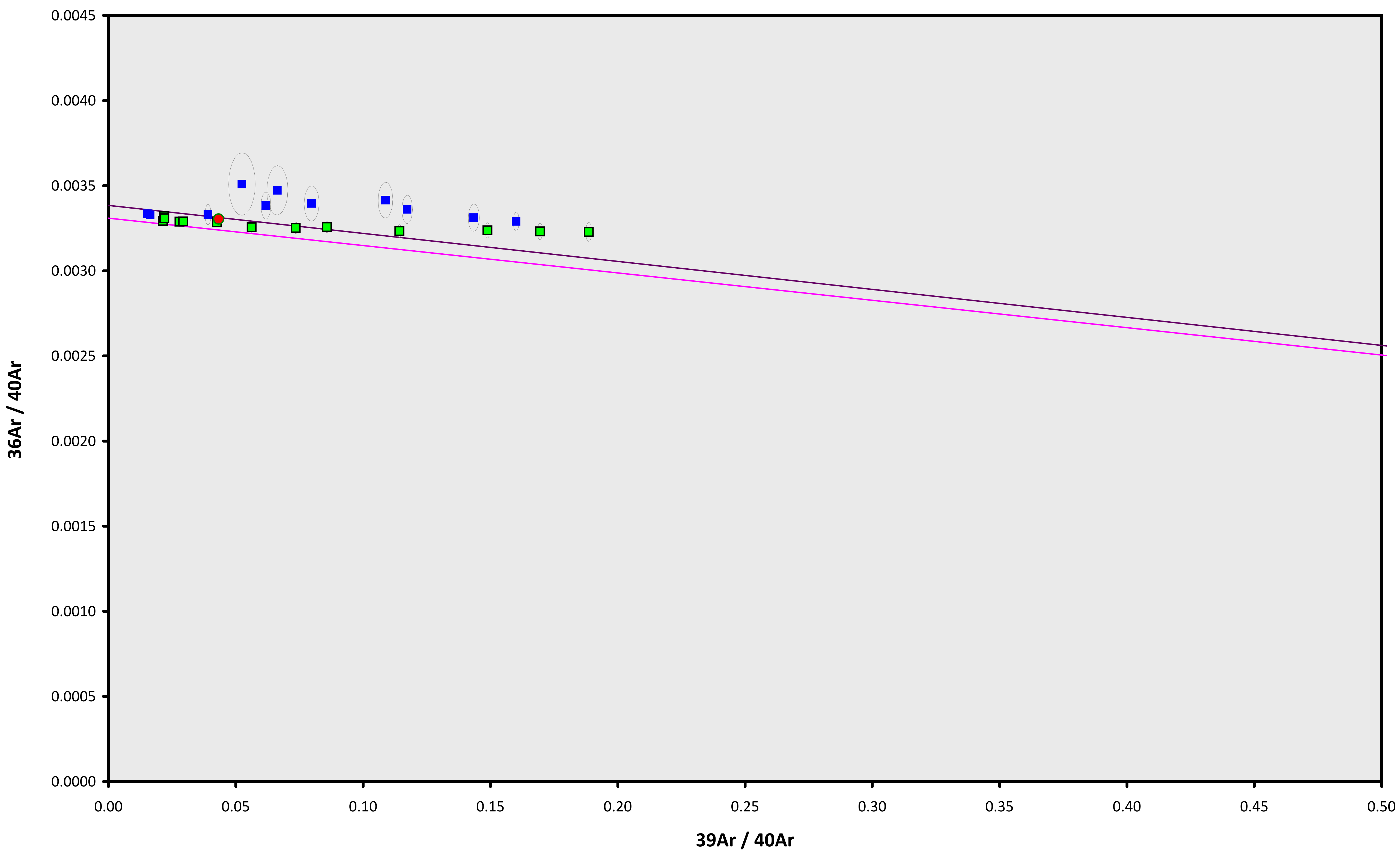

Ar-Ages in ka

WEIGHTED PLATEAU
589.9 ± 116.2
TOTAL FUSION
324.2 ± 175.4
NORMAL ISOCHRON
489.6 ± 168.3
INVERSE ISOCHRON
490.3 ± 148.6

MSWD (PROBABILITY)
0.87 (57%)

SPREADING FACTOR
2.8%

40AR/36AR INTERCEPT
302.2 ± 1.2

Sample Info

Groundmass
Gakkel Ridge
Dan Miggins

IRR = 17-OSU-05 (5B12-17)
J = 0.00162534 ± 0.00000133

Relative Abundances

			36Ar [fA]	%1σ	37Ar [fA]	%1σ	38Ar [fA]	%1σ	39Ar [fA]	%1σ	40Ar [fA]	%1σ	40(r)/39(k) ± 2σ	Age ± 2σ (ka)	40Ar(r) (%)	39Ar(k) (%)	K/Ca ± 2σ
17D24042	1.8 %	✓	4.195136	0.286	35.4660	0.386	1.4680109	1.667	44.29848	0.088	1243.3086	0.035	0.08673 ± 0.20991	254.0 ± 614.8	0.31	13.78	0.5368 ± 0.0042
17D24044	1.9 %	✓	1.827346	0.308	25.1896	0.470	0.7822699	3.077	30.33573	0.101	544.0097	0.081	0.05100 ± 0.14204	149.3 ± 415.9	0.28	9.44	0.5176 ± 0.0050
17D24045	2.0 %	✓	1.039750	0.348	21.9887	0.492	0.4773481	4.835	20.11359	0.138	309.7376	0.141	0.08426 ± 0.13637	246.7 ± 399.3	0.55	6.26	0.3931 ± 0.0040
17D24046	2.1 %	✓	0.574865	0.430	13.7083	0.744	0.2655325	9.253	11.34308	0.206	171.9034	0.254	0.15126 ± 0.16688	442.9 ± 488.6	1.00	3.53	0.3555 ± 0.0055
17D24048	2.2 %	✓	0.394636	0.525	13.6378	0.736	0.2238953	10.678	9.10383	0.266	117.9375	0.371	0.15898 ± 0.17690	465.5 ± 517.9	1.23	2.83	0.2868 ± 0.0045
17D24049	2.3 %	✓	0.380765	0.539	17.0272	0.610	0.1997678	11.664	8.74609	0.262	112.9869	0.387	0.10314 ± 0.18232	302.0 ± 533.8	0.80	2.72	0.2206 ± 0.0029
17D24050	2.4 %	✓	0.269568	0.662	15.4766	0.669	0.1493088	16.282	6.93752	0.346	79.5130	0.550	0.06281 ± 0.20580	183.9 ± 602.6	0.55	2.16	0.1925 ± 0.0029
17D24052	2.5 %	✓	0.226798	0.783	17.2821	0.615	0.1025668	23.797	6.49481	0.370	66.6980	0.655	0.07856 ± 0.21709	230.0 ± 635.6	0.76	2.02	0.1613 ± 0.0023
17D24053	2.7 %	✓	0.213059	0.815	20.6913	0.520	0.0940463	25.759	6.52273	0.341	62.1762	0.703	0.05462 ± 0.21282	159.9 ± 623.1	0.57	2.03	0.1353 ± 0.0017
17D24054	3.0 %	✓	0.169902	0.969	17.7367	0.611	0.1059718	23.031	5.13990	0.434	49.5270	0.883	0.06411 ± 0.26021	187.7 ± 761.9	0.66	1.60	0.1243 ± 0.0019
17D24056	3.4 %	✓	0.219421	0.786	34.1315	0.380	0.1490179	15.552	7.77632	0.290	63.3451	0.690	0.09169 ± 0.17808	268.5 ± 521.4	1.12	2.41	0.0977 ± 0.0009
17D24057	3.8 %	✓	0.221339	0.789	47.6390	0.340	0.1343261	18.428	8.95818	0.254	62.2806	0.702	0.01814 ± 0.15570	53.1 ± 455.9	0.26	2.78	0.0806 ± 0.0007
17D24058	4.2 %	✓	0.254698	0.691	66.4635	0.307	0.2255352	10.937	10.74525	0.222	71.4487	0.612	0.08470 ± 0.13138	248.0 ± 384.6	1.27	3.33	0.0692 ± 0.0005
17D24060	4.6 %	✓	0.203719	0.829	68.1797	0.310	0.1455893	17.115	9.84652	0.248	55.4202	0.789	0.02094 ± 0.13857	61.3 ± 405.8	0.37	3.05	0.0618 ± 0.0005
17D24061	5.2 %	✓	0.191853	0.862	75.6335	0.304	0.1842822	12.676	9.73953	0.242	51.1038	0.855	0.00264 ± 0.13825	7.7 ± 404.8	0.05	3.02	0.0551 ± 0.0004
17D24062	5.8 %	✓	0.276807	0.670	139.1757	0.281	0.2522065	9.074	14.86109	0.178	72.0624	0.607	0.05315 ± 0.09818	155.6 ± 287.5	1.09	4.60	0.0456 ± 0.0003
17D24064	6.5 %	✓	0.223135	0.753	145.0965	0.281	0.1980703	11.798	13.51578	0.189	55.2440	0.791	0.03251 ± 0.10107	95.2 ± 295.9	0.79	4.18	0.0398 ± 0.0003
17D24065	7.2 %	✓	0.217160	0.786	167.7047	0.280	0.2211202	10.606	13.75603	0.187	51.9811	0.841	0.05676 ± 0.10006	166.2 ± 293.0	1.49	4.25	0.0350 ± 0.0002
17D24066	8.0 %	✓	0.194875	0.860	186.2898	0.278	0.2424577	9.737	13.77515	0.188	43.6506	1.001	0.04257 ± 0.09857	124.6 ± 288.6	1.33	4.25	0.0315 ± 0.0002
17D24068	8.9 %	✓	0.191360	0.879	196.1508	0.278	0.1921540	12.490	13.49623	0.182	42.0023	1.040	0.05791 ± 0.10083	169.6 ± 295.2	1.84	4.16	0.0293 ± 0.0002
17D24069	9.7 %	✓	0.161852	0.999	165.4503	0.279	0.1715548	14.240	10.54840	0.230	35.3433	1.237	0.04182 ± 0.12585	122.5 ± 368.5	1.24	3.25	0.0271 ± 0.0002
17D24070	10.6 %	✓	0.152009	1.067	150.6364	0.281	0.1540330	16.710	9.19371	0.263	33.3235	1.311	0.01730 ± 0.14452	50.7 ± 423.2	0.47	2.83	0.0260 ± 0.0002
17D24072	11.6 %	✓	0.143612	1.113	139.3070	0.282	0.1456967	16.646	8.03795	0.294	31.7159	1.378	0.01764 ± 0.16395	51.7 ± 480.1	0.44	2.47	0.0245 ± 0.0002
17D24073	12.5 %	✓	0.116234	1.341	107.9424	0.287	0.1029670	23.941	5.89831	0.369	26.2054	1.668	0.04500 ± 0.22007	131.8 ± 644.4	1.00	1.81	0.0232 ± 0.0002
17D24074	13.4 %	✓	0.109199	1.428	93.0036	0.291	0.0856382	28.633	5.02928	0.471	25.2369	1.732	0.03768 ± 0.25803	110.3 ± 755.5	0.74	1.55	0.0230 ± 0.0003
17D24076	14.6 %	✓	0.127926	1.237	87.5836	0.295	0.0939185	26.085	4.69442	0.502	31.0935	1.405	0.00598 ± 0.27916	17.5 ± 817.4	0.09	1.44	0.0228 ± 0.0003
17D24077	16.0 %	✓	0.120547	1.298	78.6142	0.299	0.1074126	22.131	4.44096	0.511	29.6635	1.473	0.01706 ± 0.29296	50.0 ± 857.8	0.25	1.37	0.0240 ± 0.0003
17D24078	17.6 %	✓	0.180065	0.917	80.1830	0.300	0.0792379	28.499	4.21714	0.564	47.1249	0.927	0.01727 ± 0.32063	50.6 ± 938.9	0.15	1.30	0.0223 ± 0.0003
17D24080	18.6 %	✓	0.143223	1.110	57.3601	0.321	0.0785475	31.042	2.75735	0.812	37.6006	1.163	0.16751 ± 0.47868	490.6 ± 1402.1	1.21	0.85	0.0204 ± 0.0004
17D24082	20.0 %	✓	0.161182	1.006	61.9469	0.313	0.0680381	34.927	2.47055	0.897	43.2138	1.011	0.07231 ± 0.54265	211.7 ± 1588.8	0.41	0.76	0.0169 ± 0.0003
		Σ	12.902038	0.128	2346.6966	0.066	6.9005220	1.910	322.79390	0.042	3666.8580	0.065					

Information on Analysis and Constants Used in Calculations

Project = **O-CONNOR (16-22)**
Sample = **HLY0102-D90-18**
Material = **Groundmass**
Location = **Gakkel Ridge**
Region = **Artic Ocean**
Analyst = **Dan Miggins**
Irradiation = **17-OSU-05 (5A14-17)**
Position = **X: 0 | Y: 0 | Z/H: 20.1194 mm**
FCT-NM Age = **28.201 ± 0.023 Ma**
FCT-NM Reference = **Kuiper et al (2008)**
FCT-NM 40Ar/39Ar Ratio = **9.70619 ± 0.01116**
FCT-NM J-value = **0.00161932 ± 0.00000186**
Air Shot 40Ar/36Ar = **302.6970 ± 0.4268**
Air Shot MDF = **0.99404825 ± 0.00067391 (LIN)**
Experiment Type = **Incremental Heating**
Extraction Method = **Bulk Laser Heating**
Heating = **64 sec**
Isolation = **3.00 min**
Instrument = **ARGUS-VI-D**
Preferred Age = **Plateau Age**
Age Classification = **Crystallization Age**
IGSN = Undefined
Rock Class = **Igneous>Volcanic**
Lithology = **Basaltic Lava**
Lat-Lon = Undefined - Undefined

Age Equations = **Min et al. (2000)**
Negative Intensities = **Allowed**
Collector Calibrations = **36Ar**
Decay 40K = **5.530 ± 0.048 E-10 1/a**
Decay 39Ar = **2.940 ± 0.016 E-07 1/h**
Decay 37Ar = **8.230 ± 0.012 E-04 1/h**
Decay 36Cl = **2.257 ± 0.015 E-06 1/a**
Decay 40K(EC,β⁺) = **0.580 ± 0.009 E-10 1/a**
Decay 40K(β⁻) = **4.950 ± 0.043 E-10 1/a**
Atmospheric 40/36(a) = **297.96 ± 0.70**
Atmospheric 38/36(a) = **0.1869**
Production 39/37(ca) = **0.0006425 ± 0.0000059**
Production 38/37(ca) = **0.0001800 ± 0.0000173**
Production 36/37(ca) = **0.0002703 ± 0.0000005**
Production 40/39(k) = **0.000607 ± 0.000059**
Production 38/39(k) = **0.012077 ± 0.000011**
Production 36/38(cl) = **262.80 ± 1.71**
Scaling Ratio K/Ca = **0.430**
Abundance Ratio 40K/K = **1.1700 ± 0.0100 E-04**
Atomic Weight K = **39.0983 ± 0.0001 g**

Results

	40(a)/36(a) ± 2σ		40(r)/39(k) ± 2σ		Age ± 2σ (ka)		MSWD	39Ar(k) (%,n)	K/Ca ± 2σ
**Age Plateau**			0.05000	± 0.02807	**146.4**	**± 82.2**	0.29	100.00	0.0308 ± 0.0068
Overestimated Error				± 56.14%		**± 56.14%**	100%	30	
					Full External Error	± 82.2	1.53	2σ Confidence Limit	
					Analytical Error	± 82.2	1.0000	Error Magnification	
**Total Fusion Age**			0.03544	± 0.03995	**103.8**	**± 117.0**		30	0.0589 ± 0.0001
				± 112.72%		**± 112.72%**			
					Full External Error	± 117.0			
					Analytical Error	± 117.0			
**Normal Isochron**	**297.95**	**± 1.40**	0.05005	± 0.04505	**146.6**	**± 131.9**	0.37	100.00	
		**± 0.47%**		± 90.01%		**± 90.00%**	100%	30	
					Full External Error	± 132.0	1.53	2σ Confidence Limit	
					Analytical Error	± 131.9	1.0000	Error Magnification	
							3	Number of Iterations	
							0.0000000765	Convergence	
**Inverse Isochron**	**297.96**	**± 1.40**	0.05010	± 0.02563	**146.7**	**± 75.1**	0.37	100.00	
Clustered Points		**± 0.47%**		± 51.16%		**± 51.16%**	100%	30	
					Full External Error	± 75.1	1.53	2σ Confidence Limit	
					Analytical Error	± 75.1	1.0000	Error Magnification	
							3	Number of Iterations	
							0.0001125628	Convergence	
							1%	Spreading Factor	

Notes
Excess Initial 40Ar/36Ar = 297.96 ± 0.24 (%SD).

Incremental Heating			36Ar(a) [fA]	37Ar(ca) [fA]	38Ar(cl) [fA]	39Ar(k) [fA]	40Ar(r) [fA]	Age ± 2σ (ka)	40Ar(r) (%)	39Ar(k) (%)	K/Ca ± 2σ
17D24042	1.8 %	✓	4.185534	35.4660	0.1446331	44.27569	3.840177	254.0 ± 614.8	0.31	13.78	0.5368 ± 0.0042
17D24044	1.9 %	✓	1.820530	25.1896	0.0713097	30.31954	1.546302	149.3 ± 415.9	0.28	9.44	0.5176 ± 0.0050
17D24045	2.0 %	✓	1.033803	21.9887	0.0374312	20.09946	1.693575	246.7 ± 399.3	0.55	6.26	0.3931 ± 0.0040
17D24046	2.1 %	✓	0.571157	13.7083	0.0194317	11.33427	1.714470	442.9 ± 488.6	1.00	3.53	0.3555 ± 0.0055
17D24048	2.2 %	✓	0.390945	13.6378	0.0385316	9.09507	1.445927	465.5 ± 517.9	1.23	2.83	0.2868 ± 0.0045
17D24049	2.3 %	✓	0.376160	17.0272	0.0209042	8.73515	0.900974	302.0 ± 533.8	0.80	2.72	0.2206 ± 0.0029
17D24050	2.4 %	✓	0.265384	15.4766	0.0132585	6.92757	0.435097	183.9 ± 602.6	0.55	2.16	0.1925 ± 0.0029
17D24052	2.5 %	✓	0.222126	17.2821	0.0000000	6.48371	0.509336	230.0 ± 635.6	0.76	2.02	0.1613 ± 0.0023
17D24053	2.7 %	✓	0.207466	20.6913	0.0000000	6.50944	0.355575	159.9 ± 623.1	0.57	2.03	0.1353 ± 0.0017
17D24054	3.0 %	✓	0.165106	17.7367	0.0099839	5.12850	0.328803	187.7 ± 761.9	0.66	1.60	0.1243 ± 0.0019
17D24056	3.4 %	✓	0.210194	34.1315	0.0099393	7.75439	0.711016	268.5 ± 521.4	1.12	2.41	0.0977 ± 0.0009
17D24057	3.8 %	✓	0.208462	47.6390	0.0000000	8.92758	0.161902	53.1 ± 455.9	0.26	2.78	0.0806 ± 0.0007
17D24058	4.2 %	✓	0.236729	66.4635	0.0400724	10.70255	0.906463	248.0 ± 384.6	1.27	3.33	0.0692 ± 0.0005
17D24060	4.6 %	✓	0.185290	68.1797	0.0000000	9.80271	0.205237	61.3 ± 405.8	0.37	3.05	0.0618 ± 0.0005
17D24061	5.2 %	✓	0.171407	75.6335	0.0215949	9.69093	0.025549	7.7 ± 404.8	0.05	3.02	0.0551 ± 0.0004
17D24062	5.8 %	✓	0.239188	139.1757	0.0040532	14.77167	0.785063	155.6 ± 287.5	1.09	4.60	0.0456 ± 0.0003
17D24064	6.5 %	✓	0.183915	145.0965	0.0000000	13.42256	0.436429	95.2 ± 295.9	0.79	4.18	0.0398 ± 0.0003
17D24065	7.2 %	✓	0.171829	167.7047	0.0000000	13.64828	0.774709	166.2 ± 293.0	1.49	4.25	0.0350 ± 0.0002
17D24066	8.0 %	✓	0.144519	186.2898	0.0169980	13.65546	0.581268	124.6 ± 288.6	1.33	4.25	0.0315 ± 0.0002
17D24068	8.9 %	✓	0.138340	196.1508	0.0000000	13.37020	0.774307	169.6 ± 295.2	1.84	4.16	0.0293 ± 0.0002
17D24069	9.7 %	✓	0.117131	165.4503	0.0000000	10.44210	0.436724	122.5 ± 368.5	1.24	3.25	0.0271 ± 0.0002
17D24070	10.6 %	✓	0.111292	150.6364	0.0000000	9.09692	0.157399	50.7 ± 423.2	0.47	2.83	0.0260 ± 0.0002
17D24072	11.6 %	✓	0.105957	139.3070	0.0048248	7.94844	0.140232	51.7 ± 480.1	0.44	2.47	0.0245 ± 0.0002
17D24073	12.5 %	✓	0.087057	107.9424	0.0000000	5.82896	0.262312	131.8 ± 644.4	1.00	1.81	0.0232 ± 0.0002
17D24074	13.4 %	✓	0.084060	93.0036	0.0000000	4.96952	0.187257	110.3 ± 755.5	0.74	1.55	0.0230 ± 0.0003
17D24076	14.6 %	✓	0.104252	87.5836	0.0026539	4.63815	0.027734	17.5 ± 817.4	0.09	1.44	0.0228 ± 0.0003
17D24077	16.0 %	✓	0.099295	78.6142	0.0216803	4.39045	0.074918	50.0 ± 857.8	0.25	1.37	0.0240 ± 0.0003
17D24078	17.6 %	✓	0.158391	80.1830	0.0000000	4.16563	0.071942	50.6 ± 938.9	0.15	1.30	0.0223 ± 0.0003
17D24080	18.6 %	✓	0.127717	57.3601	0.0114968	2.72050	0.455710	490.6 ± 1402.1	1.21	0.85	0.0204 ± 0.0004
17D24082	20.0 %	✓	0.144437	61.9469	0.0005362	2.43074	0.175760	211.7 ± 1588.8	0.41	0.76	0.0169 ± 0.0003
		Σ	12.267675	2346.6966	0.4893337	321.28614	11.386506				

Information on Analysis

Project = **O-CONNOR (16-22)**
Sample = **HLY0102-D90-18**
Material = **Groundmass**
Location = **Gakkel Ridge**
Region = **Artic Ocean**
Analyst = **Dan Miggins**
Irradiation = **17-OSU-05 (5A14-17)**
J = **0.00161932 ± 0.00000186**
FCT-NM = **28.201 ± 0.023 Ma**

Results	40(r)/39(k) ± 2σ		Age ± 2σ (ka)		MSWD	39Ar(k) (%,n)	K/Ca ± 2σ
**Age Plateau** Overestimated Error	0.05000	± 0.02807 ± 56.14%	**146.4**	**± 82.2 ± 56.14%**	0.29 100%	100.00 30	0.0308 ± 0.0068
		Full External Error	± 82.2		1.53	2σ Confidence Limit	
		Analytical Error	± 82.2		1.0000	Error Magnification	
**Total Fusion Age**	0.03544	± 0.03995 ± 112.72%	**103.8**	**± 117.0 ± 112.72%**		30	0.0589 ± 0.0001
		Full External Error	± 117.0				
		Analytical Error	± 117.0				

Normal Isochron			39(k)/36(a) ± 2σ	40(a+r)/36(a) ± 2σ	r.i.
17D24042	1.8 %	✓	10.58 ± 0.06	297.04 ± 1.72	0.9489
17D24044	1.9 %	✓	16.65 ± 0.11	298.81 ± 1.91	0.9198
17D24045	2.0 %	✓	19.44 ± 0.15	299.60 ± 2.26	0.8629
17D24046	2.1 %	✓	19.84 ± 0.19	300.96 ± 3.02	0.7784
17D24048	2.2 %	✓	23.26 ± 0.28	301.66 ± 3.90	0.7319
17D24049	2.3 %	✓	23.22 ± 0.28	300.36 ± 4.02	0.7351
17D24050	2.4 %	✓	26.10 ± 0.39	299.60 ± 5.20	0.6880
17D24052	2.5 %	✓	29.19 ± 0.51	300.25 ± 6.21	0.7016
17D24053	2.7 %	✓	31.38 ± 0.57	299.67 ± 6.55	0.7088
17D24054	3.0 %	✓	31.06 ± 0.68	299.95 ± 7.99	0.6863
17D24056	3.4 %	✓	36.89 ± 0.64	301.34 ± 6.46	0.7212
17D24057	3.8 %	✓	42.83 ± 0.75	298.74 ± 6.53	0.7333
17D24058	4.2 %	✓	45.21 ± 0.70	301.79 ± 5.82	0.7401
17D24060	4.6 %	✓	52.90 ± 1.00	299.07 ± 7.21	0.7297
17D24061	5.2 %	✓	56.54 ± 1.13	298.11 ± 7.69	0.7259
17D24062	5.8 %	✓	61.76 ± 0.99	301.24 ± 5.94	0.7680
17D24064	6.5 %	✓	72.98 ± 1.37	300.33 ± 7.27	0.7412
17D24065	7.2 %	✓	79.43 ± 1.61	302.47 ± 7.89	0.7513
17D24066	8.0 %	✓	94.49 ± 2.23	301.98 ± 9.28	0.7485
17D24068	8.9 %	✓	96.65 ± 2.39	303.56 ± 9.75	0.7531
17D24069	9.7 %	✓	89.15 ± 2.51	301.69 ± 11.21	0.7359
17D24070	10.6 %	✓	81.74 ± 2.43	299.37 ± 11.76	0.7324
17D24072	11.6 %	✓	75.02 ± 2.31	299.28 ± 12.25	0.7253
17D24073	12.5 %	✓	66.96 ± 2.45	300.97 ± 14.75	0.7170
17D24074	13.4 %	✓	59.12 ± 2.27	300.19 ± 15.25	0.7086
17D24076	14.6 %	✓	44.49 ± 1.43	298.23 ± 12.35	0.6963
17D24077	16.0 %	✓	44.22 ± 1.47	298.71 ± 12.90	0.6945
17D24078	17.6 %	✓	26.30 ± 0.63	297.51 ± 8.31	0.6558
17D24080	18.6 %	✓	21.30 ± 0.64	294.39 ± 10.03	0.6101
17D24082	20.0 %	✓	16.83 ± 0.49	299.18 ± 9.04	0.5771

Results	40(a)/36(a) ± 2σ		40(r)/39(k) ± 2σ		Age ± 2σ (ka)		MSWD
**Normal Isochron**	**297.95**	**± 1.40**<br>**± 0.47%**	0.05005	± 0.04505<br>± 90.01%	**146.6**	**± 131.9**<br>**± 90.00%**	0.37<br>100%
					Full External Error	± 132.0	
					Analytical Error	± 131.9	

Statistics				
	2σ Confidence Limit	1.53	Convergence	0.000000076543
	Error Magnification	1.0000	Number of Iterations	3
	Number of Data Points	30	Calculated Line	Weighted York-2

Inverse Isochron			39(k)/40(a+r) ± 2σ	36(a)/40(a+r) ± 2σ	r.i.
17D24042	1.8 %	✓	0.0356120 ± 0.0000675	0.00336652 ± 0.00001947	0.0458
17D24044	1.9 %	✓	0.0557354 ± 0.0001443	0.00334662 ± 0.00002141	0.1567
17D24045	2.0 %	✓	0.0648945 ± 0.0002562	0.00333780 ± 0.00002520	0.2678
17D24046	2.1 %	✓	0.0659366 ± 0.0004319	0.00332268 ± 0.00003338	0.3936
17D24048	2.2 %	✓	0.0771213 ± 0.0007045	0.00331501 ± 0.00004288	0.4654
17D24049	2.3 %	✓	0.0773148 ± 0.0007226	0.00332939 ± 0.00004453	0.4793
17D24050	2.4 %	✓	0.0871296 ± 0.0011325	0.00333779 ± 0.00005797	0.5354
17D24052	2.5 %	✓	0.0972157 ± 0.0014641	0.00333052 ± 0.00006887	0.5516
17D24053	2.7 %	✓	0.1047000 ± 0.0016370	0.00333696 ± 0.00007295	0.5783
17D24054	3.0 %	✓	0.1035560 ± 0.0020384	0.00333387 ± 0.00008881	0.5944
17D24056	3.4 %	✓	0.1224240 ± 0.0018339	0.00331848 ± 0.00007115	0.5931
17D24057	3.8 %	✓	0.1433568 ± 0.0021408	0.00334743 ± 0.00007316	0.6035
17D24058	4.2 %	✓	0.1498071 ± 0.0019510	0.00331357 ± 0.00006386	0.5965
17D24060	4.6 %	✓	0.1768989 ± 0.0029260	0.00334373 ± 0.00008063	0.6239
17D24061	5.2 %	✓	0.1896543 ± 0.0033732	0.00335448 ± 0.00008656	0.6378
17D24062	5.8 %	✓	0.2050099 ± 0.0025943	0.00331959 ± 0.00006545	0.5901
17D24064	6.5 %	✓	0.2430044 ± 0.0039561	0.00332964 ± 0.00008065	0.6352
17D24065	7.2 %	✓	0.2626039 ± 0.0045260	0.00330613 ± 0.00008627	0.6288
17D24066	8.0 %	✓	0.3128952 ± 0.0063779	0.00331145 ± 0.00010175	0.6403
17D24068	8.9 %	✓	0.3183826 ± 0.0067283	0.00329427 ± 0.00010578	0.6382
17D24069	9.7 %	✓	0.2955008 ± 0.0074373	0.00331468 ± 0.00012315	0.6543
17D24070	10.6 %	✓	0.2730333 ± 0.0073072	0.00334030 ± 0.00013122	0.6544
17D24072	11.6 %	✓	0.2506517 ± 0.0070684	0.00334131 ± 0.00013675	0.6583
17D24073	12.5 %	✓	0.2224639 ± 0.0076057	0.00332256 ± 0.00016280	0.6644
17D24074	13.4 %	✓	0.1969387 ± 0.0070756	0.00333125 ± 0.00016924	0.6574
17D24076	14.6 %	✓	0.1491813 ± 0.0044588	0.00335316 ± 0.00013882	0.6386
17D24077	16.0 %	✓	0.1480219 ± 0.0046219	0.00334768 ± 0.00014452	0.6441
17D24078	17.6 %	✓	0.0884002 ± 0.0019259	0.00336128 ± 0.00009387	0.5655
17D24080	18.6 %	✓	0.0723556 ± 0.0020615	0.00339683 ± 0.00011578	0.5568
17D24082	20.0 %	✓	0.0562512 ± 0.0015321	0.00334250 ± 0.00010105	0.4967

Results	40(a)/36(a)	± 2σ	40(r)/39(k)	± 2σ	Age (ka)	± 2σ	MSWD
**Inverse Isochron** Clustered Points	**297.96**	**± 1.40**<br>**± 0.47%**	0.05010	± 0.02563<br>± 51.16%	**146.7**	**± 75.1**<br>**± 51.16%**	0.37<br>100%
					Full External Error	± 75.1	
					Analytical Error	± 75.1	

Statistics				
	2σ Confidence Limit	1.53	Convergence	0.0001125628
	Error Magnification	1.0000	Number of Iterations	3
	Number of Data Points	30	Calculated Line	Weighted York-2
	Spreading Factor	1.4%		

Degassing Patterns			36Ar(a) [fA]	%1σ	36Ar(c) [fA]	%1σ	36Ar(ca) [fA]	%1σ	36Ar(cl) [fA]	%1σ	37Ar(ca) [fA]	%1σ	38Ar(a) [fA]	%1σ	38Ar(c) [fA]	%1σ
17D24042	1.8 %	✓	4.185534	0.29	0.0000000	0.00	0.0095865	0.42	0.0000150	17.03	35.4660	0.39	0.7822764	0.29	0.0000000	0.00
17D24044	1.9 %	✓	1.820530	0.31	0.0000000	0.00	0.0068088	0.50	0.0000074	33.81	25.1896	0.47	0.3402570	0.31	0.0000000	0.00
17D24045	2.0 %	✓	1.033803	0.35	0.0000000	0.00	0.0059435	0.52	0.0000039	61.72	21.9887	0.49	0.1932177	0.35	0.0000000	0.00
17D24046	2.1 %	✓	0.571157	0.43	0.0000000	0.00	0.0037054	0.76	0.0000020	126.49	13.7083	0.74	0.1067493	0.43	0.0000000	0.00
17D24048	2.2 %	✓	0.390945	0.53	0.0000000	0.00	0.0036863	0.76	0.0000040	62.07	13.6378	0.74	0.0730677	0.53	0.0000000	0.00
17D24049	2.3 %	✓	0.376160	0.55	0.0000000	0.00	0.0046025	0.63	0.0000022	111.50	17.0272	0.61	0.0703043	0.55	0.0000000	0.00
17D24050	2.4 %	✓	0.265384	0.67	0.0000000	0.00	0.0041833	0.69	0.0000014	183.41	15.4766	0.67	0.0496002	0.67	0.0000000	0.00
17D24052	2.5 %	✓	0.222126	0.80	0.0000000	0.00	0.0046714	0.64	0.0000000	0.00	17.2821	0.62	0.0415154	0.80	0.0000000	0.00
17D24053	2.7 %	✓	0.207466	0.84	0.0000000	0.00	0.0055929	0.55	0.0000000	0.00	20.6913	0.52	0.0387755	0.84	0.0000000	0.00
17D24054	3.0 %	✓	0.165106	1.00	0.0000000	0.00	0.0047942	0.63	0.0000010	244.51	17.7367	0.61	0.0308584	1.00	0.0000000	0.00
17D24056	3.4 %	✓	0.210194	0.82	0.0000000	0.00	0.0092257	0.42	0.0000010	233.29	34.1315	0.38	0.0392852	0.82	0.0000000	0.00
17D24057	3.8 %	✓	0.208462	0.84	0.0000000	0.00	0.0128768	0.38	0.0000000	0.00	47.6390	0.34	0.0389615	0.84	0.0000000	0.00
17D24058	4.2 %	✓	0.236729	0.74	0.0000000	0.00	0.0179651	0.35	0.0000042	61.64	66.4635	0.31	0.0442446	0.74	0.0000000	0.00
17D24060	4.6 %	✓	0.185290	0.91	0.0000000	0.00	0.0184290	0.35	0.0000000	0.00	68.1797	0.31	0.0346307	0.91	0.0000000	0.00
17D24061	5.2 %	✓	0.171407	0.97	0.0000000	0.00	0.0204437	0.35	0.0000022	108.36	75.6335	0.30	0.0320359	0.97	0.0000000	0.00
17D24062	5.8 %	✓	0.239188	0.78	0.0000000	0.00	0.0376192	0.33	0.0000004	567.90	139.1757	0.28	0.0447042	0.78	0.0000000	0.00
17D24064	6.5 %	✓	0.183915	0.92	0.0000000	0.00	0.0392196	0.33	0.0000000	0.00	145.0965	0.28	0.0343738	0.92	0.0000000	0.00
17D24065	7.2 %	✓	0.171829	1.00	0.0000000	0.00	0.0453306	0.33	0.0000000	0.00	167.7047	0.28	0.0321148	1.00	0.0000000	0.00
17D24066	8.0 %	✓	0.144519	1.17	0.0000000	0.00	0.0503541	0.33	0.0000018	140.21	186.2898	0.28	0.0270107	1.17	0.0000000	0.00
17D24068	8.9 %	✓	0.138340	1.22	0.0000000	0.00	0.0530196	0.33	0.0000000	0.00	196.1508	0.28	0.0258558	1.22	0.0000000	0.00
17D24069	9.7 %	✓	0.117131	1.39	0.0000000	0.00	0.0447212	0.33	0.0000000	0.00	165.4503	0.28	0.0218917	1.39	0.0000000	0.00
17D24070	10.6 %	✓	0.111292	1.46	0.0000000	0.00	0.0407170	0.33	0.0000000	0.00	150.6364	0.28	0.0208005	1.46	0.0000000	0.00
17D24072	11.6 %	✓	0.105957	1.51	0.0000000	0.00	0.0376547	0.33	0.0000005	505.23	139.3070	0.28	0.0198033	1.51	0.0000000	0.00
17D24073	12.5 %	✓	0.087057	1.79	0.0000000	0.00	0.0291768	0.33	0.0000000	0.00	107.9424	0.29	0.0162710	1.79	0.0000000	0.00
17D24074	13.4 %	✓	0.084060	1.86	0.0000000	0.00	0.0251389	0.34	0.0000000	0.00	93.0036	0.29	0.0157109	1.86	0.0000000	0.00
17D24076	14.6 %	✓	0.104252	1.52	0.0000000	0.00	0.0236739	0.34	0.0000003	925.06	87.5836	0.30	0.0194847	1.52	0.0000000	0.00
17D24077	16.0 %	✓	0.099295	1.58	0.0000000	0.00	0.0212494	0.34	0.0000023	109.85	78.6142	0.30	0.0185582	1.58	0.0000000	0.00
17D24078	17.6 %	✓	0.158391	1.04	0.0000000	0.00	0.0216735	0.34	0.0000000	0.00	80.1830	0.30	0.0296034	1.04	0.0000000	0.00
17D24080	18.6 %	✓	0.127717	1.25	0.0000000	0.00	0.0155044	0.36	0.0000012	212.30	57.3601	0.32	0.0238704	1.25	0.0000000	0.00
17D24082	20.0 %	✓	0.144437	1.12	0.0000000	0.00	0.0167442	0.36	0.0000001	######	61.9469	0.31	0.0269954	1.12	0.0000000	0.00
		Σ	12.267675	0.13	0.0000000	0.00	0.6343121	0.08	0.0000506	20.81	2346.6966	0.07	2.2928284	0.13	0.0000000	0.00
		Σ							12.902038	0.13	2346.6966	0.07				

Degassing Patterns	38Ar(k) [fA]	%1σ	38Ar(ca) [fA]	%1σ	38Ar(cl) [fA]	%1σ	39Ar(k) [fA]	%1σ	39Ar(ca) [fA]	%1σ	40Ar(r) [fA]	%1σ	40Ar(a) [fA]	%1σ	40Ar(c) [fA]	%1σ	40Ar(k) [fA]	%1σ
17D24042	0.5347175	0.13	0.0063839	9.64	0.1446331	17.06	44.27569	0.09	0.0227869	1.00	3.840177	121.01	1247.1219	0.37	0.0000000	0.00	0.0268753	9.65
17D24044	0.3661691	0.14	0.0045341	9.64	0.0713097	33.83	30.31954	0.10	0.0161843	1.03	1.546302	139.26	542.4450	0.39	0.0000000	0.00	0.0184040	9.65
17D24045	0.2427412	0.16	0.0039580	9.64	0.0374312	61.72	20.09946	0.14	0.0141277	1.04	1.693575	80.92	308.0318	0.42	0.0000000	0.00	0.0122004	9.65
17D24046	0.1368840	0.22	0.0024675	9.66	0.0194317	126.49	11.33427	0.21	0.0088076	1.18	1.714470	55.16	170.1820	0.49	0.0000000	0.00	0.0068799	9.65
17D24048	0.1098412	0.28	0.0024548	9.66	0.0385316	62.08	9.09507	0.27	0.0087623	1.18	1.445927	55.64	116.4861	0.58	0.0000000	0.00	0.0055207	9.65
17D24049	0.1054944	0.28	0.0030649	9.65	0.0209042	111.50	8.73515	0.26	0.0109400	1.10	0.900974	88.38	112.0806	0.59	0.0000000	0.00	0.0053022	9.65
17D24050	0.0836643	0.36	0.0027858	9.65	0.0132585	183.41	6.92757	0.35	0.0099437	1.14	0.435097	163.83	79.0737	0.71	0.0000000	0.00	0.0042050	9.66
17D24052	0.0783037	0.38	0.0031108	9.65	0.0000000	0.00	6.48371	0.37	0.0111038	1.11	0.509336	138.18	66.1847	0.83	0.0000000	0.00	0.0039356	9.66
17D24053	0.0786145	0.35	0.0037244	9.64	0.0000000	0.00	6.50944	0.34	0.0132941	1.06	0.355575	194.80	61.8167	0.87	0.0000000	0.00	0.0039512	9.66
17D24054	0.0619369	0.44	0.0031926	9.65	0.0099839	244.52	5.12850	0.44	0.0113959	1.10	0.328803	202.93	49.1951	1.02	0.0000000	0.00	0.0031130	9.66
17D24056	0.0936497	0.30	0.0061437	9.64	0.0099393	233.29	7.75439	0.29	0.0219295	1.00	0.711016	97.11	62.6294	0.85	0.0000000	0.00	0.0047069	9.65
17D24057	0.1078183	0.27	0.0085750	9.64	0.0000000	0.00	8.92758	0.26	0.0306080	0.98	0.161902	429.29	62.1133	0.87	0.0000000	0.00	0.0054190	9.65
17D24058	0.1292547	0.24	0.0119634	9.63	0.0400724	61.65	10.70255	0.22	0.0427028	0.97	0.906463	77.56	70.5357	0.78	0.0000000	0.00	0.0064964	9.65
17D24060	0.1183873	0.26	0.0122724	9.63	0.0000000	0.00	9.80271	0.25	0.0438055	0.97	0.205237	330.93	55.2090	0.94	0.0000000	0.00	0.0059502	9.65
17D24061	0.1170374	0.26	0.0136140	9.63	0.0215949	108.37	9.69093	0.24	0.0485946	0.97	0.025549	######	51.0723	0.99	0.0000000	0.00	0.0058824	9.65
17D24062	0.1783975	0.20	0.0250516	9.63	0.0040532	567.90	14.77167	0.18	0.0894204	0.96	0.785063	92.37	71.2684	0.81	0.0000000	0.00	0.0089664	9.65
17D24064	0.1621043	0.21	0.0261174	9.63	0.0000000	0.00	13.42256	0.19	0.0932245	0.96	0.436429	155.42	54.7994	0.95	0.0000000	0.00	0.0081475	9.65
17D24065	0.1648303	0.21	0.0301868	9.63	0.0000000	0.00	13.64828	0.19	0.1077502	0.96	0.774709	88.14	51.1982	1.02	0.0000000	0.00	0.0082845	9.65
17D24066	0.1649169	0.21	0.0335322	9.63	0.0169980	140.22	13.65546	0.19	0.1196912	0.96	0.581268	115.78	43.0610	1.19	0.0000000	0.00	0.0082889	9.65
17D24068	0.1614719	0.20	0.0353071	9.63	0.0000000	0.00	13.37020	0.18	0.1260269	0.96	0.774307	87.05	41.2198	1.25	0.0000000	0.00	0.0081157	9.65
17D24069	0.1261092	0.25	0.0297810	9.63	0.0000000	0.00	10.44210	0.23	0.1063018	0.96	0.436724	150.45	34.9002	1.41	0.0000000	0.00	0.0063384	9.65
17D24070	0.1098635	0.28	0.0271146	9.63	0.0000000	0.00	9.09692	0.27	0.0967839	0.96	0.157399	417.62	33.1606	1.48	0.0000000	0.00	0.0055218	9.65
17D24072	0.0959933	0.31	0.0250753	9.63	0.0048248	505.23	7.94844	0.30	0.0895047	0.96	0.140232	464.64	31.5709	1.53	0.0000000	0.00	0.0048247	9.65
17D24073	0.0703963	0.38	0.0194296	9.63	0.0000000	0.00	5.82896	0.37	0.0693530	0.96	0.262312	244.51	25.9395	1.81	0.0000000	0.00	0.0035382	9.66
17D24074	0.0600169	0.49	0.0167406	9.63	0.0000000	0.00	4.96952	0.48	0.0597548	0.97	0.187257	342.39	25.0466	1.87	0.0000000	0.00	0.0030165	9.66
17D24076	0.0560149	0.52	0.0157651	9.63	0.0026539	925.06	4.63815	0.51	0.0562725	0.97	0.027734	######	31.0629	1.54	0.0000000	0.00	0.0028154	9.66
17D24077	0.0530235	0.52	0.0141506	9.63	0.0216803	109.85	4.39045	0.52	0.0505096	0.97	0.074918	858.44	29.5859	1.59	0.0000000	0.00	0.0026650	9.66
17D24078	0.0503083	0.58	0.0144329	9.63	0.0000000	0.00	4.16563	0.57	0.0515176	0.97	0.071942	928.27	47.1943	1.07	0.0000000	0.00	0.0025285	9.67
17D24080	0.0328555	0.83	0.0103248	9.64	0.0114968	212.30	2.72050	0.82	0.0368539	0.97	0.455710	142.88	38.0547	1.27	0.0000000	0.00	0.0016513	9.69
17D24082	0.0293561	0.92	0.0111504	9.64	0.0005362	######	2.43074	0.91	0.0398009	0.97	0.175760	375.24	43.0366	1.15	0.0000000	0.00	0.0014755	9.69
Σ	3.8801728	0.05	0.4224054	2.18	0.4893337	20.79	321.28614	0.04	1.5077526	0.22	11.386506	56.36	3655.2764	0.16	0.0000000	0.00	0.1950207	2.23
Σ					7.0847403	1.44			322.79390	0.04							3666.8580	0.24

Additional Parameters			40Ar/39Ar	1σ	37Ar/39Ar	1σ	36Ar/39Ar	1σ	Time (days)	37Ar (decay)	39Ar (decay)	40Ar (moles)
17D24042	1.8 %	✓	28.066621	0.026596	0.800615	0.003166	0.094702	0.000284	63.653	3.550835	1.00045279	5.968E-11
17D24044	1.9 %	✓	17.932969	0.023200	0.830361	0.003994	0.060237	0.000196	63.671	3.552102	1.00045292	2.611E-11
17D24045	2.0 %	✓	15.399417	0.030385	1.093225	0.005587	0.051694	0.000194	63.680	3.552735	1.00045298	1.487E-11
17D24046	2.1 %	✓	15.154918	0.049621	1.208517	0.009335	0.050680	0.000242	63.689	3.553369	1.00045305	8.251E-12
17D24048	2.2 %	✓	12.954713	0.059145	1.498024	0.011731	0.043348	0.000255	63.707	3.554636	1.00045317	5.661E-12
17D24049	2.3 %	✓	12.918560	0.060347	1.946835	0.012922	0.043535	0.000261	63.716	3.555270	1.00045324	5.423E-12
17D24050	2.4 %	✓	11.461307	0.074455	2.230857	0.016811	0.038857	0.000290	63.725	3.555904	1.00045330	3.817E-12
17D24052	2.5 %	✓	10.269428	0.077296	2.660914	0.019112	0.034920	0.000302	63.743	3.557173	1.00045343	3.202E-12
17D24053	2.7 %	✓	9.532237	0.074484	3.172179	0.019730	0.032664	0.000289	63.752	3.557807	1.00045349	2.984E-12
17D24054	3.0 %	✓	9.635804	0.094788	3.450798	0.025870	0.033055	0.000351	63.761	3.558441	1.00045356	2.377E-12
17D24056	3.4 %	✓	8.145902	0.060984	4.389155	0.020981	0.028217	0.000236	63.779	3.559711	1.00045368	3.041E-12
17D24057	3.8 %	✓	6.952371	0.051887	5.317927	0.022577	0.024708	0.000205	63.788	3.560346	1.00045375	2.989E-12
17D24058	4.2 %	✓	6.649326	0.043273	6.185379	0.023460	0.023703	0.000172	63.797	3.560981	1.00045381	3.430E-12
17D24060	4.6 %	✓	5.628403	0.046525	6.924251	0.027454	0.020689	0.000179	63.815	3.562251	1.00045394	2.660E-12
17D24061	5.2 %	✓	5.247048	0.046639	7.765627	0.030140	0.019698	0.000176	63.824	3.562886	1.00045400	2.453E-12
17D24062	5.8 %	✓	4.849067	0.030661	9.365108	0.031200	0.018626	0.000129	63.833	3.563521	1.00045407	3.459E-12
17D24064	6.5 %	✓	4.087371	0.033252	10.735339	0.036342	0.016509	0.000128	63.851	3.564792	1.00045419	2.652E-12
17D24065	7.2 %	✓	3.778791	0.032545	12.191358	0.041028	0.015786	0.000128	63.860	3.565428	1.00045426	2.495E-12
17D24066	8.0 %	✓	3.168791	0.032279	13.523619	0.045364	0.014147	0.000125	63.869	3.566064	1.00045432	2.095E-12
17D24068	8.9 %	✓	3.112147	0.032868	14.533744	0.048333	0.014179	0.000127	63.888	3.567336	1.00045445	2.016E-12
17D24069	9.7 %	✓	3.350584	0.042142	15.684870	0.056759	0.015344	0.000157	63.897	3.567972	1.00045451	1.696E-12
17D24070	10.6 %	✓	3.624601	0.048474	16.384736	0.062986	0.016534	0.000182	63.906	3.568608	1.00045458	1.600E-12
17D24072	11.6 %	✓	3.945775	0.055598	17.331170	0.070587	0.017867	0.000206	63.924	3.569881	1.00045470	1.522E-12
17D24073	12.5 %	✓	4.442857	0.075893	18.300558	0.085600	0.019706	0.000274	63.933	3.570518	1.00045477	1.258E-12
17D24074	13.4 %	✓	5.017990	0.090056	18.492439	0.102434	0.021713	0.000327	63.942	3.571155	1.00045483	1.211E-12
17D24076	14.6 %	✓	6.623501	0.098836	18.656965	0.108598	0.027251	0.000364	63.960	3.572429	1.00045496	1.492E-12
17D24077	16.0 %	✓	6.679518	0.104143	17.702067	0.104703	0.027144	0.000379	63.969	3.573066	1.00045502	1.424E-12
17D24078	17.6 %	✓	11.174602	0.121306	19.013580	0.121497	0.042698	0.000460	63.978	3.573703	1.00045509	2.262E-12
17D24080	18.6 %	✓	13.636502	0.193384	20.802612	0.181668	0.051942	0.000715	63.996	3.574978	1.00045521	1.805E-12
17D24082	20.0 %	✓	17.491614	0.236467	25.074180	0.238269	0.065241	0.000880	64.014	3.576253	1.00045534	2.074E-12

Procedure Blanks		36Ar ± 1σ (SE) [fA]	37Ar ± 1σ (SE) [fA]	38Ar ± 1σ (SE) [fA]	39Ar ± 1σ (SE) [fA]	40Ar ± 1σ (SE) [fA]
17D24042	1.8 %	0.0133963 ± 0.0013716	0.1565079 ± 0.0183752	0.0495445 ± 0.0167311	0.0120129 ± 0.0158626	4.0866433 ± 0.4365870
17D24044	1.9 %	0.0118935 ± 0.0013716	0.1562969 ± 0.0183752	0.0444202 ± 0.0167311	0.0119376 ± 0.0158626	3.6254817 ± 0.4365870
17D24045	2.0 %	0.0112420 ± 0.0013716	0.1557800 ± 0.0183752	0.0424299 ± 0.0167311	0.0121495 ± 0.0158626	3.4219354 ± 0.4365870
17D24046	2.1 %	0.0106529 ± 0.0013716	0.1550342 ± 0.0183752	0.0407813 ± 0.0167311	0.0124844 ± 0.0158626	3.2356230 ± 0.4365870
17D24048	2.2 %	0.0096490 ± 0.0013716	0.1529855 ± 0.0183752	0.0383952 ± 0.0167311	0.0134090 ± 0.0158626	2.9122635 ± 0.4365870
17D24049	2.3 %	0.0092281 ± 0.0013716	0.1517441 ± 0.0183752	0.0376034 ± 0.0167311	0.0139482 ± 0.0158626	2.7739690 ± 0.4365870
17D24050	2.4 %	0.0088572 ± 0.0013716	0.1503969 ± 0.0183752	0.0370443 ± 0.0167311	0.0145096 ± 0.0158626	2.6504137 ± 0.4365870
17D24052	2.5 %	0.0082529 ± 0.0013716	0.1474951 ± 0.0183752	0.0365258 ± 0.0167311	0.0156274 ± 0.0158626	2.4448983 ± 0.4365870
17D24053	2.7 %	0.0080138 ± 0.0013716	0.1459923 ± 0.0183752	0.0365195 ± 0.0167311	0.0161547 ± 0.0158626	2.3615980 ± 0.4365870
17D24054	3.0 %	0.0078127 ± 0.0013716	0.1444872 ± 0.0183752	0.0366521 ± 0.0167311	0.0166460 ± 0.0158626	2.2903565 ± 0.4365870
17D24056	3.4 %	0.0075133 ± 0.0013716	0.1415607 ± 0.0183752	0.0372503 ± 0.0167311	0.0174921 ± 0.0158626	2.1812416 ± 0.4365870
17D24057	3.8 %	0.0074093 ± 0.0013716	0.1401813 ± 0.0183752	0.0376766 ± 0.0167311	0.0178391 ± 0.0158626	2.1419352 ± 0.4365870
17D24058	4.2 %	0.0073321 ± 0.0013716	0.1388836 ± 0.0183752	0.0381628 ± 0.0167311	0.0181348 ± 0.0158626	2.1118214 ± 0.4365870
17D24060	4.6 %	0.0072473 ± 0.0013716	0.1366043 ± 0.0183752	0.0392467 ± 0.0167311	0.0185860 ± 0.0158626	2.0761778 ± 0.4365870
17D24061	5.2 %	0.0072343 ± 0.0013716	0.1356549 ± 0.0183752	0.0398125 ± 0.0167311	0.0187553 ± 0.0158626	2.0691222 ± 0.4365870
17D24062	5.8 %	0.0072374 ± 0.0013716	0.1348518 ± 0.0183752	0.0403744 ± 0.0167311	0.0189006 ± 0.0158626	2.0682073 ± 0.4365870
17D24064	6.5 %	0.0072816 ± 0.0013716	0.1337352 ± 0.0183752	0.0414328 ± 0.0167311	0.0191758 ± 0.0158626	2.0816205 ± 0.4365870
17D24065	7.2 %	0.0073177 ± 0.0013716	0.1334443 ± 0.0183752	0.0419050 ± 0.0167311	0.0193408 ± 0.0158626	2.0943298 ± 0.4365870
17D24066	8.0 %	0.0073597 ± 0.0013716	0.1333445 ± 0.0183752	0.0423244 ± 0.0167311	0.0195519 ± 0.0158626	2.1099423 ± 0.4365870
17D24068	8.9 %	0.0074520 ± 0.0013716	0.1337499 ± 0.0183752	0.0429661 ± 0.0167311	0.0202116 ± 0.0158626	2.1465119 ± 0.4365870
17D24069	9.7 %	0.0074975 ± 0.0013716	0.1342678 ± 0.0183752	0.0431715 ± 0.0167311	0.0207167 ± 0.0158626	2.1657575 ± 0.4365870
17D24070	10.6 %	0.0075394 ± 0.0013716	0.1350021 ± 0.0183752	0.0432902 ± 0.0167311	0.0213806 ± 0.0158626	2.1844831 ± 0.4365870
17D24072	11.6 %	0.0076034 ± 0.0013716	0.1371322 ± 0.0183752	0.0432437 ± 0.0167311	0.0233272 ± 0.0158626	2.2168235 ± 0.4365870
17D24073	12.5 %	0.0076210 ± 0.0013716	0.1385309 ± 0.0183752	0.0430691 ± 0.0167311	0.0246876 ± 0.0158626	2.2286339 ± 0.4365870
17D24074	13.4 %	0.0076261 ± 0.0013716	0.1401518 ± 0.0183752	0.0427889 ± 0.0167311	0.0263623 ± 0.0158626	2.2363154 ± 0.4365870
17D24076	14.6 %	0.0075902 ± 0.0013716	0.1440531 ± 0.0183752	0.0419027 ± 0.0167311	0.0308399 ± 0.0158626	2.2355553 ± 0.4365870
17D24077	16.0 %	0.0075449 ± 0.0013716	0.1463264 ± 0.0183752	0.0412947 ± 0.0167311	0.0337418 ± 0.0158626	2.2252164 ± 0.4365870
17D24078	17.6 %	0.0074788 ± 0.0013716	0.1488083 ± 0.0183752	0.0405772 ± 0.0167311	0.0371563 ± 0.0158626	2.2069540 ± 0.4365870
17D24080	18.6 %	0.0072757 ± 0.0013716	0.1543707 ± 0.0183752	0.0388195 ± 0.0167311	0.0457509 ± 0.0158626	2.1427366 ± 0.4365870
17D24082	20.0 %	0.0069655 ± 0.0013716	0.1606734 ± 0.0183752	0.0366517 ± 0.0167311	0.0571054 ± 0.0158626	2.0349425 ± 0.4365870

Intercept Values		36Ar ± 1σ (SE) [fA]	r2	Regression (type,n)	37Ar ± 1σ (SE) [fA]	r2	Regression (type,n)	38Ar ± 1σ (SE) [fA]	r2	Regression (type,n)	39Ar ± 1σ (SE) [fA]	r2	Regression (type,n)	40Ar ± 1σ (SE) [fA]	r2	Regression (type,n)
17D24042	1.8 %	4.0431538 ± 0.0027870	0.9905	EXP 150 of 150	9.653497 ± 0.019472	0.8896	EXP 150 of 150	1.4009950 ± 0.0173509	0.1966	EXP 150 of 150	44.0033901 ± 0.0189075	0.9953	EXP 150 of 150	1247.395199 ± 0.060675	0.9998	EXP 150 of 150
17D24044	1.9 %	1.7672025 ± 0.0019986	0.9725	EXP 150 of 150	6.808737 ± 0.019357	0.7882	EXP 150 of 150	0.7285397 ± 0.0168711	0.0366	EXP 150 of 150	30.1299376 ± 0.0162367	0.9922	EXP 150 of 150	547.635158 ± 0.036857	0.9989	EXP 150 of 150
17D24045	2.0 %	1.0100035 ± 0.0016178	0.9429	EXP 150 of 150	5.923099 ± 0.016820	0.8051	EXP 149 of 150	0.4292370 ± 0.0154864	0.0087	EXP 150 of 150	19.9729094 ± 0.0179841	0.9784	EXP 150 of 150	313.159530 ± 0.029024	0.9831	EXP 150 of 150
17D24046	2.1 %	0.5628555 ± 0.0012016	0.8710	EXP 150 of 150	3.634016 ± 0.018748	0.4542	EXP 150 of 150	0.2215910 ± 0.0175885	0.0037	EXP 150 of 150	11.2581044 ± 0.0151507	0.9481	EXP 149 of 150	175.139003 ± 0.027023	0.9898	EXP 150 of 150
17D24048	2.2 %	0.3887275 ± 0.0009926	0.8101	EXP 150 of 150	3.615222 ± 0.018081	0.5728	EXP 149 of 150	0.1828354 ± 0.0166752	0.0499	EXP 150 of 150	9.0322444 ± 0.0170779	0.8978	EXP 149 of 150	120.849789 ± 0.024390	0.9957	EXP 150 of 150
17D24049	2.3 %	0.3749824 ± 0.0009913	0.8132	EXP 150 of 150	4.552150 ± 0.017928	0.6659	EXP 150 of 150	0.1597869 ± 0.0158132	0.0273	EXP 150 of 150	8.6762497 ± 0.0151893	0.9141	EXP 150 of 150	115.760867 ± 0.024143	0.9957	EXP 150 of 150
17D24050	2.4 %	0.2677987 ± 0.0007382	0.7348	EXP 150 of 150	4.124374 ± 0.018581	0.6173	EXP 150 of 150	0.1104875 ± 0.0172352	0.0084	EXP 150 of 150	6.8786725 ± 0.0172124	0.8365	EXP 150 of 150	82.163432 ± 0.020014	0.9982	EXP 150 of 150
17D24052	2.5 %	0.2261098 ± 0.0008172	0.5389	EXP 150 of 150	4.624276 ± 0.018856	0.6098	EXP 150 of 150	0.0648203 ± 0.0173692	0.0074	EXP 150 of 150	6.4376773 ± 0.0173306	0.7773	EXP 150 of 150	69.142900 ± 0.020517	0.9980	EXP 150 of 150
17D24053	2.7 %	0.2126739 ± 0.0007624	0.5870	EXP 150 of 150	5.566056 ± 0.017378	0.7697	EXP 150 of 150	0.0564075 ± 0.0171187	0.0058	EXP 150 of 150	6.4648897 ± 0.0147784	0.8637	EXP 150 of 150	64.537806 ± 0.020066	0.9981	EXP 150 of 150
17D24054	3.0 %	0.1710166 ± 0.0006459	0.4046	EXP 150 of 150	4.751058 ± 0.019472	0.6849	EXP 150 of 150	0.0680585 ± 0.0173669	0.0003	EXP 150 of 150	5.0904020 ± 0.0151097	0.7464	EXP 150 of 150	51.817395 ± 0.022283	0.9980	EXP 150 of 150
17D24056	3.4 %	0.2182841 ± 0.0007232	0.6114	EXP 150 of 150	9.275754 ± 0.016794	0.9065	EXP 150 of 150	0.1099941 ± 0.0156334	0.0026	EXP 150 of 150	7.7091244 ± 0.0149724	0.8917	EXP 150 of 150	65.526346 ± 0.021617	0.9972	EXP 150 of 150
17D24057	3.8 %	0.2200225 ± 0.0007660	0.5917	EXP 150 of 150	13.001691 ± 0.019375	0.9307	EXP 150 of 150	0.0950509 ± 0.0178396	0.0122	EXP 150 of 150	8.8830925 ± 0.0149625	0.9231	EXP 150 of 150	64.422557 ± 0.019592	0.9977	EXP 150 of 150
17D24058	4.2 %	0.2519896 ± 0.0007248	0.7081	EXP 150 of 150	18.192720 ± 0.018452	0.9688	EXP 150 of 150	0.1846882 ± 0.0177206	0.0488	EXP 150 of 150	10.6584447 ± 0.0160702	0.9353	EXP 150 of 150	73.560516 ± 0.020755	0.9968	EXP 150 of 150
17D24060	4.6 %	0.2029353 ± 0.0006763	0.6160	EXP 150 of 150	18.661670 ± 0.020554	0.9636	EXP 150 of 150	0.1046099 ± 0.0180613	0.0031	EXP 150 of 150	9.7649984 ± 0.0170708	0.9178	EXP 150 of 150	57.496336 ± 0.020524	0.9972	EXP 150 of 150
17D24061	5.2 %	0.1915239 ± 0.0006198	0.5948	EXP 150 of 150	20.714036 ± 0.020903	0.9696	EXP 150 of 150	0.1422765 ± 0.0158979	0.0113	EXP 150 of 150	9.6585244 ± 0.0158951	0.9230	EXP 150 of 150	53.172893 ± 0.021561	0.9971	EXP 150 of 150
17D24062	5.8 %	0.2731326 ± 0.0008685	0.6706	EXP 150 of 150	38.224494 ± 0.019091	0.9922	EXP 150 of 150	0.2088305 ± 0.0152083	0.0118	EXP 150 of 150	14.7472065 ± 0.0185037	0.9586	EXP 150 of 150	74.130628 ± 0.022258	0.9952	EXP 150 of 150
17D24064	6.5 %	0.2216203 ± 0.0006144	0.7035	EXP 150 of 150	39.843230 ± 0.018890	0.9931	EXP 150 of 150	0.1542802 ± 0.0159118	0.0000	EXP 150 of 150	13.4102217 ± 0.0176680	0.9571	EXP 150 of 150	57.325639 ± 0.021048	0.9966	EXP 150 of 150
17D24065	7.2 %	0.2159164 ± 0.0006918	0.5855	EXP 150 of 150	46.064273 ± 0.021478	0.9934	EXP 150 of 150	0.1765836 ± 0.0160310	0.0080	EXP 150 of 150	13.6487641 ± 0.0178389	0.9580	EXP 149 of 150	54.075479 ± 0.018553	0.9976	EXP 150 of 150
17D24066	8.0 %	0.1945527 ± 0.0006661	0.5072	EXP 150 of 150	51.174893 ± 0.018898	0.9958	EXP 150 of 150	0.1972478 ± 0.0162511	0.0205	EXP 150 of 150	13.6675487 ± 0.0180742	0.9552	EXP 150 of 150	45.760498 ± 0.018545	0.9980	EXP 150 of 150
17D24068	8.9 %	0.1912680 ± 0.0006886	0.5574	EXP 150 of 150	53.871135 ± 0.022931	0.9944	EXP 150 of 150	0.1469010 ± 0.0168041	0.0004	EXP 150 of 150	13.3897535 ± 0.0162091	0.9626	EXP 150 of 150	44.148766 ± 0.017553	0.9981	EXP 150 of 150
17D24069	9.7 %	0.1629688 ± 0.0005890	0.4236	EXP 150 of 150	45.409934 ± 0.020458	0.9937	EXP 150 of 150	0.1263415 ± 0.0173991	0.0061	EXP 150 of 150	10.4602587 ± 0.0166899	0.9313	EXP 150 of 150	37.509053 ± 0.019874	0.9978	EXP 150 of 150
17D24070	10.6 %	0.1535561 ± 0.0006189	0.4239	EXP 150 of 150	41.323938 ± 0.020104	0.9929	EXP 150 of 150	0.1089096 ± 0.0191524	0.0037	EXP 150 of 150	9.1135593 ± 0.0169077	0.9165	EXP 150 of 150	35.507993 ± 0.018335	0.9981	EXP 150 of 150
17D24072	11.6 %	0.1455539 ± 0.0005744	0.3539	EXP 150 of 150	38.189990 ± 0.019125	0.9923	EXP 150 of 150	0.1007190 ± 0.0171544	0.0022	EXP 150 of 150	7.9632393 ± 0.0164790	0.8874	EXP 150 of 150	33.932745 ± 0.019915	0.9976	EXP 150 of 150
17D24073	12.5 %	0.1192728 ± 0.0005164	0.0726	EXP 150 of 150	29.554052 ± 0.018912	0.9874	EXP 150 of 150	0.0586724 ± 0.0177018	0.0034	EXP 150 of 150	5.8359226 ± 0.0141821	0.8403	EXP 150 of 150	28.433995 ± 0.020224	0.9976	EXP 150 of 150
17D24074	13.4 %	0.1125204 ± 0.0005288	0.0757	EXP 150 of 150	25.438531 ± 0.018557	0.9838	EXP 150 of 150	0.0418301 ± 0.0175243	0.0007	EXP 150 of 150	4.9707667 ± 0.0170602	0.7154	EXP 150 of 150	27.473180 ± 0.019771	0.9977	EXP 150 of 150
17D24076	14.6 %	0.1304733 ± 0.0005601	0.2766	EXP 150 of 150	23.935388 ± 0.020050	0.9783	EXP 150 of 150	0.0508981 ± 0.0174940	0.0010	EXP 150 of 150	4.6335714 ± 0.0169016	0.7018	EXP 150 of 150	33.329047 ± 0.018659	0.9974	EXP 150 of 150
17D24077	16.0 %	0.1233394 ± 0.0005249	0.2181	EXP 150 of 150	21.463300 ± 0.018526	0.9778	EXP 150 of 150	0.0648395 ± 0.0164848	0.0055	EXP 150 of 150	4.3788334 ± 0.0157200	0.6580	EXP 150 of 150	31.888714 ± 0.018757	0.9975	EXP 150 of 150
17D24078	17.6 %	0.1804453 ± 0.0006390	0.5176	EXP 150 of 150	21.888119 ± 0.020205	0.9739	EXP 150 of 150	0.0377176 ± 0.0147628	0.0037	EXP 150 of 150	4.1530305 ± 0.0173033	0.5922	EXP 149 of 150	49.331867 ± 0.019400	0.9961	EXP 150 of 150
17D24080	18.6 %	0.1448527 ± 0.0005556	0.4138	EXP 150 of 150	15.604452 ± 0.019271	0.9546	EXP 150 of 150	0.0387931 ± 0.0173355	0.0030	EXP 150 of 150	2.6939742 ± 0.0154915	0.3835	EXP 150 of 150	39.743366 ± 0.021871	0.9958	EXP 150 of 150
17D24082	20.0 %	0.1617932 ± 0.0006027	0.4733	EXP 149 of 150	16.852230 ± 0.018247	0.9652	EXP 150 of 150	0.0305767 ± 0.0164743	0.0136	EXP 150 of 150	2.3976465 ± 0.0151934	0.3204	EXP 150 of 150	45.248768 ± 0.017675	0.9965	EXP 150 of 150

Project Info		Analyst	Irradiation	X-pos	Y-pos	Z/H-pos	Project	Experiment	Nmb
17D24042	1.8 %	Dan Miggins	17-OSU-05	0.00	0.00	20.12	Arctic\O-Connor (16-22)	17D24038	01
17D24044	1.9 %	Dan Miggins	17-OSU-05	0.00	0.00	20.12	Arctic\O-Connor (16-22)	17D24038	01
17D24045	2.0 %	Dan Miggins	17-OSU-05	0.00	0.00	20.12	Arctic\O-Connor (16-22)	17D24038	01
17D24046	2.1 %	Dan Miggins	17-OSU-05	0.00	0.00	20.12	Arctic\O-Connor (16-22)	17D24038	01
17D24048	2.2 %	Dan Miggins	17-OSU-05	0.00	0.00	20.12	Arctic\O-Connor (16-22)	17D24038	01
17D24049	2.3 %	Dan Miggins	17-OSU-05	0.00	0.00	20.12	Arctic\O-Connor (16-22)	17D24038	01
17D24050	2.4 %	Dan Miggins	17-OSU-05	0.00	0.00	20.12	Arctic\O-Connor (16-22)	17D24038	01
17D24052	2.5 %	Dan Miggins	17-OSU-05	0.00	0.00	20.12	Arctic\O-Connor (16-22)	17D24038	01
17D24053	2.7 %	Dan Miggins	17-OSU-05	0.00	0.00	20.12	Arctic\O-Connor (16-22)	17D24038	01
17D24054	3.0 %	Dan Miggins	17-OSU-05	0.00	0.00	20.12	Arctic\O-Connor (16-22)	17D24038	01
17D24056	3.4 %	Dan Miggins	17-OSU-05	0.00	0.00	20.12	Arctic\O-Connor (16-22)	17D24038	01
17D24057	3.8 %	Dan Miggins	17-OSU-05	0.00	0.00	20.12	Arctic\O-Connor (16-22)	17D24038	01
17D24058	4.2 %	Dan Miggins	17-OSU-05	0.00	0.00	20.12	Arctic\O-Connor (16-22)	17D24038	01
17D24060	4.6 %	Dan Miggins	17-OSU-05	0.00	0.00	20.12	Arctic\O-Connor (16-22)	17D24038	01
17D24061	5.2 %	Dan Miggins	17-OSU-05	0.00	0.00	20.12	Arctic\O-Connor (16-22)	17D24038	01
17D24062	5.8 %	Dan Miggins	17-OSU-05	0.00	0.00	20.12	Arctic\O-Connor (16-22)	17D24038	01
17D24064	6.5 %	Dan Miggins	17-OSU-05	0.00	0.00	20.12	Arctic\O-Connor (16-22)	17D24038	01
17D24065	7.2 %	Dan Miggins	17-OSU-05	0.00	0.00	20.12	Arctic\O-Connor (16-22)	17D24038	01
17D24066	8.0 %	Dan Miggins	17-OSU-05	0.00	0.00	20.12	Arctic\O-Connor (16-22)	17D24038	01
17D24068	8.9 %	Dan Miggins	17-OSU-05	0.00	0.00	20.12	Arctic\O-Connor (16-22)	17D24038	01
17D24069	9.7 %	Dan Miggins	17-OSU-05	0.00	0.00	20.12	Arctic\O-Connor (16-22)	17D24038	01
17D24070	10.6 %	Dan Miggins	17-OSU-05	0.00	0.00	20.12	Arctic\O-Connor (16-22)	17D24038	01
17D24072	11.6 %	Dan Miggins	17-OSU-05	0.00	0.00	20.12	Arctic\O-Connor (16-22)	17D24038	01
17D24073	12.5 %	Dan Miggins	17-OSU-05	0.00	0.00	20.12	Arctic\O-Connor (16-22)	17D24038	01
17D24074	13.4 %	Dan Miggins	17-OSU-05	0.00	0.00	20.12	Arctic\O-Connor (16-22)	17D24038	01
17D24076	14.6 %	Dan Miggins	17-OSU-05	0.00	0.00	20.12	Arctic\O-Connor (16-22)	17D24038	01
17D24077	16.0 %	Dan Miggins	17-OSU-05	0.00	0.00	20.12	Arctic\O-Connor (16-22)	17D24038	01
17D24078	17.6 %	Dan Miggins	17-OSU-05	0.00	0.00	20.12	Arctic\O-Connor (16-22)	17D24038	01
17D24080	18.6 %	Dan Miggins	17-OSU-05	0.00	0.00	20.12	Arctic\O-Connor (16-22)	17D24038	01
17D24082	20.0 %	Dan Miggins	17-OSU-05	0.00	0.00	20.12	Arctic\O-Connor (16-22)	17D24038	01

**Sample Parameters**		Sample	Material	Location	Standard Name	Standard (in Ma)	%1σ	Standard Reference	Standard 40Ar/39Ar	%1σ	J	%1σ	Air 40Ar/36Ar	%1σ	MDF (lin)	%1σ	Volume Ratio	Sensitivity (mol/volt)	Day	Month	Year	Hour	Min	Resist
17D24042	1.8 %	HLY0102-D90-18	Groundmass	Gakkel Ridge	FCT-NM (5A14-17)	28.201	0.082	Kuiper et al (2008)	9.70619	0.115	0.00161932	0.115	302.697	0.141	0.99404825	0.068	1	4.8E-14	14	JUL	2017	3	30	1
17D24044	1.9 %	HLY0102-D90-18	Groundmass	Gakkel Ridge	FCT-NM (5A14-17)	28.201	0.082	Kuiper et al (2008)	9.70619	0.115	0.00161932	0.115	302.697	0.141	0.99404825	0.068	1	4.8E-14	14	JUL	2017	3	56	1
17D24045	2.0 %	HLY0102-D90-18	Groundmass	Gakkel Ridge	FCT-NM (5A14-17)	28.201	0.082	Kuiper et al (2008)	9.70619	0.115	0.00161932	0.115	302.697	0.141	0.99404825	0.068	1	4.8E-14	14	JUL	2017	4	9	1
17D24046	2.1 %	HLY0102-D90-18	Groundmass	Gakkel Ridge	FCT-NM (5A14-17)	28.201	0.082	Kuiper et al (2008)	9.70619	0.115	0.00161932	0.115	302.697	0.141	0.99404825	0.068	1	4.8E-14	14	JUL	2017	4	22	1
17D24048	2.2 %	HLY0102-D90-18	Groundmass	Gakkel Ridge	FCT-NM (5A14-17)	28.201	0.082	Kuiper et al (2008)	9.70619	0.115	0.00161932	0.115	302.697	0.141	0.99404825	0.068	1	4.8E-14	14	JUL	2017	4	48	1
17D24049	2.3 %	HLY0102-D90-18	Groundmass	Gakkel Ridge	FCT-NM (5A14-17)	28.201	0.082	Kuiper et al (2008)	9.70619	0.115	0.00161932	0.115	302.697	0.141	0.99404825	0.068	1	4.8E-14	14	JUL	2017	5	1	1
17D24050	2.4 %	HLY0102-D90-18	Groundmass	Gakkel Ridge	FCT-NM (5A14-17)	28.201	0.082	Kuiper et al (2008)	9.70619	0.115	0.00161932	0.115	302.697	0.141	0.99404825	0.068	1	4.8E-14	14	JUL	2017	5	14	1
17D24052	2.5 %	HLY0102-D90-18	Groundmass	Gakkel Ridge	FCT-NM (5A14-17)	28.201	0.082	Kuiper et al (2008)	9.70619	0.115	0.00161932	0.115	302.697	0.141	0.99404825	0.068	1	4.8E-14	14	JUL	2017	5	40	1
17D24053	2.7 %	HLY0102-D90-18	Groundmass	Gakkel Ridge	FCT-NM (5A14-17)	28.201	0.082	Kuiper et al (2008)	9.70619	0.115	0.00161932	0.115	302.697	0.141	0.99404825	0.068	1	4.8E-14	14	JUL	2017	5	53	1
17D24054	3.0 %	HLY0102-D90-18	Groundmass	Gakkel Ridge	FCT-NM (5A14-17)	28.201	0.082	Kuiper et al (2008)	9.70619	0.115	0.00161932	0.115	302.697	0.141	0.99404825	0.068	1	4.8E-14	14	JUL	2017	6	6	1
17D24056	3.4 %	HLY0102-D90-18	Groundmass	Gakkel Ridge	FCT-NM (5A14-17)	28.201	0.082	Kuiper et al (2008)	9.70619	0.115	0.00161932	0.115	302.697	0.141	0.99404825	0.068	1	4.8E-14	14	JUL	2017	6	32	1
17D24057	3.8 %	HLY0102-D90-18	Groundmass	Gakkel Ridge	FCT-NM (5A14-17)	28.201	0.082	Kuiper et al (2008)	9.70619	0.115	0.00161932	0.115	302.697	0.141	0.99404825	0.068	1	4.8E-14	14	JUL	2017	6	45	1
17D24058	4.2 %	HLY0102-D90-18	Groundmass	Gakkel Ridge	FCT-NM (5A14-17)	28.201	0.082	Kuiper et al (2008)	9.70619	0.115	0.00161932	0.115	302.697	0.141	0.99404825	0.068	1	4.8E-14	14	JUL	2017	6	58	1
17D24060	4.6 %	HLY0102-D90-18	Groundmass	Gakkel Ridge	FCT-NM (5A14-17)	28.201	0.082	Kuiper et al (2008)	9.70619	0.115	0.00161932	0.115	302.697	0.141	0.99404825	0.068	1	4.8E-14	14	JUL	2017	7	24	1
17D24061	5.2 %	HLY0102-D90-18	Groundmass	Gakkel Ridge	FCT-NM (5A14-17)	28.201	0.082	Kuiper et al (2008)	9.70619	0.115	0.00161932	0.115	302.697	0.141	0.99404825	0.068	1	4.8E-14	14	JUL	2017	7	37	1
17D24062	5.8 %	HLY0102-D90-18	Groundmass	Gakkel Ridge	FCT-NM (5A14-17)	28.201	0.082	Kuiper et al (2008)	9.70619	0.115	0.00161932	0.115	302.697	0.141	0.99404825	0.068	1	4.8E-14	14	JUL	2017	7	50	1
17D24064	6.5 %	HLY0102-D90-18	Groundmass	Gakkel Ridge	FCT-NM (5A14-17)	28.201	0.082	Kuiper et al (2008)	9.70619	0.115	0.00161932	0.115	302.697	0.141	0.99404825	0.068	1	4.8E-14	14	JUL	2017	8	16	1
17D24065	7.2 %	HLY0102-D90-18	Groundmass	Gakkel Ridge	FCT-NM (5A14-17)	28.201	0.082	Kuiper et al (2008)	9.70619	0.115	0.00161932	0.115	302.697	0.141	0.99404825	0.068	1	4.8E-14	14	JUL	2017	8	29	1
17D24066	8.0 %	HLY0102-D90-18	Groundmass	Gakkel Ridge	FCT-NM (5A14-17)	28.201	0.082	Kuiper et al (2008)	9.70619	0.115	0.00161932	0.115	302.697	0.141	0.99404825	0.068	1	4.8E-14	14	JUL	2017	8	42	1
17D24068	8.9 %	HLY0102-D90-18	Groundmass	Gakkel Ridge	FCT-NM (5A14-17)	28.201	0.082	Kuiper et al (2008)	9.70619	0.115	0.00161932	0.115	302.697	0.141	0.99404825	0.068	1	4.8E-14	14	JUL	2017	9	8	1
17D24069	9.7 %	HLY0102-D90-18	Groundmass	Gakkel Ridge	FCT-NM (5A14-17)	28.201	0.082	Kuiper et al (2008)	9.70619	0.115	0.00161932	0.115	302.697	0.141	0.99404825	0.068	1	4.8E-14	14	JUL	2017	9	21	1
17D24070	10.6 %	HLY0102-D90-18	Groundmass	Gakkel Ridge	FCT-NM (5A14-17)	28.201	0.082	Kuiper et al (2008)	9.70619	0.115	0.00161932	0.115	302.697	0.141	0.99404825	0.068	1	4.8E-14	14	JUL	2017	9	34	1
17D24072	11.6 %	HLY0102-D90-18	Groundmass	Gakkel Ridge	FCT-NM (5A14-17)	28.201	0.082	Kuiper et al (2008)	9.70619	0.115	0.00161932	0.115	302.697	0.141	0.99404825	0.068	1	4.8E-14	14	JUL	2017	10	0	1
17D24073	12.5 %	HLY0102-D90-18	Groundmass	Gakkel Ridge	FCT-NM (5A14-17)	28.201	0.082	Kuiper et al (2008)	9.70619	0.115	0.00161932	0.115	302.697	0.141	0.99404825	0.068	1	4.8E-14	14	JUL	2017	10	13	1
17D24074	13.4 %	HLY0102-D90-18	Groundmass	Gakkel Ridge	FCT-NM (5A14-17)	28.201	0.082	Kuiper et al (2008)	9.70619	0.115	0.00161932	0.115	302.697	0.141	0.99404825	0.068	1	4.8E-14	14	JUL	2017	10	26	1
17D24076	14.6 %	HLY0102-D90-18	Groundmass	Gakkel Ridge	FCT-NM (5A14-17)	28.201	0.082	Kuiper et al (2008)	9.70619	0.115	0.00161932	0.115	302.697	0.141	0.99404825	0.068	1	4.8E-14	14	JUL	2017	10	52	1
17D24077	16.0 %	HLY0102-D90-18	Groundmass	Gakkel Ridge	FCT-NM (5A14-17)	28.201	0.082	Kuiper et al (2008)	9.70619	0.115	0.00161932	0.115	302.697	0.141	0.99404825	0.068	1	4.8E-14	14	JUL	2017	11	5	1
17D24078	17.6 %	HLY0102-D90-18	Groundmass	Gakkel Ridge	FCT-NM (5A14-17)	28.201	0.082	Kuiper et al (2008)	9.70619	0.115	0.00161932	0.115	302.697	0.141	0.99404825	0.068	1	4.8E-14	14	JUL	2017	11	18	1
17D24080	18.6 %	HLY0102-D90-18	Groundmass	Gakkel Ridge	FCT-NM (5A14-17)	28.201	0.082	Kuiper et al (2008)	9.70619	0.115	0.00161932	0.115	302.697	0.141	0.99404825	0.068	1	4.8E-14	14	JUL	2017	11	44	1
17D24082	20.0 %	HLY0102-D90-18	Groundmass	Gakkel Ridge	FCT-NM (5A14-17)	28.201	0.082	Kuiper et al (2008)	9.70619	0.115	0.00161932	0.115	302.697	0.141	0.99404825	0.068	1	4.8E-14	14	JUL	2017	12	10	1

Irradiation Constants		40/36(a)	%1σ	40/36(c)	%1σ	38/36(a)	%1σ	38/36(c)	%1σ	39/37(ca)	%1σ	38/37(ca)	%1σ	36/37(ca)	%1σ	40/39(k)	%1σ	38/39(k)	%1σ	36/38(cl)	%1σ	K/Ca	%1σ	K/Cl	%1σ	Ca/Cl	%1σ
17D24042	1.8 %	297.96	0.235	0.018	35	0.1869	0	1.493	3	0.0006425	0.92	0.00018	9.63	0.0002703	0.17	0.000607	9.65	0.012077	0.09	0	0	0.43	0	0	0	0	0
17D24044	1.9 %	297.96	0.235	0.018	35	0.1869	0	1.493	3	0.0006425	0.92	0.00018	9.63	0.0002703	0.17	0.000607	9.65	0.012077	0.09	0	0	0.43	0	0	0	0	0
17D24045	2.0 %	297.96	0.235	0.018	35	0.1869	0	1.493	3	0.0006425	0.92	0.00018	9.63	0.0002703	0.17	0.000607	9.65	0.012077	0.09	0	0	0.43	0	0	0	0	0
17D24046	2.1 %	297.96	0.235	0.018	35	0.1869	0	1.493	3	0.0006425	0.92	0.00018	9.63	0.0002703	0.17	0.000607	9.65	0.012077	0.09	0	0	0.43	0	0	0	0	0
17D24048	2.2 %	297.96	0.235	0.018	35	0.1869	0	1.493	3	0.0006425	0.92	0.00018	9.63	0.0002703	0.17	0.000607	9.65	0.012077	0.09	0	0	0.43	0	0	0	0	0
17D24049	2.3 %	297.96	0.235	0.018	35	0.1869	0	1.493	3	0.0006425	0.92	0.00018	9.63	0.0002703	0.17	0.000607	9.65	0.012077	0.09	0	0	0.43	0	0	0	0	0
17D24050	2.4 %	297.96	0.235	0.018	35	0.1869	0	1.493	3	0.0006425	0.92	0.00018	9.63	0.0002703	0.17	0.000607	9.65	0.012077	0.09	0	0	0.43	0	0	0	0	0
17D24052	2.5 %	297.96	0.235	0.018	35	0.1869	0	1.493	3	0.0006425	0.92	0.00018	9.63	0.0002703	0.17	0.000607	9.65	0.012077	0.09	0	0	0.43	0	0	0	0	0
17D24053	2.7 %	297.96	0.235	0.018	35	0.1869	0	1.493	3	0.0006425	0.92	0.00018	9.63	0.0002703	0.17	0.000607	9.65	0.012077	0.09	0	0	0.43	0	0	0	0	0
17D24054	3.0 %	297.96	0.235	0.018	35	0.1869	0	1.493	3	0.0006425	0.92	0.00018	9.63	0.0002703	0.17	0.000607	9.65	0.012077	0.09	0	0	0.43	0	0	0	0	0
17D24056	3.4 %	297.96	0.235	0.018	35	0.1869	0	1.493	3	0.0006425	0.92	0.00018	9.63	0.0002703	0.17	0.000607	9.65	0.012077	0.09	0	0	0.43	0	0	0	0	0
17D24057	3.8 %	297.96	0.235	0.018	35	0.1869	0	1.493	3	0.0006425	0.92	0.00018	9.63	0.0002703	0.17	0.000607	9.65	0.012077	0.09	0	0	0.43	0	0	0	0	0
17D24058	4.2 %	297.96	0.235	0.018	35	0.1869	0	1.493	3	0.0006425	0.92	0.00018	9.63	0.0002703	0.17	0.000607	9.65	0.012077	0.09	0	0	0.43	0	0	0	0	0
17D24060	4.6 %	297.96	0.235	0.018	35	0.1869	0	1.493	3	0.0006425	0.92	0.00018	9.63	0.0002703	0.17	0.000607	9.65	0.012077	0.09	0	0	0.43	0	0	0	0	0
17D24061	5.2 %	297.96	0.235	0.018	35	0.1869	0	1.493	3	0.0006425	0.92	0.00018	9.63	0.0002703	0.17	0.000607	9.65	0.012077	0.09	0	0	0.43	0	0	0	0	0
17D24062	5.8 %	297.96	0.235	0.018	35	0.1869	0	1.493	3	0.0006425	0.92	0.00018	9.63	0.0002703	0.17	0.000607	9.65	0.012077	0.09	0	0	0.43	0	0	0	0	0
17D24064	6.5 %	297.96	0.235	0.018	35	0.1869	0	1.493	3	0.0006425	0.92	0.00018	9.63	0.0002703	0.17	0.000607	9.65	0.012077	0.09	0	0	0.43	0	0	0	0	0
17D24065	7.2 %	297.96	0.235	0.018	35	0.1869	0	1.493	3	0.0006425	0.92	0.00018	9.63	0.0002703	0.17	0.000607	9.65	0.012077	0.09	0	0	0.43	0	0	0	0	0
17D24066	8.0 %	297.96	0.235	0.018	35	0.1869	0	1.493	3	0.0006425	0.92	0.00018	9.63	0.0002703	0.17	0.000607	9.65	0.012077	0.09	0	0	0.43	0	0	0	0	0
17D24068	8.9 %	297.96	0.235	0.018	35	0.1869	0	1.493	3	0.0006425	0.92	0.00018	9.63	0.0002703	0.17	0.000607	9.65	0.012077	0.09	0	0	0.43	0	0	0	0	0
17D24069	9.7 %	297.96	0.235	0.018	35	0.1869	0	1.493	3	0.0006425	0.92	0.00018	9.63	0.0002703	0.17	0.000607	9.65	0.012077	0.09	0	0	0.43	0	0	0	0	0
17D24070	10.6 %	297.96	0.235	0.018	35	0.1869	0	1.493	3	0.0006425	0.92	0.00018	9.63	0.0002703	0.17	0.000607	9.65	0.012077	0.09	0	0	0.43	0	0	0	0	0
17D24072	11.6 %	297.96	0.235	0.018	35	0.1869	0	1.493	3	0.0006425	0.92	0.00018	9.63	0.0002703	0.17	0.000607	9.65	0.012077	0.09	0	0	0.43	0	0	0	0	0
17D24073	12.5 %	297.96	0.235	0.018	35	0.1869	0	1.493	3	0.0006425	0.92	0.00018	9.63	0.0002703	0.17	0.000607	9.65	0.012077	0.09	0	0	0.43	0	0	0	0	0
17D24074	13.4 %	297.96	0.235	0.018	35	0.1869	0	1.493	3	0.0006425	0.92	0.00018	9.63	0.0002703	0.17	0.000607	9.65	0.012077	0.09	0	0	0.43	0	0	0	0	0
17D24076	14.6 %	297.96	0.235	0.018	35	0.1869	0	1.493	3	0.0006425	0.92	0.00018	9.63	0.0002703	0.17	0.000607	9.65	0.012077	0.09	0	0	0.43	0	0	0	0	0
17D24077	16.0 %	297.96	0.235	0.018	35	0.1869	0	1.493	3	0.0006425	0.92	0.00018	9.63	0.0002703	0.17	0.000607	9.65	0.012077	0.09	0	0	0.43	0	0	0	0	0
17D24078	17.6 %	297.96	0.235	0.018	35	0.1869	0	1.493	3	0.0006425	0.92	0.00018	9.63	0.0002703	0.17	0.000607	9.65	0.012077	0.09	0	0	0.43	0	0	0	0	0
17D24080	18.6 %	297.96	0.235	0.018	35	0.1869	0	1.493	3	0.0006425	0.92	0.00018	9.63	0.0002703	0.17	0.000607	9.65	0.012077	0.09	0	0	0.43	0	0	0	0	0
17D24082	20.0 %	297.96	0.235	0.018	35	0.1869	0	1.493	3	0.0006425	0.92	0.00018	9.63	0.0002703	0.17	0.000607	9.65	0.012077	0.09	0	0	0.43	0	0	0	0	0

17D24038.AGE >>> HLY0102-D90-18 >>> ARCTIC | O-CONNOR (16-22) PROJECT

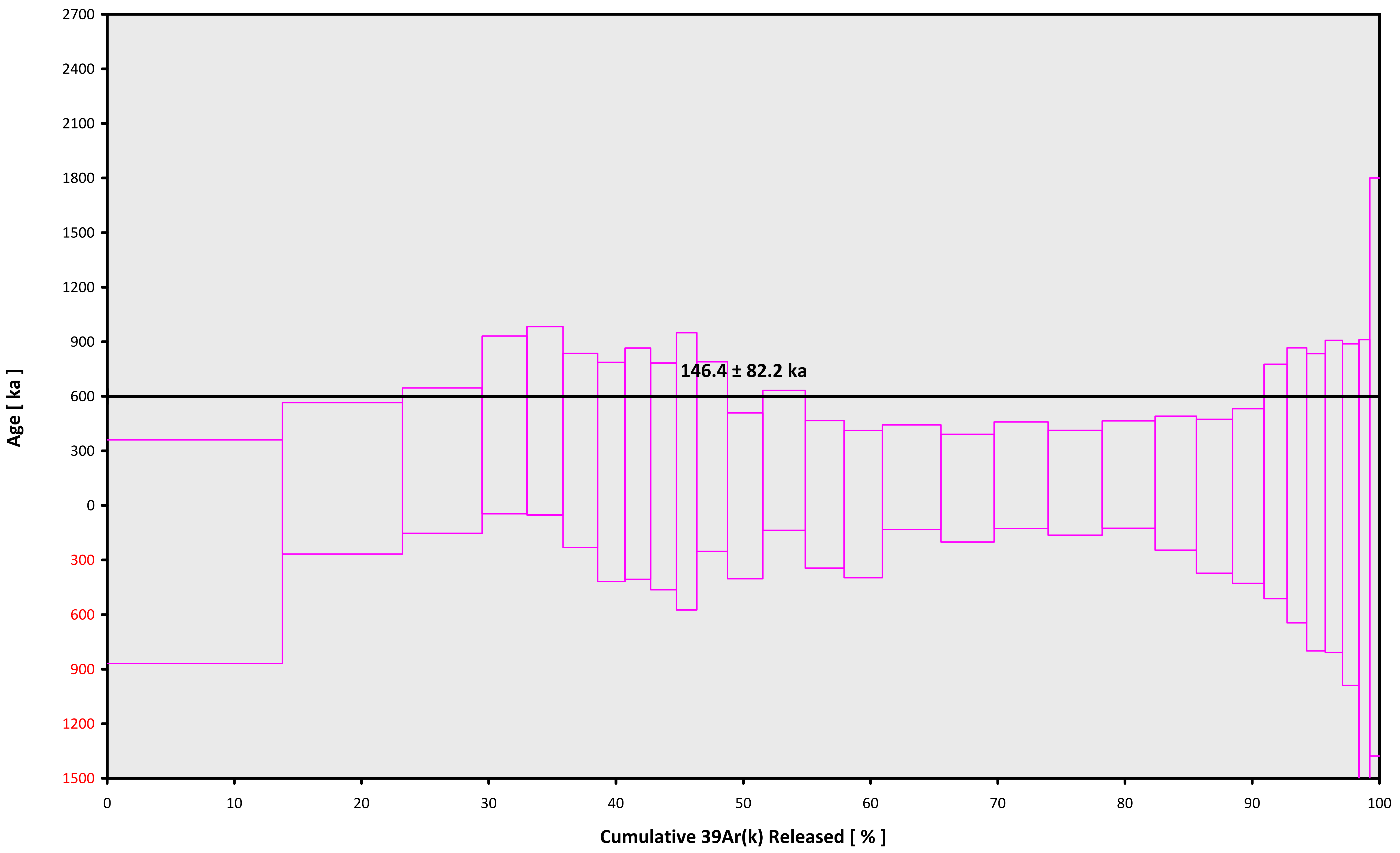

Ar-Ages in ka

WEIGHTED PLATEAU
146.4 ± 82.2
TOTAL FUSION
103.8 ± 117.0
NORMAL ISOCHRON
146.6 ± 131.9
INVERSE ISOCHRON
146.7 ± 75.1

MSWD (PROBABILITY)
0.29 (100%)

Sample Info

Groundmass
Gakkel Ridge
Dan Miggins

IRR = 17-OSU-05 (5A14-17)
J = 0.00161932 ± 0.00000186

17D24038.AGE >>> HLY0102-D90-18 >>> ARCTIC | O-CONNOR (16-22) PROJECT

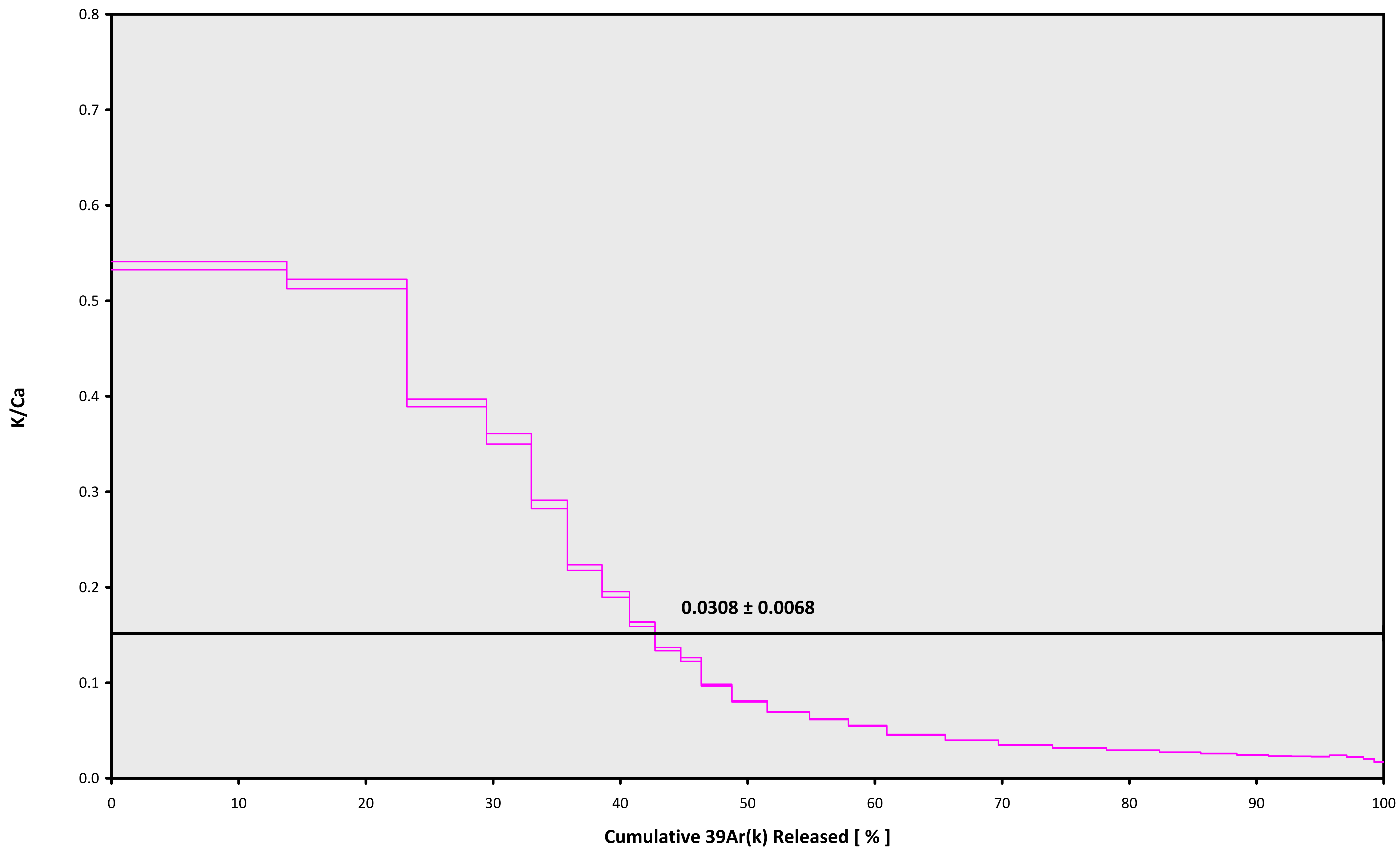

Ar-Ages in ka

WEIGHTED PLATEAU
146.4 ± 82.2
TOTAL FUSION
103.8 ± 117.0
NORMAL ISOCHRON
146.6 ± 131.9
INVERSE ISOCHRON
146.7 ± 75.1

Sample Info

Groundmass
Gakkel Ridge
Dan Miggins

IRR = 17-OSU-05 (5A14-17)
J = 0.00161932 ± 0.00000186

17D24038.AGE >>> HLY0102-D90-18 >>> ARCTIC | O-CONNOR (16-22) PROJECT

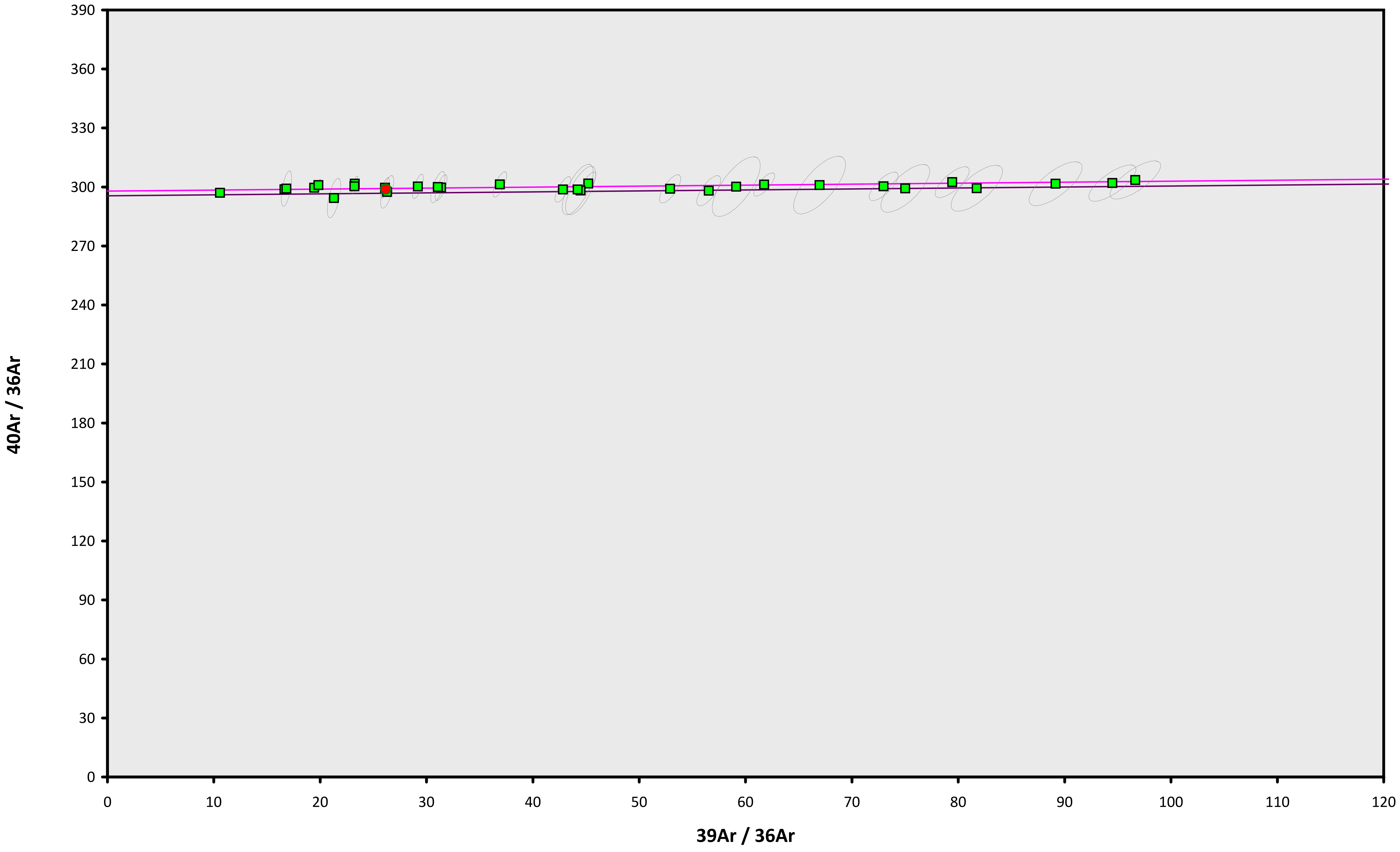

Ar-Ages in ka

WEIGHTED PLATEAU
146.4 ± 82.2
TOTAL FUSION
103.8 ± 117.0
NORMAL ISOCHRON
146.6 ± 131.9
INVERSE ISOCHRON
146.7 ± 75.1

MSWD (PROBABILITY)
0.37 (100%)

40AR/36AR INTERCEPT
297.9 ± 1.4

Sample Info

Groundmass
Gakkel Ridge
Dan Miggins

IRR = 17-OSU-05 (5A14-17)
J = 0.00161932 ± 0.00000186

17D24038.AGE >>> HLY0102-D90-18 >>> ARCTIC | O-CONNOR (16-22) PROJECT

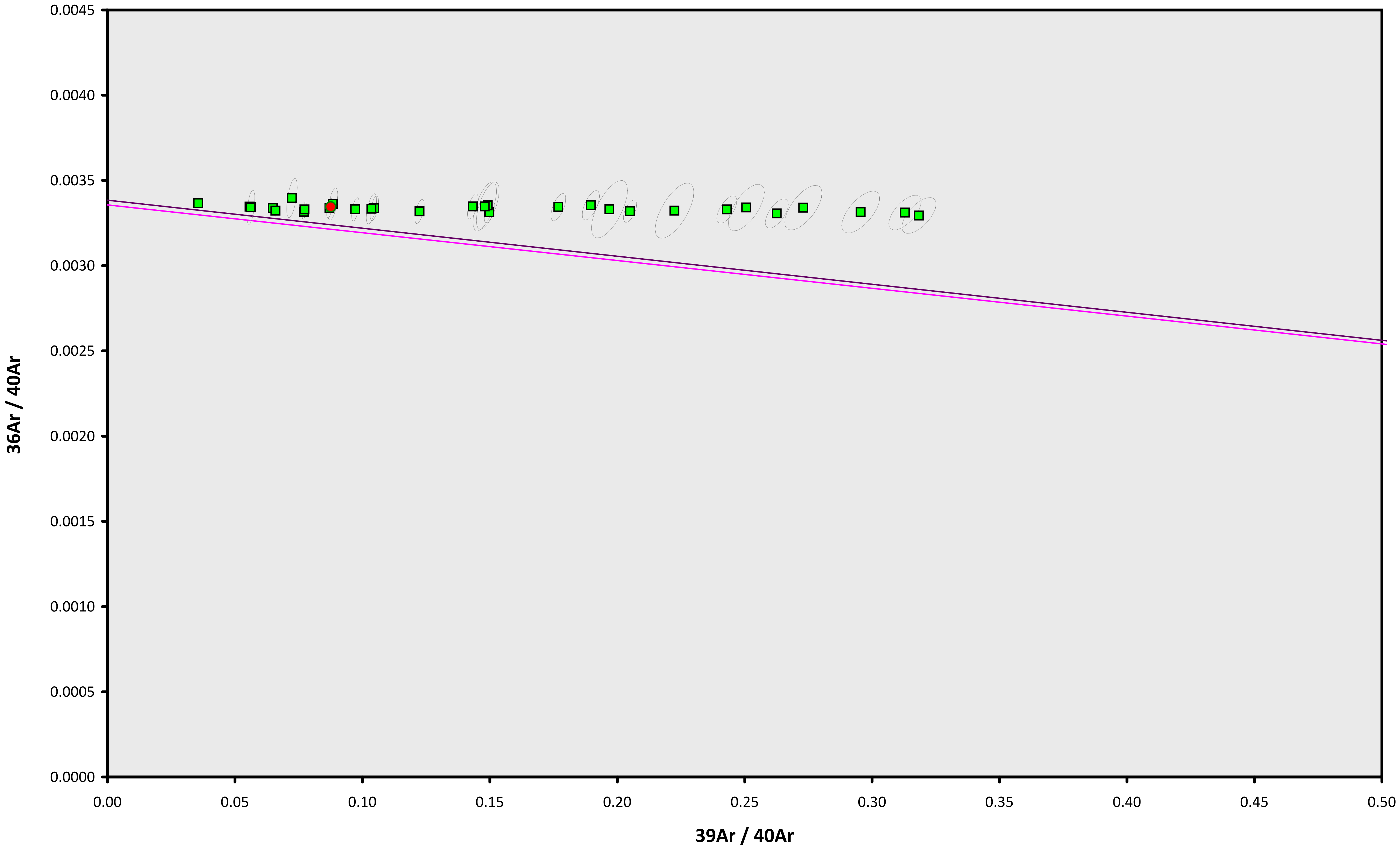

Ar-Ages in ka

WEIGHTED PLATEAU
146.4 ± 82.2
TOTAL FUSION
103.8 ± 117.0
NORMAL ISOCHRON
146.6 ± 131.9
INVERSE ISOCHRON
146.7 ± 75.1

MSWD (PROBABILITY)
0.37 (100%)

SPREADING FACTOR
1.4%

40AR/36AR INTERCEPT
298.0 ± 1.4

Sample Info

Groundmass
Gakkel Ridge
Dan Miggins

IRR = 17-OSU-05 (5A14-17)
J = 0.00161932 ± 0.00000186

Relative Abundances

Relative Abundances			36Ar [fA]	%1σ	37Ar [fA]	%1σ	38Ar [fA]	%1σ	39Ar [fA]	%1σ	40Ar [fA]	%1σ	40(r)/39(k) ± 2σ	Age ± 2σ (Ma)	40Ar(r) (%)	39Ar(k) (%)	K/Ca ± 2σ
17D17887	1.8 %		0.3612969	0.381	52.3359	0.732	0.2065574	11.681	10.27939	0.236	110.0771	0.079	0.73088 ± 0.09423	2.19 ± 0.28	6.80	4.99	0.0842 ± 0.0013
17D17889	1.9 %		0.3083723	0.391	63.8343	0.623	0.1893065	12.070	9.52851	0.250	91.2463	0.057	0.54974 ± 0.08751	1.65 ± 0.26	5.72	4.62	0.0639 ± 0.0009
17D17890	2.0 %	✓	0.2052323	0.449	47.7260	0.762	0.1344906	18.158	6.41180	0.354	59.7634	0.069	0.45857 ± 0.09657	1.38 ± 0.29	4.90	3.10	0.0575 ± 0.0010
17D17892	2.2 %	✓	0.2300840	0.447	75.5897	0.577	0.1165336	22.210	8.49596	0.266	65.9176	0.065	0.46881 ± 0.08109	1.41 ± 0.24	6.01	4.11	0.0481 ± 0.0006
17D17893	2.4 %	✓	0.1958867	0.491	72.4392	0.588	0.1344417	18.306	7.38159	0.309	55.1384	0.072	0.41382 ± 0.08571	1.24 ± 0.26	5.51	3.57	0.0435 ± 0.0006
17D17895	2.7 %	✓	0.2359271	0.439	112.3232	0.492	0.1869985	12.541	10.16438	0.234	65.1002	0.063	0.43100 ± 0.06817	1.29 ± 0.20	6.68	4.91	0.0386 ± 0.0004
17D17896	3.0 %	✓	0.2170436	0.446	118.5394	0.480	0.1996011	11.562	9.90132	0.245	59.1207	0.069	0.45272 ± 0.06529	1.36 ± 0.20	7.52	4.78	0.0356 ± 0.0004
17D17898	3.4 %	✓	0.2395023	0.446	153.7601	0.456	0.1932707	12.487	11.49868	0.206	64.0501	0.063	0.48699 ± 0.06183	1.46 ± 0.19	8.67	5.55	0.0319 ± 0.0003
17D17899	3.9 %	✓	0.2545931	0.407	182.7055	0.444	0.2498608	10.057	12.50239	0.203	66.4717	0.061	0.47049 ± 0.05618	1.41 ± 0.17	8.77	6.03	0.0291 ± 0.0003
17D17901	4.5 %	✓	0.2597527	0.432	196.5668	0.439	0.2199727	10.680	12.14756	0.198	66.1890	0.061	0.42636 ± 0.06188	1.28 ± 0.19	7.74	5.85	0.0263 ± 0.0003
17D17902	5.2 %	✓	0.3310177	0.370	268.7324	0.424	0.2363857	9.961	15.01554	0.159	83.9808	0.055	0.51342 ± 0.05645	1.54 ± 0.17	9.07	7.22	0.0238 ± 0.0002
17D17904	6.0 %	✓	0.2922926	0.386	237.2470	0.430	0.1996510	12.171	12.23273	0.198	73.3032	0.056	0.48618 ± 0.06330	1.46 ± 0.19	8.01	5.88	0.0219 ± 0.0002
17D17905	6.9 %	✓	0.2723465	0.406	215.5299	0.431	0.1989291	12.458	10.76526	0.231	68.9087	0.061	0.53065 ± 0.06977	1.59 ± 0.21	8.18	5.17	0.0212 ± 0.0002
17D17907	7.9 %	✓	0.2753941	0.399	213.9420	0.433	0.2156321	10.912	10.35526	0.231	69.2082	0.057	0.48074 ± 0.07238	1.44 ± 0.22	7.10	4.97	0.0205 ± 0.0002
17D17908	9.0 %		0.2385429	0.423	177.7264	0.446	0.1639660	14.592	8.44080	0.274	59.3967	0.067	0.37205 ± 0.08077	1.12 ± 0.24	5.22	4.05	0.0201 ± 0.0002
17D17910	10.3 %		0.2658741	0.411	178.9203	0.447	0.2056599	11.464	8.92621	0.271	68.3750	0.059	0.46491 ± 0.08303	1.40 ± 0.25	5.99	4.29	0.0212 ± 0.0002
17D17911	11.6 %		0.2637642	0.435	156.2508	0.456	0.1742405	14.226	7.64029	0.297	68.0803	0.059	0.34676 ± 0.10089	1.04 ± 0.30	3.84	3.67	0.0207 ± 0.0002
17D17912	12.5 %		0.3288880	0.381	160.5354	0.450	0.2062707	11.860	7.26172	0.304	86.9898	0.047	0.36654 ± 0.11931	1.10 ± 0.36	3.02	3.48	0.0192 ± 0.0002
17D17914	13.4 %		0.3062658	0.410	146.2678	0.464	0.1503684	15.537	5.61598	0.392	80.3253	0.050	0.27234 ± 0.15263	0.82 ± 0.46	1.87	2.69	0.0162 ± 0.0002
17D17915	14.6 %		0.5069331	0.347	245.9673	0.431	0.1884415	12.520	7.64718	0.295	132.4722	0.032	0.30915 ± 0.16369	0.93 ± 0.49	1.75	3.64	0.0131 ± 0.0001
17D17916	16.0 %		0.5895155	0.337	285.6612	0.423	0.2040946	12.058	6.94867	0.327	154.5944	0.028	0.47379 ± 0.20606	1.42 ± 0.62	2.07	3.29	0.0102 ± 0.0001
17D17918	17.6 %		0.4334448	0.365	223.9991	0.433	0.1800054	13.581	4.64538	0.488	111.9667	0.038	0.39401 ± 0.24146	1.18 ± 0.72	1.58	2.19	0.0086 ± 0.0001
17D17919	19.3 %		0.2606840	0.438	162.4413	0.451	0.0991881	24.491	3.18668	0.748	65.1261	0.060	0.34613 ± 0.24469	1.04 ± 0.73	1.64	1.50	0.0082 ± 0.0001
17D17921	21.0 %		0.0734573	0.679	47.4990	0.725	0.0361282	67.016	0.96712	2.221	18.1703	0.206	0.27478 ± 0.34260	0.82 ± 1.03	1.42	0.46	0.0085 ± 0.0004
		Σ	6.9461115	0.085	3796.5402	0.100	4.2899947	2.754	207.96040	0.055	1843.9723	0.012					

Information on Analysis and Constants Used in Calculations

Project = **O-CONNOR (16-22)**
Sample = **HLY0102-D89-004**
Material = **Groundmass**
Location = **Gakkel Ridge**
Region = **Artic Ocean**
Analyst = **Dan Miggins**
Irradiation = **17-OSU-01 (1C2-17)**
Position = **X: 0 | Y: 0 | Z/H: 3.663492 mm**
FCT-NM Age = **28.201 ± 0.023 Ma**
FCT-NM Reference = **Kuiper et al (2008)**
FCT-NM 40Ar/39Ar Ratio = **9.46726 ± 0.00852**
FCT-NM J-value = **0.00166018 ± 0.00000149**
Air Shot 40Ar/36Ar = **302.6190 ± 0.4025**
Air Shot MDF = **0.99411124 ± 0.00066430 (LIN)**
Experiment Type = **Incremental Heating**
Extraction Method = **Bulk Laser Heating**
Heating = **64 sec**
Isolation = **3.00 min**
Instrument = **ARGUS-VI-D**
Preferred Age = **Plateau Age**
Age Classification = **Crystallization Age**
IGSN = Undefined
Rock Class = **Igneous>Volcanic**
Lithology = **Basaltic Lava**
Lat-Lon = Undefined - Undefined

Age Equations = **Min et al. (2000)**
Negative Intensities = **Allowed**
Collector Calibrations = **36Ar**
Decay 40K = **5.530 ± 0.048 E-10 1/a**
Decay 39Ar = **2.940 ± 0.016 E-07 1/h**
Decay 37Ar = **8.230 ± 0.012 E-04 1/h**
Decay 36Cl = **2.257 ± 0.015 E-06 1/a**
Decay 40K(EC,β⁺) = **0.580 ± 0.009 E-10 1/a**
Decay 40K(β⁻) = **4.950 ± 0.043 E-10 1/a**
Atmospheric 40/36(a) = **295.50 ± 0.70**
Atmospheric 38/36(a) = **0.1869**
Production 39/37(ca) = **0.0006425 ± 0.0000059**
Production 38/37(ca) = **0.0001800 ± 0.0000173**
Production 36/37(ca) = **0.0002703 ± 0.0000005**
Production 40/39(k) = **0.000607 ± 0.000059**
Production 38/39(k) = **0.012077 ± 0.000011**
Production 36/38(cl) = **262.80 ± 1.71**
Scaling Ratio K/Ca = **0.430**
Abundance Ratio 40K/K = **1.1700 ± 0.0100 E-04**
Atomic Weight K = **39.0983 ± 0.0001 g**

Results

Results	40(a)/36(a) ± 2σ	40(r)/39(k) ± 2σ	Age ± 2σ (Ma)	MSWD	39Ar(k) (%,n)	K/Ca ± 2σ
**Age Plateau**		0.47147 ± 0.01977 ± 4.19%	**1.41 ± 0.06 ± 4.20%**	1.03	61.14 41% 12	0.0260 ± 0.0041
		Full External Error	± 0.07	1.85	2σ Confidence Limit	
		Analytical Error	± 0.06	1.0168	Error Magnification	
**Total Fusion Age**		0.45994 ± 0.01945 ± 4.23%	**1.38 ± 0.06 ± 4.23%**		24	0.0233 ± 0.0001
		Full External Error	± 0.07			
		Analytical Error	± 0.06			
**Normal Isochron**	**293.75 ± 6.30 ± 2.15%**	0.50541 ± 0.12509 ± 24.75%	**1.52 ± 0.38 ± 24.74%**	1.32	61.14 21% 12	
		Full External Error	± 0.38	1.89	2σ Confidence Limit	
		Analytical Error	± 0.38	1.1500	Error Magnification	
				4	Number of Iterations	
				0.0000019598	Convergence	
**Inverse Isochron** Clustered Points	**293.73 ± 6.31 ± 2.15%**	0.50637 ± 0.11529 ± 22.77%	**1.52 ± 0.35 ± 22.76%**	1.32	61.14 21% 12	
		Full External Error	± 0.35	1.89	2σ Confidence Limit	
		Analytical Error	± 0.35	1.1500	Error Magnification	
				3	Number of Iterations	
				0.0000887893	Convergence	
				4%	Spreading Factor	

**Incremental Heating**			36Ar(a) [fA]	37Ar(ca) [fA]	38Ar(cl) [fA]	39Ar(k) [fA]	40Ar(r) [fA]	Age ± 2σ (Ma)	40Ar(r) (%)	39Ar(k) (%)	K/Ca ± 2σ
17D17887	1.8 %		0.3471488	52.3359	0.0085167	10.24577	7.488450	2.19 ± 0.28	6.80	4.99	0.0842 ± 0.0013
17D17889	1.9 %		0.2911161	63.8343	0.0088262	9.48750	5.215675	1.65 ± 0.26	5.72	4.62	0.0639 ± 0.0009
17D17890	2.0 %	✓	0.1923293	47.7260	0.0128886	6.38114	2.926190	1.38 ± 0.29	4.90	3.10	0.0575 ± 0.0010
17D17892	2.2 %	✓	0.2096521	75.5897	0.0000000	8.44739	3.960251	1.41 ± 0.24	6.01	4.11	0.0481 ± 0.0006
17D17893	2.4 %	✓	0.1763063	72.4392	0.0000000	7.33505	3.035426	1.24 ± 0.26	5.51	3.57	0.0435 ± 0.0006
17D17895	2.7 %	✓	0.2055648	112.3232	0.0064766	10.09221	4.349700	1.29 ± 0.20	6.68	4.91	0.0386 ± 0.0004
17D17896	3.0 %	✓	0.1849973	118.5394	0.0250296	9.82515	4.448029	1.36 ± 0.20	7.52	4.78	0.0356 ± 0.0004
17D17898	3.4 %	✓	0.1979409	153.7601	0.0000000	11.39989	5.551640	1.46 ± 0.19	8.67	5.55	0.0319 ± 0.0003
17D17899	3.9 %	✓	0.2052019	182.7055	0.0290478	12.38500	5.827027	1.41 ± 0.17	8.77	6.03	0.0291 ± 0.0003
17D17901	4.5 %	✓	0.2066205	196.5668	0.0007925	12.02127	5.125346	1.28 ± 0.19	7.74	5.85	0.0263 ± 0.0003
17D17902	5.2 %	✓	0.2583793	268.7324	0.0000000	14.84288	7.620665	1.54 ± 0.17	9.07	7.22	0.0238 ± 0.0002
17D17904	6.0 %	✓	0.2281647	237.2470	0.0000000	12.08029	5.873176	1.46 ± 0.19	8.01	5.88	0.0219 ± 0.0002
17D17905	6.9 %	✓	0.2140887	215.5299	0.0000000	10.62678	5.639052	1.59 ± 0.21	8.18	5.17	0.0212 ± 0.0002
17D17907	7.9 %	✓	0.2175630	213.9420	0.0130597	10.21780	4.912115	1.44 ± 0.22	7.10	4.97	0.0205 ± 0.0002
17D17908	9.0 %		0.1905034	177.7264	0.0000000	8.32661	3.097880	1.12 ± 0.24	5.22	4.05	0.0201 ± 0.0002
17D17910	10.3 %		0.2175065	178.9203	0.0263888	8.81125	4.096438	1.40 ± 0.25	5.99	4.29	0.0212 ± 0.0002
17D17911	11.6 %		0.2215268	156.2508	0.0136527	7.53989	2.614529	1.04 ± 0.30	3.84	3.67	0.0207 ± 0.0002
17D17912	12.5 %		0.2854876	160.5354	0.0375625	7.15858	2.623905	1.10 ± 0.36	3.02	3.48	0.0192 ± 0.0002
17D17914	13.4 %		0.2667281	146.2678	0.0074995	5.52200	1.503840	0.82 ± 0.46	1.87	2.69	0.0162 ± 0.0002
17D17915	14.6 %		0.4404482	245.9673	0.0000000	7.48914	2.315264	0.93 ± 0.49	1.75	3.64	0.0131 ± 0.0001
17D17916	16.0 %		0.5123013	285.6612	0.0000000	6.76513	3.205279	1.42 ± 0.62	2.07	3.29	0.0102 ± 0.0001
17D17918	17.6 %		0.3728947	223.9991	0.0156275	4.50146	1.773638	1.18 ± 0.72	1.58	2.19	0.0086 ± 0.0001
17D17919	19.3 %		0.2167761	162.4413	0.0000000	3.08232	1.066889	1.04 ± 0.73	1.64	1.50	0.0082 ± 0.0001
17D17921	21.0 %		0.0606173	47.4990	0.0049376	0.93661	0.257356	0.82 ± 1.03	1.42	0.46	0.0085 ± 0.0004
		Σ	5.9198639	3796.5402	0.2103062	205.52112	94.527760				

Information on Analysis

Project = **O-CONNOR (16-22)**
Sample = **HLY0102-D89-004**
Material = **Groundmass**
Location = **Gakkel Ridge**
Region = **Artic Ocean**
Analyst = **Dan Miggins**
Irradiation = **17-OSU-01 (1C2-17)**
J = **0.00166018 ± 0.00000149**
FCT-NM = **28.201 ± 0.023 Ma**

**Results**	40(r)/39(k) ± 2σ		Age ± 2σ (Ma)		MSWD	39Ar(k) (%,n)	K/Ca ± 2σ
**Age Plateau**	0.47147	± 0.01977<br>± 4.19%	**1.41**	**± 0.06**<br>**± 4.20%**	1.03<br>41%	61.14<br>12	0.0260 ± 0.0041
			Full External Error	± 0.07	1.85	2σ Confidence Limit	
			Analytical Error	± 0.06	1.0168	Error Magnification	
**Total Fusion Age**	0.45994	± 0.01945<br>± 4.23%	**1.38**	**± 0.06**<br>**± 4.23%**		24	0.0233 ± 0.0001
			Full External Error	± 0.07			
			Analytical Error	± 0.06			

Normal Isochron			39(k)/36(a) ± 2σ	40(a+r)/36(a) ± 2σ	r.i.
17D17887	1.8 %		29.51 ± 0.27	317.07 ± 2.57	0.8424
17D17889	1.9 %		32.59 ± 0.32	313.42 ± 2.63	0.8480
17D17890	2.0 %	✓	33.18 ± 0.40	310.71 ± 3.02	0.7965
17D17892	2.2 %	✓	40.29 ± 0.45	314.39 ± 3.14	0.8722
17D17893	2.4 %	✓	41.60 ± 0.53	312.72 ± 3.46	0.8624
17D17895	2.7 %	✓	49.10 ± 0.55	316.66 ± 3.25	0.9003
17D17896	3.0 %	✓	53.11 ± 0.62	319.54 ± 3.42	0.8993
17D17898	3.4 %	✓	57.59 ± 0.68	323.55 ± 3.58	0.9292
17D17899	3.9 %	✓	60.36 ± 0.67	323.90 ± 3.38	0.9235
17D17901	4.5 %	✓	58.18 ± 0.69	320.31 ± 3.59	0.9355
17D17902	5.2 %	✓	57.45 ± 0.59	324.99 ± 3.21	0.9440
17D17904	6.0 %	✓	52.95 ± 0.58	321.24 ± 3.30	0.9254
17D17905	6.9 %	✓	49.64 ± 0.58	321.84 ± 3.44	0.9091
17D17907	7.9 %	✓	46.96 ± 0.54	318.08 ± 3.33	0.9062
17D17908	9.0 %		43.71 ± 0.53	311.76 ± 3.41	0.8836
17D17910	10.3 %		40.51 ± 0.47	314.33 ± 3.25	0.8758
17D17911	11.6 %		34.04 ± 0.41	307.30 ± 3.26	0.8629
17D17912	12.5 %		25.07 ± 0.27	304.69 ± 2.72	0.8168
17D17914	13.4 %		20.70 ± 0.26	301.14 ± 2.89	0.7629
17D17915	14.6 %		17.00 ± 0.17	300.76 ± 2.45	0.7995
17D17916	16.0 %		13.21 ± 0.14	301.76 ± 2.38	0.7574
17D17918	17.6 %		12.07 ± 0.16	300.26 ± 2.60	0.6471
17D17919	19.3 %		14.22 ± 0.27	300.42 ± 3.24	0.5654
17D17921	21.0 %		15.45 ± 0.75	299.75 ± 5.17	0.3331

Results	40(a)/36(a) ± 2σ		40(r)/39(k) ± 2σ		Age ± 2σ (Ma)		MSWD
**Normal Isochron**	**293.75**	**± 6.30**<br>**± 2.15%**	0.50541	± 0.12509<br>± 24.75%	**1.52**	**± 0.38**<br>**± 24.74%**	1.32<br>21%
					Full External Error	± 0.38	
					Analytical Error	± 0.38	

Statistics				
	2σ Confidence Limit	1.89	Convergence	0.000001959777
	Error Magnification	1.1500	Number of Iterations	4
	Number of Data Points	12	Calculated Line	Weighted York-2

Inverse Isochron			39(k)/40(a+r) ± 2σ	36(a)/40(a+r) ± 2σ	r.i.
17D17887	1.8 %		0.0930833 ± 0.0004651	0.00315386 ± 0.00002555	0.0610
17D17889	1.9 %		0.1039834 ± 0.0005357	0.00319065 ± 0.00002677	0.0297
17D17890	2.0 %	✓	0.1067803 ± 0.0007736	0.00321839 ± 0.00003133	0.0270
17D17892	2.2 %	✓	0.1281608 ± 0.0007051	0.00318077 ± 0.00003172	0.0306
17D17893	2.4 %	✓	0.1330405 ± 0.0008510	0.00319778 ± 0.00003543	0.0295
17D17895	2.7 %	✓	0.1550404 ± 0.0007584	0.00315796 ± 0.00003243	0.0318
17D17896	3.0 %	✓	0.1662048 ± 0.0008519	0.00312946 ± 0.00003352	0.0350
17D17898	3.4 %	✓	0.1780033 ± 0.0007734	0.00309074 ± 0.00003418	0.0331
17D17899	3.9 %	✓	0.1863410 ± 0.0007964	0.00308741 ± 0.00003219	0.0335
17D17901	4.5 %	✓	0.1816403 ± 0.0007602	0.00312202 ± 0.00003499	0.0322
17D17902	5.2 %	✓	0.1767604 ± 0.0006031	0.00307698 ± 0.00003040	0.0355
17D17904	6.0 %	✓	0.1648155 ± 0.0006863	0.00311293 ± 0.00003202	0.0296
17D17905	6.9 %	✓	0.1542297 ± 0.0007464	0.00310714 ± 0.00003324	0.0288
17D17907	7.9 %	✓	0.1476519 ± 0.0007127	0.00314388 ± 0.00003290	0.0261
17D17908	9.0 %		0.1401984 ± 0.0008017	0.00320758 ± 0.00003513	0.0291
17D17910	10.3 %		0.1288767 ± 0.0007247	0.00318133 ± 0.00003287	0.0236
17D17911	11.6 %		0.1107575 ± 0.0006792	0.00325412 ± 0.00003450	0.0216
17D17912	12.5 %		0.0822962 ± 0.0005140	0.00328201 ± 0.00002934	0.0156
17D17914	13.4 %		0.0687483 ± 0.0005522	0.00332074 ± 0.00003182	0.0128
17D17915	14.6 %		0.0565356 ± 0.0003436	0.00332495 ± 0.00002706	0.0081
17D17916	16.0 %		0.0437617 ± 0.0002963	0.00331393 ± 0.00002616	0.0057
17D17918	17.6 %		0.0402045 ± 0.0004065	0.00333049 ± 0.00002880	0.0065
17D17919	19.3 %		0.0473298 ± 0.0007346	0.00332866 ± 0.00003586	0.0085
17D17921	21.0 %		0.0515475 ± 0.0023742	0.00333616 ± 0.00005754	0.0213

Results	40(a)/36(a) ± 2σ		40(r)/39(k)	± 2σ	Age ± 2σ (Ma)		MSWD
**Inverse Isochron**<br>Clustered Points	**293.73**	**± 6.31**<br>**± 2.15%**	0.50637	± 0.11529<br>± 22.77%	**1.52**	**± 0.35**<br>**± 22.76%**	1.32<br>21%
					Full External Error	± 0.35	
					Analytical Error	± 0.35	

Statistics				
	2σ Confidence Limit	1.89	Convergence	0.0000887893
	Error Magnification	1.1500	Number of Iterations	3
	Number of Data Points	12	Calculated Line	Weighted York-2
	Spreading Factor	4.0%		

Degassing Patterns			36Ar(a) [fA]	%1σ	36Ar(c) [fA]	%1σ	36Ar(ca) [fA]	%1σ	36Ar(cl) [fA]	%1σ	37Ar(ca) [fA]	%1σ	38Ar(a) [fA]	%1σ	38Ar(c) [fA]	%1σ	38Ar(k) [fA]	%1σ	38Ar(ca) [fA]	%1σ	38Ar(cl) [fA]	%1σ	39Ar(k) [fA]	%1σ	39Ar(ca) [fA]	%1σ	40Ar(r) [fA]	%1σ	40Ar(a) [fA]	%1σ	40Ar(c) [fA]	%1σ	40Ar(k) [fA]	%1σ
17D17887	1.8 %		0.3471488	0.40	0.0000000	0.00	0.0141464	0.75	0.0000017	283.56	52.3359	0.73	0.0648821	0.40	0.0000000	0.00	0.1237381	0.25	0.0094205	9.66	0.0085167	283.56	10.24577	0.24	0.0336258	1.18	7.488450	6.44	102.5825	0.46	0.0000000	0.00	0.0062192	9.65
17D17889	1.9 %		0.2911161	0.42	0.0000000	0.00	0.0172544	0.65	0.0000018	259.24	63.8343	0.62	0.0544096	0.42	0.0000000	0.00	0.1145805	0.27	0.0114902	9.65	0.0088262	259.24	9.48750	0.25	0.0410135	1.11	5.215675	7.95	86.0248	0.48	0.0000000	0.00	0.0057589	9.65
17D17890	2.0 %	✓	0.1923293	0.48	0.0000000	0.00	0.0129003	0.78	0.0000026	189.61	47.7260	0.76	0.0359463	0.48	0.0000000	0.00	0.0770650	0.37	0.0085907	9.66	0.0128886	189.62	6.38114	0.36	0.0306640	1.19	2.926190	10.52	56.8333	0.54	0.0000000	0.00	0.0038734	9.66
17D17892	2.2 %	✓	0.2096521	0.49	0.0000000	0.00	0.0204319	0.60	0.0000000	0.00	75.5897	0.58	0.0391840	0.49	0.0000000	0.00	0.1020192	0.28	0.0136062	9.65	0.0000000	0.00	8.44739	0.27	0.0485664	1.09	3.960251	8.64	61.9522	0.55	0.0000000	0.00	0.0051276	9.65
17D17893	2.4 %	✓	0.1763063	0.55	0.0000000	0.00	0.0195803	0.61	0.0000000	0.00	72.4392	0.59	0.0329517	0.55	0.0000000	0.00	0.0885854	0.32	0.0130391	9.65	0.0000000	0.00	7.33505	0.31	0.0465422	1.09	3.035426	10.35	52.0985	0.60	0.0000000	0.00	0.0044524	9.66
17D17895	2.7 %	✓	0.2055648	0.51	0.0000000	0.00	0.0303610	0.52	0.0000013	363.42	112.3232	0.49	0.0384201	0.51	0.0000000	0.00	0.1218837	0.25	0.0202182	9.64	0.0064766	363.42	10.09221	0.24	0.0721677	1.04	4.349700	7.90	60.7444	0.56	0.0000000	0.00	0.0061260	9.65
17D17896	3.0 %	✓	0.1849973	0.53	0.0000000	0.00	0.0320412	0.51	0.0000051	92.59	118.5394	0.48	0.0345760	0.53	0.0000000	0.00	0.1186584	0.26	0.0213371	9.64	0.0250296	92.59	9.82515	0.25	0.0761616	1.04	4.448029	7.21	54.6667	0.58	0.0000000	0.00	0.0059639	9.65
17D17898	3.4 %	✓	0.1979409	0.55	0.0000000	0.00	0.0415613	0.49	0.0000000	0.00	153.7601	0.46	0.0369952	0.55	0.0000000	0.00	0.1376765	0.23	0.0276768	9.64	0.0000000	0.00	11.39989	0.21	0.0987908	1.03	5.551640	6.34	58.4915	0.60	0.0000000	0.00	0.0069197	9.65
17D17899	3.9 %	✓	0.2052019	0.52	0.0000000	0.00	0.0493853	0.47	0.0000059	87.21	182.7055	0.44	0.0383522	0.52	0.0000000	0.00	0.1495737	0.22	0.0328870	9.64	0.0290478	87.22	12.38500	0.20	0.1173883	1.02	5.827027	5.97	60.6372	0.57	0.0000000	0.00	0.0075177	9.65
17D17901	4.5 %	✓	0.2066205	0.56	0.0000000	0.00	0.0531320	0.47	0.0000002	######	196.5668	0.44	0.0386174	0.56	0.0000000	0.00	0.1451808	0.22	0.0353820	9.64	0.0007925	######	12.02127	0.20	0.1262942	1.02	5.125346	7.25	61.0564	0.61	0.0000000	0.00	0.0072969	9.65
17D17902	5.2 %	✓	0.2583793	0.49	0.0000000	0.00	0.0726384	0.46	0.0000000	0.00	268.7324	0.42	0.0482911	0.49	0.0000000	0.00	0.1792575	0.18	0.0483718	9.64	0.0000000	0.00	14.84288	0.16	0.1726606	1.01	7.620665	5.49	76.3511	0.55	0.0000000	0.00	0.0090096	9.65
17D17904	6.0 %	✓	0.2281647	0.51	0.0000000	0.00	0.0641279	0.46	0.0000000	0.00	237.2470	0.43	0.0426440	0.51	0.0000000	0.00	0.1458937	0.22	0.0427045	9.64	0.0000000	0.00	12.08029	0.20	0.1524312	1.02	5.873176	6.51	67.4227	0.56	0.0000000	0.00	0.0073327	9.65
17D17905	6.9 %	✓	0.2140887	0.53	0.0000000	0.00	0.0582577	0.46	0.0000000	0.00	215.5299	0.43	0.0400132	0.53	0.0000000	0.00	0.1283396	0.25	0.0387954	9.64	0.0000000	0.00	10.62678	0.23	0.1384780	1.02	5.639052	6.57	63.2632	0.58	0.0000000	0.00	0.0064505	9.65
17D17907	7.9 %	✓	0.2175630	0.52	0.0000000	0.00	0.0578285	0.47	0.0000027	182.43	213.9420	0.43	0.0406625	0.52	0.0000000	0.00	0.1234004	0.25	0.0385096	9.64	0.0130597	182.43	10.21780	0.23	0.1374577	1.02	4.912115	7.52	64.2899	0.57	0.0000000	0.00	0.0062022	9.65
17D17908	9.0 %		0.1905034	0.54	0.0000000	0.00	0.0480395	0.48	0.0000000	0.00	177.7264	0.45	0.0356051	0.54	0.0000000	0.00	0.1005605	0.29	0.0319908	9.64	0.0000000	0.00	8.32661	0.28	0.1141892	1.02	3.097880	10.85	56.2938	0.59	0.0000000	0.00	0.0050543	9.65
17D17910	10.3 %		0.2175065	0.51	0.0000000	0.00	0.0483622	0.48	0.0000054	90.13	178.9203	0.45	0.0406520	0.51	0.0000000	0.00	0.1064135	0.29	0.0322057	9.64	0.0263888	90.14	8.81125	0.28	0.1149563	1.02	4.096438	8.93	64.2732	0.57	0.0000000	0.00	0.0053484	9.65
17D17911	11.6 %		0.2215268	0.53	0.0000000	0.00	0.0422346	0.49	0.0000028	182.67	156.2508	0.46	0.0414034	0.53	0.0000000	0.00	0.0910593	0.31	0.0281251	9.64	0.0136527	182.67	7.53989	0.30	0.1003911	1.03	2.614529	14.54	65.4612	0.58	0.0000000	0.00	0.0045767	9.65
17D17912	12.5 %		0.2854876	0.44	0.0000000	0.00	0.0433927	0.48	0.0000076	65.57	160.5354	0.45	0.0533576	0.44	0.0000000	0.00	0.0864541	0.32	0.0288964	9.64	0.0375625	65.57	7.15858	0.31	0.1031440	1.02	2.623905	16.27	84.3616	0.50	0.0000000	0.00	0.0043453	9.65
17D17914	13.4 %		0.2667281	0.48	0.0000000	0.00	0.0395362	0.49	0.0000015	313.40	146.2678	0.46	0.0498515	0.48	0.0000000	0.00	0.0666892	0.41	0.0263282	9.64	0.0074995	313.40	5.52200	0.40	0.0939771	1.03	1.503840	28.02	78.8181	0.53	0.0000000	0.00	0.0033519	9.66
17D17915	14.6 %		0.4404482	0.41	0.0000000	0.00	0.0664850	0.46	0.0000000	0.00	245.9673	0.43	0.0823198	0.41	0.0000000	0.00	0.0904464	0.32	0.0442741	9.64	0.0000000	0.00	7.48914	0.30	0.1580340	1.02	2.315264	26.47	130.1524	0.47	0.0000000	0.00	0.0045459	9.65
17D17916	16.0 %		0.5123013	0.39	0.0000000	0.00	0.0772142	0.46	0.0000000	0.00	285.6612	0.42	0.0957491	0.39	0.0000000	0.00	0.0817025	0.35	0.0514190	9.64	0.0000000	0.00	6.76513	0.34	0.1835373	1.01	3.205279	21.74	151.3850	0.46	0.0000000	0.00	0.0041064	9.66
17D17918	17.6 %		0.3728947	0.43	0.0000000	0.00	0.0605470	0.47	0.0000032	158.43	223.9991	0.43	0.0696940	0.43	0.0000000	0.00	0.0543641	0.51	0.0403198	9.64	0.0156275	158.43	4.50146	0.50	0.1439194	1.02	1.773638	30.64	110.1904	0.49	0.0000000	0.00	0.0027324	9.66
17D17919	19.3 %		0.2167761	0.54	0.0000000	0.00	0.0439079	0.48	0.0000000	0.00	162.4413	0.45	0.0405155	0.54	0.0000000	0.00	0.0372251	0.78	0.0292394	9.64	0.0000000	0.00	3.08232	0.77	0.1043686	1.02	1.066889	35.34	64.0573	0.59	0.0000000	0.00	0.0018710	9.68
17D17921	21.0 %		0.0606173	0.84	0.0000000	0.00	0.0128390	0.74	0.0000010	490.69	47.4990	0.72	0.0113294	0.84	0.0000000	0.00	0.0113114	2.30	0.0085498	9.66	0.0049376	490.69	0.93661	2.29	0.0305181	1.17	0.257356	62.30	17.9124	0.87	0.0000000	0.00	0.0005685	9.92
		Σ	5.9198639	0.10	0.0000000	0.00	1.0262048	0.11	0.0000428	42.83	3796.5402	0.10	1.1064226	0.10	0.0000000	0.00	2.4820786	0.06	0.6833772	2.15	0.2103062	42.83	205.52112	0.06	2.4392771	0.23	94.527760	2.11	1749.3198	0.11	0.0000000	0.00	0.1247513	2.09
		Σ							6.9461115	0.09	3796.5402	0.10									4.4821845	2.04			207.96040	0.06							1843.9723	0.15

Additional Parameters			40Ar/39Ar	1σ	37Ar/39Ar	1σ	36Ar/39Ar	1σ	Time (days)	37Ar (decay)	39Ar (decay)	40Ar (moles)
17D17887	1.8 %		10.708527	0.026673	5.091345	0.039146	0.035148	0.000157	125.150	11.874946	1.00088433	5.284E-12
17D17889	1.9 %		9.576131	0.024560	6.699295	0.044974	0.032363	0.000150	125.164	11.878204	1.00088443	4.380E-12
17D17890	2.0 %	✓	9.320838	0.033604	7.443463	0.062544	0.032009	0.000183	125.171	11.879833	1.00088448	2.869E-12
17D17892	2.2 %	✓	7.758695	0.021221	8.897138	0.056501	0.027082	0.000141	125.185	11.883093	1.00088458	3.164E-12
17D17893	2.4 %	✓	7.469719	0.023741	9.813502	0.065172	0.026537	0.000154	125.192	11.884723	1.00088463	2.647E-12
17D17895	2.7 %	✓	6.404740	0.015554	11.050669	0.060220	0.023211	0.000116	125.206	11.887984	1.00088472	3.125E-12
17D17896	3.0 %	✓	5.970995	0.015185	11.972086	0.064459	0.021921	0.000112	125.213	11.889614	1.00088477	2.838E-12
17D17898	3.4 %	✓	5.570211	0.011995	13.371970	0.066928	0.020829	0.000102	125.226	11.892877	1.00088487	3.074E-12
17D17899	3.9 %	✓	5.316719	0.011252	14.613642	0.071257	0.020364	0.000093	125.233	11.894508	1.00088492	3.191E-12
17D17901	4.5 %	✓	5.448749	0.011279	16.181587	0.077841	0.021383	0.000102	125.247	11.897772	1.00088502	3.177E-12
17D17902	5.2 %	✓	5.592924	0.009421	17.896955	0.081077	0.022045	0.000089	125.254	11.899404	1.00088507	4.031E-12
17D17904	6.0 %	✓	5.992383	0.012308	19.394448	0.091819	0.023894	0.000104	125.268	11.902668	1.00088516	3.519E-12
17D17905	6.9 %	✓	6.401029	0.015280	20.020880	0.097922	0.025299	0.000118	125.275	11.904301	1.00088521	3.308E-12
17D17907	7.9 %	✓	6.683385	0.015901	20.660225	0.101387	0.026595	0.000123	125.290	11.907731	1.00088532	3.322E-12
17D17908	9.0 %		7.036854	0.019838	21.055633	0.110109	0.028261	0.000142	125.297	11.909364	1.00088537	2.851E-12
17D17910	10.3 %		7.660022	0.021249	20.044379	0.104769	0.029786	0.000147	125.310	11.912632	1.00088546	3.282E-12
17D17911	11.6 %		8.910697	0.026949	20.450906	0.111237	0.034523	0.000182	125.317	11.914266	1.00088551	3.268E-12
17D17912	12.5 %		11.979233	0.036852	22.107080	0.120142	0.045291	0.000221	125.324	11.915900	1.00088556	4.176E-12
17D17914	13.4 %		14.303002	0.056446	26.044940	0.158115	0.054535	0.000309	125.338	11.919170	1.00088566	3.856E-12
17D17915	14.6 %		17.323029	0.051432	32.164466	0.167991	0.066290	0.000302	125.345	11.920805	1.00088571	6.359E-12
17D17916	16.0 %		22.248062	0.073097	41.110200	0.219844	0.084839	0.000399	125.352	11.922440	1.00088576	7.421E-12
17D17918	17.6 %		24.102831	0.117856	48.219780	0.314385	0.093307	0.000568	125.366	11.925711	1.00088586	5.374E-12
17D17919	19.3 %		20.436950	0.153293	50.975040	0.445178	0.081804	0.000709	125.373	11.927347	1.00088591	3.126E-12
17D17921	21.0 %		18.788002	0.419067	49.113612	1.147438	0.075954	0.001764	125.387	11.930620	1.00088600	8.722E-13

Procedure Blanks		36Ar ± 1σ (SE) [fA]	37Ar ± 1σ (SE) [fA]	38Ar ± 1σ (SE) [fA]	39Ar ± 1σ (SE) [fA]	40Ar ± 1σ (SE) [fA]
17D17887	1.8 %	0.0037463 ± 0.0001627	0.1402745 ± 0.0180613	0.0377749 ± 0.0167677	0.0059387 ± 0.0153959	1.0586128 ± 0.0338369
17D17889	1.9 %	0.0037168 ± 0.0001627	0.1418771 ± 0.0180613	0.0492509 ± 0.0167677	0.0158479 ± 0.0153959	1.1138918 ± 0.0338369
17D17890	2.0 %	0.0037380 ± 0.0001627	0.1415927 ± 0.0180613	0.0535486 ± 0.0167677	0.0183205 ± 0.0153959	1.1289928 ± 0.0338369
17D17892	2.2 %	0.0038229 ± 0.0001627	0.1395148 ± 0.0180613	0.0596773 ± 0.0167677	0.0195611 ± 0.0153959	1.1396415 ± 0.0338369
17D17893	2.4 %	0.0038765 ± 0.0001627	0.1379556 ± 0.0180613	0.0616643 ± 0.0167677	0.0187837 ± 0.0153959	1.1372043 ± 0.0338369
17D17895	2.7 %	0.0039840 ± 0.0001627	0.1343069 ± 0.0180613	0.0638507 ± 0.0167677	0.0154478 ± 0.0153959	1.1213583 ± 0.0338369
17D17896	3.0 %	0.0040308 ± 0.0001627	0.1323909 ± 0.0180613	0.0641874 ± 0.0167677	0.0132461 ± 0.0153959	1.1095757 ± 0.0338369
17D17898	3.4 %	0.0040963 ± 0.0001627	0.1287333 ± 0.0180613	0.0636680 ± 0.0167677	0.0085445 ± 0.0153959	1.0818705 ± 0.0338369
17D17899	3.9 %	0.0041109 ± 0.0001627	0.1271044 ± 0.0180613	0.0629306 ± 0.0167677	0.0063033 ± 0.0153959	1.0671852 ± 0.0338369
17D17901	4.5 %	0.0040972 ± 0.0001627	0.1244517 ± 0.0180613	0.0607738 ± 0.0167677	0.0025650 ± 0.0153959	1.0387562 ± 0.0338369
17D17902	5.2 %	0.0040679 ± 0.0001627	0.1234797 ± 0.0180613	0.0594544 ± 0.0167677	0.0012287 ± 0.0153959	1.0258610 ± 0.0338369
17D17904	6.0 %	0.0039652 ± 0.0001627	0.1222977 ± 0.0180613	0.0565601 ± 0.0167677	0.0000989 ± 0.0153959	1.0043436 ± 0.0338369
17D17905	6.9 %	0.0038937 ± 0.0001627	0.1220787 ± 0.0180613	0.0550664 ± 0.0167677	0.0000271 ± 0.0153959	0.9961810 ± 0.0338369
17D17907	7.9 %	0.0037098 ± 0.0001627	0.1223147 ± 0.0180613	0.0520276 ± 0.0167677	0.0017557 ± 0.0153959	0.9853974 ± 0.0338369
17D17908	9.0 %	0.0036114 ± 0.0001627	0.1226833 ± 0.0180613	0.0506931 ± 0.0167677	0.0033430 ± 0.0153959	0.9833577 ± 0.0338369
17D17910	10.3 %	0.0034103 ± 0.0001627	0.1236566 ± 0.0180613	0.0483762 ± 0.0167677	0.0077261 ± 0.0153959	0.9849289 ± 0.0338369
17D17911	11.6 %	0.0033158 ± 0.0001627	0.1241282 ± 0.0180613	0.0474372 ± 0.0167677	0.0103856 ± 0.0153959	0.9882084 ± 0.0338369
17D17912	12.5 %	0.0032315 ± 0.0001627	0.1244844 ± 0.0180613	0.0466681 ± 0.0167677	0.0132391 ± 0.0153959	0.9928217 ± 0.0338369
17D17914	13.4 %	0.0031154 ± 0.0001627	0.1244726 ± 0.0180613	0.0456917 ± 0.0167677	0.0190754 ± 0.0153959	1.0046740 ± 0.0338369
17D17915	14.6 %	0.0030958 ± 0.0001627	0.1238907 ± 0.0180613	0.0455028 ± 0.0167677	0.0217914 ± 0.0153959	1.0110632 ± 0.0338369
17D17916	16.0 %	0.0031107 ± 0.0001627	0.1227649 ± 0.0180613	0.0455203 ± 0.0167677	0.0241675 ± 0.0153959	1.0170865 ± 0.0338369
17D17918	17.6 %	0.0032741 ± 0.0001627	0.1183425 ± 0.0180613	0.0461773 ± 0.0167677	0.0271866 ± 0.0153959	1.0256230 ± 0.0338369
17D17919	19.3 %	0.0034388 ± 0.0001627	0.1147504 ± 0.0180613	0.0468100 ± 0.0167677	0.0274321 ± 0.0153959	1.0267678 ± 0.0338369
17D17921	21.0 %	0.0039784 ± 0.0001627	0.1039904 ± 0.0180613	0.0486441 ± 0.0167677	0.0242788 ± 0.0153959	1.0189036 ± 0.0338369

**Intercept Values**		36Ar ± 1σ (SE) [fA]	r2	Regression (type,n)	37Ar ± 1σ (SE) [fA]	r2	Regression (type,n)	38Ar ± 1σ (SE) [fA]	r2	Regression (type,n)	39Ar ± 1σ (SE) [fA]	r2	Regression (type,n)	40Ar ± 1σ (SE) [fA]	r2	Regression (type,n)
17D17887	1.8 %	0.3516405 ± 0.0009128	0.7245	EXP 150 of 150	4.1892357 ± 0.0191206	0.5308	EXP 150 of 150	0.1663502 ± 0.0169505	0.0090	EXP 150 of 150	10.2040075 ± 0.0172924	0.9196	EXP 150 of 150	111.135751 ± 0.079559	0.8242	EXP 150 of 150
17D17889	1.9 %	0.3006497 ± 0.0008178	0.7796	EXP 150 of 150	5.1373916 ± 0.0170905	0.7278	EXP 150 of 150	0.1378264 ± 0.0151220	0.0161	EXP 150 of 150	9.4482879 ± 0.0168386	0.9136	EXP 150 of 150	92.360144 ± 0.039074	0.9248	EXP 150 of 150
17D17890	2.0 %	0.2013569 ± 0.0006869	0.5980	EXP 150 of 150	3.8049363 ± 0.0178727	0.6265	EXP 150 of 150	0.0793583 ± 0.0173561	0.0198	EXP 150 of 150	6.3501644 ± 0.0158992	0.8148	EXP 148 of 150	60.892365 ± 0.023555	0.9911	EXP 150 of 150
17D17892	2.2 %	0.2253717 ± 0.0007702	0.6541	EXP 150 of 150	6.1093891 ± 0.0179840	0.7817	EXP 150 of 150	0.0554841 ± 0.0193137	0.0138	EXP 150 of 150	8.4190015 ± 0.0153009	0.9106	EXP 150 of 150	67.057213 ± 0.026062	0.9828	EXP 150 of 150
17D17893	2.4 %	0.1924965 ± 0.0007529	0.5379	EXP 150 of 150	5.8496795 ± 0.0177557	0.7842	EXP 150 of 150	0.0711942 ± 0.0176164	0.0000	EXP 150 of 150	7.3129355 ± 0.0159360	0.8616	EXP 150 of 150	56.275607 ± 0.021058	0.9924	EXP 150 of 150
17D17895	2.7 %	0.2311591 ± 0.0007658	0.6708	EXP 150 of 150	9.1474845 ± 0.0180368	0.8986	EXP 150 of 150	0.1209458 ± 0.0159971	0.0043	EXP 150 of 150	10.0802618 ± 0.0166755	0.9299	EXP 150 of 150	66.221580 ± 0.023374	0.9857	EXP 150 of 150
17D17896	3.0 %	0.2130229 ± 0.0007221	0.5602	EXP 150 of 150	9.6617294 ± 0.0168602	0.9177	EXP 150 of 150	0.1330633 ± 0.0154560	0.0173	EXP 150 of 150	9.8211739 ± 0.0172932	0.9205	EXP 150 of 150	60.230283 ± 0.023092	0.9894	EXP 150 of 150
17D17898	3.4 %	0.2347139 ± 0.0007993	0.6350	EXP 150 of 150	12.5719518 ± 0.0186220	0.9396	EXP 150 of 150	0.1273269 ± 0.0169574	0.0022	EXP 150 of 150	11.4124521 ± 0.0160612	0.9451	EXP 150 of 150	65.131969 ± 0.022080	0.9860	EXP 150 of 150
17D17899	3.9 %	0.2492596 ± 0.0007247	0.6901	EXP 150 of 150	14.9624230 ± 0.0190659	0.9544	EXP 150 of 150	0.1839879 ± 0.0183132	0.0376	EXP 150 of 150	12.4116196 ± 0.0180977	0.9435	EXP 150 of 150	67.538900 ± 0.022428	0.9860	EXP 150 of 150
17D17901	4.5 %	0.2542141 ± 0.0008255	0.6870	EXP 150 of 150	16.1054188 ± 0.0188683	0.9587	EXP 150 of 150	0.1566086 ± 0.0160557	0.0047	EXP 150 of 150	12.0629215 ± 0.0163396	0.9517	EXP 150 of 150	67.227769 ± 0.022565	0.9821	EXP 150 of 150
17D17902	5.2 %	0.3228061 ± 0.0007829	0.8248	EXP 150 of 150	22.0618248 ± 0.0182578	0.9805	EXP 150 of 150	0.1741478 ± 0.0161311	0.0050	EXP 150 of 150	14.9128588 ± 0.0151175	0.9742	EXP 150 of 150	85.006633 ± 0.031072	0.9340	EXP 150 of 150
17D17904	6.0 %	0.2854148 ± 0.0007541	0.7691	EXP 149 of 150	19.4583389 ± 0.0197085	0.9688	EXP 150 of 150	0.1407399 ± 0.0171867	0.0000	EXP 150 of 150	12.1501731 ± 0.0165334	0.9517	EXP 150 of 150	74.307520 ± 0.023610	0.9715	EXP 150 of 150
17D17905	6.9 %	0.2661372 ± 0.0007731	0.7146	EXP 150 of 150	17.6637526 ± 0.0170189	0.9726	EXP 150 of 150	0.1415202 ± 0.0178497	0.0008	EXP 150 of 150	10.6925476 ± 0.0179173	0.9274	EXP 150 of 150	69.904901 ± 0.024983	0.9770	EXP 150 of 150
17D17907	7.9 %	0.2688879 ± 0.0007579	0.7448	EXP 150 of 150	17.5273902 ± 0.0181009	0.9691	EXP 150 of 150	0.1610654 ± 0.0161066	0.0177	EXP 150 of 150	10.2835347 ± 0.0167301	0.9322	EXP 149 of 150	70.193572 ± 0.020811	0.9787	EXP 150 of 150
17D17908	9.0 %	0.2333053 ± 0.0007274	0.7064	EXP 150 of 150	14.5373162 ± 0.0190226	0.9502	EXP 150 of 150	0.1113422 ± 0.0166682	0.0067	EXP 150 of 150	8.3804269 ± 0.0160721	0.8990	EXP 150 of 150	60.380054 ± 0.021474	0.9861	EXP 150 of 150
17D17910	10.3 %	0.2594215 ± 0.0007719	0.7203	EXP 150 of 150	14.6307758 ± 0.0198682	0.9475	EXP 150 of 150	0.1548620 ± 0.0161747	0.0593	EXP 150 of 150	8.8581711 ± 0.0174902	0.9019	EXP 150 of 150	69.359897 ± 0.021406	0.9707	EXP 150 of 150
17D17911	11.6 %	0.2572953 ± 0.0008488	0.7126	EXP 150 of 150	12.7591195 ± 0.0189654	0.9379	EXP 150 of 150	0.1247515 ± 0.0178551	0.0107	EXP 150 of 150	7.5782751 ± 0.0156156	0.8846	EXP 150 of 150	69.068481 ± 0.021982	0.9697	EXP 150 of 150
17D17912	12.5 %	0.3199191 ± 0.0008282	0.8198	EXP 150 of 150	13.1102299 ± 0.0175931	0.9468	EXP 150 of 150	0.1571736 ± 0.0174148	0.0156	EXP 150 of 150	7.1994147 ± 0.0148604	0.8780	EXP 150 of 150	87.982670 ± 0.022385	0.7666	EXP 150 of 150
17D17914	13.4 %	0.2980200 ± 0.0008908	0.7884	EXP 150 of 150	11.9306929 ± 0.0195149	0.9253	EXP 150 of 150	0.1029060 ± 0.0158688	0.0066	EXP 150 of 150	5.5589542 ± 0.0150353	0.8079	EXP 149 of 150	81.330008 ± 0.021065	0.8482	EXP 149 of 150
17D17915	14.6 %	0.4912237 ± 0.0010420	0.8917	EXP 150 of 150	20.1455762 ± 0.0213204	0.9672	EXP 150 of 150	0.1407197 ± 0.0161974	0.0078	EXP 149 of 150	7.5737112 ± 0.0154951	0.8904	EXP 150 of 150	133.483305 ± 0.024597	0.9763	EXP 150 of 150
17D17916	16.0 %	0.5707575 ± 0.0011180	0.9112	EXP 150 of 150	23.4145300 ± 0.0186188	0.9816	EXP 150 of 150	0.1561709 ± 0.0176136	0.0001	EXP 150 of 150	6.8775480 ± 0.0158854	0.8493	EXP 150 of 150	155.611499 ± 0.025956	0.9886	EXP 150 of 150
17D17918	17.6 %	0.4206398 ± 0.0010028	0.8542	EXP 150 of 150	18.3331900 ± 0.0194835	0.9680	EXP 150 of 150	0.1317085 ± 0.0173900	0.0076	EXP 149 of 150	4.5868009 ± 0.0161087	0.6940	EXP 150 of 150	112.992373 ± 0.025061	0.9018	EXP 150 of 150
17D17919	19.3 %	0.2544524 ± 0.0008457	0.7367	EXP 150 of 150	13.2642357 ± 0.0183113	0.9468	EXP 150 of 150	0.0512101 ± 0.0171785	0.0015	EXP 150 of 150	3.1377184 ± 0.0178488	0.3730	EXP 150 of 150	66.152870 ± 0.019121	0.9750	EXP 150 of 150
17D17921	21.0 %	0.0747106 ± 0.0004087	0.0014	EXP 150 of 150	3.8070439 ± 0.0149200	0.7027	EXP 150 of 150	0.0129413 ± 0.0170683	0.0012	EXP 150 of 150	0.9363102 ± 0.0147553	0.0015	EXP 150 of 150	19.189235 ± 0.015945	0.9971	EXP 150 of 150

Project Info		Analyst	Irradiation	X-pos	Y-pos	Z/H-pos	Project	Experiment	Nmb
17D17887	1.8 %	Dan Miggins	17-OSU-01	0.00	0.00	3.66	Arctic\O-Connor (16-22)	17D17883	01
17D17889	1.9 %	Dan Miggins	17-OSU-01	0.00	0.00	3.66	Arctic\O-Connor (16-22)	17D17883	01
17D17890	2.0 %	Dan Miggins	17-OSU-01	0.00	0.00	3.66	Arctic\O-Connor (16-22)	17D17883	01
17D17892	2.2 %	Dan Miggins	17-OSU-01	0.00	0.00	3.66	Arctic\O-Connor (16-22)	17D17883	01
17D17893	2.4 %	Dan Miggins	17-OSU-01	0.00	0.00	3.66	Arctic\O-Connor (16-22)	17D17883	01
17D17895	2.7 %	Dan Miggins	17-OSU-01	0.00	0.00	3.66	Arctic\O-Connor (16-22)	17D17883	01
17D17896	3.0 %	Dan Miggins	17-OSU-01	0.00	0.00	3.66	Arctic\O-Connor (16-22)	17D17883	01
17D17898	3.4 %	Dan Miggins	17-OSU-01	0.00	0.00	3.66	Arctic\O-Connor (16-22)	17D17883	01
17D17899	3.9 %	Dan Miggins	17-OSU-01	0.00	0.00	3.66	Arctic\O-Connor (16-22)	17D17883	01
17D17901	4.5 %	Dan Miggins	17-OSU-01	0.00	0.00	3.66	Arctic\O-Connor (16-22)	17D17883	01
17D17902	5.2 %	Dan Miggins	17-OSU-01	0.00	0.00	3.66	Arctic\O-Connor (16-22)	17D17883	01
17D17904	6.0 %	Dan Miggins	17-OSU-01	0.00	0.00	3.66	Arctic\O-Connor (16-22)	17D17883	01
17D17905	6.9 %	Dan Miggins	17-OSU-01	0.00	0.00	3.66	Arctic\O-Connor (16-22)	17D17883	01
17D17907	7.9 %	Dan Miggins	17-OSU-01	0.00	0.00	3.66	Arctic\O-Connor (16-22)	17D17883	01
17D17908	9.0 %	Dan Miggins	17-OSU-01	0.00	0.00	3.66	Arctic\O-Connor (16-22)	17D17883	01
17D17910	10.3 %	Dan Miggins	17-OSU-01	0.00	0.00	3.66	Arctic\O-Connor (16-22)	17D17883	01
17D17911	11.6 %	Dan Miggins	17-OSU-01	0.00	0.00	3.66	Arctic\O-Connor (16-22)	17D17883	01
17D17912	12.5 %	Dan Miggins	17-OSU-01	0.00	0.00	3.66	Arctic\O-Connor (16-22)	17D17883	01
17D17914	13.4 %	Dan Miggins	17-OSU-01	0.00	0.00	3.66	Arctic\O-Connor (16-22)	17D17883	01
17D17915	14.6 %	Dan Miggins	17-OSU-01	0.00	0.00	3.66	Arctic\O-Connor (16-22)	17D17883	01
17D17916	16.0 %	Dan Miggins	17-OSU-01	0.00	0.00	3.66	Arctic\O-Connor (16-22)	17D17883	01
17D17918	17.6 %	Dan Miggins	17-OSU-01	0.00	0.00	3.66	Arctic\O-Connor (16-22)	17D17883	01
17D17919	19.3 %	Dan Miggins	17-OSU-01	0.00	0.00	3.66	Arctic\O-Connor (16-22)	17D17883	01
17D17921	21.0 %	Dan Miggins	17-OSU-01	0.00	0.00	3.66	Arctic\O-Connor (16-22)	17D17883	01

Sample Parameters		Sample	Material	Location	Standard Name	Standard (in Ma)	%1σ	Standard Reference	Standard 40Ar/39Ar	%1σ	J	%1σ	Air 40Ar/36Ar	%1σ	MDF (lin)	%1σ	Volume Ratio	Sensitivity (mol/volt)	Day	Month	Year	Hour	Min	Resist
17D17887	1.8 %	HLY0102-D89-004	Groundmass	Gakkel Ridge	FCT-NM (1C2-17)	28.201	0.082	Kuiper et al (2008)	9.46726	0.09	0.00166018	0.090	302.619	0.133	0.9941112	0.067	1	4.8E-14	24	MAY	2017	19	15	1
17D17889	1.9 %	HLY0102-D89-004	Groundmass	Gakkel Ridge	FCT-NM (1C2-17)	28.201	0.082	Kuiper et al (2008)	9.46726	0.09	0.00166018	0.090	302.619	0.133	0.9941112	0.067	1	4.8E-14	24	MAY	2017	19	35	1
17D17890	2.0 %	HLY0102-D89-004	Groundmass	Gakkel Ridge	FCT-NM (1C2-17)	28.201	0.082	Kuiper et al (2008)	9.46726	0.09	0.00166018	0.090	302.619	0.133	0.9941112	0.067	1	4.8E-14	24	MAY	2017	19	45	1
17D17892	2.2 %	HLY0102-D89-004	Groundmass	Gakkel Ridge	FCT-NM (1C2-17)	28.201	0.082	Kuiper et al (2008)	9.46726	0.09	0.00166018	0.090	302.619	0.133	0.9941112	0.067	1	4.8E-14	24	MAY	2017	20	5	1
17D17893	2.4 %	HLY0102-D89-004	Groundmass	Gakkel Ridge	FCT-NM (1C2-17)	28.201	0.082	Kuiper et al (2008)	9.46726	0.09	0.00166018	0.090	302.619	0.133	0.9941112	0.067	1	4.8E-14	24	MAY	2017	20	15	1
17D17895	2.7 %	HLY0102-D89-004	Groundmass	Gakkel Ridge	FCT-NM (1C2-17)	28.201	0.082	Kuiper et al (2008)	9.46726	0.09	0.00166018	0.090	302.619	0.133	0.9941112	0.067	1	4.8E-14	24	MAY	2017	20	35	1
17D17896	3.0 %	HLY0102-D89-004	Groundmass	Gakkel Ridge	FCT-NM (1C2-17)	28.201	0.082	Kuiper et al (2008)	9.46726	0.09	0.00166018	0.090	302.619	0.133	0.9941112	0.067	1	4.8E-14	24	MAY	2017	20	45	1
17D17898	3.4 %	HLY0102-D89-004	Groundmass	Gakkel Ridge	FCT-NM (1C2-17)	28.201	0.082	Kuiper et al (2008)	9.46726	0.09	0.00166018	0.090	302.619	0.133	0.9941112	0.067	1	4.8E-14	24	MAY	2017	21	5	1
17D17899	3.9 %	HLY0102-D89-004	Groundmass	Gakkel Ridge	FCT-NM (1C2-17)	28.201	0.082	Kuiper et al (2008)	9.46726	0.09	0.00166018	0.090	302.619	0.133	0.9941112	0.067	1	4.8E-14	24	MAY	2017	21	15	1
17D17901	4.5 %	HLY0102-D89-004	Groundmass	Gakkel Ridge	FCT-NM (1C2-17)	28.201	0.082	Kuiper et al (2008)	9.46726	0.09	0.00166018	0.090	302.619	0.133	0.9941112	0.067	1	4.8E-14	24	MAY	2017	21	35	1
17D17902	5.2 %	HLY0102-D89-004	Groundmass	Gakkel Ridge	FCT-NM (1C2-17)	28.201	0.082	Kuiper et al (2008)	9.46726	0.09	0.00166018	0.090	302.619	0.133	0.9941112	0.067	1	4.8E-14	24	MAY	2017	21	45	1
17D17904	6.0 %	HLY0102-D89-004	Groundmass	Gakkel Ridge	FCT-NM (1C2-17)	28.201	0.082	Kuiper et al (2008)	9.46726	0.09	0.00166018	0.090	302.619	0.133	0.9941112	0.067	1	4.8E-14	24	MAY	2017	22	5	1
17D17905	6.9 %	HLY0102-D89-004	Groundmass	Gakkel Ridge	FCT-NM (1C2-17)	28.201	0.082	Kuiper et al (2008)	9.46726	0.09	0.00166018	0.090	302.619	0.133	0.9941112	0.067	1	4.8E-14	24	MAY	2017	22	15	1
17D17907	7.9 %	HLY0102-D89-004	Groundmass	Gakkel Ridge	FCT-NM (1C2-17)	28.201	0.082	Kuiper et al (2008)	9.46726	0.09	0.00166018	0.090	302.619	0.133	0.9941112	0.067	1	4.8E-14	24	MAY	2017	22	36	1
17D17908	9.0 %	HLY0102-D89-004	Groundmass	Gakkel Ridge	FCT-NM (1C2-17)	28.201	0.082	Kuiper et al (2008)	9.46726	0.09	0.00166018	0.090	302.619	0.133	0.9941112	0.067	1	4.8E-14	24	MAY	2017	22	46	1
17D17910	10.3 %	HLY0102-D89-004	Groundmass	Gakkel Ridge	FCT-NM (1C2-17)	28.201	0.082	Kuiper et al (2008)	9.46726	0.09	0.00166018	0.090	302.619	0.133	0.9941112	0.067	1	4.8E-14	24	MAY	2017	23	6	1
17D17911	11.6 %	HLY0102-D89-004	Groundmass	Gakkel Ridge	FCT-NM (1C2-17)	28.201	0.082	Kuiper et al (2008)	9.46726	0.09	0.00166018	0.090	302.619	0.133	0.9941112	0.067	1	4.8E-14	24	MAY	2017	23	16	1
17D17912	12.5 %	HLY0102-D89-004	Groundmass	Gakkel Ridge	FCT-NM (1C2-17)	28.201	0.082	Kuiper et al (2008)	9.46726	0.09	0.00166018	0.090	302.619	0.133	0.9941112	0.067	1	4.8E-14	24	MAY	2017	23	26	1
17D17914	13.4 %	HLY0102-D89-004	Groundmass	Gakkel Ridge	FCT-NM (1C2-17)	28.201	0.082	Kuiper et al (2008)	9.46726	0.09	0.00166018	0.090	302.619	0.133	0.9941112	0.067	1	4.8E-14	24	MAY	2017	23	46	1
17D17915	14.6 %	HLY0102-D89-004	Groundmass	Gakkel Ridge	FCT-NM (1C2-17)	28.201	0.082	Kuiper et al (2008)	9.46726	0.09	0.00166018	0.090	302.619	0.133	0.9941112	0.067	1	4.8E-14	24	MAY	2017	23	56	1
17D17916	16.0 %	HLY0102-D89-004	Groundmass	Gakkel Ridge	FCT-NM (1C2-17)	28.201	0.082	Kuiper et al (2008)	9.46726	0.09	0.00166018	0.090	302.619	0.133	0.9941112	0.067	1	4.8E-14	25	MAY	2017	0	6	1
17D17918	17.6 %	HLY0102-D89-004	Groundmass	Gakkel Ridge	FCT-NM (1C2-17)	28.201	0.082	Kuiper et al (2008)	9.46726	0.09	0.00166018	0.090	302.619	0.133	0.9941112	0.067	1	4.8E-14	25	MAY	2017	0	26	1
17D17919	19.3 %	HLY0102-D89-004	Groundmass	Gakkel Ridge	FCT-NM (1C2-17)	28.201	0.082	Kuiper et al (2008)	9.46726	0.09	0.00166018	0.090	302.619	0.133	0.9941112	0.067	1	4.8E-14	25	MAY	2017	0	36	1
17D17921	21.0 %	HLY0102-D89-004	Groundmass	Gakkel Ridge	FCT-NM (1C2-17)	28.201	0.082	Kuiper et al (2008)	9.46726	0.09	0.00166018	0.090	302.619	0.133	0.9941112	0.067	1	4.8E-14	25	MAY	2017	0	56	1

Irradiation Constants		40/36(a)	%1σ	40/36(c)	%1σ	38/36(a)	%1σ	38/36(c)	%1σ	39/37(ca)	%1σ	38/37(ca)	%1σ	36/37(ca)	%1σ	40/39(k)	%1σ	38/39(k)	%1σ	36/38(cl)	%1σ	K/Ca	%1σ	K/Cl	%1σ	Ca/Cl	%1σ
17D17887	1.8 %	295.5	0.237	0.018	35	0.1869	0	1.493	3	0.000643	0.92	0.00018	9.63	0.00027	0.17	0.000607	9.65	0.012077	0.09	0	0	0.43	0	0	0	0	0
17D17889	1.9 %	295.5	0.237	0.018	35	0.1869	0	1.493	3	0.000643	0.92	0.00018	9.63	0.00027	0.17	0.000607	9.65	0.012077	0.09	0	0	0.43	0	0	0	0	0
17D17890	2.0 %	295.5	0.237	0.018	35	0.1869	0	1.493	3	0.000643	0.92	0.00018	9.63	0.00027	0.17	0.000607	9.65	0.012077	0.09	0	0	0.43	0	0	0	0	0
17D17892	2.2 %	295.5	0.237	0.018	35	0.1869	0	1.493	3	0.000643	0.92	0.00018	9.63	0.00027	0.17	0.000607	9.65	0.012077	0.09	0	0	0.43	0	0	0	0	0
17D17893	2.4 %	295.5	0.237	0.018	35	0.1869	0	1.493	3	0.000643	0.92	0.00018	9.63	0.00027	0.17	0.000607	9.65	0.012077	0.09	0	0	0.43	0	0	0	0	0
17D17895	2.7 %	295.5	0.237	0.018	35	0.1869	0	1.493	3	0.000643	0.92	0.00018	9.63	0.00027	0.17	0.000607	9.65	0.012077	0.09	0	0	0.43	0	0	0	0	0
17D17896	3.0 %	295.5	0.237	0.018	35	0.1869	0	1.493	3	0.000643	0.92	0.00018	9.63	0.00027	0.17	0.000607	9.65	0.012077	0.09	0	0	0.43	0	0	0	0	0
17D17898	3.4 %	295.5	0.237	0.018	35	0.1869	0	1.493	3	0.000643	0.92	0.00018	9.63	0.00027	0.17	0.000607	9.65	0.012077	0.09	0	0	0.43	0	0	0	0	0
17D17899	3.9 %	295.5	0.237	0.018	35	0.1869	0	1.493	3	0.000643	0.92	0.00018	9.63	0.00027	0.17	0.000607	9.65	0.012077	0.09	0	0	0.43	0	0	0	0	0
17D17901	4.5 %	295.5	0.237	0.018	35	0.1869	0	1.493	3	0.000643	0.92	0.00018	9.63	0.00027	0.17	0.000607	9.65	0.012077	0.09	0	0	0.43	0	0	0	0	0
17D17902	5.2 %	295.5	0.237	0.018	35	0.1869	0	1.493	3	0.000643	0.92	0.00018	9.63	0.00027	0.17	0.000607	9.65	0.012077	0.09	0	0	0.43	0	0	0	0	0
17D17904	6.0 %	295.5	0.237	0.018	35	0.1869	0	1.493	3	0.000643	0.92	0.00018	9.63	0.00027	0.17	0.000607	9.65	0.012077	0.09	0	0	0.43	0	0	0	0	0
17D17905	6.9 %	295.5	0.237	0.018	35	0.1869	0	1.493	3	0.000643	0.92	0.00018	9.63	0.00027	0.17	0.000607	9.65	0.012077	0.09	0	0	0.43	0	0	0	0	0
17D17907	7.9 %	295.5	0.237	0.018	35	0.1869	0	1.493	3	0.000643	0.92	0.00018	9.63	0.00027	0.17	0.000607	9.65	0.012077	0.09	0	0	0.43	0	0	0	0	0
17D17908	9.0 %	295.5	0.237	0.018	35	0.1869	0	1.493	3	0.000643	0.92	0.00018	9.63	0.00027	0.17	0.000607	9.65	0.012077	0.09	0	0	0.43	0	0	0	0	0
17D17910	10.3 %	295.5	0.237	0.018	35	0.1869	0	1.493	3	0.000643	0.92	0.00018	9.63	0.00027	0.17	0.000607	9.65	0.012077	0.09	0	0	0.43	0	0	0	0	0
17D17911	11.6 %	295.5	0.237	0.018	35	0.1869	0	1.493	3	0.000643	0.92	0.00018	9.63	0.00027	0.17	0.000607	9.65	0.012077	0.09	0	0	0.43	0	0	0	0	0
17D17912	12.5 %	295.5	0.237	0.018	35	0.1869	0	1.493	3	0.000643	0.92	0.00018	9.63	0.00027	0.17	0.000607	9.65	0.012077	0.09	0	0	0.43	0	0	0	0	0
17D17914	13.4 %	295.5	0.237	0.018	35	0.1869	0	1.493	3	0.000643	0.92	0.00018	9.63	0.00027	0.17	0.000607	9.65	0.012077	0.09	0	0	0.43	0	0	0	0	0
17D17915	14.6 %	295.5	0.237	0.018	35	0.1869	0	1.493	3	0.000643	0.92	0.00018	9.63	0.00027	0.17	0.000607	9.65	0.012077	0.09	0	0	0.43	0	0	0	0	0
17D17916	16.0 %	295.5	0.237	0.018	35	0.1869	0	1.493	3	0.000643	0.92	0.00018	9.63	0.00027	0.17	0.000607	9.65	0.012077	0.09	0	0	0.43	0	0	0	0	0
17D17918	17.6 %	295.5	0.237	0.018	35	0.1869	0	1.493	3	0.000643	0.92	0.00018	9.63	0.00027	0.17	0.000607	9.65	0.012077	0.09	0	0	0.43	0	0	0	0	0
17D17919	19.3 %	295.5	0.237	0.018	35	0.1869	0	1.493	3	0.000643	0.92	0.00018	9.63	0.00027	0.17	0.000607	9.65	0.012077	0.09	0	0	0.43	0	0	0	0	0
17D17921	21.0 %	295.5	0.237	0.018	35	0.1869	0	1.493	3	0.000643	0.92	0.00018	9.63	0.00027	0.17	0.000607	9.65	0.012077	0.09	0	0	0.43	0	0	0	0	0

17D17883.AGE >>> HLY0102-D89-004 >>> ARCTIC | O-CONNOR (16-22) PROJECT

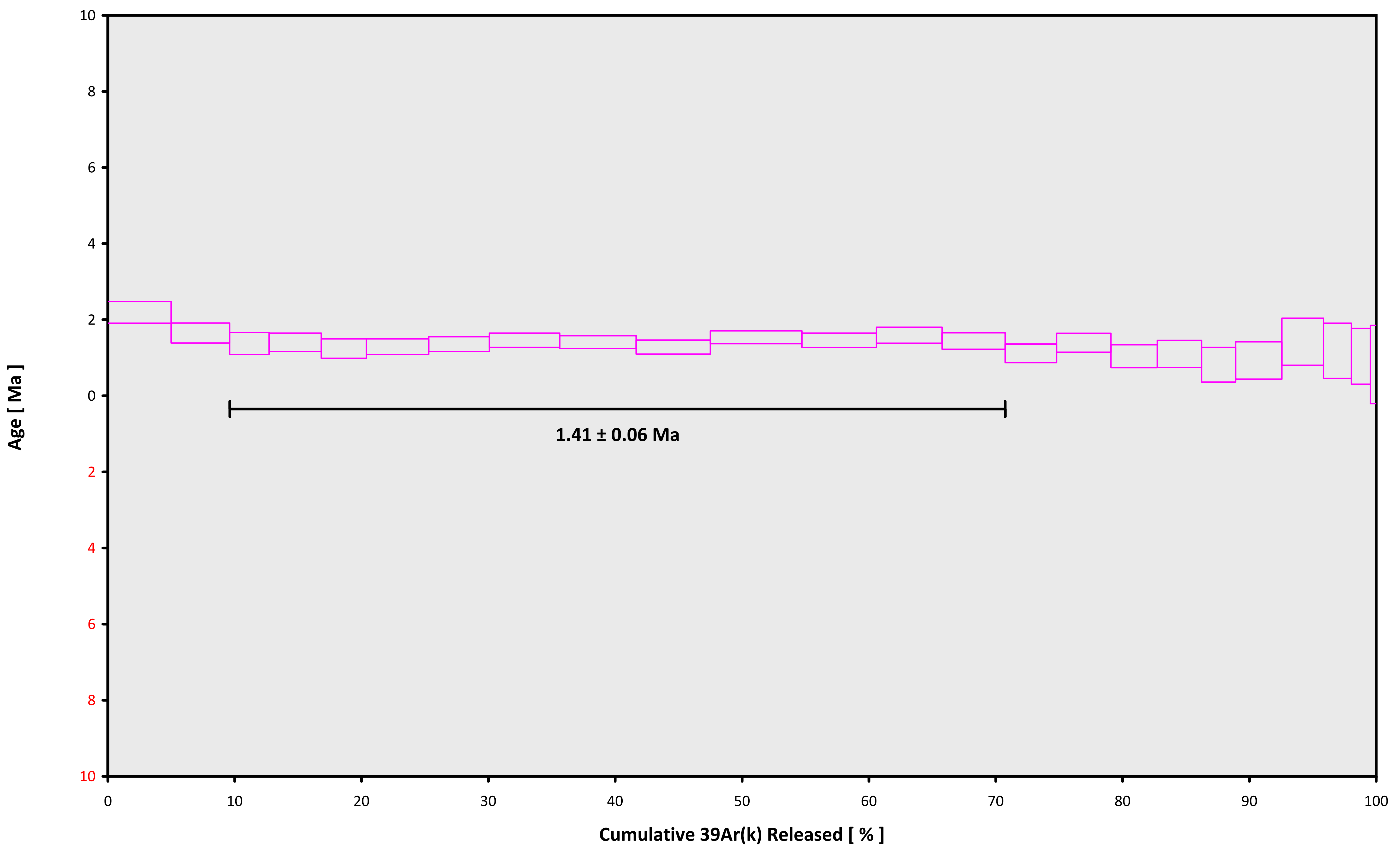

Ar-Ages in Ma

WEIGHTED PLATEAU
1.41 ± 0.06
TOTAL FUSION
1.38 ± 0.06
NORMAL ISOCHRON
1.52 ± 0.38
INVERSE ISOCHRON
1.52 ± 0.35

MSWD (PROBABILITY)
1.03 (41%)

Sample Info

Groundmass
Gakkel Ridge
Dan Miggins

IRR = 17-OSU-01 (1C2-17)
J = 0.00166018 ± 0.00000149

17D17883.AGE >>> HLY0102-D89-004 >>> ARCTIC | O-CONNOR (16-22) PROJECT

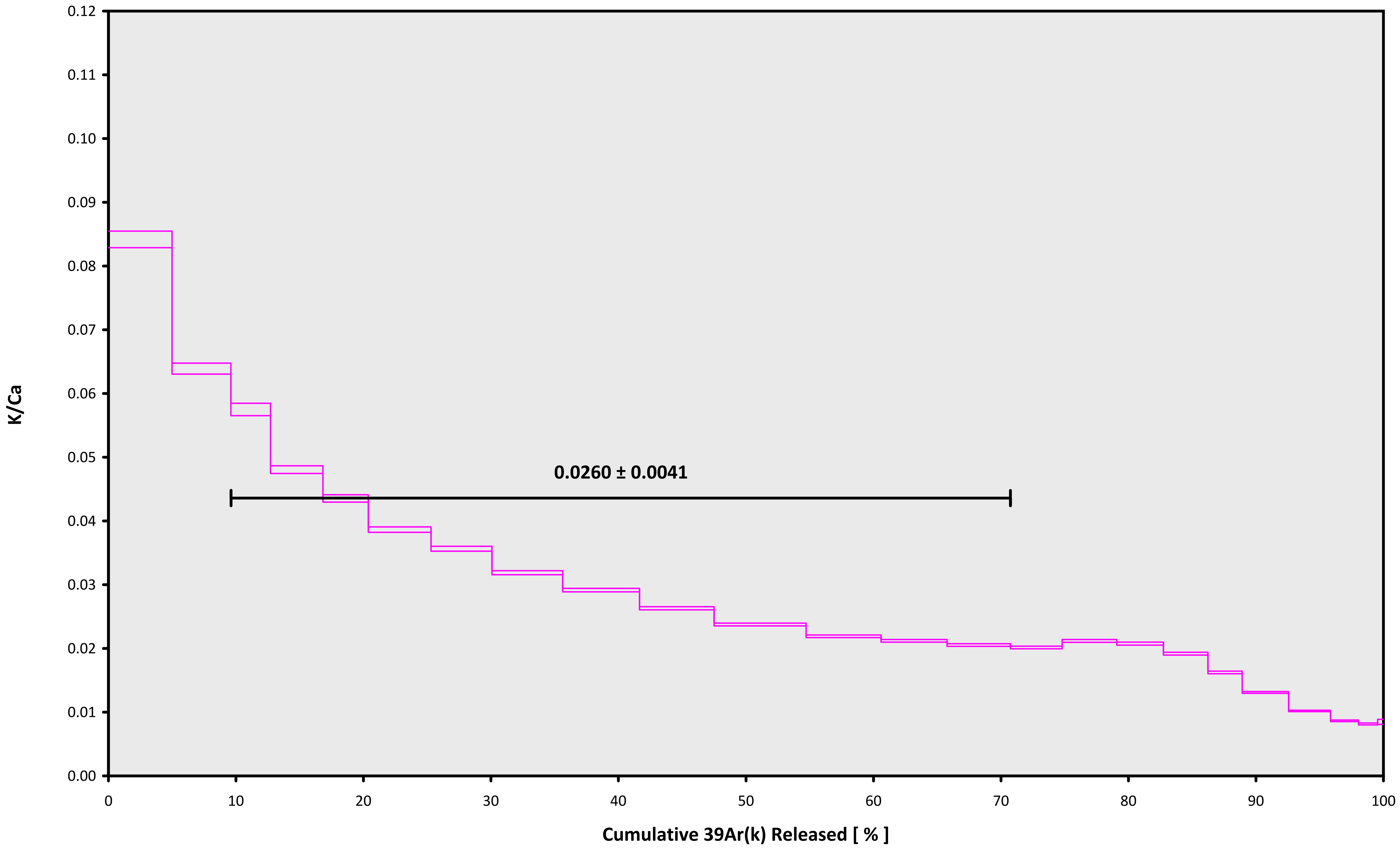

Ar-Ages in Ma

WEIGHTED PLATEAU
1.41 ± 0.06
TOTAL FUSION
1.38 ± 0.06
NORMAL ISOCHRON
1.52 ± 0.38
INVERSE ISOCHRON
1.52 ± 0.35

Sample Info

Groundmass
Gakkel Ridge
Dan Miggins

IRR = 17-OSU-01 (1C2-17)
J = 0.00166018 ± 0.00000149

17D17883.AGE >>> HLY0102-D89-004 >>> ARCTIC | O-CONNOR (16-22) PROJECT

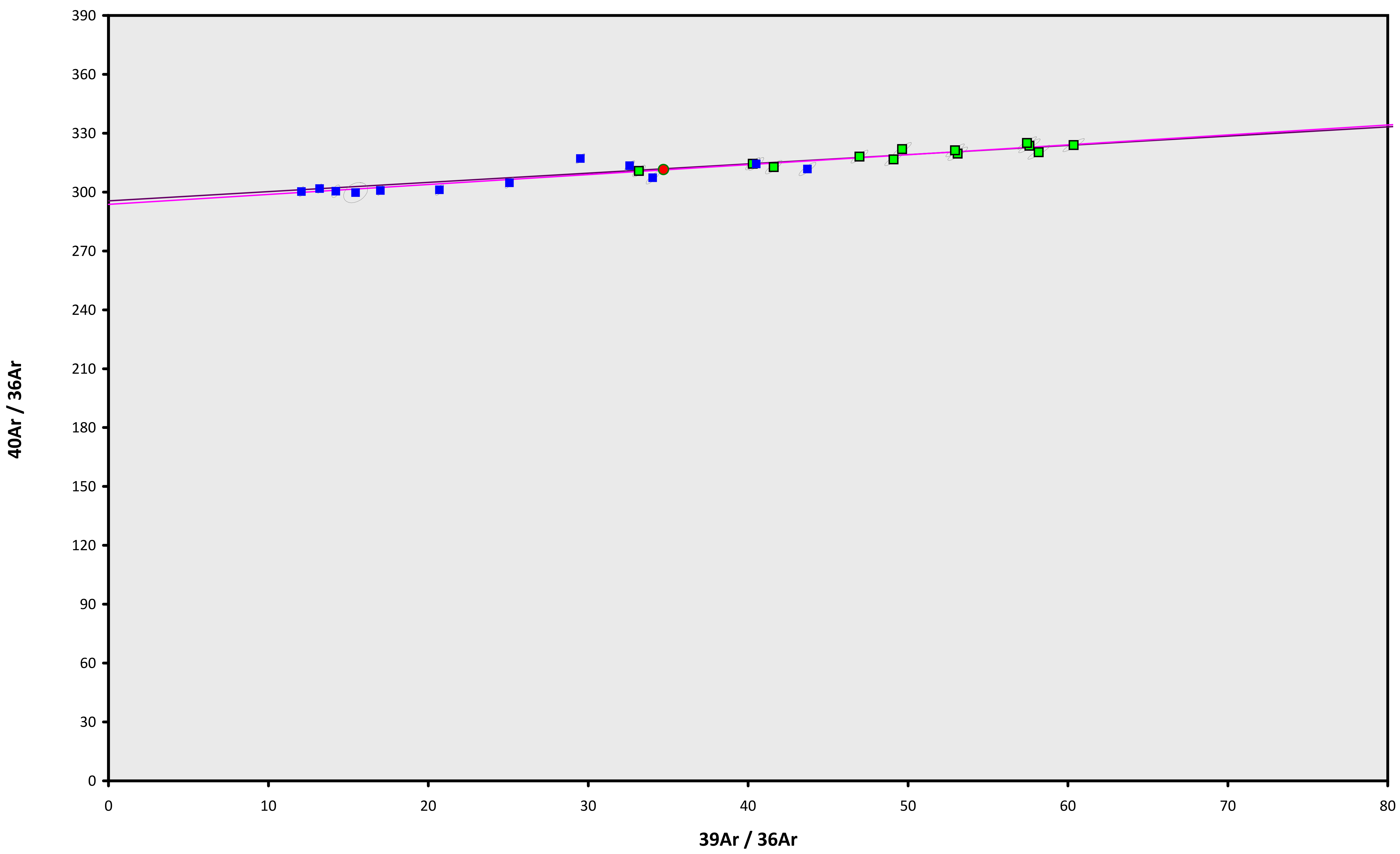

Ar-Ages in Ma

WEIGHTED PLATEAU
1.41 ± 0.06
TOTAL FUSION
1.38 ± 0.06
NORMAL ISOCHRON
1.52 ± 0.38
INVERSE ISOCHRON
1.52 ± 0.35

MSWD (PROBABILITY)
1.32 (21%)

40AR/36AR INTERCEPT
293.7 ± 6.3

Sample Info

Groundmass
Gakkel Ridge
Dan Miggins

IRR = 17-OSU-01 (1C2-17)
J = 0.00166018 ± 0.00000149

17D17883.AGE >>> HLY0102-D89-004 >>> ARCTIC | O-CONNOR (16-22) PROJECT

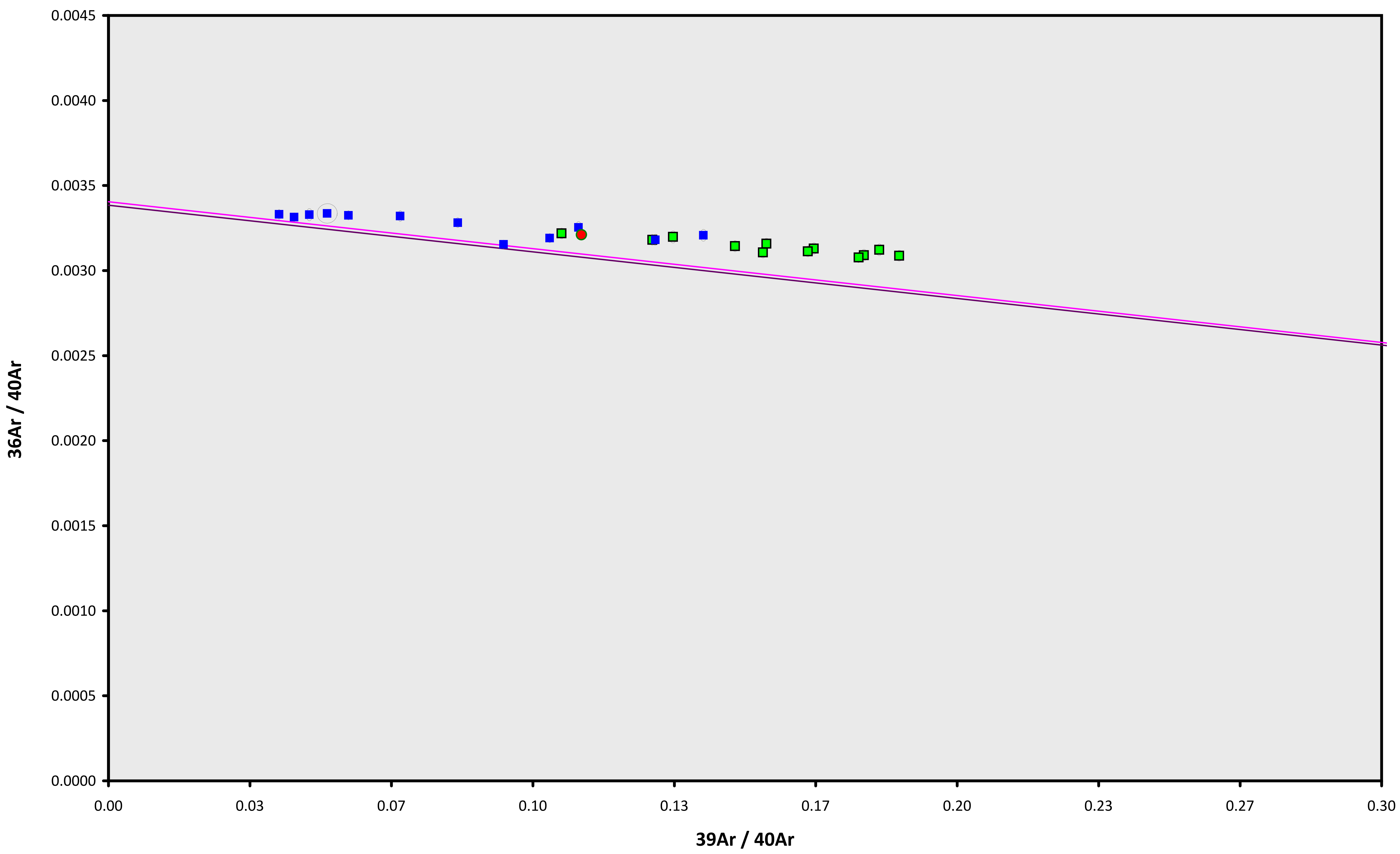

Ar-Ages in Ma

WEIGHTED PLATEAU
1.41 ± 0.06
TOTAL FUSION
1.38 ± 0.06
NORMAL ISOCHRON
1.52 ± 0.38
INVERSE ISOCHRON
1.52 ± 0.35

MSWD (PROBABILITY)
1.32 (21%)

SPREADING FACTOR
4.0%

40AR/36AR INTERCEPT
293.7 ± 6.3

Sample Info

Groundmass
Gakkel Ridge
Dan Miggins

IRR = 17-OSU-01 (1C2-17)
J = 0.00166018 ± 0.00000149

Relative Abundances			36Ar [fA]	%1σ	37Ar [fA]	%1σ	38Ar [fA]	%1σ	39Ar [fA]	%1σ	40Ar [fA]	%1σ	40(r)/39(k) ± 2σ	Age ± 2σ (Ma)	40Ar(r) (%)	39Ar(k) (%)	K/Ca ± 2σ
17D30285	1.8 %	✓	5.558419	0.288	46.0371	0.820	1.6753942	1.507	40.15543	0.093	1658.3840	0.007	0.48679 ± 0.23604	1.44 ± 0.70	1.18	15.40	0.3748 ± 0.0062
17D30287	1.9 %	✓	2.576616	0.300	48.6995	0.785	0.8757575	2.727	24.29756	0.120	770.2121	0.013	0.52348 ± 0.18862	1.55 ± 0.56	1.65	9.32	0.2143 ± 0.0034
17D30288	2.0 %	✓	1.231939	0.317	34.2283	1.009	0.4620805	5.387	13.59291	0.186	368.9813	0.028	0.56539 ± 0.17085	1.67 ± 0.51	2.08	5.21	0.1705 ± 0.0035
17D30290	2.2 %	✓	0.931733	0.323	39.7283	0.910	0.3497072	6.920	11.39598	0.208	279.2100	0.034	0.62014 ± 0.15729	1.84 ± 0.47	2.53	4.37	0.1231 ± 0.0023
17D30291	2.4 %	✓	0.709186	0.340	42.4935	0.808	0.2735539	8.644	9.70111	0.248	212.6130	0.046	0.66549 ± 0.14866	1.97 ± 0.44	3.03	3.71	0.0979 ± 0.0017
17D30293	2.7 %	✓	0.714299	0.353	66.6109	0.638	0.3086884	7.877	11.12080	0.227	213.1149	0.045	0.66394 ± 0.13596	1.97 ± 0.40	3.45	4.25	0.0715 ± 0.0010
17D30294	3.0 %	✓	0.475872	0.378	58.0497	0.696	0.2075322	11.269	8.51754	0.267	143.0550	0.073	0.83333 ± 0.12816	2.47 ± 0.38	4.94	3.26	0.0628 ± 0.0009
17D30296	3.4 %	✓	0.518774	0.369	84.9500	0.570	0.2125827	11.598	10.25381	0.228	153.2925	0.066	0.66415 ± 0.11303	1.97 ± 0.33	4.42	3.92	0.0516 ± 0.0006
17D30297	3.9 %	✓	0.552097	0.370	139.2096	0.469	0.2607735	9.120	13.49119	0.174	161.2630	0.065	0.68867 ± 0.09175	2.04 ± 0.27	5.72	5.14	0.0414 ± 0.0004
17D30299	4.5 %	✓	0.446362	0.373	157.0114	0.455	0.2872493	8.291	13.08799	0.193	127.9535	0.080	0.66129 ± 0.07808	1.96 ± 0.23	6.71	4.99	0.0356 ± 0.0004
17D30300	5.2 %	✓	0.456990	0.385	225.2272	0.435	0.3232463	7.536	16.33802	0.158	127.1058	0.081	0.62037 ± 0.06635	1.84 ± 0.20	7.90	6.22	0.0309 ± 0.0003
17D30302	6.0 %	✓	0.366460	0.395	227.3175	0.430	0.2548667	9.324	14.95723	0.165	100.1749	0.098	0.67744 ± 0.06039	2.01 ± 0.18	10.02	5.69	0.0280 ± 0.0003
17D30303	6.9 %	✓	0.328650	0.410	236.0414	0.429	0.2665706	8.917	14.61991	0.171	86.3978	0.110	0.56167 ± 0.05789	1.66 ± 0.17	9.41	5.55	0.0264 ± 0.0002
17D30305	7.9 %	✓	0.273306	0.430	215.4787	0.433	0.2228076	10.681	12.88486	0.185	71.0719	0.132	0.58943 ± 0.05791	1.75 ± 0.17	10.57	4.89	0.0254 ± 0.0002
17D30306	9.0 %	✓	0.249656	0.459	199.5951	0.438	0.1888019	13.026	11.52956	0.204	64.3270	0.145	0.56916 ± 0.06301	1.69 ± 0.19	10.09	4.38	0.0246 ± 0.0002
17D30308	10.3 %	✓	0.205432	0.489	185.6782	0.443	0.2005827	12.167	9.42433	0.255	50.7518	0.181	0.52371 ± 0.06851	1.55 ± 0.20	9.60	3.57	0.0215 ± 0.0002
17D30309	11.6 %	✓	0.157291	0.532	149.6168	0.459	0.1558820	15.145	6.68620	0.346	37.9743	0.241	0.52242 ± 0.08206	1.55 ± 0.24	9.07	2.53	0.0189 ± 0.0002
17D30311	12.5 %		0.107468	0.677	98.1053	0.524	0.1104013	20.614	4.63234	0.479	26.0388	0.350	0.46323 ± 0.10409	1.37 ± 0.31	8.13	1.75	0.0200 ± 0.0003
17D30312	13.4 %		0.088343	0.794	70.6562	0.624	0.0757449	31.274	3.38143	0.631	21.7527	0.421	0.38652 ± 0.13769	1.14 ± 0.41	5.93	1.28	0.0203 ± 0.0004
17D30314	14.6 %		0.095483	0.731	80.8105	0.566	0.0652333	36.479	3.10978	0.733	23.1343	0.393	0.44862 ± 0.14976	1.33 ± 0.44	5.93	1.17	0.0163 ± 0.0003
17D30315	16.0 %		0.089466	0.787	80.8326	0.574	0.0548765	44.268	2.61085	0.863	21.1025	0.432	0.43771 ± 0.18028	1.30 ± 0.53	5.31	0.98	0.0136 ± 0.0003
17D30317	17.6 %		0.097950	0.719	110.6665	0.512	0.0887450	28.801	2.64048	0.863	21.1147	0.432	0.39298 ± 0.18092	1.16 ± 0.54	4.78	0.99	0.0100 ± 0.0002
17D30318	19.3 %		0.093753	0.747	124.1751	0.490	0.0643698	37.873	2.33528	0.981	18.5940	0.490	0.35793 ± 0.20575	1.06 ± 0.61	4.34	0.87	0.0078 ± 0.0002
17D30320	21.0 %		0.056173	1.036	65.4760	0.637	0.0255733	94.352	1.51254	1.474	11.7451	0.781	0.25501 ± 0.26935	0.76 ± 0.80	3.19	0.56	0.0097 ± 0.0003
		Σ	16.381718	0.119	2786.6954	0.111	7.0110212	1.685	262.27716	0.046	4769.3648	0.010					

Information on Analysis and Constants Used in Calculations

Project = **O-CONNOR (16-22)**
Sample = **HLY0102-D85-90**
Material = **Groundmass**
Location = **Gakkel Ridge**
Region = **Artic Ocean**
Analyst = **Dan Miggins**
Irradiation = **17-OSU-05 (5B6-17)**
Position = **X: 0 | Y: 0 | Z/H: 9.527553 mm**
FCT-NM Age = **28.201 ± 0.023 Ma**
FCT-NM Reference = **Kuiper et al (2008)**
FCT-NM 40Ar/39Ar Ratio = **9.59452 ± 0.00950**
FCT-NM J-value = **0.00163816 ± 0.00000162**
Air Shot 40Ar/36Ar = **302.8530 ± 0.4573**
Air Shot MDF = **0.99392238 ± 0.00068637 (LIN)**
Experiment Type = **Incremental Heating**
Extraction Method = **Bulk Laser Heating**
Heating = **64 sec**
Isolation = **3.00 min**
Instrument = **ARGUS-VI-D**
Preferred Age = **Plateau Age**
Age Classification = **Crystallization Age**
IGSN = Undefined
Rock Class = **Igneous>Volcanic**
Lithology = **Basaltic Lava**
Lat-Lon = Undefined - Undefined

Age Equations = **Min et al. (2000)**
Negative Intensities = **Allowed**
Collector Calibrations = **36Ar**
Decay 40K = **5.530 ± 0.048 E-10 1/a**
Decay 39Ar = **2.940 ± 0.016 E-07 1/h**
Decay 37Ar = **8.230 ± 0.012 E-04 1/h**
Decay 36Cl = **2.257 ± 0.015 E-06 1/a**
Decay 40K(EC,β⁺) = **0.580 ± 0.009 E-10 1/a**
Decay 40K(β⁻) = **4.950 ± 0.043 E-10 1/a**
Atmospheric 40/36(a) = **295.50**
Atmospheric 38/36(a) = **0.1869**
Production 39/37(ca) = **0.0006425 ± 0.0000059**
Production 38/37(ca) = **0.0001800 ± 0.0000173**
Production 36/37(ca) = **0.0002703 ± 0.0000005**
Production 40/39(k) = **0.000607 ± 0.000059**
Production 38/39(k) = **0.012077 ± 0.000011**
Production 36/38(cl) = **262.80 ± 1.71**
Scaling Ratio K/Ca = **0.430**
Abundance Ratio 40K/K = **1.1700 ± 0.0100 E-04**
Atomic Weight K = **39.0983 ± 0.0001 g**

Results

Results	40(a)/36(a) ± 2σ	40(r)/39(k) ± 2σ	Age ± 2σ (Ma)	MSWD	39Ar(k) (%,n)	K/Ca ± 2σ
**Age Plateau**		0.60740 ± 0.03341	**1.80** ± 0.10	2.63	92.39	0.0276 ± 0.0061
Error Mean		± 5.50%	± 5.50%	0%	17	
			Full External Error ± 0.11	1.71	2σ Confidence Limit	
			Analytical Error ± 0.10	1.6209	Error Magnification	
**Total Fusion Age**		0.57978 ± 0.04452	**1.72** ± 0.13		24	0.0402 ± 0.0001
		± 7.68%	± 7.68%			
			Full External Error ± 0.14			
			Analytical Error ± 0.13			
**Normal Isochron**	296.30 ± 1.59	0.58326 ± 0.05649	**1.73** ± 0.17	2.62	92.39	
Error Chron	± 0.54%	± 9.69%	± 9.68%	0%	17	
			Full External Error ± 0.17	1.73	2σ Confidence Limit	
			Analytical Error ± 0.17	1.6174	Error Magnification	
				5	Number of Iterations	
				0.0000006683	Convergence	
**Inverse Isochron**	296.30 ± 1.60	0.58456 ± 0.05575	**1.73** ± 0.17	2.63	92.39	
Error Chron	± 0.54%	± 9.54%	± 9.53%	0%	17	
			Full External Error ± 0.17	1.73	2σ Confidence Limit	
			Analytical Error ± 0.16	1.6204	Error Magnification	
**Notes**				3	Number of Iterations	
Bumpy spectrum				0.0000649926	Convergence	
				9%	Spreading Factor	

Incremental Heating			36Ar(a) [fA]	37Ar(ca) [fA]	38Ar(cl) [fA]	39Ar(k) [fA]	40Ar(r) [fA]	Age ± 2σ (Ma)	40Ar(r) (%)	39Ar(k) (%)	K/Ca ± 2σ
17D30285	1.8 %	✓	5.545945	46.0371	0.1459704	40.12585	19.53280	1.44 ± 0.70	1.18	15.40	0.3748 ± 0.0062
17D30287	1.9 %	✓	2.563433	48.6995	0.0948221	24.26627	12.70288	1.55 ± 0.56	1.65	9.32	0.2143 ± 0.0034
17D30288	2.0 %	✓	1.222674	34.2283	0.0635056	13.57092	7.67292	1.67 ± 0.51	2.08	5.21	0.1705 ± 0.0035
17D30290	2.2 %	✓	0.920987	39.7283	0.0331026	11.37045	7.05132	1.84 ± 0.47	2.53	4.37	0.1231 ± 0.0023
17D30291	2.4 %	✓	0.697697	42.4935	0.0186750	9.67381	6.43778	1.97 ± 0.44	3.03	3.71	0.0979 ± 0.0017
17D30293	2.7 %	✓	0.696288	66.6109	0.0327731	11.07801	7.35515	1.97 ± 0.40	3.45	4.25	0.0715 ± 0.0010
17D30294	3.0 %	✓	0.460179	58.0497	0.0086599	8.48025	7.06687	2.47 ± 0.38	4.94	3.26	0.0628 ± 0.0009
17D30296	3.4 %	✓	0.495812	84.9500	0.0000000	10.19923	6.77378	1.97 ± 0.33	4.42	3.92	0.0516 ± 0.0006
17D30297	3.9 %	✓	0.514469	139.2096	0.0000000	13.40175	9.22935	2.04 ± 0.27	5.72	5.14	0.0414 ± 0.0004
17D30299	4.5 %	✓	0.403916	157.0114	0.0266499	12.98711	8.58829	1.96 ± 0.23	6.71	4.99	0.0356 ± 0.0004
17D30300	5.2 %	✓	0.396109	225.2272	0.0131060	16.19331	10.04580	1.84 ± 0.20	7.90	6.22	0.0309 ± 0.0003
17D30302	6.0 %	✓	0.305016	227.3175	0.0000000	14.81118	10.03363	2.01 ± 0.18	10.02	5.69	0.0280 ± 0.0003
17D30303	6.9 %	✓	0.264848	236.0414	0.0000000	14.46825	8.12641	1.66 ± 0.17	9.41	5.55	0.0264 ± 0.0002
17D30305	7.9 %	✓	0.215062	215.4787	0.0000000	12.74642	7.51316	1.75 ± 0.17	10.57	4.89	0.0254 ± 0.0002
17D30306	9.0 %	✓	0.195705	199.5951	0.0000000	11.40132	6.48920	1.69 ± 0.19	10.09	4.38	0.0246 ± 0.0002
17D30308	10.3 %	✓	0.155238	185.6782	0.0257697	9.30503	4.87316	1.55 ± 0.20	9.60	3.57	0.0215 ± 0.0002
17D30309	11.6 %	✓	0.116844	149.6168	0.0275244	6.59008	3.44279	1.55 ± 0.24	9.07	2.53	0.0189 ± 0.0002
17D30311	12.5 %		0.080946	98.1053	0.0224301	4.56931	2.11663	1.37 ± 0.31	8.13	1.75	0.0200 ± 0.0003
17D30312	13.4 %		0.069243	70.6562	0.0097960	3.33604	1.28946	1.14 ± 0.41	5.93	1.28	0.0203 ± 0.0004
17D30314	14.6 %		0.073640	80.8105	0.0000000	3.05786	1.37180	1.33 ± 0.44	5.93	1.17	0.0163 ± 0.0003
17D30315	16.0 %		0.067617	80.8326	0.0000000	2.55892	1.12005	1.30 ± 0.53	5.31	0.98	0.0136 ± 0.0003
17D30317	17.6 %		0.068032	110.6665	0.0250795	2.56938	1.00971	1.16 ± 0.54	4.78	0.99	0.0100 ± 0.0002
17D30318	19.3 %		0.060187	124.1751	0.0035297	2.25550	0.80731	1.06 ± 0.61	4.34	0.87	0.0078 ± 0.0002
17D30320	21.0 %		0.038475	65.4760	0.0000000	1.47047	0.37498	0.76 ± 0.80	3.19	0.56	0.0097 ± 0.0003
		Σ	15.628364	2786.6954	0.5513940	260.48671	151.02525				

Information on Analysis

Project = **O-CONNOR (16-22)**
Sample = **HLY0102-D85-90**
Material = **Groundmass**
Location = **Gakkel Ridge**
Region = **Artic Ocean**
Analyst = **Dan Miggins**
Irradiation = **17-OSU-05 (5B6-17)**
J = **0.00163816 ± 0.00000162**
FCT-NM = **28.201 ± 0.023 Ma**

Results

Results	40(r)/39(k) ± 2σ		Age ± 2σ (Ma)		MSWD	39Ar(k) (%,n)	K/Ca ± 2σ
**Age Plateau**	0.60740	± 0.03341	**1.80**	**± 0.10**	2.63	92.39	0.0276 ± 0.0061
Error Mean		± 5.50%		**± 5.50%**	0%	17	
			Full External Error	± 0.11	1.71	2σ Confidence Limit	
			Analytical Error	± 0.10	1.6209	Error Magnification	
**Total Fusion Age**	0.57978	± 0.04452	**1.72**	**± 0.13**		24	0.0402 ± 0.0001
		± 7.68%		**± 7.68%**			
			Full External Error	± 0.14			
			Analytical Error	± 0.13			

Normal Isochron			39(k)/36(a) ± 2σ	40(a+r)/36(a) ± 2σ	r.i.
17D30285	1.8 %	✓	7.24 ± 0.04	299.02 ± 1.73	0.9515
17D30287	1.9 %	✓	9.47 ± 0.06	300.46 ± 1.82	0.9283
17D30288	2.0 %	✓	11.10 ± 0.08	301.78 ± 1.94	0.8610
17D30290	2.2 %	✓	12.35 ± 0.10	303.16 ± 1.99	0.8385
17D30291	2.4 %	✓	13.87 ± 0.12	304.73 ± 2.12	0.8040
17D30293	2.7 %	✓	15.91 ± 0.14	306.06 ± 2.24	0.8408
17D30294	3.0 %	✓	18.43 ± 0.17	310.86 ± 2.48	0.8114
17D30296	3.4 %	✓	20.57 ± 0.19	309.16 ± 2.43	0.8479
17D30297	3.9 %	✓	26.05 ± 0.23	313.44 ± 2.53	0.9032
17D30299	4.5 %	✓	32.15 ± 0.30	316.76 ± 2.68	0.8896
17D30300	5.2 %	✓	40.88 ± 0.39	320.86 ± 2.94	0.9276
17D30302	6.0 %	✓	48.56 ± 0.50	328.40 ± 3.24	0.9265
17D30303	6.9 %	✓	54.63 ± 0.60	326.18 ± 3.47	0.9283
17D30305	7.9 %	✓	59.27 ± 0.70	330.43 ± 3.81	0.9236
17D30306	9.0 %	✓	58.26 ± 0.74	328.66 ± 4.05	0.9191
17D30308	10.3 %	✓	59.94 ± 0.86	326.89 ± 4.50	0.8992
17D30309	11.6 %	✓	56.40 ± 0.92	324.96 ± 5.03	0.8575
17D30311	12.5 %		56.45 ± 1.17	321.65 ± 6.32	0.8254
17D30312	13.4 %		48.18 ± 1.17	314.12 ± 6.98	0.7858
17D30314	14.6 %		41.52 ± 1.01	314.13 ± 6.54	0.7325
17D30315	16.0 %		37.84 ± 1.04	312.06 ± 7.14	0.7123
17D30317	17.6 %		37.77 ± 1.05	310.34 ± 7.12	0.7108
17D30318	19.3 %		37.47 ± 1.18	308.91 ± 8.00	0.7059
17D30320	21.0 %		38.22 ± 1.65	305.25 ± 10.56	0.6364

Results	40(a)/36(a) ± 2σ		40(r)/39(k) ± 2σ		Age ± 2σ (Ma)		MSWD
Normal Isochron<br>Error Chron	**296.30**	**± 1.59**<br>**± 0.54%**	0.58326	± 0.05649<br>± 9.69%	**1.73**	**± 0.17**<br>**± 9.68%**	2.62<br>0%
					Full External Error	± 0.17	
					Analytical Error	± 0.17	

Statistics				
	2σ Confidence Limit	1.73	Convergence	0.000000668317
	Error Magnification	1.6174	Number of Iterations	5
	Number of Data Points	17	Calculated Line	Weighted York-2

Inverse Isochron			39(k)/40(a+r) ± 2σ	36(a)/40(a+r) ± 2σ	r.i.
17D30285	1.8 %	✓	0.0241961 ± 0.0000452	0.00334424 ± 0.00001933	0.0016
17D30287	1.9 %	✓	0.0315066 ± 0.0000761	0.00332828 ± 0.00002011	0.0048
17D30288	2.0 %	✓	0.0367803 ± 0.0001383	0.00331372 ± 0.00002126	0.0131
17D30290	2.2 %	✓	0.0407247 ± 0.0001720	0.00329863 ± 0.00002167	0.0167
17D30291	2.4 %	✓	0.0455009 ± 0.0002304	0.00328162 ± 0.00002287	0.0243
17D30293	2.7 %	✓	0.0519830 ± 0.0002411	0.00326730 ± 0.00002390	0.0241
17D30294	3.0 %	✓	0.0592818 ± 0.0003293	0.00321692 ± 0.00002565	0.0485
17D30296	3.4 %	✓	0.0665371 ± 0.0003179	0.00323455 ± 0.00002541	0.0466
17D30297	3.9 %	✓	0.0831091 ± 0.0003110	0.00319041 ± 0.00002576	0.0558
17D30299	4.5 %	✓	0.1015049 ± 0.0004268	0.00315694 ± 0.00002674	0.0711
17D30300	5.2 %	✓	0.1274101 ± 0.0004562	0.00311661 ± 0.00002852	0.0810
17D30302	6.0 %	✓	0.1478665 ± 0.0005723	0.00304511 ± 0.00003006	0.1009
17D30303	6.9 %	✓	0.1674780 ± 0.0006878	0.00306576 ± 0.00003264	0.1102
17D30305	7.9 %	✓	0.1793650 ± 0.0008210	0.00302632 ± 0.00003490	0.1312
17D30306	9.0 %	✓	0.1772592 ± 0.0008938	0.00304268 ± 0.00003753	0.1345
17D30308	10.3 %	✓	0.1833645 ± 0.0011576	0.00305912 ± 0.00004215	0.1509
17D30309	11.6 %	✓	0.1735589 ± 0.0014799	0.00307726 ± 0.00004767	0.1758
17D30311	12.5 %		0.1754994 ± 0.0021032	0.00310898 ± 0.00006105	0.2085
17D30312	13.4 %		0.1533760 ± 0.0023500	0.00318347 ± 0.00007076	0.2081
17D30314	14.6 %		0.1321890 ± 0.0022293	0.00318341 ± 0.00006631	0.1759
17D30315	16.0 %		0.1212703 ± 0.0023778	0.00320446 ± 0.00007332	0.1661
17D30317	17.6 %		0.1216959 ± 0.0024032	0.00322225 ± 0.00007392	0.1648
17D30318	19.3 %		0.1213110 ± 0.0027382	0.00323715 ± 0.00008386	0.1646
17D30320	21.0 %		0.1252084 ± 0.0042712	0.00327604 ± 0.00011331	0.2066

Results	40(a)/36(a) ± 2σ	40(r)/39(k) ± 2σ	Age ± 2σ (Ma)	MSWD
**Inverse Isochron**<br>Error Chron	**296.30** **± 1.60**<br>**± 0.54%**	0.58456 ± 0.05575<br>± 9.54%	**1.73** **± 0.17**<br>**± 9.53%**<br>Full External Error ± 0.17<br>Analytical Error ± 0.16	2.63<br>0%

Statistics					
	2σ Confidence Limit	1.73	Convergence	0.0000649926
	Error Magnification	1.6204	Number of Iterations	3
	Number of Data Points	17	Calculated Line	Weighted York-2
	Spreading Factor	9.3%		

Degassing Patterns			36Ar(a) [fA]	%1σ	36Ar(c) [fA]	%1σ	36Ar(ca) [fA]	%1σ	36Ar(cl) [fA]	%1σ	37Ar(ca) [fA]	%1σ	38Ar(a) [fA]	%1σ
17D30285	1.8 %	✓	5.545945	0.29	0.0000000	0.00	0.0124438	0.84	0.0000294	17.45	46.0371	0.82	1.0365372	0.29
17D30287	1.9 %	✓	2.563433	0.30	0.0000000	0.00	0.0131635	0.80	0.0000191	25.27	48.6995	0.79	0.4791056	0.30
17D30288	2.0 %	✓	1.222674	0.32	0.0000000	0.00	0.0092519	1.02	0.0000128	39.24	34.2283	1.01	0.2285177	0.32
17D30290	2.2 %	✓	0.920987	0.33	0.0000000	0.00	0.0107386	0.93	0.0000067	73.17	39.7283	0.91	0.1721325	0.33
17D30291	2.4 %	✓	0.697697	0.35	0.0000000	0.00	0.0114860	0.83	0.0000038	126.72	42.4935	0.81	0.1303995	0.35
17D30293	2.7 %	✓	0.696288	0.36	0.0000000	0.00	0.0180049	0.66	0.0000066	74.31	66.6109	0.64	0.1301362	0.36
17D30294	3.0 %	✓	0.460179	0.39	0.0000000	0.00	0.0156908	0.72	0.0000017	270.36	58.0497	0.70	0.0860075	0.39
17D30296	3.4 %	✓	0.495812	0.39	0.0000000	0.00	0.0229620	0.59	0.0000000	0.00	84.9500	0.57	0.0926673	0.39
17D30297	3.9 %	✓	0.514469	0.40	0.0000000	0.00	0.0376284	0.50	0.0000000	0.00	139.2096	0.47	0.0961542	0.40
17D30299	4.5 %	✓	0.403916	0.42	0.0000000	0.00	0.0424402	0.49	0.0000054	89.98	157.0114	0.46	0.0754920	0.42
17D30300	5.2 %	✓	0.396109	0.45	0.0000000	0.00	0.0608789	0.47	0.0000026	188.30	225.2272	0.43	0.0740327	0.45
17D30302	6.0 %	✓	0.305016	0.48	0.0000000	0.00	0.0614439	0.46	0.0000000	0.00	227.3175	0.43	0.0570075	0.48
17D30303	6.9 %	✓	0.264848	0.52	0.0000000	0.00	0.0638020	0.46	0.0000000	0.00	236.0414	0.43	0.0495001	0.52
17D30305	7.9 %	✓	0.215062	0.56	0.0000000	0.00	0.0582439	0.47	0.0000000	0.00	215.4787	0.43	0.0401952	0.56
17D30306	9.0 %	✓	0.195705	0.60	0.0000000	0.00	0.0539506	0.47	0.0000000	0.00	199.5951	0.44	0.0365773	0.60
17D30308	10.3 %	✓	0.155238	0.66	0.0000000	0.00	0.0501888	0.47	0.0000052	95.54	185.6782	0.44	0.0290141	0.66
17D30309	11.6 %	✓	0.116844	0.74	0.0000000	0.00	0.0404414	0.49	0.0000056	86.31	149.6168	0.46	0.0218382	0.74
17D30311	12.5 %		0.080946	0.92	0.0000000	0.00	0.0265179	0.55	0.0000045	101.76	98.1053	0.52	0.0151287	0.92
17D30312	13.4 %		0.069243	1.03	0.0000000	0.00	0.0190984	0.65	0.0000020	242.17	70.6562	0.62	0.0129415	1.03
17D30314	14.6 %		0.073640	0.96	0.0000000	0.00	0.0218431	0.59	0.0000000	0.00	80.8105	0.57	0.0137634	0.96
17D30315	16.0 %		0.067617	1.06	0.0000000	0.00	0.0218490	0.60	0.0000000	0.00	80.8326	0.57	0.0126377	1.06
17D30317	17.6 %		0.068032	1.06	0.0000000	0.00	0.0299132	0.54	0.0000051	102.22	110.6665	0.51	0.0127152	1.06
17D30318	19.3 %		0.060187	1.20	0.0000000	0.00	0.0335645	0.52	0.0000007	693.47	124.1751	0.49	0.0112490	1.20
17D30320	21.0 %		0.038475	1.54	0.0000000	0.00	0.0176982	0.66	0.0000000	0.00	65.4760	0.64	0.0071909	1.54
		Σ	15.628364	0.13	0.0000000	0.00	0.7532438	0.12	0.0001111	17.05	2786.6954	0.11	2.9209412	0.13
		Σ							16.381718	0.12	2786.6954	0.11		

Degassing Patterns			38Ar(c) [fA]	%1σ	38Ar(k) [fA]	%1σ	38Ar(ca) [fA]	%1σ	38Ar(cl) [fA]	%1σ	39Ar(k) [fA]	%1σ	39Ar(ca) [fA]	%1σ	40Ar(r) [fA]	%1σ	40Ar(a) [fA]	%1σ	40Ar(c) [fA]	%1σ	40Ar(k) [fA]	%1σ
17D30285	1.8 %	✓	0.0000000	0.00	0.4845999	0.13	0.0082867	9.66	0.1459704	17.48	40.12585	0.09	0.0295788	1.23	19.53280	24.24	1638.8269	0.29	0.0000000	0.00	0.0243564	9.65
17D30287	1.9 %	✓	0.0000000	0.00	0.2930638	0.15	0.0087659	9.66	0.0948221	25.28	24.26627	0.12	0.0312894	1.21	12.70288	18.02	757.4945	0.30	0.0000000	0.00	0.0147296	9.65
17D30288	2.0 %	✓	0.0000000	0.00	0.1638960	0.21	0.0061611	9.68	0.0635056	39.25	13.57092	0.19	0.0219917	1.37	7.67292	15.11	361.3001	0.32	0.0000000	0.00	0.0082375	9.65
17D30290	2.2 %	✓	0.0000000	0.00	0.1373210	0.23	0.0071511	9.67	0.0331026	73.18	11.37045	0.21	0.0255254	1.29	7.05132	12.68	272.1518	0.33	0.0000000	0.00	0.0069019	9.65
17D30291	2.4 %	✓	0.0000000	0.00	0.1168305	0.26	0.0076488	9.66	0.0186750	126.72	9.67381	0.25	0.0273021	1.22	6.43778	11.17	206.1693	0.35	0.0000000	0.00	0.0058720	9.65
17D30293	2.7 %	✓	0.0000000	0.00	0.1337891	0.24	0.0119900	9.65	0.0327731	74.32	11.07801	0.23	0.0427975	1.12	7.35515	10.24	205.7530	0.36	0.0000000	0.00	0.0067243	9.65
17D30294	3.0 %	✓	0.0000000	0.00	0.1024159	0.28	0.0104489	9.66	0.0086599	270.37	8.48025	0.27	0.0372969	1.15	7.06687	7.68	135.9830	0.39	0.0000000	0.00	0.0051475	9.65
17D30296	3.4 %	✓	0.0000000	0.00	0.1231761	0.25	0.0152910	9.65	0.0000000	0.00	10.19923	0.23	0.0545804	1.08	6.77378	8.51	146.5126	0.39	0.0000000	0.00	0.0061909	9.65
17D30297	3.9 %	✓	0.0000000	0.00	0.1618529	0.20	0.0250577	9.64	0.0000000	0.00	13.40175	0.18	0.0894422	1.03	9.22935	6.66	152.0255	0.40	0.0000000	0.00	0.0081349	9.65
17D30299	4.5 %	✓	0.0000000	0.00	0.1568453	0.21	0.0282621	9.64	0.0266499	89.98	12.98711	0.19	0.1008798	1.03	8.58829	5.90	119.3573	0.42	0.0000000	0.00	0.0078832	9.65
17D30300	5.2 %	✓	0.0000000	0.00	0.1955666	0.18	0.0405409	9.64	0.0131060	188.30	16.19331	0.16	0.1447085	1.02	10.04580	5.35	117.0502	0.45	0.0000000	0.00	0.0098293	9.65
17D30302	6.0 %	✓	0.0000000	0.00	0.1788746	0.19	0.0409171	9.64	0.0000000	0.00	14.81118	0.17	0.1460515	1.02	10.03363	4.45	90.1322	0.48	0.0000000	0.00	0.0089904	9.65
17D30303	6.9 %	✓	0.0000000	0.00	0.1747331	0.20	0.0424875	9.64	0.0000000	0.00	14.46825	0.17	0.1516566	1.02	8.12641	5.15	78.2626	0.52	0.0000000	0.00	0.0087822	9.65
17D30305	7.9 %	✓	0.0000000	0.00	0.1539385	0.21	0.0387862	9.64	0.0000000	0.00	12.74642	0.19	0.1384450	1.02	7.51316	4.91	63.5510	0.56	0.0000000	0.00	0.0077371	9.65
17D30306	9.0 %	✓	0.0000000	0.00	0.1376937	0.23	0.0359271	9.64	0.0000000	0.00	11.40132	0.21	0.1282399	1.02	6.48920	5.53	57.8308	0.60	0.0000000	0.00	0.0069206	9.65
17D30308	10.3 %	✓	0.0000000	0.00	0.1123769	0.27	0.0334221	9.64	0.0257697	95.55	9.30503	0.26	0.1192982	1.02	4.87316	6.54	45.8729	0.66	0.0000000	0.00	0.0056482	9.65
17D30309	11.6 %	✓	0.0000000	0.00	0.0795883	0.36	0.0269310	9.64	0.0275244	86.31	6.59008	0.35	0.0961288	1.03	3.44279	7.85	34.5275	0.74	0.0000000	0.00	0.0040002	9.66
17D30311	12.5 %		0.0000000	0.00	0.0551836	0.49	0.0176590	9.64	0.0224301	101.77	4.56931	0.49	0.0630327	1.06	2.11663	11.22	23.9194	0.92	0.0000000	0.00	0.0027736	9.66
17D30312	13.4 %		0.0000000	0.00	0.0402893	0.65	0.0127181	9.65	0.0097960	242.18	3.33604	0.64	0.0453966	1.11	1.28946	17.80	20.4613	1.03	0.0000000	0.00	0.0020250	9.67
17D30314	14.6 %		0.0000000	0.00	0.0369298	0.75	0.0145459	9.65	0.0000000	0.00	3.05786	0.75	0.0519207	1.08	1.37180	16.67	21.7607	0.96	0.0000000	0.00	0.0018561	9.68
17D30315	16.0 %		0.0000000	0.00	0.0309040	0.88	0.0145499	9.65	0.0000000	0.00	2.55892	0.88	0.0519349	1.08	1.12005	20.57	19.9809	1.06	0.0000000	0.00	0.0015533	9.69
17D30317	17.6 %		0.0000000	0.00	0.0310304	0.89	0.0199200	9.64	0.0250795	102.22	2.56938	0.89	0.0711032	1.05	1.00971	23.00	20.1034	1.06	0.0000000	0.00	0.0015596	9.69
17D30318	19.3 %		0.0000000	0.00	0.0272396	1.02	0.0223515	9.64	0.0035297	693.47	2.25550	1.02	0.0797825	1.04	0.80731	28.72	17.7854	1.20	0.0000000	0.00	0.0013691	9.70
17D30320	21.0 %		0.0000000	0.00	0.0177589	1.52	0.0117857	9.65	0.0000000	0.00	1.47047	1.52	0.0420683	1.12	0.37498	52.79	11.3692	1.54	0.0000000	0.00	0.0008926	9.77
		Σ	0.0000000	0.00	3.1458979	0.05	0.5016052	2.27	0.5513940	17.04	260.48671	0.05	1.7904518	0.24	151.02525	3.84	4618.1814	0.13	0.0000000	0.00	0.1581154	2.47
		Σ							7.1198383	1.33			262.27716	0.05							4769.3648	0.17

Additional Parameters			40Ar/39Ar	1σ	37Ar/39Ar	1σ	36Ar/39Ar	1σ	Time (days)	37Ar (decay)	39Ar (decay)	40Ar (moles)
17D30285	1.8 %	✓	41.299124	0.038519	1.146472	0.009456	0.138423	0.000419	124.056	11.707999	1.00087928	7.960E-11
17D30287	1.9 %	✓	31.699150	0.038223	2.004295	0.015923	0.106044	0.000343	124.070	11.711211	1.00087938	3.697E-11
17D30288	2.0 %	✓	27.145121	0.050959	2.518100	0.025828	0.090631	0.000333	124.077	11.712817	1.00087943	1.771E-11
17D30290	2.2 %	✓	24.500744	0.051611	3.486169	0.032546	0.081760	0.000314	124.091	11.716031	1.00087953	1.340E-11
17D30291	2.4 %	✓	21.916363	0.055342	4.380270	0.037026	0.073104	0.000307	124.098	11.717638	1.00087958	1.021E-11
17D30293	2.7 %	✓	19.163625	0.044276	5.989759	0.040533	0.064231	0.000270	124.112	11.720853	1.00087967	1.023E-11
17D30294	3.0 %	✓	16.795337	0.046455	6.815308	0.050812	0.055870	0.000259	124.119	11.722461	1.00087972	6.867E-12
17D30296	3.4 %	✓	14.949808	0.035526	8.284728	0.050839	0.050593	0.000220	124.133	11.725677	1.00087982	7.358E-12
17D30297	3.9 %	✓	11.953205	0.022221	10.318553	0.051644	0.040923	0.000167	124.140	11.727286	1.00087987	7.741E-12
17D30299	4.5 %	✓	9.776407	0.020401	11.996602	0.059329	0.034105	0.000143	124.154	11.730664	1.00087997	6.142E-12
17D30300	5.2 %	✓	7.779755	0.013813	13.785464	0.063753	0.027971	0.000116	124.161	11.732273	1.00088002	6.101E-12
17D30302	6.0 %	✓	6.697422	0.012848	15.197836	0.070037	0.024501	0.000105	124.175	11.735492	1.00088012	4.808E-12
17D30303	6.9 %	✓	5.909597	0.012028	16.145202	0.074659	0.022480	0.000100	124.182	11.737102	1.00088017	4.147E-12
17D30305	7.9 %	✓	5.515919	0.012518	16.723398	0.078716	0.021211	0.000099	124.196	11.740323	1.00088027	3.411E-12
17D30306	9.0 %	✓	5.579308	0.013946	17.311601	0.083659	0.021654	0.000109	124.203	11.741933	1.00088032	3.088E-12
17D30308	10.3 %	✓	5.385184	0.016839	19.702001	0.100690	0.021798	0.000120	124.217	11.745155	1.00088042	2.436E-12
17D30309	11.6 %	✓	5.679494	0.023962	22.376947	0.128594	0.023525	0.000149	124.224	11.746766	1.00088046	1.823E-12
17D30311	12.5 %		5.621092	0.033370	21.178337	0.150430	0.023199	0.000193	124.238	11.749989	1.00088056	1.250E-12
17D30312	13.4 %		6.432994	0.048810	20.895336	0.185422	0.026126	0.000265	124.244	11.751601	1.00088061	1.044E-12
17D30314	14.6 %		7.439222	0.061895	25.985913	0.240700	0.030704	0.000318	124.258	11.754825	1.00088071	1.110E-12
17D30315	16.0 %		8.082610	0.077952	30.960238	0.320708	0.034267	0.000400	124.265	11.756437	1.00088076	1.013E-12
17D30317	17.6 %		7.996523	0.077208	41.911453	0.420747	0.037096	0.000417	124.279	11.759663	1.00088086	1.014E-12
17D30318	19.3 %		7.962241	0.087332	53.173591	0.583208	0.040146	0.000495	124.287	11.761438	1.00088091	8.925E-13
17D30320	21.0 %		7.765144	0.129516	43.288708	0.695019	0.037138	0.000669	124.301	11.764665	1.00088101	5.638E-13

Procedure Blanks		36Ar ± 1σ (SE) [fA]	37Ar ± 1σ (SE) [fA]	38Ar ± 1σ (SE) [fA]	39Ar ± 1σ (SE) [fA]	40Ar ± 1σ (SE) [fA]
17D30285	1.8 %	0.0137436 ± 0.0003555	0.0062314 ± 0.0194652	0.0211148 ± 0.0167244	0.0251584 ± 0.0159129	4.0958869 ± 0.0890970
17D30287	1.9 %	0.0135139 ± 0.0003555	0.0060651 ± 0.0194652	0.0265811 ± 0.0167244	0.0362936 ± 0.0159129	4.0280429 ± 0.0890970
17D30288	2.0 %	0.0133962 ± 0.0003555	0.0094433 ± 0.0194652	0.0289529 ± 0.0167244	0.0395074 ± 0.0159129	4.0024141 ± 0.0890970
17D30290	2.2 %	0.0131626 ± 0.0003555	0.0204308 ± 0.0194652	0.0329597 ± 0.0167244	0.0424378 ± 0.0159129	3.9632662 ± 0.0890970
17D30291	2.4 %	0.0130493 ± 0.0003555	0.0270976 ± 0.0194652	0.0345937 ± 0.0167244	0.0425885 ± 0.0159129	3.9482052 ± 0.0890970
17D30293	2.7 %	0.0128357 ± 0.0003555	0.0407564 ± 0.0194652	0.0371319 ± 0.0167244	0.0412201 ± 0.0159129	3.9238760 ± 0.0890970
17D30294	3.0 %	0.0127378 ± 0.0003555	0.0470745 ± 0.0194652	0.0380441 ± 0.0167244	0.0400348 ± 0.0159129	3.9135633 ± 0.0890970
17D30296	3.4 %	0.0125649 ± 0.0003555	0.0573381 ± 0.0194652	0.0391860 ± 0.0167244	0.0373706 ± 0.0159129	3.8946525 ± 0.0890970
17D30297	3.9 %	0.0124918 ± 0.0003555	0.0608781 ± 0.0194652	0.0394329 ± 0.0167244	0.0361251 ± 0.0159129	3.8855074 ± 0.0890970
17D30299	4.5 %	0.0123723 ± 0.0003555	0.0641130 ± 0.0194652	0.0393079 ± 0.0167244	0.0342019 ± 0.0159129	3.8661621 ± 0.0890970
17D30300	5.2 %	0.0123332 ± 0.0003555	0.0635133 ± 0.0194652	0.0389698 ± 0.0167244	0.0337549 ± 0.0159129	3.8568394 ± 0.0890970
17D30302	6.0 %	0.0122927 ± 0.0003555	0.0581812 ± 0.0194652	0.0378333 ± 0.0167244	0.0339734 ± 0.0159129	3.8385197 ± 0.0890970
17D30303	6.9 %	0.0122926 ± 0.0003555	0.0535890 ± 0.0194652	0.0370702 ± 0.0167244	0.0346683 ± 0.0159129	3.8299871 ± 0.0890970
17D30305	7.9 %	0.0123351 ± 0.0003555	0.0412382 ± 0.0194652	0.0352545 ± 0.0167244	0.0371771 ± 0.0159129	3.8159597 ± 0.0890970
17D30306	9.0 %	0.0123785 ± 0.0003555	0.0338882 ± 0.0194652	0.0342464 ± 0.0167244	0.0389201 ± 0.0159129	3.8114267 ± 0.0890970
17D30308	10.3 %	0.0125110 ± 0.0003555	0.0181962 ± 0.0194652	0.0321521 ± 0.0167244	0.0430808 ± 0.0159129	3.8103492 ± 0.0890970
17D30309	11.6 %	0.0126006 ± 0.0003555	0.0105312 ± 0.0194652	0.0311194 ± 0.0167244	0.0453272 ± 0.0159129	3.8152640 ± 0.0890970
17D30311	12.5 %	0.0128272 ± 0.0003555	0.0024087 ± 0.0194652	0.0292282 ± 0.0167244	0.0495984 ± 0.0159129	3.8402713 ± 0.0890970
17D30312	13.4 %	0.0129641 ± 0.0003555	0.0067382 ± 0.0194652	0.0284322 ± 0.0167244	0.0513517 ± 0.0159129	3.8623206 ± 0.0890970
17D30314	14.6 %	0.0132852 ± 0.0003555	0.0084167 ± 0.0194652	0.0273072 ± 0.0167244	0.0532894 ± 0.0159129	3.9310246 ± 0.0890970
17D30315	16.0 %	0.0134690 ± 0.0003555	0.0045519 ± 0.0194652	0.0270498 ± 0.0167244	0.0531018 ± 0.0159129	3.9801337 ± 0.0890970
17D30317	17.6 %	0.0138819 ± 0.0003555	0.0159560 ± 0.0194652	0.0273356 ± 0.0167244	0.0493589 ± 0.0159129	4.1146237 ± 0.0890970
17D30318	19.3 %	0.0141340 ± 0.0003555	0.0362274 ± 0.0194652	0.0280418 ± 0.0167244	0.0448351 ± 0.0159129	4.2126517 ± 0.0890970
17D30320	21.0 %	0.0146353 ± 0.0003555	0.0937049 ± 0.0194652	0.0305487 ± 0.0167244	0.0307471 ± 0.0159129	4.4430823 ± 0.0890970

**Intercept Values**		**36Ar ± 1σ (SE) [fA]**	**r2**	**Regression (type,n)**	**37Ar ± 1σ (SE) [fA]**	**r2**	**Regression (type,n)**	**38Ar ± 1σ (SE) [fA]**	**r2**	**Regression (type,n)**	**39Ar ± 1σ (SE) [fA]**	**r2**	**Regression (type,n)**	**40Ar ± 1σ (SE) [fA]**	**r2**	**Regression (type,n)**
17D30285	1.8 %	5.3241198 ± 0.0033858	0.9920	EXP 150 of 150	3.8667471 ± 0.0193938	0.5830	EXP 150 of 150	1.6761477 ± 0.0183499	0.2079	EXP 150 of 150	39.9019453 ± 0.0191752	0.9935	EXP 150 of 150	1662.479913 ± 0.064247	0.9999	EXP 150 of 150
17D30287	1.9 %	2.4751489 ± 0.0025777	0.9786	EXP 150 of 150	4.0887216 ± 0.0193397	0.5760	EXP 150 of 150	0.8916954 ± 0.0165926	0.0763	EXP 150 of 150	24.1652509 ± 0.0175083	0.9846	EXP 150 of 150	774.240125 ± 0.049335	0.9996	EXP 150 of 150
17D30288	2.0 %	1.1903600 ± 0.0016912	0.9551	EXP 150 of 150	2.8785357 ± 0.0179677	0.4746	EXP 150 of 150	0.4854176 ± 0.0180146	0.0650	EXP 150 of 150	13.5380966 ± 0.0169711	0.9547	EXP 150 of 150	372.983689 ± 0.052755	0.9960	EXP 150 of 150
17D30290	2.2 %	0.9033170 ± 0.0013671	0.9481	EXP 149 of 150	3.3496307 ± 0.0188726	0.4853	EXP 150 of 150	0.3784169 ± 0.0170750	0.0160	EXP 150 of 150	11.3593376 ± 0.0154736	0.9426	EXP 150 of 150	283.173261 ± 0.032873	0.9957	EXP 150 of 150
17D30291	2.4 %	0.6905884 ± 0.0012413	0.9211	EXP 150 of 150	3.5875270 ± 0.0154724	0.5892	EXP 150 of 150	0.3048230 ± 0.0163018	0.0061	EXP 150 of 150	9.6763763 ± 0.0165710	0.9157	EXP 150 of 150	216.561195 ± 0.041766	0.9672	EXP 150 of 150
17D30293	2.7 %	0.6952596 ± 0.0014182	0.9025	EXP 150 of 150	5.6204017 ± 0.0193049	0.7275	EXP 150 of 150	0.3420687 ± 0.0172376	0.0344	EXP 150 of 150	11.0848506 ± 0.0177472	0.9258	EXP 150 of 150	217.038770 ± 0.036522	0.9821	EXP 150 of 150
17D30294	3.0 %	0.4673741 ± 0.0010944	0.8374	EXP 150 of 150	4.9089221 ± 0.0194016	0.6679	EXP 150 of 150	0.2430541 ± 0.0159347	0.0107	EXP 150 of 150	8.4984703 ± 0.0148898	0.9126	EXP 150 of 150	146.968570 ± 0.055300	0.0681	EXP 150 of 150
17D30296	3.4 %	0.5081891 ± 0.0011313	0.8643	EXP 150 of 150	7.1702265 ± 0.0206576	0.7987	EXP 150 of 150	0.2491852 ± 0.0177034	0.0003	EXP 150 of 150	10.2200261 ± 0.0154197	0.9351	EXP 150 of 150	157.187185 ± 0.048225	0.6753	EXP 150 of 150
17D30297	3.9 %	0.5399517 ± 0.0012159	0.8707	EXP 150 of 150	11.7153318 ± 0.0191590	0.9189	EXP 149 of 150	0.2970372 ± 0.0164969	0.0005	EXP 150 of 150	13.4336928 ± 0.0143587	0.9680	EXP 150 of 150	165.148497 ± 0.054956	0.5931	EXP 149 of 150
17D30299	4.5 %	0.4388156 ± 0.0009871	0.8457	EXP 150 of 150	13.2051269 ± 0.0186570	0.9456	EXP 150 of 150	0.3230662 ± 0.0165431	0.0389	EXP 150 of 150	13.0313634 ± 0.0171828	0.9547	EXP 150 of 150	131.819665 ± 0.049197	0.3006	EXP 150 of 150
17D30300	5.2 %	0.4489305 ± 0.0010940	0.8354	EXP 150 of 150	18.9112376 ± 0.0215249	0.9643	EXP 150 of 150	0.3582876 ± 0.0172984	0.0280	EXP 150 of 150	16.2583917 ± 0.0166441	0.9697	EXP 149 of 150	130.962627 ± 0.052673	0.5921	EXP 150 of 150
17D30302	6.0 %	0.3623996 ± 0.0009048	0.7964	EXP 150 of 150	19.0756120 ± 0.0184573	0.9715	EXP 150 of 150	0.2896025 ± 0.0164689	0.0004	EXP 150 of 150	14.8873996 ± 0.0155389	0.9699	EXP 150 of 150	104.013386 ± 0.041536	0.9065	EXP 150 of 150
17D30303	6.9 %	0.3262768 ± 0.0008676	0.7866	EXP 150 of 150	19.7981569 ± 0.0191593	0.9724	EXP 150 of 150	0.3004012 ± 0.0164766	0.0072	EXP 150 of 150	14.5531180 ± 0.0163122	0.9660	EXP 150 of 150	90.227775 ± 0.032368	0.9735	EXP 150 of 150
17D30305	7.9 %	0.2734452 ± 0.0007737	0.7303	EXP 149 of 150	18.0608127 ± 0.0183251	0.9686	EXP 150 of 150	0.2553543 ± 0.0165189	0.0026	EXP 150 of 150	12.8326165 ± 0.0151316	0.9631	EXP 150 of 150	74.887814 ± 0.028309	0.9838	EXP 150 of 150
17D30306	9.0 %	0.2508933 ± 0.0007888	0.6795	EXP 150 of 150	16.7229005 ± 0.0190269	0.9608	EXP 150 of 150	0.2207538 ± 0.0176200	0.0036	EXP 150 of 150	11.4884629 ± 0.0151507	0.9475	EXP 149 of 150	68.138383 ± 0.026678	0.9884	EXP 150 of 150
17D30308	10.3 %	0.2087761 ± 0.0006998	0.6317	EXP 150 of 150	15.5392934 ± 0.0190780	0.9552	EXP 150 of 150	0.2302971 ± 0.0173620	0.0060	EXP 150 of 150	9.4020048 ± 0.0165607	0.9123	EXP 150 of 150	54.562106 ± 0.022630	0.9923	EXP 150 of 150
17D30309	11.6 %	0.1628728 ± 0.0005786	0.4228	EXP 150 of 150	12.5154950 ± 0.0179809	0.9406	EXP 150 of 150	0.1851069 ± 0.0162527	0.0064	EXP 149 of 150	6.6851273 ± 0.0159574	0.8363	EXP 150 of 150	41.789521 ± 0.020818	0.9952	EXP 150 of 150
17D30311	12.5 %	0.1154994 ± 0.0005234	0.1613	EXP 150 of 150	8.1949764 ± 0.0188616	0.8675	EXP 150 of 150	0.1382877 ± 0.0150234	0.0000	EXP 150 of 150	4.6497906 ± 0.0149325	0.7264	EXP 150 of 150	29.879091 ± 0.019413	0.9962	EXP 150 of 150
17D30312	13.4 %	0.0973651 ± 0.0005162	0.0871	EXP 150 of 150	5.8962732 ± 0.0199914	0.7446	EXP 150 of 150	0.1032566 ± 0.0163671	0.0000	EXP 150 of 150	3.4093179 ± 0.0138154	0.5991	EXP 150 of 150	25.615069 ± 0.021036	0.9957	EXP 150 of 150
17D30314	14.6 %	0.1045076 ± 0.0005032	0.1418	EXP 150 of 150	6.7410870 ± 0.0180603	0.8169	EXP 150 of 150	0.0917477 ± 0.0165189	0.0013	EXP 150 of 150	3.1414864 ± 0.0159716	0.4203	EXP 150 of 150	27.065372 ± 0.018176	0.9962	EXP 150 of 150
17D30315	16.0 %	0.0989429 ± 0.0005184	0.0472	EXP 150 of 150	6.7458718 ± 0.0191502	0.7909	EXP 150 of 150	0.0812594 ± 0.0172093	0.0040	EXP 150 of 150	2.6458327 ± 0.0156100	0.2656	EXP 150 of 150	25.082629 ± 0.018831	0.9964	EXP 150 of 150
17D30317	17.6 %	0.1074610 ± 0.0005075	0.1591	EXP 150 of 150	9.2553107 ± 0.0211399	0.8598	EXP 149 of 150	0.1150021 ± 0.0189147	0.0110	EXP 150 of 150	2.6715161 ± 0.0160045	0.2211	EXP 150 of 150	25.229312 ± 0.019575	0.9962	EXP 150 of 150
17D30318	19.3 %	0.1037029 ± 0.0005076	0.0191	EXP 150 of 150	10.4018277 ± 0.0206527	0.8895	EXP 150 of 150	0.0916293 ± 0.0173282	0.0002	EXP 150 of 150	2.3639047 ± 0.0161821	0.2677	EXP 150 of 150	22.806698 ± 0.019454	0.9969	EXP 150 of 150
17D30320	21.0 %	0.0683013 ± 0.0004002	0.2345	EXP 150 of 150	5.5578565 ± 0.0183538	0.7492	EXP 150 of 150	0.0558113 ± 0.0169832	0.0041	EXP 150 of 150	1.5327906 ± 0.0153575	0.0512	EXP 150 of 150	16.188186 ± 0.021652	0.9968	EXP 150 of 150

Project Info		Analyst	Irradiation	X-pos	Y-pos	Z/H-pos	Project	Experiment	Nmb
17D30285	1.8 %	Dan Miggins	17-OSU-05	0.00	0.00	9.53	Arctic\O-Connor (16-22)	17D30281	01
17D30287	1.9 %	Dan Miggins	17-OSU-05	0.00	0.00	9.53	Arctic\O-Connor (16-22)	17D30281	01
17D30288	2.0 %	Dan Miggins	17-OSU-05	0.00	0.00	9.53	Arctic\O-Connor (16-22)	17D30281	01
17D30290	2.2 %	Dan Miggins	17-OSU-05	0.00	0.00	9.53	Arctic\O-Connor (16-22)	17D30281	01
17D30291	2.4 %	Dan Miggins	17-OSU-05	0.00	0.00	9.53	Arctic\O-Connor (16-22)	17D30281	01
17D30293	2.7 %	Dan Miggins	17-OSU-05	0.00	0.00	9.53	Arctic\O-Connor (16-22)	17D30281	01
17D30294	3.0 %	Dan Miggins	17-OSU-05	0.00	0.00	9.53	Arctic\O-Connor (16-22)	17D30281	01
17D30296	3.4 %	Dan Miggins	17-OSU-05	0.00	0.00	9.53	Arctic\O-Connor (16-22)	17D30281	01
17D30297	3.9 %	Dan Miggins	17-OSU-05	0.00	0.00	9.53	Arctic\O-Connor (16-22)	17D30281	01
17D30299	4.5 %	Dan Miggins	17-OSU-05	0.00	0.00	9.53	Arctic\O-Connor (16-22)	17D30281	01
17D30300	5.2 %	Dan Miggins	17-OSU-05	0.00	0.00	9.53	Arctic\O-Connor (16-22)	17D30281	01
17D30302	6.0 %	Dan Miggins	17-OSU-05	0.00	0.00	9.53	Arctic\O-Connor (16-22)	17D30281	01
17D30303	6.9 %	Dan Miggins	17-OSU-05	0.00	0.00	9.53	Arctic\O-Connor (16-22)	17D30281	01
17D30305	7.9 %	Dan Miggins	17-OSU-05	0.00	0.00	9.53	Arctic\O-Connor (16-22)	17D30281	01
17D30306	9.0 %	Dan Miggins	17-OSU-05	0.00	0.00	9.53	Arctic\O-Connor (16-22)	17D30281	01
17D30308	10.3 %	Dan Miggins	17-OSU-05	0.00	0.00	9.53	Arctic\O-Connor (16-22)	17D30281	01
17D30309	11.6 %	Dan Miggins	17-OSU-05	0.00	0.00	9.53	Arctic\O-Connor (16-22)	17D30281	01
17D30311	12.5 %	Dan Miggins	17-OSU-05	0.00	0.00	9.53	Arctic\O-Connor (16-22)	17D30281	01
17D30312	13.4 %	Dan Miggins	17-OSU-05	0.00	0.00	9.53	Arctic\O-Connor (16-22)	17D30281	01
17D30314	14.6 %	Dan Miggins	17-OSU-05	0.00	0.00	9.53	Arctic\O-Connor (16-22)	17D30281	01
17D30315	16.0 %	Dan Miggins	17-OSU-05	0.00	0.00	9.53	Arctic\O-Connor (16-22)	17D30281	01
17D30317	17.6 %	Dan Miggins	17-OSU-05	0.00	0.00	9.53	Arctic\O-Connor (16-22)	17D30281	01
17D30318	19.3 %	Dan Miggins	17-OSU-05	0.00	0.00	9.53	Arctic\O-Connor (16-22)	17D30281	01
17D30320	21.0 %	Dan Miggins	17-OSU-05	0.00	0.00	9.53	Arctic\O-Connor (16-22)	17D30281	01

**Sample Parameters**		Sample	Material	Location	Standard Name	Standard (in Ma)	%1σ	Standard Reference	Standard 40Ar/39Ar	%1σ	J	%1σ	Air 40Ar/36Ar	%1σ	MDF (lin)	%1σ	Volume Ratio	Sensitivity (mol/volt)	Day	Month	Year	Hour	Min	Resist
17D30285	1.8 %	HLY0102-D85-90	Groundmass	Gakkel Ridge	FCT-NM (5B6-17)	28.201	0.082	Kuiper et al (2008)	9.59452	0.099	0.00163816	0.099	302.853	0.151	0.9939224	0.069	1	4.8E-14	12	SEP	2017	13	11	1
17D30287	1.9 %	HLY0102-D85-90	Groundmass	Gakkel Ridge	FCT-NM (5B6-17)	28.201	0.082	Kuiper et al (2008)	9.59452	0.099	0.00163816	0.099	302.853	0.151	0.9939224	0.069	1	4.8E-14	12	SEP	2017	13	31	1
17D30288	2.0 %	HLY0102-D85-90	Groundmass	Gakkel Ridge	FCT-NM (5B6-17)	28.201	0.082	Kuiper et al (2008)	9.59452	0.099	0.00163816	0.099	302.853	0.151	0.9939224	0.069	1	4.8E-14	12	SEP	2017	13	41	1
17D30290	2.2 %	HLY0102-D85-90	Groundmass	Gakkel Ridge	FCT-NM (5B6-17)	28.201	0.082	Kuiper et al (2008)	9.59452	0.099	0.00163816	0.099	302.853	0.151	0.9939224	0.069	1	4.8E-14	12	SEP	2017	14	1	1
17D30291	2.4 %	HLY0102-D85-90	Groundmass	Gakkel Ridge	FCT-NM (5B6-17)	28.201	0.082	Kuiper et al (2008)	9.59452	0.099	0.00163816	0.099	302.853	0.151	0.9939224	0.069	1	4.8E-14	12	SEP	2017	14	11	1
17D30293	2.7 %	HLY0102-D85-90	Groundmass	Gakkel Ridge	FCT-NM (5B6-17)	28.201	0.082	Kuiper et al (2008)	9.59452	0.099	0.00163816	0.099	302.853	0.151	0.9939224	0.069	1	4.8E-14	12	SEP	2017	14	31	1
17D30294	3.0 %	HLY0102-D85-90	Groundmass	Gakkel Ridge	FCT-NM (5B6-17)	28.201	0.082	Kuiper et al (2008)	9.59452	0.099	0.00163816	0.099	302.853	0.151	0.9939224	0.069	1	4.8E-14	12	SEP	2017	14	41	1
17D30296	3.4 %	HLY0102-D85-90	Groundmass	Gakkel Ridge	FCT-NM (5B6-17)	28.201	0.082	Kuiper et al (2008)	9.59452	0.099	0.00163816	0.099	302.853	0.151	0.9939224	0.069	1	4.8E-14	12	SEP	2017	15	1	1
17D30297	3.9 %	HLY0102-D85-90	Groundmass	Gakkel Ridge	FCT-NM (5B6-17)	28.201	0.082	Kuiper et al (2008)	9.59452	0.099	0.00163816	0.099	302.853	0.151	0.9939224	0.069	1	4.8E-14	12	SEP	2017	15	11	1
17D30299	4.5 %	HLY0102-D85-90	Groundmass	Gakkel Ridge	FCT-NM (5B6-17)	28.201	0.082	Kuiper et al (2008)	9.59452	0.099	0.00163816	0.099	302.853	0.151	0.9939224	0.069	1	4.8E-14	12	SEP	2017	15	32	1
17D30300	5.2 %	HLY0102-D85-90	Groundmass	Gakkel Ridge	FCT-NM (5B6-17)	28.201	0.082	Kuiper et al (2008)	9.59452	0.099	0.00163816	0.099	302.853	0.151	0.9939224	0.069	1	4.8E-14	12	SEP	2017	15	42	1
17D30302	6.0 %	HLY0102-D85-90	Groundmass	Gakkel Ridge	FCT-NM (5B6-17)	28.201	0.082	Kuiper et al (2008)	9.59452	0.099	0.00163816	0.099	302.853	0.151	0.9939224	0.069	1	4.8E-14	12	SEP	2017	16	2	1
17D30303	6.9 %	HLY0102-D85-90	Groundmass	Gakkel Ridge	FCT-NM (5B6-17)	28.201	0.082	Kuiper et al (2008)	9.59452	0.099	0.00163816	0.099	302.853	0.151	0.9939224	0.069	1	4.8E-14	12	SEP	2017	16	12	1
17D30305	7.9 %	HLY0102-D85-90	Groundmass	Gakkel Ridge	FCT-NM (5B6-17)	28.201	0.082	Kuiper et al (2008)	9.59452	0.099	0.00163816	0.099	302.853	0.151	0.9939224	0.069	1	4.8E-14	12	SEP	2017	16	32	1
17D30306	9.0 %	HLY0102-D85-90	Groundmass	Gakkel Ridge	FCT-NM (5B6-17)	28.201	0.082	Kuiper et al (2008)	9.59452	0.099	0.00163816	0.099	302.853	0.151	0.9939224	0.069	1	4.8E-14	12	SEP	2017	16	42	1
17D30308	10.3 %	HLY0102-D85-90	Groundmass	Gakkel Ridge	FCT-NM (5B6-17)	28.201	0.082	Kuiper et al (2008)	9.59452	0.099	0.00163816	0.099	302.853	0.151	0.9939224	0.069	1	4.8E-14	12	SEP	2017	17	2	1
17D30309	11.6 %	HLY0102-D85-90	Groundmass	Gakkel Ridge	FCT-NM (5B6-17)	28.201	0.082	Kuiper et al (2008)	9.59452	0.099	0.00163816	0.099	302.853	0.151	0.9939224	0.069	1	4.8E-14	12	SEP	2017	17	12	1
17D30311	12.5 %	HLY0102-D85-90	Groundmass	Gakkel Ridge	FCT-NM (5B6-17)	28.201	0.082	Kuiper et al (2008)	9.59452	0.099	0.00163816	0.099	302.853	0.151	0.9939224	0.069	1	4.8E-14	12	SEP	2017	17	32	1
17D30312	13.4 %	HLY0102-D85-90	Groundmass	Gakkel Ridge	FCT-NM (5B6-17)	28.201	0.082	Kuiper et al (2008)	9.59452	0.099	0.00163816	0.099	302.853	0.151	0.9939224	0.069	1	4.8E-14	12	SEP	2017	17	42	1
17D30314	14.6 %	HLY0102-D85-90	Groundmass	Gakkel Ridge	FCT-NM (5B6-17)	28.201	0.082	Kuiper et al (2008)	9.59452	0.099	0.00163816	0.099	302.853	0.151	0.9939224	0.069	1	4.8E-14	12	SEP	2017	18	2	1
17D30315	16.0 %	HLY0102-D85-90	Groundmass	Gakkel Ridge	FCT-NM (5B6-17)	28.201	0.082	Kuiper et al (2008)	9.59452	0.099	0.00163816	0.099	302.853	0.151	0.9939224	0.069	1	4.8E-14	12	SEP	2017	18	12	1
17D30317	17.6 %	HLY0102-D85-90	Groundmass	Gakkel Ridge	FCT-NM (5B6-17)	28.201	0.082	Kuiper et al (2008)	9.59452	0.099	0.00163816	0.099	302.853	0.151	0.9939224	0.069	1	4.8E-14	12	SEP	2017	18	32	1
17D30318	19.3 %	HLY0102-D85-90	Groundmass	Gakkel Ridge	FCT-NM (5B6-17)	28.201	0.082	Kuiper et al (2008)	9.59452	0.099	0.00163816	0.099	302.853	0.151	0.9939224	0.069	1	4.8E-14	12	SEP	2017	18	43	1
17D30320	21.0 %	HLY0102-D85-90	Groundmass	Gakkel Ridge	FCT-NM (5B6-17)	28.201	0.082	Kuiper et al (2008)	9.59452	0.099	0.00163816	0.099	302.853	0.151	0.9939224	0.069	1	4.8E-14	12	SEP	2017	19	3	1

Irradiation Constants		40/36(a)	%1σ	40/36(c)	%1σ	38/36(a)	%1σ	38/36(c)	%1σ	39/37(ca)	%1σ	38/37(ca)	%1σ	36/37(ca)	%1σ	40/39(k)	%1σ	38/39(k)	%1σ	36/38(cl)	%1σ	K/Ca	%1σ	K/Cl	%1σ	Ca/Cl	%1σ
17D30285	1.8 %	295.5	0	0.018	35	0.1869	0	1.493	3	0.000643	0.92	0.00018	9.63	0.00027	0.17	0.000607	9.65	0.012077	0.09	0	0	0.43	0	0	0	0	0
17D30287	1.9 %	295.5	0	0.018	35	0.1869	0	1.493	3	0.000643	0.92	0.00018	9.63	0.00027	0.17	0.000607	9.65	0.012077	0.09	0	0	0.43	0	0	0	0	0
17D30288	2.0 %	295.5	0	0.018	35	0.1869	0	1.493	3	0.000643	0.92	0.00018	9.63	0.00027	0.17	0.000607	9.65	0.012077	0.09	0	0	0.43	0	0	0	0	0
17D30290	2.2 %	295.5	0	0.018	35	0.1869	0	1.493	3	0.000643	0.92	0.00018	9.63	0.00027	0.17	0.000607	9.65	0.012077	0.09	0	0	0.43	0	0	0	0	0
17D30291	2.4 %	295.5	0	0.018	35	0.1869	0	1.493	3	0.000643	0.92	0.00018	9.63	0.00027	0.17	0.000607	9.65	0.012077	0.09	0	0	0.43	0	0	0	0	0
17D30293	2.7 %	295.5	0	0.018	35	0.1869	0	1.493	3	0.000643	0.92	0.00018	9.63	0.00027	0.17	0.000607	9.65	0.012077	0.09	0	0	0.43	0	0	0	0	0
17D30294	3.0 %	295.5	0	0.018	35	0.1869	0	1.493	3	0.000643	0.92	0.00018	9.63	0.00027	0.17	0.000607	9.65	0.012077	0.09	0	0	0.43	0	0	0	0	0
17D30296	3.4 %	295.5	0	0.018	35	0.1869	0	1.493	3	0.000643	0.92	0.00018	9.63	0.00027	0.17	0.000607	9.65	0.012077	0.09	0	0	0.43	0	0	0	0	0
17D30297	3.9 %	295.5	0	0.018	35	0.1869	0	1.493	3	0.000643	0.92	0.00018	9.63	0.00027	0.17	0.000607	9.65	0.012077	0.09	0	0	0.43	0	0	0	0	0
17D30299	4.5 %	295.5	0	0.018	35	0.1869	0	1.493	3	0.000643	0.92	0.00018	9.63	0.00027	0.17	0.000607	9.65	0.012077	0.09	0	0	0.43	0	0	0	0	0
17D30300	5.2 %	295.5	0	0.018	35	0.1869	0	1.493	3	0.000643	0.92	0.00018	9.63	0.00027	0.17	0.000607	9.65	0.012077	0.09	0	0	0.43	0	0	0	0	0
17D30302	6.0 %	295.5	0	0.018	35	0.1869	0	1.493	3	0.000643	0.92	0.00018	9.63	0.00027	0.17	0.000607	9.65	0.012077	0.09	0	0	0.43	0	0	0	0	0
17D30303	6.9 %	295.5	0	0.018	35	0.1869	0	1.493	3	0.000643	0.92	0.00018	9.63	0.00027	0.17	0.000607	9.65	0.012077	0.09	0	0	0.43	0	0	0	0	0
17D30305	7.9 %	295.5	0	0.018	35	0.1869	0	1.493	3	0.000643	0.92	0.00018	9.63	0.00027	0.17	0.000607	9.65	0.012077	0.09	0	0	0.43	0	0	0	0	0
17D30306	9.0 %	295.5	0	0.018	35	0.1869	0	1.493	3	0.000643	0.92	0.00018	9.63	0.00027	0.17	0.000607	9.65	0.012077	0.09	0	0	0.43	0	0	0	0	0
17D30308	10.3 %	295.5	0	0.018	35	0.1869	0	1.493	3	0.000643	0.92	0.00018	9.63	0.00027	0.17	0.000607	9.65	0.012077	0.09	0	0	0.43	0	0	0	0	0
17D30309	11.6 %	295.5	0	0.018	35	0.1869	0	1.493	3	0.000643	0.92	0.00018	9.63	0.00027	0.17	0.000607	9.65	0.012077	0.09	0	0	0.43	0	0	0	0	0
17D30311	12.5 %	295.5	0	0.018	35	0.1869	0	1.493	3	0.000643	0.92	0.00018	9.63	0.00027	0.17	0.000607	9.65	0.012077	0.09	0	0	0.43	0	0	0	0	0
17D30312	13.4 %	295.5	0	0.018	35	0.1869	0	1.493	3	0.000643	0.92	0.00018	9.63	0.00027	0.17	0.000607	9.65	0.012077	0.09	0	0	0.43	0	0	0	0	0
17D30314	14.6 %	295.5	0	0.018	35	0.1869	0	1.493	3	0.000643	0.92	0.00018	9.63	0.00027	0.17	0.000607	9.65	0.012077	0.09	0	0	0.43	0	0	0	0	0
17D30315	16.0 %	295.5	0	0.018	35	0.1869	0	1.493	3	0.000643	0.92	0.00018	9.63	0.00027	0.17	0.000607	9.65	0.012077	0.09	0	0	0.43	0	0	0	0	0
17D30317	17.6 %	295.5	0	0.018	35	0.1869	0	1.493	3	0.000643	0.92	0.00018	9.63	0.00027	0.17	0.000607	9.65	0.012077	0.09	0	0	0.43	0	0	0	0	0
17D30318	19.3 %	295.5	0	0.018	35	0.1869	0	1.493	3	0.000643	0.92	0.00018	9.63	0.00027	0.17	0.000607	9.65	0.012077	0.09	0	0	0.43	0	0	0	0	0
17D30320	21.0 %	295.5	0	0.018	35	0.1869	0	1.493	3	0.000643	0.92	0.00018	9.63	0.00027	0.17	0.000607	9.65	0.012077	0.09	0	0	0.43	0	0	0	0	0

17D30281.AGE >>> HLY0102-D85-90 >>> ARCTIC | O-CONNOR (16-22) PROJECT

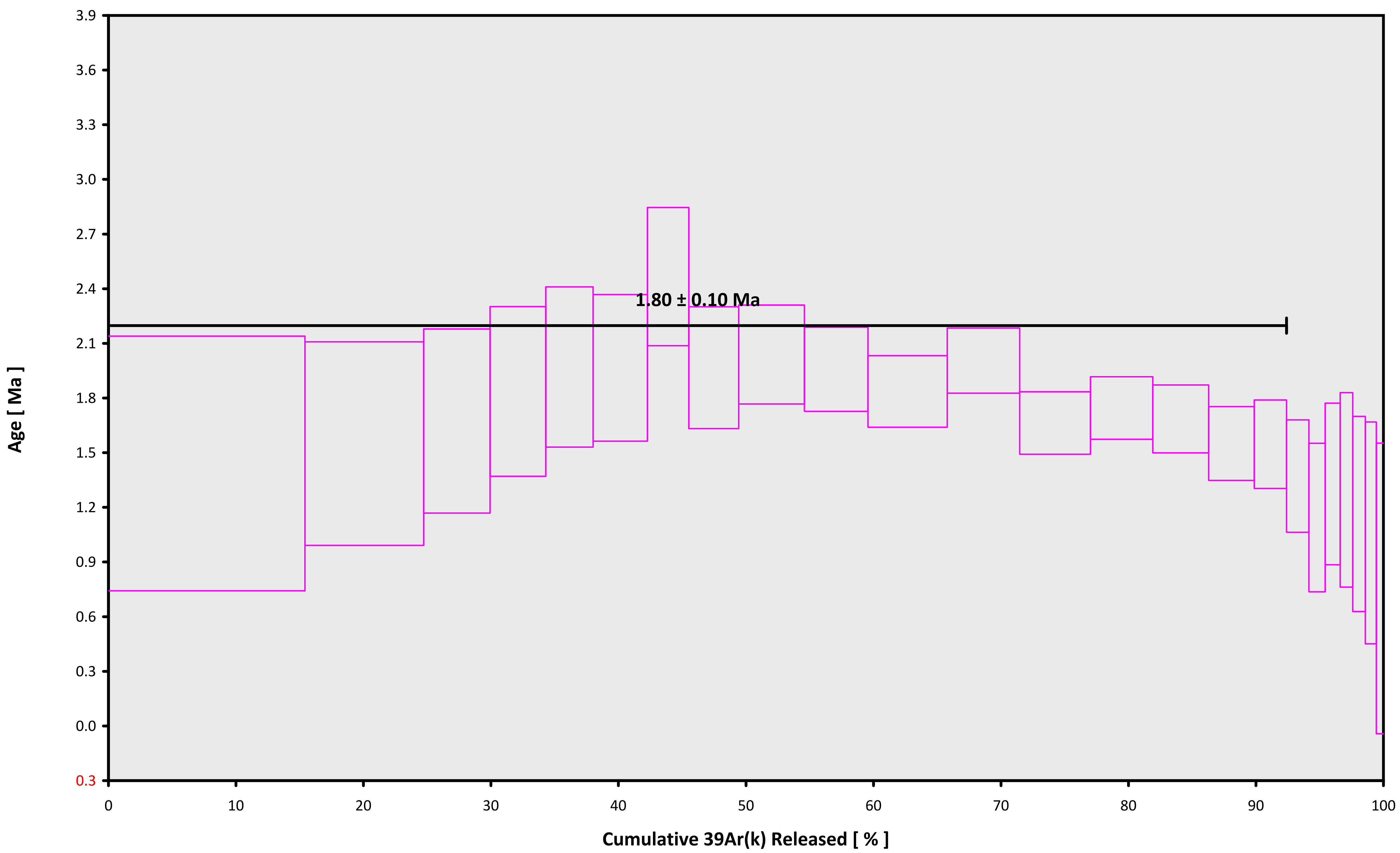

Ar-Ages in Ma

WEIGHTED PLATEAU
1.80 ± 0.10
TOTAL FUSION
1.72 ± 0.13
NORMAL ISOCHRON
1.73 ± 0.17
INVERSE ISOCHRON
1.73 ± 0.17

MSWD (PROBABILITY)
2.63 (0%)

Sample Info

Groundmass
Gakkel Ridge
Dan Miggins

IRR = 17-OSU-05 (5B6-17)
J = 0.00163816 ± 0.00000162

17D30281.AGE >>> HLY0102-D85-90 >>> ARCTIC | O-CONNOR (16-22) PROJECT

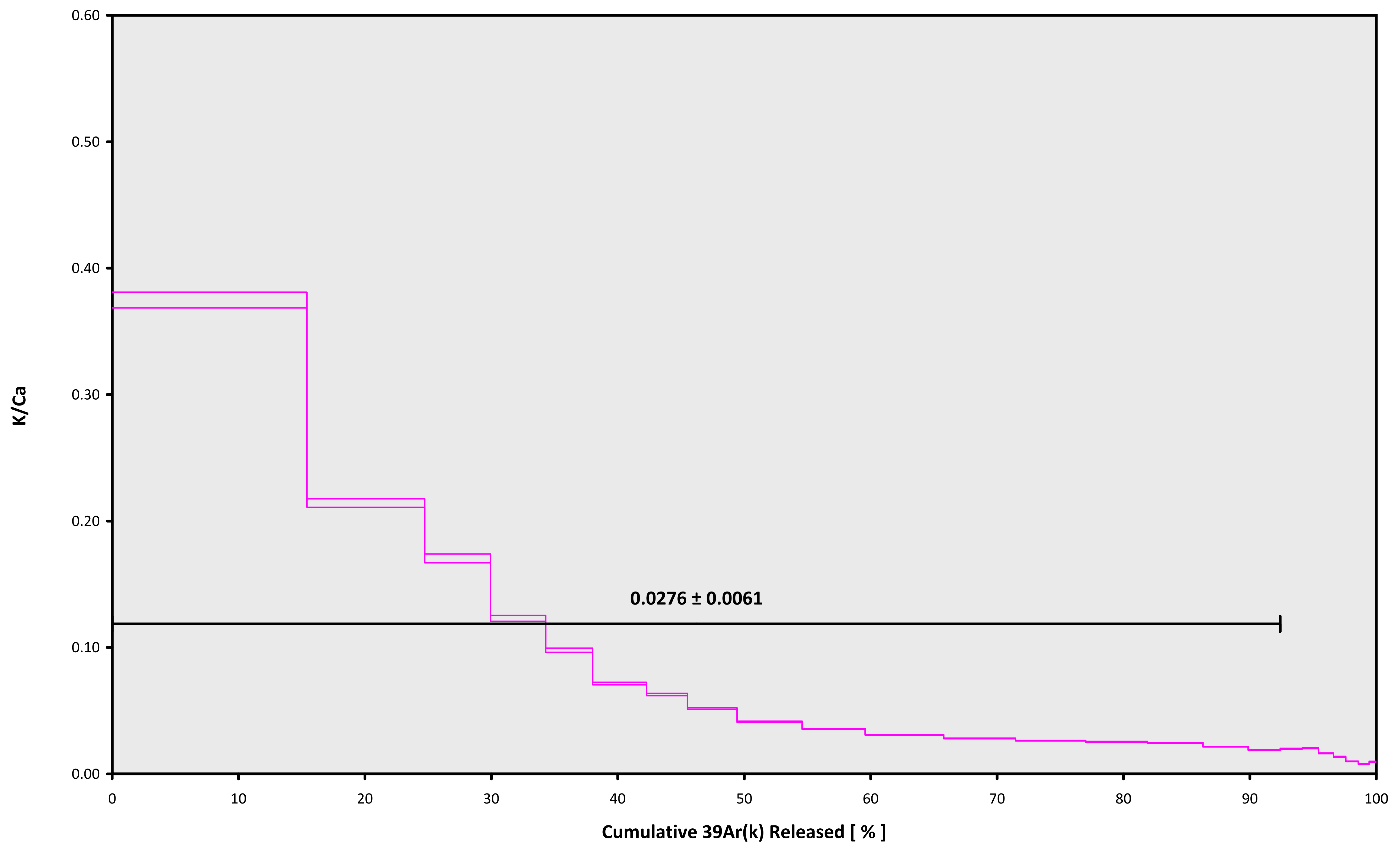

Ar-Ages in Ma

WEIGHTED PLATEAU
1.80 ± 0.10
TOTAL FUSION
1.72 ± 0.13
NORMAL ISOCHRON
1.73 ± 0.17
INVERSE ISOCHRON
1.73 ± 0.17

Sample Info

Groundmass
Gakkel Ridge
Dan Miggins

IRR = 17-OSU-05 (5B6-17)
J = 0.00163816 ± 0.00000162

17D30281.AGE >>> HLY0102-D85-90 >>> ARCTIC | O-CONNOR (16-22) PROJECT

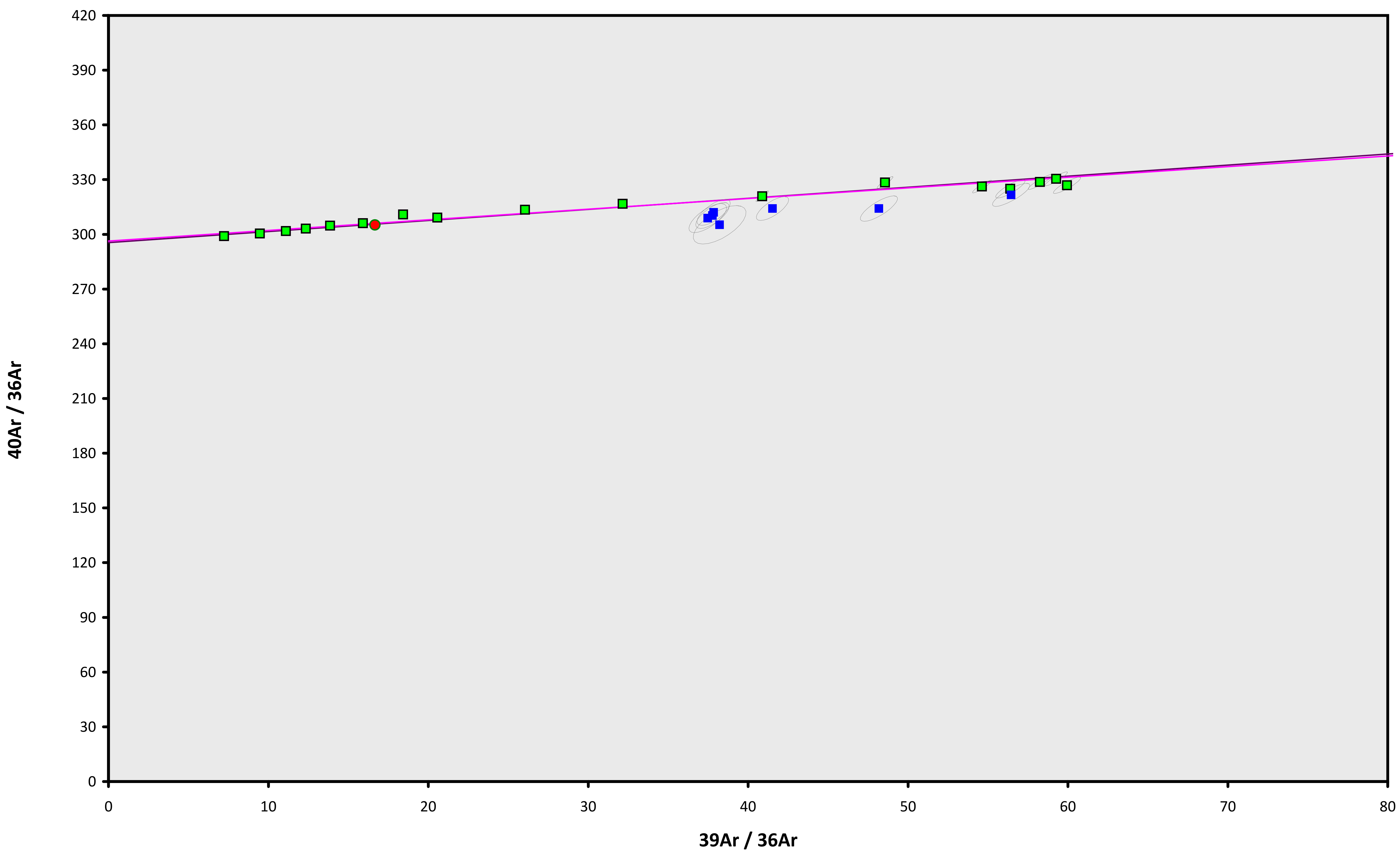

Ar-Ages in Ma

WEIGHTED PLATEAU
1.80 ± 0.10
TOTAL FUSION
1.72 ± 0.13
NORMAL ISOCHRON
1.73 ± 0.17
INVERSE ISOCHRON
1.73 ± 0.17

MSWD (PROBABILITY)
2.62 (0%)

40AR/36AR INTERCEPT
296.3 ± 1.6

Sample Info

Groundmass
Gakkel Ridge
Dan Miggins

IRR = 17-OSU-05 (5B6-17)
J = 0.00163816 ± 0.00000162

17D30281.AGE >>> HLY0102-D85-90 >>> ARCTIC | O-CONNOR (16-22) PROJECT

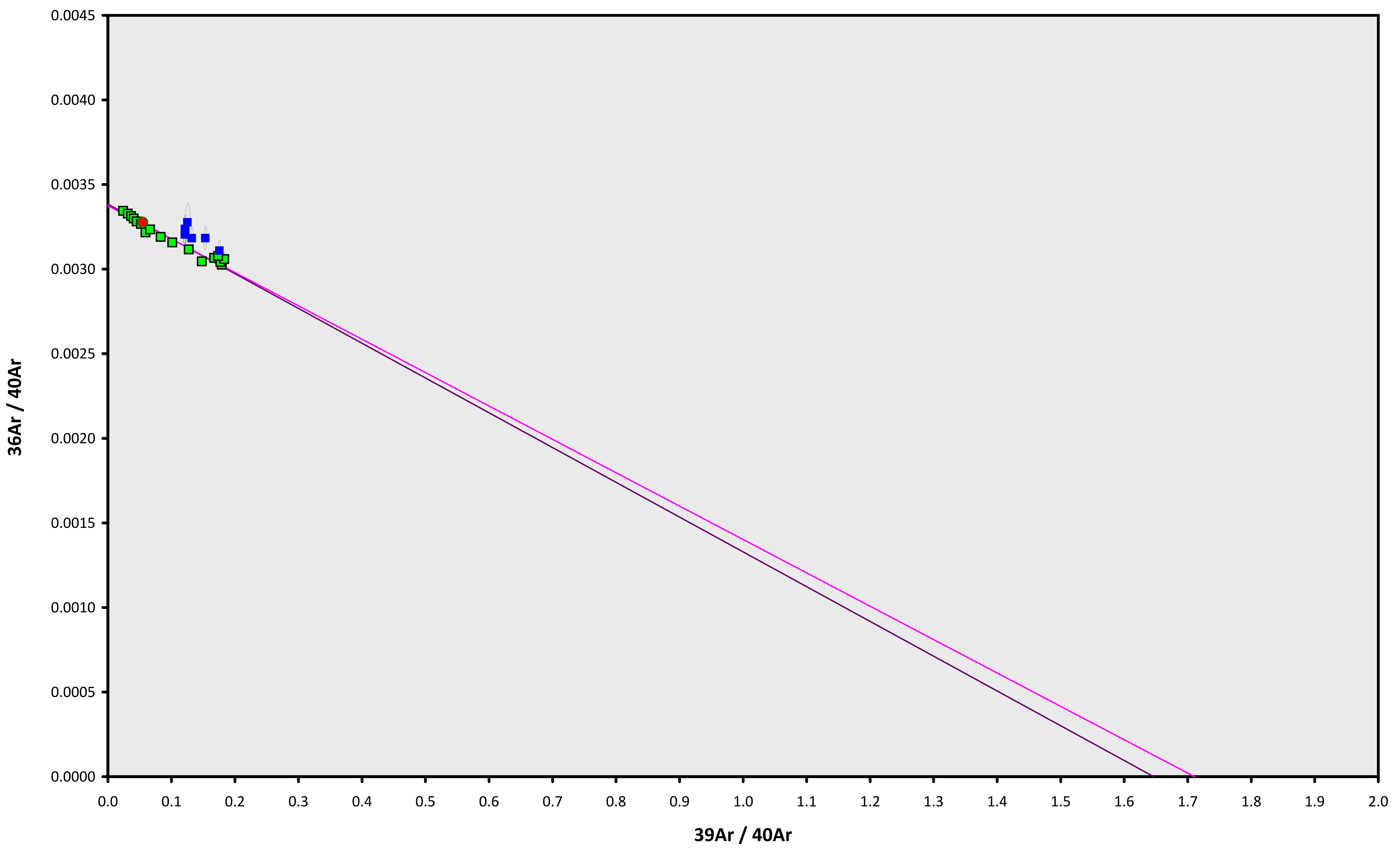

Ar-Ages in Ma

WEIGHTED PLATEAU
1.80 ± 0.10
TOTAL FUSION
1.72 ± 0.13
NORMAL ISOCHRON
1.73 ± 0.17
INVERSE ISOCHRON
1.73 ± 0.17

MSWD (PROBABILITY)
2.63 (0%)

SPREADING FACTOR
9.3%

40AR/36AR INTERCEPT
296.3 ± 1.6

Sample Info

Groundmass
Gakkel Ridge
Dan Miggins

IRR = 17-OSU-05 (5B6-17)
J = 0.00163816 ± 0.00000162

Relative Abundances

			36Ar [fA]	%1σ	37Ar [fA]	%1σ	38Ar [fA]	%1σ	39Ar [fA]	%1σ	40Ar [fA]	%1σ	40(r)/39(k) ± 2σ	Age ± 2σ (ka)	40Ar(r) (%)	39Ar(k) (%)	K/Ca ± 2σ
17D20353	1.8 %		13.118647	0.284	19.2235	2.458	2.831588	0.849	27.04112	0.110	3865.693	0.003	1.63859 ± 1.00131	4607.8 ± 2812.1	1.15	6.10	0.605 ± 0.030
17D20355	1.9 %		6.874698	0.288	19.0122	2.300	1.601425	1.482	20.16057	0.136	2026.858	0.005	1.24043 ± 0.71038	3489.2 ± 1996.3	1.23	4.54	0.456 ± 0.021
17D20356	2.0 %		3.756188	0.290	16.3281	2.877	0.908720	2.646	13.67536	0.186	1108.307	0.009	1.09748 ± 0.57563	3087.5 ± 1618.0	1.35	3.08	0.360 ± 0.021
17D20358	2.2 %		2.742669	0.295	16.9035	2.883	0.666378	3.506	12.30167	0.204	809.783	0.012	0.96546 ± 0.47222	2716.3 ± 1327.6	1.47	2.77	0.313 ± 0.018
17D20359	2.4 %		2.146032	0.297	20.4683	2.285	0.539653	4.453	12.02412	0.204	632.195	0.014	0.70142 ± 0.38037	1973.9 ± 1069.8	1.33	2.71	0.252 ± 0.012
17D20361	2.7 %		1.968762	0.303	27.7958	1.742	0.516630	4.540	13.78349	0.184	580.571	0.015	0.65639 ± 0.30802	1847.2 ± 866.4	1.56	3.11	0.213 ± 0.007
17D20362	3.0 %		1.683903	0.305	29.3466	1.682	0.492380	4.965	13.15610	0.194	496.212	0.018	0.59456 ± 0.27767	1673.3 ± 781.1	1.57	2.96	0.192 ± 0.007
17D20364	3.4 %		1.597914	0.308	46.5667	1.076	0.514469	4.448	17.31244	0.155	470.445	0.019	0.48920 ± 0.20109	1376.9 ± 565.8	1.80	3.90	0.160 ± 0.003
17D20365	3.9 %	✓	1.467051	0.310	59.3857	0.926	0.502273	4.950	19.10847	0.142	429.311	0.020	0.33894 ± 0.16817	954.1 ± 473.3	1.51	4.30	0.138 ± 0.003
17D20367	4.5 %	✓	1.150892	0.319	58.7910	0.940	0.407833	6.038	16.64316	0.158	336.725	0.026	0.35917 ± 0.15458	1011.0 ± 435.0	1.77	3.75	0.121 ± 0.002
17D20368	5.2 %	✓	1.484641	0.310	137.9227	0.583	0.654855	3.639	30.78981	0.104	432.007	0.020	0.33265 ± 0.10519	936.4 ± 296.0	2.36	6.93	0.096 ± 0.001
17D20370	6.0 %	✓	1.122035	0.314	146.6216	0.564	0.566496	4.431	27.96672	0.111	325.497	0.026	0.36084 ± 0.08818	1015.7 ± 248.2	3.09	6.29	0.082 ± 0.001
17D20371	6.9 %	✓	1.037696	0.321	189.5054	0.520	0.559006	4.277	30.37301	0.105	298.681	0.029	0.37004 ± 0.07617	1041.6 ± 214.3	3.75	6.82	0.069 ± 0.001
17D20373	7.9 %	✓	0.893712	0.335	235.4515	0.504	0.540949	4.374	31.88295	0.104	252.915	0.034	0.34682 ± 0.06427	976.3 ± 180.9	4.35	7.16	0.058 ± 0.001
17D20374	9.0 %	✓	0.708366	0.335	255.5415	0.497	0.476587	4.947	30.04157	0.106	196.406	0.043	0.33775 ± 0.05409	950.7 ± 152.2	5.14	6.74	0.050 ± 0.001
17D20376	10.3 %	✓	0.547045	0.350	261.7900	0.498	0.443138	5.239	27.56998	0.110	147.549	0.057	0.31891 ± 0.04712	897.7 ± 132.6	5.92	6.18	0.045 ± 0.000
17D20377	11.6 %	✓	0.412653	0.380	234.5109	0.507	0.341723	7.216	23.09050	0.124	109.308	0.076	0.32758 ± 0.04548	922.1 ± 128.0	6.87	5.17	0.042 ± 0.000
17D20379	12.5 %	✓	0.273556	0.445	172.3840	0.542	0.254187	8.878	16.32510	0.163	71.608	0.116	0.33672 ± 0.04913	947.8 ± 138.3	7.62	3.66	0.040 ± 0.000
17D20380	13.4 %	✓	0.200253	0.487	133.7169	0.580	0.157219	14.396	12.41158	0.205	52.518	0.157	0.38034 ± 0.05184	1070.6 ± 145.9	8.93	2.78	0.040 ± 0.000
17D20382	14.6 %	✓	0.183948	0.486	130.6327	0.583	0.188811	13.040	11.70130	0.206	47.035	0.174	0.31960 ± 0.05076	899.7 ± 142.8	7.89	2.62	0.038 ± 0.000
17D20383	16.0 %	✓	0.167520	0.498	123.4845	0.598	0.186847	12.366	10.82344	0.225	42.771	0.191	0.34204 ± 0.05143	962.8 ± 144.7	8.59	2.42	0.037 ± 0.000
17D20385	17.6 %	✓	0.158244	0.500	117.3632	0.601	0.179260	13.334	10.11931	0.227	40.681	0.203	0.37898 ± 0.05250	1066.8 ± 147.7	9.36	2.27	0.037 ± 0.000
17D20386	19.3 %	✓	0.135009	0.579	98.7437	0.653	0.136104	17.934	8.62178	0.276	34.946	0.235	0.39449 ± 0.05998	1110.4 ± 168.8	9.66	1.93	0.037 ± 0.001
17D20388	21.0 %	✓	0.138642	0.565	117.2098	0.617	0.095528	25.528	8.11417	0.283	34.047	0.241	0.35734 ± 0.06427	1005.9 ± 180.9	8.44	1.81	0.029 ± 0.000
		Σ	43.970073	0.106	2668.6999	0.145	13.762060	0.850	445.03773	0.030	12842.069	0.003					

Information on Analysis and Constants Used in Calculations

Project = **O-CONNOR (16-22)**
Sample = **PS59-305-1**
Material = **Groundmass**
Location = **Gakkel Ridge**
Region = **Artic Ocean**
Analyst = **Dan Miggins**
Irradiation = **17-OSU-01 (1C35-17)**
Position = **X: 0 | Y: 0 | Z/H: 53.4427 mm**
FCT-NM Age = **28.201 ± 0.023 Ma**
FCT-NM Reference = **Kuiper et al (2008)**
FCT-NM 40Ar/39Ar Ratio = **10.09436 ± 0.00848**
FCT-NM J-value = **0.00155705 ± 0.00000131**
Air Shot 40Ar/36Ar = **302.4360 ± 0.4537**
Air Shot MDF = **0.99425915 ± 0.00068600 (LIN)**
Experiment Type = **Incremental Heating**
Extraction Method = **Bulk Laser Heating**
Heating = **64 sec**
Isolation = **3.00 min**
Instrument = **ARGUS-VI-D**
Preferred Age = **Plateau Age**
Age Classification = **Crystallization Age**
IGSN = Undefined
Rock Class = **Igneous>Volcanic**
Lithology = **Basaltic Lava**
Lat-Lon = Undefined - Undefined

Age Equations = **Min et al. (2000)**
Negative Intensities = **Allowed**
Collector Calibrations = **36Ar**
Decay 40K = **5.530 ± 0.048 E-10 1/a**
Decay 39Ar = **2.940 ± 0.016 E-07 1/h**
Decay 37Ar = **8.230 ± 0.012 E-04 1/h**
Decay 36Cl = **2.257 ± 0.015 E-06 1/a**
Decay 40K(EC,β⁺) = **0.580 ± 0.009 E-10 1/a**
Decay 40K(β⁻) = **4.950 ± 0.043 E-10 1/a**
Atmospheric 40/36(a) = **291.41 ± 0.62**
Atmospheric 38/36(a) = **0.1869**
Production 39/37(ca) = **0.0006425 ± 0.0000059**
Production 38/37(ca) = **0.0001800 ± 0.0000173**
Production 36/37(ca) = **0.0002703 ± 0.0000005**
Production 40/39(k) = **0.000607 ± 0.000059**
Production 38/39(k) = **0.012077 ± 0.000011**
Production 36/38(cl) = **262.80 ± 1.71**
Scaling Ratio K/Ca = **0.430**
Abundance Ratio 40K/K = **1.1700 ± 0.0100 E-04**
Atomic Weight K = **39.0983 ± 0.0001 g**

Results

Results	40(a)/36(a) ± 2σ	40(r)/39(k) ± 2σ	Age ± 2σ (ka)	MSWD	39Ar(k) (%,n)	K/Ca ± 2σ
**Age Plateau**		0.34754 ± 0.01504 ± 4.33%	**978.3 ± 42.4 ± 4.33%**	0.66 82%	70.83 16	0.044 ± 0.007
			Full External Error ± 47.8	1.73	2σ Confidence Limit	
			Analytical Error ± 42.3	1.0000	Error Magnification	
**Total Fusion Age**		0.53844 ± 0.07642 ± 14.19%	**1515.4 ± 215.0 ± 14.19%**		24	0.071 ± 0.000
			Full External Error ± 217.7			
			Analytical Error ± 215.0			
**Normal Isochron**	**291.42 ± 1.23 ± 0.42%**	0.34639 ± 0.02734 ± 7.89%	**975.1 ± 76.9 ± 7.89%**	0.81 66%	70.83 16	
			Full External Error ± 80.0	1.76	2σ Confidence Limit	
			Analytical Error ± 76.9	1.0000	Error Magnification	
				4	Number of Iterations	
				0.0000025356	Convergence	
**Inverse Isochron**	**291.41 ± 1.23 ± 0.42%**	0.34706 ± 0.02706 ± 7.80%	**976.9 ± 76.2 ± 7.80%**	0.82 65%	70.83 16	
			Full External Error ± 79.3	1.76	2σ Confidence Limit	
			Analytical Error ± 76.1	1.0000	Error Magnification	
				3	Number of Iterations	
				0.0009773618	Convergence	
				7%	Spreading Factor	

Notes

Subatmospheric Initial 40Ar/36Ar = 291.41 ± 0.21 (%SD).

Incremental Heating			36Ar(a) [fA]	37Ar(ca) [fA]	38Ar(cl) [fA]	39Ar(k) [fA]	40Ar(r) [fA]	Age ± 2σ (ka)	40Ar(r) (%)	39Ar(k) (%)	K/Ca ± 2σ
17D20353	1.8 %		13.113439	19.2235	0.0507997	27.02877	44.28900	4607.8 ± 2812.1	1.15	6.10	0.605 ± 0.030
17D20355	1.9 %		6.869542	19.0122	0.0707541	20.14836	24.99267	3489.2 ± 1996.3	1.23	4.54	0.456 ± 0.021
17D20356	2.0 %		3.751765	16.3281	0.0395455	13.66487	14.99696	3087.5 ± 1618.0	1.35	3.08	0.360 ± 0.021
17D20358	2.2 %		2.738099	16.9035	0.0031489	12.29081	11.86625	2716.3 ± 1327.6	1.47	2.77	0.313 ± 0.018
17D20359	2.4 %		2.140499	20.4683	0.0000000	12.01097	8.42473	1973.9 ± 1069.8	1.33	2.71	0.252 ± 0.012
17D20361	2.7 %		1.961248	27.7958	0.0000000	13.76563	9.03568	1847.2 ± 866.4	1.56	3.11	0.213 ± 0.007
17D20362	3.0 %		1.675967	29.3466	0.0152009	13.13724	7.81083	1673.3 ± 781.1	1.57	2.96	0.192 ± 0.007
17D20364	3.4 %		1.585327	46.5667	0.0010682	17.28252	8.45453	1376.9 ± 565.8	1.80	3.90	0.160 ± 0.003
17D20365	3.9 %	✓	1.450999	59.3857	0.0000000	19.07031	6.46361	954.1 ± 473.3	1.51	4.30	0.138 ± 0.003
17D20367	4.5 %	✓	1.135001	58.7910	0.0000000	16.60539	5.96421	1011.0 ± 435.0	1.77	3.75	0.121 ± 0.002
17D20368	5.2 %	✓	1.447361	137.9227	0.0000000	30.70120	10.21266	936.4 ± 296.0	2.36	6.93	0.096 ± 0.001
17D20370	6.0 %	✓	1.082402	146.6216	0.0011863	27.87252	10.05741	1015.7 ± 248.2	3.09	6.29	0.082 ± 0.001
17D20371	6.9 %	✓	0.986472	189.5054	0.0000000	30.25126	11.19428	1041.6 ± 214.3	3.75	6.82	0.069 ± 0.001
17D20373	7.9 %	✓	0.830069	235.4515	0.0000000	31.73167	11.00512	976.3 ± 180.9	4.35	7.16	0.058 ± 0.001
17D20374	9.0 %	✓	0.639293	255.5415	0.0000000	29.87738	10.09098	950.7 ± 152.2	5.14	6.74	0.050 ± 0.001
17D20376	10.3 %	✓	0.476283	261.7900	0.0000000	27.40178	8.73871	897.7 ± 132.6	5.92	6.18	0.045 ± 0.000
17D20377	11.6 %	✓	0.349264	234.5109	0.0000000	22.93983	7.51457	922.1 ± 128.0	6.87	5.17	0.042 ± 0.000
17D20379	12.5 %	✓	0.226960	172.3840	0.0000000	16.21435	5.45974	947.8 ± 138.3	7.62	3.66	0.040 ± 0.000
17D20380	13.4 %	✓	0.164109	133.7169	0.0000000	12.32566	4.68795	1070.6 ± 145.9	8.93	2.78	0.040 ± 0.000
17D20382	14.6 %	✓	0.148638	130.6327	0.0000000	11.61737	3.71295	899.7 ± 142.8	7.89	2.62	0.038 ± 0.000
17D20383	16.0 %	✓	0.134140	123.4845	0.0097929	10.74410	3.67490	962.8 ± 144.7	8.59	2.42	0.037 ± 0.000
17D20385	17.6 %	✓	0.126518	117.3632	0.0131886	10.04391	3.80644	1066.8 ± 147.7	9.36	2.27	0.037 ± 0.000
17D20386	19.3 %	✓	0.108318	98.7437	0.0000000	8.55834	3.37617	1110.4 ± 168.8	9.66	1.93	0.037 ± 0.001
17D20388	21.0 %	✓	0.106960	117.2098	0.0000000	8.03886	2.87259	1005.9 ± 180.9	8.44	1.81	0.029 ± 0.000
		Σ	43.248675	2668.6999	0.2046851	443.32309	238.70292				

Information on Analysis

Project = **O-CONNOR (16-22)**
Sample = **PS59-305-1**
Material = **Groundmass**
Location = **Gakkel Ridge**
Region = **Artic Ocean**
Analyst = **Dan Miggins**
Irradiation = **17-OSU-01 (1C35-17)**
J = **0.00155705 ± 0.00000131**
FCT-NM = **28.201 ± 0.023 Ma**

Results	40(r)/39(k) ± 2σ		Age ± 2σ (ka)		MSWD	39Ar(k) (%,n)	K/Ca ± 2σ
**Age Plateau**	0.34754	± 0.01504<br>± 4.33%	**978.3**	**± 42.4**<br>**± 4.33%**	0.66<br>82%	70.83<br>16	0.044 ± 0.007
			Full External Error	± 47.8	1.73	2σ Confidence Limit	
			Analytical Error	± 42.3	1.0000	Error Magnification	
**Total Fusion Age**	0.53844	± 0.07642<br>± 14.19%	**1515.4**	**± 215.0**<br>**± 14.19%**		24	0.071 ± 0.000
			Full External Error	± 217.7			
			Analytical Error	± 215.0			

Normal Isochron			39(k)/36(a) ± 2σ	40(a+r)/36(a) ± 2σ	r.i.
17D20353	1.8 %		2.06 ± 0.01	294.79 ± 1.67	0.9318
17D20355	1.9 %		2.93 ± 0.02	295.05 ± 1.70	0.9038
17D20356	2.0 %		3.64 ± 0.03	295.41 ± 1.72	0.8415
17D20358	2.2 %		4.49 ± 0.03	295.74 ± 1.75	0.8217
17D20359	2.4 %		5.61 ± 0.04	295.35 ± 1.76	0.8239
17D20361	2.7 %		7.02 ± 0.05	296.02 ± 1.80	0.8546
17D20362	3.0 %		7.84 ± 0.06	296.07 ± 1.82	0.8441
17D20364	3.4 %		10.90 ± 0.08	296.74 ± 1.84	0.8930
17D20365	3.9 %	✓	13.14 ± 0.09	295.86 ± 1.86	0.9084
17D20367	4.5 %	✓	14.63 ± 0.11	296.66 ± 1.93	0.8955
17D20368	5.2 %	✓	21.21 ± 0.14	298.47 ± 1.90	0.9487
17D20370	6.0 %	✓	25.75 ± 0.18	300.70 ± 1.97	0.9429
17D20371	6.9 %	✓	30.67 ± 0.22	302.76 ± 2.06	0.9512
17D20373	7.9 %	✓	38.23 ± 0.29	304.67 ± 2.22	0.9566
17D20374	9.0 %	✓	46.74 ± 0.37	307.19 ± 2.32	0.9556
17D20376	10.3 %	✓	57.53 ± 0.49	309.76 ± 2.56	0.9560
17D20377	11.6 %	✓	65.68 ± 0.63	312.93 ± 2.91	0.9517
17D20379	12.5 %	✓	71.44 ± 0.82	315.47 ± 3.54	0.9372
17D20380	13.4 %	✓	75.11 ± 0.97	319.98 ± 4.03	0.9172
17D20382	14.6 %	✓	78.16 ± 1.02	316.39 ± 4.06	0.9125
17D20383	16.0 %	✓	80.10 ± 1.09	318.81 ± 4.26	0.9033
17D20385	17.6 %	✓	79.39 ± 1.09	321.50 ± 4.35	0.8987
17D20386	19.3 %	✓	79.01 ± 1.25	322.58 ± 5.01	0.8920
17D20388	21.0 %	✓	75.16 ± 1.21	318.27 ± 5.05	0.8912

Results	40(a)/36(a) ± 2σ	40(r)/39(k) ± 2σ	Age ± 2σ (ka)	MSWD
Normal Isochron	**291.42 ± 1.23 ± 0.42%**	0.34639 ± 0.02734 ± 7.89%	**975.1 ± 76.9 ± 7.89%**<br>Full External Error ± 80.0<br>Analytical Error ± 76.9	0.81<br>66%

Statistics				
	2σ Confidence Limit	1.76	Convergence	0.000002535646
	Error Magnification	1.0000	Number of Iterations	4
	Number of Data Points	16	Calculated Line	Weighted York-2

Inverse Isochron			39(k)/40(a+r) ± 2σ	36(a)/40(a+r) ± 2σ	r.i.
17D20353	1.8 %		0.0069920 ± 0.0000154	0.00339228 ± 0.00001924	0.0004
17D20355	1.9 %		0.0099407 ± 0.0000271	0.00338928 ± 0.00001951	0.0008
17D20356	2.0 %		0.0123296 ± 0.0000460	0.00338516 ± 0.00001968	0.0014
17D20358	2.2 %		0.0151780 ± 0.0000621	0.00338131 ± 0.00001998	0.0022
17D20359	2.4 %		0.0189991 ± 0.0000779	0.00338586 ± 0.00002022	0.0033
17D20361	2.7 %		0.0237108 ± 0.0000875	0.00337818 ± 0.00002056	0.0041
17D20362	3.0 %		0.0264755 ± 0.0001031	0.00337757 ± 0.00002077	0.0053
17D20364	3.4 %		0.0367373 ± 0.0001146	0.00336992 ± 0.00002094	0.0072
17D20365	3.9 %	✓	0.0444220 ± 0.0001280	0.00337992 ± 0.00002125	0.0092
17D20367	4.5 %	✓	0.0493159 ± 0.0001583	0.00337081 ± 0.00002191	0.0127
17D20368	5.2 %	✓	0.0710695 ± 0.0001504	0.00335046 ± 0.00002136	0.0120
17D20370	6.0 %	✓	0.0856350 ± 0.0001964	0.00332555 ± 0.00002174	0.0187
17D20371	6.9 %	✓	0.1012892 ± 0.0002219	0.00330297 ± 0.00002246	0.0218
17D20373	7.9 %	✓	0.1254734 ± 0.0002762	0.00328226 ± 0.00002391	0.0287
17D20374	9.0 %	✓	0.1521349 ± 0.0003504	0.00325527 ± 0.00002461	0.0425
17D20376	10.3 %	✓	0.1857342 ± 0.0004633	0.00322833 ± 0.00002670	0.0631
17D20377	11.6 %	✓	0.2098916 ± 0.0006160	0.00319565 ± 0.00002976	0.0855
17D20379	12.5 %	✓	0.2264628 ± 0.0009125	0.00316991 ± 0.00003559	0.1190
17D20380	13.4 %	✓	0.2347256 ± 0.0012177	0.00312523 ± 0.00003933	0.1505
17D20382	14.6 %	✓	0.2470326 ± 0.0013390	0.00316066 ± 0.00004061	0.1746
17D20383	16.0 %	✓	0.2512387 ± 0.0014899	0.00313670 ± 0.00004194	0.1848
17D20385	17.6 %	✓	0.2469305 ± 0.0015123	0.00311046 ± 0.00004205	0.1993
17D20386	19.3 %	✓	0.2449352 ± 0.0017852	0.00310002 ± 0.00004814	0.1952
17D20388	21.0 %	✓	0.2361468 ± 0.0017659	0.00314202 ± 0.00004988	0.1964

Results	40(a)/36(a) ± 2σ		40(r)/39(k) ± 2σ		Age ± 2σ (ka)		MSWD
**Inverse Isochron**	**291.41**	**± 1.23**<br>**± 0.42%**	0.34706	± 0.02706<br>± 7.80%	**976.9**	**± 76.2**<br>**± 7.80%**	0.82<br>65%
					Full External Error	± 79.3	
					Analytical Error	± 76.1	

Statistics				
**Statistics**	2σ Confidence Limit	1.76	Convergence	0.0009773618
	Error Magnification	1.0000	Number of Iterations	3
	Number of Data Points	16	Calculated Line	Weighted York-2
	Spreading Factor	7.2%		

**Degassing Patterns**			36Ar(a) [fA]	%1σ	36Ar(c) [fA]	%1σ	36Ar(ca) [fA]	%1σ	36Ar(cl) [fA]	%1σ	37Ar(ca) [fA]	%1σ	38Ar(a) [fA]	%1σ	38Ar(c) [fA]	%1σ
17D20353	1.8 %		13.113439	0.28	0.0000000	0.00	0.0051961	2.46	0.0000120	49.31	19.2235	2.46	2.4509018	0.28	0.0000000	0.00
17D20355	1.9 %		6.869542	0.29	0.0000000	0.00	0.0051390	2.31	0.0000167	33.96	19.0122	2.30	1.2839174	0.29	0.0000000	0.00
17D20356	2.0 %		3.751765	0.29	0.0000000	0.00	0.0044135	2.88	0.0000093	61.04	16.3281	2.88	0.7012049	0.29	0.0000000	0.00
17D20358	2.2 %		2.738099	0.30	0.0000000	0.00	0.0045690	2.89	0.0000007	743.59	16.9035	2.88	0.5117507	0.30	0.0000000	0.00
17D20359	2.4 %		2.140499	0.30	0.0000000	0.00	0.0055326	2.29	0.0000000	0.00	20.4683	2.29	0.4000593	0.30	0.0000000	0.00
17D20361	2.7 %		1.961248	0.30	0.0000000	0.00	0.0075132	1.75	0.0000000	0.00	27.7958	1.74	0.3665573	0.30	0.0000000	0.00
17D20362	3.0 %		1.675967	0.31	0.0000000	0.00	0.0079324	1.69	0.0000036	161.00	29.3466	1.68	0.3132381	0.31	0.0000000	0.00
17D20364	3.4 %		1.585327	0.31	0.0000000	0.00	0.0125870	1.09	0.0000003	######	46.5667	1.08	0.2962976	0.31	0.0000000	0.00
17D20365	3.9 %	✓	1.450999	0.31	0.0000000	0.00	0.0160519	0.94	0.0000000	0.00	59.3857	0.93	0.2711916	0.31	0.0000000	0.00
17D20367	4.5 %	✓	1.135001	0.32	0.0000000	0.00	0.0158912	0.96	0.0000000	0.00	58.7910	0.94	0.2121316	0.32	0.0000000	0.00
17D20368	5.2 %	✓	1.447361	0.32	0.0000000	0.00	0.0372805	0.61	0.0000000	0.00	137.9227	0.58	0.2705118	0.32	0.0000000	0.00
17D20370	6.0 %	✓	1.082402	0.33	0.0000000	0.00	0.0396318	0.59	0.0000003	######	146.6216	0.56	0.2023010	0.33	0.0000000	0.00
17D20371	6.9 %	✓	0.986472	0.34	0.0000000	0.00	0.0512233	0.55	0.0000000	0.00	189.5054	0.52	0.1843717	0.34	0.0000000	0.00
17D20373	7.9 %	✓	0.830069	0.36	0.0000000	0.00	0.0636426	0.53	0.0000000	0.00	235.4515	0.50	0.1551400	0.36	0.0000000	0.00
17D20374	9.0 %	✓	0.639293	0.38	0.0000000	0.00	0.0690729	0.53	0.0000000	0.00	255.5415	0.50	0.1194839	0.38	0.0000000	0.00
17D20376	10.3 %	✓	0.476283	0.41	0.0000000	0.00	0.0707618	0.53	0.0000000	0.00	261.7900	0.50	0.0890172	0.41	0.0000000	0.00
17D20377	11.6 %	✓	0.349264	0.46	0.0000000	0.00	0.0633883	0.54	0.0000000	0.00	234.5109	0.51	0.0652775	0.46	0.0000000	0.00
17D20379	12.5 %	✓	0.226960	0.55	0.0000000	0.00	0.0465954	0.57	0.0000000	0.00	172.3840	0.54	0.0424189	0.55	0.0000000	0.00
17D20380	13.4 %	✓	0.164109	0.61	0.0000000	0.00	0.0361437	0.60	0.0000000	0.00	133.7169	0.58	0.0306720	0.61	0.0000000	0.00
17D20382	14.6 %	✓	0.148638	0.62	0.0000000	0.00	0.0353100	0.61	0.0000000	0.00	130.6327	0.58	0.0277805	0.62	0.0000000	0.00
17D20383	16.0 %	✓	0.134140	0.64	0.0000000	0.00	0.0333779	0.62	0.0000023	237.00	123.4845	0.60	0.0250707	0.64	0.0000000	0.00
17D20385	17.6 %	✓	0.126518	0.64	0.0000000	0.00	0.0317233	0.62	0.0000031	181.92	117.3632	0.60	0.0236462	0.64	0.0000000	0.00
17D20386	19.3 %	✓	0.108318	0.74	0.0000000	0.00	0.0266904	0.67	0.0000000	0.00	98.7437	0.65	0.0202447	0.74	0.0000000	0.00
17D20388	21.0 %	✓	0.106960	0.76	0.0000000	0.00	0.0316818	0.64	0.0000000	0.00	117.2098	0.62	0.0199908	0.76	0.0000000	0.00
		Σ	43.248675	0.11	0.0000000	0.00	0.7213496	0.15	0.0000484	35.28	2668.6999	0.14	8.0831774	0.11	0.0000000	0.00
		Σ							43.970073	0.11	2668.6999	0.14				

**Degassing Patterns**	38Ar(k) [fA]	%1σ	38Ar(ca) [fA]	%1σ	38Ar(cl) [fA]	%1σ	39Ar(k) [fA]	%1σ	39Ar(ca) [fA]	%1σ	40Ar(r) [fA]	%1σ	40Ar(a) [fA]	%1σ	40Ar(c) [fA]	%1σ	40Ar(k) [fA]	%1σ
17D20353	0.3264264	0.14	0.0034602	9.94	0.0507997	49.31	27.02877	0.11	0.0123511	2.62	44.28900	30.55	3821.387	0.35	0.0000000	0.00	0.0164065	9.65
17D20355	0.2433317	0.16	0.0034222	9.90	0.0707541	33.97	20.14836	0.14	0.0122153	2.48	24.99267	28.63	2001.853	0.36	0.0000000	0.00	0.0122301	9.65
17D20356	0.1650307	0.21	0.0029391	10.05	0.0395455	61.05	13.66487	0.19	0.0104908	3.02	14.99696	26.22	1093.302	0.36	0.0000000	0.00	0.0082946	9.65
17D20358	0.1484361	0.22	0.0030426	10.05	0.0031489	743.59	12.29081	0.20	0.0108605	3.03	11.86625	24.46	797.909	0.36	0.0000000	0.00	0.0074605	9.65
17D20359	0.1450564	0.22	0.0036843	9.90	0.0000000	0.00	12.01097	0.20	0.0131509	2.46	8.42473	27.11	623.763	0.37	0.0000000	0.00	0.0072907	9.65
17D20361	0.1662475	0.20	0.0050032	9.79	0.0000000	0.00	13.76563	0.18	0.0178588	1.97	9.03568	23.46	571.527	0.37	0.0000000	0.00	0.0083557	9.65
17D20362	0.1586585	0.21	0.0052824	9.78	0.0152009	161.01	13.13724	0.19	0.0188552	1.92	7.81083	23.35	488.393	0.37	0.0000000	0.00	0.0079743	9.65
17D20364	0.2087210	0.18	0.0083820	9.69	0.0010682	######	17.28252	0.15	0.0299191	1.42	8.45453	20.55	461.980	0.38	0.0000000	0.00	0.0104905	9.65
17D20365	0.2303122	0.17	0.0106894	9.67	0.0000000	0.00	19.07031	0.14	0.0381553	1.31	6.46361	24.81	422.836	0.38	0.0000000	0.00	0.0115757	9.65
17D20367	0.2005433	0.18	0.0105824	9.68	0.0000000	0.00	16.60539	0.16	0.0377732	1.32	5.96421	21.52	330.751	0.39	0.0000000	0.00	0.0100795	9.65
17D20368	0.3707784	0.14	0.0248261	9.65	0.0000000	0.00	30.70120	0.10	0.0886153	1.09	10.21266	15.81	421.775	0.38	0.0000000	0.00	0.0186356	9.65
17D20370	0.3366164	0.14	0.0263919	9.65	0.0011863	######	27.87252	0.11	0.0942044	1.08	10.05741	12.22	315.423	0.39	0.0000000	0.00	0.0169186	9.65
17D20371	0.3653444	0.14	0.0341110	9.64	0.0000000	0.00	30.25126	0.11	0.1217572	1.06	11.19428	10.29	287.468	0.40	0.0000000	0.00	0.0183625	9.65
17D20373	0.3832234	0.14	0.0423813	9.64	0.0000000	0.00	31.73167	0.10	0.1512776	1.05	11.00512	9.26	241.891	0.42	0.0000000	0.00	0.0192611	9.65
17D20374	0.3608292	0.14	0.0459975	9.64	0.0000000	0.00	29.87738	0.11	0.1641854	1.05	10.09098	8.01	186.296	0.43	0.0000000	0.00	0.0181356	9.65
17D20376	0.3309313	0.14	0.0471222	9.64	0.0000000	0.00	27.40178	0.11	0.1682001	1.05	8.73871	7.39	138.794	0.46	0.0000000	0.00	0.0166329	9.65
17D20377	0.2770443	0.15	0.0422120	9.64	0.0000000	0.00	22.93983	0.13	0.1506733	1.05	7.51457	6.94	101.779	0.51	0.0000000	0.00	0.0139245	9.65
17D20379	0.1958207	0.19	0.0310291	9.65	0.0000000	0.00	16.21435	0.16	0.1107567	1.07	5.45974	7.29	66.139	0.59	0.0000000	0.00	0.0098421	9.65
17D20380	0.1488570	0.23	0.0240690	9.65	0.0000000	0.00	12.32566	0.21	0.0859131	1.09	4.68795	6.81	47.823	0.65	0.0000000	0.00	0.0074817	9.65
17D20382	0.1403029	0.23	0.0235139	9.65	0.0000000	0.00	11.61737	0.21	0.0839315	1.09	3.71295	7.94	43.315	0.65	0.0000000	0.00	0.0070517	9.65
17D20383	0.1297565	0.24	0.0222272	9.65	0.0097929	237.00	10.74410	0.23	0.0793388	1.10	3.67490	7.51	39.090	0.67	0.0000000	0.00	0.0065217	9.65
17D20385	0.1213003	0.25	0.0211254	9.65	0.0131886	181.92	10.04391	0.23	0.0754058	1.10	3.80644	6.92	36.869	0.68	0.0000000	0.00	0.0060967	9.65
17D20386	0.1033590	0.29	0.0177739	9.65	0.0000000	0.00	8.55834	0.28	0.0634428	1.13	3.37617	7.60	31.565	0.77	0.0000000	0.00	0.0051949	9.65
17D20388	0.0970854	0.30	0.0210978	9.65	0.0000000	0.00	8.03886	0.29	0.0753073	1.11	2.87259	8.99	31.169	0.79	0.0000000	0.00	0.0048796	9.65
Σ	5.3540130	0.04	0.4803660	2.43	0.2046851	35.27	443.32309	0.03	1.7146397	0.27	238.70292	7.10	12603.097	0.13	0.0000000	0.00	0.2690971	2.14
Σ					14.122242	0.52			445.03773	0.03							12842.069	0.19

Additional Parameters			40Ar/39Ar	1σ	37Ar/39Ar	1σ	36Ar/39Ar	1σ	Time (days)	37Ar (decay)	39Ar (decay)	40Ar (moles)
17D20353	1.8 %		142.956101	0.157826	0.710897	0.017494	0.485137	0.001476	145.499	17.749729	1.00102805	1.856E-10
17D20355	1.9 %		100.535752	0.136882	0.943038	0.021726	0.340997	0.001085	145.513	17.754599	1.00102815	9.729E-11
17D20356	2.0 %		81.044076	0.150957	1.193980	0.034428	0.274668	0.000947	145.520	17.757035	1.00102820	5.320E-11
17D20358	2.2 %		65.827076	0.134596	1.374085	0.039720	0.222951	0.000799	145.534	17.761907	1.00102830	3.887E-11
17D20359	2.4 %		52.577246	0.107600	1.702272	0.039054	0.178477	0.000644	145.541	17.764343	1.00102835	3.035E-11
17D20361	2.7 %		42.120783	0.077611	2.016598	0.035318	0.142835	0.000506	145.555	17.769217	1.00102845	2.787E-11
17D20362	3.0 %		37.717279	0.073297	2.230649	0.037773	0.127994	0.000463	145.562	17.771655	1.00102849	2.382E-11
17D20364	3.4 %		27.173826	0.042306	2.689784	0.029234	0.092299	0.000318	145.576	17.776531	1.00102859	2.258E-11
17D20365	3.9 %	✓	22.467039	0.032300	3.107820	0.029107	0.076775	0.000262	145.583	17.778969	1.00102864	2.061E-11
17D20367	4.5 %	✓	20.232020	0.032397	3.532443	0.033678	0.069151	0.000246	145.597	17.783847	1.00102874	1.616E-11
17D20368	5.2 %	✓	14.030833	0.014803	4.479490	0.026532	0.048219	0.000157	145.603	17.786287	1.00102879	2.074E-11
17D20370	6.0 %	✓	11.638733	0.013298	5.242716	0.030162	0.040120	0.000133	145.617	17.791167	1.00102889	1.562E-11
17D20371	6.9 %	✓	9.833749	0.010723	6.239269	0.033130	0.034165	0.000115	145.624	17.793608	1.00102894	1.434E-11
17D20373	7.9 %	✓	7.932607	0.008685	7.384873	0.038006	0.028031	0.000098	145.639	17.798734	1.00102904	1.214E-11
17D20374	9.0 %	✓	6.537795	0.007485	8.506262	0.043211	0.023580	0.000083	145.646	17.801175	1.00102909	9.427E-12
17D20376	10.3 %	✓	5.351795	0.006633	9.495473	0.048463	0.019842	0.000073	145.660	17.806059	1.00102919	7.082E-12
17D20377	11.6 %	✓	4.733878	0.006905	10.156165	0.053063	0.017871	0.000071	145.667	17.808502	1.00102924	5.247E-12
17D20379	12.5 %	✓	4.386381	0.008791	10.559446	0.059749	0.016757	0.000079	145.681	17.813388	1.00102933	3.437E-12
17D20380	13.4 %	✓	4.231407	0.010922	10.773567	0.066281	0.016134	0.000085	145.688	17.815832	1.00102938	2.521E-12
17D20382	14.6 %	✓	4.019615	0.010843	11.163949	0.069050	0.015720	0.000083	145.701	17.820720	1.00102948	2.258E-12
17D20383	16.0 %	✓	3.951704	0.011662	11.408989	0.072914	0.015477	0.000085	145.708	17.823164	1.00102953	2.053E-12
17D20385	17.6 %	✓	4.020148	0.012254	11.597936	0.074542	0.015638	0.000086	145.722	17.828055	1.00102963	1.953E-12
17D20386	19.3 %	✓	4.053272	0.014702	11.452823	0.081209	0.015659	0.000100	145.729	17.830500	1.00102968	1.677E-12
17D20388	21.0 %	✓	4.195955	0.015597	14.445069	0.098084	0.017086	0.000108	145.743	17.835392	1.00102977	1.634E-12

Procedure Blanks		36Ar ± 1σ (SE) [fA]	37Ar ± 1σ (SE) [fA]	38Ar ± 1σ (SE) [fA]	39Ar ± 1σ (SE) [fA]	40Ar ± 1σ (SE) [fA]
17D20353	1.8 %	0.0065341 ± 0.0002311	0.1410154 ± 0.0183797	0.0266686 ± 0.0167959	0.0241245 ± 0.0161085	1.8117119 ± 0.0798128
17D20355	1.9 %	0.0064007 ± 0.0002311	0.1367666 ± 0.0183797	0.0330496 ± 0.0167959	0.0341359 ± 0.0161085	1.8204695 ± 0.0798128
17D20356	2.0 %	0.0064077 ± 0.0002311	0.1366979 ± 0.0183797	0.0348457 ± 0.0167959	0.0366741 ± 0.0161085	1.8360481 ± 0.0798128
17D20358	2.2 %	0.0065227 ± 0.0002311	0.1394965 ± 0.0183797	0.0362554 ± 0.0167959	0.0379070 ± 0.0161085	1.8815404 ± 0.0798128
17D20359	2.4 %	0.0066143 ± 0.0002311	0.1419419 ± 0.0183797	0.0360905 ± 0.0167959	0.0370030 ± 0.0161085	1.9085852 ± 0.0798128
17D20361	2.7 %	0.0068291 ± 0.0002311	0.1479908 ± 0.0183797	0.0345233 ± 0.0167959	0.0330693 ± 0.0161085	1.9649914 ± 0.0798128
17D20362	3.0 %	0.0069401 ± 0.0002311	0.1512685 ± 0.0183797	0.0333006 ± 0.0167959	0.0303707 ± 0.0161085	1.9921975 ± 0.0798128
17D20364	3.4 %	0.0071428 ± 0.0002311	0.1576368 ± 0.0183797	0.0303747 ± 0.0167959	0.0242493 ± 0.0161085	2.0401194 ± 0.0798128
17D20365	3.9 %	0.0072263 ± 0.0002311	0.1604980 ± 0.0183797	0.0288095 ± 0.0167959	0.0210871 ± 0.0161085	2.0593935 ± 0.0798128
17D20367	4.5 %	0.0073414 ± 0.0002311	0.1651214 ± 0.0183797	0.0257672 ± 0.0167959	0.0151236 ± 0.0161085	2.0858557 ± 0.0798128
17D20368	5.2 %	0.0073687 ± 0.0002311	0.1667506 ± 0.0183797	0.0243865 ± 0.0167959	0.0125126 ± 0.0161085	2.0923157 ± 0.0798128
17D20370	6.0 %	0.0073571 ± 0.0002311	0.1684315 ± 0.0183797	0.0220942 ± 0.0167959	0.0084196 ± 0.0161085	2.0907655 ± 0.0798128
17D20371	6.9 %	0.0073178 ± 0.0002311	0.1684464 ± 0.0183797	0.0212371 ± 0.0167959	0.0070577 ± 0.0161085	2.0827408 ± 0.0798128
17D20373	7.9 %	0.0071678 ± 0.0002311	0.1667198 ± 0.0183797	0.0201564 ± 0.0167959	0.0059159 ± 0.0161085	2.0511211 ± 0.0798128
17D20374	9.0 %	0.0070682 ± 0.0002311	0.1651253 ± 0.0183797	0.0199970 ± 0.0167959	0.0062417 ± 0.0161085	2.0297850 ± 0.0798128
17D20376	10.3 %	0.0068296 ± 0.0002311	0.1607217 ± 0.0183797	0.0203419 ± 0.0167959	0.0086085 ± 0.0161085	1.9776980 ± 0.0798128
17D20377	11.6 %	0.0066986 ± 0.0002311	0.1580716 ± 0.0183797	0.0208158 ± 0.0167959	0.0106265 ± 0.0161085	1.9483837 ± 0.0798128
17D20379	12.5 %	0.0064378 ± 0.0002311	0.1523928 ± 0.0183797	0.0222381 ± 0.0167959	0.0161858 ± 0.0161085	1.8876973 ± 0.0798128
17D20380	13.4 %	0.0063201 ± 0.0002311	0.1496191 ± 0.0183797	0.0231142 ± 0.0167959	0.0196337 ± 0.0161085	1.8584753 ± 0.0798128
17D20382	14.6 %	0.0061452 ± 0.0002311	0.1449620 ± 0.0183797	0.0249634 ± 0.0167959	0.0275444 ± 0.0161085	1.8085418 ± 0.0798128
17D20383	16.0 %	0.0061040 ± 0.0002311	0.1434300 ± 0.0183797	0.0258225 ± 0.0167959	0.0318435 ± 0.0161085	1.7906941 ± 0.0798128
17D20385	17.6 %	0.0061595 ± 0.0002311	0.1429581 ± 0.0183797	0.0270722 ± 0.0167959	0.0406316 ± 0.0161085	1.7772887 ± 0.0798128
17D20386	19.3 %	0.0062762 ± 0.0002311	0.1444661 ± 0.0183797	0.0273070 ± 0.0167959	0.0448865 ± 0.0161085	1.7853085 ± 0.0798128
17D20388	21.0 %	0.0067430 ± 0.0002311	0.1522096 ± 0.0183797	0.0265547 ± 0.0167959	0.0524447 ± 0.0161085	1.8406291 ± 0.0798128

Intercept Values		36Ar ± 1σ (SE) [fA]	r2	Regression (type,n)	37Ar ± 1σ (SE) [fA]	r2	Regression (type,n)	38Ar ± 1σ (SE) [fA]	r2	Regression (type,n)	39Ar ± 1σ (SE) [fA]	r2	Regression (type,n)	40Ar ± 1σ (SE) [fA]	r2	Regression (type,n)
17D20353	1.8 %	12.6322517 ± 0.0052081	0.9968	EXP 150 of 150	0.9233873 ± 0.0179667	0.0291	EXP 150 of 150	2.7724138 ± 0.0163721	0.3888	EXP 150 of 150	26.8344416 ± 0.0166372	0.9832	EXP 150 of 150	3867.504473 ± 0.108645	0.9999	EXP 150 of 150
17D20355	1.9 %	6.6227834 ± 0.0041924	0.9921	EXP 150 of 150	0.9156495 ± 0.0149802	0.0436	EXP 150 of 150	1.5499919 ± 0.0162228	0.2156	EXP 150 of 150	19.9903310 ± 0.0171014	0.9720	EXP 150 of 150	2028.678587 ± 0.077735	0.9999	EXP 150 of 150
17D20356	2.0 %	3.6214578 ± 0.0026708	0.9889	EXP 150 of 150	0.7670180 ± 0.0179162	0.1229	EXP 150 of 150	0.8634425 ± 0.0167731	0.0649	EXP 150 of 150	13.5463655 ± 0.0170805	0.9427	EXP 150 of 150	1110.143160 ± 0.054066	0.9996	EXP 150 of 150
17D20358	2.2 %	2.6461366 ± 0.0023795	0.9840	EXP 150 of 150	0.7958113 ± 0.0192581	0.0355	EXP 150 of 150	0.6224732 ± 0.0158221	0.0103	EXP 150 of 150	12.1807162 ± 0.0170839	0.9309	EXP 150 of 150	811.664736 ± 0.050072	0.9993	EXP 150 of 150
17D20359	2.4 %	2.0720095 ± 0.0020308	0.9796	EXP 150 of 150	0.9904568 ± 0.0174489	0.1738	EXP 149 of 150	0.4973675 ± 0.0167846	0.0003	EXP 150 of 150	11.9059360 ± 0.0163518	0.9404	EXP 150 of 150	634.103482 ± 0.041125	0.9989	EXP 150 of 150
17D20361	2.7 %	1.9016151 ± 0.0021470	0.9743	EXP 150 of 150	1.3893726 ± 0.0181334	0.1478	EXP 150 of 150	0.4761756 ± 0.0159693	0.0008	EXP 150 of 150	13.6573659 ± 0.0168403	0.9509	EXP 150 of 150	582.536441 ± 0.038471	0.9990	EXP 150 of 150
17D20362	3.0 %	1.6275704 ± 0.0019425	0.9698	EXP 150 of 150	1.4716504 ± 0.0187475	0.1668	EXP 150 of 150	0.4534269 ± 0.0173606	0.0237	EXP 150 of 150	13.0369062 ± 0.0172913	0.9458	EXP 150 of 150	498.204413 ± 0.037756	0.9981	EXP 150 of 150
17D20364	3.4 %	1.5450158 ± 0.0019264	0.9676	EXP 150 of 150	2.4168751 ± 0.0169732	0.3636	EXP 149 of 150	0.4781881 ± 0.0151354	0.0081	EXP 149 of 150	17.1713040 ± 0.0175116	0.9709	EXP 150 of 150	472.485263 ± 0.036820	0.9982	EXP 150 of 150
17D20365	3.9 %	1.4191528 ± 0.0018548	0.9650	EXP 150 of 150	3.1222792 ± 0.0188677	0.4755	EXP 150 of 150	0.4676976 ± 0.0179268	0.0160	EXP 150 of 150	18.9583706 ± 0.0172996	0.9768	EXP 150 of 150	431.370080 ± 0.035740	0.9972	EXP 150 of 150
17D20367	4.5 %	1.1149887 ± 0.0016717	0.9527	EXP 150 of 150	3.0838928 ± 0.0192445	0.4886	EXP 150 of 150	0.3773840 ± 0.0176096	0.0001	EXP 150 of 150	16.5156712 ± 0.0171318	0.9711	EXP 150 of 150	338.810654 ± 0.033751	0.9933	EXP 150 of 150
17D20368	5.2 %	1.4362251 ± 0.0018636	0.9646	EXP 150 of 150	7.4543320 ± 0.0199867	0.8169	EXP 150 of 150	0.6229508 ± 0.0164947	0.0294	EXP 150 of 150	30.5694208 ± 0.0173280	0.9917	EXP 150 of 150	434.099066 ± 0.034017	0.9980	EXP 150 of 150
17D20370	6.0 %	1.0872314 ± 0.0014956	0.9583	EXP 150 of 150	7.9310978 ± 0.0188121	0.8424	EXP 150 of 150	0.5378982 ± 0.0182461	0.0361	EXP 150 of 150	27.7694794 ± 0.0181099	0.9891	EXP 150 of 150	327.587980 ± 0.032254	0.9937	EXP 150 of 150
17D20371	6.9 %	1.0060225 ± 0.0015406	0.9459	EXP 150 of 150	10.2985942 ± 0.0171507	0.9175	EXP 150 of 150	0.5313515 ± 0.0166108	0.0154	EXP 150 of 150	30.1608856 ± 0.0178048	0.9912	EXP 150 of 150	300.763329 ± 0.029667	0.9896	EXP 150 of 150
17D20373	7.9 %	0.8672989 ± 0.0015528	0.9252	EXP 150 of 150	12.8343397 ± 0.0187562	0.9383	EXP 150 of 150	0.5145823 ± 0.0162614	0.0130	EXP 150 of 150	31.6617661 ± 0.0187575	0.9910	EXP 150 of 150	254.966051 ± 0.031239	0.9659	EXP 150 of 150
17D20374	9.0 %	0.6888178 ± 0.0012286	0.9237	EXP 150 of 150	13.9433155 ± 0.0181682	0.9483	EXP 150 of 150	0.4511187 ± 0.0161443	0.0012	EXP 150 of 150	29.8324893 ± 0.0179011	0.9908	EXP 149 of 150	198.435393 ± 0.027749	0.8510	EXP 150 of 150
17D20376	10.3 %	0.5333191 ± 0.0010777	0.8841	EXP 150 of 150	14.2887368 ± 0.0198752	0.9425	EXP 150 of 150	0.4177090 ± 0.0156255	0.0229	EXP 150 of 150	27.3752192 ± 0.0171174	0.9899	EXP 150 of 150	149.526584 ± 0.026574	0.9839	EXP 150 of 150
17D20377	11.6 %	0.4038459 ± 0.0009914	0.8204	EXP 150 of 150	12.7839465 ± 0.0200808	0.9331	EXP 150 of 150	0.3169845 ± 0.0176597	0.0000	EXP 150 of 150	22.9239645 ± 0.0174096	0.9854	EXP 150 of 150	111.255992 ± 0.024620	0.9951	EXP 150 of 150
17D20379	12.5 %	0.2697149 ± 0.0008811	0.6895	EXP 150 of 150	9.3584029 ± 0.0196720	0.8789	EXP 150 of 150	0.2290307 ± 0.0146765	0.0214	EXP 150 of 150	16.1986874 ± 0.0178357	0.9676	EXP 150 of 150	73.495825 ± 0.023012	0.9974	EXP 150 of 150
17D20380	13.4 %	0.1990483 ± 0.0007325	0.4867	EXP 150 of 150	7.2268179 ± 0.0182160	0.8335	EXP 150 of 150	0.1323003 ± 0.0147782	0.0005	EXP 150 of 150	12.3081365 ± 0.0175482	0.9480	EXP 150 of 150	54.376907 ± 0.020024	0.9984	EXP 150 of 150
17D20382	14.6 %	0.1831818 ± 0.0006631	0.5500	EXP 150 of 150	7.0593567 ± 0.0178976	0.8356	EXP 150 of 150	0.1616805 ± 0.0176119	0.0002	EXP 150 of 150	11.5947421 ± 0.0157752	0.9532	EXP 150 of 150	48.843251 ± 0.018775	0.9985	EXP 150 of 150
17D20383	16.0 %	0.1673293 ± 0.0006212	0.4576	EXP 150 of 150	6.6657385 ± 0.0182326	0.8320	EXP 149 of 150	0.1588799 ± 0.0154769	0.0233	EXP 150 of 150	10.7185149 ± 0.0164067	0.9382	EXP 150 of 150	44.561737 ± 0.018082	0.9987	EXP 150 of 150
17D20385	17.6 %	0.1584579 ± 0.0005865	0.4238	EXP 150 of 150	6.3268912 ± 0.0167815	0.8188	EXP 150 of 150	0.1501305 ± 0.0166161	0.0063	EXP 150 of 150	10.0103526 ± 0.0146514	0.9438	EXP 150 of 150	42.458428 ± 0.021298	0.9980	EXP 150 of 150
17D20386	19.3 %	0.1362121 ± 0.0006155	0.2275	EXP 150 of 150	5.2982078 ± 0.0171202	0.7469	EXP 150 of 150	0.1072349 ± 0.0173220	0.0001	EXP 150 of 150	8.5186753 ± 0.0163006	0.8963	EXP 150 of 150	36.731728 ± 0.019313	0.9985	EXP 150 of 150
17D20388	21.0 %	0.1401754 ± 0.0006119	0.3426	EXP 150 of 150	6.3065259 ± 0.0190181	0.7968	EXP 150 of 150	0.0678767 ± 0.0172912	0.0086	EXP 150 of 150	8.0069357 ± 0.0151223	0.9088	EXP 150 of 150	35.887327 ± 0.019618	0.9983	EXP 150 of 150

Project Info		Analyst	Irradiation	X-pos	Y-pos	Z/H-pos	Project	Experiment	Nmb
17D20353	1.8 %	Dan Miggins	17-OSU-01	0.00	0.00	53.44	Arctic\O-Connor (16-22)	17D20349	01
17D20355	1.9 %	Dan Miggins	17-OSU-01	0.00	0.00	53.44	Arctic\O-Connor (16-22)	17D20349	01
17D20356	2.0 %	Dan Miggins	17-OSU-01	0.00	0.00	53.44	Arctic\O-Connor (16-22)	17D20349	01
17D20358	2.2 %	Dan Miggins	17-OSU-01	0.00	0.00	53.44	Arctic\O-Connor (16-22)	17D20349	01
17D20359	2.4 %	Dan Miggins	17-OSU-01	0.00	0.00	53.44	Arctic\O-Connor (16-22)	17D20349	01
17D20361	2.7 %	Dan Miggins	17-OSU-01	0.00	0.00	53.44	Arctic\O-Connor (16-22)	17D20349	01
17D20362	3.0 %	Dan Miggins	17-OSU-01	0.00	0.00	53.44	Arctic\O-Connor (16-22)	17D20349	01
17D20364	3.4 %	Dan Miggins	17-OSU-01	0.00	0.00	53.44	Arctic\O-Connor (16-22)	17D20349	01
17D20365	3.9 %	Dan Miggins	17-OSU-01	0.00	0.00	53.44	Arctic\O-Connor (16-22)	17D20349	01
17D20367	4.5 %	Dan Miggins	17-OSU-01	0.00	0.00	53.44	Arctic\O-Connor (16-22)	17D20349	01
17D20368	5.2 %	Dan Miggins	17-OSU-01	0.00	0.00	53.44	Arctic\O-Connor (16-22)	17D20349	01
17D20370	6.0 %	Dan Miggins	17-OSU-01	0.00	0.00	53.44	Arctic\O-Connor (16-22)	17D20349	01
17D20371	6.9 %	Dan Miggins	17-OSU-01	0.00	0.00	53.44	Arctic\O-Connor (16-22)	17D20349	01
17D20373	7.9 %	Dan Miggins	17-OSU-01	0.00	0.00	53.44	Arctic\O-Connor (16-22)	17D20349	01
17D20374	9.0 %	Dan Miggins	17-OSU-01	0.00	0.00	53.44	Arctic\O-Connor (16-22)	17D20349	01
17D20376	10.3 %	Dan Miggins	17-OSU-01	0.00	0.00	53.44	Arctic\O-Connor (16-22)	17D20349	01
17D20377	11.6 %	Dan Miggins	17-OSU-01	0.00	0.00	53.44	Arctic\O-Connor (16-22)	17D20349	01
17D20379	12.5 %	Dan Miggins	17-OSU-01	0.00	0.00	53.44	Arctic\O-Connor (16-22)	17D20349	01
17D20380	13.4 %	Dan Miggins	17-OSU-01	0.00	0.00	53.44	Arctic\O-Connor (16-22)	17D20349	01
17D20382	14.6 %	Dan Miggins	17-OSU-01	0.00	0.00	53.44	Arctic\O-Connor (16-22)	17D20349	01
17D20383	16.0 %	Dan Miggins	17-OSU-01	0.00	0.00	53.44	Arctic\O-Connor (16-22)	17D20349	01
17D20385	17.6 %	Dan Miggins	17-OSU-01	0.00	0.00	53.44	Arctic\O-Connor (16-22)	17D20349	01
17D20386	19.3 %	Dan Miggins	17-OSU-01	0.00	0.00	53.44	Arctic\O-Connor (16-22)	17D20349	01
17D20388	21.0 %	Dan Miggins	17-OSU-01	0.00	0.00	53.44	Arctic\O-Connor (16-22)	17D20349	01

Sample Parameters		Sample	Material	Location	Standard Name	Standard (in Ma)	%1σ	Standard Reference	Standard 40Ar/39Ar	%1σ	J	%1σ	Air 40Ar/36Ar	%1σ	MDF (lin)	%1σ	Volume Ratio	Sensitivity (mol/volt)	Day	Month	Year	Hour	Min	Resist
17D20353	1.8 %	PS59-305-1	Groundmass	Gakkel Ridge	FCT-NM (1C35-17)	28.201	0.082	Kuiper et al (2008)	10.09436	0.084	0.00155705	0.084	302.436	0.15	0.9942591	0.069	1	4.8E-14	14	JUN	2017	3	38	1
17D20355	1.9 %	PS59-305-1	Groundmass	Gakkel Ridge	FCT-NM (1C35-17)	28.201	0.082	Kuiper et al (2008)	10.09436	0.084	0.00155705	0.084	302.436	0.15	0.9942591	0.069	1	4.8E-14	14	JUN	2017	3	58	1
17D20356	2.0 %	PS59-305-1	Groundmass	Gakkel Ridge	FCT-NM (1C35-17)	28.201	0.082	Kuiper et al (2008)	10.09436	0.084	0.00155705	0.084	302.436	0.15	0.9942591	0.069	1	4.8E-14	14	JUN	2017	4	8	1
17D20358	2.2 %	PS59-305-1	Groundmass	Gakkel Ridge	FCT-NM (1C35-17)	28.201	0.082	Kuiper et al (2008)	10.09436	0.084	0.00155705	0.084	302.436	0.15	0.9942591	0.069	1	4.8E-14	14	JUN	2017	4	28	1
17D20359	2.4 %	PS59-305-1	Groundmass	Gakkel Ridge	FCT-NM (1C35-17)	28.201	0.082	Kuiper et al (2008)	10.09436	0.084	0.00155705	0.084	302.436	0.15	0.9942591	0.069	1	4.8E-14	14	JUN	2017	4	38	1
17D20361	2.7 %	PS59-305-1	Groundmass	Gakkel Ridge	FCT-NM (1C35-17)	28.201	0.082	Kuiper et al (2008)	10.09436	0.084	0.00155705	0.084	302.436	0.15	0.9942591	0.069	1	4.8E-14	14	JUN	2017	4	58	1
17D20362	3.0 %	PS59-305-1	Groundmass	Gakkel Ridge	FCT-NM (1C35-17)	28.201	0.082	Kuiper et al (2008)	10.09436	0.084	0.00155705	0.084	302.436	0.15	0.9942591	0.069	1	4.8E-14	14	JUN	2017	5	8	1
17D20364	3.4 %	PS59-305-1	Groundmass	Gakkel Ridge	FCT-NM (1C35-17)	28.201	0.082	Kuiper et al (2008)	10.09436	0.084	0.00155705	0.084	302.436	0.15	0.9942591	0.069	1	4.8E-14	14	JUN	2017	5	28	1
17D20365	3.9 %	PS59-305-1	Groundmass	Gakkel Ridge	FCT-NM (1C35-17)	28.201	0.082	Kuiper et al (2008)	10.09436	0.084	0.00155705	0.084	302.436	0.15	0.9942591	0.069	1	4.8E-14	14	JUN	2017	5	38	1
17D20367	4.5 %	PS59-305-1	Groundmass	Gakkel Ridge	FCT-NM (1C35-17)	28.201	0.082	Kuiper et al (2008)	10.09436	0.084	0.00155705	0.084	302.436	0.15	0.9942591	0.069	1	4.8E-14	14	JUN	2017	5	58	1
17D20368	5.2 %	PS59-305-1	Groundmass	Gakkel Ridge	FCT-NM (1C35-17)	28.201	0.082	Kuiper et al (2008)	10.09436	0.084	0.00155705	0.084	302.436	0.15	0.9942591	0.069	1	4.8E-14	14	JUN	2017	6	8	1
17D20370	6.0 %	PS59-305-1	Groundmass	Gakkel Ridge	FCT-NM (1C35-17)	28.201	0.082	Kuiper et al (2008)	10.09436	0.084	0.00155705	0.084	302.436	0.15	0.9942591	0.069	1	4.8E-14	14	JUN	2017	6	28	1
17D20371	6.9 %	PS59-305-1	Groundmass	Gakkel Ridge	FCT-NM (1C35-17)	28.201	0.082	Kuiper et al (2008)	10.09436	0.084	0.00155705	0.084	302.436	0.15	0.9942591	0.069	1	4.8E-14	14	JUN	2017	6	38	1
17D20373	7.9 %	PS59-305-1	Groundmass	Gakkel Ridge	FCT-NM (1C35-17)	28.201	0.082	Kuiper et al (2008)	10.09436	0.084	0.00155705	0.084	302.436	0.15	0.9942591	0.069	1	4.8E-14	14	JUN	2017	6	59	1
17D20374	9.0 %	PS59-305-1	Groundmass	Gakkel Ridge	FCT-NM (1C35-17)	28.201	0.082	Kuiper et al (2008)	10.09436	0.084	0.00155705	0.084	302.436	0.15	0.9942591	0.069	1	4.8E-14	14	JUN	2017	7	9	1
17D20376	10.3 %	PS59-305-1	Groundmass	Gakkel Ridge	FCT-NM (1C35-17)	28.201	0.082	Kuiper et al (2008)	10.09436	0.084	0.00155705	0.084	302.436	0.15	0.9942591	0.069	1	4.8E-14	14	JUN	2017	7	29	1
17D20377	11.6 %	PS59-305-1	Groundmass	Gakkel Ridge	FCT-NM (1C35-17)	28.201	0.082	Kuiper et al (2008)	10.09436	0.084	0.00155705	0.084	302.436	0.15	0.9942591	0.069	1	4.8E-14	14	JUN	2017	7	39	1
17D20379	12.5 %	PS59-305-1	Groundmass	Gakkel Ridge	FCT-NM (1C35-17)	28.201	0.082	Kuiper et al (2008)	10.09436	0.084	0.00155705	0.084	302.436	0.15	0.9942591	0.069	1	4.8E-14	14	JUN	2017	7	59	1
17D20380	13.4 %	PS59-305-1	Groundmass	Gakkel Ridge	FCT-NM (1C35-17)	28.201	0.082	Kuiper et al (2008)	10.09436	0.084	0.00155705	0.084	302.436	0.15	0.9942591	0.069	1	4.8E-14	14	JUN	2017	8	9	1
17D20382	14.6 %	PS59-305-1	Groundmass	Gakkel Ridge	FCT-NM (1C35-17)	28.201	0.082	Kuiper et al (2008)	10.09436	0.084	0.00155705	0.084	302.436	0.15	0.9942591	0.069	1	4.8E-14	14	JUN	2017	8	29	1
17D20383	16.0 %	PS59-305-1	Groundmass	Gakkel Ridge	FCT-NM (1C35-17)	28.201	0.082	Kuiper et al (2008)	10.09436	0.084	0.00155705	0.084	302.436	0.15	0.9942591	0.069	1	4.8E-14	14	JUN	2017	8	39	1
17D20385	17.6 %	PS59-305-1	Groundmass	Gakkel Ridge	FCT-NM (1C35-17)	28.201	0.082	Kuiper et al (2008)	10.09436	0.084	0.00155705	0.084	302.436	0.15	0.9942591	0.069	1	4.8E-14	14	JUN	2017	8	59	1
17D20386	19.3 %	PS59-305-1	Groundmass	Gakkel Ridge	FCT-NM (1C35-17)	28.201	0.082	Kuiper et al (2008)	10.09436	0.084	0.00155705	0.084	302.436	0.15	0.9942591	0.069	1	4.8E-14	14	JUN	2017	9	9	1
17D20388	21.0 %	PS59-305-1	Groundmass	Gakkel Ridge	FCT-NM (1C35-17)	28.201	0.082	Kuiper et al (2008)	10.09436	0.084	0.00155705	0.084	302.436	0.15	0.9942591	0.069	1	4.8E-14	14	JUN	2017	9	29	1

**Irradiation Constants**		40/36(a)	%1σ	40/36(c)	%1σ	38/36(a)	%1σ	38/36(c)	%1σ	39/37(ca)	%1σ	38/37(ca)	%1σ	36/37(ca)	%1σ	40/39(k)	%1σ	38/39(k)	%1σ	36/38(cl)	%1σ	K/Ca	%1σ	K/Cl	%1σ	Ca/Cl	%1σ
17D20353	1.8 %	291.41	0.212	0.018	35	0.1869	0	1.493	3	0.000643	0.92	0.00018	9.63	0.00027	0.17	0.000607	9.65	0.012077	0.09	0	0	0.43	0	0	0	0	0
17D20355	1.9 %	291.41	0.212	0.018	35	0.1869	0	1.493	3	0.000643	0.92	0.00018	9.63	0.00027	0.17	0.000607	9.65	0.012077	0.09	0	0	0.43	0	0	0	0	0
17D20356	2.0 %	291.41	0.212	0.018	35	0.1869	0	1.493	3	0.000643	0.92	0.00018	9.63	0.00027	0.17	0.000607	9.65	0.012077	0.09	0	0	0.43	0	0	0	0	0
17D20358	2.2 %	291.41	0.212	0.018	35	0.1869	0	1.493	3	0.000643	0.92	0.00018	9.63	0.00027	0.17	0.000607	9.65	0.012077	0.09	0	0	0.43	0	0	0	0	0
17D20359	2.4 %	291.41	0.212	0.018	35	0.1869	0	1.493	3	0.000643	0.92	0.00018	9.63	0.00027	0.17	0.000607	9.65	0.012077	0.09	0	0	0.43	0	0	0	0	0
17D20361	2.7 %	291.41	0.212	0.018	35	0.1869	0	1.493	3	0.000643	0.92	0.00018	9.63	0.00027	0.17	0.000607	9.65	0.012077	0.09	0	0	0.43	0	0	0	0	0
17D20362	3.0 %	291.41	0.212	0.018	35	0.1869	0	1.493	3	0.000643	0.92	0.00018	9.63	0.00027	0.17	0.000607	9.65	0.012077	0.09	0	0	0.43	0	0	0	0	0
17D20364	3.4 %	291.41	0.212	0.018	35	0.1869	0	1.493	3	0.000643	0.92	0.00018	9.63	0.00027	0.17	0.000607	9.65	0.012077	0.09	0	0	0.43	0	0	0	0	0
17D20365	3.9 %	291.41	0.212	0.018	35	0.1869	0	1.493	3	0.000643	0.92	0.00018	9.63	0.00027	0.17	0.000607	9.65	0.012077	0.09	0	0	0.43	0	0	0	0	0
17D20367	4.5 %	291.41	0.212	0.018	35	0.1869	0	1.493	3	0.000643	0.92	0.00018	9.63	0.00027	0.17	0.000607	9.65	0.012077	0.09	0	0	0.43	0	0	0	0	0
17D20368	5.2 %	291.41	0.212	0.018	35	0.1869	0	1.493	3	0.000643	0.92	0.00018	9.63	0.00027	0.17	0.000607	9.65	0.012077	0.09	0	0	0.43	0	0	0	0	0
17D20370	6.0 %	291.41	0.212	0.018	35	0.1869	0	1.493	3	0.000643	0.92	0.00018	9.63	0.00027	0.17	0.000607	9.65	0.012077	0.09	0	0	0.43	0	0	0	0	0
17D20371	6.9 %	291.41	0.212	0.018	35	0.1869	0	1.493	3	0.000643	0.92	0.00018	9.63	0.00027	0.17	0.000607	9.65	0.012077	0.09	0	0	0.43	0	0	0	0	0
17D20373	7.9 %	291.41	0.212	0.018	35	0.1869	0	1.493	3	0.000643	0.92	0.00018	9.63	0.00027	0.17	0.000607	9.65	0.012077	0.09	0	0	0.43	0	0	0	0	0
17D20374	9.0 %	291.41	0.212	0.018	35	0.1869	0	1.493	3	0.000643	0.92	0.00018	9.63	0.00027	0.17	0.000607	9.65	0.012077	0.09	0	0	0.43	0	0	0	0	0
17D20376	10.3 %	291.41	0.212	0.018	35	0.1869	0	1.493	3	0.000643	0.92	0.00018	9.63	0.00027	0.17	0.000607	9.65	0.012077	0.09	0	0	0.43	0	0	0	0	0
17D20377	11.6 %	291.41	0.212	0.018	35	0.1869	0	1.493	3	0.000643	0.92	0.00018	9.63	0.00027	0.17	0.000607	9.65	0.012077	0.09	0	0	0.43	0	0	0	0	0
17D20379	12.5 %	291.41	0.212	0.018	35	0.1869	0	1.493	3	0.000643	0.92	0.00018	9.63	0.00027	0.17	0.000607	9.65	0.012077	0.09	0	0	0.43	0	0	0	0	0
17D20380	13.4 %	291.41	0.212	0.018	35	0.1869	0	1.493	3	0.000643	0.92	0.00018	9.63	0.00027	0.17	0.000607	9.65	0.012077	0.09	0	0	0.43	0	0	0	0	0
17D20382	14.6 %	291.41	0.212	0.018	35	0.1869	0	1.493	3	0.000643	0.92	0.00018	9.63	0.00027	0.17	0.000607	9.65	0.012077	0.09	0	0	0.43	0	0	0	0	0
17D20383	16.0 %	291.41	0.212	0.018	35	0.1869	0	1.493	3	0.000643	0.92	0.00018	9.63	0.00027	0.17	0.000607	9.65	0.012077	0.09	0	0	0.43	0	0	0	0	0
17D20385	17.6 %	291.41	0.212	0.018	35	0.1869	0	1.493	3	0.000643	0.92	0.00018	9.63	0.00027	0.17	0.000607	9.65	0.012077	0.09	0	0	0.43	0	0	0	0	0
17D20386	19.3 %	291.41	0.212	0.018	35	0.1869	0	1.493	3	0.000643	0.92	0.00018	9.63	0.00027	0.17	0.000607	9.65	0.012077	0.09	0	0	0.43	0	0	0	0	0
17D20388	21.0 %	291.41	0.212	0.018	35	0.1869	0	1.493	3	0.000643	0.92	0.00018	9.63	0.00027	0.17	0.000607	9.65	0.012077	0.09	0	0	0.43	0	0	0	0	0

17D20349.AGE >>> PS59-305-1 >>> ARCTIC | O-CONNOR (16-22) PROJECT

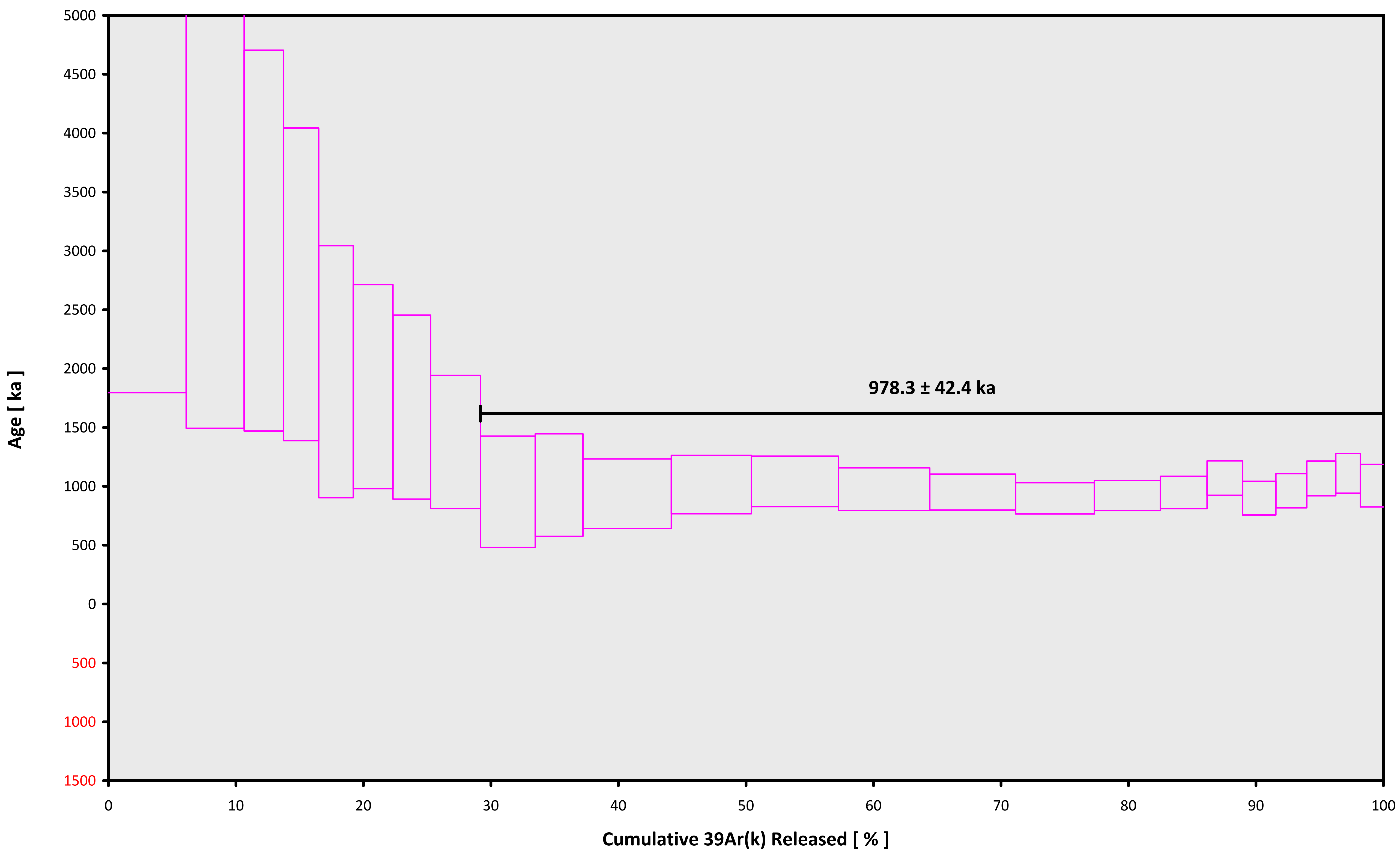

Ar-Ages in ka

WEIGHTED PLATEAU
978.3 ± 42.4
TOTAL FUSION
1515.4 ± 215.0
NORMAL ISOCHRON
975.1 ± 76.9
INVERSE ISOCHRON
976.9 ± 76.2

MSWD (PROBABILITY)
0.66 (82%)

Sample Info

Groundmass
Gakkel Ridge
Dan Miggins

IRR = 17-OSU-01 (1C35-17)
J = 0.00155705 ± 0.00000131

17D20349.AGE >>> PS59-305-1 >>> ARCTIC | O-CONNOR (16-22) PROJECT

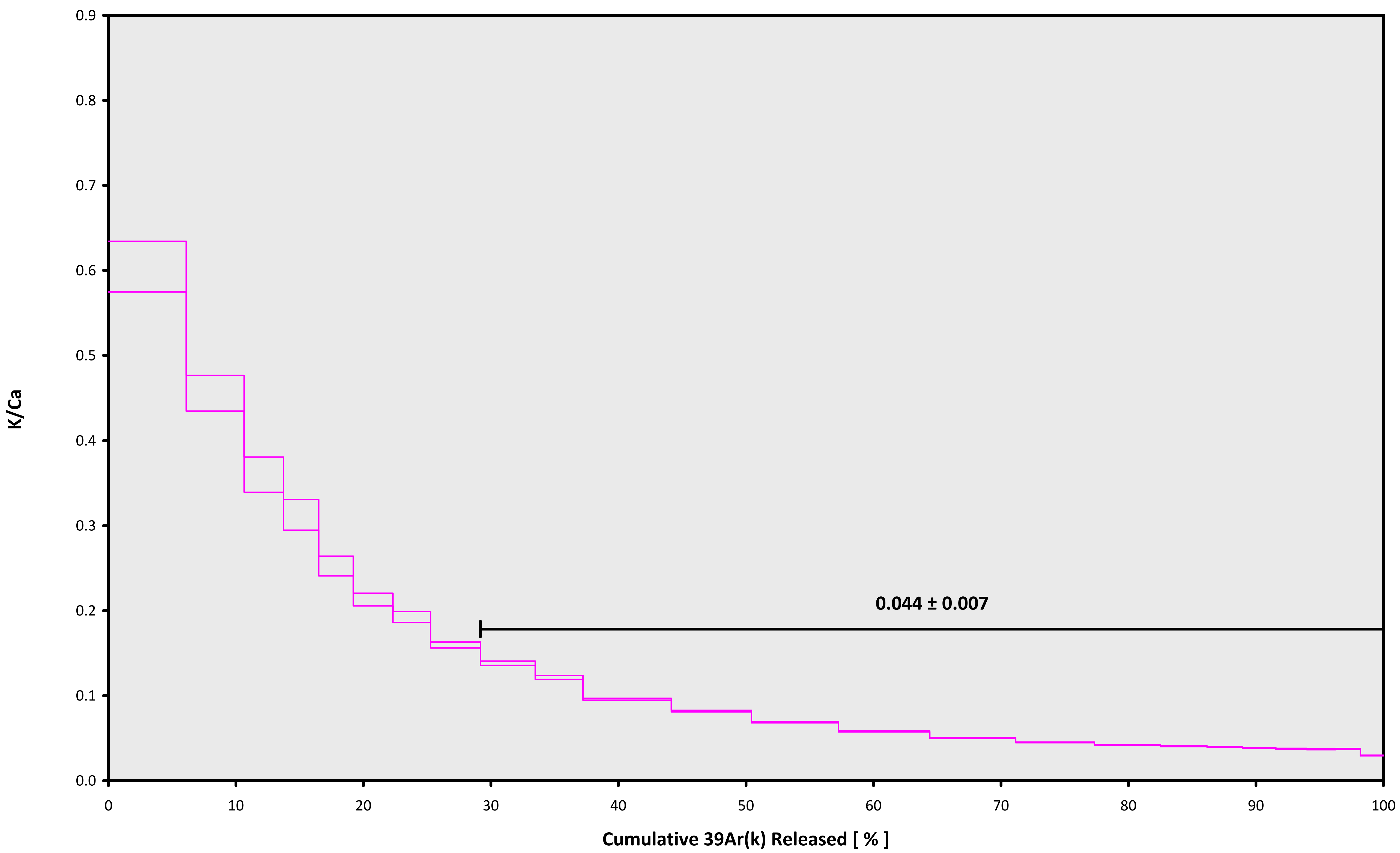

Ar-Ages in ka

WEIGHTED PLATEAU
978.3 ± 42.4
TOTAL FUSION
1515.4 ± 215.0
NORMAL ISOCHRON
975.1 ± 76.9
INVERSE ISOCHRON
976.9 ± 76.2

Sample Info

Groundmass
Gakkel Ridge
Dan Miggins

IRR = 17-OSU-01 (1C35-17)
J = 0.00155705 ± 0.00000131

17D20349.AGE >>> PS59-305-1 >>> ARCTIC | O-CONNOR (16-22) PROJECT

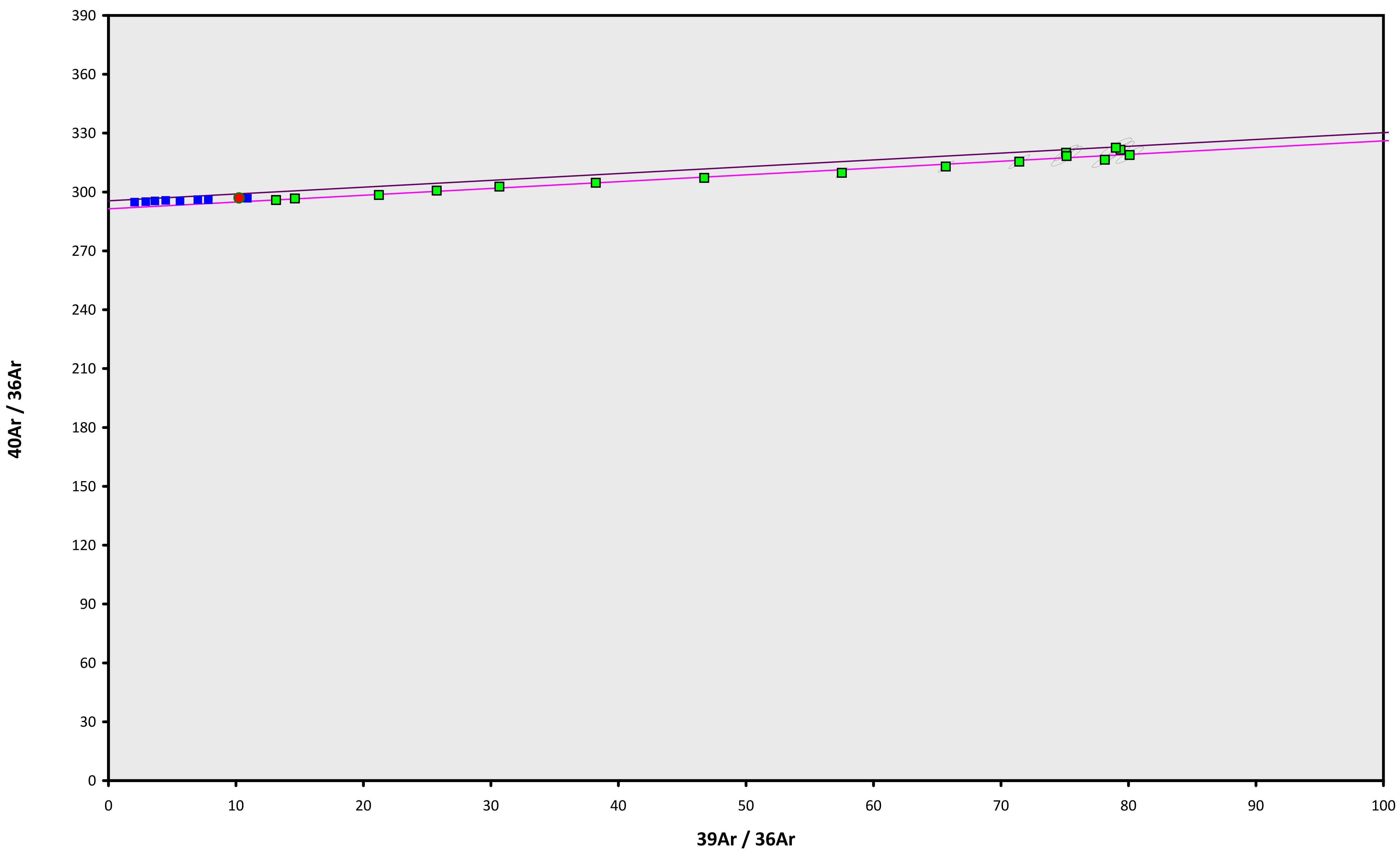

Ar-Ages in ka

WEIGHTED PLATEAU
978.3 ± 42.4
TOTAL FUSION
1515.4 ± 215.0
NORMAL ISOCHRON
975.1 ± 76.9
INVERSE ISOCHRON
976.9 ± 76.2

MSWD (PROBABILITY)
0.81 (66%)

40AR/36AR INTERCEPT
291.4 ± 1.2

Sample Info

Groundmass
Gakkel Ridge
Dan Miggins

IRR = 17-OSU-01 (1C35-17)
J = 0.00155705 ± 0.00000131

17D20349.AGE >>> PS59-305-1 >>> ARCTIC | O-CONNOR (16-22) PROJECT

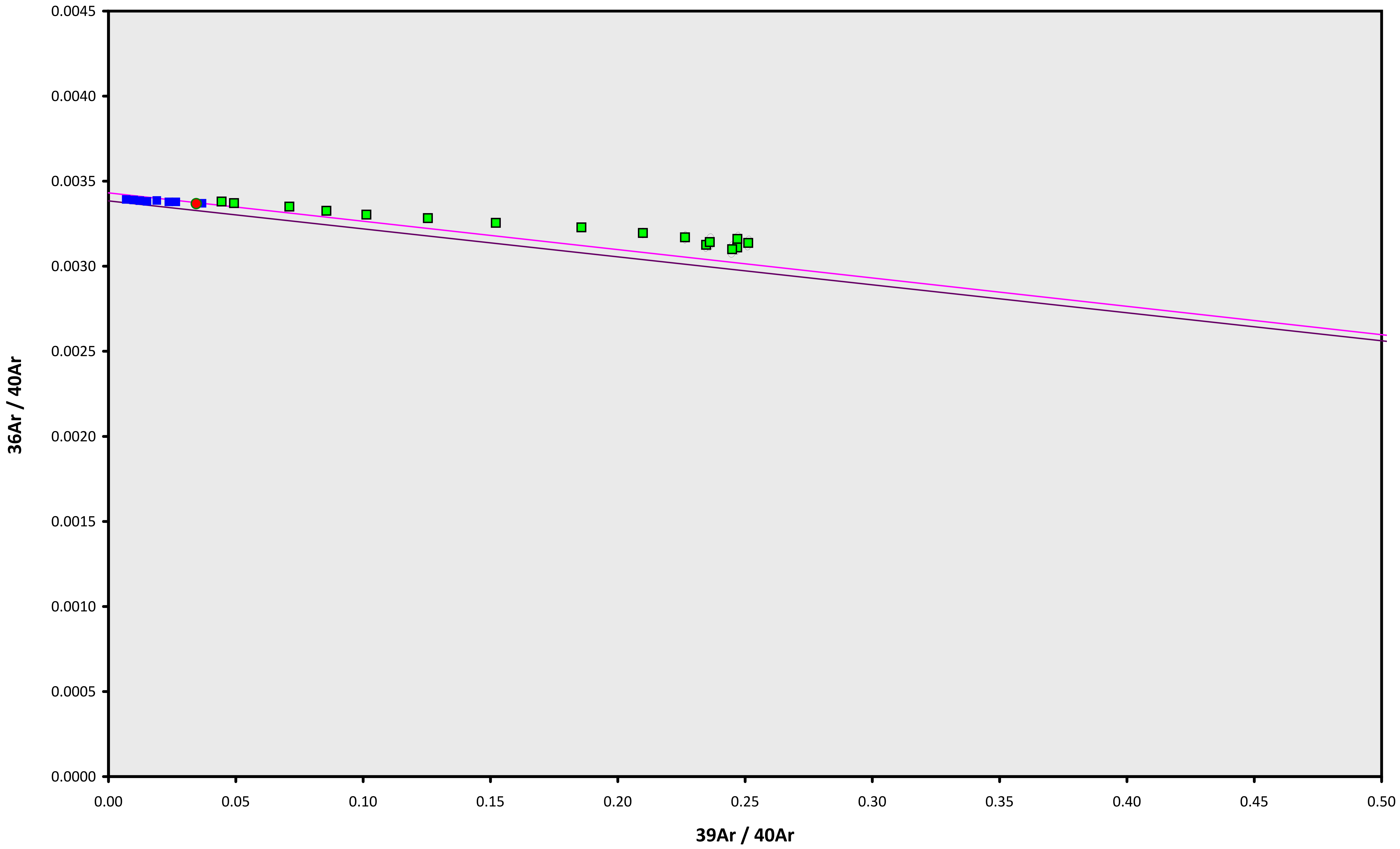

Ar-Ages in ka

WEIGHTED PLATEAU
978.3 ± 42.4
TOTAL FUSION
1515.4 ± 215.0
NORMAL ISOCHRON
975.1 ± 76.9
INVERSE ISOCHRON
976.9 ± 76.2

MSWD (PROBABILITY)
0.82 (65%)

SPREADING FACTOR
7.2%

40AR/36AR INTERCEPT
291.4 ± 1.2

Sample Info

Groundmass
Gakkel Ridge
Dan Miggins

IRR = 17-OSU-01 (1C35-17)
J = 0.00155705 ± 0.00000131

Relative Abundances			36Ar [fA]	%1σ	37Ar [fA]	%1σ	38Ar [fA]	%1σ	39Ar [fA]	%1σ	40Ar [fA]	%1σ	40(r)/39(k) ± 2σ	Age ± 2σ (ka)	40Ar(r) (%)	39Ar(k) (%)	K/Ca ± 2σ
17D19832	1.8 %	✓	1.5742202	0.302	48.4898	0.996	1.4621838	1.680	84.92136	0.074	483.3614	0.043	0.25928 ± 0.03344	757.8 ± 97.7	4.55	15.28	0.753 ± 0.015
17D19834	1.9 %	✓	0.8407941	0.330	36.5783	1.239	0.9208896	2.619	57.15248	0.080	261.0249	0.083	0.27065 ± 0.02974	791.0 ± 86.9	5.92	10.29	0.672 ± 0.017
17D19835	2.0 %	✓	0.5633249	0.353	29.6292	1.555	0.6570196	3.650	41.05179	0.090	175.8257	0.118	0.28534 ± 0.03043	834.0 ± 88.9	6.66	7.39	0.595 ± 0.019
17D19837	2.2 %	✓	0.5389248	0.368	36.5507	1.261	0.6410916	3.660	39.84008	0.091	166.7411	0.124	0.26089 ± 0.03129	762.5 ± 91.4	6.23	7.17	0.468 ± 0.012
17D19838	2.4 %	✓	0.3948614	0.406	28.3237	1.618	0.4709635	5.099	28.34252	0.109	120.8837	0.168	0.22775 ± 0.03650	665.7 ± 106.6	5.34	5.10	0.430 ± 0.014
17D19840	2.7 %	✓	0.4949852	0.375	49.7760	0.980	0.5418945	4.485	33.49453	0.102	151.0641	0.135	0.26161 ± 0.03509	764.6 ± 102.5	5.79	6.02	0.289 ± 0.006
17D19841	3.0 %	✓	0.3539488	0.430	38.1347	1.230	0.3673107	6.520	23.80492	0.118	107.3806	0.190	0.24478 ± 0.04166	715.4 ± 121.7	5.42	4.28	0.268 ± 0.007
17D19843	3.4 %	✓	0.4489851	0.381	73.4620	0.740	0.4609132	5.371	27.70695	0.109	134.1004	0.153	0.26314 ± 0.03959	769.1 ± 115.7	5.43	4.98	0.162 ± 0.002
17D19844	3.9 %	✓	0.4268182	0.412	100.7957	0.633	0.4619489	5.395	26.63680	0.111	125.4726	0.163	0.27796 ± 0.04219	812.4 ± 123.3	5.89	4.78	0.113 ± 0.001
17D19846	4.5 %	✓	0.3860348	0.396	124.8023	0.585	0.4109118	5.794	24.10968	0.118	110.4949	0.184	0.26541 ± 0.04154	775.7 ± 121.4	5.77	4.33	0.083 ± 0.001
17D19847	5.2 %	✓	0.3889303	0.392	185.4653	0.505	0.4151091	5.638	24.13842	0.115	105.4542	0.193	0.22176 ± 0.04174	648.2 ± 122.0	5.05	4.32	0.056 ± 0.001
17D19849	6.0 %	✓	0.3594580	0.429	231.3596	0.491	0.3681488	6.696	21.57769	0.127	94.0371	0.216	0.29328 ± 0.04739	857.2 ± 138.5	6.68	3.86	0.040 ± 0.000
17D19850	6.9 %	✓	0.3701799	0.407	292.7130	0.477	0.3277825	7.780	20.23914	0.130	90.5413	0.225	0.22547 ± 0.05026	659.0 ± 146.9	4.99	3.61	0.029 ± 0.000
17D19852	7.9 %	✓	0.3631519	0.405	375.7587	0.467	0.3326824	7.354	20.00797	0.139	82.6697	0.246	0.27114 ± 0.05086	792.5 ± 148.6	6.48	3.56	0.023 ± 0.000
17D19853	9.0 %	✓	0.3601348	0.421	426.7315	0.463	0.3235605	7.362	18.70382	0.137	77.0956	0.264	0.25769 ± 0.05640	753.2 ± 164.8	6.16	3.32	0.019 ± 0.000
17D19855	10.3 %	✓	0.3456997	0.442	406.2923	0.464	0.2644466	9.260	16.13669	0.150	73.4573	0.277	0.23593 ± 0.06553	689.6 ± 191.5	5.10	2.86	0.017 ± 0.000
17D19856	11.6 %	✓	0.2782653	0.468	313.3750	0.474	0.2222838	10.799	12.09031	0.200	60.5084	0.336	0.27792 ± 0.07629	812.3 ± 222.9	5.46	2.14	0.016 ± 0.000
17D19858	12.5 %	✓	0.2067307	0.552	204.9704	0.501	0.1301694	18.963	7.78430	0.283	46.0813	0.441	0.17765 ± 0.10529	519.3 ± 307.7	2.95	1.38	0.016 ± 0.000
17D19859	13.4 %	✓	0.1826712	0.570	145.7918	0.542	0.1038477	23.402	5.60649	0.393	43.3189	0.469	0.17798 ± 0.13590	520.2 ± 397.2	2.26	0.99	0.016 ± 0.000
17D19861	14.6 %	✓	0.1802150	0.603	139.9635	0.538	0.1010461	24.746	5.35702	0.418	43.5389	0.466	0.27749 ± 0.14625	811.0 ± 427.4	3.36	0.95	0.016 ± 0.000
17D19862	16.0 %		0.1873710	0.581	126.0298	0.572	0.1126679	20.607	5.10403	0.435	45.6796	0.444	0.07464 ± 0.15335	218.2 ± 448.3	0.82	0.90	0.017 ± 0.000
17D19864	17.6 %		0.2228625	0.499	129.4106	0.574	0.1200924	19.731	5.53142	0.398	56.2818	0.361	0.13933 ± 0.14362	407.3 ± 419.8	1.35	0.98	0.018 ± 0.000
17D19865	19.3 %		0.2052839	0.539	115.0060	0.580	0.1391256	17.404	4.76704	0.475	51.6897	0.393	0.04547 ± 0.16568	132.9 ± 484.3	0.41	0.84	0.018 ± 0.000
17D19867	21.0 %		0.1794270	0.569	109.4787	0.598	0.1245607	18.445	3.73176	0.612	44.8765	0.452	0.16396 ± 0.20089	479.2 ± 587.1	1.34	0.66	0.014 ± 0.000
		Σ	9.8532787	0.090	3768.8885	0.129	9.4806507	1.250	557.83719	0.027	2751.5798	0.036					

Information on Analysis and Constants Used in Calculations

Project = **O-CONNOR (16-22)**
Sample = **PS59-305-20**
Material = **Groundmass**
Location = **Gakkel Ridge**
Region = **Artic Ocean**
Analyst = **Dan Miggins**
Irradiation = **17-OSU-01 (1F26-17)**
Position = **X: 0 | Y: 0 | Z/H: 34.82958 mm**
FCT-NM Age = **28.201 ± 0.023 Ma**
FCT-NM Reference = **Kuiper et al (2008)**
FCT-NM 40Ar/39Ar Ratio = **9.72237 ± 0.00914**
FCT-NM J-value = **0.00161662 ± 0.00000152**
Air Shot 40Ar/36Ar = **302.5200 ± 0.4205**
Air Shot MDF = **0.99419123 ± 0.00067181 (LIN)**
Experiment Type = **Incremental Heating**
Extraction Method = **Bulk Laser Heating**
Heating = **64 sec**
Isolation = **3.00 min**
Instrument = **ARGUS-VI-D**
Preferred Age = **Plateau Age**
Age Classification = **Crystallization Age**
IGSN = Undefined
Rock Class = **Igneous>Volcanic**
Lithology = **Basaltic Lava**
Lat-Lon = Undefined - Undefined

Age Equations = **Min et al. (2000)**
Negative Intensities = **Allowed**
Collector Calibrations = **36Ar**
Decay 40K = **5.530 ± 0.048 E-10 1/a**
Decay 39Ar = **2.940 ± 0.016 E-07 1/h**
Decay 37Ar = **8.230 ± 0.012 E-04 1/h**
Decay 36Cl = **2.257 ± 0.015 E-06 1/a**
Decay 40K(EC,β⁺) = **0.580 ± 0.009 E-10 1/a**
Decay 40K(β⁻) = **4.950 ± 0.043 E-10 1/a**
Atmospheric 40/36(a) = **295.50**
Atmospheric 38/36(a) = **0.1869**
Production 39/37(ca) = **0.0006425 ± 0.0000059**
Production 38/37(ca) = **0.0001800 ± 0.0000173**
Production 36/37(ca) = **0.0002703 ± 0.0000005**
Production 40/39(k) = **0.000607 ± 0.000059**
Production 38/39(k) = **0.012077 ± 0.000011**
Production 36/38(cl) = **262.80 ± 1.71**
Scaling Ratio K/Ca = **0.430**
Abundance Ratio 40K/K = **1.1700 ± 0.0100 E-04**
Atomic Weight K = **39.0983 ± 0.0001 g**

Results

Results	40(a)/36(a) ± 2σ	40(r)/39(k) ± 2σ	Age ± 2σ (ka)	MSWD	39Ar(k) (%,n)	K/Ca ± 2σ
**Age Plateau**		0.25930 ± 0.00987 ± 3.80%	**757.9 ± 28.9 ± 3.81%**	1.05	96.61 40% 20	0.020 ± 0.006
			Full External Error ± 33.6	1.65	2σ Confidence Limit	
			Analytical Error ± 28.8	1.0232	Error Magnification	
**Total Fusion Age**		0.25328 ± 0.01021 ± 4.03%	**740.3 ± 29.9 ± 4.03%**		24	0.063 ± 0.000
			Full External Error ± 34.2			
			Analytical Error ± 29.8			
**Normal Isochron**	**293.59 ± 5.46 ± 1.86%**	0.28707 ± 0.08095 ± 28.20%	**839.0 ± 236.6 ± 28.19%**	1.08	96.61 36% 20	
			Full External Error ± 237.3	1.67	2σ Confidence Limit	
			Analytical Error ± 236.5	1.0404	Error Magnification	
				4	Number of Iterations	
				0.0000015407	Convergence	
**Inverse Isochron** Clustered Points	**293.61 ± 5.45 ± 1.86%**	0.28715 ± 0.06836 ± 23.80%	**839.3 ± 199.7 ± 23.80%**	1.07	96.61 37% 20	
			Full External Error ± 200.6	1.67	2σ Confidence Limit	
			Analytical Error ± 199.7	1.0367	Error Magnification	
				4	Number of Iterations	
				0.0001551287	Convergence	
				3%	Spreading Factor	

Notes
Mostly atmospheric

Incremental Heating			36Ar(a) [fA]	37Ar(ca) [fA]	38Ar(cl) [fA]	39Ar(k) [fA]	40Ar(r) [fA]	Age ± 2σ (ka)	40Ar(r) (%)	39Ar(k) (%)	K/Ca ± 2σ
17D19832	1.8 %	✓	1.5610819	48.4898	0.1364705	84.89020	22.01015	757.8 ± 97.7	4.55	15.28	0.753 ± 0.015
17D19834	1.9 %	✓	0.8308910	36.5783	0.0690653	57.12898	15.46196	791.0 ± 86.9	5.92	10.29	0.672 ± 0.017
17D19835	2.0 %	✓	0.5553041	29.6292	0.0523475	41.03275	11.70843	834.0 ± 88.9	6.66	7.39	0.595 ± 0.019
17D19837	2.2 %	✓	0.5290325	36.5507	0.0547712	39.81660	10.38780	762.5 ± 91.4	6.23	7.17	0.468 ± 0.012
17D19838	2.4 %	✓	0.3871936	28.3237	0.0514259	28.32432	6.45083	665.7 ± 106.6	5.34	5.10	0.430 ± 0.014
17D19840	2.7 %	✓	0.4815218	49.7760	0.0388112	33.46255	8.75407	764.6 ± 102.5	5.79	6.02	0.289 ± 0.006
17D19841	3.0 %	✓	0.3436389	38.1347	0.0090243	23.78042	5.82086	715.4 ± 121.7	5.42	4.28	0.268 ± 0.007
17D19843	3.4 %	✓	0.4291206	73.4620	0.0334406	27.65975	7.27852	769.1 ± 115.7	5.43	4.98	0.162 ± 0.002
17D19844	3.9 %	✓	0.3995620	100.7957	0.0482171	26.57203	7.38592	812.4 ± 123.3	5.89	4.78	0.113 ± 0.001
17D19846	4.5 %	✓	0.3522933	124.8023	0.0323996	24.02949	6.37765	775.7 ± 121.4	5.77	4.33	0.083 ± 0.001
17D19847	5.2 %	✓	0.3387925	185.4653	0.0283244	24.01926	5.32644	648.2 ± 122.0	5.05	4.32	0.056 ± 0.001
17D19849	6.0 %	✓	0.2969186	231.3596	0.0122115	21.42904	6.28463	857.2 ± 138.5	6.68	3.86	0.040 ± 0.000
17D19850	6.9 %	✓	0.2910596	292.7130	0.0000000	20.05108	4.52098	659.0 ± 146.9	4.99	3.61	0.029 ± 0.000
17D19852	7.9 %	✓	0.2615843	375.7587	0.0000000	19.76655	5.35957	792.5 ± 148.6	6.48	3.56	0.023 ± 0.000
17D19853	9.0 %	✓	0.2447892	426.7315	0.0000000	18.42965	4.74921	753.2 ± 164.8	6.16	3.32	0.019 ± 0.000
17D19855	10.3 %	✓	0.2358789	406.2923	0.0000000	15.87565	3.74549	689.6 ± 191.5	5.10	2.86	0.017 ± 0.000
17D19856	11.6 %	✓	0.1935601	313.3750	0.0000000	11.88896	3.30415	812.3 ± 222.9	5.46	2.14	0.016 ± 0.000
17D19858	12.5 %	✓	0.1513272	204.9704	0.0000000	7.65261	1.35949	519.3 ± 307.7	2.95	1.38	0.016 ± 0.000
17D19859	13.4 %	✓	0.1432637	145.7918	0.0000000	5.51281	0.98115	520.2 ± 397.2	2.26	0.99	0.016 ± 0.000
17D19861	14.6 %	✓	0.1423828	139.9635	0.0000000	5.26709	1.46158	811.0 ± 427.4	3.36	0.95	0.016 ± 0.000
17D19862	16.0 %		0.1533050	126.0298	0.0006664	5.02306	0.37493	218.2 ± 448.3	0.82	0.90	0.017 ± 0.000
17D19864	17.6 %		0.1878828	129.4106	0.0000000	5.44827	0.75909	407.3 ± 419.8	1.35	0.98	0.018 ± 0.000
17D19865	19.3 %		0.1741910	115.0060	0.0291891	4.69315	0.21340	132.9 ± 484.3	0.41	0.84	0.018 ± 0.000
17D19867	21.0 %		0.1498274	109.4787	0.0326329	3.66142	0.60032	479.2 ± 587.1	1.34	0.66	0.014 ± 0.000
		Σ	8.8344030	3768.8885	0.6289973	555.41568	140.67661				

Information on Analysis

Project = **O-CONNOR (16-22)**
Sample = **PS59-305-20**
Material = **Groundmass**
Location = **Gakkel Ridge**
Region = **Artic Ocean**
Analyst = **Dan Miggins**
Irradiation = **17-OSU-01 (1F26-17)**
J = **0.00161662 ± 0.00000152**
FCT-NM = **28.201 ± 0.023 Ma**

Results	40(r)/39(k) ± 2σ		Age ± 2σ (ka)		MSWD	39Ar(k) (%,n)	K/Ca ± 2σ
**Age Plateau**	0.25930	± 0.00987<br>± 3.80%	**757.9**	**± 28.9**<br>**± 3.81%**	1.05	96.61<br>40% 20	0.020 ± 0.006
		Full External Error		± 33.6	1.65	2σ Confidence Limit	
		Analytical Error		± 28.8	1.0232	Error Magnification	
**Total Fusion Age**	0.25328	± 0.01021<br>± 4.03%	**740.3**	**± 29.9**<br>**± 4.03%**		24	0.063 ± 0.000
		Full External Error		± 34.2			
		Analytical Error		± 29.8			

Normal Isochron			39(k)/36(a) ± 2σ	40(a+r)/36(a) ± 2σ	r.i.
17D19832	1.8 %	✓	54.38 ± 0.34	309.60 ± 1.90	0.9624
17D19834	1.9 %	✓	68.76 ± 0.47	314.11 ± 2.17	0.9442
17D19835	2.0 %	✓	73.89 ± 0.55	316.58 ± 2.39	0.9218
17D19837	2.2 %	✓	75.26 ± 0.58	315.14 ± 2.49	0.9232
17D19838	2.4 %	✓	73.15 ± 0.63	312.16 ± 2.80	0.8963
17D19840	2.7 %	✓	69.49 ± 0.56	313.68 ± 2.57	0.9128
17D19841	3.0 %	✓	69.20 ± 0.64	312.44 ± 3.02	0.8886
17D19843	3.4 %	✓	64.46 ± 0.53	312.46 ± 2.68	0.9008
17D19844	3.9 %	✓	66.50 ± 0.61	313.99 ± 2.96	0.9095
17D19846	4.5 %	✓	68.21 ± 0.62	313.60 ± 2.98	0.8892
17D19847	5.2 %	✓	70.90 ± 0.67	311.22 ± 3.09	0.8929
17D19849	6.0 %	✓	72.17 ± 0.79	316.67 ± 3.63	0.8999
17D19850	6.9 %	✓	68.89 ± 0.76	311.03 ± 3.62	0.8956
17D19852	7.9 %	✓	75.56 ± 0.92	315.99 ± 4.07	0.8992
17D19853	9.0 %	✓	75.29 ± 1.02	314.90 ± 4.48	0.9084
17D19855	10.3 %	✓	67.30 ± 0.95	311.38 ± 4.61	0.9051
17D19856	11.6 %	✓	61.42 ± 0.91	312.57 ± 4.90	0.8681
17D19858	12.5 %	✓	50.57 ± 0.84	304.48 ± 5.45	0.8156
17D19859	13.4 %	✓	38.48 ± 0.65	302.35 ± 5.32	0.7449
17D19861	14.6 %	✓	36.99 ± 0.66	305.77 ± 5.55	0.7525
17D19862	16.0 %		32.77 ± 0.56	297.95 ± 5.05	0.7265
17D19864	17.6 %		29.00 ± 0.42	299.54 ± 4.21	0.7121
17D19865	19.3 %		26.94 ± 0.43	296.73 ± 4.48	0.6834
17D19867	21.0 %		24.44 ± 0.46	299.51 ± 4.96	0.6217

Results	40(a)/36(a) ± 2σ	40(r)/39(k) ± 2σ	Age ± 2σ (ka)	MSWD
Normal Isochron	**293.59** **± 5.46** **± 1.86%**	0.28707 ± 0.08095 ± 28.20%	**839.0** **± 236.6** **± 28.19%**	1.08 36%
			Full External Error ± 237.3	
			Analytical Error ± 236.5	

Statistics				
	2σ Confidence Limit	1.67	Convergence	0.000001540731
	Error Magnification	1.0404	Number of Iterations	4
	Number of Data Points	20	Calculated Line	Weighted York-2

Inverse Isochron			39(k)/40(a+r) ± 2σ	36(a)/40(a+r) ± 2σ	r.i.
17D19832	1.8 %	✓	0.1756434 ± 0.0002997	0.00322998 ± 0.00001985	0.0691
17D19834	1.9 %	✓	0.2188932 ± 0.0005036	0.00318361 ± 0.00002195	0.1732
17D19835	2.0 %	✓	0.2334048 ± 0.0006909	0.00315871 ± 0.00002386	0.2473
17D19837	2.2 %	✓	0.2388276 ± 0.0007338	0.00317324 ± 0.00002512	0.2513
17D19838	2.4 %	✓	0.2343438 ± 0.0009401	0.00320348 ± 0.00002871	0.3154
17D19840	2.7 %	✓	0.2215421 ± 0.0007503	0.00318796 ± 0.00002613	0.2622
17D19841	3.0 %	✓	0.2214890 ± 0.0009916	0.00320063 ± 0.00003095	0.3330
17D19843	3.4 %	✓	0.2062873 ± 0.0007770	0.00320039 ± 0.00002743	0.2907
17D19844	3.9 %	✓	0.2118028 ± 0.0008378	0.00318487 ± 0.00003001	0.2859
17D19846	4.5 %	✓	0.2175002 ± 0.0009548	0.00318874 ± 0.00003028	0.3266
17D19847	5.2 %	✓	0.2278011 ± 0.0010270	0.00321314 ± 0.00003191	0.3343
17D19849	6.0 %	✓	0.2279101 ± 0.0011466	0.00315790 ± 0.00003617	0.3247
17D19850	6.9 %	✓	0.2214877 ± 0.0011535	0.00321509 ± 0.00003737	0.3338
17D19852	7.9 %	✓	0.2391373 ± 0.0013555	0.00316467 ± 0.00004073	0.3312
17D19853	9.0 %	✓	0.2390839 ± 0.0014297	0.00317560 ± 0.00004521	0.3275
17D19855	10.3 %	✓	0.2161490 ± 0.0013690	0.00321152 ± 0.00004756	0.3270
17D19856	11.6 %	✓	0.1965081 ± 0.0015463	0.00319928 ± 0.00005018	0.3663
17D19858	12.5 %	✓	0.1660842 ± 0.0017509	0.00328425 ± 0.00005874	0.4125
17D19859	13.4 %	✓	0.1272710 ± 0.0015687	0.00330744 ± 0.00005814	0.4059
17D19861	14.6 %	✓	0.1209832 ± 0.0015272	0.00327048 ± 0.00005932	0.3802
17D19862	16.0 %		0.1099702 ± 0.0013790	0.00335632 ± 0.00005694	0.3710
17D19864	17.6 %		0.0968092 ± 0.0010496	0.00333845 ± 0.00004688	0.3420
17D19865	19.3 %		0.0907997 ± 0.0011298	0.00337012 ± 0.00005085	0.3286
17D19867	21.0 %		0.0815927 ± 0.0012585	0.00333882 ± 0.00005524	0.3209

Results	40(a)/36(a) ± 2σ	40(r)/39(k) ± 2σ	Age ± 2σ (ka)	MSWD
**Inverse Isochron** Clustered Points	**293.61** **± 5.45** **± 1.86%**	0.28715 ± 0.06836 ± 23.80%	**839.3** **± 199.7** **± 23.80%**	1.07 37%
			Full External Error ± 200.6	
			Analytical Error ± 199.7	

Statistics				
2σ Confidence Limit	1.67	Convergence	0.0001551287
Error Magnification	1.0367	Number of Iterations	4
Number of Data Points	20	Calculated Line	Weighted York-2
Spreading Factor	3.4%		

Degassing Patterns			36Ar(a) [fA]	%1σ	36Ar(c) [fA]	%1σ	36Ar(ca) [fA]	%1σ	36Ar(cl) [fA]	%1σ	37Ar(ca) [fA]	%1σ	38Ar(a) [fA]	%1σ	38Ar(c) [fA]	%1σ	38Ar(k) [fA]	%1σ	38Ar(ca) [fA]	%1σ	38Ar(cl) [fA]	%1σ	39Ar(k) [fA]	%1σ	39Ar(ca) [fA]	%1σ	40Ar(r) [fA]	%1σ	40Ar(a) [fA]	%1σ	40Ar(c) [fA]	%1σ	40Ar(k) [fA]	%1σ
17D19832	1.8 %	✓	1.5610819	0.30	0.0000000	0.00	0.0131068	1.01	0.0000315	18.07	48.4898	1.00	0.2917662	0.30	0.0000000	0.00	1.0252190	0.12	0.0087282	9.68	0.1364705	18.09	84.89020	0.07	0.0311547	1.36	22.01015	6.45	461.2997	0.30	0.0000000	0.00	0.0515284	9.65
17D19834	1.9 %	✓	0.8308910	0.33	0.0000000	0.00	0.0098871	1.25	0.0000159	34.98	36.5783	1.24	0.1552935	0.33	0.0000000	0.00	0.6899467	0.12	0.0065841	9.71	0.0690653	34.99	57.12898	0.08	0.0235016	1.54	15.46196	5.49	245.5283	0.33	0.0000000	0.00	0.0346773	9.65
17D19835	2.0 %	✓	0.5553041	0.36	0.0000000	0.00	0.0080088	1.56	0.0000121	45.86	29.6292	1.55	0.1037863	0.36	0.0000000	0.00	0.4955525	0.13	0.0053333	9.75	0.0523475	45.86	41.03275	0.09	0.0190367	1.81	11.70843	5.33	164.0924	0.36	0.0000000	0.00	0.0249069	9.65
17D19837	2.2 %	✓	0.5290325	0.38	0.0000000	0.00	0.0098796	1.27	0.0000126	42.88	36.5507	1.26	0.0988762	0.38	0.0000000	0.00	0.4808650	0.13	0.0065791	9.71	0.0547712	42.89	39.81660	0.09	0.0234838	1.56	10.38780	6.00	156.3291	0.38	0.0000000	0.00	0.0241687	9.65
17D19838	2.4 %	✓	0.3871936	0.42	0.0000000	0.00	0.0076559	1.63	0.0000119	46.73	28.3237	1.62	0.0723665	0.42	0.0000000	0.00	0.3420729	0.14	0.0050983	9.76	0.0514259	46.74	28.32432	0.11	0.0181980	1.86	6.45083	8.01	114.4157	0.42	0.0000000	0.00	0.0171929	9.65
17D19840	2.7 %	✓	0.4815218	0.39	0.0000000	0.00	0.0134544	0.99	0.0000090	62.69	49.7760	0.98	0.0899964	0.39	0.0000000	0.00	0.4041272	0.14	0.0089597	9.68	0.0388112	62.69	33.46255	0.10	0.0319811	1.34	8.75407	6.71	142.2897	0.39	0.0000000	0.00	0.0203118	9.65
17D19841	3.0 %	✓	0.3436389	0.44	0.0000000	0.00	0.0103078	1.24	0.0000021	265.54	38.1347	1.23	0.0642261	0.44	0.0000000	0.00	0.2871961	0.15	0.0068643	9.71	0.0090243	265.54	23.78042	0.12	0.0245016	1.54	5.82086	8.51	101.5453	0.44	0.0000000	0.00	0.0144347	9.65
17D19843	3.4 %	✓	0.4291206	0.40	0.0000000	0.00	0.0198568	0.76	0.0000077	74.16	73.4620	0.74	0.0802026	0.40	0.0000000	0.00	0.3340468	0.14	0.0132232	9.66	0.0334406	74.16	27.65975	0.11	0.0471993	1.18	7.27852	7.52	126.8051	0.40	0.0000000	0.00	0.0167895	9.65
17D19844	3.9 %	✓	0.3995620	0.44	0.0000000	0.00	0.0272451	0.66	0.0000111	51.83	100.7957	0.63	0.0746781	0.44	0.0000000	0.00	0.3209105	0.14	0.0181432	9.65	0.0482171	51.84	26.57203	0.11	0.0647612	1.12	7.38592	7.59	118.0706	0.44	0.0000000	0.00	0.0161292	9.65
17D19846	4.5 %	✓	0.3522933	0.44	0.0000000	0.00	0.0337340	0.61	0.0000075	73.81	124.8023	0.58	0.0658436	0.44	0.0000000	0.00	0.2902041	0.15	0.0224644	9.65	0.0323996	73.81	24.02949	0.12	0.0801854	1.09	6.37765	7.82	104.1027	0.44	0.0000000	0.00	0.0145859	9.65
17D19847	5.2 %	✓	0.3387925	0.46	0.0000000	0.00	0.0501313	0.53	0.0000065	83.43	185.4653	0.51	0.0633203	0.46	0.0000000	0.00	0.2900806	0.15	0.0333838	9.64	0.0283244	83.44	24.01926	0.12	0.1191614	1.05	5.32644	9.41	100.1132	0.46	0.0000000	0.00	0.0145797	9.65
17D19849	6.0 %	✓	0.2969186	0.53	0.0000000	0.00	0.0625365	0.52	0.0000028	204.58	231.3596	0.49	0.0554941	0.53	0.0000000	0.00	0.2587985	0.16	0.0416447	9.64	0.0122115	204.58	21.42904	0.13	0.1486486	1.04	6.28463	8.08	87.7395	0.53	0.0000000	0.00	0.0130074	9.65
17D19850	6.9 %	✓	0.2910596	0.54	0.0000000	0.00	0.0791203	0.51	0.0000000	0.00	292.7130	0.48	0.0543990	0.54	0.0000000	0.00	0.2421568	0.16	0.0526883	9.64	0.0000000	0.00	20.05108	0.13	0.1880681	1.04	4.52098	11.15	86.0081	0.54	0.0000000	0.00	0.0121710	9.65
17D19852	7.9 %	✓	0.2615843	0.59	0.0000000	0.00	0.1015676	0.50	0.0000000	0.00	375.7587	0.47	0.0488901	0.59	0.0000000	0.00	0.2387206	0.17	0.0676366	9.64	0.0000000	0.00	19.76655	0.14	0.2414249	1.03	5.35957	9.38	77.2982	0.59	0.0000000	0.00	0.0119983	9.65
17D19853	9.0 %	✓	0.2447892	0.66	0.0000000	0.00	0.1153455	0.49	0.0000000	0.00	426.7315	0.46	0.0457511	0.66	0.0000000	0.00	0.2225748	0.17	0.0768117	9.64	0.0000000	0.00	18.42965	0.14	0.2741750	1.03	4.74921	10.94	72.3352	0.66	0.0000000	0.00	0.0111868	9.65
17D19855	10.3 %	✓	0.2358789	0.69	0.0000000	0.00	0.1098208	0.49	0.0000000	0.00	406.2923	0.46	0.0440858	0.69	0.0000000	0.00	0.1917302	0.18	0.0731326	9.64	0.0000000	0.00	15.87565	0.15	0.2610428	1.03	3.74549	13.89	69.7022	0.69	0.0000000	0.00	0.0096365	9.65
17D19856	11.6 %	✓	0.1935601	0.71	0.0000000	0.00	0.0847053	0.50	0.0000000	0.00	313.3750	0.47	0.0361764	0.71	0.0000000	0.00	0.1435830	0.22	0.0564075	9.64	0.0000000	0.00	11.88896	0.20	0.2013434	1.03	3.30415	13.72	57.1970	0.71	0.0000000	0.00	0.0072166	9.65
17D19858	12.5 %	✓	0.1513272	0.78	0.0000000	0.00	0.0554035	0.53	0.0000000	0.00	204.9704	0.50	0.0282831	0.78	0.0000000	0.00	0.0924206	0.30	0.0368947	9.64	0.0000000	0.00	7.65261	0.29	0.1316935	1.05	1.35949	29.63	44.7172	0.78	0.0000000	0.00	0.0046451	9.65
17D19859	13.4 %	✓	0.1432637	0.74	0.0000000	0.00	0.0394075	0.57	0.0000000	0.00	145.7918	0.54	0.0267760	0.74	0.0000000	0.00	0.0665783	0.41	0.0262425	9.65	0.0000000	0.00	5.51281	0.40	0.0936712	1.07	0.98115	38.18	42.3344	0.74	0.0000000	0.00	0.0033463	9.66
17D19861	14.6 %	✓	0.1423828	0.78	0.0000000	0.00	0.0378321	0.56	0.0000000	0.00	139.9635	0.54	0.0266113	0.78	0.0000000	0.00	0.0636106	0.43	0.0251934	9.64	0.0000000	0.00	5.26709	0.43	0.0899266	1.07	1.46158	26.35	42.0741	0.78	0.0000000	0.00	0.0031971	9.66
17D19862	16.0 %		0.1533050	0.72	0.0000000	0.00	0.0340659	0.60	0.0000002	######	126.0298	0.57	0.0286527	0.72	0.0000000	0.00	0.0606635	0.45	0.0226854	9.65	0.0006664	######	5.02306	0.44	0.0809741	1.08	0.37493	102.73	45.3016	0.72	0.0000000	0.00	0.0030490	9.66
17D19864	17.6 %		0.1878828	0.60	0.0000000	0.00	0.0349797	0.60	0.0000000	0.00	129.4106	0.57	0.0351153	0.60	0.0000000	0.00	0.0657988	0.41	0.0232939	9.65	0.0000000	0.00	5.44827	0.40	0.0831463	1.08	0.75909	51.54	55.5194	0.60	0.0000000	0.00	0.0033071	9.66
17D19865	19.3 %		0.1741910	0.64	0.0000000	0.00	0.0310861	0.60	0.0000067	83.25	115.0060	0.58	0.0325563	0.64	0.0000000	0.00	0.0566791	0.49	0.0207011	9.65	0.0291891	83.26	4.69315	0.48	0.0738914	1.09	0.21340	182.18	51.4734	0.64	0.0000000	0.00	0.0028487	9.66
17D19867	21.0 %		0.1498274	0.69	0.0000000	0.00	0.0295921	0.62	0.0000075	70.66	109.4787	0.60	0.0280027	0.69	0.0000000	0.00	0.0442189	0.63	0.0197062	9.65	0.0326329	70.67	3.66142	0.62	0.0703400	1.10	0.60032	61.26	44.2740	0.69	0.0000000	0.00	0.0022225	9.67
		Σ	8.8344030	0.10	0.0000000	0.00	1.0187306	0.14	0.0001452	14.85	3768.8885	0.13	1.6511499	0.10	0.0000000	0.00	6.7077552	0.04	0.6783999	2.48	0.6289973	14.85	555.41568	0.03	2.4215109	0.27	140.67661	2.01	2610.5661	0.10	0.0000000	0.00	0.3371373	2.52
		Σ							9.8532787	0.09	3768.8885	0.13									9.6663023	0.98			557.83719	0.03							2751.5798	0.14

Additional Parameters			40Ar/39Ar	1σ	37Ar/39Ar	1σ	36Ar/39Ar	1σ	Time (days)	37Ar (decay)	39Ar (decay)	40Ar (moles)
17D19832	1.8 %	✓	5.691871	0.004854	0.570996	0.005700	0.018537	0.000058	142.113	16.601413	1.00100414	2.320E-11
17D19834	1.9 %	✓	4.567167	0.005252	0.640013	0.007949	0.014711	0.000050	142.127	16.605968	1.00100423	1.253E-11
17D19835	2.0 %	✓	4.283021	0.006337	0.721751	0.011240	0.013722	0.000050	142.134	16.608246	1.00100428	8.440E-12
17D19837	2.2 %	✓	4.185260	0.006427	0.917435	0.011595	0.013527	0.000051	142.148	16.612803	1.00100438	8.004E-12
17D19838	2.4 %	✓	4.265102	0.008552	0.999337	0.016206	0.013932	0.000059	142.155	16.615081	1.00100443	5.802E-12
17D19840	2.7 %	✓	4.510112	0.007634	1.486093	0.014642	0.014778	0.000057	142.169	16.619640	1.00100453	7.251E-12
17D19841	3.0 %	✓	4.510857	0.010093	1.601968	0.019795	0.014869	0.000066	142.176	16.621920	1.00100458	5.154E-12
17D19843	3.4 %	✓	4.839957	0.009109	2.651393	0.019845	0.016205	0.000064	142.190	16.626480	1.00100468	6.437E-12
17D19844	3.9 %	✓	4.710500	0.009307	3.784076	0.024319	0.016024	0.000068	142.197	16.628761	1.00100473	6.023E-12
17D19846	4.5 %	✓	4.583011	0.010048	5.176439	0.030883	0.016012	0.000066	142.211	16.633552	1.00100483	5.304E-12
17D19847	5.2 %	✓	4.368728	0.009831	7.683406	0.039803	0.016113	0.000066	142.218	16.635834	1.00100488	5.062E-12
17D19849	6.0 %	✓	4.358071	0.010937	10.722169	0.054368	0.016659	0.000074	142.232	16.640398	1.00100498	4.514E-12
17D19850	6.9 %	✓	4.473572	0.011612	14.462717	0.071436	0.018290	0.000078	142.239	16.642681	1.00100502	4.346E-12
17D19852	7.9 %	✓	4.131840	0.011663	18.780448	0.091470	0.018150	0.000078	142.253	16.647247	1.00100512	3.968E-12
17D19853	9.0 %	✓	4.121918	0.012267	22.815205	0.110254	0.019255	0.000085	142.260	16.649530	1.00100517	3.701E-12
17D19855	10.3 %	✓	4.552194	0.014339	25.178166	0.122821	0.021423	0.000100	142.274	16.654099	1.00100527	3.526E-12
17D19856	11.6 %	✓	5.004700	0.019582	25.919522	0.133323	0.023016	0.000117	142.281	16.656383	1.00100532	2.904E-12
17D19858	12.5 %	✓	5.919777	0.031027	26.331250	0.151539	0.026557	0.000165	142.294	16.660953	1.00100542	2.212E-12
17D19859	13.4 %	✓	7.726572	0.047261	26.004128	0.174076	0.032582	0.000226	142.301	16.663239	1.00100547	2.079E-12
17D19861	14.6 %	✓	8.127453	0.050884	26.127144	0.177864	0.033641	0.000247	142.315	16.667811	1.00100556	2.090E-12
17D19862	16.0 %		8.949705	0.055648	24.692193	0.177391	0.036710	0.000267	142.322	16.670097	1.00100561	2.193E-12
17D19864	17.6 %		10.174925	0.054671	23.395559	0.163384	0.040290	0.000257	142.336	16.674671	1.00100571	2.702E-12
17D19865	19.3 %		10.843144	0.066806	24.125249	0.180876	0.043063	0.000309	142.343	16.676958	1.00100576	2.481E-12
17D19867	21.0 %		12.025582	0.091561	29.337036	0.251026	0.048081	0.000402	142.357	16.681534	1.00100586	2.154E-12

Procedure Blanks		36Ar ± 1σ (SE) [fA]	37Ar ± 1σ (SE) [fA]	38Ar ± 1σ (SE) [fA]	39Ar ± 1σ (SE) [fA]	40Ar ± 1σ (SE) [fA]
17D19832	1.8 %	0.0089771 ± 0.0005919	0.1481484 ± 0.0184023	0.0390829 ± 0.0172778	0.0092043 ± 0.0155248	2.5133646 ± 0.2020485
17D19834	1.9 %	0.0096180 ± 0.0005919	0.1438613 ± 0.0184023	0.0246968 ± 0.0172778	0.0049927 ± 0.0155248	2.7569863 ± 0.2020485
17D19835	2.0 %	0.0098231 ± 0.0005919	0.1419036 ± 0.0184023	0.0207981 ± 0.0172778	0.0087128 ± 0.0155248	2.8400876 ± 0.2020485
17D19837	2.2 %	0.0100489 ± 0.0005919	0.1386945 ± 0.0184023	0.0180491 ± 0.0172778	0.0108359 ± 0.0155248	2.9429055 ± 0.2020485
17D19838	2.4 %	0.0100863 ± 0.0005919	0.1375477 ± 0.0184023	0.0186404 ± 0.0172778	0.0097674 ± 0.0155248	2.9677977 ± 0.2020485
17D19840	2.7 %	0.0100487 ± 0.0005919	0.1363596 ± 0.0184023	0.0225014 ± 0.0172778	0.0045719 ± 0.0155248	2.9763908 ± 0.2020485
17D19841	3.0 %	0.0099874 ± 0.0005919	0.1363650 ± 0.0184023	0.0253277 ± 0.0172778	0.0008778 ± 0.0155248	2.9643903 ± 0.2020485
17D19843	3.4 %	0.0098103 ± 0.0005919	0.1376200 ± 0.0184023	0.0318061 ± 0.0172778	0.0077397 ± 0.0155248	2.9174459 ± 0.2020485
17D19844	3.9 %	0.0097050 ± 0.0005919	0.1388585 ± 0.0184023	0.0351295 ± 0.0172778	0.0123256 ± 0.0155248	2.8859237 ± 0.2020485
17D19846	4.5 %	0.0094730 ± 0.0005919	0.1426735 ± 0.0184023	0.0415437 ± 0.0172778	0.0217573 ± 0.0155248	2.8104923 ± 0.2020485
17D19847	5.2 %	0.0093651 ± 0.0005919	0.1449944 ± 0.0184023	0.0441046 ± 0.0172778	0.0258991 ± 0.0155248	2.7728551 ± 0.2020485
17D19849	6.0 %	0.0091694 ± 0.0005919	0.1503589 ± 0.0184023	0.0478800 ± 0.0172778	0.0330165 ± 0.0155248	2.6995722 ± 0.2020485
17D19850	6.9 %	0.0090858 ± 0.0005919	0.1532740 ± 0.0184023	0.0489995 ± 0.0172778	0.0358483 ± 0.0155248	2.6655653 ± 0.2020485
17D19852	7.9 %	0.0089541 ± 0.0005919	0.1591754 ± 0.0184023	0.0496080 ± 0.0172778	0.0398184 ± 0.0155248	2.6058218 ± 0.2020485
17D19853	9.0 %	0.0089071 ± 0.0005919	0.1619753 ± 0.0184023	0.0491169 ± 0.0172778	0.0409084 ± 0.0155248	2.5808471 ± 0.2020485
17D19855	10.3 %	0.0088498 ± 0.0005919	0.1667347 ± 0.0184023	0.0467374 ± 0.0172778	0.0412967 ± 0.0155248	2.5415008 ± 0.2020485
17D19856	11.6 %	0.0088376 ± 0.0005919	0.1684497 ± 0.0184023	0.0449839 ± 0.0172778	0.0406421 ± 0.0155248	2.5270144 ± 0.2020485
17D19858	12.5 %	0.0088371 ± 0.0005919	0.1698671 ± 0.0184023	0.0408297 ± 0.0172778	0.0378724 ± 0.0155248	2.5070316 ± 0.2020485
17D19859	13.4 %	0.0088439 ± 0.0005919	0.1692671 ± 0.0184023	0.0386789 ± 0.0172778	0.0358997 ± 0.0155248	2.5005436 ± 0.2020485
17D19861	14.6 %	0.0088545 ± 0.0005919	0.1646215 ± 0.0184023	0.0349977 ± 0.0172778	0.0312542 ± 0.0155248	2.4909991 ± 0.2020485
17D19862	16.0 %	0.0088503 ± 0.0005919	0.1602158 ± 0.0184023	0.0338320 ± 0.0172778	0.0288192 ± 0.0155248	2.4860741 ± 0.2020485
17D19864	17.6 %	0.0087983 ± 0.0005919	0.1462655 ± 0.0184023	0.0339057 ± 0.0172778	0.0244385 ± 0.0155248	2.4701510 ± 0.2020485
17D19865	19.3 %	0.0087392 ± 0.0005919	0.1363029 ± 0.0184023	0.0356246 ± 0.0172778	0.0228260 ± 0.0155248	2.4564075 ± 0.2020485
17D19867	21.0 %	0.0085225 ± 0.0005919	0.1092852 ± 0.0184023	0.0437694 ± 0.0172778	0.0217090 ± 0.0155248	2.4093973 ± 0.2020485

Intercept Values		36Ar ± 1σ (SE) [fA]	r2	Regression (type,n)	37Ar ± 1σ (SE) [fA]	r2	Regression (type,n)	38Ar ± 1σ (SE) [fA]	r2	Regression (type,n)	39Ar ± 1σ (SE) [fA]	r2	Regression (type,n)	40Ar ± 1σ (SE) [fA]	r2	Regression (type,n)
17D19832	1.8 %	1.5240367 ± 0.0017944	0.9698	EXP 150 of 150	2.7218482 ± 0.0176124	0.4439	EXP 150 of 150	1.4061169 ± 0.0169402	0.1451	EXP 150 of 150	84.3351218 ± 0.0202583	0.9987	EXP 150 of 150	485.874740 ± 0.038111	0.9978	EXP 150 of 150
17D19834	1.9 %	0.8188143 ± 0.0013600	0.9274	EXP 150 of 150	2.0205315 ± 0.0169032	0.4075	EXP 150 of 150	0.8854962 ± 0.0163778	0.0397	EXP 150 of 150	56.7691276 ± 0.0185159	0.9974	EXP 150 of 150	263.781918 ± 0.076992	0.1975	EXP 150 of 150
17D19835	2.0 %	0.5519779 ± 0.0010467	0.8841	EXP 150 of 150	1.6110569 ± 0.0184813	0.1975	EXP 150 of 150	0.6285899 ± 0.0162029	0.0252	EXP 150 of 150	40.7815542 ± 0.0185775	0.9949	EXP 150 of 150	178.665781 ± 0.043529	0.9839	EXP 150 of 150
17D19837	2.2 %	0.5287205 ± 0.0011268	0.8583	EXP 150 of 150	2.0231717 ± 0.0175760	0.3356	EXP 150 of 150	0.6155958 ± 0.0154418	0.0326	EXP 150 of 150	39.5801973 ± 0.0186298	0.9947	EXP 150 of 150	169.683997 ± 0.040628	0.9794	EXP 150 of 150
17D19838	2.4 %	0.3901085 ± 0.0009690	0.8122	EXP 150 of 150	1.5374897 ± 0.0184058	0.2074	EXP 150 of 150	0.4468526 ± 0.0162628	0.0506	EXP 150 of 150	28.1596966 ± 0.0184092	0.9894	EXP 150 of 150	123.851531 ± 0.024149	0.9969	EXP 150 of 150
17D19840	2.7 %	0.4864320 ± 0.0010652	0.8245	EXP 149 of 150	2.8065339 ± 0.0177996	0.4616	EXP 150 of 150	0.5130987 ± 0.0166703	0.0454	EXP 150 of 150	33.2714965 ± 0.0203268	0.9913	EXP 150 of 150	154.040467 ± 0.026202	0.9884	EXP 150 of 150
17D19841	3.0 %	0.3506345 ± 0.0009594	0.7583	EXP 150 of 150	2.1179563 ± 0.0180758	0.3004	EXP 150 of 150	0.3377166 ± 0.0161694	0.0021	EXP 150 of 150	23.6440354 ± 0.0170091	0.9865	EXP 150 of 150	110.344966 ± 0.026792	0.9959	EXP 150 of 150
17D19843	3.4 %	0.4419221 ± 0.0009750	0.8668	EXP 150 of 150	4.2038709 ± 0.0176365	0.6632	EXP 150 of 150	0.4237534 ± 0.0173140	0.0337	EXP 150 of 150	27.5109259 ± 0.0179101	0.9894	EXP 150 of 150	137.017875 ± 0.036748	0.9826	EXP 150 of 150
17D19844	3.9 %	0.4204831 ± 0.0011108	0.7800	EXP 149 of 150	5.8171921 ± 0.0189849	0.7616	EXP 150 of 150	0.4214536 ± 0.0175435	0.0470	EXP 150 of 150	26.4434613 ± 0.0175875	0.9887	EXP 150 of 150	128.358555 ± 0.033099	0.9901	EXP 150 of 150
17D19846	4.5 %	0.3810003 ± 0.0008771	0.8370	EXP 150 of 150	7.2298108 ± 0.0203276	0.8156	EXP 150 of 150	0.3645951 ± 0.0159637	0.0083	EXP 150 of 150	23.9240791 ± 0.0174067	0.9866	EXP 150 of 150	113.305395 ± 0.026864	0.9934	EXP 150 of 150
17D19847	5.2 %	0.3836791 ± 0.0008660	0.8277	EXP 150 of 150	10.8095543 ± 0.0167262	0.9385	EXP 150 of 150	0.3661827 ± 0.0153691	0.0412	EXP 150 of 150	23.9484864 ± 0.0160594	0.9887	EXP 149 of 150	108.227061 ± 0.027962	0.9942	EXP 150 of 150
17D19849	6.0 %	0.3551186 ± 0.0009717	0.7396	EXP 150 of 150	13.5112008 ± 0.0188901	0.9473	EXP 150 of 150	0.3159925 ± 0.0171715	0.0043	EXP 150 of 150	21.3980368 ± 0.0171739	0.9839	EXP 150 of 150	96.736668 ± 0.022995	0.9957	EXP 150 of 150
17D19850	6.9 %	0.3653540 ± 0.0008928	0.7941	EXP 150 of 150	17.1287714 ± 0.0190184	0.9646	EXP 150 of 150	0.2749757 ± 0.0183451	0.0007	EXP 150 of 150	20.0657547 ± 0.0160359	0.9836	EXP 150 of 150	93.206823 ± 0.023996	0.9958	EXP 150 of 150
17D19852	7.9 %	0.3584584 ± 0.0008562	0.8090	EXP 150 of 150	22.0198777 ± 0.0190324	0.9788	EXP 150 of 150	0.2792101 ± 0.0169125	0.0001	EXP 150 of 150	19.8321816 ± 0.0184722	0.9785	EXP 150 of 150	85.275566 ± 0.021082	0.9965	EXP 150 of 150
17D19853	9.0 %	0.3555077 ± 0.0009315	0.7797	EXP 150 of 150	25.0222797 ± 0.0188525	0.9838	EXP 150 of 150	0.2706852 ± 0.0159891	0.0051	EXP 150 of 150	18.5358026 ± 0.0159392	0.9808	EXP 150 of 150	79.676461 ± 0.024271	0.9960	EXP 150 of 150
17D19855	10.3 %	0.3415578 ± 0.0009858	0.7607	EXP 150 of 150	23.8046910 ± 0.0182177	0.9827	EXP 150 of 150	0.2146375 ± 0.0169455	0.0019	EXP 150 of 150	15.9857299 ± 0.0148909	0.9772	EXP 150 of 150	75.998844 ± 0.023276	0.9957	EXP 150 of 150
17D19856	11.6 %	0.2766455 ± 0.0008259	0.6802	EXP 150 of 150	18.3182777 ± 0.0192436	0.9671	EXP 150 of 150	0.1747180 ± 0.0162586	0.0011	EXP 150 of 150	11.9674989 ± 0.0164793	0.9488	EXP 150 of 150	63.035374 ± 0.023361	0.9966	EXP 150 of 150
17D19858	12.5 %	0.2077988 ± 0.0007449	0.5297	EXP 150 of 150	11.9185040 ± 0.0187161	0.9297	EXP 150 of 150	0.0878277 ± 0.0172247	0.0050	EXP 150 of 150	7.6935276 ± 0.0145522	0.8961	EXP 149 of 150	48.588368 ± 0.021386	0.9972	EXP 150 of 150
17D19859	13.4 %	0.1846501 ± 0.0006486	0.5337	EXP 150 of 150	8.4277930 ± 0.0180716	0.8715	EXP 150 of 150	0.0639626 ± 0.0166861	0.0019	EXP 150 of 150	5.5324827 ± 0.0149464	0.7939	EXP 150 of 150	45.819453 ± 0.020600	0.9974	EXP 150 of 150
17D19861	14.6 %	0.1822969 ± 0.0007184	0.4555	EXP 150 of 150	8.0864959 ± 0.0155464	0.8900	EXP 149 of 150	0.0648747 ± 0.0176715	0.0032	EXP 150 of 150	5.2893543 ± 0.0154842	0.7874	EXP 150 of 150	46.029897 ± 0.020607	0.9968	EXP 150 of 150
17D19862	16.0 %	0.1891797 ± 0.0007090	0.5976	EXP 150 of 150	7.2684620 ± 0.0184383	0.8318	EXP 150 of 150	0.0775272 ± 0.0151008	0.0042	EXP 150 of 150	5.0405263 ± 0.0152899	0.7750	EXP 150 of 150	48.165676 ± 0.018644	0.9973	EXP 149 of 150
17D19864	17.6 %	0.2232855 ± 0.0006693	0.6872	EXP 150 of 150	7.4795997 ± 0.0197478	0.8238	EXP 150 of 150	0.0847917 ± 0.0158100	0.0033	EXP 148 of 150	5.4693867 ± 0.0149590	0.8197	EXP 150 of 150	58.751926 ± 0.020166	0.9950	EXP 150 of 150
17D19865	19.3 %	0.2063083 ± 0.0006989	0.6598	EXP 150 of 150	6.6398015 ± 0.0164723	0.8452	EXP 150 of 150	0.1018849 ± 0.0165587	0.0133	EXP 150 of 150	4.7118141 ± 0.0159379	0.7064	EXP 150 of 150	54.146100 ± 0.019517	0.9960	EXP 150 of 150
17D19867	21.0 %	0.1812065 ± 0.0006248	0.5975	EXP 150 of 150	6.3393820 ± 0.0172815	0.8189	EXP 150 of 150	0.0793444 ± 0.0147353	0.0215	EXP 150 of 150	3.6846844 ± 0.0163670	0.6299	EXP 150 of 150	47.285943 ± 0.020089	0.9960	EXP 150 of 150

Project Info		Analyst	Irradiation	X-pos	Y-pos	Z/H-pos	Project	Experiment	Nmb
17D19832	1.8 %	Dan Miggins	17-OSU-01	0.00	0.00	34.83	Arctic\O-Connor (16-22)	17D19828	01
17D19834	1.9 %	Dan Miggins	17-OSU-01	0.00	0.00	34.83	Arctic\O-Connor (16-22)	17D19828	01
17D19835	2.0 %	Dan Miggins	17-OSU-01	0.00	0.00	34.83	Arctic\O-Connor (16-22)	17D19828	01
17D19837	2.2 %	Dan Miggins	17-OSU-01	0.00	0.00	34.83	Arctic\O-Connor (16-22)	17D19828	01
17D19838	2.4 %	Dan Miggins	17-OSU-01	0.00	0.00	34.83	Arctic\O-Connor (16-22)	17D19828	01
17D19840	2.7 %	Dan Miggins	17-OSU-01	0.00	0.00	34.83	Arctic\O-Connor (16-22)	17D19828	01
17D19841	3.0 %	Dan Miggins	17-OSU-01	0.00	0.00	34.83	Arctic\O-Connor (16-22)	17D19828	01
17D19843	3.4 %	Dan Miggins	17-OSU-01	0.00	0.00	34.83	Arctic\O-Connor (16-22)	17D19828	01
17D19844	3.9 %	Dan Miggins	17-OSU-01	0.00	0.00	34.83	Arctic\O-Connor (16-22)	17D19828	01
17D19846	4.5 %	Dan Miggins	17-OSU-01	0.00	0.00	34.83	Arctic\O-Connor (16-22)	17D19828	01
17D19847	5.2 %	Dan Miggins	17-OSU-01	0.00	0.00	34.83	Arctic\O-Connor (16-22)	17D19828	01
17D19849	6.0 %	Dan Miggins	17-OSU-01	0.00	0.00	34.83	Arctic\O-Connor (16-22)	17D19828	01
17D19850	6.9 %	Dan Miggins	17-OSU-01	0.00	0.00	34.83	Arctic\O-Connor (16-22)	17D19828	01
17D19852	7.9 %	Dan Miggins	17-OSU-01	0.00	0.00	34.83	Arctic\O-Connor (16-22)	17D19828	01
17D19853	9.0 %	Dan Miggins	17-OSU-01	0.00	0.00	34.83	Arctic\O-Connor (16-22)	17D19828	01
17D19855	10.3 %	Dan Miggins	17-OSU-01	0.00	0.00	34.83	Arctic\O-Connor (16-22)	17D19828	01
17D19856	11.6 %	Dan Miggins	17-OSU-01	0.00	0.00	34.83	Arctic\O-Connor (16-22)	17D19828	01
17D19858	12.5 %	Dan Miggins	17-OSU-01	0.00	0.00	34.83	Arctic\O-Connor (16-22)	17D19828	01
17D19859	13.4 %	Dan Miggins	17-OSU-01	0.00	0.00	34.83	Arctic\O-Connor (16-22)	17D19828	01
17D19861	14.6 %	Dan Miggins	17-OSU-01	0.00	0.00	34.83	Arctic\O-Connor (16-22)	17D19828	01
17D19862	16.0 %	Dan Miggins	17-OSU-01	0.00	0.00	34.83	Arctic\O-Connor (16-22)	17D19828	01
17D19864	17.6 %	Dan Miggins	17-OSU-01	0.00	0.00	34.83	Arctic\O-Connor (16-22)	17D19828	01
17D19865	19.3 %	Dan Miggins	17-OSU-01	0.00	0.00	34.83	Arctic\O-Connor (16-22)	17D19828	01
17D19867	21.0 %	Dan Miggins	17-OSU-01	0.00	0.00	34.83	Arctic\O-Connor (16-22)	17D19828	01

**Sample Parameters**		Sample	Material	Location	Standard Name	Standard (in Ma)	%1σ	Standard Reference	Standard 40Ar/39Ar	%1σ	J	%1σ	Air 40Ar/36Ar	%1σ	MDF (lin)	%1σ	Volume Ratio	Sensitivity (mol/volt)	Day	Month	Year	Hour	Min	Resist
17D19832	1.8 %	PS59-305-20	Groundmass	Gakkel Ridge	FCT-NM (1F26-17)	28.201	0.082	Kuiper et al (2008)	9.72237	0.094	0.00161662	0.094	302.52	0.139	0.9941912	0.068	1	4.8E-14	10	JUN	2017	18	22	1
17D19834	1.9 %	PS59-305-20	Groundmass	Gakkel Ridge	FCT-NM (1F26-17)	28.201	0.082	Kuiper et al (2008)	9.72237	0.094	0.00161662	0.094	302.52	0.139	0.9941912	0.068	1	4.8E-14	10	JUN	2017	18	42	1
17D19835	2.0 %	PS59-305-20	Groundmass	Gakkel Ridge	FCT-NM (1F26-17)	28.201	0.082	Kuiper et al (2008)	9.72237	0.094	0.00161662	0.094	302.52	0.139	0.9941912	0.068	1	4.8E-14	10	JUN	2017	18	52	1
17D19837	2.2 %	PS59-305-20	Groundmass	Gakkel Ridge	FCT-NM (1F26-17)	28.201	0.082	Kuiper et al (2008)	9.72237	0.094	0.00161662	0.094	302.52	0.139	0.9941912	0.068	1	4.8E-14	10	JUN	2017	19	12	1
17D19838	2.4 %	PS59-305-20	Groundmass	Gakkel Ridge	FCT-NM (1F26-17)	28.201	0.082	Kuiper et al (2008)	9.72237	0.094	0.00161662	0.094	302.52	0.139	0.9941912	0.068	1	4.8E-14	10	JUN	2017	19	22	1
17D19840	2.7 %	PS59-305-20	Groundmass	Gakkel Ridge	FCT-NM (1F26-17)	28.201	0.082	Kuiper et al (2008)	9.72237	0.094	0.00161662	0.094	302.52	0.139	0.9941912	0.068	1	4.8E-14	10	JUN	2017	19	42	1
17D19841	3.0 %	PS59-305-20	Groundmass	Gakkel Ridge	FCT-NM (1F26-17)	28.201	0.082	Kuiper et al (2008)	9.72237	0.094	0.00161662	0.094	302.52	0.139	0.9941912	0.068	1	4.8E-14	10	JUN	2017	19	52	1
17D19843	3.4 %	PS59-305-20	Groundmass	Gakkel Ridge	FCT-NM (1F26-17)	28.201	0.082	Kuiper et al (2008)	9.72237	0.094	0.00161662	0.094	302.52	0.139	0.9941912	0.068	1	4.8E-14	10	JUN	2017	20	12	1
17D19844	3.9 %	PS59-305-20	Groundmass	Gakkel Ridge	FCT-NM (1F26-17)	28.201	0.082	Kuiper et al (2008)	9.72237	0.094	0.00161662	0.094	302.52	0.139	0.9941912	0.068	1	4.8E-14	10	JUN	2017	20	22	1
17D19846	4.5 %	PS59-305-20	Groundmass	Gakkel Ridge	FCT-NM (1F26-17)	28.201	0.082	Kuiper et al (2008)	9.72237	0.094	0.00161662	0.094	302.52	0.139	0.9941912	0.068	1	4.8E-14	10	JUN	2017	20	43	1
17D19847	5.2 %	PS59-305-20	Groundmass	Gakkel Ridge	FCT-NM (1F26-17)	28.201	0.082	Kuiper et al (2008)	9.72237	0.094	0.00161662	0.094	302.52	0.139	0.9941912	0.068	1	4.8E-14	10	JUN	2017	20	53	1
17D19849	6.0 %	PS59-305-20	Groundmass	Gakkel Ridge	FCT-NM (1F26-17)	28.201	0.082	Kuiper et al (2008)	9.72237	0.094	0.00161662	0.094	302.52	0.139	0.9941912	0.068	1	4.8E-14	10	JUN	2017	21	13	1
17D19850	6.9 %	PS59-305-20	Groundmass	Gakkel Ridge	FCT-NM (1F26-17)	28.201	0.082	Kuiper et al (2008)	9.72237	0.094	0.00161662	0.094	302.52	0.139	0.9941912	0.068	1	4.8E-14	10	JUN	2017	21	23	1
17D19852	7.9 %	PS59-305-20	Groundmass	Gakkel Ridge	FCT-NM (1F26-17)	28.201	0.082	Kuiper et al (2008)	9.72237	0.094	0.00161662	0.094	302.52	0.139	0.9941912	0.068	1	4.8E-14	10	JUN	2017	21	43	1
17D19853	9.0 %	PS59-305-20	Groundmass	Gakkel Ridge	FCT-NM (1F26-17)	28.201	0.082	Kuiper et al (2008)	9.72237	0.094	0.00161662	0.094	302.52	0.139	0.9941912	0.068	1	4.8E-14	10	JUN	2017	21	53	1
17D19855	10.3 %	PS59-305-20	Groundmass	Gakkel Ridge	FCT-NM (1F26-17)	28.201	0.082	Kuiper et al (2008)	9.72237	0.094	0.00161662	0.094	302.52	0.139	0.9941912	0.068	1	4.8E-14	10	JUN	2017	22	13	1
17D19856	11.6 %	PS59-305-20	Groundmass	Gakkel Ridge	FCT-NM (1F26-17)	28.201	0.082	Kuiper et al (2008)	9.72237	0.094	0.00161662	0.094	302.52	0.139	0.9941912	0.068	1	4.8E-14	10	JUN	2017	22	23	1
17D19858	12.5 %	PS59-305-20	Groundmass	Gakkel Ridge	FCT-NM (1F26-17)	28.201	0.082	Kuiper et al (2008)	9.72237	0.094	0.00161662	0.094	302.52	0.139	0.9941912	0.068	1	4.8E-14	10	JUN	2017	22	43	1
17D19859	13.4 %	PS59-305-20	Groundmass	Gakkel Ridge	FCT-NM (1F26-17)	28.201	0.082	Kuiper et al (2008)	9.72237	0.094	0.00161662	0.094	302.52	0.139	0.9941912	0.068	1	4.8E-14	10	JUN	2017	22	53	1
17D19861	14.6 %	PS59-305-20	Groundmass	Gakkel Ridge	FCT-NM (1F26-17)	28.201	0.082	Kuiper et al (2008)	9.72237	0.094	0.00161662	0.094	302.52	0.139	0.9941912	0.068	1	4.8E-14	10	JUN	2017	23	13	1
17D19862	16.0 %	PS59-305-20	Groundmass	Gakkel Ridge	FCT-NM (1F26-17)	28.201	0.082	Kuiper et al (2008)	9.72237	0.094	0.00161662	0.094	302.52	0.139	0.9941912	0.068	1	4.8E-14	10	JUN	2017	23	23	1
17D19864	17.6 %	PS59-305-20	Groundmass	Gakkel Ridge	FCT-NM (1F26-17)	28.201	0.082	Kuiper et al (2008)	9.72237	0.094	0.00161662	0.094	302.52	0.139	0.9941912	0.068	1	4.8E-14	10	JUN	2017	23	43	1
17D19865	19.3 %	PS59-305-20	Groundmass	Gakkel Ridge	FCT-NM (1F26-17)	28.201	0.082	Kuiper et al (2008)	9.72237	0.094	0.00161662	0.094	302.52	0.139	0.9941912	0.068	1	4.8E-14	10	JUN	2017	23	53	1
17D19867	21.0 %	PS59-305-20	Groundmass	Gakkel Ridge	FCT-NM (1F26-17)	28.201	0.082	Kuiper et al (2008)	9.72237	0.094	0.00161662	0.094	302.52	0.139	0.9941912	0.068	1	4.8E-14	11	JUN	2017	0	13	1

**Irradiation Constants**		40/36(a)	%1σ	40/36(c)	%1σ	38/36(a)	%1σ	38/36(c)	%1σ	39/37(ca)	%1σ	38/37(ca)	%1σ	36/37(ca)	%1σ	40/39(k)	%1σ	38/39(k)	%1σ	36/38(cl)	%1σ	K/Ca	%1σ	K/Cl	%1σ	Ca/Cl	%1σ
17D19832	1.8 %	295.5	0	0.018	35	0.1869	0	1.493	3	0.000643	0.92	0.00018	9.63	0.00027	0.17	0.000607	9.65	0.012077	0.09	0	0	0.43	0	0	0	0	0
17D19834	1.9 %	295.5	0	0.018	35	0.1869	0	1.493	3	0.000643	0.92	0.00018	9.63	0.00027	0.17	0.000607	9.65	0.012077	0.09	0	0	0.43	0	0	0	0	0
17D19835	2.0 %	295.5	0	0.018	35	0.1869	0	1.493	3	0.000643	0.92	0.00018	9.63	0.00027	0.17	0.000607	9.65	0.012077	0.09	0	0	0.43	0	0	0	0	0
17D19837	2.2 %	295.5	0	0.018	35	0.1869	0	1.493	3	0.000643	0.92	0.00018	9.63	0.00027	0.17	0.000607	9.65	0.012077	0.09	0	0	0.43	0	0	0	0	0
17D19838	2.4 %	295.5	0	0.018	35	0.1869	0	1.493	3	0.000643	0.92	0.00018	9.63	0.00027	0.17	0.000607	9.65	0.012077	0.09	0	0	0.43	0	0	0	0	0
17D19840	2.7 %	295.5	0	0.018	35	0.1869	0	1.493	3	0.000643	0.92	0.00018	9.63	0.00027	0.17	0.000607	9.65	0.012077	0.09	0	0	0.43	0	0	0	0	0
17D19841	3.0 %	295.5	0	0.018	35	0.1869	0	1.493	3	0.000643	0.92	0.00018	9.63	0.00027	0.17	0.000607	9.65	0.012077	0.09	0	0	0.43	0	0	0	0	0
17D19843	3.4 %	295.5	0	0.018	35	0.1869	0	1.493	3	0.000643	0.92	0.00018	9.63	0.00027	0.17	0.000607	9.65	0.012077	0.09	0	0	0.43	0	0	0	0	0
17D19844	3.9 %	295.5	0	0.018	35	0.1869	0	1.493	3	0.000643	0.92	0.00018	9.63	0.00027	0.17	0.000607	9.65	0.012077	0.09	0	0	0.43	0	0	0	0	0
17D19846	4.5 %	295.5	0	0.018	35	0.1869	0	1.493	3	0.000643	0.92	0.00018	9.63	0.00027	0.17	0.000607	9.65	0.012077	0.09	0	0	0.43	0	0	0	0	0
17D19847	5.2 %	295.5	0	0.018	35	0.1869	0	1.493	3	0.000643	0.92	0.00018	9.63	0.00027	0.17	0.000607	9.65	0.012077	0.09	0	0	0.43	0	0	0	0	0
17D19849	6.0 %	295.5	0	0.018	35	0.1869	0	1.493	3	0.000643	0.92	0.00018	9.63	0.00027	0.17	0.000607	9.65	0.012077	0.09	0	0	0.43	0	0	0	0	0
17D19850	6.9 %	295.5	0	0.018	35	0.1869	0	1.493	3	0.000643	0.92	0.00018	9.63	0.00027	0.17	0.000607	9.65	0.012077	0.09	0	0	0.43	0	0	0	0	0
17D19852	7.9 %	295.5	0	0.018	35	0.1869	0	1.493	3	0.000643	0.92	0.00018	9.63	0.00027	0.17	0.000607	9.65	0.012077	0.09	0	0	0.43	0	0	0	0	0
17D19853	9.0 %	295.5	0	0.018	35	0.1869	0	1.493	3	0.000643	0.92	0.00018	9.63	0.00027	0.17	0.000607	9.65	0.012077	0.09	0	0	0.43	0	0	0	0	0
17D19855	10.3 %	295.5	0	0.018	35	0.1869	0	1.493	3	0.000643	0.92	0.00018	9.63	0.00027	0.17	0.000607	9.65	0.012077	0.09	0	0	0.43	0	0	0	0	0
17D19856	11.6 %	295.5	0	0.018	35	0.1869	0	1.493	3	0.000643	0.92	0.00018	9.63	0.00027	0.17	0.000607	9.65	0.012077	0.09	0	0	0.43	0	0	0	0	0
17D19858	12.5 %	295.5	0	0.018	35	0.1869	0	1.493	3	0.000643	0.92	0.00018	9.63	0.00027	0.17	0.000607	9.65	0.012077	0.09	0	0	0.43	0	0	0	0	0
17D19859	13.4 %	295.5	0	0.018	35	0.1869	0	1.493	3	0.000643	0.92	0.00018	9.63	0.00027	0.17	0.000607	9.65	0.012077	0.09	0	0	0.43	0	0	0	0	0
17D19861	14.6 %	295.5	0	0.018	35	0.1869	0	1.493	3	0.000643	0.92	0.00018	9.63	0.00027	0.17	0.000607	9.65	0.012077	0.09	0	0	0.43	0	0	0	0	0
17D19862	16.0 %	295.5	0	0.018	35	0.1869	0	1.493	3	0.000643	0.92	0.00018	9.63	0.00027	0.17	0.000607	9.65	0.012077	0.09	0	0	0.43	0	0	0	0	0
17D19864	17.6 %	295.5	0	0.018	35	0.1869	0	1.493	3	0.000643	0.92	0.00018	9.63	0.00027	0.17	0.000607	9.65	0.012077	0.09	0	0	0.43	0	0	0	0	0
17D19865	19.3 %	295.5	0	0.018	35	0.1869	0	1.493	3	0.000643	0.92	0.00018	9.63	0.00027	0.17	0.000607	9.65	0.012077	0.09	0	0	0.43	0	0	0	0	0
17D19867	21.0 %	295.5	0	0.018	35	0.1869	0	1.493	3	0.000643	0.92	0.00018	9.63	0.00027	0.17	0.000607	9.65	0.012077	0.09	0	0	0.43	0	0	0	0	0

17D19828.AGE >>> PS59-305-20 >>> ARCTIC | O-CONNOR (16-22) PROJECT

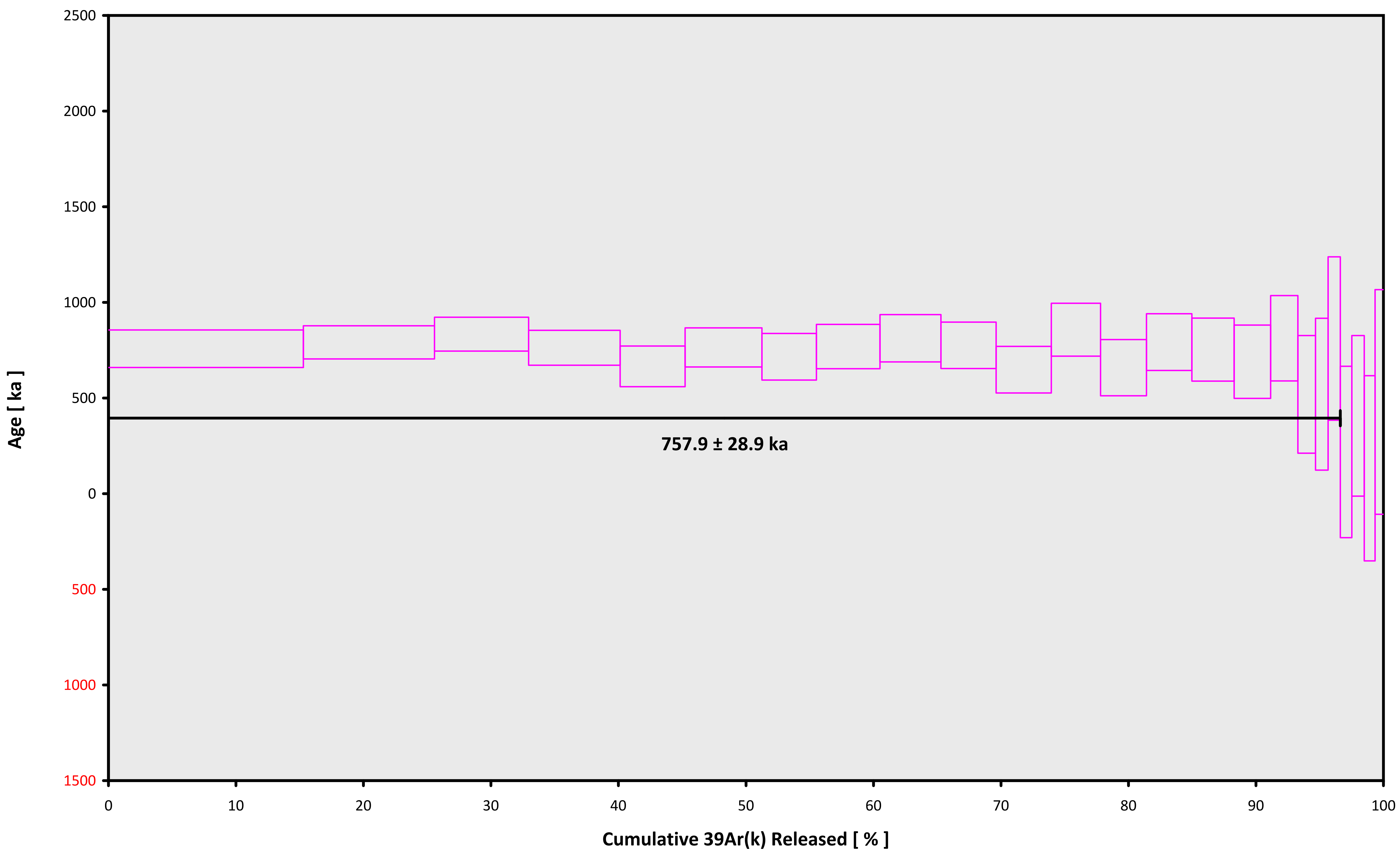

Ar-Ages in ka

WEIGHTED PLATEAU
757.9 ± 28.9
TOTAL FUSION
740.3 ± 29.9
NORMAL ISOCHRON
839.0 ± 236.6
INVERSE ISOCHRON
839.3 ± 199.7

MSWD (PROBABILITY)
1.05 (40%)

Sample Info

Groundmass
Gakkel Ridge
Dan Miggins

IRR = 17-OSU-01 (1F26-17)
J = 0.00161662 ± 0.00000152

17D19828.AGE >>> PS59-305-20 >>> ARCTIC | O-CONNOR (16-22) PROJECT

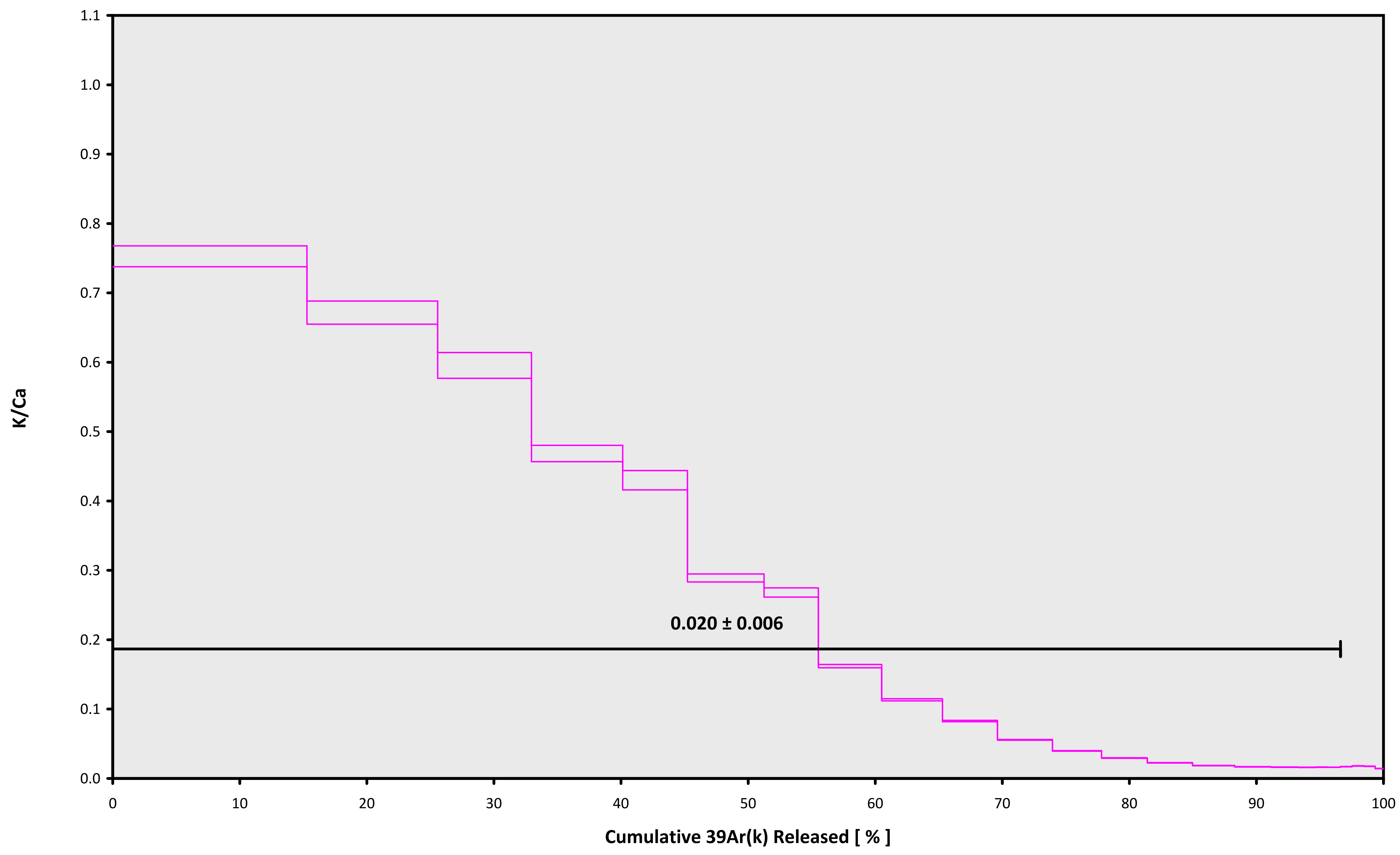

Ar-Ages in ka

WEIGHTED PLATEAU
757.9 ± 28.9
TOTAL FUSION
740.3 ± 29.9
NORMAL ISOCHRON
839.0 ± 236.6
INVERSE ISOCHRON
839.3 ± 199.7

Sample Info

Groundmass
Gakkel Ridge
Dan Miggins

IRR = 17-OSU-01 (1F26-17)
J = 0.00161662 ± 0.00000152

17D19828.AGE >>> PS59-305-20 >>> ARCTIC | O-CONNOR (16-22) PROJECT

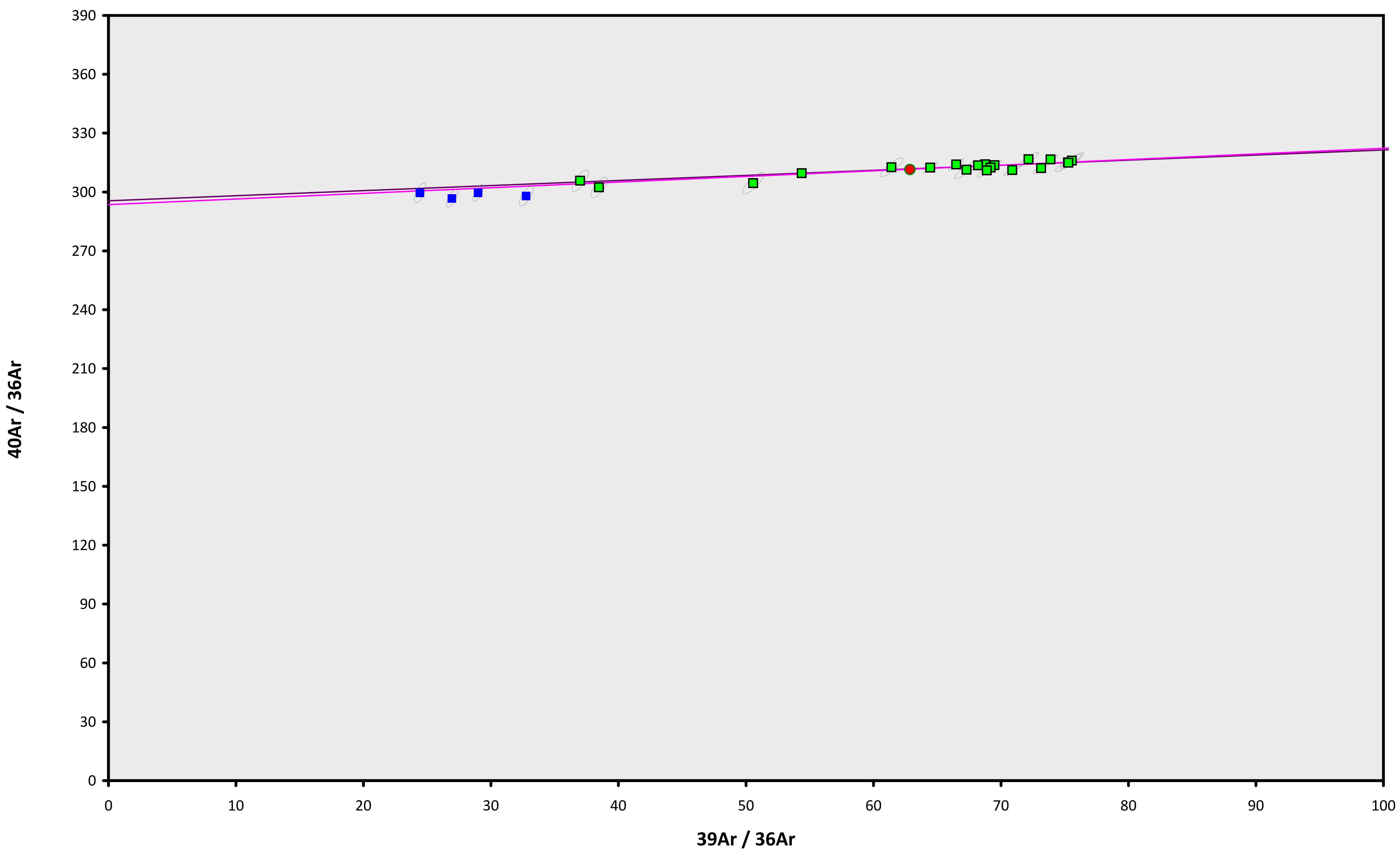

Ar-Ages in ka

WEIGHTED PLATEAU
757.9 ± 28.9
TOTAL FUSION
740.3 ± 29.9
NORMAL ISOCHRON
839.0 ± 236.6
INVERSE ISOCHRON
839.3 ± 199.7

MSWD (PROBABILITY)
1.08 (36%)

40AR/36AR INTERCEPT
293.6 ± 5.5

Sample Info

Groundmass
Gakkel Ridge
Dan Miggins

IRR = 17-OSU-01 (1F26-17)
J = 0.00161662 ± 0.00000152

17D19828.AGE >>> PS59-305-20 >>> ARCTIC | O-CONNOR (16-22) PROJECT

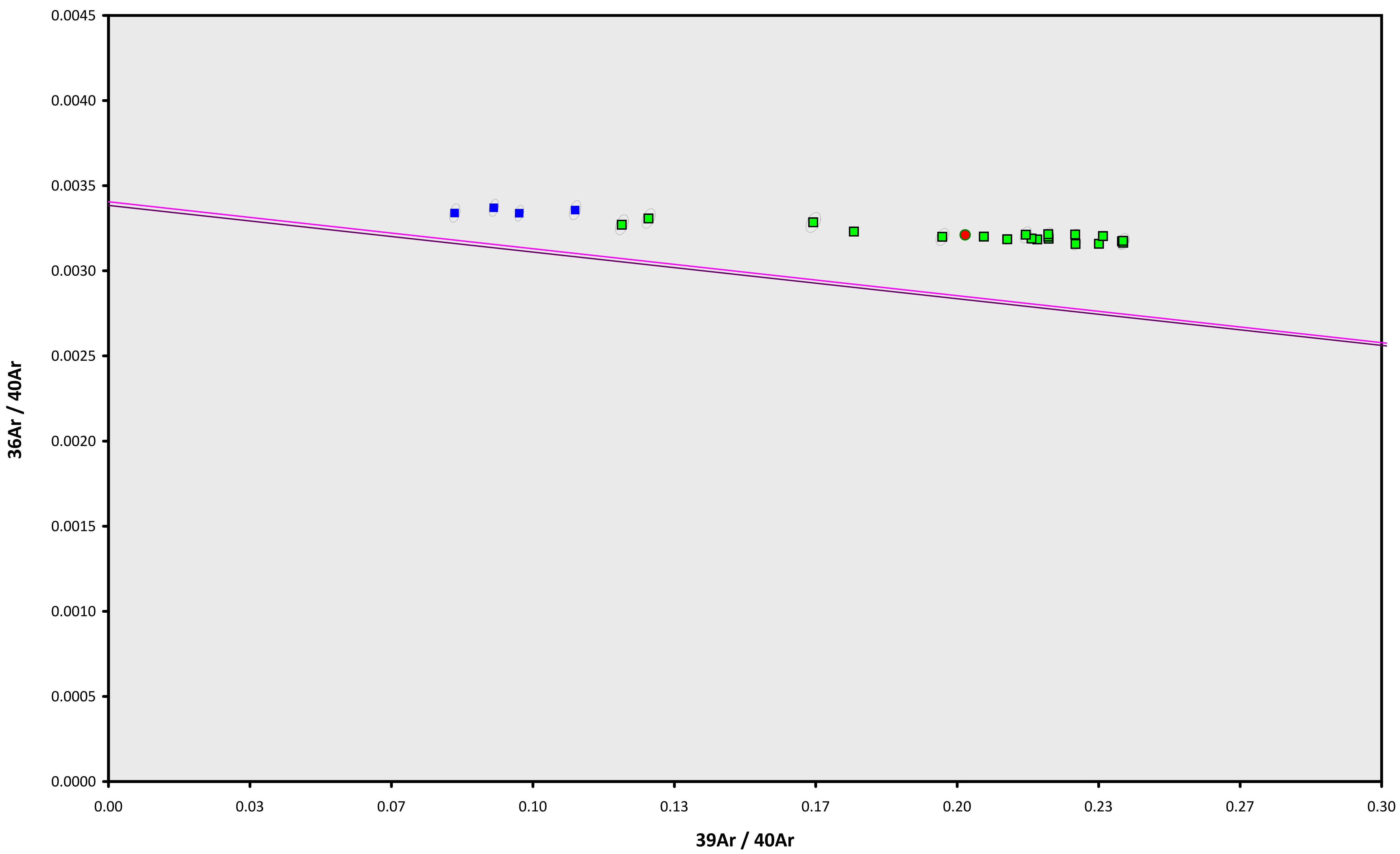

Ar-Ages in ka

WEIGHTED PLATEAU
757.9 ± 28.9
TOTAL FUSION
740.3 ± 29.9
NORMAL ISOCHRON
839.0 ± 236.6
INVERSE ISOCHRON
839.3 ± 199.7

MSWD (PROBABILITY)
1.07 (37%)

SPREADING FACTOR
3.4%

40AR/36AR INTERCEPT
293.6 ± 5.4

Sample Info

Groundmass
Gakkel Ridge
Dan Miggins

IRR = 17-OSU-01 (1F26-17)
J = 0.00161662 ± 0.00000152

Relative Abundances

			36Ar [fA]	%1σ	37Ar [fA]	%1σ	38Ar [fA]	%1σ	39Ar [fA]	%1σ	40Ar [fA]	%1σ	40(r)/39(k) ± 2σ	Age ± 2σ (Ma)	40Ar(r) (%)	39Ar(k) (%)	K/Ca ± 2σ
17D19765	1.8 %		3.2126987	0.274	33.6981	1.328	0.8315099	2.861	16.27485	0.150	953.7570	0.009	0.43612 ± 0.31978	1.29 ± 0.95	0.74	10.86	0.2074 ± 0.0055
17D19767	1.9 %		1.2742908	0.295	25.9885	1.692	0.3680833	6.090	8.59508	0.257	381.2705	0.019	0.79149 ± 0.25993	2.34 ± 0.77	1.78	5.73	0.1419 ± 0.0049
17D19768	2.0 %		0.9220193	0.309	27.8215	1.584	0.2747722	8.415	7.28739	0.325	276.8785	0.026	0.91346 ± 0.23304	2.71 ± 0.69	2.40	4.86	0.1124 ± 0.0036
17D19770	2.2 %		0.6517207	0.328	33.3406	1.346	0.1854076	11.940	6.26791	0.388	195.8411	0.036	0.94724 ± 0.20364	2.81 ± 0.60	3.02	4.17	0.0806 ± 0.0023
17D19771	2.4 %		0.4951469	0.367	39.8324	1.238	0.1637342	14.328	5.81841	0.381	147.8400	0.049	0.81172 ± 0.18783	2.40 ± 0.56	3.18	3.87	0.0625 ± 0.0016
17D19773	2.7 %		0.3645822	0.372	47.4420	0.976	0.1283153	17.691	5.48117	0.405	109.6825	0.063	1.05206 ± 0.14993	3.12 ± 0.44	5.23	3.64	0.0494 ± 0.0010
17D19774	3.0 %		0.2851264	0.405	53.2670	0.918	0.1336796	17.438	5.12679	0.416	85.8258	0.080	1.14349 ± 0.13779	3.39 ± 0.41	6.79	3.40	0.0411 ± 0.0008
17D19776	3.4 %		0.3834375	0.374	121.0296	0.570	0.1607065	14.217	8.77998	0.270	112.4803	0.063	1.01541 ± 0.09964	3.01 ± 0.29	7.86	5.81	0.0309 ± 0.0004
17D19777	3.9 %		0.2578738	0.437	119.5711	0.582	0.1378569	17.387	7.40020	0.295	74.2114	0.093	1.03174 ± 0.09444	3.06 ± 0.28	10.18	4.89	0.0263 ± 0.0003
17D19779	4.5 %	✓	0.2988563	0.394	214.2232	0.487	0.1880827	12.347	11.12400	0.211	84.7425	0.080	1.23195 ± 0.06675	3.65 ± 0.20	15.97	7.34	0.0221 ± 0.0002
17D19780	5.2 %	✓	0.2018073	0.471	184.4822	0.499	0.1567406	14.195	8.82097	0.264	55.2717	0.124	1.19135 ± 0.06913	3.53 ± 0.20	18.76	5.81	0.0203 ± 0.0002
17D19782	6.0 %	✓	0.2407476	0.422	281.7393	0.473	0.2174125	11.112	12.35513	0.192	63.1125	0.108	1.18840 ± 0.05406	3.52 ± 0.16	22.92	8.13	0.0186 ± 0.0002
17D19783	6.9 %	✓	0.1769121	0.483	235.4017	0.482	0.1505569	16.082	9.73251	0.257	45.1798	0.150	1.22102 ± 0.05847	3.62 ± 0.17	25.89	6.40	0.0175 ± 0.0002
17D19785	7.9 %	✓	0.1434915	0.542	210.8588	0.493	0.1388674	16.814	8.30799	0.257	35.7487	0.190	1.24613 ± 0.06283	3.69 ± 0.19	28.49	5.46	0.0167 ± 0.0002
17D19786	9.0 %	✓	0.1274128	0.552	193.6321	0.497	0.1219762	19.076	7.38033	0.313	31.3476	0.216	1.26226 ± 0.06481	3.74 ± 0.19	29.22	4.85	0.0161 ± 0.0002
17D19788	10.3 %	✓	0.1250128	0.600	174.5969	0.515	0.1220724	19.440	6.53885	0.365	30.3479	0.223	1.14342 ± 0.07633	3.39 ± 0.23	24.21	4.29	0.0158 ± 0.0002
17D19789	11.6 %	✓	0.1084879	0.620	135.0443	0.562	0.0774754	31.170	4.77462	0.457	26.8713	0.250	1.19389 ± 0.09411	3.53 ± 0.28	20.83	3.13	0.0149 ± 0.0002
17D19791	12.5 %	✓	0.0823559	0.769	93.3636	0.634	0.0528837	43.463	3.20420	0.714	20.4925	0.329	1.14868 ± 0.13130	3.40 ± 0.39	17.62	2.10	0.0145 ± 0.0003
17D19792	13.4 %	✓	0.0648711	0.878	66.3063	0.797	0.0537534	43.816	2.21039	0.980	16.3592	0.412	1.14638 ± 0.17342	3.39 ± 0.51	15.19	1.45	0.0141 ± 0.0004
17D19794	14.6 %		0.0663824	0.877	59.8632	0.834	0.0519357	48.674	1.77000	1.257	16.5237	0.410	0.97535 ± 0.22014	2.89 ± 0.65	10.22	1.16	0.0124 ± 0.0004
17D19795	16.0 %		0.0630411	0.909	59.3883	0.846	0.0324151	69.733	1.35354	1.639	15.1244	0.447	0.94156 ± 0.28601	2.79 ± 0.85	8.19	0.88	0.0095 ± 0.0004
17D19797	17.6 %		0.0653494	0.825	79.8669	0.715	0.0780515	30.351	1.35473	1.605	14.3441	0.469	1.08525 ± 0.27725	3.21 ± 0.82	9.86	0.87	0.0070 ± 0.0003
17D19798	19.3 %		0.0373673	1.249	45.0018	1.037	0.0479706	48.049	0.69478	3.347	8.1315	0.828	1.02933 ± 0.48009	3.05 ± 1.42	8.43	0.44	0.0064 ± 0.0005
17D19800	21.0 %		0.0668655	0.861	118.3452	0.576	0.0750943	29.429	0.72935	2.997	10.6816	0.634	0.57820 ± 0.58818	1.71 ± 1.74	3.54	0.44	0.0024 ± 0.0002
		Σ	9.7158573	0.114	2654.1043	0.136	3.9493540	2.893	151.38319	0.074	2812.0662	0.012					

Information on Analysis and Constants Used in Calculations

Project = **O-CONNOR (16-22)**
Sample = **PS59-300-18**
Material = **Groundmass**
Location = **Gakkel Ridge**
Region = **Artic Ocean**
Analyst = **Dan Miggins**
Irradiation = **17-OSU-01 (1F20-17)**
Position = **X: 0 | Y: 0 | Z/H: 25.3719 mm**
FCT-NM Age = **28.201 ± 0.023 Ma**
FCT-NM Reference = **Kuiper et al (2008)**
FCT-NM 40Ar/39Ar Ratio = **9.58986 ± 0.00921**
FCT-NM J-value = **0.00163896 ± 0.00000157**
Air Shot 40Ar/36Ar = **302.5630 ± 0.3358**
Air Shot MDF = **0.99415648 ± 0.00063982 (LIN)**
Experiment Type = **Incremental Heating**
Extraction Method = **Bulk Laser Heating**
Heating = **64 sec**
Isolation = **3.00 min**
Instrument = **ARGUS-VI-D**
Preferred Age = **Plateau Age**
Age Classification = **Crystallization Age**
IGSN = Undefined
Rock Class = **Igneous>Volcanic**
Lithology = **Basaltic Lava**
Lat-Lon = Undefined - Undefined

Age Equations = **Min et al. (2000)**
Negative Intensities = **Allowed**
Collector Calibrations = **36Ar**
Decay 40K = **5.530 ± 0.048 E-10 1/a**
Decay 39Ar = **2.940 ± 0.016 E-07 1/h**
Decay 37Ar = **8.230 ± 0.012 E-04 1/h**
Decay 36Cl = **2.257 ± 0.015 E-06 1/a**
Decay 40K(EC,β⁺) = **0.580 ± 0.009 E-10 1/a**
Decay 40K(β⁻) = **4.950 ± 0.043 E-10 1/a**
Atmospheric 40/36(a) = **295.50**
Atmospheric 38/36(a) = **0.1869**
Production 39/37(ca) = **0.0006425 ± 0.0000059**
Production 38/37(ca) = **0.0001800 ± 0.0000173**
Production 36/37(ca) = **0.0002703 ± 0.0000005**
Production 40/39(k) = **0.000607 ± 0.000059**
Production 38/39(k) = **0.012077 ± 0.000011**
Production 36/38(cl) = **262.80 ± 1.71**
Scaling Ratio K/Ca = **0.430**
Abundance Ratio 40K/K = **1.1700 ± 0.0100 E-04**
Atomic Weight K = **39.0983 ± 0.0001 g**

Results

Results	40(a)/36(a) ± 2σ	40(r)/39(k) ± 2σ	Age ± 2σ (Ma)	MSWD	39Ar(k) (%,n)	K/Ca ± 2σ
**Age Plateau**		1.20976 ± 0.02396 ± 1.98%	**3.58** ± 0.07 ± 1.99%	1.11 35%	48.97 10	0.0172 ± 0.0015
			Full External Error ± 0.11	1.94	2σ Confidence Limit	
			Analytical Error ± 0.07	1.0540	Error Magnification	
**Total Fusion Age**		1.02183 ± 0.04411 ± 4.32%	**3.03** ± 0.13 ± 4.32%		24	0.0242 ± 0.0001
			Full External Error ± 0.15			
			Analytical Error ± 0.13			
**Normal Isochron**	**293.45** ± 6.52 ± 2.22%	1.23795 ± 0.09561 ± 7.72%	**3.67** ± 0.28 ± 7.72%	1.19 30%	48.97 10	
			Full External Error ± 0.29	2.00	2σ Confidence Limit	
			Analytical Error ± 0.28	1.0929	Error Magnification	
				8	Number of Iterations	
				0.0000056038	Convergence	
**Inverse Isochron**	**293.37** ± 6.47 ± 2.21%	1.24000 ± 0.09396 ± 7.58%	**3.67** ± 0.28 ± 7.57%	1.19 30%	48.97 10	
			Full External Error ± 0.29	2.00	2σ Confidence Limit	
			Analytical Error ± 0.28	1.0888	Error Magnification	
				4	Number of Iterations	
				0.0000374340	Convergence	
				13%	Spreading Factor	

Notes
Mostly atmospheric

Incremental Heating			36Ar(a) [fA]	37Ar(ca) [fA]	38Ar(cl) [fA]	39Ar(k) [fA]	40Ar(r) [fA]	Age ± 2σ (Ma)	40Ar(r) (%)	39Ar(k) (%)	K/Ca ± 2σ
17D19765	1.8 %		3.2035832	33.6981	0.0304046	16.25320	7.08836	1.29 ± 0.95	0.74	10.86	0.2074 ± 0.0055
17D19767	1.9 %		1.2672609	25.9885	0.0229532	8.57839	6.78968	2.34 ± 0.77	1.78	5.73	0.1419 ± 0.0049
17D19768	2.0 %		0.9144966	27.8215	0.0110510	7.26951	6.64039	2.71 ± 0.69	2.40	4.86	0.1124 ± 0.0036
17D19770	2.2 %		0.6427087	33.3406	0.0000000	6.24649	5.91692	2.81 ± 0.60	3.02	4.17	0.0806 ± 0.0023
17D19771	2.4 %		0.4843802	39.8324	0.0000000	5.79282	4.70213	2.40 ± 0.56	3.18	3.87	0.0625 ± 0.0016
17D19773	2.7 %		0.3517586	47.4420	0.0000000	5.45069	5.73447	3.12 ± 0.44	5.23	3.64	0.0494 ± 0.0010
17D19774	3.0 %		0.2707256	53.2670	0.0119900	5.09257	5.82333	3.39 ± 0.41	6.79	3.40	0.0411 ± 0.0008
17D19776	3.4 %		0.3507232	121.0296	0.0000000	8.70222	8.83631	3.01 ± 0.29	7.86	5.81	0.0309 ± 0.0004
17D19777	3.9 %		0.2255537	119.5711	0.0000000	7.32338	7.55581	3.06 ± 0.28	10.18	4.89	0.0263 ± 0.0003
17D19779	4.5 %	✓	0.2409518	214.2232	0.0000000	10.98636	13.53460	3.65 ± 0.20	15.97	7.34	0.0221 ± 0.0002
17D19780	5.2 %	✓	0.1519417	184.4822	0.0000000	8.70244	10.36765	3.53 ± 0.20	18.76	5.81	0.0203 ± 0.0002
17D19782	6.0 %	✓	0.1645934	281.7393	0.0000000	12.17411	14.46771	3.52 ± 0.16	22.92	8.13	0.0186 ± 0.0002
17D19783	6.9 %	✓	0.1132830	235.4017	0.0000000	9.58127	11.69889	3.62 ± 0.17	25.89	6.40	0.0175 ± 0.0002
17D19785	7.9 %	✓	0.0864964	210.8588	0.0000000	8.17252	10.18405	3.69 ± 0.19	28.49	5.46	0.0167 ± 0.0002
17D19786	9.0 %	✓	0.0750741	193.6321	0.0000000	7.25592	9.15884	3.74 ± 0.19	29.22	4.85	0.0161 ± 0.0002
17D19788	10.3 %	✓	0.0778193	174.5969	0.0000000	6.42668	7.34839	3.39 ± 0.23	24.21	4.29	0.0158 ± 0.0002
17D19789	11.6 %	✓	0.0719854	135.0443	0.0000000	4.68785	5.59679	3.53 ± 0.28	20.83	3.13	0.0149 ± 0.0002
17D19791	12.5 %	✓	0.0571197	93.3636	0.0000000	3.14421	3.61168	3.40 ± 0.39	17.62	2.10	0.0145 ± 0.0003
17D19792	13.4 %	✓	0.0469469	66.3063	0.0068635	2.16779	2.48511	3.39 ± 0.51	15.19	1.45	0.0141 ± 0.0004
17D19794	14.6 %		0.0501988	59.8632	0.0108664	1.73154	1.68885	2.89 ± 0.65	10.22	1.16	0.0124 ± 0.0004
17D19795	16.0 %		0.0469884	59.3883	0.0000000	1.31539	1.23851	2.79 ± 0.85	8.19	0.88	0.0095 ± 0.0004
17D19797	17.6 %		0.0437522	79.8669	0.0397569	1.30341	1.41452	3.21 ± 0.82	9.86	0.87	0.0070 ± 0.0003
17D19798	19.3 %		0.0251971	45.0018	0.0271193	0.66586	0.68539	3.05 ± 1.42	8.43	0.44	0.0064 ± 0.0005
17D19800	21.0 %		0.0348677	118.3452	0.0393853	0.65332	0.37775	1.71 ± 1.74	3.54	0.44	0.0024 ± 0.0002
		Σ	8.9984067	2654.1043	0.2003902	149.67793	152.94611				

Information on Analysis

Project = **O-CONNOR (16-22)**
Sample = **PS59-300-18**
Material = **Groundmass**
Location = **Gakkel Ridge**
Region = **Artic Ocean**
Analyst = **Dan Miggins**
Irradiation = **17-OSU-01 (1F20-17)**
J = **0.00163896 ± 0.00000157**
FCT-NM = **28.201 ± 0.023 Ma**

Results	40(r)/39(k) ± 2σ			Age ± 2σ (Ma)	MSWD	39Ar(k) (%,n)	K/Ca ± 2σ
**Age Plateau**	1.20976	± 0.02396<br>± 1.98%	**3.58**	**± 0.07**<br>**± 1.99%**	1.11<br>35%	48.97<br>10	0.0172 ± 0.0015
			Full External Error	± 0.11	1.94	2σ Confidence Limit	
			Analytical Error	± 0.07	1.0540	Error Magnification	
**Total Fusion Age**	1.02183	± 0.04411<br>± 4.32%	**3.03**	**± 0.13**<br>**± 4.32%**		24	0.0242 ± 0.0001
			Full External Error	± 0.15			
			Analytical Error	± 0.13			

**Normal Isochron**			**39(k)/36(a) ± 2σ**	**40(a+r)/36(a) ± 2σ**	**r.i.**
17D19765	1.8 %		5.07 ± 0.03	297.71 ± 1.63	0.8771
17D19767	1.9 %		6.77 ± 0.05	300.86 ± 1.79	0.7544
17D19768	2.0 %		7.95 ± 0.07	302.76 ± 1.90	0.6894
17D19770	2.2 %		9.72 ± 0.10	304.71 ± 2.04	0.6458
17D19771	2.4 %		11.96 ± 0.13	305.21 ± 2.32	0.6952
17D19773	2.7 %		15.50 ± 0.17	311.80 ± 2.44	0.6799
17D19774	3.0 %		18.81 ± 0.23	317.01 ± 2.77	0.7030
17D19776	3.4 %		24.81 ± 0.25	320.69 ± 2.67	0.8242
17D19777	3.9 %		32.47 ± 0.38	329.00 ± 3.39	0.8482
17D19779	4.5 %	✓	45.60 ± 0.50	351.67 ± 3.59	0.9092
17D19780	5.2 %	✓	57.27 ± 0.80	363.73 ± 4.81	0.9079
17D19782	6.0 %	✓	73.96 ± 1.02	383.40 ± 5.12	0.9462
17D19783	6.9 %	✓	84.58 ± 1.43	398.77 ± 6.54	0.9352
17D19785	7.9 %	✓	94.48 ± 1.89	413.24 ± 8.11	0.9468
17D19786	9.0 %	✓	96.65 ± 2.04	417.50 ± 8.60	0.9321
17D19788	10.3 %	✓	82.58 ± 1.79	389.93 ± 8.13	0.9175
17D19789	11.6 %	✓	65.12 ± 1.41	373.25 ± 7.55	0.8753
17D19791	12.5 %	✓	55.05 ± 1.49	358.73 ± 8.55	0.8114
17D19792	13.4 %	✓	46.18 ± 1.48	348.43 ± 9.19	0.7424
17D19794	14.6 %		34.49 ± 1.21	329.14 ± 8.30	0.6429
17D19795	16.0 %		27.99 ± 1.18	321.86 ± 8.58	0.5624
17D19797	17.6 %		29.79 ± 1.25	327.83 ± 8.96	0.5727
17D19798	19.3 %		26.43 ± 2.11	322.70 ± 13.50	0.4425
17D19800	21.0 %		18.74 ± 1.41	306.33 ± 11.35	0.4335

**Results**	**40(a)/36(a) ± 2σ**	**40(r)/39(k) ± 2σ**	**Age ± 2σ (Ma)**	**MSWD**
**Normal Isochron**	**293.45 ± 6.52<br>± 2.22%**	1.23795 ± 0.09561<br>± 7.72%	**3.67 ± 0.28<br>± 7.72%**<br>Full External Error ± 0.29<br>Analytical Error ± 0.28	1.19<br>30%

**Statistics**				
	2σ Confidence Limit	2.00	Convergence	0.000005603810
	Error Magnification	1.0929	Number of Iterations	8
	Number of Data Points	10	Calculated Line	Weighted York-2

**Inverse Isochron**			**39(k)/40(a+r) ± 2σ**	**36(a)/40(a+r) ± 2σ**	**r.i.**
17D19765	1.8 %		0.0170414 ± 0.0000512	0.00335894 ± 0.00001844	0.0018
17D19767	1.9 %		0.0224998 ± 0.0001161	0.00332383 ± 0.00001979	0.0048
17D19768	2.0 %		0.0262557 ± 0.0001717	0.00330293 ± 0.00002070	0.0066
17D19770	2.2 %		0.0318963 ± 0.0002495	0.00328185 ± 0.00002196	0.0097
17D19771	2.4 %		0.0391840 ± 0.0003026	0.00327646 ± 0.00002488	0.0162
17D19773	2.7 %		0.0496967 ± 0.0004095	0.00320716 ± 0.00002514	0.0243
17D19774	3.0 %		0.0593382 ± 0.0005066	0.00315447 ± 0.00002753	0.0344
17D19776	3.4 %		0.0773703 ± 0.0004334	0.00311823 ± 0.00002600	0.0338
17D19777	3.9 %		0.0986886 ± 0.0006161	0.00303952 ± 0.00003135	0.0532
17D19779	4.5 %	✓	0.1296542 ± 0.0005926	0.00284356 ± 0.00002905	0.0548
17D19780	5.2 %	✓	0.1574634 ± 0.0009306	0.00274926 ± 0.00003635	0.0787
17D19782	6.0 %	✓	0.1929182 ± 0.0008613	0.00260824 ± 0.00003485	0.0789
17D19783	6.9 %	✓	0.2120969 ± 0.0012793	0.00250770 ± 0.00004115	0.0908
17D19785	7.9 %	✓	0.2286419 ± 0.0014782	0.00241990 ± 0.00004751	0.1134
17D19786	9.0 %	✓	0.2314989 ± 0.0017822	0.00239522 ± 0.00004931	0.1180
17D19788	10.3 %	✓	0.2117940 ± 0.0018379	0.00256457 ± 0.00005347	0.1103
17D19789	11.6 %	✓	0.1744739 ± 0.0018439	0.00267918 ± 0.00005420	0.1168
17D19791	12.5 %	✓	0.1534468 ± 0.0024497	0.00278761 ± 0.00006644	0.1135
17D19792	13.4 %	✓	0.1325222 ± 0.0028662	0.00286998 ± 0.00007567	0.1188
17D19794	14.6 %		0.1047979 ± 0.0028282	0.00303819 ± 0.00007659	0.0989
17D19795	16.0 %		0.0869758 ± 0.0030351	0.00310696 ± 0.00008280	0.0860
17D19797	17.6 %		0.0908725 ± 0.0031510	0.00305036 ± 0.00008340	0.0929
17D19798	19.3 %		0.0818907 ± 0.0058784	0.00309884 ± 0.00012960	0.0913
17D19800	21.0 %		0.0611652 ± 0.0041683	0.00326441 ± 0.00012096	0.0636

**Results**	**40(a)/36(a) ± 2σ**	**40(r)/39(k) ± 2σ**	**Age ± 2σ (Ma)**	**MSWD**
**Inverse Isochron**	**293.37 ± 6.47 ± 2.21%**	1.24000 ± 0.09396 ± 7.58%	**3.67 ± 0.28 ± 7.57%**	1.19 30%
		Full External Error	± 0.29	
		Analytical Error	± 0.28	

**Statistics**				
	2σ Confidence Limit	2.00	Convergence	0.0000374340
	Error Magnification	1.0888	Number of Iterations	4
	Number of Data Points	10	Calculated Line	Weighted York-2
	Spreading Factor	12.6%		

**Degassing Patterns**			36Ar(a) [fA]	%1σ	36Ar(c) [fA]	%1σ	36Ar(ca) [fA]	%1σ	36Ar(cl) [fA]	%1σ	37Ar(ca) [fA]	%1σ	38Ar(a) [fA]	%1σ	38Ar(c) [fA]	%1σ	38Ar(k) [fA]	%1σ	38Ar(ca) [fA]	%1σ	38Ar(cl) [fA]	%1σ	39Ar(k) [fA]	%1σ	39Ar(ca) [fA]	%1σ	40Ar(r) [fA]	%1σ	40Ar(a) [fA]	%1σ	40Ar(c) [fA]	%1σ	40Ar(k) [fA]	%1σ
17D19765	1.8 %		3.2035832	0.27	0.0000000	0.00	0.0091086	1.34	0.0000070	78.46	33.6981	1.33	0.5987497	0.27	0.0000000	0.00	0.1962899	0.17	0.0060657	9.72	0.0304046	78.46	16.25320	0.15	0.0216510	1.62	7.08836	36.66	946.6588	0.27	0.0000000	0.00	0.0098657	9.65
17D19767	1.9 %		1.2672609	0.30	0.0000000	0.00	0.0070247	1.70	0.0000053	97.75	25.9885	1.69	0.2368511	0.30	0.0000000	0.00	0.1036012	0.27	0.0046779	9.78	0.0229532	97.75	8.57839	0.26	0.0166976	1.93	6.78968	16.42	374.4756	0.30	0.0000000	0.00	0.0052071	9.65
17D19768	2.0 %		0.9144966	0.31	0.0000000	0.00	0.0075202	1.59	0.0000025	209.37	27.8215	1.58	0.1709194	0.31	0.0000000	0.00	0.0877939	0.34	0.0050079	9.76	0.0110510	209.37	7.26951	0.33	0.0178753	1.83	6.64039	12.75	270.2337	0.31	0.0000000	0.00	0.0044126	9.66
17D19770	2.2 %		0.6427087	0.33	0.0000000	0.00	0.0090120	1.36	0.0000000	0.00	33.3406	1.35	0.1201223	0.33	0.0000000	0.00	0.0754389	0.40	0.0060013	9.72	0.0000000	0.00	6.24649	0.39	0.0214213	1.63	5.91692	10.74	189.9204	0.33	0.0000000	0.00	0.0037916	9.66
17D19771	2.4 %		0.4843802	0.38	0.0000000	0.00	0.0107667	1.25	0.0000000	0.00	39.8324	1.24	0.0905307	0.38	0.0000000	0.00	0.0699599	0.39	0.0071698	9.71	0.0000000	0.00	5.79282	0.38	0.0255923	1.54	4.70213	11.56	143.1344	0.38	0.0000000	0.00	0.0035162	9.66
17D19773	2.7 %		0.3517586	0.39	0.0000000	0.00	0.0128236	0.99	0.0000000	0.00	47.4420	0.98	0.0657437	0.39	0.0000000	0.00	0.0658280	0.42	0.0085396	9.68	0.0000000	0.00	5.45069	0.41	0.0304815	1.34	5.73447	7.11	103.9447	0.39	0.0000000	0.00	0.0033086	9.66
17D19774	3.0 %		0.2707256	0.43	0.0000000	0.00	0.0143981	0.93	0.0000028	194.61	53.2670	0.92	0.0505986	0.43	0.0000000	0.00	0.0615030	0.43	0.0095881	9.67	0.0119900	194.61	5.09257	0.42	0.0342240	1.30	5.82333	6.01	79.9994	0.43	0.0000000	0.00	0.0030912	9.66
17D19776	3.4 %		0.3507232	0.41	0.0000000	0.00	0.0327143	0.59	0.0000000	0.00	121.0296	0.57	0.0655502	0.41	0.0000000	0.00	0.1050967	0.29	0.0217853	9.65	0.0000000	0.00	8.70222	0.27	0.0777615	1.08	8.83631	4.90	103.6387	0.41	0.0000000	0.00	0.0052822	9.65
17D19777	3.9 %		0.2255537	0.51	0.0000000	0.00	0.0323201	0.61	0.0000000	0.00	119.5711	0.58	0.0421560	0.51	0.0000000	0.00	0.0884444	0.31	0.0215228	9.65	0.0000000	0.00	7.32338	0.30	0.0768245	1.09	7.55581	4.57	66.6511	0.51	0.0000000	0.00	0.0044453	9.65
17D19779	4.5 %	✓	0.2409518	0.50	0.0000000	0.00	0.0579045	0.52	0.0000000	0.00	214.2232	0.49	0.0450339	0.50	0.0000000	0.00	0.1326823	0.23	0.0385602	9.64	0.0000000	0.00	10.98636	0.21	0.1376384	1.04	13.53460	2.70	71.2012	0.50	0.0000000	0.00	0.0066687	9.65
17D19780	5.2 %	✓	0.1519417	0.65	0.0000000	0.00	0.0498655	0.53	0.0000000	0.00	184.4822	0.50	0.0283979	0.65	0.0000000	0.00	0.1050994	0.28	0.0332068	9.64	0.0000000	0.00	8.70244	0.27	0.1185298	1.05	10.36765	2.89	44.8988	0.65	0.0000000	0.00	0.0052824	9.65
17D19782	6.0 %	✓	0.1645934	0.66	0.0000000	0.00	0.0761541	0.50	0.0000000	0.00	281.7393	0.47	0.0307625	0.66	0.0000000	0.00	0.1470268	0.21	0.0507131	9.64	0.0000000	0.00	12.17411	0.20	0.1810175	1.03	14.46771	2.27	48.6374	0.66	0.0000000	0.00	0.0073897	9.65
17D19783	6.9 %	✓	0.1132830	0.81	0.0000000	0.00	0.0636291	0.51	0.0000000	0.00	235.4017	0.48	0.0211726	0.81	0.0000000	0.00	0.1157130	0.28	0.0423723	9.64	0.0000000	0.00	9.58127	0.26	0.1512456	1.04	11.69889	2.38	33.4751	0.81	0.0000000	0.00	0.0058158	9.65
17D19785	7.9 %	✓	0.0864964	0.96	0.0000000	0.00	0.0569951	0.52	0.0000000	0.00	210.8588	0.49	0.0161662	0.96	0.0000000	0.00	0.0986995	0.28	0.0379546	9.64	0.0000000	0.00	8.17252	0.26	0.1354768	1.04	10.18405	2.51	25.5597	0.96	0.0000000	0.00	0.0049607	9.65
17D19786	9.0 %	✓	0.0750741	1.01	0.0000000	0.00	0.0523387	0.52	0.0000000	0.00	193.6321	0.50	0.0140313	1.01	0.0000000	0.00	0.0876298	0.33	0.0348538	9.64	0.0000000	0.00	7.25592	0.32	0.1244086	1.05	9.15884	2.55	22.1844	1.01	0.0000000	0.00	0.0044043	9.66
17D19788	10.3 %	✓	0.0778193	1.02	0.0000000	0.00	0.0471935	0.54	0.0000000	0.00	174.5969	0.51	0.0145444	1.02	0.0000000	0.00	0.0776150	0.38	0.0314274	9.64	0.0000000	0.00	6.42668	0.37	0.1121785	1.05	7.34839	3.32	22.9956	1.02	0.0000000	0.00	0.0039010	9.66
17D19789	11.6 %	✓	0.0719854	0.98	0.0000000	0.00	0.0365025	0.59	0.0000000	0.00	135.0443	0.56	0.0134541	0.98	0.0000000	0.00	0.0566152	0.47	0.0243080	9.65	0.0000000	0.00	4.68785	0.47	0.0867660	1.08	5.59679	3.91	21.2717	0.98	0.0000000	0.00	0.0028455	9.66
17D19791	12.5 %	✓	0.0571197	1.15	0.0000000	0.00	0.0252362	0.66	0.0000000	0.00	93.3636	0.63	0.0106757	1.15	0.0000000	0.00	0.0379726	0.73	0.0168054	9.65	0.0000000	0.00	3.14421	0.73	0.0599861	1.12	3.61168	5.67	16.8789	1.15	0.0000000	0.00	0.0019085	9.68
17D19792	13.4 %	✓	0.0469469	1.25	0.0000000	0.00	0.0179226	0.81	0.0000016	343.61	66.3063	0.80	0.0087744	1.25	0.0000000	0.00	0.0261804	1.00	0.0119351	9.66	0.0068635	343.61	2.16779	1.00	0.0426018	1.22	2.48511	7.50	13.8728	1.25	0.0000000	0.00	0.0013158	9.70
17D19794	14.6 %		0.0501988	1.19	0.0000000	0.00	0.0161810	0.85	0.0000025	232.86	59.8632	0.83	0.0093822	1.19	0.0000000	0.00	0.0209117	1.29	0.0107754	9.67	0.0108664	232.86	1.73154	1.29	0.0384621	1.24	1.68885	11.21	14.8338	1.19	0.0000000	0.00	0.0010510	9.74
17D19795	16.0 %		0.0469884	1.26	0.0000000	0.00	0.0160527	0.86	0.0000000	0.00	59.3883	0.85	0.0087821	1.26	0.0000000	0.00	0.0158859	1.69	0.0106899	9.67	0.0000000	0.00	1.31539	1.69	0.0381570	1.25	1.23851	15.09	13.8851	1.26	0.0000000	0.00	0.0007984	9.80
17D19797	17.6 %		0.0437522	1.28	0.0000000	0.00	0.0215880	0.73	0.0000092	59.70	79.8669	0.71	0.0081773	1.28	0.0000000	0.00	0.0157413	1.67	0.0143760	9.66	0.0397569	59.71	1.30341	1.67	0.0513145	1.17	1.41452	12.66	12.9288	1.28	0.0000000	0.00	0.0007912	9.79
17D19798	19.3 %		0.0251971	1.92	0.0000000	0.00	0.0121640	1.05	0.0000063	85.06	45.0018	1.04	0.0047093	1.92	0.0000000	0.00	0.0080416	3.49	0.0081003	9.69	0.0271193	85.06	0.66586	3.49	0.0289136	1.39	0.68539	23.06	7.4457	1.92	0.0000000	0.00	0.0004042	10.26
17D19800	21.0 %		0.0348677	1.74	0.0000000	0.00	0.0319887	0.60	0.0000091	56.37	118.3452	0.58	0.0065168	1.74	0.0000000	0.00	0.0078901	3.35	0.0213021	9.65	0.0393853	56.38	0.65332	3.35	0.0760368	1.09	0.37775	50.75	10.3034	1.74	0.0000000	0.00	0.0003966	10.21
		Σ	8.9984067	0.12	0.0000000	0.00	0.7174044	0.14	0.0000462	35.06	2654.1043	0.14	1.6818022	0.12	0.0000000	0.00	1.8076603	0.08	0.4777388	2.38	0.2003902	35.07	149.67793	0.07	1.7052620	0.26	152.94611	2.16	2659.0292	0.12	0.0000000	0.00	0.0908545	2.32
		Σ							9.7158573	0.11	2654.1043	0.14									4.1675916	1.71			151.38319	0.07							2812.0662	0.17

Additional Parameters			40Ar/39Ar	1σ	37Ar/39Ar	1σ	36Ar/39Ar	1σ	Time (days)	37Ar (decay)	39Ar (decay)	40Ar (moles)
17D19765	1.8 %		58.603115	0.087882	2.070562	0.027678	0.197403	0.000616	141.707	16.468732	1.00100127	4.578E-11
17D19767	1.9 %		44.359139	0.114179	3.023645	0.051750	0.148258	0.000580	141.721	16.473251	1.00100137	1.830E-11
17D19768	2.0 %		37.994212	0.123897	3.817761	0.061730	0.126523	0.000568	141.728	16.475510	1.00100141	1.329E-11
17D19770	2.2 %		31.245026	0.121753	5.319250	0.074494	0.103977	0.000528	141.742	16.480031	1.00100151	9.400E-12
17D19771	2.4 %		25.408997	0.097680	6.845917	0.088688	0.085100	0.000451	141.749	16.482291	1.00100156	7.096E-12
17D19773	2.7 %		20.010768	0.081988	8.655452	0.091465	0.066515	0.000366	141.762	16.486814	1.00100166	5.265E-12
17D19774	3.0 %		16.740640	0.070991	10.389920	0.104780	0.055615	0.000323	141.769	16.489075	1.00100171	4.120E-12
17D19776	3.4 %		12.810994	0.035561	13.784718	0.086905	0.043672	0.000201	141.783	16.493599	1.00100181	5.399E-12
17D19777	3.9 %		10.028293	0.030985	16.157822	0.105394	0.034847	0.000184	141.790	16.495862	1.00100186	3.562E-12
17D19779	4.5 %	✓	7.617991	0.017192	19.257753	0.102280	0.026866	0.000120	141.804	16.500388	1.00100195	4.068E-12
17D19780	5.2 %	✓	6.265944	0.018289	20.914045	0.118151	0.022878	0.000124	141.811	16.502651	1.00100200	2.653E-12
17D19782	6.0 %	✓	5.108198	0.011249	22.803425	0.116300	0.019486	0.000090	141.825	16.507179	1.00100210	3.029E-12
17D19783	6.9 %	✓	4.642154	0.013816	24.187137	0.132089	0.018177	0.000099	141.832	16.509444	1.00100215	2.169E-12
17D19785	7.9 %	✓	4.302928	0.013741	25.380228	0.141074	0.017272	0.000104	141.846	16.513973	1.00100225	1.716E-12
17D19786	9.0 %	✓	4.247456	0.016143	26.236224	0.153953	0.017264	0.000110	141.853	16.516239	1.00100230	1.505E-12
17D19788	10.3 %	✓	4.641163	0.019866	26.701449	0.168487	0.019118	0.000134	141.867	16.520770	1.00100240	1.457E-12
17D19789	11.6 %	✓	5.627957	0.029299	28.283808	0.204723	0.022722	0.000175	141.874	16.523036	1.00100244	1.290E-12
17D19791	12.5 %	✓	6.395508	0.050240	29.137922	0.278150	0.025703	0.000270	141.888	16.527797	1.00100255	9.836E-13
17D19792	13.4 %	✓	7.401063	0.078699	29.997554	0.378955	0.029348	0.000386	141.895	16.530064	1.00100260	7.852E-13
17D19794	14.6 %		9.335414	0.123462	33.821073	0.510359	0.037504	0.000575	141.909	16.534599	1.00100269	7.931E-13
17D19795	16.0 %		11.173929	0.189791	43.876169	0.809047	0.046575	0.000873	141.916	16.536867	1.00100274	7.260E-13
17D19797	17.6 %		10.588181	0.177075	58.954240	1.035927	0.048238	0.000871	141.930	16.541404	1.00100284	6.885E-13
17D19798	19.3 %		11.703798	0.403482	64.771581	2.269322	0.053783	0.001921	141.937	16.543674	1.00100289	3.903E-13
17D19800	21.0 %		14.645259	0.448588	162.260461	4.951612	0.091678	0.002859	141.951	16.548213	1.00100299	5.127E-13

**Procedure Blanks**		36Ar ± 1σ (SE) [fA]	37Ar ± 1σ (SE) [fA]	38Ar ± 1σ (SE) [fA]	39Ar ± 1σ (SE) [fA]	40Ar ± 1σ (SE) [fA]
17D19765	1.8 %	0.0084857 ± 0.0003109	0.1396199 ± 0.0179545	0.0386306 ± 0.0163090	0.0282446 ± 0.0154250	2.4604964 ± 0.0650525
17D19767	1.9 %	0.0091090 ± 0.0003109	0.1441408 ± 0.0179545	0.0326749 ± 0.0163090	0.0266852 ± 0.0154250	2.5540459 ± 0.0650525
17D19768	2.0 %	0.0092816 ± 0.0003109	0.1443374 ± 0.0179545	0.0309413 ± 0.0163090	0.0266300 ± 0.0154250	2.5783812 ± 0.0650525
17D19770	2.2 %	0.0094109 ± 0.0003109	0.1419587 ± 0.0179545	0.0294182 ± 0.0163090	0.0273822 ± 0.0154250	2.5918486 ± 0.0650525
17D19771	2.4 %	0.0093904 ± 0.0003109	0.1398656 ± 0.0179545	0.0294319 ± 0.0163090	0.0279869 ± 0.0154250	2.5845288 ± 0.0650525
17D19773	2.7 %	0.0092306 ± 0.0003109	0.1349262 ± 0.0179545	0.0305707 ± 0.0163090	0.0292259 ± 0.0154250	2.5498474 ± 0.0650525
17D19774	3.0 %	0.0091095 ± 0.0003109	0.1324413 ± 0.0179545	0.0315408 ± 0.0163090	0.0297213 ± 0.0154250	2.5253899 ± 0.0650525
17D19776	3.4 %	0.0088248 ± 0.0003109	0.1281939 ± 0.0179545	0.0339476 ± 0.0163090	0.0301952 ± 0.0154250	2.4686964 ± 0.0650525
17D19777	3.9 %	0.0086750 ± 0.0003109	0.1266723 ± 0.0179545	0.0352713 ± 0.0163090	0.0300985 ± 0.0154250	2.4387205 ± 0.0650525
17D19779	4.5 %	0.0083894 ± 0.0003109	0.1252842 ± 0.0179545	0.0379293 ± 0.0163090	0.0291283 ± 0.0154250	2.3803551 ± 0.0650525
17D19780	5.2 %	0.0082627 ± 0.0003109	0.1255379 ± 0.0179545	0.0391925 ± 0.0163090	0.0282431 ± 0.0154250	2.3535817 ± 0.0650525
17D19782	6.0 %	0.0080594 ± 0.0003109	0.1280905 ± 0.0179545	0.0414618 ± 0.0163090	0.0257225 ± 0.0154250	2.3080881 ± 0.0650525
17D19783	6.9 %	0.0079874 ± 0.0003109	0.1303888 ± 0.0179545	0.0424386 ± 0.0163090	0.0241390 ± 0.0154250	2.2903399 ± 0.0650525
17D19785	7.9 %	0.0079090 ± 0.0003109	0.1368774 ± 0.0179545	0.0440561 ± 0.0163090	0.0205346 ± 0.0154250	2.2664652 ± 0.0650525
17D19786	9.0 %	0.0079029 ± 0.0003109	0.1409466 ± 0.0179545	0.0447094 ± 0.0163090	0.0186292 ± 0.0154250	2.2606666 ± 0.0650525
17D19788	10.3 %	0.0079518 ± 0.0003109	0.1502811 ± 0.0179545	0.0457891 ± 0.0163090	0.0149803 ± 0.0154250	2.2613613 ± 0.0650525
17D19789	11.6 %	0.0080026 ± 0.0003109	0.1553047 ± 0.0179545	0.0462700 ± 0.0163090	0.0134159 ± 0.0154250	2.2675384 ± 0.0650525
17D19791	12.5 %	0.0081481 ± 0.0003109	0.1657885 ± 0.0179545	0.0473618 ± 0.0163090	0.0112803 ± 0.0154250	2.2913802 ± 0.0650525
17D19792	13.4 %	0.0082265 ± 0.0003109	0.1703636 ± 0.0179545	0.0480235 ± 0.0163090	0.0110690 ± 0.0154250	2.3068811 ± 0.0650525
17D19794	14.6 %	0.0083729 ± 0.0003109	0.1776206 ± 0.0179545	0.0499236 ± 0.0163090	0.0129100 ± 0.0154250	2.3425996 ± 0.0650525
17D19795	16.0 %	0.0084273 ± 0.0003109	0.1798154 ± 0.0179545	0.0513015 ± 0.0163090	0.0152709 ± 0.0154250	2.3611914 ± 0.0650525
17D19797	17.6 %	0.0084596 ± 0.0003109	0.1799690 ± 0.0179545	0.0553149 ± 0.0163090	0.0237244 ± 0.0154250	2.3949705 ± 0.0650525
17D19798	19.3 %	0.0084195 ± 0.0003109	0.1773201 ± 0.0179545	0.0581320 ± 0.0163090	0.0301891 ± 0.0154250	2.4078880 ± 0.0650525
17D19800	21.0 %	0.0081764 ± 0.0003109	0.1649007 ± 0.0179545	0.0658929 ± 0.0163090	0.0486048 ± 0.0154250	2.4192988 ± 0.0650525

Intercept Values		36Ar ± 1σ (SE) [fA]	r2	Regression (type,n)	37Ar ± 1σ (SE) [fA]	r2	Regression (type,n)	38Ar ± 1σ (SE) [fA]	r2	Regression (type,n)	39Ar ± 1σ (SE) [fA]	r2	Regression (type,n)	40Ar ± 1σ (SE) [fA]	r2	Regression (type,n)
17D19765	1.8 %	3.1016876 ± 0.0024455	0.9876	EXP 150 of 150	1.8707476 ± 0.0176214	0.2588	EXP 150 of 150	0.7831631 ± 0.0168963	0.0461	EXP 150 of 150	16.1355048 ± 0.0154879	0.9716	EXP 150 of 150	956.217544 ± 0.050154	0.9998	EXP 150 of 150
17D19767	1.9 %	1.2360024 ± 0.0016477	0.9650	EXP 150 of 150	1.4058601 ± 0.0178283	0.1875	EXP 150 of 150	0.3311073 ± 0.0149869	0.0127	EXP 150 of 150	8.5097209 ± 0.0145630	0.9079	EXP 150 of 150	383.824517 ± 0.034164	0.9988	EXP 150 of 150
17D19768	2.0 %	0.8970062 ± 0.0014314	0.9464	EXP 150 of 150	1.5147608 ± 0.0177068	0.1232	EXP 150 of 150	0.2406202 ± 0.0160050	0.0116	EXP 150 of 150	7.2110077 ± 0.0171443	0.8327	EXP 150 of 150	279.456925 ± 0.031291	0.9965	EXP 150 of 150
17D19770	2.2 %	0.6368907 ± 0.0011959	0.9253	EXP 149 of 150	1.8457182 ± 0.0177354	0.2947	EXP 150 of 150	0.1538230 ± 0.0145833	0.0003	EXP 150 of 150	6.1977415 ± 0.0181554	0.7678	EXP 150 of 150	198.432996 ± 0.024747	0.9902	EXP 150 of 150
17D19771	2.4 %	0.4861203 ± 0.0011889	0.8722	EXP 149 of 150	2.2345071 ± 0.0207322	0.2765	EXP 150 of 150	0.1323891 ± 0.0164798	0.0014	EXP 150 of 150	5.7507026 ± 0.0152922	0.7944	EXP 150 of 150	150.424528 ± 0.031141	0.6905	EXP 150 of 150
17D19773	2.7 %	0.3602521 ± 0.0008724	0.8494	EXP 150 of 150	2.6922756 ± 0.0167438	0.4694	EXP 150 of 150	0.0962452 ± 0.0154044	0.0000	EXP 150 of 150	5.4145253 ± 0.0153526	0.8001	EXP 150 of 150	112.232299 ± 0.021953	0.9008	EXP 150 of 150
17D19774	3.0 %	0.2836305 ± 0.0007879	0.7637	EXP 150 of 150	3.0414511 ± 0.0180914	0.5006	EXP 150 of 150	0.1005768 ± 0.0162716	0.0187	EXP 150 of 150	5.0620720 ± 0.0141774	0.7922	EXP 150 of 150	88.351210 ± 0.022060	0.9817	EXP 150 of 150
17D19776	3.4 %	0.3780003 ± 0.0009334	0.8591	EXP 150 of 150	7.0813278 ± 0.0181835	0.8218	EXP 150 of 150	0.1248811 ± 0.0156169	0.0023	EXP 150 of 150	8.6898438 ± 0.0169292	0.8870	EXP 150 of 150	114.948987 ± 0.027537	0.6705	EXP 150 of 150
17D19777	3.9 %	0.2569572 ± 0.0008121	0.7336	EXP 150 of 150	6.9949960 ± 0.0196686	0.8132	EXP 150 of 150	0.1009747 ± 0.0171797	0.0057	EXP 150 of 150	7.3195798 ± 0.0144637	0.9003	EXP 150 of 150	76.650109 ± 0.022022	0.9869	EXP 150 of 150
17D19779	4.5 %	0.2961297 ± 0.0007893	0.8032	EXP 150 of 150	12.6303701 ± 0.0175670	0.9427	EXP 150 of 150	0.1479557 ± 0.0161474	0.0004	EXP 150 of 150	11.0189219 ± 0.0159776	0.9455	EXP 150 of 150	87.122865 ± 0.019045	0.9799	EXP 150 of 150
17D19780	5.2 %	0.2025638 ± 0.0006950	0.6140	EXP 150 of 150	10.8577184 ± 0.0169520	0.9277	EXP 150 of 150	0.1157166 ± 0.0147474	0.0086	EXP 150 of 150	8.7325038 ± 0.0163197	0.9140	EXP 150 of 150	57.625297 ± 0.021481	0.9927	EXP 150 of 150
17D19782	6.0 %	0.2398523 ± 0.0007007	0.7337	EXP 150 of 150	16.6408242 ± 0.0192005	0.9616	EXP 150 of 150	0.1734103 ± 0.0174377	0.0271	EXP 150 of 150	12.2450535 ± 0.0159028	0.9567	EXP 150 of 150	65.420547 ± 0.021340	0.9893	EXP 150 of 150
17D19783	6.9 %	0.1783192 ± 0.0006172	0.5671	EXP 150 of 150	13.8786222 ± 0.0181972	0.9490	EXP 150 of 150	0.1063590 ± 0.0175108	0.0013	EXP 150 of 150	9.6419258 ± 0.0184726	0.9036	EXP 150 of 150	47.470171 ± 0.018775	0.9954	EXP 150 of 150
17D19785	7.9 %	0.1460634 ± 0.0005780	0.3712	EXP 150 of 150	12.4081184 ± 0.0192731	0.9324	EXP 150 of 150	0.0931886 ± 0.0163243	0.0232	EXP 149 of 150	8.2307355 ± 0.0135437	0.9303	EXP 150 of 150	38.015163 ± 0.019082	0.9959	EXP 150 of 150
17D19786	9.0 %	0.1305766 ± 0.0005094	0.3168	EXP 150 of 150	11.3775691 ± 0.0176401	0.9308	EXP 150 of 150	0.0758414 ± 0.0162112	0.0033	EXP 150 of 150	7.3113127 ± 0.0162742	0.8769	EXP 150 of 150	33.608302 ± 0.019009	0.9963	EXP 150 of 150
17D19788	10.3 %	0.1283148 ± 0.0005705	0.3488	EXP 150 of 150	10.2330476 ± 0.0197112	0.9060	EXP 150 of 150	0.0748569 ± 0.0168548	0.0092	EXP 150 of 150	6.4792280 ± 0.0175200	0.8303	EXP 150 of 150	32.609252 ± 0.019056	0.9960	EXP 150 of 150
17D19789	11.6 %	0.1124553 ± 0.0004976	0.1622	EXP 149 of 150	7.8747192 ± 0.0206879	0.8317	EXP 150 of 150	0.0303002 ± 0.0174250	0.0011	EXP 150 of 150	4.7285994 ± 0.0148968	0.7588	EXP 150 of 150	29.138869 ± 0.016551	0.9973	EXP 150 of 150
17D19791	12.5 %	0.0874407 ± 0.0004813	0.0460	EXP 150 of 150	5.3842117 ± 0.0174214	0.7572	EXP 150 of 150	0.0049040 ± 0.0158124	0.0008	EXP 150 of 150	3.1710370 ± 0.0165373	0.4637	EXP 150 of 150	22.783838 ± 0.017395	0.9971	EXP 150 of 150
17D19792	13.4 %	0.0706848 ± 0.0004210	0.0176	EXP 150 of 150	3.7706762 ± 0.0188197	0.5774	EXP 150 of 150	0.0051018 ± 0.0166084	0.0164	EXP 150 of 150	2.1842295 ± 0.0149459	0.3114	EXP 150 of 150	18.666124 ± 0.017339	0.9973	EXP 150 of 150
17D19794	14.6 %	0.0722861 ± 0.0004354	0.0035	EXP 150 of 150	3.3794843 ± 0.0175123	0.5721	EXP 150 of 150	0.0014053 ± 0.0189266	0.0029	EXP 150 of 150	1.7450014 ± 0.0157904	0.1677	EXP 150 of 150	18.866255 ± 0.018928	0.9966	EXP 150 of 150
17D19795	16.0 %	0.0691236 ± 0.0004275	0.0057	EXP 150 of 150	3.3485853 ± 0.0178920	0.5797	EXP 150 of 150	0.0192652 ± 0.0152673	0.0001	EXP 150 of 150	1.3290299 ± 0.0157017	0.0793	EXP 150 of 150	17.485584 ± 0.018494	0.9969	EXP 150 of 150
17D19797	17.6 %	0.0713783 ± 0.0003814	0.0102	EXP 150 of 150	4.5638137 ± 0.0194766	0.6624	EXP 148 of 150	0.0218245 ± 0.0167976	0.0026	EXP 150 of 150	1.3217520 ± 0.0150930	0.0889	EXP 150 of 150	16.739063 ± 0.017212	0.9973	EXP 150 of 150
17D19798	19.3 %	0.0443969 ± 0.0003104	0.3207	EXP 150 of 150	2.4952445 ± 0.0174256	0.4205	EXP 150 of 150	0.0107219 ± 0.0159041	0.0032	EXP 150 of 150	0.6598435 ± 0.0171795	0.0128	EXP 150 of 150	10.539415 ± 0.017315	0.9976	EXP 150 of 150
17D19800	21.0 %	0.0725549 ± 0.0004270	0.0071	EXP 150 of 150	6.8614494 ± 0.0182308	0.8252	EXP 150 of 150	0.0083239 ± 0.0145278	0.0030	EXP 149 of 150	0.6757679 ± 0.0152673	0.0054	EXP 150 of 150	13.100863 ± 0.018631	0.9972	EXP 150 of 150

Project Info		Analyst	Irradiation	X-pos	Y-pos	Z/H-pos	Project	Experiment	Nmb
17D19765	1.8 %	Dan Miggins	17-OSU-01	0.00	0.00	25.37	Arctic\O-Connor (16-22)	17D19761	01
17D19767	1.9 %	Dan Miggins	17-OSU-01	0.00	0.00	25.37	Arctic\O-Connor (16-22)	17D19761	01
17D19768	2.0 %	Dan Miggins	17-OSU-01	0.00	0.00	25.37	Arctic\O-Connor (16-22)	17D19761	01
17D19770	2.2 %	Dan Miggins	17-OSU-01	0.00	0.00	25.37	Arctic\O-Connor (16-22)	17D19761	01
17D19771	2.4 %	Dan Miggins	17-OSU-01	0.00	0.00	25.37	Arctic\O-Connor (16-22)	17D19761	01
17D19773	2.7 %	Dan Miggins	17-OSU-01	0.00	0.00	25.37	Arctic\O-Connor (16-22)	17D19761	01
17D19774	3.0 %	Dan Miggins	17-OSU-01	0.00	0.00	25.37	Arctic\O-Connor (16-22)	17D19761	01
17D19776	3.4 %	Dan Miggins	17-OSU-01	0.00	0.00	25.37	Arctic\O-Connor (16-22)	17D19761	01
17D19777	3.9 %	Dan Miggins	17-OSU-01	0.00	0.00	25.37	Arctic\O-Connor (16-22)	17D19761	01
17D19779	4.5 %	Dan Miggins	17-OSU-01	0.00	0.00	25.37	Arctic\O-Connor (16-22)	17D19761	01
17D19780	5.2 %	Dan Miggins	17-OSU-01	0.00	0.00	25.37	Arctic\O-Connor (16-22)	17D19761	01
17D19782	6.0 %	Dan Miggins	17-OSU-01	0.00	0.00	25.37	Arctic\O-Connor (16-22)	17D19761	01
17D19783	6.9 %	Dan Miggins	17-OSU-01	0.00	0.00	25.37	Arctic\O-Connor (16-22)	17D19761	01
17D19785	7.9 %	Dan Miggins	17-OSU-01	0.00	0.00	25.37	Arctic\O-Connor (16-22)	17D19761	01
17D19786	9.0 %	Dan Miggins	17-OSU-01	0.00	0.00	25.37	Arctic\O-Connor (16-22)	17D19761	01
17D19788	10.3 %	Dan Miggins	17-OSU-01	0.00	0.00	25.37	Arctic\O-Connor (16-22)	17D19761	01
17D19789	11.6 %	Dan Miggins	17-OSU-01	0.00	0.00	25.37	Arctic\O-Connor (16-22)	17D19761	01
17D19791	12.5 %	Dan Miggins	17-OSU-01	0.00	0.00	25.37	Arctic\O-Connor (16-22)	17D19761	01
17D19792	13.4 %	Dan Miggins	17-OSU-01	0.00	0.00	25.37	Arctic\O-Connor (16-22)	17D19761	01
17D19794	14.6 %	Dan Miggins	17-OSU-01	0.00	0.00	25.37	Arctic\O-Connor (16-22)	17D19761	01
17D19795	16.0 %	Dan Miggins	17-OSU-01	0.00	0.00	25.37	Arctic\O-Connor (16-22)	17D19761	01
17D19797	17.6 %	Dan Miggins	17-OSU-01	0.00	0.00	25.37	Arctic\O-Connor (16-22)	17D19761	01
17D19798	19.3 %	Dan Miggins	17-OSU-01	0.00	0.00	25.37	Arctic\O-Connor (16-22)	17D19761	01
17D19800	21.0 %	Dan Miggins	17-OSU-01	0.00	0.00	25.37	Arctic\O-Connor (16-22)	17D19761	01

**Sample Parameters**		Sample	Material	Location	Standard Name	Standard (in Ma)	%1σ	Standard Reference	Standard 40Ar/39Ar	%1σ	J	%1σ	Air 40Ar/36Ar	%1σ	MDF (lin)	%1σ	Volume Ratio	Sensitivity (mol/volt)	Day	Month	Year	Hour	Min	Resist
17D19765	1.8 %	PS59-300-18	Groundmass	Gakkel Ridge	FCT-NM (1F20-17)	28.201	0.082	Kuiper et al (2008)	9.58986	0.096	0.00163896	0.096	302.563	0.111	0.9941565	0.064	1	4.8E-14	10	JUN	2017	8	37	1
17D19767	1.9 %	PS59-300-18	Groundmass	Gakkel Ridge	FCT-NM (1F20-17)	28.201	0.082	Kuiper et al (2008)	9.58986	0.096	0.00163896	0.096	302.563	0.111	0.9941565	0.064	1	4.8E-14	10	JUN	2017	8	57	1
17D19768	2.0 %	PS59-300-18	Groundmass	Gakkel Ridge	FCT-NM (1F20-17)	28.201	0.082	Kuiper et al (2008)	9.58986	0.096	0.00163896	0.096	302.563	0.111	0.9941565	0.064	1	4.8E-14	10	JUN	2017	9	7	1
17D19770	2.2 %	PS59-300-18	Groundmass	Gakkel Ridge	FCT-NM (1F20-17)	28.201	0.082	Kuiper et al (2008)	9.58986	0.096	0.00163896	0.096	302.563	0.111	0.9941565	0.064	1	4.8E-14	10	JUN	2017	9	27	1
17D19771	2.4 %	PS59-300-18	Groundmass	Gakkel Ridge	FCT-NM (1F20-17)	28.201	0.082	Kuiper et al (2008)	9.58986	0.096	0.00163896	0.096	302.563	0.111	0.9941565	0.064	1	4.8E-14	10	JUN	2017	9	37	1
17D19773	2.7 %	PS59-300-18	Groundmass	Gakkel Ridge	FCT-NM (1F20-17)	28.201	0.082	Kuiper et al (2008)	9.58986	0.096	0.00163896	0.096	302.563	0.111	0.9941565	0.064	1	4.8E-14	10	JUN	2017	9	57	1
17D19774	3.0 %	PS59-300-18	Groundmass	Gakkel Ridge	FCT-NM (1F20-17)	28.201	0.082	Kuiper et al (2008)	9.58986	0.096	0.00163896	0.096	302.563	0.111	0.9941565	0.064	1	4.8E-14	10	JUN	2017	10	7	1
17D19776	3.4 %	PS59-300-18	Groundmass	Gakkel Ridge	FCT-NM (1F20-17)	28.201	0.082	Kuiper et al (2008)	9.58986	0.096	0.00163896	0.096	302.563	0.111	0.9941565	0.064	1	4.8E-14	10	JUN	2017	10	27	1
17D19777	3.9 %	PS59-300-18	Groundmass	Gakkel Ridge	FCT-NM (1F20-17)	28.201	0.082	Kuiper et al (2008)	9.58986	0.096	0.00163896	0.096	302.563	0.111	0.9941565	0.064	1	4.8E-14	10	JUN	2017	10	37	1
17D19779	4.5 %	PS59-300-18	Groundmass	Gakkel Ridge	FCT-NM (1F20-17)	28.201	0.082	Kuiper et al (2008)	9.58986	0.096	0.00163896	0.096	302.563	0.111	0.9941565	0.064	1	4.8E-14	10	JUN	2017	10	57	1
17D19780	5.2 %	PS59-300-18	Groundmass	Gakkel Ridge	FCT-NM (1F20-17)	28.201	0.082	Kuiper et al (2008)	9.58986	0.096	0.00163896	0.096	302.563	0.111	0.9941565	0.064	1	4.8E-14	10	JUN	2017	11	7	1
17D19782	6.0 %	PS59-300-18	Groundmass	Gakkel Ridge	FCT-NM (1F20-17)	28.201	0.082	Kuiper et al (2008)	9.58986	0.096	0.00163896	0.096	302.563	0.111	0.9941565	0.064	1	4.8E-14	10	JUN	2017	11	27	1
17D19783	6.9 %	PS59-300-18	Groundmass	Gakkel Ridge	FCT-NM (1F20-17)	28.201	0.082	Kuiper et al (2008)	9.58986	0.096	0.00163896	0.096	302.563	0.111	0.9941565	0.064	1	4.8E-14	10	JUN	2017	11	37	1
17D19785	7.9 %	PS59-300-18	Groundmass	Gakkel Ridge	FCT-NM (1F20-17)	28.201	0.082	Kuiper et al (2008)	9.58986	0.096	0.00163896	0.096	302.563	0.111	0.9941565	0.064	1	4.8E-14	10	JUN	2017	11	57	1
17D19786	9.0 %	PS59-300-18	Groundmass	Gakkel Ridge	FCT-NM (1F20-17)	28.201	0.082	Kuiper et al (2008)	9.58986	0.096	0.00163896	0.096	302.563	0.111	0.9941565	0.064	1	4.8E-14	10	JUN	2017	12	7	1
17D19788	10.3 %	PS59-300-18	Groundmass	Gakkel Ridge	FCT-NM (1F20-17)	28.201	0.082	Kuiper et al (2008)	9.58986	0.096	0.00163896	0.096	302.563	0.111	0.9941565	0.064	1	4.8E-14	10	JUN	2017	12	27	1
17D19789	11.6 %	PS59-300-18	Groundmass	Gakkel Ridge	FCT-NM (1F20-17)	28.201	0.082	Kuiper et al (2008)	9.58986	0.096	0.00163896	0.096	302.563	0.111	0.9941565	0.064	1	4.8E-14	10	JUN	2017	12	37	1
17D19791	12.5 %	PS59-300-18	Groundmass	Gakkel Ridge	FCT-NM (1F20-17)	28.201	0.082	Kuiper et al (2008)	9.58986	0.096	0.00163896	0.096	302.563	0.111	0.9941565	0.064	1	4.8E-14	10	JUN	2017	12	58	1
17D19792	13.4 %	PS59-300-18	Groundmass	Gakkel Ridge	FCT-NM (1F20-17)	28.201	0.082	Kuiper et al (2008)	9.58986	0.096	0.00163896	0.096	302.563	0.111	0.9941565	0.064	1	4.8E-14	10	JUN	2017	13	8	1
17D19794	14.6 %	PS59-300-18	Groundmass	Gakkel Ridge	FCT-NM (1F20-17)	28.201	0.082	Kuiper et al (2008)	9.58986	0.096	0.00163896	0.096	302.563	0.111	0.9941565	0.064	1	4.8E-14	10	JUN	2017	13	28	1
17D19795	16.0 %	PS59-300-18	Groundmass	Gakkel Ridge	FCT-NM (1F20-17)	28.201	0.082	Kuiper et al (2008)	9.58986	0.096	0.00163896	0.096	302.563	0.111	0.9941565	0.064	1	4.8E-14	10	JUN	2017	13	38	1
17D19797	17.6 %	PS59-300-18	Groundmass	Gakkel Ridge	FCT-NM (1F20-17)	28.201	0.082	Kuiper et al (2008)	9.58986	0.096	0.00163896	0.096	302.563	0.111	0.9941565	0.064	1	4.8E-14	10	JUN	2017	13	58	1
17D19798	19.3 %	PS59-300-18	Groundmass	Gakkel Ridge	FCT-NM (1F20-17)	28.201	0.082	Kuiper et al (2008)	9.58986	0.096	0.00163896	0.096	302.563	0.111	0.9941565	0.064	1	4.8E-14	10	JUN	2017	14	8	1
17D19800	21.0 %	PS59-300-18	Groundmass	Gakkel Ridge	FCT-NM (1F20-17)	28.201	0.082	Kuiper et al (2008)	9.58986	0.096	0.00163896	0.096	302.563	0.111	0.9941565	0.064	1	4.8E-14	10	JUN	2017	14	28	1

**Irradiation Constants**		**40/36(a)**	**%1σ**	**40/36(c)**	**%1σ**	**38/36(a)**	**%1σ**	**38/36(c)**	**%1σ**	**39/37(ca)**	**%1σ**	**38/37(ca)**	**%1σ**	**36/37(ca)**	**%1σ**	**40/39(k)**	**%1σ**	**38/39(k)**	**%1σ**	**36/38(cl)**	**%1σ**	**K/Ca**	**%1σ**	**K/Cl**	**%1σ**	**Ca/Cl**	**%1σ**
17D19765	1.8 %	295.5	0	0.018	35	0.1869	0	1.493	3	0.000643	0.92	0.00018	9.63	0.00027	0.17	0.000607	9.65	0.012077	0.09	0	0	0.43	0	0	0	0	0
17D19767	1.9 %	295.5	0	0.018	35	0.1869	0	1.493	3	0.000643	0.92	0.00018	9.63	0.00027	0.17	0.000607	9.65	0.012077	0.09	0	0	0.43	0	0	0	0	0
17D19768	2.0 %	295.5	0	0.018	35	0.1869	0	1.493	3	0.000643	0.92	0.00018	9.63	0.00027	0.17	0.000607	9.65	0.012077	0.09	0	0	0.43	0	0	0	0	0
17D19770	2.2 %	295.5	0	0.018	35	0.1869	0	1.493	3	0.000643	0.92	0.00018	9.63	0.00027	0.17	0.000607	9.65	0.012077	0.09	0	0	0.43	0	0	0	0	0
17D19771	2.4 %	295.5	0	0.018	35	0.1869	0	1.493	3	0.000643	0.92	0.00018	9.63	0.00027	0.17	0.000607	9.65	0.012077	0.09	0	0	0.43	0	0	0	0	0
17D19773	2.7 %	295.5	0	0.018	35	0.1869	0	1.493	3	0.000643	0.92	0.00018	9.63	0.00027	0.17	0.000607	9.65	0.012077	0.09	0	0	0.43	0	0	0	0	0
17D19774	3.0 %	295.5	0	0.018	35	0.1869	0	1.493	3	0.000643	0.92	0.00018	9.63	0.00027	0.17	0.000607	9.65	0.012077	0.09	0	0	0.43	0	0	0	0	0
17D19776	3.4 %	295.5	0	0.018	35	0.1869	0	1.493	3	0.000643	0.92	0.00018	9.63	0.00027	0.17	0.000607	9.65	0.012077	0.09	0	0	0.43	0	0	0	0	0
17D19777	3.9 %	295.5	0	0.018	35	0.1869	0	1.493	3	0.000643	0.92	0.00018	9.63	0.00027	0.17	0.000607	9.65	0.012077	0.09	0	0	0.43	0	0	0	0	0
17D19779	4.5 %	295.5	0	0.018	35	0.1869	0	1.493	3	0.000643	0.92	0.00018	9.63	0.00027	0.17	0.000607	9.65	0.012077	0.09	0	0	0.43	0	0	0	0	0
17D19780	5.2 %	295.5	0	0.018	35	0.1869	0	1.493	3	0.000643	0.92	0.00018	9.63	0.00027	0.17	0.000607	9.65	0.012077	0.09	0	0	0.43	0	0	0	0	0
17D19782	6.0 %	295.5	0	0.018	35	0.1869	0	1.493	3	0.000643	0.92	0.00018	9.63	0.00027	0.17	0.000607	9.65	0.012077	0.09	0	0	0.43	0	0	0	0	0
17D19783	6.9 %	295.5	0	0.018	35	0.1869	0	1.493	3	0.000643	0.92	0.00018	9.63	0.00027	0.17	0.000607	9.65	0.012077	0.09	0	0	0.43	0	0	0	0	0
17D19785	7.9 %	295.5	0	0.018	35	0.1869	0	1.493	3	0.000643	0.92	0.00018	9.63	0.00027	0.17	0.000607	9.65	0.012077	0.09	0	0	0.43	0	0	0	0	0
17D19786	9.0 %	295.5	0	0.018	35	0.1869	0	1.493	3	0.000643	0.92	0.00018	9.63	0.00027	0.17	0.000607	9.65	0.012077	0.09	0	0	0.43	0	0	0	0	0
17D19788	10.3 %	295.5	0	0.018	35	0.1869	0	1.493	3	0.000643	0.92	0.00018	9.63	0.00027	0.17	0.000607	9.65	0.012077	0.09	0	0	0.43	0	0	0	0	0
17D19789	11.6 %	295.5	0	0.018	35	0.1869	0	1.493	3	0.000643	0.92	0.00018	9.63	0.00027	0.17	0.000607	9.65	0.012077	0.09	0	0	0.43	0	0	0	0	0
17D19791	12.5 %	295.5	0	0.018	35	0.1869	0	1.493	3	0.000643	0.92	0.00018	9.63	0.00027	0.17	0.000607	9.65	0.012077	0.09	0	0	0.43	0	0	0	0	0
17D19792	13.4 %	295.5	0	0.018	35	0.1869	0	1.493	3	0.000643	0.92	0.00018	9.63	0.00027	0.17	0.000607	9.65	0.012077	0.09	0	0	0.43	0	0	0	0	0
17D19794	14.6 %	295.5	0	0.018	35	0.1869	0	1.493	3	0.000643	0.92	0.00018	9.63	0.00027	0.17	0.000607	9.65	0.012077	0.09	0	0	0.43	0	0	0	0	0
17D19795	16.0 %	295.5	0	0.018	35	0.1869	0	1.493	3	0.000643	0.92	0.00018	9.63	0.00027	0.17	0.000607	9.65	0.012077	0.09	0	0	0.43	0	0	0	0	0
17D19797	17.6 %	295.5	0	0.018	35	0.1869	0	1.493	3	0.000643	0.92	0.00018	9.63	0.00027	0.17	0.000607	9.65	0.012077	0.09	0	0	0.43	0	0	0	0	0
17D19798	19.3 %	295.5	0	0.018	35	0.1869	0	1.493	3	0.000643	0.92	0.00018	9.63	0.00027	0.17	0.000607	9.65	0.012077	0.09	0	0	0.43	0	0	0	0	0
17D19800	21.0 %	295.5	0	0.018	35	0.1869	0	1.493	3	0.000643	0.92	0.00018	9.63	0.00027	0.17	0.000607	9.65	0.012077	0.09	0	0	0.43	0	0	0	0	0

17D19761.AGE >>> PS59-300-18 >>> ARCTIC | O-CONNOR (16-22) PROJECT

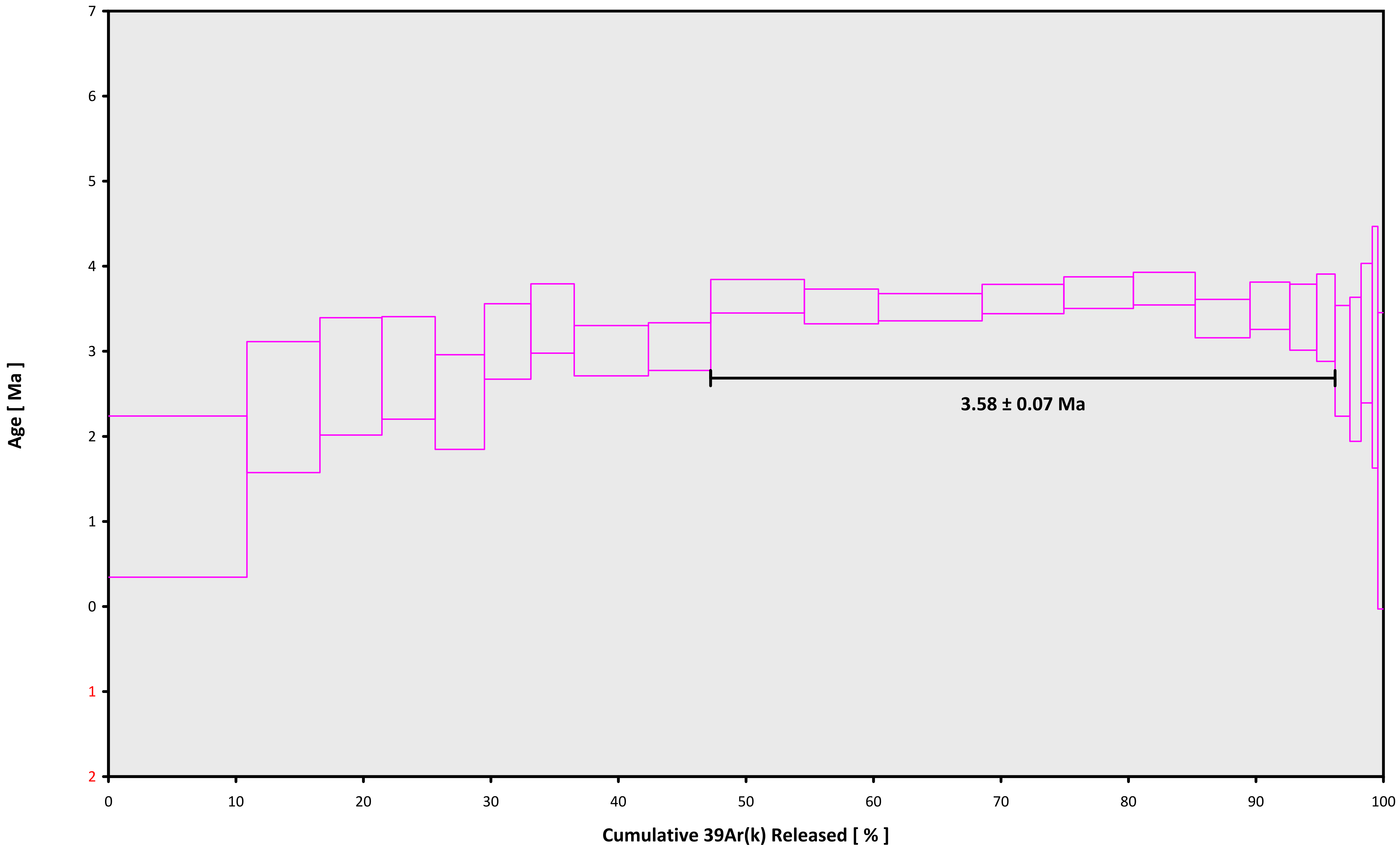

Ar-Ages in Ma

WEIGHTED PLATEAU
3.58 ± 0.07
TOTAL FUSION
3.03 ± 0.13
NORMAL ISOCHRON
3.67 ± 0.28
INVERSE ISOCHRON
3.67 ± 0.28

MSWD (PROBABILITY)
1.11 (35%)

Sample Info

Groundmass
Gakkel Ridge
Dan Miggins

IRR = 17-OSU-01 (1F20-17)
J = 0.00163896 ± 0.00000157

17D19761.AGE >>> PS59-300-18 >>> ARCTIC | O-CONNOR (16-22) PROJECT

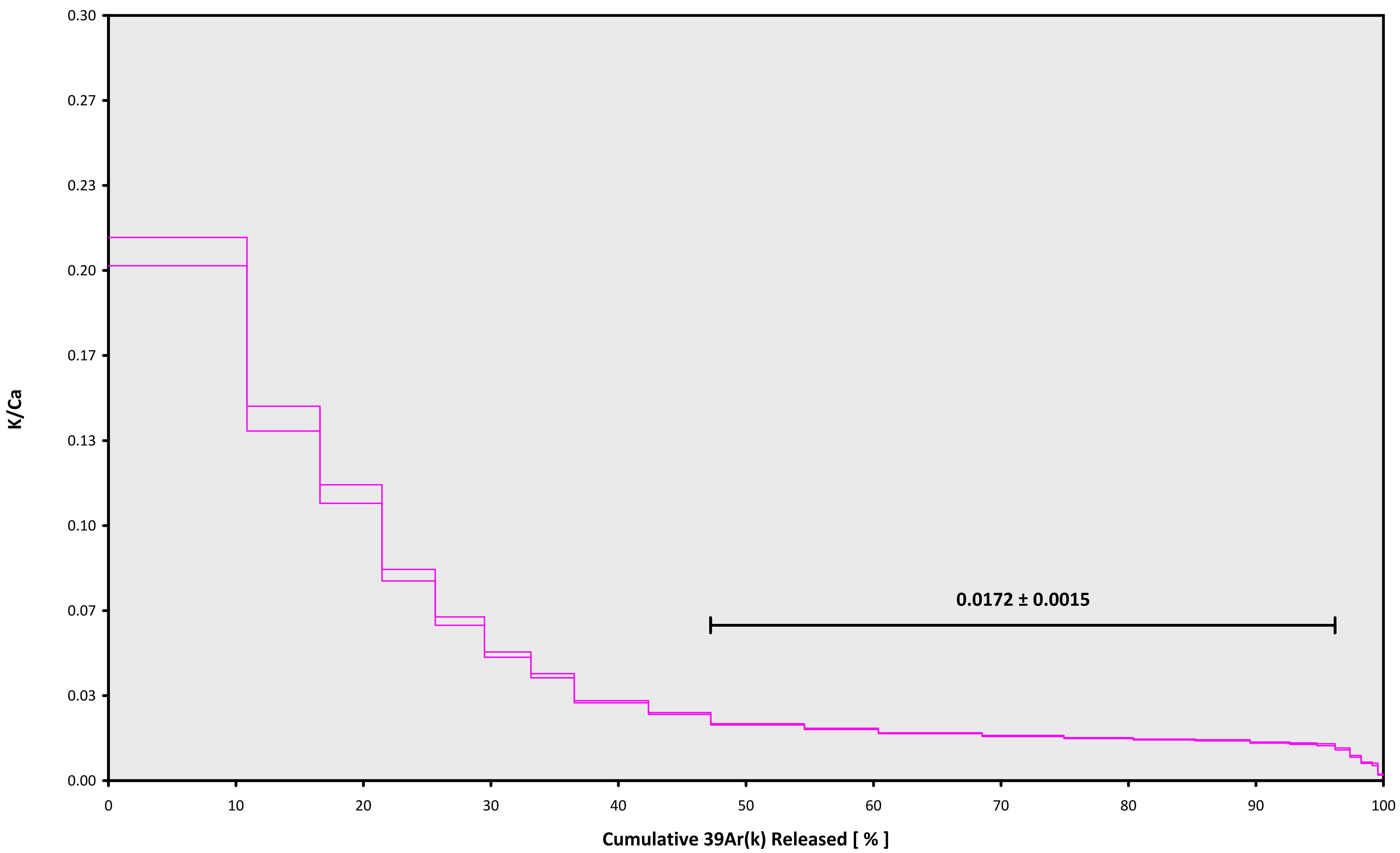

Ar-Ages in Ma

WEIGHTED PLATEAU
3.58 ± 0.07
TOTAL FUSION
3.03 ± 0.13
NORMAL ISOCHRON
3.67 ± 0.28
INVERSE ISOCHRON
3.67 ± 0.28

Sample Info

Groundmass
Gakkel Ridge
Dan Miggins

IRR = 17-OSU-01 (1F20-17)
J = 0.00163896 ± 0.00000157

17D19761.AGE >>> PS59-300-18 >>> ARCTIC | O-CONNOR (16-22) PROJECT

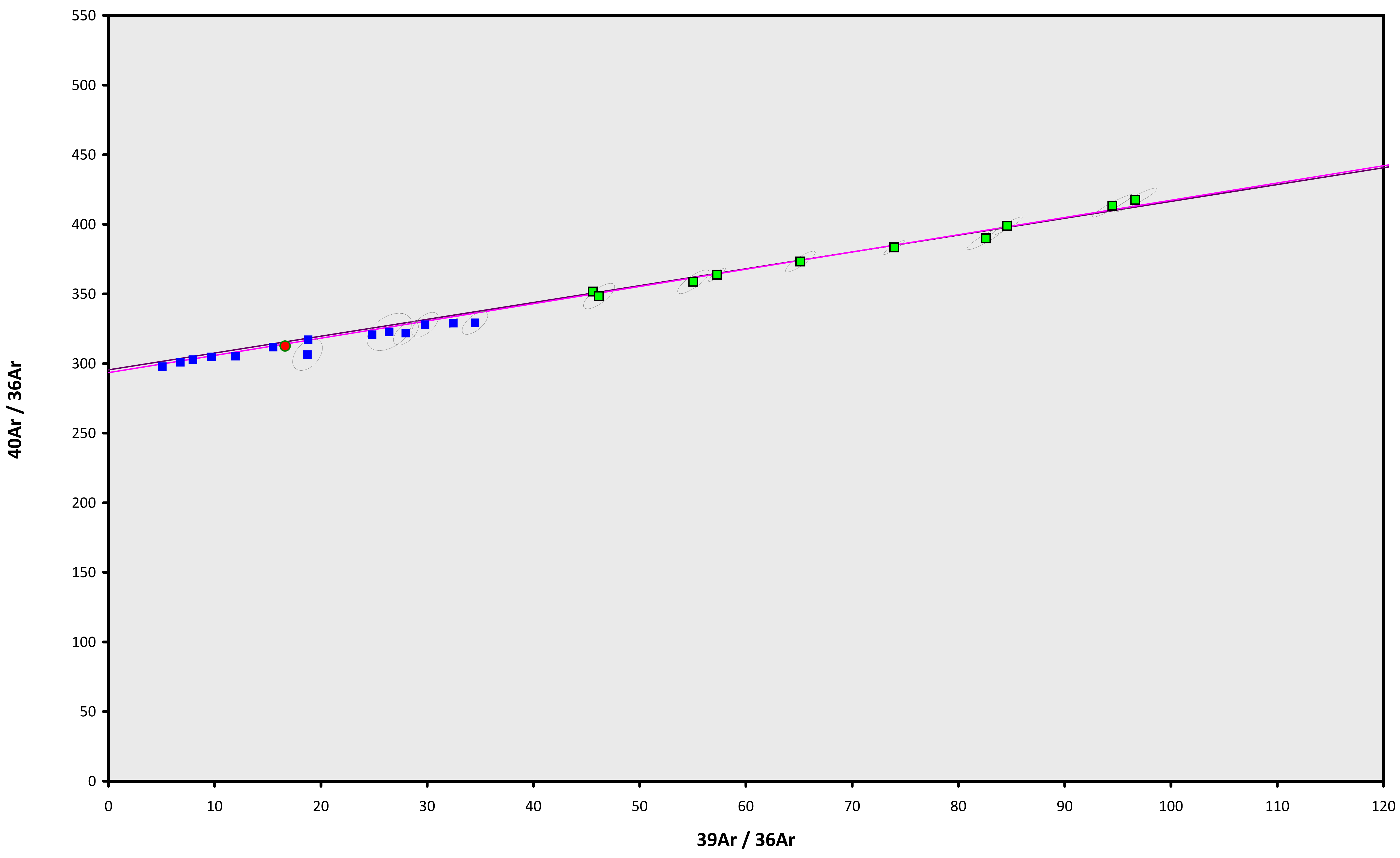

Ar-Ages in Ma

WEIGHTED PLATEAU
3.58 ± 0.07
TOTAL FUSION
3.03 ± 0.13
NORMAL ISOCHRON
3.67 ± 0.28
INVERSE ISOCHRON
3.67 ± 0.28

MSWD (PROBABILITY)
1.19 (30%)

40AR/36AR INTERCEPT
293.4 ± 6.5

Sample Info

Groundmass
Gakkel Ridge
Dan Miggins

IRR = 17-OSU-01 (1F20-17)
J = 0.00163896 ± 0.00000157

17D19761.AGE >>> PS59-300-18 >>> ARCTIC | O-CONNOR (16-22) PROJECT

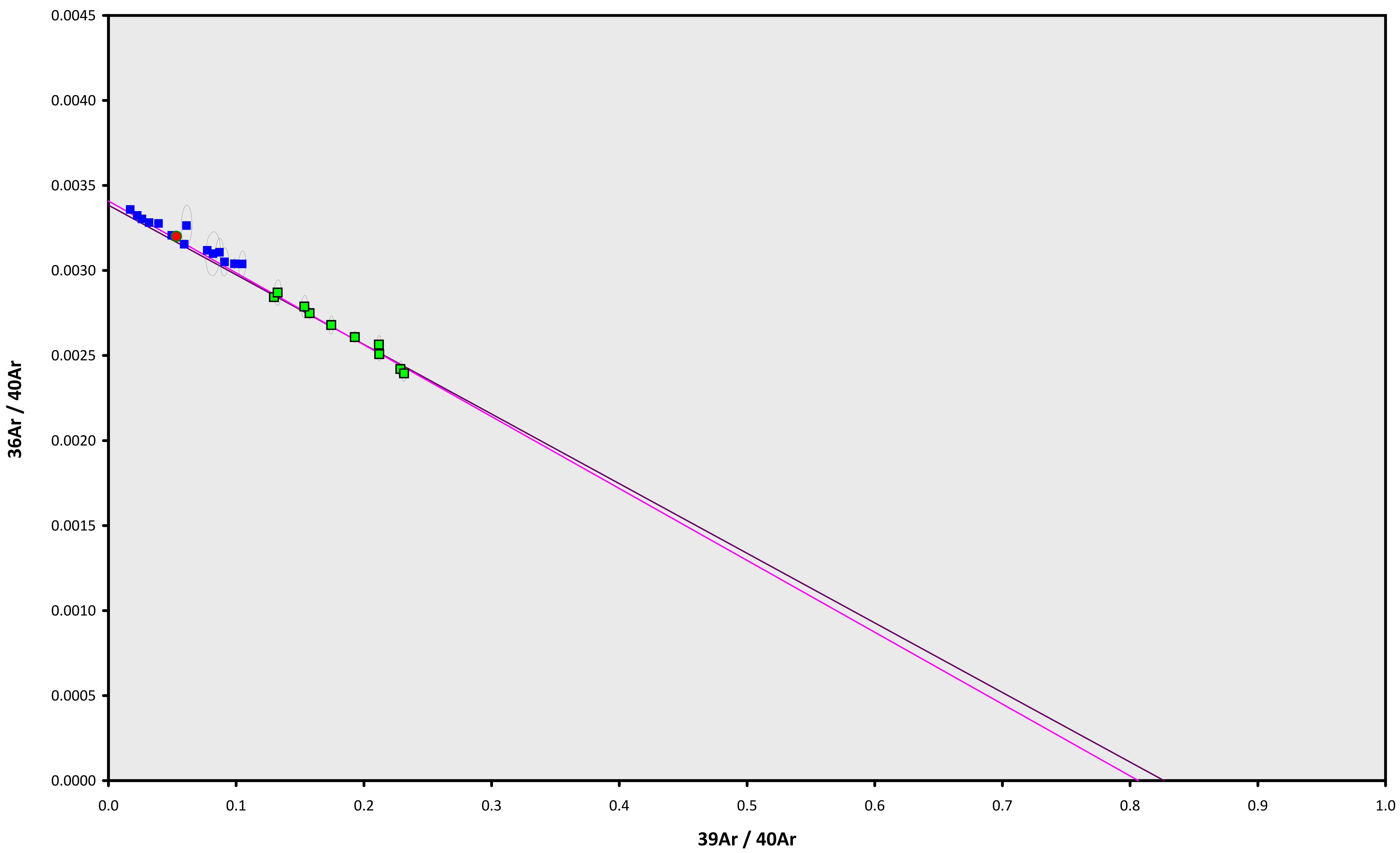

Ar-Ages in Ma

WEIGHTED PLATEAU
3.58 ± 0.07
TOTAL FUSION
3.03 ± 0.13
NORMAL ISOCHRON
3.67 ± 0.28
INVERSE ISOCHRON
3.67 ± 0.28

MSWD (PROBABILITY)
1.19 (30%)

SPREADING FACTOR
12.6%

40AR/36AR INTERCEPT
293.4 ± 6.5

Sample Info

Groundmass
Gakkel Ridge
Dan Miggins

IRR = 17-OSU-01 (1F20-17)
J = 0.00163896 ± 0.00000157

Relative Abundances			36Ar [fA]	%1σ	37Ar [fA]	%1σ	38Ar [fA]	%1σ	39Ar [fA]	%1σ	40Ar [fA]	%1σ	40(r)/39(k) ± 2σ	Age ± 2σ (Ma)	40Ar(r) (%)	39Ar(k) (%)	K/Ca ± 2σ
18D00428	1.8 %		1.3263036	0.315	154.9923	2.008	0.2722071	8.606	2.397993	0.937	389.9450	0.089	4.65369 ± 2.45467	12.87 ± 6.77	2.74	4.60	0.0064 ± 0.0003
18D00430	1.9 %		0.6286474	0.363	114.9635	2.583	0.0901700	25.656	1.764639	1.289	180.3739	0.107	2.32293 ± 1.63188	6.44 ± 4.51	2.18	3.38	0.0063 ± 0.0004
18D00431	2.0 %		0.5344817	0.369	150.6668	2.009	0.1210037	19.988	2.219097	1.043	148.4852	0.126	1.26865 ± 1.09796	3.52 ± 3.04	1.81	4.25	0.0061 ± 0.0003
18D00433	2.2 %	✓	0.3911130	0.394	161.7878	1.893	0.0761691	28.700	2.294960	0.921	104.3975	0.161	0.83284 ± 0.79191	2.31 ± 2.20	1.75	4.38	0.0058 ± 0.0002
18D00434	2.4 %	✓	0.2908885	0.451	152.6725	1.923	0.0379327	65.151	2.150295	1.020	74.1936	0.213	0.23719 ± 0.66555	0.66 ± 1.85	0.66	4.11	0.0058 ± 0.0003
18D00436	2.7 %	✓	0.1878199	0.541	112.8130	2.620	0.0140149	166.233	1.587237	1.400	46.0431	0.320	0.27174 ± 0.67515	0.75 ± 1.87	0.89	3.03	0.0058 ± 0.0003
18D00437	3.0 %	✓	0.2710328	0.462	232.1130	1.453	0.0584907	39.090	2.943155	0.760	62.3219	0.249	0.29264 ± 0.45181	0.81 ± 1.25	1.31	5.59	0.0052 ± 0.0002
18D00439	3.4 %	✓	0.2498741	0.511	226.9865	1.461	0.0482453	48.891	2.747544	0.830	56.8706	0.266	0.46309 ± 0.46816	1.29 ± 1.30	2.12	5.21	0.0049 ± 0.0002
18D00440	3.9 %	✓	0.2373469	0.500	277.7050	1.290	0.0689451	33.636	3.228348	0.661	48.7789	0.314	0.28188 ± 0.37626	0.78 ± 1.04	1.76	6.10	0.0047 ± 0.0001
18D00442	4.5 %	✓	0.2085326	0.516	239.4133	1.397	0.0429599	52.257	2.723594	0.815	42.5462	0.349	0.03079 ± 0.40698	0.09 ± 1.13	0.19	5.14	0.0046 ± 0.0002
18D00443	5.2 %	✓	0.2527366	0.460	378.0586	1.036	0.0842915	26.178	3.939441	0.546	46.3223	0.331	0.50533 ± 0.30961	1.40 ± 0.86	4.03	7.40	0.0042 ± 0.0001
18D00445	6.0 %	✓	0.2421424	0.481	404.0059	0.996	0.0994009	24.089	4.010941	0.567	41.2965	0.367	0.54412 ± 0.29899	1.51 ± 0.83	4.94	7.51	0.0040 ± 0.0001
18D00446	6.9 %	✓	0.2304357	0.501	390.6419	1.018	0.0991324	23.503	3.823844	0.584	38.0578	0.398	0.33380 ± 0.30757	0.93 ± 0.85	3.13	7.15	0.0039 ± 0.0001
18D00448	7.9 %	✓	0.2295074	0.472	392.5409	0.999	0.0705246	32.178	3.756969	0.583	37.8347	0.394	0.39809 ± 0.30356	1.10 ± 0.84	3.69	7.01	0.0038 ± 0.0001
18D00449	9.0 %	✓	0.1744797	0.591	320.1170	1.130	0.0271716	88.876	3.022434	0.702	26.7731	0.553	0.28466 ± 0.34004	0.79 ± 0.94	2.99	5.64	0.0038 ± 0.0001
18D00451	10.3 %	✓	0.1635249	0.594	271.8558	1.310	0.0766676	30.956	2.430173	0.929	27.4923	0.534	0.40086 ± 0.41291	1.11 ± 1.15	3.29	4.51	0.0036 ± 0.0001
18D00452	11.6 %		0.1683396	0.577	306.4084	1.232	0.0538509	42.249	2.493299	0.880	27.8180	0.541	1.11733 ± 0.41366	3.10 ± 1.15	9.22	4.60	0.0032 ± 0.0001
18D00454	12.5 %		0.0882274	0.830	157.8977	1.958	0.0159406	143.024	1.288404	1.768	14.8779	0.985	1.20333 ± 0.62630	3.34 ± 1.74	9.60	2.38	0.0032 ± 0.0002
18D00455	13.4 %		0.0805143	0.865	131.5122	2.348	0.0072880	328.297	0.966604	2.289	14.5655	1.003	1.45979 ± 0.82823	4.05 ± 2.29	8.84	1.77	0.0029 ± 0.0002
18D00457	14.6 %		0.0837815	0.837	127.5189	2.252	0.0140868	165.801	0.852765	2.450	17.0984	0.854	3.29148 ± 0.93927	9.11 ± 2.59	14.84	1.54	0.0026 ± 0.0002
18D00458	16.0 %		0.0892851	0.857	131.3608	2.413	0.0281680	83.337	0.843271	2.558	18.9519	0.769	4.04851 ± 1.03848	11.20 ± 2.86	16.21	1.52	0.0025 ± 0.0002
18D00460	17.6 %		0.1398532	0.609	163.9855	1.971	0.0612807	37.583	0.817685	2.809	33.3552	0.438	7.22827 ± 1.29859	19.95 ± 3.57	15.44	1.43	0.0019 ± 0.0001
18D00461	19.3 %		0.0927103	0.822	160.9561	1.842	0.0258745	89.887	0.659847	3.151	19.5456	0.747	9.01612 ± 1.49531	24.86 ± 4.09	25.67	1.11	0.0015 ± 0.0001
18D00463	21.0 %		0.0546003	1.161	99.8250	2.915	0.0183579	127.126	0.391065	5.818	11.2094	1.300	9.34311 ± 2.43814	25.75 ± 6.67	27.25	0.65	0.0014 ± 0.0002
		Σ	6.4161791	0.110	5260.7984	0.312	1.5121746	7.542	53.353602	0.203	1529.1546	0.053					

Information on Analysis and Constants Used in Calculations

Project = **O-CONNOR (16-22)**
Sample = **PS59-299-1**
Material = **Groundmass**
Location = **Gakkel Ridge**
Region = **Artic Ocean**
Analyst = **Dan Miggins**
Irradiation = **17-OSU-05 (5B33-17)**
Position = **X: 0 | Y: 0 | Z/H: 48.74548 mm**
FCT-NM Age = **28.201 ± 0.023 Ma**
FCT-NM Reference = **Kuiper et al (2008)**
FCT-NM 40Ar/39Ar Ratio = **10.23897 ± 0.00788**
FCT-NM J-value = **0.00153506 ± 0.00000118**
Air Shot 40Ar/36Ar = **302.3240 ± 0.4686**
Air Shot MDF = **0.99434975 ± 0.00069288 (LIN)**
Experiment Type = **Incremental Heating**
Extraction Method = **Bulk Laser Heating**
Heating = **77 sec**
Isolation = **3.00 min**
Instrument = **ARGUS-VI-D**
Preferred Age = **Plateau Age**
Age Classification = **Crystallization Age**
IGSN = Undefined
Rock Class = Undefined
Lithology = **Basaltic Lava**
Lat-Lon = Undefined - Undefined

Age Equations = **Min et al. (2000)**
Negative Intensities = **Allowed**
Collector Calibrations = **36Ar**
Decay 40K = **5.530 ± 0.048 E-10 1/a**
Decay 39Ar = **2.940 ± 0.016 E-07 1/h**
Decay 37Ar = **8.230 ± 0.012 E-04 1/h**
Decay 36Cl = **2.257 ± 0.015 E-06 1/a**
Decay 40K(EC,β⁺) = **0.580 ± 0.009 E-10 1/a**
Decay 40K(β⁻) = **4.950 ± 0.043 E-10 1/a**
Atmospheric 40/36(a) = **295.27 ± 1.95**
Atmospheric 38/36(a) = **0.1869**
Production 39/37(ca) = **0.0006425 ± 0.0000059**
Production 38/37(ca) = **0.0001800 ± 0.0000173**
Production 36/37(ca) = **0.0002703 ± 0.0000005**
Production 40/39(k) = **0.000607 ± 0.000059**
Production 38/39(k) = **0.012077 ± 0.000011**
Production 36/38(cl) = **262.80 ± 1.71**
Scaling Ratio K/Ca = **0.430**
Abundance Ratio 40K/K = **1.1700 ± 0.0100 E-04**
Atomic Weight K = **39.0983 ± 0.0001 g**

Results

Results	40(a)/36(a) ± 2σ	40(r)/39(k) ± 2σ	Age ± 2σ (Ma)	MSWD	39Ar(k) (%,n)	K/Ca ± 2σ
**Age Plateau**		0.35991 ± 0.10773 ± 29.93%	**1.00 ± 0.30 ± 29.93%**	0.91 54%	72.78 13	0.0042 ± 0.0003
		Full External Error	± 0.30	1.82	2σ Confidence Limit	
		Analytical Error	± 0.30	1.0000	Error Magnification	
**Total Fusion Age**		1.09041 ± 0.16476 ± 15.11%	**3.02 ± 0.46 ± 15.10%**		24	0.0041 ± 0.0000
		Full External Error	± 0.46			
		Analytical Error	± 0.46			
**Normal Isochron**	**295.18** ± 3.93 ± 1.33%	0.36165 ± 0.24010 ± 66.39%	**1.00 ± 0.67 ± 66.37%**	1.58 10%	72.78 13	
		Full External Error	± 0.67	1.85	2σ Confidence Limit	
		Analytical Error	± 0.67	1.2585	Error Magnification	
				5	Number of Iterations	
				0.0000001467	Convergence	
**Inverse Isochron** Clustered Points	**295.27** ± 3.89 ± 1.32%	0.36188 ± 0.15946 ± 44.07%	**1.00 ± 0.44 ± 44.05%**	1.56 10%	72.78 13	
		Full External Error	± 0.44	1.85	2σ Confidence Limit	
		Analytical Error	± 0.44	1.2508	Error Magnification	
				4	Number of Iterations	
				0.0000342661	Convergence	
				3%	Spreading Factor	

Notes
Mostly atmospheric

Incremental Heating			36Ar(a) [fA]	37Ar(ca) [fA]	38Ar(cl) [fA]	39Ar(k) [fA]	40Ar(r) [fA]	Age ± 2σ (Ma)	40Ar(r) (%)	39Ar(k) (%)	K/Ca ± 2σ
18D00428	1.8 %		1.2844092	154.9923	0.0000000	2.298411	10.696101	12.87 ± 6.77	2.74	4.60	0.0064 ± 0.0003
18D00430	1.9 %		0.5975728	114.9635	0.0000000	1.690775	3.927546	6.44 ± 4.51	2.18	3.38	0.0063 ± 0.0004
18D00431	2.0 %		0.4937564	150.6668	0.0000000	2.122293	2.692450	3.52 ± 3.04	1.81	4.25	0.0061 ± 0.0003
18D00433	2.2 %	✓	0.3473817	161.7878	0.0000000	2.191011	1.824755	2.31 ± 2.20	1.75	4.38	0.0058 ± 0.0002
18D00434	2.4 %	✓	0.2496212	152.6725	0.0000000	2.052203	0.486754	0.66 ± 1.85	0.66	4.11	0.0058 ± 0.0003
18D00436	2.7 %	✓	0.1573265	112.8130	0.0000000	1.514754	0.411614	0.75 ± 1.87	0.89	3.03	0.0058 ± 0.0003
18D00437	3.0 %	✓	0.2082927	232.1130	0.0000000	2.794022	0.817633	0.81 ± 1.25	1.31	5.59	0.0052 ± 0.0002
18D00439	3.4 %	✓	0.1885196	226.9865	0.0000000	2.601706	1.204836	1.29 ± 1.30	2.12	5.21	0.0049 ± 0.0002
18D00440	3.9 %	✓	0.1622832	277.7050	0.0000000	3.049922	0.859697	0.78 ± 1.04	1.76	6.10	0.0047 ± 0.0001
18D00442	4.5 %	✓	0.1438192	239.4133	0.0000000	2.569771	0.079112	0.09 ± 1.13	0.19	5.14	0.0046 ± 0.0002
18D00443	5.2 %	✓	0.1505473	378.0586	0.0000000	3.696538	1.867967	1.40 ± 0.86	4.03	7.40	0.0042 ± 0.0001
18D00445	6.0 %	✓	0.1329396	404.0059	0.0000000	3.751368	2.041181	1.51 ± 0.83	4.94	7.51	0.0040 ± 0.0001
18D00446	6.9 %	✓	0.1248452	390.6419	0.0000000	3.572857	1.192628	0.93 ± 0.85	3.13	7.15	0.0039 ± 0.0001
18D00448	7.9 %	✓	0.1234036	392.5409	0.0000000	3.504761	1.395194	1.10 ± 0.84	3.69	7.01	0.0038 ± 0.0001
18D00449	9.0 %	✓	0.0879521	320.1170	0.0000000	2.816758	0.801821	0.79 ± 0.94	2.99	5.64	0.0038 ± 0.0001
18D00451	10.3 %	✓	0.0900423	271.8558	0.0000000	2.255506	0.904145	1.11 ± 1.15	3.29	4.51	0.0036 ± 0.0001
18D00452	11.6 %		0.0855174	306.4084	0.0000000	2.296431	2.565864	3.10 ± 1.15	9.22	4.60	0.0032 ± 0.0001
18D00454	12.5 %		0.0455477	157.8977	0.0000000	1.186954	1.428299	3.34 ± 1.74	9.60	2.38	0.0032 ± 0.0002
18D00455	13.4 %		0.0449666	131.5122	0.0000000	0.882107	1.287692	4.05 ± 2.29	8.84	1.77	0.0029 ± 0.0002
18D00457	14.6 %		0.0493132	127.5189	0.0000000	0.770834	2.537184	9.11 ± 2.59	14.84	1.54	0.0026 ± 0.0002
18D00458	16.0 %		0.0537783	131.3608	0.0000000	0.758871	3.072295	11.20 ± 2.86	16.21	1.52	0.0025 ± 0.0002
18D00460	17.6 %		0.0955259	163.9855	0.0053068	0.712324	5.148874	19.95 ± 3.57	15.44	1.43	0.0019 ± 0.0001
18D00461	19.3 %		0.0492039	160.9561	0.0000000	0.556432	5.016859	24.86 ± 4.09	25.67	1.11	0.0015 ± 0.0001
18D00463	21.0 %		0.0276176	99.8250	0.0000000	0.326928	3.054523	25.75 ± 6.67	27.25	0.65	0.0014 ± 0.0002
		Σ	4.9941832	5260.7984	0.0053068	49.973539	54.491797				

Information on Analysis

Project = **O-CONNOR (16-22)**
Sample = **PS59-299-1**
Material = **Groundmass**
Location = **Gakkel Ridge**
Region = **Artic Ocean**
Analyst = **Dan Miggins**
Irradiation = **17-OSU-05 (5B33-17)**
J = **0.00153506 ± 0.00000118**
FCT-NM = **28.201 ± 0.023 Ma**

Results	40(r)/39(k) ± 2σ		Age ± 2σ (Ma)		MSWD	39Ar(k) (%,n)	K/Ca ± 2σ
**Age Plateau**	0.35991	± 0.10773<br>± 29.93%	**1.00**	**± 0.30<br>± 29.93%**	0.91<br>54%	72.78<br>13	0.0042 ± 0.0003
		Full External Error		± 0.30	1.82	2σ Confidence Limit	
		Analytical Error		± 0.30	1.0000	Error Magnification	
**Total Fusion Age**	1.09041	± 0.16476<br>± 15.11%	**3.02**	**± 0.46<br>± 15.10%**		24	0.0041 ± 0.0000
		Full External Error		± 0.46			
		Analytical Error		± 0.46			

Normal Isochron			39(k)/36(a) ± 2σ	40(a+r)/36(a) ± 2σ	r.i.
18D00428	1.8 %		1.79 ± 0.04	303.60 ± 2.08	0.3088
18D00430	1.9 %		2.83 ± 0.08	301.84 ± 2.53	0.2778
18D00431	2.0 %		4.30 ± 0.10	300.72 ± 2.71	0.3527
18D00433	2.2 %	✓	6.31 ± 0.14	300.52 ± 3.18	0.4391
18D00434	2.4 %	✓	8.22 ± 0.20	297.22 ± 3.87	0.4694
18D00436	2.7 %	✓	9.63 ± 0.32	292.65 ± 5.17	0.4543
18D00437	3.0 %	✓	13.41 ± 0.29	299.20 ± 4.70	0.6439
18D00439	3.4 %	✓	13.80 ± 0.33	301.66 ± 5.26	0.6524
18D00440	3.9 %	✓	18.79 ± 0.44	300.57 ± 6.00	0.7611
18D00442	4.5 %	✓	17.87 ± 0.47	295.82 ± 6.15	0.7045
18D00443	5.2 %	✓	24.55 ± 0.59	307.68 ± 6.78	0.8317
18D00445	6.0 %	✓	28.22 ± 0.76	310.62 ± 7.84	0.8531
18D00446	6.9 %	✓	28.62 ± 0.81	304.82 ± 8.13	0.8545
18D00448	7.9 %	✓	28.40 ± 0.79	306.58 ± 7.96	0.8482
18D00449	9.0 %	✓	32.03 ± 1.15	304.39 ± 10.45	0.8573
18D00451	10.3 %	✓	25.05 ± 0.92	305.31 ± 9.87	0.7872
18D00452	11.6 %		26.85 ± 1.03	325.27 ± 11.33	0.8213
18D00454	12.5 %		26.06 ± 1.62	326.63 ± 17.22	0.7283
18D00455	13.4 %		19.62 ± 1.37	323.91 ± 16.98	0.6402
18D00457	14.6 %		15.63 ± 1.08	346.72 ± 15.88	0.5709
18D00458	16.0 %		14.11 ± 1.01	352.40 ± 16.02	0.5639
18D00460	17.6 %		7.46 ± 0.52	349.17 ± 9.44	0.3474
18D00461	19.3 %		11.31 ± 0.99	397.23 ± 18.85	0.4882
18D00463	21.0 %		11.84 ± 1.87	405.87 ± 31.55	0.4376

Results	40(a)/36(a) ± 2σ		40(r)/39(k) ± 2σ		Age ± 2σ (Ma)		MSWD
**Normal Isochron**	**295.18**	**± 3.93**<br>**± 1.33%**	0.36165	± 0.24010<br>± 66.39%	**1.00**	**± 0.67**<br>**± 66.37%**	1.58<br>10%
					Full External Error	± 0.67	
					Analytical Error	± 0.67	

Statistics				
2σ Confidence Limit	1.85	Convergence	0.000000146748
Error Magnification	1.2585	Number of Iterations	5
Number of Data Points	13	Calculated Line	Weighted York-2

Inverse Isochron			39(k)/40(a+r) ± 2σ	36(a)/40(a+r) ± 2σ	r.i.
18D00428	1.8 %		0.0058942 ± 0.0001162	0.00329383 ± 0.00002261	0.0235
18D00430	1.9 %		0.0093738 ± 0.0002541	0.00331299 ± 0.00002777	0.0202
18D00431	2.0 %		0.0142931 ± 0.0003153	0.00332532 ± 0.00002999	0.0321
18D00433	2.2 %	✓	0.0209875 ± 0.0004128	0.00332753 ± 0.00003521	0.0498
18D00434	2.4 %	✓	0.0276606 ± 0.0006058	0.00336451 ± 0.00004379	0.0638
18D00436	2.7 %	✓	0.0328993 ± 0.0009916	0.00341701 ± 0.00006032	0.0772
18D00437	3.0 %	✓	0.0448333 ± 0.0007561	0.00334230 ± 0.00005251	0.0938
18D00439	3.4 %	✓	0.0457491 ± 0.0008431	0.00331498 ± 0.00005777	0.0881
18D00440	3.9 %	✓	0.0625278 ± 0.0009662	0.00332704 ± 0.00006640	0.1279
18D00442	4.5 %	✓	0.0604018 ± 0.0011320	0.00338043 ± 0.00007031	0.1249
18D00443	5.2 %	✓	0.0798042 ± 0.0010791	0.00325015 ± 0.00007159	0.1476
18D00445	6.0 %	✓	0.0908448 ± 0.0012979	0.00321932 ± 0.00008123	0.1494
18D00446	6.9 %	✓	0.0938850 ± 0.0014033	0.00328059 ± 0.00008745	0.1592
18D00448	7.9 %	✓	0.0926387 ± 0.0013808	0.00326183 ± 0.00008466	0.1609
18D00449	9.0 %	✓	0.1052151 ± 0.0019800	0.00328530 ± 0.00011279	0.1894
18D00451	10.3 %	✓	0.0820454 ± 0.0018733	0.00327535 ± 0.00010588	0.1547
18D00452	11.6 %		0.0825562 ± 0.0018259	0.00307433 ± 0.00010706	0.1518
18D00454	12.5 %		0.0797836 ± 0.0034541	0.00306158 ± 0.00016137	0.1700
18D00455	13.4 %		0.0605636 ± 0.0032852	0.00308731 ± 0.00016185	0.1415
18D00457	14.6 %		0.0450836 ± 0.0025728	0.00288417 ± 0.00013211	0.1116
18D00458	16.0 %		0.0400430 ± 0.0023696	0.00283769 ± 0.00012900	0.0880
18D00460	17.6 %		0.0213560 ± 0.0013965	0.00286393 ± 0.00007745	0.0434
18D00461	19.3 %		0.0284689 ± 0.0021804	0.00251743 ± 0.00011948	0.0614
18D00463	21.0 %		0.0291661 ± 0.0041445	0.00246384 ± 0.00019150	0.0612

Results	40(a)/36(a) ± 2σ	40(r)/39(k) ± 2σ	Age ± 2σ (Ma)	MSWD
**Inverse Isochron** Clustered Points	**295.27** **± 3.89** **± 1.32%**	0.36188 ± 0.15946 ± 44.07%	**1.00** **± 0.44** **± 44.05%**	1.56 10%
			Full External Error ± 0.44	
			Analytical Error ± 0.44	

Statistics					
	2σ Confidence Limit	1.85	Convergence	0.0000342661
	Error Magnification	1.2508	Number of Iterations	4
	Number of Data Points	13	Calculated Line	Weighted York-2
	Spreading Factor	3.0%		

Degassing Patterns			36Ar(a) [fA]	%1σ	36Ar(c) [fA]	%1σ	36Ar(ca) [fA]	%1σ	36Ar(cl) [fA]	%1σ	37Ar(ca) [fA]	%1σ	38Ar(a) [fA]	%1σ	38Ar(c) [fA]	%1σ	38Ar(k) [fA]	%1σ	38Ar(ca) [fA]	%1σ	38Ar(cl) [fA]	%1σ	39Ar(k) [fA]	%1σ	39Ar(ca) [fA]	%1σ	40Ar(r) [fA]	%1σ	40Ar(a) [fA]	%1σ	40Ar(c) [fA]	%1σ	40Ar(k) [fA]	%1σ
18D00428	1.8 %		1.2844092	0.33	0.0000000	0.00	0.0418944	2.02	0.0000000	0.00	154.9923	2.01	0.2400561	0.33	0.0000000	0.00	0.0277579	0.99	0.0278986	9.84	0.0000000	0.00	2.298411	0.98	0.0995826	2.21	10.696101	26.36	379.2475	0.74	0.0000000	0.00	0.0013951	9.70
18D00430	1.9 %		0.5975728	0.41	0.0000000	0.00	0.0310746	2.59	0.0000000	0.00	114.9635	2.58	0.1116864	0.41	0.0000000	0.00	0.0204195	1.35	0.0206934	9.97	0.0000000	0.00	1.690775	1.35	0.0738640	2.74	3.927546	35.10	176.4453	0.77	0.0000000	0.00	0.0010263	9.74
18D00431	2.0 %		0.4937564	0.43	0.0000000	0.00	0.0407252	2.02	0.0000000	0.00	150.6668	2.01	0.0922831	0.43	0.0000000	0.00	0.0256309	1.10	0.0271200	9.84	0.0000000	0.00	2.122293	1.10	0.0968034	2.21	2.692450	43.26	145.7915	0.79	0.0000000	0.00	0.0012882	9.71
18D00433	2.2 %	✓	0.3473817	0.50	0.0000000	0.00	0.0437312	1.90	0.0000000	0.00	161.7878	1.89	0.0649256	0.50	0.0000000	0.00	0.0264608	0.97	0.0291218	9.81	0.0000000	0.00	2.191011	0.97	0.1039487	2.10	1.824755	47.53	102.5714	0.83	0.0000000	0.00	0.0013299	9.70
18D00434	2.4 %	✓	0.2496212	0.61	0.0000000	0.00	0.0412674	1.93	0.0000000	0.00	152.6725	1.92	0.0466542	0.61	0.0000000	0.00	0.0247845	1.08	0.0274810	9.82	0.0000000	0.00	2.052203	1.07	0.0980921	2.13	0.486754	140.30	73.7056	0.90	0.0000000	0.00	0.0012457	9.71
18D00436	2.7 %	✓	0.1573265	0.82	0.0000000	0.00	0.0304933	2.63	0.0000000	0.00	112.8130	2.62	0.0294043	0.82	0.0000000	0.00	0.0182937	1.48	0.0203063	9.98	0.0000000	0.00	1.514754	1.47	0.0724823	2.78	0.411614	124.22	46.4538	1.05	0.0000000	0.00	0.0009195	9.76
18D00437	3.0 %	✓	0.2082927	0.74	0.0000000	0.00	0.0627401	1.46	0.0000000	0.00	232.1130	1.45	0.0389299	0.74	0.0000000	0.00	0.0337434	0.81	0.0417803	9.74	0.0000000	0.00	2.794022	0.81	0.1491326	1.72	0.817633	77.19	61.5026	0.99	0.0000000	0.00	0.0016960	9.68
18D00439	3.4 %	✓	0.1885196	0.83	0.0000000	0.00	0.0613545	1.47	0.0000000	0.00	226.9865	1.46	0.0352343	0.83	0.0000000	0.00	0.0314208	0.89	0.0408576	9.74	0.0000000	0.00	2.601706	0.88	0.1458388	1.73	1.204836	50.54	55.6642	1.06	0.0000000	0.00	0.0015792	9.69
18D00440	3.9 %	✓	0.1622832	0.95	0.0000000	0.00	0.0750637	1.30	0.0000000	0.00	277.7050	1.29	0.0303307	0.95	0.0000000	0.00	0.0368339	0.71	0.0499869	9.72	0.0000000	0.00	3.049922	0.71	0.1784255	1.58	0.859697	66.74	47.9174	1.15	0.0000000	0.00	0.0018513	9.68
18D00442	4.5 %	✓	0.1438192	0.98	0.0000000	0.00	0.0647134	1.41	0.0000000	0.00	239.4133	1.40	0.0268798	0.98	0.0000000	0.00	0.0310351	0.87	0.0430944	9.73	0.0000000	0.00	2.569771	0.87	0.1538231	1.67	0.079112	660.98	42.4655	1.18	0.0000000	0.00	0.0015599	9.69
18D00443	5.2 %	✓	0.1505473	1.05	0.0000000	0.00	0.1021892	1.05	0.0000000	0.00	378.0586	1.04	0.0281373	1.05	0.0000000	0.00	0.0446431	0.60	0.0680506	9.69	0.0000000	0.00	3.696538	0.59	0.2429027	1.39	1.867967	30.63	44.4521	1.24	0.0000000	0.00	0.0022438	9.67
18D00445	6.0 %	✓	0.1329396	1.21	0.0000000	0.00	0.1092028	1.01	0.0000000	0.00	404.0059	1.00	0.0248464	1.21	0.0000000	0.00	0.0453053	0.62	0.0727211	9.68	0.0000000	0.00	3.751368	0.61	0.2595738	1.36	2.041181	27.47	39.2531	1.38	0.0000000	0.00	0.0022771	9.67
18D00446	6.9 %	✓	0.1248452	1.27	0.0000000	0.00	0.1055905	1.03	0.0000000	0.00	390.6419	1.02	0.0233336	1.27	0.0000000	0.00	0.0431494	0.64	0.0703155	9.68	0.0000000	0.00	3.572857	0.63	0.2509874	1.37	1.192628	46.07	36.8630	1.43	0.0000000	0.00	0.0021687	9.67
18D00448	7.9 %	✓	0.1234036	1.24	0.0000000	0.00	0.1061038	1.01	0.0000000	0.00	392.5409	1.00	0.0230641	1.24	0.0000000	0.00	0.0423270	0.64	0.0706574	9.68	0.0000000	0.00	3.504761	0.63	0.2522076	1.36	1.395194	38.12	36.4374	1.40	0.0000000	0.00	0.0021274	9.67
18D00449	9.0 %	✓	0.0879521	1.63	0.0000000	0.00	0.0865276	1.14	0.0000000	0.00	320.1170	1.13	0.0164382	1.63	0.0000000	0.00	0.0340180	0.77	0.0576211	9.70	0.0000000	0.00	2.816758	0.76	0.2056752	1.46	0.801821	59.72	25.9696	1.75	0.0000000	0.00	0.0017098	9.68
18D00451	10.3 %	✓	0.0900423	1.53	0.0000000	0.00	0.0734826	1.32	0.0000000	0.00	271.8558	1.31	0.0168289	1.53	0.0000000	0.00	0.0272397	1.01	0.0489340	9.72	0.0000000	0.00	2.255506	1.01	0.1746673	1.60	0.904145	51.49	26.5868	1.66	0.0000000	0.00	0.0013691	9.70
18D00452	11.6 %		0.0855174	1.66	0.0000000	0.00	0.0828222	1.24	0.0000000	0.00	306.4084	1.23	0.0159832	1.66	0.0000000	0.00	0.0277340	0.97	0.0551535	9.71	0.0000000	0.00	2.296431	0.96	0.1968674	1.54	2.565864	18.49	25.2507	1.78	0.0000000	0.00	0.0013939	9.70
18D00454	12.5 %		0.0455477	2.44	0.0000000	0.00	0.0426798	1.97	0.0000000	0.00	157.8977	1.96	0.0085129	2.44	0.0000000	0.00	0.0143348	1.93	0.0284216	9.83	0.0000000	0.00	1.186954	1.93	0.1014493	2.16	1.428299	25.95	13.4489	2.53	0.0000000	0.00	0.0007205	9.84
18D00455	13.4 %		0.0449666	2.42	0.0000000	0.00	0.0355477	2.35	0.0000000	0.00	131.5122	2.35	0.0084042	2.42	0.0000000	0.00	0.0106532	2.52	0.0236722	9.91	0.0000000	0.00	0.882107	2.52	0.0844966	2.52	1.287692	28.26	13.2773	2.51	0.0000000	0.00	0.0005354	9.97
18D00457	14.6 %		0.0493132	2.13	0.0000000	0.00	0.0344684	2.26	0.0000000	0.00	127.5189	2.25	0.0092166	2.13	0.0000000	0.00	0.0093094	2.72	0.0229534	9.89	0.0000000	0.00	0.770834	2.72	0.0819309	2.43	2.537184	14.01	14.5607	2.22	0.0000000	0.00	0.0004679	10.03
18D00458	16.0 %		0.0537783	2.14	0.0000000	0.00	0.0355068	2.42	0.0000000	0.00	131.3608	2.41	0.0100512	2.14	0.0000000	0.00	0.0091649	2.86	0.0236449	9.93	0.0000000	0.00	0.758871	2.86	0.0843993	2.58	3.072295	12.50	15.8791	2.24	0.0000000	0.00	0.0004606	10.06
18D00460	17.6 %		0.0955259	1.28	0.0000000	0.00	0.0443253	1.98	0.0000021	437.51	163.9855	1.97	0.0178538	1.28	0.0000000	0.00	0.0086027	3.24	0.0295174	9.83	0.0053068	437.51	0.712324	3.24	0.1053607	2.17	5.148874	8.38	28.2059	1.44	0.0000000	0.00	0.0004324	10.18
18D00461	19.3 %		0.0492039	2.25	0.0000000	0.00	0.0435064	1.85	0.0000000	0.00	160.9561	1.84	0.0091962	2.25	0.0000000	0.00	0.0067200	3.76	0.0289721	9.80	0.0000000	0.00	0.556432	3.76	0.1034143	2.06	5.016859	7.39	14.5284	2.35	0.0000000	0.00	0.0003378	10.36
18D00463	21.0 %		0.0276176	3.66	0.0000000	0.00	0.0269827	2.92	0.0000000	0.00	99.8250	2.92	0.0051617	3.66	0.0000000	0.00	0.0039483	6.99	0.0179685	10.06	0.0000000	0.00	0.326928	6.99	0.0641376	3.06	3.054523	11.02	8.1546	3.72	0.0000000	0.00	0.0001984	11.91
		Σ	4.9941832	0.17	0.0000000	0.00	1.4219938	0.31	0.0000021	437.51	5260.7984	0.31	0.9334128	0.17	0.0000000	0.00	0.6035304	0.22	0.9469437	2.18	0.0053068	437.51	49.973539	0.22	3.3800630	0.37	54.491797	7.55	1474.6325	0.27	0.0000000	0.00	0.0303339	2.21
		Σ							6.4161791	0.15	5260.7984	0.31									2.4891938	1.25			53.353602	0.21							1529.1546	0.38

**Additional Parameters**			**40Ar/39Ar**	**1σ**	**37Ar/39Ar**	**1σ**	**36Ar/39Ar**	**1σ**	**Time (days)**	**37Ar (decay)**	**39Ar (decay)**	**40Ar (moles)**
18D00428	1.8 %		162.613037	1.529877	64.634169	1.432329	0.553089	0.005464	238.216	111.626121	1.00168583	1.872E-11
18D00430	1.9 %		102.215738	1.322353	65.148424	1.880916	0.356247	0.004772	238.230	111.656748	1.00168592	8.658E-12
18D00431	2.0 %		66.912445	0.703242	67.895566	1.536734	0.240855	0.002666	238.237	111.672064	1.00168597	7.127E-12
18D00433	2.2 %	✓	45.489897	0.425434	70.497005	1.484287	0.170423	0.001708	238.251	111.702704	1.00168607	5.011E-12
18D00434	2.4 %	✓	34.503937	0.359701	71.000703	1.545661	0.135278	0.001509	238.258	111.718027	1.00168612	3.561E-12
18D00436	2.7 %	✓	29.008349	0.416486	71.075092	2.111281	0.118331	0.001776	238.272	111.750212	1.00168622	2.210E-12
18D00437	3.0 %	✓	21.175203	0.169323	78.865354	1.293192	0.092089	0.000819	238.279	111.765541	1.00168627	2.991E-12
18D00439	3.4 %	✓	20.698702	0.180474	82.614321	1.388474	0.090945	0.000887	238.293	111.796206	1.00168637	2.730E-12
18D00440	3.9 %	✓	15.109563	0.110592	86.020785	1.247291	0.073520	0.000609	238.300	111.811542	1.00168642	2.341E-12
18D00442	4.5 %	✓	15.621337	0.138499	87.903465	1.421915	0.076565	0.000738	238.314	111.842220	1.00168652	2.042E-12
18D00443	5.2 %	✓	11.758601	0.075133	95.967580	1.123813	0.064155	0.000458	238.321	111.857562	1.00168657	2.223E-12
18D00445	6.0 %	✓	10.295971	0.069492	100.725946	1.153804	0.060370	0.000449	238.335	111.888253	1.00168667	1.982E-12
18D00446	6.9 %	✓	9.952771	0.070349	102.159469	1.199330	0.060263	0.000464	238.342	111.903601	1.00168671	1.827E-12
18D00448	7.9 %	✓	10.070538	0.070869	104.483421	1.208689	0.061088	0.000458	238.356	111.934304	1.00168681	1.816E-12
18D00449	9.0 %	✓	8.858140	0.079193	105.913665	1.409471	0.057728	0.000530	238.363	111.949659	1.00168686	1.285E-12
18D00451	10.3 %	✓	11.312900	0.121262	111.866843	1.796477	0.067289	0.000742	238.377	111.981911	1.00168697	1.320E-12
18D00452	11.6 %		11.157101	0.115250	122.892765	1.860485	0.067517	0.000711	238.384	111.997272	1.00168701	1.335E-12
18D00454	12.5 %		11.547534	0.233666	122.553012	3.232954	0.068478	0.001337	238.398	112.028001	1.00168711	7.141E-13
18D00455	13.4 %		15.068743	0.376589	136.055911	4.461642	0.083296	0.002038	238.405	112.043368	1.00168716	6.991E-13
18D00457	14.6 %		20.050488	0.520193	149.535783	4.975823	0.098247	0.002544	238.419	112.074110	1.00168726	8.207E-13
18D00458	16.0 %		22.474252	0.600342	155.775395	5.478356	0.105880	0.002856	238.426	112.089484	1.00168731	9.097E-13
18D00460	17.6 %		40.792278	1.159555	200.548537	6.880837	0.171036	0.004915	238.440	112.120238	1.00168741	1.601E-12
18D00461	19.3 %		29.621474	0.959153	243.929588	8.902407	0.140503	0.004575	238.447	112.135618	1.00168746	9.382E-13
18D00463	21.0 %		28.663672	1.708751	255.264278	16.611219	0.139619	0.008283	238.460	112.166385	1.00168755	5.380E-13

**Procedure Blanks**		36Ar ± 1σ (SE) [fA]	37Ar ± 1σ (SE) [fA]	38Ar ± 1σ (SE) [fA]	39Ar ± 1σ (SE) [fA]	40Ar ± 1σ (SE) [fA]
18D00428	1.8 %	0.0210363 ± 0.0003940	0.0088318 ± 0.0180027	0.0111082 ± 0.0161408	0.0707564 ± 0.0152272	5.9611840 ± 0.1443033
18D00430	1.9 %	0.0212576 ± 0.0003940	0.0194937 ± 0.0180027	0.0261116 ± 0.0161408	0.0581183 ± 0.0152272	6.1686639 ± 0.1443033
18D00431	2.0 %	0.0212685 ± 0.0003940	0.0204618 ± 0.0180027	0.0306400 ± 0.0161408	0.0533915 ± 0.0152272	6.2191898 ± 0.1443033
18D00433	2.2 %	0.0211335 ± 0.0003940	0.0161908 ± 0.0180027	0.0351206 ± 0.0161408	0.0464848 ± 0.0152272	6.2367295 ± 0.1443033
18D00434	2.4 %	0.0210033 ± 0.0003940	0.0118509 ± 0.0180027	0.0355673 ± 0.0161408	0.0440735 ± 0.0152272	6.2121258 ± 0.1443033
18D00436	2.7 %	0.0206335 ± 0.0003940	0.0001366 ± 0.0180027	0.0338310 ± 0.0161408	0.0406954 ± 0.0152272	6.1090238 ± 0.1443033
18D00437	3.0 %	0.0204253 ± 0.0003940	0.0059610 ± 0.0180027	0.0321391 ± 0.0161408	0.0396943 ± 0.0152272	6.0426248 ± 0.1443033
18D00439	3.4 %	0.0199765 ± 0.0003940	0.0177109 ± 0.0180027	0.0279257 ± 0.0161408	0.0384585 ± 0.0152272	5.8916144 ± 0.1443033
18D00440	3.9 %	0.0197461 ± 0.0003940	0.0229047 ± 0.0180027	0.0256810 ± 0.0161408	0.0380869 ± 0.0152272	5.8121968 ± 0.1443033
18D00442	4.5 %	0.0192957 ± 0.0003940	0.0310543 ± 0.0180027	0.0214736 ± 0.0161408	0.0375518 ± 0.0152272	5.6565608 ± 0.1443033
18D00443	5.2 %	0.0190833 ± 0.0003940	0.0337682 ± 0.0180027	0.0196807 ± 0.0161408	0.0372981 ± 0.0152272	5.5839681 ± 0.1443033
18D00445	6.0 %	0.0186998 ± 0.0003940	0.0361401 ± 0.0180027	0.0170068 ± 0.0161408	0.0366503 ± 0.0152272	5.4563369 ± 0.1443033
18D00446	6.9 %	0.0185335 ± 0.0003940	0.0357729 ± 0.0180027	0.0161884 ± 0.0161408	0.0362124 ± 0.0152272	5.4033558 ± 0.1443033
18D00448	7.9 %	0.0182611 ± 0.0003940	0.0321397 ± 0.0180027	0.0156116 ± 0.0161408	0.0350573 ± 0.0152272	5.3222462 ± 0.1443033
18D00449	9.0 %	0.0181571 ± 0.0003940	0.0290651 ± 0.0180027	0.0158087 ± 0.0161408	0.0343428 ± 0.0152272	5.2946068 ± 0.1443033
18D00451	10.3 %	0.0180111 ± 0.0003940	0.0207131 ± 0.0180027	0.0170024 ± 0.0161408	0.0326181 ± 0.0152272	5.2637387 ± 0.1443033
18D00452	11.6 %	0.0179756 ± 0.0003940	0.0162694 ± 0.0180027	0.0177798 ± 0.0161408	0.0317431 ± 0.0152272	5.2608094 ± 0.1443033
18D00454	12.5 %	0.0179655 ± 0.0003940	0.0078096 ± 0.0180027	0.0192092 ± 0.0161408	0.0300769 ± 0.0152272	5.2728806 ± 0.1443033
18D00455	13.4 %	0.0179878 ± 0.0003940	0.0044255 ± 0.0180027	0.0195990 ± 0.0161408	0.0293831 ± 0.0152272	5.2851816 ± 0.1443033
18D00457	14.6 %	0.0180757 ± 0.0003940	0.0011995 ± 0.0180027	0.0189394 ± 0.0161408	0.0285759 ± 0.0152272	5.3136059 ± 0.1443033
18D00458	16.0 %	0.0181354 ± 0.0003940	0.0022063 ± 0.0180027	0.0175208 ± 0.0161408	0.0286064 ± 0.0152272	5.3254617 ± 0.1443033
18D00460	17.6 %	0.0182683 ± 0.0003940	0.0118517 ± 0.0180027	0.0114485 ± 0.0161408	0.0299534 ± 0.0152272	5.3318312 ± 0.1443033
18D00461	19.3 %	0.0183329 ± 0.0003940	0.0215556 ± 0.0180027	0.0063186 ± 0.0161408	0.0314605 ± 0.0152272	5.3205092 ± 0.1443033
18D00463	21.0 %	0.0184337 ± 0.0003940	0.0536601 ± 0.0180027	0.0094538 ± 0.0161408	0.0366762 ± 0.0152272	5.2523023 ± 0.1443033

Intercept Values		36Ar ± 1σ (SE) [fA]	r2	Regression (type,n)	37Ar ± 1σ (SE) [fA]	r2	Regression (type,n)	38Ar ± 1σ (SE) [fA]	r2	Regression (type,n)	39Ar ± 1σ (SE) [fA]	r2	Regression (type,n)	40Ar ± 1σ (SE) [fA]	r2	Regression (type,n)
18D00428	1.8 %	1.2845300 ± 0.0016836	0.9409	EXP 150 of 150	1.3738253 ± 0.0183296	0.1530	EXP 150 of 150	0.2802398 ± 0.0166078	0.0000	EXP 150 of 150	2.4512137 ± 0.0162000	0.5424	EXP 150 of 150	395.906188 ± 0.316674	0.7783	EXP 150 of 150
18D00430	1.9 %	0.6201341 ± 0.0013043	0.7693	EXP 150 of 150	1.0316815 ± 0.0175864	0.0757	EXP 150 of 150	0.1152628 ± 0.0162054	0.0313	EXP 150 of 150	1.8098528 ± 0.0166343	0.2884	EXP 150 of 150	186.542557 ± 0.128234	0.9823	EXP 150 of 150
18D00431	2.0 %	0.5304386 ± 0.0011402	0.7085	EXP 150 of 150	1.3468155 ± 0.0172978	0.1622	EXP 150 of 150	0.1502766 ± 0.0176424	0.0012	EXP 150 of 150	2.2562601 ± 0.0171477	0.1987	EXP 149 of 150	154.704387 ± 0.120018	0.9883	EXP 150 of 150
18D00433	2.2 %	0.3937245 ± 0.0009410	0.4952	EXP 150 of 150	1.4400542 ± 0.0174062	0.2108	EXP 150 of 150	0.1104291 ± 0.0143742	0.0148	EXP 150 of 150	2.3246615 ± 0.0143570	0.0800	EXP 150 of 150	110.634221 ± 0.086142	0.9936	EXP 150 of 150
18D00434	2.4 %	0.2981161 ± 0.0008894	0.0840	EXP 150 of 150	1.3553076 ± 0.0159579	0.0958	EXP 150 of 150	0.0730715 ± 0.0183442	0.0097	EXP 150 of 150	2.1786431 ± 0.0155049	0.1081	EXP 150 of 150	80.405767 ± 0.064807	0.9968	EXP 150 of 150
18D00436	2.7 %	0.1995588 ± 0.0007249	0.0275	EXP 150 of 150	0.9925599 ± 0.0174260	0.0146	EXP 150 of 150	0.0476875 ± 0.0164332	0.0233	EXP 149 of 150	1.6163241 ± 0.0159121	0.0899	EXP 150 of 150	52.152134 ± 0.030735	0.9991	EXP 150 of 150
18D00437	3.0 %	0.2786228 ± 0.0008549	0.1626	EXP 150 of 150	2.0356721 ± 0.0187416	0.2843	EXP 150 of 150	0.0899690 ± 0.0158266	0.0022	EXP 150 of 150	2.9613251 ± 0.0160231	0.0074	EXP 150 of 150	68.364528 ± 0.057600	0.9972	EXP 150 of 150
18D00439	3.4 %	0.2580172 ± 0.0009338	0.0561	EXP 150 of 150	1.9782832 ± 0.0181873	0.2788	EXP 150 of 150	0.0756258 ± 0.0168328	0.0128	EXP 150 of 150	2.7659092 ± 0.0166572	0.0259	EXP 150 of 150	62.762220 ± 0.045238	0.9979	EXP 150 of 150
18D00440	3.9 %	0.2458529 ± 0.0008444	0.0314	EXP 150 of 150	2.4187448 ± 0.0193844	0.3215	EXP 150 of 150	0.0938471 ± 0.0162845	0.0013	EXP 150 of 150	3.2428243 ± 0.0145647	0.1622	EXP 150 of 150	54.591120 ± 0.051296	0.9976	EXP 150 of 150
18D00442	4.5 %	0.2179528 ± 0.0007597	0.0767	EXP 150 of 150	2.0733480 ± 0.0179672	0.2799	EXP 150 of 150	0.0639481 ± 0.0152359	0.0127	EXP 150 of 150	2.7412265 ± 0.0158182	0.0435	EXP 150 of 150	48.202736 ± 0.034795	0.9987	EXP 150 of 150
18D00443	5.2 %	0.2598510 ± 0.0007774	0.0796	EXP 150 of 150	3.2888469 ± 0.0178265	0.5430	EXP 150 of 150	0.1030198 ± 0.0146766	0.0033	EXP 150 of 150	3.9479277 ± 0.0147348	0.2378	EXP 150 of 150	51.906284 ± 0.052426	0.9974	EXP 150 of 150
18D00445	6.0 %	0.2493750 ± 0.0008067	0.0929	EXP 150 of 150	3.5135417 ± 0.0174783	0.5683	EXP 150 of 150	0.1152846 ± 0.0173178	0.0001	EXP 150 of 150	4.0182570 ± 0.0164097	0.2824	EXP 150 of 150	46.752875 ± 0.046157	0.9978	EXP 150 of 150
18D00446	6.9 %	0.2380565 ± 0.0008174	0.0149	EXP 150 of 150	3.3960195 ± 0.0178466	0.5192	EXP 150 of 150	0.1142008 ± 0.0164349	0.0093	EXP 150 of 150	3.8320901 ± 0.0158922	0.3637	EXP 149 of 150	43.461200 ± 0.046300	0.9979	EXP 150 of 150
18D00448	7.9 %	0.2368996 ± 0.0007241	0.1289	EXP 150 of 150	3.4153899 ± 0.0166885	0.5854	EXP 150 of 150	0.0853394 ± 0.0155852	0.0037	EXP 150 of 150	3.7645481 ± 0.0152905	0.3287	EXP 150 of 150	43.156940 ± 0.038087	0.9984	EXP 150 of 150
18D00449	9.0 %	0.1843739 ± 0.0007677	0.0071	EXP 150 of 150	2.7820085 ± 0.0172316	0.3476	EXP 150 of 150	0.0426733 ± 0.0175940	0.0202	EXP 150 of 150	3.0346706 ± 0.0144209	0.1159	EXP 150 of 150	32.067747 ± 0.033152	0.9988	EXP 150 of 150
18D00451	10.3 %	0.1737919 ± 0.0007124	0.0098	EXP 150 of 150	2.3658720 ± 0.0193112	0.3209	EXP 150 of 150	0.0928038 ± 0.0170311	0.0104	EXP 150 of 150	2.4450168 ± 0.0163662	0.0006	EXP 150 of 150	32.756042 ± 0.027407	0.9991	EXP 150 of 150
18D00452	11.6 %	0.1783431 ± 0.0007030	0.1154	EXP 149 of 150	2.6732794 ± 0.0204384	0.3453	EXP 150 of 150	0.0710223 ± 0.0156673	0.0004	EXP 150 of 150	2.5068058 ± 0.0154836	0.0000	EXP 150 of 150	33.078795 ± 0.042412	0.9981	EXP 150 of 150
18D00454	12.5 %	0.1020148 ± 0.0005240	0.6292	EXP 150 of 150	1.3777831 ± 0.0178211	0.1602	EXP 150 of 150	0.0349697 ± 0.0157349	0.0031	EXP 150 of 150	1.3090570 ± 0.0166889	0.0495	EXP 150 of 150	20.150765 ± 0.025305	0.9992	EXP 150 of 150
18D00455	13.4 %	0.0946892 ± 0.0004881	0.5550	EXP 149 of 150	1.1494687 ± 0.0185615	0.0823	EXP 150 of 150	0.0268047 ± 0.0172938	0.0095	EXP 150 of 150	0.9889170 ± 0.0158161	0.1594	EXP 150 of 150	19.850685 ± 0.022587	0.9993	EXP 150 of 150
18D00457	14.6 %	0.0978896 ± 0.0004904	0.4500	EXP 150 of 150	1.1173505 ± 0.0157734	0.2535	EXP 150 of 150	0.0328671 ± 0.0165144	0.0003	EXP 150 of 150	0.8751035 ± 0.0140664	0.1356	EXP 150 of 150	22.411959 ± 0.022407	0.9991	EXP 150 of 150
18D00458	16.0 %	0.1031923 ± 0.0005636	0.3067	EXP 150 of 150	1.1498860 ± 0.0195889	0.0868	EXP 150 of 150	0.0453705 ± 0.0166773	0.0008	EXP 150 of 150	0.8657091 ± 0.0150441	0.1347	EXP 150 of 150	24.277340 ± 0.020999	0.9992	EXP 150 of 150
18D00460	17.6 %	0.1514984 ± 0.0006000	0.0447	EXP 150 of 150	1.4259787 ± 0.0194172	0.2066	EXP 150 of 150	0.0720368 ± 0.0160620	0.0098	EXP 150 of 150	0.8416574 ± 0.0169575	0.1306	EXP 150 of 150	38.687057 ± 0.022679	0.9987	EXP 150 of 150
18D00461	19.3 %	0.1066527 ± 0.0005562	0.1945	EXP 150 of 150	1.3895198 ± 0.0159175	0.2741	EXP 150 of 150	0.0319008 ± 0.0163782	0.0003	EXP 150 of 150	0.6864808 ± 0.0139233	0.2548	EXP 149 of 150	24.866143 ± 0.021829	0.9990	EXP 150 of 150
18D00463	21.0 %	0.0704482 ± 0.0004336	0.5715	EXP 150 of 150	0.8212488 ± 0.0169947	0.0563	EXP 150 of 150	0.0086968 ± 0.0164889	0.0018	EXP 150 of 150	0.4248810 ± 0.0166781	0.1207	EXP 150 of 150	16.461669 ± 0.020332	0.9991	EXP 150 of 150

Project Info		Analyst	Irradiation	X-pos	Y-pos	Z/H-pos	Project	Experiment	Nmb
18D00428	1.8 %	Dan Miggins	17-OSU-05	0.00	0.00	48.75	Arctic\O-Connor (16-22)	18D00424	01
18D00430	1.9 %	Dan Miggins	17-OSU-05	0.00	0.00	48.75	Arctic\O-Connor (16-22)	18D00424	01
18D00431	2.0 %	Dan Miggins	17-OSU-05	0.00	0.00	48.75	Arctic\O-Connor (16-22)	18D00424	01
18D00433	2.2 %	Dan Miggins	17-OSU-05	0.00	0.00	48.75	Arctic\O-Connor (16-22)	18D00424	01
18D00434	2.4 %	Dan Miggins	17-OSU-05	0.00	0.00	48.75	Arctic\O-Connor (16-22)	18D00424	01
18D00436	2.7 %	Dan Miggins	17-OSU-05	0.00	0.00	48.75	Arctic\O-Connor (16-22)	18D00424	01
18D00437	3.0 %	Dan Miggins	17-OSU-05	0.00	0.00	48.75	Arctic\O-Connor (16-22)	18D00424	01
18D00439	3.4 %	Dan Miggins	17-OSU-05	0.00	0.00	48.75	Arctic\O-Connor (16-22)	18D00424	01
18D00440	3.9 %	Dan Miggins	17-OSU-05	0.00	0.00	48.75	Arctic\O-Connor (16-22)	18D00424	01
18D00442	4.5 %	Dan Miggins	17-OSU-05	0.00	0.00	48.75	Arctic\O-Connor (16-22)	18D00424	01
18D00443	5.2 %	Dan Miggins	17-OSU-05	0.00	0.00	48.75	Arctic\O-Connor (16-22)	18D00424	01
18D00445	6.0 %	Dan Miggins	17-OSU-05	0.00	0.00	48.75	Arctic\O-Connor (16-22)	18D00424	01
18D00446	6.9 %	Dan Miggins	17-OSU-05	0.00	0.00	48.75	Arctic\O-Connor (16-22)	18D00424	01
18D00448	7.9 %	Dan Miggins	17-OSU-05	0.00	0.00	48.75	Arctic\O-Connor (16-22)	18D00424	01
18D00449	9.0 %	Dan Miggins	17-OSU-05	0.00	0.00	48.75	Arctic\O-Connor (16-22)	18D00424	01
18D00451	10.3 %	Dan Miggins	17-OSU-05	0.00	0.00	48.75	Arctic\O-Connor (16-22)	18D00424	01
18D00452	11.6 %	Dan Miggins	17-OSU-05	0.00	0.00	48.75	Arctic\O-Connor (16-22)	18D00424	01
18D00454	12.5 %	Dan Miggins	17-OSU-05	0.00	0.00	48.75	Arctic\O-Connor (16-22)	18D00424	01
18D00455	13.4 %	Dan Miggins	17-OSU-05	0.00	0.00	48.75	Arctic\O-Connor (16-22)	18D00424	01
18D00457	14.6 %	Dan Miggins	17-OSU-05	0.00	0.00	48.75	Arctic\O-Connor (16-22)	18D00424	01
18D00458	16.0 %	Dan Miggins	17-OSU-05	0.00	0.00	48.75	Arctic\O-Connor (16-22)	18D00424	01
18D00460	17.6 %	Dan Miggins	17-OSU-05	0.00	0.00	48.75	Arctic\O-Connor (16-22)	18D00424	01
18D00461	19.3 %	Dan Miggins	17-OSU-05	0.00	0.00	48.75	Arctic\O-Connor (16-22)	18D00424	01
18D00463	21.0 %	Dan Miggins	17-OSU-05	0.00	0.00	48.75	Arctic\O-Connor (16-22)	18D00424	01

**Sample Parameters**		Sample	Material	Location	Standard Name	Standard (in Ma)	%1σ	Standard Reference	Standard 40Ar/39Ar	%1σ	J	%1σ	Air 40Ar/36Ar	%1σ	MDF (lin)	%1σ	Volume Ratio	Sensitivity (mol/volt)	Day	Month	Year	Hour	Min	Resist
18D00428	1.8 %	PS59-299-1	Groundmass	Gakkel Ridge	FCT-NM (5B33-17)	28.201	0.082	Kuiper et al (2008)	10.23897	0.077	0.00153506	0.077	302.324	0.155	0.9943498	0.070	1	4.8E-14	4	JAN	2018	17	1	1
18D00430	1.9 %	PS59-299-1	Groundmass	Gakkel Ridge	FCT-NM (5B33-17)	28.201	0.082	Kuiper et al (2008)	10.23897	0.077	0.00153506	0.077	302.324	0.155	0.9943498	0.070	1	4.8E-14	4	JAN	2018	17	21	1
18D00431	2.0 %	PS59-299-1	Groundmass	Gakkel Ridge	FCT-NM (5B33-17)	28.201	0.082	Kuiper et al (2008)	10.23897	0.077	0.00153506	0.077	302.324	0.155	0.9943498	0.070	1	4.8E-14	4	JAN	2018	17	31	1
18D00433	2.2 %	PS59-299-1	Groundmass	Gakkel Ridge	FCT-NM (5B33-17)	28.201	0.082	Kuiper et al (2008)	10.23897	0.077	0.00153506	0.077	302.324	0.155	0.9943498	0.070	1	4.8E-14	4	JAN	2018	17	51	1
18D00434	2.4 %	PS59-299-1	Groundmass	Gakkel Ridge	FCT-NM (5B33-17)	28.201	0.082	Kuiper et al (2008)	10.23897	0.077	0.00153506	0.077	302.324	0.155	0.9943498	0.070	1	4.8E-14	4	JAN	2018	18	1	1
18D00436	2.7 %	PS59-299-1	Groundmass	Gakkel Ridge	FCT-NM (5B33-17)	28.201	0.082	Kuiper et al (2008)	10.23897	0.077	0.00153506	0.077	302.324	0.155	0.9943498	0.070	1	4.8E-14	4	JAN	2018	18	22	1
18D00437	3.0 %	PS59-299-1	Groundmass	Gakkel Ridge	FCT-NM (5B33-17)	28.201	0.082	Kuiper et al (2008)	10.23897	0.077	0.00153506	0.077	302.324	0.155	0.9943498	0.070	1	4.8E-14	4	JAN	2018	18	32	1
18D00439	3.4 %	PS59-299-1	Groundmass	Gakkel Ridge	FCT-NM (5B33-17)	28.201	0.082	Kuiper et al (2008)	10.23897	0.077	0.00153506	0.077	302.324	0.155	0.9943498	0.070	1	4.8E-14	4	JAN	2018	18	52	1
18D00440	3.9 %	PS59-299-1	Groundmass	Gakkel Ridge	FCT-NM (5B33-17)	28.201	0.082	Kuiper et al (2008)	10.23897	0.077	0.00153506	0.077	302.324	0.155	0.9943498	0.070	1	4.8E-14	4	JAN	2018	19	2	1
18D00442	4.5 %	PS59-299-1	Groundmass	Gakkel Ridge	FCT-NM (5B33-17)	28.201	0.082	Kuiper et al (2008)	10.23897	0.077	0.00153506	0.077	302.324	0.155	0.9943498	0.070	1	4.8E-14	4	JAN	2018	19	22	1
18D00443	5.2 %	PS59-299-1	Groundmass	Gakkel Ridge	FCT-NM (5B33-17)	28.201	0.082	Kuiper et al (2008)	10.23897	0.077	0.00153506	0.077	302.324	0.155	0.9943498	0.070	1	4.8E-14	4	JAN	2018	19	32	1
18D00445	6.0 %	PS59-299-1	Groundmass	Gakkel Ridge	FCT-NM (5B33-17)	28.201	0.082	Kuiper et al (2008)	10.23897	0.077	0.00153506	0.077	302.324	0.155	0.9943498	0.070	1	4.8E-14	4	JAN	2018	19	52	1
18D00446	6.9 %	PS59-299-1	Groundmass	Gakkel Ridge	FCT-NM (5B33-17)	28.201	0.082	Kuiper et al (2008)	10.23897	0.077	0.00153506	0.077	302.324	0.155	0.9943498	0.070	1	4.8E-14	4	JAN	2018	20	2	1
18D00448	7.9 %	PS59-299-1	Groundmass	Gakkel Ridge	FCT-NM (5B33-17)	28.201	0.082	Kuiper et al (2008)	10.23897	0.077	0.00153506	0.077	302.324	0.155	0.9943498	0.070	1	4.8E-14	4	JAN	2018	20	22	1
18D00449	9.0 %	PS59-299-1	Groundmass	Gakkel Ridge	FCT-NM (5B33-17)	28.201	0.082	Kuiper et al (2008)	10.23897	0.077	0.00153506	0.077	302.324	0.155	0.9943498	0.070	1	4.8E-14	4	JAN	2018	20	32	1
18D00451	10.3 %	PS59-299-1	Groundmass	Gakkel Ridge	FCT-NM (5B33-17)	28.201	0.082	Kuiper et al (2008)	10.23897	0.077	0.00153506	0.077	302.324	0.155	0.9943498	0.070	1	4.8E-14	4	JAN	2018	20	53	1
18D00452	11.6 %	PS59-299-1	Groundmass	Gakkel Ridge	FCT-NM (5B33-17)	28.201	0.082	Kuiper et al (2008)	10.23897	0.077	0.00153506	0.077	302.324	0.155	0.9943498	0.070	1	4.8E-14	4	JAN	2018	21	3	1
18D00454	12.5 %	PS59-299-1	Groundmass	Gakkel Ridge	FCT-NM (5B33-17)	28.201	0.082	Kuiper et al (2008)	10.23897	0.077	0.00153506	0.077	302.324	0.155	0.9943498	0.070	1	4.8E-14	4	JAN	2018	21	23	1
18D00455	13.4 %	PS59-299-1	Groundmass	Gakkel Ridge	FCT-NM (5B33-17)	28.201	0.082	Kuiper et al (2008)	10.23897	0.077	0.00153506	0.077	302.324	0.155	0.9943498	0.070	1	4.8E-14	4	JAN	2018	21	33	1
18D00457	14.6 %	PS59-299-1	Groundmass	Gakkel Ridge	FCT-NM (5B33-17)	28.201	0.082	Kuiper et al (2008)	10.23897	0.077	0.00153506	0.077	302.324	0.155	0.9943498	0.070	1	4.8E-14	4	JAN	2018	21	53	1
18D00458	16.0 %	PS59-299-1	Groundmass	Gakkel Ridge	FCT-NM (5B33-17)	28.201	0.082	Kuiper et al (2008)	10.23897	0.077	0.00153506	0.077	302.324	0.155	0.9943498	0.070	1	4.8E-14	4	JAN	2018	22	3	1
18D00460	17.6 %	PS59-299-1	Groundmass	Gakkel Ridge	FCT-NM (5B33-17)	28.201	0.082	Kuiper et al (2008)	10.23897	0.077	0.00153506	0.077	302.324	0.155	0.9943498	0.070	1	4.8E-14	4	JAN	2018	22	23	1
18D00461	19.3 %	PS59-299-1	Groundmass	Gakkel Ridge	FCT-NM (5B33-17)	28.201	0.082	Kuiper et al (2008)	10.23897	0.077	0.00153506	0.077	302.324	0.155	0.9943498	0.070	1	4.8E-14	4	JAN	2018	22	33	1
18D00463	21.0 %	PS59-299-1	Groundmass	Gakkel Ridge	FCT-NM (5B33-17)	28.201	0.082	Kuiper et al (2008)	10.23897	0.077	0.00153506	0.077	302.324	0.155	0.9943498	0.070	1	4.8E-14	4	JAN	2018	22	53	1

Irradiation Constants		40/36(a)	%1σ	40/36(c)	%1σ	38/36(a)	%1σ	38/36(c)	%1σ	39/37(ca)	%1σ	38/37(ca)	%1σ	36/37(ca)	%1σ	40/39(k)	%1σ	38/39(k)	%1σ	36/38(cl)	%1σ	K/Ca	%1σ	K/Cl	%1σ	Ca/Cl	%1σ
18D00428	1.8 %	295.27	0.659	0.018	35	0.1869	0	1.493	3	0.000643	0.92	0.00018	9.63	0.00027	0.17	0.000607	9.65	0.012077	0.09	0	0	0.43	0	0	0	0	0
18D00430	1.9 %	295.27	0.659	0.018	35	0.1869	0	1.493	3	0.000643	0.92	0.00018	9.63	0.00027	0.17	0.000607	9.65	0.012077	0.09	0	0	0.43	0	0	0	0	0
18D00431	2.0 %	295.27	0.659	0.018	35	0.1869	0	1.493	3	0.000643	0.92	0.00018	9.63	0.00027	0.17	0.000607	9.65	0.012077	0.09	0	0	0.43	0	0	0	0	0
18D00433	2.2 %	295.27	0.659	0.018	35	0.1869	0	1.493	3	0.000643	0.92	0.00018	9.63	0.00027	0.17	0.000607	9.65	0.012077	0.09	0	0	0.43	0	0	0	0	0
18D00434	2.4 %	295.27	0.659	0.018	35	0.1869	0	1.493	3	0.000643	0.92	0.00018	9.63	0.00027	0.17	0.000607	9.65	0.012077	0.09	0	0	0.43	0	0	0	0	0
18D00436	2.7 %	295.27	0.659	0.018	35	0.1869	0	1.493	3	0.000643	0.92	0.00018	9.63	0.00027	0.17	0.000607	9.65	0.012077	0.09	0	0	0.43	0	0	0	0	0
18D00437	3.0 %	295.27	0.659	0.018	35	0.1869	0	1.493	3	0.000643	0.92	0.00018	9.63	0.00027	0.17	0.000607	9.65	0.012077	0.09	0	0	0.43	0	0	0	0	0
18D00439	3.4 %	295.27	0.659	0.018	35	0.1869	0	1.493	3	0.000643	0.92	0.00018	9.63	0.00027	0.17	0.000607	9.65	0.012077	0.09	0	0	0.43	0	0	0	0	0
18D00440	3.9 %	295.27	0.659	0.018	35	0.1869	0	1.493	3	0.000643	0.92	0.00018	9.63	0.00027	0.17	0.000607	9.65	0.012077	0.09	0	0	0.43	0	0	0	0	0
18D00442	4.5 %	295.27	0.659	0.018	35	0.1869	0	1.493	3	0.000643	0.92	0.00018	9.63	0.00027	0.17	0.000607	9.65	0.012077	0.09	0	0	0.43	0	0	0	0	0
18D00443	5.2 %	295.27	0.659	0.018	35	0.1869	0	1.493	3	0.000643	0.92	0.00018	9.63	0.00027	0.17	0.000607	9.65	0.012077	0.09	0	0	0.43	0	0	0	0	0
18D00445	6.0 %	295.27	0.659	0.018	35	0.1869	0	1.493	3	0.000643	0.92	0.00018	9.63	0.00027	0.17	0.000607	9.65	0.012077	0.09	0	0	0.43	0	0	0	0	0
18D00446	6.9 %	295.27	0.659	0.018	35	0.1869	0	1.493	3	0.000643	0.92	0.00018	9.63	0.00027	0.17	0.000607	9.65	0.012077	0.09	0	0	0.43	0	0	0	0	0
18D00448	7.9 %	295.27	0.659	0.018	35	0.1869	0	1.493	3	0.000643	0.92	0.00018	9.63	0.00027	0.17	0.000607	9.65	0.012077	0.09	0	0	0.43	0	0	0	0	0
18D00449	9.0 %	295.27	0.659	0.018	35	0.1869	0	1.493	3	0.000643	0.92	0.00018	9.63	0.00027	0.17	0.000607	9.65	0.012077	0.09	0	0	0.43	0	0	0	0	0
18D00451	10.3 %	295.27	0.659	0.018	35	0.1869	0	1.493	3	0.000643	0.92	0.00018	9.63	0.00027	0.17	0.000607	9.65	0.012077	0.09	0	0	0.43	0	0	0	0	0
18D00452	11.6 %	295.27	0.659	0.018	35	0.1869	0	1.493	3	0.000643	0.92	0.00018	9.63	0.00027	0.17	0.000607	9.65	0.012077	0.09	0	0	0.43	0	0	0	0	0
18D00454	12.5 %	295.27	0.659	0.018	35	0.1869	0	1.493	3	0.000643	0.92	0.00018	9.63	0.00027	0.17	0.000607	9.65	0.012077	0.09	0	0	0.43	0	0	0	0	0
18D00455	13.4 %	295.27	0.659	0.018	35	0.1869	0	1.493	3	0.000643	0.92	0.00018	9.63	0.00027	0.17	0.000607	9.65	0.012077	0.09	0	0	0.43	0	0	0	0	0
18D00457	14.6 %	295.27	0.659	0.018	35	0.1869	0	1.493	3	0.000643	0.92	0.00018	9.63	0.00027	0.17	0.000607	9.65	0.012077	0.09	0	0	0.43	0	0	0	0	0
18D00458	16.0 %	295.27	0.659	0.018	35	0.1869	0	1.493	3	0.000643	0.92	0.00018	9.63	0.00027	0.17	0.000607	9.65	0.012077	0.09	0	0	0.43	0	0	0	0	0
18D00460	17.6 %	295.27	0.659	0.018	35	0.1869	0	1.493	3	0.000643	0.92	0.00018	9.63	0.00027	0.17	0.000607	9.65	0.012077	0.09	0	0	0.43	0	0	0	0	0
18D00461	19.3 %	295.27	0.659	0.018	35	0.1869	0	1.493	3	0.000643	0.92	0.00018	9.63	0.00027	0.17	0.000607	9.65	0.012077	0.09	0	0	0.43	0	0	0	0	0
18D00463	21.0 %	295.27	0.659	0.018	35	0.1869	0	1.493	3	0.000643	0.92	0.00018	9.63	0.00027	0.17	0.000607	9.65	0.012077	0.09	0	0	0.43	0	0	0	0	0

18D00424.AGE >>> PS59-299-1 >>> ARCTIC | O-CONNOR (16-22) PROJECT

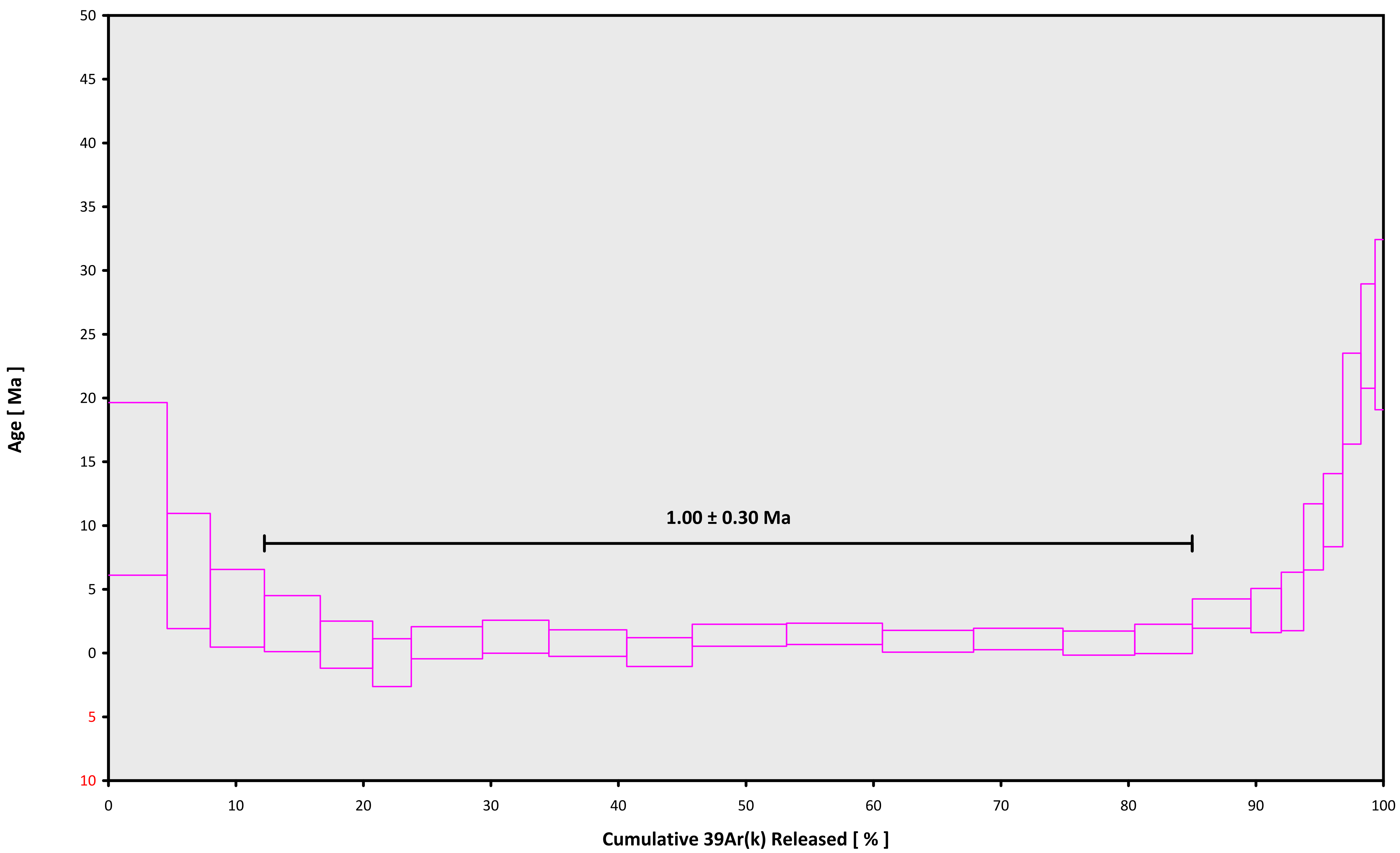

Ar-Ages in Ma

WEIGHTED PLATEAU
1.00 ± 0.30
TOTAL FUSION
3.02 ± 0.46
NORMAL ISOCHRON
1.00 ± 0.67
INVERSE ISOCHRON
1.00 ± 0.44

MSWD (PROBABILITY)
0.91 (54%)

Sample Info

Groundmass
Gakkel Ridge
Dan Miggins

IRR = 17-OSU-05 (5B33-17)
J = 0.00153506 ± 0.00000118

18D00424.AGE >>> PS59-299-1 >>> ARCTIC | O-CONNOR (16-22) PROJECT

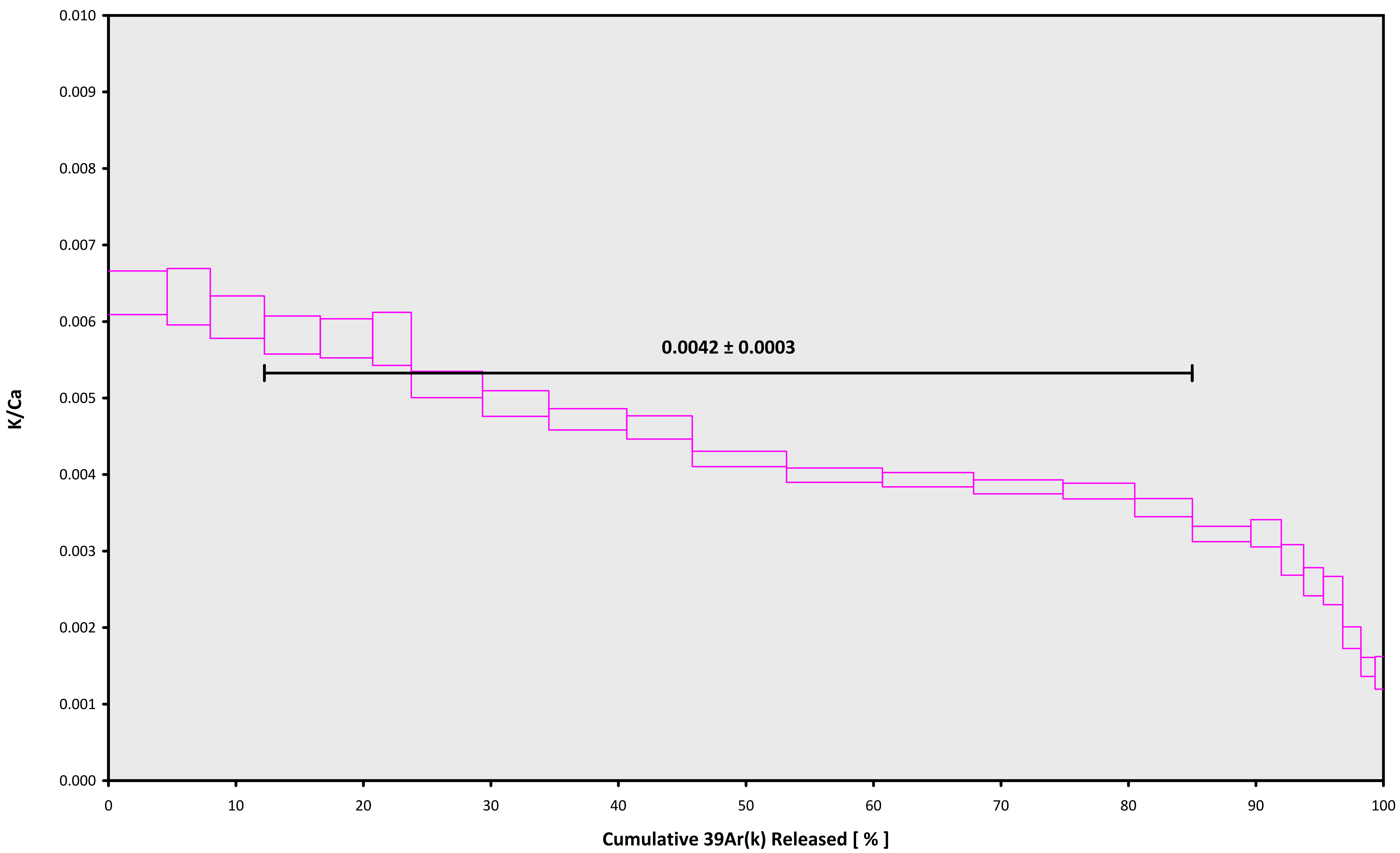

Ar-Ages in Ma

WEIGHTED PLATEAU
1.00 ± 0.30
TOTAL FUSION
3.02 ± 0.46
NORMAL ISOCHRON
1.00 ± 0.67
INVERSE ISOCHRON
1.00 ± 0.44

Sample Info

Groundmass
Gakkel Ridge
Dan Miggins

IRR = 17-OSU-05 (5B33-17)
J = 0.00153506 ± 0.00000118

18D00424.AGE >>> PS59-299-1 >>> ARCTIC | O-CONNOR (16-22) PROJECT

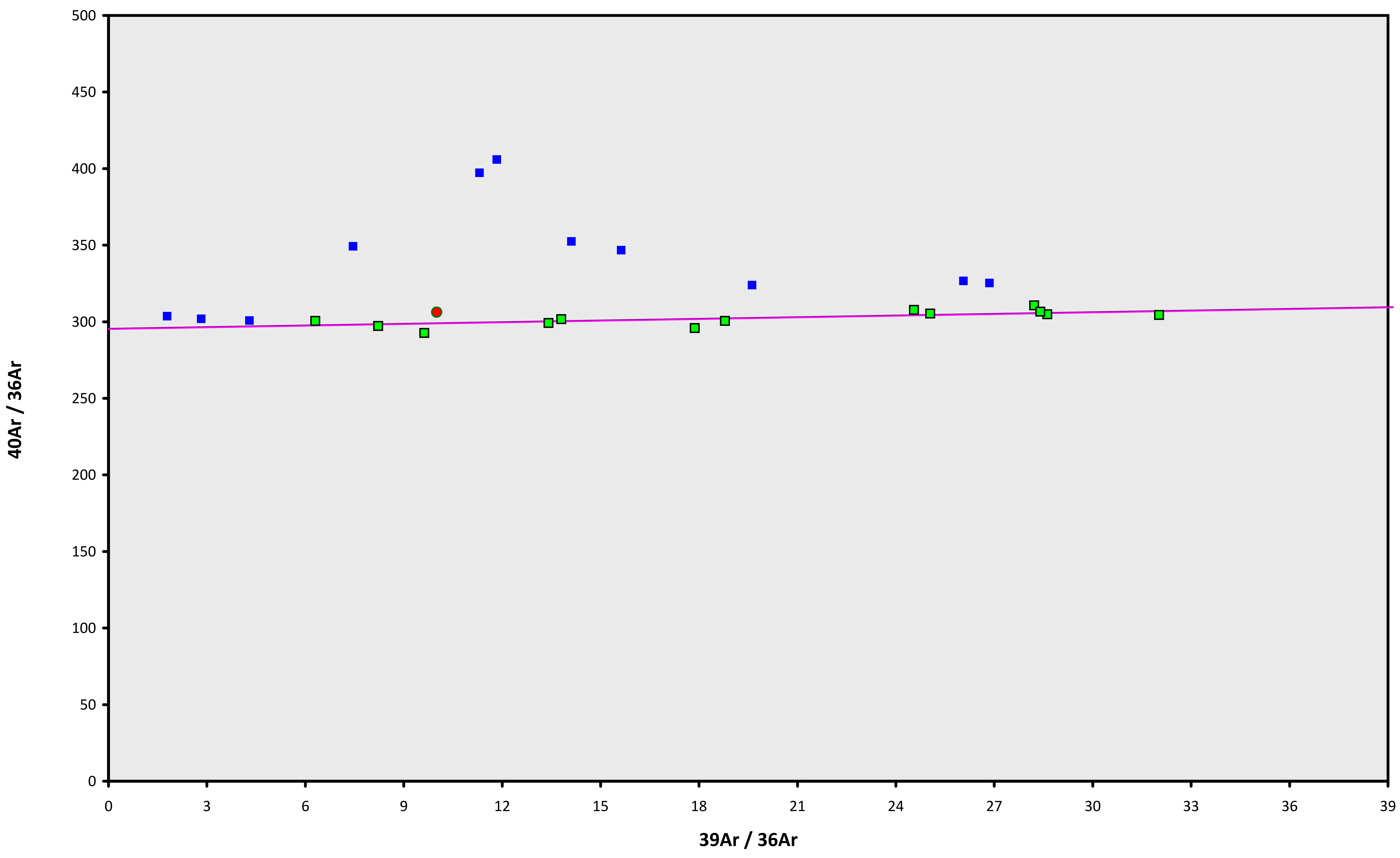

Ar-Ages in Ma

WEIGHTED PLATEAU
1.00 ± 0.30
TOTAL FUSION
3.02 ± 0.46
NORMAL ISOCHRON
1.00 ± 0.67
INVERSE ISOCHRON
1.00 ± 0.44

MSWD (PROBABILITY)
1.58 (10%)

40AR/36AR INTERCEPT
295.2 ± 3.9

Sample Info

Groundmass
Gakkel Ridge
Dan Miggins

IRR = 17-OSU-05 (5B33-17)
J = 0.00153506 ± 0.00000118

18D00424.AGE >>> PS59-299-1 >>> ARCTIC | O-CONNOR (16-22) PROJECT

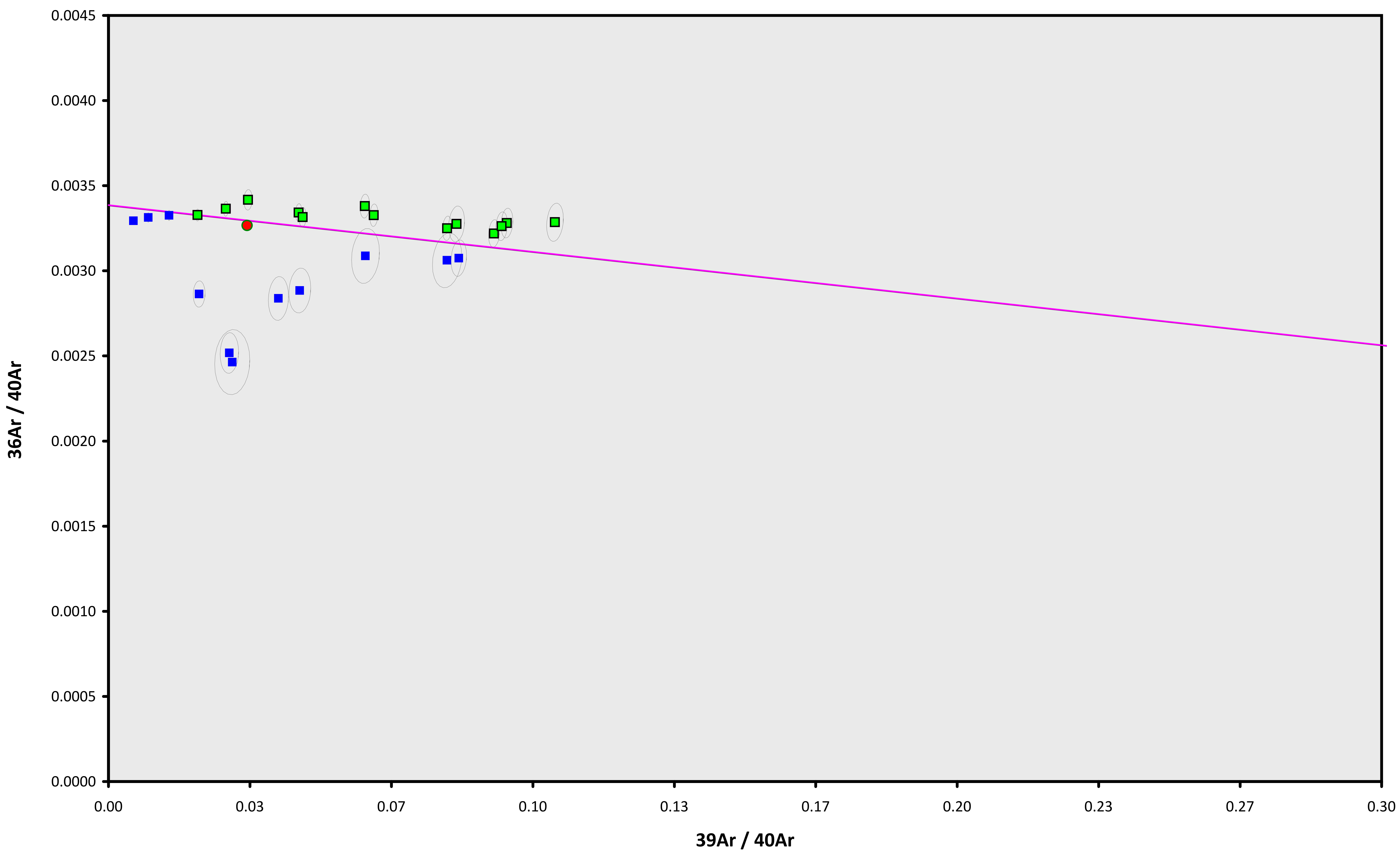

Ar-Ages in Ma

WEIGHTED PLATEAU
1.00 ± 0.30
TOTAL FUSION
3.02 ± 0.46
NORMAL ISOCHRON
1.00 ± 0.67
INVERSE ISOCHRON
1.00 ± 0.44

MSWD (PROBABILITY)
1.56 (10%)

SPREADING FACTOR
3.0%

40AR/36AR INTERCEPT
295.3 ± 3.9

Sample Info

Groundmass
Gakkel Ridge
Dan Miggins

IRR = 17-OSU-05 (5B33-17)
J = 0.00153506 ± 0.00000118

Relative Abundances			36Ar [fA]	%1σ	37Ar [fA]	%1σ	38Ar [fA]	%1σ	39Ar [fA]	%1σ	40Ar [fA]	%1σ	40(r)/39(k) ± 2σ	Age ± 2σ (Ma)	40Ar(r) (%)	39Ar(k) (%)	K/Ca ± 2σ
18D00468	1.8 %	✓	1.0477390	0.318	90.0542	3.411	0.3451181	7.149	11.03724	0.219	316.2708	0.087	0.40027 ± 0.37343	1.18 ± 1.10	1.39	4.79	0.0524 ± 0.0036
18D00470	1.9 %	✓	1.1212737	0.318	165.3697	1.868	0.4250846	5.579	20.02838	0.134	337.1474	0.089	0.45477 ± 0.21738	1.34 ± 0.64	2.69	8.68	0.0518 ± 0.0019
18D00471	2.0 %	✓	0.4058562	0.397	85.6149	3.421	0.1962163	13.281	10.50436	0.217	120.0407	0.121	0.32594 ± 0.16507	0.96 ± 0.49	2.84	4.56	0.0525 ± 0.0036
18D00473	2.2 %	✓	0.5142829	0.366	131.0822	2.330	0.2615349	9.559	15.39986	0.155	152.0251	0.094	0.39767 ± 0.13541	1.17 ± 0.40	4.01	6.68	0.0502 ± 0.0023
18D00474	2.4 %	✓	0.1784038	0.523	58.4210	4.702	0.1029794	25.031	7.02487	0.320	51.1818	0.152	0.23228 ± 0.13265	0.69 ± 0.39	3.17	3.05	0.0514 ± 0.0048
18D00476	2.7 %	✓	0.3324557	0.395	109.2158	2.714	0.1901207	13.370	11.99884	0.199	97.0064	0.094	0.39257 ± 0.11781	1.16 ± 0.35	4.83	5.20	0.0470 ± 0.0026
18D00477	3.0 %	✓	0.1481278	0.600	56.2457	5.047	0.0884413	28.552	6.17132	0.362	41.8667	0.172	0.22094 ± 0.14000	0.65 ± 0.41	3.24	2.67	0.0469 ± 0.0047
18D00479	3.4 %	✓	0.2290105	0.462	97.5786	3.152	0.1541448	16.545	10.01086	0.238	65.1658	0.115	0.34266 ± 0.10875	1.01 ± 0.32	5.23	4.34	0.0438 ± 0.0028
18D00480	3.9 %	✓	0.6854089	0.331	334.8306	1.129	0.4789425	5.307	30.88438	0.103	196.0956	0.076	0.48211 ± 0.08311	1.42 ± 0.25	7.54	13.37	0.0394 ± 0.0009
18D00482	4.5 %	✓	0.3772906	0.404	246.4503	1.368	0.3253821	7.628	20.58145	0.136	103.4223	0.092	0.42769 ± 0.07470	1.26 ± 0.22	8.45	8.90	0.0356 ± 0.0010
18D00483	5.2 %	✓	0.0886549	0.737	61.3924	4.619	0.0947505	25.753	5.03978	0.448	23.1057	0.304	0.22879 ± 0.13522	0.68 ± 0.40	4.95	2.18	0.0350 ± 0.0033
18D00485	6.0 %	✓	0.1234751	0.638	92.1949	3.038	0.0933887	27.147	7.02369	0.329	32.3500	0.215	0.33200 ± 0.10900	0.98 ± 0.32	7.15	3.04	0.0325 ± 0.0020
18D00486	6.9 %	✓	0.1532012	0.544	114.3596	2.638	0.1334184	18.891	8.19825	0.271	40.2809	0.174	0.37039 ± 0.10293	1.09 ± 0.30	7.47	3.54	0.0305 ± 0.0016
18D00488	7.9 %	✓	0.2025738	0.523	153.0200	1.998	0.1472298	16.891	10.26559	0.231	53.0823	0.135	0.38833 ± 0.09807	1.15 ± 0.29	7.44	4.43	0.0286 ± 0.0011
18D00489	9.0 %	✓	0.2279185	0.459	181.7990	1.750	0.2190916	11.230	10.68647	0.212	58.6466	0.121	0.39379 ± 0.09806	1.16 ± 0.29	7.10	4.61	0.0250 ± 0.0009
18D00491	10.3 %	✓	0.5122235	0.364	429.7827	0.924	0.3807047	6.212	19.17838	0.132	128.7258	0.065	0.42435 ± 0.10007	1.25 ± 0.30	6.23	8.24	0.0189 ± 0.0004
18D00492	11.6 %		0.2614746	0.452	216.2543	1.518	0.2032112	12.280	9.82283	0.243	64.9150	0.108	0.31409 ± 0.11822	0.93 ± 0.35	4.69	4.22	0.0193 ± 0.0006
18D00494	12.5 %		0.2763196	0.411	217.3383	1.524	0.1872568	12.913	6.69495	0.333	67.7446	0.103	0.21979 ± 0.17701	0.65 ± 0.52	2.13	2.86	0.0130 ± 0.0004
18D00495	13.4 %		0.1066785	0.672	80.4468	3.655	0.0661989	37.056	2.82947	0.770	25.9179	0.268	0.01216 ± 0.26185	0.04 ± 0.77	0.13	1.21	0.0148 ± 0.0011
18D00497	14.6 %		0.1057558	0.666	78.7088	3.720	0.0839051	28.279	2.06222	1.078	25.6955	0.269	0.02330 ± 0.35822	0.07 ± 1.06	0.18	0.88	0.0110 ± 0.0009
18D00498	16.0 %		0.1020484	0.675	84.5069	3.339	0.0748102	33.035	1.94528	1.119	23.7895	0.288	0.18316 ± 0.36799	0.54 ± 1.09	1.46	0.82	0.0096 ± 0.0007
18D00500	17.6 %		0.0725286	0.801	60.4457	4.758	0.0369284	69.121	1.33514	1.650	16.8497	0.411	0.21172 ± 0.49157	0.63 ± 1.45	1.63	0.57	0.0092 ± 0.0009
18D00501	19.3 %		0.1503163	0.544	115.2399	2.663	0.0464221	54.496	2.23382	1.010	35.4167	0.197	0.41649 ± 0.38429	1.23 ± 1.14	2.54	0.94	0.0081 ± 0.0005
18D00503	21.0 %		0.0383690	1.325	20.0959	13.580	0.0131690	187.431	0.55071	3.794	8.9822	0.775	1.96242 ± 1.07932	5.80 ± 3.20	11.75	0.23	0.0115 ± 0.0033
		Σ	7.4613868	0.099	3280.4481	0.461	4.3221118	2.822	231.50815	0.050	2085.7251	0.028					

Information on Analysis and Constants Used in Calculations

Project = **O-CONNOR (16-22)**
Sample = **PS59-271-1-14**
Material = **Groundmass**
Location = **Gakkel Ridge**
Region = **Artic Ocean**
Analyst = **Dan Miggins**
Irradiation = **17-OSU-05 (5B9-17)**
Position = **X: 0 | Y: 0 | Z/H: 13.78676 mm**
FCT-NM Age = **28.201 ± 0.023 Ma**
FCT-NM Reference = **Kuiper et al (2008)**
FCT-NM 40Ar/39Ar Ratio = **9.62643 ± 0.00789**
FCT-NM J-value = **0.00163273 ± 0.00000134**
Air Shot 40Ar/36Ar = **302.3250 ± 0.4686**
Air Shot MDF = **0.99434894 ± 0.00069288 (LIN)**
Experiment Type = **Incremental Heating**
Extraction Method = **Bulk Laser Heating**
Heating = **77 sec**
Isolation = **3.00 min**
Instrument = **ARGUS-VI-D**
Preferred Age = **Plateau Age**
Age Classification = **Crystallization Age**
IGSN = Undefined
Rock Class = Undefined
Lithology = **Basaltic Lava**
Lat-Lon = Undefined - Undefined

Age Equations = **Min et al. (2000)**
Negative Intensities = **Allowed**
Collector Calibrations = **36Ar**
Decay 40K = **5.530 ± 0.048 E-10 1/a**
Decay 39Ar = **2.940 ± 0.016 E-07 1/h**
Decay 37Ar = **8.230 ± 0.012 E-04 1/h**
Decay 36Cl = **2.257 ± 0.015 E-06 1/a**
Decay 40K(EC,β⁺) = **0.580 ± 0.009 E-10 1/a**
Decay 40K(β⁻) = **4.950 ± 0.043 E-10 1/a**
Atmospheric 40/36(a) = **304.74 ± 1.70**
Atmospheric 38/36(a) = **0.1869**
Production 39/37(ca) = **0.0006425 ± 0.0000059**
Production 38/37(ca) = **0.0001800 ± 0.0000173**
Production 36/37(ca) = **0.0002703 ± 0.0000005**
Production 40/39(k) = **0.000607 ± 0.000059**
Production 38/39(k) = **0.012077 ± 0.000011**
Production 36/38(cl) = **262.80 ± 1.71**
Scaling Ratio K/Ca = **0.430**
Abundance Ratio 40K/K = **1.1700 ± 0.0100 E-04**
Atomic Weight K = **39.0983 ± 0.0001 g**

Results

Results	40(a)/36(a) ± 2σ	40(r)/39(k) ± 2σ	Age ± 2σ (Ma)	MSWD	39Ar(k) (%,n)	K/Ca ± 2σ
**Age Plateau** / Error Mean		0.37903 ± 0.03725 ± 9.83%	**1.12** ± 0.11 ± 9.83%	1.73 / 4%	88.27 / 16	0.0258 ± 0.0050
		Full External Error	± 0.11	1.73	2σ Confidence Limit	
		Analytical Error	± 0.11	1.3164	Error Magnification	
**Total Fusion Age**		0.35760 ± 0.03632 ± 10.16%	**1.06** ± 0.11 ± 10.15%		24	0.0301 ± 0.0003
		Full External Error	± 0.11			
		Analytical Error	± 0.11			
**Normal Isochron** / Error Chron	305.10 ± 3.40 ± 1.12%	0.38241 ± 0.08343 ± 21.82%	**1.13** ± 0.25 ± 21.81%	3.28 / 0%	88.27 / 16	
		Full External Error	± 0.25	1.76	2σ Confidence Limit	
		Analytical Error	± 0.25	1.8111	Error Magnification	
				5	Number of Iterations	
				0.0000002196	Convergence	
**Inverse Isochron** / Error Chron	305.05 ± 3.37 ± 1.10%	0.38599 ± 0.07616 ± 19.73%	**1.14** ± 0.22 ± 19.72%	3.21 / 0%	88.27 / 16	
		Full External Error	± 0.23	1.76	2σ Confidence Limit	
		Analytical Error	± 0.22	1.7915	Error Magnification	
				3	Number of Iterations	
				0.0002677559	Convergence	
				7%	Spreading Factor	

Notes

Excess Initial 40Ar/36Ar = 304.74 ± 0.56 (%SD). Mostly atmospheric

Incremental Heating			36Ar(a) [fA]	37Ar(ca) [fA]	38Ar(cl) [fA]	39Ar(k) [fA]	40Ar(r) [fA]	Age ± 2σ (Ma)	40Ar(r) (%)	39Ar(k) (%)	K/Ca ± 2σ
18D00468	1.8 %	✓	1.0233954	90.0542	0.0050378	10.97938	4.394671	1.18 ± 1.10	1.39	4.79	0.0524 ± 0.0036
18D00470	1.9 %	✓	1.0765742	165.3697	0.0000000	19.92213	9.060067	1.34 ± 0.64	2.69	8.68	0.0518 ± 0.0019
18D00471	2.0 %	✓	0.3827145	85.6149	0.0000000	10.44935	3.405904	0.96 ± 0.49	2.84	4.56	0.0525 ± 0.0036
18D00473	2.2 %	✓	0.4788514	131.0822	0.0000000	15.31564	6.090624	1.17 ± 0.40	4.01	6.68	0.0502 ± 0.0023
18D00474	2.4 %	✓	0.1626126	58.4210	0.0000000	6.98734	1.623041	0.69 ± 0.39	3.17	3.05	0.0514 ± 0.0048
18D00476	2.7 %	✓	0.3029347	109.2158	0.0000000	11.92867	4.682851	1.16 ± 0.35	4.83	5.20	0.0470 ± 0.0026
18D00477	3.0 %	✓	0.1329246	56.2457	0.0000000	6.13518	1.355527	0.65 ± 0.41	3.24	2.67	0.0469 ± 0.0047
18D00479	3.4 %	✓	0.2026350	97.5786	0.0000000	9.94817	3.408811	1.01 ± 0.32	5.23	4.34	0.0438 ± 0.0028
18D00480	3.9 %	✓	0.5949042	334.8306	0.0000000	30.66925	14.785903	1.42 ± 0.25	7.54	13.37	0.0394 ± 0.0009
18D00482	4.5 %	✓	0.3106751	246.4503	0.0000000	20.42311	8.734820	1.26 ± 0.22	8.45	8.90	0.0356 ± 0.0010
18D00483	5.2 %	✓	0.0720567	61.3924	0.0098435	5.00033	1.144051	0.68 ± 0.40	4.95	2.18	0.0350 ± 0.0033
18D00485	6.0 %	✓	0.0985548	92.1949	0.0000000	6.96446	2.312211	0.98 ± 0.32	7.15	3.04	0.0325 ± 0.0020
18D00486	6.9 %	✓	0.1222898	114.3596	0.0000000	8.12478	3.009361	1.09 ± 0.30	7.47	3.54	0.0305 ± 0.0016
18D00488	7.9 %	✓	0.1612125	153.0200	0.0000000	10.16728	3.948225	1.15 ± 0.29	7.44	4.43	0.0286 ± 0.0011
18D00489	9.0 %	✓	0.1787685	181.7990	0.0253062	10.56966	4.162273	1.16 ± 0.29	7.10	4.61	0.0250 ± 0.0009
18D00491	10.3 %	✓	0.3960528	429.7827	0.0010391	18.90225	8.021183	1.25 ± 0.30	6.23	8.24	0.0189 ± 0.0004
18D00492	11.6 %		0.2030174	216.2543	0.0093892	9.68388	3.041572	0.93 ± 0.35	4.69	4.22	0.0193 ± 0.0006
18D00494	12.5 %		0.2175621	217.3383	0.0283050	6.55531	1.440777	0.65 ± 0.52	2.13	2.86	0.0130 ± 0.0004
18D00495	13.4 %		0.0849328	80.4468	0.0022972	2.77779	0.033772	0.04 ± 0.77	0.13	1.21	0.0148 ± 0.0011
18D00497	14.6 %		0.0844693	78.7088	0.0296556	2.01165	0.046871	0.07 ± 1.06	0.18	0.88	0.0110 ± 0.0009
18D00498	16.0 %		0.0791976	84.5069	0.0219594	1.89099	0.346362	0.54 ± 1.09	1.46	0.82	0.0096 ± 0.0007
18D00500	17.6 %		0.0561901	60.4457	0.0000000	1.29631	0.274459	0.63 ± 1.45	1.63	0.57	0.0092 ± 0.0009
18D00501	19.3 %		0.1191670	115.2399	0.0000000	2.15977	0.899515	1.23 ± 1.14	2.54	0.94	0.0081 ± 0.0005
18D00503	21.0 %		0.0329370	20.0959	0.0000000	0.53780	1.055382	5.80 ± 3.20	11.75	0.23	0.0115 ± 0.0033
		Σ	6.5746301	3280.4481	0.1328329	229.40046	82.033055				

Information on Analysis
Project = **O-CONNOR (16-22)**
Sample = **PS59-271-1-14**
Material = **Groundmass**
Location = **Gakkel Ridge**
Region = **Artic Ocean**
Analyst = **Dan Miggins**
Irradiation = **17-OSU-05 (5B9-17)**
J = **0.00163273 ± 0.00000134**
FCT-NM = **28.201 ± 0.023 Ma**

Results	40(r)/39(k) ± 2σ		Age ± 2σ (Ma)		MSWD	39Ar(k) (%,n)	K/Ca ± 2σ
**Age Plateau**	0.37903	± 0.03725	**1.12**	± 0.11	1.73	88.27	0.0258 ± 0.0050
Error Mean		± 9.83%		± 9.83%	4%	16	
			Full External Error	± 0.11	1.73	2σ Confidence Limit	
			Analytical Error	± 0.11	1.3164	Error Magnification	
**Total Fusion Age**	0.35760	± 0.03632	**1.06**	± 0.11		24	0.0301 ± 0.0003
		± 10.16%		± 10.15%			
			Full External Error	± 0.11			
			Analytical Error	± 0.11			

Normal Isochron			39(k)/36(a) ± 2σ	40(a+r)/36(a) ± 2σ	r.i.
18D00468	1.8 %	✓	10.73 ± 0.09	309.03 ± 2.15	0.8085
18D00470	1.9 %	✓	18.51 ± 0.14	313.16 ± 2.20	0.8991
18D00471	2.0 %	✓	27.30 ± 0.28	313.64 ± 3.04	0.8773
18D00473	2.2 %	✓	31.98 ± 0.29	317.46 ± 2.79	0.9178
18D00474	2.4 %	✓	42.97 ± 0.69	314.72 ± 4.72	0.8966
18D00476	2.7 %	✓	39.38 ± 0.43	320.20 ± 3.31	0.9145
18D00477	3.0 %	✓	46.16 ± 0.88	314.94 ± 5.67	0.9071
18D00479	3.4 %	✓	49.09 ± 0.69	321.56 ± 4.34	0.9263
18D00480	3.9 %	✓	51.55 ± 0.45	329.59 ± 2.81	0.9548
18D00482	4.5 %	✓	65.74 ± 0.77	332.86 ± 3.86	0.9600
18D00483	5.2 %	✓	69.39 ± 2.04	320.62 ± 9.18	0.9296
18D00485	6.0 %	✓	70.67 ± 1.64	328.20 ± 7.42	0.9402
18D00486	6.9 %	✓	66.44 ± 1.32	329.35 ± 6.39	0.9454
18D00488	7.9 %	✓	63.07 ± 1.09	329.23 ± 5.57	0.9505
18D00489	9.0 %	✓	59.12 ± 0.93	328.02 ± 5.04	0.9499
18D00491	10.3 %	✓	47.73 ± 0.54	324.99 ± 3.57	0.9638
18D00492	11.6 %		47.70 ± 0.73	319.72 ± 4.71	0.9367
18D00494	12.5 %		30.13 ± 0.45	311.36 ± 4.20	0.8789
18D00495	13.4 %		32.71 ± 0.97	305.14 ± 7.87	0.8297
18D00497	14.6 %		23.82 ± 0.80	304.19 ± 7.81	0.7327
18D00498	16.0 %		23.88 ± 0.83	300.37 ± 7.99	0.7294
18D00500	17.6 %		23.07 ± 1.12	299.86 ± 10.65	0.6924
18D00501	19.3 %		18.12 ± 0.52	297.19 ± 5.93	0.6687
18D00503	21.0 %		16.33 ± 1.55	272.70 ± 15.43	0.5503

Results	40(a)/36(a) ± 2σ		40(r)/39(k) ± 2σ		Age ± 2σ (Ma)		MSWD
**Normal Isochron**	**305.10**	**± 3.40**	0.38241	± 0.08343	**1.13**	**± 0.25**	3.28
Error Chron		**± 1.12%**		± 21.82%		**± 21.81%**	0%
					Full External Error	± 0.25	
					Analytical Error	± 0.25	

Statistics				
	2σ Confidence Limit	1.76	Convergence	0.000000219618
	Error Magnification	1.8111	Number of Iterations	5
	Number of Data Points	16	Calculated Line	Weighted York-2

Inverse Isochron			39(k)/40(a+r) ± 2σ		36(a)/40(a+r) ± 2σ		r.i.
18D00468	1.8 %	✓	0.0347158	± 0.0001651	0.00323589	± 0.00002246	0.0922
18D00470	1.9 %	✓	0.0590924	± 0.0001913	0.00319330	± 0.00002247	0.1404
18D00471	2.0 %	✓	0.0870530	± 0.0004355	0.00318838	± 0.00003088	0.1217
18D00473	2.2 %	✓	0.1007503	± 0.0003675	0.00315001	± 0.00002766	0.1092
18D00474	2.4 %	✓	0.1365311	± 0.0009730	0.00317742	± 0.00004762	0.0862
18D00476	2.7 %	✓	0.1229770	± 0.0005453	0.00312306	± 0.00003227	0.0774
18D00477	3.0 %	✓	0.1465538	± 0.0011834	0.00317523	± 0.00005717	0.0817
18D00479	3.4 %	✓	0.1526734	± 0.0008150	0.00310982	± 0.00004192	0.0739
18D00480	3.9 %	✓	0.1564143	± 0.0004044	0.00303403	± 0.00002587	0.1048
18D00482	4.5 %	✓	0.1974965	± 0.0006538	0.00300431	± 0.00003483	0.0887
18D00483	5.2 %	✓	0.2164401	± 0.0023620	0.00311899	± 0.00008928	0.1187
18D00485	6.0 %	✓	0.2153126	± 0.0017088	0.00304691	± 0.00006889	0.1034
18D00486	6.9 %	✓	0.2017278	± 0.0013131	0.00303630	± 0.00005894	0.0961
18D00488	7.9 %	✓	0.1915604	± 0.0010360	0.00303738	± 0.00005137	0.0792
18D00489	9.0 %	✓	0.1802460	± 0.0008918	0.00304857	± 0.00004687	0.0776
18D00491	10.3 %	✓	0.1468543	± 0.0004411	0.00307699	± 0.00003382	0.0511
18D00492	11.6 %		0.1491915	± 0.0008056	0.00312772	± 0.00004611	0.0584
18D00494	12.5 %		0.0967707	± 0.0006918	0.00321169	± 0.00004328	0.0439
18D00495	13.4 %		0.1071834	± 0.0017835	0.00327721	± 0.00008452	0.0672
18D00497	14.6 %		0.0782915	± 0.0017871	0.00328747	± 0.00008441	0.0495
18D00498	16.0 %		0.0794923	± 0.0018927	0.00332926	± 0.00008856	0.0525
18D00500	17.6 %		0.0769372	± 0.0027000	0.00333494	± 0.00011848	0.0542
18D00501	19.3 %		0.0609840	± 0.0013015	0.00336483	± 0.00006715	0.0365
18D00503	21.0 %		0.0598758	± 0.0047602	0.00366707	± 0.00020745	0.0534

Results	40(a)/36(a) ± 2σ		40(r)/39(k) ± 2σ		Age ± 2σ (Ma)		MSWD
**Inverse Isochron**<br>Error Chron	**305.05**	**± 3.37**<br>**± 1.10%**	0.38599	± 0.07616<br>± 19.73%	**1.14**	**± 0.22**<br>**± 19.72%**	3.21<br>0%
					Full External Error	± 0.23	
					Analytical Error	± 0.22	

Statistics				
	2σ Confidence Limit	1.76	Convergence	0.0002677559
	Error Magnification	1.7915	Number of Iterations	3
	Number of Data Points	16	Calculated Line	Weighted York-2
	Spreading Factor	7.0%		

Degassing Patterns			36Ar(a) [fA]	%1σ	36Ar(c) [fA]	%1σ	36Ar(ca) [fA]	%1σ	36Ar(cl) [fA]	%1σ	37Ar(ca) [fA]	%1σ	38Ar(a) [fA]	%1σ	38Ar(c) [fA]	%1σ	38Ar(k) [fA]	%1σ	38Ar(ca) [fA]	%1σ	38Ar(cl) [fA]	%1σ	39Ar(k) [fA]	%1σ	39Ar(ca) [fA]	%1σ	40Ar(r) [fA]	%1σ	40Ar(a) [fA]	%1σ	40Ar(c) [fA]	%1σ	40Ar(k) [fA]	%1σ
18D00468	1.8 %	✓	1.0233954	0.34	0.0000000	0.00	0.0243417	3.42	0.0000020	491.10	90.0542	3.41	0.1912726	0.34	0.0000000	0.00	0.1325979	0.24	0.0162098	10.22	0.0050378	491.10	10.97938	0.22	0.0578598	3.53	4.394671	46.65	311.8695	0.65	0.0000000	0.00	0.0066645	9.65
18D00470	1.9 %	✓	1.0765742	0.34	0.0000000	0.00	0.0446994	1.88	0.0000000	0.00	165.3697	1.87	0.2012117	0.34	0.0000000	0.00	0.2405996	0.16	0.0297665	9.81	0.0000000	0.00	19.92213	0.13	0.1062500	2.08	9.060067	23.90	328.0752	0.65	0.0000000	0.00	0.0120927	9.65
18D00471	2.0 %	✓	0.3827145	0.47	0.0000000	0.00	0.0231417	3.43	0.0000000	0.00	85.6149	3.42	0.0715293	0.47	0.0000000	0.00	0.1261968	0.24	0.0154107	10.22	0.0000000	0.00	10.44935	0.22	0.0550076	3.54	3.405904	25.32	116.6284	0.73	0.0000000	0.00	0.0063428	9.65
18D00473	2.2 %	✓	0.4788514	0.43	0.0000000	0.00	0.0354315	2.34	0.0000000	0.00	131.0822	2.33	0.0894973	0.43	0.0000000	0.00	0.1849670	0.18	0.0235948	9.91	0.0000000	0.00	15.31564	0.16	0.0842203	2.50	6.090624	17.02	145.9252	0.70	0.0000000	0.00	0.0092966	9.65
18D00474	2.4 %	✓	0.1626126	0.73	0.0000000	0.00	0.0157912	4.70	0.0000000	0.00	58.4210	4.70	0.0303923	0.73	0.0000000	0.00	0.0843861	0.33	0.0105158	10.72	0.0000000	0.00	6.98734	0.32	0.0375355	4.79	1.623041	28.55	49.5546	0.92	0.0000000	0.00	0.0042413	9.66
18D00476	2.7 %	✓	0.3029347	0.51	0.0000000	0.00	0.0295210	2.72	0.0000000	0.00	109.2158	2.71	0.0566185	0.51	0.0000000	0.00	0.1440625	0.22	0.0196588	10.01	0.0000000	0.00	11.92867	0.20	0.0701711	2.87	4.682851	15.00	92.3163	0.75	0.0000000	0.00	0.0072407	9.65
18D00477	3.0 %	✓	0.1329246	0.88	0.0000000	0.00	0.0152032	5.05	0.0000000	0.00	56.2457	5.05	0.0248436	0.88	0.0000000	0.00	0.0740945	0.38	0.0101242	10.87	0.0000000	0.00	6.13518	0.37	0.0361379	5.13	1.355527	31.68	40.5074	1.05	0.0000000	0.00	0.0037241	9.66
18D00479	3.4 %	✓	0.2026350	0.66	0.0000000	0.00	0.0263755	3.16	0.0000000	0.00	97.5786	3.15	0.0378725	0.66	0.0000000	0.00	0.1201440	0.26	0.0175642	10.13	0.0000000	0.00	9.94817	0.24	0.0626943	3.28	3.408811	15.87	61.7510	0.87	0.0000000	0.00	0.0060385	9.65
18D00480	3.9 %	✓	0.5949042	0.42	0.0000000	0.00	0.0905047	1.14	0.0000000	0.00	334.8306	1.13	0.1111876	0.42	0.0000000	0.00	0.3703926	0.14	0.0602695	9.70	0.0000000	0.00	30.66925	0.10	0.2151286	1.46	14.785903	8.62	181.2911	0.70	0.0000000	0.00	0.0186162	9.65
18D00482	4.5 %	✓	0.3106751	0.57	0.0000000	0.00	0.0666155	1.38	0.0000000	0.00	246.4503	1.37	0.0580652	0.57	0.0000000	0.00	0.2466498	0.16	0.0443611	9.73	0.0000000	0.00	20.42311	0.14	0.1583443	1.65	8.734820	8.73	94.6751	0.80	0.0000000	0.00	0.0123968	9.65
18D00483	5.2 %	✓	0.0720567	1.40	0.0000000	0.00	0.0165944	4.62	0.0000038	248.22	61.3924	4.62	0.0134674	1.40	0.0000000	0.00	0.0603890	0.46	0.0110506	10.68	0.0098435	248.22	5.00033	0.45	0.0394446	4.71	1.144051	29.55	21.9586	1.51	0.0000000	0.00	0.0030352	9.66
18D00485	6.0 %	✓	0.0985548	1.11	0.0000000	0.00	0.0249203	3.04	0.0000000	0.00	92.1949	3.04	0.0184199	1.11	0.0000000	0.00	0.0841098	0.35	0.0165951	10.10	0.0000000	0.00	6.96446	0.33	0.0592352	3.17	2.312211	16.41	30.0336	1.24	0.0000000	0.00	0.0042274	9.66
18D00486	6.9 %	✓	0.1222898	0.95	0.0000000	0.00	0.0309114	2.64	0.0000000	0.00	114.3596	2.64	0.0228560	0.95	0.0000000	0.00	0.0981230	0.29	0.0205847	9.98	0.0000000	0.00	8.12478	0.27	0.0734760	2.79	3.009361	13.89	37.2666	1.11	0.0000000	0.00	0.0049317	9.65
18D00488	7.9 %	✓	0.1612125	0.83	0.0000000	0.00	0.0413613	2.01	0.0000000	0.00	153.0200	2.00	0.0301306	0.83	0.0000000	0.00	0.1227902	0.25	0.0275436	9.84	0.0000000	0.00	10.16728	0.23	0.0983153	2.20	3.948225	12.63	49.1279	1.00	0.0000000	0.00	0.0061715	9.65
18D00489	9.0 %	✓	0.1787685	0.76	0.0000000	0.00	0.0491403	1.76	0.0000098	98.07	181.7990	1.75	0.0334118	0.76	0.0000000	0.00	0.1276498	0.23	0.0327238	9.79	0.0253062	98.07	10.56966	0.22	0.1168059	1.98	4.162273	12.45	54.4779	0.94	0.0000000	0.00	0.0064158	9.65
18D00491	10.3 %	✓	0.3960528	0.55	0.0000000	0.00	0.1161703	0.94	0.0000004	######	429.7827	0.92	0.0740223	0.55	0.0000000	0.00	0.2282825	0.16	0.0773609	9.67	0.0010391	######	18.90225	0.14	0.2761354	1.30	8.021183	11.79	120.6931	0.78	0.0000000	0.00	0.0114737	9.65
18D00492	11.6 %		0.2030174	0.73	0.0000000	0.00	0.0584535	1.53	0.0000036	268.88	216.2543	1.52	0.0379439	0.73	0.0000000	0.00	0.1169523	0.26	0.0389258	9.75	0.0093892	268.88	9.68388	0.25	0.1389434	1.78	3.041572	18.82	61.8675	0.92	0.0000000	0.00	0.0058781	9.65
18D00494	12.5 %		0.2175621	0.67	0.0000000	0.00	0.0587465	1.53	0.0000110	86.50	217.3383	1.52	0.0406624	0.67	0.0000000	0.00	0.0791685	0.35	0.0391209	9.75	0.0283050	86.51	6.55531	0.34	0.1396399	1.78	1.440777	40.27	66.2999	0.87	0.0000000	0.00	0.0039791	9.66
18D00495	13.4 %		0.0849328	1.26	0.0000000	0.00	0.0217448	3.66	0.0000009	######	80.4468	3.65	0.0158739	1.26	0.0000000	0.00	0.0335473	0.79	0.0144804	10.30	0.0022972	######	2.77779	0.79	0.0516870	3.77	0.033772	######	25.8824	1.38	0.0000000	0.00	0.0016861	9.68
18D00497	14.6 %		0.0844693	1.26	0.0000000	0.00	0.0212750	3.72	0.0000115	80.18	78.7088	3.72	0.0157873	1.26	0.0000000	0.00	0.0242946	1.11	0.0141676	10.32	0.0296556	80.19	2.01165	1.11	0.0505704	3.83	0.046871	768.71	25.7412	1.37	0.0000000	0.00	0.0012211	9.71
18D00498	16.0 %		0.0791976	1.30	0.0000000	0.00	0.0228422	3.34	0.0000085	112.79	84.5069	3.34	0.0148020	1.30	0.0000000	0.00	0.0228375	1.16	0.0152113	10.19	0.0219594	112.79	1.89099	1.16	0.0542957	3.46	0.346362	100.45	24.1347	1.41	0.0000000	0.00	0.0011478	9.72
18D00500	17.6 %		0.0561901	1.73	0.0000000	0.00	0.0163385	4.76	0.0000000	0.00	60.4457	4.76	0.0105019	1.73	0.0000000	0.00	0.0156555	1.71	0.0108802	10.74	0.0000000	0.00	1.29631	1.71	0.0388364	4.85	0.274459	116.08	17.1234	1.82	0.0000000	0.00	0.0007869	9.80
18D00501	19.3 %		0.1191670	0.98	0.0000000	0.00	0.0311493	2.67	0.0000000	0.00	115.2399	2.66	0.0222723	0.98	0.0000000	0.00	0.0260836	1.05	0.0207432	9.99	0.0000000	0.00	2.15977	1.05	0.0740416	2.82	0.899515	46.12	36.3149	1.13	0.0000000	0.00	0.0013110	9.71
18D00503	21.0 %		0.0329370	2.72	0.0000000	0.00	0.0054319	13.58	0.0000000	0.00	20.0959	13.58	0.0061559	2.72	0.0000000	0.00	0.0064950	3.90	0.0036173	16.65	0.0000000	0.00	0.53780	3.90	0.0129116	13.61	1.055382	27.22	10.0372	2.78	0.0000000	0.00	0.0003264	10.41
		Σ	6.5746301	0.13	0.0000000	0.00	0.8867051	0.46	0.0000515	55.63	3280.4481	0.46	1.2287984	0.13	0.0000000	0.00	2.7704694	0.06	0.5904807	2.43	0.1328329	55.63	229.40046	0.05	2.1076879	0.51	82.033055	5.08	2003.5528	0.21	0.0000000	0.00	0.1392461	2.46
		Σ							7.4613868	0.13	3280.4481	0.46									4.7225813	1.59			231.50815	0.05							2085.7251	0.28

**Additional Parameters**			40Ar/39Ar	1σ	37Ar/39Ar	1σ	36Ar/39Ar	1σ	Time (days)	37Ar (decay)	39Ar (decay)	40Ar (moles)
18D00468	1.8 %	✓	28.654895	0.067613	8.159126	0.278909	0.094928	0.000367	238.488	112.226404	1.00168775	1.518E-11
18D00470	1.9 %	✓	16.833483	0.027083	8.256768	0.154650	0.055984	0.000193	238.501	112.257196	1.00168784	1.618E-11
18D00471	2.0 %	✓	11.427698	0.028394	8.150419	0.279402	0.038637	0.000175	238.508	112.272595	1.00168789	5.762E-12
18D00473	2.2 %	✓	9.871849	0.017881	8.511909	0.198746	0.033395	0.000133	238.523	112.304939	1.00168800	7.297E-12
18D00474	2.4 %	✓	7.285805	0.025780	8.316307	0.391911	0.025396	0.000156	238.530	112.320345	1.00168804	2.457E-12
18D00476	2.7 %	✓	8.084651	0.017785	9.102196	0.247715	0.027707	0.000123	238.544	112.351162	1.00168814	4.656E-12
18D00477	3.0 %	✓	6.784079	0.027182	9.114053	0.461175	0.024003	0.000168	238.551	112.366574	1.00168819	2.010E-12
18D00479	3.4 %	✓	6.509514	0.017234	9.747277	0.308092	0.022876	0.000119	238.565	112.397405	1.00168829	3.128E-12
18D00480	3.9 %	✓	6.349346	0.008145	10.841421	0.122904	0.022193	0.000077	238.572	112.412823	1.00168834	9.413E-12
18D00482	4.5 %	✓	5.025027	0.008248	11.974390	0.164585	0.018332	0.000078	238.585	112.443666	1.00168844	4.964E-12
18D00483	5.2 %	✓	4.584658	0.024822	12.181573	0.565267	0.017591	0.000152	238.592	112.459090	1.00168849	1.109E-12
18D00485	6.0 %	✓	4.605842	0.018125	13.126267	0.401075	0.017580	0.000126	238.606	112.489946	1.00168858	1.553E-12
18D00486	6.9 %	✓	4.913348	0.015841	13.949260	0.369884	0.018687	0.000114	238.613	112.505377	1.00168863	1.933E-12
18D00488	7.9 %	✓	5.170892	0.013839	14.906102	0.299813	0.019733	0.000113	238.627	112.536245	1.00168873	2.548E-12
18D00489	9.0 %	✓	5.487932	0.013411	17.012080	0.299909	0.021328	0.000108	238.634	112.551682	1.00168878	2.815E-12
18D00491	10.3 %	✓	6.712025	0.009881	22.409743	0.209268	0.026708	0.000103	238.649	112.584107	1.00168888	6.179E-12
18D00492	11.6 %		6.608583	0.017553	22.015485	0.338470	0.026619	0.000136	238.656	112.599551	1.00168893	3.116E-12
18D00494	12.5 %		10.118762	0.035277	32.463003	0.506413	0.041273	0.000218	238.669	112.630445	1.00168903	3.252E-12
18D00495	13.4 %		9.159971	0.074695	28.431709	1.061897	0.037703	0.000385	238.676	112.645895	1.00168908	1.244E-12
18D00497	14.6 %		12.460156	0.138422	38.167102	1.478052	0.051283	0.000650	238.690	112.676802	1.00168918	1.233E-12
18D00498	16.0 %		12.229301	0.141275	43.441940	1.529969	0.052459	0.000685	238.697	112.692259	1.00168923	1.142E-12
18D00500	17.6 %		12.620125	0.214620	45.272790	2.279884	0.054323	0.000997	238.711	112.723178	1.00168933	8.088E-13
18D00501	19.3 %		15.854812	0.163096	51.588778	1.469129	0.067291	0.000772	238.718	112.738641	1.00168937	1.700E-12
18D00503	21.0 %		16.310251	0.631587	36.491014	5.145118	0.069672	0.002800	238.732	112.769573	1.00168947	4.311E-13

Procedure Blanks		36Ar ± 1σ (SE) [fA]	37Ar ± 1σ (SE) [fA]	38Ar ± 1σ (SE) [fA]	39Ar ± 1σ (SE) [fA]	40Ar ± 1σ (SE) [fA]
18D00468	1.8 %	0.0181742 ± 0.0002699	0.0019422 ± 0.0172677	0.0096479 ± 0.0184036	0.0269767 ± 0.0150091	5.3300120 ± 0.0662264
18D00470	1.9 %	0.0182430 ± 0.0002699	0.0014425 ± 0.0172677	0.0130374 ± 0.0184036	0.0226631 ± 0.0150091	5.3294886 ± 0.0662264
18D00471	2.0 %	0.0182976 ± 0.0002699	0.0026988 ± 0.0172677	0.0144388 ± 0.0184036	0.0218574 ± 0.0150091	5.3391218 ± 0.0662264
18D00473	2.2 %	0.0184330 ± 0.0002699	0.0043432 ± 0.0172677	0.0165589 ± 0.0184036	0.0223331 ± 0.0150091	5.3726070 ± 0.0662264
18D00474	2.4 %	0.0184999 ± 0.0002699	0.0046460 ± 0.0172677	0.0171254 ± 0.0184036	0.0233269 ± 0.0150091	5.3920973 ± 0.0662264
18D00476	2.7 %	0.0186228 ± 0.0002699	0.0043316 ± 0.0172677	0.0173243 ± 0.0184036	0.0262284 ± 0.0150091	5.4321887 ± 0.0662264
18D00477	3.0 %	0.0186738 ± 0.0002699	0.0037286 ± 0.0172677	0.0169456 ± 0.0184036	0.0279383 ± 0.0150091	5.4508919 ± 0.0662264
18D00479	3.4 %	0.0187454 ± 0.0002699	0.0016924 ± 0.0172677	0.0152498 ± 0.0184036	0.0314457 ± 0.0150091	5.4817479 ± 0.0662264
18D00480	3.9 %	0.0187631 ± 0.0002699	0.0002940 ± 0.0172677	0.0139582 ± 0.0184036	0.0330968 ± 0.0150091	5.4927059 ± 0.0662264
18D00482	4.5 %	0.0187578 ± 0.0002699	0.0031507 ± 0.0172677	0.0105972 ± 0.0184036	0.0358919 ± 0.0150091	5.5035732 ± 0.0662264
18D00483	5.2 %	0.0187342 ± 0.0002699	0.0051416 ± 0.0172677	0.0085900 ± 0.0184036	0.0369408 ± 0.0150091	5.5029907 ± 0.0662264
18D00485	6.0 %	0.0186458 ± 0.0002699	0.0094964 ± 0.0172677	0.0041239 ± 0.0184036	0.0381680 ± 0.0150091	5.4894427 ± 0.0662264
18D00486	6.9 %	0.0185826 ± 0.0002699	0.0117843 ± 0.0172677	0.0017638 ± 0.0184036	0.0383027 ± 0.0150091	5.4766883 ± 0.0662264
18D00488	7.9 %	0.0184241 ± 0.0002699	0.0163655 ± 0.0172677	0.0029170 ± 0.0184036	0.0375691 ± 0.0150091	5.4406251 ± 0.0662264
18D00489	9.0 %	0.0183326 ± 0.0002699	0.0185623 ± 0.0172677	0.0051021 ± 0.0184036	0.0367086 ± 0.0150091	5.4182304 ± 0.0662264
18D00491	10.3 %	0.0181264 ± 0.0002699	0.0226760 ± 0.0172677	0.0089267 ± 0.0184036	0.0339241 ± 0.0150091	5.3652515 ± 0.0662264
18D00492	11.6 %	0.0180277 ± 0.0002699	0.0242726 ± 0.0172677	0.0102009 ± 0.0184036	0.0321961 ± 0.0150091	5.3389152 ± 0.0662264
18D00494	12.5 %	0.0178467 ± 0.0002699	0.0264382 ± 0.0172677	0.0112017 ± 0.0184036	0.0281819 ± 0.0150091	5.2891623 ± 0.0662264
18D00495	13.4 %	0.0177724 ± 0.0002699	0.0268690 ± 0.0172677	0.0107180 ± 0.0184036	0.0260078 ± 0.0150091	5.2680891 ± 0.0662264
18D00497	14.6 %	0.0176787 ± 0.0002699	0.0260546 ± 0.0172677	0.0072107 ± 0.0184036	0.0216708 ± 0.0150091	5.2402866 ± 0.0662264
18D00498	16.0 %	0.0176693 ± 0.0002699	0.0246506 ± 0.0172677	0.0039400 ± 0.0184036	0.0196715 ± 0.0150091	5.2366038 ± 0.0662264
18D00500	17.6 %	0.0177534 ± 0.0002699	0.0194256 ± 0.0172677	0.0062988 ± 0.0184036	0.0164847 ± 0.0150091	5.2581697 ± 0.0662264
18D00501	19.3 %	0.0178591 ± 0.0002699	0.0154252 ± 0.0172677	0.0135508 ± 0.0184036	0.0155122 ± 0.0150091	5.2871679 ± 0.0662264
18D00503	21.0 %	0.0182309 ± 0.0002699	0.0041739 ± 0.0172677	0.0330750 ± 0.0184036	0.0154111 ± 0.0150091	5.3918469 ± 0.0662264

**Intercept Values**		**36Ar ± 1σ (SE) [fA]**	**r2**	**Regression (type,n)**	**37Ar ± 1σ (SE) [fA]**	**r2**	**Regression (type,n)**	**38Ar ± 1σ (SE) [fA]**	**r2**	**Regression (type,n)**	**39Ar ± 1σ (SE) [fA]**	**r2**	**Regression (type,n)**	**40Ar ± 1σ (SE) [fA]**	**r2**	**Regression (type,n)**
18D00468	1.8 %	1.0161249 ± 0.0014280	0.9323	EXP 150 of 150	0.7869072 ± 0.0198838	0.0240	EXP 150 of 150	0.3508661 ± 0.0160048	0.0195	EXP 150 of 150	10.9834693 ± 0.0171379	0.8785	EXP 150 of 150	321.600850 ± 0.267756	0.1779	EXP 150 of 150
18D00470	1.9 %	1.0862340 ± 0.0015226	0.9323	EXP 150 of 150	1.4496370 ± 0.0181827	0.2160	EXP 150 of 150	0.4333185 ± 0.0145155	0.0052	EXP 150 of 150	19.9045226 ± 0.0170485	0.9749	EXP 150 of 150	342.476884 ± 0.294085	0.2781	EXP 150 of 150
18D00471	2.0 %	0.4048676 ± 0.0010391	0.4271	EXP 150 of 150	0.7523529 ± 0.0182194	0.0356	EXP 150 of 150	0.2084378 ± 0.0180310	0.0101	EXP 150 of 150	10.4493708 ± 0.0152679	0.9085	EXP 149 of 150	125.379780 ± 0.129830	0.9764	EXP 150 of 150
18D00473	2.2 %	0.5082773 ± 0.0011002	0.7542	EXP 150 of 150	1.1517834 ± 0.0187522	0.0837	EXP 150 of 150	0.2751384 ± 0.0164985	0.0073	EXP 150 of 150	15.3095307 ± 0.0149699	0.9679	EXP 150 of 150	157.397705 ± 0.125781	0.9547	EXP 150 of 150
18D00474	2.4 %	0.1884260 ± 0.0006977	0.1035	EXP 150 of 150	0.5159694 ± 0.0163377	0.0007	EXP 149 of 150	0.1189411 ± 0.0176302	0.0004	EXP 150 of 150	6.9968061 ± 0.0157511	0.7977	EXP 150 of 150	56.573947 ± 0.040560	0.9975	EXP 150 of 150
18D00476	2.7 %	0.3352803 ± 0.0008288	0.5844	EXP 150 of 150	0.9599687 ± 0.0181562	0.0666	EXP 150 of 150	0.2052966 ± 0.0171141	0.0009	EXP 150 of 150	11.9372841 ± 0.0163380	0.9385	EXP 149 of 150	102.438605 ± 0.062899	0.9887	EXP 150 of 150
18D00477	3.0 %	0.1597626 ± 0.0006959	0.0598	EXP 150 of 150	0.4958105 ± 0.0175135	0.0046	EXP 150 of 150	0.1043875 ± 0.0168699	0.0026	EXP 150 of 150	6.1541043 ± 0.0157353	0.7439	EXP 150 of 150	47.317580 ± 0.028647	0.9984	EXP 150 of 150
18D00479	3.4 %	0.2368734 ± 0.0007482	0.3052	EXP 150 of 150	0.8551534 ± 0.0197367	0.0365	EXP 150 of 150	0.1676527 ± 0.0172348	0.0012	EXP 150 of 150	9.9690666 ± 0.0169692	0.9039	EXP 150 of 150	70.647590 ± 0.035467	0.9965	EXP 150 of 150
18D00480	3.9 %	0.6716015 ± 0.0010915	0.8998	EXP 150 of 150	2.9284516 ± 0.0192970	0.4156	EXP 150 of 150	0.4874885 ± 0.0170983	0.0222	EXP 150 of 150	30.6915245 ± 0.0180037	0.9907	EXP 150 of 150	201.588330 ± 0.133452	0.8570	EXP 150 of 150
18D00482	4.5 %	0.3781196 ± 0.0009973	0.6304	EXP 150 of 150	2.1515133 ± 0.0184850	0.2387	EXP 150 of 150	0.3323024 ± 0.0162260	0.0131	EXP 150 of 150	20.4667628 ± 0.0185077	0.9756	EXP 150 of 150	108.925908 ± 0.068624	0.9852	EXP 150 of 150
18D00483	5.2 %	0.1031762 ± 0.0005075	0.4766	EXP 150 of 150	0.5315260 ± 0.0173792	0.0053	EXP 149 of 150	0.1022699 ± 0.0155984	0.0037	EXP 149 of 150	5.0398455 ± 0.0162588	0.6185	EXP 150 of 150	28.608645 ± 0.023705	0.9990	EXP 150 of 150
18D00485	6.0 %	0.1362534 ± 0.0006164	0.0690	EXP 150 of 150	0.7962128 ± 0.0163976	0.0559	EXP 150 of 150	0.0964572 ± 0.0170167	0.0018	EXP 150 of 150	7.0104741 ± 0.0166954	0.8060	EXP 150 of 150	37.839468 ± 0.021560	0.9988	EXP 150 of 150
18D00486	6.9 %	0.1645037 ± 0.0006226	0.0071	EXP 150 of 150	0.9874895 ± 0.0186384	0.0899	EXP 150 of 150	0.1336746 ± 0.0168006	0.0019	EXP 149 of 150	8.1765756 ± 0.0151675	0.8960	EXP 150 of 150	45.757570 ± 0.023226	0.9986	EXP 150 of 150
18D00488	7.9 %	0.2113716 ± 0.0008050	0.1935	EXP 150 of 150	1.3203560 ± 0.0180858	0.1602	EXP 150 of 150	0.1426491 ± 0.0163028	0.0095	EXP 150 of 150	10.2280550 ± 0.0167445	0.9131	EXP 150 of 150	58.522907 ± 0.026727	0.9976	EXP 150 of 150
18D00489	9.0 %	0.2354205 ± 0.0007366	0.2833	EXP 150 of 150	1.5693433 ± 0.0187057	0.2204	EXP 150 of 150	0.2115138 ± 0.0159034	0.0112	EXP 150 of 150	10.6449855 ± 0.0150473	0.9394	EXP 150 of 150	64.064820 ± 0.026185	0.9976	EXP 150 of 150
18D00491	10.3 %	0.5060091 ± 0.0010840	0.8647	EXP 150 of 150	3.7301375 ± 0.0145421	0.6944	EXP 149 of 150	0.3674760 ± 0.0144167	0.0281	EXP 150 of 150	19.0719863 ± 0.0152331	0.9816	EXP 150 of 150	134.091051 ± 0.051010	0.9578	EXP 150 of 150
18D00492	11.6 %	0.2670770 ± 0.0008333	0.5131	EXP 150 of 150	1.8637765 ± 0.0186482	0.1711	EXP 150 of 150	0.1907140 ± 0.0164293	0.0004	EXP 150 of 150	9.7831522 ± 0.0169996	0.9004	EXP 150 of 150	70.253880 ± 0.022602	0.9977	EXP 150 of 150
18D00494	12.5 %	0.2810358 ± 0.0007342	0.7414	EXP 150 of 150	1.8705543 ± 0.0189810	0.2926	EXP 150 of 150	0.1739391 ± 0.0152568	0.0241	EXP 149 of 150	6.6741496 ± 0.0156047	0.8203	EXP 149 of 150	73.033803 ± 0.021566	0.9968	EXP 150 of 150
18D00495	13.4 %	0.1193816 ± 0.0005579	0.0204	EXP 150 of 150	0.6751977 ± 0.0183270	0.0272	EXP 150 of 150	0.0547329 ± 0.0157964	0.0035	EXP 150 of 150	2.8347781 ± 0.0154494	0.2716	EXP 150 of 150	31.185981 ± 0.021281	0.9985	EXP 150 of 150
18D00497	14.6 %	0.1184090 ± 0.0005443	0.0004	EXP 150 of 150	0.6606564 ± 0.0181939	0.0993	EXP 150 of 150	0.0757463 ± 0.0145472	0.0050	EXP 150 of 150	2.0687976 ± 0.0161086	0.0772	EXP 149 of 150	30.935821 ± 0.020110	0.9985	EXP 150 of 150
18D00498	16.0 %	0.1148684 ± 0.0005304	0.0011	EXP 150 of 150	0.7125463 ± 0.0167666	0.0826	EXP 149 of 150	0.0700248 ± 0.0160730	0.0085	EXP 150 of 150	1.9507226 ± 0.0154771	0.0335	EXP 149 of 150	29.026078 ± 0.017957	0.9989	EXP 150 of 150
18D00500	17.6 %	0.0868354 ± 0.0004420	0.3197	EXP 150 of 150	0.5077285 ± 0.0178099	0.0104	EXP 150 of 150	0.0428099 ± 0.0172685	0.0016	EXP 150 of 150	1.3418600 ± 0.0158814	0.0004	EXP 150 of 150	22.107864 ± 0.020308	0.9986	EXP 150 of 150
18D00501	19.3 %	0.1610324 ± 0.0006074	0.0672	EXP 150 of 150	0.9894569 ± 0.0191841	0.0241	EXP 150 of 150	0.0594483 ± 0.0169385	0.0018	EXP 149 of 150	2.2329834 ± 0.0165403	0.0546	EXP 150 of 150	40.703905 ± 0.022047	0.9982	EXP 150 of 150
18D00503	21.0 %	0.0547766 ± 0.0003887	0.6910	EXP 149 of 150	0.1710124 ± 0.0163175	0.0095	EXP 150 of 150	0.0200548 ± 0.0160269	0.0005	EXP 150 of 150	0.5620890 ± 0.0143096	0.1375	EXP 150 of 150	14.374026 ± 0.021453	0.9988	EXP 150 of 150

Project Info		Analyst	Irradiation	X-pos	Y-pos	Z/H-pos	Project	Experiment	Nmb
18D00468	1.8 %	Dan Miggins	17-OSU-05	0.00	0.00	13.79	Arctic\O-Connor (16-22)	18D00464	01
18D00470	1.9 %	Dan Miggins	17-OSU-05	0.00	0.00	13.79	Arctic\O-Connor (16-22)	18D00464	01
18D00471	2.0 %	Dan Miggins	17-OSU-05	0.00	0.00	13.79	Arctic\O-Connor (16-22)	18D00464	01
18D00473	2.2 %	Dan Miggins	17-OSU-05	0.00	0.00	13.79	Arctic\O-Connor (16-22)	18D00464	01
18D00474	2.4 %	Dan Miggins	17-OSU-05	0.00	0.00	13.79	Arctic\O-Connor (16-22)	18D00464	01
18D00476	2.7 %	Dan Miggins	17-OSU-05	0.00	0.00	13.79	Arctic\O-Connor (16-22)	18D00464	01
18D00477	3.0 %	Dan Miggins	17-OSU-05	0.00	0.00	13.79	Arctic\O-Connor (16-22)	18D00464	01
18D00479	3.4 %	Dan Miggins	17-OSU-05	0.00	0.00	13.79	Arctic\O-Connor (16-22)	18D00464	01
18D00480	3.9 %	Dan Miggins	17-OSU-05	0.00	0.00	13.79	Arctic\O-Connor (16-22)	18D00464	01
18D00482	4.5 %	Dan Miggins	17-OSU-05	0.00	0.00	13.79	Arctic\O-Connor (16-22)	18D00464	01
18D00483	5.2 %	Dan Miggins	17-OSU-05	0.00	0.00	13.79	Arctic\O-Connor (16-22)	18D00464	01
18D00485	6.0 %	Dan Miggins	17-OSU-05	0.00	0.00	13.79	Arctic\O-Connor (16-22)	18D00464	01
18D00486	6.9 %	Dan Miggins	17-OSU-05	0.00	0.00	13.79	Arctic\O-Connor (16-22)	18D00464	01
18D00488	7.9 %	Dan Miggins	17-OSU-05	0.00	0.00	13.79	Arctic\O-Connor (16-22)	18D00464	01
18D00489	9.0 %	Dan Miggins	17-OSU-05	0.00	0.00	13.79	Arctic\O-Connor (16-22)	18D00464	01
18D00491	10.3 %	Dan Miggins	17-OSU-05	0.00	0.00	13.79	Arctic\O-Connor (16-22)	18D00464	01
18D00492	11.6 %	Dan Miggins	17-OSU-05	0.00	0.00	13.79	Arctic\O-Connor (16-22)	18D00464	01
18D00494	12.5 %	Dan Miggins	17-OSU-05	0.00	0.00	13.79	Arctic\O-Connor (16-22)	18D00464	01
18D00495	13.4 %	Dan Miggins	17-OSU-05	0.00	0.00	13.79	Arctic\O-Connor (16-22)	18D00464	01
18D00497	14.6 %	Dan Miggins	17-OSU-05	0.00	0.00	13.79	Arctic\O-Connor (16-22)	18D00464	01
18D00498	16.0 %	Dan Miggins	17-OSU-05	0.00	0.00	13.79	Arctic\O-Connor (16-22)	18D00464	01
18D00500	17.6 %	Dan Miggins	17-OSU-05	0.00	0.00	13.79	Arctic\O-Connor (16-22)	18D00464	01
18D00501	19.3 %	Dan Miggins	17-OSU-05	0.00	0.00	13.79	Arctic\O-Connor (16-22)	18D00464	01
18D00503	21.0 %	Dan Miggins	17-OSU-05	0.00	0.00	13.79	Arctic\O-Connor (16-22)	18D00464	01

Sample Parameters		Sample	Material	Location	Standard Name	Standard (in Ma)	%1σ	Standard Reference	Standard 40Ar/39Ar	%1σ	J	%1σ	Air 40Ar/36Ar	%1σ	MDF (lin)	%1σ	Volume Ratio	Sensitivity (mol/volt)	Day	Month	Year	Hour	Min	Resist
18D00468	1.8 %	PS59-271-1-14	Groundmass	Gakkel Ridge	FCT-NM (5B9-17)	28.201	0.082	Kuiper et al (2008)	9.62643	0.082	0.00163273	0.082	302.325	0.155	0.9943489	0.070	1	4.8E-14	4	JAN	2018	23	32	1
18D00470	1.9 %	PS59-271-1-14	Groundmass	Gakkel Ridge	FCT-NM (5B9-17)	28.201	0.082	Kuiper et al (2008)	9.62643	0.082	0.00163273	0.082	302.325	0.155	0.9943489	0.070	1	4.8E-14	4	JAN	2018	23	52	1
18D00471	2.0 %	PS59-271-1-14	Groundmass	Gakkel Ridge	FCT-NM (5B9-17)	28.201	0.082	Kuiper et al (2008)	9.62643	0.082	0.00163273	0.082	302.325	0.155	0.9943489	0.070	1	4.8E-14	5	JAN	2018	0	2	1
18D00473	2.2 %	PS59-271-1-14	Groundmass	Gakkel Ridge	FCT-NM (5B9-17)	28.201	0.082	Kuiper et al (2008)	9.62643	0.082	0.00163273	0.082	302.325	0.155	0.9943489	0.070	1	4.8E-14	5	JAN	2018	0	23	1
18D00474	2.4 %	PS59-271-1-14	Groundmass	Gakkel Ridge	FCT-NM (5B9-17)	28.201	0.082	Kuiper et al (2008)	9.62643	0.082	0.00163273	0.082	302.325	0.155	0.9943489	0.070	1	4.8E-14	5	JAN	2018	0	33	1
18D00476	2.7 %	PS59-271-1-14	Groundmass	Gakkel Ridge	FCT-NM (5B9-17)	28.201	0.082	Kuiper et al (2008)	9.62643	0.082	0.00163273	0.082	302.325	0.155	0.9943489	0.070	1	4.8E-14	5	JAN	2018	0	53	1
18D00477	3.0 %	PS59-271-1-14	Groundmass	Gakkel Ridge	FCT-NM (5B9-17)	28.201	0.082	Kuiper et al (2008)	9.62643	0.082	0.00163273	0.082	302.325	0.155	0.9943489	0.070	1	4.8E-14	5	JAN	2018	1	3	1
18D00479	3.4 %	PS59-271-1-14	Groundmass	Gakkel Ridge	FCT-NM (5B9-17)	28.201	0.082	Kuiper et al (2008)	9.62643	0.082	0.00163273	0.082	302.325	0.155	0.9943489	0.070	1	4.8E-14	5	JAN	2018	1	23	1
18D00480	3.9 %	PS59-271-1-14	Groundmass	Gakkel Ridge	FCT-NM (5B9-17)	28.201	0.082	Kuiper et al (2008)	9.62643	0.082	0.00163273	0.082	302.325	0.155	0.9943489	0.070	1	4.8E-14	5	JAN	2018	1	33	1
18D00482	4.5 %	PS59-271-1-14	Groundmass	Gakkel Ridge	FCT-NM (5B9-17)	28.201	0.082	Kuiper et al (2008)	9.62643	0.082	0.00163273	0.082	302.325	0.155	0.9943489	0.070	1	4.8E-14	5	JAN	2018	1	53	1
18D00483	5.2 %	PS59-271-1-14	Groundmass	Gakkel Ridge	FCT-NM (5B9-17)	28.201	0.082	Kuiper et al (2008)	9.62643	0.082	0.00163273	0.082	302.325	0.155	0.9943489	0.070	1	4.8E-14	5	JAN	2018	2	3	1
18D00485	6.0 %	PS59-271-1-14	Groundmass	Gakkel Ridge	FCT-NM (5B9-17)	28.201	0.082	Kuiper et al (2008)	9.62643	0.082	0.00163273	0.082	302.325	0.155	0.9943489	0.070	1	4.8E-14	5	JAN	2018	2	23	1
18D00486	6.9 %	PS59-271-1-14	Groundmass	Gakkel Ridge	FCT-NM (5B9-17)	28.201	0.082	Kuiper et al (2008)	9.62643	0.082	0.00163273	0.082	302.325	0.155	0.9943489	0.070	1	4.8E-14	5	JAN	2018	2	33	1
18D00488	7.9 %	PS59-271-1-14	Groundmass	Gakkel Ridge	FCT-NM (5B9-17)	28.201	0.082	Kuiper et al (2008)	9.62643	0.082	0.00163273	0.082	302.325	0.155	0.9943489	0.070	1	4.8E-14	5	JAN	2018	2	53	1
18D00489	9.0 %	PS59-271-1-14	Groundmass	Gakkel Ridge	FCT-NM (5B9-17)	28.201	0.082	Kuiper et al (2008)	9.62643	0.082	0.00163273	0.082	302.325	0.155	0.9943489	0.070	1	4.8E-14	5	JAN	2018	3	3	1
18D00491	10.3 %	PS59-271-1-14	Groundmass	Gakkel Ridge	FCT-NM (5B9-17)	28.201	0.082	Kuiper et al (2008)	9.62643	0.082	0.00163273	0.082	302.325	0.155	0.9943489	0.070	1	4.8E-14	5	JAN	2018	3	24	1
18D00492	11.6 %	PS59-271-1-14	Groundmass	Gakkel Ridge	FCT-NM (5B9-17)	28.201	0.082	Kuiper et al (2008)	9.62643	0.082	0.00163273	0.082	302.325	0.155	0.9943489	0.070	1	4.8E-14	5	JAN	2018	3	34	1
18D00494	12.5 %	PS59-271-1-14	Groundmass	Gakkel Ridge	FCT-NM (5B9-17)	28.201	0.082	Kuiper et al (2008)	9.62643	0.082	0.00163273	0.082	302.325	0.155	0.9943489	0.070	1	4.8E-14	5	JAN	2018	3	54	1
18D00495	13.4 %	PS59-271-1-14	Groundmass	Gakkel Ridge	FCT-NM (5B9-17)	28.201	0.082	Kuiper et al (2008)	9.62643	0.082	0.00163273	0.082	302.325	0.155	0.9943489	0.070	1	4.8E-14	5	JAN	2018	4	4	1
18D00497	14.6 %	PS59-271-1-14	Groundmass	Gakkel Ridge	FCT-NM (5B9-17)	28.201	0.082	Kuiper et al (2008)	9.62643	0.082	0.00163273	0.082	302.325	0.155	0.9943489	0.070	1	4.8E-14	5	JAN	2018	4	24	1
18D00498	16.0 %	PS59-271-1-14	Groundmass	Gakkel Ridge	FCT-NM (5B9-17)	28.201	0.082	Kuiper et al (2008)	9.62643	0.082	0.00163273	0.082	302.325	0.155	0.9943489	0.070	1	4.8E-14	5	JAN	2018	4	34	1
18D00500	17.6 %	PS59-271-1-14	Groundmass	Gakkel Ridge	FCT-NM (5B9-17)	28.201	0.082	Kuiper et al (2008)	9.62643	0.082	0.00163273	0.082	302.325	0.155	0.9943489	0.070	1	4.8E-14	5	JAN	2018	4	54	1
18D00501	19.3 %	PS59-271-1-14	Groundmass	Gakkel Ridge	FCT-NM (5B9-17)	28.201	0.082	Kuiper et al (2008)	9.62643	0.082	0.00163273	0.082	302.325	0.155	0.9943489	0.070	1	4.8E-14	5	JAN	2018	5	4	1
18D00503	21.0 %	PS59-271-1-14	Groundmass	Gakkel Ridge	FCT-NM (5B9-17)	28.201	0.082	Kuiper et al (2008)	9.62643	0.082	0.00163273	0.082	302.325	0.155	0.9943489	0.070	1	4.8E-14	5	JAN	2018	5	24	1

Irradiation Constants		40/36(a)	%1σ	40/36(c)	%1σ	38/36(a)	%1σ	38/36(c)	%1σ	39/37(ca)	%1σ	38/37(ca)	%1σ	36/37(ca)	%1σ	40/39(k)	%1σ	38/39(k)	%1σ	36/38(cl)	%1σ	K/Ca	%1σ	K/Cl	%1σ	Ca/Cl	%1σ
18D00468	1.8 %	304.74	0.558	0.018	35	0.1869	0	1.493	3	0.000643	0.92	0.00018	9.63	0.00027	0.17	0.000607	9.65	0.012077	0.09	0	0	0.43	0	0	0	0	0
18D00470	1.9 %	304.74	0.558	0.018	35	0.1869	0	1.493	3	0.000643	0.92	0.00018	9.63	0.00027	0.17	0.000607	9.65	0.012077	0.09	0	0	0.43	0	0	0	0	0
18D00471	2.0 %	304.74	0.558	0.018	35	0.1869	0	1.493	3	0.000643	0.92	0.00018	9.63	0.00027	0.17	0.000607	9.65	0.012077	0.09	0	0	0.43	0	0	0	0	0
18D00473	2.2 %	304.74	0.558	0.018	35	0.1869	0	1.493	3	0.000643	0.92	0.00018	9.63	0.00027	0.17	0.000607	9.65	0.012077	0.09	0	0	0.43	0	0	0	0	0
18D00474	2.4 %	304.74	0.558	0.018	35	0.1869	0	1.493	3	0.000643	0.92	0.00018	9.63	0.00027	0.17	0.000607	9.65	0.012077	0.09	0	0	0.43	0	0	0	0	0
18D00476	2.7 %	304.74	0.558	0.018	35	0.1869	0	1.493	3	0.000643	0.92	0.00018	9.63	0.00027	0.17	0.000607	9.65	0.012077	0.09	0	0	0.43	0	0	0	0	0
18D00477	3.0 %	304.74	0.558	0.018	35	0.1869	0	1.493	3	0.000643	0.92	0.00018	9.63	0.00027	0.17	0.000607	9.65	0.012077	0.09	0	0	0.43	0	0	0	0	0
18D00479	3.4 %	304.74	0.558	0.018	35	0.1869	0	1.493	3	0.000643	0.92	0.00018	9.63	0.00027	0.17	0.000607	9.65	0.012077	0.09	0	0	0.43	0	0	0	0	0
18D00480	3.9 %	304.74	0.558	0.018	35	0.1869	0	1.493	3	0.000643	0.92	0.00018	9.63	0.00027	0.17	0.000607	9.65	0.012077	0.09	0	0	0.43	0	0	0	0	0
18D00482	4.5 %	304.74	0.558	0.018	35	0.1869	0	1.493	3	0.000643	0.92	0.00018	9.63	0.00027	0.17	0.000607	9.65	0.012077	0.09	0	0	0.43	0	0	0	0	0
18D00483	5.2 %	304.74	0.558	0.018	35	0.1869	0	1.493	3	0.000643	0.92	0.00018	9.63	0.00027	0.17	0.000607	9.65	0.012077	0.09	0	0	0.43	0	0	0	0	0
18D00485	6.0 %	304.74	0.558	0.018	35	0.1869	0	1.493	3	0.000643	0.92	0.00018	9.63	0.00027	0.17	0.000607	9.65	0.012077	0.09	0	0	0.43	0	0	0	0	0
18D00486	6.9 %	304.74	0.558	0.018	35	0.1869	0	1.493	3	0.000643	0.92	0.00018	9.63	0.00027	0.17	0.000607	9.65	0.012077	0.09	0	0	0.43	0	0	0	0	0
18D00488	7.9 %	304.74	0.558	0.018	35	0.1869	0	1.493	3	0.000643	0.92	0.00018	9.63	0.00027	0.17	0.000607	9.65	0.012077	0.09	0	0	0.43	0	0	0	0	0
18D00489	9.0 %	304.74	0.558	0.018	35	0.1869	0	1.493	3	0.000643	0.92	0.00018	9.63	0.00027	0.17	0.000607	9.65	0.012077	0.09	0	0	0.43	0	0	0	0	0
18D00491	10.3 %	304.74	0.558	0.018	35	0.1869	0	1.493	3	0.000643	0.92	0.00018	9.63	0.00027	0.17	0.000607	9.65	0.012077	0.09	0	0	0.43	0	0	0	0	0
18D00492	11.6 %	304.74	0.558	0.018	35	0.1869	0	1.493	3	0.000643	0.92	0.00018	9.63	0.00027	0.17	0.000607	9.65	0.012077	0.09	0	0	0.43	0	0	0	0	0
18D00494	12.5 %	304.74	0.558	0.018	35	0.1869	0	1.493	3	0.000643	0.92	0.00018	9.63	0.00027	0.17	0.000607	9.65	0.012077	0.09	0	0	0.43	0	0	0	0	0
18D00495	13.4 %	304.74	0.558	0.018	35	0.1869	0	1.493	3	0.000643	0.92	0.00018	9.63	0.00027	0.17	0.000607	9.65	0.012077	0.09	0	0	0.43	0	0	0	0	0
18D00497	14.6 %	304.74	0.558	0.018	35	0.1869	0	1.493	3	0.000643	0.92	0.00018	9.63	0.00027	0.17	0.000607	9.65	0.012077	0.09	0	0	0.43	0	0	0	0	0
18D00498	16.0 %	304.74	0.558	0.018	35	0.1869	0	1.493	3	0.000643	0.92	0.00018	9.63	0.00027	0.17	0.000607	9.65	0.012077	0.09	0	0	0.43	0	0	0	0	0
18D00500	17.6 %	304.74	0.558	0.018	35	0.1869	0	1.493	3	0.000643	0.92	0.00018	9.63	0.00027	0.17	0.000607	9.65	0.012077	0.09	0	0	0.43	0	0	0	0	0
18D00501	19.3 %	304.74	0.558	0.018	35	0.1869	0	1.493	3	0.000643	0.92	0.00018	9.63	0.00027	0.17	0.000607	9.65	0.012077	0.09	0	0	0.43	0	0	0	0	0
18D00503	21.0 %	304.74	0.558	0.018	35	0.1869	0	1.493	3	0.000643	0.92	0.00018	9.63	0.00027	0.17	0.000607	9.65	0.012077	0.09	0	0	0.43	0	0	0	0	0

18D00464.AGE >>> PS59-271-1-14 >>> ARCTIC | O-CONNOR (16-22) PROJECT

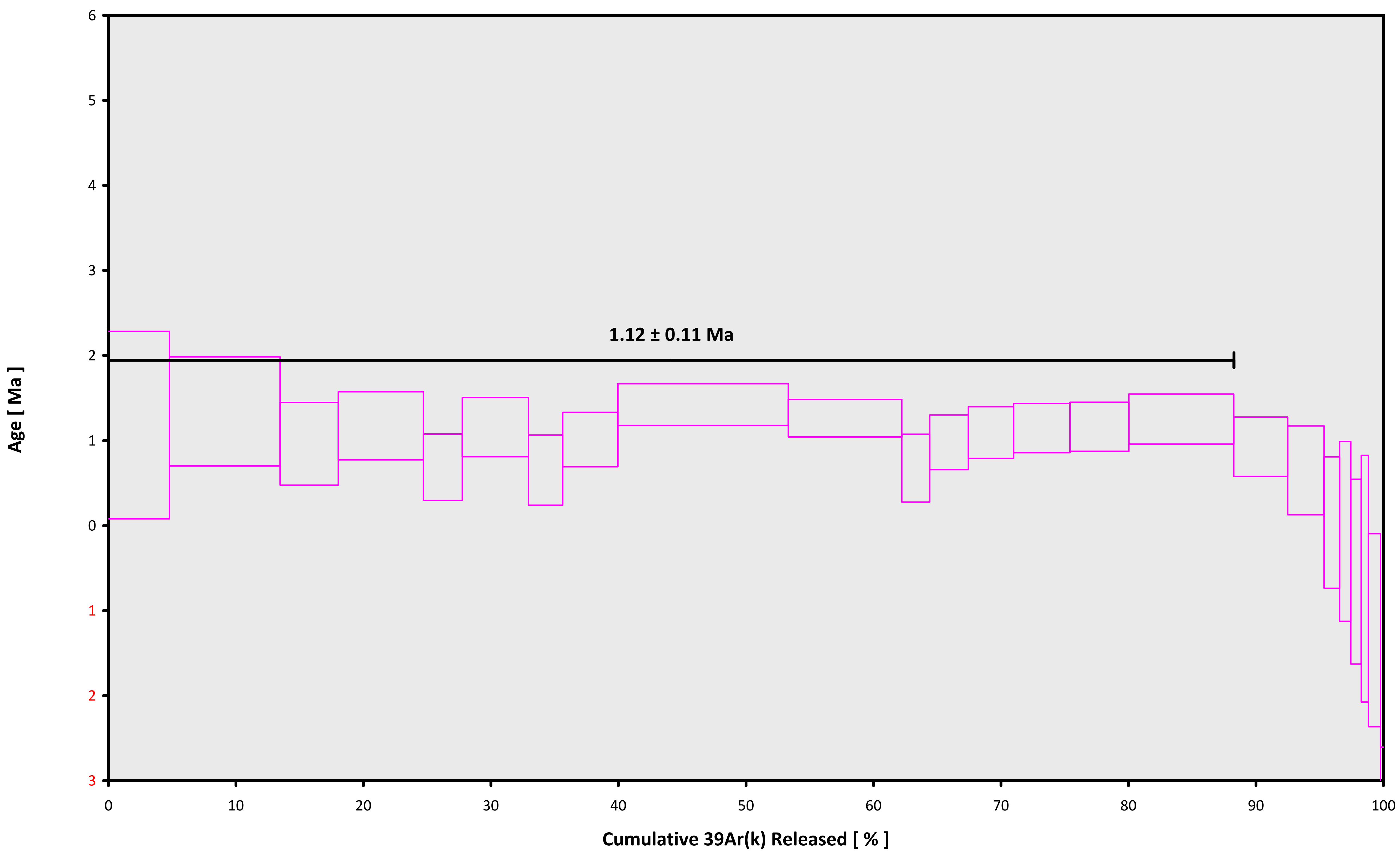

Ar-Ages in Ma

WEIGHTED PLATEAU
1.12 ± 0.11
TOTAL FUSION
1.06 ± 0.11
NORMAL ISOCHRON
1.13 ± 0.25
INVERSE ISOCHRON
1.14 ± 0.22

MSWD (PROBABILITY)
1.73 (4%)

Sample Info

Groundmass
Gakkel Ridge
Dan Miggins

IRR = 17-OSU-05 (5B9-17)
J = 0.00163273 ± 0.00000134

OSU Argon Geochronology Lab
CEOAS Oregon State University, Corvallis, USA

18D00464.AGE >>> PS59-271-1-14 >>> ARCTIC | O-CONNOR (16-22) PROJECT

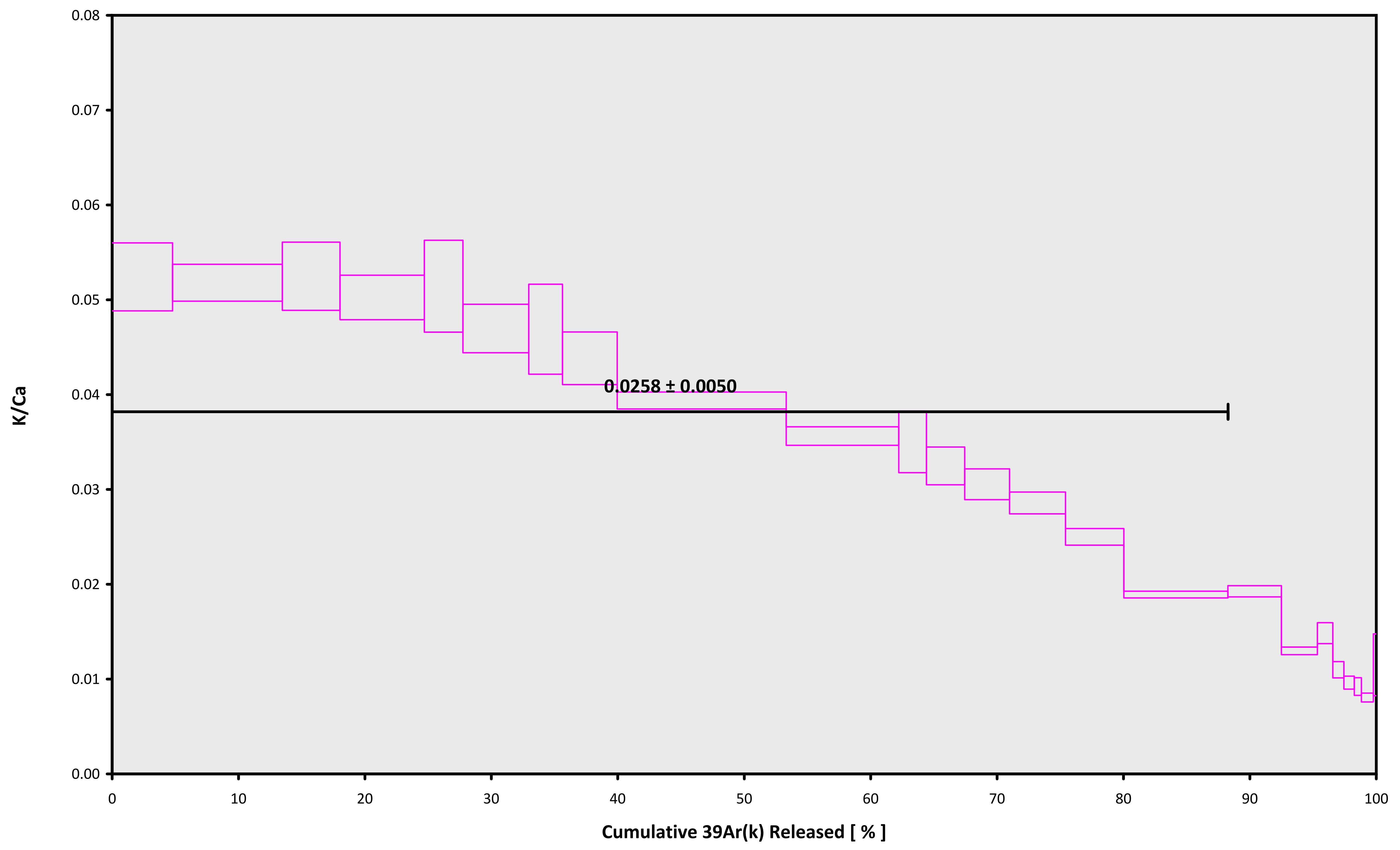

Ar-Ages in Ma

WEIGHTED PLATEAU
1.12 ± 0.11
TOTAL FUSION
1.06 ± 0.11
NORMAL ISOCHRON
1.13 ± 0.25
INVERSE ISOCHRON
1.14 ± 0.22

Sample Info

Groundmass
Gakkel Ridge
Dan Miggins

IRR = 17-OSU-05 (5B9-17)
J = 0.00163273 ± 0.00000134

18D00464.full.xls printed at 06-03-2018 (12:41)
ArArCALC v2.7.0 -- Beta Version

18D00464.AGE >>> PS59-271-1-14 >>> ARCTIC | O-CONNOR (16-22) PROJECT

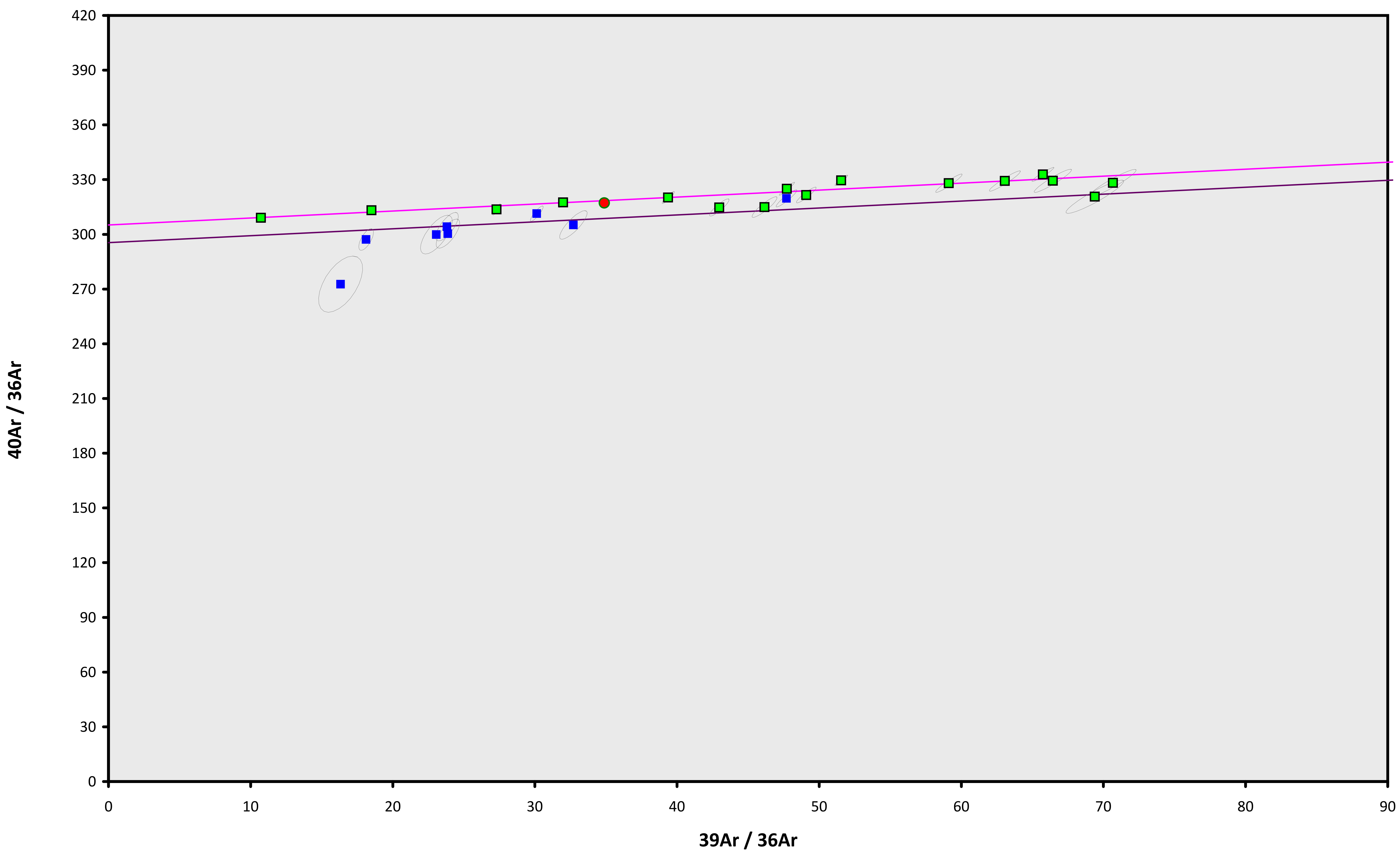

Ar-Ages in Ma

WEIGHTED PLATEAU
1.12 ± 0.11
TOTAL FUSION
1.06 ± 0.11
NORMAL ISOCHRON
1.13 ± 0.25
INVERSE ISOCHRON
1.14 ± 0.22

MSWD (PROBABILITY)
3.28 (0%)

40AR/36AR INTERCEPT
305.1 ± 3.4

Sample Info

Groundmass
Gakkel Ridge
Dan Miggins

IRR = 17-OSU-05 (5B9-17)
J = 0.00163273 ± 0.00000134

18D00464.AGE >>> PS59-271-1-14 >>> ARCTIC | O-CONNOR (16-22) PROJECT

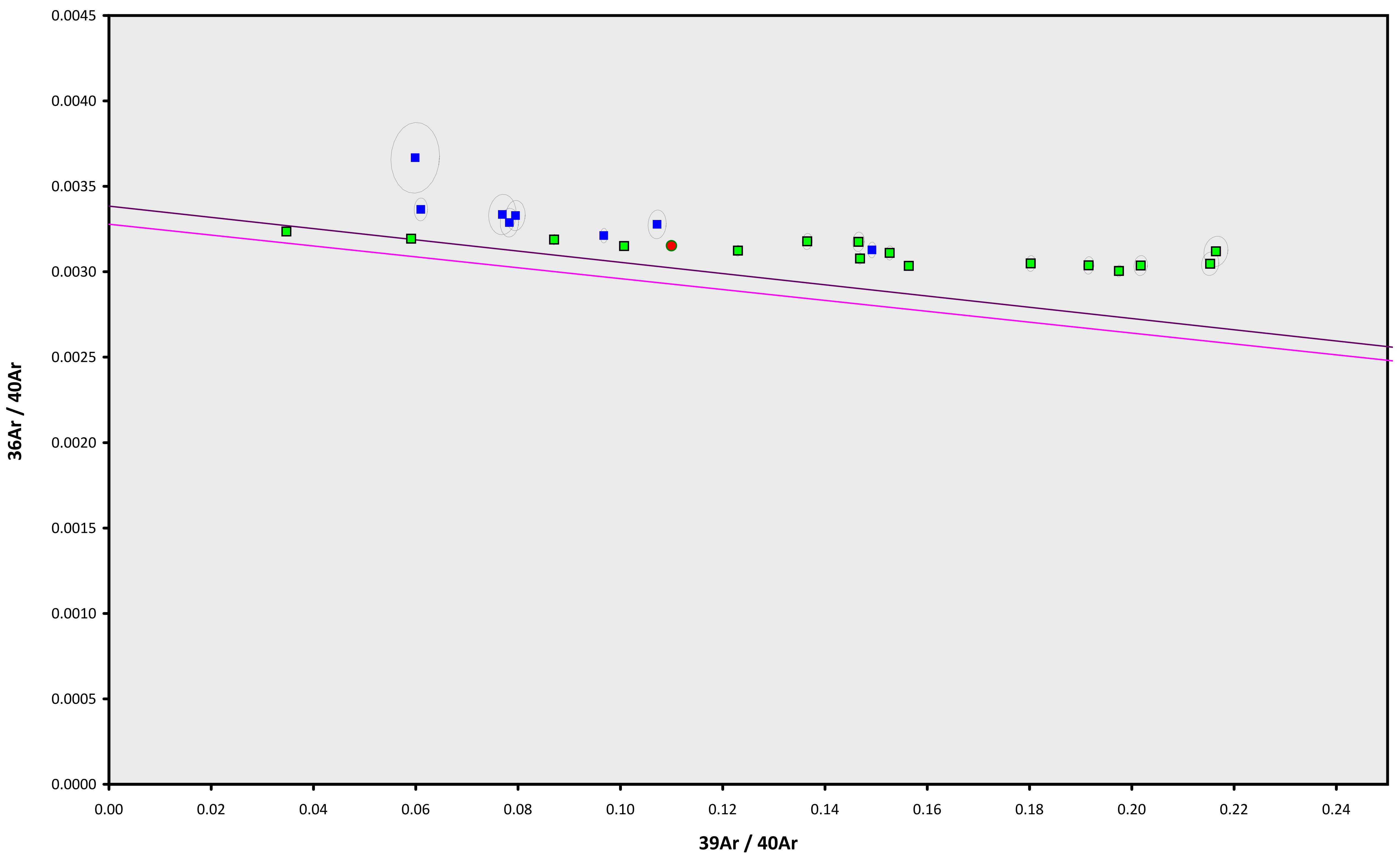

Ar-Ages in Ma

WEIGHTED PLATEAU
1.12 ± 0.11
TOTAL FUSION
1.06 ± 0.11
NORMAL ISOCHRON
1.13 ± 0.25
INVERSE ISOCHRON
1.14 ± 0.22

MSWD (PROBABILITY)
3.21 (0%)

SPREADING FACTOR
7.0%

40AR/36AR INTERCEPT
305.0 ± 3.4

Sample Info

Groundmass
Gakkel Ridge
Dan Miggins

IRR = 17-OSU-05 (5B9-17)
J = 0.00163273 ± 0.00000134

Relative Abundances			36Ar [fA]	%1σ	37Ar [fA]	%1σ	38Ar [fA]	%1σ	39Ar [fA]	%1σ	40Ar [fA]	%1σ	40(r)/39(k) ± 2σ	Age ± 2σ (ka)	40Ar(r) (%)	39Ar(k) (%)	K/Ca ± 2σ
17D20099	1.8 %		3.615282	0.276	95.5289	0.659	1.0177802	2.342	27.93507	0.107	1070.8322	0.006	0.36341 ± 0.21192	1084.5 ± 632.3	0.95	6.45	0.125 ± 0.002
17D20101	1.9 %		0.868731	0.318	23.7227	1.926	0.2764079	8.367	7.54453	0.316	257.7215	0.018	0.38560 ± 0.21740	1150.7 ± 648.6	1.13	1.74	0.136 ± 0.005
17D20102	2.0 %		0.711251	0.322	28.5626	1.567	0.2413332	9.722	8.70192	0.271	210.4185	0.022	0.29020 ± 0.15627	866.1 ± 466.3	1.20	2.01	0.131 ± 0.004
17D20104	2.2 %		0.586942	0.350	42.4297	1.110	0.2907988	8.171	11.80000	0.199	170.8724	0.026	0.06924 ± 0.10349	206.7 ± 308.9	0.48	2.72	0.119 ± 0.003
17D20105	2.4 %		0.455333	0.374	49.5489	1.027	0.2729821	8.678	13.17437	0.185	132.5167	0.034	0.14586 ± 0.07706	435.4 ± 230.0	1.45	3.04	0.114 ± 0.002
17D20107	2.7 %		0.372903	0.379	59.5060	0.858	0.2842190	8.558	15.26675	0.159	107.4053	0.042	0.12856 ± 0.05541	383.7 ± 165.4	1.82	3.52	0.110 ± 0.002
17D20108	3.0 %	✓	0.610679	0.334	212.5747	0.493	0.7379850	3.015	47.48991	0.084	166.0761	0.026	0.05433 ± 0.02580	162.2 ± 77.0	1.55	10.96	0.096 ± 0.001
17D20110	3.4 %	✓	0.403056	0.369	183.1456	0.520	0.5839905	3.975	39.65102	0.092	107.1789	0.042	0.06781 ± 0.02273	202.4 ± 67.9	2.50	9.15	0.093 ± 0.001
17D20111	3.9 %	✓	0.184360	0.499	75.9203	0.754	0.2187752	11.007	16.75270	0.145	49.5117	0.086	0.06509 ± 0.03339	194.3 ± 99.7	2.20	3.87	0.095 ± 0.001
17D20113	4.5 %	✓	0.303636	0.404	162.5587	0.529	0.4694823	4.831	34.30884	0.097	78.8568	0.052	0.06128 ± 0.02173	182.9 ± 64.8	2.66	7.91	0.090 ± 0.001
17D20114	5.2 %	✓	0.420822	0.388	272.7993	0.480	0.7855438	3.001	57.46746	0.078	106.2727	0.042	0.06414 ± 0.01734	191.5 ± 51.8	3.46	13.26	0.090 ± 0.001
17D20116	6.0 %	✓	0.195321	0.511	113.8195	0.609	0.3420718	6.539	23.82958	0.120	50.6596	0.085	0.08499 ± 0.02556	253.7 ± 76.3	3.99	5.50	0.090 ± 0.001
17D20117	6.9 %	✓	0.132468	0.569	69.9682	0.761	0.2158172	10.485	14.23649	0.171	34.4151	0.121	0.06001 ± 0.03251	179.1 ± 97.0	2.47	3.28	0.087 ± 0.001
17D20119	7.9 %	✓	0.296897	0.416	181.5283	0.511	0.4482414	5.049	31.20367	0.097	76.1696	0.056	0.09383 ± 0.02416	280.1 ± 72.1	3.83	7.19	0.074 ± 0.001
17D20120	9.0 %	✓	0.191288	0.510	105.4961	0.603	0.2278270	10.001	16.18731	0.158	49.8904	0.085	0.11051 ± 0.03676	329.9 ± 109.7	3.57	3.73	0.066 ± 0.001
17D20122	10.3 %	✓	0.144143	0.579	70.2438	0.739	0.1302032	17.556	10.38253	0.231	37.9999	0.109	0.09769 ± 0.04910	291.6 ± 146.5	2.66	2.39	0.063 ± 0.001
17D20123	11.6 %	✓	0.267655	0.432	121.9060	0.578	0.2399426	9.716	14.16764	0.179	69.8968	0.060	0.03787 ± 0.04955	113.0 ± 147.9	0.76	3.26	0.050 ± 0.001
17D20125	12.5 %	✓	0.347796	0.393	154.6859	0.542	0.2605949	8.623	13.56009	0.178	91.6493	0.047	0.09091 ± 0.06132	271.4 ± 183.0	1.34	3.11	0.037 ± 0.000
17D20126	13.4 %	✓	0.250270	0.449	98.2336	0.640	0.1399340	17.020	7.00289	0.338	66.5347	0.063	0.06080 ± 0.09768	181.5 ± 291.6	0.63	1.61	0.030 ± 0.000
17D20128	14.6 %	✓	0.180050	0.523	66.8480	0.834	0.1125604	20.890	3.98184	0.562	48.1195	0.086	0.06427 ± 0.14464	191.8 ± 431.7	0.53	0.91	0.025 ± 0.001
17D20129	16.0 %	✓	0.483154	0.353	229.4486	0.491	0.2135562	11.266	7.16666	0.313	124.4860	0.035	0.00532 ± 0.14665	15.9 ± 437.8	0.03	1.62	0.013 ± 0.000
17D20131	17.6 %	✓	0.147915	0.566	81.5523	0.702	0.0967794	23.702	2.62764	0.838	37.6018	0.110	0.15808 ± 0.19809	471.8 ± 591.2	1.08	0.60	0.014 ± 0.000
17D20132	19.3 %	✓	0.492775	0.348	292.9486	0.478	0.2010992	12.456	5.76124	0.409	123.2221	0.023	0.17993 ± 0.18720	537.1 ± 558.7	0.81	1.29	0.008 ± 0.000
17D20134	21.0 %		0.334444	0.393	245.0501	0.484	0.1570401	14.705	3.92413	0.585	80.6966	0.033	0.38236 ± 0.21370	1141.0 ± 637.5	1.78	0.87	0.007 ± 0.000
		Σ	11.997172	0.103	3038.0263	0.130	7.9649656	1.435	434.12429	0.031	3349.0043	0.006					

Information on Analysis and Constants Used in Calculations

Project = **O-CONNOR (16-22)**
Sample = **PS59-276-1-005**
Material = **Groundmass**
Location = **Gakkel Ridge**
Region = **Artic Ocean**
Analyst = **Dan Miggins**
Irradiation = **17-OSU-01 (1C11-17)**
Position = **X: 0 | Y: 0 | Z/H: 17.68106 mm**
FCT-NM Age = **28.201 ± 0.023 Ma**
FCT-NM Reference = **Kuiper et al (2008)**
FCT-NM 40Ar/39Ar Ratio = **9.52112 ± 0.00847**
FCT-NM J-value = **0.00165079 ± 0.00000147**
Air Shot 40Ar/36Ar = **302.6420 ± 0.3511**
Air Shot MDF = **0.99409266 ± 0.00064493 (LIN)**
Experiment Type = **Incremental Heating**
Extraction Method = **Bulk Laser Heating**
Heating = **64 sec**
Isolation = **3.00 min**
Instrument = **ARGUS-VI-D**
Preferred Age = **Plateau Age**
Age Classification = **Crystallization Age**
IGSN = Undefined
Rock Class = **Igneous>Volcanic**
Lithology = **Basaltic Lava**
Lat-Lon = Undefined - Undefined

Age Equations = **Min et al. (2000)**
Negative Intensities = **Allowed**
Collector Calibrations = **36Ar**
Decay 40K = **5.530 ± 0.048 E-10 1/a**
Decay 39Ar = **2.940 ± 0.016 E-07 1/h**
Decay 37Ar = **8.230 ± 0.012 E-04 1/h**
Decay 36Cl = **2.257 ± 0.015 E-06 1/a**
Decay 40K(EC,β^+) = **0.580 ± 0.009 E-10 1/a**
Decay 40K(β^-) = **4.950 ± 0.043 E-10 1/a**
Atmospheric 40/36(a) = **295.50**
Atmospheric 38/36(a) = **0.1869**
Production 39/37(ca) = **0.0006425 ± 0.0000059**
Production 38/37(ca) = **0.0001800 ± 0.0000173**
Production 36/37(ca) = **0.0002703 ± 0.0000005**
Production 40/39(k) = **0.000607 ± 0.000059**
Production 38/39(k) = **0.012077 ± 0.000011**
Production 36/38(cl) = **262.80 ± 1.71**
Scaling Ratio K/Ca = **0.430**
Abundance Ratio 40K/K = **1.1700 ± 0.0100 E-04**
Atomic Weight K = **39.0983 ± 0.0001 g**

Results

Results	40(a)/36(a) ± 2σ	40(r)/39(k) ± 2σ	Age ± 2σ (ka)	MSWD	39Ar(k) (%,n)	K/Ca ± 2σ
**Age Plateau**		0.07120 ± 0.00872 ± 12.25%	**212.5 ± 26.0 ± 12.25%**	1.22 24%	79.64 17	0.017 ± 0.009
			Full External Error ± 26.5	1.71	2σ Confidence Limit	
			Analytical Error ± 26.0	1.1047	Error Magnification	
**Total Fusion Age**		0.10702 ± 0.01693 ± 15.82%	**319.5 ± 50.5 ± 15.82%**		24	0.061 ± 0.000
			Full External Error ± 51.1			
			Analytical Error ± 50.5			
**Normal Isochron**	**295.68 ± 1.57 ± 0.53%**	0.06924 ± 0.01709 ± 24.68%	**206.7 ± 51.0 ± 24.68%**	1.29 20%	79.64 17	
			Full External Error ± 51.2	1.73	2σ Confidence Limit	
			Analytical Error ± 51.0	1.1337	Error Magnification	
				3	Number of Iterations	
				0.0000004103	Convergence	
**Inverse Isochron** Clustered Points	**295.70 ± 1.58 ± 0.53%**	0.06938 ± 0.01543 ± 22.24%	**207.1 ± 46.1 ± 22.24%**	1.30 19%	79.64 17	
			Full External Error ± 46.3	1.73	2σ Confidence Limit	
			Analytical Error ± 46.0	1.1386	Error Magnification	
				3	Number of Iterations	
				0.0000777587	Convergence	
				3%	Spreading Factor	

Incremental Heating			36Ar(a) [fA]	37Ar(ca) [fA]	38Ar(cl) [fA]	39Ar(k) [fA]	40Ar(r) [fA]	Age ± 2σ (ka)	40Ar(r) (%)	39Ar(k) (%)	K/Ca ± 2σ
17D20099	1.8 %		3.589461	95.5289	0.0000000	27.87369	10.129654	1084.5 ± 632.3	0.95	6.45	0.125 ± 0.002
17D20101	1.9 %		0.862314	23.7227	0.0200402	7.52928	2.903263	1150.7 ± 648.6	1.13	1.74	0.136 ± 0.005
17D20102	2.0 %		0.703530	28.5626	0.0000000	8.68357	2.519997	866.1 ± 466.3	1.20	2.01	0.131 ± 0.004
17D20104	2.2 %		0.575466	42.4297	0.0334275	11.77274	0.815121	206.7 ± 308.9	0.48	2.72	0.119 ± 0.003
17D20105	2.4 %		0.441935	49.5489	0.0227433	13.14253	1.916939	435.4 ± 230.0	1.45	3.04	0.114 ± 0.002
17D20107	2.7 %		0.356813	59.5060	0.0229048	15.22852	1.957844	383.7 ± 165.4	1.82	3.52	0.110 ± 0.002
17D20108	3.0 %	✓	0.553214	212.5747	0.0244397	47.35333	2.572487	162.2 ± 77.0	1.55	10.96	0.096 ± 0.001
17D20110	3.4 %	✓	0.353550	183.1456	0.0075015	39.53335	2.680721	202.4 ± 67.9	2.50	9.15	0.093 ± 0.001
17D20111	3.9 %	✓	0.163839	75.9203	0.0000000	16.70392	1.087217	194.3 ± 99.7	2.20	3.87	0.095 ± 0.001
17D20113	4.5 %	✓	0.259696	162.5587	0.0000000	34.20439	2.095898	182.9 ± 64.8	2.66	7.91	0.090 ± 0.001
17D20114	5.2 %	✓	0.347084	272.7993	0.0000000	57.29219	3.674605	191.5 ± 51.8	3.46	13.26	0.090 ± 0.001
17D20116	6.0 %	✓	0.164555	113.8195	0.0039223	23.75646	2.019177	253.7 ± 76.3	3.99	5.50	0.090 ± 0.001
17D20117	6.9 %	✓	0.113553	69.9682	0.0106088	14.19153	0.851681	179.1 ± 97.0	2.47	3.28	0.087 ± 0.001
17D20119	7.9 %	✓	0.247830	181.5283	0.0000000	31.08703	2.916892	280.1 ± 72.1	3.83	7.19	0.074 ± 0.001
17D20120	9.0 %	✓	0.162772	105.4961	0.0000000	16.11953	1.781419	329.9 ± 109.7	3.57	3.73	0.066 ± 0.001
17D20122	10.3 %	✓	0.125157	70.2438	0.0000000	10.33740	1.009869	291.6 ± 146.5	2.66	2.39	0.063 ± 0.001
17D20123	11.6 %	✓	0.234703	121.9060	0.0039769	14.08932	0.533552	113.0 ± 147.9	0.76	3.26	0.050 ± 0.001
17D20125	12.5 %	✓	0.305981	154.6859	0.0129986	13.46071	1.223751	271.4 ± 183.0	1.34	3.11	0.037 ± 0.000
17D20126	13.4 %	✓	0.223718	98.2336	0.0000000	6.93977	0.421917	181.5 ± 291.6	0.63	1.61	0.030 ± 0.000
17D20128	14.6 %	✓	0.161976	66.8480	0.0226844	3.93889	0.253150	191.8 ± 431.7	0.53	0.91	0.025 ± 0.001
17D20129	16.0 %	✓	0.421132	229.4486	0.0087745	7.01924	0.037345	15.9 ± 437.8	0.03	1.62	0.013 ± 0.000
17D20131	17.6 %	✓	0.125865	81.5523	0.0274746	2.57524	0.407102	471.8 ± 591.2	1.08	0.60	0.014 ± 0.000
17D20132	19.3 %	✓	0.413590	292.9486	0.0037631	5.57302	1.002779	537.1 ± 558.7	0.81	1.29	0.008 ± 0.000
17D20134	21.0 %		0.268203	245.0501	0.0173136	3.76669	1.440229	1141.0 ± 637.5	1.78	0.87	0.007 ± 0.000
		Σ	11.175937	3038.0263	0.2425738	432.17236	46.252609				

Information on Analysis

Project = **O-CONNOR (16-22)**
Sample = **PS59-276-1-005**
Material = **Groundmass**
Location = **Gakkel Ridge**
Region = **Artic Ocean**
Analyst = **Dan Miggins**
Irradiation = **17-OSU-01 (1C11-17)**
J = **0.00165079 ± 0.00000147**
FCT-NM = **28.201 ± 0.023 Ma**

Results	40(r)/39(k) ± 2σ		Age ± 2σ (ka)		MSWD	39Ar(k) (%,n)	K/Ca ± 2σ
**Age Plateau**	0.07120	± 0.00872<br>± 12.25%	**212.5**	**± 26.0**<br>**± 12.25%**	1.22<br>24%	79.64<br>17	0.017 ± 0.009
		Full External Error	± 26.5		1.71	2σ Confidence Limit	
		Analytical Error	± 26.0		1.1047	Error Magnification	
**Total Fusion Age**	0.10702	± 0.01693<br>± 15.82%	**319.5**	**± 50.5**<br>**± 15.82%**		24	0.061 ± 0.000
		Full External Error	± 51.1				
		Analytical Error	± 50.5				

Normal Isochron			39(k)/36(a) ± 2σ	40(a+r)/36(a) ± 2σ	r.i.
17D20099	1.8 %		7.77 ± 0.05	298.32 ± 1.66	0.9330
17D20101	1.9 %		8.73 ± 0.08	298.87 ± 1.92	0.7108
17D20102	2.0 %		12.34 ± 0.10	299.08 ± 1.95	0.7664
17D20104	2.2 %		20.46 ± 0.17	296.92 ± 2.13	0.8706
17D20105	2.4 %		29.74 ± 0.25	299.84 ± 2.33	0.8978
17D20107	2.7 %		42.68 ± 0.37	300.99 ± 2.41	0.9232
17D20108	3.0 %	✓	85.60 ± 0.65	300.15 ± 2.24	0.9732
17D20110	3.4 %	✓	111.82 ± 0.98	303.08 ± 2.61	0.9731
17D20111	3.9 %	✓	101.95 ± 1.20	302.14 ± 3.48	0.9579
17D20113	4.5 %	✓	131.71 ± 1.29	303.57 ± 2.94	0.9746
17D20114	5.2 %	✓	165.07 ± 1.61	306.09 ± 2.96	0.9833
17D20116	6.0 %	✓	144.37 ± 1.82	307.77 ± 3.84	0.9724
17D20117	6.9 %	✓	124.98 ± 1.74	303.00 ± 4.16	0.9543
17D20119	7.9 %	✓	125.44 ± 1.30	307.27 ± 3.15	0.9764
17D20120	9.0 %	✓	99.03 ± 1.25	306.44 ± 3.77	0.9584
17D20122	10.3 %	✓	82.60 ± 1.18	303.57 ± 4.16	0.9339
17D20123	11.6 %	✓	60.03 ± 0.64	297.77 ± 3.00	0.9338
17D20125	12.5 %	✓	43.99 ± 0.43	299.50 ± 2.73	0.9248
17D20126	13.4 %	✓	31.02 ± 0.38	297.39 ± 3.05	0.8242
17D20128	14.6 %	✓	24.32 ± 0.40	297.06 ± 3.54	0.7122
17D20129	16.0 %	✓	16.67 ± 0.17	295.59 ± 2.45	0.7871
17D20131	17.6 %	✓	20.46 ± 0.45	298.73 ± 4.10	0.6123
17D20132	19.3 %	✓	13.47 ± 0.16	297.92 ± 2.54	0.7073
17D20134	21.0 %		14.04 ± 0.22	300.87 ± 3.05	0.6370

Results	40(a)/36(a) ± 2σ	40(r)/39(k) ± 2σ	Age ± 2σ (ka)	MSWD
**Normal Isochron**	**295.68 ± 1.57 ± 0.53%**	0.06924 ± 0.01709 ± 24.68%	**206.7 ± 51.0 ± 24.68%**	1.29 20%
			Full External Error ± 51.2	
			Analytical Error ± 51.0	

Statistics				
	2σ Confidence Limit	1.73	Convergence	0.000000410263
	Error Magnification	1.1337	Number of Iterations	3
	Number of Data Points	17	Calculated Line	Weighted York-2

Inverse Isochron			39(k)/40(a+r) ± 2σ	36(a)/40(a+r) ± 2σ	r.i.
17D20099	1.8 %		0.0260303 ± 0.0000559	0.00335208 ± 0.00001867	0.0011
17D20101	1.9 %		0.0292153 ± 0.0001851	0.00334597 ± 0.00002149	0.0030
17D20102	2.0 %		0.0412691 ± 0.0002247	0.00334357 ± 0.00002182	0.0055
17D20104	2.2 %		0.0689007 ± 0.0002774	0.00336795 ± 0.00002413	0.0095
17D20105	2.4 %		0.0991824 ± 0.0003743	0.00333514 ± 0.00002586	0.0156
17D20107	2.7 %		0.1417977 ± 0.0004671	0.00332240 ± 0.00002658	0.0268
17D20108	3.0 %	✓	0.2851796 ± 0.0005016	0.00333167 ± 0.00002490	0.0210
17D20110	3.4 %	✓	0.3689366 ± 0.0007446	0.00329943 ± 0.00002838	0.0399
17D20111	3.9 %	✓	0.3374420 ± 0.0011424	0.00330977 ± 0.00003811	0.0757
17D20113	4.5 %	✓	0.4338674 ± 0.0009561	0.00329413 ± 0.00003189	0.0506
17D20114	5.2 %	✓	0.5392820 ± 0.0009590	0.00326704 ± 0.00003164	0.0416
17D20116	6.0 %	✓	0.4690767 ± 0.0013838	0.00324917 ± 0.00004054	0.0781
17D20117	6.9 %	✓	0.4124666 ± 0.0017281	0.00330033 ± 0.00004534	0.1016
17D20119	7.9 %	✓	0.4082302 ± 0.0009153	0.00325447 ± 0.00003336	0.0538
17D20120	9.0 %	✓	0.3231621 ± 0.0011648	0.00326324 ± 0.00004017	0.0657
17D20122	10.3 %	✓	0.2720823 ± 0.0013960	0.00329415 ± 0.00004517	0.0675
17D20123	11.6 %	✓	0.2015977 ± 0.0007668	0.00335826 ± 0.00003380	0.0381
17D20125	12.5 %	✓	0.1468850 ± 0.0005457	0.00333890 ± 0.00003048	0.0261
17D20126	13.4 %	✓	0.1043096 ± 0.0007240	0.00336263 ± 0.00003448	0.0220
17D20128	14.6 %	✓	0.0818606 ± 0.0009405	0.00336629 ± 0.00004006	0.0216
17D20129	16.0 %	✓	0.0563877 ± 0.0003629	0.00338308 ± 0.00002798	0.0093
17D20131	17.6 %	✓	0.0684900 ± 0.0011815	0.00334745 ± 0.00004590	0.0205
17D20132	19.3 %	✓	0.0452287 ± 0.0003847	0.00335655 ± 0.00002865	0.0029
17D20134	21.0 %		0.0466785 ± 0.0005710	0.00332370 ± 0.00003375	0.0036

Results	40(a)/36(a) ± 2σ	40(r)/39(k) ± 2σ	Age ± 2σ (ka)	MSWD
**Inverse Isochron** Clustered Points	**295.70** **± 1.58** **± 0.53%**	0.06938 ± 0.01543 ± 22.24%	**207.1** **± 46.1** **± 22.24%**	1.30 19%
			Full External Error ± 46.3	
			Analytical Error ± 46.0	

Statistics				
	2σ Confidence Limit	1.73	Convergence	0.0000777587
	Error Magnification	1.1386	Number of Iterations	3
	Number of Data Points	17	Calculated Line	Weighted York-2
	Spreading Factor	3.4%		

Degassing Patterns			36Ar(a) [fA]	%1σ	36Ar(c) [fA]	%1σ	36Ar(ca) [fA]	%1σ	36Ar(cl) [fA]	%1σ	37Ar(ca) [fA]	%1σ	38Ar(a) [fA]	%1σ	38Ar(c) [fA]	%1σ	38Ar(k) [fA]	%1σ	38Ar(ca) [fA]	%1σ	38Ar(cl) [fA]	%1σ	39Ar(k) [fA]	%1σ	39Ar(ca) [fA]	%1σ	40Ar(r) [fA]	%1σ	40Ar(a) [fA]	%1σ	40Ar(c) [fA]	%1σ	40Ar(k) [fA]	%1σ
17D20099	1.8 %		3.589461	0.28	0.0000000	0.00	0.0258215	0.68	0.0000000	0.00	95.5289	0.66	0.6708702	0.28	0.0000000	0.00	0.3366306	0.14	0.0171952	9.65	0.0000000	0.00	27.87369	0.11	0.0613773	1.13	10.129654	29.16	1060.6856	0.28	0.0000000	0.00	0.0169193	9.65
17D20101	1.9 %		0.862314	0.32	0.0000000	0.00	0.0064122	1.93	0.0000047	115.46	23.7227	1.93	0.1611664	0.32	0.0000000	0.00	0.0909312	0.33	0.0042701	9.82	0.0200402	115.47	7.52928	0.32	0.0152418	2.13	2.903263	28.19	254.8137	0.32	0.0000000	0.00	0.0045703	9.66
17D20102	2.0 %		0.703530	0.33	0.0000000	0.00	0.0077205	1.58	0.0000000	0.00	28.5626	1.57	0.1314898	0.33	0.0000000	0.00	0.1048715	0.29	0.0051413	9.76	0.0000000	0.00	8.68357	0.27	0.0183515	1.82	2.519997	26.92	207.8932	0.33	0.0000000	0.00	0.0052709	9.65
17D20104	2.2 %		0.575466	0.36	0.0000000	0.00	0.0114687	1.12	0.0000078	71.14	42.4297	1.11	0.1075545	0.36	0.0000000	0.00	0.1421794	0.22	0.0076373	9.69	0.0334275	71.15	11.77274	0.20	0.0272611	1.44	0.815121	74.74	170.0501	0.36	0.0000000	0.00	0.0071461	9.65
17D20105	2.4 %		0.441935	0.39	0.0000000	0.00	0.0133931	1.04	0.0000053	104.26	49.5489	1.03	0.0825976	0.39	0.0000000	0.00	0.1587224	0.21	0.0089188	9.68	0.0227433	104.26	13.14253	0.19	0.0318352	1.38	1.916939	26.42	130.5918	0.39	0.0000000	0.00	0.0079775	9.65
17D20107	2.7 %		0.356813	0.40	0.0000000	0.00	0.0160845	0.87	0.0000054	106.32	59.5060	0.86	0.0666883	0.40	0.0000000	0.00	0.1839148	0.18	0.0107111	9.67	0.0229048	106.32	15.22852	0.16	0.0382326	1.26	1.957844	21.55	105.4382	0.40	0.0000000	0.00	0.0092437	9.65
17D20108	3.0 %	✓	0.553214	0.37	0.0000000	0.00	0.0574589	0.52	0.0000057	92.35	212.5747	0.49	0.1033958	0.37	0.0000000	0.00	0.5718861	0.12	0.0382634	9.64	0.0244397	92.35	47.35333	0.08	0.1365792	1.04	2.572487	23.75	163.4749	0.37	0.0000000	0.00	0.0287435	9.65
17D20110	3.4 %	✓	0.353550	0.43	0.0000000	0.00	0.0495043	0.55	0.0000018	312.50	183.1456	0.52	0.0660786	0.43	0.0000000	0.00	0.4774443	0.13	0.0329662	9.64	0.0075015	312.50	39.53335	0.09	0.1176710	1.06	2.680721	16.76	104.4741	0.43	0.0000000	0.00	0.0239967	9.65
17D20111	3.9 %	✓	0.163839	0.57	0.0000000	0.00	0.0205213	0.77	0.0000000	0.00	75.9203	0.75	0.0306215	0.57	0.0000000	0.00	0.2017333	0.17	0.0136657	9.66	0.0000000	0.00	16.70392	0.15	0.0487788	1.19	1.087217	25.65	48.4144	0.57	0.0000000	0.00	0.0101393	9.65
17D20113	4.5 %	✓	0.259696	0.48	0.0000000	0.00	0.0439396	0.56	0.0000000	0.00	162.5587	0.53	0.0485372	0.48	0.0000000	0.00	0.4130864	0.13	0.0292606	9.64	0.0000000	0.00	34.20439	0.10	0.1044440	1.06	2.095898	17.73	76.7402	0.48	0.0000000	0.00	0.0207621	9.65
17D20114	5.2 %	✓	0.347084	0.48	0.0000000	0.00	0.0737377	0.51	0.0000000	0.00	272.7993	0.48	0.0648700	0.48	0.0000000	0.00	0.6919177	0.12	0.0491039	9.64	0.0000000	0.00	57.29219	0.08	0.1752735	1.04	3.674605	13.52	102.5633	0.48	0.0000000	0.00	0.0347764	9.65
17D20116	6.0 %	✓	0.164555	0.62	0.0000000	0.00	0.0307654	0.63	0.0000009	572.62	113.8195	0.61	0.0307553	0.62	0.0000000	0.00	0.2869067	0.15	0.0204875	9.65	0.0039223	572.62	23.75646	0.12	0.0731290	1.10	2.019177	15.04	48.6260	0.62	0.0000000	0.00	0.0144202	9.65
17D20117	6.9 %	✓	0.113553	0.68	0.0000000	0.00	0.0189124	0.78	0.0000025	213.65	69.9682	0.76	0.0212230	0.68	0.0000000	0.00	0.1713912	0.19	0.0125943	9.66	0.0106088	213.66	14.19153	0.17	0.0449546	1.19	0.851681	27.08	33.5548	0.68	0.0000000	0.00	0.0086143	9.65
17D20119	7.9 %	✓	0.247830	0.51	0.0000000	0.00	0.0490671	0.54	0.0000000	0.00	181.5283	0.51	0.0463195	0.51	0.0000000	0.00	0.3754381	0.13	0.0326751	9.64	0.0000000	0.00	31.08703	0.10	0.1166320	1.05	2.916892	12.88	73.2339	0.51	0.0000000	0.00	0.0188698	9.65
17D20120	9.0 %	✓	0.162772	0.61	0.0000000	0.00	0.0285156	0.63	0.0000000	0.00	105.4961	0.60	0.0304222	0.61	0.0000000	0.00	0.1946756	0.18	0.0189893	9.65	0.0000000	0.00	16.11953	0.16	0.0677812	1.10	1.781419	16.63	48.0992	0.61	0.0000000	0.00	0.0097846	9.65
17D20122	10.3 %	✓	0.125157	0.68	0.0000000	0.00	0.0189869	0.76	0.0000000	0.00	70.2438	0.74	0.0233918	0.68	0.0000000	0.00	0.1248447	0.25	0.0126439	9.66	0.0000000	0.00	10.33740	0.23	0.0451317	1.18	1.009869	25.13	36.9838	0.68	0.0000000	0.00	0.0062748	9.65
17D20123	11.6 %	✓	0.234703	0.50	0.0000000	0.00	0.0329512	0.60	0.0000009	588.71	121.9060	0.58	0.0438660	0.50	0.0000000	0.00	0.1701567	0.20	0.0219431	9.65	0.0039769	588.71	14.08932	0.18	0.0783246	1.09	0.533552	65.42	69.3547	0.50	0.0000000	0.00	0.0085522	9.65
17D20125	12.5 %	✓	0.305981	0.45	0.0000000	0.00	0.0418116	0.57	0.0000030	174.13	154.6859	0.54	0.0571878	0.45	0.0000000	0.00	0.1625650	0.20	0.0278435	9.65	0.0129986	174.14	13.46071	0.18	0.0993857	1.07	1.223751	33.73	90.4174	0.45	0.0000000	0.00	0.0081706	9.65
17D20126	13.4 %	✓	0.223718	0.51	0.0000000	0.00	0.0265525	0.66	0.0000000	0.00	98.2336	0.64	0.0418128	0.51	0.0000000	0.00	0.0838116	0.35	0.0176821	9.65	0.0000000	0.00	6.93977	0.34	0.0631151	1.12	0.421917	80.33	66.1086	0.51	0.0000000	0.00	0.0042124	9.66
17D20128	14.6 %	✓	0.161976	0.59	0.0000000	0.00	0.0180690	0.85	0.0000053	103.80	66.8480	0.83	0.0302733	0.59	0.0000000	0.00	0.0475700	0.58	0.0120326	9.67	0.0226844	103.81	3.93889	0.57	0.0429498	1.24	0.253150	112.52	47.8639	0.59	0.0000000	0.00	0.0023909	9.67
17D20129	16.0 %	✓	0.421132	0.41	0.0000000	0.00	0.0620199	0.52	0.0000021	277.98	229.4486	0.49	0.0787095	0.41	0.0000000	0.00	0.0847714	0.33	0.0413007	9.64	0.0087745	277.99	7.01924	0.32	0.1474207	1.04	0.037345	######	124.4444	0.41	0.0000000	0.00	0.0042607	9.66
17D20131	17.6 %	✓	0.125865	0.68	0.0000000	0.00	0.0220436	0.72	0.0000064	83.67	81.5523	0.70	0.0235242	0.68	0.0000000	0.00	0.0311012	0.86	0.0146794	9.66	0.0274746	83.67	2.57524	0.86	0.0523973	1.16	0.407102	62.65	37.1932	0.68	0.0000000	0.00	0.0015632	9.69
17D20132	19.3 %	✓	0.413590	0.43	0.0000000	0.00	0.0791840	0.51	0.0000009	679.36	292.9486	0.48	0.0773000	0.43	0.0000000	0.00	0.0673053	0.43	0.0527307	9.64	0.0037631	679.36	5.57302	0.42	0.1882195	1.04	1.002779	52.02	122.2160	0.43	0.0000000	0.00	0.0033828	9.66
17D20134	21.0 %		0.268203	0.51	0.0000000	0.00	0.0662370	0.51	0.0000041	135.65	245.0501	0.48	0.0501272	0.51	0.0000000	0.00	0.0454903	0.62	0.0441090	9.64	0.0173136	135.66	3.76669	0.61	0.1574447	1.04	1.440229	27.94	79.2541	0.51	0.0000000	0.00	0.0022864	9.67
		Σ	11.175937	0.11	0.0000000	0.00	0.8211785	0.14	0.0000567	37.51	3038.0263	0.13	2.0887826	0.11	0.0000000	0.00	5.2193456	0.04	0.5468447	2.31	0.2425738	37.51	432.17236	0.03	1.9519319	0.26	46.252609	7.91	3302.4893	0.11	0.0000000	0.00	0.2623286	2.51
		Σ							11.997172	0.10	3038.0263	0.13									8.0975467	1.13			434.12429	0.03							3349.0043	0.15

**Additional Parameters**			**40Ar/39Ar**	**1σ**	**37Ar/39Ar**	**1σ**	**36Ar/39Ar**	**1σ**	**Time (days)**	**37Ar (decay)**	**39Ar (decay)**	**40Ar (moles)**
17D20099	1.8 %		38.332897	0.041039	3.419676	0.022844	0.129417	0.000383	143.875	17.189298	1.00101658	5.140E-11
17D20101	1.9 %		34.160062	0.108011	3.144358	0.061364	0.115147	0.000516	143.889	17.194014	1.00101668	1.237E-11
17D20102	2.0 %		24.180685	0.065696	3.282331	0.052187	0.081735	0.000344	143.896	17.196373	1.00101673	1.010E-11
17D20104	2.2 %		14.480706	0.029085	3.595733	0.040546	0.049741	0.000200	143.910	17.201091	1.00101683	8.202E-12
17D20105	2.4 %		10.058672	0.018935	3.761007	0.039266	0.034562	0.000144	143.917	17.203451	1.00101687	6.361E-12
17D20107	2.7 %		7.035245	0.011557	3.897753	0.034007	0.024426	0.000100	143.931	17.208171	1.00101697	5.155E-12
17D20108	3.0 %	✓	3.497082	0.003065	4.476208	0.022369	0.012859	0.000044	143.938	17.210531	1.00101702	7.972E-12
17D20110	3.4 %	✓	2.703054	0.002719	4.618937	0.024375	0.010165	0.000039	143.951	17.215253	1.00101712	5.145E-12
17D20111	3.9 %	✓	2.955448	0.004990	4.531826	0.034806	0.011005	0.000057	143.958	17.217615	1.00101717	2.377E-12
17D20113	4.5 %	✓	2.298440	0.002524	4.738102	0.025500	0.008850	0.000037	143.972	17.222339	1.00101727	3.785E-12
17D20114	5.2 %	✓	1.849267	0.001638	4.747022	0.023085	0.007323	0.000029	143.979	17.224701	1.00101732	5.101E-12
17D20116	6.0 %	✓	2.125910	0.003128	4.776394	0.029645	0.008197	0.000043	143.993	17.229427	1.00101741	2.432E-12
17D20117	6.9 %	✓	2.417388	0.005052	4.914709	0.038333	0.009305	0.000055	144.000	17.231791	1.00101746	1.652E-12
17D20119	7.9 %	✓	2.441047	0.002726	5.817532	0.030270	0.009515	0.000041	144.014	17.236519	1.00101756	3.656E-12
17D20120	9.0 %	✓	3.082070	0.005534	6.517205	0.040656	0.011817	0.000063	144.021	17.238883	1.00101761	2.395E-12
17D20122	10.3 %	✓	3.659986	0.009353	6.765582	0.052400	0.013883	0.000087	144.035	17.243613	1.00101771	1.824E-12
17D20123	11.6 %	✓	4.933555	0.009331	8.604536	0.052093	0.018892	0.000088	144.042	17.245978	1.00101776	3.355E-12
17D20125	12.5 %	✓	6.758751	0.012458	11.407434	0.065073	0.025648	0.000111	144.056	17.250710	1.00101786	4.399E-12
17D20126	13.4 %	✓	9.501044	0.032672	14.027593	0.101477	0.035738	0.000201	144.063	17.253077	1.00101790	3.194E-12
17D20128	14.6 %	✓	12.084717	0.068670	16.788198	0.168824	0.045218	0.000347	144.076	17.257810	1.00101800	2.310E-12
17D20129	16.0 %	✓	17.370152	0.054638	32.016091	0.186308	0.067417	0.000318	144.083	17.260178	1.00101805	5.975E-12
17D20131	17.6 %	✓	14.310109	0.120966	31.036321	0.339388	0.056292	0.000569	144.097	17.264913	1.00101815	1.805E-12
17D20132	19.3 %	✓	21.388134	0.087707	50.848203	0.320121	0.085533	0.000460	144.104	17.267282	1.00101820	5.915E-12
17D20134	21.0 %		20.564175	0.120438	62.446905	0.474169	0.085228	0.000601	144.119	17.272256	1.00101830	3.873E-12

**Procedure Blanks**		36Ar ± 1σ (SE) [fA]	37Ar ± 1σ (SE) [fA]	38Ar ± 1σ (SE) [fA]	39Ar ± 1σ (SE) [fA]	40Ar ± 1σ (SE) [fA]
17D20099	1.8 %	0.0076258 ± 0.0004163	0.1377685 ± 0.0175623	0.0396360 ± 0.0163559	0.0172676 ± 0.0156393	2.1872472 ± 0.0371003
17D20101	1.9 %	0.0075701 ± 0.0004163	0.1429957 ± 0.0175623	0.0502937 ± 0.0163559	0.0191818 ± 0.0156393	2.1769954 ± 0.0371003
17D20102	2.0 %	0.0075902 ± 0.0004163	0.1445318 ± 0.0175623	0.0536747 ± 0.0163559	0.0192928 ± 0.0156393	2.1820655 ± 0.0371003
17D20104	2.2 %	0.0076862 ± 0.0004163	0.1458619 ± 0.0175623	0.0572134 ± 0.0163559	0.0183086 ± 0.0156393	2.2031740 ± 0.0371003
17D20105	2.4 %	0.0077484 ± 0.0004163	0.1458111 ± 0.0175623	0.0576267 ± 0.0163559	0.0173912 ± 0.0156393	2.2160212 ± 0.0371003
17D20107	2.7 %	0.0078728 ± 0.0004163	0.1446416 ± 0.0175623	0.0563469 ± 0.0163559	0.0151086 ± 0.0156393	2.2397694 ± 0.0371003
17D20108	3.0 %	0.0079259 ± 0.0004163	0.1436584 ± 0.0175623	0.0548827 ± 0.0163559	0.0138880 ± 0.0156393	2.2486358 ± 0.0371003
17D20110	3.4 %	0.0079973 ± 0.0004163	0.1412096 ± 0.0175623	0.0508452 ± 0.0163559	0.0116076 ± 0.0156393	2.2567122 ± 0.0371003
17D20111	3.9 %	0.0080112 ± 0.0004163	0.1398598 ± 0.0175623	0.0484741 ± 0.0163559	0.0106592 ± 0.0156393	2.2550442 ± 0.0371003
17D20113	4.5 %	0.0079902 ± 0.0004163	0.1371748 ± 0.0175623	0.0434999 ± 0.0163559	0.0093826 ± 0.0156393	2.2395465 ± 0.0371003
17D20114	5.2 %	0.0079556 ± 0.0004163	0.1359358 ± 0.0175623	0.0410725 ± 0.0163559	0.0091325 ± 0.0156393	2.2259953 ± 0.0371003
17D20116	6.0 %	0.0078449 ± 0.0004163	0.1338807 ± 0.0175623	0.0367430 ± 0.0163559	0.0095619 ± 0.0156393	2.1894300 ± 0.0371003
17D20117	6.9 %	0.0077736 ± 0.0004163	0.1331412 ± 0.0175623	0.0349900 ± 0.0163559	0.0102863 ± 0.0156393	2.1678512 ± 0.0371003
17D20119	7.9 %	0.0076176 ± 0.0004163	0.1324051 ± 0.0175623	0.0326470 ± 0.0163559	0.0128249 ± 0.0156393	2.1231342 ± 0.0371003
17D20120	9.0 %	0.0075425 ± 0.0004163	0.1324653 ± 0.0175623	0.0321794 ± 0.0163559	0.0146507 ± 0.0156393	2.1025879 ± 0.0371003
17D20122	10.3 %	0.0074277 ± 0.0004163	0.1335598 ± 0.0175623	0.0329248 ± 0.0163559	0.0194024 ± 0.0156393	2.0730442 ± 0.0371003
17D20123	11.6 %	0.0074024 ± 0.0004163	0.1346314 ± 0.0175623	0.0342337 ± 0.0163559	0.0223066 ± 0.0156393	2.0677950 ± 0.0371003
17D20125	12.5 %	0.0074573 ± 0.0004163	0.1378914 ± 0.0175623	0.0389299 ± 0.0163559	0.0290762 ± 0.0156393	2.0871585 ± 0.0371003
17D20126	13.4 %	0.0075565 ± 0.0004163	0.1400974 ± 0.0175623	0.0423865 ± 0.0163559	0.0328865 ± 0.0156393	2.1166759 ± 0.0371003
17D20128	14.6 %	0.0079514 ± 0.0004163	0.1456805 ± 0.0175623	0.0516561 ± 0.0163559	0.0411794 ± 0.0156393	2.2290896 ± 0.0371003
17D20129	16.0 %	0.0082708 ± 0.0004163	0.1490555 ± 0.0175623	0.0575118 ± 0.0163559	0.0455738 ± 0.0156393	2.3180473 ± 0.0371003
17D20131	17.6 %	0.0092182 ± 0.0004163	0.1569422 ± 0.0175623	0.0717378 ± 0.0163559	0.0545964 ± 0.0156393	2.5780634 ± 0.0371003
17D20132	19.3 %	0.0088795 ± 0.0001776	0.1449952 ± 0.0182633	0.0654442 ± 0.0185440	0.0457354 ± 0.0152372	2.4506788 ± 0.0167840
17D20134	21.0 %	0.0075105 ± 0.0001709	0.1498792 ± 0.0157159	0.0408852 ± 0.0154690	0.0421557 ± 0.0159805	2.2167936 ± 0.0160762

**Intercept Values**		**36Ar ± 1σ (SE) [fA]**	**r2**	**Regression (type,n)**	**37Ar ± 1σ (SE) [fA]**	**r2**	**Regression (type,n)**	**38Ar ± 1σ (SE) [fA]**	**r2**	**Regression (type,n)**	**39Ar ± 1σ (SE) [fA]**	**r2**	**Regression (type,n)**	**40Ar ± 1σ (SE) [fA]**	**r2**	**Regression (type,n)**
17D20099	1.8 %	3.4854502 ± 0.0028272	0.9874	EXP 150 of 150	5.321349 ± 0.019471	0.6821	EXP 150 of 150	0.9661215 ± 0.0168958	0.0684	EXP 150 of 150	27.7248982 ± 0.0176696	0.9890	EXP 150 of 150	1073.019462 ± 0.050151	0.9998	EXP 150 of 150
17D20101	1.9 %	0.8432703 ± 0.0014233	0.9410	EXP 150 of 150	1.212296 ± 0.018312	0.1774	EXP 150 of 150	0.2228491 ± 0.0159560	0.0081	EXP 150 of 150	7.4732455 ± 0.0170699	0.8650	EXP 150 of 150	259.898493 ± 0.025924	0.9982	EXP 150 of 150
17D20102	2.0 %	0.6917983 ± 0.0011868	0.9355	EXP 150 of 150	1.487042 ± 0.017048	0.1453	EXP 150 of 150	0.1848077 ± 0.0164304	0.0008	EXP 150 of 150	8.6225388 ± 0.0164830	0.8939	EXP 149 of 150	212.600549 ± 0.028292	0.9952	EXP 150 of 150
17D20104	2.2 %	0.5723122 ± 0.0012262	0.8981	EXP 150 of 150	2.277172 ± 0.017173	0.3676	EXP 150 of 150	0.2301503 ± 0.0168439	0.0170	EXP 150 of 150	11.7002071 ± 0.0155699	0.9499	EXP 150 of 150	173.075558 ± 0.024970	0.9914	EXP 150 of 150
17D20105	2.4 %	0.4457693 ± 0.0010812	0.8658	EXP 150 of 150	2.683393 ± 0.019316	0.3917	EXP 150 of 150	0.2121308 ± 0.0167454	0.0160	EXP 150 of 150	13.0659993 ± 0.0164506	0.9584	EXP 150 of 150	134.732691 ± 0.025159	0.9451	EXP 150 of 150
17D20107	2.7 %	0.3665973 ± 0.0008815	0.8497	EXP 150 of 150	3.252175 ± 0.017468	0.5362	EXP 150 of 150	0.2245148 ± 0.0176100	0.0199	EXP 150 of 150	15.1462102 ± 0.0154527	0.9741	EXP 150 of 150	109.645088 ± 0.025714	0.2585	EXP 150 of 150
17D20108	3.0 %	0.5953862 ± 0.0011293	0.9216	EXP 150 of 150	11.989201 ± 0.016057	0.9462	EXP 150 of 150	0.6743848 ± 0.0146620	0.1291	EXP 149 of 150	47.1480575 ± 0.0194649	0.9960	EXP 150 of 150	168.324748 ± 0.022725	0.9894	EXP 150 of 150
17D20110	3.4 %	0.3957289 ± 0.0009117	0.8673	EXP 150 of 150	10.309094 ± 0.020254	0.8913	EXP 150 of 150	0.5262469 ± 0.0160678	0.0548	EXP 150 of 150	39.3655844 ± 0.0201604	0.9938	EXP 150 of 150	109.435563 ± 0.024619	0.0041	EXP 150 of 150
17D20111	3.9 %	0.1853617 ± 0.0006241	0.6618	EXP 150 of 150	4.191568 ± 0.019373	0.5940	EXP 150 of 150	0.1677168 ± 0.0172819	0.0015	EXP 150 of 150	16.6263482 ± 0.0149776	0.9792	EXP 150 of 150	51.766793 ± 0.020769	0.9911	EXP 150 of 150
17D20113	4.5 %	0.3000811 ± 0.0007891	0.8070	EXP 150 of 150	9.134626 ± 0.018557	0.8922	EXP 150 of 150	0.4204367 ± 0.0153097	0.0395	EXP 150 of 150	34.0625098 ± 0.0188891	0.9926	EXP 150 of 150	81.096361 ± 0.017220	0.9688	EXP 150 of 150
17D20114	5.2 %	0.4127770 ± 0.0010720	0.8249	EXP 150 of 150	15.421479 ± 0.017958	0.9597	EXP 150 of 150	0.7351920 ± 0.0165587	0.1175	EXP 150 of 150	57.0614432 ± 0.0191451	0.9974	EXP 150 of 150	108.498666 ± 0.025126	0.2473	EXP 150 of 150
17D20116	6.0 %	0.1957397 ± 0.0007093	0.5964	EXP 150 of 150	6.355326 ± 0.019802	0.7776	EXP 150 of 150	0.3012881 ± 0.0148596	0.0360	EXP 149 of 150	23.6554483 ± 0.0181878	0.9856	EXP 150 of 150	52.848990 ± 0.021573	0.9887	EXP 150 of 150
17D20117	6.9 %	0.1352046 ± 0.0004890	0.4894	EXP 150 of 150	3.855419 ± 0.016968	0.6459	EXP 150 of 150	0.1782778 ± 0.0152468	0.0290	EXP 150 of 150	14.1278798 ± 0.0158962	0.9653	EXP 150 of 150	36.582971 ± 0.018787	0.9945	EXP 150 of 150
17D20119	7.9 %	0.2932266 ± 0.0008185	0.7867	EXP 150 of 150	10.212839 ± 0.017338	0.9210	EXP 150 of 150	0.4102995 ± 0.0152390	0.0381	EXP 150 of 150	30.9753338 ± 0.0159539	0.9937	EXP 150 of 150	78.292754 ± 0.020306	0.9537	EXP 150 of 150
17D20120	9.0 %	0.1915574 ± 0.0006871	0.6050	EXP 150 of 150	5.878898 ± 0.016398	0.7904	EXP 150 of 150	0.1929563 ± 0.0154719	0.0078	EXP 150 of 150	16.0608660 ± 0.0170879	0.9713	EXP 150 of 150	51.993022 ± 0.020879	0.9884	EXP 150 of 150
17D20122	10.3 %	0.1460906 ± 0.0005813	0.4821	EXP 150 of 150	3.867968 ± 0.015462	0.6975	EXP 150 of 150	0.0957403 ± 0.0155790	0.0030	EXP 150 of 150	10.2914166 ± 0.0167037	0.9319	EXP 150 of 150	40.072948 ± 0.018361	0.9931	EXP 150 of 150
17D20123	11.6 %	0.2648809 ± 0.0007752	0.7852	EXP 150 of 150	6.808942 ± 0.017829	0.8395	EXP 150 of 150	0.2028746 ± 0.0162194	0.0359	EXP 150 of 150	14.0474824 ± 0.0175613	0.9603	EXP 150 of 150	71.964629 ± 0.020090	0.9517	EXP 148 of 150
17D20125	12.5 %	0.3420292 ± 0.0008835	0.8499	EXP 150 of 150	8.670357 ± 0.019570	0.8825	EXP 149 of 150	0.2185866 ± 0.0150138	0.0205	EXP 150 of 150	13.4373603 ± 0.0159805	0.9652	EXP 150 of 150	93.736448 ± 0.021999	0.0230	EXP 150 of 150
17D20126	13.4 %	0.2483111 ± 0.0007705	0.7599	EXP 150 of 150	5.452834 ± 0.018196	0.7433	EXP 150 of 150	0.0958946 ± 0.0169229	0.0023	EXP 149 of 150	6.9216316 ± 0.0169734	0.8362	EXP 150 of 150	68.651399 ± 0.018928	0.9657	EXP 150 of 150
17D20128	14.6 %	0.1811561 ± 0.0006616	0.6385	EXP 150 of 150	3.659266 ± 0.020066	0.5342	EXP 150 of 150	0.0595746 ± 0.0165037	0.0007	EXP 150 of 150	3.9131630 ± 0.0155633	0.5958	EXP 150 of 150	50.348555 ± 0.018222	0.9873	EXP 150 of 150
17D20129	16.0 %	0.4730545 ± 0.0010068	0.9005	EXP 150 of 150	12.909225 ± 0.017636	0.9478	EXP 150 of 150	0.1535218 ± 0.0172532	0.0093	EXP 150 of 150	7.0715905 ± 0.0151362	0.8882	EXP 150 of 150	126.804093 ± 0.023108	0.9745	EXP 150 of 150
17D20131	17.6 %	0.1515095 ± 0.0005774	0.5409	EXP 150 of 150	4.483054 ± 0.017697	0.6977	EXP 150 of 150	0.0238984 ± 0.0156936	0.0022	EXP 150 of 150	2.5548944 ± 0.0151948	0.3147	EXP 150 of 150	40.179879 ± 0.018364	0.9925	EXP 150 of 150
17D20132	19.3 %	0.4829188 ± 0.0010627	0.8884	EXP 150 of 150	16.520311 ± 0.018311	0.9642	EXP 150 of 150	0.1332795 ± 0.0163945	0.0063	EXP 150 of 150	5.6757087 ± 0.0174018	0.7442	EXP 150 of 150	125.672799 ± 0.022957	0.9688	EXP 150 of 150
17D20134	21.0 %	0.3292389 ± 0.0009226	0.7756	EXP 150 of 150	13.786554 ± 0.018317	0.9521	EXP 150 of 150	0.1142999 ± 0.0167763	0.0472	EXP 150 of 150	3.8548741 ± 0.0160464	0.6532	EXP 149 of 150	82.913386 ± 0.021608	0.7665	EXP 150 of 150

Project Info		Analyst	Irradiation	X-pos	Y-pos	Z/H-pos	Project	Experiment	Nmb
17D20099	1.8 %	Dan Miggins	17-OSU-01	0.00	0.00	17.68	Arctic\O-Connor (16-22)	17D20095	01
17D20101	1.9 %	Dan Miggins	17-OSU-01	0.00	0.00	17.68	Arctic\O-Connor (16-22)	17D20095	01
17D20102	2.0 %	Dan Miggins	17-OSU-01	0.00	0.00	17.68	Arctic\O-Connor (16-22)	17D20095	01
17D20104	2.2 %	Dan Miggins	17-OSU-01	0.00	0.00	17.68	Arctic\O-Connor (16-22)	17D20095	01
17D20105	2.4 %	Dan Miggins	17-OSU-01	0.00	0.00	17.68	Arctic\O-Connor (16-22)	17D20095	01
17D20107	2.7 %	Dan Miggins	17-OSU-01	0.00	0.00	17.68	Arctic\O-Connor (16-22)	17D20095	01
17D20108	3.0 %	Dan Miggins	17-OSU-01	0.00	0.00	17.68	Arctic\O-Connor (16-22)	17D20095	01
17D20110	3.4 %	Dan Miggins	17-OSU-01	0.00	0.00	17.68	Arctic\O-Connor (16-22)	17D20095	01
17D20111	3.9 %	Dan Miggins	17-OSU-01	0.00	0.00	17.68	Arctic\O-Connor (16-22)	17D20095	01
17D20113	4.5 %	Dan Miggins	17-OSU-01	0.00	0.00	17.68	Arctic\O-Connor (16-22)	17D20095	01
17D20114	5.2 %	Dan Miggins	17-OSU-01	0.00	0.00	17.68	Arctic\O-Connor (16-22)	17D20095	01
17D20116	6.0 %	Dan Miggins	17-OSU-01	0.00	0.00	17.68	Arctic\O-Connor (16-22)	17D20095	01
17D20117	6.9 %	Dan Miggins	17-OSU-01	0.00	0.00	17.68	Arctic\O-Connor (16-22)	17D20095	01
17D20119	7.9 %	Dan Miggins	17-OSU-01	0.00	0.00	17.68	Arctic\O-Connor (16-22)	17D20095	01
17D20120	9.0 %	Dan Miggins	17-OSU-01	0.00	0.00	17.68	Arctic\O-Connor (16-22)	17D20095	01
17D20122	10.3 %	Dan Miggins	17-OSU-01	0.00	0.00	17.68	Arctic\O-Connor (16-22)	17D20095	01
17D20123	11.6 %	Dan Miggins	17-OSU-01	0.00	0.00	17.68	Arctic\O-Connor (16-22)	17D20095	01
17D20125	12.5 %	Dan Miggins	17-OSU-01	0.00	0.00	17.68	Arctic\O-Connor (16-22)	17D20095	01
17D20126	13.4 %	Dan Miggins	17-OSU-01	0.00	0.00	17.68	Arctic\O-Connor (16-22)	17D20095	01
17D20128	14.6 %	Dan Miggins	17-OSU-01	0.00	0.00	17.68	Arctic\O-Connor (16-22)	17D20095	01
17D20129	16.0 %	Dan Miggins	17-OSU-01	0.00	0.00	17.68	Arctic\O-Connor (16-22)	17D20095	01
17D20131	17.6 %	Dan Miggins	17-OSU-01	0.00	0.00	17.68	Arctic\O-Connor (16-22)	17D20095	01
17D20132	19.3 %	Dan Miggins	17-OSU-01	0.00	0.00	17.68	Arctic\O-Connor (16-22)	17D20095	01
17D20134	21.0 %	Dan Miggins	17-OSU-01	0.00	0.00	17.68	Arctic\O-Connor (16-22)	17D20095	01

Sample Parameters		Sample	Material	Location	Standard Name	Standard (in Ma)	%1σ	Standard Reference	Standard 40Ar/39Ar	%1σ	J	%1σ	Air 40Ar/36Ar	%1σ	MDF (lin)	%1σ	Volume Ratio	Sensitivity (mol/volt)	Day	Month	Year	Hour	Min	Resist
17D20099	1.8 %	PS59-276-1-005	Groundmass	Gakkel Ridge	FCT-NM (1C11-17)	28.201	0.082	Kuiper et al (2008)	9.52112	0.089	0.00165079	0.089	302.642	0.116	0.9940927	0.065	1	4.8E-14	12	JUN	2017	12	39	1
17D20101	1.9 %	PS59-276-1-005	Groundmass	Gakkel Ridge	FCT-NM (1C11-17)	28.201	0.082	Kuiper et al (2008)	9.52112	0.089	0.00165079	0.089	302.642	0.116	0.9940927	0.065	1	4.8E-14	12	JUN	2017	12	59	1
17D20102	2.0 %	PS59-276-1-005	Groundmass	Gakkel Ridge	FCT-NM (1C11-17)	28.201	0.082	Kuiper et al (2008)	9.52112	0.089	0.00165079	0.089	302.642	0.116	0.9940927	0.065	1	4.8E-14	12	JUN	2017	13	9	1
17D20104	2.2 %	PS59-276-1-005	Groundmass	Gakkel Ridge	FCT-NM (1C11-17)	28.201	0.082	Kuiper et al (2008)	9.52112	0.089	0.00165079	0.089	302.642	0.116	0.9940927	0.065	1	4.8E-14	12	JUN	2017	13	29	1
17D20105	2.4 %	PS59-276-1-005	Groundmass	Gakkel Ridge	FCT-NM (1C11-17)	28.201	0.082	Kuiper et al (2008)	9.52112	0.089	0.00165079	0.089	302.642	0.116	0.9940927	0.065	1	4.8E-14	12	JUN	2017	13	39	1
17D20107	2.7 %	PS59-276-1-005	Groundmass	Gakkel Ridge	FCT-NM (1C11-17)	28.201	0.082	Kuiper et al (2008)	9.52112	0.089	0.00165079	0.089	302.642	0.116	0.9940927	0.065	1	4.8E-14	12	JUN	2017	13	59	1
17D20108	3.0 %	PS59-276-1-005	Groundmass	Gakkel Ridge	FCT-NM (1C11-17)	28.201	0.082	Kuiper et al (2008)	9.52112	0.089	0.00165079	0.089	302.642	0.116	0.9940927	0.065	1	4.8E-14	12	JUN	2017	14	9	1
17D20110	3.4 %	PS59-276-1-005	Groundmass	Gakkel Ridge	FCT-NM (1C11-17)	28.201	0.082	Kuiper et al (2008)	9.52112	0.089	0.00165079	0.089	302.642	0.116	0.9940927	0.065	1	4.8E-14	12	JUN	2017	14	29	1
17D20111	3.9 %	PS59-276-1-005	Groundmass	Gakkel Ridge	FCT-NM (1C11-17)	28.201	0.082	Kuiper et al (2008)	9.52112	0.089	0.00165079	0.089	302.642	0.116	0.9940927	0.065	1	4.8E-14	12	JUN	2017	14	39	1
17D20113	4.5 %	PS59-276-1-005	Groundmass	Gakkel Ridge	FCT-NM (1C11-17)	28.201	0.082	Kuiper et al (2008)	9.52112	0.089	0.00165079	0.089	302.642	0.116	0.9940927	0.065	1	4.8E-14	12	JUN	2017	14	59	1
17D20114	5.2 %	PS59-276-1-005	Groundmass	Gakkel Ridge	FCT-NM (1C11-17)	28.201	0.082	Kuiper et al (2008)	9.52112	0.089	0.00165079	0.089	302.642	0.116	0.9940927	0.065	1	4.8E-14	12	JUN	2017	15	9	1
17D20116	6.0 %	PS59-276-1-005	Groundmass	Gakkel Ridge	FCT-NM (1C11-17)	28.201	0.082	Kuiper et al (2008)	9.52112	0.089	0.00165079	0.089	302.642	0.116	0.9940927	0.065	1	4.8E-14	12	JUN	2017	15	29	1
17D20117	6.9 %	PS59-276-1-005	Groundmass	Gakkel Ridge	FCT-NM (1C11-17)	28.201	0.082	Kuiper et al (2008)	9.52112	0.089	0.00165079	0.089	302.642	0.116	0.9940927	0.065	1	4.8E-14	12	JUN	2017	15	39	1
17D20119	7.9 %	PS59-276-1-005	Groundmass	Gakkel Ridge	FCT-NM (1C11-17)	28.201	0.082	Kuiper et al (2008)	9.52112	0.089	0.00165079	0.089	302.642	0.116	0.9940927	0.065	1	4.8E-14	12	JUN	2017	15	59	1
17D20120	9.0 %	PS59-276-1-005	Groundmass	Gakkel Ridge	FCT-NM (1C11-17)	28.201	0.082	Kuiper et al (2008)	9.52112	0.089	0.00165079	0.089	302.642	0.116	0.9940927	0.065	1	4.8E-14	12	JUN	2017	16	9	1
17D20122	10.3 %	PS59-276-1-005	Groundmass	Gakkel Ridge	FCT-NM (1C11-17)	28.201	0.082	Kuiper et al (2008)	9.52112	0.089	0.00165079	0.089	302.642	0.116	0.9940927	0.065	1	4.8E-14	12	JUN	2017	16	29	1
17D20123	11.6 %	PS59-276-1-005	Groundmass	Gakkel Ridge	FCT-NM (1C11-17)	28.201	0.082	Kuiper et al (2008)	9.52112	0.089	0.00165079	0.089	302.642	0.116	0.9940927	0.065	1	4.8E-14	12	JUN	2017	16	39	1
17D20125	12.5 %	PS59-276-1-005	Groundmass	Gakkel Ridge	FCT-NM (1C11-17)	28.201	0.082	Kuiper et al (2008)	9.52112	0.089	0.00165079	0.089	302.642	0.116	0.9940927	0.065	1	4.8E-14	12	JUN	2017	16	59	1
17D20126	13.4 %	PS59-276-1-005	Groundmass	Gakkel Ridge	FCT-NM (1C11-17)	28.201	0.082	Kuiper et al (2008)	9.52112	0.089	0.00165079	0.089	302.642	0.116	0.9940927	0.065	1	4.8E-14	12	JUN	2017	17	9	1
17D20128	14.6 %	PS59-276-1-005	Groundmass	Gakkel Ridge	FCT-NM (1C11-17)	28.201	0.082	Kuiper et al (2008)	9.52112	0.089	0.00165079	0.089	302.642	0.116	0.9940927	0.065	1	4.8E-14	12	JUN	2017	17	29	1
17D20129	16.0 %	PS59-276-1-005	Groundmass	Gakkel Ridge	FCT-NM (1C11-17)	28.201	0.082	Kuiper et al (2008)	9.52112	0.089	0.00165079	0.089	302.642	0.116	0.9940927	0.065	1	4.8E-14	12	JUN	2017	17	39	1
17D20131	17.6 %	PS59-276-1-005	Groundmass	Gakkel Ridge	FCT-NM (1C11-17)	28.201	0.082	Kuiper et al (2008)	9.52112	0.089	0.00165079	0.089	302.642	0.116	0.9940927	0.065	1	4.8E-14	12	JUN	2017	17	59	1
17D20132	19.3 %	PS59-276-1-005	Groundmass	Gakkel Ridge	FCT-NM (1C11-17)	28.201	0.082	Kuiper et al (2008)	9.52112	0.089	0.00165079	0.089	302.642	0.116	0.9940927	0.065	1	4.8E-14	12	JUN	2017	18	9	1
17D20134	21.0 %	PS59-276-1-005	Groundmass	Gakkel Ridge	FCT-NM (1C11-17)	28.201	0.082	Kuiper et al (2008)	9.52112	0.089	0.00165079	0.089	302.642	0.116	0.9940927	0.065	1	4.8E-14	12	JUN	2017	18	30	1

Irradiation Constants		40/36(a)	%1σ	40/36(c)	%1σ	38/36(a)	%1σ	38/36(c)	%1σ	39/37(ca)	%1σ	38/37(ca)	%1σ	36/37(ca)	%1σ	40/39(k)	%1σ	38/39(k)	%1σ	36/38(cl)	%1σ	K/Ca	%1σ	K/Cl	%1σ	Ca/Cl	%1σ
17D20099	1.8 %	295.5	0	0.018	35	0.1869	0	1.493	3	0.000643	0.92	0.00018	9.63	0.00027	0.17	0.000607	9.65	0.012077	0.09	0	0	0.43	0	0	0	0	0
17D20101	1.9 %	295.5	0	0.018	35	0.1869	0	1.493	3	0.000643	0.92	0.00018	9.63	0.00027	0.17	0.000607	9.65	0.012077	0.09	0	0	0.43	0	0	0	0	0
17D20102	2.0 %	295.5	0	0.018	35	0.1869	0	1.493	3	0.000643	0.92	0.00018	9.63	0.00027	0.17	0.000607	9.65	0.012077	0.09	0	0	0.43	0	0	0	0	0
17D20104	2.2 %	295.5	0	0.018	35	0.1869	0	1.493	3	0.000643	0.92	0.00018	9.63	0.00027	0.17	0.000607	9.65	0.012077	0.09	0	0	0.43	0	0	0	0	0
17D20105	2.4 %	295.5	0	0.018	35	0.1869	0	1.493	3	0.000643	0.92	0.00018	9.63	0.00027	0.17	0.000607	9.65	0.012077	0.09	0	0	0.43	0	0	0	0	0
17D20107	2.7 %	295.5	0	0.018	35	0.1869	0	1.493	3	0.000643	0.92	0.00018	9.63	0.00027	0.17	0.000607	9.65	0.012077	0.09	0	0	0.43	0	0	0	0	0
17D20108	3.0 %	295.5	0	0.018	35	0.1869	0	1.493	3	0.000643	0.92	0.00018	9.63	0.00027	0.17	0.000607	9.65	0.012077	0.09	0	0	0.43	0	0	0	0	0
17D20110	3.4 %	295.5	0	0.018	35	0.1869	0	1.493	3	0.000643	0.92	0.00018	9.63	0.00027	0.17	0.000607	9.65	0.012077	0.09	0	0	0.43	0	0	0	0	0
17D20111	3.9 %	295.5	0	0.018	35	0.1869	0	1.493	3	0.000643	0.92	0.00018	9.63	0.00027	0.17	0.000607	9.65	0.012077	0.09	0	0	0.43	0	0	0	0	0
17D20113	4.5 %	295.5	0	0.018	35	0.1869	0	1.493	3	0.000643	0.92	0.00018	9.63	0.00027	0.17	0.000607	9.65	0.012077	0.09	0	0	0.43	0	0	0	0	0
17D20114	5.2 %	295.5	0	0.018	35	0.1869	0	1.493	3	0.000643	0.92	0.00018	9.63	0.00027	0.17	0.000607	9.65	0.012077	0.09	0	0	0.43	0	0	0	0	0
17D20116	6.0 %	295.5	0	0.018	35	0.1869	0	1.493	3	0.000643	0.92	0.00018	9.63	0.00027	0.17	0.000607	9.65	0.012077	0.09	0	0	0.43	0	0	0	0	0
17D20117	6.9 %	295.5	0	0.018	35	0.1869	0	1.493	3	0.000643	0.92	0.00018	9.63	0.00027	0.17	0.000607	9.65	0.012077	0.09	0	0	0.43	0	0	0	0	0
17D20119	7.9 %	295.5	0	0.018	35	0.1869	0	1.493	3	0.000643	0.92	0.00018	9.63	0.00027	0.17	0.000607	9.65	0.012077	0.09	0	0	0.43	0	0	0	0	0
17D20120	9.0 %	295.5	0	0.018	35	0.1869	0	1.493	3	0.000643	0.92	0.00018	9.63	0.00027	0.17	0.000607	9.65	0.012077	0.09	0	0	0.43	0	0	0	0	0
17D20122	10.3 %	295.5	0	0.018	35	0.1869	0	1.493	3	0.000643	0.92	0.00018	9.63	0.00027	0.17	0.000607	9.65	0.012077	0.09	0	0	0.43	0	0	0	0	0
17D20123	11.6 %	295.5	0	0.018	35	0.1869	0	1.493	3	0.000643	0.92	0.00018	9.63	0.00027	0.17	0.000607	9.65	0.012077	0.09	0	0	0.43	0	0	0	0	0
17D20125	12.5 %	295.5	0	0.018	35	0.1869	0	1.493	3	0.000643	0.92	0.00018	9.63	0.00027	0.17	0.000607	9.65	0.012077	0.09	0	0	0.43	0	0	0	0	0
17D20126	13.4 %	295.5	0	0.018	35	0.1869	0	1.493	3	0.000643	0.92	0.00018	9.63	0.00027	0.17	0.000607	9.65	0.012077	0.09	0	0	0.43	0	0	0	0	0
17D20128	14.6 %	295.5	0	0.018	35	0.1869	0	1.493	3	0.000643	0.92	0.00018	9.63	0.00027	0.17	0.000607	9.65	0.012077	0.09	0	0	0.43	0	0	0	0	0
17D20129	16.0 %	295.5	0	0.018	35	0.1869	0	1.493	3	0.000643	0.92	0.00018	9.63	0.00027	0.17	0.000607	9.65	0.012077	0.09	0	0	0.43	0	0	0	0	0
17D20131	17.6 %	295.5	0	0.018	35	0.1869	0	1.493	3	0.000643	0.92	0.00018	9.63	0.00027	0.17	0.000607	9.65	0.012077	0.09	0	0	0.43	0	0	0	0	0
17D20132	19.3 %	295.5	0	0.018	35	0.1869	0	1.493	3	0.000643	0.92	0.00018	9.63	0.00027	0.17	0.000607	9.65	0.012077	0.09	0	0	0.43	0	0	0	0	0
17D20134	21.0 %	295.5	0	0.018	35	0.1869	0	1.493	3	0.000643	0.92	0.00018	9.63	0.00027	0.17	0.000607	9.65	0.012077	0.09	0	0	0.43	0	0	0	0	0

17D20095.AGE >>> PS59-276-1-005 >>> ARCTIC | O-CONNOR (16-22) PROJECT

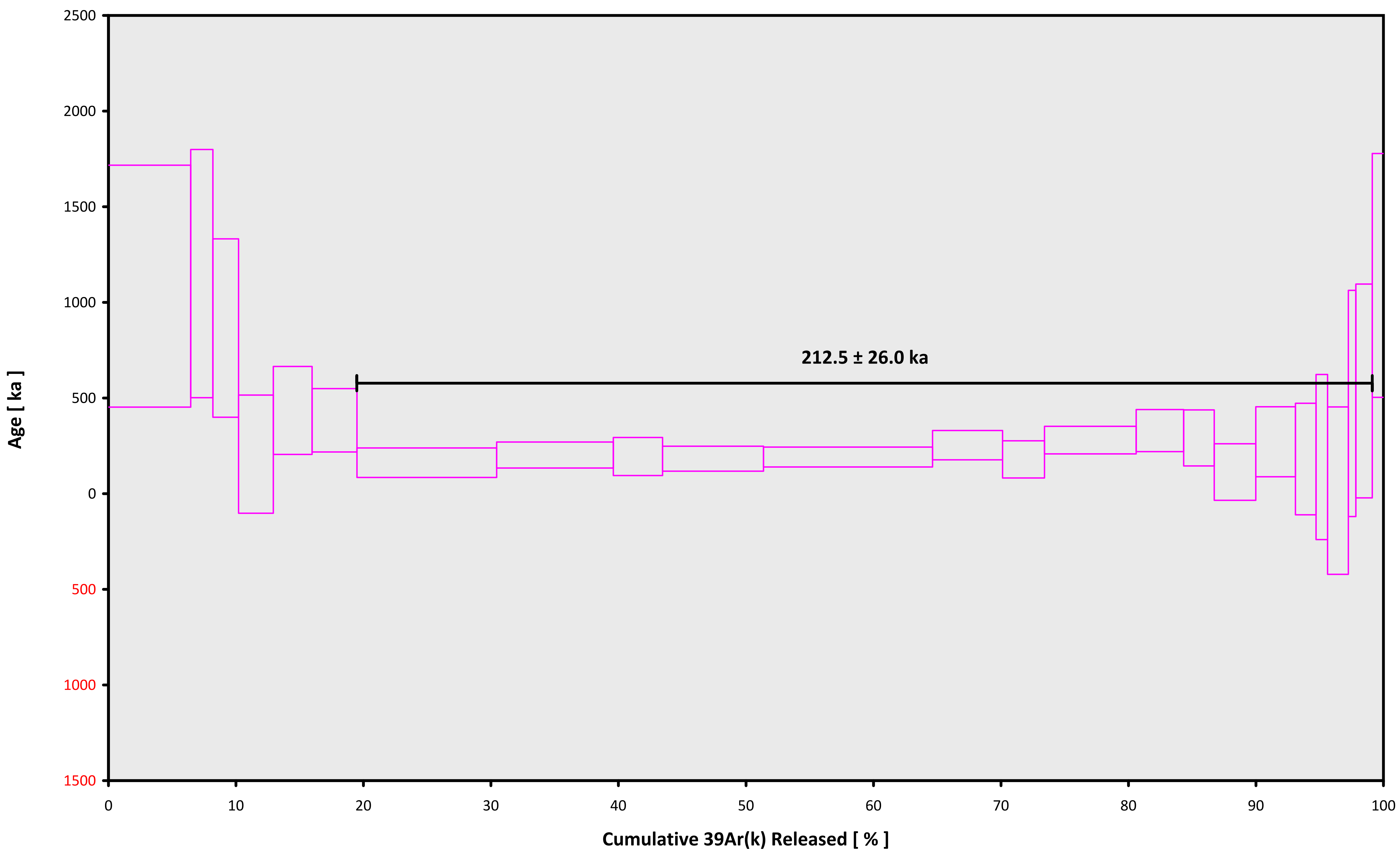

Ar-Ages in ka

WEIGHTED PLATEAU
212.5 ± 26.0
TOTAL FUSION
319.5 ± 50.5
NORMAL ISOCHRON
206.7 ± 51.0
INVERSE ISOCHRON
207.1 ± 46.1

MSWD (PROBABILITY)
1.22 (24%)

Sample Info

Groundmass
Gakkel Ridge
Dan Miggins

IRR = 17-OSU-01 (1C11-17)
J = 0.00165079 ± 0.00000147

17D20095.AGE >>> PS59-276-1-005 >>> ARCTIC | O-CONNOR (16-22) PROJECT

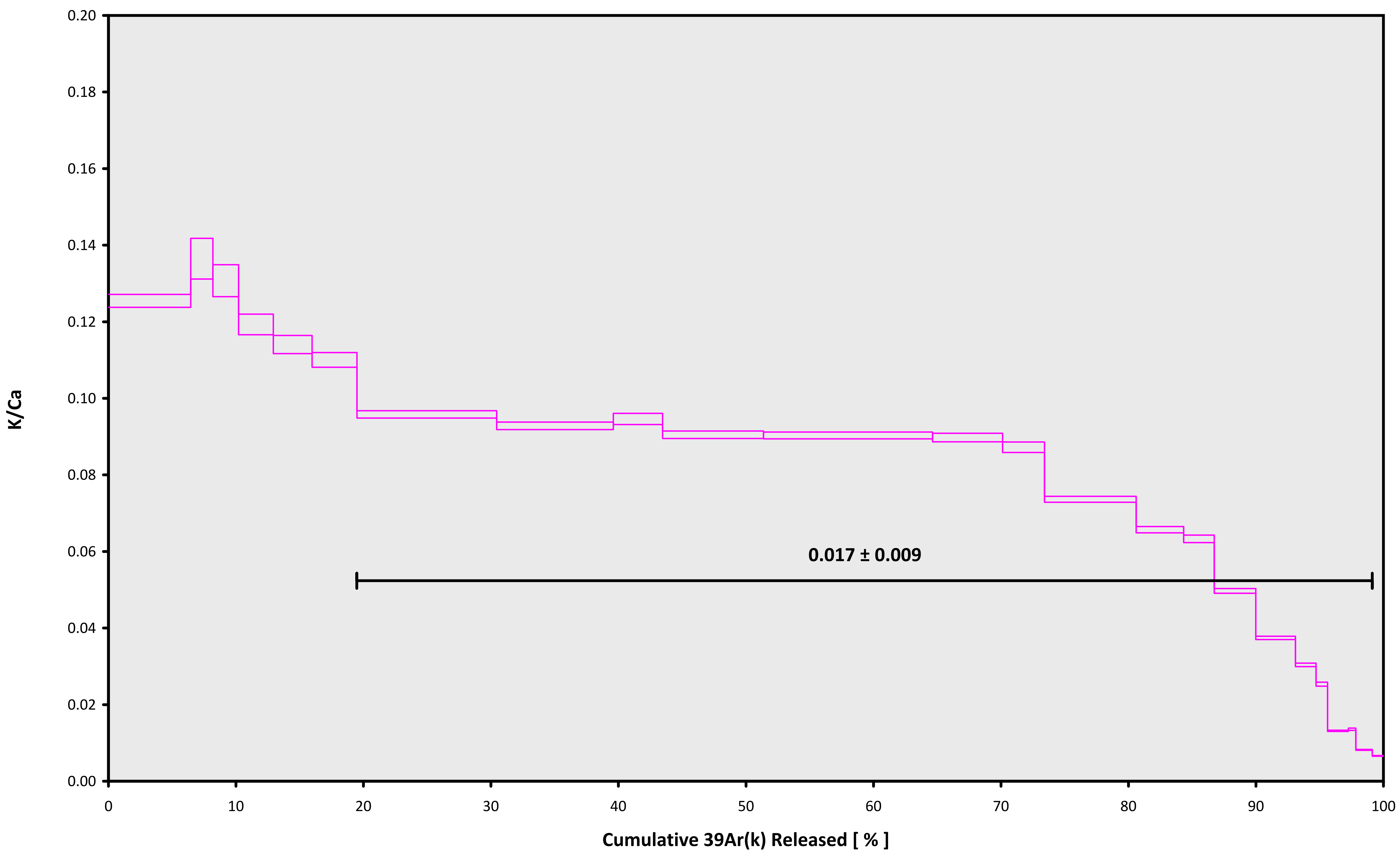

Ar-Ages in ka

WEIGHTED PLATEAU
212.5 ± 26.0
TOTAL FUSION
319.5 ± 50.5
NORMAL ISOCHRON
206.7 ± 51.0
INVERSE ISOCHRON
207.1 ± 46.1

Sample Info

Groundmass
Gakkel Ridge
Dan Miggins

IRR = 17-OSU-01 (1C11-17)
J = 0.00165079 ± 0.00000147

17D20095.AGE >>> PS59-276-1-005 >>> ARCTIC | O-CONNOR (16-22) PROJECT

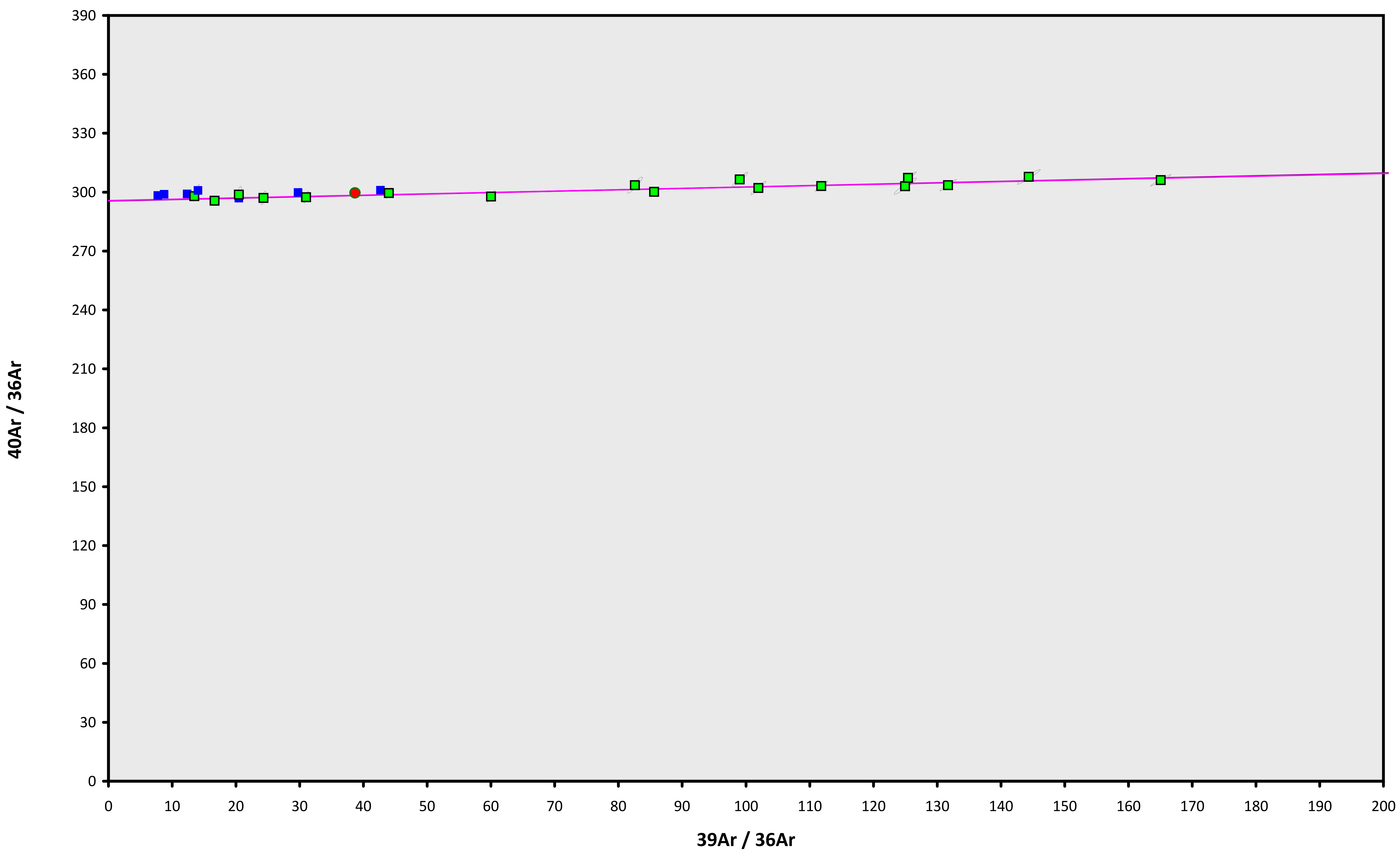

Ar-Ages in ka

WEIGHTED PLATEAU
212.5 ± 26.0
TOTAL FUSION
319.5 ± 50.5
NORMAL ISOCHRON
206.7 ± 51.0
INVERSE ISOCHRON
207.1 ± 46.1

MSWD (PROBABILITY)
1.29 (20%)

40AR/36AR INTERCEPT
295.7 ± 1.6

Sample Info

Groundmass
Gakkel Ridge
Dan Miggins

IRR = 17-OSU-01 (1C11-17)
J = 0.00165079 ± 0.00000147

17D20095.AGE >>> PS59-276-1-005 >>> ARCTIC | O-CONNOR (16-22) PROJECT

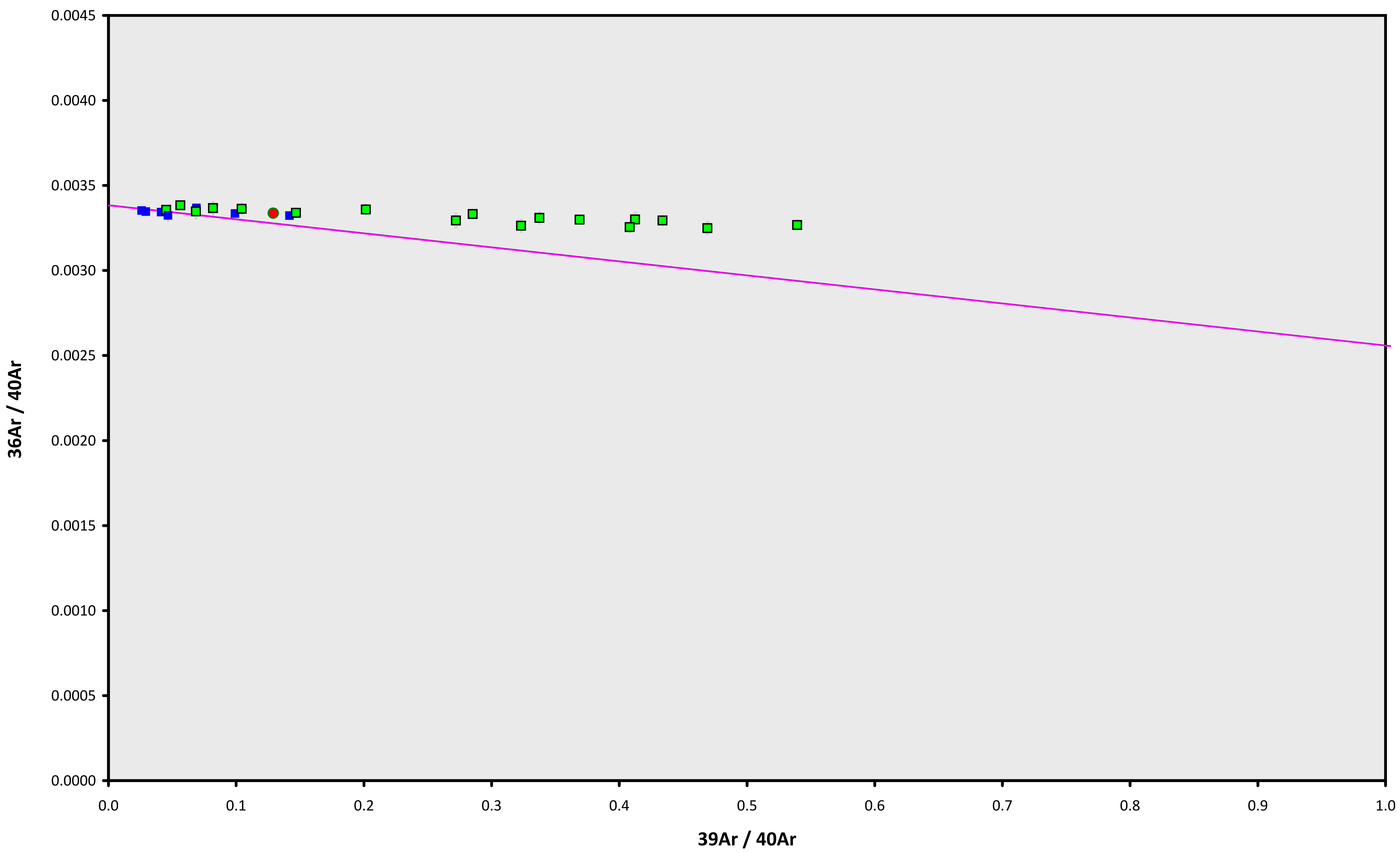

Ar-Ages in ka

WEIGHTED PLATEAU
212.5 ± 26.0
TOTAL FUSION
319.5 ± 50.5
NORMAL ISOCHRON
206.7 ± 51.0
INVERSE ISOCHRON
207.1 ± 46.1

MSWD (PROBABILITY)
1.30 (19%)

SPREADING FACTOR
3.4%

40AR/36AR INTERCEPT
295.7 ± 1.6

Sample Info

Groundmass
Gakkel Ridge
Dan Miggins

IRR = 17-OSU-01 (1C11-17)
J = 0.00165079 ± 0.00000147

Relative Abundances			36Ar [fA]	%1σ	37Ar [fA]	%1σ	38Ar [fA]	%1σ	39Ar [fA]	%1σ	40Ar [fA]	%1σ	40(r)/39(k) ± 2σ	Age ± 2σ (Ma)	40Ar(r) (%)	39Ar(k) (%)	K/Ca ± 2σ
18D00615	1.8 %	✓	0.2361434	0.502	47.0608	6.270	0.2146043	11.517	15.37581	0.166	73.2405	0.139	0.46982 ± 0.06020	1.38 ± 0.18	9.84	2.11	0.140 ± 0.018
18D00617	1.9 %	✓	0.1375072	0.659	43.8530	6.661	0.1823596	13.222	13.94294	0.188	44.1626	0.212	0.50475 ± 0.05431	1.49 ± 0.16	15.90	1.91	0.136 ± 0.018
18D00618	2.0 %	✓	0.1013028	0.805	33.8366	8.930	0.1753148	13.401	12.36339	0.204	33.1521	0.283	0.47908 ± 0.05830	1.41 ± 0.17	17.83	1.70	0.157 ± 0.028
18D00620	2.2 %	✓	0.2397270	0.490	169.2900	1.937	0.6995356	3.252	54.66647	0.081	85.3971	0.112	0.51407 ± 0.01712	1.51 ± 0.05	32.84	7.51	0.139 ± 0.005
18D00621	2.4 %	✓	0.0536772	1.199	37.5137	7.924	0.1528722	16.214	12.04126	0.200	18.7197	0.498	0.48656 ± 0.05324	1.43 ± 0.16	31.24	1.65	0.138 ± 0.022
18D00623	2.7 %	✓	0.2173518	0.507	203.0364	1.694	0.7755965	3.291	63.64864	0.079	80.2782	0.119	0.50741 ± 0.01426	1.49 ± 0.04	40.15	8.74	0.135 ± 0.005
18D00624	3.0 %	✓	0.0707002	0.981	72.3407	4.145	0.2704498	9.298	22.89128	0.122	26.4392	0.351	0.49515 ± 0.02897	1.46 ± 0.09	42.78	3.14	0.136 ± 0.011
18D00626	3.4 %	✓	0.1327824	0.642	149.9580	2.149	0.5827307	4.047	47.57245	0.086	51.3285	0.183	0.50636 ± 0.01597	1.49 ± 0.05	46.84	6.53	0.136 ± 0.006
18D00627	3.9 %	✓	0.0887232	0.853	104.0024	2.833	0.4090383	5.673	33.36820	0.097	34.4268	0.271	0.49535 ± 0.02048	1.46 ± 0.06	47.92	4.58	0.138 ± 0.008
18D00629	4.5 %	✓	0.1260623	0.661	148.5367	2.038	0.5923303	4.051	48.26021	0.084	49.1438	0.191	0.49263 ± 0.01510	1.45 ± 0.04	48.28	6.63	0.139 ± 0.006
18D00630	5.2 %	✓	0.2251259	0.500	268.2611	1.311	1.0357099	2.251	85.89946	0.076	88.6244	0.111	0.50713 ± 0.01077	1.49 ± 0.03	49.05	11.80	0.137 ± 0.004
18D00632	6.0 %	✓	0.0779642	0.905	96.2792	3.206	0.3626345	6.611	30.76998	0.104	30.3992	0.307	0.48952 ± 0.02206	1.44 ± 0.06	49.45	4.23	0.137 ± 0.009
18D00633	6.9 %	✓	0.1272480	0.655	143.5588	2.191	0.5558330	4.288	44.06507	0.089	48.3171	0.193	0.50384 ± 0.01684	1.48 ± 0.05	45.85	6.05	0.132 ± 0.006
18D00635	7.9 %	✓	0.1166734	0.706	118.0192	2.668	0.4768644	4.963	36.64018	0.093	42.7533	0.218	0.48355 ± 0.02012	1.42 ± 0.06	41.36	5.03	0.133 ± 0.007
18D00636	9.0 %	✓	0.1462667	0.623	126.6855	2.478	0.4915834	4.829	37.16390	0.096	51.4823	0.180	0.49503 ± 0.02094	1.46 ± 0.06	35.66	5.10	0.126 ± 0.006
18D00638	10.3 %	✓	0.2266270	0.484	166.3963	1.845	0.6028627	4.101	42.69150	0.088	74.3045	0.126	0.48383 ± 0.02051	1.42 ± 0.06	27.73	5.86	0.110 ± 0.004
18D00639	11.6 %	✓	0.3908800	0.412	270.2300	1.342	0.8098495	2.955	48.74909	0.084	118.0286	0.080	0.49596 ± 0.02508	1.46 ± 0.07	20.41	6.68	0.077 ± 0.002
18D00641	12.5 %	✓	0.1650771	0.558	127.2228	2.470	0.3306562	7.156	19.98327	0.130	48.4190	0.192	0.49209 ± 0.03953	1.45 ± 0.12	20.23	2.74	0.067 ± 0.003
18D00642	13.4 %	✓	0.2091281	0.487	151.4615	2.079	0.3693097	6.439	20.68313	0.128	59.3875	0.157	0.47035 ± 0.04088	1.38 ± 0.12	16.30	2.83	0.058 ± 0.002
18D00644	14.6 %		0.1309459	0.638	101.8371	2.924	0.2059769	11.443	12.41947	0.202	35.8362	0.260	0.42662 ± 0.05877	1.26 ± 0.17	14.71	1.70	0.052 ± 0.003
18D00645	16.0 %		0.0570960	1.151	41.0943	7.234	0.0800412	29.519	5.72358	0.385	15.8185	0.583	0.39064 ± 0.11305	1.15 ± 0.33	14.07	0.78	0.060 ± 0.009
18D00647	17.6 %		0.0805412	0.882	65.2418	4.614	0.1223144	19.689	6.50990	0.355	21.3511	0.434	0.42683 ± 0.10375	1.26 ± 0.31	12.93	0.89	0.043 ± 0.004
18D00648	19.3 %		0.1181795	0.648	108.4103	2.932	0.1452365	16.064	7.29219	0.307	28.7477	0.323	0.34377 ± 0.09922	1.01 ± 0.29	8.64	0.99	0.029 ± 0.002
18D00650	21.0 %		0.1189056	0.678	110.9229	2.942	0.1417941	15.994	5.82225	0.383	28.7473	0.321	0.42969 ± 0.12890	1.26 ± 0.38	8.60	0.79	0.022 ± 0.001
		Σ	3.5946361	0.127	2905.0491	0.529	9.7854985	1.196	728.54364	0.023	1188.5050	0.039					

Information on Analysis and Constants Used in Calculations

Project = **O-CONNOR (16-22)**
Sample = **HLY0102-D66-32**
Material = **Groundmass**
Location = **Gakkel Ridge**
Region = **Artic Ocean**
Analyst = **Dan Miggins**
Irradiation = **17-OSU-05 (5B11-17)**
Position = **X: 0 | Y: 0 | Z/H: 16.47832 mm**
FCT-NM Age = **28.201 ± 0.023 Ma**
FCT-NM Reference = **Kuiper et al (2008)**
FCT-NM 40Ar/39Ar Ratio = **9.65222 ± 0.00791**
FCT-NM J-value = **0.00162837 ± 0.00000134**
Air Shot 40Ar/36Ar = **302.6400 ± 0.4600**
Air Shot MDF = **0.99409428 ± 0.00068817 (LIN)**
Experiment Type = **Incremental Heating**
Extraction Method = **Bulk Laser Heating**
Heating = **77 sec**
Isolation = **3.00 min**
Instrument = **ARGUS-VI-D**
Preferred Age = **Plateau Age**
Age Classification = **Crystallization Age**
IGSN = Undefined
Rock Class = Undefined
Lithology = **Basaltic Lava**
Lat-Lon = Undefined - Undefined

Age Equations = **Min et al. (2000)**
Negative Intensities = **Allowed**
Collector Calibrations = **36Ar**
Decay 40K = **5.530 ± 0.048 E-10 1/a**
Decay 39Ar = **2.940 ± 0.016 E-07 1/h**
Decay 37Ar = **8.230 ± 0.012 E-04 1/h**
Decay 36Cl = **2.257 ± 0.015 E-06 1/a**
Decay 40K(EC,β^+) = **0.580 ± 0.009 E-10 1/a**
Decay 40K(β^-) = **4.950 ± 0.043 E-10 1/a**
Atmospheric 40/36(a) = **295.50 ± 0.70**
Atmospheric 38/36(a) = **0.1869**
Production 39/37(ca) = **0.0006425 ± 0.0000059**
Production 38/37(ca) = **0.0001800 ± 0.0000173**
Production 36/37(ca) = **0.0002703 ± 0.0000005**
Production 40/39(k) = **0.000607 ± 0.000059**
Production 38/39(k) = **0.012077 ± 0.000011**
Production 36/38(cl) = **262.80 ± 1.71**
Scaling Ratio K/Ca = **0.430**
Abundance Ratio 40K/K = **1.1700 ± 0.0100 E-04**
Atomic Weight K = **39.0983 ± 0.0001 g**

Results

Results	40(a)/36(a) ± 2σ	40(r)/39(k) ± 2σ	Age ± 2σ (Ma)	MSWD	39Ar(k) (%,n)	K/Ca ± 2σ
**Age Plateau**		0.49948 ± 0.00469 ± 0.94%	**1.47** ± 0.01 ± 0.95%	1.00	94.84	0.097 ± 0.015
				45%	19	
		Full External Error	± 0.04	1.67	2σ Confidence Limit	
		Analytical Error	± 0.01	1.0017	Error Magnification	
**Total Fusion Age**		0.49255 ± 0.00534 ± 1.09%	**1.45** ± 0.02 ± 1.10%		24	0.108 ± 0.001
		Full External Error	± 0.04			
		Analytical Error	± 0.02			
**Normal Isochron**	**293.45** ± 2.55 ± 0.87%	0.50495 ± 0.00845 ± 1.67%	**1.49** ± 0.02 ± 1.68%	0.99	94.84	
				47%	19	
		Full External Error	± 0.04	1.69	2σ Confidence Limit	
		Analytical Error	± 0.02	1.0000	Error Magnification	
				11	Number of Iterations	
				0.0000028900	Convergence	
**Inverse Isochron**	**293.43** ± 2.56 ± 0.87%	0.50532 ± 0.00844 ± 1.67%	**1.49** ± 0.02 ± 1.68%	0.98	94.84	
				48%	19	
		Full External Error	± 0.04	1.69	2σ Confidence Limit	
		Analytical Error	± 0.02	1.0000	Error Magnification	
				3	Number of Iterations	
				0.0004579159	Convergence	
				40%	Spreading Factor	

Notes
Mostly atmospheric

Incremental Heating			36Ar(a) [fA]	37Ar(ca) [fA]	38Ar(cl) [fA]	39Ar(k) [fA]	40Ar(r) [fA]	Age ± 2σ (Ma)	40Ar(r) (%)	39Ar(k) (%)	K/Ca ± 2σ
18D00615	1.8 %	✓	0.2234229	47.0608	0.0000000	15.34557	7.20971	1.38 ± 0.18	9.84	2.11	0.140 ± 0.018
18D00617	1.9 %	✓	0.1256537	43.8530	0.0000000	13.91476	7.02347	1.49 ± 0.16	15.90	1.91	0.136 ± 0.018
18D00618	2.0 %	✓	0.0921556	33.8366	0.0029502	12.34165	5.91264	1.41 ± 0.17	17.83	1.70	0.157 ± 0.028
18D00620	2.2 %	✓	0.1939679	169.2900	0.0000000	54.55770	28.04648	1.51 ± 0.05	32.84	7.51	0.139 ± 0.005
18D00621	2.4 %	✓	0.0435372	37.5137	0.0000000	12.01716	5.84712	1.43 ± 0.16	31.24	1.65	0.138 ± 0.022
18D00623	2.7 %	✓	0.1624711	203.0364	0.0000000	63.51818	32.22945	1.49 ± 0.04	40.15	8.74	0.135 ± 0.005
18D00624	3.0 %	✓	0.0511465	72.3407	0.0000000	22.84481	11.31150	1.46 ± 0.09	42.78	3.14	0.136 ± 0.011
18D00626	3.4 %	✓	0.0922488	149.9580	0.0000000	47.47610	24.04013	1.49 ± 0.05	46.84	6.53	0.136 ± 0.006
18D00627	3.9 %	✓	0.0606114	104.0024	0.0000000	33.30138	16.49594	1.46 ± 0.06	47.92	4.58	0.138 ± 0.008
18D00629	4.5 %	✓	0.0859128	148.5367	0.0000000	48.16478	23.72730	1.45 ± 0.04	48.28	6.63	0.139 ± 0.006
18D00630	5.2 %	✓	0.1526149	268.2611	0.0000000	85.72710	43.47460	1.49 ± 0.03	49.05	11.80	0.137 ± 0.004
18D00632	6.0 %	✓	0.0519399	96.2792	0.0000000	30.70812	15.03232	1.44 ± 0.06	49.45	4.23	0.137 ± 0.009
18D00633	6.9 %	✓	0.0884440	143.5588	0.0000000	43.97283	22.15516	1.48 ± 0.05	45.85	6.05	0.132 ± 0.006
18D00635	7.9 %	✓	0.0847729	118.0192	0.0000000	36.56435	17.68073	1.42 ± 0.06	41.36	5.03	0.133 ± 0.007
18D00636	9.0 %	✓	0.1120236	126.6855	0.0000000	37.08251	18.35681	1.46 ± 0.06	35.66	5.10	0.126 ± 0.006
18D00638	10.3 %	✓	0.1816405	166.3963	0.0246686	42.58459	20.60391	1.42 ± 0.06	27.73	5.86	0.110 ± 0.004
18D00639	11.6 %	✓	0.3177920	270.2300	0.1151669	48.57546	24.09162	1.46 ± 0.07	20.41	6.68	0.077 ± 0.002
18D00641	12.5 %	✓	0.1306720	127.2228	0.0429828	19.90153	9.79335	1.45 ± 0.12	20.23	2.74	0.067 ± 0.003
18D00642	13.4 %	✓	0.1681640	151.4615	0.0620018	20.58582	9.68252	1.38 ± 0.12	16.30	2.83	0.058 ± 0.002
18D00644	14.6 %		0.1034119	101.8371	0.0191188	12.35404	5.27051	1.26 ± 0.17	14.71	1.70	0.052 ± 0.003
18D00645	16.0 %		0.0459882	41.0943	0.0000000	5.69718	2.22554	1.15 ± 0.33	14.07	0.78	0.060 ± 0.009
18D00647	17.6 %		0.0628983	65.2418	0.0207014	6.46798	2.76075	1.26 ± 0.31	12.93	0.89	0.043 ± 0.004
18D00648	19.3 %		0.0888677	108.4103	0.0218867	7.22254	2.48288	1.01 ± 0.29	8.64	0.99	0.029 ± 0.002
18D00650	21.0 %		0.0889092	110.9229	0.0357562	5.75099	2.47113	1.26 ± 0.38	8.60	0.79	0.022 ± 0.001
		Σ	2.8092669	2905.0491	0.3452334	726.67714	357.92558				

Information on Analysis

Project = **O-CONNOR (16-22)**
Sample = **HLY0102-D66-32**
Material = **Groundmass**
Location = **Gakkel Ridge**
Region = **Artic Ocean**
Analyst = **Dan Miggins**
Irradiation = **17-OSU-05 (5B11-17)**
J = **0.00162837 ± 0.00000134**
FCT-NM = **28.201 ± 0.023 Ma**

Results	40(r)/39(k) ± 2σ		Age ± 2σ (Ma)		MSWD	39Ar(k) (%,n)	K/Ca ± 2σ
**Age Plateau**	0.49948	± 0.00469<br>± 0.94%	**1.47**	**± 0.01**<br>**± 0.95%**	1.00<br>45%	94.84<br>19	0.097 ± 0.015
			Full External Error	± 0.04	1.67	2σ Confidence Limit	
			Analytical Error	± 0.01	1.0017	Error Magnification	
**Total Fusion Age**	0.49255	± 0.00534<br>± 1.09%	**1.45**	**± 0.02**<br>**± 1.10%**		24	0.108 ± 0.001
			Full External Error	± 0.04			
			Analytical Error	± 0.02			

Normal Isochron			39(k)/36(a) ± 2σ	40(a+r)/36(a) ± 2σ	r.i.
18D00615	1.8 %	✓	68.68 ± 0.91	327.77 ± 4.29	0.9457
18D00617	1.9 %	✓	110.74 ± 2.16	351.40 ± 6.88	0.9579
18D00618	2.0 %	✓	133.92 ± 3.40	359.66 ± 9.24	0.9626
18D00620	2.2 %	✓	281.27 ± 4.30	440.09 ± 6.76	0.9836
18D00621	2.4 %	✓	276.02 ± 13.10	429.80 ± 20.77	0.9750
18D00623	2.7 %	✓	390.95 ± 6.98	493.87 ± 8.86	0.9872
18D00624	3.0 %	✓	446.65 ± 18.67	516.66 ± 21.86	0.9845
18D00626	3.4 %	✓	514.65 ± 13.65	556.10 ± 14.86	0.9884
18D00627	3.9 %	✓	549.42 ± 19.96	567.66 ± 20.82	0.9876
18D00629	4.5 %	✓	560.62 ± 15.30	571.68 ± 15.72	0.9884
18D00630	5.2 %	✓	561.72 ± 10.91	580.36 ± 11.32	0.9904
18D00632	6.0 %	✓	591.22 ± 24.93	584.92 ± 24.89	0.9883
18D00633	6.9 %	✓	497.18 ± 13.44	546.00 ± 14.88	0.9877
18D00635	7.9 %	✓	431.32 ± 12.09	504.07 ± 14.27	0.9859
18D00636	9.0 %	✓	331.02 ± 7.40	459.37 ± 10.36	0.9834
18D00638	10.3 %	✓	234.44 ± 3.58	408.93 ± 6.28	0.9799
18D00639	11.6 %	✓	152.85 ± 1.84	371.31 ± 4.46	0.9813
18D00641	12.5 %	✓	152.30 ± 2.95	370.45 ± 7.26	0.9715
18D00642	13.4 %	✓	122.42 ± 1.96	353.08 ± 5.69	0.9680
18D00644	14.6 %		119.46 ± 2.73	346.47 ± 7.99	0.9585
18D00645	16.0 %		123.88 ± 5.67	343.89 ± 16.03	0.9542
18D00647	17.6 %		102.83 ± 3.61	339.39 ± 12.03	0.9491
18D00648	19.3 %		81.27 ± 2.17	323.44 ± 8.64	0.9435
18D00650	21.0 %		64.68 ± 1.81	323.29 ± 8.94	0.9341

Results	40(a)/36(a) ± 2σ		40(r)/39(k) ± 2σ		Age ± 2σ (Ma)		MSWD
Normal Isochron	**293.45**	**± 2.55**<br>**± 0.87%**	0.50495	± 0.00845<br>± 1.67%	**1.49**	**± 0.02**<br>**± 1.68%**	0.99<br>47%
					Full External Error	± 0.04	
					Analytical Error	± 0.02	

Statistics				
	2σ Confidence Limit	1.69	Convergence	0.000002890047
	Error Magnification	1.0000	Number of Iterations	11
	Number of Data Points	19	Calculated Line	Weighted York-2

Inverse Isochron			39(k)/40(a+r) ± 2σ	36(a)/40(a+r) ± 2σ	r.i.
18D00615	1.8 %	✓	0.2095497 ± 0.0009089	0.00305093 ± 0.00003994	0.1355
18D00617	1.9 %	✓	0.3151405 ± 0.0017885	0.00284580 ± 0.00005576	0.1618
18D00618	2.0 %	✓	0.3723575 ± 0.0026032	0.00278041 ± 0.00007140	0.1786
18D00620	2.2 %	✓	0.6391185 ± 0.0017742	0.00227224 ± 0.00003492	0.1180
18D00621	2.4 %	✓	0.6422043 ± 0.0069023	0.00232665 ± 0.00011245	0.1913
18D00623	2.7 %	✓	0.7916058 ± 0.0022660	0.00202482 ± 0.00003633	0.1104
18D00624	3.0 %	✓	0.8645053 ± 0.0064253	0.00193551 ± 0.00008189	0.1565
18D00626	3.4 %	✓	0.9254665 ± 0.0037579	0.00179823 ± 0.00004806	0.1243
18D00627	3.9 %	✓	0.9678777 ± 0.0055723	0.00176162 ± 0.00006462	0.1388
18D00629	4.5 %	✓	0.9806623 ± 0.0040894	0.00174923 ± 0.00004811	0.1270
18D00630	5.2 %	✓	0.9678770 ± 0.0026055	0.00172305 ± 0.00003360	0.0940
18D00632	6.0 %	✓	1.0107817 ± 0.0065593	0.00170964 ± 0.00007276	0.1366
18D00633	6.9 %	✓	0.9105922 ± 0.0038787	0.00183150 ± 0.00004990	0.1289
18D00635	7.9 %	✓	0.8556846 ± 0.0040538	0.00198387 ± 0.00005617	0.1416
18D00636	9.0 %	✓	0.7206115 ± 0.0029515	0.00217692 ± 0.00004909	0.1410
18D00638	10.3 %	✓	0.5733085 ± 0.0017608	0.00244539 ± 0.00003757	0.1337
18D00639	11.6 %	✓	0.4116594 ± 0.0009558	0.00269317 ± 0.00003233	0.0917
18D00641	12.5 %	✓	0.4111297 ± 0.0019146	0.00269945 ± 0.00005288	0.1624
18D00642	13.4 %	✓	0.3467087 ± 0.0014092	0.00283224 ± 0.00004566	0.1501
18D00644	14.6 %		0.3448082 ± 0.0022808	0.00288628 ± 0.00006653	0.1776
18D00645	16.0 %		0.3602379 ± 0.0050467	0.00290788 ± 0.00013558	0.2082
18D00647	17.6 %		0.3029898 ± 0.0034103	0.00294644 ± 0.00010442	0.1888
18D00648	19.3 %		0.2512774 ± 0.0022533	0.00309177 ± 0.00008260	0.1740
18D00650	21.0 %		0.2000774 ± 0.0020226	0.00309316 ± 0.00008555	0.1479

Results	40(a)/36(a) ± 2σ	40(r)/39(k) ± 2σ	Age ± 2σ (Ma)	MSWD
Inverse Isochron	**293.43** **± 2.56** **± 0.87%**	0.50532 ± 0.00844 ± 1.67%	**1.49** **± 0.02** **± 1.68%**	0.98 48%
			Full External Error ± 0.04	
			Analytical Error ± 0.02	

Statistics				
2σ Confidence Limit	1.69	Convergence	0.0004579159
Error Magnification	1.0000	Number of Iterations	3
Number of Data Points	19	Calculated Line	Weighted York-2
Spreading Factor	40.5%		

**Degassing Patterns**			36Ar(a) [fA]	%1σ	36Ar(c) [fA]	%1σ	36Ar(ca) [fA]	%1σ	36Ar(cl) [fA]	%1σ	37Ar(ca) [fA]	%1σ	38Ar(a) [fA]	%1σ	38Ar(c) [fA]	%1σ	38Ar(k) [fA]	%1σ	38Ar(ca) [fA]	%1σ	38Ar(cl) [fA]	%1σ	39Ar(k) [fA]	%1σ	39Ar(ca) [fA]	%1σ	40Ar(r) [fA]	%1σ	40Ar(a) [fA]	%1σ	40Ar(c) [fA]	%1σ	40Ar(k) [fA]	%1σ
18D00615	1.8 %	✓	0.2234229	0.64	0.0000000	0.00	0.0127205	6.27	0.0000000	0.00	47.0608	6.27	0.0417577	0.64	0.0000000	0.00	0.1853285	0.19	0.0084709	11.49	0.0000000	0.00	15.34557	0.17	0.0302366	6.34	7.20971	6.40	66.02146	0.68	0.0000000	0.00	0.0093148	9.65
18D00617	1.9 %	✓	0.1256537	0.96	0.0000000	0.00	0.0118535	6.66	0.0000000	0.00	43.8530	6.66	0.0234847	0.96	0.0000000	0.00	0.1680486	0.21	0.0078935	11.71	0.0000000	0.00	13.91476	0.19	0.0281755	6.72	7.02347	5.38	37.13067	0.99	0.0000000	0.00	0.0084463	9.65
18D00618	2.0 %	✓	0.0921556	1.25	0.0000000	0.00	0.0091460	8.93	0.0000011	796.98	33.8366	8.93	0.0172239	1.25	0.0000000	0.00	0.1490501	0.22	0.0060906	13.13	0.0029502	796.98	12.34165	0.21	0.0217400	8.98	5.91264	6.08	27.23199	1.27	0.0000000	0.00	0.0074914	9.65
18D00620	2.2 %	✓	0.1939679	0.76	0.0000000	0.00	0.0457591	1.94	0.0000000	0.00	169.2900	1.94	0.0362526	0.76	0.0000000	0.00	0.6588934	0.12	0.0304722	9.82	0.0000000	0.00	54.55770	0.08	0.1087688	2.14	28.04648	1.66	57.31751	0.80	0.0000000	0.00	0.0331165	9.65
18D00621	2.4 %	✓	0.0435372	2.36	0.0000000	0.00	0.0101400	7.93	0.0000000	0.00	37.5137	7.92	0.0081371	2.36	0.0000000	0.00	0.1451312	0.22	0.0067525	12.47	0.0000000	0.00	12.01716	0.20	0.0241026	7.98	5.84712	5.47	12.86524	2.38	0.0000000	0.00	0.0072944	9.65
18D00623	2.7 %	✓	0.1624711	0.89	0.0000000	0.00	0.0548807	1.70	0.0000000	0.00	203.0364	1.69	0.0303658	0.89	0.0000000	0.00	0.7671091	0.12	0.0365466	9.78	0.0000000	0.00	63.51818	0.08	0.1304509	1.93	32.22945	1.40	48.01021	0.92	0.0000000	0.00	0.0385555	9.65
18D00624	3.0 %	✓	0.0511465	2.09	0.0000000	0.00	0.0195537	4.15	0.0000000	0.00	72.3407	4.14	0.0095593	2.09	0.0000000	0.00	0.2758967	0.15	0.0130213	10.48	0.0000000	0.00	22.84481	0.12	0.0464789	4.25	11.31150	2.92	15.11379	2.10	0.0000000	0.00	0.0138668	9.65
18D00626	3.4 %	✓	0.0922488	1.32	0.0000000	0.00	0.0405336	2.16	0.0000000	0.00	149.9580	2.15	0.0172413	1.32	0.0000000	0.00	0.5733689	0.12	0.0269924	9.87	0.0000000	0.00	47.47610	0.09	0.0963480	2.34	24.04013	1.57	27.25952	1.34	0.0000000	0.00	0.0288180	9.65
18D00627	3.9 %	✓	0.0606114	1.81	0.0000000	0.00	0.0281118	2.84	0.0000000	0.00	104.0024	2.83	0.0113283	1.81	0.0000000	0.00	0.4021808	0.13	0.0187204	10.04	0.0000000	0.00	33.30138	0.10	0.0668215	2.98	16.49594	2.07	17.91066	1.83	0.0000000	0.00	0.0202139	9.65
18D00629	4.5 %	✓	0.0859128	1.36	0.0000000	0.00	0.0401495	2.05	0.0000000	0.00	148.5367	2.04	0.0160571	1.36	0.0000000	0.00	0.5816860	0.12	0.0267366	9.84	0.0000000	0.00	48.16478	0.08	0.0954348	2.24	23.72730	1.53	25.38724	1.38	0.0000000	0.00	0.0292360	9.65
18D00630	5.2 %	✓	0.1526149	0.97	0.0000000	0.00	0.0725110	1.32	0.0000000	0.00	268.2611	1.31	0.0285237	0.97	0.0000000	0.00	1.0353262	0.12	0.0482870	9.72	0.0000000	0.00	85.72710	0.08	0.1723577	1.60	43.47460	1.06	45.09772	1.00	0.0000000	0.00	0.0520364	9.65
18D00632	6.0 %	✓	0.0519399	2.11	0.0000000	0.00	0.0260243	3.21	0.0000000	0.00	96.2792	3.21	0.0097076	2.11	0.0000000	0.00	0.3708620	0.14	0.0173303	10.15	0.0000000	0.00	30.70812	0.10	0.0618594	3.34	15.03232	2.25	15.34824	2.12	0.0000000	0.00	0.0186398	9.65
18D00633	6.9 %	✓	0.0884440	1.35	0.0000000	0.00	0.0388040	2.20	0.0000000	0.00	143.5588	2.19	0.0165302	1.35	0.0000000	0.00	0.5310599	0.13	0.0258406	9.88	0.0000000	0.00	43.97283	0.09	0.0922365	2.38	22.15516	1.67	26.13522	1.37	0.0000000	0.00	0.0266915	9.65
18D00635	7.9 %	✓	0.0847729	1.40	0.0000000	0.00	0.0319006	2.67	0.0000000	0.00	118.0192	2.67	0.0158440	1.40	0.0000000	0.00	0.4415877	0.13	0.0212435	9.99	0.0000000	0.00	36.56435	0.09	0.0758273	2.82	17.68073	2.08	25.05038	1.42	0.0000000	0.00	0.0221946	9.65
18D00636	9.0 %	✓	0.1120236	1.11	0.0000000	0.00	0.0342431	2.48	0.0000000	0.00	126.6855	2.48	0.0209372	1.11	0.0000000	0.00	0.4478455	0.13	0.0228034	9.94	0.0000000	0.00	37.08251	0.10	0.0813955	2.64	18.35681	2.11	33.10297	1.14	0.0000000	0.00	0.0225091	9.65
18D00638	10.3 %	✓	0.1816405	0.76	0.0000000	0.00	0.0449769	1.85	0.0000096	100.99	166.3963	1.84	0.0339486	0.76	0.0000000	0.00	0.5142941	0.13	0.0299513	9.81	0.0246686	100.99	42.58459	0.09	0.1069096	2.06	20.60391	2.12	53.67476	0.79	0.0000000	0.00	0.0258488	9.65
18D00639	11.6 %	✓	0.3177920	0.59	0.0000000	0.00	0.0730432	1.35	0.0000448	21.21	270.2300	1.34	0.0593953	0.59	0.0000000	0.00	0.5866459	0.12	0.0486414	9.72	0.1151669	21.23	48.57546	0.08	0.1736228	1.63	24.09162	2.53	93.90753	0.64	0.0000000	0.00	0.0294853	9.65
18D00641	12.5 %	✓	0.1306720	0.96	0.0000000	0.00	0.0343883	2.48	0.0000167	55.33	127.2228	2.47	0.0244226	0.96	0.0000000	0.00	0.2403507	0.16	0.0229001	9.94	0.0429828	55.33	19.90153	0.13	0.0817407	2.64	9.79335	4.01	38.61358	0.99	0.0000000	0.00	0.0120802	9.65
18D00642	13.4 %	✓	0.1681640	0.79	0.0000000	0.00	0.0409400	2.09	0.0000241	38.62	151.4615	2.08	0.0314298	0.79	0.0000000	0.00	0.2486150	0.16	0.0272631	9.85	0.0620018	38.63	20.58582	0.13	0.0973140	2.27	9.68252	4.34	49.69245	0.83	0.0000000	0.00	0.0124956	9.65
18D00644	14.6 %		0.1034119	1.12	0.0000000	0.00	0.0275266	2.93	0.0000074	123.69	101.8371	2.92	0.0193277	1.12	0.0000000	0.00	0.1491997	0.22	0.0183307	10.06	0.0191188	123.69	12.35404	0.20	0.0654303	3.07	5.27051	6.88	30.55822	1.15	0.0000000	0.00	0.0074989	9.65
18D00645	16.0 %		0.0459882	2.26	0.0000000	0.00	0.0111078	7.24	0.0000000	0.00	41.0943	7.23	0.0085952	2.26	0.0000000	0.00	0.0688048	0.40	0.0073970	12.04	0.0000000	0.00	5.69718	0.39	0.0264031	7.29	2.22554	14.47	13.58950	2.27	0.0000000	0.00	0.0034582	9.66
18D00647	17.6 %		0.0628983	1.72	0.0000000	0.00	0.0176348	4.62	0.0000081	116.51	65.2418	4.61	0.0117557	1.72	0.0000000	0.00	0.0781138	0.37	0.0117435	10.68	0.0207014	116.52	6.46798	0.36	0.0419178	4.71	2.76075	12.15	18.58644	1.73	0.0000000	0.00	0.0039261	9.66
18D00648	19.3 %		0.0888677	1.30	0.0000000	0.00	0.0293033	2.94	0.0000085	107.00	108.4103	2.93	0.0166094	1.30	0.0000000	0.00	0.0872266	0.32	0.0195139	10.07	0.0218867	107.00	7.22254	0.31	0.0696536	3.07	2.48288	14.43	26.26040	1.32	0.0000000	0.00	0.0043841	9.66
18D00650	21.0 %		0.0889092	1.34	0.0000000	0.00	0.0299825	2.95	0.0000139	63.69	110.9229	2.94	0.0166171	1.34	0.0000000	0.00	0.0694547	0.40	0.0199661	10.07	0.0357562	63.70	5.75099	0.39	0.0712680	3.08	2.47113	14.99	26.27267	1.37	0.0000000	0.00	0.0034908	9.66
		Σ	2.8092669	0.22	0.0000000	0.00	0.7852348	0.53	0.0001343	20.72	2905.0491	0.53	0.5250520	0.22	0.0000000	0.00	8.7760799	0.03	0.5229088	2.28	0.3452334	20.72	726.67714	0.02	1.8664941	0.57	357.92558	0.54	830.13838	0.23	0.0000000	0.00	0.4410930	2.38
		Σ							3.5946361	0.21	2905.0491	0.53									10.169274	0.71			728.54364	0.02							1188.5050	0.23

**Additional Parameters**			40Ar/39Ar	1σ	37Ar/39Ar	1σ	36Ar/39Ar	1σ	Time (days)	37Ar (decay)	39Ar (decay)	40Ar (moles)
18D00615	1.8 %	✓	4.763359	0.010301	3.060705	0.191983	0.015358	0.000081	239.439	114.355284	1.00169447	3.516E-12
18D00617	1.9 %	✓	3.167381	0.008968	3.145173	0.209591	0.009862	0.000068	239.453	114.388229	1.00169457	2.120E-12
18D00618	2.0 %	✓	2.681474	0.009356	2.736836	0.244453	0.008194	0.000068	239.460	114.403920	1.00169462	1.591E-12
18D00620	2.2 %	✓	1.562148	0.002164	3.096779	0.060046	0.004385	0.000022	239.474	114.435309	1.00169472	4.099E-12
18D00621	2.4 %	✓	1.554626	0.008345	3.115432	0.246958	0.004458	0.000054	239.481	114.451007	1.00169477	8.985E-13
18D00623	2.7 %	✓	1.261272	0.001802	3.189957	0.054111	0.003415	0.000018	239.495	114.482409	1.00169487	3.853E-12
18D00624	3.0 %	✓	1.154988	0.004288	3.160187	0.131036	0.003089	0.000031	239.502	114.498113	1.00169492	1.269E-12
18D00626	3.4 %	✓	1.078954	0.002187	3.152202	0.067798	0.002791	0.000018	239.516	114.529528	1.00169501	2.464E-12
18D00627	3.9 %	✓	1.031725	0.002967	3.116811	0.088352	0.002659	0.000023	239.523	114.545239	1.00169506	1.652E-12
18D00629	4.5 %	✓	1.018308	0.002120	3.077829	0.062782	0.002612	0.000017	239.537	114.576667	1.00169516	2.359E-12
18D00630	5.2 %	✓	1.031722	0.001386	3.122966	0.041010	0.002621	0.000013	239.544	114.592384	1.00169521	4.254E-12
18D00632	6.0 %	✓	0.987950	0.003202	3.128999	0.100382	0.002534	0.000023	239.558	114.623825	1.00169531	1.459E-12
18D00633	6.9 %	✓	1.096493	0.002332	3.257883	0.071434	0.002888	0.000019	239.565	114.641121	1.00169536	2.319E-12
18D00635	7.9 %	✓	1.166842	0.002760	3.221033	0.085984	0.003184	0.000023	239.579	114.672575	1.00169546	2.052E-12
18D00636	9.0 %	✓	1.385277	0.002833	3.408833	0.084519	0.003936	0.000025	239.586	114.688306	1.00169551	2.471E-12
18D00638	10.3 %	✓	1.740499	0.002668	3.897645	0.071989	0.005308	0.000026	239.600	114.719773	1.00169561	3.567E-12
18D00639	11.6 %	✓	2.421146	0.002801	5.543283	0.074517	0.008018	0.000034	239.607	114.735510	1.00169566	5.665E-12
18D00641	12.5 %	✓	2.422978	0.005627	6.366468	0.157489	0.008261	0.000047	239.621	114.766990	1.00169576	2.324E-12
18D00642	13.4 %	✓	2.871299	0.005815	7.322947	0.152518	0.010111	0.000051	239.628	114.782733	1.00169580	2.851E-12
18D00644	14.6 %		2.885488	0.009511	8.199794	0.240313	0.010544	0.000071	239.642	114.814226	1.00169590	1.720E-12
18D00645	16.0 %		2.763742	0.019306	7.179829	0.520099	0.009976	0.000121	239.649	114.829976	1.00169595	7.593E-13
18D00647	17.6 %		3.279792	0.018381	10.021932	0.463814	0.012372	0.000118	239.663	114.863057	1.00169606	1.025E-12
18D00648	19.3 %		3.942253	0.017555	14.866627	0.438205	0.016206	0.000116	239.670	114.878814	1.00169610	1.380E-12
18D00650	21.0 %		4.937485	0.024704	19.051540	0.565147	0.020423	0.000159	239.684	114.910333	1.00169620	1.380E-12

Procedure Blanks		36Ar ± 1σ (SE) [fA]	37Ar ± 1σ (SE) [fA]	38Ar ± 1σ (SE) [fA]	39Ar ± 1σ (SE) [fA]	40Ar ± 1σ (SE) [fA]
18D00615	1.8 %	0.0175263 ± 0.0004146	0.0221393 ± 0.0181643	0.0184911 ± 0.0167625	0.0280778 ± 0.0157445	5.2622421 ± 0.0905427
18D00617	1.9 %	0.0175010 ± 0.0004146	0.0193989 ± 0.0181643	0.0183887 ± 0.0167625	0.0218576 ± 0.0157445	5.2277488 ± 0.0905427
18D00618	2.0 %	0.0174903 ± 0.0004146	0.0190451 ± 0.0181643	0.0173431 ± 0.0167625	0.0191021 ± 0.0157445	5.2146696 ± 0.0905427
18D00620	2.2 %	0.0174686 ± 0.0004146	0.0195067 ± 0.0181643	0.0140403 ± 0.0167625	0.0143693 ± 0.0157445	5.1937811 ± 0.0905427
18D00621	2.4 %	0.0174563 ± 0.0004146	0.0200935 ± 0.0181643	0.0120307 ± 0.0167625	0.0125113 ± 0.0157445	5.1855457 ± 0.0905427
18D00623	2.7 %	0.0174274 ± 0.0004146	0.0214933 ± 0.0181643	0.0078302 ± 0.0167625	0.0100388 ± 0.0157445	5.1725434 ± 0.0905427
18D00624	3.0 %	0.0174102 ± 0.0004146	0.0221463 ± 0.0181643	0.0058199 ± 0.0167625	0.0094863 ± 0.0157445	5.1674429 ± 0.0905427
18D00626	3.4 %	0.0173700 ± 0.0004146	0.0230443 ± 0.0181643	0.0023469 ± 0.0167625	0.0098325 ± 0.0157445	5.1593250 ± 0.0905427
18D00627	3.9 %	0.0173471 ± 0.0004146	0.0231978 ± 0.0181643	0.0009978 ± 0.0167625	0.0107359 ± 0.0157445	5.1560666 ± 0.0905427
18D00629	4.5 %	0.0172968 ± 0.0004146	0.0227711 ± 0.0181643	0.0007252 ± 0.0167625	0.0139438 ± 0.0157445	5.1506640 ± 0.0905427
18D00630	5.2 %	0.0172700 ± 0.0004146	0.0221681 ± 0.0181643	0.0010524 ± 0.0167625	0.0161958 ± 0.0157445	5.1483713 ± 0.0905427
18D00632	6.0 %	0.0172160 ± 0.0004146	0.0202112 ± 0.0181643	0.0006053 ± 0.0167625	0.0217929 ± 0.0157445	5.1443474 ± 0.0905427
18D00633	6.9 %	0.0171874 ± 0.0004146	0.0187622 ± 0.0181643	0.0002423 ± 0.0167625	0.0253641 ± 0.0157445	5.1423891 ± 0.0905427
18D00635	7.9 %	0.0171418 ± 0.0004146	0.0156639 ± 0.0181643	0.0027328 ± 0.0167625	0.0323860 ± 0.0157445	5.1392703 ± 0.0905427
18D00636	9.0 %	0.0171240 ± 0.0004146	0.0140093 ± 0.0181643	0.0043463 ± 0.0167625	0.0359802 ± 0.0157445	5.1379644 ± 0.0905427
18D00638	10.3 %	0.0171038 ± 0.0004146	0.0108665 ± 0.0181643	0.0080023 ± 0.0167625	0.0428409 ± 0.0157445	5.1360745 ± 0.0905427
18D00639	11.6 %	0.0171039 ± 0.0004146	0.0095635 ± 0.0181643	0.0098885 ± 0.0167625	0.0458812 ± 0.0157445	5.1356226 ± 0.0905427
18D00641	12.5 %	0.0171315 ± 0.0004146	0.0080430 ± 0.0181643	0.0133021 ± 0.0167625	0.0505446 ± 0.0157445	5.1361509 ± 0.0905427
18D00642	13.4 %	0.0171620 ± 0.0004146	0.0080793 ± 0.0181643	0.0146063 ± 0.0167625	0.0518842 ± 0.0157445	5.1373557 ± 0.0905427
18D00644	14.6 %	0.0172649 ± 0.0004146	0.0104653 ± 0.0181643	0.0157670 ± 0.0167625	0.0517988 ± 0.0157445	5.1423239 ± 0.0905427
18D00645	16.0 %	0.0173409 ± 0.0004146	0.0131373 ± 0.0181643	0.0153333 ± 0.0167625	0.0500330 ± 0.0157445	5.1464044 ± 0.0905427
18D00647	17.6 %	0.0175648 ± 0.0004146	0.0229465 ± 0.0181643	0.0113242 ± 0.0167625	0.0415624 ± 0.0157445	5.1594456 ± 0.0905427
18D00648	19.3 %	0.0177065 ± 0.0004146	0.0300319 ± 0.0181643	0.0075601 ± 0.0167625	0.0348383 ± 0.0157445	5.1682207 ± 0.0905427
18D00650	21.0 %	0.0180693 ± 0.0004146	0.0499643 ± 0.0181643	0.0045590 ± 0.0167625	0.0150910 ± 0.0157445	5.1919042 ± 0.0905427

**Intercept Values**		**36Ar ± 1σ (SE) [fA]**	**r2**	**Regression (type,n)**	**37Ar ± 1σ (SE) [fA]**	**r2**	**Regression (type,n)**	**38Ar ± 1σ (SE) [fA]**	**r2**	**Regression (type,n)**	**39Ar ± 1σ (SE) [fA]**	**r2**	**Regression (type,n)**	**40Ar ± 1σ (SE) [fA]**	**r2**	**Regression (type,n)**
18D00615	1.8 %	0.2421972 ± 0.0008373	0.0319	EXP 150 of 150	0.3821118 ± 0.0174494	0.0211	EXP 150 of 150	0.2305611 ± 0.0177626	0.0008	EXP 150 of 150	15.2873988 ± 0.0167914	0.9613	EXP 149 of 150	78.502729 ± 0.046048	0.9983	EXP 150 of 150
18D00617	1.9 %	0.1483276 ± 0.0006595	0.0405	EXP 150 of 150	0.3571883 ± 0.0170971	0.0344	EXP 150 of 150	0.1985948 ± 0.0169326	0.0009	EXP 150 of 150	13.8591611 ± 0.0182982	0.9481	EXP 150 of 150	49.390338 ± 0.023909	0.9993	EXP 150 of 150
18D00618	2.0 %	0.1138716 ± 0.0005968	0.2330	EXP 150 of 150	0.2714866 ± 0.0184103	0.0010	EXP 150 of 150	0.1905875 ± 0.0160612	0.0116	EXP 150 of 150	12.2888286 ± 0.0175215	0.9364	EXP 150 of 150	38.366791 ± 0.024647	0.9993	EXP 150 of 150
18D00620	2.2 %	0.2455489 ± 0.0008168	0.1932	EXP 150 of 150	1.4336727 ± 0.0189213	0.2083	EXP 150 of 150	0.7053148 ± 0.0149487	0.1034	EXP 149 of 150	54.2666761 ± 0.0173043	0.9975	EXP 150 of 150	90.590888 ± 0.031043	0.9984	EXP 150 of 150
18D00621	2.4 %	0.0685257 ± 0.0004267	0.6623	EXP 149 of 150	0.3018790 ± 0.0177738	0.0089	EXP 150 of 150	0.1630975 ± 0.0178591	0.0001	EXP 150 of 150	11.9625457 ± 0.0159627	0.9472	EXP 150 of 150	23.905201 ± 0.022343	0.9994	EXP 150 of 150
18D00623	2.7 %	0.2242196 ± 0.0007669	0.1616	EXP 150 of 150	1.7206472 ± 0.0197768	0.2471	EXP 150 of 150	0.7742674 ± 0.0188165	0.0408	EXP 150 of 150	63.1764499 ± 0.0186804	0.9979	EXP 150 of 150	85.450760 ± 0.030227	0.9983	EXP 150 of 150
18D00624	3.0 %	0.0846756 ± 0.0004767	0.5077	EXP 150 of 150	0.5984834 ± 0.0176820	0.0294	EXP 150 of 150	0.2730758 ± 0.0183419	0.0002	EXP 150 of 150	22.7273370 ± 0.0166003	0.9858	EXP 150 of 150	31.606603 ± 0.019814	0.9994	EXP 150 of 150
18D00626	3.4 %	0.1437015 ± 0.0006000	0.1087	EXP 150 of 150	1.2631310 ± 0.0187648	0.1243	EXP 150 of 150	0.5781959 ± 0.0161742	0.0259	EXP 150 of 150	47.2218529 ± 0.0186421	0.9961	EXP 150 of 150	56.487790 ± 0.025856	0.9987	EXP 150 of 150
18D00627	3.9 %	0.1017599 ± 0.0005382	0.3173	EXP 150 of 150	0.8686984 ± 0.0164044	0.0627	EXP 150 of 150	0.4052056 ± 0.0156391	0.0033	EXP 150 of 150	33.1261228 ± 0.0163910	0.9937	EXP 150 of 150	39.582882 ± 0.021827	0.9992	EXP 150 of 150
18D00629	4.5 %	0.1372346 ± 0.0005854	0.0354	EXP 150 of 150	1.2506898 ± 0.0162271	0.1821	EXP 149 of 150	0.5846101 ± 0.0167524	0.0201	EXP 150 of 150	47.9085068 ± 0.0163605	0.9971	EXP 150 of 150	54.294441 ± 0.023985	0.9987	EXP 150 of 150
18D00630	5.2 %	0.2314587 ± 0.0007813	0.1502	EXP 150 of 150	2.2774193 ± 0.0178015	0.3394	EXP 150 of 150	1.0224264 ± 0.0157443	0.0433	EXP 150 of 150	85.2648231 ± 0.0214557	0.9985	EXP 150 of 150	93.772722 ± 0.038116	0.9964	EXP 150 of 150
18D00632	6.0 %	0.0913924 ± 0.0004850	0.3908	EXP 150 of 150	0.8048872 ± 0.0183373	0.0316	EXP 150 of 150	0.3577467 ± 0.0167323	0.0001	EXP 150 of 150	30.5586346 ± 0.0178709	0.9913	EXP 150 of 150	35.543552 ± 0.022481	0.9989	EXP 150 of 150
18D00633	6.9 %	0.1382533 ± 0.0005842	0.1000	EXP 150 of 150	1.2113296 ± 0.0179260	0.1263	EXP 150 of 150	0.5495112 ± 0.0165267	0.0170	EXP 150 of 150	43.7565608 ± 0.0186875	0.9955	EXP 150 of 150	53.459452 ± 0.022753	0.9988	EXP 150 of 150
18D00635	7.9 %	0.1281469 ± 0.0005873	0.0203	EXP 150 of 150	0.9953129 ± 0.0186237	0.0578	EXP 150 of 150	0.4739657 ± 0.0162949	0.0065	EXP 149 of 150	36.3949445 ± 0.0159384	0.9952	EXP 150 of 150	47.892574 ± 0.021568	0.9988	EXP 150 of 150
18D00636	9.0 %	0.1562846 ± 0.0006534	0.0156	EXP 150 of 150	1.0710561 ± 0.0182862	0.1475	EXP 150 of 150	0.4901244 ± 0.0163991	0.0146	EXP 150 of 150	36.9182941 ± 0.0192191	0.9932	EXP 150 of 150	56.620253 ± 0.020442	0.9988	EXP 150 of 150
18D00638	10.3 %	0.2327206 ± 0.0007393	0.5034	EXP 150 of 150	1.4139321 ± 0.0161360	0.2071	EXP 150 of 150	0.6037456 ± 0.0177580	0.0104	EXP 150 of 150	42.4108677 ± 0.0168793	0.9960	EXP 150 of 150	79.440594 ± 0.022337	0.9976	EXP 150 of 150
18D00639	11.6 %	0.3889938 ± 0.0010412	0.7627	EXP 150 of 150	2.3040121 ± 0.0191885	0.2638	EXP 150 of 150	0.8101741 ± 0.0166446	0.1102	EXP 150 of 150	48.4255897 ± 0.0165269	0.9971	EXP 150 of 150	123.164261 ± 0.026333	0.9907	EXP 150 of 150
18D00641	12.5 %	0.1741886 ± 0.0006337	0.2201	EXP 150 of 150	1.0808773 ± 0.0182992	0.1865	EXP 150 of 150	0.3400535 ± 0.0162959	0.0222	EXP 150 of 150	19.8823931 ± 0.0151550	0.9846	EXP 150 of 150	53.555158 ± 0.021930	0.9980	EXP 150 of 150
18D00642	13.4 %	0.2161301 ± 0.0006742	0.5587	EXP 150 of 150	1.2881257 ± 0.0176862	0.1433	EXP 150 of 150	0.3795546 ± 0.0164610	0.0495	EXP 150 of 150	20.5782966 ± 0.0155989	0.9844	EXP 150 of 150	64.524826 ± 0.021726	0.9977	EXP 150 of 150
18D00644	14.6 %	0.1418491 ± 0.0005805	0.1050	EXP 150 of 150	0.8608159 ± 0.0167728	0.1007	EXP 150 of 150	0.2193114 ± 0.0161688	0.0128	EXP 150 of 150	12.3771620 ± 0.0173893	0.9447	EXP 150 of 150	40.978557 ± 0.022140	0.9983	EXP 150 of 150
18D00645	16.0 %	0.0716630 ± 0.0004420	0.4551	EXP 150 of 150	0.3384026 ± 0.0176231	0.0001	EXP 150 of 150	0.0944292 ± 0.0162525	0.0022	EXP 150 of 150	5.7302429 ± 0.0146438	0.7864	EXP 150 of 150	20.964901 ± 0.017540	0.9992	EXP 150 of 150
18D00647	17.6 %	0.0941930 ± 0.0004881	0.1364	EXP 150 of 150	0.5350010 ± 0.0178175	0.0377	EXP 150 of 150	0.1321941 ± 0.0168913	0.0011	EXP 150 of 150	6.5021324 ± 0.0160433	0.8137	EXP 149 of 150	26.510562 ± 0.019582	0.9989	EXP 150 of 150
18D00648	19.3 %	0.1301445 ± 0.0005085	0.0036	EXP 150 of 150	0.8969657 ± 0.0191297	0.0622	EXP 150 of 150	0.1510815 ± 0.0158280	0.0003	EXP 150 of 150	7.2717729 ± 0.0148320	0.8599	EXP 150 of 150	33.915886 ± 0.020305	0.9989	EXP 150 of 150
18D00650	21.0 %	0.1311981 ± 0.0005608	0.0082	EXP 150 of 150	0.8982582 ± 0.0200856	0.0278	EXP 150 of 150	0.1355607 ± 0.0148744	0.0018	EXP 150 of 150	5.7932266 ± 0.0150661	0.7943	EXP 150 of 150	33.939200 ± 0.018449	0.9989	EXP 150 of 150

Project Info		Analyst	Irradiation	X-pos	Y-pos	Z/H-pos	Project	Experiment	Nmb
18D00615	1.8 %	Dan Miggins	17-OSU-05	0.00	0.00	16.48	Arctic\O-Connor (16-22)	18D00611	01
18D00617	1.9 %	Dan Miggins	17-OSU-05	0.00	0.00	16.48	Arctic\O-Connor (16-22)	18D00611	01
18D00618	2.0 %	Dan Miggins	17-OSU-05	0.00	0.00	16.48	Arctic\O-Connor (16-22)	18D00611	01
18D00620	2.2 %	Dan Miggins	17-OSU-05	0.00	0.00	16.48	Arctic\O-Connor (16-22)	18D00611	01
18D00621	2.4 %	Dan Miggins	17-OSU-05	0.00	0.00	16.48	Arctic\O-Connor (16-22)	18D00611	01
18D00623	2.7 %	Dan Miggins	17-OSU-05	0.00	0.00	16.48	Arctic\O-Connor (16-22)	18D00611	01
18D00624	3.0 %	Dan Miggins	17-OSU-05	0.00	0.00	16.48	Arctic\O-Connor (16-22)	18D00611	01
18D00626	3.4 %	Dan Miggins	17-OSU-05	0.00	0.00	16.48	Arctic\O-Connor (16-22)	18D00611	01
18D00627	3.9 %	Dan Miggins	17-OSU-05	0.00	0.00	16.48	Arctic\O-Connor (16-22)	18D00611	01
18D00629	4.5 %	Dan Miggins	17-OSU-05	0.00	0.00	16.48	Arctic\O-Connor (16-22)	18D00611	01
18D00630	5.2 %	Dan Miggins	17-OSU-05	0.00	0.00	16.48	Arctic\O-Connor (16-22)	18D00611	01
18D00632	6.0 %	Dan Miggins	17-OSU-05	0.00	0.00	16.48	Arctic\O-Connor (16-22)	18D00611	01
18D00633	6.9 %	Dan Miggins	17-OSU-05	0.00	0.00	16.48	Arctic\O-Connor (16-22)	18D00611	01
18D00635	7.9 %	Dan Miggins	17-OSU-05	0.00	0.00	16.48	Arctic\O-Connor (16-22)	18D00611	01
18D00636	9.0 %	Dan Miggins	17-OSU-05	0.00	0.00	16.48	Arctic\O-Connor (16-22)	18D00611	01
18D00638	10.3 %	Dan Miggins	17-OSU-05	0.00	0.00	16.48	Arctic\O-Connor (16-22)	18D00611	01
18D00639	11.6 %	Dan Miggins	17-OSU-05	0.00	0.00	16.48	Arctic\O-Connor (16-22)	18D00611	01
18D00641	12.5 %	Dan Miggins	17-OSU-05	0.00	0.00	16.48	Arctic\O-Connor (16-22)	18D00611	01
18D00642	13.4 %	Dan Miggins	17-OSU-05	0.00	0.00	16.48	Arctic\O-Connor (16-22)	18D00611	01
18D00644	14.6 %	Dan Miggins	17-OSU-05	0.00	0.00	16.48	Arctic\O-Connor (16-22)	18D00611	01
18D00645	16.0 %	Dan Miggins	17-OSU-05	0.00	0.00	16.48	Arctic\O-Connor (16-22)	18D00611	01
18D00647	17.6 %	Dan Miggins	17-OSU-05	0.00	0.00	16.48	Arctic\O-Connor (16-22)	18D00611	01
18D00648	19.3 %	Dan Miggins	17-OSU-05	0.00	0.00	16.48	Arctic\O-Connor (16-22)	18D00611	01
18D00650	21.0 %	Dan Miggins	17-OSU-05	0.00	0.00	16.48	Arctic\O-Connor (16-22)	18D00611	01

**Sample Parameters**		Sample	Material	Location	Standard Name	Standard (in Ma)	%1σ	Standard Reference	Standard 40Ar/39Ar	%1σ	J	%1σ	Air 40Ar/36Ar	%1σ	MDF (lin)	%1σ	Volume Ratio	Sensitivity (mol/volt)	Day	Month	Year	Hour	Min	Resist
18D00615	1.8 %	HLY0102-D66-32	Groundmass	Gakkel Ridge	FCT-NM (5B11-17)	28.201	0.082	Kuiper et al (2008)	9.65222	0.082	0.00162837	0.082	302.64	0.152	0.9940943	0.069	1	4.8E-14	5	JAN	2018	22	22	1
18D00617	1.9 %	HLY0102-D66-32	Groundmass	Gakkel Ridge	FCT-NM (5B11-17)	28.201	0.082	Kuiper et al (2008)	9.65222	0.082	0.00162837	0.082	302.64	0.152	0.9940943	0.069	1	4.8E-14	5	JAN	2018	22	43	1
18D00618	2.0 %	HLY0102-D66-32	Groundmass	Gakkel Ridge	FCT-NM (5B11-17)	28.201	0.082	Kuiper et al (2008)	9.65222	0.082	0.00162837	0.082	302.64	0.152	0.9940943	0.069	1	4.8E-14	5	JAN	2018	22	53	1
18D00620	2.2 %	HLY0102-D66-32	Groundmass	Gakkel Ridge	FCT-NM (5B11-17)	28.201	0.082	Kuiper et al (2008)	9.65222	0.082	0.00162837	0.082	302.64	0.152	0.9940943	0.069	1	4.8E-14	5	JAN	2018	23	13	1
18D00621	2.4 %	HLY0102-D66-32	Groundmass	Gakkel Ridge	FCT-NM (5B11-17)	28.201	0.082	Kuiper et al (2008)	9.65222	0.082	0.00162837	0.082	302.64	0.152	0.9940943	0.069	1	4.8E-14	5	JAN	2018	23	23	1
18D00623	2.7 %	HLY0102-D66-32	Groundmass	Gakkel Ridge	FCT-NM (5B11-17)	28.201	0.082	Kuiper et al (2008)	9.65222	0.082	0.00162837	0.082	302.64	0.152	0.9940943	0.069	1	4.8E-14	5	JAN	2018	23	43	1
18D00624	3.0 %	HLY0102-D66-32	Groundmass	Gakkel Ridge	FCT-NM (5B11-17)	28.201	0.082	Kuiper et al (2008)	9.65222	0.082	0.00162837	0.082	302.64	0.152	0.9940943	0.069	1	4.8E-14	5	JAN	2018	23	53	1
18D00626	3.4 %	HLY0102-D66-32	Groundmass	Gakkel Ridge	FCT-NM (5B11-17)	28.201	0.082	Kuiper et al (2008)	9.65222	0.082	0.00162837	0.082	302.64	0.152	0.9940943	0.069	1	4.8E-14	6	JAN	2018	0	13	1
18D00627	3.9 %	HLY0102-D66-32	Groundmass	Gakkel Ridge	FCT-NM (5B11-17)	28.201	0.082	Kuiper et al (2008)	9.65222	0.082	0.00162837	0.082	302.64	0.152	0.9940943	0.069	1	4.8E-14	6	JAN	2018	0	23	1
18D00629	4.5 %	HLY0102-D66-32	Groundmass	Gakkel Ridge	FCT-NM (5B11-17)	28.201	0.082	Kuiper et al (2008)	9.65222	0.082	0.00162837	0.082	302.64	0.152	0.9940943	0.069	1	4.8E-14	6	JAN	2018	0	43	1
18D00630	5.2 %	HLY0102-D66-32	Groundmass	Gakkel Ridge	FCT-NM (5B11-17)	28.201	0.082	Kuiper et al (2008)	9.65222	0.082	0.00162837	0.082	302.64	0.152	0.9940943	0.069	1	4.8E-14	6	JAN	2018	0	53	1
18D00632	6.0 %	HLY0102-D66-32	Groundmass	Gakkel Ridge	FCT-NM (5B11-17)	28.201	0.082	Kuiper et al (2008)	9.65222	0.082	0.00162837	0.082	302.64	0.152	0.9940943	0.069	1	4.8E-14	6	JAN	2018	1	13	1
18D00633	6.9 %	HLY0102-D66-32	Groundmass	Gakkel Ridge	FCT-NM (5B11-17)	28.201	0.082	Kuiper et al (2008)	9.65222	0.082	0.00162837	0.082	302.64	0.152	0.9940943	0.069	1	4.8E-14	6	JAN	2018	1	24	1
18D00635	7.9 %	HLY0102-D66-32	Groundmass	Gakkel Ridge	FCT-NM (5B11-17)	28.201	0.082	Kuiper et al (2008)	9.65222	0.082	0.00162837	0.082	302.64	0.152	0.9940943	0.069	1	4.8E-14	6	JAN	2018	1	44	1
18D00636	9.0 %	HLY0102-D66-32	Groundmass	Gakkel Ridge	FCT-NM (5B11-17)	28.201	0.082	Kuiper et al (2008)	9.65222	0.082	0.00162837	0.082	302.64	0.152	0.9940943	0.069	1	4.8E-14	6	JAN	2018	1	54	1
18D00638	10.3 %	HLY0102-D66-32	Groundmass	Gakkel Ridge	FCT-NM (5B11-17)	28.201	0.082	Kuiper et al (2008)	9.65222	0.082	0.00162837	0.082	302.64	0.152	0.9940943	0.069	1	4.8E-14	6	JAN	2018	2	14	1
18D00639	11.6 %	HLY0102-D66-32	Groundmass	Gakkel Ridge	FCT-NM (5B11-17)	28.201	0.082	Kuiper et al (2008)	9.65222	0.082	0.00162837	0.082	302.64	0.152	0.9940943	0.069	1	4.8E-14	6	JAN	2018	2	24	1
18D00641	12.5 %	HLY0102-D66-32	Groundmass	Gakkel Ridge	FCT-NM (5B11-17)	28.201	0.082	Kuiper et al (2008)	9.65222	0.082	0.00162837	0.082	302.64	0.152	0.9940943	0.069	1	4.8E-14	6	JAN	2018	2	44	1
18D00642	13.4 %	HLY0102-D66-32	Groundmass	Gakkel Ridge	FCT-NM (5B11-17)	28.201	0.082	Kuiper et al (2008)	9.65222	0.082	0.00162837	0.082	302.64	0.152	0.9940943	0.069	1	4.8E-14	6	JAN	2018	2	54	1
18D00644	14.6 %	HLY0102-D66-32	Groundmass	Gakkel Ridge	FCT-NM (5B11-17)	28.201	0.082	Kuiper et al (2008)	9.65222	0.082	0.00162837	0.082	302.64	0.152	0.9940943	0.069	1	4.8E-14	6	JAN	2018	3	14	1
18D00645	16.0 %	HLY0102-D66-32	Groundmass	Gakkel Ridge	FCT-NM (5B11-17)	28.201	0.082	Kuiper et al (2008)	9.65222	0.082	0.00162837	0.082	302.64	0.152	0.9940943	0.069	1	4.8E-14	6	JAN	2018	3	24	1
18D00647	17.6 %	HLY0102-D66-32	Groundmass	Gakkel Ridge	FCT-NM (5B11-17)	28.201	0.082	Kuiper et al (2008)	9.65222	0.082	0.00162837	0.082	302.64	0.152	0.9940943	0.069	1	4.8E-14	6	JAN	2018	3	45	1
18D00648	19.3 %	HLY0102-D66-32	Groundmass	Gakkel Ridge	FCT-NM (5B11-17)	28.201	0.082	Kuiper et al (2008)	9.65222	0.082	0.00162837	0.082	302.64	0.152	0.9940943	0.069	1	4.8E-14	6	JAN	2018	3	55	1
18D00650	21.0 %	HLY0102-D66-32	Groundmass	Gakkel Ridge	FCT-NM (5B11-17)	28.201	0.082	Kuiper et al (2008)	9.65222	0.082	0.00162837	0.082	302.64	0.152	0.9940943	0.069	1	4.8E-14	6	JAN	2018	4	15	1

**Irradiation Constants**		**40/36(a)**	**%1σ**	**40/36(c)**	**%1σ**	**38/36(a)**	**%1σ**	**38/36(c)**	**%1σ**	**39/37(ca)**	**%1σ**	**38/37(ca)**	**%1σ**	**36/37(ca)**	**%1σ**	**40/39(k)**	**%1σ**	**38/39(k)**	**%1σ**	**36/38(cl)**	**%1σ**	**K/Ca**	**%1σ**	**K/Cl**	**%1σ**	**Ca/Cl**	**%1σ**
18D00615	1.8 %	295.5	0.237	0.018	35	0.1869	0	1.493	3	0.000643	0.92	0.00018	9.63	0.00027	0.17	0.000607	9.65	0.012077	0.09	0	0	0.43	0	0	0	0	0
18D00617	1.9 %	295.5	0.237	0.018	35	0.1869	0	1.493	3	0.000643	0.92	0.00018	9.63	0.00027	0.17	0.000607	9.65	0.012077	0.09	0	0	0.43	0	0	0	0	0
18D00618	2.0 %	295.5	0.237	0.018	35	0.1869	0	1.493	3	0.000643	0.92	0.00018	9.63	0.00027	0.17	0.000607	9.65	0.012077	0.09	0	0	0.43	0	0	0	0	0
18D00620	2.2 %	295.5	0.237	0.018	35	0.1869	0	1.493	3	0.000643	0.92	0.00018	9.63	0.00027	0.17	0.000607	9.65	0.012077	0.09	0	0	0.43	0	0	0	0	0
18D00621	2.4 %	295.5	0.237	0.018	35	0.1869	0	1.493	3	0.000643	0.92	0.00018	9.63	0.00027	0.17	0.000607	9.65	0.012077	0.09	0	0	0.43	0	0	0	0	0
18D00623	2.7 %	295.5	0.237	0.018	35	0.1869	0	1.493	3	0.000643	0.92	0.00018	9.63	0.00027	0.17	0.000607	9.65	0.012077	0.09	0	0	0.43	0	0	0	0	0
18D00624	3.0 %	295.5	0.237	0.018	35	0.1869	0	1.493	3	0.000643	0.92	0.00018	9.63	0.00027	0.17	0.000607	9.65	0.012077	0.09	0	0	0.43	0	0	0	0	0
18D00626	3.4 %	295.5	0.237	0.018	35	0.1869	0	1.493	3	0.000643	0.92	0.00018	9.63	0.00027	0.17	0.000607	9.65	0.012077	0.09	0	0	0.43	0	0	0	0	0
18D00627	3.9 %	295.5	0.237	0.018	35	0.1869	0	1.493	3	0.000643	0.92	0.00018	9.63	0.00027	0.17	0.000607	9.65	0.012077	0.09	0	0	0.43	0	0	0	0	0
18D00629	4.5 %	295.5	0.237	0.018	35	0.1869	0	1.493	3	0.000643	0.92	0.00018	9.63	0.00027	0.17	0.000607	9.65	0.012077	0.09	0	0	0.43	0	0	0	0	0
18D00630	5.2 %	295.5	0.237	0.018	35	0.1869	0	1.493	3	0.000643	0.92	0.00018	9.63	0.00027	0.17	0.000607	9.65	0.012077	0.09	0	0	0.43	0	0	0	0	0
18D00632	6.0 %	295.5	0.237	0.018	35	0.1869	0	1.493	3	0.000643	0.92	0.00018	9.63	0.00027	0.17	0.000607	9.65	0.012077	0.09	0	0	0.43	0	0	0	0	0
18D00633	6.9 %	295.5	0.237	0.018	35	0.1869	0	1.493	3	0.000643	0.92	0.00018	9.63	0.00027	0.17	0.000607	9.65	0.012077	0.09	0	0	0.43	0	0	0	0	0
18D00635	7.9 %	295.5	0.237	0.018	35	0.1869	0	1.493	3	0.000643	0.92	0.00018	9.63	0.00027	0.17	0.000607	9.65	0.012077	0.09	0	0	0.43	0	0	0	0	0
18D00636	9.0 %	295.5	0.237	0.018	35	0.1869	0	1.493	3	0.000643	0.92	0.00018	9.63	0.00027	0.17	0.000607	9.65	0.012077	0.09	0	0	0.43	0	0	0	0	0
18D00638	10.3 %	295.5	0.237	0.018	35	0.1869	0	1.493	3	0.000643	0.92	0.00018	9.63	0.00027	0.17	0.000607	9.65	0.012077	0.09	0	0	0.43	0	0	0	0	0
18D00639	11.6 %	295.5	0.237	0.018	35	0.1869	0	1.493	3	0.000643	0.92	0.00018	9.63	0.00027	0.17	0.000607	9.65	0.012077	0.09	0	0	0.43	0	0	0	0	0
18D00641	12.5 %	295.5	0.237	0.018	35	0.1869	0	1.493	3	0.000643	0.92	0.00018	9.63	0.00027	0.17	0.000607	9.65	0.012077	0.09	0	0	0.43	0	0	0	0	0
18D00642	13.4 %	295.5	0.237	0.018	35	0.1869	0	1.493	3	0.000643	0.92	0.00018	9.63	0.00027	0.17	0.000607	9.65	0.012077	0.09	0	0	0.43	0	0	0	0	0
18D00644	14.6 %	295.5	0.237	0.018	35	0.1869	0	1.493	3	0.000643	0.92	0.00018	9.63	0.00027	0.17	0.000607	9.65	0.012077	0.09	0	0	0.43	0	0	0	0	0
18D00645	16.0 %	295.5	0.237	0.018	35	0.1869	0	1.493	3	0.000643	0.92	0.00018	9.63	0.00027	0.17	0.000607	9.65	0.012077	0.09	0	0	0.43	0	0	0	0	0
18D00647	17.6 %	295.5	0.237	0.018	35	0.1869	0	1.493	3	0.000643	0.92	0.00018	9.63	0.00027	0.17	0.000607	9.65	0.012077	0.09	0	0	0.43	0	0	0	0	0
18D00648	19.3 %	295.5	0.237	0.018	35	0.1869	0	1.493	3	0.000643	0.92	0.00018	9.63	0.00027	0.17	0.000607	9.65	0.012077	0.09	0	0	0.43	0	0	0	0	0
18D00650	21.0 %	295.5	0.237	0.018	35	0.1869	0	1.493	3	0.000643	0.92	0.00018	9.63	0.00027	0.17	0.000607	9.65	0.012077	0.09	0	0	0.43	0	0	0	0	0

18D00611.AGE >>> HLY0102-D66-32 >>> ARCTIC | O-CONNOR (16-22) PROJECT

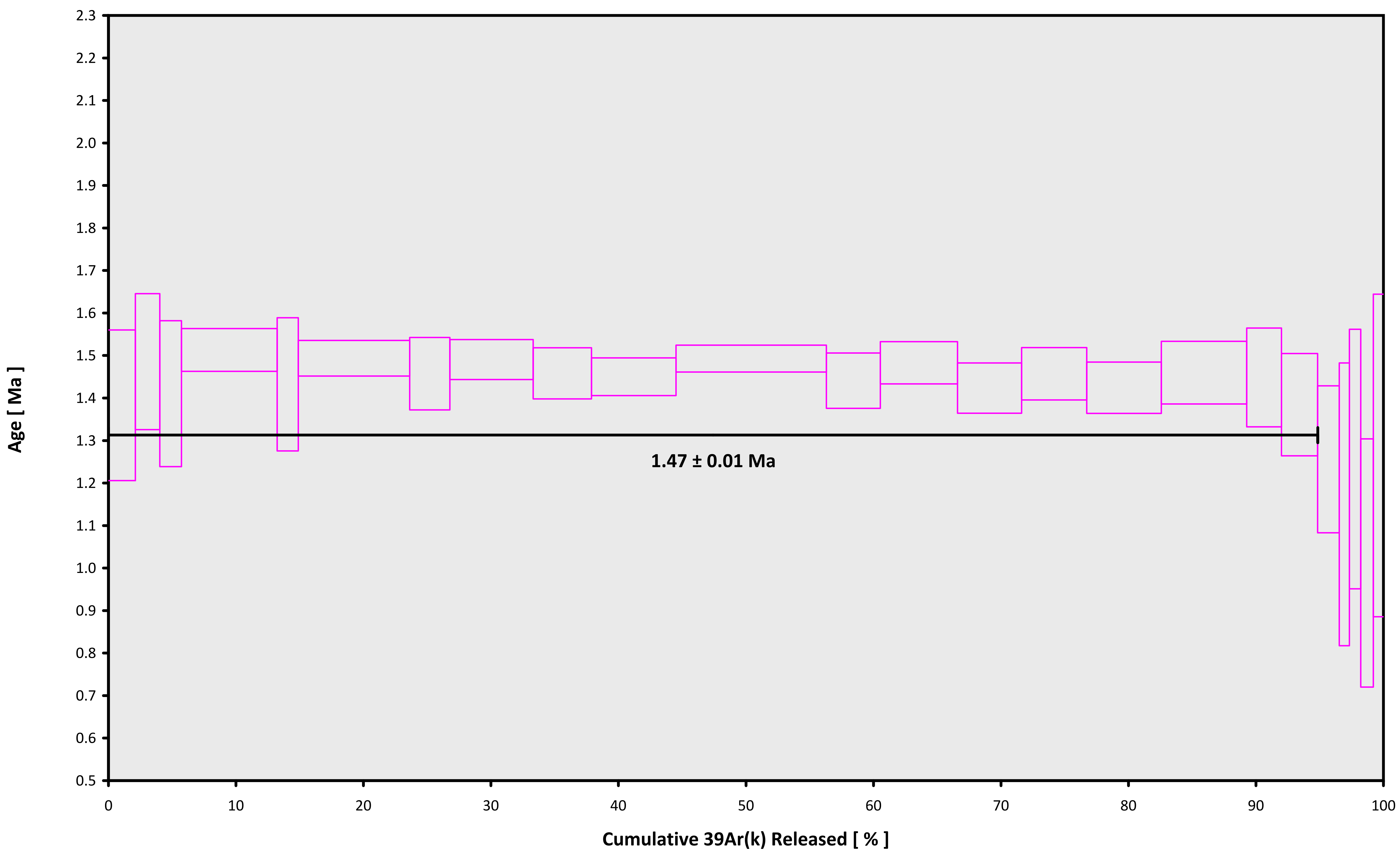

Ar-Ages in Ma

WEIGHTED PLATEAU
1.47 ± 0.01
TOTAL FUSION
1.45 ± 0.02
NORMAL ISOCHRON
1.49 ± 0.02
INVERSE ISOCHRON
1.49 ± 0.02

MSWD (PROBABILITY)
1.00 (45%)

Sample Info

Groundmass
Gakkel Ridge
Dan Miggins

IRR = 17-OSU-05 (5B11-17)
J = 0.00162837 ± 0.00000134

18D00611.AGE >>> HLY0102-D66-32 >>> ARCTIC | O-CONNOR (16-22) PROJECT

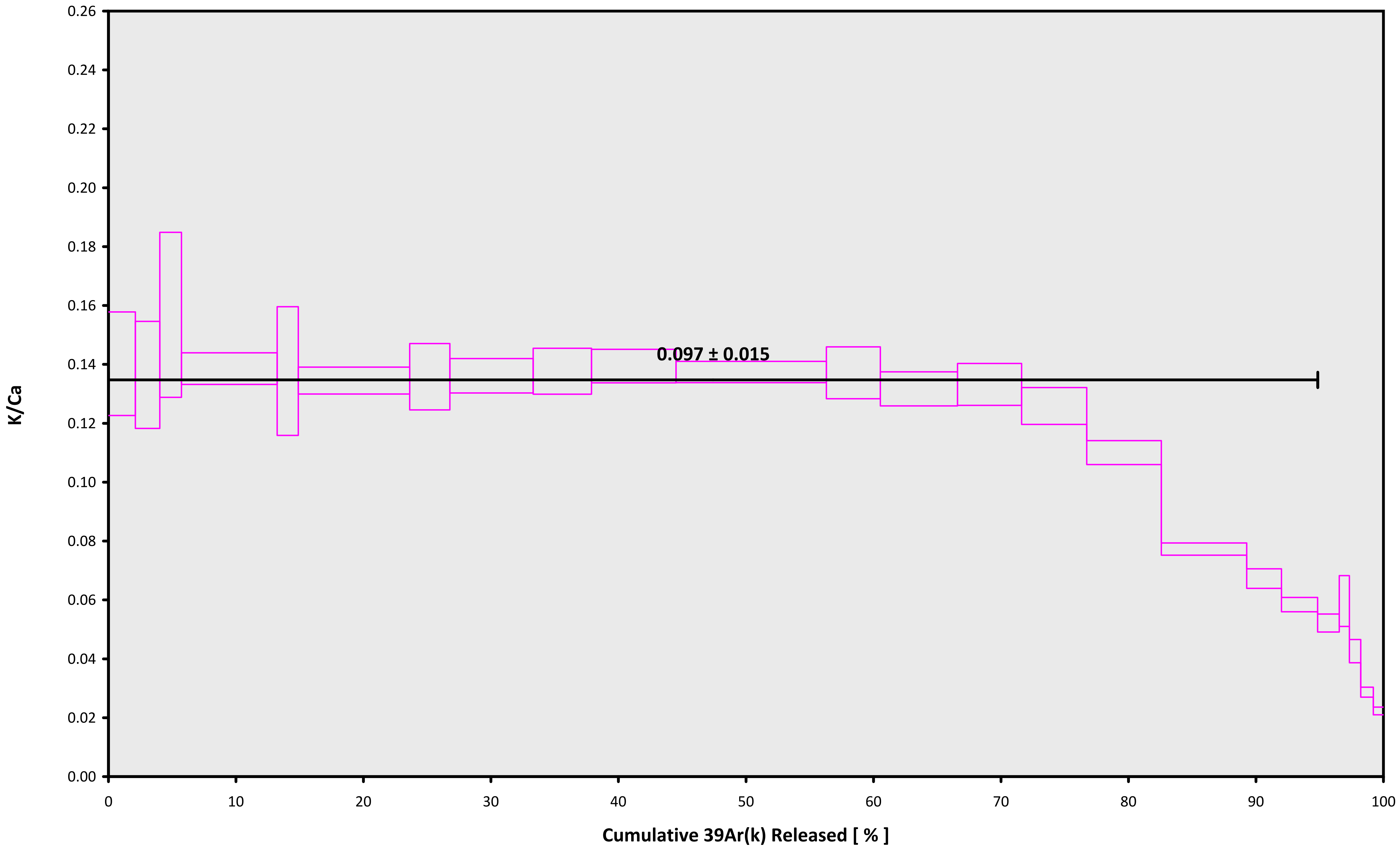

Ar-Ages in Ma

WEIGHTED PLATEAU
1.47 ± 0.01
TOTAL FUSION
1.45 ± 0.02
NORMAL ISOCHRON
1.49 ± 0.02
INVERSE ISOCHRON
1.49 ± 0.02

Sample Info

Groundmass
Gakkel Ridge
Dan Miggins

IRR = 17-OSU-05 (5B11-17)
J = 0.00162837 ± 0.00000134

18D00611.AGE >>> HLY0102-D66-32 >>> ARCTIC | O-CONNOR (16-22) PROJECT

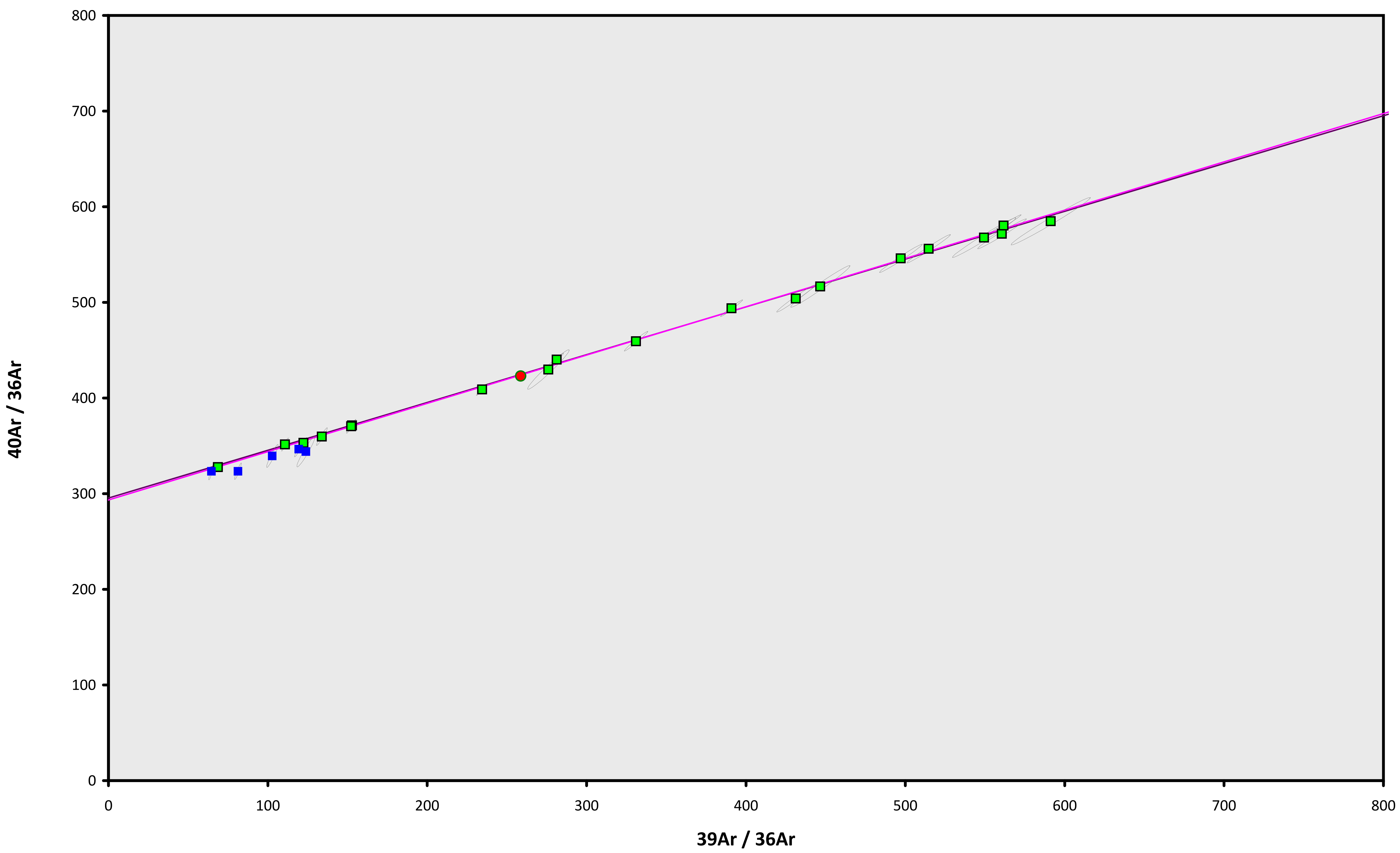

Ar-Ages in Ma

WEIGHTED PLATEAU
1.47 ± 0.01
TOTAL FUSION
1.45 ± 0.02
NORMAL ISOCHRON
1.49 ± 0.02
INVERSE ISOCHRON
1.49 ± 0.02

MSWD (PROBABILITY)
0.99 (47%)

40AR/36AR INTERCEPT
293.4 ± 2.6

Sample Info

Groundmass
Gakkel Ridge
Dan Miggins

IRR = 17-OSU-05 (5B11-17)
J = 0.00162837 ± 0.00000134

18D00611.AGE >>> HLY0102-D66-32 >>> ARCTIC | O-CONNOR (16-22) PROJECT

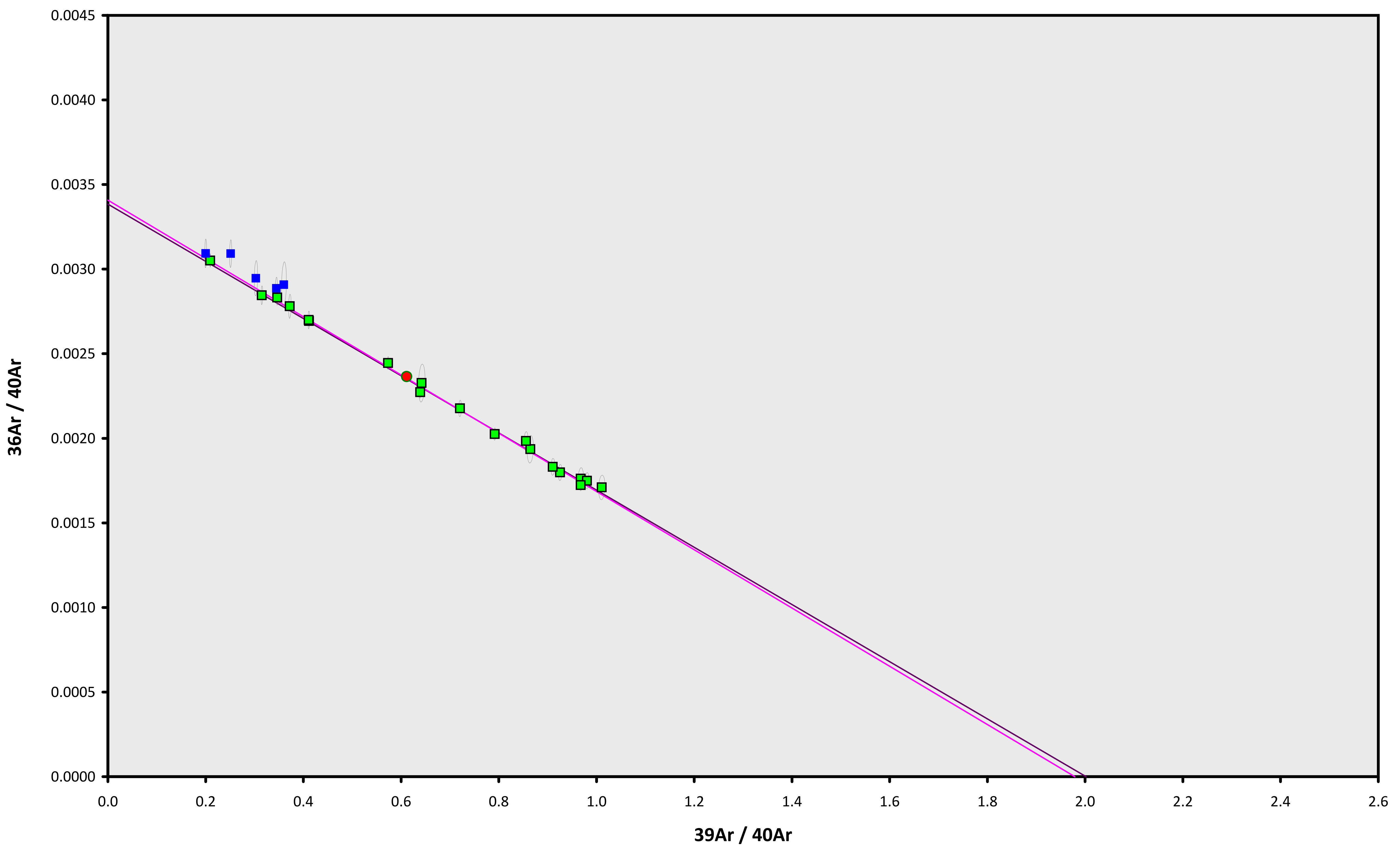

Ar-Ages in Ma

WEIGHTED PLATEAU
1.47 ± 0.01
TOTAL FUSION
1.45 ± 0.02
NORMAL ISOCHRON
1.49 ± 0.02
INVERSE ISOCHRON
1.49 ± 0.02

MSWD (PROBABILITY)
0.98 (48%)

SPREADING FACTOR
40.5%

40AR/36AR INTERCEPT
293.4 ± 2.6

Sample Info

Groundmass
Gakkel Ridge
Dan Miggins

IRR = 17-OSU-05 (5B11-17)
J = 0.00162837 ± 0.00000134

Relative Abundances			36Ar [fA]	%1σ	37Ar [fA]	%1σ	38Ar [fA]	%1σ	39Ar [fA]	%1σ	40Ar [fA]	%1σ	40(r)/39(k) ± 2σ	Age ± 2σ (Ma)	40Ar(r) (%)	39Ar(k) (%)	K/Ca ± 2σ
18D00216	1.8 %	✓	16.04502	0.275	123.8628	2.354	3.519749	0.685	37.10389	0.093	4749.927	0.023	2.31172 ± 1.42247	6.71 ± 4.12	1.80	13.04	0.1285 ± 0.0061
18D00218	1.9 %	✓	7.46898	0.280	66.7922	4.396	1.616787	1.489	16.46977	0.156	2200.671	0.050	1.83430 ± 1.50333	5.32 ± 4.36	1.37	5.79	0.1058 ± 0.0093
18D00219	2.0 %	✓	8.89277	0.280	109.9834	2.492	1.911925	1.275	19.04788	0.136	2616.899	0.042	1.84407 ± 1.54609	5.35 ± 4.48	1.34	6.68	0.0742 ± 0.0037
18D00221	2.2 %	✓	6.57259	0.282	97.3813	2.955	1.406807	1.681	13.83974	0.179	1929.576	0.057	1.63857 ± 1.58029	4.76 ± 4.58	1.17	4.85	0.0608 ± 0.0036
18D00222	2.4 %	✓	3.07494	0.308	45.9073	6.264	0.661741	3.579	6.44715	0.358	903.263	0.121	1.73184 ± 1.65263	5.03 ± 4.79	1.23	2.26	0.0601 ± 0.0075
18D00224	2.7 %	✓	3.45836	0.302	61.1437	4.531	0.726115	3.426	7.30447	0.321	1016.952	0.107	1.96917 ± 1.62517	5.71 ± 4.71	1.41	2.56	0.0511 ± 0.0046
18D00225	3.0 %	✓	6.68888	0.283	143.0284	1.984	1.436713	1.711	12.26412	0.203	1961.025	0.056	1.95064 ± 1.81799	5.66 ± 5.27	1.21	4.29	0.0366 ± 0.0015
18D00227	3.4 %	✓	3.55570	0.302	76.4112	3.497	0.753797	3.156	6.61500	0.324	1042.803	0.104	1.98059 ± 1.84525	5.75 ± 5.35	1.25	2.31	0.0369 ± 0.0026
18D00228	3.9 %	✓	7.40413	0.281	196.0015	1.567	1.566704	1.491	12.50495	0.197	2165.129	0.050	1.91107 ± 1.97153	5.55 ± 5.71	1.09	4.36	0.0272 ± 0.0009
18D00230	4.5 %	✓	4.97238	0.289	146.0211	2.048	1.040584	2.461	8.40653	0.278	1451.770	0.075	1.77548 ± 1.99426	5.15 ± 5.78	1.02	2.93	0.0245 ± 0.0010
18D00231	5.2 %	✓	4.46005	0.293	152.4733	1.948	0.950757	2.562	7.34754	0.323	1296.973	0.084	1.34060 ± 2.06101	3.89 ± 5.98	0.75	2.55	0.0204 ± 0.0008
18D00233	6.0 %	✓	6.22903	0.285	219.6697	1.458	1.294396	1.869	9.22358	0.256	1816.097	0.060	2.07095 ± 2.26948	6.01 ± 6.57	1.04	3.20	0.0178 ± 0.0005
18D00234	6.9 %	✓	7.63115	0.280	229.0008	1.431	1.583340	1.535	9.34231	0.238	2226.076	0.049	2.29175 ± 2.73178	6.65 ± 7.91	0.95	3.24	0.0173 ± 0.0005
18D00236	7.9 %	✓	11.47316	0.276	243.8026	1.316	2.288396	1.160	10.06845	0.239	3360.874	0.033	3.81551 ± 3.79430	11.06 ± 10.96	1.13	3.49	0.0175 ± 0.0005
18D00237	9.0 %		21.79434	0.274	269.0502	1.245	4.319097	0.573	12.32366	0.197	6428.375	0.017	8.28343 ± 5.87062	23.92 ± 16.84	1.57	4.28	0.0194 ± 0.0005
18D00239	10.3 %		46.10152	0.272	477.5137	0.899	9.126034	0.304	22.33314	0.121	13741.229	0.008	15.84480 ± 6.84204	45.47 ± 19.39	2.54	7.76	0.0198 ± 0.0004
18D00240	11.6 %		55.39239	0.272	564.9331	0.840	10.973038	0.272	24.67335	0.112	16514.837	0.007	17.39898 ± 7.44651	49.87 ± 21.05	2.56	8.56	0.0185 ± 0.0003
18D00242	12.5 %		29.49011	0.272	437.9195	0.918	5.823305	0.431	15.62145	0.164	8714.083	0.013	10.28675 ± 6.28072	29.65 ± 17.96	1.81	5.40	0.0151 ± 0.0003
18D00243	13.4 %		16.54012	0.275	309.7750	1.107	3.250951	0.749	9.71227	0.244	4866.031	0.022	7.58060 ± 5.69012	21.90 ± 16.34	1.48	3.35	0.0132 ± 0.0003
18D00245	14.6 %		8.59418	0.279	173.5780	1.773	1.687978	1.422	5.34552	0.424	2519.870	0.043	5.72465 ± 5.40404	16.56 ± 15.56	1.19	1.84	0.0130 ± 0.0005
18D00246	16.0 %		18.51220	0.274	449.5425	0.926	3.664692	0.654	12.29654	0.198	5448.274	0.020	7.56803 ± 5.03658	21.86 ± 14.46	1.67	4.23	0.0115 ± 0.0002
18D00248	17.6 %		11.32607	0.276	370.5108	0.998	2.262051	1.081	8.77731	0.268	3324.528	0.033	6.35899 ± 4.33757	18.39 ± 12.48	1.63	3.01	0.0099 ± 0.0002
		Σ	305.67810	0.081	4964.3021	0.314	61.864958	0.188	287.06862	0.041	90295.264	0.006					

Information on Analysis and Constants Used in Calculations

Project = **O-CONNOR (16-22)**
Sample = **PS59-229-13**
Material = **Groundmass**
Location = **Gakkel Ridge**
Region = **Artic Ocean**
Analyst = **Dan Miggins**
Irradiation = **17-OSU-05 (5B18-17)**
Position = **X: 0 | Y: 0 | Z/H: 26.49127 mm**
FCT-NM Age = **28.201 ± 0.023 Ma**
FCT-NM Reference = **Kuiper et al (2008)**
FCT-NM 40Ar/39Ar Ratio = **9.77942 ± 0.00792**
FCT-NM J-value = **0.00160719 ± 0.00000130**
Air Shot 40Ar/36Ar = **302.9580 ± 0.3969**
Air Shot MDF = **0.99383773 ± 0.00066118 (LIN)**
Experiment Type = **Incremental Heating**
Extraction Method = **Bulk Laser Heating**
Heating = **64 sec**
Isolation = **3.00 min**
Instrument = **ARGUS-VI-D**
Preferred Age = **Plateau Age**
Age Classification = **Crystallization Age**
IGSN = Undefined
Rock Class = **Igneous>Volcanic**
Lithology = **Basaltic Lava**
Lat-Lon = Undefined - Undefined

Age Equations = **Min et al. (2000)**
Negative Intensities = **Allowed**
Collector Calibrations = **36Ar**
Decay 40K = **5.530 ± 0.048 E-10 1/a**
Decay 39Ar = **2.940 ± 0.016 E-07 1/h**
Decay 37Ar = **8.230 ± 0.012 E-04 1/h**
Decay 36Cl = **2.257 ± 0.015 E-06 1/a**
Decay 40K(EC,β⁺) = **0.580 ± 0.009 E-10 1/a**
Decay 40K(β⁻) = **4.950 ± 0.043 E-10 1/a**
Atmospheric 40/36(a) = **291.31 ± 1.43**
Atmospheric 38/36(a) = **0.1869**
Production 39/37(ca) = **0.0006425 ± 0.0000059**
Production 38/37(ca) = **0.0001800 ± 0.0000173**
Production 36/37(ca) = **0.0002703 ± 0.0000005**
Production 40/39(k) = **0.000607 ± 0.000059**
Production 38/39(k) = **0.012077 ± 0.000011**
Production 36/38(cl) = **262.80 ± 1.71**
Scaling Ratio K/Ca = **0.430**
Abundance Ratio 40K/K = **1.1700 ± 0.0100 E-04**
Atomic Weight K = **39.0983 ± 0.0001 g**

Results

Results	40(a)/36(a) ± 2σ		40(r)/39(k) ± 2σ		Age ± 2σ (Ma)		MSWD	39Ar(k) (%,n)	K/Ca ± 2σ
**Age Plateau**			1.92560	± 0.48574<br>± 25.23%	**5.59**	**± 1.41<br>± 25.19%**	0.15<br>100%	61.56<br>14	0.0205 ± 0.0046
Overestimated Error				Full External Error		± 1.41	1.78	2σ Confidence Limit	
				Analytical Error		± 1.41	1.0000	Error Magnification	
**Total Fusion Age**			5.77352	± 1.04369<br>± 18.08%	**16.70**	**± 3.01<br>± 17.99%**		22	0.0246 ± 0.0002
				Full External Error		± 3.03			
				Analytical Error		± 3.01			
**Normal Isochron**	**292.77**	**± 2.08<br>± 0.71%**	1.16228	± 1.13281<br>± 97.46%	**3.37**	**± 3.29<br>± 97.37%**	0.47<br>93%	61.56<br>14	
				Full External Error		± 3.29	1.82	2σ Confidence Limit	
				Analytical Error		± 3.29	1.0000	Error Magnification	
							3	Number of Iterations	
							0.0000028765	Convergence	
**Inverse Isochron**	**292.77**	**± 2.08<br>± 0.71%**	1.16218	± 0.73542<br>± 63.28%	**3.37**	**± 2.13<br>± 63.22%**	0.47<br>93%	61.56<br>14	
Clustered Points				Full External Error		± 2.13	1.82	2σ Confidence Limit	
				Analytical Error		± 2.13	1.0000	Error Magnification	
							3	Number of Iterations	
							0.0000226994	Convergence	
							1%	Spreading Factor	

Notes

Subatmospheric Initial 40Ar/36Ar = 291.31 ± 0.49 (%SD).

**Incremental Heating**			**36Ar(a) [fA]**	**37Ar(ca) [fA]**	**38Ar(cl) [fA]**	**39Ar(k) [fA]**	**40Ar(r) [fA]**	**Age ± 2σ (Ma)**	**40Ar(r) (%)**	**39Ar(k) (%)**	**K/Ca ± 2σ**
18D00216	1.8 %	✓	16.01152	123.8628	0.0577586	37.02431	85.5898	6.71 ± 4.12	1.80	13.04	0.1285 ± 0.0061
18D00218	1.9 %	✓	7.45093	66.7922	0.0137995	16.42685	30.1319	5.32 ± 4.36	1.37	5.79	0.1058 ± 0.0093
18D00219	2.0 %	✓	8.86304	109.9834	0.0064384	18.97722	34.9953	5.35 ± 4.48	1.34	6.68	0.0742 ± 0.0037
18D00221	2.2 %	✓	6.54627	97.3813	0.0000000	13.77717	22.5748	4.76 ± 4.58	1.17	4.85	0.0608 ± 0.0036
18D00222	2.4 %	✓	3.06253	45.9073	0.0035854	6.41766	11.1144	5.03 ± 4.79	1.23	2.26	0.0601 ± 0.0075
18D00224	2.7 %	✓	3.44184	61.1437	0.0000000	7.26518	14.3063	5.71 ± 4.71	1.41	2.56	0.0511 ± 0.0046
18D00225	3.0 %	✓	6.65021	143.0284	0.0210384	12.17222	23.7437	5.66 ± 5.27	1.21	4.29	0.0366 ± 0.0015
18D00227	3.4 %	✓	3.53505	76.4112	0.0000464	6.56591	13.0044	5.75 ± 5.35	1.25	2.31	0.0369 ± 0.0026
18D00228	3.9 %	✓	7.35115	196.0015	0.0079924	12.37901	23.6571	5.55 ± 5.71	1.09	4.36	0.0272 ± 0.0009
18D00230	4.5 %	✓	4.93291	146.0211	0.0000000	8.31271	14.7590	5.15 ± 5.78	1.02	2.93	0.0245 ± 0.0010
18D00231	5.2 %	✓	4.41883	152.4733	0.0098791	7.24957	9.7188	3.89 ± 5.98	0.75	2.55	0.0204 ± 0.0008
18D00233	6.0 %	✓	6.16965	219.6697	0.0000000	9.08244	18.8093	6.01 ± 6.57	1.04	3.20	0.0178 ± 0.0005
18D00234	6.9 %	✓	7.56925	229.0008	0.0163769	9.19518	21.0731	6.65 ± 7.91	0.95	3.24	0.0173 ± 0.0005
18D00236	7.9 %	✓	11.40726	243.8026	0.0000000	9.91180	37.8186	11.06 ± 10.96	1.13	3.49	0.0175 ± 0.0005
18D00237	9.0 %		21.72159	269.0502	0.0641568	12.15080	100.6503	23.92 ± 16.84	1.57	4.28	0.0194 ± 0.0005
18D00239	10.3 %		45.97238	477.5137	0.1818325	22.02634	349.0030	45.47 ± 19.39	2.54	7.76	0.0198 ± 0.0004
18D00240	11.6 %		55.23960	564.9331	0.2534731	24.31038	422.9759	49.87 ± 21.05	2.56	8.56	0.0185 ± 0.0003
18D00242	12.5 %		29.37172	437.9195	0.0696437	15.34009	157.7996	29.65 ± 17.96	1.81	5.40	0.0151 ± 0.0003
18D00243	13.4 %		16.45639	309.7750	0.0046013	9.51324	72.1161	21.90 ± 16.34	1.48	3.35	0.0132 ± 0.0003
18D00245	14.6 %		8.54726	173.5780	0.0000000	5.23399	29.9628	16.56 ± 15.56	1.19	1.84	0.0130 ± 0.0005
18D00246	16.0 %		18.39069	449.5425	0.0015374	12.00771	90.8747	21.86 ± 14.46	1.67	4.23	0.0115 ± 0.0002
18D00248	17.6 %		11.22592	370.5108	0.0000000	8.53926	54.3011	18.39 ± 12.48	1.63	3.01	0.0099 ± 0.0002
		Σ	304.33597	4964.3021	0.7121598	283.87906	1638.9800				

Information on Analysis

Project = **O-CONNOR (16-22)**
Sample = **PS59-229-13**
Material = **Groundmass**
Location = **Gakkel Ridge**
Region = **Artic Ocean**
Analyst = **Dan Miggins**
Irradiation = **17-OSU-05 (5B18-17)**
J = **0.00160719 ± 0.00000130**
FCT-NM = **28.201 ± 0.023 Ma**

**Results**	**40(r)/39(k) ± 2σ**		**Age ± 2σ (Ma)**		**MSWD**	**39Ar(k) (%,n)**	**K/Ca ± 2σ**
**Age Plateau**<br>Overestimated Error	1.92560	± 0.48574<br>± 25.23%	**5.59**	**± 1.41**<br>**± 25.19%**	0.15<br>100%	61.56<br>14	0.0205 ± 0.0046
			Full External Error	± 1.41	1.78	2σ Confidence Limit	
			Analytical Error	± 1.41	1.0000	Error Magnification	
**Total Fusion Age**	5.77352	± 1.04369<br>± 18.08%	**16.70**	**± 3.01**<br>**± 17.99%**		22	0.0246 ± 0.0002
			Full External Error	± 3.03			
			Analytical Error	± 3.01			

Normal Isochron			39(k)/36(a) ± 2σ	40(a+r)/36(a) ± 2σ	r.i.
18D00216	1.8 %	✓	2.31 ± 0.01	296.66 ± 1.64	0.9436
18D00218	1.9 %	✓	2.20 ± 0.01	295.35 ± 1.69	0.8602
18D00219	2.0 %	✓	2.14 ± 0.01	295.26 ± 1.68	0.8895
18D00221	2.2 %	✓	2.10 ± 0.01	294.76 ± 1.71	0.8279
18D00222	2.4 %	✓	2.10 ± 0.02	294.94 ± 1.96	0.6075
18D00224	2.7 %	✓	2.11 ± 0.02	295.47 ± 1.91	0.6468
18D00225	3.0 %	✓	1.83 ± 0.01	294.88 ± 1.71	0.7957
18D00227	3.4 %	✓	1.86 ± 0.02	294.99 ± 1.90	0.6441
18D00228	3.9 %	✓	1.68 ± 0.01	294.53 ± 1.69	0.8039
18D00230	4.5 %	✓	1.69 ± 0.01	294.30 ± 1.77	0.6950
18D00231	5.2 %	✓	1.64 ± 0.01	293.51 ± 1.81	0.6438
18D00233	6.0 %	✓	1.47 ± 0.01	294.36 ± 1.73	0.7251
18D00234	6.9 %	✓	1.21 ± 0.01	294.09 ± 1.69	0.7471
18D00236	7.9 %	✓	0.87 ± 0.01	294.63 ± 1.65	0.7453
18D00237	9.0 %		0.56 ± 0.00	295.94 ± 1.63	0.8049
18D00239	10.3 %		0.48 ± 0.00	298.90 ± 1.63	0.9099
18D00240	11.6 %		0.44 ± 0.00	298.97 ± 1.63	0.9207
18D00242	12.5 %		0.52 ± 0.00	296.68 ± 1.62	0.8507
18D00243	13.4 %		0.58 ± 0.00	295.69 ± 1.64	0.7369
18D00245	14.6 %		0.61 ± 0.01	294.82 ± 1.67	0.5355
18D00246	16.0 %		0.65 ± 0.00	296.25 ± 1.64	0.7996
18D00248	17.6 %		0.76 ± 0.01	296.15 ± 1.66	0.7022

Results	40(a)/36(a) ± 2σ		40(r)/39(k) ± 2σ		Age ± 2σ (Ma)		MSWD
**Normal Isochron**	**292.77**	**± 2.08**<br>**± 0.71%**	1.16228	± 1.13281<br>± 97.46%	**3.37**	**± 3.29**<br>**± 97.37%**	0.47<br>93%
					Full External Error	± 3.29	
					Analytical Error	± 3.29	

Statistics					
	2σ Confidence Limit	1.82	Convergence	0.000002876539
	Error Magnification	1.0000	Number of Iterations	3
	Number of Data Points	14	Calculated Line	Weighted York-2

Inverse Isochron			39(k)/40(a+r) ± 2σ		36(a)/40(a+r) ± 2σ		r.i.
18D00216	1.8 %	✓	0.0077947	± 0.0000150	0.00337091	± 0.00001866	0.0200
18D00218	1.9 %	✓	0.0074645	± 0.0000245	0.00338577	± 0.00001933	0.0524
18D00219	2.0 %	✓	0.0072518	± 0.0000207	0.00338686	± 0.00001925	0.0428
18D00221	2.2 %	✓	0.0071400	± 0.0000270	0.00339261	± 0.00001963	0.0585
18D00222	2.4 %	✓	0.0071050	± 0.0000541	0.00339053	± 0.00002258	0.1146
18D00224	2.7 %	✓	0.0071441	± 0.0000487	0.00338448	± 0.00002186	0.1043
18D00225	3.0 %	✓	0.0062071	± 0.0000264	0.00339121	± 0.00001966	0.0501
18D00227	3.4 %	✓	0.0062964	± 0.0000433	0.00338996	± 0.00002183	0.0985
18D00228	3.9 %	✓	0.0057175	± 0.0000236	0.00339526	± 0.00001951	0.0428
18D00230	4.5 %	✓	0.0057259	± 0.0000335	0.00339787	± 0.00002045	0.0641
18D00231	5.2 %	✓	0.0055896	± 0.0000379	0.00340705	± 0.00002096	0.0676
18D00233	6.0 %	✓	0.0050011	± 0.0000268	0.00339722	± 0.00002000	0.0456
18D00234	6.9 %	✓	0.0041307	± 0.0000205	0.00340027	± 0.00001951	0.0337
18D00236	7.9 %	✓	0.0029492	± 0.0000145	0.00339414	± 0.00001897	0.0154
18D00237	9.0 %		0.0018902	± 0.0000076	0.00337902	± 0.00001858	0.0053
18D00239	10.3 %		0.0016029	± 0.0000040	0.00334558	± 0.00001826	0.0020
18D00240	11.6 %		0.0014720	± 0.0000034	0.00334485	± 0.00001824	0.0016
18D00242	12.5 %		0.0017604	± 0.0000059	0.00337061	± 0.00001845	0.0035
18D00243	13.4 %		0.0019550	± 0.0000099	0.00338189	± 0.00001873	0.0072
18D00245	14.6 %		0.0020771	± 0.0000182	0.00339195	± 0.00001924	0.0151
18D00246	16.0 %		0.0022040	± 0.0000091	0.00337551	± 0.00001867	0.0071
18D00248	17.6 %		0.0025686	± 0.0000144	0.00337670	± 0.00001893	0.0138

Results	40(a)/36(a) ± 2σ		40(r)/39(k) ± 2σ		Age ± 2σ (Ma)		MSWD
**Inverse Isochron** Clustered Points	**292.77**	**± 2.08** **± 0.71%**	1.16218	± 0.73542 ± 63.28%	**3.37**	**± 2.13** **± 63.22%**	0.47 93%
					Full External Error	± 2.13	
					Analytical Error	± 2.13	

Statistics				
2σ Confidence Limit	1.82	Convergence	0.0000226994
Error Magnification	1.0000	Number of Iterations	3
Number of Data Points	14	Calculated Line	Weighted York-2
Spreading Factor	0.6%		

Degassing Patterns			36Ar(a) [fA]	%1σ	36Ar(c) [fA]	%1σ	36Ar(ca) [fA]	%1σ	36Ar(cl) [fA]	%1σ	37Ar(ca) [fA]	%1σ	38Ar(a) [fA]	%1σ	38Ar(c) [fA]	%1σ	38Ar(k) [fA]	%1σ	38Ar(ca) [fA]	%1σ	38Ar(cl) [fA]	%1σ	39Ar(k) [fA]	%1σ	39Ar(ca) [fA]	%1σ	40Ar(r) [fA]	%1σ	40Ar(a) [fA]	%1σ	40Ar(c) [fA]	%1σ	40Ar(k) [fA]	%1σ
18D00216	1.8 %	✓	16.01152	0.28	0.0000000	0.00	0.0334801	2.36	0.0000222	44.29	123.8628	2.35	2.992553	0.28	0.0000000	0.00	0.4471425	0.13	0.0222953	9.91	0.0577586	44.30	37.02431	0.09	0.0795818	2.53	85.5898	30.77	4664.315	0.56	0.0000000	0.00	0.0224738	9.65
18D00218	1.9 %	✓	7.45093	0.28	0.0000000	0.00	0.0180539	4.40	0.0000053	176.96	66.7922	4.40	1.392578	0.28	0.0000000	0.00	0.1983871	0.18	0.0120226	10.59	0.0137995	176.97	16.42685	0.16	0.0429140	4.49	30.1319	40.98	2170.529	0.57	0.0000000	0.00	0.0099711	9.65
18D00219	2.0 %	✓	8.86304	0.28	0.0000000	0.00	0.0297285	2.50	0.0000025	386.68	109.9834	2.49	1.656502	0.28	0.0000000	0.00	0.2291879	0.16	0.0197970	9.95	0.0064384	386.68	18.97722	0.14	0.0706643	2.66	34.9953	41.92	2581.892	0.57	0.0000000	0.00	0.0115192	9.65
18D00221	2.2 %	✓	6.54627	0.28	0.0000000	0.00	0.0263222	2.96	0.0000000	0.00	97.3813	2.96	1.223497	0.28	0.0000000	0.00	0.1663869	0.20	0.0175286	10.07	0.0000000	0.00	13.77717	0.18	0.0625675	3.10	22.5748	48.22	1906.992	0.57	0.0000000	0.00	0.0083627	9.65
18D00222	2.4 %	✓	3.06253	0.31	0.0000000	0.00	0.0124088	6.27	0.0000014	663.07	45.9073	6.26	0.572386	0.31	0.0000000	0.00	0.0775060	0.37	0.0082633	11.49	0.0035854	663.07	6.41766	0.36	0.0294955	6.33	11.1144	47.71	892.145	0.58	0.0000000	0.00	0.0038955	9.66
18D00224	2.7 %	✓	3.44184	0.30	0.0000000	0.00	0.0165271	4.53	0.0000000	0.00	61.1437	4.53	0.643279	0.30	0.0000000	0.00	0.0877416	0.34	0.0110059	10.64	0.0000000	0.00	7.26518	0.32	0.0392848	4.62	14.3063	41.26	1002.642	0.58	0.0000000	0.00	0.0044100	9.66
18D00225	3.0 %	✓	6.65021	0.28	0.0000000	0.00	0.0386606	1.99	0.0000081	118.71	143.0284	1.98	1.242925	0.28	0.0000000	0.00	0.1470039	0.22	0.0257451	9.83	0.0210384	118.71	12.17222	0.21	0.0918957	2.19	23.7437	46.60	1937.274	0.57	0.0000000	0.00	0.0073885	9.65
18D00227	3.4 %	✓	3.53505	0.30	0.0000000	0.00	0.0206540	3.50	0.0000000	######	76.4112	3.50	0.660700	0.30	0.0000000	0.00	0.0792965	0.34	0.0137540	10.25	0.0000464	######	6.56591	0.33	0.0490942	3.62	13.0044	46.58	1029.795	0.58	0.0000000	0.00	0.0039855	9.66
18D00228	3.9 %	✓	7.35115	0.28	0.0000000	0.00	0.0529792	1.58	0.0000031	299.53	196.0015	1.57	1.373930	0.28	0.0000000	0.00	0.1495014	0.22	0.0352803	9.76	0.0079924	299.54	12.37901	0.20	0.1259310	1.82	23.6571	51.58	2141.464	0.57	0.0000000	0.00	0.0075141	9.65
18D00230	4.5 %	✓	4.93291	0.29	0.0000000	0.00	0.0394695	2.05	0.0000000	0.00	146.0211	2.05	0.921961	0.29	0.0000000	0.00	0.1003926	0.30	0.0262838	9.85	0.0000000	0.00	8.31271	0.28	0.0938185	2.24	14.7590	56.16	1437.006	0.57	0.0000000	0.00	0.0050458	9.65
18D00231	5.2 %	✓	4.41883	0.30	0.0000000	0.00	0.0412135	1.96	0.0000038	249.35	152.4733	1.95	0.825880	0.30	0.0000000	0.00	0.0875531	0.34	0.0274452	9.83	0.0098791	249.35	7.24957	0.33	0.0979641	2.15	9.7188	76.87	1287.250	0.57	0.0000000	0.00	0.0044005	9.66
18D00233	6.0 %	✓	6.16965	0.29	0.0000000	0.00	0.0593767	1.47	0.0000000	0.00	219.6697	1.46	1.153108	0.29	0.0000000	0.00	0.1096886	0.28	0.0395406	9.74	0.0000000	0.00	9.08244	0.26	0.1411378	1.72	18.8093	54.79	1797.282	0.57	0.0000000	0.00	0.0055130	9.65
18D00234	6.9 %	✓	7.56925	0.28	0.0000000	0.00	0.0618989	1.44	0.0000063	152.43	229.0008	1.43	1.414692	0.28	0.0000000	0.00	0.1110502	0.26	0.0412201	9.74	0.0163769	152.43	9.19518	0.24	0.1471330	1.70	21.0731	59.60	2204.998	0.57	0.0000000	0.00	0.0055815	9.65
18D00236	7.9 %	✓	11.40726	0.28	0.0000000	0.00	0.0658998	1.33	0.0000000	0.00	243.8026	1.32	2.132017	0.28	0.0000000	0.00	0.1197049	0.26	0.0438845	9.72	0.0000000	0.00	9.91180	0.24	0.1566432	1.61	37.8186	49.72	3323.050	0.56	0.0000000	0.00	0.0060165	9.65
18D00237	9.0 %		21.72159	0.27	0.0000000	0.00	0.0727243	1.26	0.0000247	42.98	269.0502	1.24	4.059766	0.27	0.0000000	0.00	0.1467452	0.22	0.0484290	9.71	0.0641568	42.99	12.15080	0.20	0.1728647	1.55	100.6503	35.44	6327.718	0.56	0.0000000	0.00	0.0073755	9.65
18D00239	10.3 %		45.97238	0.27	0.0000000	0.00	0.1290719	0.92	0.0000699	20.51	477.5137	0.90	8.592237	0.27	0.0000000	0.00	0.2660121	0.15	0.0859525	9.67	0.1818325	20.53	22.02634	0.12	0.3068025	1.29	349.0030	21.59	13392.213	0.56	0.0000000	0.00	0.0133700	9.65
18D00240	11.6 %		55.23960	0.27	0.0000000	0.00	0.1527014	0.86	0.0000975	16.68	564.9331	0.84	10.324281	0.27	0.0000000	0.00	0.2935965	0.15	0.1016880	9.67	0.2534731	16.70	24.31038	0.12	0.3629695	1.25	422.9759	21.40	16091.847	0.56	0.0000000	0.00	0.0147564	9.65
18D00242	12.5 %		29.37172	0.27	0.0000000	0.00	0.1183696	0.93	0.0000268	43.38	437.9195	0.92	5.489574	0.27	0.0000000	0.00	0.1852623	0.19	0.0788255	9.67	0.0696437	43.39	15.34009	0.17	0.2813633	1.30	157.7996	30.53	8556.274	0.56	0.0000000	0.00	0.0093114	9.65
18D00243	13.4 %		16.45639	0.28	0.0000000	0.00	0.0837322	1.12	0.0000018	572.95	309.7750	1.11	3.075698	0.28	0.0000000	0.00	0.1148914	0.27	0.0557595	9.69	0.0046013	572.95	9.51324	0.25	0.1990305	1.44	72.1161	37.53	4793.910	0.56	0.0000000	0.00	0.0057745	9.65
18D00245	14.6 %		8.54726	0.28	0.0000000	0.00	0.0469181	1.78	0.0000000	0.00	173.5780	1.77	1.597484	0.28	0.0000000	0.00	0.0632109	0.44	0.0312440	9.79	0.0000000	0.00	5.23399	0.43	0.1115238	2.00	29.9628	47.20	2489.904	0.57	0.0000000	0.00	0.0031770	9.66
18D00246	16.0 %		18.39069	0.28	0.0000000	0.00	0.1215113	0.94	0.0000006	######	449.5425	0.93	3.437220	0.28	0.0000000	0.00	0.1450171	0.22	0.0809177	9.67	0.0015374	######	12.00771	0.21	0.2888311	1.31	90.8747	33.27	5357.392	0.56	0.0000000	0.00	0.0072887	9.65
18D00248	17.6 %		11.22592	0.28	0.0000000	0.00	0.1001491	1.01	0.0000000	0.00	370.5108	1.00	2.098124	0.28	0.0000000	0.00	0.1031287	0.29	0.0666919	9.68	0.0000000	0.00	8.53926	0.28	0.2380532	1.36	54.3011	34.10	3270.222	0.57	0.0000000	0.00	0.0051833	9.65
		Σ	304.33597	0.08	0.0000000	0.00	1.3418508	0.32	0.0002739	15.19	4964.3021	0.31	56.880393	0.08	0.0000000	0.00	3.4284074	0.05	0.8935744	2.47	0.7121598	15.20	283.87906	0.04	3.1895641	0.39	1638.9800	9.04	88656.112	0.17	0.0000000	0.00	0.1723146	2.36
		Σ							305.67810	0.08	4964.3021	0.31									61.914535	0.19			287.06862	0.04							90295.264	0.23

**Additional Parameters**			40Ar/39Ar	1σ	37Ar/39Ar	1σ	36Ar/39Ar	1σ	Time (days)	37Ar (decay)	39Ar (decay)	40Ar (moles)
18D00216	1.8 %	✓	128.016975	0.123029	3.338269	0.078630	0.432435	0.001257	236.690	108.312391	1.00167504	2.280E-10
18D00218	1.9 %	✓	133.618803	0.218476	4.055443	0.178400	0.453497	0.001454	236.704	108.342109	1.00167514	1.056E-10
18D00219	2.0 %	✓	137.385280	0.195242	5.774048	0.144098	0.466864	0.001453	236.711	108.356971	1.00167519	1.256E-10
18D00221	2.2 %	✓	139.422814	0.261461	7.036353	0.208326	0.474907	0.001587	236.725	108.386701	1.00167529	9.262E-11
18D00222	2.4 %	✓	140.102714	0.529754	7.120561	0.446758	0.476945	0.002254	236.732	108.401569	1.00167534	4.336E-11
18D00224	2.7 %	✓	139.223391	0.470753	8.370730	0.380203	0.473459	0.002087	236.746	108.431311	1.00167544	4.881E-11
18D00225	3.0 %	✓	159.899385	0.336994	11.662345	0.232573	0.545403	0.001899	236.753	108.446185	1.00167548	9.413E-11
18D00227	3.4 %	✓	157.642098	0.536785	11.551195	0.405663	0.537521	0.002382	236.767	108.475940	1.00167558	5.005E-11
18D00228	3.9 %	✓	173.141787	0.352331	15.673921	0.247582	0.592096	0.002031	236.774	108.490820	1.00167563	1.039E-10
18D00230	4.5 %	✓	172.695462	0.497979	17.369956	0.358925	0.591490	0.002372	236.788	108.522075	1.00167573	6.968E-11
18D00231	5.2 %	✓	176.518079	0.589000	20.751615	0.409716	0.607013	0.002645	236.795	108.536962	1.00167578	6.225E-11
18D00233	6.0 %	✓	196.897254	0.517882	23.816115	0.352641	0.675338	0.002588	236.809	108.566741	1.00167588	8.717E-11
18D00234	6.9 %	✓	238.278976	0.578425	24.512218	0.355573	0.816838	0.003002	236.816	108.581634	1.00167593	1.069E-10
18D00236	7.9 %	✓	333.802633	0.806421	24.214519	0.323899	1.139517	0.004162	236.830	108.611426	1.00167603	1.613E-10
18D00237	9.0 %		521.628624	1.032590	21.831996	0.275133	1.768496	0.005963	236.837	108.626325	1.00167608	3.086E-10
18D00239	10.3 %		615.284142	0.746419	21.381391	0.193978	2.064265	0.006147	236.851	108.656129	1.00167618	6.596E-10
18D00240	11.6 %		669.339058	0.751864	22.896489	0.194107	2.245029	0.006600	236.858	108.671034	1.00167623	7.927E-10
18D00242	12.5 %		557.827892	0.915436	28.033209	0.261435	1.887795	0.005997	236.872	108.700850	1.00167632	4.183E-10
18D00243	13.4 %		501.018916	1.229651	31.895220	0.361595	1.703013	0.006259	236.878	108.715761	1.00167637	2.336E-10
18D00245	14.6 %		471.398771	2.007979	32.471699	0.591873	1.607737	0.008154	236.892	108.745589	1.00167647	1.210E-10
18D00246	16.0 %		443.073718	0.883848	36.558452	0.346304	1.505481	0.005093	236.899	108.760507	1.00167652	2.615E-10
18D00248	17.6 %		378.763704	1.024184	42.212320	0.436190	1.290379	0.004966	236.914	108.791840	1.00167662	1.596E-10

Procedure Blanks		36Ar ± 1σ (SE) [fA]	37Ar ± 1σ (SE) [fA]	38Ar ± 1σ (SE) [fA]	39Ar ± 1σ (SE) [fA]	40Ar ± 1σ (SE) [fA]
18D00216	1.8 %	0.0413340 ± 0.0034008	0.0077471 ± 0.0174366	0.0142109 ± 0.0173376	0.0129604 ± 0.0158767	12.6874212 ± 1.0879906
18D00218	1.9 %	0.0602904 ± 0.0034008	0.0205587 ± 0.0174366	0.0188333 ± 0.0173376	0.0089523 ± 0.0158767	18.6387533 ± 1.0879906
18D00219	2.0 %	0.0674947 ± 0.0034008	0.0234277 ± 0.0174366	0.0199093 ± 0.0173376	0.0129807 ± 0.0158767	20.8751175 ± 1.0879906
18D00221	2.2 %	0.0779347 ± 0.0034008	0.0241964 ± 0.0174366	0.0203385 ± 0.0173376	0.0107659 ± 0.0158767	24.0710355 ± 1.0879906
18D00222	2.4 %	0.0813897 ± 0.0034008	0.0228376 ± 0.0174366	0.0199555 ± 0.0173376	0.0058183 ± 0.0158767	25.1064578 ± 1.0879906
18D00224	2.7 %	0.0852879 ± 0.0034008	0.0182369 ± 0.0174366	0.0185645 ± 0.0173376	0.0088635 ± 0.0158767	26.2297785 ± 1.0879906
18D00225	3.0 %	0.0859260 ± 0.0034008	0.0155360 ± 0.0174366	0.0177494 ± 0.0173376	0.0175785 ± 0.0158767	26.3838477 ± 1.0879906
18D00227	3.4 %	0.0850372 ± 0.0034008	0.0104343 ± 0.0174366	0.0162732 ± 0.0173376	0.0355540 ± 0.0158767	26.0301094 ± 1.0879906
18D00228	3.9 %	0.0836810 ± 0.0034008	0.0083740 ± 0.0174366	0.0157339 ± 0.0173376	0.0440716 ± 0.0158767	25.5787751 ± 1.0879906
18D00230	4.5 %	0.0792918 ± 0.0034008	0.0057712 ± 0.0174366	0.0152702 ± 0.0173376	0.0592718 ± 0.0158767	24.1779923 ± 1.0879906
18D00231	5.2 %	0.0766223 ± 0.0034008	0.0054973 ± 0.0174366	0.0154206 ± 0.0173376	0.0647413 ± 0.0158767	23.3446198 ± 1.0879906
18D00233	6.0 %	0.0705020 ± 0.0034008	0.0069058 ± 0.0174366	0.0164747 ± 0.0173376	0.0714374 ± 0.0158767	21.4622352 ± 1.0879906
18D00234	6.9 %	0.0671721 ± 0.0034008	0.0085208 ± 0.0174366	0.0173561 ± 0.0173376	0.0724831 ± 0.0158767	20.4499779 ± 1.0879906
18D00236	7.9 %	0.0602451 ± 0.0034008	0.0131529 ± 0.0174366	0.0196831 ± 0.0173376	0.0698628 ± 0.0158767	18.3630986 ± 1.0879906
18D00237	9.0 %	0.0567445 ± 0.0034008	0.0159020 ± 0.0174366	0.0210355 ± 0.0173376	0.0662923 ± 0.0158767	17.3155337 ± 1.0879906
18D00239	10.3 %	0.0498801 ± 0.0034008	0.0213450 ± 0.0174366	0.0237960 ± 0.0173376	0.0552146 ± 0.0158767	15.2676747 ± 1.0879906
18D00240	11.6 %	0.0465886 ± 0.0034008	0.0235703 ± 0.0174366	0.0250400 ± 0.0173376	0.0480792 ± 0.0158767	14.2847402 ± 1.0879906
18D00242	12.5 %	0.0404366 ± 0.0034008	0.0256064 ± 0.0174366	0.0267565 ± 0.0173376	0.0318904 ± 0.0158767	12.4321381 ± 1.0879906
18D00243	13.4 %	0.0376241 ± 0.0034008	0.0247480 ± 0.0174366	0.0269939 ± 0.0173376	0.0234851 ± 0.0158767	11.5701326 ± 1.0879906
18D00245	14.6 %	0.0326147 ± 0.0034008	0.0173541 ± 0.0174366	0.0255506 ± 0.0173376	0.0080186 ± 0.0158767	9.9817455 ± 1.0879906
18D00246	16.0 %	0.0304414 ± 0.0034008	0.0099489 ± 0.0174366	0.0235638 ± 0.0173376	0.0018820 ± 0.0158767	9.2533284 ± 1.0879906
18D00248	17.6 %	0.0266267 ± 0.0034008	0.0163374 ± 0.0174366	0.0157003 ± 0.0173376	0.0045935 ± 0.0158767	7.8471163 ± 1.0879906

Intercept Values		36Ar ± 1σ (SE) [fA]	r2	Regression (type,n)	37Ar ± 1σ (SE) [fA]	r2	Regression (type,n)	38Ar ± 1σ (SE) [fA]	r2	Regression (type,n)	39Ar ± 1σ (SE) [fA]	r2	Regression (type,n)	40Ar ± 1σ (SE) [fA]	r2	Regression (type,n)
18D00216	1.8 %	15.294597 ± 0.006720	0.9963	EXP 150 of 150	1.1147126 ± 0.0182536	0.0777	EXP 150 of 150	3.4905881 ± 0.0156279	0.5578	EXP 150 of 150	36.8269799 ± 0.0181593	0.9898	EXP 150 of 150	4762.6149 ± 0.1393	0.9999	EXP 150 of 150
18D00218	1.9 %	7.160711 ± 0.003825	0.9944	EXP 150 of 150	0.5845545 ± 0.0196474	0.0060	EXP 149 of 150	1.6156978 ± 0.0161190	0.2034	EXP 150 of 150	16.3321476 ± 0.0166870	0.9416	EXP 150 of 150	2219.3096 ± 0.0797	0.9999	EXP 150 of 150
18D00219	2.0 %	8.521443 ± 0.004965	0.9934	EXP 150 of 150	0.9728453 ± 0.0162637	0.0800	EXP 149 of 150	1.9082751 ± 0.0165068	0.2116	EXP 150 of 150	18.8860912 ± 0.0158033	0.9619	EXP 150 of 150	2637.7739 ± 0.0754	0.9999	EXP 150 of 150
18D00221	2.2 %	6.326191 ± 0.003669	0.9935	EXP 150 of 150	0.8576800 ± 0.0183778	0.0626	EXP 150 of 150	1.4098099 ± 0.0155417	0.1518	EXP 150 of 150	13.7208512 ± 0.0163603	0.9190	EXP 150 of 150	1953.6467 ± 0.0755	0.9999	EXP 150 of 150
18D00222	2.4 %	3.004592 ± 0.002625	0.9837	EXP 150 of 150	0.3928380 ± 0.0191208	0.0240	EXP 150 of 150	0.6735424 ± 0.0156796	0.0412	EXP 150 of 150	6.3909630 ± 0.0159829	0.5996	EXP 149 of 150	928.3697 ± 0.0440	0.9997	EXP 150 of 150
18D00224	2.7 %	3.372996 ± 0.002832	0.9857	EXP 150 of 150	0.5352471 ± 0.0176084	0.0143	EXP 150 of 150	0.7357324 ± 0.0173812	0.0074	EXP 150 of 150	7.2562608 ± 0.0162799	0.6747	EXP 150 of 150	1043.1822 ± 0.0506	0.9997	EXP 150 of 150
18D00225	3.0 %	6.444740 ± 0.003871	0.9929	EXP 150 of 150	1.2790058 ± 0.0165762	0.2025	EXP 150 of 150	1.4367582 ± 0.0168976	0.1669	EXP 150 of 150	12.1858804 ± 0.0171625	0.8754	EXP 150 of 150	1987.4089 ± 0.0687	0.9999	EXP 150 of 150
18D00227	3.4 %	3.465280 ± 0.002993	0.9852	EXP 150 of 150	0.6809696 ± 0.0160465	0.0283	EXP 149 of 150	0.7607820 ± 0.0158326	0.0435	EXP 150 of 150	6.5988768 ± 0.0134685	0.6483	EXP 149 of 150	1068.8334 ± 0.0515	0.9997	EXP 150 of 150
18D00228	3.9 %	7.122451 ± 0.003929	0.9942	EXP 150 of 150	1.7648949 ± 0.0177989	0.1654	EXP 150 of 150	1.5631326 ± 0.0150901	0.1879	EXP 150 of 150	12.4513172 ± 0.0166869	0.8884	EXP 150 of 150	2190.7074 ± 0.0736	0.9999	EXP 150 of 150
18D00230	4.5 %	4.806304 ± 0.003267	0.9910	EXP 150 of 150	1.3149331 ± 0.0185245	0.0917	EXP 150 of 150	1.0430316 ± 0.0183675	0.0679	EXP 150 of 150	8.4001233 ± 0.0160157	0.7108	EXP 150 of 150	1475.9479 ± 0.0666	0.9998	EXP 150 of 150
18D00231	5.2 %	4.316587 ± 0.003253	0.9888	EXP 150 of 150	1.3733755 ± 0.0180463	0.1869	EXP 150 of 150	0.9544621 ± 0.0166339	0.0871	EXP 150 of 150	7.3548723 ± 0.0166948	0.5892	EXP 150 of 150	1320.3179 ± 0.0589	0.9998	EXP 150 of 150
18D00233	6.0 %	5.992155 ± 0.004050	0.9910	EXP 150 of 150	1.9791047 ± 0.0185672	0.3047	EXP 150 of 150	1.2949209 ± 0.0163481	0.1233	EXP 150 of 150	9.2229478 ± 0.0161312	0.7599	EXP 150 of 150	1837.5590 ± 0.0659	0.9999	EXP 150 of 150
18D00234	6.9 %	7.321759 ± 0.003953	0.9944	EXP 150 of 150	2.0615665 ± 0.0191549	0.2904	EXP 150 of 150	1.5811852 ± 0.0164744	0.2497	EXP 150 of 150	9.3418004 ± 0.0139902	0.7615	EXP 149 of 150	2246.5262 ± 0.0775	0.9999	EXP 150 of 150
18D00236	7.9 %	10.967253 ± 0.004644	0.9966	EXP 150 of 150	2.1901335 ± 0.0173989	0.3374	EXP 149 of 150	2.2798809 ± 0.0194273	0.2407	EXP 150 of 150	10.0596422 ± 0.0166073	0.5558	EXP 150 of 150	3379.2373 ± 0.1105	0.9999	EXP 150 of 150
18D00237	9.0 %	20.775625 ± 0.007205	0.9977	EXP 150 of 150	2.4152174 ± 0.0180756	0.2829	EXP 150 of 150	4.2869109 ± 0.0162863	0.6557	EXP 150 of 150	12.2936659 ± 0.0162325	0.0001	EXP 150 of 150	6445.6908 ± 0.1592	1.0000	EXP 150 of 150
18D00239	10.3 %	43.876475 ± 0.010971	0.9988	EXP 150 of 150	4.2922533 ± 0.0174101	0.5559	EXP 150 of 150	9.0373755 ± 0.0174514	0.8755	EXP 150 of 150	22.2138594 ± 0.0158336	0.2150	EXP 150 of 150	13756.4969 ± 0.3023	1.0000	EXP 150 of 150
18D00240	11.6 %	52.705593 ± 0.011474	0.9991	EXP 150 of 150	5.0790276 ± 0.0167074	0.7487	EXP 150 of 150	10.8628640 ± 0.0189802	0.8981	EXP 150 of 150	24.5286446 ± 0.0153914	0.7840	EXP 150 of 150	16529.1221 ± 0.4001	1.0000	EXP 150 of 150
18D00242	12.5 %	28.075331 ± 0.007197	0.9988	EXP 150 of 150	3.9286923 ± 0.0161264	0.5742	EXP 149 of 150	5.7783045 ± 0.0159188	0.7654	EXP 150 of 150	15.5312855 ± 0.0168850	0.0611	EXP 150 of 150	8726.5155 ± 0.1954	1.0000	EXP 150 of 150
18D00243	13.4 %	15.761555 ± 0.006211	0.9970	EXP 150 of 150	2.7720558 ± 0.0166551	0.4246	EXP 150 of 150	3.2378851 ± 0.0161236	0.5409	EXP 150 of 150	9.6598677 ± 0.0161754	0.1404	EXP 150 of 150	4877.6016 ± 0.1088	1.0000	EXP 150 of 150
18D00245	14.6 %	8.202709 ± 0.004155	0.9950	EXP 150 of 150	1.5493648 ± 0.0186821	0.1609	EXP 150 of 150	1.6927285 ± 0.0160106	0.2600	EXP 150 of 150	5.3117657 ± 0.0155128	0.1252	EXP 150 of 150	2529.8512 ± 0.0805	0.9999	EXP 150 of 150
18D00246	16.0 %	17.629142 ± 0.006473	0.9974	EXP 150 of 150	4.0470758 ± 0.0177259	0.5415	EXP 150 of 150	3.6430984 ± 0.0153671	0.6065	EXP 150 of 150	12.2023404 ± 0.0163886	0.1893	EXP 150 of 150	5457.5275 ± 0.1332	0.9999	EXP 150 of 150
18D00248	17.6 %	10.793797 ± 0.004490	0.9966	EXP 150 of 150	3.3591549 ± 0.0164201	0.5410	EXP 150 of 150	2.2498774 ± 0.0165550	0.2786	EXP 150 of 150	8.7041356 ± 0.0161500	0.1923	EXP 150 of 150	3332.3752 ± 0.1051	0.9999	EXP 150 of 150

Project Info		Analyst	Irradiation	X-pos	Y-pos	Z/H-pos	Project	Experiment	Nmb
18D00216	1.8 %	Dan Miggins	17-OSU-05	0.00	0.00	26.49	Arctic\O-Connor (16-22)	18D00212	01
18D00218	1.9 %	Dan Miggins	17-OSU-05	0.00	0.00	26.49	Arctic\O-Connor (16-22)	18D00212	01
18D00219	2.0 %	Dan Miggins	17-OSU-05	0.00	0.00	26.49	Arctic\O-Connor (16-22)	18D00212	01
18D00221	2.2 %	Dan Miggins	17-OSU-05	0.00	0.00	26.49	Arctic\O-Connor (16-22)	18D00212	01
18D00222	2.4 %	Dan Miggins	17-OSU-05	0.00	0.00	26.49	Arctic\O-Connor (16-22)	18D00212	01
18D00224	2.7 %	Dan Miggins	17-OSU-05	0.00	0.00	26.49	Arctic\O-Connor (16-22)	18D00212	01
18D00225	3.0 %	Dan Miggins	17-OSU-05	0.00	0.00	26.49	Arctic\O-Connor (16-22)	18D00212	01
18D00227	3.4 %	Dan Miggins	17-OSU-05	0.00	0.00	26.49	Arctic\O-Connor (16-22)	18D00212	01
18D00228	3.9 %	Dan Miggins	17-OSU-05	0.00	0.00	26.49	Arctic\O-Connor (16-22)	18D00212	01
18D00230	4.5 %	Dan Miggins	17-OSU-05	0.00	0.00	26.49	Arctic\O-Connor (16-22)	18D00212	01
18D00231	5.2 %	Dan Miggins	17-OSU-05	0.00	0.00	26.49	Arctic\O-Connor (16-22)	18D00212	01
18D00233	6.0 %	Dan Miggins	17-OSU-05	0.00	0.00	26.49	Arctic\O-Connor (16-22)	18D00212	01
18D00234	6.9 %	Dan Miggins	17-OSU-05	0.00	0.00	26.49	Arctic\O-Connor (16-22)	18D00212	01
18D00236	7.9 %	Dan Miggins	17-OSU-05	0.00	0.00	26.49	Arctic\O-Connor (16-22)	18D00212	01
18D00237	9.0 %	Dan Miggins	17-OSU-05	0.00	0.00	26.49	Arctic\O-Connor (16-22)	18D00212	01
18D00239	10.3 %	Dan Miggins	17-OSU-05	0.00	0.00	26.49	Arctic\O-Connor (16-22)	18D00212	01
18D00240	11.6 %	Dan Miggins	17-OSU-05	0.00	0.00	26.49	Arctic\O-Connor (16-22)	18D00212	01
18D00242	12.5 %	Dan Miggins	17-OSU-05	0.00	0.00	26.49	Arctic\O-Connor (16-22)	18D00212	01
18D00243	13.4 %	Dan Miggins	17-OSU-05	0.00	0.00	26.49	Arctic\O-Connor (16-22)	18D00212	01
18D00245	14.6 %	Dan Miggins	17-OSU-05	0.00	0.00	26.49	Arctic\O-Connor (16-22)	18D00212	01
18D00246	16.0 %	Dan Miggins	17-OSU-05	0.00	0.00	26.49	Arctic\O-Connor (16-22)	18D00212	01
18D00248	17.6 %	Dan Miggins	17-OSU-05	0.00	0.00	26.49	Arctic\O-Connor (16-22)	18D00212	01

Sample Parameters		Sample	Material	Location	Standard Name	Standard (in Ma)	%1σ	Standard Reference	Standard 40Ar/39Ar	%1σ	J	%1σ	Air 40Ar/36Ar	%1σ	MDF (lin)	%1σ	Volume Ratio	Sensitivity (mol/volt)	Day	Month	Year	Hour	Min	Resist
18D00216	1.8 %	PS59-229-13	Groundmass	Gakkel Ridge	FCT-NM (5B18-17)	28.201	0.082	Kuiper et al (2008)	9.77942	0.081	0.00160719	0.081	302.958	0.131	0.9938377	0.067	1	4.8E-14	3	JAN	2018	4	24	1
18D00218	1.9 %	PS59-229-13	Groundmass	Gakkel Ridge	FCT-NM (5B18-17)	28.201	0.082	Kuiper et al (2008)	9.77942	0.081	0.00160719	0.081	302.958	0.131	0.9938377	0.067	1	4.8E-14	3	JAN	2018	4	44	1
18D00219	2.0 %	PS59-229-13	Groundmass	Gakkel Ridge	FCT-NM (5B18-17)	28.201	0.082	Kuiper et al (2008)	9.77942	0.081	0.00160719	0.081	302.958	0.131	0.9938377	0.067	1	4.8E-14	3	JAN	2018	4	54	1
18D00221	2.2 %	PS59-229-13	Groundmass	Gakkel Ridge	FCT-NM (5B18-17)	28.201	0.082	Kuiper et al (2008)	9.77942	0.081	0.00160719	0.081	302.958	0.131	0.9938377	0.067	1	4.8E-14	3	JAN	2018	5	14	1
18D00222	2.4 %	PS59-229-13	Groundmass	Gakkel Ridge	FCT-NM (5B18-17)	28.201	0.082	Kuiper et al (2008)	9.77942	0.081	0.00160719	0.081	302.958	0.131	0.9938377	0.067	1	4.8E-14	3	JAN	2018	5	24	1
18D00224	2.7 %	PS59-229-13	Groundmass	Gakkel Ridge	FCT-NM (5B18-17)	28.201	0.082	Kuiper et al (2008)	9.77942	0.081	0.00160719	0.081	302.958	0.131	0.9938377	0.067	1	4.8E-14	3	JAN	2018	5	44	1
18D00225	3.0 %	PS59-229-13	Groundmass	Gakkel Ridge	FCT-NM (5B18-17)	28.201	0.082	Kuiper et al (2008)	9.77942	0.081	0.00160719	0.081	302.958	0.131	0.9938377	0.067	1	4.8E-14	3	JAN	2018	5	54	1
18D00227	3.4 %	PS59-229-13	Groundmass	Gakkel Ridge	FCT-NM (5B18-17)	28.201	0.082	Kuiper et al (2008)	9.77942	0.081	0.00160719	0.081	302.958	0.131	0.9938377	0.067	1	4.8E-14	3	JAN	2018	6	14	1
18D00228	3.9 %	PS59-229-13	Groundmass	Gakkel Ridge	FCT-NM (5B18-17)	28.201	0.082	Kuiper et al (2008)	9.77942	0.081	0.00160719	0.081	302.958	0.131	0.9938377	0.067	1	4.8E-14	3	JAN	2018	6	24	1
18D00230	4.5 %	PS59-229-13	Groundmass	Gakkel Ridge	FCT-NM (5B18-17)	28.201	0.082	Kuiper et al (2008)	9.77942	0.081	0.00160719	0.081	302.958	0.131	0.9938377	0.067	1	4.8E-14	3	JAN	2018	6	45	1
18D00231	5.2 %	PS59-229-13	Groundmass	Gakkel Ridge	FCT-NM (5B18-17)	28.201	0.082	Kuiper et al (2008)	9.77942	0.081	0.00160719	0.081	302.958	0.131	0.9938377	0.067	1	4.8E-14	3	JAN	2018	6	55	1
18D00233	6.0 %	PS59-229-13	Groundmass	Gakkel Ridge	FCT-NM (5B18-17)	28.201	0.082	Kuiper et al (2008)	9.77942	0.081	0.00160719	0.081	302.958	0.131	0.9938377	0.067	1	4.8E-14	3	JAN	2018	7	15	1
18D00234	6.9 %	PS59-229-13	Groundmass	Gakkel Ridge	FCT-NM (5B18-17)	28.201	0.082	Kuiper et al (2008)	9.77942	0.081	0.00160719	0.081	302.958	0.131	0.9938377	0.067	1	4.8E-14	3	JAN	2018	7	25	1
18D00236	7.9 %	PS59-229-13	Groundmass	Gakkel Ridge	FCT-NM (5B18-17)	28.201	0.082	Kuiper et al (2008)	9.77942	0.081	0.00160719	0.081	302.958	0.131	0.9938377	0.067	1	4.8E-14	3	JAN	2018	7	45	1
18D00237	9.0 %	PS59-229-13	Groundmass	Gakkel Ridge	FCT-NM (5B18-17)	28.201	0.082	Kuiper et al (2008)	9.77942	0.081	0.00160719	0.081	302.958	0.131	0.9938377	0.067	1	4.8E-14	3	JAN	2018	7	55	1
18D00239	10.3 %	PS59-229-13	Groundmass	Gakkel Ridge	FCT-NM (5B18-17)	28.201	0.082	Kuiper et al (2008)	9.77942	0.081	0.00160719	0.081	302.958	0.131	0.9938377	0.067	1	4.8E-14	3	JAN	2018	8	15	1
18D00240	11.6 %	PS59-229-13	Groundmass	Gakkel Ridge	FCT-NM (5B18-17)	28.201	0.082	Kuiper et al (2008)	9.77942	0.081	0.00160719	0.081	302.958	0.131	0.9938377	0.067	1	4.8E-14	3	JAN	2018	8	25	1
18D00242	12.5 %	PS59-229-13	Groundmass	Gakkel Ridge	FCT-NM (5B18-17)	28.201	0.082	Kuiper et al (2008)	9.77942	0.081	0.00160719	0.081	302.958	0.131	0.9938377	0.067	1	4.8E-14	3	JAN	2018	8	45	1
18D00243	13.4 %	PS59-229-13	Groundmass	Gakkel Ridge	FCT-NM (5B18-17)	28.201	0.082	Kuiper et al (2008)	9.77942	0.081	0.00160719	0.081	302.958	0.131	0.9938377	0.067	1	4.8E-14	3	JAN	2018	8	55	1
18D00245	14.6 %	PS59-229-13	Groundmass	Gakkel Ridge	FCT-NM (5B18-17)	28.201	0.082	Kuiper et al (2008)	9.77942	0.081	0.00160719	0.081	302.958	0.131	0.9938377	0.067	1	4.8E-14	3	JAN	2018	9	15	1
18D00246	16.0 %	PS59-229-13	Groundmass	Gakkel Ridge	FCT-NM (5B18-17)	28.201	0.082	Kuiper et al (2008)	9.77942	0.081	0.00160719	0.081	302.958	0.131	0.9938377	0.067	1	4.8E-14	3	JAN	2018	9	25	1
18D00248	17.6 %	PS59-229-13	Groundmass	Gakkel Ridge	FCT-NM (5B18-17)	28.201	0.082	Kuiper et al (2008)	9.77942	0.081	0.00160719	0.081	302.958	0.131	0.9938377	0.067	1	4.8E-14	3	JAN	2018	9	46	1

Irradiation Constants		40/36(a)	%1σ	40/36(c)	%1σ	38/36(a)	%1σ	38/36(c)	%1σ	39/37(ca)	%1σ	38/37(ca)	%1σ	36/37(ca)	%1σ	40/39(k)	%1σ	38/39(k)	%1σ	36/38(cl)	%1σ	K/Ca	%1σ	K/Cl	%1σ	Ca/Cl	%1σ
18D00216	1.8 %	291.31	0.492	0.018	35	0.1869	0	1.493	3	0.000643	0.92	0.00018	9.63	0.00027	0.17	0.000607	9.65	0.012077	0.09	0	0	0.43	0	0	0	0	0
18D00218	1.9 %	291.31	0.492	0.018	35	0.1869	0	1.493	3	0.000643	0.92	0.00018	9.63	0.00027	0.17	0.000607	9.65	0.012077	0.09	0	0	0.43	0	0	0	0	0
18D00219	2.0 %	291.31	0.492	0.018	35	0.1869	0	1.493	3	0.000643	0.92	0.00018	9.63	0.00027	0.17	0.000607	9.65	0.012077	0.09	0	0	0.43	0	0	0	0	0
18D00221	2.2 %	291.31	0.492	0.018	35	0.1869	0	1.493	3	0.000643	0.92	0.00018	9.63	0.00027	0.17	0.000607	9.65	0.012077	0.09	0	0	0.43	0	0	0	0	0
18D00222	2.4 %	291.31	0.492	0.018	35	0.1869	0	1.493	3	0.000643	0.92	0.00018	9.63	0.00027	0.17	0.000607	9.65	0.012077	0.09	0	0	0.43	0	0	0	0	0
18D00224	2.7 %	291.31	0.492	0.018	35	0.1869	0	1.493	3	0.000643	0.92	0.00018	9.63	0.00027	0.17	0.000607	9.65	0.012077	0.09	0	0	0.43	0	0	0	0	0
18D00225	3.0 %	291.31	0.492	0.018	35	0.1869	0	1.493	3	0.000643	0.92	0.00018	9.63	0.00027	0.17	0.000607	9.65	0.012077	0.09	0	0	0.43	0	0	0	0	0
18D00227	3.4 %	291.31	0.492	0.018	35	0.1869	0	1.493	3	0.000643	0.92	0.00018	9.63	0.00027	0.17	0.000607	9.65	0.012077	0.09	0	0	0.43	0	0	0	0	0
18D00228	3.9 %	291.31	0.492	0.018	35	0.1869	0	1.493	3	0.000643	0.92	0.00018	9.63	0.00027	0.17	0.000607	9.65	0.012077	0.09	0	0	0.43	0	0	0	0	0
18D00230	4.5 %	291.31	0.492	0.018	35	0.1869	0	1.493	3	0.000643	0.92	0.00018	9.63	0.00027	0.17	0.000607	9.65	0.012077	0.09	0	0	0.43	0	0	0	0	0
18D00231	5.2 %	291.31	0.492	0.018	35	0.1869	0	1.493	3	0.000643	0.92	0.00018	9.63	0.00027	0.17	0.000607	9.65	0.012077	0.09	0	0	0.43	0	0	0	0	0
18D00233	6.0 %	291.31	0.492	0.018	35	0.1869	0	1.493	3	0.000643	0.92	0.00018	9.63	0.00027	0.17	0.000607	9.65	0.012077	0.09	0	0	0.43	0	0	0	0	0
18D00234	6.9 %	291.31	0.492	0.018	35	0.1869	0	1.493	3	0.000643	0.92	0.00018	9.63	0.00027	0.17	0.000607	9.65	0.012077	0.09	0	0	0.43	0	0	0	0	0
18D00236	7.9 %	291.31	0.492	0.018	35	0.1869	0	1.493	3	0.000643	0.92	0.00018	9.63	0.00027	0.17	0.000607	9.65	0.012077	0.09	0	0	0.43	0	0	0	0	0
18D00237	9.0 %	291.31	0.492	0.018	35	0.1869	0	1.493	3	0.000643	0.92	0.00018	9.63	0.00027	0.17	0.000607	9.65	0.012077	0.09	0	0	0.43	0	0	0	0	0
18D00239	10.3 %	291.31	0.492	0.018	35	0.1869	0	1.493	3	0.000643	0.92	0.00018	9.63	0.00027	0.17	0.000607	9.65	0.012077	0.09	0	0	0.43	0	0	0	0	0
18D00240	11.6 %	291.31	0.492	0.018	35	0.1869	0	1.493	3	0.000643	0.92	0.00018	9.63	0.00027	0.17	0.000607	9.65	0.012077	0.09	0	0	0.43	0	0	0	0	0
18D00242	12.5 %	291.31	0.492	0.018	35	0.1869	0	1.493	3	0.000643	0.92	0.00018	9.63	0.00027	0.17	0.000607	9.65	0.012077	0.09	0	0	0.43	0	0	0	0	0
18D00243	13.4 %	291.31	0.492	0.018	35	0.1869	0	1.493	3	0.000643	0.92	0.00018	9.63	0.00027	0.17	0.000607	9.65	0.012077	0.09	0	0	0.43	0	0	0	0	0
18D00245	14.6 %	291.31	0.492	0.018	35	0.1869	0	1.493	3	0.000643	0.92	0.00018	9.63	0.00027	0.17	0.000607	9.65	0.012077	0.09	0	0	0.43	0	0	0	0	0
18D00246	16.0 %	291.31	0.492	0.018	35	0.1869	0	1.493	3	0.000643	0.92	0.00018	9.63	0.00027	0.17	0.000607	9.65	0.012077	0.09	0	0	0.43	0	0	0	0	0
18D00248	17.6 %	291.31	0.492	0.018	35	0.1869	0	1.493	3	0.000643	0.92	0.00018	9.63	0.00027	0.17	0.000607	9.65	0.012077	0.09	0	0	0.43	0	0	0	0	0

18D00212.AGE >>> PS59-229-13 >>> ARCTIC | O-CONNOR (16-22) PROJECT

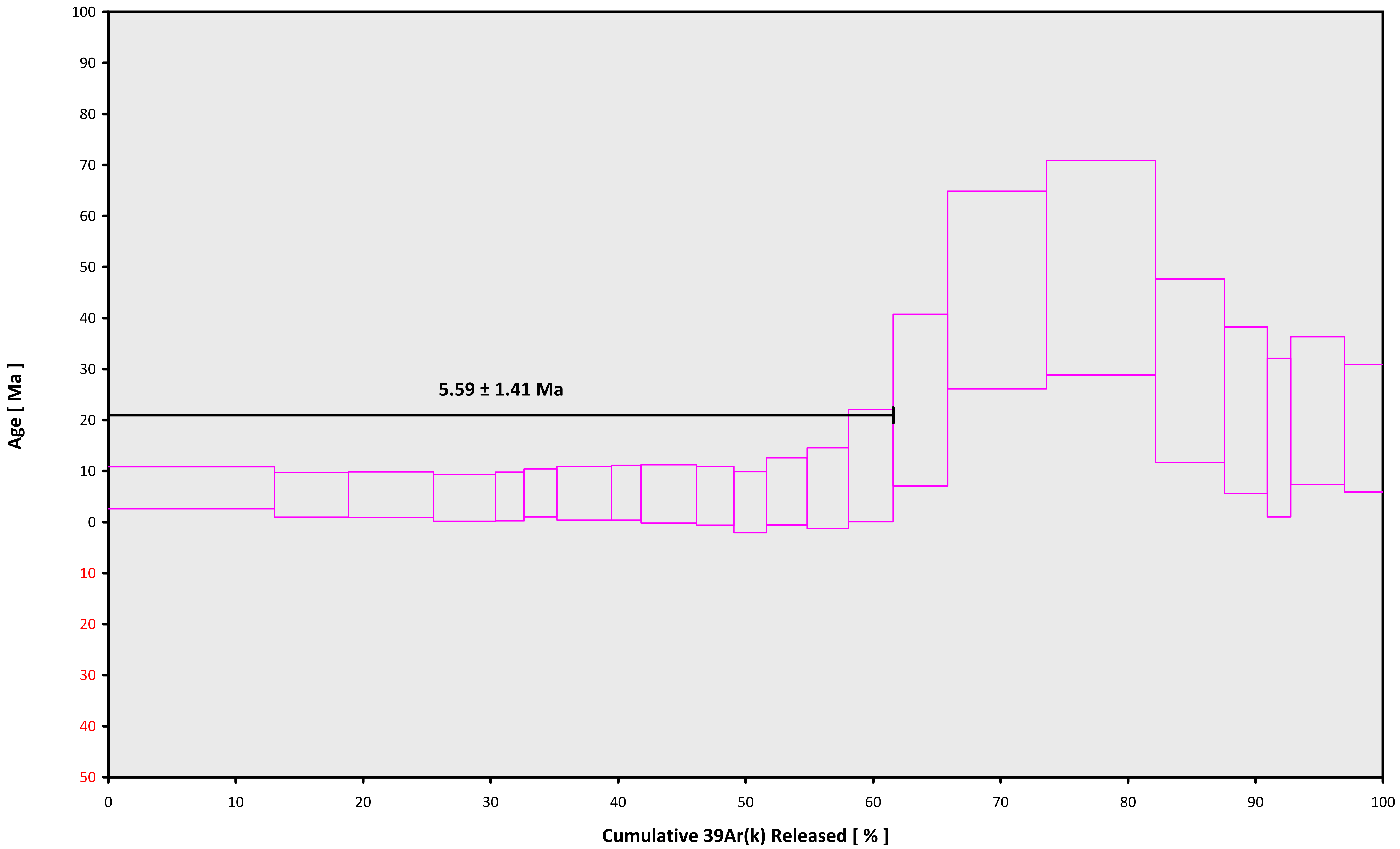

Ar-Ages in Ma

WEIGHTED PLATEAU
5.59 ± 1.41
TOTAL FUSION
16.70 ± 3.01
NORMAL ISOCHRON
3.37 ± 3.29
INVERSE ISOCHRON
3.37 ± 2.13

MSWD (PROBABILITY)
0.15 (100%)

Sample Info

Groundmass
Gakkel Ridge
Dan Miggins

IRR = 17-OSU-05 (5B18-17)
J = 0.00160719 ± 0.00000130

18D00212.AGE >>> PS59-229-13 >>> ARCTIC | O-CONNOR (16-22) PROJECT

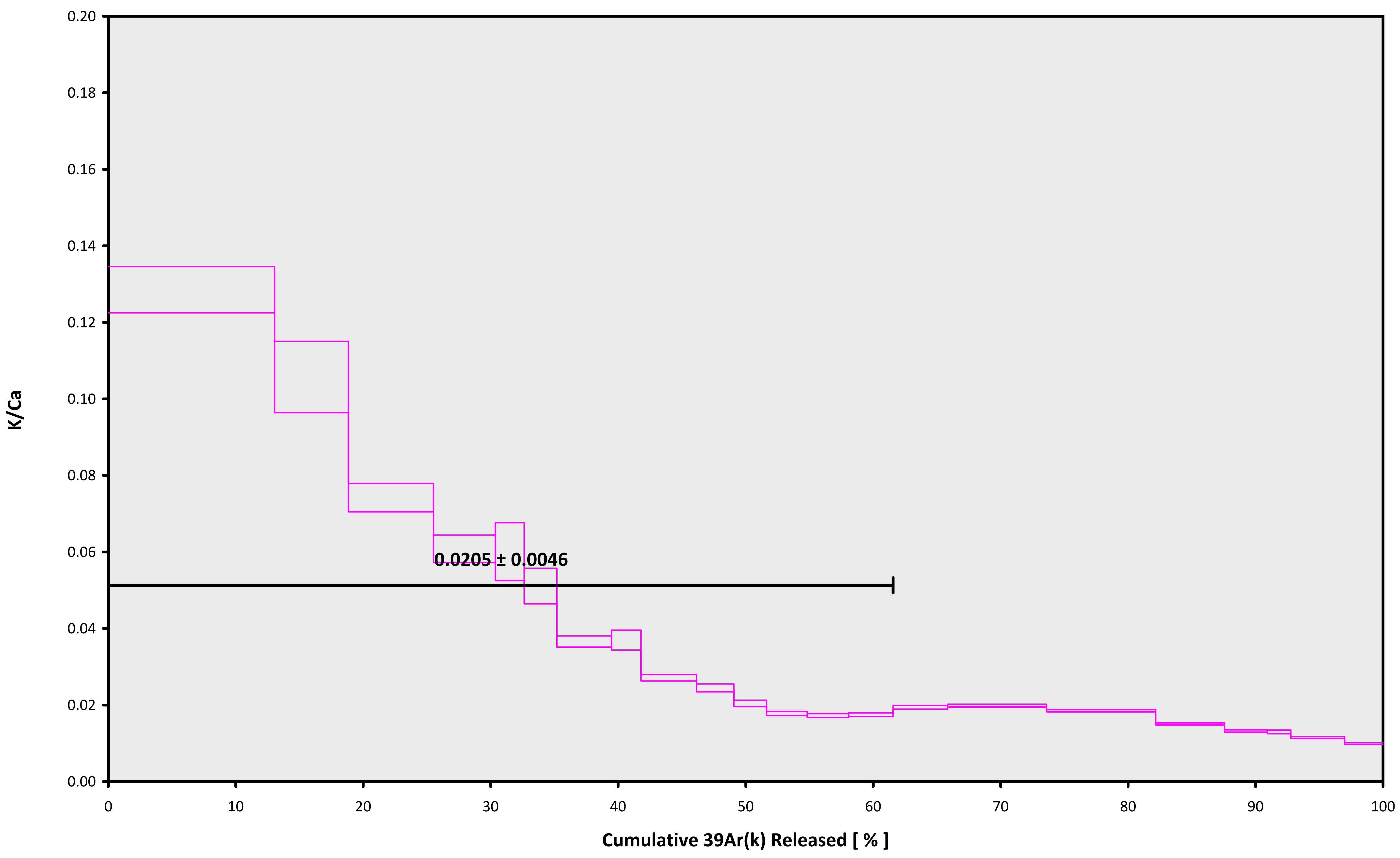

Ar-Ages in Ma

WEIGHTED PLATEAU
5.59 ± 1.41
TOTAL FUSION
16.70 ± 3.01
NORMAL ISOCHRON
3.37 ± 3.29
INVERSE ISOCHRON
3.37 ± 2.13

Sample Info

Groundmass
Gakkel Ridge
Dan Miggins

IRR = 17-OSU-05 (5B18-17)
J = 0.00160719 ± 0.00000130

18D00212.AGE >>> PS59-229-13 >>> ARCTIC | O-CONNOR (16-22) PROJECT

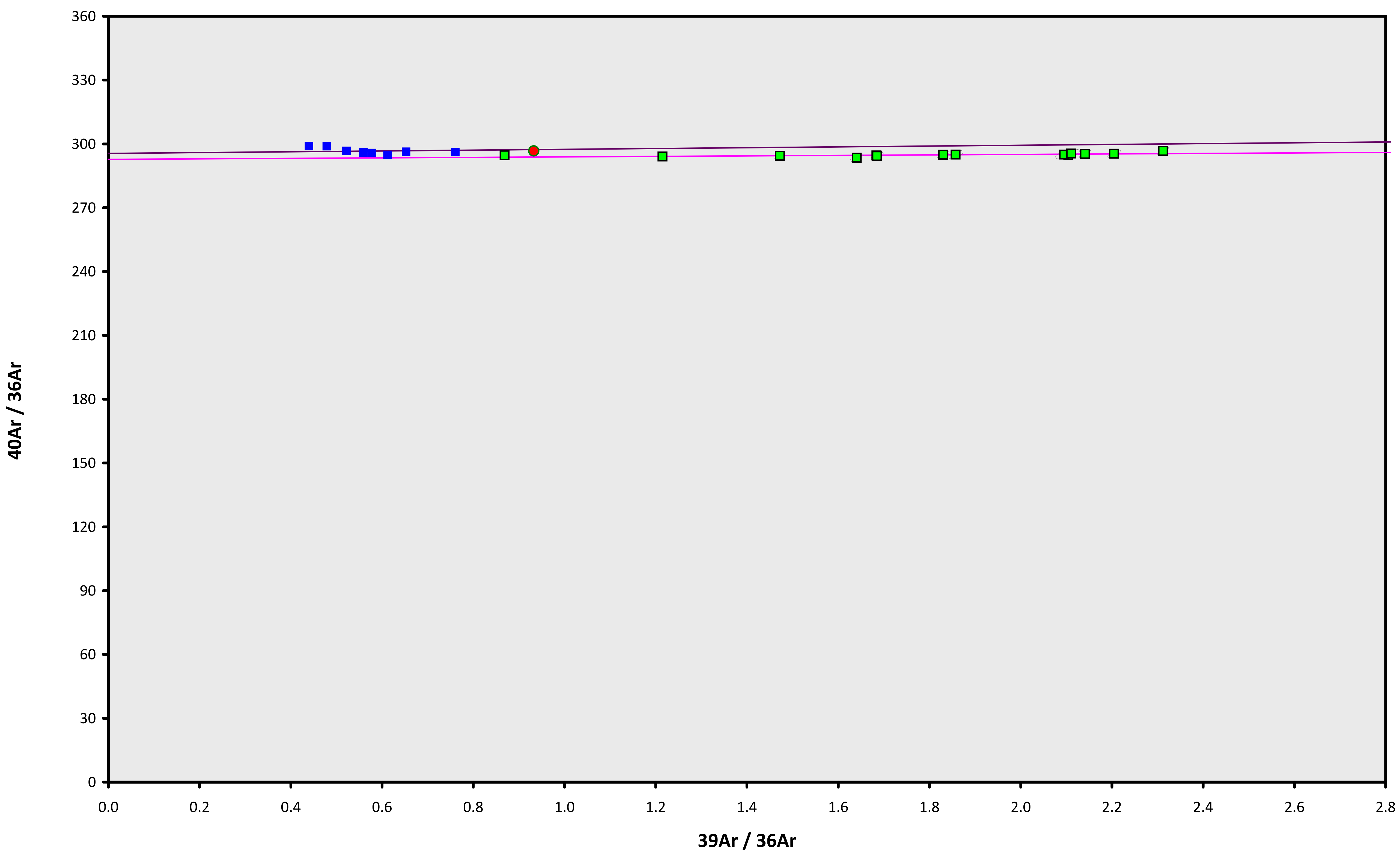

Ar-Ages in Ma

WEIGHTED PLATEAU
5.59 ± 1.41
TOTAL FUSION
16.70 ± 3.01
NORMAL ISOCHRON
3.37 ± 3.29
INVERSE ISOCHRON
3.37 ± 2.13

MSWD (PROBABILITY)
0.47 (93%)

40AR/36AR INTERCEPT
292.8 ± 2.1

Sample Info

Groundmass
Gakkel Ridge
Dan Miggins

IRR = 17-OSU-05 (5B18-17)
J = 0.00160719 ± 0.00000130

18D00212.AGE >>> PS59-229-13 >>> ARCTIC | O-CONNOR (16-22) PROJECT

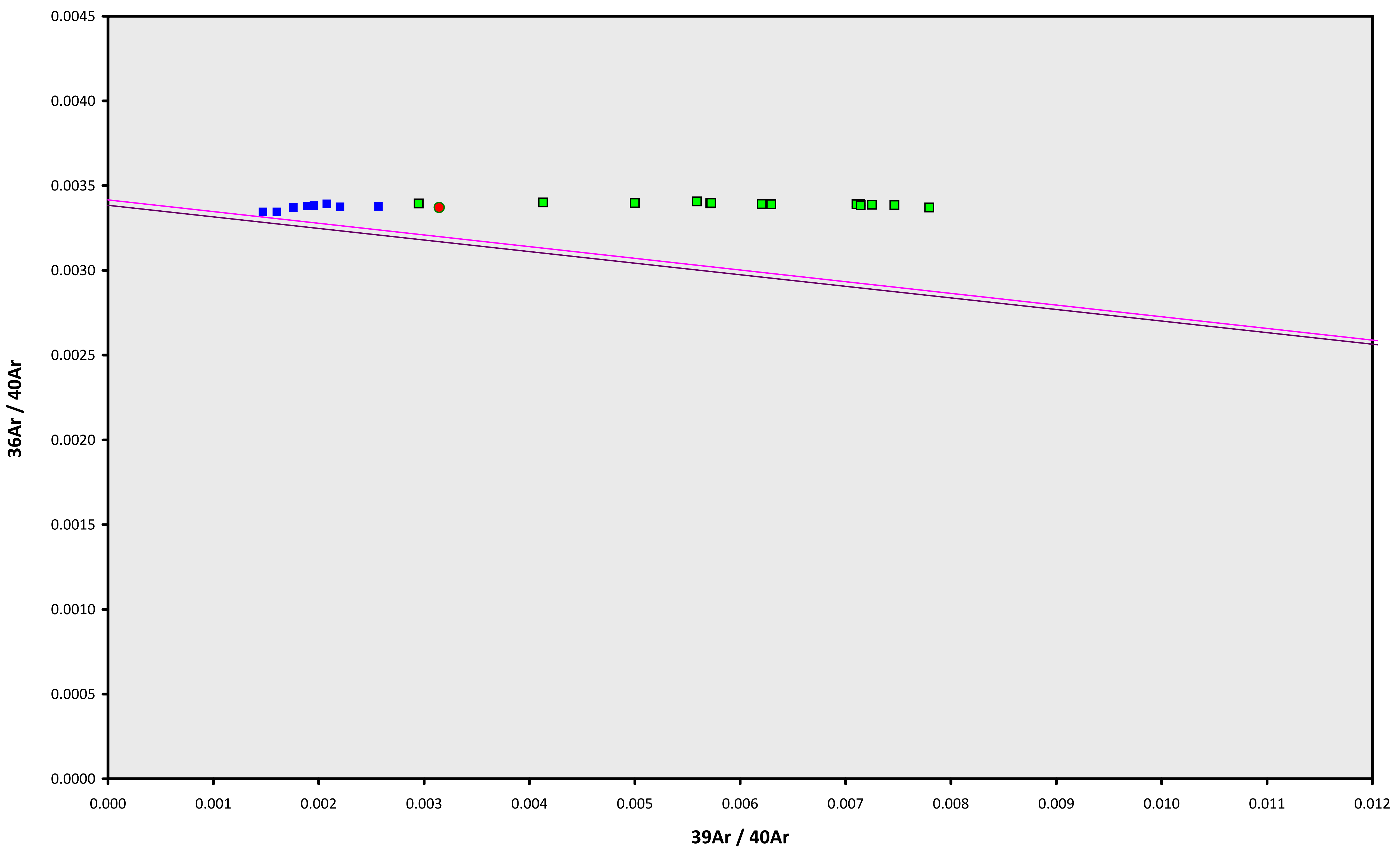

Ar-Ages in Ma

WEIGHTED PLATEAU
5.59 ± 1.41
TOTAL FUSION
16.70 ± 3.01
NORMAL ISOCHRON
3.37 ± 3.29
INVERSE ISOCHRON
3.37 ± 2.13

MSWD (PROBABILITY)
0.47 (93%)

SPREADING FACTOR
0.6%

40AR/36AR INTERCEPT
292.8 ± 2.1

Sample Info

Groundmass
Gakkel Ridge
Dan Miggins

IRR = 17-OSU-05 (5B18-17)
J = 0.00160719 ± 0.00000130

Relative Abundances			36Ar [fA]	%1σ	37Ar [fA]	%1σ	38Ar [fA]	%1σ	39Ar [fA]	%1σ	40Ar [fA]	%1σ	40(r)/39(k) ± 2σ	Age ± 2σ (Ma)	40Ar(r) (%)	39Ar(k) (%)	K/Ca ± 2σ
18D00655	1.8 %	✓	10.46148	0.286	112.1065	2.730	2.300478	1.002	24.73348	0.142	3106.599	0.006	0.98031 ± 0.93033	2.90 ± 2.75	0.78	12.94	0.0946 ± 0.0052
18D00657	1.9 %	✓	7.04912	0.288	98.7288	3.165	1.527248	1.514	16.00312	0.210	2087.253	0.008	0.76014 ± 0.97316	2.25 ± 2.87	0.58	8.36	0.0694 ± 0.0044
18D00658	2.0 %	✓	4.87048	0.289	82.9753	3.474	1.072191	2.150	10.94292	0.291	1444.302	0.012	1.07423 ± 0.98848	3.17 ± 2.92	0.81	5.71	0.0564 ± 0.0039
18D00660	2.2 %	✓	4.55561	0.293	98.6281	3.069	1.018671	2.381	9.84893	0.324	1348.423	0.012	1.03383 ± 1.03654	3.05 ± 3.06	0.75	5.13	0.0427 ± 0.0026
18D00661	2.4 %	✓	3.22508	0.300	80.8172	3.895	0.710059	3.396	7.22578	0.421	952.899	0.017	0.88398 ± 1.01650	2.61 ± 3.00	0.67	3.76	0.0382 ± 0.0030
18D00663	2.7 %	✓	3.60995	0.296	123.9396	2.502	0.798234	2.864	8.10354	0.385	1067.729	0.016	1.35638 ± 1.00815	4.01 ± 2.97	1.02	4.21	0.0278 ± 0.0014
18D00664	3.0 %	✓	3.38624	0.298	158.4131	1.918	0.768935	3.244	7.99990	0.386	997.410	0.017	1.19341 ± 0.96375	3.53 ± 2.84	0.95	4.14	0.0214 ± 0.0008
18D00666	3.4 %	✓	3.09393	0.294	184.7731	1.711	0.685330	3.488	7.33737	0.424	909.603	0.018	1.39929 ± 0.95359	4.13 ± 2.81	1.11	3.79	0.0168 ± 0.0006
18D00667	3.9 %	✓	3.06009	0.296	229.4621	1.470	0.707112	3.322	7.53729	0.427	898.915	0.018	1.75682 ± 0.92542	5.19 ± 2.73	1.44	3.88	0.0138 ± 0.0004
18D00669	4.5 %	✓	3.01822	0.298	274.7411	1.265	0.692478	3.333	7.30589	0.424	882.263	0.019	1.72787 ± 0.94850	5.10 ± 2.80	1.40	3.74	0.0112 ± 0.0003
18D00670	5.2 %	✓	3.10474	0.297	350.2117	1.114	0.668466	3.461	7.52045	0.428	903.555	0.018	1.92900 ± 0.95026	5.70 ± 2.80	1.56	3.83	0.0090 ± 0.0002
18D00672	6.0 %	✓	3.22417	0.295	457.7159	0.944	0.695314	3.437	7.85841	0.394	930.354	0.018	1.87273 ± 0.94570	5.53 ± 2.79	1.52	3.97	0.0071 ± 0.0001
18D00673	6.9 %	✓	3.01460	0.295	555.9030	0.874	0.660839	3.792	7.84098	0.399	860.859	0.019	1.92982 ± 0.89193	5.70 ± 2.63	1.68	3.93	0.0058 ± 0.0001
18D00675	7.9 %	✓	2.64034	0.297	536.9521	0.894	0.592888	4.002	7.01310	0.456	750.619	0.022	1.99200 ± 0.87967	5.88 ± 2.59	1.77	3.50	0.0053 ± 0.0001
18D00676	9.0 %		2.04607	0.300	392.1770	1.024	0.444555	5.199	5.09866	0.613	585.902	0.028	2.60215 ± 0.94776	7.68 ± 2.79	2.15	2.54	0.0053 ± 0.0001
18D00678	10.3 %		3.14174	0.295	455.3635	0.953	0.643123	3.606	6.58328	0.487	912.935	0.018	3.32534 ± 1.10737	9.81 ± 3.26	2.29	3.30	0.0059 ± 0.0001
18D00679	11.6 %		3.21155	0.296	316.0825	1.211	0.674280	3.345	5.41436	0.583	951.547	0.017	5.33023 ± 1.37451	15.69 ± 4.03	2.92	2.73	0.0071 ± 0.0002
18D00681	12.5 %		3.54974	0.292	240.9688	1.391	0.747474	3.291	4.80373	0.648	1064.146	0.016	7.40849 ± 1.69477	21.78 ± 4.95	3.24	2.44	0.0083 ± 0.0003
18D00682	13.4 %		5.44809	0.288	242.8095	1.363	1.126802	1.979	5.56236	0.560	1643.045	0.010	9.71556 ± 2.21717	28.50 ± 6.45	3.20	2.84	0.0096 ± 0.0003
18D00684	14.6 %		6.06848	0.290	230.9080	1.494	1.219794	1.904	5.49574	0.557	1832.872	0.009	10.86093 ± 2.50865	31.83 ± 7.29	3.17	2.81	0.0100 ± 0.0003
18D00685	16.0 %		7.56292	0.288	289.1533	1.227	1.575090	1.488	6.46609	0.485	2277.811	0.008	10.51958 ± 2.64593	30.84 ± 7.69	2.90	3.30	0.0093 ± 0.0002
18D00687	17.6 %		9.46370	0.286	441.2596	0.974	1.910249	1.215	8.28700	0.382	2852.192	0.006	11.35854 ± 2.58570	33.28 ± 7.51	3.19	4.20	0.0078 ± 0.0002
18D00688	19.3 %		5.42478	0.288	332.0333	1.139	1.313706	1.724	5.74167	0.537	1616.342	0.011	7.20960 ± 2.15282	21.19 ± 6.29	2.47	2.90	0.0072 ± 0.0002
18D00690	21.0 %		4.33798	0.291	267.1127	1.326	0.888730	2.652	4.07723	0.757	1307.277	0.013	11.96689 ± 2.46022	35.05 ± 7.14	3.58	2.05	0.0063 ± 0.0002
		Σ	108.56910	0.065	6553.2358	0.273	23.442044	0.491	194.80129	0.079	32184.852	0.003					

Information on Analysis and Constants Used in Calculations

Project = **O-CONNOR (16-22)**
Sample = **HLY0102-D55-9**
Material = **Groundmass**
Location = **Gakkel Ridge**
Region = **Artic Ocean**
Analyst = **Dan Miggins**
Irradiation = **17-OSU-05 (5B8-17)**
Position = **X: 0 | Y: 0 | Z/H: 12.04491 mm**
FCT-NM Age = **28.201 ± 0.023 Ma**
FCT-NM Reference = **Kuiper et al (2008)**
FCT-NM 40Ar/39Ar Ratio = **9.61157 ± 0.00788**
FCT-NM J-value = **0.00163526 ± 0.00000134**
Air Shot 40Ar/36Ar = **302.8580 ± 0.4603**
Air Shot MDF = **0.99391835 ± 0.00068767 (LIN)**
Experiment Type = **Incremental Heating**
Extraction Method = **Bulk Laser Heating**
Heating = **77 sec**
Isolation = **3.00 min**
Instrument = **ARGUS-VI-D**
Preferred Age = **Plateau Age**
Age Classification = **Crystallization Age**
IGSN = Undefined
Rock Class = Undefined
Lithology = **Basaltic Lava**
Lat-Lon = Undefined - Undefined

Age Equations = **Min et al. (2000)**
Negative Intensities = **Allowed**
Collector Calibrations = **36Ar**
Decay 40K = **5.530 ± 0.048 E-10 1/a**
Decay 39Ar = **2.940 ± 0.016 E-07 1/h**
Decay 37Ar = **8.230 ± 0.012 E-04 1/h**
Decay 36Cl = **2.257 ± 0.015 E-06 1/a**
Decay 40K(EC,β^+) = **0.580 ± 0.009 E-10 1/a**
Decay 40K(β^-) = **4.950 ± 0.043 E-10 1/a**
Atmospheric 40/36(a) = **295.50 ± 0.70**
Atmospheric 38/36(a) = **0.1869**
Production 39/37(ca) = **0.0006425 ± 0.0000059**
Production 38/37(ca) = **0.0001800 ± 0.0000173**
Production 36/37(ca) = **0.0002703 ± 0.0000005**
Production 40/39(k) = **0.000607 ± 0.000059**
Production 38/39(k) = **0.012077 ± 0.000011**
Production 36/38(cl) = **262.80 ± 1.71**
Scaling Ratio K/Ca = **0.430**
Abundance Ratio 40K/K = **1.1700 ± 0.0100 E-04**
Atomic Weight K = **39.0983 ± 0.0001 g**

Results	40(a)/36(a) ± 2σ	40(r)/39(k) ± 2σ	Age ± 2σ (Ma)	MSWD	39Ar(k) (%,n)	K/Ca ± 2σ
**Age Plateau**		1.44732 ± 0.25526 ± 17.64%	**4.27 ± 0.75 ± 17.62%**	0.85 60%	70.90 14	0.0069 ± 0.0018
		Full External Error	± 0.76	1.78	2σ Confidence Limit	
		Analytical Error	± 0.75	1.0000	Error Magnification	
**Total Fusion Age**		3.28476 ± 0.28369 ± 8.64%	**9.69 ± 0.83 ± 8.61%**		24	0.0125 ± 0.0001
		Full External Error	± 0.86			
		Analytical Error	± 0.83			
**Normal Isochron**	**281.31** ± 7.93 ± 2.82%	7.40194 ± 3.33979 ± 45.12%	**21.76 ± 9.76 ± 44.85%**	0.52 90%	70.90 14	
		Full External Error	± 9.77	1.82	2σ Confidence Limit	
		Analytical Error	± 9.76	1.0000	Error Magnification	
				4	Number of Iterations	
				0.0000245246	Convergence	
**Inverse Isochron** Clustered Points	**281.32** ± 7.94 ± 2.82%	7.39809 ± 2.86788 ± 38.77%	**21.75 ± 8.38 ± 38.53%**	0.52 90%	70.90 14	
		Full External Error	± 8.39	1.82	2σ Confidence Limit	
		Analytical Error	± 8.38	1.0000	Error Magnification	
**Notes**				3	Number of Iterations	
Mostly atmospheric				0.0000542284	Convergence	
				1%	Spreading Factor	

Incremental Heating			36Ar(a) [fA]	37Ar(ca) [fA]	38Ar(cl) [fA]	39Ar(k) [fA]	40Ar(r) [fA]	Age ± 2σ (Ma)	40Ar(r) (%)	39Ar(k) (%)	K/Ca ± 2σ
18D00655	1.8 %	✓	10.43116	112.1065	0.0328781	24.66145	24.17579	2.90 ± 2.75	0.78	12.94	0.0946 ± 0.0052
18D00657	1.9 %	✓	7.02243	98.7288	0.0044815	15.93968	12.11635	2.25 ± 2.87	0.58	8.36	0.0694 ± 0.0044
18D00658	2.0 %	✓	4.84804	82.9753	0.0196424	10.88961	11.69799	3.17 ± 2.92	0.81	5.71	0.0564 ± 0.0039
18D00660	2.2 %	✓	4.52894	98.6281	0.0362796	9.78557	10.11664	3.05 ± 3.06	0.75	5.13	0.0427 ± 0.0026
18D00661	2.4 %	✓	3.20323	80.8172	0.0101906	7.17385	6.34155	2.61 ± 3.00	0.67	3.76	0.0382 ± 0.0030
18D00663	2.7 %	✓	3.57645	123.9396	0.0105818	8.02391	10.88346	4.01 ± 2.97	1.02	4.21	0.0278 ± 0.0014
18D00664	3.0 %	✓	3.34342	158.4131	0.0201501	7.89812	9.42568	3.53 ± 2.84	0.95	4.14	0.0214 ± 0.0008
18D00666	3.4 %	✓	3.04399	184.7731	0.0000000	7.21865	10.10100	4.13 ± 2.81	1.11	3.79	0.0168 ± 0.0006
18D00667	3.9 %	✓	2.99806	229.4621	0.0162229	7.38986	12.98266	5.19 ± 2.73	1.44	3.88	0.0138 ± 0.0004
18D00669	4.5 %	✓	2.94396	274.7411	0.0066970	7.12937	12.31864	5.10 ± 2.80	1.40	3.74	0.0112 ± 0.0003
18D00670	5.2 %	✓	3.01008	350.2117	0.0000000	7.29544	14.07289	5.70 ± 2.80	1.56	3.83	0.0090 ± 0.0002
18D00672	6.0 %	✓	3.10045	457.7159	0.0000000	7.56433	14.16597	5.53 ± 2.79	1.52	3.97	0.0071 ± 0.0001
18D00673	6.9 %	✓	2.86434	555.9030	0.0000000	7.48381	14.44243	5.70 ± 2.63	1.68	3.93	0.0058 ± 0.0001
18D00675	7.9 %	✓	2.49520	536.9521	0.0000000	6.66810	13.28288	5.88 ± 2.59	1.77	3.50	0.0053 ± 0.0001
18D00676	9.0 %		1.94006	392.1770	0.0000000	4.84669	12.61180	7.68 ± 2.79	2.15	2.54	0.0053 ± 0.0001
18D00678	10.3 %		3.01866	455.3635	0.0000000	6.29071	20.91874	9.81 ± 3.26	2.29	3.30	0.0059 ± 0.0001
18D00679	11.6 %		3.12612	316.0825	0.0000000	5.21128	27.77731	15.69 ± 4.03	2.92	2.73	0.0071 ± 0.0002
18D00681	12.5 %		3.48461	240.9688	0.0000000	4.64891	34.44136	21.78 ± 4.95	3.24	2.44	0.0083 ± 0.0003
18D00682	13.4 %		5.38246	242.8095	0.0118225	5.40636	52.52582	28.50 ± 6.45	3.20	2.84	0.0096 ± 0.0003
18D00684	14.6 %		6.00606	230.9080	0.0000000	5.34738	58.07756	31.83 ± 7.29	3.17	2.81	0.0100 ± 0.0003
18D00685	16.0 %		7.48474	289.1533	0.0482977	6.28031	66.06624	30.84 ± 7.69	2.90	3.30	0.0093 ± 0.0002
18D00687	17.6 %		9.34443	441.2596	0.0000000	8.00349	90.90796	33.28 ± 7.51	3.19	4.20	0.0078 ± 0.0002
18D00688	19.3 %		5.33496	332.0333	0.1900697	5.52834	39.85710	21.19 ± 6.29	2.47	2.90	0.0072 ± 0.0002
18D00690	21.0 %		4.26578	267.1127	0.0000000	3.90561	46.73797	35.05 ± 7.14	3.58	2.05	0.0063 ± 0.0002
		Σ	106.79760	6553.2358	0.4073139	190.59083	626.04576				

Information on Analysis

Project = **O-CONNOR (16-22)**
Sample = **HLY0102-D55-9**
Material = **Groundmass**
Location = **Gakkel Ridge**
Region = **Artic Ocean**
Analyst = **Dan Miggins**
Irradiation = **17-OSU-05 (5B8-17)**
J = **0.00163526 ± 0.00000134**
FCT-NM = **28.201 ± 0.023 Ma**

Results	40(r)/39(k) ± 2σ		Age ± 2σ (Ma)	MSWD	39Ar(k) (%,n)	K/Ca ± 2σ
**Age Plateau**	1.44732	± 0.25526<br>± 17.64%	**4.27** **± 0.75**<br>**± 17.62%**	0.85<br>60%	70.90<br>14	0.0069 ± 0.0018
		Full External Error	± 0.76	1.78	2σ Confidence Limit	
		Analytical Error	± 0.75	1.0000	Error Magnification	
**Total Fusion Age**	3.28476	± 0.28369<br>± 8.64%	**9.69** **± 0.83**<br>**± 8.61%**		24	0.0125 ± 0.0001
		Full External Error	± 0.86			
		Analytical Error	± 0.83			

Normal Isochron			39(k)/36(a) ± 2σ	40(a+r)/36(a) ± 2σ	r.i.
18D00655	1.8 %	✓	2.36 ± 0.02	297.82 ± 1.71	0.8952
18D00657	1.9 %	✓	2.27 ± 0.02	297.23 ± 1.72	0.8071
18D00658	2.0 %	✓	2.25 ± 0.02	297.91 ± 1.74	0.7041
18D00660	2.2 %	✓	2.16 ± 0.02	297.73 ± 1.76	0.6700
18D00661	2.4 %	✓	2.24 ± 0.02	297.48 ± 1.81	0.5800
18D00663	2.7 %	✓	2.24 ± 0.02	298.54 ± 1.79	0.6091
18D00664	3.0 %	✓	2.36 ± 0.02	298.32 ± 1.81	0.6111
18D00666	3.4 %	✓	2.37 ± 0.02	298.82 ± 1.80	0.5694
18D00667	3.9 %	✓	2.46 ± 0.03	299.83 ± 1.83	0.5701
18D00669	4.5 %	✓	2.42 ± 0.03	299.68 ± 1.85	0.5753
18D00670	5.2 %	✓	2.42 ± 0.03	300.18 ± 1.86	0.5699
18D00672	6.0 %	✓	2.44 ± 0.03	300.07 ± 1.86	0.5997
18D00673	6.9 %	✓	2.61 ± 0.03	300.54 ± 1.89	0.5962
18D00675	7.9 %	✓	2.67 ± 0.03	300.82 ± 1.92	0.5485
18D00676	9.0 %		2.50 ± 0.04	302.00 ± 1.95	0.4423
18D00678	10.3 %		2.08 ± 0.02	302.43 ± 1.88	0.5157
18D00679	11.6 %		1.67 ± 0.02	304.39 ± 1.86	0.4480
18D00681	12.5 %		1.33 ± 0.02	305.38 ± 1.83	0.4060
18D00682	13.4 %		1.00 ± 0.01	305.26 ± 1.78	0.4509
18D00684	14.6 %		0.89 ± 0.01	305.17 ± 1.79	0.4547
18D00685	16.0 %		0.84 ± 0.01	304.33 ± 1.77	0.5017
18D00687	17.6 %		0.86 ± 0.01	305.23 ± 1.77	0.5882
18D00688	19.3 %		1.04 ± 0.01	302.97 ± 1.78	0.4629
18D00690	21.0 %		0.92 ± 0.02	306.46 ± 1.82	0.3499

Results	40(a)/36(a) ± 2σ		40(r)/39(k) ± 2σ		Age ± 2σ (Ma)		MSWD
**Normal Isochron**	**281.31**	**± 7.93**<br>**± 2.82%**	7.40194	± 3.33979<br>± 45.12%	**21.76**	**± 9.76**<br>**± 44.85%**	0.52<br>90%
					Full External Error	± 9.77	
					Analytical Error	± 9.76	

Statistics				
	2σ Confidence Limit	1.82	Convergence	0.000024524620
	Error Magnification	1.0000	Number of Iterations	4
	Number of Data Points	14	Calculated Line	Weighted York-2

Inverse Isochron			39(k)/40(a+r) ± 2σ	36(a)/40(a+r) ± 2σ	r.i.
18D00655	1.8 %	✓	0.0079384 ± 0.0000227	0.00335776 ± 0.00001927	0.0009
18D00657	1.9 %	✓	0.0076367 ± 0.0000323	0.00336445 ± 0.00001945	0.0011
18D00658	2.0 %	✓	0.0075397 ± 0.0000443	0.00335669 ± 0.00001957	0.0016
18D00660	2.2 %	✓	0.0072571 ± 0.0000475	0.00335871 ± 0.00001986	0.0016
18D00661	2.4 %	✓	0.0075285 ± 0.0000640	0.00336157 ± 0.00002041	0.0023
18D00663	2.7 %	✓	0.0075150 ± 0.0000586	0.00334960 ± 0.00002013	0.0021
18D00664	3.0 %	✓	0.0079187 ± 0.0000621	0.00335211 ± 0.00002036	0.0023
18D00666	3.4 %	✓	0.0079361 ± 0.0000686	0.00334651 ± 0.00002010	0.0025
18D00667	3.9 %	✓	0.0082209 ± 0.0000719	0.00333522 ± 0.00002031	0.0025
18D00669	4.5 %	✓	0.0080808 ± 0.0000705	0.00333684 ± 0.00002055	0.0026
18D00670	5.2 %	✓	0.0080742 ± 0.0000717	0.00333139 ± 0.00002060	0.0024
18D00672	6.0 %	✓	0.0081306 ± 0.0000671	0.00333257 ± 0.00002067	0.0025
18D00673	6.9 %	✓	0.0086935 ± 0.0000735	0.00332732 ± 0.00002096	0.0028
18D00675	7.9 %	✓	0.0088835 ± 0.0000861	0.00332421 ± 0.00002123	0.0031
18D00676	9.0 %		0.0082722 ± 0.0001074	0.00331125 ± 0.00002137	0.0037
18D00678	10.3 %		0.0068907 ± 0.0000708	0.00330655 ± 0.00002050	0.0020
18D00679	11.6 %		0.0054767 ± 0.0000667	0.00328531 ± 0.00002012	0.0016
18D00681	12.5 %		0.0043687 ± 0.0000587	0.00327457 ± 0.00001961	0.0012
18D00682	13.4 %		0.0032905 ± 0.0000380	0.00327591 ± 0.00001915	0.0006
18D00684	14.6 %		0.0029175 ± 0.0000335	0.00327686 ± 0.00001925	0.0005
18D00685	16.0 %		0.0027572 ± 0.0000277	0.00328594 ± 0.00001913	0.0004
18D00687	17.6 %		0.0028061 ± 0.0000224	0.00327623 ± 0.00001900	0.0003
18D00688	19.3 %		0.0034203 ± 0.0000384	0.00330065 ± 0.00001937	0.0007
18D00690	21.0 %		0.0029876 ± 0.0000474	0.00326311 ± 0.00001938	0.0007

Results	40(a)/36(a) ± 2σ		40(r)/39(k) ± 2σ		Age ± 2σ (Ma)		MSWD
**Inverse Isochron**<br>Clustered Points	**281.32**	**± 7.94**<br>**± 2.82%**	7.39809	± 2.86788<br>± 38.77%	**21.75**	**± 8.38**<br>**± 38.53%**	0.52<br>90%
					Full External Error	± 8.39	
					Analytical Error	± 8.38	

Statistics				
	2σ Confidence Limit	1.82	Convergence	0.0000542284
	Error Magnification	1.0000	Number of Iterations	3
	Number of Data Points	14	Calculated Line	Weighted York-2
	Spreading Factor	1.2%		

Degassing Patterns			36Ar(a) [fA]	%1σ	36Ar(c) [fA]	%1σ	36Ar(ca) [fA]	%1σ	36Ar(cl) [fA]	%1σ	37Ar(ca) [fA]	%1σ	38Ar(a) [fA]	%1σ	38Ar(c) [fA]	%1σ	38Ar(k) [fA]	%1σ	38Ar(ca) [fA]	%1σ	38Ar(cl) [fA]	%1σ	39Ar(k) [fA]	%1σ	39Ar(ca) [fA]	%1σ	40Ar(r) [fA]	%1σ	40Ar(a) [fA]	%1σ	40Ar(c) [fA]	%1σ	40Ar(k) [fA]	%1σ
18D00655	1.8 %	✓	10.43116	0.29	0.0000000	0.00	0.0303024	2.74	0.0000128	72.45	112.1065	2.73	1.949584	0.29	0.0000000	0.00	0.2978363	0.17	0.0201792	10.01	0.0328781	72.46	24.66145	0.14	0.0720284	2.88	24.17579	47.45	3082.408	0.37	0.0000000	0.00	0.0149695	9.65
18D00657	1.9 %	✓	7.02243	0.29	0.0000000	0.00	0.0266864	3.17	0.0000017	524.66	98.7288	3.16	1.312492	0.29	0.0000000	0.00	0.1925035	0.23	0.0177712	10.14	0.0044815	524.66	15.93968	0.21	0.0634333	3.30	12.11635	64.01	2075.127	0.37	0.0000000	0.00	0.0096754	9.65
18D00658	2.0 %	✓	4.84804	0.29	0.0000000	0.00	0.0224282	3.48	0.0000076	118.44	82.9753	3.47	0.906100	0.29	0.0000000	0.00	0.1315138	0.31	0.0149356	10.24	0.0196424	118.44	10.88961	0.29	0.0533116	3.59	11.69799	46.01	1432.597	0.38	0.0000000	0.00	0.0066100	9.65
18D00660	2.2 %	✓	4.52894	0.30	0.0000000	0.00	0.0266592	3.07	0.0000141	67.41	98.6281	3.07	0.846458	0.30	0.0000000	0.00	0.1181803	0.34	0.0177531	10.11	0.0362796	67.42	9.78557	0.33	0.0633686	3.20	10.11664	50.13	1338.301	0.38	0.0000000	0.00	0.0059398	9.66
18D00661	2.4 %	✓	3.20323	0.30	0.0000000	0.00	0.0218449	3.90	0.0000040	237.80	80.8172	3.89	0.598683	0.30	0.0000000	0.00	0.0866386	0.43	0.0145471	10.39	0.0101906	237.80	7.17385	0.42	0.0519250	4.00	6.34155	57.49	946.554	0.38	0.0000000	0.00	0.0043545	9.66
18D00663	2.7 %	✓	3.57645	0.30	0.0000000	0.00	0.0335009	2.51	0.0000041	217.90	123.9396	2.50	0.668438	0.30	0.0000000	0.00	0.0969048	0.40	0.0223091	9.95	0.0105818	217.90	8.02391	0.39	0.0796312	2.67	10.88346	37.16	1056.840	0.38	0.0000000	0.00	0.0048705	9.66
18D00664	3.0 %	✓	3.34342	0.30	0.0000000	0.00	0.0428191	1.93	0.0000078	124.94	158.4131	1.92	0.624885	0.30	0.0000000	0.00	0.0953856	0.40	0.0285144	9.82	0.0201501	124.95	7.89812	0.39	0.1017804	2.13	9.42568	40.38	987.980	0.38	0.0000000	0.00	0.0047942	9.66
18D00666	3.4 %	✓	3.04399	0.30	0.0000000	0.00	0.0499442	1.72	0.0000000	0.00	184.7731	1.71	0.568921	0.30	0.0000000	0.00	0.0871797	0.44	0.0332592	9.78	0.0000000	0.00	7.21865	0.43	0.1187167	1.94	10.10100	34.07	899.498	0.38	0.0000000	0.00	0.0043817	9.66
18D00667	3.9 %	✓	2.99806	0.30	0.0000000	0.00	0.0620236	1.48	0.0000063	147.33	229.4621	1.47	0.560338	0.30	0.0000000	0.00	0.0892474	0.45	0.0413032	9.74	0.0162229	147.33	7.38986	0.44	0.1474294	1.73	12.98266	26.33	885.928	0.39	0.0000000	0.00	0.0044856	9.66
18D00669	4.5 %	✓	2.94396	0.31	0.0000000	0.00	0.0742625	1.28	0.0000026	353.03	274.7411	1.27	0.550226	0.31	0.0000000	0.00	0.0861014	0.44	0.0494534	9.71	0.0066970	353.04	7.12937	0.44	0.1765211	1.56	12.31864	27.44	869.940	0.39	0.0000000	0.00	0.0043275	9.66
18D00670	5.2 %	✓	3.01008	0.31	0.0000000	0.00	0.0946622	1.13	0.0000000	0.00	350.2117	1.11	0.562583	0.31	0.0000000	0.00	0.0881071	0.45	0.0630381	9.69	0.0000000	0.00	7.29544	0.44	0.2250110	1.44	14.07289	24.63	889.477	0.39	0.0000000	0.00	0.0044283	9.66
18D00672	6.0 %	✓	3.10045	0.31	0.0000000	0.00	0.1237206	0.96	0.0000000	0.00	457.7159	0.94	0.579474	0.31	0.0000000	0.00	0.0913544	0.42	0.0823889	9.68	0.0000000	0.00	7.56433	0.41	0.2940825	1.32	14.16597	25.25	916.183	0.39	0.0000000	0.00	0.0045915	9.66
18D00673	6.9 %	✓	2.86434	0.31	0.0000000	0.00	0.1502606	0.89	0.0000000	0.00	555.9030	0.87	0.535345	0.31	0.0000000	0.00	0.0903820	0.43	0.1000625	9.67	0.0000000	0.00	7.48381	0.42	0.3571677	1.27	14.44243	23.11	846.412	0.39	0.0000000	0.00	0.0045427	9.66
18D00675	7.9 %	✓	2.49520	0.32	0.0000000	0.00	0.1451381	0.91	0.0000000	0.00	536.9521	0.89	0.466353	0.32	0.0000000	0.00	0.0805307	0.49	0.0966514	9.67	0.0000000	0.00	6.66810	0.48	0.3449917	1.28	13.28288	22.07	737.332	0.40	0.0000000	0.00	0.0040475	9.66
18D00676	9.0 %		1.94006	0.32	0.0000000	0.00	0.1060054	1.04	0.0000000	0.00	392.1770	1.02	0.362597	0.32	0.0000000	0.00	0.0585334	0.65	0.0705919	9.68	0.0000000	0.00	4.84669	0.65	0.2519737	1.38	12.61180	18.20	573.288	0.40	0.0000000	0.00	0.0029419	9.67
18D00678	10.3 %		3.01866	0.31	0.0000000	0.00	0.1230847	0.97	0.0000000	0.00	455.3635	0.95	0.564187	0.31	0.0000000	0.00	0.0759729	0.52	0.0819654	9.68	0.0000000	0.00	6.29071	0.51	0.2925710	1.32	20.91874	16.64	892.013	0.39	0.0000000	0.00	0.0038185	9.66
18D00679	11.6 %		3.12612	0.31	0.0000000	0.00	0.0854371	1.22	0.0000000	0.00	316.0825	1.21	0.584271	0.31	0.0000000	0.00	0.0629366	0.62	0.0568948	9.71	0.0000000	0.00	5.21128	0.61	0.2030830	1.52	27.77731	12.88	923.767	0.39	0.0000000	0.00	0.0031632	9.67
18D00681	12.5 %		3.48461	0.30	0.0000000	0.00	0.0651339	1.40	0.0000000	0.00	240.9688	1.39	0.651273	0.30	0.0000000	0.00	0.0561448	0.68	0.0433744	9.73	0.0000000	0.00	4.64891	0.67	0.1548225	1.67	34.44136	11.42	1029.702	0.38	0.0000000	0.00	0.0028219	9.67
18D00682	13.4 %		5.38246	0.29	0.0000000	0.00	0.0656314	1.37	0.0000046	193.71	242.8095	1.36	1.005981	0.29	0.0000000	0.00	0.0652926	0.58	0.0437057	9.73	0.0118225	193.71	5.40636	0.58	0.1560051	1.64	52.52582	11.40	1590.516	0.38	0.0000000	0.00	0.0032817	9.67
18D00684	14.6 %		6.00606	0.29	0.0000000	0.00	0.0624144	1.50	0.0000000	0.00	230.9080	1.49	1.122533	0.29	0.0000000	0.00	0.0645803	0.58	0.0415634	9.75	0.0000000	0.00	5.34738	0.57	0.1483584	1.75	58.07756	11.53	1774.791	0.38	0.0000000	0.00	0.0032459	9.67
18D00685	16.0 %		7.48474	0.29	0.0000000	0.00	0.0781581	1.24	0.0000188	50.38	289.1533	1.23	1.398898	0.29	0.0000000	0.00	0.0758473	0.51	0.0520476	9.71	0.0482977	50.39	6.28031	0.50	0.1857810	1.53	66.06624	12.57	2211.741	0.38	0.0000000	0.00	0.0038121	9.66
18D00687	17.6 %		9.34443	0.29	0.0000000	0.00	0.1192725	0.99	0.0000000	0.00	441.2596	0.97	1.746474	0.29	0.0000000	0.00	0.0966581	0.41	0.0794267	9.68	0.0000000	0.00	8.00349	0.40	0.2835093	1.34	90.90796	11.38	2761.279	0.37	0.0000000	0.00	0.0048581	9.66
18D00688	19.3 %		5.33496	0.29	0.0000000	0.00	0.0897486	1.15	0.0000741	12.43	332.0333	1.14	0.997104	0.29	0.0000000	0.00	0.0667657	0.57	0.0597660	9.70	0.1900697	12.47	5.52834	0.56	0.2133314	1.46	39.85710	14.92	1576.481	0.38	0.0000000	0.00	0.0033557	9.67
18D00690	21.0 %		4.26578	0.30	0.0000000	0.00	0.0722005	1.34	0.0000000	0.00	267.1127	1.33	0.797274	0.30	0.0000000	0.00	0.0471680	0.80	0.0480803	9.72	0.0000000	0.00	3.90561	0.79	0.1716199	1.61	46.73797	10.25	1260.537	0.38	0.0000000	0.00	0.0023707	9.68
		Σ	106.79760	0.07	0.0000000	0.00	1.7713396	0.28	0.0001587	20.27	6553.2358	0.27	19.960471	0.07	0.0000000	0.00	2.3017655	0.08	1.1795824	2.23	0.4073139	20.28	190.59083	0.08	4.2104540	0.35	626.04576	4.32	31558.691	0.09	0.0000000	0.00	0.1156886	2.23
		Σ							108.56910	0.07	6553.2358	0.27									23.849133	0.37			194.80129	0.08							32184.852	0.12

**Additional Parameters**			40Ar/39Ar	1σ	37Ar/39Ar	1σ	36Ar/39Ar	1σ	Time (days)	37Ar (decay)	39Ar (decay)	40Ar (moles)
18D00655	1.8 %	✓	125.603009	0.178464	4.532583	0.123901	0.422968	0.001350	239.711	114.971821	1.00169639	1.491E-10
18D00657	1.9 %	✓	130.427939	0.273902	6.169351	0.195670	0.440484	0.001568	239.725	115.003366	1.00169649	1.002E-10
18D00658	2.0 %	✓	131.985037	0.384812	7.582558	0.264362	0.445080	0.001828	239.732	115.019141	1.00169654	6.933E-11
18D00660	2.2 %	✓	136.910565	0.444005	10.014093	0.309083	0.462548	0.002021	239.746	115.050699	1.00169664	6.472E-11
18D00661	2.4 %	✓	131.875043	0.555254	11.184570	0.438158	0.446329	0.002306	239.753	115.066482	1.00169669	4.574E-11
18D00663	2.7 %	✓	131.760699	0.507907	15.294497	0.387144	0.445478	0.002165	239.767	115.098053	1.00169679	5.125E-11
18D00664	3.0 %	✓	124.677780	0.481521	19.801875	0.387335	0.423286	0.002065	239.774	115.113841	1.00169684	4.788E-11
18D00666	3.4 %	✓	123.968555	0.525525	25.182469	0.443954	0.421667	0.002173	239.787	115.145425	1.00169693	4.366E-11
18D00667	3.9 %	✓	119.262334	0.509767	30.443560	0.466064	0.405994	0.002110	239.794	115.161220	1.00169698	4.315E-11
18D00669	4.5 %	✓	120.760447	0.512023	37.605424	0.501799	0.413122	0.002140	239.809	115.194397	1.00169709	4.235E-11
18D00670	5.2 %	✓	120.146317	0.515257	46.567898	0.555663	0.412839	0.002153	239.816	115.210199	1.00169713	4.337E-11
18D00672	6.0 %	✓	118.389546	0.466428	58.245351	0.595910	0.410283	0.002019	239.830	115.241810	1.00169723	4.466E-11
18D00673	6.9 %	✓	109.789682	0.438410	70.897114	0.681257	0.384467	0.001908	239.837	115.257618	1.00169728	4.132E-11
18D00675	7.9 %	✓	107.031012	0.488414	76.564190	0.768111	0.376487	0.002048	239.851	115.289241	1.00169738	3.603E-11
18D00676	9.0 %		114.912995	0.705063	76.917640	0.917833	0.401295	0.002738	239.858	115.305056	1.00169743	2.812E-11
18D00678	10.3 %		138.674796	0.675450	69.169683	0.739886	0.477230	0.002716	239.872	115.336693	1.00169753	4.382E-11
18D00679	11.6 %		175.745122	1.025543	58.378544	0.784597	0.593154	0.003879	239.878	115.352514	1.00169758	4.567E-11
18D00681	12.5 %		221.525012	1.436348	50.162874	0.769960	0.738956	0.005255	239.892	115.384163	1.00169767	5.108E-11
18D00682	13.4 %		295.386145	1.653526	43.652223	0.643278	0.979456	0.006166	239.899	115.399991	1.00169772	7.887E-11
18D00684	14.6 %		333.507698	1.858501	42.015817	0.669937	1.104214	0.006937	239.913	115.431654	1.00169782	8.798E-11
18D00685	16.0 %		352.269997	1.708977	44.718387	0.590079	1.169627	0.006596	239.921	115.449072	1.00169788	1.093E-10
18D00687	17.6 %		344.176780	1.315042	53.247233	0.557085	1.141994	0.005450	239.935	115.480748	1.00169797	1.369E-10
18D00688	19.3 %		281.510785	1.513263	57.828712	0.728388	0.944810	0.005760	239.942	115.496589	1.00169802	7.758E-11
18D00690	21.0 %		320.628972	2.427772	65.513296	1.000089	1.063953	0.008629	239.956	115.528278	1.00169812	6.275E-11

Procedure Blanks		36Ar ± 1σ (SE) [fA]	37Ar ± 1σ (SE) [fA]	38Ar ± 1σ (SE) [fA]	39Ar ± 1σ (SE) [fA]	40Ar ± 1σ (SE) [fA]
18D00655	1.8 %	0.0215466 ± 0.0002993	0.0178219 ± 0.0176863	0.0044382 ± 0.0163020	0.0299599 ± 0.0261385	6.3818510 ± 0.1566428
18D00657	1.9 %	0.0263069 ± 0.0002993	0.0299044 ± 0.0176863	0.0111693 ± 0.0163020	0.0380250 ± 0.0261385	7.9601592 ± 0.1566428
18D00658	2.0 %	0.0280442 ± 0.0002993	0.0328927 ± 0.0176863	0.0133798 ± 0.0163020	0.0404897 ± 0.0261385	8.5220616 ± 0.1566428
18D00660	2.2 %	0.0304069 ± 0.0002993	0.0344626 ± 0.0176863	0.0158364 ± 0.0163020	0.0429351 ± 0.0261385	9.2630508 ± 0.1566428
18D00661	2.4 %	0.0310986 ± 0.0002993	0.0336492 ± 0.0176863	0.0162132 ± 0.0163020	0.0431584 ± 0.0261385	9.4689516 ± 0.1566428
18D00663	2.7 %	0.0316619 ± 0.0002993	0.0301521 ± 0.0176863	0.0155715 ± 0.0163020	0.0421682 ± 0.0261385	9.6139471 ± 0.1566428
18D00664	3.0 %	0.0315939 ± 0.0002993	0.0279245 ± 0.0176863	0.0146684 ± 0.0163020	0.0411616 ± 0.0261385	9.5761332 ± 0.1566428
18D00666	3.4 %	0.0309027 ± 0.0002993	0.0234653 ± 0.0176863	0.0119676 ± 0.0163020	0.0385980 ± 0.0261385	9.3329573 ± 0.1566428
18D00667	3.9 %	0.0303341 ± 0.0002993	0.0215407 ± 0.0176863	0.0102702 ± 0.0163020	0.0372122 ± 0.0261385	9.1469640 ± 0.1566428
18D00669	4.5 %	0.0287991 ± 0.0002993	0.0188010 ± 0.0176863	0.0062085 ± 0.0163020	0.0344993 ± 0.0261385	8.6649717 ± 0.1566428
18D00670	5.2 %	0.0279573 ± 0.0002993	0.0182813 ± 0.0176863	0.0041276 ± 0.0163020	0.0334442 ± 0.0261385	8.4084310 ± 0.1566428
18D00672	6.0 %	0.0261755 ± 0.0002993	0.0189644 ± 0.0176863	0.0001220 ± 0.0163020	0.0320897 ± 0.0261385	7.8779657 ± 0.1566428
18D00673	6.9 %	0.0252782 ± 0.0002993	0.0201714 ± 0.0176863	0.0022212 ± 0.0163020	0.0318886 ± 0.0261385	7.6158408 ± 0.1566428
18D00675	7.9 %	0.0235697 ± 0.0002993	0.0241404 ± 0.0176863	0.0062142 ± 0.0163020	0.0326405 ± 0.0261385	7.1226521 ± 0.1566428
18D00676	9.0 %	0.0227953 ± 0.0002993	0.0267575 ± 0.0176863	0.0080537 ± 0.0163020	0.0336561 ± 0.0261385	6.8996652 ± 0.1566428
18D00678	10.3 %	0.0214906 ± 0.0002993	0.0327086 ± 0.0176863	0.0113026 ± 0.0163020	0.0370784 ± 0.0261385	6.5164460 ± 0.1566428
18D00679	11.6 %	0.0209911 ± 0.0002993	0.0357486 ± 0.0176863	0.0126730 ± 0.0163020	0.0395120 ± 0.0261385	6.3605680 ± 0.1566428
18D00681	12.5 %	0.0203674 ± 0.0002993	0.0410369 ± 0.0176863	0.0148272 ± 0.0163020	0.0458470 ± 0.0261385	6.1265080 ± 0.1566428
18D00682	13.4 %	0.0202682 ± 0.0002993	0.0428422 ± 0.0176863	0.0155872 ± 0.0163020	0.0497394 ± 0.0261385	6.0489580 ± 0.1566428
18D00684	14.6 %	0.0205497 ± 0.0002993	0.0434819 ± 0.0176863	0.0164330 ± 0.0163020	0.0589072 ± 0.0261385	5.9697441 ± 0.1566428
18D00685	16.0 %	0.0209994 ± 0.0002993	0.0414541 ± 0.0176863	0.0165050 ± 0.0163020	0.0646833 ± 0.0261385	5.9656682 ± 0.1566428
18D00687	17.6 %	0.0223952 ± 0.0002993	0.0316785 ± 0.0176863	0.0159195 ± 0.0163020	0.0763530 ± 0.0261385	6.0168482 ± 0.1566428
18D00688	19.3 %	0.0233861 ± 0.0002993	0.0230812 ± 0.0176863	0.0152872 ± 0.0163020	0.0826696 ± 0.0261385	6.0637097 ± 0.1566428
18D00690	21.0 %	0.0259787 ± 0.0002993	0.0035829 ± 0.0176863	0.0133804 ± 0.0163020	0.0960177 ± 0.0261385	6.1779790 ± 0.1566428

Intercept Values		36Ar ± 1σ (SE) [fA]	r2	Regression (type,n)	37Ar ± 1σ (SE) [fA]	r2	Regression (type,n)	38Ar ± 1σ (SE) [fA]	r2	Regression (type,n)	39Ar ± 1σ (SE) [fA]	r2	Regression (type,n)	40Ar ± 1σ (SE) [fA]	r2	Regression (type,n)
18D00655	1.8 %	9.9678508 ± 0.0049511	0.9951	EXP 150 of 150	0.9394923 ± 0.0180189	0.0783	EXP 150 of 150	2.2680629 ± 0.0155886	0.4122	EXP 150 of 150	24.5716732 ± 0.0155990	0.9807	EXP 150 of 150	3112.9808 ± 0.0985	0.9999	EXP 150 of 150
18D00657	1.9 %	6.7282910 ± 0.0039008	0.9934	EXP 150 of 150	0.8129420 ± 0.0190637	0.0203	EXP 150 of 150	1.4975055 ± 0.0158700	0.1442	EXP 150 of 150	15.9170645 ± 0.0175163	0.9330	EXP 150 of 150	2095.2136 ± 0.0670	0.9999	EXP 150 of 150
18D00658	2.0 %	4.6586792 ± 0.0030992	0.9910	EXP 150 of 150	0.6753691 ± 0.0163646	0.0382	EXP 150 of 150	1.0457723 ± 0.0158382	0.0622	EXP 150 of 150	10.8985666 ± 0.0161604	0.8429	EXP 150 of 150	1452.8238 ± 0.0597	0.9998	EXP 150 of 150
18D00660	2.2 %	4.3616766 ± 0.0035212	0.9869	EXP 150 of 150	0.8071778 ± 0.0178720	0.0477	EXP 150 of 150	0.9904464 ± 0.0175021	0.0916	EXP 150 of 150	9.8155071 ± 0.0165581	0.8061	EXP 150 of 150	1357.6863 ± 0.0617	0.9998	EXP 150 of 150
18D00661	2.4 %	3.0973563 ± 0.0031551	0.9790	EXP 150 of 150	0.6559074 ± 0.0196192	0.0231	EXP 150 of 150	0.6852110 ± 0.0173400	0.0094	EXP 150 of 150	7.2129088 ± 0.0142132	0.6654	EXP 150 of 150	962.3684 ± 0.0544	0.9996	EXP 150 of 150
18D00663	2.7 %	3.4638435 ± 0.0031611	0.9830	EXP 149 of 150	1.0270480 ± 0.0182007	0.0801	EXP 150 of 150	0.7729549 ± 0.0155848	0.0166	EXP 150 of 150	8.0828812 ± 0.0156550	0.7678	EXP 150 of 150	1077.3426 ± 0.0544	0.9997	EXP 150 of 150
18D00664	3.0 %	3.2510827 ± 0.0031654	0.9807	EXP 150 of 150	1.3231472 ± 0.0163655	0.2037	EXP 150 of 150	0.7449151 ± 0.0184449	0.0530	EXP 150 of 150	7.9790360 ± 0.0149943	0.7894	EXP 150 of 150	1006.9862 ± 0.0559	0.9996	EXP 150 of 150
18D00666	3.4 %	2.9724725 ± 0.0024360	0.9859	EXP 150 of 150	1.5519932 ± 0.0170552	0.3750	EXP 150 of 150	0.6650284 ± 0.0170534	0.0264	EXP 150 of 150	7.3190756 ± 0.0155661	0.7334	EXP 150 of 150	918.9360 ± 0.0502	0.9996	EXP 150 of 150
18D00667	3.9 %	2.9397351 ± 0.0026711	0.9829	EXP 150 of 150	1.9346878 ± 0.0180086	0.2732	EXP 150 of 150	0.6882422 ± 0.0164888	0.0461	EXP 150 of 150	7.5160635 ± 0.0176107	0.6881	EXP 150 of 150	908.0622 ± 0.0484	0.9996	EXP 150 of 150
18D00669	4.5 %	2.8983905 ± 0.0028008	0.9798	EXP 150 of 150	2.3227693 ± 0.0171131	0.3989	EXP 150 of 150	0.6778476 ± 0.0159146	0.0728	EXP 150 of 150	7.2837403 ± 0.0153168	0.7475	EXP 150 of 150	890.9275 ± 0.0540	0.9995	EXP 150 of 150
18D00670	5.2 %	2.9798034 ± 0.0028048	0.9815	EXP 150 of 150	2.9661024 ± 0.0186962	0.4364	EXP 150 of 150	0.6562090 ± 0.0159865	0.0117	EXP 150 of 150	7.4955843 ± 0.0176779	0.6697	EXP 150 of 150	911.9631 ± 0.0496	0.9996	EXP 150 of 150
18D00672	6.0 %	3.0915735 ± 0.0027307	0.9839	EXP 150 of 150	3.8804638 ± 0.0169759	0.5956	EXP 150 of 150	0.6869797 ± 0.0170504	0.0110	EXP 150 of 150	7.8295672 ± 0.0151524	0.7434	EXP 150 of 150	938.2317 ± 0.0533	0.9996	EXP 150 of 150
18D00673	6.9 %	2.8914241 ± 0.0025504	0.9836	EXP 150 of 150	4.7150943 ± 0.0169502	0.7398	EXP 150 of 150	0.6550236 ± 0.0186040	0.0252	EXP 150 of 150	7.8120718 ± 0.0158393	0.7475	EXP 150 of 150	868.4748 ± 0.0520	0.9995	EXP 150 of 150
18D00675	7.9 %	2.5338860 ± 0.0023423	0.9828	EXP 150 of 150	4.5484435 ± 0.0178524	0.6812	EXP 149 of 150	0.5918917 ± 0.0168179	0.0464	EXP 150 of 150	6.9913565 ± 0.0173116	0.6499	EXP 150 of 150	757.7415 ± 0.0530	0.9992	EXP 150 of 150
18D00676	9.0 %	1.9681025 ± 0.0019924	0.9766	EXP 150 of 150	3.3124904 ± 0.0173826	0.6044	EXP 150 of 150	0.4472021 ± 0.0159736	0.0211	EXP 150 of 150	5.0927808 ± 0.0163133	0.4052	EXP 149 of 150	592.8020 ± 0.0456	0.9986	EXP 150 of 150
18D00678	10.3 %	3.0085165 ± 0.0026114	0.9840	EXP 150 of 150	3.8434868 ± 0.0174356	0.6238	EXP 150 of 150	0.6466042 ± 0.0160746	0.0144	EXP 150 of 150	6.5693111 ± 0.0175314	0.5057	EXP 150 of 150	919.4516 ± 0.0514	0.9996	EXP 150 of 150
18D00679	11.6 %	3.0743913 ± 0.0027572	0.9837	EXP 149 of 150	2.6544747 ± 0.0197155	0.3581	EXP 150 of 150	0.6787530 ± 0.0151597	0.0315	EXP 150 of 150	5.4118880 ± 0.0168808	0.2994	EXP 150 of 150	957.9081 ± 0.0529	0.9996	EXP 150 of 150
18D00681	12.5 %	3.3953034 ± 0.0026509	0.9876	EXP 150 of 150	2.0093205 ± 0.0170920	0.3170	EXP 150 of 150	0.7532106 ± 0.0179925	0.0706	EXP 150 of 150	4.8123248 ± 0.0161412	0.2696	EXP 150 of 150	1070.2724 ± 0.0524	0.9998	EXP 150 of 150
18D00682	13.4 %	5.2000707 ± 0.0031604	0.9927	EXP 150 of 150	2.0228941 ± 0.0163765	0.2432	EXP 150 of 150	1.1286859 ± 0.0147419	0.2029	EXP 150 of 150	5.5689691 ± 0.0160148	0.1503	EXP 150 of 150	1649.0939 ± 0.0668	0.9999	EXP 150 of 150
18D00684	14.6 %	5.7901851 ± 0.0040425	0.9907	EXP 150 of 150	1.9204622 ± 0.0188788	0.1972	EXP 150 of 150	1.2213928 ± 0.0160616	0.1044	EXP 150 of 150	5.5120311 ± 0.0150257	0.2222	EXP 150 of 150	1838.8417 ± 0.0650	0.9999	EXP 150 of 150
18D00685	16.0 %	7.2114826 ± 0.0042362	0.9934	EXP 149 of 150	2.4175124 ± 0.0172414	0.4911	EXP 149 of 150	1.5724405 ± 0.0163014	0.3270	EXP 150 of 150	6.4806346 ± 0.0163012	0.2007	EXP 150 of 150	2283.7764 ± 0.0730	0.9999	EXP 150 of 150
18D00687	17.6 %	9.0200594 ± 0.0044944	0.9952	EXP 150 of 150	3.7197748 ± 0.0179669	0.5514	EXP 150 of 150	1.9029376 ± 0.0159018	0.2083	EXP 150 of 150	8.2990857 ± 0.0164724	0.3964	EXP 150 of 150	2858.2085 ± 0.0850	0.9999	EXP 150 of 150
18D00688	19.3 %	5.1810288 ± 0.0030524	0.9927	EXP 150 of 150	2.7993766 ± 0.0180277	0.4275	EXP 150 of 150	1.3130168 ± 0.0152165	0.3676	EXP 150 of 150	5.7798132 ± 0.0154540	0.0203	EXP 150 of 150	1622.4054 ± 0.0696	0.9997	EXP 150 of 150
18D00690	21.0 %	4.1503337 ± 0.0030091	0.9893	EXP 150 of 150	2.2735581 ± 0.0183438	0.3202	EXP 150 of 150	0.8913026 ± 0.0165750	0.0342	EXP 149 of 150	4.1416274 ± 0.0157188	0.0050	EXP 150 of 150	1313.4554 ± 0.0607	0.9998	EXP 150 of 150

Project Info		Analyst	Irradiation	X-pos	Y-pos	Z/H-pos	Project	Experiment	Nmb
18D00655	1.8 %	Dan Miggins	17-OSU-05	0.00	0.00	12.04	Arctic\O-Connor (16-22)	18D00651	01
18D00657	1.9 %	Dan Miggins	17-OSU-05	0.00	0.00	12.04	Arctic\O-Connor (16-22)	18D00651	01
18D00658	2.0 %	Dan Miggins	17-OSU-05	0.00	0.00	12.04	Arctic\O-Connor (16-22)	18D00651	01
18D00660	2.2 %	Dan Miggins	17-OSU-05	0.00	0.00	12.04	Arctic\O-Connor (16-22)	18D00651	01
18D00661	2.4 %	Dan Miggins	17-OSU-05	0.00	0.00	12.04	Arctic\O-Connor (16-22)	18D00651	01
18D00663	2.7 %	Dan Miggins	17-OSU-05	0.00	0.00	12.04	Arctic\O-Connor (16-22)	18D00651	01
18D00664	3.0 %	Dan Miggins	17-OSU-05	0.00	0.00	12.04	Arctic\O-Connor (16-22)	18D00651	01
18D00666	3.4 %	Dan Miggins	17-OSU-05	0.00	0.00	12.04	Arctic\O-Connor (16-22)	18D00651	01
18D00667	3.9 %	Dan Miggins	17-OSU-05	0.00	0.00	12.04	Arctic\O-Connor (16-22)	18D00651	01
18D00669	4.5 %	Dan Miggins	17-OSU-05	0.00	0.00	12.04	Arctic\O-Connor (16-22)	18D00651	01
18D00670	5.2 %	Dan Miggins	17-OSU-05	0.00	0.00	12.04	Arctic\O-Connor (16-22)	18D00651	01
18D00672	6.0 %	Dan Miggins	17-OSU-05	0.00	0.00	12.04	Arctic\O-Connor (16-22)	18D00651	01
18D00673	6.9 %	Dan Miggins	17-OSU-05	0.00	0.00	12.04	Arctic\O-Connor (16-22)	18D00651	01
18D00675	7.9 %	Dan Miggins	17-OSU-05	0.00	0.00	12.04	Arctic\O-Connor (16-22)	18D00651	01
18D00676	9.0 %	Dan Miggins	17-OSU-05	0.00	0.00	12.04	Arctic\O-Connor (16-22)	18D00651	01
18D00678	10.3 %	Dan Miggins	17-OSU-05	0.00	0.00	12.04	Arctic\O-Connor (16-22)	18D00651	01
18D00679	11.6 %	Dan Miggins	17-OSU-05	0.00	0.00	12.04	Arctic\O-Connor (16-22)	18D00651	01
18D00681	12.5 %	Dan Miggins	17-OSU-05	0.00	0.00	12.04	Arctic\O-Connor (16-22)	18D00651	01
18D00682	13.4 %	Dan Miggins	17-OSU-05	0.00	0.00	12.04	Arctic\O-Connor (16-22)	18D00651	01
18D00684	14.6 %	Dan Miggins	17-OSU-05	0.00	0.00	12.04	Arctic\O-Connor (16-22)	18D00651	01
18D00685	16.0 %	Dan Miggins	17-OSU-05	0.00	0.00	12.04	Arctic\O-Connor (16-22)	18D00651	01
18D00687	17.6 %	Dan Miggins	17-OSU-05	0.00	0.00	12.04	Arctic\O-Connor (16-22)	18D00651	01
18D00688	19.3 %	Dan Miggins	17-OSU-05	0.00	0.00	12.04	Arctic\O-Connor (16-22)	18D00651	01
18D00690	21.0 %	Dan Miggins	17-OSU-05	0.00	0.00	12.04	Arctic\O-Connor (16-22)	18D00651	01

**Sample Parameters**		Sample	Material	Location	Standard Name	Standard (in Ma)	%1σ	Standard Reference	Standard 40Ar/39Ar	%1σ	J	%1σ	Air 40Ar/36Ar	%1σ	MDF (lin)	%1σ	Volume Ratio	Sensitivity (mol/volt)	Day	Month	Year	Hour	Min	Resist
18D00655	1.8 %	HLY0102-D55-9	Groundmass	Gakkel Ridge	FCT-NM (5B8-17)	28.201	0.082	Kuiper et al (2008)	9.61157	0.082	0.00163526	0.082	302.858	0.152	0.9939183	0.069	1	4.8E-14	6	JAN	2018	4	54	1
18D00657	1.9 %	HLY0102-D55-9	Groundmass	Gakkel Ridge	FCT-NM (5B8-17)	28.201	0.082	Kuiper et al (2008)	9.61157	0.082	0.00163526	0.082	302.858	0.152	0.9939183	0.069	1	4.8E-14	6	JAN	2018	5	14	1
18D00658	2.0 %	HLY0102-D55-9	Groundmass	Gakkel Ridge	FCT-NM (5B8-17)	28.201	0.082	Kuiper et al (2008)	9.61157	0.082	0.00163526	0.082	302.858	0.152	0.9939183	0.069	1	4.8E-14	6	JAN	2018	5	24	1
18D00660	2.2 %	HLY0102-D55-9	Groundmass	Gakkel Ridge	FCT-NM (5B8-17)	28.201	0.082	Kuiper et al (2008)	9.61157	0.082	0.00163526	0.082	302.858	0.152	0.9939183	0.069	1	4.8E-14	6	JAN	2018	5	44	1
18D00661	2.4 %	HLY0102-D55-9	Groundmass	Gakkel Ridge	FCT-NM (5B8-17)	28.201	0.082	Kuiper et al (2008)	9.61157	0.082	0.00163526	0.082	302.858	0.152	0.9939183	0.069	1	4.8E-14	6	JAN	2018	5	54	1
18D00663	2.7 %	HLY0102-D55-9	Groundmass	Gakkel Ridge	FCT-NM (5B8-17)	28.201	0.082	Kuiper et al (2008)	9.61157	0.082	0.00163526	0.082	302.858	0.152	0.9939183	0.069	1	4.8E-14	6	JAN	2018	6	14	1
18D00664	3.0 %	HLY0102-D55-9	Groundmass	Gakkel Ridge	FCT-NM (5B8-17)	28.201	0.082	Kuiper et al (2008)	9.61157	0.082	0.00163526	0.082	302.858	0.152	0.9939183	0.069	1	4.8E-14	6	JAN	2018	6	24	1
18D00666	3.4 %	HLY0102-D55-9	Groundmass	Gakkel Ridge	FCT-NM (5B8-17)	28.201	0.082	Kuiper et al (2008)	9.61157	0.082	0.00163526	0.082	302.858	0.152	0.9939183	0.069	1	4.8E-14	6	JAN	2018	6	44	1
18D00667	3.9 %	HLY0102-D55-9	Groundmass	Gakkel Ridge	FCT-NM (5B8-17)	28.201	0.082	Kuiper et al (2008)	9.61157	0.082	0.00163526	0.082	302.858	0.152	0.9939183	0.069	1	4.8E-14	6	JAN	2018	6	54	1
18D00669	4.5 %	HLY0102-D55-9	Groundmass	Gakkel Ridge	FCT-NM (5B8-17)	28.201	0.082	Kuiper et al (2008)	9.61157	0.082	0.00163526	0.082	302.858	0.152	0.9939183	0.069	1	4.8E-14	6	JAN	2018	7	15	1
18D00670	5.2 %	HLY0102-D55-9	Groundmass	Gakkel Ridge	FCT-NM (5B8-17)	28.201	0.082	Kuiper et al (2008)	9.61157	0.082	0.00163526	0.082	302.858	0.152	0.9939183	0.069	1	4.8E-14	6	JAN	2018	7	25	1
18D00672	6.0 %	HLY0102-D55-9	Groundmass	Gakkel Ridge	FCT-NM (5B8-17)	28.201	0.082	Kuiper et al (2008)	9.61157	0.082	0.00163526	0.082	302.858	0.152	0.9939183	0.069	1	4.8E-14	6	JAN	2018	7	45	1
18D00673	6.9 %	HLY0102-D55-9	Groundmass	Gakkel Ridge	FCT-NM (5B8-17)	28.201	0.082	Kuiper et al (2008)	9.61157	0.082	0.00163526	0.082	302.858	0.152	0.9939183	0.069	1	4.8E-14	6	JAN	2018	7	55	1
18D00675	7.9 %	HLY0102-D55-9	Groundmass	Gakkel Ridge	FCT-NM (5B8-17)	28.201	0.082	Kuiper et al (2008)	9.61157	0.082	0.00163526	0.082	302.858	0.152	0.9939183	0.069	1	4.8E-14	6	JAN	2018	8	15	1
18D00676	9.0 %	HLY0102-D55-9	Groundmass	Gakkel Ridge	FCT-NM (5B8-17)	28.201	0.082	Kuiper et al (2008)	9.61157	0.082	0.00163526	0.082	302.858	0.152	0.9939183	0.069	1	4.8E-14	6	JAN	2018	8	25	1
18D00678	10.3 %	HLY0102-D55-9	Groundmass	Gakkel Ridge	FCT-NM (5B8-17)	28.201	0.082	Kuiper et al (2008)	9.61157	0.082	0.00163526	0.082	302.858	0.152	0.9939183	0.069	1	4.8E-14	6	JAN	2018	8	45	1
18D00679	11.6 %	HLY0102-D55-9	Groundmass	Gakkel Ridge	FCT-NM (5B8-17)	28.201	0.082	Kuiper et al (2008)	9.61157	0.082	0.00163526	0.082	302.858	0.152	0.9939183	0.069	1	4.8E-14	6	JAN	2018	8	55	1
18D00681	12.5 %	HLY0102-D55-9	Groundmass	Gakkel Ridge	FCT-NM (5B8-17)	28.201	0.082	Kuiper et al (2008)	9.61157	0.082	0.00163526	0.082	302.858	0.152	0.9939183	0.069	1	4.8E-14	6	JAN	2018	9	15	1
18D00682	13.4 %	HLY0102-D55-9	Groundmass	Gakkel Ridge	FCT-NM (5B8-17)	28.201	0.082	Kuiper et al (2008)	9.61157	0.082	0.00163526	0.082	302.858	0.152	0.9939183	0.069	1	4.8E-14	6	JAN	2018	9	25	1
18D00684	14.6 %	HLY0102-D55-9	Groundmass	Gakkel Ridge	FCT-NM (5B8-17)	28.201	0.082	Kuiper et al (2008)	9.61157	0.082	0.00163526	0.082	302.858	0.152	0.9939183	0.069	1	4.8E-14	6	JAN	2018	9	45	1
18D00685	16.0 %	HLY0102-D55-9	Groundmass	Gakkel Ridge	FCT-NM (5B8-17)	28.201	0.082	Kuiper et al (2008)	9.61157	0.082	0.00163526	0.082	302.858	0.152	0.9939183	0.069	1	4.8E-14	6	JAN	2018	9	56	1
18D00687	17.6 %	HLY0102-D55-9	Groundmass	Gakkel Ridge	FCT-NM (5B8-17)	28.201	0.082	Kuiper et al (2008)	9.61157	0.082	0.00163526	0.082	302.858	0.152	0.9939183	0.069	1	4.8E-14	6	JAN	2018	10	16	1
18D00688	19.3 %	HLY0102-D55-9	Groundmass	Gakkel Ridge	FCT-NM (5B8-17)	28.201	0.082	Kuiper et al (2008)	9.61157	0.082	0.00163526	0.082	302.858	0.152	0.9939183	0.069	1	4.8E-14	6	JAN	2018	10	26	1
18D00690	21.0 %	HLY0102-D55-9	Groundmass	Gakkel Ridge	FCT-NM (5B8-17)	28.201	0.082	Kuiper et al (2008)	9.61157	0.082	0.00163526	0.082	302.858	0.152	0.9939183	0.069	1	4.8E-14	6	JAN	2018	10	46	1

Irradiation Constants		40/36(a)	%1σ	40/36(c)	%1σ	38/36(a)	%1σ	38/36(c)	%1σ	39/37(ca)	%1σ	38/37(ca)	%1σ	36/37(ca)	%1σ	40/39(k)	%1σ	38/39(k)	%1σ	36/38(cl)	%1σ	K/Ca	%1σ	K/Cl	%1σ	Ca/Cl	%1σ
18D00655	1.8 %	295.5	0.237	0.018	35	0.1869	0	1.493	3	0.000643	0.92	0.00018	9.63	0.00027	0.17	0.000607	9.65	0.012077	0.09	0	0	0.43	0	0	0	0	0
18D00657	1.9 %	295.5	0.237	0.018	35	0.1869	0	1.493	3	0.000643	0.92	0.00018	9.63	0.00027	0.17	0.000607	9.65	0.012077	0.09	0	0	0.43	0	0	0	0	0
18D00658	2.0 %	295.5	0.237	0.018	35	0.1869	0	1.493	3	0.000643	0.92	0.00018	9.63	0.00027	0.17	0.000607	9.65	0.012077	0.09	0	0	0.43	0	0	0	0	0
18D00660	2.2 %	295.5	0.237	0.018	35	0.1869	0	1.493	3	0.000643	0.92	0.00018	9.63	0.00027	0.17	0.000607	9.65	0.012077	0.09	0	0	0.43	0	0	0	0	0
18D00661	2.4 %	295.5	0.237	0.018	35	0.1869	0	1.493	3	0.000643	0.92	0.00018	9.63	0.00027	0.17	0.000607	9.65	0.012077	0.09	0	0	0.43	0	0	0	0	0
18D00663	2.7 %	295.5	0.237	0.018	35	0.1869	0	1.493	3	0.000643	0.92	0.00018	9.63	0.00027	0.17	0.000607	9.65	0.012077	0.09	0	0	0.43	0	0	0	0	0
18D00664	3.0 %	295.5	0.237	0.018	35	0.1869	0	1.493	3	0.000643	0.92	0.00018	9.63	0.00027	0.17	0.000607	9.65	0.012077	0.09	0	0	0.43	0	0	0	0	0
18D00666	3.4 %	295.5	0.237	0.018	35	0.1869	0	1.493	3	0.000643	0.92	0.00018	9.63	0.00027	0.17	0.000607	9.65	0.012077	0.09	0	0	0.43	0	0	0	0	0
18D00667	3.9 %	295.5	0.237	0.018	35	0.1869	0	1.493	3	0.000643	0.92	0.00018	9.63	0.00027	0.17	0.000607	9.65	0.012077	0.09	0	0	0.43	0	0	0	0	0
18D00669	4.5 %	295.5	0.237	0.018	35	0.1869	0	1.493	3	0.000643	0.92	0.00018	9.63	0.00027	0.17	0.000607	9.65	0.012077	0.09	0	0	0.43	0	0	0	0	0
18D00670	5.2 %	295.5	0.237	0.018	35	0.1869	0	1.493	3	0.000643	0.92	0.00018	9.63	0.00027	0.17	0.000607	9.65	0.012077	0.09	0	0	0.43	0	0	0	0	0
18D00672	6.0 %	295.5	0.237	0.018	35	0.1869	0	1.493	3	0.000643	0.92	0.00018	9.63	0.00027	0.17	0.000607	9.65	0.012077	0.09	0	0	0.43	0	0	0	0	0
18D00673	6.9 %	295.5	0.237	0.018	35	0.1869	0	1.493	3	0.000643	0.92	0.00018	9.63	0.00027	0.17	0.000607	9.65	0.012077	0.09	0	0	0.43	0	0	0	0	0
18D00675	7.9 %	295.5	0.237	0.018	35	0.1869	0	1.493	3	0.000643	0.92	0.00018	9.63	0.00027	0.17	0.000607	9.65	0.012077	0.09	0	0	0.43	0	0	0	0	0
18D00676	9.0 %	295.5	0.237	0.018	35	0.1869	0	1.493	3	0.000643	0.92	0.00018	9.63	0.00027	0.17	0.000607	9.65	0.012077	0.09	0	0	0.43	0	0	0	0	0
18D00678	10.3 %	295.5	0.237	0.018	35	0.1869	0	1.493	3	0.000643	0.92	0.00018	9.63	0.00027	0.17	0.000607	9.65	0.012077	0.09	0	0	0.43	0	0	0	0	0
18D00679	11.6 %	295.5	0.237	0.018	35	0.1869	0	1.493	3	0.000643	0.92	0.00018	9.63	0.00027	0.17	0.000607	9.65	0.012077	0.09	0	0	0.43	0	0	0	0	0
18D00681	12.5 %	295.5	0.237	0.018	35	0.1869	0	1.493	3	0.000643	0.92	0.00018	9.63	0.00027	0.17	0.000607	9.65	0.012077	0.09	0	0	0.43	0	0	0	0	0
18D00682	13.4 %	295.5	0.237	0.018	35	0.1869	0	1.493	3	0.000643	0.92	0.00018	9.63	0.00027	0.17	0.000607	9.65	0.012077	0.09	0	0	0.43	0	0	0	0	0
18D00684	14.6 %	295.5	0.237	0.018	35	0.1869	0	1.493	3	0.000643	0.92	0.00018	9.63	0.00027	0.17	0.000607	9.65	0.012077	0.09	0	0	0.43	0	0	0	0	0
18D00685	16.0 %	295.5	0.237	0.018	35	0.1869	0	1.493	3	0.000643	0.92	0.00018	9.63	0.00027	0.17	0.000607	9.65	0.012077	0.09	0	0	0.43	0	0	0	0	0
18D00687	17.6 %	295.5	0.237	0.018	35	0.1869	0	1.493	3	0.000643	0.92	0.00018	9.63	0.00027	0.17	0.000607	9.65	0.012077	0.09	0	0	0.43	0	0	0	0	0
18D00688	19.3 %	295.5	0.237	0.018	35	0.1869	0	1.493	3	0.000643	0.92	0.00018	9.63	0.00027	0.17	0.000607	9.65	0.012077	0.09	0	0	0.43	0	0	0	0	0
18D00690	21.0 %	295.5	0.237	0.018	35	0.1869	0	1.493	3	0.000643	0.92	0.00018	9.63	0.00027	0.17	0.000607	9.65	0.012077	0.09	0	0	0.43	0	0	0	0	0

18D00651.AGE >>> HLY0102-D55-9 >>> ARCTIC | O-CONNOR (16-22) PROJECT

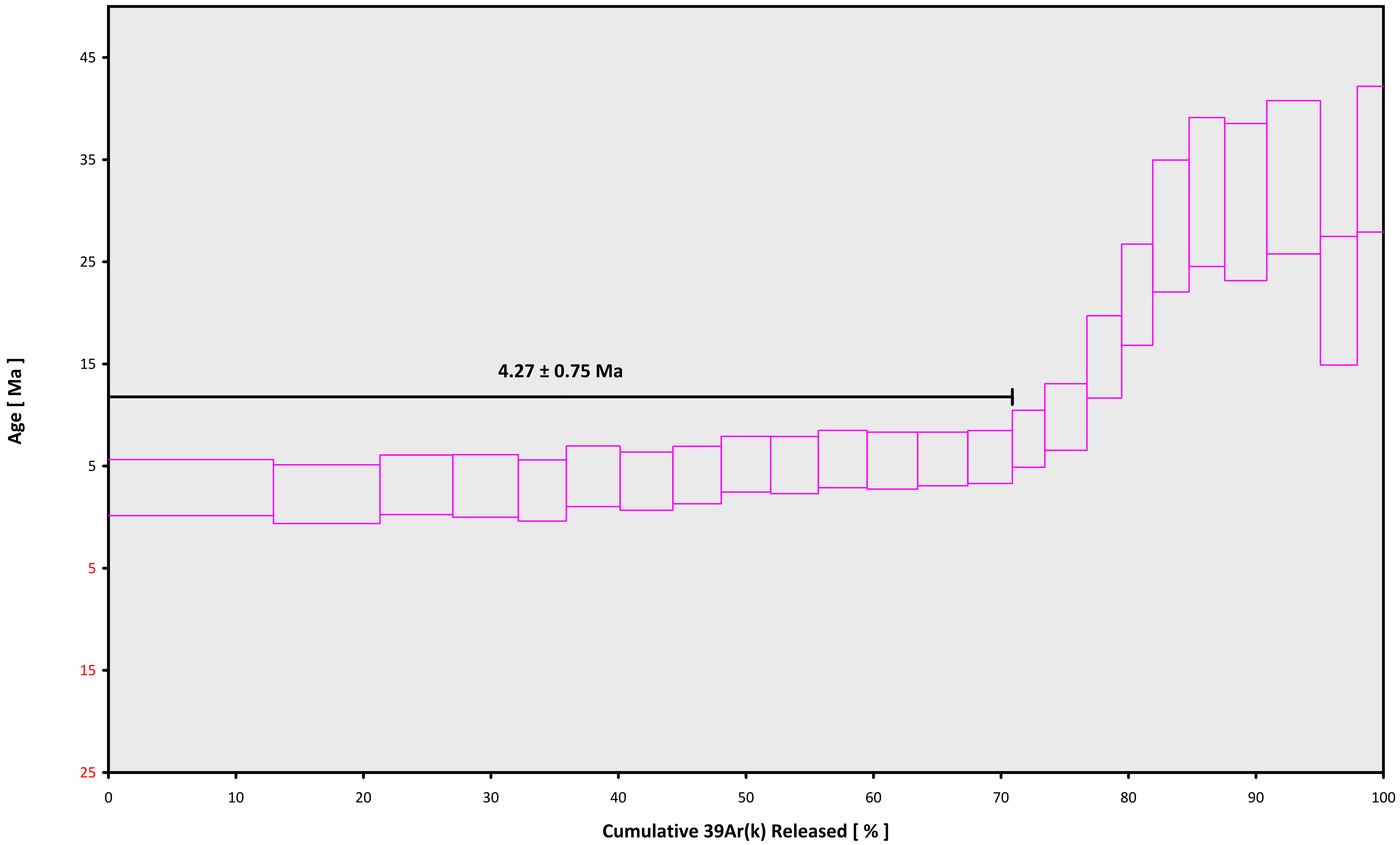

Ar-Ages in Ma

WEIGHTED PLATEAU
4.27 ± 0.75
TOTAL FUSION
9.69 ± 0.83
NORMAL ISOCHRON
21.76 ± 9.76
INVERSE ISOCHRON
21.75 ± 8.38

MSWD (PROBABILITY)
0.85 (60%)

Sample Info

Groundmass
Gakkel Ridge
Dan Miggins

IRR = 17-OSU-05 (5B8-17)
J = 0.00163526 ± 0.00000134

18D00651.AGE >>> HLY0102-D55-9 >>> ARCTIC | O-CONNOR (16-22) PROJECT

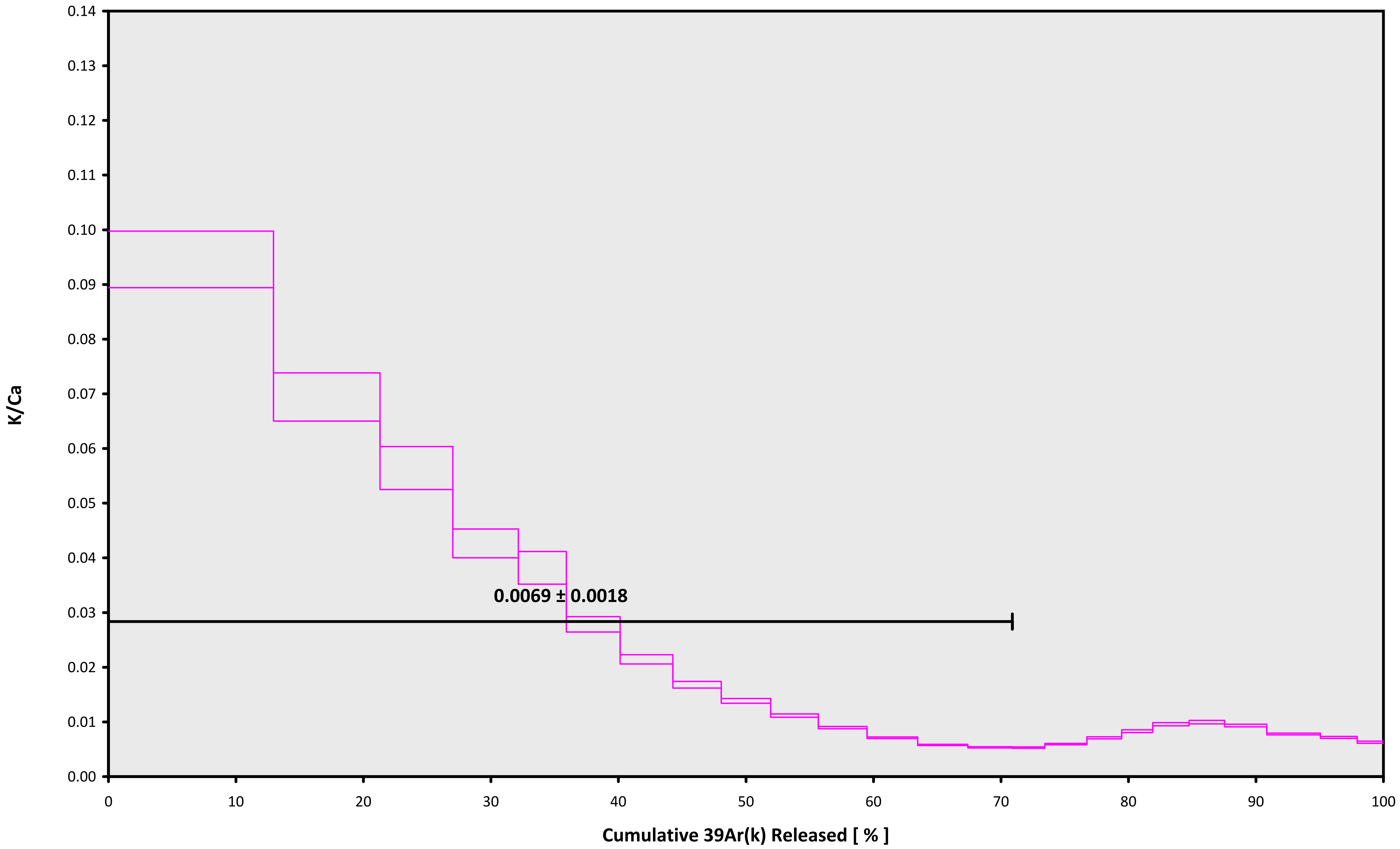

Ar-Ages in Ma

WEIGHTED PLATEAU
4.27 ± 0.75
TOTAL FUSION
9.69 ± 0.83
NORMAL ISOCHRON
21.76 ± 9.76
INVERSE ISOCHRON
21.75 ± 8.38

Sample Info

Groundmass
Gakkel Ridge
Dan Miggins

IRR = 17-OSU-05 (5B8-17)
J = 0.00163526 ± 0.00000134

18D00651.AGE >>> HLY0102-D55-9 >>> ARCTIC | O-CONNOR (16-22) PROJECT

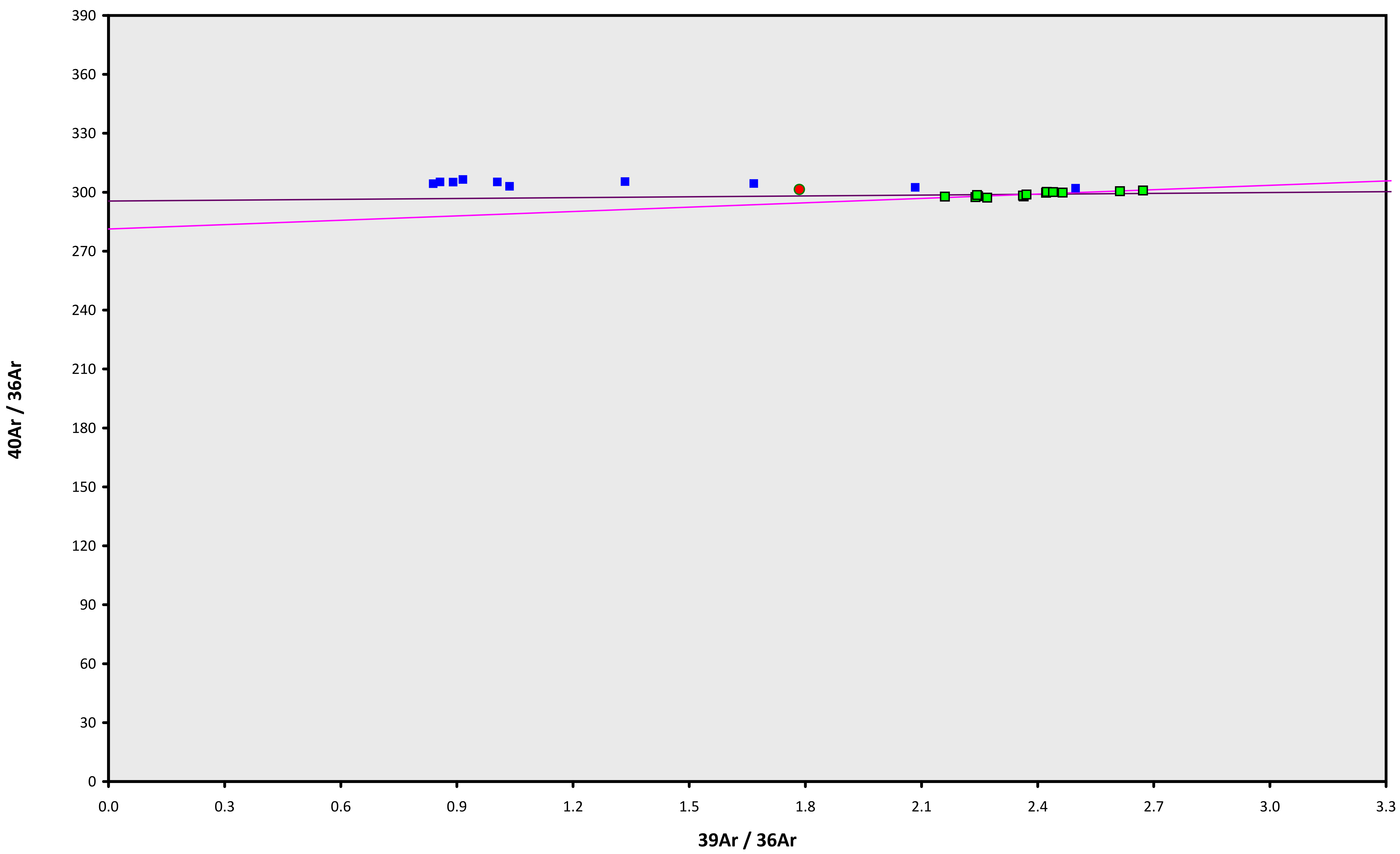

Ar-Ages in Ma

WEIGHTED PLATEAU
4.27 ± 0.75
TOTAL FUSION
9.69 ± 0.83
NORMAL ISOCHRON
21.76 ± 9.76
INVERSE ISOCHRON
21.75 ± 8.38

MSWD (PROBABILITY)
0.52 (90%)

40AR/36AR INTERCEPT
281.3 ± 7.9

Sample Info

Groundmass
Gakkel Ridge
Dan Miggins

IRR = 17-OSU-05 (5B8-17)
J = 0.00163526 ± 0.00000134

18D00651.AGE >>> HLY0102-D55-9 >>> ARCTIC | O-CONNOR (16-22) PROJECT

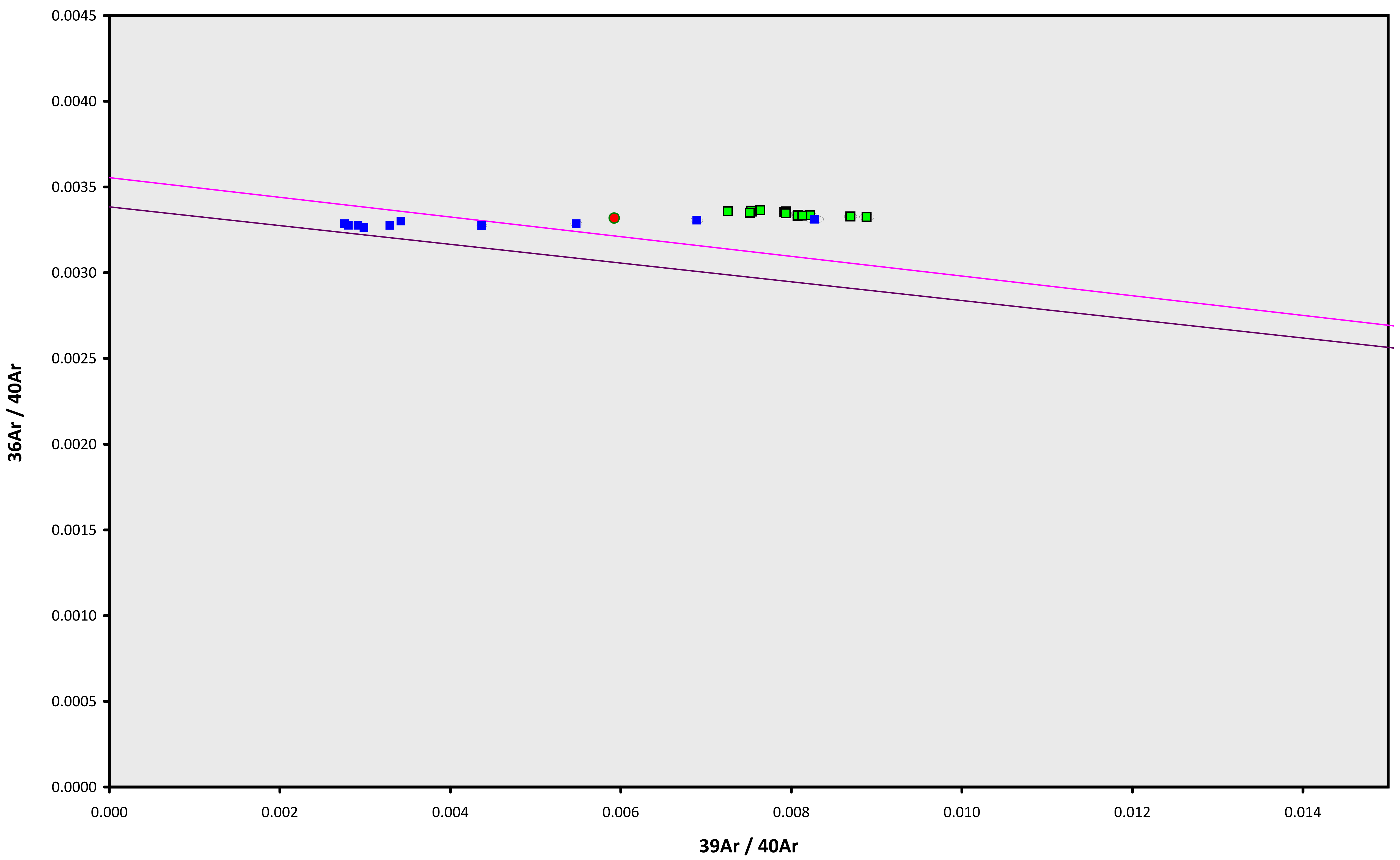

Ar-Ages in Ma

WEIGHTED PLATEAU
4.27 ± 0.75
TOTAL FUSION
9.69 ± 0.83
NORMAL ISOCHRON
21.76 ± 9.76
INVERSE ISOCHRON
21.75 ± 8.38

MSWD (PROBABILITY)
0.52 (90%)

SPREADING FACTOR
1.2%

40AR/36AR INTERCEPT
281.3 ± 7.9

Sample Info

Groundmass
Gakkel Ridge
Dan Miggins

IRR = 17-OSU-05 (5B8-17)
J = 0.00163526 ± 0.00000134

Relative Abundances			36Ar [fA]	%1σ	37Ar [fA]	%1σ	38Ar [fA]	%1σ	39Ar [fA]	%1σ	40Ar [fA]	%1σ	40(r)/39(k) ± 2σ	Age ± 2σ (Ma)	40Ar(r) (%)	39Ar(k) (%)	K/Ca ± 2σ
18D00548	1.8 %	✓	2.770800	0.307	82.7638	3.548	0.602604	4.147	6.62785	0.346	815.061	0.047	0.44058 ± 0.97185	1.28 ± 2.83	0.36	6.61	0.0342 ± 0.0024
18D00550	1.9 %	✓	1.523253	0.326	39.6391	7.691	0.304261	7.441	3.17371	0.689	447.123	0.085	0.05257 ± 1.18587	0.15 ± 3.45	0.04	3.17	0.0342 ± 0.0053
18D00551	2.0 %	✓	1.231627	0.350	35.8116	8.442	0.239664	10.470	2.54232	0.897	361.099	0.106	0.00468 ± 1.26895	0.01 ± 3.70	0.00	2.53	0.0303 ± 0.0051
18D00553	2.2 %	✓	1.226199	0.351	37.9556	8.096	0.250465	9.439	2.50174	0.888	361.243	0.105	0.77979 ± 1.28952	2.27 ± 3.75	0.53	2.49	0.0281 ± 0.0046
18D00554	2.4 %	✓	2.316621	0.313	118.2159	2.646	0.510635	4.418	5.63555	0.426	677.927	0.056	0.50440 ± 0.97576	1.47 ± 2.84	0.41	5.59	0.0202 ± 0.0011
18D00556	2.7 %	✓	2.786306	0.306	179.4611	1.771	0.610111	3.889	7.30156	0.312	812.090	0.047	0.42671 ± 0.88958	1.24 ± 2.59	0.38	7.22	0.0172 ± 0.0006
18D00557	3.0 %	✓	2.180691	0.311	159.4414	2.041	0.474518	4.988	5.86407	0.377	633.011	0.060	0.23409 ± 0.88279	0.68 ± 2.57	0.21	5.79	0.0155 ± 0.0006
18D00559	3.4 %	✓	2.023362	0.312	158.6105	1.993	0.459017	5.101	5.20567	0.459	588.238	0.065	0.58783 ± 0.92873	1.71 ± 2.70	0.51	5.13	0.0138 ± 0.0006
18D00560	3.9 %	✓	2.187095	0.307	191.4403	1.640	0.483117	4.923	5.67840	0.388	632.139	0.060	0.20528 ± 0.90999	0.60 ± 2.65	0.18	5.59	0.0125 ± 0.0004
18D00562	4.5 %	✓	1.431598	0.337	120.9473	2.480	0.325295	7.066	3.56669	0.595	413.740	0.092	0.10353 ± 1.02374	0.30 ± 2.98	0.09	3.51	0.0124 ± 0.0006
18D00563	5.2 %	✓	1.472751	0.334	136.2032	2.291	0.365281	6.196	3.71795	0.613	424.572	0.090	0.07006 ± 1.00583	0.20 ± 2.93	0.06	3.65	0.0115 ± 0.0005
18D00565	6.0 %	✓	1.560487	0.327	162.0265	2.006	0.370852	6.339	4.24943	0.529	447.985	0.085	0.04800 ± 0.91717	0.14 ± 2.67	0.04	4.17	0.0110 ± 0.0005
18D00566	6.9 %	✓	2.363936	0.308	292.9232	1.194	0.554194	4.063	7.15260	0.316	677.326	0.056	0.31230 ± 0.78253	0.91 ± 2.28	0.32	7.00	0.0102 ± 0.0003
18D00568	7.9 %	✓	2.807016	0.304	263.7287	1.290	0.617375	3.910	6.07394	0.356	807.920	0.047	0.08326 ± 1.08441	0.24 ± 3.16	0.06	5.94	0.0096 ± 0.0003
18D00569	9.0 %	✓	3.845218	0.295	245.1423	1.424	0.824809	2.943	5.68095	0.384	1122.570	0.034	1.06570 ± 1.55747	3.10 ± 4.53	0.52	5.55	0.0097 ± 0.0003
18D00571	10.3 %	✓	9.814032	0.286	334.2362	1.140	1.970378	1.271	7.64668	0.297	2890.887	0.013	2.35919 ± 2.89233	6.86 ± 8.39	0.61	7.47	0.0096 ± 0.0002
18D00572	11.6 %	✓	6.408277	0.290	186.8575	1.710	1.285290	1.798	4.57433	0.486	1883.882	0.020	1.15825 ± 3.17985	3.37 ± 9.25	0.27	4.48	0.0103 ± 0.0004
18D00574	12.5 %	✓	5.562815	0.291	139.7415	2.307	1.101105	2.090	3.43109	0.658	1633.451	0.024	0.23906 ± 3.69085	0.70 ± 10.75	0.05	3.36	0.0103 ± 0.0005
18D00575	13.4 %	✓	1.409501	0.338	55.2964	5.181	0.274265	8.955	1.30567	1.616	413.751	0.092	1.30604 ± 2.78829	3.80 ± 8.11	0.40	1.28	0.0099 ± 0.0011
18D00577	14.6 %	✓	1.986953	0.317	68.8564	4.456	0.390772	6.102	1.46570	1.498	584.350	0.065	1.90227 ± 3.31884	5.53 ± 9.64	0.46	1.43	0.0089 ± 0.0008
18D00578	16.0 %	✓	0.379069	0.618	19.4558	14.736	0.055211	41.453	0.35762	6.559	111.256	0.342	2.30481 ± 5.01035	6.70 ± 14.54	0.71	0.35	0.0076 ± 0.0025
18D00580	17.6 %	✓	3.108043	0.302	145.8356	2.107	0.614070	3.903	2.38157	0.942	912.699	0.042	2.58714 ± 3.09607	7.52 ± 8.98	0.65	2.30	0.0067 ± 0.0003
18D00581	19.3 %	✓	1.773079	0.322	93.3219	3.156	0.354820	6.930	1.52039	1.397	519.158	0.074	1.82547 ± 2.92084	5.31 ± 8.48	0.51	1.47	0.0067 ± 0.0005
18D00583	21.0 %	✓	4.576329	0.292	299.4244	1.205	0.929597	2.568	4.10628	0.504	1339.420	0.029	2.81784 ± 2.59316	8.19 ± 7.52	0.82	3.93	0.0056 ± 0.0001
		Σ	66.745057	0.075	3567.3360	0.438	13.967706	0.831	101.76176	0.108	19510.897	0.010					

Information on Analysis and Constants Used in Calculations

Project = **O-CONNOR (16-22)**
Sample = **PS59-297-37**
Material = **Groundmass**
Location = **Gakkel Ridge**
Region = **Artic Ocean**
Analyst = **Dan Miggins**
Irradiation = **17-OSU-05 (5B17-17)**
Position = **X: 0 | Y: 0 | Z/H: 24.94839 mm**
FCT-NM Age = **28.201 ± 0.023 Ma**
FCT-NM Reference = **Kuiper et al (2008)**
FCT-NM 40Ar/39Ar Ratio = **9.75664 ± 0.00790**
FCT-NM J-value = **0.00161094 ± 0.00000130**
Air Shot 40Ar/36Ar = **302.4190 ± 0.4597**
Air Shot MDF = **0.99427289 ± 0.00068867 (LIN)**
Experiment Type = **Incremental Heating**
Extraction Method = **Bulk Laser Heating**
Heating = **77 sec**
Isolation = **3.00 min**
Instrument = **ARGUS-VI-D**
Preferred Age = **Plateau Age**
Age Classification = **Crystallization Age**
IGSN = Undefined
Rock Class = Undefined
Lithology = **Basaltic Lava**
Lat-Lon = Undefined - Undefined

Age Equations = **Min et al. (2000)**
Negative Intensities = **Allowed**
Collector Calibrations = **36Ar**
Decay 40K = **5.530 ± 0.048 E-10 1/a**
Decay 39Ar = **2.940 ± 0.016 E-07 1/h**
Decay 37Ar = **8.230 ± 0.012 E-04 1/h**
Decay 36Cl = **2.257 ± 0.015 E-06 1/a**
Decay 40K(EC,β⁺) = **0.580 ± 0.009 E-10 1/a**
Decay 40K(β⁻) = **4.950 ± 0.043 E-10 1/a**
Atmospheric 40/36(a) = **295.50 ± 0.70**
Atmospheric 38/36(a) = **0.1869**
Production 39/37(ca) = **0.0006425 ± 0.0000059**
Production 38/37(ca) = **0.0001800 ± 0.0000173**
Production 36/37(ca) = **0.0002703 ± 0.0000005**
Production 40/39(k) = **0.000607 ± 0.000059**
Production 38/39(k) = **0.012077 ± 0.000011**
Production 36/38(cl) = **262.80 ± 1.71**
Scaling Ratio K/Ca = **0.430**
Abundance Ratio 40K/K = **1.1700 ± 0.0100 E-04**
Atomic Weight K = **39.0983 ± 0.0001 g**

Results

Results	40(a)/36(a) ± 2σ	40(r)/39(k) ± 2σ	Age ± 2σ (Ma)	MSWD	39Ar(k) (%,n)	K/Ca ± 2σ
**Age Plateau**		0.37810 ± 0.25075 ± 66.32%	**1.10 ± 0.73 ± 66.30%**	0.66 / 89%	100.00 / 24	0.0089 ± 0.0012
		Full External Error	± 0.73	1.59	2σ Confidence Limit	
		Analytical Error	± 0.73	1.0000	Error Magnification	
**Total Fusion Age**		0.73003 ± 0.37997 ± 52.05%	**2.13 ± 1.11 ± 52.02%**		24	0.0120 ± 0.0001
		Full External Error	± 1.11			
		Analytical Error	± 1.11			
**Normal Isochron**	297.16 ± 0.93 ± 0.31%	0.37913 ± 0.47314 ± 124.80%	**1.10 ± 1.38 ± 124.83%**	0.48 / 98%	100.00 / 24	
		Full External Error	± 1.38	1.60	2σ Confidence Limit	
		Analytical Error	± 1.38	1.0000	Error Magnification	
				3	Number of Iterations	
				0.0000001720	Convergence	
**Inverse Isochron** Clustered Points	297.16 ± 0.93 ± 0.31%	0.38037 ± 0.21075 ± 55.41%	**1.11 ± 0.61 ± 55.42%**	0.48 / 98%	100.00 / 24	
		Full External Error	± 0.61	1.60	2σ Confidence Limit	
		Analytical Error	± 0.61	1.0000	Error Magnification	
				3	Number of Iterations	
				0.0001137307	Convergence	
				0%	Spreading Factor	

Notes
Mostly atmospheric

**Incremental Heating**			36Ar(a) [fA]	37Ar(ca) [fA]	38Ar(cl) [fA]	39Ar(k) [fA]	40Ar(r) [fA]	Age ± 2σ (Ma)	40Ar(r) (%)	39Ar(k) (%)	K/Ca ± 2σ
18D00548	1.8 %	✓	2.748429	82.7638	0.0000000	6.574679	2.896651	1.28 ± 2.83	0.36	6.61	0.0342 ± 0.0024
18D00550	1.9 %	✓	1.512539	39.6391	0.0000000	3.148243	0.165508	0.15 ± 3.45	0.04	3.17	0.0342 ± 0.0053
18D00551	2.0 %	✓	1.221947	35.8116	0.0000000	2.519310	0.011798	0.01 ± 3.70	0.00	2.53	0.0303 ± 0.0051
18D00553	2.2 %	✓	1.215940	37.9556	0.0000000	2.477352	1.931802	2.27 ± 3.75	0.53	2.49	0.0281 ± 0.0046
18D00554	2.4 %	✓	2.284667	118.2159	0.0000000	5.559594	2.804247	1.47 ± 2.84	0.41	5.59	0.0202 ± 0.0011
18D00556	2.7 %	✓	2.737798	179.4611	0.0000000	7.186260	3.066465	1.24 ± 2.59	0.38	7.22	0.0172 ± 0.0006
18D00557	3.0 %	✓	2.137594	159.4414	0.0000000	5.761626	1.348766	0.68 ± 2.57	0.21	5.79	0.0155 ± 0.0006
18D00559	3.4 %	✓	1.980489	158.6105	0.0000000	5.103766	3.000141	1.71 ± 2.70	0.51	5.13	0.0138 ± 0.0006
18D00560	3.9 %	✓	2.135349	191.4403	0.0000000	5.555404	1.140439	0.60 ± 2.65	0.18	5.59	0.0125 ± 0.0004
18D00562	4.5 %	✓	1.398906	120.9473	0.0000000	3.488983	0.361214	0.30 ± 2.98	0.09	3.51	0.0124 ± 0.0006
18D00563	5.2 %	✓	1.435924	136.2032	0.0285457	3.630438	0.254345	0.20 ± 2.93	0.06	3.65	0.0115 ± 0.0005
18D00565	6.0 %	✓	1.516688	162.0265	0.0081554	4.145326	0.198960	0.14 ± 2.67	0.04	4.17	0.0110 ± 0.0005
18D00566	6.9 %	✓	2.284759	292.9232	0.0000000	6.964398	2.174986	0.91 ± 2.28	0.32	7.00	0.0102 ± 0.0003
18D00568	7.9 %	✓	2.735730	263.7287	0.0000000	5.904492	0.491580	0.24 ± 3.16	0.06	5.94	0.0096 ± 0.0003
18D00569	9.0 %	✓	3.778953	245.1423	0.0076899	5.523443	5.886343	3.10 ± 4.53	0.52	5.55	0.0097 ± 0.0003
18D00571	10.3 %	✓	9.723687	334.2362	0.0031028	7.431932	17.533310	6.86 ± 8.39	0.61	7.47	0.0096 ± 0.0002
18D00572	11.6 %	✓	6.357765	186.8575	0.0095948	4.454272	5.159161	3.37 ± 9.25	0.27	4.48	0.0103 ± 0.0004
18D00574	12.5 %	✓	5.525041	139.7415	0.0029680	3.341305	0.798772	0.70 ± 10.75	0.05	3.36	0.0103 ± 0.0005
18D00575	13.4 %	✓	1.394555	55.2964	0.0000000	1.270144	1.658863	3.80 ± 8.11	0.40	1.28	0.0099 ± 0.0011
18D00577	14.6 %	✓	1.968341	68.8564	0.0000000	1.421464	2.704009	5.53 ± 9.64	0.46	1.43	0.0089 ± 0.0008
18D00578	16.0 %	✓	0.373810	19.4558	0.0000000	0.345118	0.795430	6.70 ± 14.54	0.71	0.35	0.0076 ± 0.0025
18D00580	17.6 %	✓	3.068623	145.8356	0.0000000	2.287871	5.919046	7.52 ± 8.98	0.65	2.30	0.0067 ± 0.0003
18D00581	19.3 %	✓	1.747854	93.3219	0.0000000	1.460434	2.665983	5.31 ± 8.48	0.51	1.47	0.0067 ± 0.0005
18D00583	21.0 %	✓	4.495394	299.4244	0.0000000	3.913896	11.028718	8.19 ± 7.52	0.82	3.93	0.0056 ± 0.0001
		Σ	65.780783	3567.3360	0.0600567	99.469751	72.615456				

Information on Analysis

Project = **O-CONNOR (16-22)**
Sample = **PS59-297-37**
Material = **Groundmass**
Location = **Gakkel Ridge**
Region = **Artic Ocean**
Analyst = **Dan Miggins**
Irradiation = **17-OSU-05 (5B17-17)**
J = **0.00161094 ± 0.00000130**
FCT-NM = **28.201 ± 0.023 Ma**

**Results**	40(r)/39(k) ± 2σ		Age ± 2σ (Ma)		MSWD	39Ar(k) (%,n)	K/Ca ± 2σ
**Age Plateau**	0.37810	± 0.25075<br>± 66.32%	**1.10**	**± 0.73**<br>**± 66.30%**	0.66<br>89%	100.00<br>24	0.0089 ± 0.0012
		Full External Error		± 0.73	1.59	2σ Confidence Limit	
		Analytical Error		± 0.73	1.0000	Error Magnification	
**Total Fusion Age**	0.73003	± 0.37997<br>± 52.05%	**2.13**	**± 1.11**<br>**± 52.02%**		24	0.0120 ± 0.0001
		Full External Error		± 1.11			
		Analytical Error		± 1.11			

Normal Isochron			39(k)/36(a) ± 2σ	40(a+r)/36(a) ± 2σ	r.i.
18D00548	1.8 %	✓	2.39 ± 0.02	296.55 ± 1.86	0.6557
18D00550	1.9 %	✓	2.08 ± 0.03	295.61 ± 2.03	0.4177
18D00551	2.0 %	✓	2.06 ± 0.04	295.51 ± 2.21	0.3523
18D00553	2.2 %	✓	2.04 ± 0.04	297.09 ± 2.23	0.3572
18D00554	2.4 %	✓	2.43 ± 0.03	296.73 ± 1.93	0.5839
18D00556	2.7 %	✓	2.62 ± 0.02	296.62 ± 1.88	0.6926
18D00557	3.0 %	✓	2.70 ± 0.03	296.13 ± 1.93	0.6270
18D00559	3.4 %	✓	2.58 ± 0.03	297.01 ± 1.95	0.5538
18D00560	3.9 %	✓	2.60 ± 0.03	296.03 ± 1.91	0.6117
18D00562	4.5 %	✓	2.49 ± 0.04	295.76 ± 2.14	0.4801
18D00563	5.2 %	✓	2.53 ± 0.04	295.68 ± 2.12	0.4678
18D00565	6.0 %	✓	2.73 ± 0.04	295.37 ± 2.08	0.5150
18D00566	6.9 %	✓	3.05 ± 0.03	296.45 ± 1.94	0.6905
18D00568	7.9 %	✓	2.16 ± 0.02	295.32 ± 1.87	0.6400
18D00569	9.0 %	✓	1.46 ± 0.01	297.06 ± 1.80	0.6004
18D00571	10.3 %	✓	0.76 ± 0.01	297.30 ± 1.72	0.6833
18D00572	11.6 %	✓	0.70 ± 0.01	296.31 ± 1.74	0.5023
18D00574	12.5 %	✓	0.60 ± 0.01	295.64 ± 1.74	0.3948
18D00575	13.4 %	✓	0.91 ± 0.03	296.69 ± 2.13	0.1964
18D00577	14.6 %	✓	0.72 ± 0.02	296.87 ± 1.95	0.1994
18D00578	16.0 %	✓	0.92 ± 0.13	297.63 ± 4.42	0.0856
18D00580	17.6 %	✓	0.75 ± 0.02	297.43 ± 1.85	0.2952
18D00581	19.3 %	✓	0.84 ± 0.03	297.03 ± 2.01	0.2150
18D00583	21.0 %	✓	0.87 ± 0.01	297.95 ± 1.79	0.4850

Results	40(a)/36(a) ± 2σ		40(r)/39(k)	± 2σ	Age ± 2σ (Ma)		MSWD
Normal Isochron	**297.16**	**± 0.93**<br>**± 0.31%**	0.37913	± 0.47314<br>± 124.80%	**1.10**	**± 1.38**<br>**± 124.83%**	0.48<br>98%
				Full External Error		± 1.38	
				Analytical Error		± 1.38	

Statistics				
	2σ Confidence Limit	1.60	Convergence	0.000000172016
	Error Magnification	1.0000	Number of Iterations	3
	Number of Data Points	24	Calculated Line	Weighted York-2

**Inverse Isochron**			**39(k)/40(a+r) ± 2σ**	**36(a)/40(a+r) ± 2σ**	**r.i.**
18D00548	1.8 %	✓	0.0080665 ± 0.0000570	0.00337207 ± 0.00002117	0.0199
18D00550	1.9 %	✓	0.0070411 ± 0.0000989	0.00338284 ± 0.00002327	0.0301
18D00551	2.0 %	✓	0.0069768 ± 0.0001276	0.00338398 ± 0.00002530	0.0326
18D00553	2.2 %	✓	0.0068579 ± 0.0001243	0.00336600 ± 0.00002531	0.0326
18D00554	2.4 %	✓	0.0082009 ± 0.0000718	0.00337010 ± 0.00002186	0.0224
18D00556	2.7 %	✓	0.0088491 ± 0.0000570	0.00337132 ± 0.00002131	0.0218
18D00557	3.0 %	✓	0.0091020 ± 0.0000711	0.00337688 ± 0.00002198	0.0286
18D00559	3.4 %	✓	0.0086764 ± 0.0000823	0.00336684 ± 0.00002211	0.0270
18D00560	3.9 %	✓	0.0087883 ± 0.0000709	0.00337799 ± 0.00002182	0.0280
18D00562	4.5 %	✓	0.0084328 ± 0.0001042	0.00338114 ± 0.00002443	0.0380
18D00563	5.2 %	✓	0.0085509 ± 0.0001088	0.00338207 ± 0.00002429	0.0353
18D00565	6.0 %	✓	0.0092533 ± 0.0001021	0.00338560 ± 0.00002382	0.0373
18D00566	6.9 %	✓	0.0102823 ± 0.0000683	0.00337323 ± 0.00002204	0.0293
18D00568	7.9 %	✓	0.0073083 ± 0.0000544	0.00338615 ± 0.00002149	0.0189
18D00569	9.0 %	✓	0.0049204 ± 0.0000393	0.00336635 ± 0.00002044	0.0096
18D00571	10.3 %	✓	0.0025708 ± 0.0000159	0.00336357 ± 0.00001947	0.0020
18D00572	11.6 %	✓	0.0023644 ± 0.0000237	0.00337483 ± 0.00001978	0.0029
18D00574	12.5 %	✓	0.0020456 ± 0.0000278	0.00338244 ± 0.00001989	0.0028
18D00575	13.4 %	✓	0.0030698 ± 0.0001025	0.00337053 ± 0.00002415	0.0142
18D00577	14.6 %	✓	0.0024326 ± 0.0000755	0.00336844 ± 0.00002216	0.0084
18D00578	16.0 %	✓	0.0031020 ± 0.0004235	0.00335990 ± 0.00004994	0.0230
18D00580	17.6 %	✓	0.0025067 ± 0.0000494	0.00336215 ± 0.00002087	0.0057
18D00581	19.3 %	✓	0.0028131 ± 0.0000823	0.00336672 ± 0.00002276	0.0109
18D00583	21.0 %	✓	0.0029221 ± 0.0000313	0.00335623 ± 0.00002011	0.0051

**Results**	**40(a)/36(a) ± 2σ**		**40(r)/39(k) ± 2σ**		**Age ± 2σ (Ma)**		**MSWD**
**Inverse Isochron**<br>Clustered Points	**297.16**	**± 0.93**<br>**± 0.31%**	0.38037	± 0.21075<br>± 55.41%	**1.11**	**± 0.61**<br>**± 55.42%**	0.48<br>98%
					Full External Error	± 0.61	
					Analytical Error	± 0.61	

**Statistics**				
2σ Confidence Limit	1.60	Convergence	0.0001137307
Error Magnification	1.0000	Number of Iterations	3
Number of Data Points	24	Calculated Line	Weighted York-2
Spreading Factor	0.3%		

**Degassing Patterns**			36Ar(a) [fA]	%1σ	36Ar(c) [fA]	%1σ	36Ar(ca) [fA]	%1σ	36Ar(cl) [fA]	%1σ	37Ar(ca) [fA]	%1σ	38Ar(a) [fA]	%1σ	38Ar(c) [fA]	%1σ	38Ar(k) [fA]	%1σ	38Ar(ca) [fA]	%1σ	38Ar(cl) [fA]	%1σ	39Ar(k) [fA]	%1σ	39Ar(ca) [fA]	%1σ	40Ar(r) [fA]	%1σ	40Ar(a) [fA]	%1σ	40Ar(c) [fA]	%1σ	40Ar(k) [fA]	%1σ
18D00548	1.8 %	✓	2.748429	0.31	0.0000000	0.00	0.0223710	3.55	0.0000000	0.00	82.7638	3.55	0.513681	0.31	0.0000000	0.00	0.0794024	0.36	0.0148975	10.26	0.0000000	0.00	6.574679	0.35	0.0531757	3.67	2.896651	110.29	812.161	0.39	0.0000000	0.00	0.0039908	9.66
18D00550	1.9 %	✓	1.512539	0.33	0.0000000	0.00	0.0107144	7.69	0.0000000	0.00	39.6391	7.69	0.282694	0.33	0.0000000	0.00	0.0380213	0.70	0.0071350	12.32	0.0000000	0.00	3.148243	0.70	0.0254681	7.75	0.165508	######	446.955	0.41	0.0000000	0.00	0.0019110	9.68
18D00551	2.0 %	✓	1.221947	0.36	0.0000000	0.00	0.0096799	8.44	0.0000000	0.00	35.8116	8.44	0.228382	0.36	0.0000000	0.00	0.0304257	0.91	0.0064461	12.81	0.0000000	0.00	2.519310	0.91	0.0230090	8.49	0.011798	######	361.085	0.43	0.0000000	0.00	0.0015292	9.69
18D00553	2.2 %	✓	1.215940	0.36	0.0000000	0.00	0.0102594	8.10	0.0000000	0.00	37.9556	8.10	0.227259	0.36	0.0000000	0.00	0.0299190	0.90	0.0068320	12.58	0.0000000	0.00	2.477352	0.90	0.0243865	8.15	1.931802	82.68	359.310	0.43	0.0000000	0.00	0.0015038	9.69
18D00554	2.4 %	✓	2.284667	0.32	0.0000000	0.00	0.0319537	2.65	0.0000000	0.00	118.2159	2.65	0.427004	0.32	0.0000000	0.00	0.0671432	0.44	0.0212789	9.99	0.0000000	0.00	5.559594	0.43	0.0759537	2.80	2.804247	96.72	675.119	0.40	0.0000000	0.00	0.0033747	9.66
18D00556	2.7 %	✓	2.737798	0.31	0.0000000	0.00	0.0485083	1.78	0.0000000	0.00	179.4611	1.77	0.511694	0.31	0.0000000	0.00	0.0867885	0.33	0.0323030	9.79	0.0000000	0.00	7.186260	0.32	0.1153037	2.00	3.066465	104.24	809.019	0.39	0.0000000	0.00	0.0043621	9.66
18D00557	3.0 %	✓	2.137594	0.32	0.0000000	0.00	0.0430970	2.05	0.0000000	0.00	159.4414	2.04	0.399516	0.32	0.0000000	0.00	0.0695832	0.40	0.0286995	9.84	0.0000000	0.00	5.761626	0.39	0.1024411	2.24	1.348766	188.55	631.659	0.40	0.0000000	0.00	0.0034973	9.66
18D00559	3.4 %	✓	1.980489	0.32	0.0000000	0.00	0.0428724	2.00	0.0000000	0.00	158.6105	1.99	0.370153	0.32	0.0000000	0.00	0.0616382	0.48	0.0285499	9.83	0.0000000	0.00	5.103766	0.47	0.1019072	2.20	3.000141	79.00	585.235	0.40	0.0000000	0.00	0.0030980	9.66
18D00560	3.9 %	✓	2.135349	0.32	0.0000000	0.00	0.0517463	1.65	0.0000000	0.00	191.4403	1.64	0.399097	0.32	0.0000000	0.00	0.0670926	0.41	0.0344592	9.77	0.0000000	0.00	5.555404	0.40	0.1230004	1.88	1.140439	221.64	630.996	0.40	0.0000000	0.00	0.0033721	9.66
18D00562	4.5 %	✓	1.398906	0.35	0.0000000	0.00	0.0326920	2.49	0.0000000	0.00	120.9473	2.48	0.261456	0.35	0.0000000	0.00	0.0421364	0.62	0.0217705	9.94	0.0000000	0.00	3.488983	0.61	0.0777086	2.64	0.361214	494.42	413.377	0.42	0.0000000	0.00	0.0021178	9.67
18D00563	5.2 %	✓	1.435924	0.35	0.0000000	0.00	0.0368157	2.30	0.0000111	79.83	136.2032	2.29	0.268374	0.35	0.0000000	0.00	0.0438448	0.64	0.0245166	9.90	0.0285457	79.83	3.630438	0.63	0.0875106	2.47	0.254345	717.84	424.316	0.42	0.0000000	0.00	0.0022037	9.67
18D00565	6.0 %	✓	1.516688	0.34	0.0000000	0.00	0.0437958	2.01	0.0000032	290.69	162.0265	2.01	0.283469	0.34	0.0000000	0.00	0.0500631	0.55	0.0291648	9.84	0.0081554	290.69	4.145326	0.54	0.1041020	2.21	0.198960	955.46	448.181	0.42	0.0000000	0.00	0.0025162	9.67
18D00566	6.9 %	✓	2.284759	0.32	0.0000000	0.00	0.0791771	1.21	0.0000000	0.00	292.9232	1.19	0.427022	0.32	0.0000000	0.00	0.0841090	0.34	0.0527262	9.70	0.0000000	0.00	6.964398	0.33	0.1882031	1.51	2.174986	125.28	675.146	0.40	0.0000000	0.00	0.0042274	9.66
18D00568	7.9 %	✓	2.735730	0.31	0.0000000	0.00	0.0712859	1.30	0.0000000	0.00	263.7287	1.29	0.511308	0.31	0.0000000	0.00	0.0713086	0.38	0.0474712	9.72	0.0000000	0.00	5.904492	0.37	0.1694457	1.58	0.491580	651.26	808.408	0.39	0.0000000	0.00	0.0035840	9.66
18D00569	9.0 %	✓	3.778953	0.30	0.0000000	0.00	0.0662620	1.43	0.0000030	321.83	245.1423	1.42	0.706286	0.30	0.0000000	0.00	0.0667066	0.41	0.0441256	9.73	0.0076899	321.83	5.523443	0.40	0.1575039	1.70	5.886343	73.07	1116.681	0.38	0.0000000	0.00	0.0033527	9.66
18D00571	10.3 %	✓	9.723687	0.29	0.0000000	0.00	0.0903440	1.15	0.0000012	846.09	334.2362	1.14	1.817357	0.29	0.0000000	0.00	0.0897554	0.32	0.0601625	9.70	0.0031028	846.09	7.431932	0.31	0.2147467	1.46	17.533310	61.30	2873.349	0.37	0.0000000	0.00	0.0045112	9.65
18D00572	11.6 %	✓	6.357765	0.29	0.0000000	0.00	0.0505076	1.72	0.0000037	245.99	186.8575	1.71	1.188266	0.29	0.0000000	0.00	0.0537942	0.51	0.0336344	9.78	0.0095948	245.99	4.454272	0.50	0.1200560	1.94	5.159161	137.27	1878.720	0.38	0.0000000	0.00	0.0027037	9.66
18D00574	12.5 %	✓	5.525041	0.29	0.0000000	0.00	0.0377721	2.31	0.0000012	786.80	139.7415	2.31	1.032630	0.29	0.0000000	0.00	0.0403529	0.69	0.0251535	9.90	0.0029680	786.80	3.341305	0.68	0.0897839	2.48	0.798772	771.95	1632.650	0.38	0.0000000	0.00	0.0020282	9.67
18D00575	13.4 %	✓	1.394555	0.35	0.0000000	0.00	0.0149466	5.18	0.0000000	0.00	55.2964	5.18	0.260642	0.35	0.0000000	0.00	0.0153395	1.67	0.0099534	10.94	0.0000000	0.00	1.270144	1.67	0.0355280	5.26	1.658863	106.73	412.091	0.42	0.0000000	0.00	0.0007710	9.79
18D00577	14.6 %	✓	1.968341	0.32	0.0000000	0.00	0.0186119	4.46	0.0000000	0.00	68.8564	4.46	0.367883	0.32	0.0000000	0.00	0.0171670	1.55	0.0123941	10.61	0.0000000	0.00	1.421464	1.55	0.0442402	4.55	2.704009	87.22	581.645	0.40	0.0000000	0.00	0.0008628	9.77
18D00578	16.0 %	✓	0.373810	0.66	0.0000000	0.00	0.0052589	14.74	0.0000000	0.00	19.4558	14.74	0.069865	0.66	0.0000000	0.00	0.0041680	6.82	0.0035020	17.60	0.0000000	0.00	0.345118	6.82	0.0125003	14.76	0.795430	108.48	110.461	0.70	0.0000000	0.00	0.0002095	11.82
18D00580	17.6 %	✓	3.068623	0.31	0.0000000	0.00	0.0394194	2.11	0.0000000	0.00	145.8356	2.11	0.573526	0.31	0.0000000	0.00	0.0276306	0.99	0.0262504	9.86	0.0000000	0.00	2.287871	0.99	0.0936994	2.30	5.919046	59.83	906.778	0.39	0.0000000	0.00	0.0013887	9.70
18D00581	19.3 %	✓	1.747854	0.33	0.0000000	0.00	0.0252249	3.16	0.0000000	0.00	93.3219	3.16	0.326674	0.33	0.0000000	0.00	0.0176377	1.46	0.0167979	10.13	0.0000000	0.00	1.460434	1.46	0.0599593	3.29	2.665983	79.99	516.491	0.41	0.0000000	0.00	0.0008865	9.76
18D00583	21.0 %	✓	4.495394	0.30	0.0000000	0.00	0.0809344	1.22	0.0000000	0.00	299.4244	1.21	0.840189	0.30	0.0000000	0.00	0.0472681	0.54	0.0538964	9.71	0.0000000	0.00	3.913896	0.53	0.1923802	1.52	11.028718	46.01	1328.389	0.38	0.0000000	0.00	0.0023757	9.66
		Σ	65.780783	0.08	0.0000000	0.00	0.9642509	0.44	0.0000233	98.31	3567.3360	0.44	12.294428	0.08	0.0000000	0.00	1.2012962	0.11	0.6421205	2.32	0.0600567	98.30	99.469751	0.11	2.2920134	0.49	72.615456	26.02	19438.221	0.10	0.0000000	0.00	0.0603781	2.19
		Σ							66.745057	0.08	3567.3360	0.44									14.197902	0.43			101.76176	0.11							19510.897	0.14

Additional Parameters			40Ar/39Ar	1σ	37Ar/39Ar	1σ	36Ar/39Ar	1σ	Time (days)	37Ar (decay)	39Ar (decay)	40Ar (moles)
18D00548	1.8 %	✓	122.975148	0.429790	12.487262	0.445187	0.418054	0.001934	239.031	113.436672	1.00169158	3.912E-11
18D00550	1.9 %	✓	140.883204	0.977760	12.489823	0.964489	0.479960	0.003658	239.044	113.467796	1.00169168	2.146E-11
18D00551	2.0 %	✓	142.035149	1.282817	14.086210	1.195810	0.484450	0.004664	239.051	113.483361	1.00169173	1.733E-11
18D00553	2.2 %	✓	144.396998	1.290864	15.171699	1.235718	0.490139	0.004679	239.065	113.514498	1.00169183	1.734E-11
18D00554	2.4 %	✓	120.294757	0.517391	20.976821	0.562125	0.411073	0.002174	239.072	113.530069	1.00169188	3.254E-11
18D00556	2.7 %	✓	111.221389	0.350786	24.578442	0.442057	0.381604	0.001666	239.087	113.562776	1.00169198	3.898E-11
18D00557	3.0 %	✓	107.947477	0.412331	27.189567	0.564313	0.371873	0.001818	239.094	113.578354	1.00169203	3.038E-11
18D00559	3.4 %	✓	112.999372	0.523664	30.468774	0.623138	0.388684	0.002157	239.108	113.609517	1.00169213	2.824E-11
18D00560	3.9 %	✓	111.323414	0.437014	33.713740	0.568180	0.385160	0.001906	239.115	113.625101	1.00169218	3.034E-11
18D00562	4.5 %	✓	116.001067	0.697892	33.910210	0.864645	0.401380	0.002742	239.128	113.656277	1.00169228	1.986E-11
18D00563	5.2 %	✓	114.195265	0.706932	36.633965	0.868862	0.396119	0.002764	239.135	113.671868	1.00169232	2.038E-11
18D00565	6.0 %	✓	105.422424	0.564708	38.129018	0.791000	0.367223	0.002283	239.149	113.703056	1.00169242	2.150E-11
18D00566	6.9 %	✓	94.696401	0.304169	40.953373	0.506011	0.330500	0.001460	239.156	113.718653	1.00169247	3.251E-11
18D00568	7.9 %	✓	133.014232	0.478241	43.419716	0.581002	0.462141	0.002165	239.170	113.749855	1.00169257	3.878E-11
18D00569	9.0 %	✓	197.602703	0.761684	43.151657	0.636291	0.676862	0.003279	239.177	113.765458	1.00169262	5.388E-11
18D00571	10.3 %	✓	378.057878	1.123084	43.709976	0.514795	1.283437	0.005291	239.192	113.798233	1.00169272	1.388E-10
18D00572	11.6 %	✓	411.837888	2.003108	40.849176	0.726358	1.400922	0.007926	239.199	113.813844	1.00169277	9.043E-11
18D00574	12.5 %	✓	476.073509	3.136924	40.728042	0.977073	1.621297	0.011670	239.213	113.845071	1.00169287	7.841E-11
18D00575	13.4 %	✓	316.886974	5.129052	42.350925	2.298391	1.079521	0.017822	239.219	113.860688	1.00169292	1.986E-11
18D00577	14.6 %	✓	398.681735	5.979418	46.978360	2.208716	1.355630	0.020761	239.233	113.891928	1.00169302	2.805E-11
18D00578	16.0 %	✓	311.104158	20.432814	54.403742	8.775282	1.059982	0.069832	239.240	113.907551	1.00169307	5.340E-12
18D00580	17.6 %	✓	383.233981	3.614997	61.235059	1.413526	1.305039	0.012916	239.254	113.938804	1.00169316	4.381E-11
18D00581	19.3 %	✓	341.462802	4.778480	61.380131	2.118330	1.166198	0.016725	239.261	113.954433	1.00169321	2.492E-11
18D00583	21.0 %	✓	326.188483	1.648142	72.918722	0.952888	1.114472	0.006497	239.275	113.985699	1.00169331	6.429E-11

Procedure Blanks		36Ar ± 1σ (SE) [fA]	37Ar ± 1σ (SE) [fA]	38Ar ± 1σ (SE) [fA]	39Ar ± 1σ (SE) [fA]	40Ar ± 1σ (SE) [fA]
18D00548	1.8 %	0.0198677 ± 0.0017239	0.0182149 ± 0.0179828	0.0374153 ± 0.0166328	0.0437827 ± 0.0148256	5.8539302 ± 0.3794408
18D00550	1.9 %	0.0219917 ± 0.0017239	0.0067982 ± 0.0179828	0.0365313 ± 0.0166328	0.0410881 ± 0.0148256	6.5162004 ± 0.3794408
18D00551	2.0 %	0.0226332 ± 0.0017239	0.0132310 ± 0.0179828	0.0353343 ± 0.0166328	0.0384298 ± 0.0148256	6.7284253 ± 0.3794408
18D00553	2.2 %	0.0232514 ± 0.0017239	0.0167210 ± 0.0179828	0.0319671 ± 0.0166328	0.0316161 ± 0.0148256	6.9617207 ± 0.3794408
18D00554	2.4 %	0.0232923 ± 0.0017239	0.0147894 ± 0.0179828	0.0299865 ± 0.0166328	0.0278555 ± 0.0148256	6.9999095 ± 0.3794408
18D00556	2.7 %	0.0229573 ± 0.0017239	0.0052787 ± 0.0179828	0.0256377 ± 0.0166328	0.0201288 ± 0.0148256	6.9528567 ± 0.3794408
18D00557	3.0 %	0.0226522 ± 0.0017239	0.0009859 ± 0.0179828	0.0236238 ± 0.0166328	0.0168303 ± 0.0148256	6.8846523 ± 0.3794408
18D00559	3.4 %	0.0218770 ± 0.0017239	0.0150535 ± 0.0179828	0.0199962 ± 0.0166328	0.0115495 ± 0.0148256	6.6912109 ± 0.3794408
18D00560	3.9 %	0.0214462 ± 0.0017239	0.0222411 ± 0.0179828	0.0184709 ± 0.0166328	0.0097382 ± 0.0148256	6.5767957 ± 0.3794408
18D00562	4.5 %	0.0205821 ± 0.0017239	0.0356289 ± 0.0179828	0.0161559 ± 0.0166328	0.0080642 ± 0.0148256	6.3357598 ± 0.3794408
18D00563	5.2 %	0.0201760 ± 0.0017239	0.0414086 ± 0.0179828	0.0154046 ± 0.0166328	0.0082624 ± 0.0148256	6.2168642 ± 0.3794408
18D00565	6.0 %	0.0194685 ± 0.0017239	0.0503317 ± 0.0179828	0.0147488 ± 0.0166328	0.0107442 ± 0.0148256	5.9977598 ± 0.3794408
18D00566	6.9 %	0.0191822 ± 0.0017239	0.0532491 ± 0.0179828	0.0148328 ± 0.0166328	0.0129787 ± 0.0148256	5.9021796 ± 0.3794408
18D00568	7.9 %	0.0187668 ± 0.0017239	0.0556748 ± 0.0179828	0.0157344 ± 0.0166328	0.0191747 ± 0.0148256	5.7466632 ± 0.3794408
18D00569	9.0 %	0.0186406 ± 0.0017239	0.0551520 ± 0.0179828	0.0164907 ± 0.0166328	0.0229770 ± 0.0148256	5.6882590 ± 0.3794408
18D00571	10.3 %	0.0185431 ± 0.0017239	0.0504753 ± 0.0179828	0.0185026 ± 0.0166328	0.0318950 ± 0.0148256	5.6074915 ± 0.3794408
18D00572	11.6 %	0.0185663 ± 0.0017239	0.0467210 ± 0.0179828	0.0195440 ± 0.0166328	0.0362990 ± 0.0148256	5.5872622 ± 0.3794408
18D00574	12.5 %	0.0187072 ± 0.0017239	0.0369342 ± 0.0179828	0.0214383 ± 0.0166328	0.0445774 ± 0.0148256	5.5739546 ± 0.3794408
18D00575	13.4 %	0.0188029 ± 0.0017239	0.0312666 ± 0.0179828	0.0221287 ± 0.0166328	0.0480689 ± 0.0148256	5.5761117 ± 0.3794408
18D00577	14.6 %	0.0189747 ± 0.0017239	0.0195387 ± 0.0179828	0.0225275 ± 0.0166328	0.0526613 ± 0.0148256	5.5822654 ± 0.3794408
18D00578	16.0 %	0.0190165 ± 0.0017239	0.0140379 ± 0.0179828	0.0220235 ± 0.0166328	0.0532692 ± 0.0148256	5.5784007 ± 0.3794408
18D00580	17.6 %	0.0189113 ± 0.0017239	0.0054053 ± 0.0179828	0.0190157 ± 0.0166328	0.0497380 ± 0.0148256	5.5332647 ± 0.3794408
18D00581	19.3 %	0.0187178 ± 0.0017239	0.0030278 ± 0.0179828	0.0162497 ± 0.0166328	0.0449958 ± 0.0148256	5.4810355 ± 0.3794408
18D00583	21.0 %	0.0179179 ± 0.0017239	0.0042797 ± 0.0179828	0.0074756 ± 0.0166328	0.0279127 ± 0.0148256	5.2859892 ± 0.3794408

Intercept Values		36Ar ± 1σ (SE) [fA]	r2	Regression (type,n)	37Ar ± 1σ (SE) [fA]	r2	Regression (type,n)	38Ar ± 1σ (SE) [fA]	r2	Regression (type,n)	39Ar ± 1σ (SE) [fA]	r2	Regression (type,n)	40Ar ± 1σ (SE) [fA]	r2	Regression (type,n)
18D00548	1.8 %	2.6579807 ± 0.0026948	0.9794	EXP 150 of 150	0.6988711 ± 0.0172812	0.0797	EXP 150 of 150	0.6331184 ± 0.0182433	0.0252	EXP 150 of 150	6.6226236 ± 0.0166922	0.6822	EXP 150 of 150	820.9153 ± 0.0501	0.9996	EXP 150 of 150
18D00550	1.9 %	1.4723001 ± 0.0016600	0.9733	EXP 150 of 150	0.3501471 ± 0.0191886	0.0162	EXP 150 of 150	0.3373077 ± 0.0149675	0.0007	EXP 150 of 150	3.1913294 ± 0.0156924	0.2211	EXP 150 of 150	453.6389 ± 0.0368	0.9990	EXP 150 of 150
18D00551	2.0 %	1.1952806 ± 0.0017138	0.9552	EXP 150 of 150	0.3233845 ± 0.0189044	0.0284	EXP 150 of 150	0.2722532 ± 0.0183986	0.0010	EXP 150 of 150	2.5619481 ± 0.0170155	0.0422	EXP 150 of 150	367.8271 ± 0.0341	0.9983	EXP 150 of 150
18D00553	2.2 %	1.1907306 ± 0.0017459	0.9525	EXP 150 of 150	0.3453525 ± 0.0194738	0.0271	EXP 150 of 150	0.2795633 ± 0.0164142	0.0043	EXP 150 of 150	2.5148528 ± 0.0162239	0.0465	EXP 149 of 150	368.2052 ± 0.0320	0.9987	EXP 150 of 150
18D00554	2.4 %	2.2289759 ± 0.0024691	0.9750	EXP 150 of 150	1.0381987 ± 0.0189203	0.1203	EXP 150 of 150	0.5347740 ± 0.0148390	0.0325	EXP 150 of 150	5.6217258 ± 0.0182802	0.5854	EXP 150 of 150	684.9267 ± 0.0440	0.9996	EXP 150 of 150
18D00556	2.7 %	2.6758337 ± 0.0026345	0.9807	EXP 150 of 150	1.5584478 ± 0.0177305	0.2956	EXP 150 of 150	0.6287616 ± 0.0165173	0.0229	EXP 150 of 150	7.2676936 ± 0.0163071	0.7782	EXP 150 of 150	819.0429 ± 0.0515	0.9996	EXP 150 of 150
18D00557	3.0 %	2.0989149 ± 0.0021245	0.9782	EXP 150 of 150	1.3787314 ± 0.0193785	0.1622	EXP 150 of 150	0.4927075 ± 0.0164420	0.0074	EXP 150 of 150	5.8375290 ± 0.0156850	0.6032	EXP 150 of 150	639.8959 ± 0.0413	0.9995	EXP 150 of 150
18D00559	3.4 %	1.9483446 ± 0.0019408	0.9795	EXP 150 of 150	1.3570968 ± 0.0182073	0.1906	EXP 150 of 150	0.4737566 ± 0.0160836	0.0253	EXP 150 of 150	5.1787231 ± 0.0181548	0.4608	EXP 150 of 150	594.9290 ± 0.0421	0.9994	EXP 150 of 150
18D00560	3.9 %	2.1038066 ± 0.0018961	0.9830	EXP 150 of 150	1.6336948 ± 0.0166958	0.2689	EXP 150 of 150	0.4960549 ± 0.0166071	0.0114	EXP 150 of 150	5.6461471 ± 0.0155895	0.5840	EXP 150 of 150	638.7162 ± 0.0382	0.9995	EXP 150 of 150
18D00562	4.5 %	1.3836243 ± 0.0018283	0.9621	EXP 150 of 150	1.0102638 ± 0.0171788	0.0583	EXP 150 of 150	0.3377258 ± 0.0154733	0.0107	EXP 150 of 150	3.5483774 ± 0.0147394	0.3401	EXP 148 of 150	420.0758 ± 0.0338	0.9988	EXP 150 of 150
18D00563	5.2 %	1.4224005 ± 0.0018419	0.9649	EXP 150 of 150	1.1362486 ± 0.0183361	0.1106	EXP 150 of 150	0.3765026 ± 0.0149570	0.0506	EXP 150 of 150	3.6987140 ± 0.0168716	0.2623	EXP 150 of 150	430.7890 ± 0.0362	0.9986	EXP 150 of 150
18D00565	6.0 %	1.5052277 ± 0.0017659	0.9715	EXP 150 of 150	1.3502178 ± 0.0192101	0.1037	EXP 150 of 150	0.3813540 ± 0.0162228	0.0338	EXP 150 of 150	4.2287432 ± 0.0164107	0.4715	EXP 149 of 150	453.9827 ± 0.0374	0.9989	EXP 150 of 150
18D00566	6.9 %	2.2699153 ± 0.0022438	0.9805	EXP 150 of 150	2.4784175 ± 0.0165630	0.4211	EXP 150 of 150	0.5626803 ± 0.0147725	0.0671	EXP 150 of 150	7.1126793 ± 0.0161290	0.7412	EXP 150 of 150	683.2278 ± 0.0418	0.9995	EXP 150 of 150
18D00568	7.9 %	2.6913612 ± 0.0025304	0.9819	EXP 150 of 150	2.2230454 ± 0.0168467	0.3385	EXP 150 of 150	0.6260393 ± 0.0170892	0.0187	EXP 150 of 150	6.0481891 ± 0.0149857	0.6565	EXP 150 of 150	813.6669 ± 0.0467	0.9997	EXP 150 of 150
18D00569	9.0 %	3.6797205 ± 0.0027901	0.9888	EXP 150 of 150	2.0626838 ± 0.0190806	0.2728	EXP 150 of 150	0.8318533 ± 0.0172617	0.0718	EXP 150 of 150	5.6619064 ± 0.0152891	0.5531	EXP 150 of 150	1128.2587 ± 0.0516	0.9998	EXP 150 of 150
18D00571	10.3 %	9.3626046 ± 0.0044833	0.9956	EXP 150 of 150	2.8362299 ± 0.0186351	0.3559	EXP 150 of 150	1.9663153 ± 0.0181408	0.2280	EXP 150 of 150	7.6220174 ± 0.0161254	0.3893	EXP 150 of 150	2896.4948 ± 0.0813	0.9999	EXP 150 of 150
18D00572	11.6 %	6.1199657 ± 0.0038130	0.9927	EXP 150 of 150	1.5668944 ± 0.0175982	0.1721	EXP 150 of 150	1.2901144 ± 0.0155583	0.1766	EXP 150 of 150	4.5767937 ± 0.0160373	0.0020	EXP 150 of 150	1889.4688 ± 0.0706	0.9999	EXP 150 of 150
18D00574	12.5 %	5.3151319 ± 0.0034211	0.9919	EXP 150 of 150	1.1694782 ± 0.0194733	0.0479	EXP 150 of 150	1.1099329 ± 0.0154534	0.1087	EXP 150 of 150	3.4502886 ± 0.0166614	0.0422	EXP 150 of 150	1639.0245 ± 0.0645	0.9999	EXP 150 of 150
18D00575	13.4 %	1.3608066 ± 0.0018276	0.9604	EXP 150 of 150	0.4460514 ± 0.0166399	0.0311	EXP 150 of 150	0.2932527 ± 0.0176844	0.0015	EXP 150 of 150	1.3440839 ± 0.0147650	0.0219	EXP 150 of 150	419.3267 ± 0.0373	0.9974	EXP 150 of 150
18D00577	14.6 %	1.9107767 ± 0.0021293	0.9751	EXP 150 of 150	0.5746656 ± 0.0189832	0.0000	EXP 150 of 150	0.4088248 ± 0.0166956	0.0005	EXP 150 of 150	1.5075244 ± 0.0159498	0.0362	EXP 150 of 150	589.9318 ± 0.0445	0.9992	EXP 150 of 150
18D00578	16.0 %	0.3799326 ± 0.0009846	0.7595	EXP 150 of 150	0.1538348 ± 0.0169468	0.0020	EXP 150 of 150	0.0766023 ± 0.0153366	0.0321	EXP 150 of 150	0.4082421 ± 0.0179504	0.1579	EXP 150 of 150	116.8349 ± 0.0225	0.9957	EXP 150 of 150
18D00580	17.6 %	2.9781172 ± 0.0027758	0.9830	EXP 150 of 150	1.2525824 ± 0.0173520	0.1965	EXP 150 of 150	0.6260535 ± 0.0168503	0.0168	EXP 150 of 150	2.4136925 ± 0.0165466	0.0518	EXP 150 of 150	918.2319 ± 0.0448	0.9998	EXP 150 of 150
18D00581	19.3 %	1.7068881 ± 0.0020003	0.9708	EXP 150 of 150	0.8018631 ± 0.0170201	0.0787	EXP 149 of 150	0.3670058 ± 0.0177193	0.0125	EXP 150 of 150	1.5541429 ± 0.0149634	0.0473	EXP 150 of 150	524.6387 ± 0.0426	0.9989	EXP 150 of 150
18D00583	21.0 %	4.3750970 ± 0.0029217	0.9913	EXP 150 of 150	2.5775131 ± 0.0177659	0.4082	EXP 149 of 150	0.9264263 ± 0.0166926	0.0977	EXP 149 of 150	4.1038157 ± 0.0139653	0.0019	EXP 149 of 150	1344.7061 ± 0.0548	0.9999	EXP 150 of 150

Project Info		Analyst	Irradiation	X-pos	Y-pos	Z/H-pos	Project	Experiment	Nmb
18D00548	1.8 %	Dan Miggins	17-OSU-05	0.00	0.00	24.95	Arctic\O-Connor (16-22)	18D00544	01
18D00550	1.9 %	Dan Miggins	17-OSU-05	0.00	0.00	24.95	Arctic\O-Connor (16-22)	18D00544	01
18D00551	2.0 %	Dan Miggins	17-OSU-05	0.00	0.00	24.95	Arctic\O-Connor (16-22)	18D00544	01
18D00553	2.2 %	Dan Miggins	17-OSU-05	0.00	0.00	24.95	Arctic\O-Connor (16-22)	18D00544	01
18D00554	2.4 %	Dan Miggins	17-OSU-05	0.00	0.00	24.95	Arctic\O-Connor (16-22)	18D00544	01
18D00556	2.7 %	Dan Miggins	17-OSU-05	0.00	0.00	24.95	Arctic\O-Connor (16-22)	18D00544	01
18D00557	3.0 %	Dan Miggins	17-OSU-05	0.00	0.00	24.95	Arctic\O-Connor (16-22)	18D00544	01
18D00559	3.4 %	Dan Miggins	17-OSU-05	0.00	0.00	24.95	Arctic\O-Connor (16-22)	18D00544	01
18D00560	3.9 %	Dan Miggins	17-OSU-05	0.00	0.00	24.95	Arctic\O-Connor (16-22)	18D00544	01
18D00562	4.5 %	Dan Miggins	17-OSU-05	0.00	0.00	24.95	Arctic\O-Connor (16-22)	18D00544	01
18D00563	5.2 %	Dan Miggins	17-OSU-05	0.00	0.00	24.95	Arctic\O-Connor (16-22)	18D00544	01
18D00565	6.0 %	Dan Miggins	17-OSU-05	0.00	0.00	24.95	Arctic\O-Connor (16-22)	18D00544	01
18D00566	6.9 %	Dan Miggins	17-OSU-05	0.00	0.00	24.95	Arctic\O-Connor (16-22)	18D00544	01
18D00568	7.9 %	Dan Miggins	17-OSU-05	0.00	0.00	24.95	Arctic\O-Connor (16-22)	18D00544	01
18D00569	9.0 %	Dan Miggins	17-OSU-05	0.00	0.00	24.95	Arctic\O-Connor (16-22)	18D00544	01
18D00571	10.3 %	Dan Miggins	17-OSU-05	0.00	0.00	24.95	Arctic\O-Connor (16-22)	18D00544	01
18D00572	11.6 %	Dan Miggins	17-OSU-05	0.00	0.00	24.95	Arctic\O-Connor (16-22)	18D00544	01
18D00574	12.5 %	Dan Miggins	17-OSU-05	0.00	0.00	24.95	Arctic\O-Connor (16-22)	18D00544	01
18D00575	13.4 %	Dan Miggins	17-OSU-05	0.00	0.00	24.95	Arctic\O-Connor (16-22)	18D00544	01
18D00577	14.6 %	Dan Miggins	17-OSU-05	0.00	0.00	24.95	Arctic\O-Connor (16-22)	18D00544	01
18D00578	16.0 %	Dan Miggins	17-OSU-05	0.00	0.00	24.95	Arctic\O-Connor (16-22)	18D00544	01
18D00580	17.6 %	Dan Miggins	17-OSU-05	0.00	0.00	24.95	Arctic\O-Connor (16-22)	18D00544	01
18D00581	19.3 %	Dan Miggins	17-OSU-05	0.00	0.00	24.95	Arctic\O-Connor (16-22)	18D00544	01
18D00583	21.0 %	Dan Miggins	17-OSU-05	0.00	0.00	24.95	Arctic\O-Connor (16-22)	18D00544	01

**Sample Parameters**		Sample	Material	Location	Standard Name	Standard (in Ma)	%1σ	Standard Reference	Standard 40Ar/39Ar	%1σ	J	%1σ	Air 40Ar/36Ar	%1σ	MDF (lin)	%1σ	Volume Ratio	Sensitivity (mol/volt)	Day	Month	Year	Hour	Min	Resist
18D00548	1.8 %	PS59-297-37	Groundmass	Gakkel Ridge	FCT-NM (5B17-17)	28.201	0.082	Kuiper et al (2008)	9.75664	0.081	0.00161094	0.081	302.419	0.152	0.9942729	0.069	1	4.8E-14	5	JAN	2018	12	34	1
18D00550	1.9 %	PS59-297-37	Groundmass	Gakkel Ridge	FCT-NM (5B17-17)	28.201	0.082	Kuiper et al (2008)	9.75664	0.081	0.00161094	0.081	302.419	0.152	0.9942729	0.069	1	4.8E-14	5	JAN	2018	12	54	1
18D00551	2.0 %	PS59-297-37	Groundmass	Gakkel Ridge	FCT-NM (5B17-17)	28.201	0.082	Kuiper et al (2008)	9.75664	0.081	0.00161094	0.081	302.419	0.152	0.9942729	0.069	1	4.8E-14	5	JAN	2018	13	4	1
18D00553	2.2 %	PS59-297-37	Groundmass	Gakkel Ridge	FCT-NM (5B17-17)	28.201	0.082	Kuiper et al (2008)	9.75664	0.081	0.00161094	0.081	302.419	0.152	0.9942729	0.069	1	4.8E-14	5	JAN	2018	13	24	1
18D00554	2.4 %	PS59-297-37	Groundmass	Gakkel Ridge	FCT-NM (5B17-17)	28.201	0.082	Kuiper et al (2008)	9.75664	0.081	0.00161094	0.081	302.419	0.152	0.9942729	0.069	1	4.8E-14	5	JAN	2018	13	34	1
18D00556	2.7 %	PS59-297-37	Groundmass	Gakkel Ridge	FCT-NM (5B17-17)	28.201	0.082	Kuiper et al (2008)	9.75664	0.081	0.00161094	0.081	302.419	0.152	0.9942729	0.069	1	4.8E-14	5	JAN	2018	13	55	1
18D00557	3.0 %	PS59-297-37	Groundmass	Gakkel Ridge	FCT-NM (5B17-17)	28.201	0.082	Kuiper et al (2008)	9.75664	0.081	0.00161094	0.081	302.419	0.152	0.9942729	0.069	1	4.8E-14	5	JAN	2018	14	5	1
18D00559	3.4 %	PS59-297-37	Groundmass	Gakkel Ridge	FCT-NM (5B17-17)	28.201	0.082	Kuiper et al (2008)	9.75664	0.081	0.00161094	0.081	302.419	0.152	0.9942729	0.069	1	4.8E-14	5	JAN	2018	14	25	1
18D00560	3.9 %	PS59-297-37	Groundmass	Gakkel Ridge	FCT-NM (5B17-17)	28.201	0.082	Kuiper et al (2008)	9.75664	0.081	0.00161094	0.081	302.419	0.152	0.9942729	0.069	1	4.8E-14	5	JAN	2018	14	35	1
18D00562	4.5 %	PS59-297-37	Groundmass	Gakkel Ridge	FCT-NM (5B17-17)	28.201	0.082	Kuiper et al (2008)	9.75664	0.081	0.00161094	0.081	302.419	0.152	0.9942729	0.069	1	4.8E-14	5	JAN	2018	14	55	1
18D00563	5.2 %	PS59-297-37	Groundmass	Gakkel Ridge	FCT-NM (5B17-17)	28.201	0.082	Kuiper et al (2008)	9.75664	0.081	0.00161094	0.081	302.419	0.152	0.9942729	0.069	1	4.8E-14	5	JAN	2018	15	5	1
18D00565	6.0 %	PS59-297-37	Groundmass	Gakkel Ridge	FCT-NM (5B17-17)	28.201	0.082	Kuiper et al (2008)	9.75664	0.081	0.00161094	0.081	302.419	0.152	0.9942729	0.069	1	4.8E-14	5	JAN	2018	15	25	1
18D00566	6.9 %	PS59-297-37	Groundmass	Gakkel Ridge	FCT-NM (5B17-17)	28.201	0.082	Kuiper et al (2008)	9.75664	0.081	0.00161094	0.081	302.419	0.152	0.9942729	0.069	1	4.8E-14	5	JAN	2018	15	35	1
18D00568	7.9 %	PS59-297-37	Groundmass	Gakkel Ridge	FCT-NM (5B17-17)	28.201	0.082	Kuiper et al (2008)	9.75664	0.081	0.00161094	0.081	302.419	0.152	0.9942729	0.069	1	4.8E-14	5	JAN	2018	15	55	1
18D00569	9.0 %	PS59-297-37	Groundmass	Gakkel Ridge	FCT-NM (5B17-17)	28.201	0.082	Kuiper et al (2008)	9.75664	0.081	0.00161094	0.081	302.419	0.152	0.9942729	0.069	1	4.8E-14	5	JAN	2018	16	5	1
18D00571	10.3 %	PS59-297-37	Groundmass	Gakkel Ridge	FCT-NM (5B17-17)	28.201	0.082	Kuiper et al (2008)	9.75664	0.081	0.00161094	0.081	302.419	0.152	0.9942729	0.069	1	4.8E-14	5	JAN	2018	16	26	1
18D00572	11.6 %	PS59-297-37	Groundmass	Gakkel Ridge	FCT-NM (5B17-17)	28.201	0.082	Kuiper et al (2008)	9.75664	0.081	0.00161094	0.081	302.419	0.152	0.9942729	0.069	1	4.8E-14	5	JAN	2018	16	36	1
18D00574	12.5 %	PS59-297-37	Groundmass	Gakkel Ridge	FCT-NM (5B17-17)	28.201	0.082	Kuiper et al (2008)	9.75664	0.081	0.00161094	0.081	302.419	0.152	0.9942729	0.069	1	4.8E-14	5	JAN	2018	16	56	1
18D00575	13.4 %	PS59-297-37	Groundmass	Gakkel Ridge	FCT-NM (5B17-17)	28.201	0.082	Kuiper et al (2008)	9.75664	0.081	0.00161094	0.081	302.419	0.152	0.9942729	0.069	1	4.8E-14	5	JAN	2018	17	6	1
18D00577	14.6 %	PS59-297-37	Groundmass	Gakkel Ridge	FCT-NM (5B17-17)	28.201	0.082	Kuiper et al (2008)	9.75664	0.081	0.00161094	0.081	302.419	0.152	0.9942729	0.069	1	4.8E-14	5	JAN	2018	17	26	1
18D00578	16.0 %	PS59-297-37	Groundmass	Gakkel Ridge	FCT-NM (5B17-17)	28.201	0.082	Kuiper et al (2008)	9.75664	0.081	0.00161094	0.081	302.419	0.152	0.9942729	0.069	1	4.8E-14	5	JAN	2018	17	36	1
18D00580	17.6 %	PS59-297-37	Groundmass	Gakkel Ridge	FCT-NM (5B17-17)	28.201	0.082	Kuiper et al (2008)	9.75664	0.081	0.00161094	0.081	302.419	0.152	0.9942729	0.069	1	4.8E-14	5	JAN	2018	17	56	1
18D00581	19.3 %	PS59-297-37	Groundmass	Gakkel Ridge	FCT-NM (5B17-17)	28.201	0.082	Kuiper et al (2008)	9.75664	0.081	0.00161094	0.081	302.419	0.152	0.9942729	0.069	1	4.8E-14	5	JAN	2018	18	6	1
18D00583	21.0 %	PS59-297-37	Groundmass	Gakkel Ridge	FCT-NM (5B17-17)	28.201	0.082	Kuiper et al (2008)	9.75664	0.081	0.00161094	0.081	302.419	0.152	0.9942729	0.069	1	4.8E-14	5	JAN	2018	18	26	1

Irradiation Constants		40/36(a)	%1σ	40/36(c)	%1σ	38/36(a)	%1σ	38/36(c)	%1σ	39/37(ca)	%1σ	38/37(ca)	%1σ	36/37(ca)	%1σ	40/39(k)	%1σ	38/39(k)	%1σ	36/38(cl)	%1σ	K/Ca	%1σ	K/Cl	%1σ	Ca/Cl	%1σ
18D00548	1.8 %	295.5	0.237	0.018	35	0.1869	0	1.493	3	0.000643	0.92	0.00018	9.63	0.00027	0.17	0.000607	9.65	0.012077	0.09	0	0	0.43	0	0	0	0	0
18D00550	1.9 %	295.5	0.237	0.018	35	0.1869	0	1.493	3	0.000643	0.92	0.00018	9.63	0.00027	0.17	0.000607	9.65	0.012077	0.09	0	0	0.43	0	0	0	0	0
18D00551	2.0 %	295.5	0.237	0.018	35	0.1869	0	1.493	3	0.000643	0.92	0.00018	9.63	0.00027	0.17	0.000607	9.65	0.012077	0.09	0	0	0.43	0	0	0	0	0
18D00553	2.2 %	295.5	0.237	0.018	35	0.1869	0	1.493	3	0.000643	0.92	0.00018	9.63	0.00027	0.17	0.000607	9.65	0.012077	0.09	0	0	0.43	0	0	0	0	0
18D00554	2.4 %	295.5	0.237	0.018	35	0.1869	0	1.493	3	0.000643	0.92	0.00018	9.63	0.00027	0.17	0.000607	9.65	0.012077	0.09	0	0	0.43	0	0	0	0	0
18D00556	2.7 %	295.5	0.237	0.018	35	0.1869	0	1.493	3	0.000643	0.92	0.00018	9.63	0.00027	0.17	0.000607	9.65	0.012077	0.09	0	0	0.43	0	0	0	0	0
18D00557	3.0 %	295.5	0.237	0.018	35	0.1869	0	1.493	3	0.000643	0.92	0.00018	9.63	0.00027	0.17	0.000607	9.65	0.012077	0.09	0	0	0.43	0	0	0	0	0
18D00559	3.4 %	295.5	0.237	0.018	35	0.1869	0	1.493	3	0.000643	0.92	0.00018	9.63	0.00027	0.17	0.000607	9.65	0.012077	0.09	0	0	0.43	0	0	0	0	0
18D00560	3.9 %	295.5	0.237	0.018	35	0.1869	0	1.493	3	0.000643	0.92	0.00018	9.63	0.00027	0.17	0.000607	9.65	0.012077	0.09	0	0	0.43	0	0	0	0	0
18D00562	4.5 %	295.5	0.237	0.018	35	0.1869	0	1.493	3	0.000643	0.92	0.00018	9.63	0.00027	0.17	0.000607	9.65	0.012077	0.09	0	0	0.43	0	0	0	0	0
18D00563	5.2 %	295.5	0.237	0.018	35	0.1869	0	1.493	3	0.000643	0.92	0.00018	9.63	0.00027	0.17	0.000607	9.65	0.012077	0.09	0	0	0.43	0	0	0	0	0
18D00565	6.0 %	295.5	0.237	0.018	35	0.1869	0	1.493	3	0.000643	0.92	0.00018	9.63	0.00027	0.17	0.000607	9.65	0.012077	0.09	0	0	0.43	0	0	0	0	0
18D00566	6.9 %	295.5	0.237	0.018	35	0.1869	0	1.493	3	0.000643	0.92	0.00018	9.63	0.00027	0.17	0.000607	9.65	0.012077	0.09	0	0	0.43	0	0	0	0	0
18D00568	7.9 %	295.5	0.237	0.018	35	0.1869	0	1.493	3	0.000643	0.92	0.00018	9.63	0.00027	0.17	0.000607	9.65	0.012077	0.09	0	0	0.43	0	0	0	0	0
18D00569	9.0 %	295.5	0.237	0.018	35	0.1869	0	1.493	3	0.000643	0.92	0.00018	9.63	0.00027	0.17	0.000607	9.65	0.012077	0.09	0	0	0.43	0	0	0	0	0
18D00571	10.3 %	295.5	0.237	0.018	35	0.1869	0	1.493	3	0.000643	0.92	0.00018	9.63	0.00027	0.17	0.000607	9.65	0.012077	0.09	0	0	0.43	0	0	0	0	0
18D00572	11.6 %	295.5	0.237	0.018	35	0.1869	0	1.493	3	0.000643	0.92	0.00018	9.63	0.00027	0.17	0.000607	9.65	0.012077	0.09	0	0	0.43	0	0	0	0	0
18D00574	12.5 %	295.5	0.237	0.018	35	0.1869	0	1.493	3	0.000643	0.92	0.00018	9.63	0.00027	0.17	0.000607	9.65	0.012077	0.09	0	0	0.43	0	0	0	0	0
18D00575	13.4 %	295.5	0.237	0.018	35	0.1869	0	1.493	3	0.000643	0.92	0.00018	9.63	0.00027	0.17	0.000607	9.65	0.012077	0.09	0	0	0.43	0	0	0	0	0
18D00577	14.6 %	295.5	0.237	0.018	35	0.1869	0	1.493	3	0.000643	0.92	0.00018	9.63	0.00027	0.17	0.000607	9.65	0.012077	0.09	0	0	0.43	0	0	0	0	0
18D00578	16.0 %	295.5	0.237	0.018	35	0.1869	0	1.493	3	0.000643	0.92	0.00018	9.63	0.00027	0.17	0.000607	9.65	0.012077	0.09	0	0	0.43	0	0	0	0	0
18D00580	17.6 %	295.5	0.237	0.018	35	0.1869	0	1.493	3	0.000643	0.92	0.00018	9.63	0.00027	0.17	0.000607	9.65	0.012077	0.09	0	0	0.43	0	0	0	0	0
18D00581	19.3 %	295.5	0.237	0.018	35	0.1869	0	1.493	3	0.000643	0.92	0.00018	9.63	0.00027	0.17	0.000607	9.65	0.012077	0.09	0	0	0.43	0	0	0	0	0
18D00583	21.0 %	295.5	0.237	0.018	35	0.1869	0	1.493	3	0.000643	0.92	0.00018	9.63	0.00027	0.17	0.000607	9.65	0.012077	0.09	0	0	0.43	0	0	0	0	0

18D00544.AGE >>> PS59-297-37 >>> ARCTIC | O-CONNOR (16-22) PROJECT

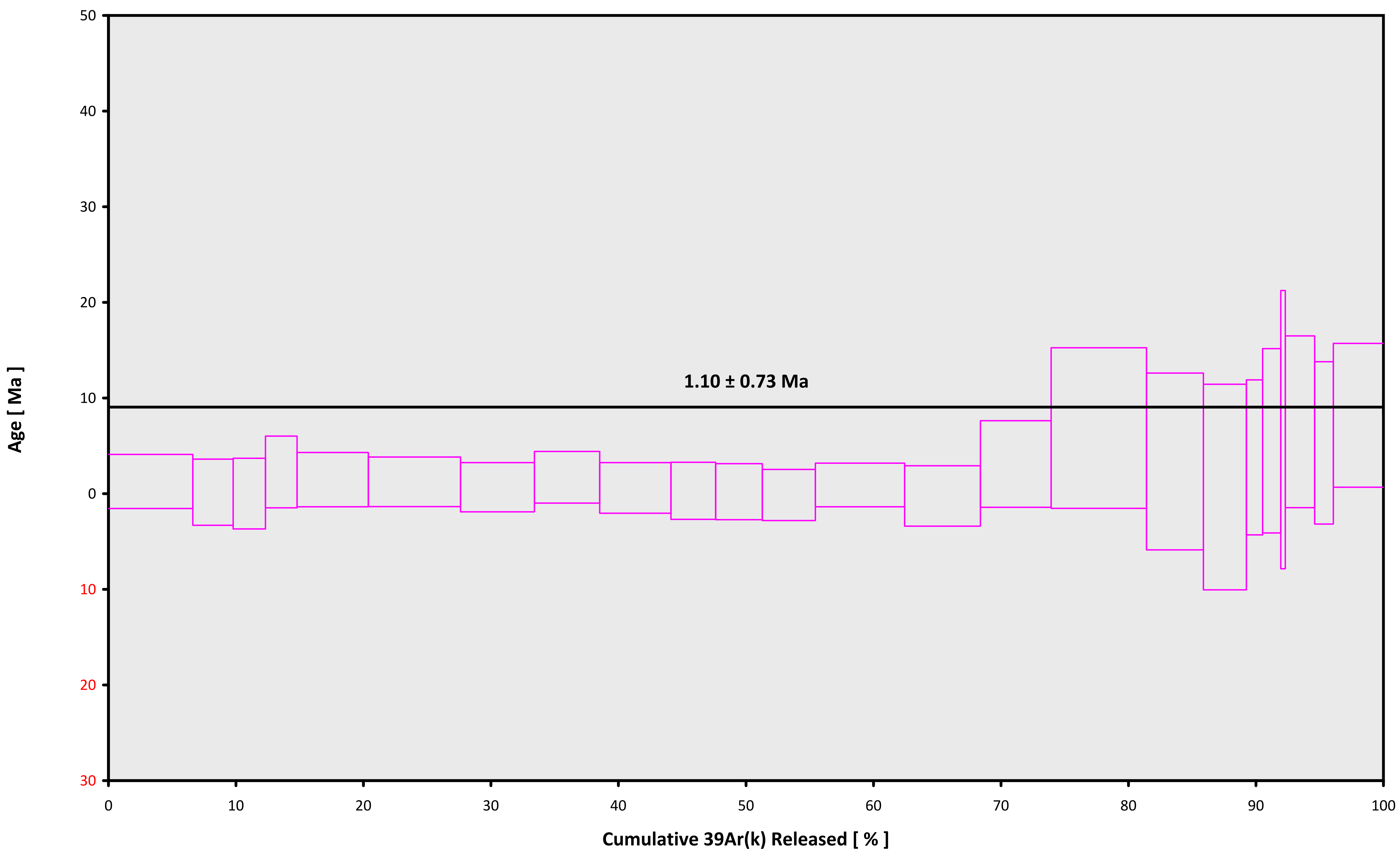

Ar-Ages in Ma

WEIGHTED PLATEAU
1.10 ± 0.73
TOTAL FUSION
2.13 ± 1.11
NORMAL ISOCHRON
1.10 ± 1.38 (NEG)
INVERSE ISOCHRON
1.11 ± 0.61 (NEG)

MSWD (PROBABILITY)
0.66 (89%)

Sample Info

Groundmass
Gakkel Ridge
Dan Miggins

IRR = 17-OSU-05 (5B17-17)
J = 0.00161094 ± 0.00000130

18D00544.AGE >>> PS59-297-37 >>> ARCTIC | O-CONNOR (16-22) PROJECT

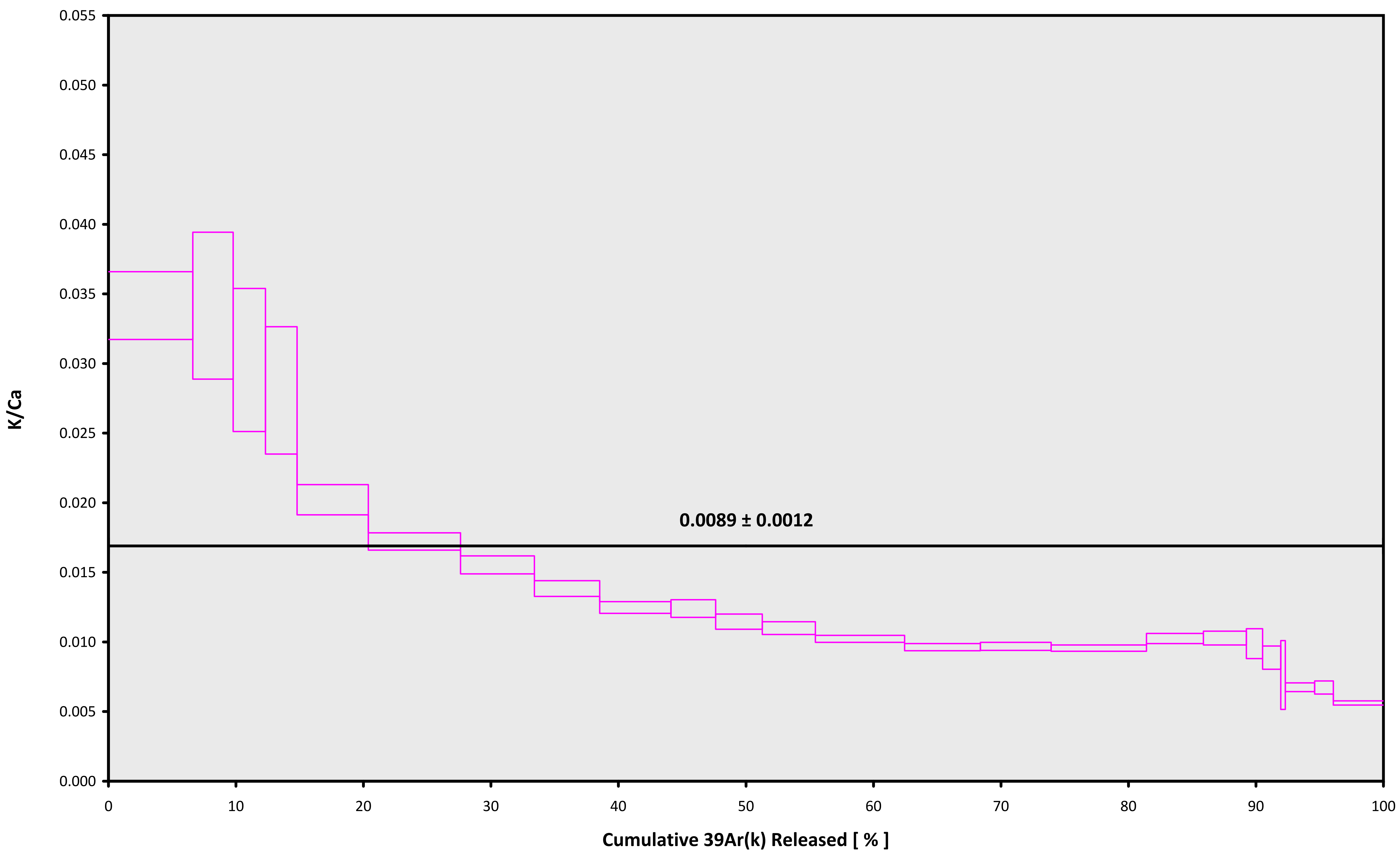

Ar-Ages in Ma

WEIGHTED PLATEAU
1.10 ± 0.73
TOTAL FUSION
2.13 ± 1.11
NORMAL ISOCHRON
1.10 ± 1.38 (NEG)
INVERSE ISOCHRON
1.11 ± 0.61 (NEG)

Sample Info

Groundmass
Gakkel Ridge
Dan Miggins

IRR = 17-OSU-05 (5B17-17)
J = 0.00161094 ± 0.00000130

18D00544.AGE >>> PS59-297-37 >>> ARCTIC | O-CONNOR (16-22) PROJECT

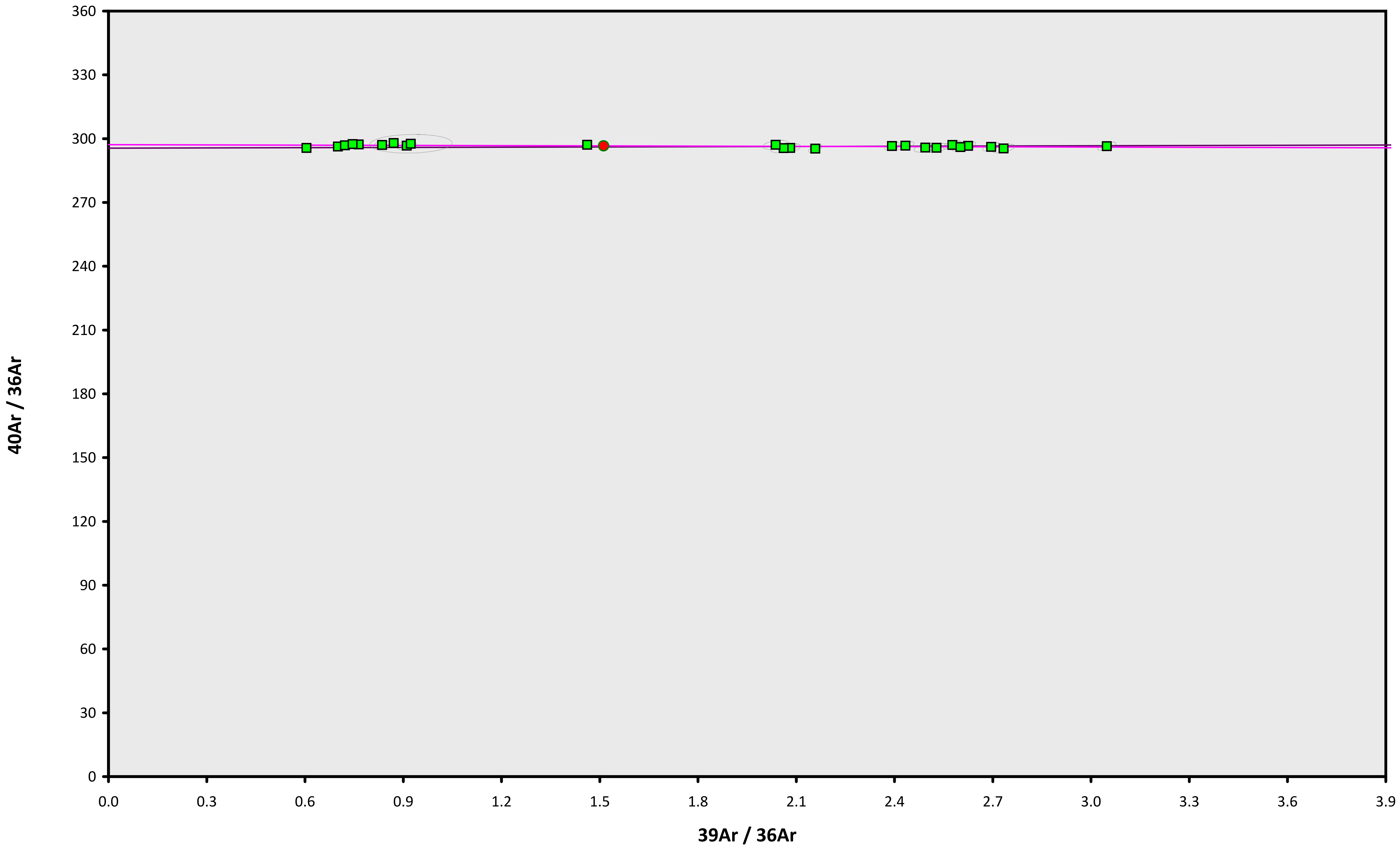

Ar-Ages in Ma

WEIGHTED PLATEAU
1.10 ± 0.73
TOTAL FUSION
2.13 ± 1.11
NORMAL ISOCHRON
1.10 ± 1.38 (NEG)
INVERSE ISOCHRON
1.11 ± 0.61 (NEG)

MSWD (PROBABILITY)
0.48 (98%)

40AR/36AR INTERCEPT
297.2 ± 0.9

Sample Info

Groundmass
Gakkel Ridge
Dan Miggins

IRR = 17-OSU-05 (5B17-17)
J = 0.00161094 ± 0.00000130

18D00544.AGE >>> PS59-297-37 >>> ARCTIC | O-CONNOR (16-22) PROJECT

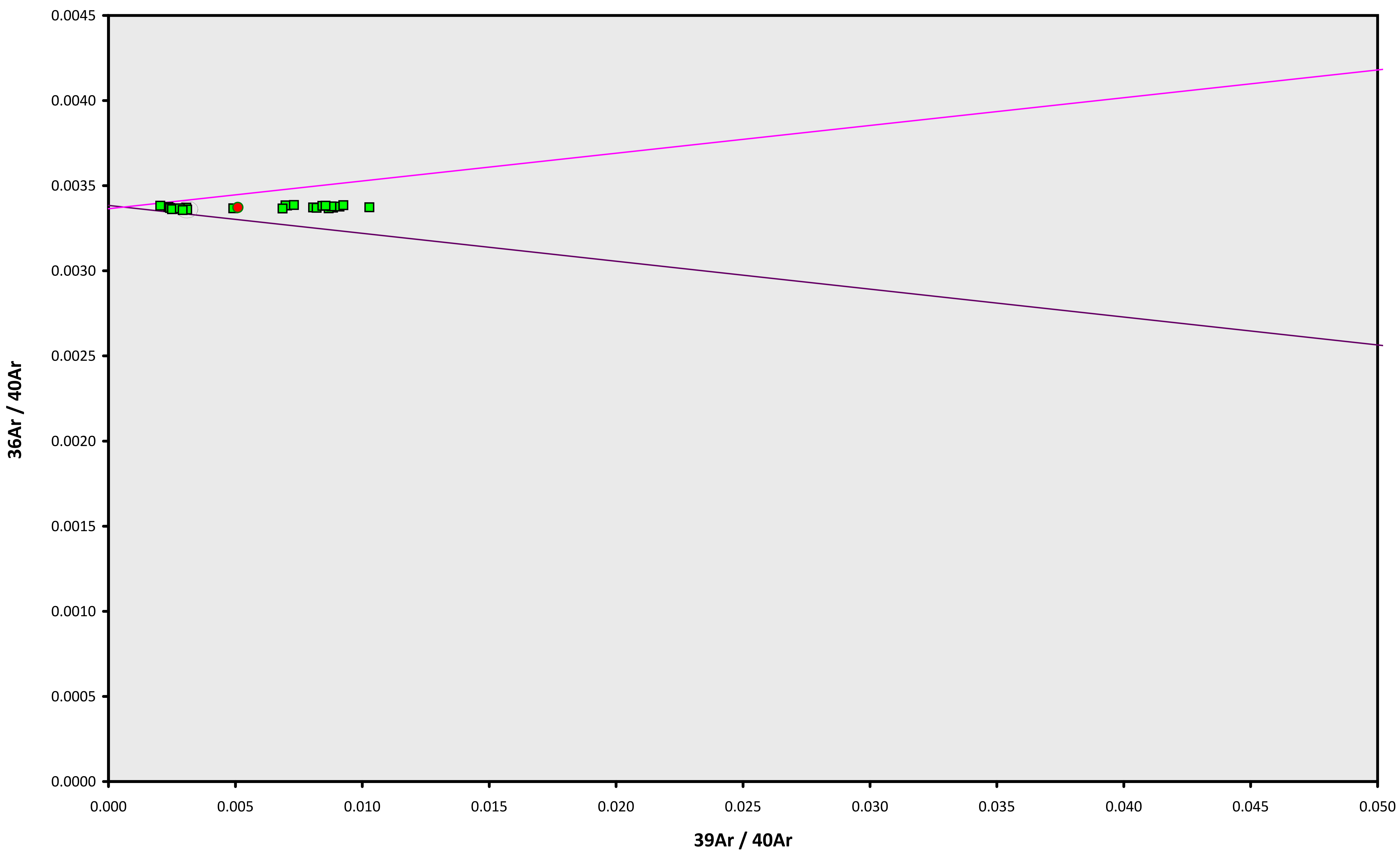

Ar-Ages in Ma

WEIGHTED PLATEAU
1.10 ± 0.73
TOTAL FUSION
2.13 ± 1.11
NORMAL ISOCHRON
1.10 ± 1.38 (NEG)
INVERSE ISOCHRON
1.11 ± 0.61 (NEG)

MSWD (PROBABILITY)
0.48 (98%)

SPREADING FACTOR
0.3%

40AR/36AR INTERCEPT
297.2 ± 0.9

Sample Info

Groundmass
Gakkel Ridge
Dan Miggins

IRR = 17-OSU-05 (5B17-17)
J = 0.00161094 ± 0.00000130
